# Lymphaticovenous Anastomosis Supermicrosurgery Decreases Oxidative Stress and Increases Antioxidant Capacity in the Serum of Lymphedema Patients

**DOI:** 10.3390/jcm10071540

**Published:** 2021-04-06

**Authors:** Johnson Chia-Shen Yang, Lien-Hung Huang, Shao-Chun Wu, Pao-Jen Kuo, Yi-Chan Wu, Chia-Jung Wu, Chia-Wei Lin, Pei-Yu Tsai, Ching-Hua Hsieh

**Affiliations:** 1 Division of Plastic and Reconstructive Surgery, Department of Surgery, Kaohsiung Chang Gung Memorial Hospital and Graduate Institute of Clinical Medical Sciences, College of Medicine, Chang Gung University, Kaohsiung 833253, Taiwan; prs.lymph@gmail.com (J.C.-S.Y.); ahonbob@gmail.com (L.-H.H.); bow110470@gmail.com (P.-J.K.); janewu0922@gmail.com (Y.-C.W.); alice8818@yahoo.com.tw (C.-J.W.); sallylin1201@gmail.com (C.-W.L.); mermaid85@cgmh.org.tw (P.-Y.T.); 2 Department of Plastic and Reconstructive Surgery, Xiamen Changgung Hospital, Xiamen 361000, China; 3 Department of Anesthesiology, Kaohsiung Chang Gung Memorial Hospital and Chang Gung University College of Medicine, Kaohsiung 833253, Taiwan; shaochunwu@gmail.com

**Keywords:** lymphedema, lymphaticovenous anastomosis, LVA, lymphovenous bypass (LVB), oxidative stress, antioxidant, Enzyme-linked immunosorbent assay (ELISA), iTRAQ, reactive oxygen species (ROS)

## Abstract

Background: Excess lymphedematous tissue causes excessive oxidative stress in lymphedema. Lymphaticovenous anastomosis (LVA) supermicrosurgery is currently emerging as the first-line surgical intervention for lymphedema. No data are available regarding the changes in serum proteins correlating to oxidative stress and antioxidant capacity before and after LVA. Methods: A total of 26 patients with unilateral lower limb lymphedema confirmed by lymphoscintigraphy were recruited, and venous serum samples were collected before (pre-LVA) and after LVA (post-LVA). In 16 patients, the serum proteins were identified by isobaric tags for relative and absolute quantitation-based quantitative proteomic analysis with subsequent validation of protein expression by enzyme-linked immunosorbent assay. An Oxidative Stress Panel Kit was used on an additional 10 patients. Magnetic resonance (MR) volumetry was used to measure t limb volume six months after LVA. Results: This study identified that catalase (CAT) was significantly downregulated after LVA (pre-LVA vs. post-LVA, 2651 ± 2101 vs. 1448 ± 593 ng/mL, respectively, *p* = 0.033). There were significantly higher levels of post-LVA serum total antioxidant capacity (pre-LVA vs. post-LVA, 441 ± 81 vs. 488 ± 59 µmole/L, respectively, *p* = 0.031) and glutathione peroxidase (pre-LVA vs. post-LVA, 73 ± 20 vs. 92 ± 29 U/g, respectively, *p* = 0.018) than pre-LVA serum. In addition, after LVA, there were significantly more differences between post-LVA and pre-LVA serum levels of CAT (good outcome vs. fair outcome, −2593 ± 2363 vs. 178 ± 603 ng/mL, respectively, *p* = 0.021) and peroxiredoxin-2 (PRDX2) (good outcome vs. fair outcome, −7782 ± 7347 vs. −397 ± 1235 pg/mL, respectively, *p* = 0.037) in those patients with good outcomes (≥40% volume reduction in MR volumetry) than those with fair outcomes (<40% volume reduction in MR volumetry). Conclusions: The study revealed that following LVA, differences in some specific oxidative stress markers and antioxidant capacity can be found in the serum of patients with lymphedema.

## 1. Introduction

The lymphatic system maintains interstitial fluid homeostasis and plays a vital role in the immune system [1]. The accumulation of interstitial fluid leads to soft tissue swelling as lymphedema is associated with chronic inflammation and subsequent deposition of abnormal adipose and tissue fibrosis [2]. Lymphedema commonly occurs when the lymphatics are damaged by cancer, infection, trauma, or radiotherapy [3]. A substantial number of patients treated for gynecologic malignancies have sustained lower limb lymphedema [4]. The deformity in aesthetics and malfunction that accompanies lower limb lymphedema can lead to a negative body image and greatly affect a patient’s ability to perform daily activities [5].

Oxidative stress is a major contributor to degenerative and chronic diseases [6,7,8,9,10,11,12,13]. It has been proposed that the regional oxygen deficiency followed by periods of reperfusion in lymphedematous tissue causes excessive oxidative stress [14,15,16,17], and the greater the volume of the lymphedema fluid, the higher the level of oxidative stress [16,17]. In addition, controlling the circumference of the lymphedematous limb may decrease oxidative stress [18]. In patients with chronic lymphedema, treatment with sodium selenite can decrease the production of reactive oxygen species (ROS) and reduce the lymphedema volume by lowering the incidence of local infections [19]. Although prophylactic antibiotics may also be beneficial [20], they do not treat obstructed lymphatic flow, and their therapeutic effect may be temporary. Therefore, mitigating the burden of lymphedema is considered essential in treating this illness. Currently, surgery such as lymphaticovenous anastomosis (LVA) [21] and vascularized lymph node transfer [22], vascularized lymph node flap transfer [23], and combined treatment with liposuction [24] are effective in decreasing the lymphedema burden. Being the least invasive procedure with evidence for the reduction of postoperative cellulitis, LVA is recommended as the first-line surgical intervention for lymphedema [25,26,27,28,29,30,31] and is effective at treating moderate-to-severe lymphedema [32] and even intractable lymphorrhea [33].

However, currently, no data are available regarding the changes in serum proteins correlating to oxidative stress and antioxidant capacity before and after LVA. To explore these changes, serum proteins were identified by isobaric tags for relative and absolute quantitation (iTRAQ)-based quantitative proteomic analysis and an Oxidative Stress Panel Kit. For clinical correlation between oxidative stress and volume reduction, magnetic resonance (MR) volumetry was implemented for precise lymphedematous limb volume measurements before and after LVA.

## 2. Material and Methods

### 2.1. Study Participants

**Figure 1:**
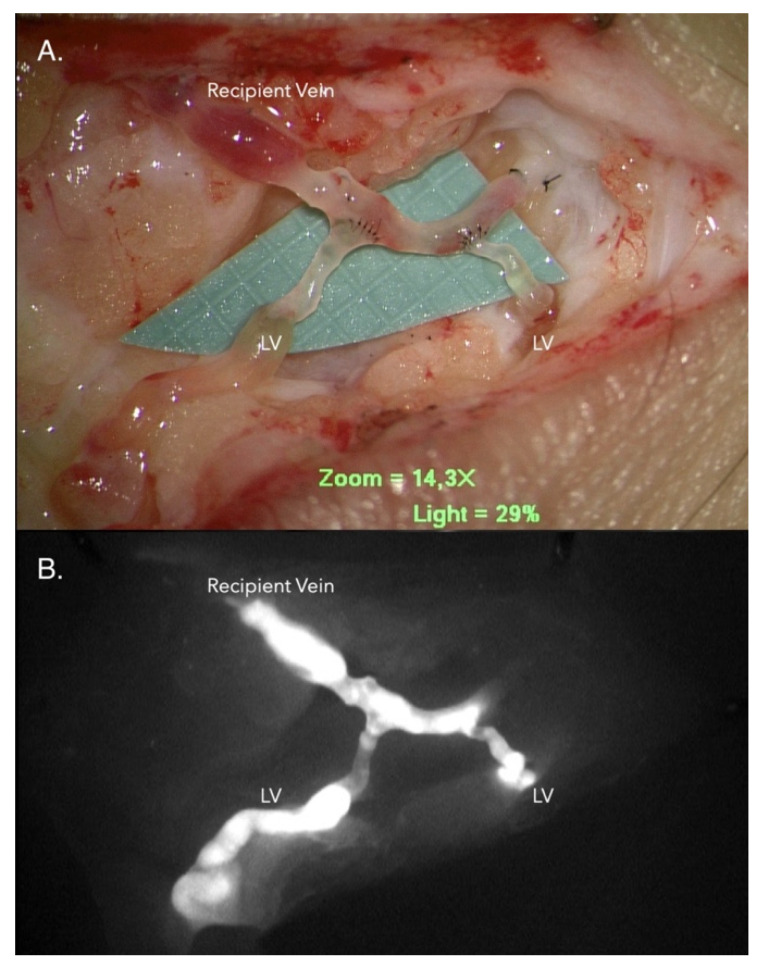
Lymphaticovenous anastomosis (LVA). (**A**) Two lymphatic vessels with diameter 0.7 mm and 0.5 mm were anastomosed to a recipient vein in an end-to-side orientation with 11-0 nylon suture. (**B**) The lumen of the recipient vein became enhanced under near-infrared lymphography. The indocyanine green (ICG)-containing lymph has flowed from the lymphatic vessel into the recipient vein, indicating good antegrade lymphatic flow. Note: the background grid is 1 * 1 mm^2^.

This prospective study was approved by the institutional review board of the Chang Gung Memorial Hospital (approval number 201800306B0) and registered on clinicaltrials.gov (https://clinicaltrials.gov/ct2/show/NCT04552938?term=NCT04552938&draw=2&rank=1, accessed on 17 September 2020). Written informed consent was obtained from all patients prior to enrollment. The recruitment criteria were as follows: (a) unilateral lower limb lymphedema; (b) lymphedema duration > 2 years; (c) no active infection; (d) no prophylactic antibiotic use for at least one month before LVA; (e) no tumor recurrence or metastasis; and (f) no consumption of antioxidants, such as vitamin E or ascorbic acid. Patients with upper limb and bilateral lower limb lymphedema were excluded. Finally, 26 patients with unilateral lower limb lymphedema confirmed by lymphoscintigraphy were recruited between June 2018 and May 2019. All patients were treated with LVA supermicrosurgery, which was performed by the same surgeon (the first author) using a microscope-integrated near-infrared camera (Pentero 900; Carl Zeiss AG, Oberkochen, Germany) according to our previously published methods (as shown in Figure 1) [17,18,19,20]. Custom-made compression stockings were used after surgery for all patients.

### 2.2. LVA Operative Technique

Immediately before operation, 0.1 mL of indocyanine green (ICG) was injected intradermally into the 1st and 3rd toe web spaces and medial and lateral malleolus. A handheld near-infrared imaging device (Fluobeam, FluoOptic, Grenoble, France) was used to detect the dermal backflow (DB) pattern immediately after injection. The ICG-enhanced lymphatic vessels with linear patterns were traced and marked with medical grade marking pen and were used as the basis for incision placement. For moderate-to-severe lymphedema patients with diffuse DB pattern and no linear pattern in sight, incisions were made along the anatomical location of the great saphenous vein. Typically, three incisions were made for each patient. As many LVAs were performed as possible within the operative site. The above-mentioned technique is as described in our previous publication [32].

### 2.3. Magnetic Resonance (MR) Volumetry

**Figure 2:**
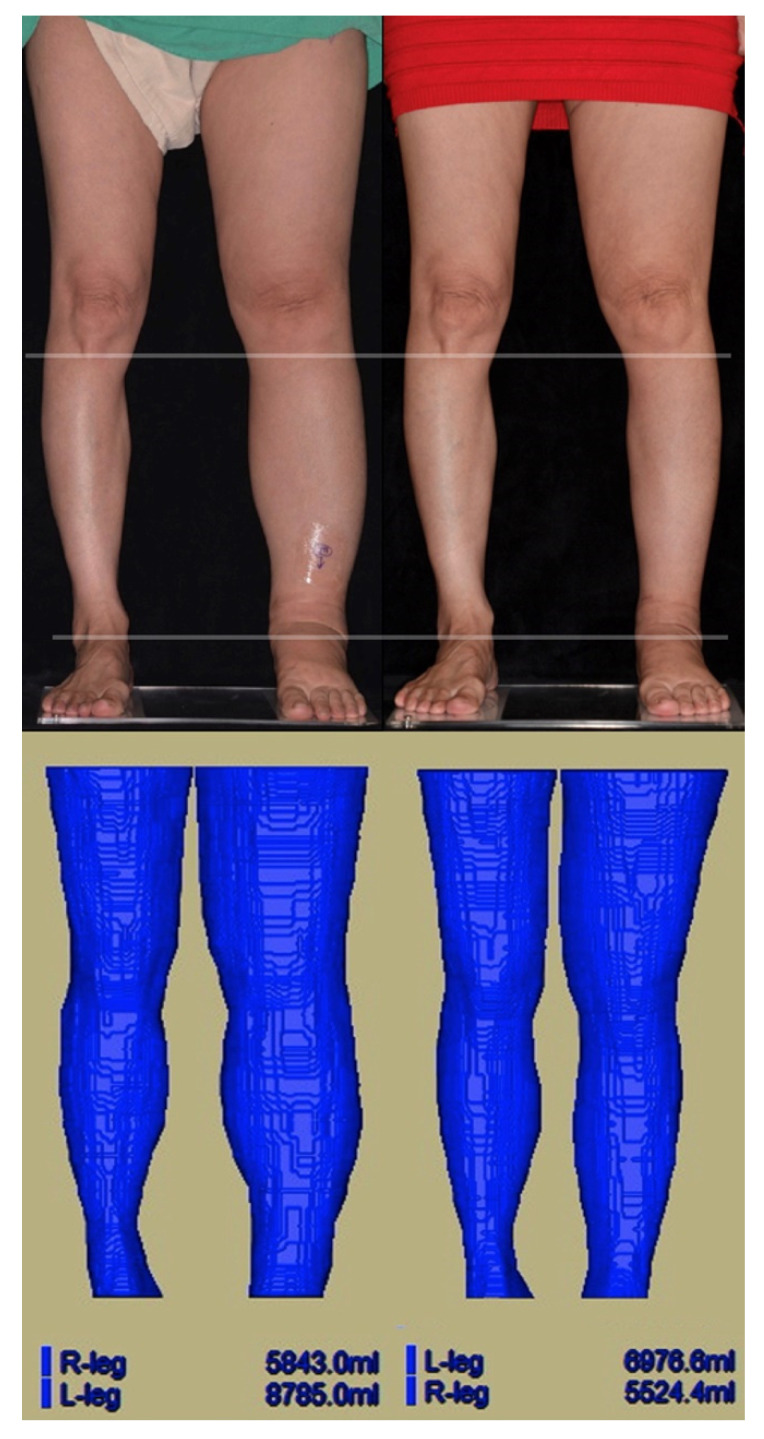
A 55-year-old woman with a BMI of 22.1 kg/m^2^ post-cervical cancer ablation and lymph node dissection (23 years prior). She has suffered from stage III lymphedema on her left lower limb for the past seven years with frequent cellulitis. A total of nine lymphaticovenous anastomoses (LVAs) were performed. (Upper left) Pre-LVA. (Upper right) Six months post-LVA follow-up. (Lower left) Preoperative MR volumetry showing a left lower limb volume of 8785.0 mL; right lower limb volume of 5843.0 mL. The volume gained in the left lower limb due to lymphedema was 2942.0 mL (8785.0 mL minus 5843.0 mL). (Lower right) Six months post-LVA MR volumetry showing left limb volume of 6976.6 mL. Post-LVA volume reduction was −1808.4 mL (a −61.5% lymphedema volume reduction).

Magnetic resonance (MR) volumetry was used for each patient to measure changes in the volume of the affected limb after LVA. Pre-LVA and six-month post-LVA, limb volumes were measured using a 3.0 T Siemens MAGNETOM Skyra scanner (Siemens Healthcare, Erlangen, Germany). MR volumetry was conducted (as shown in Figure 2) according to the protocol of our previous publication [15].

### 2.4. Sample Collection

Venous serum samples were collected from 26 patients on the day before LVA (therefore designated as pre-LVA samples) and one month after LVA (therefore designated as post-LVA samples). They were collected from the same non-dominant forearm area, at the same site, before and after LVA. Among these, serum samples from 16 patients were used for proteomic analysis, and the serum samples of the other 10 patients were assayed using an Oxidative Stress Panel Kit (Clinical Proteomics Core Laboratory, Chang Gung Memorial Hospital, Linkou, Taiwan). For iTRAQ experiments, with the serum samples of three patients being pooled as one sample, two pre-LVA pooled serum samples and two post-LVA pooled serum samples were acquired. These four pooled serum samples were labeled with 4-plex iTRAQ reagents (Sigma-aldrich, St. Louis, MO, USA). Enzyme-linked immunosorbent assay (ELISA) was used for further confirmation of the identified targets from the iTRAQ experiments in these 16 patients. Antioxidant capacity was detected, using the Oxidative Stress Panel kit, for the serum of the remaining 10 patients.

### 2.5. iTRAQ-Based Quantitative Proteomic Analysis

Before iTRAQ labeling, serum samples were individually depleted of 12 high-abundance proteins using TOP 12 abundant protein depletion columns (Thermo/Pierce^TM^, 85,165, Thermo Fisher Scientific, Waltham, MA, USA). In total, 600 µg of serum protein from each sample was added to the resin slurry in the column and incubated for 60 min at 25 °C. The pooled samples were desalted using a centrifugal filter tube (Amicon Ultra-4 tube, molecular weight cut off = 3 kDa, Millipore, UFC800396, Merck, Burlington, MA, USA). Next, 25 µg of desalted samples were dried using SpeedVac (Thermo Fisher Scientific, Waltham, MA, USA) and re-suspended in 20 µL 0.5 M triethylammonium bicarbonate (TEAB, pH 8.5). After being reduced and alkylated through tris-(2-carboxyethyl) phosphine and iodoacetamide, protein samples were resolved using sequencing grade modified trypsin (V511A, Promega, Madison, WI, USA). For iTRAQ labeling, digested samples were dried using SpeedVac and restored with 10 µL 0.5 M TEAB buffer. Samples were labeled with iTRAQ according to the manufacturer’s protocol (Applied Biosystems Inc., Foster City, CA, USA) for subsequent 2D LC-MS/MS analysis. Four-channel iTRAQ-labeled peptide mixtures were desalted using Sep-Pak C18 cartridges (Waters Corporation, Milford, MA, USA) and fractionated by reversed-phase C18 (Thermo Fisher Scientific), according to the manufacturer’s recommendations. The bound peptides were eluted in a 1% ammonia solution with various amounts of acetonitrile (5–50%). All fractions were analyzed using a Q Exactive^TM^ HF mass spectrometer (Thermo Fisher Scientific) coupled with an HPLC System (UltiMate™ 3000 RSLCnano HPLC system, Thermo Fisher Scientific).

The MS raw data were searched using the Mascot search algorithm (version 2.5, Matrix Science, Boston, MA, USA) against the Swiss-Prot human protein database using Proteome Discoverer (version 2.1, Thermo Fisher Scientific) software. The search parameters were set as follows: carbamidomethylation at cysteine as a fixed modification, oxidation at methionine, acetylation at the protein N-terminus, iTRAQ-labeled at the peptide N-terminus and lysine residue as variable modifications, 10 ppm for MS tolerance and 0.02 Da for MS/MS tolerance, and a maximum of two missing cleavage sites. Protein identification with two unique peptides and quantification with two spectrum ratio counts was contributed by the unique peptides. The relative protein levels in pre- and post-LVA were determined by total iTRAQ-labeled peptide abundance. The peptide abundance assigned for each protein was determined using Proteome Discoverer software. Next, the protein abundance data were analyzed by Partek to generate a differentially expressed protein list.

### 2.6. Validation of the Protein Expression by ELISA

Quantification of the identified protein targets from the iTRAQ-based quantitative proteomic analysis was further validated by ELISA. Serum levels of catalase (CAT), peroxiredoxin-2 (PRDX2), carbonic anhydrase 1 (CA1), and carbonic anhydrase 2 (CA2) were determined using ELISA kits (CAT: #ab171572, Abcam, Cambridge, UK; PRDX2: #EK1528, Boster, Pleasanton, CA, USA; CA1: #OKEH01283, Aviva System Biology, San Diego, CA, USA; CA2: #ab222881 Abcam, Cambridge, UK) and a TECAN Sunrise ELISA Reader (TECAN, Lake Zürich, Switzerland).

### 2.7. Oxidative Stress Panel Kit

The total antioxidant capacity (TAC), a marker of the antioxidant status of samples that possess an antioxidant response against free radicals [21], was detected by the ferric reducing ability of plasma (FRAP) [22]. In the FRAP assay, the Fe(III)/tri-pyridyltriazine complex reductants in the sample were reduced, which produced the blue ferrous form, resulting in an increase in absorbance at 593 nm. The readings of the FRAP value were taken at 4 min and expressed as μmol/g. The levels of glutathione peroxidase (GPX), 8-hydroxy-2′-deoxyguanosine (8-OHdG), and myeloperoxidase (MPO) were determined using a RANSEL kit (#RS504, RNADOX, Kearneysville, WV, USA); antibody for 8-OHdG (#AB5830, Sigma, St. Louis, MO, USA); and MPO antibody (#K86005M, Biodesign, Palo Alto, CA, USA), respectively, using a TECAN Sunrise ELISA Reader (TECAN, Reading, UK).

### 2.8. Statistical Analysis

All statistical analyses were performed using Windows version 23.0 for SPSS (IBM Inc., Chicago, IL, USA). Continuous data were expressed as mean ± standard deviation. The ELISA results were analyzed using a paired *t*-test. All statistical tests were two-tailed, and differences were considered significant at *p* < 0.05.

## 3. Results

### 3.1. Patient Demographics

Of the 26 enrolled patients, 25 were female and 1 was male, with an average age of 59.6 ± 11.1 years. The average body mass index (BMI) was 25.8 ± 5.4, kg/m^2^. The most frequent associated illness of the patients was cervical cancer (*n* = 13), followed by endometrial cancer (*n* = 5), ovarian cancer (*n* = 5), and non-cancer patients (*n* = 3). All patients were suffering from International Society of Lymphology (ISL) stage II-III lymphedema. The mean duration of lymphedema was 6.0 ± 6.5 years, with 16 affected lower limbs on the left side and 10 on the right side. The mean LVA performed was 8.1 ± 3.0 per patient. Among those 23 patients who had complete MR volumetry (three patients had incomplete MRI imaging), the mean volume gained in the lymphedema limbs as compared to the contralateral normal limbs was 2369 ± 1380 mL. After LVA, the mean volume reduction at six months post operation was 902.6 ± 855.5 mL, which correlated with a reduction percentage of volume of 40.5 ± 26.5%.

### 3.2. iTRAQ-Based Quantitative Proteomic Analysis

Among the proteins identified through iTRAQ-based quantitative proteomic analysis (Supplemental Table S1), 14 differentially expressed proteins (4 upregulated and 10 downregulated proteins) with 1.5-fold changes were identified with Partek analysis in the post-LVA serum samples as compared to the pre-LVA samples (Table 1). Only four proteins (CAT, PRDX2, CA1, and hemoglobin subunit alpha) showed statistically significant changes after LVA. Considering that hemoglobin may be easily influenced during venipuncture and that the focus of this study was to investigate the oxidative stress associated with lymphedema, we chose four other protein targets (CAT, PRDX2, CA1, and CA2) for further validation of their expression by ELISA. The ELISA assay (Table 2) revealed that only CAT was significantly downregulated after LVA (pre-LVA vs. post-LVA, 2651 ± 2101 vs. 1448 ± 593 ng/mL, respectively, *p* = 0.033). There was no significant difference in the levels of PRDX2, CA1, and CA2 between the pre-LVA and post-LVA serum samples.

### 3.3. Measurement by Oxidative Stress Panel

The Oxidative Stress Panel Kit detected significantly higher levels of post-LVA serum TAC (pre-LVA vs. post-LVA, 441 ± 81 vs. 488 ± 59 µmole/L, respectively, *p* = 0.031) and GPX (pre-LVA vs. post-LVA, 73 ± 20 vs. 92 ± 29 U/g, respectively, *p* = 0.018) than in pre-LVA serum (Table 2). No significant difference in the serum levels of 8-OHdG and MPO between pre-LVA and post-LVA samples was found.

### 3.4. Comparison of the Patients with Good vs. Fair Outcomes of Volume Reduction

As shown in Table 3, in the comparison of expression difference of iTRAQ-identified serum proteins and oxidative stress markers between those patients with good outcomes (≥40% volume reduction in MR volumetry) and fair outcomes (<40% volume reduction in MR volumetry) following LVA, there were significantly more differences between post-LVA and pre-LVA serum levels of CAT (good outcome vs. fair outcome, −2593 ± 2363 vs. 178 ± 603 ng/mL, respectively, *p* = 0.021) and PRDX2 (good outcome vs. fair outcome, −7782 ± 7347 vs. −397 ± 1235 pg/mL, respectively, *p* = 0.037) in those patients with good outcomes than those with fair outcomes. No significant difference was found between patients with good and fair outcomes regarding the expression difference of serum levels of CA1, CA2, and those markers detected in the oxidative stress panel.

## 4. Discussion

Mammalian cells express many hydrogen peroxide (H_2_O_2_)-eliminating enzymes, including CAT, PRDX, and GPX [34]. These three antioxidant enzymes play important roles in antioxidant defense in circulation [34]. CAT is a crucial antioxidant enzyme that interacts with cellular H_2_O_2_ to produce water and oxygen [35]. Under normal conditions, CAT levels are proportionate to H_2_O_2_. An increase in oxidative stress results in an increase in CAT for counteraction [36]. CAT is particularly important when a cell is exposed to high (usually exogenous) levels of H_2_O_2_ In contrast, PRDX2 and GPX are involved in exposure to low (usually endogenous from hemoglobin) levels of H_2_O_2_ [34]. In this study, the serum level of CAT was significantly downregulated after LVA, and the post-LVA reduction in CAT level was significantly higher in good outcome group (>40% volume reduction) compared to the fair outcome group. Therefore, the significant serum CAT reduction after LVA may signify a reduction in oxidative stress associated with a decrease in lymphedematous volume.

PRDX2, a family of peroxidases, has been proposed as a useful real-time marker of oxidative stress [37]. PRDXs are ROS scavengers that hydrolyze H_2_O_2_ with a catalytic cysteine residue [38]. PRDX2 is one of the most abundant PRDXs among the mammalian PRDX family [38] and works in conjunction with CAT to eliminate H_2_O_2_ [39]. PRDX2 is also involved in the maintenance of hemoglobin stability [40], suppression of apoptosis, and stimulation of cell proliferation, resulting in a rapid recovery of the cell redox state [41]. In this study, the change in PRDX2 level between post-LVA and pre-LVA was significantly different in patients with good outcomes compared to those who had a fair outcome. Furthermore, although the serum level of PRDX2 was not significantly changed after LVA (*p* = 0.066), a decreased level of PRDX2 was noted (pre-LVA vs. post-LVA, 4444 ± 5972 vs. 1083 ± 2260 pg/mL). Therefore, the reduction in serum PRDX2 level after LVA may also signify a reduction in oxidative stress associated with the improvement of the lymphedematous status in the lower limb.

During lymphedema, lymphatic stasis mirrors many of the mechanisms behind inflammation [17]. This inflammatory state can be further exacerbated by a cellulitis episode, with additional damage to the lymphatics [42]. The serum TAC levels represent the cumulative action of all antioxidants present in the body fluids [43] and may act as a prognostic marker for sepsis severity [44,45]. Lower leg cellulitis can significantly reduce serum TAC levels and therefore blood antioxidant reserves [18]. In addition, GPX is involved in the detoxification of H_2_O_2_ [46] and works synergistically with CAT and PRDX2 [47] to maintain homeostasis. In this study, significant increases in serum TAC and GPX following LVA were observed. The change in TAC and GPX levels may indicate a restoration in the antioxidant reserve after LVA with limb volume reduction, although the change in TAC and GPX levels was not significantly different between those who had good outcomes and fair outcomes.

In this study, no significant change in 8-OHdG levels was found after LVA. 8-OHdG is a product of oxidative damage of 2′-deoxyguanosine and can be used for assessing oxidative DNA damage [48]. However, the attack on DNA by reactive nitrogen or chlorine species only produces a minor product of 8-OHdG, and therefore its level may not quantitatively reflect damage to the DNA by the reactive species [49]. Notably, it is also possible that the changes in 8-OHdG levels in DNA are a result of changes in the redox state and availability of the transition metal ion and not from the sum of oxidative damage to DNA [50].

For iTRAQ-based quantitative proteomic analysis, pooling samples is a common practice. Pooled samples may also allow for chemical analysis to be conducted when individual samples are too small to provide enough material, resulting in fewer non-detects [51]. Validation with ELISA was then performed on the identified protein markers in each patient. In many biochemical studies, the results from using pooled samples or individual samples are almost the same, except for losing some of the maximal value or specific individual information; however the cost-effectiveness will be much better [52].

This is the first study to demonstrate reductions in some specific oxidative stress markers and improved antioxidant capacity in the serum of lower limb lymphedema patients after LVA. The decrease in oxidative stress and increased antioxidant capacity after lymphedematous limb volume reduction offer clues to the correlation between oxidative stress and lymphedema. Lower limb lymphedema may be a localized disease but is accompanied with systemic effects. These findings are a small step toward understanding the pathophysiology of lymphedema. More research is mandated toward the improvement of, and maybe even possibly a cure, for this debilitating disease.

The present study has some limitations. First, the 26 patients included in this study were not enrolled consecutively because not every patient was willing to participate in a study that required blood withdrawal in an outpatient clinic. This might have created bias in patient selection attributable to patient willingness to have blood withdrawn. Second, the sample size was too small to reach a solid conclusion. A larger sample size of patients with longer follow-up will be advantageous to the validation of long-term effects. Third, a decrease in antioxidant capacity has been correlated with being overweight or obese, which was defined as BMI ≧ 30 kg/m^2^ [53]. BMI is associated not only with antioxidants, but also with the severity of lymphedema. However, our patient group was not obese (BMI 25.8 ± 5.4, kg/m^2^), and the mean post-LVA volume reduction at six months was 902.6 ± 855.5 mL, which might be too small to reflect on BMI. Finally, in this study, direct evidence between volume reduction and a decrease in oxidative stress was not demonstrated.

## 5. Conclusions

The study revealed that following LVA, differences in some specific oxidative stress markers and antioxidant capacity can be found in the serum of patients with lymphedema.

## Figures and Tables

**Table 1:** List of differentially-expressed proteins identified by isobaric tags for relative and absolute quantitation (iTRAQ) following lymphaticovenous anastomosis.

Gene Names	Protein Names	Fold Change	*p* Value
*IGHV3-30*	Ig heavy chain V-III region CAM	4.58	0.384
*KRT1*	Keratin, type II cytoskeletal 1	2.18	0.408
*ALB*	Serum albumin	1.69	0.259
*KRT9*	Keratin, type I cytoskeletal 9	1.69	0.402
*THBS1*	Thrombospondin-1	−1.53	0.101
*CAT*	Catalase	−1.58	0.049
*PRDX2*	Peroxiredoxin-2	−1.68	0.022
*RPS27A*	Ubiquitin-40S ribosomal protein S27a	−1.69	0.169
*SAA1*	Serum amyloid A-1 protein	−1.73	0.289
*CA1*	Carbonic anhydrase 1	−1.89	0.039
*HBD*	Hemoglobin subunit delta	−2.42	0.140
*HBB*	Hemoglobin subunit beta	−2.62	0.067
*CA2*	Carbonic anhydrase 2	−2.64	0.066
*HBA1*	Hemoglobin subunit alpha	−3.09	0.035

iTRAQ, isobaric tags for relative and absolute quantitation; Fold change indicated the serum level of post-LVA against that of pre-LVA.

**Table 2:** Change of iTRAQ-identified serum proteins and oxidative stress markers following lymphaticovenous anastomosis (LVA).

	Pre-LVA	Post-LVA	*p* Value
iTRAQ-identified protein			
CAT, ng/mL	2651 ± 2101	1448 ± 593	0.033
PRDX2, pg/mL	4444 ± 5972	1083 ± 2260	0.066
CA1, IU/mL	576 ± 296	593 ± 378	0.703
CA2, pg/mL	436 ± 335	427 ± 391	0.752
Oxidative Stress Panel			
TAC, umole/L	441 ± 81	488 ± 59	0.031
GPX, U/g	73 ± 20	92 ± 29	0.018
8-OHdG, ng/mg	41 ± 12	45 ± 10	0.501
MPO, ng/mL	67 ± 21	79 ± 23	0.255

iTRAQ, isobaric tags for relative and absolute quantitation; CAT, catalase; PRDX2, peroxiredoxin-2; CA1, carbonic anhydrase 1; CA2, carbonic anhydrase 2; TAC, total antioxidant capacity; GPX, glutathione Peroxidase; 8-OHdG, 8-hydroxy-2′-deoxyguanosine; MPO, Myeloperoxidase. Data are expressed as mean ± standard deviation.

**Table 3:** Comparison of the expression difference of iTRAQ-identified serum proteins and oxidative stress markers between those patients with good outcomes (≥40 volume reduction in MR volumetry) and fair outcomes (<40% volume reduction in MR volumetry) following lymphaticovenous anastomosis.

	Good Outcome	Fair Outcome	*p* Value
iTRAQ-identified protein			
CAT, ng/mL	−2593 ± 2363	178 ± 603	0.021
PRDX2, pg/mL	−7782 ± 7347	−397 ± 1235	0.037
CA1, IU/mL	14 ± 175	126 ± 312	0.426
CA2, pg/mL	0.05 ± 0.04	−0.03 ± 0.14	0.199
Oxidative Stress Panel			
TAC, umole/L	21 ± 23	40 ± 39	0.406
GPX, U/g	12 ± 14	20 ± 25	0.557
8-OHdG, ng/mg	4 ± 15	7 ± 19	0.799
MPO, ng/mL	9.0 ± 13	14 ± 45	0.833

CAT, catalase; PRDX2, peroxiredoxin-2; CA1, carbonic anhydrase 1; CA2, carbonic anhydrase 2; TAC, total antioxidant capacity; GPX, glutathione Peroxidase; 8-OHdG, 8-hydroxy-2′-deoxyguanosine; MPO, Myeloperoxidase; MR, magnetic resonance. Data are presented as post-LVA serum level minus pre-LVA serum level and expressed as mean ± standard deviation.

## Data Availability

The data presented in this study are available on request from the corresponding author.

## Supplementary Materials

The following are available online at https://www.mdpi.com/article/10.3390/jcm10071540/s1: Supplementary Table S1. iTRAQ-based quantitative proteomic analysis.

## Abbreviations

8-OHdG, 8-hydroxy-2′-deoxyguanosine; BMI, body mass index; CA1, carbonic anhydrase 1; CA2, carbonic anhydrase 2; ELISA, enzyme-linked immunosorbent assay; iTRAQ, isobaric tags for relative and absolute quantitation; GPX, glutathione peroxidase; LVA, lymphaticovenous anastomosis; MPO, myeloperoxidase; MR, magnetic resonance; PRDX2, peroxiredoxin-2; and TAC, total antioxidant capacity.
