# Supplementary material for: Lymphaticovenous Anastomosis Supermicrosurgery Decreases Oxidative Stress and Increases Antioxidant Capacity in the Serum of Lymphedema Patients
Source: J Clin Med. 2021 Apr 6;10(7):1540. doi: 10.3390/jcm10071540 (PMC8038828; doi:10.3390/jcm10071540)
Supplement: Supplementary file 1 [file jcm-10-01540-s001.pdf]

Accession
P08709
P33151
Q9BXR6
P00441
P98160
Q9UJJ9
P13796
Q9HDC9
P07996
P01034
Q15113
Q9NPH3
Q13740
P02042
P36980
Q9NQ79
P04180
Q9UNW1
P43121
P05109
Q9Y4L1
Q15063
Q99784
P02452
P35908
O14786
P19320
P05154
Q9Y5Y7
O00187
P02746
P00915
P12111
P13591
P04070
P08294
P23470
P61769
P00746
P22891
Q9UGM5
P48740
P04040
P41222
P09172
O00533
Q9UNN8
Q12805
Q92820
Q9UK55

P15169
P13645
P00739
P0C0L5
P14151
P01860
P11226
P06702
P35443
P55058
O43866
P08571
P80108
P02741
P49908
P03951
P60709
O00391
Q9UHG3
P43251
P01859
P04264
Q76LX8
P05062
P17936
Q15485
Q13790
Q92954
P02747
P01876
P49747
P11021
Q6UX71
P32119
P08519
P13727
Q15582
P01591
P08833
B9A064
Q96KN2
P22105
P02763
P05452
Q16610
P20742
P55056
P22352
P00742
P02771
P23142
P01834

P00748
P12259
P35527
P06276
Q08380
P00740
P15144
P02745
O14791
Q96IY4
P20851
P02656
Q04756
P26927
P27169
P10909
Q6UXB8
P16070
O95445
P02743
P02655
P07358
P22792
P04278
P02753
P69905
P02748
P27918
P05156
P02751
Q14520
P00918
P05090
P00736
P01857
P07225
P04275
P09871
P0DJI8
Q96PD5
P01019
P02654
O75636
P19652
P08185
P07357
O75882
P01871
P36955
P35542
P10643
P02760

P29622
Q06033
P18428
Q9NZP8
P03952
P13671
P25311
P02649
P02775
P05543
P02652
P35858
P04004
P08697
P68871
P02750
P01008
P51884
P07360
P00734
P06396
P08603
P06681
P04003
P02766
P02787
P19827
P04196
P05155
P06727
P05546
P02749
P01042
P02765
P01031
P43652
P00747
P19823
P02768
P00738
P01011
P04217
P01023
P00450
P00751
Q14624
P01009
P02790
P02774
P0C0L4
P02647
P04114

P01024
Q562R1
P13598
Q02325
P0CG38
P0CG39
Q16473
Q15166
P62736
P63267
Q15195
Q6S8J3
A5A3E0
Q9BYX7
P68032
P68133
P63261
Q2TV78
P04220

Description	Exp. q-value
Coagulation factor VII OS=Homo sapiens GN=F7 PE=1 SV=1	0
Cadherin-5 OS=Homo sapiens GN=CDH5 PE=1 SV=5	0
Complement factor H-related protein 5 OS=Homo sapiens GN=CFHR5 PE=1 SV=1	0
Superoxide dismutase [Cu-Zn] OS=Homo sapiens GN=SOD1 PE=1 SV=2	0
Basement membrane-specific heparan sulfate proteoglycan core protein OS=Homo sapie	0
N-acetylglucosamine-1-phosphotransferase subunit gamma OS=Homo sapiens GN=GNPT	0
Plastin-2 OS=Homo sapiens GN=LCP1 PE=1 SV=6	0
Adipocyte plasma membrane-associated protein OS=Homo sapiens GN=APMAP PE=1 SV=	0
Thrombospondin-1 OS=Homo sapiens GN=THBS1 PE=1 SV=2	0
Cystatin-C OS=Homo sapiens GN=CST3 PE=1 SV=1	0
Procollagen C-endopeptidase enhancer 1 OS=Homo sapiens GN=PCOLCE PE=1 SV=2	0
Interleukin-1 receptor accessory protein OS=Homo sapiens GN=IL1RAP PE=1 SV=2	0
CD166 antigen OS=Homo sapiens GN=ALCAM PE=1 SV=2	0
Hemoglobin subunit delta OS=Homo sapiens GN=HBD PE=1 SV=2	0
Complement factor H-related protein 2 OS=Homo sapiens GN=CFHR2 PE=1 SV=1	0
Cartilage acidic protein 1 OS=Homo sapiens GN=CRTAC1 PE=1 SV=2	0
Phosphatidylcholine-sterol acyltransferase OS=Homo sapiens GN=LCAT PE=1 SV=1	0
Multiple inositol polyphosphate phosphatase 1 OS=Homo sapiens GN=MINPP1 PE=1 SV=	0
Cell surface glycoprotein MUC18 OS=Homo sapiens GN=MCAM PE=1 SV=2	0
Protein S100-A8 OS=Homo sapiens GN=S100A8 PE=1 SV=1	0.007
Hypoxia up-regulated protein 1 OS=Homo sapiens GN=HYOU1 PE=1 SV=1	0.007
Periostin OS=Homo sapiens GN=POSTN PE=1 SV=2	0
Noelin OS=Homo sapiens GN=OLFM1 PE=1 SV=4	0
Collagen alpha-1(I) chain OS=Homo sapiens GN=COL1A1 PE=1 SV=5	0.007
Keratin, type II cytoskeletal 2 epidermal OS=Homo sapiens GN=KRT2 PE=1 SV=2	0
Neuropilin-1 OS=Homo sapiens GN=NRP1 PE=1 SV=3	0
Vascular cell adhesion protein 1 OS=Homo sapiens GN=VCAM1 PE=1 SV=1	0
Plasma serine protease inhibitor OS=Homo sapiens GN=SERPINA5 PE=1 SV=3	0
Lymphatic vessel endothelial hyaluronic acid receptor 1 OS=Homo sapiens GN=LYVE1 PE	0
Mannan-binding lectin serine protease 2 OS=Homo sapiens GN=MASP2 PE=1 SV=4	0
Complement C1q subcomponent subunit B OS=Homo sapiens GN=C1QB PE=1 SV=3	0
Carbonic anhydrase 1 OS=Homo sapiens GN=CA1 PE=1 SV=2	0
Collagen alpha-3(VI) chain OS=Homo sapiens GN=COL6A3 PE=1 SV=5	0
Neural cell adhesion molecule 1 OS=Homo sapiens GN=NCAM1 PE=1 SV=3	0
Vitamin K-dependent protein C OS=Homo sapiens GN=PROC PE=1 SV=1	0
Extracellular superoxide dismutase [Cu-Zn] OS=Homo sapiens GN=SOD3 PE=1 SV=2	0
Receptor-type tyrosine-protein phosphatase gamma OS=Homo sapiens GN=PTPRG PE=1 S	0.007
Beta-2-microglobulin OS=Homo sapiens GN=B2M PE=1 SV=1	0
Complement factor D OS=Homo sapiens GN=CFD PE=1 SV=5	0
Vitamin K-dependent protein Z OS=Homo sapiens GN=PROZ PE=1 SV=2	0
Fetuin-B OS=Homo sapiens GN=FETUB PE=1 SV=2	0
Mannan-binding lectin serine protease 1 OS=Homo sapiens GN=MASP1 PE=1 SV=3	0
Catalase OS=Homo sapiens GN=CAT PE=1 SV=3	0
Prostaglandin-H2 D-isomerase OS=Homo sapiens GN=PTGDS PE=1 SV=1	0
Dopamine beta-hydroxylase OS=Homo sapiens GN=DBH PE=1 SV=3	0
Neural cell adhesion molecule L1-like protein OS=Homo sapiens GN=CHL1 PE=1 SV=4	0
Endothelial protein C receptor OS=Homo sapiens GN=PROCR PE=1 SV=1	0.007
EGF-containing fibulin-like extracellular matrix protein 1 OS=Homo sapiens GN=EFEMP1	0
Gamma-glutamyl hydrolase OS=Homo sapiens GN=GGH PE=1 SV=2	0
Protein Z-dependent protease inhibitor OS=Homo sapiens GN=SERPINA10 PE=1 SV=1	0

Carboxypeptidase N catalytic chain OS=Homo sapiens GN=CPN1 PE=1 SV=1	0
Keratin, type I cytoskeletal 10 OS=Homo sapiens GN=KRT10 PE=1 SV=6	0
Haptoglobin-related protein OS=Homo sapiens GN=HPR PE=2 SV=2	0
Complement C4-B OS=Homo sapiens GN=C4B PE=1 SV=2	0
L-selectin OS=Homo sapiens GN=SELL PE=1 SV=2	0
Ig gamma-3 chain C region OS=Homo sapiens GN=IGHG3 PE=1 SV=2	0
Mannose-binding protein C OS=Homo sapiens GN=MBL2 PE=1 SV=2	0
Protein S100-A9 OS=Homo sapiens GN=S100A9 PE=1 SV=1	0.007
Thrombospondin-4 OS=Homo sapiens GN=THBS4 PE=1 SV=2	0
Phospholipid transfer protein OS=Homo sapiens GN=PLTP PE=1 SV=1	0
CD5 antigen-like OS=Homo sapiens GN=CD5L PE=1 SV=1	0
Monocyte differentiation antigen CD14 OS=Homo sapiens GN=CD14 PE=1 SV=2	0
Phosphatidylinositol-glycan-specific phospholipase D OS=Homo sapiens GN=GPLD1 PE=	0
C-reactive protein OS=Homo sapiens GN=CRP PE=1 SV=1	0
Selenoprotein P OS=Homo sapiens GN=SEPP1 PE=1 SV=3	0
Coagulation factor XI OS=Homo sapiens GN=F11 PE=1 SV=1	0
Actin, cytoplasmic 1 OS=Homo sapiens GN=ACTB PE=1 SV=1	0
Sulfhydryl oxidase 1 OS=Homo sapiens GN=QSOX1 PE=1 SV=3	0
Prenylcysteine oxidase 1 OS=Homo sapiens GN=PCYOX1 PE=1 SV=3	0
Biotinidase OS=Homo sapiens GN=BTD PE=1 SV=2	0
Ig gamma-2 chain C region OS=Homo sapiens GN=IGHG2 PE=1 SV=2	0
Keratin, type II cytoskeletal 1 OS=Homo sapiens GN=KRT1 PE=1 SV=6	0
A disintegrin and metalloproteinase with thrombospondin motifs 13 OS=Homo sapiens	0
Fructose-bisphosphate aldolase B OS=Homo sapiens GN=ALDOB PE=1 SV=2	0.007
Insulin-like growth factor-binding protein 3 OS=Homo sapiens GN=IGFBP3 PE=1 SV=2	0
Ficolin-2 OS=Homo sapiens GN=FCN2 PE=1 SV=2	0
Apolipoprotein F OS=Homo sapiens GN=APOF PE=1 SV=2	0
Proteoglycan 4 OS=Homo sapiens GN=PRG4 PE=1 SV=2	0
Complement C1q subcomponent subunit C OS=Homo sapiens GN=C1QC PE=1 SV=3	0
Ig alpha-1 chain C region OS=Homo sapiens GN=IGHA1 PE=1 SV=2	0
Cartilage oligomeric matrix protein OS=Homo sapiens GN=COMP PE=1 SV=2	0
78 kDa glucose-regulated protein OS=Homo sapiens GN=HSPA5 PE=1 SV=2	0.007
Plexin domain-containing protein 2 OS=Homo sapiens GN=PLXDC2 PE=1 SV=1	0
Peroxiredoxin-2 OS=Homo sapiens GN=PRDX2 PE=1 SV=5	0
Apolipoprotein(a) OS=Homo sapiens GN=LPA PE=1 SV=1	0
Bone marrow proteoglycan OS=Homo sapiens GN=PRG2 PE=1 SV=2	0
Transforming growth factor-beta-induced protein ig-h3 OS=Homo sapiens GN=TGFBI PE=	0
Immunoglobulin J chain OS=Homo sapiens GN=JCHAIN PE=1 SV=4	0
Insulin-like growth factor-binding protein 1 OS=Homo sapiens GN=IGFBP1 PE=1 SV=1	0
Immunoglobulin lambda-like polypeptide 5 OS=Homo sapiens GN=IGLL5 PE=2 SV=2	0
Beta-Ala-His dipeptidase OS=Homo sapiens GN=CNDP1 PE=1 SV=4	0
Tenascin-X OS=Homo sapiens GN=TNXB PE=1 SV=4	0
Alpha-1-acid glycoprotein 1 OS=Homo sapiens GN=ORM1 PE=1 SV=1	0
Tetranectin OS=Homo sapiens GN=CLEC3B PE=1 SV=3	0
Extracellular matrix protein 1 OS=Homo sapiens GN=ECM1 PE=1 SV=2	0
Pregnancy zone protein OS=Homo sapiens GN=PZP PE=1 SV=4	0
Apolipoprotein C-IV OS=Homo sapiens GN=APOC4 PE=1 SV=1	0
Glutathione peroxidase 3 OS=Homo sapiens GN=GPX3 PE=1 SV=2	0
Coagulation factor X OS=Homo sapiens GN=F10 PE=1 SV=2	0
Alpha-fetoprotein OS=Homo sapiens GN=AFP PE=1 SV=1	0
Fibulin-1 OS=Homo sapiens GN=FBLN1 PE=1 SV=4	0
Ig kappa chain C region OS=Homo sapiens GN=IGKC PE=1 SV=1	0

Coagulation factor XII OS=Homo sapiens GN=F12 PE=1 SV=3	0
Coagulation factor V OS=Homo sapiens GN=F5 PE=1 SV=4	0
Keratin, type I cytoskeletal 9 OS=Homo sapiens GN=KRT9 PE=1 SV=3	0
Cholinesterase OS=Homo sapiens GN=BCHE PE=1 SV=1	0
Galectin-3-binding protein OS=Homo sapiens GN=LGALS3BP PE=1 SV=1	0
Coagulation factor IX OS=Homo sapiens GN=F9 PE=1 SV=2	0
Aminopeptidase N OS=Homo sapiens GN=ANPEP PE=1 SV=4	0
Complement C1q subcomponent subunit A OS=Homo sapiens GN=C1QA PE=1 SV=2	0
Apolipoprotein L1 OS=Homo sapiens GN=APOL1 PE=1 SV=5	0
Carboxypeptidase B2 OS=Homo sapiens GN=CPB2 PE=1 SV=2	0
C4b-binding protein beta chain OS=Homo sapiens GN=C4BPB PE=1 SV=1	0
Apolipoprotein C-III OS=Homo sapiens GN=APOC3 PE=1 SV=1	0
Hepatocyte growth factor activator OS=Homo sapiens GN=HGFAC PE=1 SV=1	0
Hepatocyte growth factor-like protein OS=Homo sapiens GN=MST1 PE=1 SV=2	0
Serum paraoxonase/arylesterase 1 OS=Homo sapiens GN=PON1 PE=1 SV=3	0
Clusterin OS=Homo sapiens GN=CLU PE=1 SV=1	0
Peptidase inhibitor 16 OS=Homo sapiens GN=PI16 PE=1 SV=1	0
CD44 antigen OS=Homo sapiens GN=CD44 PE=1 SV=3	0
Apolipoprotein M OS=Homo sapiens GN=APOM PE=1 SV=2	0
Serum amyloid P-component OS=Homo sapiens GN=APCS PE=1 SV=2	0
Apolipoprotein C-II OS=Homo sapiens GN=APOC2 PE=1 SV=1	0
Complement component C8 beta chain OS=Homo sapiens GN=C8B PE=1 SV=3	0
Carboxypeptidase N subunit 2 OS=Homo sapiens GN=CPN2 PE=1 SV=3	0
Sex hormone-binding globulin OS=Homo sapiens GN=SHBG PE=1 SV=2	0
Retinol-binding protein 4 OS=Homo sapiens GN=RBP4 PE=1 SV=3	0
Hemoglobin subunit alpha OS=Homo sapiens GN=HBA1 PE=1 SV=2	0
Complement component C9 OS=Homo sapiens GN=C9 PE=1 SV=2	0
Properdin OS=Homo sapiens GN=CFP PE=1 SV=2	0
Complement factor I OS=Homo sapiens GN=CFI PE=1 SV=2	0
Fibronectin OS=Homo sapiens GN=FN1 PE=1 SV=4	0
Hyaluronan-binding protein 2 OS=Homo sapiens GN=HABP2 PE=1 SV=1	0
Carbonic anhydrase 2 OS=Homo sapiens GN=CA2 PE=1 SV=2	0
Apolipoprotein D OS=Homo sapiens GN=APOD PE=1 SV=1	0
Complement C1r subcomponent OS=Homo sapiens GN=C1R PE=1 SV=2	0
Ig gamma-1 chain C region OS=Homo sapiens GN=IGHG1 PE=1 SV=1	0
Vitamin K-dependent protein S OS=Homo sapiens GN=PROS1 PE=1 SV=1	0
von Willebrand factor OS=Homo sapiens GN=VWF PE=1 SV=4	0
Complement C1s subcomponent OS=Homo sapiens GN=C1S PE=1 SV=1	0
Serum amyloid A-1 protein OS=Homo sapiens GN=SAA1 PE=1 SV=1	0
N-acetylmuramoyl-L-alanine amidase OS=Homo sapiens GN=PGLYRP2 PE=1 SV=1	0
Angiotensinogen OS=Homo sapiens GN=AGT PE=1 SV=1	0
Apolipoprotein C-I OS=Homo sapiens GN=APOC1 PE=1 SV=1	0
Ficolin-3 OS=Homo sapiens GN=FCN3 PE=1 SV=2	0
Alpha-1-acid glycoprotein 2 OS=Homo sapiens GN=ORM2 PE=1 SV=2	0
Corticosteroid-binding globulin OS=Homo sapiens GN=SERPINA6 PE=1 SV=1	0
Complement component C8 alpha chain OS=Homo sapiens GN=C8A PE=1 SV=2	0
Attractin OS=Homo sapiens GN=ATRN PE=1 SV=2	0
Ig mu chain C region OS=Homo sapiens GN=IGHM PE=1 SV=3	0
Pigment epithelium-derived factor OS=Homo sapiens GN=SERPINF1 PE=1 SV=4	0
Serum amyloid A-4 protein OS=Homo sapiens GN=SAA4 PE=1 SV=2	0
Complement component C7 OS=Homo sapiens GN=C7 PE=1 SV=2	0
Protein AMBP OS=Homo sapiens GN=AMBP PE=1 SV=1	0

Kallistatin OS=Homo sapiens GN=SERPINA4 PE=1 SV=3	0
Inter-alpha-trypsin inhibitor heavy chain H3 OS=Homo sapiens GN=ITIH3 PE=1 SV=2	0
Lipopolysaccharide-binding protein OS=Homo sapiens GN=LBP PE=1 SV=3	0
Complement C1r subcomponent-like protein OS=Homo sapiens GN=C1RL PE=1 SV=2	0
Plasma kallikrein OS=Homo sapiens GN=KLKB1 PE=1 SV=1	0
Complement component C6 OS=Homo sapiens GN=C6 PE=1 SV=3	0
Zinc-alpha-2-glycoprotein OS=Homo sapiens GN=AZGP1 PE=1 SV=2	0
Apolipoprotein E OS=Homo sapiens GN=APOE PE=1 SV=1	0
Platelet basic protein OS=Homo sapiens GN=PPBP PE=1 SV=3	0
Thyroxine-binding globulin OS=Homo sapiens GN=SERPINA7 PE=1 SV=2	0
Apolipoprotein A-II OS=Homo sapiens GN=APOA2 PE=1 SV=1	0
Insulin-like growth factor-binding protein complex acid labile subunit OS=Homo sapiens	0
Vitronectin OS=Homo sapiens GN=VTN PE=1 SV=1	0
Alpha-2-antiplasmin OS=Homo sapiens GN=SERPINF2 PE=1 SV=3	0
Hemoglobin subunit beta OS=Homo sapiens GN=HBB PE=1 SV=2	0
Leucine-rich alpha-2-glycoprotein OS=Homo sapiens GN=LRG1 PE=1 SV=2	0
Antithrombin-III OS=Homo sapiens GN=SERPINC1 PE=1 SV=1	0
Lumican OS=Homo sapiens GN=LUM PE=1 SV=2	0
Complement component C8 gamma chain OS=Homo sapiens GN=C8G PE=1 SV=3	0
Prothrombin OS=Homo sapiens GN=F2 PE=1 SV=2	0
Gelsolin OS=Homo sapiens GN=GSN PE=1 SV=1	0
Complement factor H OS=Homo sapiens GN=CFH PE=1 SV=4	0
Complement C2 OS=Homo sapiens GN=C2 PE=1 SV=2	0
C4b-binding protein alpha chain OS=Homo sapiens GN=C4BPA PE=1 SV=2	0
Transthyretin OS=Homo sapiens GN=TTR PE=1 SV=1	0
Serotransferrin OS=Homo sapiens GN=TF PE=1 SV=3	0
Inter-alpha-trypsin inhibitor heavy chain H1 OS=Homo sapiens GN=ITIH1 PE=1 SV=3	0
Histidine-rich glycoprotein OS=Homo sapiens GN=HRG PE=1 SV=1	0
Plasma protease C1 inhibitor OS=Homo sapiens GN=SERPING1 PE=1 SV=2	0
Apolipoprotein A-IV OS=Homo sapiens GN=APOA4 PE=1 SV=3	0
Heparin cofactor 2 OS=Homo sapiens GN=SERPIND1 PE=1 SV=3	0
Beta-2-glycoprotein 1 OS=Homo sapiens GN=APOH PE=1 SV=3	0
Kininogen-1 OS=Homo sapiens GN=KNG1 PE=1 SV=2	0
Alpha-2-HS-glycoprotein OS=Homo sapiens GN=AHSG PE=1 SV=1	0
Complement C5 OS=Homo sapiens GN=C5 PE=1 SV=4	0
Afamin OS=Homo sapiens GN=AFM PE=1 SV=1	0
Plasminogen OS=Homo sapiens GN=PLG PE=1 SV=2	0
Inter-alpha-trypsin inhibitor heavy chain H2 OS=Homo sapiens GN=ITIH2 PE=1 SV=2	0
Serum albumin OS=Homo sapiens GN=ALB PE=1 SV=2	0
Haptoglobin OS=Homo sapiens GN=HP PE=1 SV=1	0
Alpha-1-antichymotrypsin OS=Homo sapiens GN=SERPINA3 PE=1 SV=2	0
Alpha-1B-glycoprotein OS=Homo sapiens GN=A1BG PE=1 SV=4	0
Alpha-2-macroglobulin OS=Homo sapiens GN=A2M PE=1 SV=3	0
Ceruloplasmin OS=Homo sapiens GN=CP PE=1 SV=1	0
Complement factor B OS=Homo sapiens GN=CFB PE=1 SV=2	0
Inter-alpha-trypsin inhibitor heavy chain H4 OS=Homo sapiens GN=ITIH4 PE=1 SV=4	0
Alpha-1-antitrypsin OS=Homo sapiens GN=SERPINA1 PE=1 SV=3	0
Hemopexin OS=Homo sapiens GN=HPX PE=1 SV=2	0
Vitamin D-binding protein OS=Homo sapiens GN=GC PE=1 SV=1	0
Complement C4-A OS=Homo sapiens GN=C4A PE=1 SV=2	0
Apolipoprotein A-I OS=Homo sapiens GN=APOA1 PE=1 SV=1	0
Apolipoprotein B-100 OS=Homo sapiens GN=APOB PE=1 SV=2	0

Complement C3 OS=Homo sapiens GN=C3 PE=1 SV=2	0
Beta-actin-like protein 2 OS=Homo sapiens GN=ACTBL2 PE=1 SV=2	0
Intercellular adhesion molecule 2 OS=Homo sapiens GN=ICAM2 PE=1 SV=2	0.007
Plasminogen-like protein B OS=Homo sapiens GN=PLGLB1 PE=3 SV=1	0
POTE ankyrin domain family member I OS=Homo sapiens GN=POTEI PE=3 SV=1	0.007
POTE ankyrin domain family member J OS=Homo sapiens GN=POTEJ PE=3 SV=1	0.007
Putative tenascin-XA OS=Homo sapiens GN=TNXA PE=5 SV=2	0.007
Serum paraoxonase/lactonase 3 OS=Homo sapiens GN=PON3 PE=1 SV=3	0
Actin, aortic smooth muscle OS=Homo sapiens GN=ACTA2 PE=1 SV=1	0
Actin, gamma-enteric smooth muscle OS=Homo sapiens GN=ACTG2 PE=1 SV=1	0
Plasminogen-like protein A OS=Homo sapiens GN=PLGLA PE=2 SV=1	0
POTE ankyrin domain family member E OS=Homo sapiens GN=POTEE PE=1 SV=3	0
POTE ankyrin domain family member F OS=Homo sapiens GN=POTEF PE=1 SV=2	0
Putative beta-actin-like protein 3 OS=Homo sapiens GN=POTEKP PE=5 SV=1	0
Actin, alpha cardiac muscle 1 OS=Homo sapiens GN=ACTC1 PE=1 SV=1	0
Actin, alpha skeletal muscle OS=Homo sapiens GN=ACTA1 PE=1 SV=1	0
Actin, cytoplasmic 2 OS=Homo sapiens GN=ACTG1 PE=1 SV=1	0
Putative macrophage stimulating 1-like protein OS=Homo sapiens GN=MST1L PE=2 SV=2	0
Ig mu heavy chain disease protein OS=Homo sapiens PE=1 SV=1	0

Coverage	# Peptides	# PSMs	# Unique Peptides	# Protein Groups	MW [kDa]	Score A Mascot
7.081545064	2	2	2	1	51.6	34
3.698979592	2	2	2	1	87.5	51
6.502636204	3	3	2	1	64.4	46
14.28571429	2	2	2	1	15.9	52
0.72876338	3	3	3	1	468.5	77
10.81967213	2	2	2	1	34	122
7.177033493	4	4	4	1	70.2	86
6.971153846	3	3	3	1	46.5	62
3.931623932	4	4	4	1	129.3	50
24.65753425	3	5	3	1	15.8	145
9.799554566	3	3	3	1	47.9	82
4.736842105	3	3	3	1	65.4	61
4.288164666	2	2	2	1	65.1	51
52.38095238	7	15	2	1	16	508
11.48148148	3	5	2	1	30.6	114
10.13615734	5	5	5	1	71.4	151
14.54545455	4	8	4	1	49.5	161
7.802874743	3	3	3	1	55	66
7.120743034	4	5	4	1	71.6	114
15.05376344	2	2	2	1	10.8	60
1.701701702	2	2	2	1	111.3	26
2.870813397	3	3	3	1	93.3	49
3.917525773	2	2	2	1	55.3	43
1.43442623	2	4	2	1	138.9	30
9.85915493	5	6	4	1	65.4	113
3.683640303	3	3	3	1	103.1	50
8.525033829	6	6	6	1	81.2	124
16.25615764	6	10	6	1	45.6	205
8.074534161	3	3	3	1	35.2	79
7.288629738	4	4	4	1	75.7	94
20.55335968	4	7	4	1	26.7	242
24.13793103	5	8	5	1	28.9	265
2.644003777	6	6	6	1	343.5	148
6.177156177	5	6	5	1	94.5	139
5.856832972	2	8	2	1	52	88
12.08333333	2	4	2	1	25.8	84
1.176470588	2	2	2	1	161.9	50
16.80672269	2	3	2	1	13.7	109
30.83003953	5	8	5	1	27	148
14.25	5	6	5	1	44.7	106
15.44502618	5	8	5	1	42	177
14.16309013	9	12	8	1	79.2	241
7.020872865	3	3	3	1	59.7	47
13.15789474	2	2	2	1	21	66
17.17990276	8	9	8	1	69	150
7.201986755	7	7	7	1	135	92
7.56302521	2	2	2	1	26.7	21
9.53346856	4	8	4	1	54.6	135
16.35220126	5	5	5	1	35.9	104
16.66666667	6	7	6	1	50.7	98

22.48908297	8	14	8	1	52.3	207
5.136986301	3	3	3	1	58.8	68
36.20689655	13	62	3	1	39	1443
62.3853211	85	433	3	1	192.6	11605
8.870967742	3	4	3	1	42.2	117
30.76923077	9	23	3	1	41.3	685
20.96774194	5	8	5	1	26.1	211
14.03508772	2	2	2	1	13.2	39
7.180020812	5	5	2	1	105.8	116
12.1703854	5	5	5	1	54.7	137
14.4092219	4	4	4	1	38.1	105
22.13333333	6	8	6	1	40.1	129
19.04761905	12	16	12	1	92.3	339
20.08928571	4	5	4	1	25	83
6.56167979	3	5	3	1	43.2	108
19.36	11	11	11	1	70.1	209
16.53333333	5	5	5	1	41.7	77
10.84337349	6	7	6	1	82.5	174
10.2970297	5	6	5	1	56.6	92
18.96869245	8	16	8	1	61.1	435
32.20858896	8	23	3	1	35.9	575
20.1863354	12	20	11	1	66	421
1.681850035	2	2	2	1	153.5	62
9.065934066	2	2	2	1	39.4	28
27.14776632	7	11	7	1	31.7	167
9.584664537	3	4	3	1	34	110
11.65644172	3	7	3	1	35.4	245
6.98005698	11	13	11	1	151	180
25.30612245	5	15	5	1	25.8	368
43.3427762	10	18	5	1	37.6	422
14.00264201	8	12	5	1	82.8	363
2.44648318	2	2	2	1	72.3	65
8.695652174	4	5	4	1	59.5	78
23.23232323	4	5	4	1	21.9	73
28.75989446	14	21	14	1	501	498
23.87387387	4	8	4	1	25.2	194
6.588579795	5	7	5	1	74.6	122
27.04402516	4	6	4	1	18.1	188
15.44401544	4	11	4	1	27.9	269
26.63551402	4	15	2	1	23	448
16.765286	7	11	7	1	56.7	222
0.942951438	3	3	3	1	457.9	50
44.7761194	8	46	5	1	23.5	1059
32.17821782	6	21	6	1	22.5	578
30.37037037	14	21	14	1	60.6	432
15.38461538	19	35	14	1	163.8	815
27.55905512	4	9	4	1	14.5	223
25.66371681	5	7	5	1	25.5	166
15.77868852	7	15	7	1	54.7	325
1.97044335	2	4	2	1	68.6	130
15.93172119	8	12	8	1	77.2	278
80.18867925	5	24	5	1	11.6	755

16.42276423	8	9	8	1	67.7	145
9.622302158	22	26	22	1	251.5	521
20.86677368	11	16	11	1	62	514
16.94352159	10	13	10	1	68.4	320
28.03418803	14	22	14	1	65.3	522
26.68112798	10	18	10	1	51.7	428
3.929679421	4	5	4	1	109.5	95
23.26530612	4	5	4	1	26	318
21.35678392	10	19	10	1	43.9	284
15.13002364	5	11	5	1	48.4	293
47.22222222	9	17	9	1	28.3	328
58.58585859	6	31	6	1	10.8	817
20.91603053	11	17	11	1	70.6	348
27.56680731	13	17	13	1	80.3	319
39.15492958	10	27	10	1	39.7	727
30.73496659	13	36	13	1	52.5	863
16.1987041	6	10	6	1	49.4	225
3.908355795	3	5	3	1	81.5	99
51.06382979	8	17	8	1	21.2	346
30.94170404	9	22	9	1	25.4	602
49.5049505	4	23	4	1	11.3	733
38.07106599	16	34	16	1	67	768
31.74311927	13	21	13	1	60.5	566
28.10945274	8	11	8	1	43.8	383
71.64179104	10	40	10	1	23	1271
69.71830986	7	17	7	1	15.2	454
33.63148479	19	51	19	1	63.1	1245
24.30703625	9	17	9	1	51.2	299
34.47684391	17	46	17	1	65.7	1405
21.54233026	37	57	37	1	262.5	827
17.67857143	9	25	9	1	62.6	400
7.307692308	2	2	2	1	29.2	62
26.45502646	5	19	5	1	21.3	436
40.9929078	22	39	20	1	80.1	1077
41.81818182	10	37	5	1	36.1	924
31.80473373	17	37	17	1	75.1	563
14.14859581	33	38	33	1	309.1	710
34.73837209	18	47	17	1	76.6	1161
27.86885246	2	4	2	1	13.5	201
40.45138889	13	39	13	1	62.2	1153
30.72164948	10	38	10	1	53.1	1198
37.34939759	7	28	7	1	9.3	611
39.13043478	8	17	8	1	32.9	522
43.78109453	8	37	5	1	23.6	906
31.60493827	10	39	10	1	45.1	976
25.85616438	12	26	12	1	65.1	694
14.06578027	18	47	18	1	158.4	1168
37.38938053	15	52	15	1	49.3	1063
49.5215311	17	46	17	1	46.3	1385
27.69230769	4	15	4	1	14.7	286
38.79003559	26	54	26	1	93.5	1347
45.73863636	12	70	12	1	39	2290

48.94613583	17	36	17	1	48.5	708
22.92134831	17	39	17	1	99.8	567
8.316008316	4	4	4	1	53.4	54
16.42710472	7	11	5	1	53.5	272
41.06583072	24	51	24	1	71.3	1129
29.87152034	27	59	27	1	104.7	1308
48.32214765	14	45	14	1	34.2	1252
70.03154574	22	77	22	1	36.1	2172
25	3	5	3	1	13.9	111
40.48192771	14	23	14	1	46.3	698
64	8	63	8	1	11.2	1059
37.19008264	17	42	17	1	66	1113
40.37656904	15	74	15	1	54.3	1736
47.25050916	19	48	19	1	54.5	1361
93.19727891	11	28	6	1	16	891
43.80403458	13	33	13	1	38.2	1126
50	27	121	27	1	52.6	2817
43.19526627	14	66	14	1	38.4	1580
49.00990099	7	22	7	1	22.3	573
55.14469453	26	136	26	1	70	4429
35.42199488	30	84	30	1	85.6	2635
40.04874086	40	125	37	1	139	2858
32.18085106	22	64	22	1	83.2	1347
46.73366834	24	123	24	1	67	2580
65.30612245	12	77	12	1	15.9	2450
54.7277937	37	102	37	1	77	2581
37.65093304	25	141	25	1	101.3	3803
36.95238095	17	84	17	1	59.5	2105
41.2	20	103	20	1	55.1	2356
75.25252525	32	148	32	1	45.4	3409
38.0761523	18	69	18	1	57	1364
48.69565217	15	109	15	1	38.3	2291
43.94409938	30	157	30	1	71.9	4039
49.86376022	11	149	11	1	39.3	4336
35.50119332	52	128	52	1	188.2	3079
51.0851419	30	129	30	1	69	3424
61.85185185	45	162	45	1	90.5	4258
46.93446089	38	217	38	1	106.4	5469
76.68308703	50	438	50	1	69.3	9831
54.67980296	21	138	11	1	45.2	2919
50.59101655	19	201	19	1	47.6	6103
42.62626263	15	132	15	1	54.2	3098
61.46540027	63	308	58	1	163.2	8279
58.87323944	42	277	42	1	122.1	7772
48.56020942	35	168	35	1	85.5	4099
48.49462366	36	213	36	1	103.3	4735
63.15789474	25	185	25	1	46.7	3842
64.06926407	24	320	24	1	51.6	9302
80.16877637	32	303	32	1	52.9	8068
63.18807339	86	429	4	1	192.7	11495
83.52059925	31	466	31	1	30.8	11539
54.72277011	222	782	222	1	515.3	19592

81.53938665	129	1607	129	1	187	44146
7.180851064	2	2	2	1	42	25
8	2	2	2	1	30.6	18
18.75	2	16	2	1	11	353
2.23255814	2	2	2	1	121.2	44
2.312138728	2	2	2	1	117.3	44
8.038585209	2	2	2	1	33.7	47
4.519774011	2	5	2	1	39.6	85
10.0795756	3	3	3	1	42	49
10.10638298	3	3	3	1	41.9	49
16.66666667	3	8	3	1	10.9	136
3.720930233	3	3	3	1	121.3	50
3.720930233	3	3	3	1	121.4	50
10.66666667	3	3	3	1	42	50
12.99734748	4	4	4	1	42	64
12.99734748	4	4	4	1	42	64
16.53333333	5	5	5	1	41.8	77
11.32867133	5	8	5	1	79.6	138
26.59846547	9	33	9	1	43	693

Annotated Sequence	Modifications	# Protein Gr	# Proteins
[K].lHVDPENFR.[L]	N-Term(iTRAQ4plex)	2	2
[K].vVAGVANALAHk.[Y]	N-Term(iTRAQ4plex); K12(iTRAQ	2	2
[K].vVAGVANALAHk.[Y]	N-Term(iTRAQ4plex); K12(iTRAQ	2	2
[K].vVAGVANALAHk.[Y]	N-Term(iTRAQ4plex); K12(iTRAQ	2	2
[K].VVAGVANALAHk.[Y]	K12(iTRAQ4plex)	2	2
[M].vHLTPEEk.[TS]	N-Term(iTRAQ4plex); K8(iTRAQ4	2	2
[R].lLVVYPWTQR.[F]	N-Term(iTRAQ4plex)	2	5
[R].lLVVYPWTQR.[F]	N-Term(iTRAQ4plex)	2	5
[K].vLGAFSDGLAHLDNLk.[G]	N-Term(iTRAQ4plex); K16(iTRAQ	2	2
[K].lHVDPENFR.[L]	N-Term(iTRAQ4plex)	2	2
[K].tAVNALWGk.[V]	N-Term(iTRAQ4plex); K9(iTRAQ4	1	1
[K].lHVDPENFR.[L]	N-Term(iTRAQ4plex)	2	2
[K].VVAGVANALAHk.[Y]	K12(iTRAQ4plex)	2	2
[R].LLVVYPWTQR.[F]		2	5
[K].vNVDAVGGEALGR.[L]	N-Term(iTRAQ4plex)	1	1
[K].ANPTVTLFPPSSEELQANk.[A]	K19(iTRAQ4plex)	1	2
[K].yAASSYLSLTPEQWk.[S]	N-Term(iTRAQ4plex); K15(iTRAQ	3	6
[K].yAASSYLSLTPEQWk.[S]	N-Term(iTRAQ4plex); K15(iTRAQ	3	6
[K].aNPTVTLFPPSSEELQANk.[A]	N-Term(iTRAQ4plex); K19(iTRAQ	1	2
[K].aNPTVTLFPPSSEELQANk.[A]	N-Term(iTRAQ4plex); K19(iTRAQ	1	2
[RK].sYScQVTHEGSTVEk.[T]	N-Term(iTRAQ4plex); C4(Carbam	2	5
[RK].sYScQVTHEGSTVEk.[T]	N-Term(iTRAQ4plex); C4(Carbam	2	5
[K].ANPTVTLFPPSSEELQANk.[A]	K19(iTRAQ4plex)	1	2
[K].ANPTVTLFPPSSEELQANk.[A]	K19(iTRAQ4plex)	1	2
[RK].sYScQVTHEGSTVEk.[T]	N-Term(iTRAQ4plex); C4(Carbam	2	5
[K].vTVLGQPk.[A]	N-Term(iTRAQ4plex); K8(iTRAQ4	1	1
[K].vTVLGQPk.[A]	N-Term(iTRAQ4plex); K8(iTRAQ4	1	1
[K].vTVLGQPk.[A]	N-Term(iTRAQ4plex); K8(iTRAQ4	1	1
[RK].sYScQVTHEGSTVEk.[T]	N-Term(iTRAQ4plex); C4(Carbam	2	5
[K].yAASSYLSLTPEQWk.[S]	N-Term(iTRAQ4plex); K15(iTRAQ	3	6
[K].eQQcVImAENR.[K]	N-Term(iTRAQ4plex); C4(Carbam	1	2
[K].eQQcVImAENR.[K]	N-Term(iTRAQ4plex); C4(Carbam	1	2
[K].eQQcVImAENR.[K]	N-Term(iTRAQ4plex); C4(Carbam	1	2
[K].eQQcVIMAENR.[K]	N-Term(iTRAQ4plex); C4(Carbam	1	2
[K].eQQcVIMAENR.[K]	N-Term(iTRAQ4plex); C4(Carbam	1	2
[K].eQQcVIMAENR.[K]	N-Term(iTRAQ4plex); C4(Carbam	1	2
[K].eQQcVImAENR.[K]	N-Term(iTRAQ4plex); C4(Carbam	1	2
[R].aFQYHSk.[E]	N-Term(iTRAQ4plex); K7(iTRAQ4	1	3
[K].EQQcVIMAENR.[K]	C4(Carbamidomethyl)	1	2
[R].aFQYHSk.[E]	N-Term(iTRAQ4plex); K7(iTRAQ4	1	3
[R].aFQYHSk.[E]	N-Term(iTRAQ4plex); K7(iTRAQ4	1	3
[K].EQQcVIMAENR.[K]	C4(Carbamidomethyl)	1	2
[K].eQQcVImAENR.[K]	N-Term(iTRAQ4plex); C4(Carbam	1	2
[R].aFQYHSk.[E]	N-Term(iTRAQ4plex); K7(iTRAQ4	1	3
[K].eQQcVImAENR.[K]	N-Term(iTRAQ4plex); C4(Carbam	1	2
[R].aFQYHSk.[E]	N-Term(iTRAQ4plex); K7(iTRAQ4	1	3
[R].fFGHGAEDSLADQAANEWGR.[S]	N-Term(iTRAQ4plex)	1	1
[R].fFGHGAEDSLADQAANEWGR.[S]	N-Term(iTRAQ4plex)	1	1
[R].sFFSFLGEAFDGAR.[D]	N-Term(iTRAQ4plex)	1	2
[R].fFGHGAEDSLADQAANEWGR.[S]	N-Term(iTRAQ4plex)	1	1
[RK].iDVcPENAEVTLTDFR.[A]	N-Term(iTRAQ4plex); C4(Carbam	2	2

[R].wFLQHRPQVGYIR.[V]	N-Term(iTRAQ4plex)	2	2
[K].dVDIDSYPDEELPcSAR.[N]	N-Term(iTRAQ4plex); C14(Carbar	1	1
[R].nTIAEcQAcGPLk.[F]	N-Term(iTRAQ4plex); C6(Carbam	1	1
[R].aVAEPGIQLk.[A]	N-Term(iTRAQ4plex); K10(iTRAQ	2	2
[R].rHPQLAVSVILR.[V]	N-Term(iTRAQ4plex)	1	1
[R].rHPQLAVSVILR.[V]	N-Term(iTRAQ4plex)	1	1
[R].rHPQLAVSVILR.[V]	N-Term(iTRAQ4plex)	1	1
[R].hPQLAVSVILR.[V]	N-Term(iTRAQ4plex)	1	1
[K].INHGILYDEEk.[Y]	K11(iTRAQ4plex)	2	2
[K].iNHGILYDEEk.[Y]	N-Term(iTRAQ4plex); K11(iTRAQ	2	2
[K].iNHGILYDEEk.[Y]	N-Term(iTRAQ4plex); K11(iTRAQ	2	2
[R].sTISAEk.[C]	N-Term(iTRAQ4plex); K7(iTRAQ4	1	1
[R].iTcAEEGWSPTPk.[C]	N-Term(iTRAQ4plex); C3(Carbam	1	1
[K].tPEENEPTQLEGGPDSLGFETLENcR.[K]	N-Term(iTRAQ4plex); C25(Carbar	1	1
[K].vELAcGk.[S]	N-Term(iTRAQ4plex); C5(Carbam	1	1
[R].AGLAASLAGPHSIVGR.[A]		1	1
[R].aGLAASLAGPHSIVGR.[A]	N-Term(iTRAQ4plex)	1	1
[R].aVVVHAGEDDLGR.[G]	N-Term(iTRAQ4plex)	1	1
[R].aVVVHAGEDDLGR.[G]	N-Term(iTRAQ4plex)	1	1
[K].aQGFTEDTIVFLPQTDk.[C]	N-Term(iTRAQ4plex); K17(iTRAQ	1	1
[K].kAALSMck.[S]	N-Term(iTRAQ4plex); K1(iTRAQ4	1	1
[K].iLIGTVFHk.[AT]	N-Term(iTRAQ4plex); K9(iTRAQ4	1	1
[K].sTVEIFk.[F]	N-Term(iTRAQ4plex); K7(iTRAQ4	1	1
[K].ILIGTVFHk.[AT]	K9(iTRAQ4plex)	1	1
[K].iLIGTVFHk.[AT]	N-Term(iTRAQ4plex); K9(iTRAQ4	1	1
[K].iLIGTVFHk.[AT]	N-Term(iTRAQ4plex); K9(iTRAQ4	1	1
[K].lGDDLLQcHPAVk.[F]	N-Term(iTRAQ4plex); C8(Carbam	1	1
[R].rGDSPWQVVLLDSk.[K]	N-Term(iTRAQ4plex); K14(iTRAQ	1	1
[R].rGDSPWQVVLLDSk.[K]	N-Term(iTRAQ4plex); K14(iTRAQ	1	1
[R].rGDSPWQVVLLDSk.[K]	N-Term(iTRAQ4plex); K14(iTRAQ	1	1
[K].lGDDLLQcHPAVk.[F]	N-Term(iTRAQ4plex); C8(Carbam	1	1
[R].rGDSPWQVVLLDSk.[K]	N-Term(iTRAQ4plex); K14(iTRAQ	1	1
[R].rGDSPWQVVLLDSk.[K]	N-Term(iTRAQ4plex); K14(iTRAQ	1	1
[K].lGDDLLQcHPAVk.[F]	N-Term(iTRAQ4plex); C8(Carbam	1	1
[R].vNHVTLSQPk.[I]	N-Term(iTRAQ4plex); K10(iTRAQ	1	1
[K].vEHSDLSFSk.[D]	N-Term(iTRAQ4plex); K10(iTRAQ	1	1
[R].VNHVTLSQPk.[I]	K10(iTRAQ4plex)	1	1
[K].vGSAkPGLQk.[V]	N-Term(iTRAQ4plex); K5(iTRAQ4	1	1
[K].vVDVLDSIk.[T]	N-Term(iTRAQ4plex); K9(iTRAQ4	1	1
[R].iTcTEEGWSPTPk.[C]	N-Term(iTRAQ4plex); C3(Carbam	2	2
[K].gEcHVPILEANVDAQPk.[K]	N-Term(iTRAQ4plex); C3(Carbam	1	1
[K].iAGVNIk.[T]	N-Term(iTRAQ4plex); K7(iTRAQ4	1	1
[R].lFINVAPHAR.[I]	N-Term(iTRAQ4plex)	1	1
[R].sLVELTPIAAVHGR.[W]	N-Term(iTRAQ4plex)	1	1
[K].kPLIGTVLAMDPDAAR.[H]	N-Term(iTRAQ4plex); K1(iTRAQ4	1	1
[R].vDAETGDVFAIER.[L]	N-Term(iTRAQ4plex)	1	1
[K].yEkPDGSPVFIAFR.[S]	N-Term(iTRAQ4plex); K3(iTRAQ4	1	1
[K].sVQYDDVPEYk.[D]	N-Term(iTRAQ4plex); K11(iTRAQ	1	1
[R].hQGVMVGMGQk.[D]	N-Term(iTRAQ4plex); K11(iTRAQ	1	7
[KR].sYELPDGQVITIGNER.[F]	N-Term(iTRAQ4plex)	1	10
[R].vSQYIEWLQk.[L]	N-Term(iTRAQ4plex); K10(iTRAQ	1	1
[R].vAQVIIPSTYVPGTTNHDIALLR.[L]	N-Term(iTRAQ4plex)	1	1

[R].hVGDLGNVTADk.[D]	N-Term(iTRAQ4plex); K12(iTRAQ	1	1
[R].lAcGVIGIAQ.[-]	N-Term(iTRAQ4plex); C3(Carbam	1	1
[K].fQSHIIIR.[F]	N-Term(iTRAQ4plex)	1	1
[K].lTGISDPVTVk.[T]	N-Term(iTRAQ4plex); K11(iTRAQ	1	1
[K].qEYDESGPSIVHR.[K]	N-Term(iTRAQ4plex)	1	7
[K].IWHHTFYNELR.[V]		1	9
[K].qEYDESGPSIVHR.[K]	N-Term(iTRAQ4plex)	1	7
[K].IWHHTFYNELR.[V]		1	9
[R].nTVVPTk.[K]	N-Term(iTRAQ4plex); K7(iTRAQ4	1	1
[K].eTAEAYLGk.[K]	N-Term(iTRAQ4plex); K9(iTRAQ4	1	1
[K].vLcDDVIcDETk.[N]	N-Term(iTRAQ4plex); C3(Carbam	1	1
[KR].dVWkPEPcR.[I]	N-Term(iTRAQ4plex); K4(iTRAQ4	1	2
[KR].dVWkPEPcR.[I]	N-Term(iTRAQ4plex); K4(iTRAQ4	1	2
[KR].dVWkPEPcR.[I]	N-Term(iTRAQ4plex); K4(iTRAQ4	1	2
[K].lDQGGAPLAGTNk.[E]	N-Term(iTRAQ4plex); K13(iTRAQ	1	1
[R].iADQcPSSLAIQENANALAR.[Y]	N-Term(iTRAQ4plex); C5(Carbam	1	1
[R].lSQLSVTDVTTSSLR.[L]	N-Term(iTRAQ4plex)	1	2
[R].tLSPVLESPR.[D]	N-Term(iTRAQ4plex)	1	2
[R].lTWASHEk.[M]	N-Term(iTRAQ4plex); K8(iTRAQ4	1	1
[R].kDLQNFLk.[K]	N-Term(iTRAQ4plex); K1(iTRAQ4	1	1
[K].tVAILLk.[D]	N-Term(iTRAQ4plex); K7(iTRAQ4	1	1
[K].vcSSPPIHMk.[V]	N-Term(iTRAQ4plex); C2(Carbam	1	1
[K].kLAVEPk.[G]	N-Term(iTRAQ4plex); K1(iTRAQ4	1	1
[R].qVILTLQPTLVAVGk.[S]	N-Term(iTRAQ4plex); K15(iTRAQ	1	1
[K].hISAENTk.[G]	N-Term(iTRAQ4plex); K8(iTRAQ4	1	1
[R].lHMLQISYFR.[D]	N-Term(iTRAQ4plex)	1	1
[K].gADVWFk.[E]	N-Term(iTRAQ4plex); K7(iTRAQ4	1	1
[-].mLTELEk.[A]	N-Term(iTRAQ4plex); K7(iTRAQ4	1	1
[R].eVQYLLNk.[A]	N-Term(iTRAQ4plex); K8(iTRAQ4	1	1
[R].kLcQGLFFR.[V]	N-Term(iTRAQ4plex); K1(iTRAQ4	1	1
[R].gSFEFPVGDAVSk.[V]	N-Term(iTRAQ4plex); K13(iTRAQ	2	2
[R].gSFEFPVGDAVSk.[V]	N-Term(iTRAQ4plex); K13(iTRAQ	2	2
[R].gSFEFPVGDAVSk.[V]	N-Term(iTRAQ4plex); K13(iTRAQ	2	2
[R].gSFEFPVGDAVSk.[V]	N-Term(iTRAQ4plex); K13(iTRAQ	2	2
[R].gSFEFPVGDAVSk.[V]	N-Term(iTRAQ4plex); K13(iTRAQ	2	2
[R].YIYGkPVQGVAYVR.[F]	K5(iTRAQ4plex)	2	2
[R].yLDkTEQWSTLPPETk.[D]	N-Term(iTRAQ4plex); K4(iTRAQ4	2	2
[R].gSFEFPVGDAVSk.[V]	N-Term(iTRAQ4plex); K13(iTRAQ	2	2
[K].vLSLAQEQVGGSPEk.[L]	N-Term(iTRAQ4plex); K15(iTRAQ	2	2
[K].vLSLAQEQVGGSPEk.[L]	N-Term(iTRAQ4plex); K15(iTRAQ	2	2
[K].vLSLAQEQVGGSPEk.[L]	N-Term(iTRAQ4plex); K15(iTRAQ	2	2
[K].vLSLAQEQVGGSPEk.[L]	N-Term(iTRAQ4plex); K15(iTRAQ	2	2
[K].vLSLAQEQVGGSPEk.[L]	N-Term(iTRAQ4plex); K15(iTRAQ	2	2
[R].tYNVLDMk.[N]	N-Term(iTRAQ4plex); K8(iTRAQ4	2	2
[R].vHYTVcIWR.[N]	N-Term(iTRAQ4plex); C6(Carbam	2	2
[R].VGDTLNLNLR.[A]		2	2
[R].tYNVLDMk.[N]	N-Term(iTRAQ4plex); K8(iTRAQ4	2	2
[R].yLDkTEQWSTLPPETk.[D]	N-Term(iTRAQ4plex); K4(iTRAQ4	2	2
[R].yLDkTEQWSTLPPETk.[D]	N-Term(iTRAQ4plex); K4(iTRAQ4	2	2
[R].yLDkTEQWSTLPPETk.[D]	N-Term(iTRAQ4plex); K4(iTRAQ4	2	2
[R].yLDkTEQWSTLPPETk.[D]	N-Term(iTRAQ4plex); K4(iTRAQ4	2	2
[R].yLDkTEQWSTLPPETk.[D]	N-Term(iTRAQ4plex); K4(iTRAQ4	2	2

[R].rGHLFLQTDQPIYNPGQR.[V]	N-Term(iTRAQ4plex)	2	2
[R].rGHLFLQTDQPIYNPGQR.[V]	N-Term(iTRAQ4plex)	2	2
[R].gHLFLQTDQPIYNPGQR.[V]	N-Term(iTRAQ4plex)	2	2
[K].ITQVLHFTk.[D]	K9(iTRAQ4plex)	2	2
[K].ScGLHQLLR.[G]	C2(Carbamidomethyl)	2	2
[R].FGLLDEDGkk.[T]	K9(iTRAQ4plex); K10(iTRAQ4ple	2	2
[K].sHkPLNMGk.[V]	N-Term(iTRAQ4plex); K3(iTRAQ4	2	2
[K].lGQYASPTAk.[R]	N-Term(iTRAQ4plex); K10(iTRAQ	2	2
[K].lVNGQSHISLSk.[A]	N-Term(iTRAQ4plex); K12(iTRAQ	2	2
[R].kADGSYAAWLSR.[DG]	K1(iTRAQ4plex)	2	2
[R].kADGSYAAWLSR.[DG]	N-Term(iTRAQ4plex)	2	2
[R].kADGSYAAWLSR.[DG]	N-Term(iTRAQ4plex)	2	2
[R].kADGSYAAWLSR.[DG]	K1(iTRAQ4plex)	2	2
[R].tLEIPGNSDPNmIPDGDFNSYVR.[V]	N-Term(iTRAQ4plex); M12(Oxida	2	2
[K].sHALQLNNR.[Q]	N-Term(iTRAQ4plex)	2	2
[K].sHkPLNMGk.[V]	N-Term(iTRAQ4plex); K3(iTRAQ4	2	2
[R].tLEIPGNSDPNMIPDGDFNSYVR.[V]	N-Term(iTRAQ4plex)	2	2
[R].tLEIPGNSDPNMIPDGDFNSYVR.[V]	N-Term(iTRAQ4plex)	2	2
[R].TLEIPGNSDPNMIPDGDFNSYVR.[V]		2	2
[R].gLEEELQFSLGSk.[I]	N-Term(iTRAQ4plex); K13(iTRAQ	2	2
[R].gLEEELQFSLGSk.[I]	N-Term(iTRAQ4plex); K13(iTRAQ	2	2
[R].gLEEELQFSLGSk.[I]	N-Term(iTRAQ4plex); K13(iTRAQ	2	2
[R].gLEEELQFSLGSk.[I]	N-Term(iTRAQ4plex); K13(iTRAQ	2	2
[R].gLEEELQFSLGSk.[I]	N-Term(iTRAQ4plex); K13(iTRAQ	2	2
[R].gLEEELQFSLGSk.[I]	N-Term(iTRAQ4plex); K13(iTRAQ	2	2
[R].gLEEELQFSLGSk.[I]	N-Term(iTRAQ4plex); K13(iTRAQ	2	2
[R].eELVYELNPLDHR.[G]	N-Term(iTRAQ4plex)	2	2
[R].eELVYELNPLDHR.[G]	N-Term(iTRAQ4plex)	2	2
[R].eELVYELNPLDHR.[G]	N-Term(iTRAQ4plex)	2	2
[R].eELVYELNPLDHR.[G]	N-Term(iTRAQ4plex)	2	2
[R].cSVFYGAPSk.[S]	N-Term(iTRAQ4plex); C1(Carbam	2	2
[R].cSVFYGAPSk.[S]	N-Term(iTRAQ4plex); C1(Carbam	2	2
[R].vHYTVcIWR.[N]	N-Term(iTRAQ4plex); C6(Carbam	2	2
[K].vDFTLSSER.[D]	N-Term(iTRAQ4plex)	2	2
[R].vHYTVcIWR.[N]	N-Term(iTRAQ4plex); C6(Carbam	2	2
[R].vHYTVcIWR.[N]	N-Term(iTRAQ4plex); C6(Carbam	2	2
[R].kADGSYAAWLSR.[DG]	K1(iTRAQ4plex)	2	2
[K].iTQVLHFTk.[D]	N-Term(iTRAQ4plex); K9(iTRAQ4	2	2
[K].iTQVLHFTk.[D]	N-Term(iTRAQ4plex); K9(iTRAQ4	2	2
[R].kADGSYAAWLSR.[DG]	N-Term(iTRAQ4plex)	2	2
[K].iTQVLHFTk.[D]	N-Term(iTRAQ4plex); K9(iTRAQ4	2	2
[R].GHLFLQTDQPIYNPGQR.[V]		2	2
[K].mRPSTDTITVMVENSHGLR.[V]	N-Term(iTRAQ4plex)	2	2
[K].sHkPLNMGk.[V]	N-Term(iTRAQ4plex); K3(iTRAQ4	2	2
[K].sHkPLNMGk.[V]	N-Term(iTRAQ4plex); K3(iTRAQ4	2	2
[R].aLEILQEEDLIDEDDIPVR.[S]	N-Term(iTRAQ4plex)	2	2
[R].kADGSYAAWLSR.[DG]	N-Term(iTRAQ4plex); K1(iTRAQ4	2	2
[K].mRPSTDTITVMVENSHGLR.[V]	N-Term(iTRAQ4plex); M1(Oxidat	2	2
[R].rGHLFLQTDQPIYNPGQR.[V]	N-Term(iTRAQ4plex)	2	2
[R].rGHLFLQTDQPIYNPGQR.[V]	N-Term(iTRAQ4plex)	2	2
[R].rGHLFLQTDQPIYNPGQR.[V]	N-Term(iTRAQ4plex)	2	2
[R].rGHLFLQTDQPIYNPGQR.[V]	N-Term(iTRAQ4plex)	2	2

[R].yIYGkPVQGVAYVR.[F]	N-Term(iTRAQ4plex); K5(iTRAQ4	2	2
[R].yIYGkPVQGVAYVR.[F]	N-Term(iTRAQ4plex); K5(iTRAQ4	2	2
[R].rGHLFLQTDQPIYNPGQR.[V]	N-Term(iTRAQ4plex)	2	2
[R].rGHLFLQTDQPIYNPGQR.[V]	N-Term(iTRAQ4plex)	2	2
[K].scGLHQLLR.[G]	N-Term(iTRAQ4plex); C2(Carbam	2	2
[K].scGLHQLLR.[G]	N-Term(iTRAQ4plex); C2(Carbam	2	2
[K].scGLHQLLR.[G]	N-Term(iTRAQ4plex); C2(Carbam	2	2
[K].dDPDAPLQPVTPLQLFEGR.[R]	N-Term(iTRAQ4plex)	2	2
[K].dDPDAPLQPVTPLQLFEGR.[R]	N-Term(iTRAQ4plex)	2	2
[R].ecVGFEAVQEVPVGLVQPASATLYDYYNPE	N-Term(iTRAQ4plex); C2(Carbam	2	2
[R].ecVGFEAVQEVPVGLVQPASATLYDYYNPE	N-Term(iTRAQ4plex); C2(Carbam	2	2
[K].sHALQLNNR.[Q]	N-Term(iTRAQ4plex)	2	2
[K].lVNGQSHISLSk.[A]	N-Term(iTRAQ4plex); K12(iTRAQ	2	2
[K].yVLPNFEVk.[I]	N-Term(iTRAQ4plex); K9(iTRAQ4	2	2
[R].gPEVQLVAHSPWLk.[D]	N-Term(iTRAQ4plex); K14(iTRAQ	2	2
[R].gPEVQLVAHSPWLk.[D]	N-Term(iTRAQ4plex); K14(iTRAQ	2	2
[R].lLATLcSAEVcQcAEGk.[C]	N-Term(iTRAQ4plex); C6(Carbam	2	2
[R].gcGEQTMIYLAPTLAASR.[Y]	N-Term(iTRAQ4plex); C2(Carbam	2	2
[R].gPEVQLVAHSPWLk.[D]	N-Term(iTRAQ4plex); K14(iTRAQ	2	2
[R].gPEVQLVAHSPWLk.[D]	N-Term(iTRAQ4plex); K14(iTRAQ	2	2
[R].yVSHFETEGPHVLLYFDSVPTSR.[E]	N-Term(iTRAQ4plex)	2	2
[K].mRPSTDTITVMVENSHGLR.[V]	N-Term(iTRAQ4plex)	2	2
[K].mRPSTDTITVMVENSHGLR.[V]	N-Term(iTRAQ4plex)	2	2
[K].mRPSTDTITVMVENSHGLR.[V]	N-Term(iTRAQ4plex)	2	2
[K].mRPSTDTITVMVENSHGLR.[V]	N-Term(iTRAQ4plex)	2	2
[R].yVSHFETEGPHVLLYFDSVPTSR.[E]	N-Term(iTRAQ4plex)	2	2
[R].yVSHFETEGPHVLLYFDSVPTSR.[E]	N-Term(iTRAQ4plex)	2	2
[K].mRPSTDTITVMVENSHGLR.[V]	N-Term(iTRAQ4plex)	2	2
[R].yVSHFETEGPHVLLYFDSVPTSR.[E]	N-Term(iTRAQ4plex)	2	2
[K].kYVLPNFEVk.[I]	N-Term(iTRAQ4plex); K1(iTRAQ4	2	2
[K].mRPSTDTITVMVENSHGLR.[V]	N-Term(iTRAQ4plex)	2	2
[K].kYVLPNFEVk.[I]	N-Term(iTRAQ4plex); K1(iTRAQ4	2	2
[R].gHLFLQTDQPIYNPGQR.[V]	N-Term(iTRAQ4plex)	2	2
[K].kYVLPNFEVk.[I]	N-Term(iTRAQ4plex); K1(iTRAQ4	2	2
[K].kYVLPNFEVk.[I]	N-Term(iTRAQ4plex); K1(iTRAQ4	2	2
[R].gHLFLQTDQPIYNPGQR.[V]	N-Term(iTRAQ4plex)	2	2
[R].gHLFLQTDQPIYNPGQR.[V]	N-Term(iTRAQ4plex)	2	2
[K].gLcVATPVQLR.[V]	N-Term(iTRAQ4plex); C3(Carbam	2	2
[K].gLcVATPVQLR.[V]	N-Term(iTRAQ4plex); C3(Carbam	2	2
[R].vGDTLNLNLR.[A]	N-Term(iTRAQ4plex)	2	2
[K].gLcVATPVQLR.[V]	N-Term(iTRAQ4plex); C3(Carbam	2	2
[K].gLcVATPVQLR.[V]	N-Term(iTRAQ4plex); C3(Carbam	2	2
[K].kYVLPNFEVk.[I]	N-Term(iTRAQ4plex); K1(iTRAQ4	2	2
[R].gHLFLQTDQPIYNPGQR.[V]	N-Term(iTRAQ4plex)	2	2
[R].yVSHFETEGPHVLLYFDSVPTSR.[E]	N-Term(iTRAQ4plex)	2	2
[R].gPEVQLVAHSPWLk.[D]	N-Term(iTRAQ4plex); K14(iTRAQ	2	2
[R].gPEVQLVAHSPWLk.[D]	N-Term(iTRAQ4plex); K14(iTRAQ	2	2
[R].gPEVQLVAHSPWLk.[D]	N-Term(iTRAQ4plex); K14(iTRAQ	2	2
[R].gPEVQLVAHSPWLk.[D]	N-Term(iTRAQ4plex); K14(iTRAQ	2	2
[R].gPEVQLVAHSPWLk.[D]	N-Term(iTRAQ4plex); K14(iTRAQ	2	2
[R].gPEVQLVAHSPWLk.[D]	N-Term(iTRAQ4plex); K14(iTRAQ	2	2
[R].gPEVQLVAHSPWLk.[D]	N-Term(iTRAQ4plex); K14(iTRAQ	2	2

[R].GPEVQLVAHSPWLk.[D]	K14(iTRAQ4plex)	2	2
[K].eVYMPSSIFQDDFVIPDISEPGTWk.[I]	N-Term(iTRAQ4plex); K25(iTRAQ	2	2
[K].eVYMPSSIFQDDFVIPDISEPGTWk.[I]	N-Term(iTRAQ4plex); K25(iTRAQ	2	2
[K].aEmADQAAAWLTR.[Q]	N-Term(iTRAQ4plex); M3(Oxidat	1	1
[R].gHLFLQTDQPIYNPGQR.[V]	N-Term(iTRAQ4plex)	2	2
[K].sHALQLNNR.[Q]	N-Term(iTRAQ4plex)	2	2
[K].aEmADQAAAWLTR.[Q]	N-Term(iTRAQ4plex); M3(Oxidat	1	1
[K].aDGSYAAWLSR.[DG]	N-Term(iTRAQ4plex)	2	2
[R].vGDTLNLNLR.[A]	N-Term(iTRAQ4plex)	2	2
[R].vGDTLNLNLR.[A]	N-Term(iTRAQ4plex)	2	2
[R].ecVGFEAVQEVPVGLVQPASATLYDYYNPE	N-Term(iTRAQ4plex); C2(Carbam	2	2
[R].ecVGFEAVQEVPVGLVQPASATLYDYYNPE	N-Term(iTRAQ4plex); C2(Carbam	2	2
[R].vTASDPLDTLGSEGALSPGGVASLLR.[L]	N-Term(iTRAQ4plex)	2	2
[R].sTQDTVIALDALSAYWIASHTTEER.[G]	N-Term(iTRAQ4plex)	2	2
[R].STQDTVIALDALSAYWIASHTTEER.[G]		2	2
[K].eVYmPSSIFQDDFVIPDISEPGTWk.[I]	N-Term(iTRAQ4plex); M4(Oxidat	2	2
[R].lLATLcSAEVcQcAEGk.[C]	N-Term(iTRAQ4plex); C6(Carbam	2	2
[R].VTASDPLDTLGSEGALSPGGVASLLR.[L]		2	2
[R].vTASDPLDTLGSEGALSPGGVASLLR.[L]	N-Term(iTRAQ4plex)	2	2
[R].LLATLcSAEVcQcAEGk.[C]	C6(Carbamidomethyl); C11(Carb	2	2
[R].kADGSYAAWLSR.[DG]	N-Term(iTRAQ4plex); K1(iTRAQ4	2	2
[R].kADGSYAAWLSR.[DG]	N-Term(iTRAQ4plex); K1(iTRAQ4	2	2
[K].scGLHQLLR.[G]	N-Term(iTRAQ4plex); C2(Carbam	2	2
[R].vFALDQk.[M]	N-Term(iTRAQ4plex); K7(iTRAQ4	2	2
[R].kADGSYAAWLSR.[DG]	N-Term(iTRAQ4plex); K1(iTRAQ4	2	2
[R].kADGSYAAWLSR.[DG]	N-Term(iTRAQ4plex); K1(iTRAQ4	2	2
[R].kADGSYAAWLSR.[DG]	N-Term(iTRAQ4plex); K1(iTRAQ4	2	2
[K].vVEEQESR.[V]	N-Term(iTRAQ4plex)	2	2
[K].vLSLAQEQVGGSPEk.[L]	N-Term(iTRAQ4plex); K15(iTRAQ	2	2
[R].eAPkVVEEQESR.[V]	N-Term(iTRAQ4plex); K4(iTRAQ4	2	2
[R].aAcAQLNDFLQEYGTQGcQV.[-]	N-Term(iTRAQ4plex); C3(Carbam	2	2
[R].aAcAQLNDFLQEYGTQGcQV.[-]	N-Term(iTRAQ4plex); C3(Carbam	2	2
[K].aEMADQAAAWLTR.[Q]	N-Term(iTRAQ4plex)	1	1
[K].aEMADQAAAWLTR.[Q]	N-Term(iTRAQ4plex)	1	1
[R].lLATLcSAEVcQcAEGk.[C]	N-Term(iTRAQ4plex); C6(Carbam	2	2
[R].lLATLcSAEVcQcAEGk.[C]	N-Term(iTRAQ4plex); C6(Carbam	2	2
[R].lLATLcSAEVcQcAEGk.[C]	N-Term(iTRAQ4plex); C6(Carbam	2	2
[R].lLATLcSAEVcQcAEGk.[C]	N-Term(iTRAQ4plex); C6(Carbam	2	2
[R].ePFLSccQFAESLR.[K]	N-Term(iTRAQ4plex); C6(Carbam	2	2
[R].ePFLSccQFAESLR.[K]	N-Term(iTRAQ4plex); C6(Carbam	2	2
[R].ePFLSccQFAESLR.[K]	N-Term(iTRAQ4plex); C6(Carbam	2	2
[R].aVGSGATFSHYYYMILSR.[G]	N-Term(iTRAQ4plex)	2	2
[K].aSAGLLGAHAAAITAYALTLTkAPADLR.[G]	N-Term(iTRAQ4plex); K22(iTRAQ	1	1
[K].aEFQDALEk.[L]	N-Term(iTRAQ4plex); K9(iTRAQ4	2	2
[R].tTNIQGINLLFSSR.[R]	N-Term(iTRAQ4plex)	2	2
[R].tTNIQGINLLFSSR.[R]	N-Term(iTRAQ4plex)	2	2
[K].tEQWSTLPPETk.[D]	N-Term(iTRAQ4plex); K12(iTRAQ	2	2
[K].tEQWSTLPPETk.[D]	N-Term(iTRAQ4plex); K12(iTRAQ	2	2
[K].tEQWSTLPPETk.[D]	N-Term(iTRAQ4plex); K12(iTRAQ	2	2
[K].tEQWSTLPPETk.[D]	N-Term(iTRAQ4plex); K12(iTRAQ	2	2
[R].TTNIQGINLLFSSR.[R]		2	2
[K].tEQWSTLPPETk.[D]	N-Term(iTRAQ4plex); K12(iTRAQ	2	2

[K].tEQWSTLPPETk.[D]	N-Term(iTRAQ4plex); K12(iTRAQ	2	2
[K].TEQWSTLPPETk.[D]	K12(iTRAQ4plex)	2	2
[K].tEQWSTLPPETk.[D]	N-Term(iTRAQ4plex); K12(iTRAQ	2	2
[K].tEQWSTLPPETk.[D]	N-Term(iTRAQ4plex); K12(iTRAQ	2	2
[R].dFALLSLQVPLk.[D]	N-Term(iTRAQ4plex); K12(iTRAQ	2	2
[R].aLEILQEEDLIDEDDIPVR.[S]	N-Term(iTRAQ4plex)	2	2
[K].sHkPLNMGk.[V]	N-Term(iTRAQ4plex); K3(iTRAQ4	2	2
[K].lNMGITDLQGLR.[L]	N-Term(iTRAQ4plex)	2	2
[K].lNMGITDLQGLR.[L]	N-Term(iTRAQ4plex)	2	2
[R].EELVYELNPLDHR.[G]		2	2
[K].vGLSGmAIADVTLLSGFHALR.[A]	N-Term(iTRAQ4plex); M6(Oxidat	2	2
[R].rFEQLELRPVLYNYLDk.[N]	N-Term(iTRAQ4plex); K17(iTRAQ	2	2
[R].hLVPGAPFLLQALVR.[E]	N-Term(iTRAQ4plex)	2	2
[K].aSAGLLGAHAAAITAYALTLTk.[A]	N-Term(iTRAQ4plex); K22(iTRAQ	2	2
[K].aSAGLLGAHAAAITAYALTLTk.[A]	N-Term(iTRAQ4plex); K22(iTRAQ	2	2
[K].lQETSNWLLSQQQADGSFQDLSPVIHR.[S]	N-Term(iTRAQ4plex)	1	1
[R].vDVQAGAcEGk.[L]	N-Term(iTRAQ4plex); C8(Carbam	2	2
[R].vDVQAGAcEGk.[L]	N-Term(iTRAQ4plex); C8(Carbam	2	2
[R].vDVQAGAcEGk.[L]	N-Term(iTRAQ4plex); C8(Carbam	2	2
[K].lQETSNWLLSQQQADGSFQDLSPVIHR.[S]	N-Term(iTRAQ4plex)	1	1
[K].iTPGkPYILTVPGHLDEMQLDIQAR.[Y]	N-Term(iTRAQ4plex); K5(iTRAQ4	2	2
[K].dHAVDLIQk.[G]	N-Term(iTRAQ4plex); K9(iTRAQ4	2	2
[R].tTNIQGINLLFSSR.[R]	N-Term(iTRAQ4plex)	2	2
[K].dHAVDLIQk.[G]	N-Term(iTRAQ4plex); K9(iTRAQ4	2	2
[K].dHAVDLIQk.[G]	N-Term(iTRAQ4plex); K9(iTRAQ4	2	2
[K].iTPGkPYILTVPGHLDEMQLDIQAR.[Y]	N-Term(iTRAQ4plex); K5(iTRAQ4	2	2
[K].dHAVDLIQk.[G]	N-Term(iTRAQ4plex); K9(iTRAQ4	2	2
[K].dHAVDLIQk.[G]	N-Term(iTRAQ4plex); K9(iTRAQ4	2	2
[K].lNMGITDLQGLR.[L]	N-Term(iTRAQ4plex)	2	2
[R].fGLLDEDGk.[K]	N-Term(iTRAQ4plex); K9(iTRAQ4	2	2
[R].VDVQAGAcEGk.[L]	C8(Carbamidomethyl); K11(iTRA	2	2
[K].VLSLAQEQVGGSPEk.[L]	K15(iTRAQ4plex)	2	2
[R].gLQDEDGYR.[M]	N-Term(iTRAQ4plex)	2	2
[R].gLQDEDGYR.[M]	N-Term(iTRAQ4plex)	2	2
[R].DFALLSLQVPLk.[D]	K12(iTRAQ4plex)	2	2
[R].LTVAAPPSGGPGFLSIERPDSRPPR.[V]		2	2
[R].gLQDEDGYR.[M]	N-Term(iTRAQ4plex)	2	2
[R].gLQDEDGYR.[M]	N-Term(iTRAQ4plex)	2	2
[R].eMSGSPASGIPVk.[V]	N-Term(iTRAQ4plex); K13(iTRAQ	2	2
[R].fGLLDEDGkk.[T]	N-Term(iTRAQ4plex); K9(iTRAQ4	2	2
[R].fGLLDEDGkk.[T]	N-Term(iTRAQ4plex); K9(iTRAQ4	2	2
[R].fGLLDEDGk.[K]	N-Term(iTRAQ4plex); K9(iTRAQ4	2	2
[R].gLQDEDGYR.[M]	N-Term(iTRAQ4plex)	2	2
[R].gLQDEDGYR.[M]	N-Term(iTRAQ4plex)	2	2
[R].gLQDEDGYR.[M]	N-Term(iTRAQ4plex)	2	2
[R].gLQDEDGYR.[M]	N-Term(iTRAQ4plex)	2	2
[R].tYNVLDMk.[N]	N-Term(iTRAQ4plex); K8(iTRAQ4	2	2
[K].LHLETDSLALVALGALDTALYAAGSk.[S]	K26(iTRAQ4plex)	2	2
[K].lHLETDSLALVALGALDTALYAAGSk.[S]	N-Term(iTRAQ4plex); K26(iTRAQ	2	2
[R].lTVAAPPSGGPGFLSIERPDSRPPR.[V]	N-Term(iTRAQ4plex)	2	2
[R].lTVAAPPSGGPGFLSIERPDSRPPR.[V]	N-Term(iTRAQ4plex)	2	2
[R].lTVAAPPSGGPGFLSIERPDSRPPR.[V]	N-Term(iTRAQ4plex)	2	2

[R].fQILTLWLPDSLTTWEIHGLSLSk.[T]	N-Term(iTRAQ4plex); K24(iTRAQ	2	2
[R].fQILTLWLPDSLTTWEIHGLSLSk.[T]	N-Term(iTRAQ4plex); K24(iTRAQ	2	2
[K].tEQWSTLPPETk.[D]	N-Term(iTRAQ4plex); K12(iTRAQ	2	2
[R].sMQGGLVGNDETVALTAFVTIALHHGLAV	N-Term(iTRAQ4plex); K39(iTRAQ	2	2
[K].tEQWSTLPPETk.[D]	N-Term(iTRAQ4plex); K12(iTRAQ	2	2
[K].tEQWSTLPPETk.[D]	N-Term(iTRAQ4plex); K12(iTRAQ	2	2
[K].tEQWSTLPPETk.[D]	N-Term(iTRAQ4plex); K12(iTRAQ	2	2
[R].sMQGGLVGNDETVALTAFVTIALHHGLAV	N-Term(iTRAQ4plex); K39(iTRAQ	2	2
[K].tEQWSTLPPETk.[D]	N-Term(iTRAQ4plex); K12(iTRAQ	2	2
[K].tEQWSTLPPETk.[D]	N-Term(iTRAQ4plex); K12(iTRAQ	2	2
[K].lELSVDGAk.[Q]	N-Term(iTRAQ4plex); K9(iTRAQ4	2	2
[K].lGQYASPTAk.[R]	N-Term(iTRAQ4plex); K10(iTRAQ	2	2
[R].sTQDTVIALDALSAYWIASHTTEER.[G]	N-Term(iTRAQ4plex)	2	2
[K].vGLSGMAIADVTLLSGFHALR.[A]	N-Term(iTRAQ4plex)	2	2
[R].sFFPENWLWR.[V]	N-Term(iTRAQ4plex)	2	2
[R].SFFPENWLWR.[V]		2	2
[K].vGLSGMAIADVTLLSGFHALR.[A]	N-Term(iTRAQ4plex)	2	2
[K].aEmADQAAAWLTR.[Q]	N-Term(iTRAQ4plex); M3(Oxidat	1	1
[R].FGLLDEDGkk.[T]	K9(iTRAQ4plex); K10(iTRAQ4ple	2	2
[R].vTASDPLDTLGSEGALSPGGVASLLR.[L]	N-Term(iTRAQ4plex)	2	2
[R].lLLFSPSVVHLGVPLSVGVQLQDVPR.[G]	N-Term(iTRAQ4plex)	2	2
[R].lLLFSPSVVHLGVPLSVGVQLQDVPR.[G]	N-Term(iTRAQ4plex)	2	2
[R].hLVPGAPFLLQALVR.[E]	N-Term(iTRAQ4plex)	2	2
[R].dkGQAGLQR.[A]	N-Term(iTRAQ4plex); K2(iTRAQ4	2	2
[K].sHALQLNNR.[Q]	N-Term(iTRAQ4plex)	2	2
[R].cSVFYGAPSk.[S]	N-Term(iTRAQ4plex); C1(Carbam	2	2
[K].sHkPLNmGk.[V]	N-Term(iTRAQ4plex); K3(iTRAQ4	2	2
[K].sHKPLNMGk.[V]	N-Term(iTRAQ4plex); K9(iTRAQ4	2	2
[K].tEQWSTLPPETk.[D]	N-Term(iTRAQ4plex); K12(iTRAQ	2	2
[R].gPEVQLVAHSPWLk.[D]	N-Term(iTRAQ4plex); K14(iTRAQ	2	2
[R].gLQDEDGYR.[M]	N-Term(iTRAQ4plex)	2	2
[K].sHALQLNNR.[Q]	N-Term(iTRAQ4plex)	2	2
[K].LQETSNWLLSQQQADGSFQDLSPVIHR.[S		1	1
[R].eELVYELNPLDHR.[G]	N-Term(iTRAQ4plex)	2	2
[R].vEYGFQVk.[V]	N-Term(iTRAQ4plex); K8(iTRAQ4	2	2
[R].lTVAAPPSGGPGFLSIERPDSRPPR.[V]	N-Term(iTRAQ4plex)	2	2
[R].gPEVQLVAHSPWLk.[D]	N-Term(iTRAQ4plex); K14(iTRAQ	2	2
[R].gLQDEDGYR.[M]	N-Term(iTRAQ4plex)	2	2
[K].TEQWSTLPPETk.[D]	K12(iTRAQ4plex)	2	2
[R].cSVFYGAPSk.[S]	N-Term(iTRAQ4plex); C1(Carbam	2	2
[K].lGQYASPTAk.[R]	N-Term(iTRAQ4plex); K10(iTRAQ	2	2
[K].aEFQDALEk.[L]	N-Term(iTRAQ4plex); K9(iTRAQ4	2	2
[K].TEQWSTLPPETk.[D]	K12(iTRAQ4plex)	2	2
[K].scGLHQLLR.[G]	N-Term(iTRAQ4plex); C2(Carbam	2	2
[R].tLEIPGNSDPNMIPDGDFNSYVR.[V]	N-Term(iTRAQ4plex)	2	2
[R].tYNVLDMk.[N]	N-Term(iTRAQ4plex); K8(iTRAQ4	2	2
[K].tEQWSTLPPETk.[D]	N-Term(iTRAQ4plex); K12(iTRAQ	2	2
[K].tEQWSTLPPETk.[D]	N-Term(iTRAQ4plex); K12(iTRAQ	2	2
[K].vLQIEkEGAIHR.[E]	N-Term(iTRAQ4plex); K6(iTRAQ4	2	2
[K].ScGLHQLLR.[G]	C2(Carbamidomethyl)	2	2
[R].nNVPcSPk.[V]	N-Term(iTRAQ4plex); C5(Carbam	2	2
[R].GHLFLQTDQPIYNPGQR.[V]		2	2

[R].tTNIQGINLLFSSR.[R]	N-Term(iTRAQ4plex)	2	2
[R].kADGSYAAWLSR.[DG]	N-Term(iTRAQ4plex); K1(iTRAQ4	2	2
[R].vHYTVcIWR.[N]	N-Term(iTRAQ4plex); C6(Carbam	2	2
[R].GHLFLQTDQPIYNPGQR.[V]		2	2
[K].vDFTLSSER.[D]	N-Term(iTRAQ4plex)	2	2
[K].YVLPNFEVk.[I]	K9(iTRAQ4plex)	2	2
[R].RGHLFLQTDQPIYNPGQR.[V]		2	2
[R].gLQDEDGYR.[M]	N-Term(iTRAQ4plex)	2	2
[K].kYVLPNFEVk.[I]	N-Term(iTRAQ4plex); K1(iTRAQ4	2	2
[K].scGLHQLLR.[G]	N-Term(iTRAQ4plex); C2(Carbam	2	2
[K].sHALQLNNR.[Q]	N-Term(iTRAQ4plex)	2	2
[R].kADGSYAAWLSR.[DG]	N-Term(iTRAQ4plex); K1(iTRAQ4	2	2
[K].aEmADQAAAWLTR.[Q]	N-Term(iTRAQ4plex); M3(Oxidat	1	1
[K].yVLPNFEVk.[I]	N-Term(iTRAQ4plex); K9(iTRAQ4	2	2
[K].tEQWSTLPPETk.[D]	N-Term(iTRAQ4plex); K12(iTRAQ	2	2
[K].LVNGQSHISLSk.[A]	K12(iTRAQ4plex)	2	2
[K].TEQWSTLPPETk.[D]	K12(iTRAQ4plex)	2	2
[K].dHAVDLIQkGYMR.[I]	N-Term(iTRAQ4plex); K9(iTRAQ4	2	2
[R].lTVAAPPSGGPGFLSIERPDSRPPR.[V]	N-Term(iTRAQ4plex)	2	2
[K].vLSLAQEQVGGSPEk.[L]	N-Term(iTRAQ4plex); K15(iTRAQ	2	2
[K].ITQVLHFTk.[D]	K9(iTRAQ4plex)	2	2
[K].LGQYASPTAk.[R]	K10(iTRAQ4plex)	2	2
[R].QGSFQGGFR.[S]		2	2
[R].EELVYELNPLDHR.[G]		2	2
[R].RGHLFLQTDQPIYNPGQR.[V]		2	2
[R].EELVYELNPLDHR.[G]		2	2
[R].yRVFALDQk.[M]	N-Term(iTRAQ4plex); K9(iTRAQ4	2	2
[K].yVLPNFEVk.[I]	N-Term(iTRAQ4plex); K9(iTRAQ4	2	2
[R].yLDkTEQWSTLPPETk.[D]	N-Term(iTRAQ4plex); K4(iTRAQ4	2	2
[R].gSFEFPVGDAVSk.[V]	N-Term(iTRAQ4plex); K13(iTRAQ	2	2
[R].nNVPcSPk.[V]	N-Term(iTRAQ4plex); C5(Carbam	2	2
[R].VHYTVcIWR.[N]	C6(Carbamidomethyl)	2	2
[R].vEASISk.[A]	N-Term(iTRAQ4plex); K7(iTRAQ4	2	2
[R].nNVPcSPk.[V]	N-Term(iTRAQ4plex); C5(Carbam	2	2
[R].vEASISk.[A]	N-Term(iTRAQ4plex); K7(iTRAQ4	2	2
[R].tYNVLDMk.[N]	N-Term(iTRAQ4plex); K8(iTRAQ4	2	2
[K].scGLHQLLR.[G]	N-Term(iTRAQ4plex); C2(Carbam	2	2
[R].dkGQAGLQR.[A]	N-Term(iTRAQ4plex); K2(iTRAQ4	2	2
[R].tLEIPGNSDPNMIPDGDFNSYVR.[V]	N-Term(iTRAQ4plex)	2	2
[R].tLEIPGNSDPNMIPDGDFNSYVR.[V]	N-Term(iTRAQ4plex)	2	2
[K].yVLPNFEVk.[I]	N-Term(iTRAQ4plex); K9(iTRAQ4	2	2
[K].LNMGITDLQGLR.[L]		2	2
[K].lGQYASPTAk.[R]	N-Term(iTRAQ4plex); K10(iTRAQ	2	2
[R].VEASISk.[A]	K7(iTRAQ4plex)	2	2
[K].AEMADQAAAWLTR.[Q]		1	1
[R].gLEEELQFSLGSk.[I]	N-Term(iTRAQ4plex); K13(iTRAQ	2	2
[R].gLQDEDGYR.[M]	N-Term(iTRAQ4plex)	2	2
[R].cSVFYGAPSkSR.[L]	C1(Carbamidomethyl); K10(iTRA	2	2
[K].TEQWSTLPPETk.[D]	K12(iTRAQ4plex)	2	2
[R].yLDkTEQWSTLPPETk.[D]	N-Term(iTRAQ4plex); K4(iTRAQ4	2	2
[R].kADGSYAAWLSR.[DG]	N-Term(iTRAQ4plex); K1(iTRAQ4	2	2
[K].tkGLcVATPVQLR.[V]	N-Term(iTRAQ4plex); K2(iTRAQ4	2	2

[K].lELSVDGAk.[Q]	N-Term(iTRAQ4plex); K9(iTRAQ4	2	2
[R].lTVAAPPSGGPGFLSIERPDSRPPR.[V]	N-Term(iTRAQ4plex)	2	2
[K].VDFTLSSER.[D]		2	2
[K].kYVLPNFEVk.[I]	K1(iTRAQ4plex); K10(iTRAQ4plex	2	2
[K].kYVLPNFEVk.[I]	N-Term(iTRAQ4plex); K10(iTRAQ4	2	2
[K].lQETSNWLLSQQQADGSFQDLSPVIHR.[S]	N-Term(iTRAQ4plex)	1	1
[K].kYVLPNFEVk.[I]	N-Term(iTRAQ4plex); K1(iTRAQ4	2	2
[R].tYNVLDMk.[N]	N-Term(iTRAQ4plex); K8(iTRAQ4	2	2
[R].fGLLDEDGkk.[T]	N-Term(iTRAQ4plex); K9(iTRAQ4	2	2
[R].eFHLHLR.[L]	N-Term(iTRAQ4plex)	2	2
[R].RGHLFLQTDQPIYNPGQR.[V]		2	2
[R].GLQDEDGYR.[M]		2	2
[K].vLSLAQEQVGGSPEk.[L]	N-Term(iTRAQ4plex); K15(iTRAQ4	2	2
[R].YIYGkPVQGVAYVR.[F]	K5(iTRAQ4plex)	2	2
[R].vEASISk.[A]	N-Term(iTRAQ4plex); K7(iTRAQ4	2	2
[R].vEYGFQVk.[V]	N-Term(iTRAQ4plex); K8(iTRAQ4	2	2
[R].vFALDQk.[M]	N-Term(iTRAQ4plex); K7(iTRAQ4	2	2
[R].VHYTVcIWR.[N]	C6(Carbamidomethyl)	2	2
[R].vFALDQk.[M]	N-Term(iTRAQ4plex); K7(iTRAQ4	2	2
[R].nNVPcSPk.[V]	N-Term(iTRAQ4plex); C5(Carbam	2	2
[R].dkGQAGLQR.[A]	N-Term(iTRAQ4plex); K2(iTRAQ4	2	2
[K].sHkPLNMGk.[V]	N-Term(iTRAQ4plex); K3(iTRAQ4	2	2
[R].gLQDEDGYR.[M]	N-Term(iTRAQ4plex)	2	2
[R].vEYGFQVk.[V]	N-Term(iTRAQ4plex); K8(iTRAQ4	2	2
[R].cSVFYGAPSk.[S]	C1(Carbamidomethyl); K10(iTRA	2	2
[K].vVEEQESR.[V]	N-Term(iTRAQ4plex)	2	2
[K].GLcVATPVQLR.[V]	C3(Carbamidomethyl)	2	2
[R].tLEIPGNSDPNmIPDGDFNSYVR.[V]	N-Term(iTRAQ4plex); M12(Oxida	2	2
[K].vLSLAQEQVGGSPEk.[L]	N-Term(iTRAQ4plex); K15(iTRAQ4	2	2
[R].eELVYELNPLDHR.[G]	N-Term(iTRAQ4plex)	2	2
[R].QGSFQGGFR.[S]		2	2
[K].tEQWSTLPPETk.[D]	N-Term(iTRAQ4plex); K12(iTRAQ4	2	2
[R].vEASISk.[A]	N-Term(iTRAQ4plex); K7(iTRAQ4	2	2
[K].ADGSYAAWLSR.[DG]		2	2
[R].gLQDEDGYR.[M]	N-Term(iTRAQ4plex)	2	2
[R].GLQDEDGYR.[M]		2	2
[R].gQVVkGSVFLR.[N]	N-Term(iTRAQ4plex); K5(iTRAQ4	2	2
[R].yLDkTEQWSTLPPETk.[D]	N-Term(iTRAQ4plex); K4(iTRAQ4	2	2
[R].dFALLSLQVPLkDAk.[S]	N-Term(iTRAQ4plex); K12(iTRAQ4	2	2
[K].vVEEQESR.[V]	N-Term(iTRAQ4plex)	2	2
[R].aAcAQLNDFLQEYGTQGcQV.[-]	N-Term(iTRAQ4plex); C3(Carbam	2	2
[K].vVEEQESR.[V]	N-Term(iTRAQ4plex)	2	2
[R].eMSGSPASGIPVk.[V]	N-Term(iTRAQ4plex); K13(iTRAQ4	2	2
[K].FAcYYPR.[V]	C3(Carbamidomethyl)	2	2
[R].vFALDQk.[M]	N-Term(iTRAQ4plex); K7(iTRAQ4	2	2
[K].vVEEQESR.[V]	N-Term(iTRAQ4plex)	2	2
[R].QGSFQGGFR.[S]		2	2
[K].vVEEQESR.[V]	N-Term(iTRAQ4plex)	2	2
[K].iTQVLHFTk.[D]	N-Term(iTRAQ4plex); K9(iTRAQ4	2	2
[R].kADGSYAAWLSR.[DG]	N-Term(iTRAQ4plex); K1(iTRAQ4	2	2
[R].vFALDQk.[M]	N-Term(iTRAQ4plex); K7(iTRAQ4	2	2
[K].qYRNGESVk.[L]	N-Term(iTRAQ4plex); K9(iTRAQ4	2	2

[R].VFALDQk.[M]	K7(iTRAQ4plex)	2	2
[R].gLESQTk.[L]	N-Term(iTRAQ4plex); K7(iTRAQ4	2	2
[R].dkGQAGLQR.[A]	N-Term(iTRAQ4plex); K2(iTRAQ4	2	2
[K].iTQVLHFTk.[D]	N-Term(iTRAQ4plex); K9(iTRAQ4	2	2
[R].gQIVFmNR.[E]	N-Term(iTRAQ4plex); M6(Oxidat	2	2
[R].GQIVFMNR.[E]		2	2
[K].sHALQLNNR.[Q]	N-Term(iTRAQ4plex)	2	2
[R].GSFEFPVGDAVSk.[V]	K13(iTRAQ4plex)	2	2
[R].VGDTLNLNLR.[A]		2	2
[R].YIYGkPVQGVAYVR.[F]	K5(iTRAQ4plex)	2	2
[R].gLQDEDGYR.[M]	N-Term(iTRAQ4plex)	2	2
[R].dFALLSLQVPLk.[D]	N-Term(iTRAQ4plex); K12(iTRAQ	2	2
[R].gPEVQLVAHSPWLk.[D]	N-Term(iTRAQ4plex); K14(iTRAQ	2	2
[K].iTQVLHFTk.[D]	N-Term(iTRAQ4plex); K9(iTRAQ4	2	2
[R].gLESQTk.[L]	N-Term(iTRAQ4plex); K7(iTRAQ4	2	2
[K].aDGSYAAWLSR.[DG]	N-Term(iTRAQ4plex)	2	2
[R].yIYGkPVQGVAYVR.[F]	N-Term(iTRAQ4plex); K5(iTRAQ4	2	2
[K].vVEEQESR.[V]	N-Term(iTRAQ4plex)	2	2
[K].vDFTLSSER.[D]	N-Term(iTRAQ4plex)	2	2
[R].EELVYELNPLDHR.[G]		2	2
[K].lELSVDGAk.[Q]	N-Term(iTRAQ4plex); K9(iTRAQ4	2	2
[R].RGHLFLQTDQPIYNPGQR.[V]		2	2
[R].rFEQLELRPVLYNYLDk.[N]	N-Term(iTRAQ4plex); K17(iTRAQ	2	2
[R].dkGQAGLQR.[A]	N-Term(iTRAQ4plex); K2(iTRAQ4	2	2
[K].iTQVLHFTk.[D]	N-Term(iTRAQ4plex); K9(iTRAQ4	2	2
[R].eFHLHLR.[L]	N-Term(iTRAQ4plex)	2	2
[R].vEYGFQVk.[V]	N-Term(iTRAQ4plex); K8(iTRAQ4	2	2
[R].gLEEELQFSLGSk.[I]	N-Term(iTRAQ4plex); K13(iTRAQ	2	2
[R].tLEIPGNSDPNMIPDGDFNSYVR.[V]	N-Term(iTRAQ4plex)	2	2
[R].dkGQAGLQR.[A]	N-Term(iTRAQ4plex); K2(iTRAQ4	2	2
[K].sHkPLNmGk.[V]	N-Term(iTRAQ4plex); K3(iTRAQ4	2	2
[K].mRPSTDTITVMVENSHGLR.[V]	N-Term(iTRAQ4plex); M1(Oxidat	2	2
[R].gLESQTk.[L]	N-Term(iTRAQ4plex); K7(iTRAQ4	2	2
[K].TEQWSTLPPETk.[D]	K12(iTRAQ4plex)	2	2
[R].GPEVQLVAHSPWLk.[D]	K14(iTRAQ4plex)	2	2
[R].vEYGFQVk.[V]	N-Term(iTRAQ4plex); K8(iTRAQ4	2	2
[K].ScGLHQLLR.[G]	C2(Carbamidomethyl)	2	2
[R].gHLFLQTDQPIYNPGQR.[V]	N-Term(iTRAQ4plex)	2	2
[R].ePFLSccQFAESLR.[K]	N-Term(iTRAQ4plex); C6(Carbam	2	2
[R].gQIVFmNR.[E]	N-Term(iTRAQ4plex); M6(Oxidat	2	2
[R].eFHLHLR.[L]	N-Term(iTRAQ4plex)	2	2
[K].tEQWSTLPPETk.[D]	N-Term(iTRAQ4plex); K12(iTRAQ	2	2
[K].vLQIEkEGAIHR.[E]	N-Term(iTRAQ4plex); K6(iTRAQ4	2	2
[R].eELVYELNPLDHR.[G]	N-Term(iTRAQ4plex)	2	2
[R].cSVFYGAPSk.[S]	C1(Carbamidomethyl); K10(iTRA	2	2
[K].LNMGITDLQGLR.[L]		2	2
[R].VDVQAGAcEGk.[L]	C8(Carbamidomethyl); K11(iTRA	2	2
[K].vTSIQHWVQk.[T]	N-Term(iTRAQ4plex); K10(iTRAQ	1	1
[K].scAVAEYGVYVk.[V]	N-Term(iTRAQ4plex); C2(Carbam	2	2
[R].iLGGHLDAk.[G]	N-Term(iTRAQ4plex); K9(iTRAQ4	2	2
[R].iLGGHLDAk.[G]	N-Term(iTRAQ4plex); K9(iTRAQ4	2	2
[R].iLGGHLDAk.[G]	N-Term(iTRAQ4plex); K9(iTRAQ4	2	2

[K].vVLHPNYHQVDIGLIk.[L]	N-Term(iTRAQ4plex); K16(iTRAQ	1	1
[R].iLGGHLDAk.[G]	N-Term(iTRAQ4plex); K9(iTRAQ4	2	2
[K].vTSIQHWVQk.[T]	N-Term(iTRAQ4plex); K10(iTRAQ	1	1
[K].dIAPTLTLYVGk.[K]	N-Term(iTRAQ4plex); K12(iTRAQ	2	2
[K].dIAPTLTLYVGk.[K]	N-Term(iTRAQ4plex); K12(iTRAQ	2	2
[K].DIAPTLTLYVGk.[K]	K12(iTRAQ4plex)	2	2
[K].dIAPTLTLYVGk.[K]	N-Term(iTRAQ4plex); K12(iTRAQ	2	2
[K].dIAPTLTLYVGk.[K]	N-Term(iTRAQ4plex); K12(iTRAQ	2	2
[R].tEGDGVYTLNDk.[K]	N-Term(iTRAQ4plex); K12(iTRAQ	2	2
[R].tEGDGVYTLNDk.[K]	N-Term(iTRAQ4plex); K12(iTRAQ	2	2
[R].tEGDGVYTLNDk.[K]	N-Term(iTRAQ4plex); K12(iTRAQ	2	2
[K].scAVAEYGVYVk.[V]	N-Term(iTRAQ4plex); C2(Carbam	2	2
[K].scAVAEYGVYVk.[V]	N-Term(iTRAQ4plex); C2(Carbam	2	2
[K].scAVAEYGVYVk.[V]	N-Term(iTRAQ4plex); C2(Carbam	2	2
[K].scAVAEYGVYVk.[V]	N-Term(iTRAQ4plex); C2(Carbam	2	2
[K].scAVAEYGVYVk.[V]	N-Term(iTRAQ4plex); C2(Carbam	2	2
[K].scAVAEYGVYVk.[V]	N-Term(iTRAQ4plex); C2(Carbam	2	2
[K].scAVAEYGVYVk.[V]	N-Term(iTRAQ4plex); C2(Carbam	2	2
[K].scAVAEYGVYVk.[V]	N-Term(iTRAQ4plex); C2(Carbam	2	2
[K].scAVAEYGVYVk.[V]	N-Term(iTRAQ4plex); C2(Carbam	2	2
[K].scAVAEYGVYVk.[V]	N-Term(iTRAQ4plex); C2(Carbam	2	2
[K].scAVAEYGVYVk.[V]	N-Term(iTRAQ4plex); C2(Carbam	2	2
[R].vMPIcLPSk.[ND]	N-Term(iTRAQ4plex); C5(Carbam	2	2
[K].lPEcEAVcGkPk.[N]	N-Term(iTRAQ4plex); C4(Carbam	2	2
[K].lPEcEAVcGkPk.[N]	N-Term(iTRAQ4plex); C4(Carbam	2	2
[K].kQLVEIEk.[V]	N-Term(iTRAQ4plex); K1(iTRAQ4	2	2
[K].dIAPTLTLYVGkk.[Q]	N-Term(iTRAQ4plex); K12(iTRAQ	2	2
[R].VMPIcLPSk.[ND]	C5(Carbamidomethyl); K9(iTRAC	2	2
[R].qLFALYSGNDVTDISDDR.[F]	N-Term(iTRAQ4plex)	1	1
[K].scAVAEYGVYVk.[V]	N-Term(iTRAQ4plex); C2(Carbam	2	2
[K].dIAPTLTLYVGk.[K]	N-Term(iTRAQ4plex); K12(iTRAQ	2	2
[K].gSFPWQAk.[M]	N-Term(iTRAQ4plex); K8(iTRAQ4	2	2
[K].gSFPWQAk.[M]	N-Term(iTRAQ4plex); K8(iTRAQ4	2	2
[K].gSFPWQAk.[M]	N-Term(iTRAQ4plex); K8(iTRAQ4	2	2
[K].scAVAEYGVYVk.[V]	N-Term(iTRAQ4plex); C2(Carbam	2	2
[R].vMPIcLPSk.[ND]	N-Term(iTRAQ4plex); C5(Carbam	2	2
[R].VMPIcLPSk.[ND]	C5(Carbamidomethyl); K9(iTRAC	2	2
[R].iLGGHLDAk.[G]	N-Term(iTRAQ4plex); K9(iTRAQ4	2	2
[K].dIAPTLTLYVGk.[K]	N-Term(iTRAQ4plex); K12(iTRAQ	2	2
[R].vMPIcLPSk.[ND]	N-Term(iTRAQ4plex); C5(Carbam	2	2
[R].vmPIcLPSk.[ND]	N-Term(iTRAQ4plex); M2(Oxidat	2	2
[R].iLGGHLDAk.[G]	N-Term(iTRAQ4plex); K9(iTRAQ4	2	2
[K].QLVEIEk.[V]	K7(iTRAQ4plex)	2	2
[R].iLGGHLDAk.[G]	N-Term(iTRAQ4plex); K9(iTRAQ4	2	2
[R].iLGGHLDAk.[G]	N-Term(iTRAQ4plex); K9(iTRAQ4	2	2
[R].TEGDGVYTLNDk.[K]	K12(iTRAQ4plex)	2	2
[K].qLVEIEk.[V]	N-Term(iTRAQ4plex); K7(iTRAQ4	2	2
[R].ILGGHLDAk.[G]	K9(iTRAQ4plex)	2	2
[K].gSFPWQAk.[M]	N-Term(iTRAQ4plex); K8(iTRAQ4	2	2
[R].VMPIcLPSk.[ND]	C5(Carbamidomethyl); K9(iTRAC	2	2
[K].gSFPWQAk.[M]	N-Term(iTRAQ4plex); K8(iTRAQ4	2	2
[K].gSFPWQAk.[M]	N-Term(iTRAQ4plex); K8(iTRAQ4	2	2

[K].vTSIQHWVQk.[T]	N-Term(iTRAQ4plex); K10(iTRAQ	1	1
[K].gSFPWQAk.[M]	N-Term(iTRAQ4plex); K8(iTRAQ4	2	2
[R].tEGDGVYTLNDk.[K]	N-Term(iTRAQ4plex); K12(iTRAQ	2	2
[R].vmPIcLPSk.[ND]	N-Term(iTRAQ4plex); M2(Oxidat	2	2
[K].qLVEIEk.[V]	N-Term(iTRAQ4plex); K7(iTRAQ4	2	2
[K].gPSVFPLAPcSR.[S]	N-Term(iTRAQ4plex); C10(Carbar	3	3
[K].gPSVFPLAPcSR.[S]	N-Term(iTRAQ4plex); C10(Carbar	3	3
[K].wYVDGVEVHNAk.[T]	N-Term(iTRAQ4plex); K12(iTRAQ	1	1
[KR].sTSGGTAALGcLVk.[D]	N-Term(iTRAQ4plex); C11(Carbar	2	2
[K].wYVDGVEVHNAk.[T]	N-Term(iTRAQ4plex); K12(iTRAQ	1	1
[KR].sTSGGTAALGcLVk.[D]	N-Term(iTRAQ4plex); C11(Carbar	2	2
[K].gPSVFPLAPcSR.[S]	N-Term(iTRAQ4plex); C10(Carbar	3	3
[K].nQVSLTcLVk.[G]	N-Term(iTRAQ4plex); C7(Carbam	4	4
[K].nQVSLTcLVk.[G]	N-Term(iTRAQ4plex); C7(Carbam	4	4
[K].tPLGDTTHTcPR.[C]	N-Term(iTRAQ4plex); C10(Carbar	1	1
[K].aLPAPIEk.[T]	N-Term(iTRAQ4plex); K8(iTRAQ4	2	2
[K].scDTPPPcPR.[C]	N-Term(iTRAQ4plex); C2(Carbam	1	1
[R].ePQVYTLPPSR.[DE]	N-Term(iTRAQ4plex)	3	3
[R].ePQVYTLPPSR.[DE]	N-Term(iTRAQ4plex)	3	3
[R].ePQVYTLPPSR.[DE]	N-Term(iTRAQ4plex)	3	3
[R].ePQVYTLPPSR.[DE]	N-Term(iTRAQ4plex)	3	3
[R].ePQVYTLPPSR.[DE]	N-Term(iTRAQ4plex)	3	3
[R].ePQVYTLPPSR.[DE]	N-Term(iTRAQ4plex)	3	3
[R].ePQVYTLPPSR.[DE]	N-Term(iTRAQ4plex)	3	3
[KR].sTSGGTAALGcLVk.[D]	N-Term(iTRAQ4plex); C11(Carbar	2	2
[K].aLPAPIEk.[T]	N-Term(iTRAQ4plex); K8(iTRAQ4	2	2
[K].DTLmISR.[T]	M4(Oxidation)	4	4
[R].ePQVYTLPPSR.[DE]	N-Term(iTRAQ4plex)	3	3
[R].sTSESTAALGcLVk.[D]	N-Term(iTRAQ4plex); C11(Carbar	2	2
[R].sTSESTAALGcLVk.[D]	N-Term(iTRAQ4plex); C11(Carbar	2	2
[K].gPSVFPLAPcSR.[S]	N-Term(iTRAQ4plex); C10(Carbar	3	3
[K].gPSVFPLAPcSR.[S]	N-Term(iTRAQ4plex); C10(Carbar	3	3
[K].tTPPMLDSDGSFFLYSk.[L]	N-Term(iTRAQ4plex); K17(iTRAQ	1	1
[K].gPSVFPLAPcSR.[S]	N-Term(iTRAQ4plex); C10(Carbar	3	3
[K].nQVSLTcLVk.[G]	N-Term(iTRAQ4plex); C7(Carbam	4	4
[K].nQVSLTcLVk.[G]	N-Term(iTRAQ4plex); C7(Carbam	4	4
[K].ccVEcPPcPAPPVAGPSVFLFPPkPk.[D]	N-Term(iTRAQ4plex); C1(Carbam	1	1
[R].ePQVYTLPPSR.[DE]	N-Term(iTRAQ4plex)	3	3
[R].ePQVYTLPPSR.[DE]	N-Term(iTRAQ4plex)	3	3
[R].ePQVYTLPPSR.[DE]	N-Term(iTRAQ4plex)	3	3
[R].ePQVYTLPPSR.[DE]	N-Term(iTRAQ4plex)	3	3
[R].ePQVYTLPPSR.[DE]	N-Term(iTRAQ4plex)	3	3
[R].ePQVYTLPPSR.[DE]	N-Term(iTRAQ4plex)	3	3
[R].sTSESTAALGcLVk.[D]	N-Term(iTRAQ4plex); C11(Carbar	2	2
[R].ePQVYTLPPSR.[DE]	N-Term(iTRAQ4plex)	3	3
[K].gLPAPIEk.[T]	N-Term(iTRAQ4plex); K8(iTRAQ4	1	1
[K].gLPAPIEk.[T]	N-Term(iTRAQ4plex); K8(iTRAQ4	1	1
[K].ccVEcPPcPAPPVAGPSVFLFPPkPk.[D]	N-Term(iTRAQ4plex); C1(Carbam	1	1
[K].DTLmISR.[T]	M4(Oxidation)	4	4
[K].tTPPMLDSDGSFFLYSk.[L]	N-Term(iTRAQ4plex); K17(iTRAQ	1	1
[R].ePQVYTLPPSR.[DE]	N-Term(iTRAQ4plex)	3	3
[R].sGVQQLIQYYQDQk.[D]	N-Term(iTRAQ4plex); K14(iTRAQ	1	1

[R].sGVQQLIQYYQDQk.[D]	N-Term(iTRAQ4plex); K14(iTRAQ4	1	1
[R].sGVQQLIQYYQDQk.[D]	N-Term(iTRAQ4plex); K14(iTRAQ4	1	1
[K].sYDLDPGAGSLEI.[-]	N-Term(iTRAQ4plex)	1	1
[R].sLPTEDcENEk.[E]	N-Term(iTRAQ4plex); C7(Carbam	1	1
[R].sLPTEDcENEk.[E]	N-Term(iTRAQ4plex); C7(Carbam	1	1
[R].sLPTEDcENEk.[E]	N-Term(iTRAQ4plex); C7(Carbam	1	1
[R].lVGGPMDASVEEEGVR.[R]	N-Term(iTRAQ4plex)	1	1
[R].lVGGPMDASVEEEGVR.[R]	N-Term(iTRAQ4plex)	1	1
[R].lVGGPmDASVEEEGVR.[R]	N-Term(iTRAQ4plex); M6(Oxidat	1	1
[R].aLDFAVGEYNk.[A]	N-Term(iTRAQ4plex); K11(iTRAQ4	1	1
[K].aSNDMYHSR.[A]	N-Term(iTRAQ4plex)	1	1
[K].nIQSLEVIGk.[G]	N-Term(iTRAQ4plex); K10(iTRAQ4	1	1
[K].nIQSLEVIGk.[G]	N-Term(iTRAQ4plex); K10(iTRAQ4	1	1
[K].gTHcNQVEVIATLk.[D]	N-Term(iTRAQ4plex); C4(Carbam	1	1
[K].tTSGIHPk.[N]	N-Term(iTRAQ4plex); K8(iTRAQ4	1	1
[K].gTHcNQVEVIATLk.[D]	N-Term(iTRAQ4plex); C4(Carbam	1	1
[K].wNDDAcHk.[L]	N-Term(iTRAQ4plex); C6(Carbam	1	1
[K].sLTEEAENWGDGEPNNk.[K]	N-Term(iTRAQ4plex); K17(iTRAQ4	1	1
[K].sLTEEAENWGDGEPNNk.[K]	N-Term(iTRAQ4plex); K17(iTRAQ4	1	1
[R].sYYWIGIR.[K]	N-Term(iTRAQ4plex)	1	1
[R].aTSGTEHQFcGGR.[L]	N-Term(iTRAQ4plex); C10(Carbar	1	1
[K].tEESPSAPDAPTcPk.[Q]	N-Term(iTRAQ4plex); C13(Carbar	1	1
[K].tGGLDLPSPPTGASLk.[F]	N-Term(iTRAQ4plex); K16(iTRAQ4	1	1
[R].cINQLLck.[L]	N-Term(iTRAQ4plex); C1(Carbam	1	1
[K].dDFLIYDR.[C]	N-Term(iTRAQ4plex)	1	1
[R].cINQLLck.[L]	N-Term(iTRAQ4plex); C1(Carbam	1	1
[K].rVSLATVDk.[T]	N-Term(iTRAQ4plex); K9(iTRAQ4	1	1
[K].dDFLIYDR.[C]	N-Term(iTRAQ4plex)	1	1
[R].yGFIEGHVVIPR.[I]	N-Term(iTRAQ4plex)	1	1
[R].yGFIEGHVVIPR.[I]	N-Term(iTRAQ4plex)	1	1
[R].fAGVFHVEk.[N]	N-Term(iTRAQ4plex); K9(iTRAQ4	1	1
[R].fAGVFHVEk.[N]	N-Term(iTRAQ4plex); K9(iTRAQ4	1	1
[R].tEAADLck.[A]	N-Term(iTRAQ4plex); C7(Carbam	1	1
[R].aFQYHSk.[E]	N-Term(iTRAQ4plex); K7(iTRAQ4	1	3
[R].mRDVVLFEk.[-K]	N-Term(iTRAQ4plex); K9(iTRAQ4	1	2
[R].aFQYHSk.[E]	N-Term(iTRAQ4plex); K7(iTRAQ4	1	3
[R].dVVLFEk.[-K]	N-Term(iTRAQ4plex); K7(iTRAQ4	1	2
[R].dVVLFEk.[-K]	N-Term(iTRAQ4plex); K7(iTRAQ4	1	2
[R].aFQYHSk.[E]	N-Term(iTRAQ4plex); K7(iTRAQ4	1	3
[R].aFQYHSk.[E]	N-Term(iTRAQ4plex); K7(iTRAQ4	1	3
[R].aFQYHSk.[E]	N-Term(iTRAQ4plex); K7(iTRAQ4	1	3
[R].vDLVDFEDNYQFAk.[Y]	N-Term(iTRAQ4plex); K14(iTRAQ4	1	1
[R].vDLVDFEDNYQFAk.[Y]	N-Term(iTRAQ4plex); K14(iTRAQ4	1	1
[R].vDGSVDFYR.[D]	N-Term(iTRAQ4plex)	1	1
[K].vADEAEk.[Y]	N-Term(iTRAQ4plex); K7(iTRAQ4	1	2
[K].eITALAPSTMk.[I]	N-Term(iTRAQ4plex); K11(iTRAQ4	1	6
[R].hQGVMVGMGQk.[D]	N-Term(iTRAQ4plex); K11(iTRAQ4	1	7
[KR].sYELPDGQVITIGNER.[F]	N-Term(iTRAQ4plex)	1	10
[K].eITALAPSTMk.[I]	N-Term(iTRAQ4plex); K11(iTRAQ4	1	6
[R].hQGVMVGMGQk.[D]	N-Term(iTRAQ4plex); K11(iTRAQ4	1	7
[KR].sYELPDGQVITIGNER.[F]	N-Term(iTRAQ4plex)	1	10
[K].sPWcDVFDIDDAk.[V]	N-Term(iTRAQ4plex); C4(Carbam	1	1

[R].qLHGLLQAR.[G]	N-Term(iTRAQ4plex)	1	1
[K].dILQScQTSEEcELAR.[A]	N-Term(iTRAQ4plex); C6(Carbam	1	1
[R].sQYEQLAEQNR.[K]	N-Term(iTRAQ4plex)	1	1
[R].aLEESNYELEGk.[I]	N-Term(iTRAQ4plex); K12(iTRAQ4	1	1
[KR].LAADDFR.[AITLV]		1	17
[R].fVTAVGTQGAISk.[E]	N-Term(iTRAQ4plex); K13(iTRAQ4	1	1
[R].ikPATWETGISMR.[F]	N-Term(iTRAQ4plex); K2(iTRAQ4	1	1
[R].gIIIQGGk.[H]	N-Term(iTRAQ4plex); K8(iTRAQ4	1	1
[K].dQVETALk.[A]	N-Term(iTRAQ4plex); K8(iTRAQ4	1	1
[R].lLGLSLAGk.[D]	N-Term(iTRAQ4plex); K9(iTRAQ4	1	1
[R].iMGITLVSk.[K]	N-Term(iTRAQ4plex); K9(iTRAQ4	1	1
[R].nEVWWTIDGk.[K]	N-Term(iTRAQ4plex); K10(iTRAQ4	1	1
[R].tQILSIk.[K]	N-Term(iTRAQ4plex); K7(iTRAQ4	1	1
[K].vAFPLEVVQk.[D]	N-Term(iTRAQ4plex); K10(iTRAQ4	1	1
[R].vYVSGLMk.[G]	N-Term(iTRAQ4plex); K8(iTRAQ4	1	1
[K].gLFEVNPWk.[R]	N-Term(iTRAQ4plex); K9(iTRAQ4	1	1
[K].lLLSSETPIEGk.[N]	N-Term(iTRAQ4plex); K12(iTRAQ4	1	1
[R].lSQLSVTDVTTSSLR.[L]	N-Term(iTRAQ4plex)	1	2
[R].lGPISADSTTAPLEk.[E]	N-Term(iTRAQ4plex); K15(iTRAQ4	1	1
[R].tLSPVLESPR.[D]	N-Term(iTRAQ4plex)	1	2
[K].qEYDESGPSIVHR.[K]	N-Term(iTRAQ4plex)	1	7
[KR].sYELPDGQVITIGNER.[F]	N-Term(iTRAQ4plex)	1	10
[K].IWHHTFYNELR.[V]		1	9
[K].qEYDESGPSIVHR.[K]	N-Term(iTRAQ4plex)	1	7
[KR].sYELPDGQVITIGNER.[F]	N-Term(iTRAQ4plex)	1	10
[K].IWHHTFYNELR.[V]		1	9
[K].qEYDESGPSIVHR.[K]	N-Term(iTRAQ4plex)	1	7
[KR].sYELPDGQVITIGNER.[F]	N-Term(iTRAQ4plex)	1	10
[K].IWHHTFYNELR.[V]		1	9
[R].eIIkPAEk.[S]	N-Term(iTRAQ4plex); K4(iTRAQ4	1	1
[R].iITGPEIk.[Y]	N-Term(iTRAQ4plex); K8(iTRAQ4	1	1
[K].qVIELAGk.[Q]	N-Term(iTRAQ4plex); K8(iTRAQ4	1	1
[R].lFAYPDTHR.[H]	N-Term(iTRAQ4plex)	1	1
[R].dPILFPSFIHSQk.[R]	N-Term(iTRAQ4plex); K13(iTRAQ4	1	1
[R].lGPNYLHIPVNcPYR.[A]	N-Term(iTRAQ4plex); C12(Carbar	1	1
[R].YQLGSGEAR.[L]		1	1
[R].lPAVEPTDQAQYLcR.[A]	N-Term(iTRAQ4plex); C14(Carbar	1	1
[R].aLEVEEcR.[C]	N-Term(iTRAQ4plex); C7(Carbam	1	1
[R].gSFEFPVGDAVSk.[V]	N-Term(iTRAQ4plex); K13(iTRAQ4	2	2
[R].gSFEFPVGDAVSk.[V]	N-Term(iTRAQ4plex); K13(iTRAQ4	2	2
[R].gSFEFPVGDAVSk.[V]	N-Term(iTRAQ4plex); K13(iTRAQ4	2	2
[R].gSFEFPVGDAVSk.[V]	N-Term(iTRAQ4plex); K13(iTRAQ4	2	2
[R].gSFEFPVGDAVSk.[V]	N-Term(iTRAQ4plex); K13(iTRAQ4	2	2
[R].YIYGkPVQGVAYVR.[F]	K5(iTRAQ4plex)	2	2
[R].yLDkTEQWSTLPPETk.[D]	N-Term(iTRAQ4plex); K4(iTRAQ4	2	2
[R].gSFEFPVGDAVSk.[V]	N-Term(iTRAQ4plex); K13(iTRAQ4	2	2
[K].vLSLAQEQVGGSPEk.[L]	N-Term(iTRAQ4plex); K15(iTRAQ4	2	2
[K].vLSLAQEQVGGSPEk.[L]	N-Term(iTRAQ4plex); K15(iTRAQ4	2	2
[K].vLSLAQEQVGGSPEk.[L]	N-Term(iTRAQ4plex); K15(iTRAQ4	2	2
[K].vLSLAQEQVGGSPEk.[L]	N-Term(iTRAQ4plex); K15(iTRAQ4	2	2
[K].vLSLAQEQVGGSPEk.[L]	N-Term(iTRAQ4plex); K15(iTRAQ4	2	2
[R].tYNVLDMk.[N]	N-Term(iTRAQ4plex); K8(iTRAQ4	2	2

[R].vHYTVcIWR.[N]	N-Term(iTRAQ4plex); C6(Carbam	2	2
[R].VGDTLNLNLR.[A]		2	2
[R].tYNVLDMk.[N]	N-Term(iTRAQ4plex); K8(iTRAQ4	2	2
[R].yLDkTEQWSTLPPETk.[D]	N-Term(iTRAQ4plex); K4(iTRAQ4	2	2
[R].yLDkTEQWSTLPPETk.[D]	N-Term(iTRAQ4plex); K4(iTRAQ4	2	2
[R].yLDkTEQWSTLPPETk.[D]	N-Term(iTRAQ4plex); K4(iTRAQ4	2	2
[R].yLDkTEQWSTLPPETk.[D]	N-Term(iTRAQ4plex); K4(iTRAQ4	2	2
[R].yLDkTEQWSTLPPETk.[D]	N-Term(iTRAQ4plex); K4(iTRAQ4	2	2
[R].rGHLFLQTDQPIYNPGQR.[V]	N-Term(iTRAQ4plex)	2	2
[R].rGHLFLQTDQPIYNPGQR.[V]	N-Term(iTRAQ4plex)	2	2
[R].gHLFLQTDQPIYNPGQR.[V]	N-Term(iTRAQ4plex)	2	2
[K].ITQVLHFTk.[D]	K9(iTRAQ4plex)	2	2
[K].ScGLHQLLR.[G]	C2(Carbamidomethyl)	2	2
[R].FGLLDEDGkk.[T]	K9(iTRAQ4plex); K10(iTRAQ4ple	2	2
[K].sHkPLNMGk.[V]	N-Term(iTRAQ4plex); K3(iTRAQ4	2	2
[K].lGQYASPTAk.[R]	N-Term(iTRAQ4plex); K10(iTRAQ	2	2
[K].lVNGQSHISLSk.[A]	N-Term(iTRAQ4plex); K12(iTRAQ	2	2
[R].kADGSYAAWLSR.[DG]	K1(iTRAQ4plex)	2	2
[R].kADGSYAAWLSR.[DG]	N-Term(iTRAQ4plex)	2	2
[R].kADGSYAAWLSR.[DG]	N-Term(iTRAQ4plex)	2	2
[R].kADGSYAAWLSR.[DG]	K1(iTRAQ4plex)	2	2
[R].tLEIPGNSDPNmIPDGDFNSYVR.[V]	N-Term(iTRAQ4plex); M12(Oxida	2	2
[K].sHALQLNNR.[Q]	N-Term(iTRAQ4plex)	2	2
[K].sHkPLNMGk.[V]	N-Term(iTRAQ4plex); K3(iTRAQ4	2	2
[R].tLEIPGNSDPNMIPDGDFNSYVR.[V]	N-Term(iTRAQ4plex)	2	2
[R].tLEIPGNSDPNMIPDGDFNSYVR.[V]	N-Term(iTRAQ4plex)	2	2
[R].TLEIPGNSDPNMIPDGDFNSYVR.[V]		2	2
[R].gLEEELQFSLGSk.[I]	N-Term(iTRAQ4plex); K13(iTRAQ	2	2
[R].gLEEELQFSLGSk.[I]	N-Term(iTRAQ4plex); K13(iTRAQ	2	2
[R].gLEEELQFSLGSk.[I]	N-Term(iTRAQ4plex); K13(iTRAQ	2	2
[R].gLEEELQFSLGSk.[I]	N-Term(iTRAQ4plex); K13(iTRAQ	2	2
[R].gLEEELQFSLGSk.[I]	N-Term(iTRAQ4plex); K13(iTRAQ	2	2
[R].gLEEELQFSLGSk.[I]	N-Term(iTRAQ4plex); K13(iTRAQ	2	2
[R].gLEEELQFSLGSk.[I]	N-Term(iTRAQ4plex); K13(iTRAQ	2	2
[R].eELVYELNPLDHR.[G]	N-Term(iTRAQ4plex)	2	2
[R].eELVYELNPLDHR.[G]	N-Term(iTRAQ4plex)	2	2
[R].eELVYELNPLDHR.[G]	N-Term(iTRAQ4plex)	2	2
[R].eELVYELNPLDHR.[G]	N-Term(iTRAQ4plex)	2	2
[R].cSVFYGAPSk.[S]	N-Term(iTRAQ4plex); C1(Carbam	2	2
[R].cSVFYGAPSk.[S]	N-Term(iTRAQ4plex); C1(Carbam	2	2
[R].vHYTVcIWR.[N]	N-Term(iTRAQ4plex); C6(Carbam	2	2
[K].vDFTLSSER.[D]	N-Term(iTRAQ4plex)	2	2
[R].vHYTVcIWR.[N]	N-Term(iTRAQ4plex); C6(Carbam	2	2
[R].vHYTVcIWR.[N]	N-Term(iTRAQ4plex); C6(Carbam	2	2
[R].kADGSYAAWLSR.[DG]	K1(iTRAQ4plex)	2	2
[K].iTQVLHFTk.[D]	N-Term(iTRAQ4plex); K9(iTRAQ4	2	2
[K].iTQVLHFTk.[D]	N-Term(iTRAQ4plex); K9(iTRAQ4	2	2
[R].kADGSYAAWLSR.[DG]	N-Term(iTRAQ4plex)	2	2
[K].iTQVLHFTk.[D]	N-Term(iTRAQ4plex); K9(iTRAQ4	2	2
[R].GHLFLQTDQPIYNPGQR.[V]		2	2
[K].mRPSTDTITVMVENSHGLR.[V]	N-Term(iTRAQ4plex)	2	2
[K].sHkPLNMGk.[V]	N-Term(iTRAQ4plex); K3(iTRAQ4	2	2

[K].sHkPLNMGk.[V]	N-Term(iTRAQ4plex); K3(iTRAQ4	2	2
[R].aLEILQEEDLIDEDDIPVR.[S]	N-Term(iTRAQ4plex)	2	2
[R].kADGSYAAWLSR.[DG]	N-Term(iTRAQ4plex); K1(iTRAQ4	2	2
[K].mRPSTDTITVMVENSHGLR.[V]	N-Term(iTRAQ4plex); M1(Oxidat	2	2
[R].rGHLFLQTDQPIYNPGQR.[V]	N-Term(iTRAQ4plex)	2	2
[R].rGHLFLQTDQPIYNPGQR.[V]	N-Term(iTRAQ4plex)	2	2
[R].rGHLFLQTDQPIYNPGQR.[V]	N-Term(iTRAQ4plex)	2	2
[R].rGHLFLQTDQPIYNPGQR.[V]	N-Term(iTRAQ4plex)	2	2
[R].yIYGkPVQGVAYVR.[F]	N-Term(iTRAQ4plex); K5(iTRAQ4	2	2
[R].yIYGkPVQGVAYVR.[F]	N-Term(iTRAQ4plex); K5(iTRAQ4	2	2
[R].rGHLFLQTDQPIYNPGQR.[V]	N-Term(iTRAQ4plex)	2	2
[R].rGHLFLQTDQPIYNPGQR.[V]	N-Term(iTRAQ4plex)	2	2
[K].scGLHQLLR.[G]	N-Term(iTRAQ4plex); C2(Carbam	2	2
[K].scGLHQLLR.[G]	N-Term(iTRAQ4plex); C2(Carbam	2	2
[K].scGLHQLLR.[G]	N-Term(iTRAQ4plex); C2(Carbam	2	2
[K].dDPDAPLQPVTPLQLFEGR.[R]	N-Term(iTRAQ4plex)	2	2
[K].dDPDAPLQPVTPLQLFEGR.[R]	N-Term(iTRAQ4plex)	2	2
[R].ecVGFEAVQEVPVGLVQPASATLYDYYNPE	N-Term(iTRAQ4plex); C2(Carbam	2	2
[R].ecVGFEAVQEVPVGLVQPASATLYDYYNPE	N-Term(iTRAQ4plex); C2(Carbam	2	2
[K].sHALQLNNR.[Q]	N-Term(iTRAQ4plex)	2	2
[K].lVNGQSHISLSk.[A]	N-Term(iTRAQ4plex); K12(iTRAQ	2	2
[K].yVLPNFEVk.[I]	N-Term(iTRAQ4plex); K9(iTRAQ4	2	2
[R].gPEVQLVAHSPWLk.[D]	N-Term(iTRAQ4plex); K14(iTRAQ	2	2
[R].gPEVQLVAHSPWLk.[D]	N-Term(iTRAQ4plex); K14(iTRAQ	2	2
[R].lLATLcSAEVcQcAEGk.[C]	N-Term(iTRAQ4plex); C6(Carbam	2	2
[R].gcGEQTMIYLAPTLAASR.[Y]	N-Term(iTRAQ4plex); C2(Carbam	2	2
[R].gPEVQLVAHSPWLk.[D]	N-Term(iTRAQ4plex); K14(iTRAQ	2	2
[R].gPEVQLVAHSPWLk.[D]	N-Term(iTRAQ4plex); K14(iTRAQ	2	2
[R].yVSHFETEGPHVLLYFDSVPTSR.[E]	N-Term(iTRAQ4plex)	2	2
[K].mRPSTDTITVMVENSHGLR.[V]	N-Term(iTRAQ4plex)	2	2
[K].mRPSTDTITVMVENSHGLR.[V]	N-Term(iTRAQ4plex)	2	2
[K].mRPSTDTITVMVENSHGLR.[V]	N-Term(iTRAQ4plex)	2	2
[K].mRPSTDTITVMVENSHGLR.[V]	N-Term(iTRAQ4plex)	2	2
[R].yVSHFETEGPHVLLYFDSVPTSR.[E]	N-Term(iTRAQ4plex)	2	2
[R].yVSHFETEGPHVLLYFDSVPTSR.[E]	N-Term(iTRAQ4plex)	2	2
[K].mRPSTDTITVMVENSHGLR.[V]	N-Term(iTRAQ4plex)	2	2
[R].yVSHFETEGPHVLLYFDSVPTSR.[E]	N-Term(iTRAQ4plex)	2	2
[K].kYVLPNFEVk.[I]	N-Term(iTRAQ4plex); K1(iTRAQ4	2	2
[K].mRPSTDTITVMVENSHGLR.[V]	N-Term(iTRAQ4plex)	2	2
[K].kYVLPNFEVk.[I]	N-Term(iTRAQ4plex); K1(iTRAQ4	2	2
[R].gHLFLQTDQPIYNPGQR.[V]	N-Term(iTRAQ4plex)	2	2
[K].kYVLPNFEVk.[I]	N-Term(iTRAQ4plex); K1(iTRAQ4	2	2
[K].kYVLPNFEVk.[I]	N-Term(iTRAQ4plex); K1(iTRAQ4	2	2
[R].gHLFLQTDQPIYNPGQR.[V]	N-Term(iTRAQ4plex)	2	2
[R].gHLFLQTDQPIYNPGQR.[V]	N-Term(iTRAQ4plex)	2	2
[K].gLcVATPVQLR.[V]	N-Term(iTRAQ4plex); C3(Carbam	2	2
[K].gLcVATPVQLR.[V]	N-Term(iTRAQ4plex); C3(Carbam	2	2
[R].vGDTLNLNLR.[A]	N-Term(iTRAQ4plex)	2	2
[K].gLcVATPVQLR.[V]	N-Term(iTRAQ4plex); C3(Carbam	2	2
[K].gLcVATPVQLR.[V]	N-Term(iTRAQ4plex); C3(Carbam	2	2
[K].kYVLPNFEVk.[I]	N-Term(iTRAQ4plex); K1(iTRAQ4	2	2
[R].gHLFLQTDQPIYNPGQR.[V]	N-Term(iTRAQ4plex)	2	2

[R].yVSHFETEGPHVLLYFDSVPTSR.[E]	N-Term(iTRAQ4plex)	2	2
[R].gPEVQLVAHSPWLk.[D]	N-Term(iTRAQ4plex); K14(iTRAQ4	2	2
[R].gPEVQLVAHSPWLk.[D]	N-Term(iTRAQ4plex); K14(iTRAQ4	2	2
[R].gPEVQLVAHSPWLk.[D]	N-Term(iTRAQ4plex); K14(iTRAQ4	2	2
[R].gPEVQLVAHSPWLk.[D]	N-Term(iTRAQ4plex); K14(iTRAQ4	2	2
[R].gPEVQLVAHSPWLk.[D]	N-Term(iTRAQ4plex); K14(iTRAQ4	2	2
[R].gPEVQLVAHSPWLk.[D]	N-Term(iTRAQ4plex); K14(iTRAQ4	2	2
[R].gPEVQLVAHSPWLk.[D]	N-Term(iTRAQ4plex); K14(iTRAQ4	2	2
[R].GPEVQLVAHSPWLk.[D]	K14(iTRAQ4plex)	2	2
[K].eVYMPSSIFQDDFVIPDISEPGTWk.[I]	N-Term(iTRAQ4plex); K25(iTRAQ4	2	2
[K].eVYMPSSIFQDDFVIPDISEPGTWk.[I]	N-Term(iTRAQ4plex); K25(iTRAQ4	2	2
[R].gHLFLQTDQPIYNPGQR.[V]	N-Term(iTRAQ4plex)	2	2
[K].sHALQLNNR.[Q]	N-Term(iTRAQ4plex)	2	2
[K].aDGSYAAWLSR.[DG]	N-Term(iTRAQ4plex)	2	2
[R].vGDTLNLNLR.[A]	N-Term(iTRAQ4plex)	2	2
[R].vGDTLNLNLR.[A]	N-Term(iTRAQ4plex)	2	2
[R].ecVGFEAVQEVPVGLVQPASATLYDYYNPE	N-Term(iTRAQ4plex); C2(Carbam	2	2
[R].ecVGFEAVQEVPVGLVQPASATLYDYYNPE	N-Term(iTRAQ4plex); C2(Carbam	2	2
[K].aEMADQASAWLTR.[Q]	N-Term(iTRAQ4plex)	1	1
[R].vTASDPLDTLGSEGALSPGGVASLLR.[L]	N-Term(iTRAQ4plex)	2	2
[R].sTQDTVIALDALSAYWIASHTTEER.[G]	N-Term(iTRAQ4plex)	2	2
[R].STQDTVIALDALSAYWIASHTTEER.[G]		2	2
[K].eVYmPSSIFQDDFVIPDISEPGTWk.[I]	N-Term(iTRAQ4plex); M4(Oxidat	2	2
[R].lLATLcSAEVcQcAEGk.[C]	N-Term(iTRAQ4plex); C6(Carbam	2	2
[R].VTASDPLDTLGSEGALSPGGVASLLR.[L]		2	2
[R].vTASDPLDTLGSEGALSPGGVASLLR.[L]	N-Term(iTRAQ4plex)	2	2
[R].LLATLcSAEVcQcAEGk.[C]	C6(Carbamidomethyl); C11(Carb	2	2
[R].kADGSYAAWLSR.[DG]	N-Term(iTRAQ4plex); K1(iTRAQ4	2	2
[R].kADGSYAAWLSR.[DG]	N-Term(iTRAQ4plex); K1(iTRAQ4	2	2
[K].scGLHQLLR.[G]	N-Term(iTRAQ4plex); C2(Carbam	2	2
[R].vFALDQk.[M]	N-Term(iTRAQ4plex); K7(iTRAQ4	2	2
[R].kADGSYAAWLSR.[DG]	N-Term(iTRAQ4plex); K1(iTRAQ4	2	2
[R].kADGSYAAWLSR.[DG]	N-Term(iTRAQ4plex); K1(iTRAQ4	2	2
[R].kADGSYAAWLSR.[DG]	N-Term(iTRAQ4plex); K1(iTRAQ4	2	2
[K].vVEEQESR.[V]	N-Term(iTRAQ4plex)	2	2
[K].vLSLAQEQVGGSPEk.[L]	N-Term(iTRAQ4plex); K15(iTRAQ4	2	2
[R].eAPkVVEEQESR.[V]	N-Term(iTRAQ4plex); K4(iTRAQ4	2	2
[R].aAcAQLNDFLQEYGTQGcQV.[-]	N-Term(iTRAQ4plex); C3(Carbam	2	2
[R].aAcAQLNDFLQEYGTQGcQV.[-]	N-Term(iTRAQ4plex); C3(Carbam	2	2
[K].lQETSNWLLSQQQADGSFQDPcPVLDR.[S	N-Term(iTRAQ4plex); C22(Carbar	1	1
[K].lQETSNWLLSQQQADGSFQDPcPVLDR.[S	N-Term(iTRAQ4plex); C22(Carbar	1	1
[R].lLATLcSAEVcQcAEGk.[C]	N-Term(iTRAQ4plex); C6(Carbam	2	2
[R].lLATLcSAEVcQcAEGk.[C]	N-Term(iTRAQ4plex); C6(Carbam	2	2
[R].lLATLcSAEVcQcAEGk.[C]	N-Term(iTRAQ4plex); C6(Carbam	2	2
[R].lLATLcSAEVcQcAEGk.[C]	N-Term(iTRAQ4plex); C6(Carbam	2	2
[R].ePFLSccQFAESLR.[K]	N-Term(iTRAQ4plex); C6(Carbam	2	2
[R].ePFLSccQFAESLR.[K]	N-Term(iTRAQ4plex); C6(Carbam	2	2
[R].ePFLSccQFAESLR.[K]	N-Term(iTRAQ4plex); C6(Carbam	2	2
[R].aVGSGATFSHYYYMILSR.[G]	N-Term(iTRAQ4plex)	2	2
[K].aEFQDALEk.[L]	N-Term(iTRAQ4plex); K9(iTRAQ4	2	2
[R].tTNIQGINLLFSSR.[R]	N-Term(iTRAQ4plex)	2	2
[R].tTNIQGINLLFSSR.[R]	N-Term(iTRAQ4plex)	2	2

[K].tEQWSTLPPETk.[D]	N-Term(iTRAQ4plex); K12(iTRAQ	2	2
[K].tEQWSTLPPETk.[D]	N-Term(iTRAQ4plex); K12(iTRAQ	2	2
[K].tEQWSTLPPETk.[D]	N-Term(iTRAQ4plex); K12(iTRAQ	2	2
[K].tEQWSTLPPETk.[D]	N-Term(iTRAQ4plex); K12(iTRAQ	2	2
[R].TTNIQGINLLFSSR.[R]		2	2
[K].tEQWSTLPPETk.[D]	N-Term(iTRAQ4plex); K12(iTRAQ	2	2
[K].tEQWSTLPPETk.[D]	N-Term(iTRAQ4plex); K12(iTRAQ	2	2
[K].TEQWSTLPPETk.[D]	K12(iTRAQ4plex)	2	2
[K].tEQWSTLPPETk.[D]	N-Term(iTRAQ4plex); K12(iTRAQ	2	2
[K].tEQWSTLPPETk.[D]	N-Term(iTRAQ4plex); K12(iTRAQ	2	2
[R].dFALLSLQVPLk.[D]	N-Term(iTRAQ4plex); K12(iTRAQ	2	2
[R].aLEILQEEDLIDEDDIPVR.[S]	N-Term(iTRAQ4plex)	2	2
[K].sHkPLNMGk.[V]	N-Term(iTRAQ4plex); K3(iTRAQ4	2	2
[K].lNMGITDLQGLR.[L]	N-Term(iTRAQ4plex)	2	2
[K].lNMGITDLQGLR.[L]	N-Term(iTRAQ4plex)	2	2
[R].dSSTWLTAFVLk.[V]	N-Term(iTRAQ4plex); K12(iTRAQ	1	1
[R].EELVYELNPLDHR.[G]		2	2
[K].vGLSGmAIADVTLLSGFHALR.[A]	N-Term(iTRAQ4plex); M6(Oxidat	2	2
[R].rFEQLELRPVLYNYLDk.[N]	N-Term(iTRAQ4plex); K17(iTRAQ	2	2
[R].hLVPGAPFLLQALVR.[E]	N-Term(iTRAQ4plex)	2	2
[K].aSAGLLGAHAAAITAYALTLTk.[A]	N-Term(iTRAQ4plex); K22(iTRAQ	2	2
[K].aSAGLLGAHAAAITAYALTLTk.[A]	N-Term(iTRAQ4plex); K22(iTRAQ	2	2
[R].vDVQAGAcEGk.[L]	N-Term(iTRAQ4plex); C8(Carbam	2	2
[R].vDVQAGAcEGk.[L]	N-Term(iTRAQ4plex); C8(Carbam	2	2
[R].vDVQAGAcEGk.[L]	N-Term(iTRAQ4plex); C8(Carbam	2	2
[K].iTPGkPYILTVPGHLDEMQLDIQAR.[Y]	N-Term(iTRAQ4plex); K5(iTRAQ4	2	2
[K].dHAVDLIQk.[G]	N-Term(iTRAQ4plex); K9(iTRAQ4	2	2
[R].tTNIQGINLLFSSR.[R]	N-Term(iTRAQ4plex)	2	2
[K].dHAVDLIQk.[G]	N-Term(iTRAQ4plex); K9(iTRAQ4	2	2
[K].dHAVDLIQk.[G]	N-Term(iTRAQ4plex); K9(iTRAQ4	2	2
[K].iTPGkPYILTVPGHLDEMQLDIQAR.[Y]	N-Term(iTRAQ4plex); K5(iTRAQ4	2	2
[K].dHAVDLIQk.[G]	N-Term(iTRAQ4plex); K9(iTRAQ4	2	2
[K].dHAVDLIQk.[G]	N-Term(iTRAQ4plex); K9(iTRAQ4	2	2
[K].lNMGITDLQGLR.[L]	N-Term(iTRAQ4plex)	2	2
[R].fGLLDEDGk.[K]	N-Term(iTRAQ4plex); K9(iTRAQ4	2	2
[R].VDVQAGAcEGk.[L]	C8(Carbamidomethyl); K11(iTRA	2	2
[K].VLSLAQEQVGGSPEk.[L]	K15(iTRAQ4plex)	2	2
[R].gLQDEDGYR.[M]	N-Term(iTRAQ4plex)	2	2
[R].gLQDEDGYR.[M]	N-Term(iTRAQ4plex)	2	2
[R].DFALLSLQVPLk.[D]	K12(iTRAQ4plex)	2	2
[R].LTVAAPPSGGPGFLSIERPDSRPPR.[V]		2	2
[R].gLQDEDGYR.[M]	N-Term(iTRAQ4plex)	2	2
[R].gLQDEDGYR.[M]	N-Term(iTRAQ4plex)	2	2
[R].eMSGSPASGIPVk.[V]	N-Term(iTRAQ4plex); K13(iTRAQ	2	2
[R].fGLLDEDGkk.[T]	N-Term(iTRAQ4plex); K9(iTRAQ4	2	2
[R].fGLLDEDGkk.[T]	N-Term(iTRAQ4plex); K9(iTRAQ4	2	2
[R].fGLLDEDGk.[K]	N-Term(iTRAQ4plex); K9(iTRAQ4	2	2
[R].gLQDEDGYR.[M]	N-Term(iTRAQ4plex)	2	2
[R].gLQDEDGYR.[M]	N-Term(iTRAQ4plex)	2	2
[R].gLQDEDGYR.[M]	N-Term(iTRAQ4plex)	2	2
[R].gLQDEDGYR.[M]	N-Term(iTRAQ4plex)	2	2
[R].tYNVLDMk.[N]	N-Term(iTRAQ4plex); K8(iTRAQ4	2	2

[K].LHLETDSLALVALGALDTALYAAGSk.[S]	K26(iTRAQ4plex)	2	2
[K].lHLETDSLALVALGALDTALYAAGSk.[S]	N-Term(iTRAQ4plex); K26(iTRAQ4	2	2
[R].lTVAAPPSGGPGFLSIERPDSRPPR.[V]	N-Term(iTRAQ4plex)	2	2
[R].lTVAAPPSGGPGFLSIERPDSRPPR.[V]	N-Term(iTRAQ4plex)	2	2
[R].lTVAAPPSGGPGFLSIERPDSRPPR.[V]	N-Term(iTRAQ4plex)	2	2
[R].fQILTLWLPDSLTTWEIHGLSLSk.[T]	N-Term(iTRAQ4plex); K24(iTRAQ4	2	2
[R].fQILTLWLPDSLTTWEIHGLSLSk.[T]	N-Term(iTRAQ4plex); K24(iTRAQ4	2	2
[K].tEQWSTLPPETk.[D]	N-Term(iTRAQ4plex); K12(iTRAQ4	2	2
[R].sMQGGLVGNDETVALTAFVTIALHHGLAV	N-Term(iTRAQ4plex); K39(iTRAQ4	2	2
[K].tEQWSTLPPETk.[D]	N-Term(iTRAQ4plex); K12(iTRAQ4	2	2
[K].tEQWSTLPPETk.[D]	N-Term(iTRAQ4plex); K12(iTRAQ4	2	2
[K].tEQWSTLPPETk.[D]	N-Term(iTRAQ4plex); K12(iTRAQ4	2	2
[R].sMQGGLVGNDETVALTAFVTIALHHGLAV	N-Term(iTRAQ4plex); K39(iTRAQ4	2	2
[K].tEQWSTLPPETk.[D]	N-Term(iTRAQ4plex); K12(iTRAQ4	2	2
[K].tEQWSTLPPETk.[D]	N-Term(iTRAQ4plex); K12(iTRAQ4	2	2
[K].lELSVDGAk.[Q]	N-Term(iTRAQ4plex); K9(iTRAQ4	2	2
[K].lGQYASPTAk.[R]	N-Term(iTRAQ4plex); K10(iTRAQ4	2	2
[R].sTQDTVIALDALSAYWIASHTTEER.[G]	N-Term(iTRAQ4plex)	2	2
[K].vGLSGMAIADVTLLSGFHALR.[A]	N-Term(iTRAQ4plex)	2	2
[K].aNSFLGEk.[A]	N-Term(iTRAQ4plex); K8(iTRAQ4	1	1
[R].sFFPENWLWR.[V]	N-Term(iTRAQ4plex)	2	2
[R].SFFPENWLWR.[V]		2	2
[K].vGLSGMAIADVTLLSGFHALR.[A]	N-Term(iTRAQ4plex)	2	2
[R].FGLLDEDGkk.[T]	K9(iTRAQ4plex); K10(iTRAQ4plex	2	2
[R].vTASDPLDTLGSEGALSPGGVASLLR.[L]	N-Term(iTRAQ4plex)	2	2
[R].lLLFSPSVVHLGVPLSVGVQLQDVPR.[G]	N-Term(iTRAQ4plex)	2	2
[R].lLLFSPSVVHLGVPLSVGVQLQDVPR.[G]	N-Term(iTRAQ4plex)	2	2
[R].hLVPGAPFLLQALVR.[E]	N-Term(iTRAQ4plex)	2	2
[R].dkGQAGLQR.[A]	N-Term(iTRAQ4plex); K2(iTRAQ4	2	2
[K].sHALQLNNR.[Q]	N-Term(iTRAQ4plex)	2	2
[R].cSVFYGAPSk.[S]	N-Term(iTRAQ4plex); C1(Carbam	2	2
[K].sHkPLNmGk.[V]	N-Term(iTRAQ4plex); K3(iTRAQ4	2	2
[K].sHKPLNMGk.[V]	N-Term(iTRAQ4plex); K9(iTRAQ4	2	2
[K].tEQWSTLPPETk.[D]	N-Term(iTRAQ4plex); K12(iTRAQ4	2	2
[R].gPEVQLVAHSPWLk.[D]	N-Term(iTRAQ4plex); K14(iTRAQ4	2	2
[R].gLQDEDGYR.[M]	N-Term(iTRAQ4plex)	2	2
[K].sHALQLNNR.[Q]	N-Term(iTRAQ4plex)	2	2
[K].aEMADQASAWLTR.[Q]	N-Term(iTRAQ4plex)	1	1
[R].eELVYELNPLDHR.[G]	N-Term(iTRAQ4plex)	2	2
[R].vEYGFQVk.[V]	N-Term(iTRAQ4plex); K8(iTRAQ4	2	2
[R].lTVAAPPSGGPGFLSIERPDSRPPR.[V]	N-Term(iTRAQ4plex)	2	2
[R].gPEVQLVAHSPWLk.[D]	N-Term(iTRAQ4plex); K14(iTRAQ4	2	2
[R].gLQDEDGYR.[M]	N-Term(iTRAQ4plex)	2	2
[K].TEQWSTLPPETk.[D]	K12(iTRAQ4plex)	2	2
[R].cSVFYGAPSk.[S]	N-Term(iTRAQ4plex); C1(Carbam	2	2
[K].lGQYASPTAk.[R]	N-Term(iTRAQ4plex); K10(iTRAQ4	2	2
[K].aEFQDALEk.[L]	N-Term(iTRAQ4plex); K9(iTRAQ4	2	2
[K].TEQWSTLPPETk.[D]	K12(iTRAQ4plex)	2	2
[K].scGLHQLLR.[G]	N-Term(iTRAQ4plex); C2(Carbam	2	2
[R].tLEIPGNSDPNMIPDGDFNSYVR.[V]	N-Term(iTRAQ4plex)	2	2
[R].tYNVLDMk.[N]	N-Term(iTRAQ4plex); K8(iTRAQ4	2	2
[K].tEQWSTLPPETk.[D]	N-Term(iTRAQ4plex); K12(iTRAQ4	2	2

[K].tEQWSTLPPETk.[D]	N-Term(iTRAQ4plex); K12(iTRAQ	2	2
[K].vLQIEkEGAIHR.[E]	N-Term(iTRAQ4plex); K6(iTRAQ4	2	2
[K].ScGLHQLLR.[G]	C2(Carbamidomethyl)	2	2
[R].nNVPcSPk.[V]	N-Term(iTRAQ4plex); C5(Carbam	2	2
[R].GHLFLQTDQPIYNPGQR.[V]		2	2
[R].tTNIQGINLLFSSR.[R]	N-Term(iTRAQ4plex)	2	2
[R].kADGSYAAWLSR.[DG]	N-Term(iTRAQ4plex); K1(iTRAQ4	2	2
[R].vHYTVcIWR.[N]	N-Term(iTRAQ4plex); C6(Carbam	2	2
[R].GHLFLQTDQPIYNPGQR.[V]		2	2
[K].vDFTLSSER.[D]	N-Term(iTRAQ4plex)	2	2
[K].YVLPNFEVk.[I]	K9(iTRAQ4plex)	2	2
[R].RGHLFLQTDQPIYNPGQR.[V]		2	2
[R].gLQDEDGYR.[M]	N-Term(iTRAQ4plex)	2	2
[K].kYVLPNFEVk.[I]	N-Term(iTRAQ4plex); K1(iTRAQ4	2	2
[K].scGLHQLLR.[G]	N-Term(iTRAQ4plex); C2(Carbam	2	2
[K].sHALQLNNR.[Q]	N-Term(iTRAQ4plex)	2	2
[R].kADGSYAAWLSR.[DG]	N-Term(iTRAQ4plex); K1(iTRAQ4	2	2
[K].yVLPNFEVk.[I]	N-Term(iTRAQ4plex); K9(iTRAQ4	2	2
[K].tEQWSTLPPETk.[D]	N-Term(iTRAQ4plex); K12(iTRAQ	2	2
[K].LVNGQSHISLSk.[A]	K12(iTRAQ4plex)	2	2
[K].TEQWSTLPPETk.[D]	K12(iTRAQ4plex)	2	2
[K].dHAVDLIQkGYMR.[I]	N-Term(iTRAQ4plex); K9(iTRAQ4	2	2
[R].lTVAAPPSGGPGFLSIERPDSRPPR.[V]	N-Term(iTRAQ4plex)	2	2
[K].vLSLAQEQVGGSPEk.[L]	N-Term(iTRAQ4plex); K15(iTRAQ	2	2
[K].ITQVLHFTk.[D]	K9(iTRAQ4plex)	2	2
[K].LGQYASPTAk.[R]	K10(iTRAQ4plex)	2	2
[R].QGSFQGGFR.[S]		2	2
[R].EELVYELNPLDHR.[G]		2	2
[R].RGHLFLQTDQPIYNPGQR.[V]		2	2
[R].EELVYELNPLDHR.[G]		2	2
[R].yRVFALDQk.[M]	N-Term(iTRAQ4plex); K9(iTRAQ4	2	2
[K].yVLPNFEVk.[I]	N-Term(iTRAQ4plex); K9(iTRAQ4	2	2
[R].yLDkTEQWSTLPPETk.[D]	N-Term(iTRAQ4plex); K4(iTRAQ4	2	2
[R].gSFEFPVGDAVSk.[V]	N-Term(iTRAQ4plex); K13(iTRAQ	2	2
[R].nNVPcSPk.[V]	N-Term(iTRAQ4plex); C5(Carbam	2	2
[R].VHYTVcIWR.[N]	C6(Carbamidomethyl)	2	2
[R].vEASISk.[A]	N-Term(iTRAQ4plex); K7(iTRAQ4	2	2
[R].nNVPcSPk.[V]	N-Term(iTRAQ4plex); C5(Carbam	2	2
[R].vEASISk.[A]	N-Term(iTRAQ4plex); K7(iTRAQ4	2	2
[R].tYNVLDMk.[N]	N-Term(iTRAQ4plex); K8(iTRAQ4	2	2
[K].scGLHQLLR.[G]	N-Term(iTRAQ4plex); C2(Carbam	2	2
[R].dkGQAGLQR.[A]	N-Term(iTRAQ4plex); K2(iTRAQ4	2	2
[R].tLEIPGNSDPNMIPDGDFNSYVR.[V]	N-Term(iTRAQ4plex)	2	2
[R].tLEIPGNSDPNMIPDGDFNSYVR.[V]	N-Term(iTRAQ4plex)	2	2
[K].yVLPNFEVk.[I]	N-Term(iTRAQ4plex); K9(iTRAQ4	2	2
[K].LNMGITDLQGLR.[L]		2	2
[K].lGQYASPTAk.[R]	N-Term(iTRAQ4plex); K10(iTRAQ	2	2
[R].VEASISk.[A]	K7(iTRAQ4plex)	2	2
[R].gLEEELQFSLGSk.[I]	N-Term(iTRAQ4plex); K13(iTRAQ	2	2
[R].gLQDEDGYR.[M]	N-Term(iTRAQ4plex)	2	2
[R].cSVFYGAPSkSR.[L]	C1(Carbamidomethyl); K10(iTRA	2	2
[K].TEQWSTLPPETk.[D]	K12(iTRAQ4plex)	2	2

[R].yLDkTEQWSTLPPETk.[D]	N-Term(iTRAQ4plex); K4(iTRAQ4	2	2
[R].kADGSYAAWLSR.[DG]	N-Term(iTRAQ4plex); K1(iTRAQ4	2	2
[K].tkGLcVATPVQLR.[V]	N-Term(iTRAQ4plex); K2(iTRAQ4	2	2
[K].lELSVDGAk.[Q]	N-Term(iTRAQ4plex); K9(iTRAQ4	2	2
[R].lTVAAPPSGGPGFLSIERPDSRPPR.[V]	N-Term(iTRAQ4plex)	2	2
[K].VDFTLSSER.[D]		2	2
[K].kYVLPNFEVk.[I]	K1(iTRAQ4plex); K10(iTRAQ4ple	2	2
[K].kYVLPNFEVk.[I]	N-Term(iTRAQ4plex); K10(iTRAQ	2	2
[K].kYVLPNFEVk.[I]	N-Term(iTRAQ4plex); K1(iTRAQ4	2	2
[R].tYNVLDMk.[N]	N-Term(iTRAQ4plex); K8(iTRAQ4	2	2
[R].fGLLDEDGkk.[T]	N-Term(iTRAQ4plex); K9(iTRAQ4	2	2
[R].eFHLHLR.[L]	N-Term(iTRAQ4plex)	2	2
[R].RGHLFLQTDQPIYNPGQR.[V]		2	2
[R].GLQDEDGYR.[M]		2	2
[K].vLSLAQEQVGGSPEk.[L]	N-Term(iTRAQ4plex); K15(iTRAQ	2	2
[R].YIYGkPVQGVAYVR.[F]	K5(iTRAQ4plex)	2	2
[R].vEASISk.[A]	N-Term(iTRAQ4plex); K7(iTRAQ4	2	2
[R].vEYGFQVk.[V]	N-Term(iTRAQ4plex); K8(iTRAQ4	2	2
[R].vFALDQk.[M]	N-Term(iTRAQ4plex); K7(iTRAQ4	2	2
[R].VHYTVcIWR.[N]	C6(Carbamidomethyl)	2	2
[K].lQETSNWLLSQQQADGSFQDPcPVLDR.[S	N-Term(iTRAQ4plex); C22(Carbar	1	1
[R].vFALDQk.[M]	N-Term(iTRAQ4plex); K7(iTRAQ4	2	2
[R].nNVPcSPk.[V]	N-Term(iTRAQ4plex); C5(Carbam	2	2
[R].dkGQAGLQR.[A]	N-Term(iTRAQ4plex); K2(iTRAQ4	2	2
[K].sHkPLNMGk.[V]	N-Term(iTRAQ4plex); K3(iTRAQ4	2	2
[R].gLQDEDGYR.[M]	N-Term(iTRAQ4plex)	2	2
[R].vEYGFQVk.[V]	N-Term(iTRAQ4plex); K8(iTRAQ4	2	2
[R].cSVFYGAPSk.[S]	C1(Carbamidomethyl); K10(iTRA	2	2
[K].vVEEQESR.[V]	N-Term(iTRAQ4plex)	2	2
[K].GLcVATPVQLR.[V]	C3(Carbamidomethyl)	2	2
[R].tLEIPGNSDPNmIPDGDFNSYVR.[V]	N-Term(iTRAQ4plex); M12(Oxida	2	2
[K].vLSLAQEQVGGSPEk.[L]	N-Term(iTRAQ4plex); K15(iTRAQ	2	2
[R].eELVYELNPLDHR.[G]	N-Term(iTRAQ4plex)	2	2
[R].QGSFQGGFR.[S]		2	2
[K].tEQWSTLPPETk.[D]	N-Term(iTRAQ4plex); K12(iTRAQ	2	2
[R].vEASISk.[A]	N-Term(iTRAQ4plex); K7(iTRAQ4	2	2
[K].ADGSYAAWLSR.[DG]		2	2
[R].gLQDEDGYR.[M]	N-Term(iTRAQ4plex)	2	2
[R].GLQDEDGYR.[M]		2	2
[R].gQVVkGSVFLR.[N]	N-Term(iTRAQ4plex); K5(iTRAQ4	2	2
[R].yLDkTEQWSTLPPETk.[D]	N-Term(iTRAQ4plex); K4(iTRAQ4	2	2
[R].dFALLSLQVPLkDAk.[S]	N-Term(iTRAQ4plex); K12(iTRAQ	2	2
[K].vVEEQESR.[V]	N-Term(iTRAQ4plex)	2	2
[R].aAcAQLNDFLQEYGTQGcQV.[-]	N-Term(iTRAQ4plex); C3(Carbam	2	2
[K].vVEEQESR.[V]	N-Term(iTRAQ4plex)	2	2
[R].eMSGSPASGIPVk.[V]	N-Term(iTRAQ4plex); K13(iTRAQ	2	2
[K].FAcYYPR.[V]	C3(Carbamidomethyl)	2	2
[R].vFALDQk.[M]	N-Term(iTRAQ4plex); K7(iTRAQ4	2	2
[K].vVEEQESR.[V]	N-Term(iTRAQ4plex)	2	2
[R].QGSFQGGFR.[S]		2	2
[K].vVEEQESR.[V]	N-Term(iTRAQ4plex)	2	2
[K].iTQVLHFTk.[D]	N-Term(iTRAQ4plex); K9(iTRAQ4	2	2

[R].kADGSYAAWLSR.[DG]	N-Term(iTRAQ4plex); K1(iTRAQ4	2	2
[R].vFALDQk.[M]	N-Term(iTRAQ4plex); K7(iTRAQ4	2	2
[K].qYRNGESVk.[L]	N-Term(iTRAQ4plex); K9(iTRAQ4	2	2
[R].VFALDQk.[M]	K7(iTRAQ4plex)	2	2
[R].gLESQTk.[L]	N-Term(iTRAQ4plex); K7(iTRAQ4	2	2
[R].dkGQAGLQR.[A]	N-Term(iTRAQ4plex); K2(iTRAQ4	2	2
[K].iTQVLHFTk.[D]	N-Term(iTRAQ4plex); K9(iTRAQ4	2	2
[R].gQIVFmNR.[E]	N-Term(iTRAQ4plex); M6(Oxidat	2	2
[R].GQIVFMNR.[E]		2	2
[K].sHALQLNNR.[Q]	N-Term(iTRAQ4plex)	2	2
[R].GSFEFPVGDAVSk.[V]	K13(iTRAQ4plex)	2	2
[R].VGDTLNLNLR.[A]		2	2
[R].YIYGkPVQGVAYVR.[F]	K5(iTRAQ4plex)	2	2
[R].gLQDEDGYR.[M]	N-Term(iTRAQ4plex)	2	2
[R].dFALLSLQVPLk.[D]	N-Term(iTRAQ4plex); K12(iTRAQ	2	2
[R].gPEVQLVAHSPWLk.[D]	N-Term(iTRAQ4plex); K14(iTRAQ	2	2
[K].iTQVLHFTk.[D]	N-Term(iTRAQ4plex); K9(iTRAQ4	2	2
[R].gLESQTk.[L]	N-Term(iTRAQ4plex); K7(iTRAQ4	2	2
[K].aDGSYAAWLSR.[DG]	N-Term(iTRAQ4plex)	2	2
[R].yIYGkPVQGVAYVR.[F]	N-Term(iTRAQ4plex); K5(iTRAQ4	2	2
[K].LQETSNWLLSQQQADGSFQDPcPVLDR.[	C22(Carbamidomethyl)	1	1
[K].vVEEQESR.[V]	N-Term(iTRAQ4plex)	2	2
[K].vDFTLSSER.[D]	N-Term(iTRAQ4plex)	2	2
[R].EELVYELNPLDHR.[G]		2	2
[K].lELSVDGAk.[Q]	N-Term(iTRAQ4plex); K9(iTRAQ4	2	2
[R].RGHLFLQTDQPIYNPGQR.[V]		2	2
[R].rFEQLELRPVLYNYLDk.[N]	N-Term(iTRAQ4plex); K17(iTRAQ	2	2
[R].dkGQAGLQR.[A]	N-Term(iTRAQ4plex); K2(iTRAQ4	2	2
[K].iTQVLHFTk.[D]	N-Term(iTRAQ4plex); K9(iTRAQ4	2	2
[R].eFHLHLR.[L]	N-Term(iTRAQ4plex)	2	2
[R].vEYGFQVk.[V]	N-Term(iTRAQ4plex); K8(iTRAQ4	2	2
[R].gLEEELQFSLGSk.[I]	N-Term(iTRAQ4plex); K13(iTRAQ	2	2
[R].tLEIPGNSDPNMIPDGDFNSYVR.[V]	N-Term(iTRAQ4plex)	2	2
[R].dkGQAGLQR.[A]	N-Term(iTRAQ4plex); K2(iTRAQ4	2	2
[K].sHkPLNmGk.[V]	N-Term(iTRAQ4plex); K3(iTRAQ4	2	2
[K].mRPSTDTITVMVENSHGLR.[V]	N-Term(iTRAQ4plex); M1(Oxidat	2	2
[R].gLESQTk.[L]	N-Term(iTRAQ4plex); K7(iTRAQ4	2	2
[K].TEQWSTLPPETk.[D]	K12(iTRAQ4plex)	2	2
[R].GPEVQLVAHSPWLk.[D]	K14(iTRAQ4plex)	2	2
[R].vEYGFQVk.[V]	N-Term(iTRAQ4plex); K8(iTRAQ4	2	2
[K].ScGLHQLLR.[G]	C2(Carbamidomethyl)	2	2
[R].gHLFLQTDQPIYNPGQR.[V]	N-Term(iTRAQ4plex)	2	2
[R].ePFLSccQFAESLR.[K]	N-Term(iTRAQ4plex); C6(Carbam	2	2
[R].gQIVFmNR.[E]	N-Term(iTRAQ4plex); M6(Oxidat	2	2
[R].eFHLHLR.[L]	N-Term(iTRAQ4plex)	2	2
[K].tEQWSTLPPETk.[D]	N-Term(iTRAQ4plex); K12(iTRAQ	2	2
[K].vLQIEkEGAIHR.[E]	N-Term(iTRAQ4plex); K6(iTRAQ4	2	2
[R].eELVYELNPLDHR.[G]	N-Term(iTRAQ4plex)	2	2
[R].cSVFYGAPSk.[S]	C1(Carbamidomethyl); K10(iTRA	2	2
[K].LNMGITDLQGLR.[L]		2	2
[R].VDVQAGAcEGk.[L]	C8(Carbamidomethyl); K11(iTRA	2	2
[K].tYLPAVDEk.[L]	N-Term(iTRAQ4plex); K9(iTRAQ4	1	1

[K].tYLPAVDEk.[L]	N-Term(iTRAQ4plex); K9(iTRAQ4	1	1
[K].tYLPAVDEk.[L]	N-Term(iTRAQ4plex); K9(iTRAQ4	1	1
[K].tAAQNLYEk.[T]	N-Term(iTRAQ4plex); K9(iTRAQ4	1	1
[K].tAAQNLYEk.[T]	N-Term(iTRAQ4plex); K9(iTRAQ4	1	1
[K].tAAQNLYEk.[T]	N-Term(iTRAQ4plex); K9(iTRAQ4	1	1
[K].TYLPAVDEk.[L]	K9(iTRAQ4plex)	1	1
[K].tYLPAVDEk.[L]	N-Term(iTRAQ4plex); K9(iTRAQ4	1	1
[K].ESLSSYWESAk.[T]	K11(iTRAQ4plex)	1	1
[K].eSLSSYWESAk.[T]	N-Term(iTRAQ4plex); K11(iTRAQ	1	1
[K].sTAAMSTYTGIFTDQVLSVLk.[G]	N-Term(iTRAQ4plex); K21(iTRAQ	1	1
[K].sTAAMSTYTGIFTDQVLSVLk.[G]	N-Term(iTRAQ4plex); K21(iTRAQ	1	1
[K].eSLSSYWESAk.[T]	N-Term(iTRAQ4plex); K11(iTRAQ	1	1
[K].eSLSSYWESAk.[T]	N-Term(iTRAQ4plex); K11(iTRAQ	1	1
[K].eSLSSYWESAk.[T]	N-Term(iTRAQ4plex); K11(iTRAQ	1	1
[K].tAAQNLYEk.[T]	N-Term(iTRAQ4plex); K9(iTRAQ4	1	1
[K].tYLPAVDEk.[L]	N-Term(iTRAQ4plex); K9(iTRAQ4	1	1
[K].tYLPAVDEk.[L]	N-Term(iTRAQ4plex); K9(iTRAQ4	1	1
[K].TAAQNLYEk.[T]	K9(iTRAQ4plex)	1	1
[K].eSLSSYWESAk.[T]	N-Term(iTRAQ4plex); K11(iTRAQ	1	1
[K].TAAQNLYEk.[T]	K9(iTRAQ4plex)	1	1
[K].tYLPAVDEk.[L]	N-Term(iTRAQ4plex); K9(iTRAQ4	1	1
[K].sTAAMSTYTGIFTDQVLSVLk.[G]	N-Term(iTRAQ4plex); K21(iTRAQ	1	1
[K].kGHIYQGSEADSVFSGFLIFPSA.[-]	N-Term(iTRAQ4plex); K1(iTRAQ4	1	1
[K].kGHIYQGSEADSVFSGFLIFPSA.[-]	N-Term(iTRAQ4plex); K1(iTRAQ4	1	1
[K].gHIYQGSEADSVFSGFLIFPSA.[-]	N-Term(iTRAQ4plex)	1	1
[K].gLFQVVSGGMVLQLQQGDQVWVEk.[D]	N-Term(iTRAQ4plex); K24(iTRAQ	1	1
[R].sLGFcDTTNk.[G]	N-Term(iTRAQ4plex); C5(Carbam	1	1
[K].eALQGVGDMGR.[A]	N-Term(iTRAQ4plex)	1	1
[R].gPGGVWAAk.[L]	N-Term(iTRAQ4plex); K9(iTRAQ4	1	1
[K].eALQGVGDmGR.[A]	N-Term(iTRAQ4plex); M9(Oxidat	1	1
[K].EALQGVGDMGR.[A]		1	1
[K].eALQGVGDMGR.[A]	N-Term(iTRAQ4plex)	1	1
[K].eALQGVGDmGR.[A]	N-Term(iTRAQ4plex); M9(Oxidat	1	1
[K].eALQGVGDMGR.[A]	N-Term(iTRAQ4plex)	1	1
[R].fRPDGLPk.[K]	N-Term(iTRAQ4plex); K8(iTRAQ4	1	1
[R].fRPDGLPk.[K]	N-Term(iTRAQ4plex); K8(iTRAQ4	1	1
[R].gPGGVWAAk.[L]	N-Term(iTRAQ4plex); K9(iTRAQ4	1	1
[R].gNYDAAQR.[G]	N-Term(iTRAQ4plex)	1	1
[R].fRPDGLPk.[K]	N-Term(iTRAQ4plex); K8(iTRAQ4	1	1
[R].fRPDGLPk.[K]	N-Term(iTRAQ4plex); K8(iTRAQ4	1	1
[R].gPGGVWAAk.[L]	N-Term(iTRAQ4plex); K9(iTRAQ4	1	1
[R].gPGGVWAAk.[L]	N-Term(iTRAQ4plex); K9(iTRAQ4	1	1
[K].lEQGENVFLQATDk.[N]	N-Term(iTRAQ4plex); K14(iTRAQ	1	1
[K].lEQGENVFLQATDk.[N]	N-Term(iTRAQ4plex); K14(iTRAQ	1	1
[R].gNLcVNLMR.[G]	N-Term(iTRAQ4plex); C4(Carbam	1	1
[R].fDHVITNMNNNYEPR.[S]	N-Term(iTRAQ4plex)	1	1
[K].vPGLYYFTYHASSR.[G]	N-Term(iTRAQ4plex)	1	1
[R].GNLcVNLMR.[G]	C4(Carbamidomethyl)	1	1
[R].gNLcVNLMR.[G]	N-Term(iTRAQ4plex); C4(Carbam	1	1
[K].vVGIPGcQTcR.[Y]	N-Term(iTRAQ4plex); C7(Carbam	1	1
[R].gNLVSIHNFNINYR.[I]	N-Term(iTRAQ4plex)	1	1
[R].gGHcVALcTR.[G]	N-Term(iTRAQ4plex); C4(Carbam	1	1

[R].iQcSVSALNQGQVWIGGR.[I]	N-Term(iTRAQ4plex); C3(Carbam	1	1
[R].iQcSVSALNQGQVWIGGR.[I]	N-Term(iTRAQ4plex); C3(Carbam	1	1
[R].GGHcVALcTR.[G]	C4(Carbamidomethyl); C8(Carba	1	1
[K].vVGIPGcQTcR.[Y]	N-Term(iTRAQ4plex); C7(Carbam	1	1
[R].GGHcVALcTR.[G]	C4(Carbamidomethyl); C8(Carba	1	1
[R].tYIYDHGFPYTDPVGVLYEDGDDTVATR.[S	N-Term(iTRAQ4plex)	1	1
[R].sTELcGLWQGR.[Q]	N-Term(iTRAQ4plex); C5(Carbam	1	1
[K].tYSVEYLDSSk.[L]	N-Term(iTRAQ4plex); K11(iTRAQ	1	1
[R].tYIYDHGFPYTDPVGVLYEDGDDTVATR.[S	N-Term(iTRAQ4plex)	1	1
[R].sSGLVSNAPGVQIR.[V]	N-Term(iTRAQ4plex)	1	1
[K].tYSVEYLDSSk.[L]	N-Term(iTRAQ4plex); K11(iTRAQ	1	1
[K].tYSVEYLDSSk.[L]	N-Term(iTRAQ4plex); K11(iTRAQ	1	1
[R].sSGLVSNAPGVQIR.[V]	N-Term(iTRAQ4plex)	1	1
[R].aLPGEQQPLHALTR.[G]	N-Term(iTRAQ4plex)	1	1
[K].aQETSGEEISk.[F]	N-Term(iTRAQ4plex); K11(iTRAQ	1	1
[K].aQETSGEEISk.[F]	N-Term(iTRAQ4plex); K11(iTRAQ	1	1
[K].aQETSGEEISk.[F]	N-Term(iTRAQ4plex); K11(iTRAQ	1	1
[K].AQETSGEEISK.[F]		1	1
[K].aQETSGEEISk.[F]	N-Term(iTRAQ4plex); K11(iTRAQ	1	1
[K].aLHVTNIk.[K]	N-Term(iTRAQ4plex); K8(iTRAQ4	1	1
[R].ALPGEQQPLHALTR.[G]		1	1
[K].aQETSGEEISk.[F]	N-Term(iTRAQ4plex); K11(iTRAQ	1	1
[R].vVESLAk.[A]	N-Term(iTRAQ4plex); K7(iTRAQ4	1	1
[R].aLPGEQQPLHALTR.[G]	N-Term(iTRAQ4plex)	1	1
[K].dIDEcDIVPDAck.[G]	N-Term(iTRAQ4plex); C5(Carbam	1	1
[R].tcQDINEcETTNEcR.[E]	N-Term(iTRAQ4plex); C2(Carbam	1	1
[R].lNcEDIDEcR.[T]	N-Term(iTRAQ4plex); C3(Carbam	1	1
[R].eLPQSIVYk.[Y]	N-Term(iTRAQ4plex); K9(iTRAQ4	1	1
[R].eLPQSIVYk.[Y]	N-Term(iTRAQ4plex); K9(iTRAQ4	1	1
[R].tcQDINEcETTNEcR.[E]	N-Term(iTRAQ4plex); C2(Carbam	1	1
[K].dIDEcDIVPDAck.[G]	N-Term(iTRAQ4plex); C5(Carbam	1	1
[R].eLPQSIVYk.[Y]	N-Term(iTRAQ4plex); K9(iTRAQ4	1	1
[R].gFMQTYYDDHLR.[D]	N-Term(iTRAQ4plex)	1	1
[R].gFMQTYYDDHLR.[D]	N-Term(iTRAQ4plex)	1	1
[K].eLLETVVNR.[T]	N-Term(iTRAQ4plex)	1	1
[K].aWFLESk.[D]	N-Term(iTRAQ4plex); K7(iTRAQ4	1	1
[K].aWFLESk.[D]	N-Term(iTRAQ4plex); K7(iTRAQ4	1	1
[K].aWFLESk.[D]	N-Term(iTRAQ4plex); K7(iTRAQ4	1	1
[R].dLGPLTk.[A]	N-Term(iTRAQ4plex); K7(iTRAQ4	1	1
[K].AWFLESk.[D]	K7(iTRAQ4plex)	1	1
[K].aWFLESk.[D]	N-Term(iTRAQ4plex); K7(iTRAQ4	1	1
[K].mVETALTPDAcYPD.[-]	N-Term(iTRAQ4plex); C11(Carbar	1	1
[R].sSEDPNEDIVER.[N]	N-Term(iTRAQ4plex)	1	1
[R].fVYHLSDLck.[K]	N-Term(iTRAQ4plex); C9(Carbam	1	1
[R].sSEDPNEDIVER.[N]	N-Term(iTRAQ4plex)	1	1
[R].iVLVDNk.[C]	N-Term(iTRAQ4plex); K7(iTRAQ4	1	1
[R].sSEDPNEDIVER.[N]	N-Term(iTRAQ4plex)	1	1
[R].fLEQQNQVLQTk.[W]	N-Term(iTRAQ4plex); K12(iTRAQ	2	3
[R].tAAENDFVTLk.[K]	N-Term(iTRAQ4plex); K11(iTRAQ	1	1
[R].HGGGGGGFGGGGFGSR.[S]		1	1
[K].gGSISGGGYGSGGGk.[H]	N-Term(iTRAQ4plex); K15(iTRAQ	1	1
[R].sLVGLGGTk.[S]	N-Term(iTRAQ4plex); K9(iTRAQ4	1	1

[R].FLEQQNQVLQTk.[W]	K12(iTRAQ4plex)	2	3
[R].eVTVPVFYPTEk.[V]	N-Term(iTRAQ4plex); K12(iTRAQ	1	1
[R].gATLALTQVTPQDER.[I]	N-Term(iTRAQ4plex)	1	1
[R].gPVLQLHDLk.[R]	N-Term(iTRAQ4plex); K10(iTRAQ	1	1
[R].eETGQVLER.[G]	N-Term(iTRAQ4plex)	1	1
[R].gATLALTQVTPQDER.[I]	N-Term(iTRAQ4plex)	1	1
[K].nTcNHDEDTWVEcEDPFDLR.[L]	N-Term(iTRAQ4plex); C3(Carbam	1	1
[R].lADGPGHck.[G]	N-Term(iTRAQ4plex); C8(Carbam	1	1
[K].eDQVVck.[Q]	N-Term(iTRAQ4plex); C6(Carbam	1	1
[R].eATLQDcPSGPWGk.[N]	N-Term(iTRAQ4plex); C7(Carbam	1	1
[R].kEGGLGPLNIPLLADVTR.[R]	N-Term(iTRAQ4plex); K1(iTRAQ4	1	1
[R].qITVNDLPVGR.[S]	N-Term(iTRAQ4plex)	1	2
[R].qITVNDLPVGR.[S]	N-Term(iTRAQ4plex)	1	2
[R].gLFIIDGk.[G]	N-Term(iTRAQ4plex); K8(iTRAQ4	1	1
[R].iGkPAPDFk.[A]	N-Term(iTRAQ4plex); K3(iTRAQ4	1	1
[K].yEVQGEVFTkPQLWP.[-]	N-Term(iTRAQ4plex); K10(iTRAQ	1	1
[K].rQDNEILIFWSk.[D]	N-Term(iTRAQ4plex); K12(iTRAQ	1	1
[R].gYSIFSYATk.[R]	N-Term(iTRAQ4plex); K10(iTRAQ	1	1
[K].aPLTkPLk.[A]	N-Term(iTRAQ4plex); K5(iTRAQ4	1	1
[R].gYSIFSYATk.[R]	N-Term(iTRAQ4plex); K10(iTRAQ	1	1
[K].vGLSDAFVVVHR.[I]	N-Term(iTRAQ4plex)	1	1
[R].qDWVDSGcPEESk.[E]	N-Term(iTRAQ4plex); C8(Carbam	1	1
[R].aSVGQDSPEPR.[S]	N-Term(iTRAQ4plex)	1	1
[K].iHGILSNTHR.[Q]	N-Term(iTRAQ4plex)	1	1
[R].aSVGQDSPEPR.[S]	N-Term(iTRAQ4plex)	1	1
[R].vYALPEDLVEVNPk.[M]	N-Term(iTRAQ4plex); K14(iTRAQ	1	1
[R].nEALIALLR.[E]	N-Term(iTRAQ4plex)	1	1
[K].aYYHLLEQVAPk.[G]	N-Term(iTRAQ4plex); K12(iTRAQ	1	1
[R].eGESLEDLMk.[L]	N-Term(iTRAQ4plex); K10(iTRAQ	1	1
[K].eITALAPSTMk.[I]	N-Term(iTRAQ4plex); K11(iTRAQ	1	6
[R].hQGVMVGMGQk.[D]	N-Term(iTRAQ4plex); K11(iTRAQ	1	7
[KR].sYELPDGQVITIGNER.[F]	N-Term(iTRAQ4plex)	1	10
[K].IWHHTFYNELR.[V]		1	9
[K].eITALAPSTMk.[I]	N-Term(iTRAQ4plex); K11(iTRAQ	1	6
[R].hQGVMVGMGQk.[D]	N-Term(iTRAQ4plex); K11(iTRAQ	1	7
[KR].sYELPDGQVITIGNER.[F]	N-Term(iTRAQ4plex)	1	10
[K].IWHHTFYNELR.[V]		1	9
[K].vLATLcGQESTDTER.[A]	N-Term(iTRAQ4plex); C6(Carbam	1	1
[R].lASPGFPGEYANDQER.[R]	N-Term(iTRAQ4plex)	1	1
[R].rWTLTAPPGYR.[L]	N-Term(iTRAQ4plex)	1	1
[K].wPEPVFGR.[L]	N-Term(iTRAQ4plex)	1	1
[K].iTGFLkPGk.[V]	N-Term(iTRAQ4plex); K6(iTRAQ4	1	1
[R].iTLPDFTGDLR.[I]	N-Term(iTRAQ4plex)	1	1
[K].sFRPFVPR.[L]	N-Term(iTRAQ4plex)	1	1
[R].vQLYDLGLQIHk.[D]	N-Term(iTRAQ4plex); K12(iTRAQ	1	1
[R].eNSLLFDPLSSSSSNk.[E]	N-Term(iTRAQ4plex); K16(iTRAQ	1	1
[R].kIQTQLQR.[D]	N-Term(iTRAQ4plex); K1(iTRAQ4	1	1
[R].kVVATTQMQAADAR.[K]	K1(iTRAQ4plex)	1	1
[R].kVVATTQMQAADAR.[K]	N-Term(iTRAQ4plex)	1	1
[K].iQTQLQR.[D]	N-Term(iTRAQ4plex)	1	1
[K].dcVGDVTENQIcNk.[Q]	N-Term(iTRAQ4plex); C2(Carbam	1	1
[R].tIVTTLQDSIR.[K]	N-Term(iTRAQ4plex)	1	1

[R].tcHIQEcDk.[R]	N-Term(iTRAQ4plex); C2(Carbam	1	1
[R].lcNNPTPQFGGk.[D]	N-Term(iTRAQ4plex); C2(Carbam	1	1
[K].nWGLSVYADkPETTk.[E]	N-Term(iTRAQ4plex); K10(iTRAQ	1	1
[K].nWGLSVYADkPETTk.[E]	N-Term(iTRAQ4plex); K10(iTRAQ	1	1
[K].nWGLSVYADkPETTk.[E]	N-Term(iTRAQ4plex); K10(iTRAQ	1	1
[K].nWGLSVYADkPETTk.[E]	N-Term(iTRAQ4plex); K10(iTRAQ	1	1
[K].nWGLSVYADkPETTk.[E]	N-Term(iTRAQ4plex); K10(iTRAQ	1	1
[K].eQLGEFYEALDcLR.[I]	N-Term(iTRAQ4plex); C12(Carbar	1	1
[K].eQLGEFYEALDcLR.[I]	N-Term(iTRAQ4plex); C12(Carbar	1	1
[K].eQLGEFYEALDcLR.[I]	N-Term(iTRAQ4plex); C12(Carbar	1	1
[K].EQLGEFYEALDcLR.[I]	C12(Carbamidomethyl)	1	1
[K].tYmLAFDVNDEk.[N]	N-Term(iTRAQ4plex); M3(Oxidat	1	1
[K].tYMLAFDVNDEk.[N]	N-Term(iTRAQ4plex); K12(iTRAQ	1	1
[K].tYMLAFDVNDEk.[N]	N-Term(iTRAQ4plex); K12(iTRAQ	1	1
[K].tYMLAFDVNDEk.[N]	N-Term(iTRAQ4plex); K12(iTRAQ	1	1
[R].yVGGQEHFAHLLILR.[D]	N-Term(iTRAQ4plex)	1	1
[K].eQLGEFYEALDcLR.[I]	N-Term(iTRAQ4plex); C12(Carbar	1	1
[K].TEDTIFLR.[E]		2	2
[K].wFYIASAFR.[N]	N-Term(iTRAQ4plex)	2	2
[K].sDVVYTDWk.[K]	N-Term(iTRAQ4plex); K9(iTRAQ4	1	1
[R].yVGGQEHFAHLLILR.[D]	N-Term(iTRAQ4plex)	1	1
[R].yVGGQEHFAHLLILR.[D]	N-Term(iTRAQ4plex)	1	1
[R].yVGGQEHFAHLLILR.[D]	N-Term(iTRAQ4plex)	1	1
[R].yVGGQEHFAHLLILR.[D]	N-Term(iTRAQ4plex)	1	1
[K].dkcEPLEk.[Q]	N-Term(iTRAQ4plex); K2(iTRAQ4	2	2
[K].TEDTIFLR.[E]		2	2
[K].dkcEPLEk.[Q]	N-Term(iTRAQ4plex); K2(iTRAQ4	2	2
[K].dkcEPLEk.[Q]	N-Term(iTRAQ4plex); K2(iTRAQ4	2	2
[K].eQLGEFYEALDcLR.[I]	N-Term(iTRAQ4plex); C12(Carbar	1	1
[K].dkcEPLEk.[Q]	N-Term(iTRAQ4plex); K2(iTRAQ4	2	2
[K].dkcEPLEk.[Q]	N-Term(iTRAQ4plex); K2(iTRAQ4	2	2
[K].SDVVYTDWk.[K]	K9(iTRAQ4plex)	1	1
[K].wFYIASAFR.[N]	N-Term(iTRAQ4plex)	2	2
[K].NWGLSVYADkPETTk.[E]	K10(iTRAQ4plex); K15(iTRAQ4pl	1	1
[K].dkcEPLEk.[Q]	N-Term(iTRAQ4plex); K2(iTRAQ4	2	2
[K].sDVVYTDWk.[K]	N-Term(iTRAQ4plex); K9(iTRAQ4	1	1
[K].NWGLSVYADkPETTk.[E]	K10(iTRAQ4plex); K15(iTRAQ4pl	1	1
[K].NWGLSVYADkPETTk.[E]	K10(iTRAQ4plex); K15(iTRAQ4pl	1	1
[K].DkcEPLEk.[Q]	K2(iTRAQ4plex); C3(Carbamidon	2	2
[K].sDVVYTDWk.[K]	N-Term(iTRAQ4plex); K9(iTRAQ4	1	1
[K].WFYIASAFR.[N]		2	2
[R].YVGGQEHFAHLLILR.[D]		1	1
[K].SDVVYTDWk.[K]	K9(iTRAQ4plex)	1	1
[R].yVGGQEHFAHLLILR.[D]	N-Term(iTRAQ4plex)	1	1
[K].dKcEPLEk.[Q]	N-Term(iTRAQ4plex); C3(Carbam	2	2
[K].eQLGEFYEALDcLR.[I]	N-Term(iTRAQ4plex); C12(Carbar	1	1
[K].NWGLSVYADkPETTk.[E]	K10(iTRAQ4plex); K15(iTRAQ4pl	1	1
[R].yVGGQEHFAHLLILR.[D]	N-Term(iTRAQ4plex)	1	1
[KR].sTSGGTAALGcLVk.[D]	N-Term(iTRAQ4plex); C11(Carbar	2	2
[KR].sTSGGTAALGcLVk.[D]	N-Term(iTRAQ4plex); C11(Carbar	2	2
[K].gPSVFPLAPSSk.[S]	N-Term(iTRAQ4plex); K12(iTRAQ	1	1
[K].gPSVFPLAPSSk.[S]	N-Term(iTRAQ4plex); K12(iTRAQ	1	1

[K].gPSVFPLAPSSk.[S]	N-Term(iTRAQ4plex); K12(iTRAQ	1	1
[K].fNWYVDGVEVHNAk.[T]	N-Term(iTRAQ4plex); K14(iTRAQ	1	1
[K].fNWYVDGVEVHNAk.[T]	N-Term(iTRAQ4plex); K14(iTRAQ	1	1
[K].nQVSLTcLVk.[G]	N-Term(iTRAQ4plex); C7(Carbam	4	4
[K].nQVSLTcLVk.[G]	N-Term(iTRAQ4plex); C7(Carbam	4	4
[K].tTPPVLDSDGSFFLYSk.[L]	N-Term(iTRAQ4plex); K17(iTRAQ	1	1
[K].tHTcPPcPAPELLGGPSVFLFPPkPk.[D]	N-Term(iTRAQ4plex); C4(Carbam	1	1
[K].tHTcPPcPAPELLGGPSVFLFPPkPk.[D]	N-Term(iTRAQ4plex); C4(Carbam	1	1
[K].tHTcPPcPAPELLGGPSVFLFPPkPk.[D]	N-Term(iTRAQ4plex); C4(Carbam	1	1
[R].tPEVTcVVVDVSHEDPEVk.[F]	N-Term(iTRAQ4plex); C6(Carbam	1	1
[R].tPEVTcVVVDVSHEDPEVk.[F]	N-Term(iTRAQ4plex); C6(Carbam	1	1
[K].aLPAPIEk.[T]	N-Term(iTRAQ4plex); K8(iTRAQ4	2	2
[R].ePQVYTLPPSR.[DE]	N-Term(iTRAQ4plex)	3	3
[R].ePQVYTLPPSR.[DE]	N-Term(iTRAQ4plex)	3	3
[R].ePQVYTLPPSR.[DE]	N-Term(iTRAQ4plex)	3	3
[R].ePQVYTLPPSR.[DE]	N-Term(iTRAQ4plex)	3	3
[R].ePQVYTLPPSR.[DE]	N-Term(iTRAQ4plex)	3	3
[K].THTcPPcPAPELLGGPSVFLFPPkPk.[D]	C4(Carbamidomethyl); C7(Carba	1	1
[R].ePQVYTLPPSR.[DE]	N-Term(iTRAQ4plex)	3	3
[R].ePQVYTLPPSR.[DE]	N-Term(iTRAQ4plex)	3	3
[KR].sTSGGTAALGcLVk.[D]	N-Term(iTRAQ4plex); C11(Carbar	2	2
[K].tTPPVLDSDGSFFLYSk.[L]	N-Term(iTRAQ4plex); K17(iTRAQ	1	1
[K].aLPAPIEk.[T]	N-Term(iTRAQ4plex); K8(iTRAQ4	2	2
[K].GPSVFPLAPSSk.[S]	K12(iTRAQ4plex)	1	1
[K].tTPPVLDSDGSFFLYSk.[L]	N-Term(iTRAQ4plex); K17(iTRAQ	1	1
[K].tTPPVLDSDGSFFLYSk.[L]	N-Term(iTRAQ4plex); K17(iTRAQ	1	1
[K].TTPPVLDSDGSFFLYSk.[L]	K17(iTRAQ4plex)	1	1
[K].fNWYVDGVEVHNAk.[T]	N-Term(iTRAQ4plex); K14(iTRAQ	1	1
[K].DTLmISR.[T]	M4(Oxidation)	4	4
[K].tTPPVLDSDGSFFLYSk.[L]	N-Term(iTRAQ4plex); K17(iTRAQ	1	1
[K].gPSVFPLAPSSk.[S]	N-Term(iTRAQ4plex); K12(iTRAQ	1	1
[R].tPEVTcVVVDVSHEDPEVk.[F]	N-Term(iTRAQ4plex); C6(Carbam	1	1
[R].ePQVYTLPPSR.[DE]	N-Term(iTRAQ4plex)	3	3
[K].tLmFGSYLDDEk.[N]	N-Term(iTRAQ4plex); M3(Oxidat	1	1
[K].tLMFGSYLDDEk.[N]	N-Term(iTRAQ4plex); K12(iTRAQ	1	1
[K].tLMFGSYLDDEk.[N]	N-Term(iTRAQ4plex); K12(iTRAQ	1	1
[K].tLMFGSYLDDEk.[N]	N-Term(iTRAQ4plex); K12(iTRAQ	1	1
[R].eHVAHLLFLR.[D]	N-Term(iTRAQ4plex)	1	1
[R].eHVAHLLFLR.[D]	N-Term(iTRAQ4plex)	1	1
[R].eHVAHLLFLR.[D]	N-Term(iTRAQ4plex)	1	1
[R].eHVAHLLFLR.[D]	N-Term(iTRAQ4plex)	1	1
[R].eHVAHLLFLR.[D]	N-Term(iTRAQ4plex)	1	1
[K].eQLGEFYEALDcLcIPR.[S]	N-Term(iTRAQ4plex); C12(Carbar	1	1
[K].eQLGEFYEALDcLcIPR.[S]	N-Term(iTRAQ4plex); C12(Carbar	1	1
[K].nWGLSFYADkPETTk.[E]	N-Term(iTRAQ4plex); K10(iTRAQ	1	1
[K].nWGLSFYADkPETTk.[E]	N-Term(iTRAQ4plex); K10(iTRAQ	1	1
[K].nWGLSFYADkPETTk.[E]	N-Term(iTRAQ4plex); K10(iTRAQ	1	1
[K].eQLGEFYEALDcLcIPR.[S]	N-Term(iTRAQ4plex); C12(Carbar	1	1
[K].eQLGEFYEALDcLcIPR.[S]	N-Term(iTRAQ4plex); C12(Carbar	1	1
[K].TEDTIFLR.[E]		2	2
[K].wFYIASAFR.[N]	N-Term(iTRAQ4plex)	2	2
[K].dkcEPLEk.[Q]	N-Term(iTRAQ4plex); K2(iTRAQ4	2	2

[K].TEDTIFLR.[E]		2	2
[K].dkcEPLEk.[Q]	N-Term(iTRAQ4plex); K2(iTRAQ4	2	2
[R].EHVAHLLFLR.[D]		1	1
[K].dkcEPLEk.[Q]	N-Term(iTRAQ4plex); K2(iTRAQ4	2	2
[K].tLmFGSYLDDEk.[N]	N-Term(iTRAQ4plex); M3(Oxidat	1	1
[K].dkcEPLEk.[Q]	N-Term(iTRAQ4plex); K2(iTRAQ4	2	2
[R].eHVAHLLFLR.[D]	N-Term(iTRAQ4plex)	1	1
[K].dkcEPLEk.[Q]	N-Term(iTRAQ4plex); K2(iTRAQ4	2	2
[K].tLMFGSYLDDEk.[N]	N-Term(iTRAQ4plex); K12(iTRAQ	1	1
[K].wFYIASAFR.[N]	N-Term(iTRAQ4plex)	2	2
[K].dkcEPLEk.[Q]	N-Term(iTRAQ4plex); K2(iTRAQ4	2	2
[K].tLmFGSYLDDEk.[N]	N-Term(iTRAQ4plex); M3(Oxidat	1	1
[K].DkcEPLEk.[Q]	K2(iTRAQ4plex); C3(Carbamidon	2	2
[K].WFYIASAFR.[N]		2	2
[R].eHVAHLLFLR.[D]	N-Term(iTRAQ4plex)	1	1
[R].sDVMYTDWk.[K]	N-Term(iTRAQ4plex); K9(iTRAQ4	1	1
[R].eHVAHLLFLR.[D]	N-Term(iTRAQ4plex)	1	1
[K].dKcEPLEk.[Q]	N-Term(iTRAQ4plex); C3(Carbam	2	2
[K].vYAcEVTHQGLSSPVTk.[S]	N-Term(iTRAQ4plex); C4(Carbam	1	1
[K].vYAcEVTHQGLSSPVTk.[S]	N-Term(iTRAQ4plex); C4(Carbam	1	1
[-].tVAAPSVFIFPPSDEQLk.[S]	N-Term(iTRAQ4plex); K18(iTRAQ	1	1
[-].tVAAPSVFIFPPSDEQLk.[S]	N-Term(iTRAQ4plex); K18(iTRAQ	1	1
[-].tVAAPSVFIFPPSDEQLk.[S]	N-Term(iTRAQ4plex); K18(iTRAQ	1	1
[-].tVAAPSVFIFPPSDEQLk.[S]	N-Term(iTRAQ4plex); K18(iTRAQ	1	1
[K].dSTYSLSSTLTLSk.[A]	N-Term(iTRAQ4plex); K14(iTRAQ	1	1
[K].dSTYSLSSTLTLSk.[A]	N-Term(iTRAQ4plex); K14(iTRAQ	1	1
[-].TVAAPSVFIFPPSDEQLk.[S]	K18(iTRAQ4plex)	1	1
[K].sGTASVVcLLNNFYPR.[E]	N-Term(iTRAQ4plex); C8(Carbam	1	1
[K].sGTASVVcLLNNFYPR.[E]	N-Term(iTRAQ4plex); C8(Carbam	1	1
[K].sGTASVVcLLNNFYPR.[E]	N-Term(iTRAQ4plex); C8(Carbam	1	1
[K].vDNALQSGNSQESVTEQDSk.[D]	N-Term(iTRAQ4plex); K20(iTRAQ	1	1
[K].vDNALQSGNSQESVTEQDSk.[D]	N-Term(iTRAQ4plex); K20(iTRAQ	1	1
[K].vDNALQSGNSQESVTEQDSk.[D]	N-Term(iTRAQ4plex); K20(iTRAQ	1	1
[-].TVAAPSVFIFPPSDEQLk.[S]	K18(iTRAQ4plex)	1	1
[K].vYAcEVTHQGLSSPVTk.[S]	N-Term(iTRAQ4plex); C4(Carbam	1	1
[K].vYAcEVTHQGLSSPVTk.[S]	N-Term(iTRAQ4plex); C4(Carbam	1	1
[K].vDNALQSGNSQESVTEQDSk.[D]	N-Term(iTRAQ4plex); K20(iTRAQ	1	1
[K].dSTYSLSSTLTLSk.[A]	N-Term(iTRAQ4plex); K14(iTRAQ	1	1
[K].dSTYSLSSTLTLSk.[A]	N-Term(iTRAQ4plex); K14(iTRAQ	1	1
[K].dSTYSLSSTLTLSk.[A]	N-Term(iTRAQ4plex); K14(iTRAQ	1	1
[K].vDNALQSGNSQESVTEQDSk.[D]	N-Term(iTRAQ4plex); K20(iTRAQ	1	1
[-].TVAAPSVFIFPPSDEQLk.[S]	K18(iTRAQ4plex)	1	1
[K].tPLTATLSk.[S]	N-Term(iTRAQ4plex); K9(iTRAQ4	1	1
[K].TPLTATLSk.[S]	K9(iTRAQ4plex)	1	1
[R].qEPSQGTTTFAVTSILR.[V]	N-Term(iTRAQ4plex)	2	2
[R].qEPSQGTTTFAVTSILR.[V]	N-Term(iTRAQ4plex)	2	2
[K].kGDTFScMVGHEALPLAFTQk.[T]	N-Term(iTRAQ4plex); K1(iTRAQ4	2	2
[R].dASGVTFTWTPSSGk.[S]	N-Term(iTRAQ4plex); K15(iTRAQ	1	1
[R].wLQGSQELPR.[E]	N-Term(iTRAQ4plex)	2	2
[R].dLcGcYSVSSVLPGcAEPWNHGk.[T]	N-Term(iTRAQ4plex); C3(Carbam	1	1
[R].nFPPSQDASGDLYTTSSQLTLPATQcLAGk	N-Term(iTRAQ4plex); C26(Carbar	1	1
[R].nFPPSQDASGDLYTTSSQLTLPATQcLAGk	N-Term(iTRAQ4plex); C26(Carbar	1	1

[K].sAVQGPPER.[D]	N-Term(iTRAQ4plex)	2	2
[R].nFPPSQDASGDLYTTSSQLTLPATQcLAGk	N-Term(iTRAQ4plex); C26(Carbar	1	1
[K].sAVQGPPER.[D]	N-Term(iTRAQ4plex)	2	2
[K].kGDTFScMVGHEALPLAFTQk.[T]	N-Term(iTRAQ4plex); K1(iTRAQ4	2	2
[K].tPLTATLSk.[S]	N-Term(iTRAQ4plex); K9(iTRAQ4	1	1
[K].tFTcTAAYPESk.[T]	N-Term(iTRAQ4plex); C4(Carbam	1	1
[R].dASGVTFTWTPSSGk.[S]	N-Term(iTRAQ4plex); K15(iTRAQ	1	1
[K].sVTcHVk.[H]	N-Term(iTRAQ4plex); C4(Carbam	2	2
[RK].iDVcPENAEVTLTDFR.[A]	N-Term(iTRAQ4plex); C4(Carbam	2	2
[R].wFLQHRPQVGYIR.[V]	N-Term(iTRAQ4plex)	2	2
[K].dSDGDGIGDAcDNcPQk.[S]	N-Term(iTRAQ4plex); C11(Carbar	1	1
[R].dGVGDVcQDDFDADk.[V]	N-Term(iTRAQ4plex); C7(Carbam	1	1
[R].dGVGDVcQDDFDADk.[V]	N-Term(iTRAQ4plex); C7(Carbam	1	1
[R].dTDLDGFPDEk.[L]	N-Term(iTRAQ4plex); K11(iTRAQ	1	1
[R].dTDLDGFPDEk.[L]	N-Term(iTRAQ4plex); K11(iTRAQ	1	1
[R].dTDLDGFPDEk.[L]	N-Term(iTRAQ4plex); K11(iTRAQ	1	1
[R].aVAEPGIQLk.[A]	N-Term(iTRAQ4plex); K10(iTRAQ	2	2
[K].sSTGPGEQLR.[N]	N-Term(iTRAQ4plex)	1	1
[K].dSDGDGIGDAcDNcPQk.[S]	N-Term(iTRAQ4plex); C11(Carbar	1	1
[R].nALWHTGDTESQVR.[L]	N-Term(iTRAQ4plex)	1	1
[R].fNAVLTNPQGDYDTSTGk.[F]	N-Term(iTRAQ4plex); K18(iTRAQ	1	1
[R].fNAVLTNPQGDYDTSTGk.[F]	N-Term(iTRAQ4plex); K18(iTRAQ	1	1
[K].vVTFcGHTSk.[T]	N-Term(iTRAQ4plex); C5(Carbam	1	1
[K].vVTFcGHTSk.[T]	N-Term(iTRAQ4plex); C5(Carbam	1	1
[K].tNQVNSGGVLLR.[L]	N-Term(iTRAQ4plex)	1	1
[R].fNAVLTNPQGDYDTSTGk.[F]	N-Term(iTRAQ4plex); K18(iTRAQ	1	1
[R].fNAVLTNPQGDYDTSTGk.[F]	N-Term(iTRAQ4plex); K18(iTRAQ	1	1
[R].QTHQPPAPNSLIR.[F]		1	1
[R].qTHQPPAPNSLIR.[F]	N-Term(iTRAQ4plex)	1	1
[K].fQSVFTVTR.[Q]	N-Term(iTRAQ4plex)	1	1
[R].fNAVLTNPQGDYDTSTGk.[F]	N-Term(iTRAQ4plex); K18(iTRAQ	1	1
[R].QTHQPPAPNSLIR.[F]		1	1
[K].fQSVFTVTR.[Q]	N-Term(iTRAQ4plex)	1	1
[R].FNAVLTNPQGDYDTSTGk.[F]	K18(iTRAQ4plex)	1	1
[K].fQSVFTVTR.[Q]	N-Term(iTRAQ4plex)	1	1
[R].dTGTYGFLLPER.[Y]	N-Term(iTRAQ4plex)	1	1
[R].dTGTYGFLLPER.[Y]	N-Term(iTRAQ4plex)	1	1
[K].iHIGSSFEk.[Y]	N-Term(iTRAQ4plex); K9(iTRAQ4	1	1
[R].sFYANNHcIGTDLNR.[N]	N-Term(iTRAQ4plex); C8(Carbam	1	1
[K].nAIWIDcGIHAR.[E]	N-Term(iTRAQ4plex); C7(Carbam	1	1
[K].nAIWIDcGIHAR.[E]	N-Term(iTRAQ4plex); C7(Carbam	1	1
[K].skDHEELSLVASEAVR.[A]	N-Term(iTRAQ4plex); K2(iTRAQ4	1	1
[K].skDHEELSLVASEAVR.[A]	N-Term(iTRAQ4plex); K2(iTRAQ4	1	1
[K].skDHEELSLVASEAVR.[A]	N-Term(iTRAQ4plex); K2(iTRAQ4	1	1
[R].dTGTYGFLLPER.[Y]	N-Term(iTRAQ4plex)	1	1
[K].iHIGSSFEk.[Y]	N-Term(iTRAQ4plex); K9(iTRAQ4	1	1
[R].qRPEVFSDNMFcVGDETQR.[H]	N-Term(iTRAQ4plex); C12(Carbar	1	1
[R].gGGALLGDR.[W]	N-Term(iTRAQ4plex)	2	2
[K].vLSYVDWIk.[G]	N-Term(iTRAQ4plex); K9(iTRAQ4	1	1
[R].wILTAAHTIYPk.[DE]	N-Term(iTRAQ4plex); K12(iTRAQ	1	1
[R].wILTAAHTIYPk.[DE]	N-Term(iTRAQ4plex); K12(iTRAQ	1	1
[R].eAcNAWLQk.[R]	N-Term(iTRAQ4plex); C3(Carbam	1	1

[R].vVVHPDYR.[Q]	N-Term(iTRAQ4plex)	1	1
[R].gSEAINAPGDNPAk.[V]	N-Term(iTRAQ4plex); K14(iTRAQ	1	1
[R].vVVHPDYR.[Q]	N-Term(iTRAQ4plex)	1	1
[R].wILTAAHTIYPk.[DE]	N-Term(iTRAQ4plex); K12(iTRAQ	1	1
[R].wILTAAHTIYPk.[DE]	N-Term(iTRAQ4plex); K12(iTRAQ	1	1
[R].nPNLPPETVDSLk.[N]	N-Term(iTRAQ4plex); K13(iTRAQ	1	1
[R].nPNLPPETVDSLk.[N]	N-Term(iTRAQ4plex); K13(iTRAQ	1	1
[R].nPNLPPETVDSLk.[N]	N-Term(iTRAQ4plex); K13(iTRAQ	1	1
[R].nPNLPPETVDSLk.[N]	N-Term(iTRAQ4plex); K13(iTRAQ	1	1
[R].nPNLPPETVDSLk.[N]	N-Term(iTRAQ4plex); K13(iTRAQ	1	1
[R].nPNLPPETVDSLk.[N]	N-Term(iTRAQ4plex); K13(iTRAQ	1	1
[K].cPNPPVQENFDVNk.[Y]	N-Term(iTRAQ4plex); C1(Carbam	1	1
[R].nPNLPPETVDSLk.[N]	N-Term(iTRAQ4plex); K13(iTRAQ	1	1
[K].nILTSNNIDVk.[K]	N-Term(iTRAQ4plex); K11(iTRAQ	1	1
[R].nPNLPPETVDSLk.[N]	N-Term(iTRAQ4plex); K13(iTRAQ	1	1
[R].nPNLPPETVDSLk.[N]	N-Term(iTRAQ4plex); K13(iTRAQ	1	1
[K].mTVTDQVNcPk.[L]	N-Term(iTRAQ4plex); M1(Oxidat	1	1
[K].cPNPPVQENFDVNk.[Y]	N-Term(iTRAQ4plex); C1(Carbam	1	1
[K].nILTSNNIDVk.[K]	N-Term(iTRAQ4plex); K11(iTRAQ	1	1
[R].nPNLPPETVDSLk.[N]	N-Term(iTRAQ4plex); K13(iTRAQ	1	1
[K].kMTVTDQVNcPk.[L]	N-Term(iTRAQ4plex); K1(iTRAQ4	1	1
[R].nPNLPPETVDSLk.[N]	N-Term(iTRAQ4plex); K13(iTRAQ	1	1
[K].nILTSNNIDVk.[K]	N-Term(iTRAQ4plex); K11(iTRAQ	1	1
[R].nPNLPPETVDSLk.[N]	N-Term(iTRAQ4plex); K13(iTRAQ	1	1
[K].vQVLLGAHSLSQPEPSk.[R]	N-Term(iTRAQ4plex); K17(iTRAQ	1	1
[R].dVAPGTLcDVAGWGIVNHAGR.[R]	N-Term(iTRAQ4plex); C8(Carbam	1	1
[R].rPDSLQHVLLPVLDR.[A]	N-Term(iTRAQ4plex)	1	1
[R].rPDSLQHVLLPVLDR.[A]	N-Term(iTRAQ4plex)	1	1
[R].rPDSLQHVLLPVLDR.[A]	N-Term(iTRAQ4plex)	1	1
[R].aVPHPDSQPDTIDHDLLLLQLSEk.[A]	N-Term(iTRAQ4plex); K24(iTRAQ	1	1
[K].vQVLLGAHSLSQPEPSk.[R]	N-Term(iTRAQ4plex); K17(iTRAQ	1	1
[K].vQVLLGAHSLSQPEPSkR.[L]	N-Term(iTRAQ4plex); K17(iTRAQ	1	1
[R].wDLQHPHQHPFEPGk.[F]	N-Term(iTRAQ4plex); K15(iTRAQ	1	2
[R].wDLQHPHQHPFEPGk.[F]	N-Term(iTRAQ4plex); K15(iTRAQ	1	2
[R].wDLQHPHQHPFEPGk.[F]	N-Term(iTRAQ4plex); K15(iTRAQ	1	2
[R].gTELQHLLHAVVPGPWQEDVADAEEcAGI	N-Term(iTRAQ4plex); C26(Carbar	1	2
[K].eQWILTAR.[Q]	N-Term(iTRAQ4plex)	1	2
[K].eQWILTAR.[Q]	N-Term(iTRAQ4plex)	1	2
[R].vSVFVDWIHk.[V]	N-Term(iTRAQ4plex); K10(iTRAQ	1	2
[R].nPDGSERPWcYTTDPQIER.[E]	N-Term(iTRAQ4plex); C10(Carbar	1	2
[K].wLTFSLGk.[Q]	N-Term(iTRAQ4plex); K8(iTRAQ4	1	1
[K].eEAFLGITDEk.[T]	N-Term(iTRAQ4plex); K11(iTRAQ	1	1
[K].sPDGDSSLAASER.[K]	N-Term(iTRAQ4plex)	1	1
[K].tEGQFVDLTGNR.[L]	N-Term(iTRAQ4plex)	1	1
[K].tEGQFVDLTGNR.[L]	N-Term(iTRAQ4plex)	1	1
[K].eEAFLGITDEk.[T]	N-Term(iTRAQ4plex); K11(iTRAQ	1	1
[K].sPDGDSSLAASER.[K]	N-Term(iTRAQ4plex)	1	1
[R].gLQGPPGk.[L]	N-Term(iTRAQ4plex); K8(iTRAQ4	1	1
[K].hDTSLkPISVSYNPATAk.[E]	N-Term(iTRAQ4plex); K6(iTRAQ4	1	1
[K].aDGLAVIGVLMk.[V]	N-Term(iTRAQ4plex); K12(iTRAQ	1	1
[M].aSPDWGYDDk.[N]	N-Term(Prot)(Acetyl); K10(iTRAQ	1	1
[K].vLDALQAIk.[T]	N-Term(iTRAQ4plex); K9(iTRAQ4	1	1

[K].VLDALQAIk.[T]	K9(iTRAQ4plex)	1	1
[M].aSPDWGYDDk.[N]	N-Term(Prot)(Acetyl); K10(iTRAQ	1	1
[K].aDGLAVIGVLMk.[V]	N-Term(iTRAQ4plex); K12(iTRAQ	1	1
[K].eSISVSSEQLAQFR.[S]	N-Term(iTRAQ4plex)	1	1
[R].wEDILSDEVNVAR.[G]	N-Term(iTRAQ4plex)	1	1
[R].lYLQMSTHGk.[V]	N-Term(iTRAQ4plex); K10(iTRAQ	1	1
[R].eHGDPLIEELNPGDALEPEGR.[G]	N-Term(iTRAQ4plex)	1	1
[R].gVASLFAGR.[S]	N-Term(iTRAQ4plex)	1	1
[K].dEASSVEVTWPDGk.[M]	N-Term(iTRAQ4plex); K14(iTRAQ	1	1
[R].gSVQYLPDLDDk.[N]	N-Term(iTRAQ4plex); K12(iTRAQ	1	1
[K].aIFYMNNPSR.[V]	N-Term(iTRAQ4plex)	1	1
[R].aSSQWVVGPSYFVEYLIk.[E]	N-Term(iTRAQ4plex); K18(iTRAQ	1	1
[K].aIFYMNNPSR.[V]	N-Term(iTRAQ4plex)	1	1
[R].iFFESVYGQck.[A]	N-Term(iTRAQ4plex); C10(Carbar	1	1
[R].vNDAQEYR.[R]	N-Term(iTRAQ4plex)	1	1
[R].iFFESVYGQck.[A]	N-Term(iTRAQ4plex); C10(Carbar	1	1
[K].aIFYMNNPSR.[V]	N-Term(iTRAQ4plex)	1	1
[K].yVRPGGGFVPNFQLFEk.[G]	N-Term(iTRAQ4plex); K17(iTRAQ	1	1
[K].fLVGPDGIPIMR.[W]	N-Term(iTRAQ4plex)	1	1
[K].qEPGENSEILPTLk.[Y]	N-Term(iTRAQ4plex); K14(iTRAQ	1	1
[R].tTVSNVk.[M]	N-Term(iTRAQ4plex); K7(iTRAQ4	1	1
[K].fLVGPDGIPIMR.[W]	N-Term(iTRAQ4plex)	1	1
[K].yVRPGGGFVPNFQLFEk.[G]	N-Term(iTRAQ4plex); K17(iTRAQ	1	1
[K].mDILSYMR.[R]	N-Term(iTRAQ4plex)	1	1
[R].mHAAFGGTFk.[K]	N-Term(iTRAQ4plex); K10(iTRAQ	1	1
[R].fLEQELETITIPDLR.[G]	N-Term(iTRAQ4plex)	1	1
[R].aVEPQLQEEER.[M]	N-Term(iTRAQ4plex)	1	1
[R].gAFFPLTER.[N]	N-Term(iTRAQ4plex)	1	1
[R].sSVDELVGIDYSLMk.[D]	N-Term(iTRAQ4plex); K15(iTRAQ	1	1
[K].iTHVHVHMR.[Y]	N-Term(iTRAQ4plex)	1	1
[K].rAPDLQDLPWQVk.[L]	N-Term(iTRAQ4plex); K13(iTRAQ	1	1
[K].dFAEHLLIPR.[T]	N-Term(iTRAQ4plex)	1	1
[R].eNFVLTTAk.[C]	N-Term(iTRAQ4plex); K9(iTRAQ4	1	1
[R].eNFVLTTAk.[C]	N-Term(iTRAQ4plex); K9(iTRAQ4	1	1
[R].sSVAAMHWMDGSVVTR.[E]	N-Term(iTRAQ4plex)	1	1
[K].yIFSDDSSQLTIk.[K]	N-Term(iTRAQ4plex); K13(iTRAQ	1	1
[K].aGEQDATIHLk.[V]	N-Term(iTRAQ4plex); K11(iTRAQ	1	1
[K].gLGEISAASEFk.[T]	N-Term(iTRAQ4plex); K12(iTRAQ	1	1
[R].aVGEEVWHSk.[W]	N-Term(iTRAQ4plex); K10(iTRAQ	1	1
[R].vSSLTLk.[S]	N-Term(iTRAQ4plex); K7(iTRAQ4	1	1
[K].gLGEISAASEFk.[T]	N-Term(iTRAQ4plex); K12(iTRAQ	1	1
[K].sNLISGSVMYIEEk.[T]	N-Term(iTRAQ4plex); K14(iTRAQ	1	1
[K].lVcSGLLQASk.[S]	N-Term(iTRAQ4plex); C3(Carbam	1	1
[K].lFLSYDYAVk.[K]	N-Term(iTRAQ4plex); K10(iTRAQ	1	1
[K].iFSQETLTk.[A]	N-Term(iTRAQ4plex); K9(iTRAQ4	1	1
[R].tLLETLQk.[A]	N-Term(iTRAQ4plex); K8(iTRAQ4	1	1
[R].tLLETLQk.[A]	N-Term(iTRAQ4plex); K8(iTRAQ4	1	1
[K].sPYQLVLQHSR.[L]	N-Term(iTRAQ4plex)	1	1
[K].dGTPPIDAHTR.[N]	N-Term(iTRAQ4plex)	1	1
[R].kYFTNck.[Q]	N-Term(iTRAQ4plex); K1(iTRAQ4	1	1
[R].dLLNNHILk.[S]	N-Term(iTRAQ4plex); K9(iTRAQ4	1	1
[R].vLTDELk.[H]	N-Term(iTRAQ4plex); K7(iTRAQ4	1	1

[K].dGTPPIDAHTR.[N]	N-Term(iTRAQ4plex)	1	1
[K].dGTPPIDAHTR.[N]	N-Term(iTRAQ4plex)	1	1
[K].eITALAPSTMk.[I]	N-Term(iTRAQ4plex); K11(iTRAQ4	1	6
[R].hQGVMVGMGQk.[D]	N-Term(iTRAQ4plex); K11(iTRAQ4	1	7
[K].qEYDESGPSIVHR.[K]	N-Term(iTRAQ4plex)	1	7
[KR].sYELPDGQVITIGNER.[F]	N-Term(iTRAQ4plex)	1	10
[K].IWHHTFYNELR.[V]		1	9
[K].eITALAPSTMk.[I]	N-Term(iTRAQ4plex); K11(iTRAQ4	1	6
[R].hQGVMVGMGQk.[D]	N-Term(iTRAQ4plex); K11(iTRAQ4	1	7
[K].qEYDESGPSIVHR.[K]	N-Term(iTRAQ4plex)	1	7
[KR].sYELPDGQVITIGNER.[F]	N-Term(iTRAQ4plex)	1	10
[K].IWHHTFYNELR.[V]		1	9
[K].kPIIGILMQk.[C]	N-Term(iTRAQ4plex); K1(iTRAQ4	1	1
[K].kFFNVLTTNTDGk.[I]	N-Term(iTRAQ4plex); K1(iTRAQ4	1	1
[K].nLDGISHAPNAVk.[T]	N-Term(iTRAQ4plex); K13(iTRAQ4	1	1
[R].yYIAASYVk.[Y]	N-Term(iTRAQ4plex); K9(iTRAQ4	1	1
[K].dYEILFk.[S]	N-Term(iTRAQ4plex); K7(iTRAQ4	1	1
[K].lHVDPENFR.[L]	N-Term(iTRAQ4plex)	2	2
[K].vVAGVANALAHk.[Y]	N-Term(iTRAQ4plex); K12(iTRAQ4	2	2
[K].vVAGVANALAHk.[Y]	N-Term(iTRAQ4plex); K12(iTRAQ4	2	2
[K].vVAGVANALAHk.[Y]	N-Term(iTRAQ4plex); K12(iTRAQ4	2	2
[K].sAVTALWGk.[V]	N-Term(iTRAQ4plex); K9(iTRAQ4	1	1
[K].sAVTALWGk.[V]	N-Term(iTRAQ4plex); K9(iTRAQ4	1	1
[K].sAVTALWGk.[V]	N-Term(iTRAQ4plex); K9(iTRAQ4	1	1
[R].fFESFGDLSTPDAVMGNPk.[V]	N-Term(iTRAQ4plex); K19(iTRAQ4	1	1
[K].VVAGVANALAHk.[Y]	K12(iTRAQ4plex)	2	2
[M].vHLTPEEk.[TS]	N-Term(iTRAQ4plex); K8(iTRAQ4	2	2
[R].lLVVYPWTQR.[F]	N-Term(iTRAQ4plex)	2	5
[R].lLVVYPWTQR.[F]	N-Term(iTRAQ4plex)	2	5
[K].vLGAFSDGLAHLDNLk.[G]	N-Term(iTRAQ4plex); K16(iTRAQ4	2	2
[K].eFTPPVQAAYQk.[V]	N-Term(iTRAQ4plex); K12(iTRAQ4	1	1
[K].eFTPPVQAAYQk.[V]	N-Term(iTRAQ4plex); K12(iTRAQ4	1	1
[K].gTFATLSELHcDk.[L]	N-Term(iTRAQ4plex); C11(Carbar	1	1
[K].gTFATLSELHcDk.[L]	N-Term(iTRAQ4plex); C11(Carbar	1	1
[K].lHVDPENFR.[L]	N-Term(iTRAQ4plex)	2	2
[R].lLGNVLVcVLAHHFGk.[E]	N-Term(iTRAQ4plex); C8(Carbam	1	1
[K].VNVDEVGGEALGR.[L]		1	1
[K].vNVDEVGGEALGR.[L]	N-Term(iTRAQ4plex)	1	1
[K].lHVDPENFR.[L]	N-Term(iTRAQ4plex)	2	2
[R].lLGNVLVcVLAHHFGk.[E]	N-Term(iTRAQ4plex); C8(Carbam	1	1
[K].eFTPPVQAAYQk.[V]	N-Term(iTRAQ4plex); K12(iTRAQ4	1	1
[K].VVAGVANALAHk.[Y]	K12(iTRAQ4plex)	2	2
[R].LLVVYPWTQR.[F]		2	5
[K].eFTPPVQAAYQk.[V]	N-Term(iTRAQ4plex); K12(iTRAQ4	1	1
[K].eFTPPVQAAYQk.[V]	N-Term(iTRAQ4plex); K12(iTRAQ4	1	1
[K].dALSSVQESQVAQQAR.[G]	N-Term(iTRAQ4plex)	1	1
[K].tAkDALSSVQESQVAQQAR.[G]	N-Term(iTRAQ4plex); K3(iTRAQ4	1	1
[R].gWVTDGFSSLk.[D]	N-Term(iTRAQ4plex); K11(iTRAQ4	1	1
[R].gWVTDGFSSLk.[D]	N-Term(iTRAQ4plex); K11(iTRAQ4	1	1
[R].gWVTDGFSSLk.[D]	N-Term(iTRAQ4plex); K11(iTRAQ4	1	1
[R].gWVTDGFSSLk.[D]	N-Term(iTRAQ4plex); K11(iTRAQ4	1	1
[R].gWVTDGFSSLk.[D]	N-Term(iTRAQ4plex); K11(iTRAQ4	1	1

[R].gWVTDGFSSLk.[D]	N-Term(iTRAQ4plex); K11(iTRAQ	1	1
[R].gWVTDGFSSLk.[D]	N-Term(iTRAQ4plex); K11(iTRAQ	1	1
[R].gWVTDGFSSLk.[D]	N-Term(iTRAQ4plex); K11(iTRAQ	1	1
[K].fSEFWDLDPEVRPTSAVAA.[-]	N-Term(iTRAQ4plex)	1	1
[K].dALSSVQESQVAQQAR.[G]	N-Term(iTRAQ4plex)	1	1
[K].DALSSVQESQVAQQAR.[G]		1	1
[K].dALSSVQESQVAQQAR.[G]	N-Term(iTRAQ4plex)	1	1
[K].dALSSVQESQVAQQAR.[G]	N-Term(iTRAQ4plex)	1	1
[K].dALSSVQESQVAQQAR.[G]	N-Term(iTRAQ4plex)	1	1
[K].dALSSVQESQVAQQAR.[G]	N-Term(iTRAQ4plex)	1	1
[K].dYWSTVk.[D]	N-Term(iTRAQ4plex); K7(iTRAQ4	1	1
[R].gWVTDGFSSLk.[D]	N-Term(iTRAQ4plex); K11(iTRAQ	1	1
[R].gWVTDGFSSLk.[D]	N-Term(iTRAQ4plex); K11(iTRAQ	1	1
[R].GWVTDGFSSLk.[D]	K11(iTRAQ4plex)	1	1
[K].dkFSEFWDLDPEVRPTSAVAA.[-]	N-Term(iTRAQ4plex); K2(iTRAQ4	1	1
[R].gWVTDGFSSLk.[D]	N-Term(iTRAQ4plex); K11(iTRAQ	1	1
[K].dYWSTVk.[D]	N-Term(iTRAQ4plex); K7(iTRAQ4	1	1
[K].dALSSVQESQVAQQAR.[G]	N-Term(iTRAQ4plex)	1	1
[K].dYWSTVk.[D]	N-Term(iTRAQ4plex); K7(iTRAQ4	1	1
[K].dYWSTVk.[D]	N-Term(iTRAQ4plex); K7(iTRAQ4	1	1
[K].dYWSTVk.[D]	N-Term(iTRAQ4plex); K7(iTRAQ4	1	1
[K].dYWSTVk.[D]	N-Term(iTRAQ4plex); K7(iTRAQ4	1	1
[K].dALSSVQESQVAQQAR.[G]	N-Term(iTRAQ4plex)	1	1
[K].DALSSVQESQVAQQAR.[G]		1	1
[R].lDTLAQEVALLk.[E]	N-Term(iTRAQ4plex); K12(iTRAQ	1	1
[R].lDTLAQEVALLk.[E]	N-Term(iTRAQ4plex); K12(iTRAQ	1	1
[R].lDTLAQEVALLk.[E]	N-Term(iTRAQ4plex); K12(iTRAQ	1	1
[R].lDTLAQEVALLk.[E]	N-Term(iTRAQ4plex); K12(iTRAQ	1	1
[K].TFHEASEDcISR.[G]	C9(Carbamidomethyl)	1	1
[K].nWETEITAQPDGGk.[T]	N-Term(iTRAQ4plex); K14(iTRAQ	1	1
[K].nWETEITAQPDGGk.[T]	N-Term(iTRAQ4plex); K14(iTRAQ	1	1
[K].tFHEASEDcISR.[G]	N-Term(iTRAQ4plex); C9(Carbam	1	1
[K].tFHEASEDcISR.[G]	N-Term(iTRAQ4plex); C9(Carbam	1	1
[K].TFHEASEDcISR.[G]	C9(Carbamidomethyl)	1	1
[K].eQQALQTVcLk.[G]	N-Term(iTRAQ4plex); C9(Carbam	1	1
[K].TFHEASEDcISR.[G]	C9(Carbamidomethyl)	1	1
[K].TFHEASEDcISR.[G]	C9(Carbamidomethyl)	1	1
[K].TFHEASEDcISR.[G]	C9(Carbamidomethyl)	1	1
[K].kDVVNTk.[M]	K1(iTRAQ4plex); K7(iTRAQ4plex)	1	1
[K].kDVVNTk.[M]	N-Term(iTRAQ4plex); K7(iTRAQ4	1	1
[K].TFHEASEDcISR.[G]	C9(Carbamidomethyl)	1	1
[K].kDVVNTk.[M]	N-Term(iTRAQ4plex); K1(iTRAQ4	1	1
[K].TFHEASEDcISR.[G]	C9(Carbamidomethyl)	1	1
[K].cFLAFTQTk.[T]	N-Term(iTRAQ4plex); C1(Carbam	1	1
[K].kDVVNTk.[M]	N-Term(iTRAQ4plex); K1(iTRAQ4	1	1
[R].wDEELAAFAk.[A]	N-Term(iTRAQ4plex); K10(iTRAQ	1	1
[R].wDEELAAFAk.[A]	N-Term(iTRAQ4plex); K10(iTRAQ	1	1
[K].lQGVEETNIELLVcNYEPPGNVk.[G]	N-Term(iTRAQ4plex); C14(Carbar	1	1
[R].qcVWGHNk.[E]	N-Term(iTRAQ4plex); C2(Carbam	1	1
[R].qcVWGHNk.[E]	N-Term(iTRAQ4plex); C2(Carbam	1	1
[R].iGcGSHFcEk.[L]	N-Term(iTRAQ4plex); C3(Carbam	1	1
[R].lMVELHNLYR.[A]	N-Term(iTRAQ4plex)	1	1

[R].aQVSPTASDMLHMR.[W]	N-Term(iTRAQ4plex)	1	1
[R].wDEELAAFAk.[A]	N-Term(iTRAQ4plex); K10(iTRAQ4	1	1
[R].iGcGSHFcEk.[L]	N-Term(iTRAQ4plex); C3(Carbam	1	1
[R].rDVQNVAAAPELAMGALELESR.[N]	N-Term(iTRAQ4plex)	1	1
[R].sALYSPSDPLTLLQADTVR.[G]	N-Term(iTRAQ4plex)	1	1
[R].lAGAPSEDPQFPk.[V]	N-Term(iTRAQ4plex); K13(iTRAQ4	1	1
[R].eLcSAcHNER.[L]	N-Term(iTRAQ4plex); C3(Carbam	1	1
[K].fVAVLAk.[Y]	N-Term(iTRAQ4plex); K7(iTRAQ4	1	1
[R].eVALDLSQHk.[G]	N-Term(iTRAQ4plex); K10(iTRAQ4	1	1
[R].sALYSPSDPLTLLQADTVR.[G]	N-Term(iTRAQ4plex)	1	1
[K].mQILEGLGLNLQk.[S]	N-Term(iTRAQ4plex); K13(iTRAQ4	1	1
[K].tLYLADTFPTNFR.[D]	N-Term(iTRAQ4plex)	1	1
[K].aVVEVDESGTR.[A]	N-Term(iTRAQ4plex)	1	1
[K].aVVEVDESGTR.[A]	N-Term(iTRAQ4plex)	1	1
[K].aVVEVDESGTR.[A]	N-Term(iTRAQ4plex)	1	1
[R].eDQYHYLLDR.[N]	N-Term(iTRAQ4plex)	1	1
[K].fSIEGSYQLEk.[V]	N-Term(iTRAQ4plex); K11(iTRAQ4	1	1
[R].qLELYLPk.[F]	N-Term(iTRAQ4plex); K8(iTRAQ4	1	1
[K].fSIEGSYQLEk.[V]	N-Term(iTRAQ4plex); K11(iTRAQ4	1	1
[R].eDQYHYLLDR.[N]	N-Term(iTRAQ4plex)	1	1
[R].vIGGLLAGQTYHVAVVcYLR.[S]	N-Term(iTRAQ4plex); C17(Carbar	1	1
[K].lLTPITTLTSEQIQk.[L]	N-Term(iTRAQ4plex); K15(iTRAQ4	1	1
[R].lQPVLQPLPSPGVGGk.[R]	N-Term(iTRAQ4plex); K16(iTRAQ4	1	1
[K].sDDEVDDPAVELk.[Q]	N-Term(iTRAQ4plex); K13(iTRAQ4	1	1
[R].GMTQLQGTR.[A]		1	1
[R].ATYHGSFSTkk.[S]	K10(iTRAQ4plex); K11(iTRAQ4ple	1	1
[K].sLEVTFTPVIEDIGk.[V]	N-Term(iTRAQ4plex); K15(iTRAQ4	1	1
[K].sQEFLEDADR.[K]	N-Term(iTRAQ4plex)	1	1
[RK].lEIDLLk.[GS]	N-Term(iTRAQ4plex); K7(iTRAQ4	1	2
[R].lEIELLk.[G]	N-Term(iTRAQ4plex); K7(iTRAQ4	1	1
[K].lHIDDMEFEPk.[Q]	N-Term(iTRAQ4plex); K11(iTRAQ4	1	1
[K].lHIDEMDSVPTVR.[Q]	N-Term(iTRAQ4plex)	1	1
[R].aFPALTSLDLSDNPGLGER.[G]	N-Term(iTRAQ4plex)	1	1
[K].eLTLEDLk.[I]	N-Term(iTRAQ4plex); K8(iTRAQ4	1	1
[R].gLMAALcPHk.[F]	N-Term(iTRAQ4plex); C7(Carbam	1	1
[R].gLMAALcPHk.[F]	N-Term(iTRAQ4plex); C7(Carbam	1	1
[K].iTGTMPPLPLEATGLALSSLR.[L]	N-Term(iTRAQ4plex)	1	1
[R].aFPALTSLDLSDNPGLGER.[G]	N-Term(iTRAQ4plex)	1	1
[R].qYADTVk.[A]	N-Term(iTRAQ4plex); K7(iTRAQ4	1	1
[K].vLSIAQAHSPAFScEQVR.[A]	N-Term(iTRAQ4plex); C14(Carbar	1	1
[R].gLHLQALkPTkPGLLPSLFk.[G]	N-Term(iTRAQ4plex); K8(iTRAQ4	1	1
[R].lMNHYINk.[E]	N-Term(iTRAQ4plex); K8(iTRAQ4	1	1
[K].yEMHELLR.[Q]	N-Term(iTRAQ4plex)	1	1
[K].lFDEINPETk.[L]	N-Term(iTRAQ4plex); K10(iTRAQ4	1	1
[K].lFDEINPETk.[L]	N-Term(iTRAQ4plex); K10(iTRAQ4	1	1
[R].yFDTEcVPMNFR.[N]	N-Term(iTRAQ4plex); C6(Carbam	1	1
[R].nLELGLTQGSFAFIHk.[D]	N-Term(iTRAQ4plex); K16(iTRAQ4	1	1
[K].yGFcEAADQFHVLDEVR.[R]	N-Term(iTRAQ4plex); C4(Carbam	1	1
[K].yGFcEAADQFHVLDEVR.[R]	N-Term(iTRAQ4plex); C4(Carbam	1	1
[K].yGFcEAADQFHVLDEVR.[R]	N-Term(iTRAQ4plex); C4(Carbam	1	1
[R].rPASPISTIQPk.[A]	N-Term(iTRAQ4plex); K12(iTRAQ4	1	1
[R].kLDGIcWQVR.[Q]	N-Term(iTRAQ4plex); K1(iTRAQ4	1	1

[R].aEATTLHVAPQGTAMAVSTFR.[K]	N-Term(iTRAQ4plex)	1	1
[R].aEATTLHVAPQGTAMAVSTFR.[K]	N-Term(iTRAQ4plex)	1	1
[R].vQEAHLTEDQIFYFPk.[Y]	N-Term(iTRAQ4plex); K16(iTRAQ	1	1
[R].sLPVSDSVLSGFEQR.[V]	N-Term(iTRAQ4plex)	1	1
[R].kLDGIcWQVR.[Q]	N-Term(iTRAQ4plex); K1(iTRAQ4	1	1
[R].vQEAHLTEDQIFYFPk.[Y]	N-Term(iTRAQ4plex); K16(iTRAQ	1	1
[R].rPASPISTIQPk.[A]	N-Term(iTRAQ4plex); K12(iTRAQ	1	1
[R].kLDGIcWQVR.[Q]	N-Term(iTRAQ4plex); K1(iTRAQ4	1	1
[R].kLDGIcWQVR.[Q]	N-Term(iTRAQ4plex); K1(iTRAQ4	1	1
[R].sLPVSDSVLSGFEQR.[V]	N-Term(iTRAQ4plex)	1	1
[R].fLQEQGHR.[A]	N-Term(iTRAQ4plex)	1	1
[R].aEATTLHVAPQGTAMAVSTFR.[K]	N-Term(iTRAQ4plex)	1	1
[R].fLQEQGHR.[A]	N-Term(iTRAQ4plex)	1	1
[R].fLQEQGHR.[A]	N-Term(iTRAQ4plex)	1	1
[R].vQEAHLTEDQIFYFPk.[Y]	N-Term(iTRAQ4plex); K16(iTRAQ	1	1
[R].fLQEQGHR.[A]	N-Term(iTRAQ4plex)	1	1
[R].aEATTLHVAPQGTAMAVSTFR.[K]	N-Term(iTRAQ4plex)	1	1
[K].vGAHAGEYGAEALER.[M]	N-Term(iTRAQ4plex)	1	1
[M].vLSPADkTNVk.[A]	N-Term(iTRAQ4plex); K7(iTRAQ4	1	1
[K].tYFPHFDLSHGSAQVk.[G]	N-Term(iTRAQ4plex); K16(iTRAQ	1	1
[K].TYFPHFDLSHGSAQVk.[G]	K16(iTRAQ4plex)	1	1
[K].vGAHAGEYGAEALER.[M]	N-Term(iTRAQ4plex)	1	1
[K].vGAHAGEYGAEALER.[M]	N-Term(iTRAQ4plex)	1	1
[K].vADALTNAVAHVDDMPNALSALSDLHAH	N-Term(iTRAQ4plex); K29(iTRAQ	1	1
[R].mFLSFPTTk.[T]	N-Term(iTRAQ4plex); K9(iTRAQ4	1	1
[K].vGAHAGEYGAEALER.[M]	N-Term(iTRAQ4plex)	1	1
[R].vDPVNFk.[L]	N-Term(iTRAQ4plex); K7(iTRAQ4	1	2
[R].vDPVNFk.[L]	N-Term(iTRAQ4plex); K7(iTRAQ4	1	2
[R].MFLSFPTTk.[T]	K9(iTRAQ4plex)	1	1
[R].mFLSFPTTk.[T]	N-Term(iTRAQ4plex); M1(Oxidat	1	1
[K].fLASVSTVLTSk.[Y]	N-Term(iTRAQ4plex); K12(iTRAQ	1	1
[K].fLASVSTVLTSk.[Y]	N-Term(iTRAQ4plex); K12(iTRAQ	1	1
[R].VDPVNFk.[L]	K7(iTRAQ4plex)	1	2
[K].tYFPHFDLSHGSAQVk.[G]	N-Term(iTRAQ4plex); K16(iTRAQ	1	1
[R].eWFSETFQk.[V]	N-Term(iTRAQ4plex); K9(iTRAQ4	1	1
[R].eWFSETFQk.[V]	N-Term(iTRAQ4plex); K9(iTRAQ4	1	1
[R].eWFSETFQk.[V]	N-Term(iTRAQ4plex); K9(iTRAQ4	1	1
[R].EWFSETFQk.[V]	K9(iTRAQ4plex)	1	1
[R].ikQSELSAk.[M]	N-Term(iTRAQ4plex); K2(iTRAQ4	1	1
[K].eFGNTLEDk.[A]	N-Term(iTRAQ4plex); K9(iTRAQ4	1	1
[K].eFGNTLEDk.[A]	N-Term(iTRAQ4plex); K9(iTRAQ4	1	1
[K].eFGNTLEDk.[A]	N-Term(iTRAQ4plex); K9(iTRAQ4	1	1
[K].eFGNTLEDk.[A]	N-Term(iTRAQ4plex); K9(iTRAQ4	1	1
[K].lkEFGNTLEDk.[A]	N-Term(iTRAQ4plex); K2(iTRAQ4	1	1
[K].lkEFGNTLEDk.[A]	N-Term(iTRAQ4plex); K2(iTRAQ4	1	1
[R].IkQSELSAK.[M]	K2(iTRAQ4plex)	1	1
[R].ikQSELSAk.[M]	N-Term(iTRAQ4plex); K2(iTRAQ4	1	1
[R].eWFSETFQk.[V]	N-Term(iTRAQ4plex); K9(iTRAQ4	1	1
[R].eWFSETFQk.[V]	N-Term(iTRAQ4plex); K9(iTRAQ4	1	1
[K].eFGNTLEDk.[A]	N-Term(iTRAQ4plex); K9(iTRAQ4	1	1
[K].qSELSAk.[M]	N-Term(iTRAQ4plex); K7(iTRAQ4	1	1
[R].IkQSELSAkMR.[E]	K2(iTRAQ4plex); K9(iTRAQ4plex)	1	1

[R].eWFSETFQk.[V]	N-Term(iTRAQ4plex); K9(iTRAQ4	1	1
[K].EFGNTLEDk.[A]	K9(iTRAQ4plex)	1	1
[K].mREWFSETFQk.[V]	N-Term(iTRAQ4plex); K11(iTRAQ	1	1
[K].qSELSAk.[M]	N-Term(iTRAQ4plex); K7(iTRAQ4	1	1
[R].eWFSETFQk.[V]	N-Term(iTRAQ4plex); K9(iTRAQ4	1	1
[R].eWFSETFQk.[V]	N-Term(iTRAQ4plex); K9(iTRAQ4	1	1
[K].eFGNTLEDk.[A]	N-Term(iTRAQ4plex); K9(iTRAQ4	1	1
[R].iKQSELSAkMR.[E]	N-Term(iTRAQ4plex); K9(iTRAQ4	1	1
[K].qSELSAk.[M]	N-Term(iTRAQ4plex); K7(iTRAQ4	1	1
[R].EWFSETFQk.[V]	K9(iTRAQ4plex)	1	1
[K].eTYDFDIAVLR.[L]	N-Term(iTRAQ4plex)	1	1
[K].acIPTGPYPcGk.[Q]	N-Term(iTRAQ4plex); C2(Carbam	1	1
[R].mNVAPAcLPER.[D]	N-Term(iTRAQ4plex); C7(Carbam	1	1
[R].mNVAPAcLPER.[D]	N-Term(iTRAQ4plex); C7(Carbam	1	1
[K].dTYFVTGIVSWGEGcAR.[K]	N-Term(iTRAQ4plex); C15(Carbar	1	1
[K].dTYFVTGIVSWGEGcAR.[K]	N-Term(iTRAQ4plex); C15(Carbar	1	1
[R].iVGGQEck.[D]	N-Term(iTRAQ4plex); C7(Carbam	1	1
[K].eTYDFDIAVLR.[L]	N-Term(iTRAQ4plex)	1	1
[K].mLEVPYVDR.[N]	N-Term(iTRAQ4plex)	1	1
[K].mLEVPYVDR.[N]	N-Term(iTRAQ4plex)	1	1
[K].tGIVSGFGR.[T]	N-Term(iTRAQ4plex)	1	1
[R].IVGGQEck.[D]	C7(Carbamidomethyl); K8(iTRAC	1	1
[K].TGIVSGFGR.[T]		1	1
[K].mLEVPYVDR.[N]	N-Term(iTRAQ4plex)	1	1
[K].acIPTGPYPcGk.[Q]	N-Term(iTRAQ4plex); C2(Carbam	1	1
[K].sVVLIPLGAVDDGEHSQNEk.[I]	N-Term(iTRAQ4plex); K20(iTRAQ	1	1
[R].gDGWLTDPYVLTEVDGk.[L]	N-Term(iTRAQ4plex); K17(iTRAQ	1	1
[R].aLEQDLPVNIk.[F]	N-Term(iTRAQ4plex); K11(iTRAQ	1	1
[R].aLEQDLPVNIk.[F]	N-Term(iTRAQ4plex); K11(iTRAQ	1	1
[K].eEILMHLWR.[Y]	N-Term(iTRAQ4plex)	1	1
[R].mMAVAADTLQR.[L]	N-Term(iTRAQ4plex)	1	1
[R].kPAITYGTR.[G]	N-Term(iTRAQ4plex); K1(iTRAQ4	1	1
[R].aLEQDLPVNIk.[F]	N-Term(iTRAQ4plex); K11(iTRAQ	1	1
[K].sVVLIPLGAVDDGEHSQNEk.[I]	N-Term(iTRAQ4plex); K20(iTRAQ	1	1
[K].mFQEIVHk.[S]	N-Term(iTRAQ4plex); K8(iTRAQ4	1	1
[K].eEILMHLWR.[Y]	N-Term(iTRAQ4plex)	1	1
[R].aLAQcAPPPAVcAELVR.[E]	N-Term(iTRAQ4plex); C5(Carbam	1	1
[K].yGQPLPGYTTk.[G]	N-Term(iTRAQ4plex); K11(iTRAQ	1	1
[K].rETEYGPcR.[R]	N-Term(iTRAQ4plex); C8(Carbam	1	1
[K].fLNVLSPR.[G]	N-Term(iTRAQ4plex)	1	1
[R].aLAQcAPPPAVcAELVR.[E]	N-Term(iTRAQ4plex); C5(Carbam	1	1
[R].gVHIPNcDk.[K]	N-Term(iTRAQ4plex); C7(Carbam	1	1
[R].aLAQcAPPPAVcAELVR.[E]	N-Term(iTRAQ4plex); C5(Carbam	1	1
[K].fHPLHSk.[I]	N-Term(iTRAQ4plex); K7(iTRAQ4	1	1
[R].aLAQcAPPPAVcAELVR.[E]	N-Term(iTRAQ4plex); C5(Carbam	1	1
[K].vDYESQSTDTQNFSSESk.[R]	N-Term(iTRAQ4plex); K18(iTRAQ	1	1
[K].fHPLHSk.[I]	N-Term(iTRAQ4plex); K7(iTRAQ4	1	1
[K].iDPLEVEEGDPIVLPcNPPk.[G]	N-Term(iTRAQ4plex); C16(Carbar	1	1
[K].lHMLELHcESk.[C]	N-Term(iTRAQ4plex); C8(Carbam	1	1
[K].vEEVkPLEGR.[R]	N-Term(iTRAQ4plex); K5(iTRAQ4	1	1
[K].dGNPFYFTDHR.[I]	N-Term(iTRAQ4plex)	1	1
[K].gNPEPTFSWTk.[D]	N-Term(iTRAQ4plex); K11(iTRAQ	1	1

[R].vMTPAVYAPYDVk.[V]	N-Term(iTRAQ4plex); K13(iTRAQ	1	1
[K].gDLYFANVEEk.[D]	N-Term(iTRAQ4plex); K11(iTRAQ	1	1
[K].skEQLTPLIk.[K]	N-Term(iTRAQ4plex); K2(iTRAQ4	1	1
[K].skEQLTPLIk.[K]	N-Term(iTRAQ4plex); K2(iTRAQ4	1	1
[K].skEQLTPLIk.[K]	N-Term(iTRAQ4plex); K2(iTRAQ4	1	1
[K].skEQLTPLIk.[K]	N-Term(iTRAQ4plex); K2(iTRAQ4	1	1
[K].EPcVESLVSQYFQTVTDYGk.[D]	C3(Carbamidomethyl); K20(iTRA	1	1
[K].EPcVESLVSQYFQTVTDYGk.[D]	C3(Carbamidomethyl); K20(iTRA	1	1
[K].ePcVESLVSQYFQTVTDYGk.[D]	N-Term(iTRAQ4plex); C3(Carbam	1	1
[K].vkSPELQAEAk.[S]	N-Term(iTRAQ4plex); K2(iTRAQ4	1	1
[K].vkSPELQAEAk.[S]	N-Term(iTRAQ4plex); K2(iTRAQ4	1	1
[K].kAGTELVNFLSYFVELGTQPATQ.[-]	N-Term(iTRAQ4plex); K1(iTRAQ4	1	1
[K].sPELQAEAk.[S]	N-Term(iTRAQ4plex); K9(iTRAQ4	1	1
[K].sPELQAEAk.[S]	N-Term(iTRAQ4plex); K9(iTRAQ4	1	1
[K].sPELQAEAk.[S]	N-Term(iTRAQ4plex); K9(iTRAQ4	1	1
[K].sPELQAEAk.[S]	N-Term(iTRAQ4plex); K9(iTRAQ4	1	1
[K].sPELQAEAk.[S]	N-Term(iTRAQ4plex); K9(iTRAQ4	1	1
[K].sPELQAEAk.[S]	N-Term(iTRAQ4plex); K9(iTRAQ4	1	1
[K].sPELQAEAk.[S]	N-Term(iTRAQ4plex); K9(iTRAQ4	1	1
[K].sPELQAEAk.[S]	N-Term(iTRAQ4plex); K9(iTRAQ4	1	1
[K].sPELQAEAk.[S]	N-Term(iTRAQ4plex); K9(iTRAQ4	1	1
[K].aGTELVNFLSYFVELGTQPATQ.[-]	N-Term(iTRAQ4plex)	1	1
[K].ePcVESLVSQYFQTVTDYGk.[D]	N-Term(iTRAQ4plex); C3(Carbam	1	1
[K].SPELQAEAk.[S]	K9(iTRAQ4plex)	1	1
[K].VkSPELQAEAk.[S]	K2(iTRAQ4plex); K11(iTRAQ4ple	1	1
[K].SPELQAEAk.[S]	K9(iTRAQ4plex)	1	1
[K].sPELQAEAk.[S]	N-Term(iTRAQ4plex); K9(iTRAQ4	1	1
[K].sPELQAEAk.[S]	N-Term(iTRAQ4plex); K9(iTRAQ4	1	1
[K].eQLTPLIk.[K]	N-Term(iTRAQ4plex); K8(iTRAQ4	1	1
[K].sPELQAEAk.[S]	N-Term(iTRAQ4plex); K9(iTRAQ4	1	1
[K].sPELQAEAk.[S]	N-Term(iTRAQ4plex); K9(iTRAQ4	1	1
[K].skEQLTPLIk.[K]	N-Term(iTRAQ4plex); K2(iTRAQ4	1	1
[K].skEQLTPLIk.[K]	N-Term(iTRAQ4plex); K2(iTRAQ4	1	1
[K].sPELQAEAk.[S]	N-Term(iTRAQ4plex); K9(iTRAQ4	1	1
[K].skEQLTPLIk.[K]	N-Term(iTRAQ4plex); K2(iTRAQ4	1	1
[K].eQLTPLIk.[K]	N-Term(iTRAQ4plex); K8(iTRAQ4	1	1
[K].eQLTPLIk.[K]	N-Term(iTRAQ4plex); K8(iTRAQ4	1	1
[K].ePcVESLVSQYFQTVTDYGk.[D]	N-Term(iTRAQ4plex); C3(Carbam	1	1
[K].SkEQLTPLIk.[K]	K2(iTRAQ4plex); K10(iTRAQ4ple	1	1
[K].skEQLTPLIk.[K]	N-Term(iTRAQ4plex); K2(iTRAQ4	1	1
[K].sKEQLTPLIk.[K]	N-Term(iTRAQ4plex); K10(iTRAQ	1	1
[K].sPELQAEAk.[S]	N-Term(iTRAQ4plex); K9(iTRAQ4	1	1
[K].eQLTPLIk.[K]	N-Term(iTRAQ4plex); K8(iTRAQ4	1	1
[K].sPELQAEAk.[S]	N-Term(iTRAQ4plex); K9(iTRAQ4	1	1
[K].skEQLTPLIK.[K]	N-Term(iTRAQ4plex); K2(iTRAQ4	1	1
[K].eQLTPLIK.[K]	N-Term(iTRAQ4plex)	1	1
[K].aGTELVNFLSYFVELGTQPATQ.[-]	N-Term(iTRAQ4plex)	1	1
[K].skEQLTPLIkk.[A]	N-Term(iTRAQ4plex); K2(iTRAQ4	1	1
[K].eQLTPLIk.[K]	N-Term(iTRAQ4plex); K8(iTRAQ4	1	1
[K].sPELQAEAk.[S]	N-Term(iTRAQ4plex); K9(iTRAQ4	1	1
[K].skEQLTPLIk.[K]	N-Term(iTRAQ4plex); K2(iTRAQ4	1	1
[K].eQLTPLIk.[K]	N-Term(iTRAQ4plex); K8(iTRAQ4	1	1

[K].vkSPELQAEAk.[S]	N-Term(iTRAQ4plex); K2(iTRAQ4	1	1
[K].sPELQAEAk.[S]	N-Term(iTRAQ4plex); K9(iTRAQ4	1	1
[K].skEQLTPLIk.[K]	N-Term(iTRAQ4plex); K2(iTRAQ4	1	1
[K].skEQLTPLIk.[K]	N-Term(iTRAQ4plex); K2(iTRAQ4	1	1
[K].sPELQAEAk.[S]	N-Term(iTRAQ4plex); K9(iTRAQ4	1	1
[K].skEQLTPLIk.[K]	N-Term(iTRAQ4plex); K2(iTRAQ4	1	1
[K].sPELQAEAk.[S]	N-Term(iTRAQ4plex); K9(iTRAQ4	1	1
[K].eQLTPLIk.[K]	N-Term(iTRAQ4plex); K8(iTRAQ4	1	1
[K].VkSPELQAEAk.[S]	K2(iTRAQ4plex); K11(iTRAQ4ple	1	1
[K].vKSPELQAEAk.[S]	N-Term(iTRAQ4plex); K11(iTRAQ	1	1
[K].sPELQAEAk.[S]	N-Term(iTRAQ4plex); K9(iTRAQ4	1	1
[K].EQLTPLIk.[K]	K8(iTRAQ4plex)	1	1
[K].sPELQAEAk.[S]	N-Term(iTRAQ4plex); K9(iTRAQ4	1	1
[K].yGIDWASGR.[G]	N-Term(iTRAQ4plex)	1	1
[R].tFAHYATFR.[L]	N-Term(iTRAQ4plex)	1	1
[R].tFAHYATFR.[L]	N-Term(iTRAQ4plex)	1	1
[R].lLGEVDHYQLALGk.[F]	N-Term(iTRAQ4plex); K14(iTRAQ	1	1
[R].yAVSEAAAHk.[Y]	N-Term(iTRAQ4plex); K10(iTRAQ	1	1
[R].aGFGNQESEFWLGNENLHQLTLQGNWEL	N-Term(iTRAQ4plex)	1	1
[R].aGFGNQESEFWLGNENLHQLTLQGNWEL	N-Term(iTRAQ4plex)	1	1
[R].yAVSEAAAHk.[Y]	N-Term(iTRAQ4plex); K10(iTRAQ	1	1
[R].aLPVFcDMDTEGGGWLVFQR.[R]	N-Term(iTRAQ4plex); C6(Carbam	1	1
[R].qDGSVDFFR.[S]	N-Term(iTRAQ4plex)	1	1
[K].vVLLPScPGAPGSPGEk.[G]	N-Term(iTRAQ4plex); C7(Carbam	1	1
[R].tFAHYATFR.[L]	N-Term(iTRAQ4plex)	1	1
[R].tFAHYATFR.[L]	N-Term(iTRAQ4plex)	1	1
[K].YGIDWASGR.[G]		1	1
[R].lLGEVDHYQLALGk.[F]	N-Term(iTRAQ4plex); K14(iTRAQ	1	1
[K].vVLLPScPGAPGSPGEk.[G]	N-Term(iTRAQ4plex); C7(Carbam	1	1
[R].tFAHYATFR.[L]	N-Term(iTRAQ4plex)	1	1
[K].SHLIIAQVAk.[N]	K10(iTRAQ4plex)	1	1
[K].sHLIIAQVAk.[N]	N-Term(iTRAQ4plex); K10(iTRAQ	1	1
[K].sHLIIAQVAk.[N]	N-Term(iTRAQ4plex); K10(iTRAQ	1	1
[R].wNPcLEPHR.[F]	N-Term(iTRAQ4plex); C4(Carbam	1	1
[R].qEALELMNQNLDIYEQQVMTAAQk.[D]	N-Term(iTRAQ4plex); K24(iTRAQ	1	1
[K].sHLIIAQVAk.[N]	N-Term(iTRAQ4plex); K10(iTRAQ	1	1
[K].sHLIIAQVAk.[N]	N-Term(iTRAQ4plex); K10(iTRAQ	1	1
[K].iLSGDPYcEk.[D]	N-Term(iTRAQ4plex); C8(Carbam	1	1
[K].dAQEVHcDEATk.[W]	N-Term(iTRAQ4plex); C7(Carbam	1	1
[K].dAQEVHcDEATk.[W]	N-Term(iTRAQ4plex); C7(Carbam	1	1
[R].gDMFLVANLGTk.[E]	N-Term(iTRAQ4plex); K12(iTRAQ	1	1
[K].vDLITFDTPFAGR.[F]	N-Term(iTRAQ4plex)	1	1
[K].SHLIIAQVAk.[N]	K10(iTRAQ4plex)	1	1
[R].lSSGLVTAALYGR.[L]	N-Term(iTRAQ4plex)	1	1
[K].vDLITFDTPFAGR.[F]	N-Term(iTRAQ4plex)	1	1
[R].gDMFLVANLGTk.[E]	N-Term(iTRAQ4plex); K12(iTRAQ	1	1
[R].tWDPEGVIFYGDTNPk.[D]	N-Term(iTRAQ4plex); K16(iTRAQ	1	1
[R].tWDPEGVIFYGDTNPk.[D]	N-Term(iTRAQ4plex); K16(iTRAQ	1	1
[R].vVLSQGSk.[M]	N-Term(iTRAQ4plex); K8(iTRAQ4	1	1
[R].iALGGLLFPASNLR.[L]	N-Term(iTRAQ4plex)	1	1
[K].qAEISASAPTSLR.[S]	N-Term(iTRAQ4plex)	1	1
[K].vVLSSGSGPGLDLPLVLGLPLQLk.[L]	N-Term(iTRAQ4plex); K24(iTRAQ	1	1

[R].dGRPEIQLHNHWAQLTVGAGPR.[L]	N-Term(iTRAQ4plex)	1	1
[R].dGRPEIQLHNHWAQLTVGAGPR.[L]	N-Term(iTRAQ4plex)	1	1
[R].wHQVEVk.[M]	N-Term(iTRAQ4plex); K7(iTRAQ4	1	1
[K].dDWFMLGLR.[D]	N-Term(iTRAQ4plex)	1	1
[R].vVLSQGSk.[M]	N-Term(iTRAQ4plex); K8(iTRAQ4	1	1
[R].kWIYHLTEGSTDLR.[T]	N-Term(iTRAQ4plex); K1(iTRAQ4	1	1
[K].wIYHLTEGSTDLR.[T]	N-Term(iTRAQ4plex)	1	1
[K].wIYHLTEGSTDLR.[T]	N-Term(iTRAQ4plex)	1	1
[K].wIYHLTEGSTDLR.[T]	N-Term(iTRAQ4plex)	1	1
[K].wIYHLTEGSTDLR.[T]	N-Term(iTRAQ4plex)	1	1
[K].eFPEVHLGQWYFIAGAAPTk.[E]	N-Term(iTRAQ4plex); K20(iTRAQ	1	1
[K].eELATFDPVDNIVFNMAAGSAPMQLHLR.	N-Term(iTRAQ4plex)	1	1
[K].sLTScLDSk.[A]	N-Term(iTRAQ4plex); C5(Carbam	1	1
[K].wIYHLTEGSTDLR.[T]	N-Term(iTRAQ4plex)	1	1
[K].sLTScLDSk.[A]	N-Term(iTRAQ4plex); C5(Carbam	1	1
[R].nQEAcELSNN.[-]	N-Term(iTRAQ4plex); C5(Carbam	1	1
[K].eFPEVHLGQWYFIAGAAPTk.[E]	N-Term(iTRAQ4plex); K20(iTRAQ	1	1
[R].tEGRPDMk.[T]	N-Term(iTRAQ4plex); K8(iTRAQ4	1	1
[R].tEGRPDMk.[T]	N-Term(iTRAQ4plex); K8(iTRAQ4	1	1
[R].nQEAcELSNN.[-]	N-Term(iTRAQ4plex); C5(Carbam	1	1
[K].AFLLTPR.[N]		1	1
[K].SLTScLDSk.[A]	C5(Carbamidomethyl); K9(iTRAC	1	1
[R].eFTRPEEIIFLR.[A]	N-Term(iTRAQ4plex)	1	1
[R].gYHLNEEGTR.[C]	N-Term(iTRAQ4plex)	1	1
[K].sQETGDLDVGGLQETDk.[I]	N-Term(iTRAQ4plex); K17(iTRAQ	1	1
[R].dSScGTGYELTEDNSck.[D]	N-Term(iTRAQ4plex); C4(Carbam	1	1
[R].dSScGTGYELTEDNSck.[D]	N-Term(iTRAQ4plex); C4(Carbam	1	1
[R].gYHLNEEGTR.[C]	N-Term(iTRAQ4plex)	1	1
[R].mcVDVNEcQR.[Y]	N-Term(iTRAQ4plex); C2(Carbam	1	1
[R].mVQEQccHSQLEELHcATGISLANEQDR.[	N-Term(iTRAQ4plex); C6(Carbam	1	1
[R].ccHccLLGR.[A]	N-Term(iTRAQ4plex); C1(Carbam	1	1
[R].sAATLQQEk.[T]	N-Term(iTRAQ4plex); K9(iTRAQ4	1	1
[R].dSScGTGYELTEDNSck.[D]	N-Term(iTRAQ4plex); C4(Carbam	1	1
[R].sAATLQQEk.[T]	N-Term(iTRAQ4plex); K9(iTRAQ4	1	1
[R].sIPQVSPVR.[R]	N-Term(iTRAQ4plex)	1	1
[R].iVQLIQDTR.[I]	N-Term(iTRAQ4plex)	1	1
[K].yGGPNHHLPLPDNWk.[S]	N-Term(iTRAQ4plex); K15(iTRAQ	1	1
[K].yVGNmHGNEALGR.[E]	N-Term(iTRAQ4plex); M5(Oxidat	1	1
[R].iVQLIQDTR.[I]	N-Term(iTRAQ4plex)	1	1
[K].yGGPNHHLPLPDNWk.[S]	N-Term(iTRAQ4plex); K15(iTRAQ	1	1
[K].vQNEcPGITR.[V]	N-Term(iTRAQ4plex); C5(Carbam	1	1
[R].tASTPTPDDk.[L]	N-Term(iTRAQ4plex); K10(iTRAQ	1	1
[R].IVQLIQDTR.[I]		1	1
[K].sQVEPETR.[A]	N-Term(iTRAQ4plex)	1	1
[K].yGGPNHHLPLPDNWk.[S]	N-Term(iTRAQ4plex); K15(iTRAQ	1	1
[R].iHILPSMNPDGYEVAAAQGPNkPGYLVGR	N-Term(iTRAQ4plex); K22(iTRAQ	1	1
[R].sIPQVSPVR.[R]	N-Term(iTRAQ4plex)	1	1
[K].yGGPNHHLPLPDNWk.[S]	N-Term(iTRAQ4plex); K15(iTRAQ	1	1
[K].dNVEMDTFQIEcLk.[D]	N-Term(iTRAQ4plex); C12(Carba	1	1
[R].aAGNEcPELQPPVHGk.[I]	N-Term(iTRAQ4plex); C6(Carbam	1	1
[R].aAGNEcPELQPPVHGk.[I]	N-Term(iTRAQ4plex); C6(Carbam	1	1
[R].aAGNEcPELQPPVHGk.[I]	N-Term(iTRAQ4plex); C6(Carbam	1	1

[R].sDENEQHLGVk.[H]	N-Term(iTRAQ4plex); K11(iTRAQ	1	1
[RK].sDFSNEER.[F]	N-Term(iTRAQ4plex)	2	2
[K].vLGPFcGEk.[A]	N-Term(iTRAQ4plex); C6(Carbam	1	1
[R].dSDLLSPSDFk.[I]	N-Term(iTRAQ4plex); K11(iTRAQ	1	1
[R].dMIcAGEk.[E]	N-Term(iTRAQ4plex); C4(Carbam	1	1
[K].yScQEPYYk.[M]	N-Term(iTRAQ4plex); C3(Carbam	1	1
[R].sDENEQHLGVk.[H]	N-Term(iTRAQ4plex); K11(iTRAQ	1	1
[R].sLPTcLPVcGLPk.[F]	N-Term(iTRAQ4plex); C5(Carbam	1	1
[R].hVLAAWALGAk.[A]	N-Term(iTRAQ4plex); K11(iTRAQ	1	1
[R].sPAGPTVVSIGGGk.[G]	N-Term(iTRAQ4plex); K14(iTRAQ	1	1
[K].sAVDAGFLQk.[Y]	N-Term(iTRAQ4plex); K10(iTRAQ	1	1
[K].gQIHLDPQQDYQLLQVQR.[T]	N-Term(iTRAQ4plex)	1	1
[K].vISTLEEPTPQcPTSQGR.[S]	N-Term(iTRAQ4plex); C12(Carbar	1	1
[R].kVVTVLVR.[D]	N-Term(iTRAQ4plex); K1(iTRAQ4	1	1
[R].sPAGPTVVSIGGGk.[G]	N-Term(iTRAQ4plex); K14(iTRAQ	1	1
[R].eTAFILTGYcTDk.[C]	N-Term(iTRAQ4plex); C10(Carbar	1	1
[R].lEVHYHNPLVIEGR.[N]	N-Term(iTRAQ4plex)	1	1
[K].gRPGPQPWcATTPNFDQDQR.[W]	N-Term(iTRAQ4plex); C9(Carbam	1	1
[R].tTLSGAPcQPWASEATYR.[N]	N-Term(iTRAQ4plex); C8(Carbam	1	1
[R].nWGLGGHAFcR.[N]	N-Term(iTRAQ4plex); C10(Carbar	1	1
[R].lHEAFSPVSYQHDLALLR.[L]	N-Term(iTRAQ4plex)	1	1
[R].tNPcLHGGR.[C]	N-Term(iTRAQ4plex); C4(Carbam	1	1
[R].nWGLGGHAFcR.[N]	N-Term(iTRAQ4plex); C10(Carbar	1	1
[R].cLEVEGHR.[L]	N-Term(iTRAQ4plex); C1(Carbam	1	1
[R].vVGGLVALR.[G]	N-Term(iTRAQ4plex)	1	1
[R].eQPPSLTR.[N]	N-Term(iTRAQ4plex)	1	1
[K].lIcQATGFSPR.[Q]	N-Term(iTRAQ4plex); C3(Carbam	1	2
[K].lIcQATGFSPR.[Q]	N-Term(iTRAQ4plex); C3(Carbam	1	2
[K].gVALHRPDVYLLPPAR.[E]	N-Term(iTRAQ4plex)	1	2
[K].gVALHRPDVYLLPPAR.[E]	N-Term(iTRAQ4plex)	1	2
[K].gVALHRPDVYLLPPAR.[E]	N-Term(iTRAQ4plex)	1	2
[K].LIcQATGFSPR.[Q]	C3(Carbamidomethyl)	1	2
[R].fTcTVTHTDLPSPLk.[Q]	N-Term(iTRAQ4plex); C3(Carbam	1	2
[R].fTcTVTHTDLPSPLk.[Q]	N-Term(iTRAQ4plex); C3(Carbam	1	2
[R].vFAIPPSFASIFLTk.[S]	N-Term(iTRAQ4plex); K15(iTRAQ	1	2
[K].yVTSAPMPEPQAPGR.[Y]	N-Term(iTRAQ4plex)	1	2
[K].eSGPTTYk.[V]	N-Term(iTRAQ4plex); K8(iTRAQ4	1	2
[K].vTSTLTIk.[E]	N-Term(iTRAQ4plex); K8(iTRAQ4	1	2
[R].GQPLSPEk.[Y]	K8(iTRAQ4plex)	1	2
[R].gQPLSPEk.[Y]	N-Term(iTRAQ4plex); K8(iTRAQ4	1	2
[K].eSGPTTYk.[V]	N-Term(iTRAQ4plex); K8(iTRAQ4	1	2
[R].vFAIPPSFASIFLTk.[S]	N-Term(iTRAQ4plex); K15(iTRAQ	1	2
[K].VSVFVPPR.[D]		1	2
[K].yVTSAPMPEPQAPGR.[Y]	N-Term(iTRAQ4plex)	1	2
[K].vTSTLTIk.[E]	N-Term(iTRAQ4plex); K8(iTRAQ4	1	2
[R].fTcTVTHTDLPSPLk.[Q]	N-Term(iTRAQ4plex); C3(Carbam	1	2
[R].fTcTVTHTDLPSPLk.[Q]	N-Term(iTRAQ4plex); C3(Carbam	1	2
[K].vSVFVPPR.[D]	N-Term(iTRAQ4plex)	1	2
[R].GQPLSPEk.[Y]	K8(iTRAQ4plex)	1	2
[K].gVALHRPDVYLLPPAR.[E]	N-Term(iTRAQ4plex)	1	2
[R].fTcTVTHTDLPSPLk.[Q]	N-Term(iTRAQ4plex); C3(Carbam	1	2
[R].gQPLSPEk.[Y]	N-Term(iTRAQ4plex); K8(iTRAQ4	1	2

[K].vSVFVPPR.[D]	N-Term(iTRAQ4plex)	1	2
[K].vTSTLTIk.[E]	N-Term(iTRAQ4plex); K8(iTRAQ4	1	2
[K].vTSTLTIk.[E]	N-Term(iTRAQ4plex); K8(iTRAQ4	1	2
[K].vSVFVPPR.[D]	N-Term(iTRAQ4plex)	1	2
[R].gQPLSPEk.[Y]	N-Term(iTRAQ4plex); K8(iTRAQ4	1	2
[K].vSVFVPPR.[D]	N-Term(iTRAQ4plex)	1	2
[K].vSVFVPPR.[D]	N-Term(iTRAQ4plex)	1	2
[K].iTFMcNDHYILk.[G]	N-Term(iTRAQ4plex); C5(Carbam	1	1
[R].yYLVGVQEQQcVDGEWSSALPVck.[L]	N-Term(iTRAQ4plex); C11(Carbar	1	1
[R].yYLVGVQEQQcVDGEWSSALPVck.[L]	N-Term(iTRAQ4plex); C11(Carbar	1	1
[R].sQcLEDHTWAPPFPIck.[S]	N-Term(iTRAQ4plex); C3(Carbam	1	1
[K].eVEGQILGTYVcIk.[G]	N-Term(iTRAQ4plex); C12(Carbar	1	1
[K].eVEGQILGTYVcIk.[G]	N-Term(iTRAQ4plex); C12(Carbar	1	1
[K].lIQEAPkPEcEk.[A]	N-Term(iTRAQ4plex); K7(iTRAQ4	1	1
[K].lIQEAPkPEcEk.[A]	N-Term(iTRAQ4plex); K7(iTRAQ4	1	1
[K].lIQEAPkPEcEk.[A]	N-Term(iTRAQ4plex); K7(iTRAQ4	1	1
[K].nLcEAMENFMQQLk.[E]	N-Term(iTRAQ4plex); C3(Carbam	1	1
[K].aLLAFQESk.[N]	N-Term(iTRAQ4plex); K9(iTRAQ4	1	1
[K].aLLAFQESk.[N]	N-Term(iTRAQ4plex); K9(iTRAQ4	1	1
[K].iTFmcNDHYILk.[G]	N-Term(iTRAQ4plex); M4(Oxidat	1	1
[K].eSGMTMEELk.[Y]	N-Term(iTRAQ4plex); K10(iTRAQ	1	1
[K].gYHLVGk.[K]	N-Term(iTRAQ4plex); K7(iTRAQ4	1	1
[R].SQcLEDHTWAPPFPIck.[S]	C3(Carbamidomethyl); C16(Carb	1	1
[R].sQcLEDHTWAPPFPIck.[S]	N-Term(iTRAQ4plex); C3(Carbam	1	1
[R].nPDADEkPWcFIk.[V]	N-Term(iTRAQ4plex); K7(iTRAQ4	1	1
[R].nPDADEkPWcFIk.[V]	N-Term(iTRAQ4plex); K7(iTRAQ4	1	1
[R].nPDADEkPWcFIk.[V]	N-Term(iTRAQ4plex); K7(iTRAQ4	1	1
[R].gQcLITQSPPYYR.[C]	N-Term(iTRAQ4plex); C3(Carbam	1	1
[R].gQcLITQSPPYYR.[C]	N-Term(iTRAQ4plex); C3(Carbam	1	1
[R].gQcLITQSPPYYR.[C]	N-Term(iTRAQ4plex); C3(Carbam	1	1
[K].fTcAcPDQFk.[G]	N-Term(iTRAQ4plex); C3(Carbam	1	1
[K].fTcAcPDQFk.[G]	N-Term(iTRAQ4plex); C3(Carbam	1	1
[K].lPGFDScGk.[T]	N-Term(iTRAQ4plex); C7(Carbam	1	1
[K].lPGFDScGk.[T]	N-Term(iTRAQ4plex); C7(Carbam	1	1
[K].kEEFHEQSFR.[V]	N-Term(iTRAQ4plex); K1(iTRAQ4	1	1
[K].lkPVDGHcALESk.[Y]	N-Term(iTRAQ4plex); K2(iTRAQ4	1	1
[K].lkPVDGHcALESk.[Y]	N-Term(iTRAQ4plex); K2(iTRAQ4	1	1
[R].nPDADEkPWcFIk.[V]	N-Term(iTRAQ4plex); K7(iTRAQ4	1	1
[R].dEIPHNDIALLk.[L]	N-Term(iTRAQ4plex); K12(iTRAQ	1	1
[R].nPDADEkPWcFIk.[V]	N-Term(iTRAQ4plex); K7(iTRAQ4	1	1
[K].lPGFDScGk.[T]	N-Term(iTRAQ4plex); C7(Carbam	1	1
[R].gQcLITQSPPYYR.[C]	N-Term(iTRAQ4plex); C3(Carbam	1	1
[K].vVLGDQDLk.[K]	N-Term(iTRAQ4plex); K9(iTRAQ4	1	1
[K].LIANTLcNSR.[Q]	C7(Carbamidomethyl)	1	1
[R].gQcLITQSPPYYR.[C]	N-Term(iTRAQ4plex); C3(Carbam	1	1
[K].kEEFHEQSFR.[V]	N-Term(iTRAQ4plex); K1(iTRAQ4	1	1
[K].kEEFHEQSFR.[V]	N-Term(iTRAQ4plex); K1(iTRAQ4	1	1
[K].lPGFDScGk.[T]	N-Term(iTRAQ4plex); C7(Carbam	1	1
[R].nPDADEkPWcFIk.[V]	N-Term(iTRAQ4plex); K7(iTRAQ4	1	1
[R].aYSLFSYNTQGRDNELLVYkER.[V]	N-Term(iTRAQ4plex); K20(iTRAQ	1	1
[R].aYSLFSYNTQGR.[D]	N-Term(iTRAQ4plex)	1	1
[R].aYSLFSYNTQGR.[D]	N-Term(iTRAQ4plex)	1	1

[R].aYSLFSYNTQGR.[D]	N-Term(iTRAQ4plex)	1	1
[R].dNELLVYkER.[V]	N-Term(iTRAQ4plex); K8(iTRAQ4	1	1
[K].iVLGQEQDSYGGk.[F]	N-Term(iTRAQ4plex); K13(iTRAQ	1	1
[K].iVLGQEQDSYGGk.[F]	N-Term(iTRAQ4plex); K13(iTRAQ	1	1
[K].iVLGQEQDSYGGk.[F]	N-Term(iTRAQ4plex); K13(iTRAQ	1	1
[K].iVLGQEQDSYGGk.[F]	N-Term(iTRAQ4plex); K13(iTRAQ	1	1
[R].VGEYSLYIGR.[H]		1	1
[R].GYVIIkPLVWV.[-]	K6(iTRAQ4plex)	1	1
[R].aYSLFSYNTQGR.[D]	N-Term(iTRAQ4plex)	1	1
[K].iVLGQEQDSYGGk.[F]	N-Term(iTRAQ4plex); K13(iTRAQ	1	1
[R].dNELLVYk.[E]	N-Term(iTRAQ4plex); K8(iTRAQ4	1	1
[R].gYVIIkPLVWV.[-]	N-Term(iTRAQ4plex); K6(iTRAQ4	1	1
[R].dNELLVYkER.[V]	N-Term(iTRAQ4plex); K8(iTRAQ4	1	1
[R].QGYFVEAQPk.[I]	K10(iTRAQ4plex)	1	1
[K].gLRQGYFVEAQPk.[I]	N-Term(iTRAQ4plex); K13(iTRAQ	1	1
[R].qGYFVEAQPk.[I]	N-Term(iTRAQ4plex); K10(iTRAQ	1	1
[R].dNELLVYkER.[V]	N-Term(iTRAQ4plex); K8(iTRAQ4	1	1
[R].dNELLVYk.[E]	N-Term(iTRAQ4plex); K8(iTRAQ4	1	1
[R].dNELLVYkER.[V]	N-Term(iTRAQ4plex); K8(iTRAQ4	1	1
[R].hcYSIQHcPLk.[G]	N-Term(iTRAQ4plex); C2(Carbam	1	1
[R].hcYSIQHcPLk.[G]	N-Term(iTRAQ4plex); C2(Carbam	1	1
[K].gLLGGGVSVEDccLNTAFAYQk.[R]	N-Term(iTRAQ4plex); C12(Carbar	1	1
[R].tcNHPVPQHGGPFcAGDATR.[T]	N-Term(iTRAQ4plex); C2(Carbam	1	1
[R].tcNHPVPQHGGPFcAGDATR.[T]	N-Term(iTRAQ4plex); C2(Carbam	1	1
[R].hcYSIQHcPLk.[G]	N-Term(iTRAQ4plex); C2(Carbam	1	1
[K].sIScQEIPGQQSR.[G]	N-Term(iTRAQ4plex); C4(Carbam	1	1
[K].yPPTVSMVEGQGEk.[N]	N-Term(iTRAQ4plex); K14(iTRAQ	1	1
[K].rPcLHVPAck.[D]	N-Term(iTRAQ4plex); C3(Carbam	1	1
[K].rPcLHVPAck.[D]	N-Term(iTRAQ4plex); C3(Carbam	1	1
[R].lcTPLLPk.[Y]	N-Term(iTRAQ4plex); C2(Carbam	1	1
[K].rPcLHVPAck.[D]	N-Term(iTRAQ4plex); C3(Carbam	1	1
[R].cEELQGQk.[L]	N-Term(iTRAQ4plex); C1(Carbam	1	1
[R].acNHPAPk.[C]	N-Term(iTRAQ4plex); C2(Carbam	1	1
[K].rPcLHVPAck.[D]	N-Term(iTRAQ4plex); C3(Carbam	1	1
[R].lcTPLLPk.[Y]	N-Term(iTRAQ4plex); C2(Carbam	1	1
[R].acNHPAPk.[C]	N-Term(iTRAQ4plex); C2(Carbam	1	1
[K].gNDDHWIVDTDYDTYAVQYScR.[L]	N-Term(iTRAQ4plex); C21(Carbar	1	1
[K].gNDDHWIVDTDYDTYAVQYScR.[L]	N-Term(iTRAQ4plex); C21(Carbar	1	1
[K].gNDDHWIVDTDYDTYAVQYScR.[L]	N-Term(iTRAQ4plex); C21(Carbar	1	1
[R].lIVHNGYcDGR.[S]	N-Term(iTRAQ4plex); C8(Carbam	1	1
[R].lLNNWDVcADMVGTFTDTEDPAk.[F]	N-Term(iTRAQ4plex); C8(Carbam	1	1
[K].kDPEGLFLQDNIVAEFSVDETGQMSATAk.	N-Term(iTRAQ4plex); K1(iTRAQ4	1	1
[K].kDPEGLFLQDNIVAEFSVDETGQMSATAk.	N-Term(iTRAQ4plex); K1(iTRAQ4	1	1
[K].kDPEGLFLQDNIVAEFSVDETGQMSATAk.	N-Term(iTRAQ4plex); K1(iTRAQ4	1	1
[R].lLNNWDVcADMVGTFTDTEDPAk.[F]	N-Term(iTRAQ4plex); C8(Carbam	1	1
[K].kDPEGLFLQDNIVAEFSVDETGQMSATAk.	N-Term(iTRAQ4plex); K1(iTRAQ4	1	1
[K].yWGVASFLQk.[G]	N-Term(iTRAQ4plex); K10(iTRAQ	1	1
[K].yWGVASFLQk.[G]	N-Term(iTRAQ4plex); K10(iTRAQ	1	1
[R].dPNGLPPEAQk.[I]	N-Term(iTRAQ4plex); K11(iTRAQ	1	1
[R].dPNGLPPEAQk.[I]	N-Term(iTRAQ4plex); K11(iTRAQ	1	1
[R].lLNLDGTcADSYSFVFSR.[D]	N-Term(iTRAQ4plex); C8(Carbam	1	1
[R].lLNLDGTcADSYSFVFSR.[D]	N-Term(iTRAQ4plex); C8(Carbam	1	1

[K].kDPEGLFLQDNIVAEFSVDETGQMSATAk.	N-Term(iTRAQ4plex); K1(iTRAQ4	1	1
[R].LIVHNGYcDGR.[S]	C8(Carbamidomethyl)	1	1
[R].FSGTWYAMAk.[K]	K10(iTRAQ4plex)	1	1
[R].fSGTWYAMAk.[K]	N-Term(iTRAQ4plex); K10(iTRAQ	1	1
[R].qEELcLAR.[Q]	N-Term(iTRAQ4plex); C5(Carbam	1	1
[R].QRQEELcLAR.[Q]	C7(Carbamidomethyl)	1	1
[R].fSGTWYAMAk.[K]	N-Term(iTRAQ4plex); K10(iTRAQ	1	1
[R].fSGTWYAMAk.[K]	N-Term(iTRAQ4plex); K10(iTRAQ	1	1
[R].QEELcLAR.[Q]	C5(Carbamidomethyl)	1	1
[R].qEELcLAR.[Q]	N-Term(iTRAQ4plex); C5(Carbam	1	1
[R].QEELcLAR.[Q]	C5(Carbamidomethyl)	1	1
[R].dPNGLPPEAQk.[I]	N-Term(iTRAQ4plex); K11(iTRAQ	1	1
[K].yWGVASFLQk.[G]	N-Term(iTRAQ4plex); K10(iTRAQ	1	1
[K].yWGVASFLQk.[G]	N-Term(iTRAQ4plex); K10(iTRAQ	1	1
[R].qEELcLAR.[Q]	N-Term(iTRAQ4plex); C5(Carbam	1	1
[R].dPNGLPPEAQk.[I]	N-Term(iTRAQ4plex); K11(iTRAQ	1	1
[R].qRQEELcLAR.[Q]	N-Term(iTRAQ4plex); C7(Carbam	1	1
[R].fSGTWYAMAk.[K]	N-Term(iTRAQ4plex); K10(iTRAQ	1	1
[R].fSGTWYAmAk.[K]	N-Term(iTRAQ4plex); M8(Oxidat	1	1
[R].lLNNWDVcADmVGTFTDTEDPAk.[F]	N-Term(iTRAQ4plex); C8(Carbam	1	1
[R].FSGTWYAMAk.[K]	K10(iTRAQ4plex)	1	1
[R].fSGTWYAMAk.[K]	N-Term(iTRAQ4plex); K10(iTRAQ	1	1
[R].fSGTWYAmAk.[K]	N-Term(iTRAQ4plex); M8(Oxidat	1	1
[R].QRQEELcLAR.[Q]	C7(Carbamidomethyl)	1	1
[K].DPTFIPAPIQAk.[T]	K12(iTRAQ4plex)	1	1
[R].sLDFTELDVAAEk.[I]	N-Term(iTRAQ4plex); K13(iTRAQ	1	1
[R].sLDFTELDVAAEk.[I]	N-Term(iTRAQ4plex); K13(iTRAQ	1	1
[K].dPTFIPAPIQAk.[T]	N-Term(iTRAQ4plex); K12(iTRAQ	1	1
[K].dPTFIPAPIQAk.[T]	N-Term(iTRAQ4plex); K12(iTRAQ	1	1
[K].aLQDQLVLVAAk.[L]	N-Term(iTRAQ4plex); K12(iTRAQ	1	1
[K].aLQDQLVLVAAk.[L]	N-Term(iTRAQ4plex); K12(iTRAQ	1	1
[K].aLQDQLVLVAAk.[L]	N-Term(iTRAQ4plex); K12(iTRAQ	1	1
[K].qPFVQGLALYTPVVLPR.[S]	N-Term(iTRAQ4plex)	1	1
[K].qPFVQGLALYTPVVLPR.[S]	N-Term(iTRAQ4plex)	1	1
[R].SLDFTELDVAAEk.[I]	K13(iTRAQ4plex)	1	1
[R].lQAILGVPWk.[D]	N-Term(iTRAQ4plex); K10(iTRAQ	1	1
[R].lQAILGVPWk.[D]	N-Term(iTRAQ4plex); K10(iTRAQ	1	1
[R].fMQAVTGWk.[T]	N-Term(iTRAQ4plex); K9(iTRAQ4	1	1
[R].fMQAVTGWk.[T]	N-Term(iTRAQ4plex); K9(iTRAQ4	1	1
[R].fMQAVTGWk.[T]	N-Term(iTRAQ4plex); K9(iTRAQ4	1	1
[R].fMQAVTGWk.[T]	N-Term(iTRAQ4plex); K9(iTRAQ4	1	1
[K].vLSALQAVQGLLVAQGR.[A]	N-Term(iTRAQ4plex)	1	1
[K].vLSALQAVQGLLVAQGR.[A]	N-Term(iTRAQ4plex)	1	1
[R].tIHLTMPQLVLQGSYDLQDLLAQAELPAIL	N-Term(iTRAQ4plex); K38(iTRAQ	1	1
[R].aAMVGMLANFLGFR.[I]	N-Term(iTRAQ4plex)	1	1
[R].sLDFTELDVAAEk.[I]	N-Term(iTRAQ4plex); K13(iTRAQ	1	1
[R].fmQAVTGWk.[T]	N-Term(iTRAQ4plex); M2(Oxidat	1	1
[R].LQAILGVPWk.[D]	K10(iTRAQ4plex)	1	1
[K].aLQDQLVLVAAk.[L]	N-Term(iTRAQ4plex); K12(iTRAQ	1	1
[K].aLQDQLVLVAAk.[L]	N-Term(iTRAQ4plex); K12(iTRAQ	1	1
[R].FMQAVTGWk.[T]	K9(iTRAQ4plex)	1	1
[K].DPTFIPAPIQAk.[T]	K12(iTRAQ4plex)	1	1

[R].fMQAVTGWk.[T]	N-Term(iTRAQ4plex); K9(iTRAQ4	1	1
[K].dPTFIPAPIQAk.[T]	N-Term(iTRAQ4plex); K12(iTRAQ	1	1
[K].TSPVDEk.[A]	K7(iTRAQ4plex)	1	1
[R].lQAILGVPWk.[D]	N-Term(iTRAQ4plex); K10(iTRAQ	1	1
[K].tSPVDEk.[A]	N-Term(iTRAQ4plex); K7(iTRAQ4	1	1
[K].dPTFIPAPIQAk.[T]	N-Term(iTRAQ4plex); K12(iTRAQ	1	1
[K].tSPVDEk.[A]	N-Term(iTRAQ4plex); K7(iTRAQ4	1	1
[R].FMQAVTGWk.[T]	K9(iTRAQ4plex)	1	1
[K].tSPVDEk.[A]	N-Term(iTRAQ4plex); K7(iTRAQ4	1	1
[K].tSPVDEk.[A]	N-Term(iTRAQ4plex); K7(iTRAQ4	1	1
[K].hLVALSPk.[K]	N-Term(iTRAQ4plex); K8(iTRAQ4	1	1
[R].sETEIHQGFQHLHQLFAk.[S]	N-Term(iTRAQ4plex); K18(iTRAQ	1	1
[R].sETEIHQGFQHLHQLFAk.[S]	N-Term(iTRAQ4plex); K18(iTRAQ	1	1
[R].sETEIHQGFQHLHQLFAk.[S]	N-Term(iTRAQ4plex); K18(iTRAQ	1	1
[R].sETEIHQGFQHLHQLFAk.[S]	N-Term(iTRAQ4plex); K18(iTRAQ	1	1
[R].sETEIHQGFQHLHQLFAk.[S]	N-Term(iTRAQ4plex); K18(iTRAQ	1	1
[R].sETEIHQGFQHLHQLFAk.[S]	N-Term(iTRAQ4plex); K18(iTRAQ	1	1
[K].HLVALSPk.[K]	K8(iTRAQ4plex)	1	1
[K].hYYESEVLAMNFQDWATASR.[Q]	N-Term(iTRAQ4plex)	1	1
[K].hYYESEVLAMNFQDWATASR.[Q]	N-Term(iTRAQ4plex)	1	1
[K].gTWTQPFDLASTR.[E]	N-Term(iTRAQ4plex)	1	1
[K].gTWTQPFDLASTR.[E]	N-Term(iTRAQ4plex)	1	1
[R].wSAGLTSSQVDLYIPk.[V]	N-Term(iTRAQ4plex); K16(iTRAQ	1	1
[K].hLVALSPk.[K]	N-Term(iTRAQ4plex); K8(iTRAQ4	1	1
[K].hLVALSPk.[K]	N-Term(iTRAQ4plex); K8(iTRAQ4	1	1
[K].hYYESEVLAMNFQDWATASR.[Q]	N-Term(iTRAQ4plex)	1	1
[R].eENFYVDETTVVk.[V]	N-Term(iTRAQ4plex); K13(iTRAQ	1	1
[R].gLASANVDFAFSLYk.[H]	N-Term(iTRAQ4plex); K15(iTRAQ	1	1
[R].gLASANVDFAFSLYk.[H]	N-Term(iTRAQ4plex); K15(iTRAQ	1	1
[K].mNTVIAALSR.[D]	N-Term(iTRAQ4plex)	1	1
[R].sETEIHQGFQHLHQLFAk.[S]	N-Term(iTRAQ4plex); K18(iTRAQ	1	1
[R].sETEIHQGFQHLHQLFAk.[S]	N-Term(iTRAQ4plex); K18(iTRAQ	1	1
[R].eENFYVDETTVVk.[V]	N-Term(iTRAQ4plex); K13(iTRAQ	1	1
[K].GTWTQPFDLASTR.[E]		1	1
[R].wSAGLTSSQVDLYIPk.[V]	N-Term(iTRAQ4plex); K16(iTRAQ	1	1
[K].hLVALSPk.[K]	N-Term(iTRAQ4plex); K8(iTRAQ4	1	1
[R].gLASANVDFAFSLYk.[H]	N-Term(iTRAQ4plex); K15(iTRAQ	1	1
[R].iTQDAQLk.[S]	N-Term(iTRAQ4plex); K8(iTRAQ4	1	1
[R].gLASANVDFAFSLYk.[H]	N-Term(iTRAQ4plex); K15(iTRAQ	1	1
[K].hLVALSPk.[K]	N-Term(iTRAQ4plex); K8(iTRAQ4	1	1
[K].HLVALSPk.[K]	K8(iTRAQ4plex)	1	1
[R].qINSYVk.[N]	N-Term(iTRAQ4plex); K7(iTRAQ4	1	1
[R].wSAGLTSSQVDLYIPk.[V]	N-Term(iTRAQ4plex); K16(iTRAQ	1	1
[R].SETEIHQGFQHLHQLFAk.[S]	K18(iTRAQ4plex)	1	1
[K].mNTVIAALSR.[D]	N-Term(iTRAQ4plex)	1	1
[R].gLASANVDFAFSLYk.[H]	N-Term(iTRAQ4plex); K15(iTRAQ	1	1
[K].gTWTQPFDLASTR.[E]	N-Term(iTRAQ4plex)	1	1
[R].wSAGLTSSQVDLYIPk.[V]	N-Term(iTRAQ4plex); K16(iTRAQ	1	1
[R].wSAGLTSSQVDLYIPk.[V]	N-Term(iTRAQ4plex); K16(iTRAQ	1	1
[R].iQNILTEEPk.[V]	N-Term(iTRAQ4plex); K10(iTRAQ	1	1
[R].iQNILTEEPk.[V]	N-Term(iTRAQ4plex); K10(iTRAQ	1	1
[K].lLIGTVFHk.[AT]	N-Term(iTRAQ4plex); K9(iTRAQ4	1	1

[R].eVQPVELPNcNLVk.[G]	N-Term(iTRAQ4plex); C10(Carbar	1	1
[R].eVQPVELPNcNLVk.[G]	N-Term(iTRAQ4plex); C10(Carbar	1	1
[K].iFFYDSENPPASEVLR.[I]	N-Term(iTRAQ4plex)	1	1
[K].iFFYDSENPPASEVLR.[I]	N-Term(iTRAQ4plex)	1	1
[K].iFFYDSENPPASEVLR.[I]	N-Term(iTRAQ4plex)	1	1
[K].yVYIAELLAHk.[I]	N-Term(iTRAQ4plex); K11(iTRAQ	1	1
[K].yVYIAELLAHk.[I]	N-Term(iTRAQ4plex); K11(iTRAQ	1	1
[K].yVYIAELLAHk.[I]	N-Term(iTRAQ4plex); K11(iTRAQ	1	1
[K].fDVSSFNPHGISTFTDEDNAMYLLVVNHPD	N-Term(iTRAQ4plex); K32(iTRAQ	1	1
[K].fDVSSFNPHGISTFTDEDNAMYLLVVNHPD	N-Term(iTRAQ4plex); K32(iTRAQ	1	1
[R].iQNILTEEPk.[V]	N-Term(iTRAQ4plex); K10(iTRAQ	1	1
[R].iQNILTEEPk.[V]	N-Term(iTRAQ4plex); K10(iTRAQ	1	1
[K].iLLMDLNEEDPTVLELGITGSk.[F]	N-Term(iTRAQ4plex); K22(iTRAQ	1	1
[K].iFFYDSENPPASEVLR.[I]	N-Term(iTRAQ4plex)	1	1
[K].yVYIAELLAHk.[I]	N-Term(iTRAQ4plex); K11(iTRAQ	1	1
[K].sTVELFk.[F]	N-Term(iTRAQ4plex); K7(iTRAQ4	1	1
[R].nHQSSYQTR.[L]	N-Term(iTRAQ4plex)	1	1
[K].sFNPNSPGk.[I]	N-Term(iTRAQ4plex); K9(iTRAQ4	1	1
[K].LLIGTVFHk.[AT]	K9(iTRAQ4plex)	1	1
[R].iQNILTEEPk.[V]	N-Term(iTRAQ4plex); K10(iTRAQ	1	1
[K].lLIGTVFHk.[AT]	N-Term(iTRAQ4plex); K9(iTRAQ4	1	1
[K].lLIGTVFHk.[AT]	N-Term(iTRAQ4plex); K9(iTRAQ4	1	1
[K].sFNPNSPGk.[I]	N-Term(iTRAQ4plex); K9(iTRAQ4	1	1
[R].iQNILTEEPk.[V]	N-Term(iTRAQ4plex); K10(iTRAQ	1	1
[K].dDINSYEcWcPFGFEGk.[N]	N-Term(iTRAQ4plex); C8(Carbam	1	1
[K].fGSGYVSGWGR.[V]	N-Term(iTRAQ4plex)	1	1
[R].iIPHHNYNAAINk.[Y]	N-Term(iTRAQ4plex); K13(iTRAQ	1	1
[R].iIPHHNYNAAINk.[Y]	N-Term(iTRAQ4plex); K13(iTRAQ	1	1
[K].wIVTAAHcVETGVk.[I]	N-Term(iTRAQ4plex); C8(Carbam	1	1
[K].wIVTAAHcVETGVk.[I]	N-Term(iTRAQ4plex); C8(Carbam	1	1
[K].wIVTAAHcVETGVk.[I]	N-Term(iTRAQ4plex); C8(Carbam	1	1
[K].iTVVAGEHNIEETEHTEQk.[R]	N-Term(iTRAQ4plex); K19(iTRAQ	1	1
[R].vSVSQTSk.[L]	N-Term(iTRAQ4plex); K8(iTRAQ4	1	1
[R].iIPHHNYNAAINk.[Y]	N-Term(iTRAQ4plex); K13(iTRAQ	1	1
[K].eYTNIFLk.[F]	N-Term(iTRAQ4plex); K8(iTRAQ4	1	1
[K].ncELDVTcNIk.[N]	N-Term(iTRAQ4plex); C2(Carbam	1	1
[R].sALVLQYLR.[V]	N-Term(iTRAQ4plex)	1	1
[R].iIPHHNYNAAINk.[Y]	N-Term(iTRAQ4plex); K13(iTRAQ	1	1
[K].fGSGYVSGWGR.[V]	N-Term(iTRAQ4plex)	1	1
[K].vDAFcGGSIVNEk.[W]	N-Term(iTRAQ4plex); C5(Carbam	1	1
[R].SALVLQYLR.[V]		1	1
[K].eYTNIFLk.[F]	N-Term(iTRAQ4plex); K8(iTRAQ4	1	1
[R].aNLQSVPHASASRPR.[V]	N-Term(iTRAQ4plex)	1	1
[R].aNLQSVPHASASRPR.[V]	N-Term(iTRAQ4plex)	1	1
[R].aLADGVQk.[V]	N-Term(iTRAQ4plex); K8(iTRAQ4	1	1
[R].aLADGVQk.[V]	N-Term(iTRAQ4plex); K8(iTRAQ4	1	1
[K].sETAEELkk.[V]	N-Term(iTRAQ4plex); K8(iTRAQ4	1	1
[K].wWTQAQAHDLVIk.[S]	N-Term(iTRAQ4plex); K13(iTRAQ	1	1
[K].sELEDNIR.[R]	N-Term(iTRAQ4plex)	1	1
[K].vAQELEEk.[L]	N-Term(iTRAQ4plex); K8(iTRAQ4	1	1
[K].lNILNNNYk.[I]	N-Term(iTRAQ4plex); K9(iTRAQ4	1	1
[K].sETAEELkk.[V]	N-Term(iTRAQ4plex); K8(iTRAQ4	1	1

[K].vAQELEEk.[L]	N-Term(iTRAQ4plex); K8(iTRAQ4	1	1
[K].vAQELEEk.[L]	N-Term(iTRAQ4plex); K8(iTRAQ4	1	1
[K].iLQADQEL.[-]	N-Term(iTRAQ4plex)	1	1
[R].nEADELR.[K]	N-Term(iTRAQ4plex)	1	1
[K].vAQELEEk.[L]	N-Term(iTRAQ4plex); K8(iTRAQ4	1	1
[K].vAQELEEk.[L]	N-Term(iTRAQ4plex); K8(iTRAQ4	1	1
[K].sETAEELk.[K]	N-Term(iTRAQ4plex); K8(iTRAQ4	1	1
[K].iLQADQEL.[-]	N-Term(iTRAQ4plex)	1	1
[K].lNILNNNYk.[I]	N-Term(iTRAQ4plex); K9(iTRAQ4	1	1
[K].tQILVGVNk.[D]	N-Term(iTRAQ4plex); K9(iTRAQ4	1	1
[K].tQILVGVNk.[D]	N-Term(iTRAQ4plex); K9(iTRAQ4	1	1
[K].nIAAFGGNPk.[S]	N-Term(iTRAQ4plex); K10(iTRAQ	1	1
[K].eSILFHYTDWVDDQRPENYR.[E]	N-Term(iTRAQ4plex)	1	1
[K].dEGTAFLVYGAPGFSk.[D]	N-Term(iTRAQ4plex); K16(iTRAQ	1	1
[K].iFFPGVSEFGk.[E]	N-Term(iTRAQ4plex); K11(iTRAQ	1	1
[K].iFFPGVSEFGk.[E]	N-Term(iTRAQ4plex); K11(iTRAQ	1	1
[K].yLTLNTESTR.[I]	N-Term(iTRAQ4plex)	1	1
[K].kTQILVGVNk.[D]	N-Term(iTRAQ4plex); K1(iTRAQ4	1	1
[R].kEFQEGLk.[I]	N-Term(iTRAQ4plex); K1(iTRAQ4	1	1
[K].nQFNDYTSk.[K]	N-Term(iTRAQ4plex); K9(iTRAQ4	1	1
[R].fWTSFFPk.[V]	N-Term(iTRAQ4plex); K8(iTRAQ4	1	1
[K].IFFPGVSEFGk.[E]	K11(iTRAQ4plex)	1	1
[R].hTFmGVVSLGSPSGEVSHPR.[K]	N-Term(iTRAQ4plex); M4(Oxidat	1	1
[R].hTFmGVVSLGSPSGEVSHPR.[K]	N-Term(iTRAQ4plex); M4(Oxidat	1	1
[R].hTFmGVVSLGSPSGEVSHPR.[K]	N-Term(iTRAQ4plex); M4(Oxidat	1	1
[R].hTFMGVVSLGSPSGEVSHPR.[K]	N-Term(iTRAQ4plex)	1	1
[R].hTFmGVVSLGSPSGEVSHPR.[K]	N-Term(iTRAQ4plex); M4(Oxidat	1	1
[R].hTFmGVVSLGSPSGEVSHPR.[K]	N-Term(iTRAQ4plex); M4(Oxidat	1	1
[R].hTFMGVVSLGSPSGEVSHPR.[K]	N-Term(iTRAQ4plex)	1	1
[R].hTFmGVVSLGSPSGEVSHPR.[K]	N-Term(iTRAQ4plex); M4(Oxidat	1	1
[R].hTFmGVVSLGSPSGEVSHPR.[K]	N-Term(iTRAQ4plex); M4(Oxidat	1	1
[R].hTFmGVVSLGSPSGEVSHPR.[K]	N-Term(iTRAQ4plex); M4(Oxidat	1	1
[R].TVVQPSVGAAAGPVVPPcPGR.[I]	C18(Carbamidomethyl)	1	1
[R].TVVQPSVGAAAGPVVPPcPGR.[I]	C18(Carbamidomethyl)	1	1
[R].TVVQPSVGAAAGPVVPPcPGR.[I]	C18(Carbamidomethyl)	1	1
[R].HTFmGVVSLGSPSGEVSHPR.[K]	M4(Oxidation)	1	1
[R].hTFmGVVSLGSPSGEVSHPR.[K]	N-Term(iTRAQ4plex); M4(Oxidat	1	1
[R].hTFmGVVSLGSPSGEVSHPR.[K]	N-Term(iTRAQ4plex); M4(Oxidat	1	1
[R].hTFmGVVSLGSPSGEVSHPR.[K]	N-Term(iTRAQ4plex); M4(Oxidat	1	1
[R].hTFmGVVSLGSPSGEVSHPR.[K]	N-Term(iTRAQ4plex); M4(Oxidat	1	1
[R].hTFmGVVSLGSPSGEVSHPR.[K]	N-Term(iTRAQ4plex); M4(Oxidat	1	1
[R].tVVQPSVGAAAGPVVPPcPGR.[I]	N-Term(iTRAQ4plex); C18(Carbar	1	1
[R].hTFMGVVSLGSPSGEVSHPR.[K]	N-Term(iTRAQ4plex)	1	1
[R].hTFMGVVSLGSPSGEVSHPR.[K]	N-Term(iTRAQ4plex)	1	1
[R].hTFMGVVSLGSPSGEVSHPR.[K]	N-Term(iTRAQ4plex)	1	1
[R].hTFMGVVSLGSPSGEVSHPR.[K]	N-Term(iTRAQ4plex)	1	1
[R].HTFMGVVSLGSPSGEVSHPR.[K]		1	1
[R].hTFmGVVSLGSPSGEVSHPR.[K]	N-Term(iTRAQ4plex); M4(Oxidat	1	1
[R].hTFmGVVSLGSPSGEVSHPR.[K]	N-Term(iTRAQ4plex); M4(Oxidat	1	1
[R].hTFMGVVSLGSPSGEVSHPR.[K]	N-Term(iTRAQ4plex)	1	1
[R].hTFMGVVSLGSPSGEVSHPR.[K]	N-Term(iTRAQ4plex)	1	1
[R].hTFMGVVSLGSPSGEVSHPR.[K]	N-Term(iTRAQ4plex)	1	1

[R].hTFMGVVSLGSPSGEVSHPR.[K]	N-Term(iTRAQ4plex)	1	1
[R].HTFMGVVSLGSPSGEVSHPR.[K]		1	1
[R].hTFMGVVSLGSPSGEVSHPR.[K]	N-Term(iTRAQ4plex)	1	1
[R].hTFmGVVSLGSPSGEVSHPR.[K]	N-Term(iTRAQ4plex); M4(Oxidat	1	1
[R].hTFmGVVSLGSPSGEVSHPR.[K]	N-Term(iTRAQ4plex); M4(Oxidat	1	1
[R].hTFmGVVSLGSPSGEVSHPR.[K]	N-Term(iTRAQ4plex); M4(Oxidat	1	1
[R].HTFMGVVSLGSPSGEVSHPR.[K]		1	1
[R].HTFMGVVSLGSPSGEVSHPR.[K]		1	1
[R].HTFMGVVSLGSPSGEVSHPR.[K]		1	1
[R].HTFMGVVSLGSPSGEVSHPR.[K]		1	1
[R].qPNcDDPETEEAALVAIDYINQNLPWGYk.	N-Term(iTRAQ4plex); C4(Carbam	1	1
[R].qPNcDDPETEEAALVAIDYINQNLPWGYk.	N-Term(iTRAQ4plex); C4(Carbam	1	1
[R].tVVQPSVGAAAGPVVPPcPGR.[I]	N-Term(iTRAQ4plex); C18(Carbar	1	1
[R].tVVQPSVGAAAGPVVPPcPGR.[I]	N-Term(iTRAQ4plex); C18(Carbar	1	1
[R].tVVQPSVGAAAGPVVPPcPGR.[I]	N-Term(iTRAQ4plex); C18(Carbar	1	1
[R].tVVQPSVGAAAGPVVPPcPGR.[I]	N-Term(iTRAQ4plex); C18(Carbar	1	1
[K].vWPQQPSGELFEIEIDTLETTcHVLDPTPV/	N-Term(iTRAQ4plex); C22(Carbar	1	1
[K].vWPQQPSGELFEIEIDTLETTcHVLDPTPV/	N-Term(iTRAQ4plex); C22(Carbar	1	1
[R].tVVQPSVGAAAGPVVPPcPGR.[I]	N-Term(iTRAQ4plex); C18(Carbar	1	1
[R].TVVQPSVGAAAGPVVPPcPGR.[I]	C18(Carbamidomethyl)	1	1
[K].vWPQQPSGELFEIEIDTLETTcHVLDPTPV/	N-Term(iTRAQ4plex); C22(Carbar	1	1
[R].aQLVPLPPSTYVEFTVSGTDcVAk.[E]	N-Term(iTRAQ4plex); C21(Carbar	1	1
[K].hTLNQIDEVk.[V]	N-Term(iTRAQ4plex); K10(iTRAQ	1	1
[K].fSVVYAk.[C]	N-Term(iTRAQ4plex); K7(iTRAQ4	1	1
[R].AQLVPLPPSTYVEFTVSGTDcVAk.[E]	C21(Carbamidomethyl); K24(iTR	1	1
[R].aQLVPLPPSTYVEFTVSGTDcVAk.[E]	N-Term(iTRAQ4plex); C21(Carbar	1	1
[R].aQLVPLPPSTYVEFTVSGTDcVAk.[E]	N-Term(iTRAQ4plex); C21(Carbar	1	1
[K].eHAVEGDcDFQLLk.[L]	N-Term(iTRAQ4plex); C8(Carbam	1	1
[K].eHAVEGDcDFQLLk.[L]	N-Term(iTRAQ4plex); C8(Carbam	1	1
[K].eHAVEGDcDFQLLk.[L]	N-Term(iTRAQ4plex); C8(Carbam	1	1
[K].EHAVEGDcDFQLLk.[L]	C8(Carbamidomethyl); K14(iTRA	1	1
[K].eHAVEGDcDFQLLk.[L]	N-Term(iTRAQ4plex); C8(Carbam	1	1
[K].eHAVEGDcDFQLLk.[L]	N-Term(iTRAQ4plex); C8(Carbam	1	1
[K].eHAVEGDcDFQLLk.[L]	N-Term(iTRAQ4plex); C8(Carbam	1	1
[K].eHAVEGDcDFQLLk.[L]	N-Term(iTRAQ4plex); C8(Carbam	1	1
[K].eHAVEGDcDFQLLk.[L]	N-Term(iTRAQ4plex); C8(Carbam	1	1
[K].eHAVEGDcDFQLLk.[L]	N-Term(iTRAQ4plex); C8(Carbam	1	1
[K].eHAVEGDcDFQLLk.[L]	N-Term(iTRAQ4plex); C8(Carbam	1	1
[K].eHAVEGDcDFQLLk.[L]	N-Term(iTRAQ4plex); C8(Carbam	1	1
[K].eHAVEGDcDFQLLk.[L]	N-Term(iTRAQ4plex); C8(Carbam	1	1
[K].eHAVEGDcDFQLLk.[L]	N-Term(iTRAQ4plex); C8(Carbam	1	1
[K].EHAVEGDcDFQLLk.[L]	C8(Carbamidomethyl); K14(iTRA	1	1
[K].eHAVEGDcDFQLLk.[L]	N-Term(iTRAQ4plex); C8(Carbam	1	1
[R].aQLVPLPPSTYVEFTVSGTDcVAk.[E]	N-Term(iTRAQ4plex); C21(Carbar	1	1
[K].hTLNQIDEVk.[V]	N-Term(iTRAQ4plex); K10(iTRAQ	1	1
[K].hTLNQIDEVk.[V]	N-Term(iTRAQ4plex); K10(iTRAQ	1	1
[K].hTLNQIDEVk.[V]	N-Term(iTRAQ4plex); K10(iTRAQ	1	1
[K].hTLNQIDEVk.[V]	N-Term(iTRAQ4plex); K10(iTRAQ	1	1
[K].hTLNQIDEVk.[V]	N-Term(iTRAQ4plex); K10(iTRAQ	1	1
[K].HTLNQIDEVk.[V]	K10(iTRAQ4plex)	1	1
[K].eHAVEGDcDFQLLk.[L]	N-Term(iTRAQ4plex); C8(Carbam	1	1
[K].cDSSPDSAEDVR.[K]	C1(Carbamidomethyl)	1	1

[K].eHAVEGDcDFQLLk.[L]	N-Term(iTRAQ4plex); C8(Carbam	1	1
[R].aQLVPLPPSTYVEFTVSGTDcVAk.[E]	N-Term(iTRAQ4plex); C21(Carbai	1	1
[K].hTLNQIDEVk.[V]	N-Term(iTRAQ4plex); K10(iTRAQ	1	1
[K].cDSSPDSAEDVR.[K]	N-Term(iTRAQ4plex); C1(Carbam	1	1
[R].hTFmGVVSLGSPSGEVSHPR.[K]	N-Term(iTRAQ4plex); M4(Oxidat	1	1
[K].eATEAAk.[C]	N-Term(iTRAQ4plex); K7(iTRAQ4	1	1
[R].TVVQPSVGAAAGPVVPPcPGR.[I]	C18(Carbamidomethyl)	1	1
[K].eATEAAk.[C]	N-Term(iTRAQ4plex); K7(iTRAQ4	1	1
[K].hTLNQIDEVk.[V]	N-Term(iTRAQ4plex); K10(iTRAQ	1	1
[K].hTLNQIDEVk.[V]	N-Term(iTRAQ4plex); K10(iTRAQ	1	1
[K].eHAVEGDcDFQLLk.[L]	N-Term(iTRAQ4plex); C8(Carbam	1	1
[K].HTLNQIDEVk.[V]	K10(iTRAQ4plex)	1	1
[K].eHAVEGDcDFQLLk.[L]	N-Term(iTRAQ4plex); C8(Carbam	1	1
[K].fSVVYAk.[C]	N-Term(iTRAQ4plex); K7(iTRAQ4	1	1
[R].hTFMGVVSLGSPSGEVSHPR.[K]	N-Term(iTRAQ4plex)	1	1
[K].hTLNQIDEVk.[V]	N-Term(iTRAQ4plex); K10(iTRAQ	1	1
[K].HTLNQIDEVk.[V]	K10(iTRAQ4plex)	1	1
[K].eATEAAk.[C]	N-Term(iTRAQ4plex); K7(iTRAQ4	1	1
[K].fSVVYAk.[C]	N-Term(iTRAQ4plex); K7(iTRAQ4	1	1
[K].eATEAAk.[C]	N-Term(iTRAQ4plex); K7(iTRAQ4	1	1
[R].HTFmGVVSLGSPSGEVSHPR.[K]	M4(Oxidation)	1	1
[R].hTFmGVVSLGSPSGEVSHPR.[K]	N-Term(iTRAQ4plex); M4(Oxidat	1	1
[K].hTLNQIDEVk.[V]	N-Term(iTRAQ4plex); K10(iTRAQ	1	1
[R].hTFmGVVSLGSPSGEVSHPR.[K]	N-Term(iTRAQ4plex); M4(Oxidat	1	1
[K].fSVVYAk.[C]	N-Term(iTRAQ4plex); K7(iTRAQ4	1	1
[K].fSVVYAk.[C]	N-Term(iTRAQ4plex); K7(iTRAQ4	1	1
[K].hTLNQIDEVk.[V]	N-Term(iTRAQ4plex); K10(iTRAQ	1	1
[K].cDSSPDSAEDVR.[K]	C1(Carbamidomethyl)	1	1
[K].eATEAAk.[C]	N-Term(iTRAQ4plex); K7(iTRAQ4	1	1
[R].tVVQPSVGAAAGPVVPPcPGR.[I]	N-Term(iTRAQ4plex); C18(Carbai	1	1
[K].eHAVEGDcDFQLLk.[L]	N-Term(iTRAQ4plex); C8(Carbam	1	1
[K].FSVVYAk.[C]	K7(iTRAQ4plex)	1	1
[K].fSVVYAk.[C]	N-Term(iTRAQ4plex); K7(iTRAQ4	1	1
[R].hTFmGVVSLGSPSGEVSHPR.[K]	N-Term(iTRAQ4plex); M4(Oxidat	1	1
[K].eATEAAk.[C]	N-Term(iTRAQ4plex); K7(iTRAQ4	1	1
[K].eATEAAk.[C]	N-Term(iTRAQ4plex); K7(iTRAQ4	1	1
[K].fSVVYAk.[C]	N-Term(iTRAQ4plex); K7(iTRAQ4	1	1
[R].hTFmGVVSLGSPSGEVSHPR.[K]	N-Term(iTRAQ4plex); M4(Oxidat	1	1
[K].hTLNQIDEVk.[V]	N-Term(iTRAQ4plex); K10(iTRAQ	1	1
[R].hTFMGVVSLGSPSGEVSHPR.[K]	N-Term(iTRAQ4plex)	1	1
[R].hTFmGVVSLGSPSGEVSHPR.[K]	N-Term(iTRAQ4plex); M4(Oxidat	1	1
[K].fSVVYAk.[C]	N-Term(iTRAQ4plex); K7(iTRAQ4	1	1
[K].eATEAAk.[C]	N-Term(iTRAQ4plex); K7(iTRAQ4	1	1
[K].cNLLAEk.[Q]	N-Term(iTRAQ4plex); C1(Carbam	1	1
[K].FSVVYAk.[C]	K7(iTRAQ4plex)	1	1
[R].TVVQPSVGAAAGPVVPPcPGR.[I]	C18(Carbamidomethyl)	1	1
[K].HTLNQIDEVk.[V]	K10(iTRAQ4plex)	1	1
[K].FSVVYAk.[C]	K7(iTRAQ4plex)	1	1
[K].eHAVEGDcDFQLLk.[L]	N-Term(iTRAQ4plex); C8(Carbam	1	1
[R].hTFMGVVSLGSPSGEVSHPR.[K]	N-Term(iTRAQ4plex)	1	1
[K].HTLNQIDEVk.[V]	K10(iTRAQ4plex)	1	1
[K].fSVVYAk.[C]	N-Term(iTRAQ4plex); K7(iTRAQ4	1	1

[K].eHAVEGDcDFQLLk.[L]	N-Term(iTRAQ4plex); C8(Carbam	1	1
[R].hTFMGVVSLGSPSGEVSHPR.[K]	N-Term(iTRAQ4plex)	1	1
[R].tVVQPSVGAAAGPVVPPcPGR.[I]	N-Term(iTRAQ4plex); C18(Carbar	1	1
[K].eHAVEGDcDFQLLk.[L]	N-Term(iTRAQ4plex); C8(Carbam	1	1
[K].fSVVYAk.[C]	N-Term(iTRAQ4plex); K7(iTRAQ4	1	1
[K].HTLNQIDEVk.[V]	K10(iTRAQ4plex)	1	1
[K].EHAVEGDcDFQLLk.[L]	C8(Carbamidomethyl); K14(iTRA	1	1
[K].fSVVYAk.[C]	N-Term(iTRAQ4plex); K7(iTRAQ4	1	1
[K].eHAVEGDcDFQLLk.[L]	N-Term(iTRAQ4plex); C8(Carbam	1	1
[R].hTFMGVVSLGSPSGEVSHPR.[K]	N-Term(iTRAQ4plex)	1	1
[R].hTFmGVVSLGSPSGEVSHPR.[K]	N-Term(iTRAQ4plex); M4(Oxidat	1	1
[R].aQLVPLPPSTYVEFTVSGTDcVAk.[E]	N-Term(iTRAQ4plex); C21(Carbar	1	1
[K].EHAVEGDcDFQLLk.[L]	C8(Carbamidomethyl); K14(iTRA	1	1
[K].cDSSPDSAEDVR.[K]	N-Term(iTRAQ4plex); C1(Carbam	1	1
[K].FSVVYAk.[C]	K7(iTRAQ4plex)	1	1
[K].yVmLPVADQDQcIR.[H]	N-Term(iTRAQ4plex); M3(Oxidat	1	1
[K].yVmLPVADQDQcIR.[H]	N-Term(iTRAQ4plex); M3(Oxidat	1	1
[K].scAVAEYGVYVk.[V]	N-Term(iTRAQ4plex); C2(Carbam	2	2
[R].iLGGHLDAk.[G]	N-Term(iTRAQ4plex); K9(iTRAQ4	2	2
[R].iLGGHLDAk.[G]	N-Term(iTRAQ4plex); K9(iTRAQ4	2	2
[R].iLGGHLDAk.[G]	N-Term(iTRAQ4plex); K9(iTRAQ4	2	2
[R].nANFkFTDHLk.[Y]	N-Term(iTRAQ4plex); K5(iTRAQ4	1	1
[K].vTSIQDWVQk.[T]	N-Term(iTRAQ4plex); K10(iTRAQ	1	1
[R].iLGGHLDAk.[G]	N-Term(iTRAQ4plex); K9(iTRAQ4	2	2
[K].vVLHPNYSQVDIGLIk.[L]	N-Term(iTRAQ4plex); K16(iTRAQ	1	1
[K].sPVGVQPILNEHTFcAGMSk.[Y]	N-Term(iTRAQ4plex); C15(Carbar	1	1
[K].sPVGVQPILNEHTFcAGMSk.[Y]	N-Term(iTRAQ4plex); C15(Carbar	1	1
[K].dIAPTLTLYVGk.[K]	N-Term(iTRAQ4plex); K12(iTRAQ	2	2
[K].dIAPTLTLYVGk.[K]	N-Term(iTRAQ4plex); K12(iTRAQ	2	2
[K].sPVGVQPILNEHTFcAGMSk.[Y]	N-Term(iTRAQ4plex); C15(Carbar	1	1
[K].SPVGVQPILNEHTFcAGMSk.[Y]	C15(Carbamidomethyl); K20(iTR	1	1
[K].vTSIQDWVQk.[T]	N-Term(iTRAQ4plex); K10(iTRAQ	1	1
[K].vTSIQDWVQk.[T]	N-Term(iTRAQ4plex); K10(iTRAQ	1	1
[K].vTSIQDWVQk.[T]	N-Term(iTRAQ4plex); K10(iTRAQ	1	1
[K].vTSIQDWVQk.[T]	N-Term(iTRAQ4plex); K10(iTRAQ	1	1
[K].yVmLPVADQDQcIR.[H]	N-Term(iTRAQ4plex); M3(Oxidat	1	1
[K].yVmLPVADQDQcIR.[H]	N-Term(iTRAQ4plex); M3(Oxidat	1	1
[K].yVmLPVADQDQcIR.[H]	N-Term(iTRAQ4plex); M3(Oxidat	1	1
[K].vTSIQDWVQk.[T]	N-Term(iTRAQ4plex); K10(iTRAQ	1	1
[K].yVMLPVADQDQcIR.[H]	N-Term(iTRAQ4plex); C12(Carbar	1	1
[K].yVMLPVADQDQcIR.[H]	N-Term(iTRAQ4plex); C12(Carbar	1	1
[K].yVMLPVADQDQcIR.[H]	N-Term(iTRAQ4plex); C12(Carbar	1	1
[K].yVMLPVADQDQcIR.[H]	N-Term(iTRAQ4plex); C12(Carbar	1	1
[K].DIAPTLTLYVGk.[K]	K12(iTRAQ4plex)	2	2
[K].dIAPTLTLYVGk.[K]	N-Term(iTRAQ4plex); K12(iTRAQ	2	2
[K].dIAPTLTLYVGk.[K]	N-Term(iTRAQ4plex); K12(iTRAQ	2	2
[R].hYEGSTVPEkk.[T]	N-Term(iTRAQ4plex); K10(iTRAQ	1	1
[R].hYEGSTVPEk.[K]	N-Term(iTRAQ4plex); K10(iTRAQ	1	1
[R].hYEGSTVPEk.[K]	N-Term(iTRAQ4plex); K10(iTRAQ	1	1
[R].hYEGSTVPEk.[K]	N-Term(iTRAQ4plex); K10(iTRAQ	1	1
[R].hYEGSTVPEk.[K]	N-Term(iTRAQ4plex); K10(iTRAQ	1	1
[R].tEGDGVYTLNDk.[K]	N-Term(iTRAQ4plex); K12(iTRAQ	2	2

[R].tEGDGVYTLNDk.[K]	N-Term(iTRAQ4plex); K12(iTRAQ	2	2
[R].tEGDGVYTLNNEk.[Q]	N-Term(iTRAQ4plex); K13(iTRAQ	1	1
[R].tEGDGVYTLNNEk.[Q]	N-Term(iTRAQ4plex); K13(iTRAQ	1	1
[R].tEGDGVYTLNDk.[K]	N-Term(iTRAQ4plex); K12(iTRAQ	2	2
[R].tEGDGVYTLNNEk.[Q]	N-Term(iTRAQ4plex); K13(iTRAQ	1	1
[R].TEGDGVYTLNNEk.[Q]	K13(iTRAQ4plex)	1	1
[R].tEGDGVYTLNNEk.[Q]	N-Term(iTRAQ4plex); K13(iTRAQ	1	1
[R].tEGDGVYTLNNEk.[Q]	N-Term(iTRAQ4plex); K13(iTRAQ	1	1
[R].tEGDGVYTLNNEk.[Q]	N-Term(iTRAQ4plex); K13(iTRAQ	1	1
[R].tEGDGVYTLNNEk.[Q]	N-Term(iTRAQ4plex); K13(iTRAQ	1	1
[K].scAVAEYGVYVk.[V]	N-Term(iTRAQ4plex); C2(Carbam	2	2
[K].scAVAEYGVYVk.[V]	N-Term(iTRAQ4plex); C2(Carbam	2	2
[K].scAVAEYGVYVk.[V]	N-Term(iTRAQ4plex); C2(Carbam	2	2
[K].scAVAEYGVYVk.[V]	N-Term(iTRAQ4plex); C2(Carbam	2	2
[K].scAVAEYGVYVk.[V]	N-Term(iTRAQ4plex); C2(Carbam	2	2
[K].lPEcEADDGcPkPPEIAHGYVEHSVR.[Y]	N-Term(iTRAQ4plex); C4(Carbam	1	1
[K].lPEcEADDGcPkPPEIAHGYVEHSVR.[Y]	N-Term(iTRAQ4plex); C4(Carbam	1	1
[K].scAVAEYGVYVk.[V]	N-Term(iTRAQ4plex); C2(Carbam	2	2
[K].scAVAEYGVYVk.[V]	N-Term(iTRAQ4plex); C2(Carbam	2	2
[K].scAVAEYGVYVk.[V]	N-Term(iTRAQ4plex); C2(Carbam	2	2
[K].lPEcEADDGcPkPPEIAHGYVEHSVR.[Y]	N-Term(iTRAQ4plex); C4(Carbam	1	1
[K].scAVAEYGVYVk.[V]	N-Term(iTRAQ4plex); C2(Carbam	2	2
[K].scAVAEYGVYVk.[V]	N-Term(iTRAQ4plex); C2(Carbam	2	2
[K].scAVAEYGVYVk.[V]	N-Term(iTRAQ4plex); C2(Carbam	2	2
[R].vMPIcLPSk.[ND]	N-Term(iTRAQ4plex); C5(Carbam	2	2
[K].lPEcEAVcGkPk.[N]	N-Term(iTRAQ4plex); C4(Carbam	2	2
[K].lPEcEAVcGkPk.[N]	N-Term(iTRAQ4plex); C4(Carbam	2	2
[K].kQLVEIEk.[V]	N-Term(iTRAQ4plex); K1(iTRAQ4	2	2
[K].dIAPTLTLYVGkk.[Q]	N-Term(iTRAQ4plex); K12(iTRAQ	2	2
[K].vTSIQDWVQk.[T]	N-Term(iTRAQ4plex); K10(iTRAQ	1	1
[R].hYEGSTVPEk.[K]	N-Term(iTRAQ4plex); K10(iTRAQ	1	1
[R].vGYVSGWGR.[N]	N-Term(iTRAQ4plex)	1	1
[R].hYEGSTVPEk.[K]	N-Term(iTRAQ4plex); K10(iTRAQ	1	1
[R].VMPIcLPSk.[ND]	C5(Carbamidomethyl); K9(iTRAC	2	2
[R].hYEGSTVPEk.[K]	N-Term(iTRAQ4plex); K10(iTRAQ	1	1
[R].hYEGSTVPEk.[K]	N-Term(iTRAQ4plex); K10(iTRAQ	1	1
[K].scAVAEYGVYVk.[V]	N-Term(iTRAQ4plex); C2(Carbam	2	2
[K].dIAPTLTLYVGk.[K]	N-Term(iTRAQ4plex); K12(iTRAQ	2	2
[K].gSFPWQAk.[M]	N-Term(iTRAQ4plex); K8(iTRAQ4	2	2
[R].hYEGSTVPEk.[K]	N-Term(iTRAQ4plex); K10(iTRAQ	1	1
[K].gSFPWQAk.[M]	N-Term(iTRAQ4plex); K8(iTRAQ4	2	2
[K].gSFPWQAk.[M]	N-Term(iTRAQ4plex); K8(iTRAQ4	2	2
[K].scAVAEYGVYVk.[V]	N-Term(iTRAQ4plex); C2(Carbam	2	2
[K].yVMLPVADQDQcIR.[H]	N-Term(iTRAQ4plex); C12(Carbar	1	1
[R].hYEGSTVPEkk.[T]	N-Term(iTRAQ4plex); K10(iTRAQ	1	1
[R].hYEGSTVPEk.[K]	N-Term(iTRAQ4plex); K10(iTRAQ	1	1
[R].hYEGSTVPEk.[K]	N-Term(iTRAQ4plex); K10(iTRAQ	1	1
[R].vMPIcLPSk.[ND]	N-Term(iTRAQ4plex); C5(Carbam	2	2
[K].VTSIQDWVQk.[T]	K10(iTRAQ4plex)	1	1
[R].hYEGSTVPEk.[K]	N-Term(iTRAQ4plex); K10(iTRAQ	1	1
[R].VMPIcLPSk.[ND]	C5(Carbamidomethyl); K9(iTRAC	2	2
[R].iLGGHLDAk.[G]	N-Term(iTRAQ4plex); K9(iTRAQ4	2	2

[R].hYEGSTVPEk.[K]	N-Term(iTRAQ4plex); K10(iTRAQ	1	1
[K].dIAPTLTLYVGk.[K]	N-Term(iTRAQ4plex); K12(iTRAQ	2	2
[R].vMPIcLPSk.[ND]	N-Term(iTRAQ4plex); C5(Carbam	2	2
[R].vmPIcLPSk.[ND]	N-Term(iTRAQ4plex); M2(Oxidat	2	2
[R].tEGDGVYTLNNEk.[Q]	N-Term(iTRAQ4plex); K13(iTRAQ	1	1
[R].iLGGHLDAk.[G]	N-Term(iTRAQ4plex); K9(iTRAQ4	2	2
[K].QLVEIEk.[V]	K7(iTRAQ4plex)	2	2
[R].iLGGHLDAk.[G]	N-Term(iTRAQ4plex); K9(iTRAQ4	2	2
[K].VTSIQDWVQk.[T]	K10(iTRAQ4plex)	1	1
[R].iLGGHLDAk.[G]	N-Term(iTRAQ4plex); K9(iTRAQ4	2	2
[R].vGYVSGWGR.[N]	N-Term(iTRAQ4plex)	1	1
[K].lkQkVSVNER.[V]	N-Term(iTRAQ4plex); K2(iTRAQ4	1	1
[R].TEGDGVYTLNDk.[K]	K12(iTRAQ4plex)	2	2
[K].qLVEIEk.[V]	N-Term(iTRAQ4plex); K7(iTRAQ4	2	2
[R].ILGGHLDAk.[G]	K9(iTRAQ4plex)	2	2
[K].yVmLPVADQDQcIR.[H]	N-Term(iTRAQ4plex); M3(Oxidat	1	1
[K].yVMLPVADQDQcIR.[H]	N-Term(iTRAQ4plex); C12(Carbar	1	1
[R].hYEGSTVPEk.[K]	N-Term(iTRAQ4plex); K10(iTRAQ	1	1
[K].YVMLPVADQDQcIR.[H]	C12(Carbamidomethyl)	1	1
[R].hYEGSTVPEkk.[T]	N-Term(iTRAQ4plex); K10(iTRAQ	1	1
[K].gSFPWQAk.[M]	N-Term(iTRAQ4plex); K8(iTRAQ4	2	2
[R].VMPIcLPSk.[ND]	C5(Carbamidomethyl); K9(iTRAC	2	2
[K].gSFPWQAk.[M]	N-Term(iTRAQ4plex); K8(iTRAQ4	2	2
[K].vTSIQDWVQk.[T]	N-Term(iTRAQ4plex); K10(iTRAQ	1	1
[K].yVMLPVADQDQcIR.[H]	N-Term(iTRAQ4plex); C12(Carbar	1	1
[K].gSFPWQAk.[M]	N-Term(iTRAQ4plex); K8(iTRAQ4	2	2
[R].vGYVSGWGR.[N]	N-Term(iTRAQ4plex)	1	1
[R].vGYVSGWGR.[N]	N-Term(iTRAQ4plex)	1	1
[R].hYEGSTVPEk.[K]	N-Term(iTRAQ4plex); K10(iTRAQ	1	1
[K].YVMLPVADQDQcIR.[H]	C12(Carbamidomethyl)	1	1
[R].VGYVSGWGR.[N]		1	1
[K].gSFPWQAk.[M]	N-Term(iTRAQ4plex); K8(iTRAQ4	2	2
[R].vGYVSGWGR.[N]	N-Term(iTRAQ4plex)	1	1
[R].tEGDGVYTLNDk.[K]	N-Term(iTRAQ4plex); K12(iTRAQ	2	2
[R].vGYVSGWGR.[N]	N-Term(iTRAQ4plex)	1	1
[R].hYEGSTVPEk.[K]	N-Term(iTRAQ4plex); K10(iTRAQ	1	1
[R].vmPIcLPSk.[ND]	N-Term(iTRAQ4plex); M2(Oxidat	2	2
[K].yVMLPVADQDQcIR.[H]	N-Term(iTRAQ4plex); C12(Carbar	1	1
[K].yVMLPVADQDQcIR.[H]	N-Term(iTRAQ4plex); C12(Carbar	1	1
[R].tEGDGVYTLNNEk.[Q]	N-Term(iTRAQ4plex); K13(iTRAQ	1	1
[K].vVLHPNYSQVDIGLIk.[L]	N-Term(iTRAQ4plex); K16(iTRAQ	1	1
[K].vVLHPNYSQVDIGLIk.[L]	N-Term(iTRAQ4plex); K16(iTRAQ	1	1
[K].VTSIQDWVQk.[T]	K10(iTRAQ4plex)	1	1
[K].YVMLPVADQDQcIR.[H]	C12(Carbamidomethyl)	1	1
[R].hYEGSTVPEkk.[T]	N-Term(iTRAQ4plex); K10(iTRAQ	1	1
[K].vVLHPNYSQVDIGLIk.[L]	N-Term(iTRAQ4plex); K16(iTRAQ	1	1
[K].yVmLPVADQDQcIR.[H]	N-Term(iTRAQ4plex); M3(Oxidat	1	1
[R].tEGDGVYTLNNEk.[Q]	N-Term(iTRAQ4plex); K13(iTRAQ	1	1
[K].qLVEIEk.[V]	N-Term(iTRAQ4plex); K7(iTRAQ4	2	2
[R].GGSGGSYGGGGSGGGYGGGSGSR.[G]		1	1
[R].SGGGGGGGLGSGGSIR.[S]		1	1
[R].FSSSSGYGGGSSR.[V]		1	1

[K].vQALEEANNDLENk.[I]	N-Term(iTRAQ4plex); K14(iTRAQ	1	1
[R].sGGGGGGGLGSGGSIR.[S]	N-Term(iTRAQ4plex)	1	1
[R].fSSSSGYGGGSSR.[V]	N-Term(iTRAQ4plex)	1	1
[K].tLLDIDNTR.[M]	N-Term(iTRAQ4plex)	1	1
[R].qEYEQLIAk.[N]	N-Term(iTRAQ4plex); K9(iTRAQ4	1	1
[R].fSSSGGGGGGGR.[F]	N-Term(iTRAQ4plex)	1	1
[R].qVLDNLTmEk.[S]	N-Term(iTRAQ4plex); M8(Oxidat	1	1
[K].fEMEQNLR.[Q]	N-Term(iTRAQ4plex)	1	1
[K].STmQELNSR.[L]	M3(Oxidation)	1	1
[R].SGGGGGGGLGSGGSIR.[S]		1	1
[R].FSSSSGYGGGSSR.[V]		1	1
[R].mTLDDFR.[I]	N-Term(iTRAQ4plex)	1	1
[K].STMQELNSR.[L]		1	1
[R].sLVNLGGSk.[S]	N-Term(iTRAQ4plex); K9(iTRAQ4	1	1
[K].wELLQQVDTSTR.[T]	N-Term(iTRAQ4plex)	1	1
[K].wELLQQVDTSTR.[T]	N-Term(iTRAQ4plex)	1	1
[R].fLEQQNQVLQTk.[W]	N-Term(iTRAQ4plex); K12(iTRAQ	2	3
[R].sLDLDSIIAEVk.[A]	N-Term(iTRAQ4plex); K12(iTRAQ	1	1
[R].tNAENEFVTIk.[K]	N-Term(iTRAQ4plex); K11(iTRAQ	1	1
[R].tLLEGEESR.[M]	N-Term(iTRAQ4plex)	1	1
[R].sLVNLGGSk.[S]	N-Term(iTRAQ4plex); K9(iTRAQ4	1	1
[R].tLLEGEESR.[M]	N-Term(iTRAQ4plex)	1	1
[R].tLLEGEESR.[M]	N-Term(iTRAQ4plex)	1	1
[K].wELLQQVDTSTR.[T]	N-Term(iTRAQ4plex)	1	1
[K].dVDGAYMTk.[V]	N-Term(iTRAQ4plex); K9(iTRAQ4	1	1
[R].TLLEGEESR.[M]		1	1
[R].fSScGGGGGSFGAGGGFGSR.[S]	N-Term(iTRAQ4plex); C4(Carbam	1	1
[K].aQYEDIAQk.[S]	N-Term(iTRAQ4plex); K9(iTRAQ4	1	1
[K].sISISVAR.[G]	N-Term(iTRAQ4plex)	1	1
[R].sEIDNVk.[K]	N-Term(iTRAQ4plex); K7(iTRAQ4	1	1
[K].sLNNQFASFIDk.[V]	N-Term(iTRAQ4plex); K12(iTRAQ	1	1
[K].dVDGAYMTk.[V]	N-Term(iTRAQ4plex); K9(iTRAQ4	1	1
[R].FLEQQNQVLQTk.[W]	K12(iTRAQ4plex)	2	3
[K].wcATTHNYDR.[D]	N-Term(iTRAQ4plex); C2(Carbam	1	1
[R].sQFVQPIcLPEPGSTFPAGHk.[C]	N-Term(iTRAQ4plex); C8(Carbam	1	1
[R].kWcATTHNYDR.[D]	N-Term(iTRAQ4plex); K1(iTRAQ4	1	1
[R].mLHAcTSEGSAHR.[K]	N-Term(iTRAQ4plex); C5(Carbam	1	1
[R].vQLSPDLLATLPEPASPGR.[Q]	N-Term(iTRAQ4plex)	1	1
[R].tTDVTQTFGIEk.[Y]	N-Term(iTRAQ4plex); K12(iTRAQ	1	1
[K].wcATTHNYDR.[D]	N-Term(iTRAQ4plex); C2(Carbam	1	1
[K].sDAcQGDSGGPLAcEk.[N]	N-Term(iTRAQ4plex); C4(Carbam	1	1
[R].sQFVQPIcLPEPGSTFPAGHk.[C]	N-Term(iTRAQ4plex); C8(Carbam	1	1
[R].qGHVEQcEcFGGR.[T]	N-Term(iTRAQ4plex); C7(Carbam	1	1
[K].sDAcQGDSGGPLAcEk.[N]	N-Term(iTRAQ4plex); C4(Carbam	1	1
[R].lcNIEPDER.[C]	N-Term(iTRAQ4plex); C2(Carbam	1	1
[R].nPDNDERPWcYVVk.[D]	N-Term(iTRAQ4plex); C10(Carbar	1	1
[R].lcNIEPDER.[C]	N-Term(iTRAQ4plex); C2(Carbam	1	1
[R].yEYLEGGDR.[W]	N-Term(iTRAQ4plex)	1	1
[R].lcNIEPDER.[C]	N-Term(iTRAQ4plex); C2(Carbam	1	1
[K].sDAcQGDSGGPLAcEk.[N]	N-Term(iTRAQ4plex); C4(Carbam	1	1
[K].aLSGFSLQScR.[H]	N-Term(iTRAQ4plex); C10(Carbar	1	1
[R].nVIYTDcWVTGWGYR.[K]	N-Term(iTRAQ4plex); C7(Carbam	1	1

[K].qcSHQISAcNk.[D]	N-Term(iTRAQ4plex); C2(Carbam	1	1
[R].qFPSLEHR.[N]	N-Term(iTRAQ4plex)	1	1
[K].iPLVTNEEcQk.[R]	N-Term(iTRAQ4plex); C9(Carbam	1	1
[K].scALSNLAcIR.[D]	N-Term(iTRAQ4plex); C2(Carbam	1	1
[K].mAESGYDIALLk.[L]	N-Term(iTRAQ4plex); K12(iTRAQ	1	1
[K].dTcFEGGDITTVFTPSAk.[Y]	N-Term(iTRAQ4plex); C3(Carbam	1	1
[K].vVSGFSLk.[S]	N-Term(iTRAQ4plex); K8(iTRAQ4	1	1
[K].dSVTETLPR.[V]	N-Term(iTRAQ4plex)	1	1
[K].iQNTLQk.[A]	N-Term(iTRAQ4plex); K7(iTRAQ4	1	1
[R].kPDGYDYYAFSk.[D]	N-Term(iTRAQ4plex); K1(iTRAQ4	1	1
[K].aLENSPk.[E]	N-Term(iTRAQ4plex); K7(iTRAQ4	1	1
[K].eTTVETk.[E]	N-Term(iTRAQ4plex); K7(iTRAQ4	1	1
[K].nWPESVYFFk.[R]	N-Term(iTRAQ4plex); K10(iTRAQ	1	1
[R].gLPNVVTSAISLPNIR.[K]	N-Term(iTRAQ4plex)	1	1
[K].rVcTAELSck.[G]	N-Term(iTRAQ4plex); C3(Carbam	1	1
[K].eTQSIEk.[T]	N-Term(iTRAQ4plex); K7(iTRAQ4	1	1
[K].eTSLTVNk.[E]	N-Term(iTRAQ4plex); K8(iTRAQ4	1	1
[K].dQYYNIDVPSR.[T]	N-Term(iTRAQ4plex)	1	1
[R].gGSIQQYIYk.[Q]	N-Term(iTRAQ4plex); K10(iTRAQ	1	1
[K].nWPESVYFFk.[R]	N-Term(iTRAQ4plex); K10(iTRAQ	1	1
[R].vcTAELSck.[G]	N-Term(iTRAQ4plex); C2(Carbam	1	1
[K].aLENSPk.[E]	N-Term(iTRAQ4plex); K7(iTRAQ4	1	1
[R].kAADDTWEPFASGk.[T]	K1(iTRAQ4plex); K14(iTRAQ4ple	1	1
[R].kAADDTWEPFASGk.[T]	N-Term(iTRAQ4plex); K14(iTRAQ	1	1
[R].kAADDTWEPFASGk.[T]	K1(iTRAQ4plex); K14(iTRAQ4ple	1	1
[R].kAADDTWEPFASGk.[T]	N-Term(iTRAQ4plex); K14(iTRAQ	1	1
[R].kAADDTWEPFASGk.[T]	K1(iTRAQ4plex); K14(iTRAQ4ple	1	1
[R].kAADDTWEPFASGk.[T]	N-Term(iTRAQ4plex); K14(iTRAQ	1	1
[R].GSPAINVAVHVFRk.[A]	K14(iTRAQ4plex)	1	1
[R].gSPAINVAVHVFR.[K]	N-Term(iTRAQ4plex)	1	1
[R].gSPAINVAVHVFR.[K]	N-Term(iTRAQ4plex)	1	1
[R].gSPAINVAVHVFR.[K]	N-Term(iTRAQ4plex)	1	1
[K].aADDTWEPFASGk.[T]	N-Term(iTRAQ4plex); K13(iTRAQ	1	1
[R].gSPAINVAVHVFR.[K]	N-Term(iTRAQ4plex)	1	1
[R].gSPAINVAVHVFRk.[A]	N-Term(iTRAQ4plex); K14(iTRAQ	1	1
[R].gSPAINVAVHVFRk.[A]	N-Term(iTRAQ4plex); K14(iTRAQ	1	1
[R].gSPAINVAVHVFRk.[A]	N-Term(iTRAQ4plex); K14(iTRAQ	1	1
[R].gSPAINVAVHVFRk.[A]	N-Term(iTRAQ4plex); K14(iTRAQ	1	1
[R].gSPAINVAVHVFR.[K]	N-Term(iTRAQ4plex)	1	1
[R].gSPAINVAVHVFR.[K]	N-Term(iTRAQ4plex)	1	1
[R].gSPAINVAVHVFR.[K]	N-Term(iTRAQ4plex)	1	1
[R].GSPAINVAVHVFR.[K]		1	1
[R].gSPAINVAVHVFR.[K]	N-Term(iTRAQ4plex)	1	1
[R].gSPAINVAVHVFR.[K]	N-Term(iTRAQ4plex)	1	1
[R].GSPAINVAVHVFR.[K]		1	1
[R].GSPAINVAVHVFR.[K]		1	1
[R].GSPAINVAVHVFR.[K]		1	1
[R].GSPAINVAVHVFR.[K]		1	1
[R].GSPAINVAVHVFR.[K]		1	1
[R].GSPAINVAVHVFR.[K]		1	1
[R].GSPAINVAVHVFR.[K]		1	1
[R].GSPAINVAVHVFR.[K]		1	1

[K].aLGISPFHEHAEVVFTANDSGPR.[R]	N-Term(iTRAQ4plex)	1	1
[K].aLGISPFHEHAEVVFTANDSGPR.[R]	N-Term(iTRAQ4plex)	1	1
[K].aLGISPFHEHAEVVFTANDSGPR.[R]	N-Term(iTRAQ4plex)	1	1
[K].aLGISPFHEHAEVVFTANDSGPR.[R]	N-Term(iTRAQ4plex)	1	1
[K].aLGISPFHEHAEVVFTANDSGPRR.[Y]	N-Term(iTRAQ4plex)	1	1
[K].aADDTWEPFASGk.[T]	N-Term(iTRAQ4plex); K13(iTRAQ	1	1
[R].yTIAALLSPYSYSTTAVVTNPkE.[-]	N-Term(iTRAQ4plex); K22(iTRAQ	1	1
[K].aADDTWEPFASGk.[T]	N-Term(iTRAQ4plex); K13(iTRAQ	1	1
[K].aADDTWEPFASGk.[T]	N-Term(iTRAQ4plex); K13(iTRAQ	1	1
[R].rYTIAALLSPYSYSTTAVVTNPkE.[-]	N-Term(iTRAQ4plex); K23(iTRAQ	1	1
[R].yTIAALLSPYSYSTTAVVTNPkE.[-]	N-Term(iTRAQ4plex); K22(iTRAQ	1	1
[R].yTIAALLSPYSYSTTAVVTNPk.[E]	N-Term(iTRAQ4plex); K22(iTRAQ	1	1
[R].YTIAALLSPYSYSTTAVVTNPkE.[-]	K22(iTRAQ4plex)	1	1
[R].YTIAALLSPYSYSTTAVVTNPkE.[-]	K22(iTRAQ4plex)	1	1
[K].tSESGELHGLTTEEEFVEGIYk.[V]	N-Term(iTRAQ4plex); K22(iTRAQ	1	1
[R].RYTIAALLSPYSYSTTAVVTNPkE.[-]	K23(iTRAQ4plex)	1	1
[R].rYTIAALLSPYSYSTTAVVTNPkE.[-]	N-Term(iTRAQ4plex); K23(iTRAQ	1	1
[R].rYTIAALLSPYSYSTTAVVTNPkE.[-]	N-Term(iTRAQ4plex); K23(iTRAQ	1	1
[R].rYTIAALLSPYSYSTTAVVTNPk.[E]	N-Term(iTRAQ4plex); K23(iTRAQ	1	1
[R].kAADDTWEPFASGk.[T]	N-Term(iTRAQ4plex); K14(iTRAQ	1	1
[R].kAADDTWEPFASGk.[T]	K1(iTRAQ4plex); K14(iTRAQ4ple	1	1
[R].kAADDTWEPFASGk.[T]	N-Term(iTRAQ4plex); K1(iTRAQ4	1	1
[R].kAADDTWEPFASGk.[T]	N-Term(iTRAQ4plex); K1(iTRAQ4	1	1
[R].kAADDTWEPFASGk.[T]	N-Term(iTRAQ4plex); K1(iTRAQ4	1	1
[R].kAADDTWEPFASGk.[T]	N-Term(iTRAQ4plex); K1(iTRAQ4	1	1
[K].aADDTWEPFASGk.[T]	N-Term(iTRAQ4plex); K13(iTRAQ	1	1
[K].aADDTWEPFASGk.[T]	N-Term(iTRAQ4plex); K13(iTRAQ	1	1
[R].rYTIAALLSPYSYSTTAVVTNPkE.[-]	N-Term(iTRAQ4plex); K23(iTRAQ	1	1
[K].tSESGELHGLTTEEEFVEGIYk.[V]	N-Term(iTRAQ4plex); K22(iTRAQ	1	1
[R].yTIAALLSPYSYSTTAVVTNPkE.[-]	N-Term(iTRAQ4plex); K22(iTRAQ	1	1
[R].GSPAINVAVHVFRk.[A]	K14(iTRAQ4plex)	1	1
[R].RYTIAALLSPYSYSTTAVVTNPkE.[-]	K23(iTRAQ4plex)	1	1
[R].RYTIAALLSPYSYSTTAVVTNPkE.[-]	K23(iTRAQ4plex)	1	1
[R].GSPAINVAVHVFRK.[A]		1	1
[R].gSPAINVAVHVFRk.[A]	N-Term(iTRAQ4plex); K14(iTRAQ	1	1
[K].aLGISPFHEHAEVVFTANDSGPR.[R]	N-Term(iTRAQ4plex)	1	1
[R].gSPAINVAVHVFR.[K]	N-Term(iTRAQ4plex)	1	1
[R].gSPAINVAVHVFR.[K]	N-Term(iTRAQ4plex)	1	1
[R].GSPAINVAVHVFRkAADDTWEPFASGk.[T	K14(iTRAQ4plex); K27(iTRAQ4pl	1	1
[K].AADDTWEPFASGk.[T]	K13(iTRAQ4plex)	1	1
[R].gSPAINVAVHVFR.[K]	N-Term(iTRAQ4plex)	1	1
[R].gSPAINVAVHVFR.[K]	N-Term(iTRAQ4plex)	1	1
[K].aLGISPFHEHAEVVFTANDSGPR.[R]	N-Term(iTRAQ4plex)	1	1
[R].yTIAALLSPYSYSTTAVVTNPkE.[-]	N-Term(iTRAQ4plex); K22(iTRAQ	1	1
[R].rYTIAALLSPYSYSTTAVVTNPk.[E]	N-Term(iTRAQ4plex); K23(iTRAQ	1	1
[K].aLGISPFHEHAEVVFTANDSGPRR.[Y]	N-Term(iTRAQ4plex)	1	1
[K].aLGISPFHEHAEVVFTANDSGPR.[R]	N-Term(iTRAQ4plex)	1	1
[R].kGVcEETSGAYEk.[T]	N-Term(iTRAQ4plex); K1(iTRAQ4	1	1
[R].TVAAcNLPIVR.[G]	C5(Carbamidomethyl)	1	1
[R].TVAAcNLPIVR.[G]	C5(Carbamidomethyl)	1	1
[R].kGVcEETSGAYEk.[T]	N-Term(iTRAQ4plex); K1(iTRAQ4	1	1
[R].TVAAcNLPIVR.[G]	C5(Carbamidomethyl)	1	1

[R].tVAAcNLPIVR.[G]	N-Term(iTRAQ4plex); C5(Carbam	1	1
[R].tVAAcNLPIVR.[G]	N-Term(iTRAQ4plex); C5(Carbam	1	1
[R].tVAAcNLPIVR.[G]	N-Term(iTRAQ4plex); C5(Carbam	1	1
[R].tVAAcNLPIVR.[G]	N-Term(iTRAQ4plex); C5(Carbam	1	1
[R].vVAQGVGIPEDSIFTMADR.[G]	N-Term(iTRAQ4plex)	1	1
[R].mTVSTLVLGEGATEAEISMTSTR.[W]	N-Term(iTRAQ4plex)	1	1
[R].mTVSTLVLGEGATEAEISMTSTR.[W]	N-Term(iTRAQ4plex)	1	1
[R].vVAQGVGIPEDSIFTmADR.[G]	N-Term(iTRAQ4plex); M16(Oxida	1	1
[R].vVAQGVGIPEDSIFTmADR.[G]	N-Term(iTRAQ4plex); M16(Oxida	1	1
[R].eYcGVPGDGDEELLR.[F]	N-Term(iTRAQ4plex); C3(Carbam	1	1
[R].eYcGVPGDGDEELLR.[F]	N-Term(iTRAQ4plex); C3(Carbam	1	1
[R].gEcVPGEQEPEPILIPR.[V]	N-Term(iTRAQ4plex); C3(Carbam	1	1
[R].gEcVPGEQEPEPILIPR.[V]	N-Term(iTRAQ4plex); C3(Carbam	1	1
[R].gEcVPGEQEPEPILIPR.[V]	N-Term(iTRAQ4plex); C3(Carbam	1	1
[R].hHGPTITAk.[L]	N-Term(iTRAQ4plex); K9(iTRAQ4	1	1
[R].hHGPTITAk.[L]	N-Term(iTRAQ4plex); K9(iTRAQ4	1	1
[R].hHGPTITAk.[L]	N-Term(iTRAQ4plex); K9(iTRAQ4	1	1
[R].hHGPTITAk.[L]	N-Term(iTRAQ4plex); K9(iTRAQ4	1	1
[R].eYcGVPGDGDEELLR.[F]	N-Term(iTRAQ4plex); C3(Carbam	1	1
[R].eYcGVPGDGDEELLR.[F]	N-Term(iTRAQ4plex); C3(Carbam	1	1
[R].eYcGVPGDGDEELLR.[F]	N-Term(iTRAQ4plex); C3(Carbam	1	1
[R].eYcGVPGDGDEELLR.[F]	N-Term(iTRAQ4plex); C3(Carbam	1	1
[R].eYcGVPGDGDEELLR.[F]	N-Term(iTRAQ4plex); C3(Carbam	1	1
[K].wYNLAIGSTcPWLk.[K]	N-Term(iTRAQ4plex); C10(Carbar	1	1
[R].eYcGVPGDGDEELLR.[F]	N-Term(iTRAQ4plex); C3(Carbam	1	1
[R].eYcGVPGDGDEELLR.[F]	N-Term(iTRAQ4plex); C3(Carbam	1	1
[R].eYcGVPGDGDEELLR.[F]	N-Term(iTRAQ4plex); C3(Carbam	1	1
[R].eYcGVPGDGDEELLR.[F]	N-Term(iTRAQ4plex); C3(Carbam	1	1
[R].eYcGVPGDGDEELLR.[F]	N-Term(iTRAQ4plex); C3(Carbam	1	1
[R].kGVcEETSGAYEk.[T]	N-Term(iTRAQ4plex); K1(iTRAQ4	1	1
[R].kGVcEETSGAYEk.[T]	N-Term(iTRAQ4plex); K1(iTRAQ4	1	1
[R].kGVcEETSGAYEk.[T]	N-Term(iTRAQ4plex); K1(iTRAQ4	1	1
[R].eYcGVPGDGDEELLR.[F]	N-Term(iTRAQ4plex); C3(Carbam	1	1
[R].eYcGVPGDGDEELLR.[F]	N-Term(iTRAQ4plex); C3(Carbam	1	1
[R].EYcGVPGDGDEELLR.[F]	C3(Carbamidomethyl)	1	1
[K].kEDScQLGYSAGPcMGMTSR.[Y]	N-Term(iTRAQ4plex); K1(iTRAQ4	1	1
[K].kEDScQLGYSAGPcMGMTSR.[Y]	N-Term(iTRAQ4plex); K1(iTRAQ4	1	1
[K].gVcEETSGAYEk.[T]	N-Term(iTRAQ4plex); C3(Carbam	1	1
[R].aFIQLWAFDAVk.[G]	N-Term(iTRAQ4plex); K12(iTRAQ	1	1
[R].aFIQLWAFDAVk.[G]	N-Term(iTRAQ4plex); K12(iTRAQ	1	1
[R].hHGPTITAk.[L]	N-Term(iTRAQ4plex); K9(iTRAQ4	1	1
[R].TVAAcNLPIVR.[G]	C5(Carbamidomethyl)	1	1
[R].hHGPTITAk.[L]	N-Term(iTRAQ4plex); K9(iTRAQ4	1	1
[R].eYcGVPGDGDEELLR.[F]	N-Term(iTRAQ4plex); C3(Carbam	1	1
[R].vVAQGVGIPEDSIFTMADR.[G]	N-Term(iTRAQ4plex)	1	1
[K].wYNLAIGSTcPWLk.[K]	N-Term(iTRAQ4plex); C10(Carbar	1	1
[R].vVAQGVGIPEDSIFTMADR.[G]	N-Term(iTRAQ4plex)	1	1
[R].AFIQLWAFDAVk.[G]	K12(iTRAQ4plex)	1	1
[R].eYcGVPGDGDEELLR.[F]	N-Term(iTRAQ4plex); C3(Carbam	1	1
[K].gVcEETSGAYEk.[T]	N-Term(iTRAQ4plex); C3(Carbam	1	1
[R].kGVcEETSGAYEk.[T]	N-Term(iTRAQ4plex); K1(iTRAQ4	1	1
[R].TVAAcNLPIVR.[G]	C5(Carbamidomethyl)	1	1

[R].eTLLQDFR.[V]	N-Term(iTRAQ4plex)	1	1
[K].kEDScQLGYSAGPcMGMTSR.[Y]	N-Term(iTRAQ4plex); K1(iTRAQ4	1	1
[R].eYcGVPGDGDEELLR.[F]	N-Term(iTRAQ4plex); C3(Carbam	1	1
[R].hHGPTITAk.[L]	N-Term(iTRAQ4plex); K9(iTRAQ4	1	1
[R].tVAAcNLPIVR.[G]	N-Term(iTRAQ4plex); C5(Carbam	1	1
[R].vVAQGVGIPEDSIFTMADR.[G]	N-Term(iTRAQ4plex)	1	1
[R].GEcVPGEQEPEPILIPR.[V]	C3(Carbamidomethyl)	1	1
[K].wYNLAIGSTcPWLk.[K]	N-Term(iTRAQ4plex); C10(Carbar	1	1
[R].gEcVPGEQEPEPILIPR.[V]	N-Term(iTRAQ4plex); C3(Carbam	1	1
[R].mTVSTLVLGEGATEAEISMTSTR.[W]	N-Term(iTRAQ4plex)	1	1
[R].tVAAcNLPIVR.[G]	N-Term(iTRAQ4plex); C5(Carbam	1	1
[R].gEcVPGEQEPEPILIPR.[V]	N-Term(iTRAQ4plex); C3(Carbam	1	1
[R].GEcVPGEQEPEPILIPR.[V]	C3(Carbamidomethyl)	1	1
[K].aMAVEDIISR.[V]	N-Term(iTRAQ4plex)	1	1
[K].iNVGGGLSGDHck.[K]	N-Term(iTRAQ4plex); C12(Carbar	1	1
[K].mESLGITSR.[D]	N-Term(iTRAQ4plex)	1	1
[K].amAVEDIISR.[V]	N-Term(iTRAQ4plex); M2(Oxidat	1	1
[K].iNVGGGLSGDHck.[K]	N-Term(iTRAQ4plex); C12(Carbar	1	1
[R].kPYNFLk.[Y]	N-Term(iTRAQ4plex); K1(iTRAQ4	1	1
[K].fGGTIcSGDIWDQAScSSSTTcVR.[Q]	N-Term(iTRAQ4plex); C6(Carbam	1	1
[K].yNPVVIDFEMQPIHEVLR.[H]	N-Term(iTRAQ4plex)	1	1
[K].yNPVVIDFEMQPIHEVLR.[H]	N-Term(iTRAQ4plex)	1	1
[R].aIDEDcSQYEPIPGSQk.[A]	N-Term(iTRAQ4plex); C6(Carbam	1	1
[R].aIDEDcSQYEPIPGSQk.[A]	N-Term(iTRAQ4plex); C6(Carbam	1	1
[R].qAQcGQDFQck.[E]	N-Term(iTRAQ4plex); C4(Carbam	1	1
[R].aIDEDcSQYEPIPGSQk.[A]	N-Term(iTRAQ4plex); C6(Carbam	1	1
[R].lYYGDDEk.[Y]	N-Term(iTRAQ4plex); K8(iTRAQ4	1	1
[R].lGSLGAAcEQTQTEGAk.[A]	N-Term(iTRAQ4plex); C8(Carbam	1	1
[R].hTSLGPLEAk.[R]	N-Term(iTRAQ4plex); K10(iTRAQ4	1	1
[K].INVGGGLSGDHck.[K]	C12(Carbamidomethyl); K13(iTR	1	1
[K].fGGTIcSGDIWDQAScSSSTTcVR.[Q]	N-Term(iTRAQ4plex); C6(Carbam	1	1
[K].aMAVEDIISR.[V]	N-Term(iTRAQ4plex)	1	1
[K].mESLGITSR.[D]	N-Term(iTRAQ4plex)	1	1
[R].kPYNFLk.[Y]	N-Term(iTRAQ4plex); K1(iTRAQ4	1	1
[K].vQTAHFk.[M]	N-Term(iTRAQ4plex); K7(iTRAQ4	1	1
[R].hTSLGPLEAk.[R]	N-Term(iTRAQ4plex); K10(iTRAQ4	1	1
[R].aIDEDcSQYEPIPGSQk.[A]	N-Term(iTRAQ4plex); C6(Carbam	1	1
[R].AIDEDcSQYEPIPGSQk.[A]	C6(Carbamidomethyl); K17(iTRA	1	1
[R].lGSLGAAcEQTQTEGAk.[A]	N-Term(iTRAQ4plex); C8(Carbam	1	1
[K].aQYVLISPEASSR.[F]	N-Term(iTRAQ4plex)	1	1
[K].vAFLTVTLHQGGATR.[M]	N-Term(iTRAQ4plex)	1	1
[R].tMFIGGSQLSQk.[H]	N-Term(iTRAQ4plex); K12(iTRAQ4	1	1
[K].gIVAAFYSGPSLSDk.[E]	N-Term(iTRAQ4plex); K15(iTRAQ4	1	1
[K].gAVYVYFGSk.[Q]	N-Term(iTRAQ4plex); K10(iTRAQ4	1	1
[R].eLLLEHQDAYQAGIVFPDcFYPSIck.[G]	N-Term(iTRAQ4plex); C19(Carbar	1	1
[K].eTTLGDMTGk.[C]	N-Term(iTRAQ4plex); K10(iTRAQ4	1	1
[R].iLEGFQPSGR.[F]	N-Term(iTRAQ4plex)	1	1
[K].sWITPcPEEk.[A]	N-Term(iTRAQ4plex); C6(Carbam	1	1
[R].iLEGFQPSGR.[F]	N-Term(iTRAQ4plex)	1	1
[K].gAVYVYFGSk.[Q]	N-Term(iTRAQ4plex); K10(iTRAQ4	1	1
[K].qVLLVGAPTYDDVSk.[V]	N-Term(iTRAQ4plex); K15(iTRAQ4	1	1
[R].eNYPLPWEk.[D]	N-Term(iTRAQ4plex); K9(iTRAQ4	1	1

[R].iADVTSGLIGGEDGR.[V]	N-Term(iTRAQ4plex)	1	1
[K].sWITPcPEEk.[A]	N-Term(iTRAQ4plex); C6(Carbam	1	1
[K].qVLLVGAPTYDDVSk.[V]	N-Term(iTRAQ4plex); K15(iTRAQ	1	1
[R].gWHWVGAHTLGHNSR.[G]	N-Term(iTRAQ4plex)	1	1
[R].gWHWVGAHTLGHNSR.[G]	N-Term(iTRAQ4plex)	1	1
[R].hTASAWLMSAPNSGPHNR.[L]	N-Term(iTRAQ4plex)	1	1
[R].hTASAWLMSAPNSGPHNR.[L]	N-Term(iTRAQ4plex)	1	1
[R].aGLLRPDYALLGHR.[Q]	N-Term(iTRAQ4plex)	1	1
[R].aGLLRPDYALLGHR.[Q]	N-Term(iTRAQ4plex)	1	1
[R].aGLLRPDYALLGHR.[Q]	N-Term(iTRAQ4plex)	1	1
[R].aGLLRPDYALLGHR.[Q]	N-Term(iTRAQ4plex)	1	1
[R].TDcPGDALFDLLR.[T]	C3(Carbamidomethyl)	1	1
[R].tDcPGDALFDLLR.[T]	N-Term(iTRAQ4plex); C3(Carbam	1	1
[K].eFTEAFLGcPAIHPR.[C]	N-Term(iTRAQ4plex); C9(Carbam	1	1
[K].eFTEAFLGcPAIHPR.[C]	N-Term(iTRAQ4plex); C9(Carbam	1	1
[K].gcPDVQASLPDAk.[A]	N-Term(iTRAQ4plex); C2(Carbam	1	1
[K].gcPDVQASLPDAk.[A]	N-Term(iTRAQ4plex); C2(Carbam	1	1
[K].gcPDVQASLPDAk.[A]	N-Term(iTRAQ4plex); C2(Carbam	1	1
[R].dTLPScAVR.[A]	N-Term(iTRAQ4plex); C6(Carbam	1	1
[K].lLQLPLGFLYVHHTYVPAPPcTDFTR.[C]	N-Term(iTRAQ4plex); C21(Carba	1	1
[K].lLQLPLGFLYVHHTYVPAPPcTDFTR.[C]	N-Term(iTRAQ4plex); C21(Carba	1	1
[R].vINLPLDSMAAPWETGDTFPDVVAIAPDV	N-Term(iTRAQ4plex)	1	1
[R].vINLPLDSMAAPWETGDTFPDVVAIAPDV	N-Term(iTRAQ4plex)	1	1
[K].eYGVVLAPDGSTVAVEPLLAGLEAGLQGR.	N-Term(iTRAQ4plex)	1	1
[R].gSQTQSHPDLGTEGcWDQLSAPR.[T]	N-Term(iTRAQ4plex); C15(Carba	1	1
[R].gSQTQSHPDLGTEGcWDQLSAPR.[T]	N-Term(iTRAQ4plex); C15(Carba	1	1
[R].GSQTQSHPDLGTEGcWDQLSAPR.[T]	C15(Carbamidomethyl)	1	1
[K].gcPDVQASLPDAk.[A]	N-Term(iTRAQ4plex); C2(Carbam	1	1
[K].eFTEAFLGcPAIHPR.[C]	N-Term(iTRAQ4plex); C9(Carbam	1	1
[R].AGLLRPDYALLGHR.[Q]		1	1
[R].tFTLLDPk.[A]	N-Term(iTRAQ4plex); K8(iTRAQ4	1	1
[R].dGSPDVTTADIGANTPDATk.[G]	N-Term(iTRAQ4plex); K20(iTRAQ	1	1
[R].GSQTQSHPDLGTEGcWDQLSAPR.[T]	C15(Carbamidomethyl)	1	1
[R].TFTLLDPk.[A]	K8(iTRAQ4plex)	1	1
[R].dGSPDVTTADIGANTPDATk.[G]	N-Term(iTRAQ4plex); K20(iTRAQ	1	1
[R].hTASAWLMSAPNSGPHNR.[L]	N-Term(iTRAQ4plex)	1	1
[K].eFTEAFLGcPAIHPR.[C]	N-Term(iTRAQ4plex); C9(Carbam	1	1
[K].eFTEAFLGcPAIHPR.[C]	N-Term(iTRAQ4plex); C9(Carbam	1	1
[R].dGSPDVTTADIGANTPDATk.[G]	N-Term(iTRAQ4plex); K20(iTRAQ	1	1
[R].tFTLLDPk.[A]	N-Term(iTRAQ4plex); K8(iTRAQ4	1	1
[R].AGLLRPDYALLGHR.[Q]		1	1
[R].gWHWVGAHTLGHNSR.[G]	N-Term(iTRAQ4plex)	1	1
[K].dLLLPQPDLR.[Y]	N-Term(iTRAQ4plex)	1	1
[K].dLLLPQPDLR.[Y]	N-Term(iTRAQ4plex)	1	1
[R].lHLEGNkLQVLGk.[D]	N-Term(iTRAQ4plex); K7(iTRAQ4	1	1
[K].aLGHLDLSGNRLR.[K]	N-Term(iTRAQ4plex)	1	1
[K].gQTLLAVAk.[S]	N-Term(iTRAQ4plex); K9(iTRAQ4	1	1
[K].aLGHLDLSGNR.[L]	N-Term(iTRAQ4plex)	1	1
[K].aLGHLDLSGNR.[L]	N-Term(iTRAQ4plex)	1	1
[K].aLGHLDLSGNR.[L]	N-Term(iTRAQ4plex)	1	1
[R].VAAGAFQGLR.[Q]		1	1
[R].vAAGAFQGLR.[Q]	N-Term(iTRAQ4plex)	1	1

[R].vAAGAFQGLR.[Q]	N-Term(iTRAQ4plex)	1	1
[R].dGFDISGNPWIcDQNLSDLYR.[W]	N-Term(iTRAQ4plex); C12(Carbar	1	1
[R].dGFDISGNPWIcDQNLSDLYR.[W]	N-Term(iTRAQ4plex); C12(Carbar	1	1
[R].tLDLGENQLETLPPDLLR.[G]	N-Term(iTRAQ4plex)	1	1
[R].tLDLGENQLETLPPDLLR.[G]	N-Term(iTRAQ4plex)	1	1
[K].eNQLEVLEVSWLHGLk.[A]	N-Term(iTRAQ4plex); K16(iTRAQ	1	1
[K].eNQLEVLEVSWLHGLk.[A]	N-Term(iTRAQ4plex); K16(iTRAQ	1	1
[K].eNQLEVLEVSWLHGLk.[A]	N-Term(iTRAQ4plex); K16(iTRAQ	1	1
[K].ENQLEVLEVSWLHGLk.[A]	K16(iTRAQ4plex)	1	1
[R].cAGPEAVk.[G]	N-Term(iTRAQ4plex); C1(Carbam	1	1
[R].nALTGLPPGLFQASATLDTLVLk.[E]	N-Term(iTRAQ4plex); K23(iTRAQ	1	1
[K].dLLLPQPDLR.[Y]	N-Term(iTRAQ4plex)	1	1
[K].dLLLPQPDLR.[Y]	N-Term(iTRAQ4plex)	1	1
[K].aLGHLDLSGNR.[L]	N-Term(iTRAQ4plex)	1	1
[K].gQTLLAVAk.[S]	N-Term(iTRAQ4plex); K9(iTRAQ4	1	1
[K].dLLLPQPDLR.[Y]	N-Term(iTRAQ4plex)	1	1
[K].aLGHLDLSGNR.[L]	N-Term(iTRAQ4plex)	1	1
[R].yLFLNGNkLAR.[V]	N-Term(iTRAQ4plex); K8(iTRAQ4	1	1
[R].YLFLNGNkLAR.[V]	K8(iTRAQ4plex)	1	1
[K].ALGHLDLSGNR.[L]		1	1
[R].yLFLNGNk.[L]	N-Term(iTRAQ4plex); K8(iTRAQ4	1	1
[R].yLFLNGNk.[L]	N-Term(iTRAQ4plex); K8(iTRAQ4	1	1
[R].YLFLNGNkLAR.[V]	K8(iTRAQ4plex)	1	1
[R].rPHFFFPk.[S]	N-Term(iTRAQ4plex); K8(iTRAQ4	1	1
[R].rPHFFFPk.[S]	N-Term(iTRAQ4plex); K8(iTRAQ4	1	1
[R].rPHFFFPk.[S]	N-Term(iTRAQ4plex); K8(iTRAQ4	1	1
[K].tLLSNLEEAk.[K]	N-Term(iTRAQ4plex); K10(iTRAQ	1	1
[R].kTLLSNLEEAk.[K]	N-Term(iTRAQ4plex); K1(iTRAQ4	1	1
[K].lFDSDPITVTVPVEVSR.[K]	N-Term(iTRAQ4plex)	1	1
[K].tLLSNLEEAk.[K]	N-Term(iTRAQ4plex); K10(iTRAQ	1	1
[R].qQTHMLDVMQDHFSR.[A]	N-Term(iTRAQ4plex)	1	1
[R].qQTHMLDVMQDHFSR.[A]	N-Term(iTRAQ4plex)	1	1
[R].qQTHMLDVMQDHFSR.[A]	N-Term(iTRAQ4plex)	1	1
[R].aSSIIDELFQDR.[F]	N-Term(iTRAQ4plex)	1	1
[R].aSSIIDELFQDR.[F]	N-Term(iTRAQ4plex)	1	1
[R].eLDESLQVAER.[L]	N-Term(iTRAQ4plex)	1	1
[R].eLDESLQVAER.[L]	N-Term(iTRAQ4plex)	1	1
[R].eLDESLQVAER.[L]	N-Term(iTRAQ4plex)	1	1
[K].TLLSNLEEAk.[K]	K10(iTRAQ4plex)	1	1
[R].vTTVASHTSDSDVPSGVTEVVVk.[L]	N-Term(iTRAQ4plex); K23(iTRAQ	1	1
[R].iDSLLENDR.[Q]	N-Term(iTRAQ4plex)	1	1
[K].tLLSNLEEAk.[K]	N-Term(iTRAQ4plex); K10(iTRAQ	1	1
[K].fMETVAEk.[A]	N-Term(iTRAQ4plex); K8(iTRAQ4	1	1
[K].tLLSNLEEAk.[K]	N-Term(iTRAQ4plex); K10(iTRAQ	1	1
[R].eILSVDcSTNNPSQAk.[L]	N-Term(iTRAQ4plex); C7(Carbam	1	1
[R].rPHFFFPk.[S]	N-Term(iTRAQ4plex); K8(iTRAQ4	1	1
[R].eILSVDcSTNNPSQAk.[L]	N-Term(iTRAQ4plex); C7(Carbam	1	1
[R].RPHFFFPk.[S]	K8(iTRAQ4plex)	1	1
[R].kTLLSNLEEAk.[K]	N-Term(iTRAQ4plex); K1(iTRAQ4	1	1
[R].ASSIIDELFQDR.[F]		1	1
[R].rPHFFFPk.[S]	N-Term(iTRAQ4plex); K8(iTRAQ4	1	1
[R].rELDESLQVAER.[L]	N-Term(iTRAQ4plex)	1	1

[R].eILSVDcSTNNPSQAk.[L]	N-Term(iTRAQ4plex); C7(Carbam	1	1
[R].IDSLLENDR.[Q]		1	1
[R].kYNELLk.[S]	N-Term(iTRAQ4plex); K1(iTRAQ4	1	1
[R].ASSIIDELFQDR.[F]		1	1
[R].aSSIIDELFQDR.[F]	N-Term(iTRAQ4plex)	1	1
[R].aSSIIDELFQDR.[F]	N-Term(iTRAQ4plex)	1	1
[R].vTTVASHTSDSDVPSGVTEVVVk.[L]	N-Term(iTRAQ4plex); K23(iTRAQ	1	1
[R].rLFQPLTHLk.[T]	N-Term(iTRAQ4plex); K10(iTRAQ	1	1
[R].dHLGFQVTWPDESk.[A]	N-Term(iTRAQ4plex); K14(iTRAQ	1	1
[R].dHLGFQVTWPDESk.[A]	N-Term(iTRAQ4plex); K14(iTRAQ	1	1
[R].LFQPLTHLk.[T]	K9(iTRAQ4plex)	1	1
[R].lFQPLTHLk.[T]	N-Term(iTRAQ4plex); K9(iTRAQ4	1	1
[R].lFQPLTHLk.[T]	N-Term(iTRAQ4plex); K9(iTRAQ4	1	1
[R].sQcTYSNPEGTVVLAcDQAQcR.[W]	N-Term(iTRAQ4plex); C3(Carbam	1	1
[R].sLMLSYNAITHLPAGIFR.[D]	N-Term(iTRAQ4plex)	1	1
[K].lSNNALSGLPQGVFGk.[L]	N-Term(iTRAQ4plex); K16(iTRAQ	1	1
[K].vVFLNTQLcQFRPDAFGGLPR.[L]	N-Term(iTRAQ4plex); C9(Carbam	1	1
[K].tLNLAQNLLAQLPEELFHPLTSLQTLk.[L]	N-Term(iTRAQ4plex); K27(iTRAQ	1	1
[K].aGGSWDLAVQER.[A]	N-Term(iTRAQ4plex)	1	1
[K].lELLSLSk.[N]	N-Term(iTRAQ4plex); K8(iTRAQ4	1	1
[K].gQVVPALNEk.[Q]	N-Term(iTRAQ4plex); K10(iTRAQ	1	1
[R].dLEELVk.[L]	N-Term(iTRAQ4plex); K7(iTRAQ4	1	1
[R].lFQPLTHLk.[T]	N-Term(iTRAQ4plex); K9(iTRAQ4	1	1
[K].aGGSWDLAVQER.[A]	N-Term(iTRAQ4plex)	1	1
[R].lTVSIEAR.[A]	N-Term(iTRAQ4plex)	1	1
[R].DLEELVk.[L]	K7(iTRAQ4plex)	1	1
[K].gQVVPALNEk.[Q]	N-Term(iTRAQ4plex); K10(iTRAQ	1	1
[R].lFQPLTHLk.[T]	N-Term(iTRAQ4plex); K9(iTRAQ4	1	1
[R].wDLQHPHQHPFEPGk.[F]	N-Term(iTRAQ4plex); K15(iTRAQ	1	2
[R].wDLQHPHQHPFEPGk.[F]	N-Term(iTRAQ4plex); K15(iTRAQ	1	2
[R].wDLQHPHQHPFEPGk.[F]	N-Term(iTRAQ4plex); K15(iTRAQ	1	2
[R].vVGGHPGNSPWTVSLR.[N]	N-Term(iTRAQ4plex)	1	1
[R].wDAQIPHQHR.[F]	N-Term(iTRAQ4plex)	1	1
[R].gTMATTVGGLPcQAWSHk.[F]	N-Term(iTRAQ4plex); C12(Carbar	1	1
[R].gTELQHLLHAVVPGPWQEDVADAEEcAGI	N-Term(iTRAQ4plex); C26(Carbar	1	2
[R].nPDGDPGGPWcYTTDPAVR.[F]	N-Term(iTRAQ4plex); C11(Carbar	1	1
[R].nPDGDPGGPWcYTTDPAVR.[F]	N-Term(iTRAQ4plex); C11(Carbar	1	1
[K].mVcGPSGSQLVLLk.[L]	N-Term(iTRAQ4plex); C3(Carbam	1	1
[K].eQWILTAR.[Q]	N-Term(iTRAQ4plex)	1	2
[K].eQWILTAR.[Q]	N-Term(iTRAQ4plex)	1	2
[R].vSVFVDWIHk.[V]	N-Term(iTRAQ4plex); K10(iTRAQ	1	2
[R].cADDQPPSILDPPDQVQFEk.[C]	N-Term(iTRAQ4plex); C1(Carbam	1	1
[R].qEATTVScFR.[G]	N-Term(iTRAQ4plex); C8(Carbam	1	1
[R].nPDGSERPWcYTTDPQIER.[E]	N-Term(iTRAQ4plex); C10(Carbar	1	2
[R].aAFcYQIR.[R]	N-Term(iTRAQ4plex); C4(Carbam	1	1
[K].cLAYDFYPGk.[I]	N-Term(iTRAQ4plex); C1(Carbam	1	1
[R].eDIFMETLk.[D]	N-Term(iTRAQ4plex); K9(iTRAQ4	1	1
[K].EIPAWVPFDPAAQITk.[Q]	K16(iTRAQ4plex)	1	1
[K].eIPAWVPFDPAAQITk.[Q]	N-Term(iTRAQ4plex); K16(iTRAQ	1	1
[K].eIPAWVPFDPAAQITk.[Q]	N-Term(iTRAQ4plex); K16(iTRAQ	1	1
[R].qVEGMEDWk.[Q]	N-Term(iTRAQ4plex); K9(iTRAQ4	1	1
[K].aYLEEEcPATLR.[K]	N-Term(iTRAQ4plex); C7(Carbam	1	1

[K].aYLEEEcPATLR.[K]	N-Term(iTRAQ4plex); C7(Carbam	1	1
[K].hVEDVPAFQALGSLNDLQFFR.[Y]	N-Term(iTRAQ4plex)	1	1
[R].ySLTYIYTGLSk.[H]	N-Term(iTRAQ4plex); K12(iTRAQ	1	1
[R].qDPPSVVVTSHQAPGEk.[K]	N-Term(iTRAQ4plex); K17(iTRAQ	1	1
[R].qDPPSVVVTSHQAPGEk.[K]	N-Term(iTRAQ4plex); K17(iTRAQ	1	1
[K].aREDIFMETLk.[D]	N-Term(iTRAQ4plex); K11(iTRAQ	1	1
[K].hVEDVPAFQALGSLNDLQFFR.[Y]	N-Term(iTRAQ4plex)	1	1
[K].aREDIFMETLk.[D]	N-Term(iTRAQ4plex); K11(iTRAQ	1	1
[K].qkWEAEPVYVQR.[A]	N-Term(iTRAQ4plex); K2(iTRAQ4	1	1
[R].aGEVQEPELR.[G]	N-Term(iTRAQ4plex)	1	1
[R].aGEVQEPELR.[G]	N-Term(iTRAQ4plex)	1	1
[K].WEAEPVYVQR.[A]		1	1
[K].AYLEEEcPATLR.[K]	C7(Carbamidomethyl)	1	1
[R].AGEVQEPELR.[G]		1	1
[R].aGEVQEPELR.[G]	N-Term(iTRAQ4plex)	1	1
[K].dYIEFNk.[E]	N-Term(iTRAQ4plex); K7(iTRAQ4	1	1
[K].wEAEPVYVQR.[A]	N-Term(iTRAQ4plex)	1	1
[R].qVEGMEDWk.[Q]	N-Term(iTRAQ4plex); K9(iTRAQ4	1	1
[K].qkWEAEPVYVQR.[A]	N-Term(iTRAQ4plex); K2(iTRAQ4	1	1
[K].qkWEAEPVYVQR.[A]	N-Term(iTRAQ4plex); K2(iTRAQ4	1	1
[R].qVEGmEDWk.[Q]	N-Term(iTRAQ4plex); M5(Oxidat	1	1
[R].EDIFMETLk.[D]	K9(iTRAQ4plex)	1	1
[R].sSGAFWk.[Y]	N-Term(iTRAQ4plex); K7(iTRAQ4	1	1
[R].aGEVQEPELR.[G]	N-Term(iTRAQ4plex)	1	1
[R].sSGAFWk.[Y]	N-Term(iTRAQ4plex); K7(iTRAQ4	1	1
[K].cLAYDFYPGk.[I]	N-Term(iTRAQ4plex); C1(Carbam	1	1
[K].dYIEFNk.[E]	N-Term(iTRAQ4plex); K7(iTRAQ4	1	1
[R].AGEVQEPELR.[G]		1	1
[R].eDIFmETLk.[D]	N-Term(iTRAQ4plex); M5(Oxidat	1	1
[R].qVEGmEDWk.[Q]	N-Term(iTRAQ4plex); M5(Oxidat	1	1
[K].qkWEAEPVYVQR.[A]	N-Term(iTRAQ4plex); K2(iTRAQ4	1	1
[K].AREDIFMETLk.[D]	K11(iTRAQ4plex)	1	1
[R].sSGAFWk.[Y]	N-Term(iTRAQ4plex); K7(iTRAQ4	1	1
[K].eIPAWVPFDPAAQITk.[Q]	N-Term(iTRAQ4plex); K16(iTRAQ	1	1
[R].eDIFMETLk.[D]	N-Term(iTRAQ4plex); K9(iTRAQ4	1	1
[K].wEAEPVYVQR.[A]	N-Term(iTRAQ4plex)	1	1
[R].qVEGMEDWk.[Q]	N-Term(iTRAQ4plex); K9(iTRAQ4	1	1
[R].aGEVQEPELR.[G]	N-Term(iTRAQ4plex)	1	1
[K].iSNIPDEYFk.[R]	N-Term(iTRAQ4plex); K10(iTRAQ	1	1
[K].iSNIPDEYFk.[R]	N-Term(iTRAQ4plex); K10(iTRAQ	1	1
[R].iSETSLPPDMYEcLR.[V]	N-Term(iTRAQ4plex); C13(Carba	1	1
[R].iSETSLPPDMYEcLR.[V]	N-Term(iTRAQ4plex); C13(Carba	1	1
[R].iSETSLPPDMYEcLR.[V]	N-Term(iTRAQ4plex); C13(Carba	1	1
[R].iSETSLPPDMYEcLR.[V]	N-Term(iTRAQ4plex); C13(Carba	1	1
[R].iSETSLPPDMYEcLR.[V]	N-Term(iTRAQ4plex); C13(Carba	1	1
[R].iSETSLPPDMYEcLR.[V]	N-Term(iTRAQ4plex); C13(Carba	1	1
[R].iSETSLPPDMYEcLR.[V]	N-Term(iTRAQ4plex); C13(Carba	1	1
[K].nIPTVNENLENYYLEVNQLEk.[F]	N-Term(iTRAQ4plex); K21(iTRAQ	1	1
[K].nIPTVNENLENYYLEVNQLEk.[F]	N-Term(iTRAQ4plex); K21(iTRAQ	1	1
[K].iLGPLSYSk.[I]	N-Term(iTRAQ4plex); K9(iTRAQ4	1	1
[K].sLEDLQLTHNk.[I]	N-Term(iTRAQ4plex); K11(iTRAQ	1	1
[R].lkEDAVSAAFk.[G]	N-Term(iTRAQ4plex); K2(iTRAQ4	1	1

[R].lkEDAVSAAFk.[G]	N-Term(iTRAQ4plex); K2(iTRAQ4	1	1
[R].lkEDAVSAAFk.[G]	N-Term(iTRAQ4plex); K2(iTRAQ4	1	1
[R].lkEDAVSAAFk.[G]	N-Term(iTRAQ4plex); K2(iTRAQ4	1	1
[R].lkEDAVSAAFk.[G]	N-Term(iTRAQ4plex); K2(iTRAQ4	1	1
[R].nNQIDHIDEk.[A]	N-Term(iTRAQ4plex); K10(iTRAQ	1	1
[K].sVPMVPPGIk.[Y]	N-Term(iTRAQ4plex); K10(iTRAQ	1	1
[R].FNALQYLR.[L]		1	1
[K].sVPMVPPGIk.[Y]	N-Term(iTRAQ4plex); K10(iTRAQ	1	1
[K].sVPMVPPGIk.[Y]	N-Term(iTRAQ4plex); K10(iTRAQ	1	1
[K].sVPMVPPGIk.[Y]	N-Term(iTRAQ4plex); K10(iTRAQ	1	1
[K].nIPTVNENLENYYLEVNQLEk.[F]	N-Term(iTRAQ4plex); K21(iTRAQ	1	1
[K].sLEYLDLSFNQIAR.[L]	N-Term(iTRAQ4plex)	1	1
[K].sLEYLDLSFNQIAR.[L]	N-Term(iTRAQ4plex)	1	1
[R].nNQIDHIDEk.[A]	N-Term(iTRAQ4plex); K10(iTRAQ	1	1
[R].nNQIDHIDEk.[A]	N-Term(iTRAQ4plex); K10(iTRAQ	1	1
[R].nNQIDHIDEk.[A]	N-Term(iTRAQ4plex); K10(iTRAQ	1	1
[R].nNQIDHIDEk.[A]	N-Term(iTRAQ4plex); K10(iTRAQ	1	1
[R].nNQIDHIDEk.[A]	N-Term(iTRAQ4plex); K10(iTRAQ	1	1
[R].nNQIDHIDEk.[A]	N-Term(iTRAQ4plex); K10(iTRAQ	1	1
[R].lPSGLPVSLLTLYLDNNk.[I]	N-Term(iTRAQ4plex); K18(iTRAQ	1	1
[R].nNQIDHIDEk.[A]	N-Term(iTRAQ4plex); K10(iTRAQ	1	1
[R].nNQIDHIDEk.[A]	N-Term(iTRAQ4plex); K10(iTRAQ	1	1
[R].lPSGLPVSLLTLYLDNNk.[I]	N-Term(iTRAQ4plex); K18(iTRAQ	1	1
[K].sLEDLQLTHNk.[I]	N-Term(iTRAQ4plex); K11(iTRAQ	1	1
[K].sLEDLQLTHNk.[I]	N-Term(iTRAQ4plex); K11(iTRAQ	1	1
[K].eDAVSAAFk.[G]	N-Term(iTRAQ4plex); K9(iTRAQ4	1	1
[R].iSETSLPPDMYEcLR.[V]	N-Term(iTRAQ4plex); C13(Carbai	1	1
[K].sLEYLDLSFNQIAR.[L]	N-Term(iTRAQ4plex)	1	1
[K].sLEYLDLSFNQIAR.[L]	N-Term(iTRAQ4plex)	1	1
[R].lkEDAVSAAFk.[G]	N-Term(iTRAQ4plex); K2(iTRAQ4	1	1
[R].fNALQYLR.[L]	N-Term(iTRAQ4plex)	1	1
[R].nNQIDHIDEk.[A]	N-Term(iTRAQ4plex); K10(iTRAQ	1	1
[K].sLEDLQLTHNk.[I]	N-Term(iTRAQ4plex); K11(iTRAQ	1	1
[K].ILGPLSYSk.[I]	K9(iTRAQ4plex)	1	1
[R].LkEDAVSAAFk.[G]	K2(iTRAQ4plex); K11(iTRAQ4plex	1	1
[K].iLGPLSYSk.[I]	N-Term(iTRAQ4plex); K9(iTRAQ4	1	1
[R].lkEDAVSAAFk.[G]	N-Term(iTRAQ4plex); K2(iTRAQ4	1	1
[R].lkEDAVSAAFk.[G]	N-Term(iTRAQ4plex); K2(iTRAQ4	1	1
[K].iLGPLSYSk.[I]	N-Term(iTRAQ4plex); K9(iTRAQ4	1	1
[R].nNQIDHIDEk.[A]	N-Term(iTRAQ4plex); K10(iTRAQ	1	1
[K].iLGPLSYSk.[I]	N-Term(iTRAQ4plex); K9(iTRAQ4	1	1
[R].nNQIDHIDEk.[A]	N-Term(iTRAQ4plex); K10(iTRAQ	1	1
[K].rFNALQYLR.[L]	N-Term(iTRAQ4plex)	1	1
[K].SLEYLDLSFNQIAR.[L]		1	1
[R].fNALQYLR.[L]	N-Term(iTRAQ4plex)	1	1
[R].NNQIDHIDEk.[A]	K10(iTRAQ4plex)	1	1
[K].sVPMVPPGIk.[Y]	N-Term(iTRAQ4plex); K10(iTRAQ	1	1
[K].SVPMVPPGIk.[Y]	K10(iTRAQ4plex)	1	1
[K].SLEDLQLTHNk.[I]	K11(iTRAQ4plex)	1	1
[R].vANEVTLN.[-]	N-Term(iTRAQ4plex)	1	1
[K].nIPTVNENLENYYLEVNQLEk.[F]	N-Term(iTRAQ4plex); K21(iTRAQ	1	1
[K].sLEYLDLSFNQIAR.[L]	N-Term(iTRAQ4plex)	1	1

[K].fEIENcLANk.[V]	N-Term(iTRAQ4plex); C6(Carbam	1	1
[R].SSGSLLNNAIk.[G]	K11(iTRAQ4plex)	2	2
[K].mVSGFIPLkPTVk.[M]	N-Term(iTRAQ4plex); M1(Oxidat	2	2
[K].mVSGFIPLkPTVk.[M]	N-Term(iTRAQ4plex); M1(Oxidat	2	2
[K].aVGYLITGYQR.[Q]	N-Term(iTRAQ4plex)	1	1
[K].aTVLNYLPk.[C]	N-Term(iTRAQ4plex); K9(iTRAQ4	2	2
[R].sLFTDLVAEk.[D]	N-Term(iTRAQ4plex); K10(iTRAQ	1	1
[K].aGAFcLSEDAGLGISSTASLR.[A]	N-Term(iTRAQ4plex); C5(Carbam	2	2
[K].aGAFcLSEDAGLGISSTASLR.[A]	N-Term(iTRAQ4plex); C5(Carbam	2	2
[K].aGAFcLSEDAGLGISSTASLR.[A]	N-Term(iTRAQ4plex); C5(Carbam	2	2
[K].mVSGFIPLkPTVk.[M]	N-Term(iTRAQ4plex); K9(iTRAQ4	2	2
[K].aGAFcLSEDAGLGISSTASLR.[A]	N-Term(iTRAQ4plex); C5(Carbam	2	2
[K].mVSGFIPLkPTVk.[M]	N-Term(iTRAQ4plex); K9(iTRAQ4	2	2
[K].eDSPFALk.[V]	N-Term(iTRAQ4plex); K8(iTRAQ4	1	1
[R].aFQPFFVELTMPYSVIR.[G]	N-Term(iTRAQ4plex)	2	2
[R].aFQPFFVELTMPYSVIR.[G]	N-Term(iTRAQ4plex)	2	2
[K].aGAFcLSEDAGLGISSTASLR.[A]	N-Term(iTRAQ4plex); C5(Carbam	2	2
[K].lEAGINQLSFPLSSEPIQGSYR.[V]	N-Term(iTRAQ4plex)	1	1
[R].sSGSLLNNAIk.[G]	N-Term(iTRAQ4plex); K11(iTRAQ	2	2
[K].hQDGSYSTFGER.[Y]	N-Term(iTRAQ4plex)	1	1
[K].aTVLNYLPk.[C]	N-Term(iTRAQ4plex); K9(iTRAQ4	2	2
[R].sSGSLLNNAIk.[G]	N-Term(iTRAQ4plex); K11(iTRAQ	2	2
[R].iSEITNIVSk.[L]	N-Term(iTRAQ4plex); K10(iTRAQ	1	1
[K].mVSGFIPLkPTVk.[M]	N-Term(iTRAQ4plex); K9(iTRAQ4	2	2
[R].vVSVDENFRPR.[N]	N-Term(iTRAQ4plex)	1	1
[R].sSGSLLNNAIk.[G]	N-Term(iTRAQ4plex); K11(iTRAQ	2	2
[R].eILNSLDk.[E]	N-Term(iTRAQ4plex); K8(iTRAQ4	1	1
[K].eGTHGSHVYTk.[A]	N-Term(iTRAQ4plex); K11(iTRAQ	1	1
[R].sSGSLLNNAIk.[G]	N-Term(iTRAQ4plex); K11(iTRAQ	2	2
[K].aTVLNYLPk.[C]	N-Term(iTRAQ4plex); K9(iTRAQ4	2	2
[K].mLQITNTGFEMk.[L]	N-Term(iTRAQ4plex); K12(iTRAQ	1	1
[K].vQTVPQTcDGHk.[A]	N-Term(iTRAQ4plex); C8(Carbam	1	1
[K].aSPAFLASQNTk.[G]	N-Term(iTRAQ4plex); K12(iTRAQ	1	1
[K].aTVLNYLPk.[C]	N-Term(iTRAQ4plex); K9(iTRAQ4	2	2
[R].iAQWQSLk.[L]	N-Term(iTRAQ4plex); K8(iTRAQ4	1	1
[K].aQWANPFDPSk.[T]	N-Term(iTRAQ4plex); K11(iTRAQ	1	1
[K].aQWANPFDPSk.[T]	N-Term(iTRAQ4plex); K11(iTRAQ	1	1
[K].aQWANPFDPSk.[T]	N-Term(iTRAQ4plex); K11(iTRAQ	1	1
[K].GTEAAAVPEVELSDQPENTFLHPIIQIDR.[S		1	1
[K].TEDSSSFLIDk.[T]	K11(iTRAQ4plex)	1	1
[K].tEDSSSFLIDk.[T]	N-Term(iTRAQ4plex); K11(iTRAQ	1	1
[K].tEDSSSFLIDk.[T]	N-Term(iTRAQ4plex); K11(iTRAQ	1	1
[K].eGQMESVEAAMSSk.[T]	N-Term(iTRAQ4plex); K14(iTRAQ	1	1
[K].fSISATYDLGATLLk.[M]	N-Term(iTRAQ4plex); K15(iTRAQ	1	1
[K].fSISATYDLGATLLk.[M]	N-Term(iTRAQ4plex); K15(iTRAQ	1	1
[K].gWVDLFVPk.[F]	N-Term(iTRAQ4plex); K9(iTRAQ4	1	1
[K].nALALFVLPk.[E]	N-Term(iTRAQ4plex); K10(iTRAQ	1	1
[K].vVGLIQDLkPNTIMVLVNYIHFk.[A]	N-Term(iTRAQ4plex); K9(iTRAQ4	1	1
[K].gWVDLFVPk.[F]	N-Term(iTRAQ4plex); K9(iTRAQ4	1	1
[R].rFTVETPDk.[N]	N-Term(iTRAQ4plex); K9(iTRAQ4	1	1
[K].aVLHIGEk.[G]	N-Term(iTRAQ4plex); K8(iTRAQ4	1	1
[R].sILFLGk.[V]	N-Term(iTRAQ4plex); K7(iTRAQ4	1	1

[R].FTVETPDk.[N]	K8(iTRAQ4plex)	1	1
[K].hLkPLAk.[F]	N-Term(iTRAQ4plex); K3(iTRAQ4	1	1
[R].sILFLGk.[V]	N-Term(iTRAQ4plex); K7(iTRAQ4	1	1
[K].aVLHIGEk.[G]	N-Term(iTRAQ4plex); K8(iTRAQ4	1	1
[K].kELELQIGNALFIGk.[H]	N-Term(iTRAQ4plex); K1(iTRAQ4	1	1
[K].aVLHIGEk.[G]	N-Term(iTRAQ4plex); K8(iTRAQ4	1	1
[R].aAFGQGSGPIMLDEVQcTGTEASLADck.[S	N-Term(iTRAQ4plex); C17(Carbar	1	1
[R].kSQLVYQSR.[R]	N-Term(iTRAQ4plex); K1(iTRAQ4	1	1
[R].rIDITLSSVk.[C]	N-Term(iTRAQ4plex); K10(iTRAQ	1	1
[R].eLSEALGQIFDSQR.[G]	N-Term(iTRAQ4plex)	1	1
[R].eLSEALGQIFDSQR.[G]	N-Term(iTRAQ4plex)	1	1
[R].sDLAVPSELALLk.[A]	N-Term(iTRAQ4plex); K13(iTRAQ	1	1
[R].gQWGTVcDNLWDLTDASVVcR.[A]	N-Term(iTRAQ4plex); C7(Carbam	1	1
[R].gQWGTVcDNLWDLTDASVVcR.[A]	N-Term(iTRAQ4plex); C7(Carbam	1	1
[R].lADGGATNQGR.[V]	N-Term(iTRAQ4plex)	1	1
[R].iYTSPTWSAFVTDSSWSAR.[K]	N-Term(iTRAQ4plex)	1	1
[R].aSHEEVEGLVEk.[I]	N-Term(iTRAQ4plex); K12(iTRAQ	1	1
[R].kSQLVYQSR.[R]	N-Term(iTRAQ4plex); K1(iTRAQ4	1	1
[R].ASHEEVEGLVEk.[I]	K12(iTRAQ4plex)	1	1
[K].lASAYGAR.[Q]	N-Term(iTRAQ4plex)	1	1
[R].rIDITLSSVk.[C]	N-Term(iTRAQ4plex); K10(iTRAQ	1	1
[R].iDITLSSVk.[C]	N-Term(iTRAQ4plex); K9(iTRAQ4	1	1
[K].aVDTWSWGER.[A]	N-Term(iTRAQ4plex)	1	1
[R].aAFGQGSGPIMLDEVQcTGTEASLADck.[S	N-Term(iTRAQ4plex); C17(Carbar	1	1
[R].iDITLSSVk.[C]	N-Term(iTRAQ4plex); K9(iTRAQ4	1	1
[R].sTHTLDLSR.[E]	N-Term(iTRAQ4plex)	1	1
[R].sDLAVPSELALLk.[A]	N-Term(iTRAQ4plex); K13(iTRAQ	1	1
[K].sQLVYQSR.[R]	N-Term(iTRAQ4plex)	1	1
[R].fScFQEEAPQPHYQLR.[A]	N-Term(iTRAQ4plex); C3(Carbam	1	1
[R].vTPNLMGHLcGNQR.[V]	N-Term(iTRAQ4plex); C10(Carbar	1	1
[K].eVGPPLPQEAVPLQk.[E]	N-Term(iTRAQ4plex); K15(iTRAQ	1	1
[R].dPALccYLSPGDEQVNcFNINYLR.[N]	N-Term(iTRAQ4plex); C5(Carbam	1	1
[K].tRPHWccTR.[Q]	N-Term(iTRAQ4plex); C6(Carbam	1	1
[K].lTFINDLcGPR.[R]	N-Term(iTRAQ4plex); C8(Carbam	1	1
[R].rAPYPNYDR.[D]	N-Term(iTRAQ4plex)	1	1
[R].rAPYPNYDR.[D]	N-Term(iTRAQ4plex)	1	1
[R].nVALVSGDTENAk.[G]	N-Term(iTRAQ4plex); K13(iTRAQ	1	1
[R].fcEAEFSVk.[T]	N-Term(iTRAQ4plex); C2(Carbam	1	1
[K].aWEDTLDk.[Y]	N-Term(iTRAQ4plex); K8(iTRAQ4	1	1
[R].fScFQEEAPQPHYQLR.[A]	N-Term(iTRAQ4plex); C3(Carbam	1	1
[K].eVGPPLPQEAVPLQk.[E]	N-Term(iTRAQ4plex); K15(iTRAQ	1	1
[R].eLLALIQLER.[E]	N-Term(iTRAQ4plex)	1	1
[K].tHHHLccR.[H]	N-Term(iTRAQ4plex); C6(Carbam	1	1
[K].lVWEEAMSR.[F]	N-Term(iTRAQ4plex)	1	1
[K].tHHHLccR.[H]	N-Term(iTRAQ4plex); C6(Carbam	1	1
[R].dILTIDIGR.[V]	N-Term(iTRAQ4plex)	1	1
[R].fcEAEFSVk.[T]	N-Term(iTRAQ4plex); C2(Carbam	1	1
[K].lTFINDLcGPR.[R]	N-Term(iTRAQ4plex); C8(Carbam	1	1
[R].vTPNLMGHLcGNQR.[V]	N-Term(iTRAQ4plex); C10(Carbar	1	1
[R].tPAYYPNAGLIk.[N]	N-Term(iTRAQ4plex); K12(iTRAQ	1	1
[R].nPDPVAAPWcYTTDPSVR.[W]	N-Term(iTRAQ4plex); C10(Carbar	1	1
[R].rIPLYYPNAGLTR.[N]	N-Term(iTRAQ4plex)	1	1

[R].lFLEPTQADIALLk.[L]	N-Term(iTRAQ4plex); K14(iTRAQ4	1	1
[R].lFLEPTQADIALLk.[L]	N-Term(iTRAQ4plex); K14(iTRAQ4	1	1
[R].gTDScQGDSGGPLVcFEk.[D]	N-Term(iTRAQ4plex); C5(Carbam	1	1
[R].gTYSTTVTGR.[T]	N-Term(iTRAQ4plex)	1	1
[R].gTYSTTVTGR.[T]	N-Term(iTRAQ4plex)	1	1
[R].gTDScQGDSGGPLVcFEk.[D]	N-Term(iTRAQ4plex); C5(Carbam	1	1
[R].nPDAVAAPYcYTR.[D]	N-Term(iTRAQ4plex); C10(Carbar	1	1
[R].gTFSTTVTGR.[T]	N-Term(iTRAQ4plex)	1	1
[K].yIcAEHLAR.[G]	N-Term(iTRAQ4plex); C3(Carbam	1	1
[R].gTYSTTVTGR.[T]	N-Term(iTRAQ4plex)	1	1
[R].gTYSTTVTGR.[T]	N-Term(iTRAQ4plex)	1	1
[K].sPVVQDcYHGDGR.[S]	N-Term(iTRAQ4plex); C7(Carbam	1	1
[R].tPEYYPNAGLIMNYcR.[N]	N-Term(iTRAQ4plex); C15(Carbar	1	1
[R].nPDAEISPWcYTMDPNVR.[W]	N-Term(iTRAQ4plex); C10(Carbar	1	1
[R].GTYSTTVTGR.[T]		1	1
[K].vILGAHQEVNLESHVQEIEVSR.[L]	N-Term(iTRAQ4plex)	1	1
[R].gISSTTVTGR.[T]	N-Term(iTRAQ4plex)	1	1
[R].gTYSTTVTGR.[T]	N-Term(iTRAQ4plex)	1	1
[R].aTWSGAVLAGR.[D]	N-Term(iTRAQ4plex)	1	1
[R].lELHVDGPPPRPQLR.[A]	N-Term(iTRAQ4plex)	1	1
[R].lELHVDGPPPRPQLR.[A]	N-Term(iTRAQ4plex)	1	1
[R].lELHVDGPPPRPQLR.[A]	N-Term(iTRAQ4plex)	1	1
[K].hQFLLTGDTQGR.[Y]	N-Term(iTRAQ4plex)	1	1
[K].hQFLLTGDTQGR.[Y]	N-Term(iTRAQ4plex)	1	1
[K].hQFLLTGDTQGR.[Y]	N-Term(iTRAQ4plex)	1	1
[K].hQFLLTGDTQGR.[Y]	N-Term(iTRAQ4plex)	1	1
[K].HQFLLTGDTQGR.[Y]		1	1
[K].hQFLLTGDTQGR.[Y]	N-Term(iTRAQ4plex)	1	1
[K].hQFLLTGDTQGR.[Y]	N-Term(iTRAQ4plex)	1	1
[K].hQFLLTGDTQGR.[Y]	N-Term(iTRAQ4plex)	1	1
[K].hQFLLTGDTQGR.[Y]	N-Term(iTRAQ4plex)	1	1
[K].HQFLLTGDTQGR.[Y]		1	1
[K].hQFLLTGDTQGR.[Y]	N-Term(iTRAQ4plex)	1	1
[K].hQFLLTGDTQGR.[Y]	N-Term(iTRAQ4plex)	1	1
[K].hQFLLTGDTQGR.[Y]	N-Term(iTRAQ4plex)	1	1
[K].hQFLLTGDTQGR.[Y]	N-Term(iTRAQ4plex)	1	1
[R].lELHVDGPPPRPQLR.[A]	N-Term(iTRAQ4plex)	1	1
[R].lELHVDGPPPRPQLR.[A]	N-Term(iTRAQ4plex)	1	1
[R].lELHVDGPPPRPQLR.[A]	N-Term(iTRAQ4plex)	1	1
[K].HQFLLTGDTQGR.[Y]		1	1
[K].HQFLLTGDTQGR.[Y]		1	1
[R].lELHVDGPPPRPQLR.[A]	N-Term(iTRAQ4plex)	1	1
[R].aTWSGAVLAGR.[D]	N-Term(iTRAQ4plex)	1	1
[R].aTWSGAVLAGR.[D]	N-Term(iTRAQ4plex)	1	1
[R].aTWSGAVLAGR.[D]	N-Term(iTRAQ4plex)	1	1
[R].aTWSGAVLAGR.[D]	N-Term(iTRAQ4plex)	1	1
[R].sGLSTGWTQLSk.[L]	N-Term(iTRAQ4plex); K12(iTRAQ4	1	1
[R].lELHVDGPPPRPQLR.[A]	N-Term(iTRAQ4plex)	1	1
[R].lELHVDGPPPRPQLR.[A]	N-Term(iTRAQ4plex)	1	1
[R].lELHVDGPPPRPQLR.[A]	N-Term(iTRAQ4plex)	1	1
[R].lELHVDGPPPRPQLR.[A]	N-Term(iTRAQ4plex)	1	1
[R].lELHVDGPPPRPQLR.[A]	N-Term(iTRAQ4plex)	1	1

[R].lELHVDGPPPRPQLR.[A]	N-Term(iTRAQ4plex)	1	1
[R].lELHVDGPPPRPQLR.[A]	N-Term(iTRAQ4plex)	1	1
[R].lELHVDGPPPRPQLR.[A]	N-Term(iTRAQ4plex)	1	1
[R].lELHVDGPPPRPQLR.[A]	N-Term(iTRAQ4plex)	1	1
[R].lELHVDGPPPRPQLR.[A]	N-Term(iTRAQ4plex)	1	1
[R].lELHVDGPPPRPQLR.[A]	N-Term(iTRAQ4plex)	1	1
[R].lELHVDGPPPRPQLR.[A]	N-Term(iTRAQ4plex)	1	1
[R].lELHVDGPPPRPQLR.[A]	N-Term(iTRAQ4plex)	1	1
[R].lELHVDGPPPRPQLR.[A]	N-Term(iTRAQ4plex)	1	1
[R].lELHVDGPPPRPQLR.[A]	N-Term(iTRAQ4plex)	1	1
[R].sWVPHTFESELSDPVELLVAES.[-]	N-Term(iTRAQ4plex)	1	1
[R].sWVPHTFESELSDPVELLVAES.[-]	N-Term(iTRAQ4plex)	1	1
[K].hQFLLTGDTQGR.[Y]	N-Term(iTRAQ4plex)	1	1
[R].ATWSGAVLAGR.[D]		1	1
[R].sGLSTGWTQLSk.[L]	N-Term(iTRAQ4plex); K12(iTRAQ	1	1
[R].sGLSTGWTQLSk.[L]	N-Term(iTRAQ4plex); K12(iTRAQ	1	1
[R].sGLSTGWTQLSk.[L]	N-Term(iTRAQ4plex); K12(iTRAQ	1	1
[K].lLELTGPk.[S]	N-Term(iTRAQ4plex); K8(iTRAQ4	1	1
[K].lLELTGPk.[S]	N-Term(iTRAQ4plex); K8(iTRAQ4	1	1
[K].lLELTGPk.[S]	N-Term(iTRAQ4plex); K8(iTRAQ4	1	1
[K].lLELTGPk.[S]	N-Term(iTRAQ4plex); K8(iTRAQ4	1	1
[R].lELHVDGPPPRPQLR.[A]	N-Term(iTRAQ4plex)	1	1
[R].lELHVDGPPPRPQLR.[A]	N-Term(iTRAQ4plex)	1	1
[R].lELHVDGPPPRPQLR.[A]	N-Term(iTRAQ4plex)	1	1
[R].lELHVDGPPPRPQLR.[A]	N-Term(iTRAQ4plex)	1	1
[R].lELHVDGPPPRPQLR.[A]	N-Term(iTRAQ4plex)	1	1
[R].lELHVDGPPPRPQLR.[A]	N-Term(iTRAQ4plex)	1	1
[R].lELHVDGPPPRPQLR.[A]	N-Term(iTRAQ4plex)	1	1
[R].lELHVDGPPPRPQLR.[A]	N-Term(iTRAQ4plex)	1	1
[R].lELHVDGPPPRPQLR.[A]	N-Term(iTRAQ4plex)	1	1
[R].lELHVDGPPPRPQLR.[A]	N-Term(iTRAQ4plex)	1	1
[R].cEGPIPDVTFELLR.[E]	N-Term(iTRAQ4plex); C1(Carbam	1	1
[R].cEGPIPDVTFELLR.[E]	N-Term(iTRAQ4plex); C1(Carbam	1	1
[R].tPGAAANLELIFVGPQHAGNYR.[C]	N-Term(iTRAQ4plex)	1	1
[R].TPGAAANLELIFVGPQHAGNYR.[C]		1	1
[R].tPGAAANLELIFVGPQHAGNYR.[C]	N-Term(iTRAQ4plex)	1	1
[R].tPGAAANLELIFVGPQHAGNYR.[C]	N-Term(iTRAQ4plex)	1	1
[R].tPGAAANLELIFVGPQHAGNYR.[C]	N-Term(iTRAQ4plex)	1	1
[R].tPGAAANLELIFVGPQHAGNYR.[C]	N-Term(iTRAQ4plex)	1	1
[K].vTLTcVAPLSGVDFQLR.[R]	N-Term(iTRAQ4plex); C5(Carbam	1	1
[K].vTLTcVAPLSGVDFQLR.[R]	N-Term(iTRAQ4plex); C5(Carbam	1	1
[K].vTLTcVAPLSGVDFQLR.[R]	N-Term(iTRAQ4plex); C5(Carbam	1	1
[K].sLPAPWLSMAPVSWITPGLk.[T]	N-Term(iTRAQ4plex); K20(iTRAQ	1	1
[K].nGVAQEPVHLDSPAIk.[H]	N-Term(iTRAQ4plex); K16(iTRAQ	1	1
[K].sLPAPWLSMAPVSWITPGLk.[T]	N-Term(iTRAQ4plex); K20(iTRAQ	1	1
[K].sLPAPWLSMAPVSWITPGLk.[T]	N-Term(iTRAQ4plex); K20(iTRAQ	1	1
[R].iFFHLNAVALGDGGHYTcR.[Y]	N-Term(iTRAQ4plex); C18(Carbar	1	1
[R].iFFHLNAVALGDGGHYTcR.[Y]	N-Term(iTRAQ4plex); C18(Carbar	1	1
[R].iFFHLNAVALGDGGHYTcR.[Y]	N-Term(iTRAQ4plex); C18(Carbar	1	1
[K].sLPAPWLSmAPVSWITPGLk.[T]	N-Term(iTRAQ4plex); M9(Oxidat	1	1
[R].iFFHLNAVALGDGGHYTcR.[Y]	N-Term(iTRAQ4plex); C18(Carbar	1	1
[R].iFFHLNAVALGDGGHYTcR.[Y]	N-Term(iTRAQ4plex); C18(Carbar	1	1

[R].tPGAAANLELIFVGPQHAGNYR.[C]	N-Term(iTRAQ4plex)	1	1
[K].hQFLLTGDTQGR.[Y]	N-Term(iTRAQ4plex)	1	1
[K].hQFLLTGDTQGR.[Y]	N-Term(iTRAQ4plex)	1	1
[K].HQFLLTGDTQGR.[Y]		1	1
[R].tPGAAANLELIFVGPQHAGNYR.[C]	N-Term(iTRAQ4plex)	1	1
[R].lELHVDGPPPRPQLR.[A]	N-Term(iTRAQ4plex)	1	1
[R].lELHVDGPPPRPQLR.[A]	N-Term(iTRAQ4plex)	1	1
[R].lELHVDGPPPRPQLR.[A]	N-Term(iTRAQ4plex)	1	1
[R].lELHVDGPPPRPQLR.[A]	N-Term(iTRAQ4plex)	1	1
[K].lLELTGPK.[S]	N-Term(iTRAQ4plex)	1	1
[K].vTLTcVAPLSGVDFQLR.[R]	N-Term(iTRAQ4plex); C5(Carbam	1	1
[K].lLELTGPk.[S]	N-Term(iTRAQ4plex); K8(iTRAQ4	1	1
[R].aTWSGAVLAGR.[D]	N-Term(iTRAQ4plex)	1	1
[R].ATWSGAVLAGR.[D]		1	1
[R].aTWSGAVLAGR.[D]	N-Term(iTRAQ4plex)	1	1
[K].lLELTGPk.[S]	N-Term(iTRAQ4plex); K8(iTRAQ4	1	1
[R].LELHVDGPPPRPQLR.[A]		1	1
[R].ATWSGAVLAGRDAVLR.[C]		1	1
[K].hQFLLTGDTQGR.[Y]	N-Term(iTRAQ4plex)	1	1
[K].lLELTGPk.[S]	N-Term(iTRAQ4plex); K8(iTRAQ4	1	1
[R].tPGAAANLELIFVGPQHAGNYR.[C]	N-Term(iTRAQ4plex)	1	1
[R].aTWSGAVLAGR.[D]	N-Term(iTRAQ4plex)	1	1
[K].LLELTGPk.[S]	K8(iTRAQ4plex)	1	1
[R].aTWSGAVLAGR.[D]	N-Term(iTRAQ4plex)	1	1
[R].cLAPLEGAR.[F]	C1(Carbamidomethyl)	1	1
[R].lELHVDGPPPRPQLR.[A]	N-Term(iTRAQ4plex)	1	1
[K].LLELTGPk.[S]	K8(iTRAQ4plex)	1	1
[R].sGLSTGWTQLSk.[L]	N-Term(iTRAQ4plex); K12(iTRAQ	1	1
[K].hQFLLTGDTQGR.[Y]	N-Term(iTRAQ4plex)	1	1
[R].LELHVDGPPPRPQLR.[A]		1	1
[R].IFFHLNAVALGDGGHYTcR.[Y]	C18(Carbamidomethyl)	1	1
[K].LLELTGPk.[S]	K8(iTRAQ4plex)	1	1
[R].LELHVDGPPPRPQLR.[A]		1	1
[K].lLELTGPk.[S]	N-Term(iTRAQ4plex); K8(iTRAQ4	1	1
[R].sGLSTGWTQLSk.[L]	N-Term(iTRAQ4plex); K12(iTRAQ	1	1
[K].lLELTGPk.[S]	N-Term(iTRAQ4plex); K8(iTRAQ4	1	1
[R].GEkELLVPR.[S]	K3(iTRAQ4plex)	1	1
[K].hQFLLTGDTQGR.[Y]	N-Term(iTRAQ4plex)	1	1
[R].aTWSGAVLAGR.[D]	N-Term(iTRAQ4plex)	1	1
[R].LELHVDGPPPRPQLR.[A]		1	1
[R].LELHVDGPPPRPQLR.[A]		1	1
[R].aTWSGAVLAGR.[D]	N-Term(iTRAQ4plex)	1	1
[R].aTWSGAVLAGR.[D]	N-Term(iTRAQ4plex)	1	1
[R].LELHVDGPPPRPQLR.[A]		1	1
[K].hQFLLTGDTQGR.[Y]	N-Term(iTRAQ4plex)	1	1
[K].LLELTGPk.[S]	K8(iTRAQ4plex)	1	1
[K].tFYEPGEEITYSckPGYVSR.[G]	N-Term(iTRAQ4plex); C13(Carbar	1	1
[K].tFYEPGEEITYSckPGYVSR.[G]	N-Term(iTRAQ4plex); C13(Carbar	1	1
[K].tFYEPGEEITYSckPGYVSR.[G]	N-Term(iTRAQ4plex); C13(Carbar	1	1
[K].tFYEPGEEITYSckPGYVSR.[G]	N-Term(iTRAQ4plex); C13(Carbar	1	1
[K].tFYEPGEEITYSckPGYVSR.[G]	N-Term(iTRAQ4plex); C13(Carbar	1	1
[K].kATVVYQGER.[V]	N-Term(iTRAQ4plex); K1(iTRAQ4	1	1

[K].eHSSLAFWk.[T]	N-Term(iTRAQ4plex); K9(iTRAQ4	1	1
[R].tcPkPDDLPFSTVVPLk.[T]	N-Term(iTRAQ4plex); C2(Carbam	1	1
[R].tcPkPDDLPFSTVVPLk.[T]	N-Term(iTRAQ4plex); C2(Carbam	1	1
[R].tcPkPDDLPFSTVVPLk.[T]	N-Term(iTRAQ4plex); C2(Carbam	1	1
[K].aTVVYQGER.[V]	N-Term(iTRAQ4plex)	1	1
[K].eHSSLAFWk.[T]	N-Term(iTRAQ4plex); K9(iTRAQ4	1	1
[K].eHSSLAFWk.[T]	N-Term(iTRAQ4plex); K9(iTRAQ4	1	1
[K].eHSSLAFWk.[T]	N-Term(iTRAQ4plex); K9(iTRAQ4	1	1
[K].eHSSLAFWk.[T]	N-Term(iTRAQ4plex); K9(iTRAQ4	1	1
[K].eHSSLAFWk.[T]	N-Term(iTRAQ4plex); K9(iTRAQ4	1	1
[R].tcPkPDDLPFSTVVPLk.[T]	N-Term(iTRAQ4plex); C2(Carbam	1	1
[R].tcPkPDDLPFSTVVPLk.[T]	N-Term(iTRAQ4plex); C2(Carbam	1	1
[R].TcPkPDDLPFSTVVPLk.[T]	C2(Carbamidomethyl); K4(iTRAC	1	1
[R].TcPkPDDLPFSTVVPLk.[T]	C2(Carbamidomethyl); K4(iTRAC	1	1
[R].tcPKPDDLPFSTVVPLk.[T]	N-Term(iTRAQ4plex); C2(Carbam	1	1
[R].TcPkPDDLPFSTVVPLk.[T]	C2(Carbamidomethyl); K4(iTRAC	1	1
[R].tcPkPDDLPFSTVVPLk.[T]	N-Term(iTRAQ4plex); C2(Carbam	1	1
[R].tcPkPDDLPFSTVVPLk.[T]	N-Term(iTRAQ4plex); C2(Carbam	1	1
[R].tcPkPDDLPFSTVVPLk.[T]	N-Term(iTRAQ4plex); C2(Carbam	1	1
[K].kcSYTEDAQcIDGTIEVPk.[C]	N-Term(iTRAQ4plex); K1(iTRAQ4	1	1
[K].kcSYTEDAQcIDGTIEVPk.[C]	N-Term(iTRAQ4plex); K1(iTRAQ4	1	1
[K].kcSYTEDAQcIDGTIEVPk.[C]	N-Term(iTRAQ4plex); K1(iTRAQ4	1	1
[K].ATFGcHDGYSLDGPEEIEcTk.[L]	C5(Carbamidomethyl); C19(Carb	1	1
[K].kcSYTEDAQcIDGTIEVPk.[C]	N-Term(iTRAQ4plex); K1(iTRAQ4	1	1
[K].aTFGcHDGYSLDGPEEIEcTk.[L]	N-Term(iTRAQ4plex); C5(Carbam	1	1
[K].aTFGcHDGYSLDGPEEIEcTk.[L]	N-Term(iTRAQ4plex); C5(Carbam	1	1
[K].kATVVYQGER.[V]	N-Term(iTRAQ4plex); K1(iTRAQ4	1	1
[K].aTFGcHDGYSLDGPEEIEcTk.[L]	N-Term(iTRAQ4plex); C5(Carbam	1	1
[K].aTFGcHDGYSLDGPEEIEcTk.[L]	N-Term(iTRAQ4plex); C5(Carbam	1	1
[K].aTFGcHDGYSLDGPEEIEcTk.[L]	N-Term(iTRAQ4plex); C5(Carbam	1	1
[K].aTFGcHDGYSLDGPEEIEcTk.[L]	N-Term(iTRAQ4plex); C5(Carbam	1	1
[K].aTFGcHDGYSLDGPEEIEcTk.[L]	N-Term(iTRAQ4plex); C5(Carbam	1	1
[K].aTFGcHDGYSLDGPEEIEcTk.[L]	N-Term(iTRAQ4plex); C5(Carbam	1	1
[K].aTFGcHDGYSLDGPEEIEcTk.[L]	N-Term(iTRAQ4plex); C5(Carbam	1	1
[K].aTFGcHDGYSLDGPEEIEcTk.[L]	N-Term(iTRAQ4plex); C5(Carbam	1	1
[K].aTFGcHDGYSLDGPEEIEcTk.[L]	N-Term(iTRAQ4plex); C5(Carbam	1	1
[K].aTFGcHDGYSLDGPEEIEcTk.[L]	N-Term(iTRAQ4plex); C5(Carbam	1	1
[K].aTFGcHDGYSLDGPEEIEcTk.[L]	N-Term(iTRAQ4plex); C5(Carbam	1	1
[K].cSYTEDAQcIDGTIEVPk.[C]	N-Term(iTRAQ4plex); C1(Carbam	1	1
[K].cSYTEDAQcIDGTIEVPk.[C]	N-Term(iTRAQ4plex); C1(Carbam	1	1
[R].kFIcPLTGLWPINTLk.[C]	K1(iTRAQ4plex); C4(Carbamidon	1	1
[K].tFYEPGEEITYSckPGYVSR.[G]	N-Term(iTRAQ4plex); C13(Carbar	1	1
[R].kFIcPLTGLWPINTLk.[C]	N-Term(iTRAQ4plex); K1(iTRAQ4	1	1
[R].kFIcPLTGLWPINTLk.[C]	N-Term(iTRAQ4plex); K1(iTRAQ4	1	1
[R].kFIcPLTGLWPINTLk.[C]	N-Term(iTRAQ4plex); C4(Carbam	1	1
[R].kFIcPLTGLWPINTLk.[C]	N-Term(iTRAQ4plex); K1(iTRAQ4	1	1
[R].kFIcPLTGLWPINTLk.[C]	N-Term(iTRAQ4plex); K1(iTRAQ4	1	1
[K].tDASDVkPc.[-]	N-Term(iTRAQ4plex); K7(iTRAQ4	1	1
[K].wSPELPVcAPIIcPPPSIPTFATLR.[V]	N-Term(iTRAQ4plex); C8(Carbam	1	1
[K].wSPELPVcAPIIcPPPSIPTFATLR.[V]	N-Term(iTRAQ4plex); C8(Carbam	1	1
[K].WSPELPVcAPIIcPPPSIPTFATLR.[V]	C8(Carbamidomethyl); C13(Carb	1	1
[K].wSPELPVcAPIIcPPPSIPTFATLR.[V]	N-Term(iTRAQ4plex); C8(Carbam	1	1

[K].ATVVYQGER.[V]		1	1
[K].kcSYTEDAQcIDGTIEVPk.[C]	N-Term(iTRAQ4plex); K1(iTRAQ4	1	1
[K].TFYEPGEEITYSckPGYVSR.[G]	C13(Carbamidomethyl); K14(iTR	1	1
[K].FIcPLTGLWPINTLk.[C]	C3(Carbamidomethyl); K15(iTRA	1	1
[K].tFYEPGEEITYSckPGYVSR.[G]	N-Term(iTRAQ4plex); C13(Carbar	1	1
[K].tFYEPGEEITYSckPGYVSR.[G]	N-Term(iTRAQ4plex); C13(Carbar	1	1
[K].aTFGcHDGYSLDGPEEIEcTk.[L]	N-Term(iTRAQ4plex); C5(Carbam	1	1
[K].aTFGcHDGYSLDGPEEIEcTk.[L]	N-Term(iTRAQ4plex); C5(Carbam	1	1
[K].kATVVYQGER.[V]	N-Term(iTRAQ4plex); K1(iTRAQ4	1	1
[R].TcPkPDDLPFSTVVPLk.[T]	C2(Carbamidomethyl); K4(iTRAC	1	1
[K].aTFGcHDGYSLDGPEEIEcTk.[L]	N-Term(iTRAQ4plex); C5(Carbam	1	1
[R].tcPkPDDLPFSTVVPLk.[T]	N-Term(iTRAQ4plex); C2(Carbam	1	1
[K].aTFGcHDGYSLDGPEEIEcTk.[L]	N-Term(iTRAQ4plex); C5(Carbam	1	1
[K].nGmLHGDk.[V]	N-Term(iTRAQ4plex); M3(Oxidat	1	1
[K].aTVVYQGER.[V]	N-Term(iTRAQ4plex)	1	1
[K].aTVVYQGER.[V]	N-Term(iTRAQ4plex)	1	1
[K].TDASDVkPc.[-]	K7(iTRAQ4plex); C9(Carbamidon	1	1
[K].TDASDVkPc.[-]	K7(iTRAQ4plex); C9(Carbamidon	1	1
[K].cFkEHSSLAFWk.[T]	N-Term(iTRAQ4plex); C1(Carbam	1	1
[R].tcPkPDDLPFSTVVPLk.[T]	N-Term(iTRAQ4plex); C2(Carbam	1	1
[R].tcPkPDDLPFSTVVPLK.[T]	N-Term(iTRAQ4plex); C2(Carbam	1	1
[R].tcPkPDDLPFSTVVPLk.[T]	N-Term(iTRAQ4plex); C2(Carbam	1	1
[K].tDASDVkPc.[-]	N-Term(iTRAQ4plex); K7(iTRAQ4	1	1
[K].aTVVYQGER.[V]	N-Term(iTRAQ4plex)	1	1
[K].tDASDVkPc.[-]	N-Term(iTRAQ4plex); K7(iTRAQ4	1	1
[K].aTVVYQGER.[V]	N-Term(iTRAQ4plex)	1	1
[R].TcPkPDDLPFSTVVPLk.[T]	C2(Carbamidomethyl); K4(iTRAC	1	1
[R].tcPKPDDLPFSTVVPLk.[T]	N-Term(iTRAQ4plex); C2(Carbam	1	1
[K].lGNWSAMPSck.[A]	N-Term(iTRAQ4plex); C10(Carbar	1	1
[K].tDASDVkPc.[-]	N-Term(iTRAQ4plex); K7(iTRAQ4	1	1
[K].aTFGcHDGYSLDGPEEIEcTk.[L]	N-Term(iTRAQ4plex); C5(Carbam	1	1
[K].nGMLHGDk.[V]	N-Term(iTRAQ4plex); K8(iTRAQ4	1	1
[K].tDASDVkPc.[-]	N-Term(iTRAQ4plex); K7(iTRAQ4	1	1
[K].tDASDVkPc.[-]	N-Term(iTRAQ4plex); K7(iTRAQ4	1	1
[K].aTVVYQGER.[V]	N-Term(iTRAQ4plex)	1	1
[K].tDASDVkPc.[-]	N-Term(iTRAQ4plex); K7(iTRAQ4	1	1
[R].tcPKPDDLPFSTVVPLk.[T]	N-Term(iTRAQ4plex); C2(Carbam	1	1
[R].TcPkPDDLPFSTVVPLk.[T]	C2(Carbamidomethyl); K4(iTRAC	1	1
[K].eHSSLAFWk.[T]	N-Term(iTRAQ4plex); K9(iTRAQ4	1	1
[K].ATVVYQGER.[V]		1	1
[R].kFIcPLTGLWPINTLk.[C]	N-Term(iTRAQ4plex); C4(Carbam	1	1
[R].kFIcPLTGLWPINTLk.[C]	K1(iTRAQ4plex); C4(Carbamidon	1	1
[K].ATVVYQGER.[V]		1	1
[R].tcPKPDDLPFSTVVPLk.[T]	N-Term(iTRAQ4plex); C2(Carbam	1	1
[R].TcPkPDDLPFSTVVPLk.[T]	C2(Carbamidomethyl); K4(iTRAC	1	1
[R].tcPkPDDLPFSTVVPLk.[T]	N-Term(iTRAQ4plex); C2(Carbam	1	1
[K].aTVVYQGER.[V]	N-Term(iTRAQ4plex)	1	1
[R].tcPKPDDLPFSTVVPLk.[T]	N-Term(iTRAQ4plex); C2(Carbam	1	1
[R].TcPkPDDLPFSTVVPLk.[T]	C2(Carbamidomethyl); K4(iTRAC	1	1
[K].aTFGcHDGYSLDGPEEIEcTk.[L]	N-Term(iTRAQ4plex); C5(Carbam	1	1
[R].tcPkPDDLPFSTVVPLk.[T]	N-Term(iTRAQ4plex); C2(Carbam	1	1
[R].dWHGVPGQVDAAMAGR.[I]	N-Term(iTRAQ4plex)	1	1

[R].dWHGVPGQVDAAMAGR.[I]	N-Term(iTRAQ4plex)	1	1
[R].dWHGVPGQVDAAMAGR.[I]	N-Term(iTRAQ4plex)	1	1
[R].dWHGVPGQVDAAMAGR.[I]	N-Term(iTRAQ4plex)	1	1
[R].dWHGVPGQVDAAMAGR.[I]	N-Term(iTRAQ4plex)	1	1
[R].dWHGVPGQVDAAMAGR.[I]	N-Term(iTRAQ4plex)	1	1
[R].dWHGVPGQVDAAMAGR.[I]	N-Term(iTRAQ4plex)	1	1
[R].dWHGVPGQVDAAMAGR.[I]	N-Term(iTRAQ4plex)	1	1
[R].IYISGMAPRPSLAk.[K]	K14(iTRAQ4plex)	1	1
[R].dWHGVPGQVDAAMAGR.[I]	N-Term(iTRAQ4plex)	1	1
[K].aVRPGYPk.[L]	N-Term(iTRAQ4plex); K8(iTRAQ4	1	1
[R].fEDGVLDPDYPR.[N]	N-Term(iTRAQ4plex)	1	1
[R].fEDGVLDPDYPR.[N]	N-Term(iTRAQ4plex)	1	1
[R].dWHGVPGQVDAAmAGR.[I]	N-Term(iTRAQ4plex); M13(Oxida	1	1
[R].dWHGVPGQVDAAMAGR.[I]	N-Term(iTRAQ4plex)	1	1
[K].aVRPGYPk.[L]	N-Term(iTRAQ4plex); K8(iTRAQ4	1	1
[R].iYISGMAPRPSLAk.[K]	N-Term(iTRAQ4plex); K14(iTRAQ	1	1
[R].DVWGIEGPIDAAFTR.[I]		1	1
[R].fEDGVLDPDYPR.[N]	N-Term(iTRAQ4plex)	1	1
[R].fEDGVLDPDYPR.[N]	N-Term(iTRAQ4plex)	1	1
[R].fEDGVLDPDYPR.[N]	N-Term(iTRAQ4plex)	1	1
[R].fEDGVLDPDYPR.[N]	N-Term(iTRAQ4plex)	1	1
[R].fEDGVLDPDYPR.[N]	N-Term(iTRAQ4plex)	1	1
[R].fEDGVLDPDYPR.[N]	N-Term(iTRAQ4plex)	1	1
[R].sIAQYWLGcPAPGHL.[-]	N-Term(iTRAQ4plex); C9(Carbam	1	1
[R].FEDGVLDPDYPR.[N]		1	1
[R].FEDGVLDPDYPR.[N]		1	1
[R].cTEGFNVDk.[K]	N-Term(iTRAQ4plex); C1(Carbam	1	1
[R].gQYcYELDEk.[A]	N-Term(iTRAQ4plex); C4(Carbam	1	1
[R].rVDTVDPPYPR.[S]	N-Term(iTRAQ4plex)	1	1
[R].rVDTVDPPYPR.[S]	N-Term(iTRAQ4plex)	1	1
[R].cTEGFNVDk.[K]	N-Term(iTRAQ4plex); C1(Carbam	1	1
[R].rVDTVDPPYPR.[S]	N-Term(iTRAQ4plex)	1	1
[R].rVDTVDPPYPR.[S]	N-Term(iTRAQ4plex)	1	1
[R].tSAGTRQPQFISR.[D]	N-Term(iTRAQ4plex)	1	1
[R].mDWLVPATcEPIQSVFFFSGDk.[Y]	N-Term(iTRAQ4plex); M1(Oxidat	1	1
[R].DWHGVPGQVDAAMAGR.[I]		1	1
[R].mDWLVPATcEPIQSVFFFSGDk.[Y]	N-Term(iTRAQ4plex); C9(Carbam	1	1
[R].mDWLVPATcEPIQSVFFFSGDk.[Y]	N-Term(iTRAQ4plex); C9(Carbam	1	1
[R].rVDTVDPPYPR.[S]	N-Term(iTRAQ4plex)	1	1
[R].gDVFTMPEDEYTVYDDGEEk.[N]	N-Term(iTRAQ4plex); K20(iTRAQ	1	1
[R].rVDTVDPPYPR.[S]	N-Term(iTRAQ4plex)	1	1
[R].gQYcYELDEk.[A]	N-Term(iTRAQ4plex); C4(Carbam	1	1
[K].cQcDELcSYYQSccTDYTAEckPQVTR.[G]	N-Term(iTRAQ4plex); C1(Carbam	1	1
[R].TSAGTRQPQFISR.[D]		1	1
[R].sIAQYWLGcPAPGHL.[-]	N-Term(iTRAQ4plex); C9(Carbam	1	1
[R].cTEGFNVDk.[K]	N-Term(iTRAQ4plex); C1(Carbam	1	1
[R].vDTVDPPYPR.[S]	N-Term(iTRAQ4plex)	1	1
[R].RVDTVDPPYPR.[S]		1	1
[K].aVRPGYPk.[L]	N-Term(iTRAQ4plex); K8(iTRAQ4	1	1
[R].dWHGVPGQVDAAMAGR.[I]	N-Term(iTRAQ4plex)	1	1
[R].DWHGVPGQVDAAMAGR.[I]		1	1
[R].dVWGIEGPIDAAFTR.[I]	N-Term(iTRAQ4plex)	1	1

[R].cTEGFNVDkk.[C]	N-Term(iTRAQ4plex); C1(Carbam	1	1
[R].DWHGVPGQVDAAMAGR.[I]		1	1
[R].vDTVDPPYPR.[S]	N-Term(iTRAQ4plex)	1	1
[R].RVDTVDPPYPR.[S]		1	1
[R].fEDGVLDPDYPR.[N]	N-Term(iTRAQ4plex)	1	1
[R].fEDGVLDPDYPR.[N]	N-Term(iTRAQ4plex)	1	1
[R].cTEGFNVDk.[K]	C1(Carbamidomethyl); K9(iTRAC	1	1
[R].dVWGIEGPIDAAFTR.[I]	N-Term(iTRAQ4plex)	1	1
[R].fEDGVLDPDYPR.[N]	N-Term(iTRAQ4plex)	1	1
[R].DWHGVPGQVDAAmAGR.[I]	M13(Oxidation)	1	1
[R].rVDTVDPPYPR.[S]	N-Term(iTRAQ4plex)	1	1
[R].gQYcYELDEk.[A]	N-Term(iTRAQ4plex); C4(Carbam	1	1
[R].RVDTVDPPYPR.[S]		1	1
[R].sIAQYWLGcPAPGHL.[-]	N-Term(iTRAQ4plex); C9(Carbam	1	1
[R].iYISGMAPRPSLAk.[K]	N-Term(iTRAQ4plex); K14(iTRAQ	1	1
[R].RVDTVDPPYPR.[S]		1	1
[R].gQYcYELDEk.[A]	N-Term(iTRAQ4plex); C4(Carbam	1	1
[R].iYISGMAPRPSLAk.[K]	N-Term(iTRAQ4plex); K14(iTRAQ	1	1
[R].DWHGVPGQVDAAMAGR.[I]		1	1
[R].dVWGIEGPIDAAFTR.[I]	N-Term(iTRAQ4plex)	1	1
[K].aVRPGYPk.[L]	N-Term(iTRAQ4plex); K8(iTRAQ4	1	1
[K].lIcQATGFSPR.[Q]	N-Term(iTRAQ4plex); C3(Carbam	1	2
[K].lIcQATGFSPR.[Q]	N-Term(iTRAQ4plex); C3(Carbam	1	2
[K].gVALHRPDVYLLPPAR.[E]	N-Term(iTRAQ4plex)	1	2
[K].gVALHRPDVYLLPPAR.[E]	N-Term(iTRAQ4plex)	1	2
[K].gVALHRPDVYLLPPAR.[E]	N-Term(iTRAQ4plex)	1	2
[K].LIcQATGFSPR.[Q]	C3(Carbamidomethyl)	1	2
[K].nVPLPVIAELPPk.[V]	N-Term(iTRAQ4plex); K13(iTRAQ	1	1
[K].nVPLPVIAELPPk.[V]	N-Term(iTRAQ4plex); K13(iTRAQ	1	1
[R].fTcTVTHTDLPSPLk.[Q]	N-Term(iTRAQ4plex); C3(Carbam	1	2
[R].fTcTVTHTDLPSPLk.[Q]	N-Term(iTRAQ4plex); C3(Carbam	1	2
[K].yAATSQVLLPSk.[D]	N-Term(iTRAQ4plex); K12(iTRAQ	1	1
[K].dVMQGTDEHVVck.[V]	N-Term(iTRAQ4plex); C12(Carbar	1	1
[K].qVGSGVTTDQVQAEAk.[E]	N-Term(iTRAQ4plex); K16(iTRAQ	1	1
[K].qVGSGVTTDQVQAEAk.[E]	N-Term(iTRAQ4plex); K16(iTRAQ	1	1
[K].dVmQGTDEHVVck.[V]	N-Term(iTRAQ4plex); M3(Oxidat	1	1
[K].dVMQGTDEHVVck.[V]	N-Term(iTRAQ4plex); C12(Carbar	1	1
[R].vFAIPPSFASIFLTk.[S]	N-Term(iTRAQ4plex); K15(iTRAQ	1	2
[K].yVTSAPMPEPQAPGR.[Y]	N-Term(iTRAQ4plex)	1	2
[K].eSGPTTYk.[V]	N-Term(iTRAQ4plex); K8(iTRAQ4	1	2
[K].vTSTLTIk.[E]	N-Term(iTRAQ4plex); K8(iTRAQ4	1	2
[R].GQPLSPEk.[Y]	K8(iTRAQ4plex)	1	2
[R].gQPLSPEk.[Y]	N-Term(iTRAQ4plex); K8(iTRAQ4	1	2
[K].dVMQGTDEHVVck.[V]	N-Term(iTRAQ4plex); C12(Carbar	1	1
[K].eSGPTTYk.[V]	N-Term(iTRAQ4plex); K8(iTRAQ4	1	2
[K].yAATSQVLLPSk.[D]	N-Term(iTRAQ4plex); K12(iTRAQ	1	1
[R].vFAIPPSFASIFLTk.[S]	N-Term(iTRAQ4plex); K15(iTRAQ	1	2
[K].nVPLPVIAELPPk.[V]	N-Term(iTRAQ4plex); K13(iTRAQ	1	1
[K].VSVFVPPR.[D]		1	2
[K].yVTSAPMPEPQAPGR.[Y]	N-Term(iTRAQ4plex)	1	2
[K].nVPLPVIAELPPk.[V]	N-Term(iTRAQ4plex); K13(iTRAQ	1	1
[K].yAATSQVLLPSk.[D]	N-Term(iTRAQ4plex); K12(iTRAQ	1	1

[K].vTSTLTIk.[E]	N-Term(iTRAQ4plex); K8(iTRAQ4	1	2
[R].fTcTVTHTDLPSPLk.[Q]	N-Term(iTRAQ4plex); C3(Carbam	1	2
[R].fTcTVTHTDLPSPLk.[Q]	N-Term(iTRAQ4plex); C3(Carbam	1	2
[K].vSVFVPPR.[D]	N-Term(iTRAQ4plex)	1	2
[R].GQPLSPEk.[Y]	K8(iTRAQ4plex)	1	2
[K].gVALHRPDVYLLPPAR.[E]	N-Term(iTRAQ4plex)	1	2
[K].nVPLPVIAELPPk.[V]	N-Term(iTRAQ4plex); K13(iTRAQ	1	1
[R].fTcTVTHTDLPSPLk.[Q]	N-Term(iTRAQ4plex); C3(Carbam	1	2
[K].nVPLPVIAELPPk.[V]	N-Term(iTRAQ4plex); K13(iTRAQ	1	1
[R].gQPLSPEk.[Y]	N-Term(iTRAQ4plex); K8(iTRAQ4	1	2
[K].vQHPNGNk.[E]	N-Term(iTRAQ4plex); K8(iTRAQ4	1	1
[K].vSVFVPPR.[D]	N-Term(iTRAQ4plex)	1	2
[K].vTSTLTIk.[E]	N-Term(iTRAQ4plex); K8(iTRAQ4	1	2
[R].eGKQVGSGVTTDQVQAEAk.[E]	N-Term(iTRAQ4plex); K19(iTRAQ	1	1
[R].EGkQVGSGVTTDQVQAEAk.[E]	K3(iTRAQ4plex); K19(iTRAQ4plex	1	1
[K].vTSTLTIk.[E]	N-Term(iTRAQ4plex); K8(iTRAQ4	1	2
[K].qVGSGVTTDQVQAEAk.[E]	N-Term(iTRAQ4plex); K16(iTRAQ	1	1
[K].vSVFVPPR.[D]	N-Term(iTRAQ4plex)	1	2
[R].gQPLSPEk.[Y]	N-Term(iTRAQ4plex); K8(iTRAQ4	1	2
[K].vSVFVPPR.[D]	N-Term(iTRAQ4plex)	1	2
[K].vSVFVPPR.[D]	N-Term(iTRAQ4plex)	1	2
[K].rLPLEYSYGEYR.[D]	N-Term(iTRAQ4plex)	1	1
[K].rLPLEYSYGEYR.[D]	N-Term(iTRAQ4plex)	1	1
[K].sGFSFGFk.[I]	N-Term(iTRAQ4plex); K8(iTRAQ4	1	1
[R].cDcIcPVGSQGLAcEVSYR.[K]	N-Term(iTRAQ4plex); C1(Carbam	1	1
[R].cDcIcPVGSQGLAcEVSYR.[K]	N-Term(iTRAQ4plex); C1(Carbam	1	1
[R].yAYLLQPSQFHGEPcNFSDk.[E]	N-Term(iTRAQ4plex); C15(Carbar	1	1
[R].yAYLLQPSQFHGEPcNFSDk.[E]	N-Term(iTRAQ4plex); C15(Carbar	1	1
[R].gGASEHITTLAYQELPTADLMQEWGDAVC	N-Term(iTRAQ4plex); K36(iTRAQ	1	1
[K].rLPLEYSYGEYR.[D]	N-Term(iTRAQ4plex)	1	1
[K].vEPLYELVTATDFAYSSTVR.[Q]	N-Term(iTRAQ4plex)	1	1
[R].dFGTHYITEAVLGGIYEYTLVMNk.[E]	N-Term(iTRAQ4plex); K24(iTRAQ	1	1
[K].eYESYSDFER.[N]	N-Term(iTRAQ4plex)	1	1
[K].eYESYSDFER.[N]	N-Term(iTRAQ4plex)	1	1
[K].eYESYSDFER.[N]	N-Term(iTRAQ4plex)	1	1
[R].lPLEYSYGEYR.[D]	N-Term(iTRAQ4plex)	1	1
[R].lPLEYSYGEYR.[D]	N-Term(iTRAQ4plex)	1	1
[K].sGFSFGFk.[I]	N-Term(iTRAQ4plex); K8(iTRAQ4	1	1
[R].sDLEVAHYk.[L]	N-Term(iTRAQ4plex); K9(iTRAQ4	1	1
[R].sLMLHYEFLQR.[V]	N-Term(iTRAQ4plex)	1	1
[R].sLMLHYEFLQR.[V]	N-Term(iTRAQ4plex)	1	1
[K].eYESYSDFER.[N]	N-Term(iTRAQ4plex)	1	1
[R].gILNEIk.[D]	N-Term(iTRAQ4plex); K7(iTRAQ4	1	1
[R].gILNEIk.[D]	N-Term(iTRAQ4plex); K7(iTRAQ4	1	1
[K].rLPLEYSYGEYR.[D]	N-Term(iTRAQ4plex)	1	1
[K].iPGIFELGISSQSDR.[G]	N-Term(iTRAQ4plex)	1	1
[K].sGFSFGFk.[I]	N-Term(iTRAQ4plex); K8(iTRAQ4	1	1
[R].kPYNVESYTPQTQGk.[Y]	N-Term(iTRAQ4plex); K1(iTRAQ4	1	1
[R].cEGFVcAQTGR.[C]	N-Term(iTRAQ4plex); C1(Carbam	1	1
[R].kNTPIDGk.[W]	N-Term(iTRAQ4plex); K1(iTRAQ4	1	1
[R].yAYLLQPSQFHGEPcNFSDk.[E]	N-Term(iTRAQ4plex); C15(Carbar	1	1
[R].lPLEYSYGEYR.[D]	N-Term(iTRAQ4plex)	1	1

[R].sLmLHYEFLQR.[V]	N-Term(iTRAQ4plex); M3(Oxidat	1	1
[R].kNTPIDGk.[W]	N-Term(iTRAQ4plex); K1(iTRAQ4	1	1
[K].sGFSFGFk.[I]	N-Term(iTRAQ4plex); K8(iTRAQ4	1	1
[R].gGEGTGYFVDFSVR.[N]	N-Term(iTRAQ4plex)	1	1
[R].aDLFYDVEALDLESPk.[N]	N-Term(iTRAQ4plex); K16(iTRAQ	1	1
[R].aDLFYDVEALDLESPk.[N]	N-Term(iTRAQ4plex); K16(iTRAQ	1	1
[R].sSTTkPPFkPHGSR.[D]	N-Term(iTRAQ4plex); K5(iTRAQ4	1	1
[K].dSPVLIDFFEDTER.[Y]	N-Term(iTRAQ4plex)	1	1
[K].dSPVLIDFFEDTER.[Y]	N-Term(iTRAQ4plex)	1	1
[R].sSTTkPPFkPHGSR.[D]	N-Term(iTRAQ4plex); K5(iTRAQ4	1	1
[R].SSTTkPPFkPHGSR.[D]	K5(iTRAQ4plex); K9(iTRAQ4plex)	1	1
[K].DSPVLIDFFEDTER.[Y]		1	1
[K].dSPVLIDFFEDTER.[Y]	N-Term(iTRAQ4plex)	1	1
[K].dSPVLIDFFEDTER.[Y]	N-Term(iTRAQ4plex)	1	1
[R].aDLFYDVEALDLESPk.[N]	N-Term(iTRAQ4plex); K16(iTRAQ	1	1
[R].aDLFYDVEALDLESPk.[N]	N-Term(iTRAQ4plex); K16(iTRAQ	1	1
[R].aDLFYDVEALDLESPk.[N]	N-Term(iTRAQ4plex); K16(iTRAQ	1	1
[R].ADLFYDVEALDLESPk.[N]	K16(iTRAQ4plex)	1	1
[R].ADLFYDVEALDLESPk.[N]	K16(iTRAQ4plex)	1	1
[R].sSTTkPPFkPHGSR.[D]	N-Term(iTRAQ4plex); K5(iTRAQ4	1	1
[R].rPSEIVIGQck.[V]	N-Term(iTRAQ4plex); C10(Carbar	1	1
[R].aDLFYDVEALDLESPk.[N]	N-Term(iTRAQ4plex); K16(iTRAQ	1	1
[R].hPNVFGFcR.[A]	N-Term(iTRAQ4plex); C8(Carbam	1	1
[R].hPNVFGFcR.[A]	N-Term(iTRAQ4plex); C8(Carbam	1	1
[K].ykEENDDFASFR.[V]	N-Term(iTRAQ4plex); K2(iTRAQ4	1	1
[K].ykEENDDFASFR.[V]	N-Term(iTRAQ4plex); K2(iTRAQ4	1	1
[R].kGEVLPLPEANFPSFPLPHHk.[H]	N-Term(iTRAQ4plex); K1(iTRAQ4	1	1
[R].kGEVLPLPEANFPSFPLPHHk.[H]	N-Term(iTRAQ4plex); K1(iTRAQ4	1	1
[R].kGEVLPLPEANFPSFPLPHHk.[H]	N-Term(iTRAQ4plex); K1(iTRAQ4	1	1
[R].kGEVLPLPEANFPSFPLPHHk.[H]	N-Term(iTRAQ4plex); K1(iTRAQ4	1	1
[R].kGEVLPLPEANFPSFPLPHHk.[H]	N-Term(iTRAQ4plex); K1(iTRAQ4	1	1
[R].kYWNDcEPPDSR.[R]	N-Term(iTRAQ4plex); K1(iTRAQ4	1	1
[R].hSHESQDLR.[V]	N-Term(iTRAQ4plex)	1	1
[R].kYWNDcEPPDSR.[R]	N-Term(iTRAQ4plex); K1(iTRAQ4	1	1
[R].kYWNDcEPPDSR.[R]	N-Term(iTRAQ4plex); K1(iTRAQ4	1	1
[K].sGFPQVSMFFTHTFPk.[-]	N-Term(iTRAQ4plex); K16(iTRAQ	1	1
[K].gEVLPLPEANFPSFPLPHHk.[H]	N-Term(iTRAQ4plex); K20(iTRAQ	1	1
[K].yWNDcEPPDSR.[R]	N-Term(iTRAQ4plex); C5(Carbam	1	1
[K].ykEENDDFASFR.[V]	N-Term(iTRAQ4plex); K2(iTRAQ4	1	1
[R].hSHESQDLR.[V]	N-Term(iTRAQ4plex)	1	1
[R].hSHESQDLR.[V]	N-Term(iTRAQ4plex)	1	1
[R].RPSEIVIGQck.[V]	C10(Carbamidomethyl); K11(iTR	1	1
[R].rPSEIVIGQck.[V]	N-Term(iTRAQ4plex); C10(Carbar	1	1
[R].rPSEIVIGQck.[V]	N-Term(iTRAQ4plex); C10(Carbar	1	1
[R].rPSEIVIGQck.[V]	N-Term(iTRAQ4plex); C10(Carbar	1	1
[K].hPLkPDNQPFPQSVSEScPGk.[F]	N-Term(iTRAQ4plex); K4(iTRAQ4	1	1
[K].hPLkPDNQPFPQSVSEScPGk.[F]	N-Term(iTRAQ4plex); K4(iTRAQ4	1	1
[K].hPLkPDNQPFPQSVSEScPGk.[F]	N-Term(iTRAQ4plex); K4(iTRAQ4	1	1
[R].rDGYLFQLLR.[I]	N-Term(iTRAQ4plex)	1	1
[K].hPLkPDNQPFPQSVSEScPGk.[F]	N-Term(iTRAQ4plex); K4(iTRAQ4	1	1
[R].hPNVFGFcR.[A]	N-Term(iTRAQ4plex); C8(Carbam	1	1
[K].hPLkPDNQPFPQSVSEScPGk.[F]	N-Term(iTRAQ4plex); K4(iTRAQ4	1	1

[K].sGFPQVSMFFTHTFPk.[-]	N-Term(iTRAQ4plex); K16(iTRAQ	1	1
[R].rPSEIVIGQck.[V]	N-Term(iTRAQ4plex); C10(Carbar	1	1
[R].rPSEIVIGQck.[V]	N-Term(iTRAQ4plex); C10(Carbar	1	1
[R].kYWNDcEPPDSR.[R]	N-Term(iTRAQ4plex); K1(iTRAQ4	1	1
[K].sGFPQVSMFFTHTFPk.[-]	N-Term(iTRAQ4plex); K16(iTRAQ	1	1
[R].hPNVFGFcR.[A]	N-Term(iTRAQ4plex); C8(Carbam	1	1
[K].aLDLINk.[R]	N-Term(iTRAQ4plex); K7(iTRAQ4	1	1
[K].aLDLINk.[R]	N-Term(iTRAQ4plex); K7(iTRAQ4	1	1
[K].yWNDcEPPDSR.[R]	N-Term(iTRAQ4plex); C5(Carbam	1	1
[K].yWNDcEPPDSR.[R]	N-Term(iTRAQ4plex); C5(Carbam	1	1
[R].gGEGTGYFVDFSVR.[N]	N-Term(iTRAQ4plex)	1	1
[R].hPNVFGFcR.[A]	N-Term(iTRAQ4plex); C8(Carbam	1	1
[R].HSHESQDLR.[V]		1	1
[K].aLDLINk.[R]	N-Term(iTRAQ4plex); K7(iTRAQ4	1	1
[R].hPNVFGFcR.[A]	N-Term(iTRAQ4plex); C8(Carbam	1	1
[R].kYWNDcEPPDSR.[R]	N-Term(iTRAQ4plex); K1(iTRAQ4	1	1
[K].aLDLINk.[R]	N-Term(iTRAQ4plex); K7(iTRAQ4	1	1
[K].aLDLINk.[R]	N-Term(iTRAQ4plex); K7(iTRAQ4	1	1
[R].HPNVFGFcR.[A]	C8(Carbamidomethyl)	1	1
[R].HSHESQDLR.[V]		1	1
[K].gEVLPLPEANFPSFPLPHHk.[H]	N-Term(iTRAQ4plex); K20(iTRAQ	1	1
[R].hPNVFGFcR.[A]	N-Term(iTRAQ4plex); C8(Carbam	1	1
[R].gGEGTGYFVDFSVR.[N]	N-Term(iTRAQ4plex)	1	1
[R].iADAHLDR.[V]	N-Term(iTRAQ4plex)	1	1
[R].IADAHLDR.[V]		1	1
[K].aLDLINk.[R]	N-Term(iTRAQ4plex); K7(iTRAQ4	1	1
[K].ykEENDDFASFR.[V]	N-Term(iTRAQ4plex); K2(iTRAQ4	1	1
[R].gGEGTGYFVDFSVR.[N]	N-Term(iTRAQ4plex)	1	1
[R].HPNVFGFcR.[A]	C8(Carbamidomethyl)	1	1
[K].aLDLINk.[R]	N-Term(iTRAQ4plex); K7(iTRAQ4	1	1
[R].gGEGTGYFVDFSVR.[N]	N-Term(iTRAQ4plex)	1	1
[R].kYWNDcEPPDSR.[R]	N-Term(iTRAQ4plex); K1(iTRAQ4	1	1
[R].hSHESQDLR.[V]	N-Term(iTRAQ4plex)	1	1
[K].aLDLINk.[R]	N-Term(iTRAQ4plex); K7(iTRAQ4	1	1
[R].iADAHLDR.[V]	N-Term(iTRAQ4plex)	1	1
[R].DTDTGALLFIGk.[I]	K12(iTRAQ4plex)	1	1
[K].tVQAVLTVPk.[L]	N-Term(iTRAQ4plex); K10(iTRAQ	1	1
[K].tVQAVLTVPk.[L]	N-Term(iTRAQ4plex); K10(iTRAQ	1	1
[K].tVQAVLTVPk.[L]	N-Term(iTRAQ4plex); K10(iTRAQ	1	1
[K].lQSLFDSPDFSk.[I]	N-Term(iTRAQ4plex); K12(iTRAQ	1	1
[K].lQSLFDSPDFSk.[I]	N-Term(iTRAQ4plex); K12(iTRAQ	1	1
[K].lQSLFDSPDFSk.[I]	N-Term(iTRAQ4plex); K12(iTRAQ	1	1
[R].aLYYDLISSPDIHGTYk.[E]	N-Term(iTRAQ4plex); K17(iTRAQ	1	1
[K].LQSLFDSPDFSk.[I]	K12(iTRAQ4plex)	1	1
[R].dTDTGALLFIGk.[I]	N-Term(iTRAQ4plex); K12(iTRAQ	1	1
[R].dTDTGALLFIGk.[I]	N-Term(iTRAQ4plex); K12(iTRAQ	1	1
[R].aLYYDLISSPDIHGTYk.[E]	N-Term(iTRAQ4plex); K17(iTRAQ	1	1
[R].aLYYDLISSPDIHGTYk.[E]	N-Term(iTRAQ4plex); K17(iTRAQ	1	1
[R].aLYYDLISSPDIHGTYk.[E]	N-Term(iTRAQ4plex); K17(iTRAQ	1	1
[K].tSLEDFYLDEER.[T]	N-Term(iTRAQ4plex)	1	1
[K].tSLEDFYLDEER.[T]	N-Term(iTRAQ4plex)	1	1
[K].tSLEDFYLDEER.[T]	N-Term(iTRAQ4plex)	1	1

[K].lSYEGEVTk.[S]	N-Term(iTRAQ4plex); K9(iTRAQ4	1	1
[K].lAAAVSNFGYDLYR.[V]	N-Term(iTRAQ4plex)	1	1
[K].iAQLPLTGSMSIIFFLPLk.[V]	N-Term(iTRAQ4plex); K19(iTRAQ	1	1
[K].eIPDEISILLLGVAHFk.[G]	N-Term(iTRAQ4plex); K17(iTRAQ	1	1
[R].sSTSPTTNVLLSPLSVATALSALSLGAEQR.[T	N-Term(iTRAQ4plex)	1	1
[K].eLLDTVTAPQk.[N]	N-Term(iTRAQ4plex); K11(iTRAQ	1	1
[R].kTSLEDFYLDEER.[T]	N-Term(iTRAQ4plex); K1(iTRAQ4	1	1
[R].kTSLEDFYLDEER.[T]	N-Term(iTRAQ4plex); K1(iTRAQ4	1	1
[K].lSYEGEVTk.[S]	N-Term(iTRAQ4plex); K9(iTRAQ4	1	1
[R].lDLQEINNWVQAQMk.[G]	N-Term(iTRAQ4plex); K15(iTRAQ	1	1
[K].TSLEDFYLDEER.[T]		1	1
[K].sSFVAPLEk.[S]	N-Term(iTRAQ4plex); K9(iTRAQ4	1	1
[R].dTDTGALLFIGk.[I]	N-Term(iTRAQ4plex); K12(iTRAQ	1	1
[K].lAAAVSNFGYDLYR.[V]	N-Term(iTRAQ4plex)	1	1
[K].sSFVAPLEk.[S]	N-Term(iTRAQ4plex); K9(iTRAQ4	1	1
[K].iTGkPIk.[L]	N-Term(iTRAQ4plex); K4(iTRAQ4	1	1
[R].yGLDSDLSck.[I]	N-Term(iTRAQ4plex); C9(Carbam	1	1
[K].tSLEDFYLDEER.[T]	N-Term(iTRAQ4plex)	1	1
[K].iTGkPIk.[L]	N-Term(iTRAQ4plex); K4(iTRAQ4	1	1
[R].DTDTGALLFIGk.[I]	K12(iTRAQ4plex)	1	1
[R].lDLQEINNWVQAQmKGK.[L]	N-Term(iTRAQ4plex); M14(Oxida	1	1
[R].kTSLEDFYLDEER.[T]	N-Term(iTRAQ4plex)	1	1
[R].kTSLEDFYLDEER.[T]	K1(iTRAQ4plex)	1	1
[K].iTGkPIk.[L]	N-Term(iTRAQ4plex); K4(iTRAQ4	1	1
[K].lSYEGEVTk.[S]	N-Term(iTRAQ4plex); K9(iTRAQ4	1	1
[R].dTDTGALLFIGk.[I]	N-Term(iTRAQ4plex); K12(iTRAQ	1	1
[K].sSFVAPLEk.[S]	N-Term(iTRAQ4plex); K9(iTRAQ4	1	1
[K].lQSLFDSPDFSk.[I]	N-Term(iTRAQ4plex); K12(iTRAQ	1	1
[K].tVQAVLTVPk.[L]	N-Term(iTRAQ4plex); K10(iTRAQ	1	1
[R].iRPHTFTGLSGLR.[R]	N-Term(iTRAQ4plex)	1	1
[R].iRPHTFTGLSGLR.[R]	N-Term(iTRAQ4plex)	1	1
[R].iRPHTFTGLSGLR.[R]	N-Term(iTRAQ4plex)	1	1
[R].lSHNAIASLRPR.[T]	N-Term(iTRAQ4plex)	1	1
[K].aNVFVQLPR.[L]	N-Term(iTRAQ4plex)	1	1
[R].aFWLDVSHNR.[L]	N-Term(iTRAQ4plex)	1	1
[R].aFWLDVSHNR.[L]	N-Term(iTRAQ4plex)	1	1
[R].lWLEGNPWDcGcPLk.[A]	N-Term(iTRAQ4plex); C10(Carbar	1	1
[K].ANVFVQLPR.[L]		1	1
[R].AFWLDVSHNR.[L]		1	1
[R].LSHNAIASLRPR.[T]		1	1
[K].lHSLHLEGScLGR.[I]	N-Term(iTRAQ4plex); C10(Carbar	1	1
[R].wLDLSHNR.[V]	N-Term(iTRAQ4plex)	1	1
[R].wLDLSHNR.[V]	N-Term(iTRAQ4plex)	1	1
[R].sFEGLGQLEVLTLDHNQLQEVk.[A]	N-Term(iTRAQ4plex); K22(iTRAQ	1	1
[R].sFEGLGQLEVLTLDHNQLQEVk.[A]	N-Term(iTRAQ4plex); K22(iTRAQ	1	1
[R].lAELPADALGPLQR.[A]	N-Term(iTRAQ4plex)	1	1
[R].lAELPADALGPLQR.[A]	N-Term(iTRAQ4plex)	1	1
[R].sFEGLGQLEVLTLDHNQLQEVk.[A]	N-Term(iTRAQ4plex); K22(iTRAQ	1	1
[R].LAELPADALGPLQR.[A]		1	1
[K].dLHFLEELQLGHNR.[I]	N-Term(iTRAQ4plex)	1	1
[R].nLIAAVAPGAFLGLk.[A]	N-Term(iTRAQ4plex); K15(iTRAQ	1	1
[R].sLALGTFAHTPALASLGLSNNR.[L]	N-Term(iTRAQ4plex)	1	1

[R].vAGLLEDTFPGLLGLR.[V]	N-Term(iTRAQ4plex)	1	1
[R].vAGLLEDTFPGLLGLR.[V]	N-Term(iTRAQ4plex)	1	1
[R].tFTPQPPGLER.[L]	N-Term(iTRAQ4plex)	1	1
[R].lAYLQPALFSGLAELR.[E]	N-Term(iTRAQ4plex)	1	1
[K].lHSLHLEGScLGR.[I]	N-Term(iTRAQ4plex); C10(Carbar	1	1
[K].LEYLLLSR.[N]		1	1
[R].lWLEGNPWDcGcPLk.[A]	N-Term(iTRAQ4plex); C10(Carbar	1	1
[R].sFEGLGQLEVLTLDHNQLQEVk.[A]	N-Term(iTRAQ4plex); K22(iTRAQ	1	1
[R].sFEGLGQLEVLTLDHNQLQEVk.[A]	N-Term(iTRAQ4plex); K22(iTRAQ	1	1
[K].aNVFVQLPR.[L]	N-Term(iTRAQ4plex)	1	1
[K].aNVFVQLPR.[L]	N-Term(iTRAQ4plex)	1	1
[R].TFTPQPPGLER.[L]		1	1
[R].iRPHTFTGLSGLR.[R]	N-Term(iTRAQ4plex)	1	1
[R].lFQGLGk.[L]	N-Term(iTRAQ4plex); K7(iTRAQ4	1	1
[R].tFTPQPPGLER.[L]	N-Term(iTRAQ4plex)	1	1
[R].aFWLDVSHNR.[L]	N-Term(iTRAQ4plex)	1	1
[K].lHSLHLEGScLGR.[I]	N-Term(iTRAQ4plex); C10(Carbar	1	1
[R].sFEGLGQLEVLTLDHNQLQEVk.[A]	N-Term(iTRAQ4plex); K22(iTRAQ	1	1
[R].tFTPQPPGLER.[L]	N-Term(iTRAQ4plex)	1	1
[R].dVVQITcLDGFEVVEGR.[V]	N-Term(iTRAQ4plex); C7(Carbam	1	1
[R].dVVQITcLDGFEVVEGR.[V]	N-Term(iTRAQ4plex); C7(Carbam	1	1
[R].rEDFDVEAADSAGNcLDSLVFVAGDR.[Q]	N-Term(iTRAQ4plex); C15(Carbar	1	1
[R].rEDFDVEAADSAGNcLDSLVFVAGDR.[Q]	N-Term(iTRAQ4plex); C15(Carbar	1	1
[R].dVVQITcLDGFEVVEGR.[V]	N-Term(iTRAQ4plex); C7(Carbam	1	1
[R].dVVQITcLDGFEVVEGR.[V]	N-Term(iTRAQ4plex); C7(Carbam	1	1
[R].rEDFDVEAADSAGNcLDSLVFVAGDR.[Q]	N-Term(iTRAQ4plex); C15(Carbar	1	1
[K].sNALDIIFQTDLTGQk.[K]	N-Term(iTRAQ4plex); K16(iTRAQ	1	1
[K].sNALDIIFQTDLTGQk.[K]	N-Term(iTRAQ4plex); K16(iTRAQ	1	1
[R].qFGPYcGHGFPGPLNIETk.[S]	N-Term(iTRAQ4plex); C6(Carbam	1	1
[K].mLTPEHVFIHPGWk.[L]	N-Term(iTRAQ4plex); M1(Oxidat	1	1
[K].vEDPESTLFGSVIR.[Y]	N-Term(iTRAQ4plex)	1	1
[K].nYVDWIMk.[T]	N-Term(iTRAQ4plex); K8(iTRAQ4	1	1
[K].sNALDIIFQTDLTGQk.[K]	N-Term(iTRAQ4plex); K16(iTRAQ	1	1
[K].eDTPNSVWEPAk.[A]	N-Term(iTRAQ4plex); K12(iTRAQ	1	1
[K].gDSGGAFAVQDPNDk.[T]	N-Term(iTRAQ4plex); K15(iTRAQ	1	1
[R].ePTMYVGSTSVQTSR.[L]	N-Term(iTRAQ4plex)	1	1
[R].iIGGSDADIk.[N]	N-Term(iTRAQ4plex); K10(iTRAQ	1	1
[K].gFQVVVTLR.[R]	N-Term(iTRAQ4plex)	1	1
[K].mLTPEHVFIHPGWk.[L]	N-Term(iTRAQ4plex); K14(iTRAQ	1	1
[R].tNFDNDIALVR.[L]	N-Term(iTRAQ4plex)	1	1
[R].TNFDNDIALVR.[L]		1	1
[R].sSNNPHSPIVEEFQVPYNk.[L]	N-Term(iTRAQ4plex); K19(iTRAQ	1	1
[R].yHGDPMPcPk.[E]	N-Term(iTRAQ4plex); C8(Carbam	1	1
[K].gFQVVVTLR.[R]	N-Term(iTRAQ4plex)	1	1
[R].qFGPYcGHGFPGPLNIETk.[S]	N-Term(iTRAQ4plex); C6(Carbam	1	1
[K].gDSGGAFAVQDPNDk.[T]	N-Term(iTRAQ4plex); K15(iTRAQ	1	1
[K].sNALDIIFQTDLTGQk.[K]	N-Term(iTRAQ4plex); K16(iTRAQ	1	1
[RK].sDFSNEER.[F]	N-Term(iTRAQ4plex)	2	2
[R].sSNNPHSPIVEEFQVPYNk.[L]	N-Term(iTRAQ4plex); K19(iTRAQ	1	1
[K].gDSGGAFAVQDPNDk.[T]	N-Term(iTRAQ4plex); K15(iTRAQ	1	1
[K].gFQVVVTLR.[R]	N-Term(iTRAQ4plex)	1	1
[K].gDSGGAFAVQDPNDk.[T]	N-Term(iTRAQ4plex); K15(iTRAQ	1	1

[R].dVVQITcLDGFEVVEGR.[V]	N-Term(iTRAQ4plex); C7(Carbam	1	1
[R].iIGGSDADIk.[N]	N-Term(iTRAQ4plex); K10(iTRAQ	1	1
[K].mLTPEHVFIHPGWk.[L]	N-Term(iTRAQ4plex); M1(Oxidat	1	1
[K].mLTPEHVFIHPGWk.[L]	N-Term(iTRAQ4plex); K14(iTRAQ	1	1
[K].lLEVPEGR.[T]	N-Term(iTRAQ4plex)	1	1
[R].YHGDPMPcPk.[E]	C8(Carbamidomethyl); K10(iTRA	1	1
[R].TNFDNDIALVR.[L]		1	1
[K].gDSGGAFAVQDPNDk.[T]	N-Term(iTRAQ4plex); K15(iTRAQ	1	1
[K].eDTPNSVWEPAk.[A]	N-Term(iTRAQ4plex); K12(iTRAQ	1	1
[R].qFGPYcGHGFPGPLNIETk.[S]	N-Term(iTRAQ4plex); C6(Carbam	1	1
[K].gFQVVVTLR.[R]	N-Term(iTRAQ4plex)	1	1
[R].lPVAPLRk.[C]	N-Term(iTRAQ4plex); K8(iTRAQ4	1	1
[K].lLEVPEGR.[T]	N-Term(iTRAQ4plex)	1	1
[K].mLTPEHVFIHPGWk.[L]	N-Term(iTRAQ4plex); K14(iTRAQ	1	1
[R].eANVAcLDLGFQQGADTQR.[R]	N-Term(iTRAQ4plex); C6(Carbam	1	1
[R].gLETSLAEcTFTk.[R]	N-Term(iTRAQ4plex); C9(Carbam	1	1
[R].gLETSLAEcTFTk.[R]	N-Term(iTRAQ4plex); C9(Carbam	1	1
[R].gLETSLAEcTFTk.[R]	N-Term(iTRAQ4plex); C9(Carbam	1	1
[R].eANVAcLDLGFQQGADTQR.[R]	N-Term(iTRAQ4plex); C6(Carbam	1	1
[R].eANVAcLDLGFQQGADTQR.[R]	N-Term(iTRAQ4plex); C6(Carbam	1	1
[R].iIFHENYNAGTYQNDIALIEMk.[K]	N-Term(iTRAQ4plex); K22(iTRAQ	1	1
[R].tmGYQDFADVVcYTQk.[A]	N-Term(iTRAQ4plex); M2(Oxidat	1	1
[R].tMGYQDFADVVcYTQk.[A]	N-Term(iTRAQ4plex); C12(Carbar	1	1
[K].hGNTDSEGIVEVk.[L]	N-Term(iTRAQ4plex); K13(iTRAQ	1	1
[K].hGNTDSEGIVEVk.[L]	N-Term(iTRAQ4plex); K13(iTRAQ	1	1
[K].kYTHLScDk.[V]	N-Term(iTRAQ4plex); K1(iTRAQ4	1	1
[R].vFSLQWGEVk.[L]	N-Term(iTRAQ4plex); K10(iTRAQ	1	1
[K].yTHLScDk.[V]	N-Term(iTRAQ4plex); C6(Carbam	1	1
[K].emEcAGTYDGSIDAck.[G]	N-Term(iTRAQ4plex); M2(Oxidat	1	1
[K].eMEcAGTYDGSIDAck.[G]	N-Term(iTRAQ4plex); C4(Carbam	1	1
[K].eMEcAGTYDGSIDAck.[G]	N-Term(iTRAQ4plex); C4(Carbam	1	1
[K].eMEcAGTYDGSIDAck.[G]	N-Term(iTRAQ4plex); C4(Carbam	1	1
[K].eMEcAGTYDGSIDAck.[G]	N-Term(iTRAQ4plex); C4(Carbam	1	1
[K].acDGINDcGDQSDELcck.[A]	N-Term(iTRAQ4plex); C2(Carbam	1	1
[K].acDGINDcGDQSDELcck.[A]	N-Term(iTRAQ4plex); C2(Carbam	1	1
[K].acDGINDcGDQSDELcck.[A]	N-Term(iTRAQ4plex); C2(Carbam	1	1
[K].acDGINDcGDQSDELcck.[A]	N-Term(iTRAQ4plex); C2(Carbam	1	1
[R].yQIWTTVVDWIHPDLk.[R]	N-Term(iTRAQ4plex); K16(iTRAQ	1	1
[K].acDGINDcGDQSDELcck.[A]	N-Term(iTRAQ4plex); C2(Carbam	1	1
[K].AcDGINDcGDQSDELcck.[A]	C2(Carbamidomethyl); C8(Carba	1	1
[K].acDGINDcGDQSDELcck.[A]	N-Term(iTRAQ4plex); C2(Carbam	1	1
[K].acDGINDcGDQSDELcck.[A]	N-Term(iTRAQ4plex); C2(Carbam	1	1
[K].acDGINDcGDQSDELcck.[A]	N-Term(iTRAQ4plex); C2(Carbam	1	1
[K].acDGINDcGDQSDELcck.[A]	N-Term(iTRAQ4plex); C2(Carbam	1	1
[K].sLEcLHPGTk.[F]	N-Term(iTRAQ4plex); C4(Carbam	1	1
[R].aQLGDLPWQVAIk.[D]	N-Term(iTRAQ4plex); K13(iTRAQ	1	1
[K].rAQLGDLPWQVAIk.[D]	N-Term(iTRAQ4plex); K14(iTRAQ	1	1
[K].sLEcLHPGTk.[F]	N-Term(iTRAQ4plex); C4(Carbam	1	1
[R].vFSLQWGEVk.[L]	N-Term(iTRAQ4plex); K10(iTRAQ	1	1
[R].cIEGTcVck.[L]	N-Term(iTRAQ4plex); C1(Carbam	1	1
[R].vFSLQWGEVk.[L]	N-Term(iTRAQ4plex); K10(iTRAQ	1	1
[R].iVIEYVDR.[I]	N-Term(iTRAQ4plex)	1	1

[K].acDGINDcGDQSDELcck.[A]	N-Term(iTRAQ4plex); C2(Carbam	1	1
[R].aQLGDLPWQVAIk.[D]	N-Term(iTRAQ4plex); K13(iTRAQ	1	1
[K].kYTHLScDk.[V]	N-Term(iTRAQ4plex); K1(iTRAQ4	1	1
[K].AcDGINDcGDQSDELcck.[A]	C2(Carbamidomethyl); C8(Carba	1	1
[K].vFcQPWQR.[C]	N-Term(iTRAQ4plex); C3(Carbam	1	1
[R].AQLGDLPWQVAIk.[D]	K13(iTRAQ4plex)	1	1
[R].tMGYQDFADVVcYTQk.[A]	N-Term(iTRAQ4plex); C12(Carbai	1	1
[R].iVIEYVDR.[I]	N-Term(iTRAQ4plex)	1	1
[K].kLELHLPk.[F]	N-Term(iTRAQ4plex); K1(iTRAQ4	1	1
[R].eIEEVLTPEMLMR.[W]	N-Term(iTRAQ4plex)	1	1
[K].aTLDVDEAGTEAAAATSFAIk.[F]	N-Term(iTRAQ4plex); K21(iTRAQ	1	1
[R].eIEEVLTPEMLMR.[W]	N-Term(iTRAQ4plex)	1	1
[R].vGSALFLSHNLk.[F]	N-Term(iTRAQ4plex); K12(iTRAQ	1	1
[R].vGSALFLSHNLk.[F]	N-Term(iTRAQ4plex); K12(iTRAQ	1	1
[R].vGSALFLSHNLk.[F]	N-Term(iTRAQ4plex); K12(iTRAQ	1	1
[K].iAPANADFAFR.[F]	N-Term(iTRAQ4plex)	1	1
[K].wADLSGITk.[Q]	N-Term(iTRAQ4plex); K9(iTRAQ4	1	1
[K].iVDLVSELk.[K]	N-Term(iTRAQ4plex); K9(iTRAQ4	1	1
[R].lGFTDLFSk.[W]	N-Term(iTRAQ4plex); K9(iTRAQ4	1	1
[K].LFHTNFYDTVGTIQLINDHVk.[K]	K21(iTRAQ4plex)	1	1
[K].lFHTNFYDTVGTIQLINDHVk.[K]	N-Term(iTRAQ4plex); K21(iTRAQ	1	1
[K].gDATVFFILPNQGk.[M]	N-Term(iTRAQ4plex); K14(iTRAQ	1	1
[K].fSISGSYVLDQILPR.[L]	N-Term(iTRAQ4plex)	1	1
[R].gFQHLLHTLNLPGHGLETR.[V]	N-Term(iTRAQ4plex)	1	1
[R].gFQHLLHTLNLPGHGLETR.[V]	N-Term(iTRAQ4plex)	1	1
[K].aLWEkPFISSR.[T]	N-Term(iTRAQ4plex); K5(iTRAQ4	1	1
[R].vGSALFLSHNLk.[F]	N-Term(iTRAQ4plex); K12(iTRAQ	1	1
[R].gFQHLLHTLNLPGHGLETR.[V]	N-Term(iTRAQ4plex)	1	1
[R].eIEEVLTPEMLMR.[W]	N-Term(iTRAQ4plex)	1	1
[R].fYYLIASETPGk.[N]	N-Term(iTRAQ4plex); K12(iTRAQ	1	1
[K].aTLDVDEAGTEAAAATSFAIk.[F]	N-Term(iTRAQ4plex); K21(iTRAQ	1	1
[K].aLWEkPFISSR.[T]	N-Term(iTRAQ4plex); K5(iTRAQ4	1	1
[K].wADLSGITk.[Q]	N-Term(iTRAQ4plex); K9(iTRAQ4	1	1
[R].vPMMLQDQEHHWYLHDR.[Y]	N-Term(iTRAQ4plex)	1	1
[K].lELHLPk.[F]	N-Term(iTRAQ4plex); K7(iTRAQ4	1	1
[K].fFSAQTNR.[H]	N-Term(iTRAQ4plex)	1	1
[R].eIEEVLTPEMLMR.[W]	N-Term(iTRAQ4plex)	1	1
[K].iAPANADFAFR.[F]	N-Term(iTRAQ4plex)	1	1
[R].FYYLIASETPGk.[N]	K12(iTRAQ4plex)	1	1
[K].FFSAQTNR.[H]		1	1
[K].lELHLPk.[F]	N-Term(iTRAQ4plex); K7(iTRAQ4	1	1
[K].wADLSGITk.[Q]	N-Term(iTRAQ4plex); K9(iTRAQ4	1	1
[K].lELHLPk.[F]	N-Term(iTRAQ4plex); K7(iTRAQ4	1	1
[K].FFSAQTNR.[H]		1	1
[R].kVESELIkPINPR.[L]	N-Term(iTRAQ4plex); K1(iTRAQ4	1	1
[R].kVESELIkPINPR.[L]	N-Term(iTRAQ4plex); K1(iTRAQ4	1	1
[R].kVESELIkPINPR.[L]	N-Term(iTRAQ4plex); K1(iTRAQ4	1	1
[R].kVESELIkPINPR.[L]	N-Term(iTRAQ4plex); K1(iTRAQ4	1	1
[K].aSFTcTckPGWQGEk.[C]	N-Term(iTRAQ4plex); C5(Carbam	1	1
[K].aSFTcTckPGWQGEk.[C]	N-Term(iTRAQ4plex); C5(Carbam	1	1
[R].sFQTGLFTAAR.[Q]	N-Term(iTRAQ4plex)	1	1
[R].iQALSLcSDQQSHLEFR.[V]	N-Term(iTRAQ4plex); C7(Carbam	1	1

[R].iQALSLcSDQQSHLEFR.[V]	N-Term(iTRAQ4plex); C7(Carbam	1	1
[K].scEVVSVcLPLNLDTk.[Y]	N-Term(iTRAQ4plex); C2(Carbam	1	1
[K].nIPGDFEcEcPEGYR.[Y]	N-Term(iTRAQ4plex); C8(Carbam	1	1
[K].nIPGDFEcEcPEGYR.[Y]	N-Term(iTRAQ4plex); C8(Carbam	1	1
[K].iETISHEDLQR.[Q]	N-Term(iTRAQ4plex)	1	1
[K].hcLVTVEk.[G]	N-Term(iTRAQ4plex); C2(Carbam	1	1
[R].kVESELIkPINPR.[L]	N-Term(iTRAQ4plex); K1(iTRAQ4	1	1
[K].dVDEcSLkPSIcGTAVck.[N]	N-Term(iTRAQ4plex); C5(Carbam	1	1
[R].NNLELSTPLk.[I]	K10(iTRAQ4plex)	1	1
[K].nIPGDFEcEcPEGYR.[Y]	N-Term(iTRAQ4plex); C8(Carbam	1	1
[R].aHScPSVWk.[K]	N-Term(iTRAQ4plex); C4(Carbam	1	1
[K].aSFTcTckPGWQGEk.[C]	N-Term(iTRAQ4plex); C5(Carbam	1	1
[R].fSAEFDFR.[T]	N-Term(iTRAQ4plex)	1	1
[R].kVESELIkPINPR.[L]	K1(iTRAQ4plex); K8(iTRAQ4plex)	1	1
[R].kVESELIkPINPR.[L]	N-Term(iTRAQ4plex); K8(iTRAQ4	1	1
[K].vYFAGFPR.[K]	N-Term(iTRAQ4plex)	1	1
[R].qSTNAYPDLR.[S]	N-Term(iTRAQ4plex)	1	1
[R].qLAVLDk.[A]	N-Term(iTRAQ4plex); K7(iTRAQ4	1	1
[K].IETISHEDLQR.[Q]		1	1
[K].sQDILLSVENTVIYR.[I]	N-Term(iTRAQ4plex)	1	1
[R].scVNAIPDQcSPLPcNEDGYMSck.[D]	N-Term(iTRAQ4plex); C2(Carbam	1	1
[R].qSTNAYPDLR.[S]	N-Term(iTRAQ4plex)	1	1
[R].nNLELSTPLk.[I]	N-Term(iTRAQ4plex); K10(iTRAQ	1	1
[K].hcLVTVEk.[G]	N-Term(iTRAQ4plex); C2(Carbam	1	1
[K].nIPGDFEcEcPEGYR.[Y]	N-Term(iTRAQ4plex); C8(Carbam	1	1
[K].sQDILLSVENTVIYR.[I]	N-Term(iTRAQ4plex)	1	1
[K].sQDILLSVENTVIYR.[I]	N-Term(iTRAQ4plex)	1	1
[R].kVESELIkPINPR.[L]	N-Term(iTRAQ4plex); K1(iTRAQ4	1	1
[R].aHScPSVWk.[K]	N-Term(iTRAQ4plex); C4(Carbam	1	1
[R].dYIFGNYIER.[L]	N-Term(iTRAQ4plex)	1	1
[R].dYIFGNYIER.[L]	N-Term(iTRAQ4plex)	1	1
[R].aLDLSLk.[Y]	N-Term(iTRAQ4plex); K7(iTRAQ4	1	1
[K].gMTNINDGLLR.[G]	N-Term(iTRAQ4plex)	1	1
[K].gMTNINDGLLR.[G]	N-Term(iTRAQ4plex)	1	1
[K].gHVSFkPSLDQQR.[S]	N-Term(iTRAQ4plex); K6(iTRAQ4	1	1
[K].kGHVSFkPSLDQQR.[S]	N-Term(iTRAQ4plex); K1(iTRAQ4	1	1
[K].kGHVSFkPSLDQQR.[S]	N-Term(iTRAQ4plex); K1(iTRAQ4	1	1
[K].kGHVSFkPSLDQQR.[S]	N-Term(iTRAQ4plex); K1(iTRAQ4	1	1
[R].sTSIVIMLTDGDANVGESRPEk.[I]	N-Term(iTRAQ4plex); K22(iTRAQ	1	1
[R].sTSIVIMLTDGDANVGESRPEk.[I]	N-Term(iTRAQ4plex); K22(iTRAQ	1	1
[R].sTSIVIMLTDGDANVGESRPEk.[I]	N-Term(iTRAQ4plex); K22(iTRAQ	1	1
[K].eHLVQATPENLQEAR.[T]	N-Term(iTRAQ4plex)	1	1
[K].eHLVQATPENLQEAR.[T]	N-Term(iTRAQ4plex)	1	1
[K].fPLYNLGFGNNLNYNFLENMALENHGFAR	N-Term(iTRAQ4plex)	1	1
[K].kGHVSFkPSLDQQR.[S]	N-Term(iTRAQ4plex); K1(iTRAQ4	1	1
[K].GMTNINDGLLR.[G]		1	1
[K].EHLVQATPENLQEAR.[T]		1	1
[R].dFLGFYVVDSHR.[M]	N-Term(iTRAQ4plex)	1	1
[R].lVDEDmNSFk.[A]	N-Term(iTRAQ4plex); M6(Oxidat	1	1
[K].nHQLIVTR.[G]	N-Term(iTRAQ4plex)	1	1
[K].vSDIRPGSDPTkPDATLVVk.[N]	N-Term(iTRAQ4plex); K12(iTRAQ	1	1
[R].gISMLNk.[A]	N-Term(iTRAQ4plex); K7(iTRAQ4	1	1

[K].kGHVSFkPSLDQQR.[S]	K1(iTRAQ4plex); K7(iTRAQ4plex)	1	1
[K].kGHVSFkPSLDQQR.[S]	N-Term(iTRAQ4plex); K7(iTRAQ4	1	1
[R].aLDLSLk.[Y]	N-Term(iTRAQ4plex); K7(iTRAQ4	1	1
[R].fAHNVVTMR.[A]	N-Term(iTRAQ4plex)	1	1
[R].fAHNVVTmR.[A]	N-Term(iTRAQ4plex); M8(Oxidat	1	1
[R].fAHNVVTMR.[A]	N-Term(iTRAQ4plex)	1	1
[K].dASIGTk.[V]	N-Term(iTRAQ4plex); K7(iTRAQ4	1	1
[R].fAHNVVTMR.[A]	N-Term(iTRAQ4plex)	1	1
[R].fAHNVVTMR.[A]	N-Term(iTRAQ4plex)	1	1
[K].nHQLIVTR.[G]	N-Term(iTRAQ4plex)	1	1
[R].ALDLSLk.[Y]	K7(iTRAQ4plex)	1	1
[R].dYIFGNYIER.[L]	N-Term(iTRAQ4plex)	1	1
[R].kDASIGTk.[V]	N-Term(iTRAQ4plex); K1(iTRAQ4	1	1
[R].gISMLNk.[A]	N-Term(iTRAQ4plex); K7(iTRAQ4	1	1
[K].yHFVTPLTSMVVTkPEDNEDER.[A]	N-Term(iTRAQ4plex); K14(iTRAQ	1	1
[K].yHFVTPLTSMVVTkPEDNEDER.[A]	N-Term(iTRAQ4plex); K14(iTRAQ	1	1
[K].nYNLVESLk.[L]	N-Term(iTRAQ4plex); K9(iTRAQ4	1	1
[K].nYNLVESLk.[L]	N-Term(iTRAQ4plex); K9(iTRAQ4	1	1
[K].fPVEmTHNHNFR.[L]	N-Term(iTRAQ4plex); M5(Oxidat	1	1
[K].fPVEMTHNHNFR.[L]	N-Term(iTRAQ4plex)	1	1
[K].gETHEQVHSILHFk.[D]	N-Term(iTRAQ4plex); K14(iTRAQ	1	1
[K].gETHEQVHSILHFk.[D]	N-Term(iTRAQ4plex); K14(iTRAQ	1	1
[K].gETHEQVHSILHFk.[D]	N-Term(iTRAQ4plex); K14(iTRAQ	1	1
[K].gETHEQVHSILHFk.[D]	N-Term(iTRAQ4plex); K14(iTRAQ	1	1
[K].gETHEQVHSILHFk.[D]	N-Term(iTRAQ4plex); K14(iTRAQ	1	1
[K].gETHEQVHSILHFk.[D]	N-Term(iTRAQ4plex); K14(iTRAQ	1	1
[K].gETHEQVHSILHFk.[D]	N-Term(iTRAQ4plex); K14(iTRAQ	1	1
[K].gETHEQVHSILHFk.[D]	N-Term(iTRAQ4plex); K14(iTRAQ	1	1
[K].gETHEQVHSILHFk.[D]	N-Term(iTRAQ4plex); K14(iTRAQ	1	1
[K].gETHEQVHSILHFk.[D]	N-Term(iTRAQ4plex); K14(iTRAQ	1	1
[R].tScLLFMGR.[V]	N-Term(iTRAQ4plex); C3(Carbam	1	1
[R].sVNDLYIQk.[Q]	N-Term(iTRAQ4plex); K9(iTRAQ4	1	1
[R].eYYFAEAQIADFSDPAFISk.[T]	N-Term(iTRAQ4plex); K20(iTRAQ	1	1
[K].fPVEMTHNHNFR.[L]	N-Term(iTRAQ4plex)	1	1
[K].fPVEMTHNHNFR.[L]	N-Term(iTRAQ4plex)	1	1
[K].fPVEMTHNHNFR.[L]	N-Term(iTRAQ4plex)	1	1
[K].gETHEQVHSILHFk.[D]	N-Term(iTRAQ4plex); K14(iTRAQ	1	1
[K].gGETAQSADPQWEQLNNk.[N]	N-Term(iTRAQ4plex); K18(iTRAQ	1	1
[K].gPLDQLEk.[G]	N-Term(iTRAQ4plex); K8(iTRAQ4	1	1
[R].fTVDRPFLFLIYEHR.[T]	N-Term(iTRAQ4plex)	1	1
[K].qFPILLDFk.[T]	N-Term(iTRAQ4plex); K9(iTRAQ4	1	1
[K].TLEAQLTPR.[V]		1	1
[R].sVNDLYIQk.[Q]	N-Term(iTRAQ4plex); K9(iTRAQ4	1	1
[K].yEITTIHNLFR.[K]	N-Term(iTRAQ4plex)	1	1
[K].tLEAQLTPR.[V]	N-Term(iTRAQ4plex)	1	1
[K].tLEAQLTPR.[V]	N-Term(iTRAQ4plex)	1	1
[K].NYNLVESLk.[L]	K9(iTRAQ4plex)	1	1
[K].YEITTIHNLFR.[K]		1	1
[K].yEITTIHNLFR.[K]	N-Term(iTRAQ4plex)	1	1
[K].GPLDQLEk.[G]	K8(iTRAQ4plex)	1	1
[R].fTVDRPFLFLIYEHR.[T]	N-Term(iTRAQ4plex)	1	1
[R].rNFGYTLR.[S]	N-Term(iTRAQ4plex)	1	1

[R].iAIDLFk.[H]	N-Term(iTRAQ4plex); K7(iTRAQ4	1	1
[K].qFPILLDFk.[T]	N-Term(iTRAQ4plex); K9(iTRAQ4	1	1
[K].nYNLVESLk.[L]	N-Term(iTRAQ4plex); K9(iTRAQ4	1	1
[K].fPVEMTHNHNFR.[L]	N-Term(iTRAQ4plex)	1	1
[K].TLEAQLTPR.[V]		1	1
[K].fPVEmTHNHNFR.[L]	N-Term(iTRAQ4plex); M5(Oxidat	1	1
[K].GGETAQSADPQWEQLNNk.[N]	K18(iTRAQ4plex)	1	1
[R].TScLLFMGR.[V]	C3(Carbamidomethyl)	1	1
[R].iAIDLFk.[H]	N-Term(iTRAQ4plex); K7(iTRAQ4	1	1
[K].tLEAQLTPR.[V]	N-Term(iTRAQ4plex)	1	1
[K].fPVEMTHNHNFR.[L]	N-Term(iTRAQ4plex)	1	1
[K].gPLDQLEk.[G]	N-Term(iTRAQ4plex); K8(iTRAQ4	1	1
[R].EYYFAEAQIADFSDPAFISk.[T]	K20(iTRAQ4plex)	1	1
[R].iAIDLFk.[H]	N-Term(iTRAQ4plex); K7(iTRAQ4	1	1
[K].nYNLVESLk.[L]	N-Term(iTRAQ4plex); K9(iTRAQ4	1	1
[K].qFPILLDFk.[T]	N-Term(iTRAQ4plex); K9(iTRAQ4	1	1
[K].QFPILLDFk.[T]	K9(iTRAQ4plex)	1	1
[K].gETHEQVHSILHFk.[D]	N-Term(iTRAQ4plex); K14(iTRAQ	1	1
[R].IAIDLFk.[H]	K7(iTRAQ4plex)	1	1
[K].tLEAQLTPR.[V]	N-Term(iTRAQ4plex)	1	1
[K].nGNMAGISDQR.[I]	N-Term(iTRAQ4plex)	1	1
[R].sVNDLYIQk.[Q]	N-Term(iTRAQ4plex); K9(iTRAQ4	1	1
[K].vSMMQTk.[G]	N-Term(iTRAQ4plex); K7(iTRAQ4	1	1
[R].lNILNAk.[F]	N-Term(iTRAQ4plex); K7(iTRAQ4	1	1
[K].fAFNLYR.[V]	N-Term(iTRAQ4plex)	1	1
[R].lNILNAk.[F]	N-Term(iTRAQ4plex); K7(iTRAQ4	1	1
[K].TLEAQLTPR.[V]		1	1
[K].gPLDQLEk.[G]	N-Term(iTRAQ4plex); K8(iTRAQ4	1	1
[K].gPLDQLEk.[G]	N-Term(iTRAQ4plex); K8(iTRAQ4	1	1
[K].fPVEMTHNHNFR.[L]	N-Term(iTRAQ4plex)	1	1
[K].fPVEmTHNHNFR.[L]	N-Term(iTRAQ4plex); M5(Oxidat	1	1
[K].fAFNLYR.[V]	N-Term(iTRAQ4plex)	1	1
[K].fPVEMTHNHNFR.[L]	N-Term(iTRAQ4plex)	1	1
[R].lTGSSGFVTDGPGNYk.[Y]	N-Term(iTRAQ4plex); K16(iTRAQ	1	1
[R].lTGSSGFVTDGPGNYk.[Y]	N-Term(iTRAQ4plex); K16(iTRAQ	1	1
[R].wSVLPRPDLHHDVNR.[F]	N-Term(iTRAQ4plex)	1	1
[R].wSVLPRPDLHHDVNR.[F]	N-Term(iTRAQ4plex)	1	1
[R].wSVLPRPDLHHDVNR.[F]	N-Term(iTRAQ4plex)	1	1
[R].nHNALLASLTTQk.[K]	N-Term(iTRAQ4plex); K13(iTRAQ	1	1
[R].nHNALLASLTTQk.[K]	N-Term(iTRAQ4plex); K13(iTRAQ	1	1
[K].cFSSDFMAYDIAcDR.[W]	N-Term(iTRAQ4plex); C1(Carbam	1	1
[R].iMQSSQSMSk.[L]	N-Term(iTRAQ4plex); K10(iTRAQ	1	1
[R].aLYVHGGYk.[A]	N-Term(iTRAQ4plex); K9(iTRAQ4	1	1
[R].nHNALLASLTTQk.[K]	N-Term(iTRAQ4plex); K13(iTRAQ	1	1
[R].IMQSSQSMSk.[L]	K10(iTRAQ4plex)	1	1
[R].sVNNVVVR.[Y]	N-Term(iTRAQ4plex)	1	1
[R].SEAAcLAAGPGIR.[C]	C5(Carbamidomethyl)	1	1
[K].dNPMYYcNk.[K]	N-Term(iTRAQ4plex); C7(Carbam	1	1
[K].dNPMYYcNk.[K]	N-Term(iTRAQ4plex); C7(Carbam	1	1
[K].gEAcDIPHcTDNcGFPHR.[G]	N-Term(iTRAQ4plex); C4(Carbam	1	1
[R].sEAAcLAAGPGIR.[C]	N-Term(iTRAQ4plex); C5(Carbam	1	1
[R].sEAAcLAAGPGIR.[C]	N-Term(iTRAQ4plex); C5(Carbam	1	1

[K].gDEcQLcEVENR.[Y]	N-Term(iTRAQ4plex); C4(Carbam	1	1
[K].gDEcQLcEVENR.[Y]	N-Term(iTRAQ4plex); C4(Carbam	1	1
[K].gDEcQLcEVENR.[Y]	N-Term(iTRAQ4plex); C4(Carbam	1	1
[K].gDEcQLcEVENR.[Y]	N-Term(iTRAQ4plex); C4(Carbam	1	1
[K].gDEcQLcEVENR.[Y]	N-Term(iTRAQ4plex); C4(Carbam	1	1
[K].gDEcQLcEVENR.[Y]	N-Term(iTRAQ4plex); C4(Carbam	1	1
[K].gDEcQLcEVENR.[Y]	N-Term(iTRAQ4plex); C4(Carbam	1	1
[K].kVEFVLk.[Q]	N-Term(iTRAQ4plex); K1(iTRAQ4	1	1
[R].lTGSSGFVTDGPGNYk.[Y]	N-Term(iTRAQ4plex); K16(iTRAQ	1	1
[K].cFSSDFMAYDIAcDR.[W]	N-Term(iTRAQ4plex); C1(Carbam	1	1
[K].dSFSNEk.[F]	N-Term(iTRAQ4plex); K7(iTRAQ4	1	1
[K].gDEcQLcEVENR.[Y]	N-Term(iTRAQ4plex); C4(Carbam	1	1
[R].yDVDTQMWTILk.[D]	N-Term(iTRAQ4plex); K12(iTRAQ	1	1
[K].dNPmYYcNk.[K]	N-Term(iTRAQ4plex); M4(Oxidat	1	1
[R].sVNNVVVR.[Y]	N-Term(iTRAQ4plex)	1	1
[R].yGHSLALYk.[D]	N-Term(iTRAQ4plex); K9(iTRAQ4	1	1
[R].eEYSNLk.[L]	N-Term(iTRAQ4plex); K7(iTRAQ4	1	1
[R].imQSSQSMSk.[L]	N-Term(iTRAQ4plex); M2(Oxidat	1	1
[R].iMQSSQSMSk.[L]	N-Term(iTRAQ4plex); K10(iTRAQ	1	1
[R].sEAAcLAAGPGIR.[C]	N-Term(iTRAQ4plex); C5(Carbam	1	1
[R].aLYVHGGYk.[A]	N-Term(iTRAQ4plex); K9(iTRAQ4	1	1
[K].cTWLIEGQPNR.[I]	N-Term(iTRAQ4plex); C1(Carbam	1	1
[K].lTLTPWVGLR.[K]	N-Term(iTRAQ4plex)	1	1
[K].kVEFVLk.[Q]	N-Term(iTRAQ4plex); K1(iTRAQ4	1	1
[R].nHNALLASLTTQk.[K]	N-Term(iTRAQ4plex); K13(iTRAQ	1	1
[K].cTWLIEGQPNR.[I]	N-Term(iTRAQ4plex); C1(Carbam	1	1
[R].iMQSSQSmSk.[L]	N-Term(iTRAQ4plex); M8(Oxidat	1	1
[K].gEAcDIPHcTDNcGFPHR.[G]	N-Term(iTRAQ4plex); C4(Carbam	1	1
[K].aVLDVFEEGTEASAATAVk.[I]	N-Term(iTRAQ4plex); K19(iTRAQ	1	1
[R].lYGSEAFATDFQDSAAAk.[K]	N-Term(iTRAQ4plex); K18(iTRAQ	1	1
[R].lYGSEAFATDFQDSAAAk.[K]	N-Term(iTRAQ4plex); K18(iTRAQ	1	1
[R].lYGSEAFATDFQDSAAAk.[K]	N-Term(iTRAQ4plex); K18(iTRAQ	1	1
[K].wEMPFDPQDTHQSR.[F]	N-Term(iTRAQ4plex)	1	1
[K].wEMPFDPQDTHQSR.[F]	N-Term(iTRAQ4plex)	1	1
[K].wEMPFDPQDTHQSR.[F]	N-Term(iTRAQ4plex)	1	1
[K].wEMPFDPQDTHQSR.[F]	N-Term(iTRAQ4plex)	1	1
[R].nLAVSQVVHk.[A]	N-Term(iTRAQ4plex); K10(iTRAQ	1	1
[K].ADLSGITGAR.[N]		1	1
[K].mEEVEAMLLPETLk.[R]	N-Term(iTRAQ4plex); M1(Oxidat	1	1
[K].aVLDVFEEGTEASAATAVk.[I]	N-Term(iTRAQ4plex); K19(iTRAQ	1	1
[K].mEEVEAmLLPETLk.[R]	N-Term(iTRAQ4plex); M7(Oxidat	1	1
[K].mEEVEAMLLPETLk.[R]	N-Term(iTRAQ4plex); M1(Oxidat	1	1
[K].mEEVEAMLLPETLk.[R]	N-Term(iTRAQ4plex); M1(Oxidat	1	1
[K].aVLDVFEEGTEASAATAVk.[I]	N-Term(iTRAQ4plex); K19(iTRAQ	1	1
[K].aVLDVFEEGTEASAATAVk.[I]	N-Term(iTRAQ4plex); K19(iTRAQ	1	1
[K].mEEVEAMLLPETLk.[R]	N-Term(iTRAQ4plex); M1(Oxidat	1	1
[K].aVLDVFEEGTEASAATAVk.[I]	N-Term(iTRAQ4plex); K19(iTRAQ	1	1
[K].mEEVEAmLLPETLk.[R]	N-Term(iTRAQ4plex); M7(Oxidat	1	1
[K].mEEVEAmLLPETLk.[R]	N-Term(iTRAQ4plex); M7(Oxidat	1	1
[K].mEEVEAmLLPETLk.[R]	N-Term(iTRAQ4plex); M7(Oxidat	1	1
[K].mEEVEAMLLPETLk.[R]	N-Term(iTRAQ4plex); K14(iTRAQ	1	1
[K].mEEVEAMLLPETLk.[R]	N-Term(iTRAQ4plex); K14(iTRAQ	1	1

[K].mEEVEAMLLPETLk.[R]	N-Term(iTRAQ4plex); K14(iTRAQ	1	1
[K].mEEVEAMLLPETLk.[R]	N-Term(iTRAQ4plex); M1(Oxidat	1	1
[K].mEEVEAmLLPETLk.[R]	N-Term(iTRAQ4plex); M7(Oxidat	1	1
[K].mEEVEAmLLPETLk.[R]	N-Term(iTRAQ4plex); M7(Oxidat	1	1
[K].mEEVEAMLLPETLk.[R]	N-Term(iTRAQ4plex); M1(Oxidat	1	1
[K].mEEVEAMLLPETLk.[R]	N-Term(iTRAQ4plex); M1(Oxidat	1	1
[K].mEEVEAMLLPETLk.[R]	N-Term(iTRAQ4plex); M1(Oxidat	1	1
[K].mEEVEAMLLPETLk.[R]	N-Term(iTRAQ4plex); M1(Oxidat	1	1
[K].mEEVEAmLLPETLk.[R]	N-Term(iTRAQ4plex); M7(Oxidat	1	1
[K].mEEVEAmLLPETLk.[R]	N-Term(iTRAQ4plex); M7(Oxidat	1	1
[K].mEEVEAmLLPETLk.[R]	N-Term(iTRAQ4plex); M7(Oxidat	1	1
[K].aVLDVFEEGTEASAATAVk.[I]	N-Term(iTRAQ4plex); K19(iTRAQ	1	1
[K].mEEVEAMLLPETLk.[R]	N-Term(iTRAQ4plex); M1(Oxidat	1	1
[K].aVLDVFEEGTEASAATAVk.[I]	N-Term(iTRAQ4plex); K19(iTRAQ	1	1
[K].aVLDVFEEGTEASAATAVk.[I]	N-Term(iTRAQ4plex); K19(iTRAQ	1	1
[K].mEEVEAMLLPETLk.[R]	N-Term(iTRAQ4plex); M1(Oxidat	1	1
[R].lYGSEAFATDFQDSAAAk.[K]	N-Term(iTRAQ4plex); K18(iTRAQ	1	1
[K].aVLDVFEEGTEASAATAVk.[I]	N-Term(iTRAQ4plex); K19(iTRAQ	1	1
[K].mEEVEAmLLPETLk.[R]	N-Term(iTRAQ4plex); M7(Oxidat	1	1
[K].aVLDVFEEGTEASAATAVk.[I]	N-Term(iTRAQ4plex); K19(iTRAQ	1	1
[K].aVLDVFEEGTEASAATAVk.[I]	N-Term(iTRAQ4plex); K19(iTRAQ	1	1
[K].aVLDVFEEGTEASAATAVk.[I]	N-Term(iTRAQ4plex); K19(iTRAQ	1	1
[R].lYGSEAFATDFQDSAAAk.[K]	N-Term(iTRAQ4plex); K18(iTRAQ	1	1
[K].aVLDVFEEGTEASAATAVk.[I]	N-Term(iTRAQ4plex); K19(iTRAQ	1	1
[R].lYGSEAFATDFQDSAAAk.[K]	N-Term(iTRAQ4plex); K18(iTRAQ	1	1
[R].lYGSEAFATDFQDSAAAk.[K]	N-Term(iTRAQ4plex); K18(iTRAQ	1	1
[R].lYGSEAFATDFQDSAAAk.[K]	N-Term(iTRAQ4plex); K18(iTRAQ	1	1
[R].lYGSEAFATDFQDSAAAk.[K]	N-Term(iTRAQ4plex); K18(iTRAQ	1	1
[R].lYGSEAFATDFQDSAAAk.[K]	N-Term(iTRAQ4plex); K18(iTRAQ	1	1
[R].lYGSEAFATDFQDSAAAk.[K]	N-Term(iTRAQ4plex); K18(iTRAQ	1	1
[R].lYGSEAFATDFQDSAAAk.[K]	N-Term(iTRAQ4plex); K18(iTRAQ	1	1
[R].lYGSEAFATDFQDSAAAk.[K]	N-Term(iTRAQ4plex); K18(iTRAQ	1	1
[K].aVLDVFEEGTEASAATAVk.[I]	N-Term(iTRAQ4plex); K19(iTRAQ	1	1
[K].aVLDVFEEGTEASAATAVk.[I]	N-Term(iTRAQ4plex); K19(iTRAQ	1	1
[K].aVLDVFEEGTEASAATAVk.[I]	N-Term(iTRAQ4plex); K19(iTRAQ	1	1
[K].aVLDVFEEGTEASAATAVk.[I]	N-Term(iTRAQ4plex); K19(iTRAQ	1	1
[K].aVLDVFEEGTEASAATAVk.[I]	N-Term(iTRAQ4plex); K19(iTRAQ	1	1
[K].aVLDVFEEGTEASAATAVk.[I]	N-Term(iTRAQ4plex); K19(iTRAQ	1	1
[K].aVLDVFEEGTEASAATAVk.[I]	N-Term(iTRAQ4plex); K19(iTRAQ	1	1
[K].aVLDVFEEGTEASAATAVk.[I]	N-Term(iTRAQ4plex); K19(iTRAQ	1	1
[K].aVLDVFEEGTEASAATAVk.[I]	N-Term(iTRAQ4plex); K19(iTRAQ	1	1
[K].aVLDVFEEGTEASAATAVk.[I]	N-Term(iTRAQ4plex); K19(iTRAQ	1	1
[R].lYGSEAFATDFQDSAAAk.[K]	N-Term(iTRAQ4plex); K18(iTRAQ	1	1
[K].aVLDVFEEGTEASAATAVk.[I]	N-Term(iTRAQ4plex); K19(iTRAQ	1	1
[K].aVLDVFEEGTEASAATAVk.[I]	N-Term(iTRAQ4plex); K19(iTRAQ	1	1
[R].lYGSEAFATDFQDSAAAk.[K]	N-Term(iTRAQ4plex); K18(iTRAQ	1	1
[K].aVLDVFEEGTEASAATAVk.[I]	N-Term(iTRAQ4plex); K19(iTRAQ	1	1
[K].aVLDVFEEGTEASAATAVk.[I]	N-Term(iTRAQ4plex); K19(iTRAQ	1	1
[K].rLYGSEAFATDFQDSAAAk.[K]	N-Term(iTRAQ4plex); K19(iTRAQ	1	1
[K].rLYGSEAFATDFQDSAAAk.[K]	N-Term(iTRAQ4plex); K19(iTRAQ	1	1
[R].eIGELYLPk.[F]	N-Term(iTRAQ4plex); K9(iTRAQ4	1	1
[R].eIGELYLPk.[F]	N-Term(iTRAQ4plex); K9(iTRAQ4	1	1

[R].eIGELYLPk.[F]	N-Term(iTRAQ4plex); K9(iTRAQ4	1	1
[R].lYGSEAFATDFQDSAAAk.[K]	N-Term(iTRAQ4plex); K18(iTRAQ4	1	1
[R].lYGSEAFATDFQDSAAAk.[K]	N-Term(iTRAQ4plex); K18(iTRAQ4	1	1
[R].lYGSEAFATDFQDSAAAk.[K]	N-Term(iTRAQ4plex); K18(iTRAQ4	1	1
[R].lYGSEAFATDFQDSAAAk.[K]	N-Term(iTRAQ4plex); K18(iTRAQ4	1	1
[R].lYGSEAFATDFQDSAAAk.[K]	N-Term(iTRAQ4plex); K18(iTRAQ4	1	1
[R].lYGSEAFATDFQDSAAAk.[K]	N-Term(iTRAQ4plex); K18(iTRAQ4	1	1
[R].lYGSEAFATDFQDSAAAk.[K]	N-Term(iTRAQ4plex); K18(iTRAQ4	1	1
[R].lYGSEAFATDFQDSAAAk.[K]	N-Term(iTRAQ4plex); K18(iTRAQ4	1	1
[R].lYGSEAFATDFQDSAAAk.[K]	N-Term(iTRAQ4plex); K18(iTRAQ4	1	1
[R].lYGSEAFATDFQDSAAAk.[K]	N-Term(iTRAQ4plex); K18(iTRAQ4	1	1
[R].lYGSEAFATDFQDSAAAk.[K]	N-Term(iTRAQ4plex); K18(iTRAQ4	1	1
[R].lYGSEAFATDFQDSAAAk.[K]	N-Term(iTRAQ4plex); K18(iTRAQ4	1	1
[R].lYGSEAFATDFQDSAAAk.[K]	N-Term(iTRAQ4plex); K18(iTRAQ4	1	1
[R].lYGSEAFATDFQDSAAAk.[K]	N-Term(iTRAQ4plex); K18(iTRAQ4	1	1
[K].aDLSGITGAR.[N]	N-Term(iTRAQ4plex)	1	1
[R].nLAVSQVVHk.[A]	N-Term(iTRAQ4plex); K10(iTRAQ4	1	1
[K].kLINDYVk.[N]	N-Term(iTRAQ4plex); K1(iTRAQ4	1	1
[R].nLAVSQVVHk.[A]	N-Term(iTRAQ4plex); K10(iTRAQ4	1	1
[K].kLINDYVk.[N]	N-Term(iTRAQ4plex); K1(iTRAQ4	1	1
[R].nLAVSQVVHk.[A]	N-Term(iTRAQ4plex); K10(iTRAQ4	1	1
[R].nLAVSQVVHk.[A]	N-Term(iTRAQ4plex); K10(iTRAQ4	1	1
[K].aDLSGITGAR.[N]	N-Term(iTRAQ4plex)	1	1
[R].nLAVSQVVHk.[A]	N-Term(iTRAQ4plex); K10(iTRAQ4	1	1
[K].aVLDVFEEGTEASAATAVk.[I]	N-Term(iTRAQ4plex); K19(iTRAQ4	1	1
[R].lYGSEAFATDFQDSAAAk.[K]	N-Term(iTRAQ4plex); K18(iTRAQ4	1	1
[K].aVLDVFEEGTEASAATAVk.[I]	N-Term(iTRAQ4plex); K19(iTRAQ4	1	1
[R].lYGSEAFATDFQDSAAAk.[K]	N-Term(iTRAQ4plex); K18(iTRAQ4	1	1
[R].lYGSEAFATDFQDSAAAk.[K]	N-Term(iTRAQ4plex); K18(iTRAQ4	1	1
[R].lYGSEAFATDFQDSAAAk.[K]	N-Term(iTRAQ4plex); K18(iTRAQ4	1	1
[K].mEEVEAMLLPETLk.[R]	N-Term(iTRAQ4plex); K14(iTRAQ4	1	1
[K].mEEVEAMLLPETLk.[R]	N-Term(iTRAQ4plex); K14(iTRAQ4	1	1
[R].nLAVSQVVHk.[A]	N-Term(iTRAQ4plex); K10(iTRAQ4	1	1
[K].wEMPFDPQDTHQSR.[F]	N-Term(iTRAQ4plex)	1	1
[R].lYGSEAFATDFQDSAAAk.[K]	N-Term(iTRAQ4plex); K18(iTRAQ4	1	1
[R].lYGSEAFATDFQDSAAAk.[K]	N-Term(iTRAQ4plex); K18(iTRAQ4	1	1
[R].nLAVSQVVHkAVLDVFEEGTEASAATAVk.[	N-Term(iTRAQ4plex); K10(iTRAQ4	1	1
[R].dEELScTVVELk.[Y]	N-Term(iTRAQ4plex); C6(Carbam	1	1
[R].NLAVSQVVHkAVLDVFEEGTEASAATAVk.[	K10(iTRAQ4plex); K29(iTRAQ4pl	1	1
[K].ITLLSALVETR.[T]		1	1
[R].gTHVDLGLASANVDFAFSLYk.[Q]	N-Term(iTRAQ4plex); K21(iTRAQ4	1	1
[R].dEELScTVVELk.[Y]	N-Term(iTRAQ4plex); C6(Carbam	1	1
[R].dEELScTVVELk.[Y]	N-Term(iTRAQ4plex); C6(Carbam	1	1
[R].gTHVDLGLASANVDFAFSLYk.[Q]	N-Term(iTRAQ4plex); K21(iTRAQ4	1	1
[R].DEELScTVVELk.[Y]	C6(Carbamidomethyl); K12(iTRA	1	1
[K].iTLLSALVETR.[T]	N-Term(iTRAQ4plex)	1	1
[K].iTLLSALVETR.[T]	N-Term(iTRAQ4plex)	1	1
[K].iTLLSALVETR.[T]	N-Term(iTRAQ4plex)	1	1
[K].iTLLSALVETR.[T]	N-Term(iTRAQ4plex)	1	1
[K].iTLLSALVETR.[T]	N-Term(iTRAQ4plex)	1	1
[K].iTLLSALVETR.[T]	N-Term(iTRAQ4plex)	1	1
[R].dYNLNDILLQLGIEEAFTSk.[A]	N-Term(iTRAQ4plex); K20(iTRAQ4	1	1

[R].dYNLNDILLQLGIEEAFTSk.[A]	N-Term(iTRAQ4plex); K20(iTRAQ	1	1
[K].dLDSQTMMVLVNYIFFk.[A]	N-Term(iTRAQ4plex); K17(iTRAQ	1	1
[K].dLDSQTMMVLVNYIFFk.[A]	N-Term(iTRAQ4plex); K17(iTRAQ	1	1
[R].fNRPFLMIIVPTDTQNIFFMSk.[V]	N-Term(iTRAQ4plex); K22(iTRAQ	1	1
[R].fNRPFLMIIVPTDTQNIFFMSk.[V]	N-Term(iTRAQ4plex); K22(iTRAQ	1	1
[K].mEEVEAmLLPETLk.[R]	N-Term(iTRAQ4plex); M7(Oxidat	1	1
[R].nLAVSQVVHk.[A]	N-Term(iTRAQ4plex); K10(iTRAQ	1	1
[K].eQLSLLDR.[F]	N-Term(iTRAQ4plex)	1	1
[R].LYGSEAFATDFQDSAAAk.[K]	K18(iTRAQ4plex)	1	1
[K].lINDYVk.[N]	N-Term(iTRAQ4plex); K7(iTRAQ4	1	1
[K].AVLDVFEEGTEASAATAVk.[I]	K19(iTRAQ4plex)	1	1
[R].lYGSEAFATDFQDSAAAk.[K]	N-Term(iTRAQ4plex); K18(iTRAQ	1	1
[K].aVLDVFEEGTEASAATAVk.[I]	N-Term(iTRAQ4plex); K19(iTRAQ	1	1
[K].aVLDVFEEGTEASAATAVkITLLSALVETR.[T	N-Term(iTRAQ4plex); K19(iTRAQ	1	1
[R].NLAVSQVVHk.[A]	K10(iTRAQ4plex)	1	1
[R].DEELScTVVELk.[Y]	C6(Carbamidomethyl); K12(iTRA	1	1
[R].eIGELYLPk.[F]	N-Term(iTRAQ4plex); K9(iTRAQ4	1	1
[K].aDLSGITGAR.[N]	N-Term(iTRAQ4plex)	1	1
[R].lYGSEAFATDFQDSAAAk.[K]	N-Term(iTRAQ4plex); K18(iTRAQ	1	1
[K].aVLDVFEEGTEASAATAVk.[I]	N-Term(iTRAQ4plex); K19(iTRAQ	1	1
[R].lYGSEAFATDFQDSAAAk.[K]	N-Term(iTRAQ4plex); K18(iTRAQ	1	1
[K].wEMPFDPQDTHQSR.[F]	N-Term(iTRAQ4plex)	1	1
[K].aVLDVFEEGTEASAATAVk.[I]	N-Term(iTRAQ4plex); K19(iTRAQ	1	1
[R].lYGSEAFATDFQDSAAAk.[K]	N-Term(iTRAQ4plex); K18(iTRAQ	1	1
[K].eQLSLLDR.[F]	N-Term(iTRAQ4plex)	1	1
[K].aDLSGITGAR.[N]	N-Term(iTRAQ4plex)	1	1
[R].lYGSEAFATDFQDSAAAk.[K]	N-Term(iTRAQ4plex); K18(iTRAQ	1	1
[K].aVLDVFEEGTEASAATAVk.[I]	N-Term(iTRAQ4plex); K19(iTRAQ	1	1
[K].rLYGSEAFATDFQDSAAAk.[K]	N-Term(iTRAQ4plex); K19(iTRAQ	1	1
[R].eIGELYLPk.[F]	N-Term(iTRAQ4plex); K9(iTRAQ4	1	1
[R].nLAVSQVVHk.[A]	N-Term(iTRAQ4plex); K10(iTRAQ	1	1
[R].lYGSEAFATDFQDSAAAk.[K]	N-Term(iTRAQ4plex); K18(iTRAQ	1	1
[R].EIGELYLPk.[F]	K9(iTRAQ4plex)	1	1
[R].lYGSEAFATDFQDSAAAk.[K]	N-Term(iTRAQ4plex); K18(iTRAQ	1	1
[R].nLAVSQVVHk.[A]	N-Term(iTRAQ4plex); K10(iTRAQ	1	1
[R].lYGSEAFATDFQDSAAAk.[K]	N-Term(iTRAQ4plex); K18(iTRAQ	1	1
[R].eIGELYLPk.[F]	N-Term(iTRAQ4plex); K9(iTRAQ4	1	1
[K].ADLSGITGAR.[N]		1	1
[K].aVLDVFEEGTEASAATAVk.[I]	N-Term(iTRAQ4plex); K19(iTRAQ	1	1
[R].NLAVSQVVHK.[A]		1	1
[K].MEEVEAMLLPETLk.[R]	K14(iTRAQ4plex)	1	1
[R].lYGSEAFATDFQDSAAAk.[K]	N-Term(iTRAQ4plex); K18(iTRAQ	1	1
[R].lYGSEAFATDFQDSAAAk.[K]	N-Term(iTRAQ4plex); K18(iTRAQ	1	1
[R].lYGSEAFATDFQDSAAAk.[K]	N-Term(iTRAQ4plex); K18(iTRAQ	1	1
[R].lYGSEAFATDFQDSAAAk.[K]	N-Term(iTRAQ4plex); K18(iTRAQ	1	1
[K].mEEVEAMLLPETLk.[R]	N-Term(iTRAQ4plex); M1(Oxidat	1	1
[R].lYGSEAFATDFQDSAAAk.[K]	N-Term(iTRAQ4plex); K18(iTRAQ	1	1
[K].wEMPFDPQDTHQSR.[F]	N-Term(iTRAQ4plex)	1	1
[K].lINDYVk.[N]	N-Term(iTRAQ4plex); K7(iTRAQ4	1	1
[K].aVLDVFEEGTEASAATAVk.[I]	N-Term(iTRAQ4plex); K19(iTRAQ	1	1
[R].nLAVSQVVHk.[A]	N-Term(iTRAQ4plex); K10(iTRAQ	1	1
[R].lYGSEAFATDFQDSAAAk.[K]	N-Term(iTRAQ4plex); K18(iTRAQ	1	1

[R].lYGSEAFATDFQDSAAAk.[K]	N-Term(iTRAQ4plex); K18(iTRAQ4	1	1
[K].LINDYVk.[N]	K7(iTRAQ4plex)	1	1
[K].wEmPFDPQDTHQSR.[F]	N-Term(iTRAQ4plex); M3(Oxidat	1	1
[R].eIGELYLPk.[F]	N-Term(iTRAQ4plex); K9(iTRAQ4	1	1
[R].DEELScTVVELk.[Y]	C6(Carbamidomethyl); K12(iTRA	1	1
[K].lINDYVk.[N]	N-Term(iTRAQ4plex); K7(iTRAQ4	1	1
[K].lINDYVk.[N]	N-Term(iTRAQ4plex); K7(iTRAQ4	1	1
[R].dEELScTVVELk.[Y]	N-Term(iTRAQ4plex); C6(Carbam	1	1
[R].eIGELYLPk.[F]	N-Term(iTRAQ4plex); K9(iTRAQ4	1	1
[K].lINDYVk.[N]	N-Term(iTRAQ4plex); K7(iTRAQ4	1	1
[R].NLAVSQVVHk.[A]	K10(iTRAQ4plex)	1	1
[R].eIGELYLPK.[F]	N-Term(iTRAQ4plex)	1	1
[K].wEmPFDPQDTHQSR.[F]	N-Term(iTRAQ4plex); M3(Oxidat	1	1
[K].wEMPFDPQDTHQSR.[F]	N-Term(iTRAQ4plex)	1	1
[K].iTLLSALVETR.[T]	N-Term(iTRAQ4plex)	1	1
[R].eIGELYLPk.[F]	N-Term(iTRAQ4plex); K9(iTRAQ4	1	1
[K].aVLDVFEEGTEASAATAVk.[I]	N-Term(iTRAQ4plex); K19(iTRAQ4	1	1
[K].aVLDVFEEGTEASAATAVk.[I]	N-Term(iTRAQ4plex); K19(iTRAQ4	1	1
[K].lINDYVk.[N]	N-Term(iTRAQ4plex); K7(iTRAQ4	1	1
[R].lYGSEAFATDFQDSAAAk.[K]	N-Term(iTRAQ4plex); K18(iTRAQ4	1	1
[K].mEEVEAmLLPETLk.[R]	N-Term(iTRAQ4plex); M7(Oxidat	1	1
[K].qEDDLANINQWVk.[E]	N-Term(iTRAQ4plex); K13(iTRAQ4	1	1
[R].lcQDLGPGAFR.[L]	N-Term(iTRAQ4plex); C2(Carbam	1	1
[R].qLTSGPNQEQVSPLTLLk.[L]	N-Term(iTRAQ4plex); K18(iTRAQ4	1	1
[R].dPTPEQTHR.[L]	N-Term(iTRAQ4plex)	1	1
[R].lcQDLGPGAFR.[L]	N-Term(iTRAQ4plex); C2(Carbam	1	1
[R].lcQDLGPGAFR.[L]	N-Term(iTRAQ4plex); C2(Carbam	1	1
[R].lcQDLGPGAFR.[L]	N-Term(iTRAQ4plex); C2(Carbam	1	1
[R].dSFHLDEQFTVPVEMMQAR.[T]	N-Term(iTRAQ4plex)	1	1
[R].dSFHLDEQFTVPVEMMQAR.[T]	N-Term(iTRAQ4plex)	1	1
[K].gFPIkEDFLEQSEQLFGAkPVSLTGk.[Q]	N-Term(iTRAQ4plex); K5(iTRAQ4	1	1
[R].wFLLEQPEIQVAHFPFk.[N]	N-Term(iTRAQ4plex); K17(iTRAQ4	1	1
[R].wFLLEQPEIQVAHFPFk.[N]	N-Term(iTRAQ4plex); K17(iTRAQ4	1	1
[R].WFLLEQPEIQVAHFPFk.[N]	K17(iTRAQ4plex)	1	1
[R].wFLLEQPEIQVAHFPFk.[N]	N-Term(iTRAQ4plex); K17(iTRAQ4	1	1
[R].gDkLFGPDLk.[L]	N-Term(iTRAQ4plex); K3(iTRAQ4	1	1
[R].lQQVLHAGSGPcLPHLLSR.[L]	N-Term(iTRAQ4plex); C12(Carbar	1	1
[R].lQQVLHAGSGPcLPHLLSR.[L]	N-Term(iTRAQ4plex); C12(Carbar	1	1
[R].lQQVLHAGSGPcLPHLLSR.[L]	N-Term(iTRAQ4plex); C12(Carbar	1	1
[K].hQMDLVATLSQLGLQELFQAPDLR.[G]	N-Term(iTRAQ4plex)	1	1
[K].hQMDLVATLSQLGLQELFQAPDLR.[G]	N-Term(iTRAQ4plex)	1	1
[R].eLkEQQDSPGNk.[D]	N-Term(iTRAQ4plex); K3(iTRAQ4	1	1
[R].eLkEQQDSPGNk.[D]	N-Term(iTRAQ4plex); K3(iTRAQ4	1	1
[K].lGNQEPGGQTALk.[S]	N-Term(iTRAQ4plex); K13(iTRAQ4	1	1
[K].lGNQEPGGQTALk.[S]	N-Term(iTRAQ4plex); K13(iTRAQ4	1	1
[K].eQQDSPGNk.[D]	N-Term(iTRAQ4plex); K9(iTRAQ4	1	1
[R].dSFHLDEQFTVPVEMMQAR.[T]	N-Term(iTRAQ4plex)	1	1
[R].LcQDLGPGAFR.[L]	C2(Carbamidomethyl)	1	1
[R].qLTSGPNQEQVSPLTLLk.[L]	N-Term(iTRAQ4plex); K18(iTRAQ4	1	1
[K].dFLQSLk.[G]	N-Term(iTRAQ4plex); K7(iTRAQ4	1	1
[R].lQQVLHAGSGPcLPHLLSR.[L]	N-Term(iTRAQ4plex); C12(Carbar	1	1
[R].NPNPSAPR.[E]		1	1

[K].qEDDLANINQWVk.[E]	N-Term(iTRAQ4plex); K13(iTRAQ	1	1
[R].NkFDPSLTQR.[D]	K2(iTRAQ4plex)	1	1
[K].fDPSLTQR.[D]	N-Term(iTRAQ4plex)	1	1
[K].dFLQSLk.[G]	N-Term(iTRAQ4plex); K7(iTRAQ4	1	1
[R].dPTPEQTHR.[L]	N-Term(iTRAQ4plex)	1	1
[R].nKFDPSLTQR.[D]	N-Term(iTRAQ4plex)	1	1
[R].nkFDPSLTQR.[D]	N-Term(iTRAQ4plex); K2(iTRAQ4	1	1
[R].LcQDLGPGAFR.[L]	C2(Carbamidomethyl)	1	1
[K].lFGPDLk.[L]	N-Term(iTRAQ4plex); K7(iTRAQ4	1	1
[K].lVPPMEEDYPQFGSPk.[-]	N-Term(iTRAQ4plex); K16(iTRAQ	1	1
[R].nPNPSAPR.[E]	N-Term(iTRAQ4plex)	1	1
[K].lGNQEPGGQTALk.[S]	N-Term(iTRAQ4plex); K13(iTRAQ	1	1
[K].qEDDLANINQWVk.[E]	N-Term(iTRAQ4plex); K13(iTRAQ	1	1
[K].dFLQSLk.[G]	N-Term(iTRAQ4plex); K7(iTRAQ4	1	1
[R].dPTPEQTHR.[L]	N-Term(iTRAQ4plex)	1	1
[K].lFGPDLk.[L]	N-Term(iTRAQ4plex); K7(iTRAQ4	1	1
[K].fDPSLTQR.[D]	N-Term(iTRAQ4plex)	1	1
[K].fEGIAcEISk.[Q]	N-Term(iTRAQ4plex); C6(Carbam	1	1
[K].fEGIAcEISk.[Q]	N-Term(iTRAQ4plex); C6(Carbam	1	1
[K].fEGIAcEISk.[Q]	N-Term(iTRAQ4plex); C6(Carbam	1	1
[K].aEQccEETASSISLHGkGSFR.[F]	N-Term(iTRAQ4plex); C4(Carbam	1	1
[R].kYAFELk.[ME]	N-Term(iTRAQ4plex); K1(iTRAQ4	1	2
[R].dVVLTTTFVDDIk.[A]	N-Term(iTRAQ4plex); K13(iTRAQ	1	1
[R].dVVLTTTFVDDIk.[A]	N-Term(iTRAQ4plex); K13(iTRAQ	1	1
[R].dVVLTTTFVDDIk.[A]	N-Term(iTRAQ4plex); K13(iTRAQ	1	1
[R].kYAFELk.[ME]	N-Term(iTRAQ4plex); K1(iTRAQ4	1	2
[K].TSNFNAAISLk.[F]	K11(iTRAQ4plex)	1	1
[K].tSNFNAAISLk.[F]	N-Term(iTRAQ4plex); K11(iTRAQ	1	1
[K].tSNFNAAISLk.[F]	N-Term(iTRAQ4plex); K11(iTRAQ	1	1
[R].aIEDYINEFSVR.[K]	N-Term(iTRAQ4plex)	1	1
[R].aIEDYINEFSVR.[K]	N-Term(iTRAQ4plex)	1	1
[K].lSPIYNLVPVk.[M]	N-Term(iTRAQ4plex); K11(iTRAQ	1	1
[R].dRDGNTLTYYR.[R]	N-Term(iTRAQ4plex)	1	1
[R].tEHYEEQIEAFk.[S]	N-Term(iTRAQ4plex); K12(iTRAQ	1	1
[K].lSPIYNLVPVk.[M]	N-Term(iTRAQ4plex); K11(iTRAQ	1	1
[R].tEHYEEQIEAFk.[S]	N-Term(iTRAQ4plex); K12(iTRAQ	1	1
[R].tEHYEEQIEAFk.[S]	N-Term(iTRAQ4plex); K12(iTRAQ	1	1
[R].vVEESELAR.[T]	N-Term(iTRAQ4plex)	1	1
[K].aEQccEETASSISLHGk.[G]	N-Term(iTRAQ4plex); C4(Carbam	1	1
[R].rPWNVASLIYETk.[G]	N-Term(iTRAQ4plex); K13(iTRAQ	1	1
[R].rPWNVASLIYETk.[G]	N-Term(iTRAQ4plex); K13(iTRAQ	1	1
[R].qcVPTEPcEDAEDDcGNDFQcSTGR.[C]	N-Term(iTRAQ4plex); C2(Carbam	1	1
[K].aLPTTYEk.[G]	N-Term(iTRAQ4plex); K8(iTRAQ4	1	1
[R].rPWNVASLIYETk.[G]	N-Term(iTRAQ4plex); K13(iTRAQ	1	1
[R].qcVPTEPcEDAEDDcGNDFQcSTGR.[C]	N-Term(iTRAQ4plex); C2(Carbam	1	1
[R].qcVPTEPcEDAEDDcGNDFQcSTGR.[C]	N-Term(iTRAQ4plex); C2(Carbam	1	1
[R].vVEESELAR.[T]	N-Term(iTRAQ4plex)	1	1
[R].rPWNVASLIYETk.[G]	N-Term(iTRAQ4plex); K13(iTRAQ	1	1
[R].dGNTLTYYR.[R]	N-Term(iTRAQ4plex)	1	1
[K].aEQccEETASSISLHGk.[G]	N-Term(iTRAQ4plex); C4(Carbam	1	1
[R].dVVLTTTFVDDIk.[A]	N-Term(iTRAQ4plex); K13(iTRAQ	1	1
[K].fTPTETNk.[A]	N-Term(iTRAQ4plex); K8(iTRAQ4	1	1

[R].NRDVVLTTTFVDDIk.[A]	K15(iTRAQ4plex)	1	1
[R].TEHYEEQIEAFk.[S]	K12(iTRAQ4plex)	1	1
[R].VVEESELAR.[T]		1	1
[R].dVVLTTTFVDDIk.[A]	N-Term(iTRAQ4plex); K13(iTRAQ	1	1
[R].vVEESELAR.[T]	N-Term(iTRAQ4plex)	1	1
[K].FTPTETNk.[A]	K8(iTRAQ4plex)	1	1
[K].FEGIAcEISk.[Q]	C6(Carbamidomethyl); K10(iTRA	1	1
[R].DGNTLTYYR.[R]		1	1
[K].cLcAcPFk.[F]	N-Term(iTRAQ4plex); C1(Carbam	1	1
[R].qcVPTEPcEDAEDDcGNDFQcSTGR.[C]	N-Term(iTRAQ4plex); C2(Carbam	1	1
[K].lSPIYNLVPVk.[M]	N-Term(iTRAQ4plex); K11(iTRAQ	1	1
[K].tSNFNAAISLk.[F]	N-Term(iTRAQ4plex); K11(iTRAQ	1	1
[K].gEIHLGR.[F]	N-Term(iTRAQ4plex)	1	1
[R].VVEESELAR.[T]		1	1
[K].cLcAcPFk.[F]	N-Term(iTRAQ4plex); C1(Carbam	1	1
[R].dVVLTTTFVDDIk.[A]	N-Term(iTRAQ4plex); K13(iTRAQ	1	1
[K].VTTSQDMLSIMEk.[L]	K13(iTRAQ4plex)	1	1
[R].lLDSLPSDTR.[L]	N-Term(iTRAQ4plex)	1	1
[K].dFTcVHQALk.[G]	N-Term(iTRAQ4plex); C4(Carbam	1	1
[K].dFTcVHQALk.[G]	N-Term(iTRAQ4plex); C4(Carbam	1	1
[K].dFTcVHQALk.[G]	N-Term(iTRAQ4plex); C4(Carbam	1	1
[K].lYHAFSAMk.[K]	N-Term(iTRAQ4plex); K9(iTRAQ4	1	1
[K].dFTcVHQALk.[G]	N-Term(iTRAQ4plex); C4(Carbam	1	1
[K].vATTVISk.[M]	N-Term(iTRAQ4plex); K8(iTRAQ4	1	1
[K].kYPVAHFIDQTLk.[A]	N-Term(iTRAQ4plex); K1(iTRAQ4	1	1
[K].kYPVAHFIDQTLk.[A]	N-Term(iTRAQ4plex); K1(iTRAQ4	1	1
[K].tNLESILSYPk.[D]	N-Term(iTRAQ4plex); K11(iTRAQ	1	1
[K].tNLESILSYPk.[D]	N-Term(iTRAQ4plex); K11(iTRAQ	1	1
[K].tNLESILSYPk.[D]	N-Term(iTRAQ4plex); K11(iTRAQ	1	1
[K].tNLESILSYPk.[D]	N-Term(iTRAQ4plex); K11(iTRAQ	1	1
[K].yPVAHFIDQTLk.[A]	N-Term(iTRAQ4plex); K12(iTRAQ	1	1
[K].yPVAHFIDQTLk.[A]	N-Term(iTRAQ4plex); K12(iTRAQ	1	1
[K].GVTSVSQIFHSPDLAIR.[D]		1	1
[K].gVTSVSQIFHSPDLAIR.[D]	N-Term(iTRAQ4plex)	1	1
[K].kYPVAHFIDQTLk.[A]	N-Term(iTRAQ4plex); K1(iTRAQ4	1	1
[K].kYPVAHFIDQTLk.[A]	N-Term(iTRAQ4plex); K1(iTRAQ4	1	1
[K].kYPVAHFIDQTLk.[A]	N-Term(iTRAQ4plex); K1(iTRAQ4	1	1
[K].kYPVAHFIDQTLk.[A]	N-Term(iTRAQ4plex); K1(iTRAQ4	1	1
[K].kYPVAHFIDQTLk.[A]	N-Term(iTRAQ4plex); K1(iTRAQ4	1	1
[K].kYPVAHFIDQTLk.[A]	N-Term(iTRAQ4plex); K1(iTRAQ4	1	1
[K].kYPVAHFIDQTLk.[A]	N-Term(iTRAQ4plex); K1(iTRAQ4	1	1
[K].kYPVAHFIDQTLk.[A]	N-Term(iTRAQ4plex); K1(iTRAQ4	1	1
[K].kYPVAHFIDQTLk.[A]	N-Term(iTRAQ4plex); K1(iTRAQ4	1	1
[K].kYPVAHFIDQTLk.[A]	N-Term(iTRAQ4plex); K1(iTRAQ4	1	1
[K].kYPVAHFIDQTLk.[A]	N-Term(iTRAQ4plex); K1(iTRAQ4	1	1
[K].hRLEDMEQALSPSVFk.[A]	N-Term(iTRAQ4plex); K16(iTRAQ	1	1
[R].lEDmEQALSPSVFk.[A]	N-Term(iTRAQ4plex); M4(Oxidat	1	1
[R].lEDmEQALSPSVFk.[A]	N-Term(iTRAQ4plex); M4(Oxidat	1	1
[K].vTTSQDmLSIMEk.[L]	N-Term(iTRAQ4plex); M7(Oxidat	1	1
[K].vTTSQDmLSIMEk.[L]	N-Term(iTRAQ4plex); M7(Oxidat	1	1
[R].lEDMEQALSPSVFk.[A]	N-Term(iTRAQ4plex); K14(iTRAQ	1	1
[R].lEDMEQALSPSVFk.[A]	N-Term(iTRAQ4plex); K14(iTRAQ	1	1

[R].lEDMEQALSPSVFk.[A]	N-Term(iTRAQ4plex); K14(iTRAQ4	1	1
[K].TNLESILSYPk.[D]	K11(iTRAQ4plex)	1	1
[K].vTTSQDMLSIMEk.[L]	N-Term(iTRAQ4plex); K13(iTRAQ4	1	1
[R].lVLLNAIYLSAk.[W]	N-Term(iTRAQ4plex); K12(iTRAQ4	1	1
[K].fQPTLLTLPR.[I]	N-Term(iTRAQ4plex)	1	1
[K].gVTSVSQIFHSPDLAIR.[D]	N-Term(iTRAQ4plex)	1	1
[K].gVTSVSQIFHSPDLAIR.[D]	N-Term(iTRAQ4plex)	1	1
[R].lVLLNAIYLSAk.[W]	N-Term(iTRAQ4plex); K12(iTRAQ4	1	1
[K].gVTSVSQIFHSPDLAIR.[D]	N-Term(iTRAQ4plex)	1	1
[R].tLLVFEVQQPFLFVLWDQQHkFPVFMGR.	N-Term(iTRAQ4plex); K21(iTRAQ4	1	1
[R].LLDSLPSDTR.[L]		1	1
[K].vATTVISk.[M]	N-Term(iTRAQ4plex); K8(iTRAQ4	1	1
[R].lLDSLPSDTR.[L]	N-Term(iTRAQ4plex)	1	1
[K].lYHAFSAMk.[K]	N-Term(iTRAQ4plex); K9(iTRAQ4	1	1
[R].lLDSLPSDTR.[L]	N-Term(iTRAQ4plex)	1	1
[K].dFTcVHQALk.[G]	N-Term(iTRAQ4plex); C4(Carbam	1	1
[K].dFTcVHQALk.[G]	N-Term(iTRAQ4plex); C4(Carbam	1	1
[K].kYPVAHFIDQTLk.[A]	N-Term(iTRAQ4plex); K13(iTRAQ4	1	1
[K].kYPVAHFIDQTLk.[A]	K1(iTRAQ4plex); K13(iTRAQ4plex	1	1
[R].lEDMEQALSPSVFk.[A]	N-Term(iTRAQ4plex); K14(iTRAQ4	1	1
[K].dFTcVHQALk.[G]	N-Term(iTRAQ4plex); C4(Carbam	1	1
[K].gVTSVSQIFHSPDLAIR.[D]	N-Term(iTRAQ4plex)	1	1
[R].lLDSLPSDTR.[L]	N-Term(iTRAQ4plex)	1	1
[K].kYPVAHFIDQTLk.[A]	K1(iTRAQ4plex); K13(iTRAQ4plex	1	1
[K].kYPVAHFIDQTLk.[A]	N-Term(iTRAQ4plex); K13(iTRAQ4	1	1
[K].dFTcVHQALk.[G]	N-Term(iTRAQ4plex); C4(Carbam	1	1
[R].LEDMEQALSPSVFk.[A]	K14(iTRAQ4plex)	1	1
[K].kYPVAHFIDQTLk.[A]	N-Term(iTRAQ4plex); K1(iTRAQ4	1	1
[K].DFTcVHQALk.[G]	C4(Carbamidomethyl); K10(iTRA	1	1
[K].FQPTLLTLPR.[I]		1	1
[R].lEDMEQALSPSVFk.[A]	N-Term(iTRAQ4plex); K14(iTRAQ4	1	1
[R].mEPFHFk.[N]	N-Term(iTRAQ4plex); K7(iTRAQ4	1	1
[K].tNLESILSYPk.[D]	N-Term(iTRAQ4plex); K11(iTRAQ4	1	1
[K].DFTcVHQALk.[G]	C4(Carbamidomethyl); K10(iTRA	1	1
[R].mEPFHFk.[N]	N-Term(iTRAQ4plex); K7(iTRAQ4	1	1
[R].mEPFHFk.[N]	N-Term(iTRAQ4plex); K7(iTRAQ4	1	1
[K].TNLESILSYPk.[D]	K11(iTRAQ4plex)	1	1
[K].LYHAFSAMk.[K]	K9(iTRAQ4plex)	1	1
[K].dFTcVHQALk.[G]	N-Term(iTRAQ4plex); C4(Carbam	1	1
[R].TLYSSSPR.[V]		1	1
[R].vLSNNSDANLELINTWVAk.[N]	N-Term(iTRAQ4plex); K19(iTRAQ4	1	1
[K].yPVAHFIDQTLk.[A]	N-Term(iTRAQ4plex); K12(iTRAQ4	1	1
[R].lLDSLPSDTR.[L]	N-Term(iTRAQ4plex)	1	1
[R].mEPFHFk.[N]	N-Term(iTRAQ4plex); M1(Oxidat	1	1
[K].FPVFMGR.[V]		1	1
[R].lLDSLPSDTR.[L]	N-Term(iTRAQ4plex)	1	1
[K].kYPVAHFIDQTLk.[A]	N-Term(iTRAQ4plex); K1(iTRAQ4	1	1
[K].gVTSVSQIFHSPDLAIR.[D]	N-Term(iTRAQ4plex)	1	1
[K].nTNNkISR.[L]	N-Term(iTRAQ4plex); K5(iTRAQ4	1	1
[K].tNLESILSYPk.[D]	N-Term(iTRAQ4plex); K11(iTRAQ4	1	1
[K].gVTSVSQIFHSPDLAIR.[D]	N-Term(iTRAQ4plex)	1	1
[K].vPMMNSk.[K]	N-Term(iTRAQ4plex); K7(iTRAQ4	1	1

[R].LLDSLPSDTR.[L]		1	1
[K].dFTcVHQALk.[G]	N-Term(iTRAQ4plex); C4(Carbam	1	1
[R].mEPFHFk.[N]	N-Term(iTRAQ4plex); K7(iTRAQ4	1	1
[R].LEDMEQALSPSVFk.[A]	K14(iTRAQ4plex)	1	1
[R].lVLLNAIYLSAk.[W]	N-Term(iTRAQ4plex); K12(iTRAQ	1	1
[R].mEPFHFk.[N]	N-Term(iTRAQ4plex); K7(iTRAQ4	1	1
[R].TLYSSSPR.[V]		1	1
[K].hRLEDMEQALSPSVFk.[A]	N-Term(iTRAQ4plex); K16(iTRAQ	1	1
[R].lEDmEQALSPSVFk.[A]	N-Term(iTRAQ4plex); M4(Oxidat	1	1
[K].DFTcVHQALk.[G]	C4(Carbamidomethyl); K10(iTRA	1	1
[R].lEDMEQALSPSVFK.[A]	N-Term(iTRAQ4plex)	1	1
[R].TLYSSSPR.[V]		1	1
[K].gVTSVSQIFHSPDLAIR.[D]	N-Term(iTRAQ4plex)	1	1
[K].tNLESILSYPk.[D]	N-Term(iTRAQ4plex); K11(iTRAQ	1	1
[R].TLYSSSPR.[V]		1	1
[R].cLPVcGkPVNPVEQR.[Q]	N-Term(iTRAQ4plex); C1(Carbam	1	1
[K].QRPPDLDTSSNAVDLLFFTDESGDSR.[G]		1	1
[R].iQYYcHEPYYk.[M]	N-Term(iTRAQ4plex); C5(Carbam	1	1
[R].iQYYcHEPYYk.[M]	N-Term(iTRAQ4plex); C5(Carbam	1	1
[K].lVFQQFDLEPSEGcFYDYVk.[I]	N-Term(iTRAQ4plex); C14(Carba	1	1
[R].lPVANPQAcENWLR.[G]	N-Term(iTRAQ4plex); C9(Carbam	1	1
[R].lPVANPQAcENWLR.[G]	N-Term(iTRAQ4plex); C9(Carbam	1	1
[R].fcGQLGSPLGNPPGk.[K]	N-Term(iTRAQ4plex); C2(Carbam	1	1
[R].fcGQLGSPLGNPPGk.[K]	N-Term(iTRAQ4plex); C2(Carbam	1	1
[K].qGYQLIEGNQVLHSFTAVcQDDGTWHR.[	N-Term(iTRAQ4plex); C19(Carba	1	1
[K].gFLAYYQAVDLDEcASR.[S]	N-Term(iTRAQ4plex); C14(Carba	1	1
[K].gFLAYYQAVDLDEcASR.[S]	N-Term(iTRAQ4plex); C14(Carba	1	1
[K].qRPPDLDTSSNAVDLLFFTDESGDSR.[G]	N-Term(iTRAQ4plex)	1	1
[K].qRPPDLDTSSNAVDLLFFTDESGDSR.[G]	N-Term(iTRAQ4plex)	1	1
[K].lVFQQFDLEPSEGcFYDYVk.[I]	N-Term(iTRAQ4plex); C14(Carba	1	1
[K].lVFQQFDLEPSEGcFYDYVk.[I]	N-Term(iTRAQ4plex); C14(Carba	1	1
[K].kEFMSQGNk.[M]	N-Term(iTRAQ4plex); K1(iTRAQ4	1	1
[K].mGNFPWQVFTNIHGR.[G]	N-Term(iTRAQ4plex)	1	1
[K].mGNFPWQVFTNIHGR.[G]	N-Term(iTRAQ4plex)	1	1
[R].gGGALLGDR.[W]	N-Term(iTRAQ4plex)	2	2
[R].iQYYcHEPYYk.[M]	N-Term(iTRAQ4plex); C5(Carbam	1	1
[R].dYFIATck.[Q]	N-Term(iTRAQ4plex); C7(Carbam	1	1
[K].tLDEFTIIQNLQPQYQFR.[D]	N-Term(iTRAQ4plex)	1	1
[R].eSEQGVYTcTAQGIWk.[N]	N-Term(iTRAQ4plex); C9(Carbam	1	1
[R].wILTAAHTLYPk.[DE]	N-Term(iTRAQ4plex); K12(iTRAQ	1	1
[R].wILTAAHTLYPk.[DE]	N-Term(iTRAQ4plex); K12(iTRAQ	1	1
[R].eSEQGVYTcTAQGIWk.[N]	N-Term(iTRAQ4plex); C9(Carbam	1	1
[R].gLTLHLk.[F]	N-Term(iTRAQ4plex); K7(iTRAQ4	1	1
[R].yTTTMGVNTYk.[A]	N-Term(iTRAQ4plex); K11(iTRAQ	1	1
[R].lPVANPQAcENWLR.[G]	N-Term(iTRAQ4plex); C9(Carbam	1	1
[K].vLNYVDWIk.[K]	N-Term(iTRAQ4plex); K9(iTRAQ4	1	1
[R].gYGFYTk.[V]	N-Term(iTRAQ4plex); K7(iTRAQ4	1	1
[K].gFLAYYQAVDLDEcASR.[S]	N-Term(iTRAQ4plex); C14(Carba	1	1
[R].YTTEIIk.[C]	K7(iTRAQ4plex)	1	1
[K].nIGEFcGk.[Q]	N-Term(iTRAQ4plex); C6(Carbam	1	1
[R].wILTAAHTLYPk.[DE]	N-Term(iTRAQ4plex); K12(iTRAQ	1	1
[R].vSVHPDYR.[Q]	N-Term(iTRAQ4plex)	1	1

[R].dYFIATck.[Q]	N-Term(iTRAQ4plex); C7(Carbam	1	1
[R].wILTAAHTLYPk.[DE]	N-Term(iTRAQ4plex); K12(iTRAQ	1	1
[R].aATVGSLAGQPLQER.[A]	N-Term(iTRAQ4plex)	1	1
[R].LGPLVEQGR.[V]		1	1
[K].SWFEPLVEDMQR.[Q]		1	1
[K].SWFEPLVEDMQR.[Q]		1	1
[R].gEVQAMLGQSTEELR.[V]	N-Term(iTRAQ4plex)	1	1
[R].gEVQAMLGQSTEELR.[V]	N-Term(iTRAQ4plex)	1	1
[R].gEVQAMLGQSTEELR.[V]	N-Term(iTRAQ4plex)	1	1
[R].lQAEAFQAR.[L]	N-Term(iTRAQ4plex)	1	1
[R].aATVGSLAGQPLQER.[A]	N-Term(iTRAQ4plex)	1	1
[R].aATVGSLAGQPLQER.[A]	N-Term(iTRAQ4plex)	1	1
[R].qWAGLVEk.[V]	N-Term(iTRAQ4plex); K8(iTRAQ4	1	1
[R].qWAGLVEk.[V]	N-Term(iTRAQ4plex); K8(iTRAQ4	1	1
[R].lAVYQAGAR.[E]	N-Term(iTRAQ4plex)	1	1
[R].aATVGSLAGQPLQER.[A]	N-Term(iTRAQ4plex)	1	1
[R].LQAEAFQAR.[L]		1	1
[K].sWFEPLVEDMQR.[Q]	N-Term(iTRAQ4plex)	1	1
[K].sWFEPLVEDMQR.[Q]	N-Term(iTRAQ4plex)	1	1
[R].WVQTLSEQVQEELLSSQVTQELR.[A]		1	1
[R].akLEEQAQQIR.[L]	N-Term(iTRAQ4plex); K2(iTRAQ4	1	1
[R].LGADMEDVcGR.[L]	C9(Carbamidomethyl)	1	1
[R].aLMDETMk.[E]	N-Term(iTRAQ4plex); K8(iTRAQ4	1	1
[K].SELEEQLTPVAEETR.[A]		1	1
[K].sELEEQLTPVAEETR.[A]	N-Term(iTRAQ4plex)	1	1
[K].sELEEQLTPVAEETR.[A]	N-Term(iTRAQ4plex)	1	1
[K].sELEEQLTPVAEETR.[A]	N-Term(iTRAQ4plex)	1	1
[K].sELEEQLTPVAEETR.[A]	N-Term(iTRAQ4plex)	1	1
[R].gEVQAmLGQSTEELR.[V]	N-Term(iTRAQ4plex); M6(Oxidat	1	1
[R].gEVQAmLGQSTEELR.[V]	N-Term(iTRAQ4plex); M6(Oxidat	1	1
[R].dADDLQk.[R]	N-Term(iTRAQ4plex); K7(iTRAQ4	1	1
[K].vQAAVGTSAAPVPSDNH.[-]	N-Term(iTRAQ4plex)	1	1
[R].qQTEWQSGQR.[W]	N-Term(iTRAQ4plex)	1	1
[R].qQTEWQSGQR.[W]	N-Term(iTRAQ4plex)	1	1
[K].LEEQAQQIR.[L]		1	1
[K].vEQAVETEPEPELR.[Q]	N-Term(iTRAQ4plex)	1	1
[K].vEQAVETEPEPELR.[Q]	N-Term(iTRAQ4plex)	1	1
[K].vEQAVETEPEPELR.[Q]	N-Term(iTRAQ4plex)	1	1
[R].dADDLQk.[R]	N-Term(iTRAQ4plex); K7(iTRAQ4	1	1
[R].ARMEEMGSR.[T]		1	1
[R].dRLDEVk.[E]	N-Term(iTRAQ4plex); K7(iTRAQ4	1	1
[R].LSkELQAAQAR.[L]	K3(iTRAQ4plex)	1	1
[K].sELEEQLTPVAEETR.[A]	N-Term(iTRAQ4plex)	1	1
[R].dRLDEVk.[E]	N-Term(iTRAQ4plex); K7(iTRAQ4	1	1
[R].lGADmEDVcGR.[L]	N-Term(iTRAQ4plex); M5(Oxidat	1	1
[R].lSkELQAAQAR.[L]	N-Term(iTRAQ4plex); K3(iTRAQ4	1	1
[R].akLEEQAQQIR.[L]	N-Term(iTRAQ4plex); K2(iTRAQ4	1	1
[R].lQAEAFQAR.[L]	N-Term(iTRAQ4plex)	1	1
[R].AkLEEQAQQIR.[L]	K2(iTRAQ4plex)	1	1
[R].lGPLVEQGR.[V]	N-Term(iTRAQ4plex)	1	1
[R].qWAGLVEk.[V]	N-Term(iTRAQ4plex); K8(iTRAQ4	1	1
[R].LAVYQAGAR.[E]		1	1

[R].dADDLQk.[R]	N-Term(iTRAQ4plex); K7(iTRAQ4	1	1
[R].dRLDEVk.[E]	N-Term(iTRAQ4plex); K7(iTRAQ4	1	1
[R].aLmDETMk.[E]	N-Term(iTRAQ4plex); M3(Oxidat	1	1
[R].qWAGLVEk.[V]	N-Term(iTRAQ4plex); K8(iTRAQ4	1	1
[K].eLQAAQAR.[L]	N-Term(iTRAQ4plex)	1	1
[K].lEEQAQQIR.[L]	N-Term(iTRAQ4plex)	1	1
[R].QQTEWQSGQR.[W]		1	1
[K].lEEQAQQIR.[L]	N-Term(iTRAQ4plex)	1	1
[R].LGPLVEQGRVR.[A]		1	1
[R].lGPLVEQGR.[V]	N-Term(iTRAQ4plex)	1	1
[R].AATVGSLAGQPLQER.[A]		1	1
[R].dADDLQk.[R]	N-Term(iTRAQ4plex); K7(iTRAQ4	1	1
[R].lGPLVEQGR.[V]	N-Term(iTRAQ4plex)	1	1
[K].SELEEQLTPVAEETR.[A]		1	1
[R].dADDLQk.[R]	N-Term(iTRAQ4plex); K7(iTRAQ4	1	1
[R].dRLDEVk.[E]	N-Term(iTRAQ4plex); K7(iTRAQ4	1	1
[R].aLmDETMk.[E]	N-Term(iTRAQ4plex); M3(Oxidat	1	1
[R].qQTEWQSGQR.[W]	N-Term(iTRAQ4plex)	1	1
[K].vEQAVETEPEPELR.[Q]	N-Term(iTRAQ4plex)	1	1
[R].LAVYQAGAR.[E]		1	1
[R].dADDLQk.[R]	N-Term(iTRAQ4plex); K7(iTRAQ4	1	1
[R].aLMDETmk.[E]	N-Term(iTRAQ4plex); M7(Oxidat	1	1
[R].dADDLQk.[R]	N-Term(iTRAQ4plex); K7(iTRAQ4	1	1
[R].aLMDETmk.[E]	N-Term(iTRAQ4plex); M7(Oxidat	1	1
[K].sELEEQLTPVAEETR.[A]	N-Term(iTRAQ4plex)	1	1
[K].eLQAAQAR.[L]	N-Term(iTRAQ4plex)	1	1
[R].aATVGSLAGQPLQER.[A]	N-Term(iTRAQ4plex)	1	1
[R].tPWHVTIkPk.[S]	N-Term(iTRAQ4plex); K8(iTRAQ4	1	1
[R].tPWHVTIkPk.[S]	N-Term(iTRAQ4plex); K8(iTRAQ4	1	1
[R].tPWHVTIkPk.[S]	N-Term(iTRAQ4plex); K8(iTRAQ4	1	1
[R].dFHINLFR.[M]	N-Term(iTRAQ4plex)	1	1
[K].mGVEWTScAEVVSQEk.[T]	N-Term(iTRAQ4plex); C8(Carbam	1	1
[R].eVVTDQFLcSGTQEDESPckGESGGAVFLE	N-Term(iTRAQ4plex); C9(Carbam	1	1
[R].tPWHVTIkPk.[S]	N-Term(iTRAQ4plex); K8(iTRAQ4	1	1
[R].hAIILLTDGk.[S]	N-Term(iTRAQ4plex); K10(iTRAQ	1	1
[R].hAIILLTDGk.[S]	N-Term(iTRAQ4plex); K10(iTRAQ	1	1
[R].hAIILLTDGk.[S]	N-Term(iTRAQ4plex); K10(iTRAQ	1	1
[R].hAIILLTDGk.[S]	N-Term(iTRAQ4plex); K10(iTRAQ	1	1
[K].aVISPGFDVFAk.[K]	N-Term(iTRAQ4plex); K12(iTRAQ	1	1
[K].aVISPGFDVFAk.[K]	N-Term(iTRAQ4plex); K12(iTRAQ	1	1
[R].hAIILLTDGk.[S]	N-Term(iTRAQ4plex); K10(iTRAQ	1	1
[K].kNQGILEFYGDDIALLk.[L]	N-Term(iTRAQ4plex); K1(iTRAQ4	1	1
[K].nQGILEFYGDDIALLk.[L]	N-Term(iTRAQ4plex); K16(iTRAQ	1	1
[K].nQGILEFYGDDIALLk.[L]	N-Term(iTRAQ4plex); K16(iTRAQ	1	1
[K].rNDYLDIYAIGVGk.[L]	N-Term(iTRAQ4plex); K14(iTRAQ	1	1
[R].eVVTDQFLcSGTQEDESPck.[G]	N-Term(iTRAQ4plex); C9(Carbam	1	1
[R].eVVTDQFLcSGTQEDESPck.[G]	N-Term(iTRAQ4plex); C9(Carbam	1	1
[R].dHENELLNk.[Q]	N-Term(iTRAQ4plex); K9(iTRAQ4	1	1
[R].dHENELLNk.[Q]	N-Term(iTRAQ4plex); K9(iTRAQ4	1	1
[R].eLNELGSk.[K]	N-Term(iTRAQ4plex); K8(iTRAQ4	1	1
[R].dGNDHSLWR.[V]	N-Term(iTRAQ4plex)	1	1
[K].sSGQWQTPGATR.[S]	N-Term(iTRAQ4plex)	1	1

[K].sSGQWQTPGATR.[S]	N-Term(iTRAQ4plex)	1	1
[K].aLHQVFEHMLDVSk.[L]	N-Term(iTRAQ4plex); K14(iTRAQ	1	1
[K].aLHQVFEHMLDVSk.[L]	N-Term(iTRAQ4plex); K14(iTRAQ	1	1
[K].aLHQVFEHMLDVSk.[L]	N-Term(iTRAQ4plex); K14(iTRAQ	1	1
[K].vLMSVLNDNSR.[D]	N-Term(iTRAQ4plex)	1	1
[R].fFQVGLVSWGLYNPcLGSADk.[N]	N-Term(iTRAQ4plex); C15(Carbar	1	1
[R].tPWHVTIkPk.[S]	N-Term(iTRAQ4plex); K8(iTRAQ4	1	1
[K].gESGGAVFLER.[R]	N-Term(iTRAQ4plex)	1	1
[K].aVISPGFDVFAk.[K]	N-Term(iTRAQ4plex); K12(iTRAQ	1	1
[R].dGNDHSLWR.[V]	N-Term(iTRAQ4plex)	1	1
[R].hAFILQDTk.[A]	N-Term(iTRAQ4plex); K9(iTRAQ4	1	1
[R].HAIILLTDGk.[S]	K10(iTRAQ4plex)	1	1
[R].dFHINLFR.[M]	N-Term(iTRAQ4plex)	1	1
[R].tPWHVTIkPk.[S]	N-Term(iTRAQ4plex); K8(iTRAQ4	1	1
[K].sNMGGSPk.[T]	N-Term(iTRAQ4plex); K8(iTRAQ4	1	1
[K].vLMSVLNDNSR.[D]	N-Term(iTRAQ4plex)	1	1
[R].DHENELLNk.[Q]	K9(iTRAQ4plex)	1	1
[R].dHENELLNk.[Q]	N-Term(iTRAQ4plex); K9(iTRAQ4	1	1
[K].ALHQVFEHMLDVSk.[L]	K14(iTRAQ4plex)	1	1
[R].hAFILQDTk.[A]	N-Term(iTRAQ4plex); K9(iTRAQ4	1	1
[R].dHENELLNk.[Q]	N-Term(iTRAQ4plex); K9(iTRAQ4	1	1
[R].hAFILQDTk.[A]	N-Term(iTRAQ4plex); K9(iTRAQ4	1	1
[R].tPWHVTIkPk.[S]	N-Term(iTRAQ4plex); K8(iTRAQ4	1	1
[R].eILNINQk.[R]	N-Term(iTRAQ4plex); K8(iTRAQ4	1	1
[R].hAIILLTDGk.[S]	N-Term(iTRAQ4plex); K10(iTRAQ	1	1
[R].hAIILLTDGk.[S]	N-Term(iTRAQ4plex); K10(iTRAQ	1	1
[R].hAFILQDTk.[A]	N-Term(iTRAQ4plex); K9(iTRAQ4	1	1
[K].gESGGAVFLER.[R]	N-Term(iTRAQ4plex)	1	1
[R].TPWHVTIkPk.[S]	K8(iTRAQ4plex); K10(iTRAQ4ple	1	1
[R].dMTEVISSLENANYk.[D]	N-Term(iTRAQ4plex); K15(iTRAQ	1	1
[R].dMTEVISSLENANYk.[D]	N-Term(iTRAQ4plex); K15(iTRAQ	1	1
[K].VLMSVLNDNSR.[D]		1	1
[R].hAIILLTDGk.[S]	N-Term(iTRAQ4plex); K10(iTRAQ	1	1
[R].hAFILQDTk.[A]	N-Term(iTRAQ4plex); K9(iTRAQ4	1	1
[R].dMTEVISSLENANYk.[D]	N-Term(iTRAQ4plex); K15(iTRAQ	1	1
[R].dGNDHSLWR.[V]	N-Term(iTRAQ4plex)	1	1
[R].dFHINLFR.[M]	N-Term(iTRAQ4plex)	1	1
[R].eVVTDQFLcSGTQEDESPck.[G]	N-Term(iTRAQ4plex); C9(Carbam	1	1
[K].aVISPGFDVFAk.[K]	N-Term(iTRAQ4plex); K12(iTRAQ	1	1
[K].eVIITGIQTQGAk.[H]	N-Term(iTRAQ4plex); K13(iTRAQ	1	1
[K].eVIITGIQTQGAk.[H]	N-Term(iTRAQ4plex); K13(iTRAQ	1	1
[K].aDkPLSIHPQGIR.[Y]	N-Term(iTRAQ4plex); K3(iTRAQ4	1	1
[R].aADIEQQAVFAVFDENk.[S]	N-Term(iTRAQ4plex); K17(iTRAQ	1	1
[K].iTAIITQGck.[S]	N-Term(iTRAQ4plex); C9(Carbam	1	1
[R].gEYEEHLGILGPIIR.[A]	N-Term(iTRAQ4plex)	1	1
[K].gTLTEGGTQk.[T]	N-Term(iTRAQ4plex); K10(iTRAQ	1	1
[K].nFFNPPIISR.[F]	N-Term(iTRAQ4plex)	1	1
[R].eTDIEDSDDIPEDTTYk.[K]	N-Term(iTRAQ4plex); K17(iTRAQ	1	1
[R].eTDIEDSDDIPEDTTYk.[K]	N-Term(iTRAQ4plex); K17(iTRAQ	1	1
[R].sEAYNTFSER.[R]	N-Term(iTRAQ4plex)	1	1
[K].kITAIITQGck.[S]	N-Term(iTRAQ4plex); K1(iTRAQ4	1	1
[K].eDGILGPIIR.[A]	N-Term(iTRAQ4plex)	1	1

[K].fcENPDEVkR.[D]	N-Term(iTRAQ4plex); C2(Carbam	1	1
[K].iTAIITQGck.[S]	N-Term(iTRAQ4plex); C9(Carbam	1	1
[K].mDDAVAPGR.[E]	N-Term(iTRAQ4plex)	1	1
[K].tYEDDSPEWFk.[E]	N-Term(iTRAQ4plex); K11(iTRAQ4	1	1
[K].hSLVLHk.[S]	N-Term(iTRAQ4plex); K7(iTRAQ4	1	1
[R].lAAALGIR.[S]	N-Term(iTRAQ4plex)	1	1
[K].fTVNNLAEPQk.[A]	N-Term(iTRAQ4plex); K11(iTRAQ4	1	1
[K].rHEDTLTLFPMR.[G]	N-Term(iTRAQ4plex)	1	1
[K].fcENPDEVk.[R]	N-Term(iTRAQ4plex); C2(Carbam	1	1
[R].sQHLDNFSNQIGk.[H]	N-Term(iTRAQ4plex); K13(iTRAQ4	1	1
[R].gEYEEHLGILGPIIR.[A]	N-Term(iTRAQ4plex)	1	1
[R].lTSSEMk.[K]	N-Term(iTRAQ4plex); K7(iTRAQ4	1	1
[R].hEDTLTLFPMR.[G]	N-Term(iTRAQ4plex)	1	1
[R].gEcQAEGVLFFQGDR.[E]	N-Term(iTRAQ4plex); C3(Carbam	1	1
[R].gEcQAEGVLFFQGDR.[E]	N-Term(iTRAQ4plex); C3(Carbam	1	1
[R].gEcQAEGVLFFQGDR.[E]	N-Term(iTRAQ4plex); C3(Carbam	1	1
[R].YYcFQGNQFLR.[F]	C3(Carbamidomethyl)	1	1
[K].SGAQATWTELPWPHEk.[V]	K16(iTRAQ4plex)	1	1
[K].SGAQATWTELPWPHEk.[V]	K16(iTRAQ4plex)	1	1
[R].qGHNSVFLIk.[G]	N-Term(iTRAQ4plex); K10(iTRAQ4	1	1
[R].QGHNSVFLIk.[G]	K10(iTRAQ4plex)	1	1
[R].qGHNSVFLIk.[G]	N-Term(iTRAQ4plex); K10(iTRAQ4	1	1
[R].qGHNSVFLIk.[G]	N-Term(iTRAQ4plex); K10(iTRAQ4	1	1
[R].qGHNSVFLIk.[G]	N-Term(iTRAQ4plex); K10(iTRAQ4	1	1
[R].QGHNSVFLIk.[G]	K10(iTRAQ4plex)	1	1
[R].qGHNSVFLIk.[G]	N-Term(iTRAQ4plex); K10(iTRAQ4	1	1
[R].gEcQAEGVLFFQGDR.[E]	N-Term(iTRAQ4plex); C3(Carbam	1	1
[R].GEcQAEGVLFFQGDR.[E]	C3(Carbamidomethyl)	1	1
[R].gEcQAEGVLFFQGDR.[E]	N-Term(iTRAQ4plex); C3(Carbam	1	1
[R].gEcQAEGVLFFQGDR.[E]	N-Term(iTRAQ4plex); C3(Carbam	1	1
[R].gEcQAEGVLFFQGDR.[E]	N-Term(iTRAQ4plex); C3(Carbam	1	1
[R].gEcQAEGVLFFQGDR.[E]	N-Term(iTRAQ4plex); C3(Carbam	1	1
[R].gEcQAEGVLFFQGDR.[E]	N-Term(iTRAQ4plex); C3(Carbam	1	1
[R].gEcQAEGVLFFQGDR.[E]	N-Term(iTRAQ4plex); C3(Carbam	1	1
[R].gEcQAEGVLFFQGDR.[E]	N-Term(iTRAQ4plex); C3(Carbam	1	1
[R].gEcQAEGVLFFQGDR.[E]	N-Term(iTRAQ4plex); C3(Carbam	1	1
[R].GEcQAEGVLFFQGDR.[E]	C3(Carbamidomethyl)	1	1
[R].gEcQAEGVLFFQGDR.[E]	N-Term(iTRAQ4plex); C3(Carbam	1	1
[R].gEcQAEGVLFFQGDR.[E]	N-Term(iTRAQ4plex); C3(Carbam	1	1
[R].gEcQAEGVLFFQGDR.[E]	N-Term(iTRAQ4plex); C3(Carbam	1	1
[R].gEcQAEGVLFFQGDR.[E]	N-Term(iTRAQ4plex); C3(Carbam	1	1
[R].gEcQAEGVLFFQGDR.[E]	N-Term(iTRAQ4plex); C3(Carbam	1	1
[R].gEcQAEGVLFFQGDR.[E]	N-Term(iTRAQ4plex); C3(Carbam	1	1
[K].gGYTLVSGYPk.[R]	N-Term(iTRAQ4plex); K11(iTRAQ4	1	1
[K].gGYTLVSGYPk.[R]	N-Term(iTRAQ4plex); K11(iTRAQ4	1	1
[K].gGYTLVSGYPk.[R]	N-Term(iTRAQ4plex); K11(iTRAQ4	1	1
[K].gGYTLVSGYPk.[R]	N-Term(iTRAQ4plex); K11(iTRAQ4	1	1
[K].gGYTLVSGYPk.[R]	N-Term(iTRAQ4plex); K11(iTRAQ4	1	1
[K].gGYTLVSGYPk.[R]	N-Term(iTRAQ4plex); K11(iTRAQ4	1	1
[K].gGYTLVSGYPk.[R]	N-Term(iTRAQ4plex); K11(iTRAQ4	1	1
[R].gEcQAEGVLFFQGDR.[E]	N-Term(iTRAQ4plex); C3(Carbam	1	1
[K].eVGTPHGIILDSVDAAFIcPGSSR.[L]	N-Term(iTRAQ4plex); C19(Carbar	1	1

[K].lLQDEFPGIPSPLDAAVEcHR.[G]	N-Term(iTRAQ4plex); C19(Carbar	1	1
[K].eVGTPHGIILDSVDAAFIcPGSSR.[L]	N-Term(iTRAQ4plex); C19(Carbar	1	1
[K].LLQDEFPGIPSPLDAAVEcHR.[G]	C19(Carbamidomethyl)	1	1
[K].eVGTPHGIILDSVDAAFIcPGSSR.[L]	N-Term(iTRAQ4plex); C19(Carbar	1	1
[K].LLQDEFPGIPSPLDAAVEcHR.[G]	C19(Carbamidomethyl)	1	1
[K].eVGTPHGIILDSVDAAFIcPGSSR.[L]	N-Term(iTRAQ4plex); C19(Carbar	1	1
[K].eVGTPHGIILDSVDAAFIcPGSSR.[L]	N-Term(iTRAQ4plex); C19(Carbar	1	1
[K].LLQDEFPGIPSPLDAAVEcHR.[G]	C19(Carbamidomethyl)	1	1
[K].eVGTPHGIILDSVDAAFIcPGSSR.[L]	N-Term(iTRAQ4plex); C19(Carbar	1	1
[K].eVGTPHGIILDSVDAAFIcPGSSR.[L]	N-Term(iTRAQ4plex); C19(Carbar	1	1
[K].eVGTPHGIILDSVDAAFIcPGSSR.[L]	N-Term(iTRAQ4plex); C19(Carbar	1	1
[K].eVGTPHGIILDSVDAAFIcPGSSR.[L]	N-Term(iTRAQ4plex); C19(Carbar	1	1
[K].eVGTPHGIILDSVDAAFIcPGSSR.[L]	N-Term(iTRAQ4plex); C19(Carbar	1	1
[K].EVGTPHGIILDSVDAAFIcPGSSR.[L]	C19(Carbamidomethyl)	1	1
[K].eVGTPHGIILDSVDAAFIcPGSSR.[L]	N-Term(iTRAQ4plex); C19(Carbar	1	1
[K].eVGTPHGIILDSVDAAFIcPGSSR.[L]	N-Term(iTRAQ4plex); C19(Carbar	1	1
[K].eVGTPHGIILDSVDAAFIcPGSSR.[L]	N-Term(iTRAQ4plex); C19(Carbar	1	1
[R].eWFWDLATGTmk.[E]	N-Term(iTRAQ4plex); M11(Oxida	1	1
[K].lLQDEFPGIPSPLDAAVEcHR.[G]	N-Term(iTRAQ4plex); C19(Carbar	1	1
[K].lLQDEFPGIPSPLDAAVEcHR.[G]	N-Term(iTRAQ4plex); C19(Carbar	1	1
[K].lLQDEFPGIPSPLDAAVEcHR.[G]	N-Term(iTRAQ4plex); C19(Carbar	1	1
[K].lLQDEFPGIPSPLDAAVEcHR.[G]	N-Term(iTRAQ4plex); C19(Carbar	1	1
[K].lLQDEFPGIPSPLDAAVEcHR.[G]	N-Term(iTRAQ4plex); C19(Carbar	1	1
[K].lLQDEFPGIPSPLDAAVEcHR.[G]	N-Term(iTRAQ4plex); C19(Carbar	1	1
[K].lLQDEFPGIPSPLDAAVEcHR.[G]	N-Term(iTRAQ4plex); C19(Carbar	1	1
[K].lLQDEFPGIPSPLDAAVEcHR.[G]	N-Term(iTRAQ4plex); C19(Carbar	1	1
[R].dGWHSWPIAHQWPQGPSAVDAAFSWE	N-Term(iTRAQ4plex); K28(iTRAQ	1	1
[K].lLQDEFPGIPSPLDAAVEcHR.[G]	N-Term(iTRAQ4plex); C19(Carbar	1	1
[K].lLQDEFPGIPSPLDAAVEcHR.[G]	N-Term(iTRAQ4plex); C19(Carbar	1	1
[K].eVGTPHGIILDSVDAAFIcPGSSR.[L]	N-Term(iTRAQ4plex); C19(Carbar	1	1
[R].eWFWDLATGTmk.[E]	N-Term(iTRAQ4plex); M11(Oxida	1	1
[K].eVGTPHGIILDSVDAAFIcPGSSR.[L]	N-Term(iTRAQ4plex); C19(Carbar	1	1
[K].eVGTPHGIILDSVDAAFIcPGSSR.[L]	N-Term(iTRAQ4plex); C19(Carbar	1	1
[R].qGHNSVFLIk.[G]	N-Term(iTRAQ4plex); K10(iTRAQ	1	1
[R].qGHNSVFLIk.[G]	N-Term(iTRAQ4plex); K10(iTRAQ	1	1
[K].EVGTPHGIILDSVDAAFIcPGSSR.[L]	C19(Carbamidomethyl)	1	1
[K].eVGTPHGIILDSVDAAFIcPGSSR.[L]	N-Term(iTRAQ4plex); C19(Carbar	1	1
[K].eVGTPHGIILDSVDAAFIcPGSSR.[L]	N-Term(iTRAQ4plex); C19(Carbar	1	1
[K].eVGTPHGIILDSVDAAFIcPGSSR.[L]	N-Term(iTRAQ4plex); C19(Carbar	1	1
[K].sGAQATWTELPWPHEk.[V]	N-Term(iTRAQ4plex); K16(iTRAQ	1	1
[K].sGAQATWTELPWPHEk.[V]	N-Term(iTRAQ4plex); K16(iTRAQ	1	1
[K].sGAQATWTELPWPHEk.[V]	N-Term(iTRAQ4plex); K16(iTRAQ	1	1
[R].yYcFQGNQFLR.[F]	N-Term(iTRAQ4plex); C3(Carbam	1	1
[R].yYcFQGNQFLR.[F]	N-Term(iTRAQ4plex); C3(Carbam	1	1
[R].yYcFQGNQFLR.[F]	N-Term(iTRAQ4plex); C3(Carbam	1	1
[R].yYcFQGNQFLR.[F]	N-Term(iTRAQ4plex); C3(Carbam	1	1
[K].eVGTPHGIILDSVDAAFIcPGSSR.[L]	N-Term(iTRAQ4plex); C19(Carbar	1	1
[K].eVGTPHGIILDSVDAAFIcPGSSR.[L]	N-Term(iTRAQ4plex); C19(Carbar	1	1
[K].eVGTPHGIILDSVDAAFIcPGSSR.[L]	N-Term(iTRAQ4plex); C19(Carbar	1	1
[K].sLGPNScSANGPGLYLIHGPNLYcYSDVEk.	N-Term(iTRAQ4plex); C7(Carbam	1	1
[K].eVGTPHGIILDSVDAAFIcPGSSR.[L]	N-Term(iTRAQ4plex); C19(Carbar	1	1
[K].eVGTPHGIILDSVDAAFIcPGSSR.[L]	N-Term(iTRAQ4plex); C19(Carbar	1	1

[K].eVGTPHGIILDSVDAAFIcPGSSR.[L]	N-Term(iTRAQ4plex); C19(Carbar	1	1
[K].eVGTPHGIILDSVDAAFIcPGSSR.[L]	N-Term(iTRAQ4plex); C19(Carbar	1	1
[K].eVGTPHGIILDSVDAAFIcPGSSR.[L]	N-Term(iTRAQ4plex); C19(Carbar	1	1
[K].sGAQATWTELPWPHEk.[V]	N-Term(iTRAQ4plex); K16(iTRAQ	1	1
[K].sGAQATWTELPWPHEk.[V]	N-Term(iTRAQ4plex); K16(iTRAQ	1	1
[K].SGAQATWTELPWPHEk.[V]	K16(iTRAQ4plex)	1	1
[K].SGAQATWTELPWPHEk.[V]	K16(iTRAQ4plex)	1	1
[K].sGAQATWTELPWPHEk.[V]	N-Term(iTRAQ4plex); K16(iTRAQ	1	1
[K].SGAQATWTELPWPHEk.[V]	K16(iTRAQ4plex)	1	1
[K].sGAQATWTELPWPHEk.[V]	N-Term(iTRAQ4plex); K16(iTRAQ	1	1
[K].sGAQATWTELPWPHEk.[V]	N-Term(iTRAQ4plex); K16(iTRAQ	1	1
[K].SGAQATWTELPWPHEk.[V]	K16(iTRAQ4plex)	1	1
[K].sGAQATWTELPWPHEk.[V]	N-Term(iTRAQ4plex); K16(iTRAQ	1	1
[K].sGAQATWTELPWPHEk.[V]	N-Term(iTRAQ4plex); K16(iTRAQ	1	1
[K].sGAQATWTELPWPHEk.[V]	N-Term(iTRAQ4plex); K16(iTRAQ	1	1
[K].sGAQATWTELPWPHEk.[V]	N-Term(iTRAQ4plex); K16(iTRAQ	1	1
[K].sGAQATWTELPWPHEk.[V]	N-Term(iTRAQ4plex); K16(iTRAQ	1	1
[R].EWFWDLATGTMk.[E]	K12(iTRAQ4plex)	1	1
[K].sGAQATWTELPWPHEk.[V]	N-Term(iTRAQ4plex); K16(iTRAQ	1	1
[K].sGAQATWTELPWPHEk.[V]	N-Term(iTRAQ4plex); K16(iTRAQ	1	1
[K].nFPSPVDAAFR.[Q]	N-Term(iTRAQ4plex)	1	1
[K].nFPSPVDAAFR.[Q]	N-Term(iTRAQ4plex)	1	1
[K].NFPSPVDAAFR.[Q]		1	1
[K].NFPSPVDAAFR.[Q]		1	1
[K].nFPSPVDAAFR.[Q]	N-Term(iTRAQ4plex)	1	1
[K].nFPSPVDAAFR.[Q]	N-Term(iTRAQ4plex)	1	1
[K].nFPSPVDAAFR.[Q]	N-Term(iTRAQ4plex)	1	1
[K].nFPSPVDAAFR.[Q]	N-Term(iTRAQ4plex)	1	1
[K].gGYTLVSGYPk.[R]	N-Term(iTRAQ4plex); K11(iTRAQ	1	1
[K].gGYTLVSGYPk.[R]	N-Term(iTRAQ4plex); K11(iTRAQ	1	1
[R].sWPAVGNcSSALR.[W]	N-Term(iTRAQ4plex); C8(Carbam	1	1
[K].vWVYPPEk.[K]	N-Term(iTRAQ4plex); K8(iTRAQ4	1	1
[R].dGWHSWPIAHQWPQGPSAVDAAFSWE	N-Term(iTRAQ4plex); K28(iTRAQ	1	1
[R].dGWHSWPIAHQWPQGPSAVDAAFSWE	N-Term(iTRAQ4plex); K28(iTRAQ	1	1
[K].eVGTPHGIILDSVDAAFIcPGSSR.[L]	N-Term(iTRAQ4plex); C19(Carbar	1	1
[K].lLQDEFPGIPSPLDAAVEcHR.[G]	N-Term(iTRAQ4plex); C19(Carbar	1	1
[K].lLQDEFPGIPSPLDAAVEcHR.[G]	N-Term(iTRAQ4plex); C19(Carbar	1	1
[K].LLQDEFPGIPSPLDAAVEcHR.[G]	C19(Carbamidomethyl)	1	1
[R].dGWHSWPIAHQWPQGPSAVDAAFSWE	N-Term(iTRAQ4plex); K28(iTRAQ	1	1
[K].LLQDEFPGIPSPLDAAVEcHR.[G]	C19(Carbamidomethyl)	1	1
[R].dGWHSWPIAHQWPQGPSAVDAAFSWE	N-Term(iTRAQ4plex); K28(iTRAQ	1	1
[K].LLQDEFPGIPSPLDAAVEcHR.[G]	C19(Carbamidomethyl)	1	1
[R].dGWHSWPIAHQWPQGPSAVDAAFSWE	N-Term(iTRAQ4plex); K28(iTRAQ	1	1
[K].lLQDEFPGIPSPLDAAVEcHR.[G]	N-Term(iTRAQ4plex); C19(Carbar	1	1
[K].lLQDEFPGIPSPLDAAVEcHR.[G]	N-Term(iTRAQ4plex); C19(Carbar	1	1
[K].lLQDEFPGIPSPLDAAVEcHR.[G]	N-Term(iTRAQ4plex); C19(Carbar	1	1
[K].lLQDEFPGIPSPLDAAVEcHR.[G]	N-Term(iTRAQ4plex); C19(Carbar	1	1
[K].lLQDEFPGIPSPLDAAVEcHR.[G]	N-Term(iTRAQ4plex); C19(Carbar	1	1
[K].lLQDEFPGIPSPLDAAVEcHR.[G]	N-Term(iTRAQ4plex); C19(Carbar	1	1
[K].lLQDEFPGIPSPLDAAVEcHR.[G]	N-Term(iTRAQ4plex); C19(Carbar	1	1
[K].lLQDEFPGIPSPLDAAVEcHR.[G]	N-Term(iTRAQ4plex); C19(Carbar	1	1
[K].eVGTPHGIILDSVDAAFIcPGSSR.[L]	N-Term(iTRAQ4plex); C19(Carbar	1	1

[R].rLWWLDLKSGAQATWTELPWPHEk.[V]	N-Term(iTRAQ4plex); K24(iTRAQ4	1	1
[K].eVGTPHGIILDSVDAAFIcPGSSR.[L]	N-Term(iTRAQ4plex); C19(Carbar	1	1
[R].RLWWLDLkSGAQATWTELPWPHEk.[V]	K8(iTRAQ4plex); K24(iTRAQ4plex	1	1
[K].eVGTPHGIILDSVDAAFIcPGSSR.[L]	N-Term(iTRAQ4plex); C19(Carbar	1	1
[R].cSPHLVLSALTSDNHGATYAFSGTHYWR.[	N-Term(iTRAQ4plex); C1(Carbam	1	1
[K].lYLVQGTQVYVFLTk.[G]	N-Term(iTRAQ4plex); K15(iTRAQ4	1	1
[K].lYLVQGTQVYVFLTk.[G]	N-Term(iTRAQ4plex); K15(iTRAQ4	1	1
[K].eVGTPHGIILDSVDAAFIcPGSSR.[L]	N-Term(iTRAQ4plex); C19(Carbar	1	1
[R].lWWLDLk.[S]	N-Term(iTRAQ4plex); K7(iTRAQ4	1	1
[K].lLQDEFPGIPSPLDAAVEcHR.[G]	N-Term(iTRAQ4plex); C19(Carbar	1	1
[R].eWFWDLATGTmk.[E]	N-Term(iTRAQ4plex); M11(Oxida	1	1
[R].lWWLDLk.[S]	N-Term(iTRAQ4plex); K7(iTRAQ4	1	1
[R].lWWLDLk.[S]	N-Term(iTRAQ4plex); K7(iTRAQ4	1	1
[R].dGWHSWPIAHQWPQGPSAVDAAFSWE	N-Term(iTRAQ4plex); K28(iTRAQ4	1	1
[R].dGWHSWPIAHQWPQGPSAVDAAFSWE	N-Term(iTRAQ4plex); K28(iTRAQ4	1	1
[R].eWFWDLATGTmk.[E]	N-Term(iTRAQ4plex); M11(Oxida	1	1
[R].wkNFPSPVDAAFR.[Q]	N-Term(iTRAQ4plex); K2(iTRAQ4	1	1
[R].wkNFPSPVDAAFR.[Q]	N-Term(iTRAQ4plex); K2(iTRAQ4	1	1
[R].yYcFQGNQFLR.[F]	N-Term(iTRAQ4plex); C3(Carbam	1	1
[K].vDGALcMEk.[S]	N-Term(iTRAQ4plex); C6(Carbam	1	1
[R].wkNFPSPVDAAFR.[Q]	N-Term(iTRAQ4plex); K2(iTRAQ4	1	1
[K].vDGALcMEk.[S]	N-Term(iTRAQ4plex); C6(Carbam	1	1
[R].wKNFPSPVDAAFR.[Q]	N-Term(iTRAQ4plex)	1	1
[R].WkNFPSPVDAAFR.[Q]	K2(iTRAQ4plex)	1	1
[K].GGYTLVSGYPk.[R]	K11(iTRAQ4plex)	1	1
[K].lLQDEFPGIPSPLDAAVEcHR.[G]	N-Term(iTRAQ4plex); C19(Carbar	1	1
[R].eWFWDLATGTMk.[E]	N-Term(iTRAQ4plex); K12(iTRAQ4	1	1
[R].eWFWDLATGTMk.[E]	N-Term(iTRAQ4plex); K12(iTRAQ4	1	1
[R].eWFWDLATGTMk.[E]	N-Term(iTRAQ4plex); K12(iTRAQ4	1	1
[R].gEcQAEGVLFFQGDR.[E]	N-Term(iTRAQ4plex); C3(Carbam	1	1
[K].lLQDEFPGIPSPLDAAVEcHR.[G]	N-Term(iTRAQ4plex); C19(Carbar	1	1
[R].gEcQAEGVLFFQGDR.[E]	N-Term(iTRAQ4plex); C3(Carbam	1	1
[R].sWPAVGNcSSALR.[W]	N-Term(iTRAQ4plex); C8(Carbam	1	1
[K].NFPSPVDAAFR.[Q]		1	1
[R].gEcQAEGVLFFQGDR.[E]	N-Term(iTRAQ4plex); C3(Carbam	1	1
[K].vWVYPPEk.[K]	N-Term(iTRAQ4plex); K8(iTRAQ4	1	1
[R].gEcQAEGVLFFQGDR.[E]	N-Term(iTRAQ4plex); C3(Carbam	1	1
[K].vDGALcMEk.[S]	N-Term(iTRAQ4plex); C6(Carbam	1	1
[K].eVGTPHGIILDSVDAAFIcPGSSR.[L]	N-Term(iTRAQ4plex); C19(Carbar	1	1
[R].dYFMPcPGR.[G]	N-Term(iTRAQ4plex); C6(Carbam	1	1
[R].lWWLDLk.[S]	N-Term(iTRAQ4plex); K7(iTRAQ4	1	1
[K].VDGALcmEk.[S]	C6(Carbamidomethyl); M7(Oxid	1	1
[R].qGHNSVFLIk.[G]	N-Term(iTRAQ4plex); K10(iTRAQ4	1	1
[R].rLWWLDLk.[S]	N-Term(iTRAQ4plex); K8(iTRAQ4	1	1
[K].VDGALcMEk.[S]	C6(Carbamidomethyl); K9(iTRAC	1	1
[K].gGYTLVSGYPk.[R]	N-Term(iTRAQ4plex); K11(iTRAQ4	1	1
[R].lWWLDLk.[S]	N-Term(iTRAQ4plex); K7(iTRAQ4	1	1
[K].sGAQATWTELPWPHEk.[V]	N-Term(iTRAQ4plex); K16(iTRAQ4	1	1
[R].yYcFQGNQFLR.[F]	N-Term(iTRAQ4plex); C3(Carbam	1	1
[K].lLQDEFPGIPSPLDAAVEcHR.[G]	N-Term(iTRAQ4plex); C19(Carbar	1	1
[K].gGYTLVSGYPk.[R]	N-Term(iTRAQ4plex); K11(iTRAQ4	1	1
[K].lLQDEFPGIPSPLDAAVEcHR.[G]	N-Term(iTRAQ4plex); C19(Carbar	1	1

[K].eVGTPHGIILDSVDAAFIcPGSSR.[L]	N-Term(iTRAQ4plex); C19(Carbar	1	1
[R].dYFmPcPGR.[G]	N-Term(iTRAQ4plex); M4(Oxidat	1	1
[R].rLWWLDLk.[S]	N-Term(iTRAQ4plex); K8(iTRAQ4	1	1
[K].vWVYPPEk.[K]	N-Term(iTRAQ4plex); K8(iTRAQ4	1	1
[R].dYFMPcPGR.[G]	N-Term(iTRAQ4plex); C6(Carbam	1	1
[K].vWVYPPEk.[K]	N-Term(iTRAQ4plex); K8(iTRAQ4	1	1
[K].vWVYPPEk.[K]	N-Term(iTRAQ4plex); K8(iTRAQ4	1	1
[R].gEcQAEGVLFFQGDR.[E]	N-Term(iTRAQ4plex); C3(Carbam	1	1
[R].gEcQAEGVLFFQGDR.[E]	N-Term(iTRAQ4plex); C3(Carbam	1	1
[K].nFPSPVDAAFR.[Q]	N-Term(iTRAQ4plex)	1	1
[R].rLWWLDLk.[S]	N-Term(iTRAQ4plex); K8(iTRAQ4	1	1
[K].gGYTLVSGYPk.[R]	N-Term(iTRAQ4plex); K11(iTRAQ	1	1
[R].qGHNSVFLIk.[G]	N-Term(iTRAQ4plex); K10(iTRAQ	1	1
[R].eWFWDLATGTmk.[E]	N-Term(iTRAQ4plex); M11(Oxida	1	1
[R].lWWLDLk.[S]	N-Term(iTRAQ4plex); K7(iTRAQ4	1	1
[K].gGYTLVSGYPk.[R]	N-Term(iTRAQ4plex); K11(iTRAQ	1	1
[R].QGHNSVFLIk.[G]	K10(iTRAQ4plex)	1	1
[K].vWVYPPEk.[K]	N-Term(iTRAQ4plex); K8(iTRAQ4	1	1
[K].vWVYPPEk.[K]	N-Term(iTRAQ4plex); K8(iTRAQ4	1	1
[K].vWVYPPEk.[K]	N-Term(iTRAQ4plex); K8(iTRAQ4	1	1
[K].nFPSPVDAAFR.[Q]	N-Term(iTRAQ4plex)	1	1
[K].eVGTPHGIILDSVDAAFIcPGSSR.[L]	N-Term(iTRAQ4plex); C19(Carbar	1	1
[R].yYcFQGNQFLR.[F]	N-Term(iTRAQ4plex); C3(Carbam	1	1
[R].dYFMPcPGR.[G]	N-Term(iTRAQ4plex); C6(Carbam	1	1
[K].lLQDEFPGIPSPLDAAVEcHR.[G]	N-Term(iTRAQ4plex); C19(Carbar	1	1
[R].eWFWDLATGTmk.[E]	N-Term(iTRAQ4plex); M11(Oxida	1	1
[K].vDGALcMEk.[S]	N-Term(iTRAQ4plex); C6(Carbam	1	1
[K].vWVYPPEk.[K]	N-Term(iTRAQ4plex); K8(iTRAQ4	1	1
[K].SGAQATWTELPWPHEk.[V]	K16(iTRAQ4plex)	1	1
[R].gEcQAEGVLFFQGDR.[E]	N-Term(iTRAQ4plex); C3(Carbam	1	1
[K].gGYTLVSGYPk.[R]	N-Term(iTRAQ4plex); K11(iTRAQ	1	1
[K].LLQDEFPGIPSPLDAAVEcHR.[G]	C19(Carbamidomethyl)	1	1
[K].vWVYPPEk.[K]	N-Term(iTRAQ4plex); K8(iTRAQ4	1	1
[R].dYFMPcPGR.[G]	N-Term(iTRAQ4plex); C6(Carbam	1	1
[R].yYcFQGNQFLR.[F]	N-Term(iTRAQ4plex); C3(Carbam	1	1
[R].YYcFQGNQFLR.[F]	C3(Carbamidomethyl)	1	1
[K].NFPSPVDAAFR.[Q]		1	1
[R].qGHNSVFLIk.[G]	N-Term(iTRAQ4plex); K10(iTRAQ	1	1
[R].dYFMPcPGR.[G]	N-Term(iTRAQ4plex); C6(Carbam	1	1
[R].eWFWDLATGTMk.[E]	N-Term(iTRAQ4plex); K12(iTRAQ	1	1
[K].vWVYPPEk.[K]	N-Term(iTRAQ4plex); K8(iTRAQ4	1	1
[K].vWVYPPEk.[K]	N-Term(iTRAQ4plex); K8(iTRAQ4	1	1
[R].QGHNSVFLIk.[G]	K10(iTRAQ4plex)	1	1
[K].NFPSPVDAAFR.[Q]		1	1
[R].dYFMPcPGR.[G]	N-Term(iTRAQ4plex); C6(Carbam	1	1
[K].vWVYPPEk.[K]	N-Term(iTRAQ4plex); K8(iTRAQ4	1	1
[K].vWVYPPEkk.[E]	N-Term(iTRAQ4plex); K8(iTRAQ4	1	1
[K].SGAQATWTELPWPHEk.[V]	K16(iTRAQ4plex)	1	1
[K].vDGALcMEk.[S]	N-Term(iTRAQ4plex); C6(Carbam	1	1
[K].vDGALcmEk.[S]	N-Term(iTRAQ4plex); C6(Carbam	1	1
[K].NFPSPVDAAFR.[Q]		1	1
[K].vWVYPPEkk.[E]	N-Term(iTRAQ4plex); K8(iTRAQ4	1	1

[R].yYcFQGNQFLR.[F]	N-Term(iTRAQ4plex); C3(Carbam	1	1
[K].gGYTLVSGYPk.[R]	N-Term(iTRAQ4plex); K11(iTRAQ	1	1
[R].qGHNSVFLIk.[G]	N-Term(iTRAQ4plex); K10(iTRAQ	1	1
[K].gGYTLVSGYPk.[R]	N-Term(iTRAQ4plex); K11(iTRAQ	1	1
[K].vDGALcmEk.[S]	N-Term(iTRAQ4plex); C6(Carbam	1	1
[K].gGYTLVSGYPk.[R]	N-Term(iTRAQ4plex); K11(iTRAQ	1	1
[K].vDGALcMEk.[S]	N-Term(iTRAQ4plex); C6(Carbam	1	1
[R].LWWLDLk.[S]	K7(iTRAQ4plex)	1	1
[R].rLWWLDLk.[S]	N-Term(iTRAQ4plex); K8(iTRAQ4	1	1
[K].vWVYPPEk.[K]	N-Term(iTRAQ4plex); K8(iTRAQ4	1	1
[K].EVGTPHGIILDSVDAAFIcPGSSR.[L]	C19(Carbamidomethyl)	1	1
[K].vWVYPPEk.[K]	N-Term(iTRAQ4plex); K8(iTRAQ4	1	1
[R].dYFMPcPGR.[G]	N-Term(iTRAQ4plex); C6(Carbam	1	1
[K].vDGALcmEk.[S]	N-Term(iTRAQ4plex); C6(Carbam	1	1
[K].vWVYPPEk.[K]	N-Term(iTRAQ4plex); K8(iTRAQ4	1	1
[R].YYcFQGNQFLR.[F]	C3(Carbamidomethyl)	1	1
[R].gEcQAEGVLFFQGDR.[E]	N-Term(iTRAQ4plex); C3(Carbam	1	1
[K].vDGALcMEk.[S]	N-Term(iTRAQ4plex); C6(Carbam	1	1
[R].rLWWLDLk.[S]	N-Term(iTRAQ4plex); K8(iTRAQ4	1	1
[K].eVGTPHGIILDSVDAAFIcPGSSR.[L]	N-Term(iTRAQ4plex); C19(Carbar	1	1
[K].LLQDEFPGIPSPLDAAVEcHR.[G]	C19(Carbamidomethyl)	1	1
[K].vDGALcmEk.[S]	N-Term(iTRAQ4plex); C6(Carbam	1	1
[R].gEVPPRYPR.[D]	N-Term(iTRAQ4plex)	1	1
[K].eVGTPHGIILDSVDAAFIcPGSSR.[L]	N-Term(iTRAQ4plex); C19(Carbar	1	1
[R].dYFmPcPGR.[G]	N-Term(iTRAQ4plex); M4(Oxidat	1	1
[R].LWWLDLk.[S]	K7(iTRAQ4plex)	1	1
[R].fDPVRGEVPPR.[Y]	N-Term(iTRAQ4plex)	1	1
[K].gGYTLVSGYPk.[R]	N-Term(iTRAQ4plex); K11(iTRAQ	1	1
[R].lWWLDLk.[S]	N-Term(iTRAQ4plex); K7(iTRAQ4	1	1
[K].NFPSPVDAAFR.[Q]		1	1
[R].dYFMPcPGR.[G]	N-Term(iTRAQ4plex); C6(Carbam	1	1
[K].nFPSPVDAAFR.[Q]	N-Term(iTRAQ4plex)	1	1
[R].dYFmPcPGR.[G]	N-Term(iTRAQ4plex); M4(Oxidat	1	1
[K].vWVYPPEk.[K]	N-Term(iTRAQ4plex); K8(iTRAQ4	1	1
[K].vWVYPPEk.[K]	N-Term(iTRAQ4plex); K8(iTRAQ4	1	1
[R].LWWLDLk.[S]	K7(iTRAQ4plex)	1	1
[K].nFPSPVDAAFR.[Q]	N-Term(iTRAQ4plex)	1	1
[R].dYFMPcPGR.[G]	N-Term(iTRAQ4plex); C6(Carbam	1	1
[R].eWFWDLATGTmk.[E]	N-Term(iTRAQ4plex); M11(Oxida	1	1
[K].vDGALcMEk.[S]	N-Term(iTRAQ4plex); C6(Carbam	1	1
[R].dYFMPcPGR.[G]	N-Term(iTRAQ4plex); C6(Carbam	1	1
[K].NFPSPVDAAFR.[Q]		1	1
[K].GGYTLVSGYPk.[R]	K11(iTRAQ4plex)	1	1
[K].EVGTPHGIILDSVDAAFIcPGSSR.[L]	C19(Carbamidomethyl)	1	1
[R].gEcQAEGVLFFQGDR.[E]	N-Term(iTRAQ4plex); C3(Carbam	1	1
[R].dYFmPcPGR.[G]	N-Term(iTRAQ4plex); M4(Oxidat	1	1
[R].dYFMPcPGR.[G]	N-Term(iTRAQ4plex); C6(Carbam	1	1
[K].vDGALcMEk.[S]	N-Term(iTRAQ4plex); C6(Carbam	1	1
[R].gEcQAEGVLFFQGDR.[E]	N-Term(iTRAQ4plex); C3(Carbam	1	1
[R].QGHNSVFLIk.[G]	K10(iTRAQ4plex)	1	1
[K].NFPSPVDAAFR.[Q]		1	1
[R].eWFWDLATGTmk.[E]	N-Term(iTRAQ4plex); M11(Oxida	1	1

[K].vDGALcMEk.[S]	N-Term(iTRAQ4plex); C6(Carbam	1	1
[K].vDGALcmEk.[S]	N-Term(iTRAQ4plex); C6(Carbam	1	1
[K].lYLVQGTQVYVFLTk.[G]	N-Term(iTRAQ4plex); K15(iTRAQ	1	1
[K].eVGTPHGIILDSVDAAFIcPGSSR.[L]	N-Term(iTRAQ4plex); C19(Carbar	1	1
[K].lLQDEFPGIPSPLDAAVEcHR.[G]	N-Term(iTRAQ4plex); C19(Carbar	1	1
[R].dYFmPcPGR.[G]	N-Term(iTRAQ4plex); M4(Oxidat	1	1
[K].nFPSPVDAAFR.[Q]	N-Term(iTRAQ4plex)	1	1
[K].sGAQATWTELPWPHEk.[V]	N-Term(iTRAQ4plex); K16(iTRAQ	1	1
[R].eWFWDLATGTMk.[E]	N-Term(iTRAQ4plex); K12(iTRAQ	1	1
[K].vDGALcmEk.[S]	N-Term(iTRAQ4plex); C6(Carbam	1	1
[R].qGHNSVFLIk.[G]	N-Term(iTRAQ4plex); K10(iTRAQ	1	1
[K].gGYTLVSGYPk.[R]	N-Term(iTRAQ4plex); K11(iTRAQ	1	1
[K].eVGTPHGIILDSVDAAFIcPGSSR.[L]	N-Term(iTRAQ4plex); C19(Carbar	1	1
[R].qGHNSVFLIk.[G]	N-Term(iTRAQ4plex); K10(iTRAQ	1	1
[K].vWVYPPEk.[K]	N-Term(iTRAQ4plex); K8(iTRAQ4	1	1
[K].nFPSPVDAAFR.[Q]	N-Term(iTRAQ4plex)	1	1
[R].dYFmPcPGR.[G]	N-Term(iTRAQ4plex); M4(Oxidat	1	1
[R].YYcFQGNQFLR.[F]	C3(Carbamidomethyl)	1	1
[K].SGAQATWTELPWPHEk.[V]	K16(iTRAQ4plex)	1	1
[R].dYFmPcPGR.[G]	N-Term(iTRAQ4plex); M4(Oxidat	1	1
[R].dYFmPcPGR.[G]	N-Term(iTRAQ4plex); M4(Oxidat	1	1
[R].lMQcLPNPEDVk.[M]	N-Term(iTRAQ4plex); C4(Carbam	1	1
[K].cEWETPEGcEQVLTGkR.[L]	N-Term(iTRAQ4plex); C1(Carbam	1	1
[R].FSAIcQGDGTWSPR.[T]	C5(Carbamidomethyl)	1	1
[R].wTPYQGcEALccPEPk.[L]	N-Term(iTRAQ4plex); C7(Carbam	1	1
[R].wTPYQGcEALccPEPk.[L]	N-Term(iTRAQ4plex); C7(Carbam	1	1
[R].wTPYQGcEALccPEPk.[L]	N-Term(iTRAQ4plex); C7(Carbam	1	1
[R].wTPYQGcEALccPEPk.[L]	N-Term(iTRAQ4plex); C7(Carbam	1	1
[R].cHPGYkPTTDEPTTVIcQk.[N]	C1(Carbamidomethyl); K6(iTRAC	1	1
[K].eDVYVVGTVLR.[Y]	N-Term(iTRAQ4plex)	1	1
[K].eDVYVVGTVLR.[Y]	N-Term(iTRAQ4plex)	1	1
[K].eDVYVVGTVLR.[Y]	N-Term(iTRAQ4plex)	1	1
[R].wTPYQGcEALccPEPk.[L]	N-Term(iTRAQ4plex); C7(Carbam	1	1
[K].qSSSYSFFk.[E]	N-Term(iTRAQ4plex); K9(iTRAQ4	1	1
[K].qSSSYSFFk.[E]	N-Term(iTRAQ4plex); K9(iTRAQ4	1	1
[K].qSSSYSFFk.[E]	N-Term(iTRAQ4plex); K9(iTRAQ4	1	1
[R].fSAIcQGDGTWSPR.[T]	N-Term(iTRAQ4plex); C5(Carbam	1	1
[R].fSAIcQGDGTWSPR.[T]	N-Term(iTRAQ4plex); C5(Carbam	1	1
[R].fSAIcQGDGTWSPR.[T]	N-Term(iTRAQ4plex); C5(Carbam	1	1
[R].fSAIcQGDGTWSPR.[T]	N-Term(iTRAQ4plex); C5(Carbam	1	1
[R].kPDVSHGEMVSGFGPIYNYk.[D]	N-Term(iTRAQ4plex); K1(iTRAQ4	1	1
[R].GSSVIHcDADSk.[W]	C7(Carbamidomethyl); K12(iTRA	1	1
[R].fSAIcQGDGTWSPR.[T]	N-Term(iTRAQ4plex); C5(Carbam	1	1
[R].fSAIcQGDGTWSPR.[T]	N-Term(iTRAQ4plex); C5(Carbam	1	1
[R].kPDVSHGEMVSGFGPIYNYk.[D]	N-Term(iTRAQ4plex); K1(iTRAQ4	1	1
[R].kPDVSHGEMVSGFGPIYNYk.[D]	N-Term(iTRAQ4plex); K1(iTRAQ4	1	1
[R].lMQcLPNPEDVk.[M]	N-Term(iTRAQ4plex); C4(Carbam	1	1
[R].lMQcLPNPEDVk.[M]	N-Term(iTRAQ4plex); C4(Carbam	1	1
[R].kPDVSHGEMVSGFGPIYNYk.[D]	N-Term(iTRAQ4plex); K1(iTRAQ4	1	1
[K].gYILVGQAk.[L]	N-Term(iTRAQ4plex); K9(iTRAQ4	1	1
[K].lScSYSHWSAPAPQck.[A]	N-Term(iTRAQ4plex); C3(Carbam	1	1
[R].gVGWSHPLPQcEIVk.[C]	N-Term(iTRAQ4plex); C11(Carbar	1	1

[R].gVGWSHPLPQcEIVk.[C]	N-Term(iTRAQ4plex); C11(Carbar	1	1
[R].gVGWSHPLPQcEIVk.[C]	N-Term(iTRAQ4plex); C11(Carbar	1	1
[R].GVGWSHPLPQcEIVk.[C]	C11(Carbamidomethyl); K15(iTR	1	1
[R].lmQcLPNPEDVk.[M]	N-Term(iTRAQ4plex); M2(Oxidat	1	1
[K].lSLEIEQLELQR.[D]	N-Term(iTRAQ4plex)	1	1
[K].lSLEIEQLELQR.[D]	N-Term(iTRAQ4plex)	1	1
[R].gSSVIHcDADSk.[W]	N-Term(iTRAQ4plex); C7(Carbam	1	1
[R].gSSVIHcDADSk.[W]	N-Term(iTRAQ4plex); C7(Carbam	1	1
[R].gSSVIHcDADSk.[W]	N-Term(iTRAQ4plex); C7(Carbam	1	1
[K].cEWETPEGcEQVLTGk.[R]	N-Term(iTRAQ4plex); C1(Carbam	1	1
[R].gSSVIHcDADSk.[W]	N-Term(iTRAQ4plex); C7(Carbam	1	1
[K].tDLSFGSQIEFScSEGFFLIGSTTSR.[C]	N-Term(iTRAQ4plex); C13(Carbar	1	1
[R].tPScGDIcNFPPk.[I]	N-Term(iTRAQ4plex); C4(Carbam	1	1
[R].tPScGDIcNFPPk.[I]	N-Term(iTRAQ4plex); C4(Carbam	1	1
[R].tPScGDIcNFPPk.[I]	N-Term(iTRAQ4plex); C4(Carbam	1	1
[R].tPScGDIcNFPPk.[I]	N-Term(iTRAQ4plex); C4(Carbam	1	1
[R].tPScGDIcNFPPk.[I]	N-Term(iTRAQ4plex); C4(Carbam	1	1
[R].tPScGDIcNFPPk.[I]	N-Term(iTRAQ4plex); C4(Carbam	1	1
[R].tPScGDIcNFPPk.[I]	N-Term(iTRAQ4plex); C4(Carbam	1	1
[R].tPScGDIcNFPPk.[I]	N-Term(iTRAQ4plex); C4(Carbam	1	1
[R].tPScGDIcNFPPk.[I]	N-Term(iTRAQ4plex); C4(Carbam	1	1
[K].eEIIYEcDk.[G]	N-Term(iTRAQ4plex); C7(Carbam	1	1
[K].eEIIYEcDk.[G]	N-Term(iTRAQ4plex); C7(Carbam	1	1
[K].eEIIYEcDk.[G]	N-Term(iTRAQ4plex); C7(Carbam	1	1
[K].eEIIYEcDk.[G]	N-Term(iTRAQ4plex); C7(Carbam	1	1
[K].eEIIYEcDk.[G]	N-Term(iTRAQ4plex); C7(Carbam	1	1
[K].eEIIYEcDk.[G]	N-Term(iTRAQ4plex); C7(Carbam	1	1
[R].lMQcLPNPEDVk.[M]	N-Term(iTRAQ4plex); C4(Carbam	1	1
[K].gYILVGQAk.[L]	N-Term(iTRAQ4plex); K9(iTRAQ4	1	1
[R].tWYPEVPk.[C]	N-Term(iTRAQ4plex); K8(iTRAQ4	1	1
[K].ckPPPDIR.[N]	N-Term(iTRAQ4plex); C1(Carbam	1	1
[K].eEIIYEcDk.[G]	N-Term(iTRAQ4plex); C7(Carbam	1	1
[K].eDVYVVGTVLR.[Y]	N-Term(iTRAQ4plex)	1	1
[K].mALEVYk.[L]	N-Term(iTRAQ4plex); K7(iTRAQ4	1	1
[K].eEIIYEcDk.[G]	N-Term(iTRAQ4plex); C7(Carbam	1	1
[R].cHPGYkPTTDEPTTVIcQk.[N]	N-Term(iTRAQ4plex); C1(Carbam	1	1
[R].gVGWSHPLPQcEIVk.[C]	N-Term(iTRAQ4plex); C11(Carbar	1	1
[K].gYILVGQAk.[L]	N-Term(iTRAQ4plex); K9(iTRAQ4	1	1
[R].tWYPEVPk.[C]	N-Term(iTRAQ4plex); K8(iTRAQ4	1	1
[R].gSSVIHcDADSk.[W]	N-Term(iTRAQ4plex); C7(Carbam	1	1
[R].kPDVSHGEmVSGFGPIYNYk.[D]	N-Term(iTRAQ4plex); K1(iTRAQ4	1	1
[K].eDVYVVGTVLR.[Y]	N-Term(iTRAQ4plex)	1	1
[R].tPScGDIcNFPPk.[I]	N-Term(iTRAQ4plex); C4(Carbam	1	1
[R].kPDVSHGEMVSGFGPIYNYk.[D]	N-Term(iTRAQ4plex); K1(iTRAQ4	1	1
[K].eDVYVVGTVLR.[Y]	N-Term(iTRAQ4plex)	1	1
[K].EDVYVVGTVLR.[Y]		1	1
[K].iAHGHYk.[Q]	N-Term(iTRAQ4plex); K7(iTRAQ4	1	1
[K].gYILVGQAk.[L]	N-Term(iTRAQ4plex); K9(iTRAQ4	1	1
[R].tPScGDIcNFPPk.[I]	N-Term(iTRAQ4plex); C4(Carbam	1	1
[K].mALEVYk.[L]	N-Term(iTRAQ4plex); K7(iTRAQ4	1	1
[R].gSSVIHcDADSk.[W]	N-Term(iTRAQ4plex); C7(Carbam	1	1
[R].fkTGTTLk.[Y]	N-Term(iTRAQ4plex); K2(iTRAQ4	1	1

[R].gSSVIHcDADSk.[W]	N-Term(iTRAQ4plex); C7(Carbam	1	1
[K].gYILVGQAk.[L]	N-Term(iTRAQ4plex); K9(iTRAQ4	1	1
[K].eDVYVVGTVLR.[Y]	N-Term(iTRAQ4plex)	1	1
[R].tWYPEVPk.[C]	N-Term(iTRAQ4plex); K8(iTRAQ4	1	1
[K].gYILVGQAk.[L]	N-Term(iTRAQ4plex); K9(iTRAQ4	1	1
[R].YRcHPGYkPTTDEPTTVIcQk.[N]	C3(Carbamidomethyl); K8(iTRAC	1	1
[R].GSSVIHcDADSk.[W]	C7(Carbamidomethyl); K12(iTRA	1	1
[K].yTcLPGYVR.[S]	N-Term(iTRAQ4plex); C3(Carbam	1	1
[R].LMQcLPNPEDVk.[M]	C4(Carbamidomethyl); K12(iTRA	1	1
[R].tPScGDIcNFPPk.[I]	N-Term(iTRAQ4plex); C4(Carbam	1	1
[K].iAHGHYk.[Q]	N-Term(iTRAQ4plex); K7(iTRAQ4	1	1
[R].tPScGDIcNFPPk.[I]	N-Term(iTRAQ4plex); C4(Carbam	1	1
[R].tPScGDIcNFPPk.[I]	N-Term(iTRAQ4plex); C4(Carbam	1	1
[R].wTPYQGcEALccPEPk.[L]	N-Term(iTRAQ4plex); C7(Carbam	1	1
[R].tPScGDIcNFPPk.[I]	N-Term(iTRAQ4plex); C4(Carbam	1	1
[K].gYILVGQAk.[L]	N-Term(iTRAQ4plex); K9(iTRAQ4	1	1
[R].tPScGDIcNFPPk.[I]	N-Term(iTRAQ4plex); C4(Carbam	1	1
[R].TWYPEVPk.[C]	K8(iTRAQ4plex)	1	1
[R].cHPGYkPTTDEPTTVIcQk.[N]	N-Term(iTRAQ4plex); C1(Carbam	1	1
[K].eEIIYEcDk.[G]	N-Term(iTRAQ4plex); C7(Carbam	1	1
[K].GYILVGQAk.[L]	K9(iTRAQ4plex)	1	1
[K].cEWETPEGcEQVLTGk.[R]	C1(Carbamidomethyl); C9(Carba	1	1
[K].yTcLPGYVR.[S]	N-Term(iTRAQ4plex); C3(Carbam	1	1
[K].lScSYSHWSAPAPQck.[A]	N-Term(iTRAQ4plex); C3(Carbam	1	1
[R].gVGWSHPLPQcEIVk.[C]	N-Term(iTRAQ4plex); C11(Carbar	1	1
[R].tPScGDIcNFPPk.[I]	N-Term(iTRAQ4plex); C4(Carbam	1	1
[R].tWYPEVPk.[C]	N-Term(iTRAQ4plex); K8(iTRAQ4	1	1
[R].tWYPEVPk.[C]	N-Term(iTRAQ4plex); K8(iTRAQ4	1	1
[R].GVGWSHPLPQcEIVk.[C]	C11(Carbamidomethyl); K15(iTR	1	1
[R].tWYPEVPk.[C]	N-Term(iTRAQ4plex); K8(iTRAQ4	1	1
[R].tPScGDIcNFPPk.[I]	N-Term(iTRAQ4plex); C4(Carbam	1	1
[R].tWYPEVPk.[C]	N-Term(iTRAQ4plex); K8(iTRAQ4	1	1
[R].gSSVIHcDADSk.[W]	N-Term(iTRAQ4plex); C7(Carbam	1	1
[R].gVGWSHPLPQcEIVk.[C]	N-Term(iTRAQ4plex); C11(Carbar	1	1
[K].yTcLPGYVR.[S]	N-Term(iTRAQ4plex); C3(Carbam	1	1
[R].tPScGDIcNFPPk.[I]	N-Term(iTRAQ4plex); C4(Carbam	1	1
[R].tPScGDIcNFPPk.[I]	N-Term(iTRAQ4plex); C4(Carbam	1	1
[K].eEIIYEcDk.[G]	N-Term(iTRAQ4plex); C7(Carbam	1	1
[R].cHPGYkPTTDEPTTVIcQk.[N]	C1(Carbamidomethyl); K6(iTRAC	1	1
[R].tPScGDIcNFPPk.[I]	N-Term(iTRAQ4plex); C4(Carbam	1	1
[K].tSESGTPSSSTPQENTISGYSLLTck.[R]	N-Term(iTRAQ4plex); C25(Carbar	1	1
[R].iAYGTQGSSGYSLR.[L]	N-Term(iTRAQ4plex)	1	1
[R].iAYGTQGSSGYSLR.[L]	N-Term(iTRAQ4plex)	1	1
[K].ekGEIQNILQk.[V]	N-Term(iTRAQ4plex); K2(iTRAQ4	1	1
[K].ekGEIQNILQk.[V]	N-Term(iTRAQ4plex); K2(iTRAQ4	1	1
[K].ekGEIQNILQk.[V]	N-Term(iTRAQ4plex); K2(iTRAQ4	1	1
[R].LVGITSWGEGcAR.[R]	C11(Carbamidomethyl)	1	1
[R].lVGITSWGEGcAR.[R]	N-Term(iTRAQ4plex); C11(Carbar	1	1
[R].vLTPDAFVcR.[T]	N-Term(iTRAQ4plex); C9(Carbam	1	1
[K].vNIPLVTNEEcQk.[R]	N-Term(iTRAQ4plex); C11(Carbar	1	1
[K].vAEYMDWILEk.[T]	N-Term(iTRAQ4plex); K11(iTRAQ	1	1
[K].vAEYMDWILEk.[T]	N-Term(iTRAQ4plex); K11(iTRAQ	1	1

[R].rEQPGVYTk.[V]	N-Term(iTRAQ4plex); K9(iTRAQ4	1	1
[K].gDTSTIYTNcWVTGWGFSk.[E]	N-Term(iTRAQ4plex); C10(Carbar	1	1
[K].dSVTGTLPk.[V]	N-Term(iTRAQ4plex); K9(iTRAQ4	1	1
[K].gVNVcQETcTk.[M]	N-Term(iTRAQ4plex); C5(Carbam	1	1
[R].tLPEPcHSk.[I]	N-Term(iTRAQ4plex); C6(Carbam	1	1
[R].gGDVASMYTPNAQYcQMR.[C]	N-Term(iTRAQ4plex); C15(Carbar	1	1
[R].lVGITSWGEGcAR.[R]	N-Term(iTRAQ4plex); C11(Carbar	1	1
[R].lVGITSWGEGcAR.[R]	N-Term(iTRAQ4plex); C11(Carbar	1	1
[K].qcGHQISAcHR.[D]	N-Term(iTRAQ4plex); C2(Carbam	1	1
[K].qcGHQISAcHR.[D]	N-Term(iTRAQ4plex); C2(Carbam	1	1
[K].eIIIHQNYk.[V]	N-Term(iTRAQ4plex); K9(iTRAQ4	1	1
[K].vSEGNHDIALIk.[L]	N-Term(iTRAQ4plex); K12(iTRAQ	1	1
[K].ekGEIQNILQk.[V]	N-Term(iTRAQ4plex); K2(iTRAQ4	1	1
[R].LSMDGSPTR.[I]		1	1
[K].ekGEIQNILQk.[V]	N-Term(iTRAQ4plex); K2(iTRAQ4	1	1
[R].tGAVSGHSLk.[Q]	N-Term(iTRAQ4plex); K10(iTRAQ	1	1
[K].eIIIHQNYk.[V]	N-Term(iTRAQ4plex); K9(iTRAQ4	1	1
[K].gEIQNILQk.[V]	N-Term(iTRAQ4plex); K9(iTRAQ4	1	1
[K].dTPFSQIk.[E]	N-Term(iTRAQ4plex); K8(iTRAQ4	1	1
[R].TLPEPcHSk.[I]	C6(Carbamidomethyl); K9(iTRAC	1	1
[K].ySPGGTPTAIk.[V]	N-Term(iTRAQ4plex); K11(iTRAQ	1	1
[K].gDSGGPLVck.[HD]	N-Term(iTRAQ4plex); C9(Carbam	1	2
[K].ySPGGTPTAIk.[V]	N-Term(iTRAQ4plex); K11(iTRAQ	1	1
[R].vLTPDAFVcR.[T]	N-Term(iTRAQ4plex); C9(Carbam	1	1
[K].vNIPLVTNEEcQk.[R]	N-Term(iTRAQ4plex); C11(Carbar	1	1
[R].lVGITSWGEGcAR.[R]	N-Term(iTRAQ4plex); C11(Carbar	1	1
[K].vSEGNHDIALIk.[L]	N-Term(iTRAQ4plex); K12(iTRAQ	1	1
[R].tLPEPcHSk.[I]	N-Term(iTRAQ4plex); C6(Carbam	1	1
[R].LSMDGSPTR.[I]		1	1
[K].vSSVEEcQk.[R]	N-Term(iTRAQ4plex); C7(Carbam	1	1
[R].lVGITSWGEGcAR.[R]	N-Term(iTRAQ4plex); C11(Carbar	1	1
[K].ekGEIQNILQk.[V]	N-Term(iTRAQ4plex); K2(iTRAQ4	1	1
[K].EkGEIQNILQk.[V]	K2(iTRAQ4plex); K11(iTRAQ4plex	1	1
[K].eKGEIQNILQk.[V]	N-Term(iTRAQ4plex); K11(iTRAQ	1	1
[R].TLPEPcHSk.[I]	C6(Carbamidomethyl); K9(iTRAC	1	1
[R].eQPGVYTk.[V]	N-Term(iTRAQ4plex); K8(iTRAQ4	1	1
[K].dTPFSQIk.[E]	N-Term(iTRAQ4plex); K8(iTRAQ4	1	1
[R].lVGITSWGEGcAR.[R]	N-Term(iTRAQ4plex); C11(Carbar	1	1
[K].dTPFSQIk.[E]	N-Term(iTRAQ4plex); K8(iTRAQ4	1	1
[R].FAHYVVTSQVVNTANEAR.[E]		1	1
[R].gMADQDGLkPTIDkPSEDSPPLEmLGPR.[	N-Term(iTRAQ4plex); K9(iTRAQ4	1	1
[R].gMADQDGLkPTIDkPSEDSPPLEmLGPR.[	N-Term(iTRAQ4plex); K9(iTRAQ4	1	1
[R].gMADQDGLkPTIDkPSEDSPPLEmLGPR.[	N-Term(iTRAQ4plex); K9(iTRAQ4	1	1
[R].FAHYVVTSQVVNTANEAR.[E]		1	1
[R].gMADQDGLkPTIDkPSEDSPPLEMLGPR.[	N-Term(iTRAQ4plex); K9(iTRAQ4	1	1
[R].gMADQDGLkPTIDkPSEDSPPLEMLGPR.[	N-Term(iTRAQ4plex); K9(iTRAQ4	1	1
[R].gMADQDGLkPTIDkPSEDSPPLEMLGPR.[	N-Term(iTRAQ4plex); K9(iTRAQ4	1	1
[R].gMADQDGLkPTIDkPSEDSPPLEMLGPR.[	N-Term(iTRAQ4plex); K9(iTRAQ4	1	1
[R].gMADQDGLkPTIDkPSEDSPPLEMLGPR.[	N-Term(iTRAQ4plex); K9(iTRAQ4	1	1
[R].gMADQDGLkPTIDkPSEDSPPLEMLGPR.[	N-Term(iTRAQ4plex); K9(iTRAQ4	1	1
[R].gmADQDGLkPTIDkPSEDSPPLEMLGPR.[	N-Term(iTRAQ4plex); M2(Oxidat	1	1
[R].fAHYVVTSQVVNTANEAR.[E]	N-Term(iTRAQ4plex)	1	1

[R].fAHYVVTSQVVNTANEAR.[E]	N-Term(iTRAQ4plex)	1	1
[R].gHMLENHVER.[L]	N-Term(iTRAQ4plex)	1	1
[R].eVAFDLEIPk.[T]	N-Term(iTRAQ4plex); K10(iTRAQ4	1	1
[R].eVAFDLEIPk.[T]	N-Term(iTRAQ4plex); K10(iTRAQ4	1	1
[R].qAVDTAVDGVFIR.[S]	N-Term(iTRAQ4plex)	1	1
[R].qAVDTAVDGVFIR.[S]	N-Term(iTRAQ4plex)	1	1
[R].QAVDTAVDGVFIR.[S]		1	1
[R].gMADQDGLkPTIDkPSEDSPPLEMLGPR.[	N-Term(iTRAQ4plex); K9(iTRAQ4	1	1
[R].gMADQDGLkPTIDkPSEDSPPLEMLGPR.[	N-Term(iTRAQ4plex); K9(iTRAQ4	1	1
[R].eVAFDLEIPk.[T]	N-Term(iTRAQ4plex); K10(iTRAQ4	1	1
[R].fAHYVVTSQVVNTANEAR.[E]	N-Term(iTRAQ4plex)	1	1
[R].fAHYVVTSQVVNTANEAR.[E]	N-Term(iTRAQ4plex)	1	1
[R].fAHYVVTSQVVNTANEAR.[E]	N-Term(iTRAQ4plex)	1	1
[R].fAHYVVTSQVVNTANEAR.[E]	N-Term(iTRAQ4plex)	1	1
[R].fAHYVVTSQVVNTANEAR.[E]	N-Term(iTRAQ4plex)	1	1
[R].fAHYVVTSQVVNTANEAR.[E]	N-Term(iTRAQ4plex)	1	1
[R].nHMQYEIVIk.[V]	N-Term(iTRAQ4plex); K10(iTRAQ4	1	1
[R].nHMQYEIVIk.[V]	N-Term(iTRAQ4plex); K10(iTRAQ4	1	1
[R].gHMLENHVER.[L]	N-Term(iTRAQ4plex)	1	1
[R].kAAISGENAGLVR.[A]	N-Term(iTRAQ4plex); K1(iTRAQ4	1	1
[R].kAAISGENAGLVR.[A]	N-Term(iTRAQ4plex); K1(iTRAQ4	1	1
[R].FAHYVVTSQVVNTANEAR.[E]		1	1
[R].FAHYVVTSQVVNTANEAR.[E]		1	1
[R].FAHYVVTSQVVNTANEAR.[E]		1	1
[R].FAHYVVTSQVVNTANEAR.[E]		1	1
[R].fAHYVVTSQVVNTANEAR.[E]	N-Term(iTRAQ4plex)	1	1
[R].fAHYVVTSQVVNTANEAR.[E]	N-Term(iTRAQ4plex)	1	1
[R].tMEQFTIHLTVNPQSk.[V]	N-Term(iTRAQ4plex); K16(iTRAQ4	1	1
[R].tMEQFTIHLTVNPQSk.[V]	N-Term(iTRAQ4plex); K16(iTRAQ4	1	1
[R].tMEQFTIHLTVNPQSk.[V]	N-Term(iTRAQ4plex); K16(iTRAQ4	1	1
[R].tMEQFTIHLTVNPQSk.[V]	N-Term(iTRAQ4plex); K16(iTRAQ4	1	1
[K].lDAQASFLPk.[E]	N-Term(iTRAQ4plex); K10(iTRAQ4	1	1
[K].lDAQASFLPk.[E]	N-Term(iTRAQ4plex); K10(iTRAQ4	1	1
[K].lDAQASFLPk.[E]	N-Term(iTRAQ4plex); K10(iTRAQ4	1	1
[R].GHMLENHVER.[L]		1	1
[R].gHMLENHVER.[L]	N-Term(iTRAQ4plex)	1	1
[K].lDAQASFLPk.[E]	N-Term(iTRAQ4plex); K10(iTRAQ4	1	1
[R].kAAISGENAGLVR.[A]	N-Term(iTRAQ4plex)	1	1
[R].kAAISGENAGLVR.[A]	K1(iTRAQ4plex)	1	1
[R].nHMQYEIVIk.[V]	N-Term(iTRAQ4plex); K10(iTRAQ4	1	1
[K].qLVHHFEIDVDIFEPQGISk.[L]	N-Term(iTRAQ4plex); K20(iTRAQ4	1	1
[K].qLVHHFEIDVDIFEPQGISk.[L]	N-Term(iTRAQ4plex); K20(iTRAQ4	1	1
[K].vTFQLTYEEVLk.[R]	N-Term(iTRAQ4plex); K12(iTRAQ4	1	1
[K].vTFQLTYEEVLk.[R]	N-Term(iTRAQ4plex); K12(iTRAQ4	1	1
[K].tAFISDFAVTADGNAFIGDIk.[D]	N-Term(iTRAQ4plex); K21(iTRAQ4	1	1
[K].gSLVQASEANLQAAQDFVR.[G]	N-Term(iTRAQ4plex)	1	1
[K].gSLVQASEANLQAAQDFVR.[G]	N-Term(iTRAQ4plex)	1	1
[K].gSLVQASEANLQAAQDFVR.[G]	N-Term(iTRAQ4plex)	1	1
[K].gSLVQASEANLQAAQDFVR.[G]	N-Term(iTRAQ4plex)	1	1
[K].qYYEGSEIVVAGR.[I]	N-Term(iTRAQ4plex)	1	1
[K].qYYEGSEIVVAGR.[I]	N-Term(iTRAQ4plex)	1	1
[K].qYYEGSEIVVAGR.[I]	N-Term(iTRAQ4plex)	1	1

[K].qYYEGSEIVVAGR.[I]	N-Term(iTRAQ4plex)	1	1
[R].gHMLENHVER.[L]	N-Term(iTRAQ4plex)	1	1
[R].gHMLENHVER.[L]	N-Term(iTRAQ4plex)	1	1
[K].tAFISDFAVTADGNAFIGDIk.[D]	N-Term(iTRAQ4plex); K21(iTRAQ	1	1
[R].iADNkQSSFk.[A]	N-Term(iTRAQ4plex); K5(iTRAQ4	1	1
[K].nVVFVIDISGSMR.[G]	N-Term(iTRAQ4plex)	1	1
[K].aDVQAHGEGQEFSITcLVDEEEMk.[K]	N-Term(iTRAQ4plex); C16(Carbar	1	1
[K].eLAAQTIk.[K]	N-Term(iTRAQ4plex); K8(iTRAQ4	1	1
[R].gHmLENHVER.[L]	N-Term(iTRAQ4plex); M3(Oxidat	1	1
[R].gHmLENHVER.[L]	N-Term(iTRAQ4plex); M3(Oxidat	1	1
[R].gHmLENHVER.[L]	N-Term(iTRAQ4plex); M3(Oxidat	1	1
[R].gHmLENHVER.[L]	N-Term(iTRAQ4plex); M3(Oxidat	1	1
[R].gHmLENHVER.[L]	N-Term(iTRAQ4plex); M3(Oxidat	1	1
[K].AAISGENAGLVR.[A]		1	1
[R].gHmLENHVER.[L]	N-Term(iTRAQ4plex); M3(Oxidat	1	1
[R].sPGQHDGTYFGR.[L]	N-Term(iTRAQ4plex)	1	1
[R].gHMLENHVER.[L]	N-Term(iTRAQ4plex)	1	1
[R].gHmLENHVER.[L]	N-Term(iTRAQ4plex); M3(Oxidat	1	1
[R].gHMLENHVER.[L]	N-Term(iTRAQ4plex)	1	1
[R].nHMQYEIVIk.[V]	N-Term(iTRAQ4plex); K10(iTRAQ	1	1
[R].nHMQYEIVIk.[V]	N-Term(iTRAQ4plex); K10(iTRAQ	1	1
[R].nHMQYEIVIk.[V]	N-Term(iTRAQ4plex); K10(iTRAQ	1	1
[R].nHMQYEIVIk.[V]	N-Term(iTRAQ4plex); K10(iTRAQ	1	1
[K].tAFISDFAVTADGNAFIGDIk.[D]	N-Term(iTRAQ4plex); K21(iTRAQ	1	1
[K].iLGDMQPGDYFDLVLFGTR.[V]	N-Term(iTRAQ4plex)	1	1
[K].qYYEGSEIVVAGR.[I]	N-Term(iTRAQ4plex)	1	1
[R].tMEQFTIHLTVNPQSk.[V]	N-Term(iTRAQ4plex); K16(iTRAQ	1	1
[R].lWAYLTIQELLAk.[R]	N-Term(iTRAQ4plex); K13(iTRAQ	1	1
[K].iLGDMQPGDYFDLVLFGTR.[V]	N-Term(iTRAQ4plex)	1	1
[R].qAVDTAVDGVFIR.[S]	N-Term(iTRAQ4plex)	1	1
[R].GHMLENHVER.[L]		1	1
[R].nHmQYEIVIk.[V]	N-Term(iTRAQ4plex); M3(Oxidat	1	1
[R].nHmQYEIVIk.[V]	N-Term(iTRAQ4plex); M3(Oxidat	1	1
[R].gHmLENHVER.[L]	N-Term(iTRAQ4plex); M3(Oxidat	1	1
[K].aAISGENAGLVR.[A]	N-Term(iTRAQ4plex)	1	1
[K].aAISGENAGLVR.[A]	N-Term(iTRAQ4plex)	1	1
[K].GSLVQASEANLQAAQDFVR.[G]		1	1
[K].qLVHHFEIDVDIFEPQGISk.[L]	N-Term(iTRAQ4plex); K20(iTRAQ	1	1
[K].lDAQASFLPk.[E]	N-Term(iTRAQ4plex); K10(iTRAQ	1	1
[K].vTFQLTYEEVLk.[R]	N-Term(iTRAQ4plex); K12(iTRAQ	1	1
[K].aAISGENAGLVR.[A]	N-Term(iTRAQ4plex)	1	1
[R].fAHYVVTSQVVNTANEAR.[E]	N-Term(iTRAQ4plex)	1	1
[K].vTFQLTYEEVLk.[R]	N-Term(iTRAQ4plex); K12(iTRAQ	1	1
[R].QAVDTAVDGVFIR.[S]		1	1
[R].tMEQFTIHLTVNPQSk.[V]	N-Term(iTRAQ4plex); K16(iTRAQ	1	1
[K].gSLVQASEANLQAAQDFVR.[G]	N-Term(iTRAQ4plex)	1	1
[R].tFVLSALQPSPTHSSSNTQR.[L]	N-Term(iTRAQ4plex)	1	1
[R].gMADQDGLkPTIDkPSEDSPPLEMLGPR.[	N-Term(iTRAQ4plex); K9(iTRAQ4	1	1
[R].FAHYVVTSQVVNTANEAR.[E]		1	1
[R].gMADQDGLkPTIDkPSEDSPPLEMLGPR.[	N-Term(iTRAQ4plex); K9(iTRAQ4	1	1
[R].GHmLENHVER.[L]	M3(Oxidation)	1	1
[K].aAISGENAGLVR.[A]	N-Term(iTRAQ4plex)	1	1

[R].iADNkQSSFk.[A]	N-Term(iTRAQ4plex); K5(iTRAQ4	1	1
[K].nVVFVIDISGSMR.[G]	N-Term(iTRAQ4plex)	1	1
[K].eLAAQTIk.[K]	N-Term(iTRAQ4plex); K8(iTRAQ4	1	1
[K].nVVFVIDISGSMR.[G]	N-Term(iTRAQ4plex)	1	1
[R].FAHYVVTSQVVNTANEAR.[E]		1	1
[K].LDAQASFLPk.[E]	K10(iTRAQ4plex)	1	1
[R].FAHYVVTSQVVNTANEAR.[E]		1	1
[K].qLVHHFEIDVDIFEPQGISk.[L]	N-Term(iTRAQ4plex); K20(iTRAQ	1	1
[K].rQAVDTAVDGVFIR.[S]	N-Term(iTRAQ4plex)	1	1
[K].gSLVQASEANLQAAQDFVR.[G]	N-Term(iTRAQ4plex)	1	1
[K].QYYEGSEIVVAGR.[I]		1	1
[R].qDGVVVTINk.[K]	N-Term(iTRAQ4plex); K10(iTRAQ	1	1
[R].fAHYVVTSQVVNTANEAR.[E]	N-Term(iTRAQ4plex)	1	1
[R].fAHYVVTSQVVNTANEAR.[E]	N-Term(iTRAQ4plex)	1	1
[K].vTFQLTYEEVLk.[R]	N-Term(iTRAQ4plex); K12(iTRAQ	1	1
[R].GHmLENHVER.[L]	M3(Oxidation)	1	1
[R].qAVDTAVDGVFIR.[S]	N-Term(iTRAQ4plex)	1	1
[K].qLVHHFEIDVDIFEPQGISk.[L]	N-Term(iTRAQ4plex); K20(iTRAQ	1	1
[R].FAHYVVTSQVVNTANEAR.[E]		1	1
[R].FAHYVVTSQVVNTANEAR.[E]		1	1
[K].lDAQASFLPk.[E]	N-Term(iTRAQ4plex); K10(iTRAQ	1	1
[R].gMADQDGLkPTIDkPSEDSPPLEmLGPR.[	N-Term(iTRAQ4plex); K9(iTRAQ4	1	1
[K].nVVFVIDISGSMR.[G]	N-Term(iTRAQ4plex)	1	1
[K].qLVHHFEIDVDIFEPQGISk.[L]	N-Term(iTRAQ4plex); K20(iTRAQ	1	1
[K].lQHLENELTHDIITk.[F]	N-Term(iTRAQ4plex); K15(iTRAQ	1	1
[K].lYHSEAFTVNFGDTEEAk.[K]	N-Term(iTRAQ4plex); K18(iTRAQ	1	1
[K].lYHSEAFTVNFGDTEEAk.[K]	N-Term(iTRAQ4plex); K18(iTRAQ	1	1
[K].lYHSEAFTVNFGDTEEAk.[K]	N-Term(iTRAQ4plex); K18(iTRAQ	1	1
[K].lYHSEAFTVNFGDTEEAk.[K]	N-Term(iTRAQ4plex); K18(iTRAQ	1	1
[R].lGMFNIQHck.[K]	N-Term(iTRAQ4plex); C9(Carbam	1	1
[R].lGMFNIQHck.[K]	N-Term(iTRAQ4plex); C9(Carbam	1	1
[R].lGMFNIQHck.[K]	N-Term(iTRAQ4plex); C9(Carbam	1	1
[R].lGMFNIQHck.[K]	N-Term(iTRAQ4plex); C9(Carbam	1	1
[K].vFSNGADLSGVTEEAPLk.[L]	N-Term(iTRAQ4plex); K18(iTRAQ	1	1
[R].lGmFNIQHck.[K]	N-Term(iTRAQ4plex); M3(Oxidat	1	1
[K].rLGMFNIQHck.[K]	N-Term(iTRAQ4plex); C10(Carbar	1	1
[K].lQHLENELTHDIITk.[F]	N-Term(iTRAQ4plex); K15(iTRAQ	1	1
[K].lQHLENELTHDIITk.[F]	N-Term(iTRAQ4plex); K15(iTRAQ	1	1
[K].lQHLENELTHDIITk.[F]	N-Term(iTRAQ4plex); K15(iTRAQ	1	1
[K].lQHLENELTHDIITk.[F]	N-Term(iTRAQ4plex); K15(iTRAQ	1	1
[K].lQHLENELTHDIITk.[F]	N-Term(iTRAQ4plex); K15(iTRAQ	1	1
[K].lQHLENELTHDIITk.[F]	N-Term(iTRAQ4plex); K15(iTRAQ	1	1
[K].lQHLENELTHDIITk.[F]	N-Term(iTRAQ4plex); K15(iTRAQ	1	1
[K].lQHLENELTHDIITk.[F]	N-Term(iTRAQ4plex); K15(iTRAQ	1	1
[K].lQHLENELTHDIITk.[F]	N-Term(iTRAQ4plex); K15(iTRAQ	1	1
[K].lQHLENELTHDIITk.[F]	N-Term(iTRAQ4plex); K15(iTRAQ	1	1
[K].lQHLENELTHDIITk.[F]	N-Term(iTRAQ4plex); K15(iTRAQ	1	1
[K].lQHLENELTHDIITk.[F]	N-Term(iTRAQ4plex); K15(iTRAQ	1	1
[K].lQHLENELTHDIITk.[F]	N-Term(iTRAQ4plex); K15(iTRAQ	1	1
[K].lQHLENELTHDIITk.[F]	N-Term(iTRAQ4plex); K15(iTRAQ	1	1
[K].lQHLENELTHDIITk.[F]	N-Term(iTRAQ4plex); K15(iTRAQ	1	1
[K].lQHLENELTHDIITk.[F]	N-Term(iTRAQ4plex); K15(iTRAQ	1	1

[K].lQHLENELTHDIITk.[F]	N-Term(iTRAQ4plex); K15(iTRAQ	1	1
[K].lQHLENELTHDIITk.[F]	N-Term(iTRAQ4plex); K15(iTRAQ	1	1
[K].lYHSEAFTVNFGDTEEAk.[K]	N-Term(iTRAQ4plex); K18(iTRAQ	1	1
[K].tDTSHHDQDHPTFNk.[I]	N-Term(iTRAQ4plex); K15(iTRAQ	1	1
[K].tDTSHHDQDHPTFNk.[I]	N-Term(iTRAQ4plex); K15(iTRAQ	1	1
[K].LYHSEAFTVNFGDTEEAk.[K]	K18(iTRAQ4plex)	1	1
[K].LYHSEAFTVNFGDTEEAk.[K]	K18(iTRAQ4plex)	1	1
[K].LYHSEAFTVNFGDTEEAk.[K]	K18(iTRAQ4plex)	1	1
[K].aVLTIDEk.[G]	N-Term(iTRAQ4plex); K8(iTRAQ4	1	1
[K].tDTSHHDQDHPTFNk.[I]	N-Term(iTRAQ4plex); K15(iTRAQ	1	1
[K].tDTSHHDQDHPTFNk.[I]	N-Term(iTRAQ4plex); K15(iTRAQ	1	1
[R].lGMFNIQHck.[K]	N-Term(iTRAQ4plex); C9(Carbam	1	1
[R].LGMFNIQHck.[K]	C9(Carbamidomethyl); K10(iTRA	1	1
[R].LGMFNIQHck.[K]	C9(Carbamidomethyl); K10(iTRA	1	1
[K].lSITGTYDLk.[S]	N-Term(iTRAQ4plex); K10(iTRAQ	1	1
[K].lSITGTYDLk.[S]	N-Term(iTRAQ4plex); K10(iTRAQ	1	1
[K].gTEAAGAMFLEAIPMSIPPEVk.[F]	N-Term(iTRAQ4plex); K22(iTRAQ	1	1
[R].tLNQPDSQLQLTTGNGLFLSEGLk.[L]	N-Term(iTRAQ4plex); K24(iTRAQ	1	1
[K].gTEAAGAMFLEAIPmSIPPEVk.[F]	N-Term(iTRAQ4plex); M15(Oxida	1	1
[K].LYHSEAFTVNFGDTEEAk.[K]	K18(iTRAQ4plex)	1	1
[K].tDTSHHDQDHPTFNk.[I]	N-Term(iTRAQ4plex); K15(iTRAQ	1	1
[K].tDTSHHDQDHPTFNk.[I]	N-Term(iTRAQ4plex); K15(iTRAQ	1	1
[K].tDTSHHDQDHPTFNk.[I]	N-Term(iTRAQ4plex); K15(iTRAQ	1	1
[K].tDTSHHDQDHPTFNk.[I]	N-Term(iTRAQ4plex); K15(iTRAQ	1	1
[K].tDTSHHDQDHPTFNk.[I]	N-Term(iTRAQ4plex); K15(iTRAQ	1	1
[K].tDTSHHDQDHPTFNk.[I]	N-Term(iTRAQ4plex); K15(iTRAQ	1	1
[K].tDTSHHDQDHPTFNk.[I]	N-Term(iTRAQ4plex); K15(iTRAQ	1	1
[K].tDTSHHDQDHPTFNk.[I]	N-Term(iTRAQ4plex); K15(iTRAQ	1	1
[K].tDTSHHDQDHPTFNk.[I]	N-Term(iTRAQ4plex); K15(iTRAQ	1	1
[K].tDTSHHDQDHPTFNk.[I]	N-Term(iTRAQ4plex); K15(iTRAQ	1	1
[K].tDTSHHDQDHPTFNk.[I]	N-Term(iTRAQ4plex); K15(iTRAQ	1	1
[K].dTEEEDFHVDQVTTVk.[V]	N-Term(iTRAQ4plex); K16(iTRAQ	1	1
[K].dTEEEDFHVDQVTTVk.[V]	N-Term(iTRAQ4plex); K16(iTRAQ	1	1
[K].lSSWVLLMk.[Y]	N-Term(iTRAQ4plex); K9(iTRAQ4	1	1
[K].lSSWVLLMk.[Y]	N-Term(iTRAQ4plex); K9(iTRAQ4	1	1
[K].fNkPFVFLMIEQNTk.[S]	N-Term(iTRAQ4plex); K3(iTRAQ4	1	1
[K].fNkPFVFLMIEQNTk.[S]	N-Term(iTRAQ4plex); K3(iTRAQ4	1	1
[K].aVLTIDEk.[G]	N-Term(iTRAQ4plex); K8(iTRAQ4	1	1
[K].dTEEEDFHVDQVTTVk.[V]	N-Term(iTRAQ4plex); K16(iTRAQ	1	1
[K].dTEEEDFHVDQVTTVk.[V]	N-Term(iTRAQ4plex); K16(iTRAQ	1	1
[K].dTEEEDFHVDQVTTVk.[V]	N-Term(iTRAQ4plex); K16(iTRAQ	1	1
[K].dTEEEDFHVDQVTTVk.[V]	N-Term(iTRAQ4plex); K16(iTRAQ	1	1
[K].dTEEEDFHVDQVTTVk.[V]	N-Term(iTRAQ4plex); K16(iTRAQ	1	1
[K].dTEEEDFHVDQVTTVk.[V]	N-Term(iTRAQ4plex); K16(iTRAQ	1	1
[K].LQHLENELTHDIITk.[F]	K15(iTRAQ4plex)	1	1
[K].sVLGQLGITk.[V]	N-Term(iTRAQ4plex); K10(iTRAQ	1	1
[K].sVLGQLGITk.[V]	N-Term(iTRAQ4plex); K10(iTRAQ	1	1
[K].LQHLENELTHDIITk.[F]	K15(iTRAQ4plex)	1	1
[K].LQHLENELTHDIITk.[F]	K15(iTRAQ4plex)	1	1
[K].LQHLENELTHDIITk.[F]	K15(iTRAQ4plex)	1	1
[K].aVLTIDEk.[G]	N-Term(iTRAQ4plex); K8(iTRAQ4	1	1
[K].aVLTIDEk.[G]	N-Term(iTRAQ4plex); K8(iTRAQ4	1	1

[R].dTVFALVNYIFFk.[G]	N-Term(iTRAQ4plex); K13(iTRAQ4	1	1
[K].eLDRDTVFALVNYIFFk.[G]	N-Term(iTRAQ4plex); K17(iTRAQ4	1	1
[K].kLSSWVLLMk.[Y]	N-Term(iTRAQ4plex); K1(iTRAQ4	1	1
[K].kQINDYVEk.[G]	N-Term(iTRAQ4plex); K1(iTRAQ4	1	1
[K].iTPNLAEFAFSLYR.[Q]	N-Term(iTRAQ4plex)	1	1
[K].fNkPFVFLMIEQNTk.[S]	N-Term(iTRAQ4plex); K3(iTRAQ4	1	1
[K].lSITGTYDLk.[S]	N-Term(iTRAQ4plex); K10(iTRAQ4	1	1
[K].sVLGQLGITk.[V]	N-Term(iTRAQ4plex); K10(iTRAQ4	1	1
[K].qINDYVEk.[G]	N-Term(iTRAQ4plex); K8(iTRAQ4	1	1
[K].lYHSEAFTVNFGDTEEAk.[K]	N-Term(iTRAQ4plex); K18(iTRAQ4	1	1
[K].SVLGQLGITk.[V]	K10(iTRAQ4plex)	1	1
[K].dTEEEDFHVDQVTTVk.[V]	N-Term(iTRAQ4plex); K16(iTRAQ4	1	1
[K].tDTSHHDQDHPTFNk.[I]	N-Term(iTRAQ4plex); K15(iTRAQ4	1	1
[R].lGmFNIQHck.[K]	N-Term(iTRAQ4plex); M3(Oxidati	1	1
[K].eLDRDTVFALVNYIFFk.[G]	N-Term(iTRAQ4plex); K17(iTRAQ4	1	1
[R].lGMFNIQHck.[K]	N-Term(iTRAQ4plex); C9(Carbam	1	1
[K].dTEEEDFHVDQVTTVk.[V]	N-Term(iTRAQ4plex); K16(iTRAQ4	1	1
[R].lGmFNIQHck.[K]	N-Term(iTRAQ4plex); M3(Oxidati	1	1
[K].lQHLENELTHDIITk.[F]	N-Term(iTRAQ4plex); K15(iTRAQ4	1	1
[K].LQHLENELTHDIITk.[F]	K15(iTRAQ4plex)	1	1
[K].lSSWVLLMk.[Y]	N-Term(iTRAQ4plex); K9(iTRAQ4	1	1
[K].dTEEEDFHVDQVTTVk.[V]	N-Term(iTRAQ4plex); K16(iTRAQ4	1	1
[K].rLGMFNIQHck.[K]	N-Term(iTRAQ4plex); C10(Carban	1	1
[K].sVLGQLGITk.[V]	N-Term(iTRAQ4plex); K10(iTRAQ4	1	1
[K].gkWERPFEVk.[D]	N-Term(iTRAQ4plex); K2(iTRAQ4	1	1
[K].AVLTIDEk.[G]	K8(iTRAQ4plex)	1	1
[K].fNkPFVFLMIEQNTkSPLFMGkVVNPTQk.[	N-Term(iTRAQ4plex); K3(iTRAQ4	1	1
[K].TDTSHHDQDHPTFNk.[I]	K15(iTRAQ4plex)	1	1
[K].gkWERPFEVk.[D]	N-Term(iTRAQ4plex); K2(iTRAQ4	1	1
[R].lGmFNIQHck.[K]	N-Term(iTRAQ4plex); M3(Oxidati	1	1
[R].lGMFNIQHck.[K]	N-Term(iTRAQ4plex); C9(Carbam	1	1
[K].lSITGTYDLk.[S]	N-Term(iTRAQ4plex); K10(iTRAQ4	1	1
[K].kLSSWVLLMk.[Y]	K1(iTRAQ4plex); K10(iTRAQ4plex	1	1
[K].kLSSWVLLMk.[Y]	N-Term(iTRAQ4plex); K10(iTRAQ4	1	1
[K].lYHSEAFTVNFGDTEEAk.[K]	N-Term(iTRAQ4plex); K18(iTRAQ4	1	1
[R].lGMFNIQHck.[K]	N-Term(iTRAQ4plex); C9(Carbam	1	1
[K].lQHLENELTHDIITk.[F]	N-Term(iTRAQ4plex); K15(iTRAQ4	1	1
[R].lGmFNIQHck.[K]	N-Term(iTRAQ4plex); M3(Oxidati	1	1
[K].LQHLENELTHDIITk.[F]	K15(iTRAQ4plex)	1	1
[K].sPLFMGk.[V]	N-Term(iTRAQ4plex); K7(iTRAQ4	1	1
[K].rLGMFNIQHck.[K]	N-Term(iTRAQ4plex); C10(Carban	1	1
[K].DTEEEDFHVDQVTTVk.[V]	K16(iTRAQ4plex)	1	1
[R].lGMFNIQHck.[K]	N-Term(iTRAQ4plex); C9(Carbam	1	1
[K].tDTSHHDQDHPTFNk.[I]	N-Term(iTRAQ4plex); K15(iTRAQ4	1	1
[R].lGmFNIQHck.[K]	N-Term(iTRAQ4plex); M3(Oxidati	1	1
[R].lGMFNIQHck.[K]	N-Term(iTRAQ4plex); C9(Carbam	1	1
[R].lGmFNIQHck.[K]	N-Term(iTRAQ4plex); M3(Oxidati	1	1
[K].tDTSHHDQDHPTFNk.[I]	N-Term(iTRAQ4plex); K15(iTRAQ4	1	1
[K].aVLTIDEk.[G]	N-Term(iTRAQ4plex); K8(iTRAQ4	1	1
[K].kLSSWVLLmk.[Y]	N-Term(iTRAQ4plex); K1(iTRAQ4	1	1
[K].tDTSHHDQDHPTFNk.[I]	N-Term(iTRAQ4plex); K15(iTRAQ4	1	1
[R].sASLHLPk.[L]	N-Term(iTRAQ4plex); K8(iTRAQ4	1	1

[K].QINDYVEk.[G]	K8(iTRAQ4plex)	1	1
[K].qINDYVEk.[G]	N-Term(iTRAQ4plex); K8(iTRAQ4	1	1
[R].sASLHLPk.[L]	N-Term(iTRAQ4plex); K8(iTRAQ4	1	1
[R].LGmFNIQHck.[K]	M3(Oxidation); C9(Carbamidom	1	1
[K].sPLFMGk.[V]	N-Term(iTRAQ4plex); K7(iTRAQ4	1	1
[K].sPLFMGk.[V]	N-Term(iTRAQ4plex); K7(iTRAQ4	1	1
[K].TDTSHHDQDHPTFNk.[I]	K15(iTRAQ4plex)	1	1
[K].LYHSEAFTVNFGDTEEAk.[K]	K18(iTRAQ4plex)	1	1
[K].sPLFMGk.[V]	N-Term(iTRAQ4plex); K7(iTRAQ4	1	1
[R].lGMFNIQHcK.[K]	N-Term(iTRAQ4plex); C9(Carbam	1	1
[K].wERPFEVk.[D]	N-Term(iTRAQ4plex); K8(iTRAQ4	1	1
[R].sASLHLPk.[L]	N-Term(iTRAQ4plex); K8(iTRAQ4	1	1
[K].DTEEEDFHVDQVTTVk.[V]	K16(iTRAQ4plex)	1	1
[K].TDTSHHDQDHPTFNk.[I]	K15(iTRAQ4plex)	1	1
[K].SVLGQLGITk.[V]	K10(iTRAQ4plex)	1	1
[K].aVLTIDEk.[G]	N-Term(iTRAQ4plex); K8(iTRAQ4	1	1
[R].lGmFNIQHck.[K]	N-Term(iTRAQ4plex); M3(Oxidat	1	1
[K].wERPFEVk.[D]	N-Term(iTRAQ4plex); K8(iTRAQ4	1	1
[R].sASLHLPk.[L]	N-Term(iTRAQ4plex); K8(iTRAQ4	1	1
[K].SVLGQLGITk.[V]	K10(iTRAQ4plex)	1	1
[K].LYHSEAFTVNFGDTEEAk.[K]	K18(iTRAQ4plex)	1	1
[K].sPLFmGk.[V]	N-Term(iTRAQ4plex); M5(Oxidat	1	1
[K].sPLFMGk.[V]	N-Term(iTRAQ4plex); K7(iTRAQ4	1	1
[K].TDTSHHDQDHPTFNk.[I]	K15(iTRAQ4plex)	1	1
[K].wERPFEVk.[D]	N-Term(iTRAQ4plex); K8(iTRAQ4	1	1
[K].sPLFMGk.[V]	N-Term(iTRAQ4plex); K7(iTRAQ4	1	1
[R].lGmFNIQHck.[K]	N-Term(iTRAQ4plex); M3(Oxidat	1	1
[R].lGMFNIQHck.[K]	N-Term(iTRAQ4plex); C9(Carbam	1	1
[R].sASLHLPk.[L]	N-Term(iTRAQ4plex); K8(iTRAQ4	1	1
[R].lGmFNIQHck.[K]	N-Term(iTRAQ4plex); M3(Oxidat	1	1
[K].wERPFEVk.[D]	N-Term(iTRAQ4plex); K8(iTRAQ4	1	1
[R].lGmFNIQHck.[K]	N-Term(iTRAQ4plex); M3(Oxidat	1	1
[K].wERPFEVk.[D]	N-Term(iTRAQ4plex); K8(iTRAQ4	1	1
[K].lYHSEAFTVNFGDTEEAk.[K]	N-Term(iTRAQ4plex); K18(iTRAQ	1	1
[K].rLGMFNIQHck.[K]	N-Term(iTRAQ4plex); C10(Carbar	1	1
[K].lSITGTYDLk.[S]	N-Term(iTRAQ4plex); K10(iTRAQ	1	1
[R].sASLHLPk.[L]	N-Term(iTRAQ4plex); K8(iTRAQ4	1	1
[R].lGmFNIQHck.[K]	N-Term(iTRAQ4plex); M3(Oxidat	1	1
[K].TDTSHHDQDHPTFNk.[I]	K15(iTRAQ4plex)	1	1
[K].aVLTIDEk.[G]	N-Term(iTRAQ4plex); K8(iTRAQ4	1	1
[K].lSITGTYDLk.[S]	N-Term(iTRAQ4plex); K10(iTRAQ	1	1
[R].lGmFNIQHck.[K]	N-Term(iTRAQ4plex); M3(Oxidat	1	1
[K].sPLFmGk.[V]	N-Term(iTRAQ4plex); M5(Oxidat	1	1
[K].wERPFEVk.[D]	N-Term(iTRAQ4plex); K8(iTRAQ4	1	1
[R].lGmFNIQHck.[K]	N-Term(iTRAQ4plex); M3(Oxidat	1	1
[K].lYHSEAFTVNFGDTEEAk.[K]	N-Term(iTRAQ4plex); K18(iTRAQ	1	1
[K].tDTSHHDQDHPTFNk.[I]	N-Term(iTRAQ4plex); K15(iTRAQ	1	1
[R].sASLHLPk.[L]	N-Term(iTRAQ4plex); K8(iTRAQ4	1	1
[R].lGmFNIQHck.[K]	N-Term(iTRAQ4plex); M3(Oxidat	1	1
[K].LQHLENELTHDIITk.[F]	K15(iTRAQ4plex)	1	1
[K].TDTSHHDQDHPTFNk.[I]	K15(iTRAQ4plex)	1	1
[R].lGmFNIQHck.[K]	N-Term(iTRAQ4plex); M3(Oxidat	1	1

[K].lQHLENELTHDIITk.[F]	N-Term(iTRAQ4plex); K15(iTRAQ	1	1
[R].eTAASLLQAGYk.[G]	N-Term(iTRAQ4plex); K12(iTRAQ	1	1
[R].eTAASLLQAGYk.[G]	N-Term(iTRAQ4plex); K12(iTRAQ	1	1
[R].eTAASLLQAGYk.[G]	N-Term(iTRAQ4plex); K12(iTRAQ	1	1
[R].rQEcSIPVcGQDQVTVAMTPR.[S]	N-Term(iTRAQ4plex); C4(Carbam	1	1
[R].rQEcSIPVcGQDQVTVAMTPR.[S]	N-Term(iTRAQ4plex); C4(Carbam	1	1
[R].rQEcSIPVcGQDQVTVAMTPR.[S]	N-Term(iTRAQ4plex); C4(Carbam	1	1
[R].iTDNMFcAGYkPDEGk.[R]	N-Term(iTRAQ4plex); C7(Carbam	1	1
[R].iTDNMFcAGYkPDEGk.[R]	N-Term(iTRAQ4plex); C7(Carbam	1	1
[R].iTDNMFcAGYkPDEGk.[R]	N-Term(iTRAQ4plex); C7(Carbam	1	1
[R].rQEcSIPVcGQDQVTVAMTPR.[S]	N-Term(iTRAQ4plex); C4(Carbam	1	1
[R].sGIEcQLWR.[S]	N-Term(iTRAQ4plex); C5(Carbam	1	1
[R].sGIEcQLWR.[S]	N-Term(iTRAQ4plex); C5(Carbam	1	1
[R].rQEcSIPVcGQDQVTVAMTPR.[S]	N-Term(iTRAQ4plex); C4(Carbam	1	1
[R].tATSEYQTFFNPR.[T]	N-Term(iTRAQ4plex)	1	1
[R].tATSEYQTFFNPR.[T]	N-Term(iTRAQ4plex)	1	1
[K].lAAcLEGNcAEGLGTNYR.[G]	N-Term(iTRAQ4plex); C4(Carbam	1	1
[K].lAAcLEGNcAEGLGTNYR.[G]	N-Term(iTRAQ4plex); C4(Carbam	1	1
[K].lAAcLEGNcAEGLGTNYR.[G]	N-Term(iTRAQ4plex); C4(Carbam	1	1
[K].lAAcLEGNcAEGLGTNYR.[G]	N-Term(iTRAQ4plex); C4(Carbam	1	1
[R].TATSEYQTFFNPR.[T]		1	1
[R].eLLESYIDGR.[I]	N-Term(iTRAQ4plex)	1	1
[R].eLLESYIDGR.[I]	N-Term(iTRAQ4plex)	1	1
[R].nPDSSTTGPWcYTTDPTVR.[R]	N-Term(iTRAQ4plex); C11(Carbar	1	1
[R].sEGSSVNLSPPLEQcVPDR.[G]	N-Term(iTRAQ4plex); C15(Carbar	1	1
[R].sEGSSVNLSPPLEQcVPDR.[G]	N-Term(iTRAQ4plex); C15(Carbar	1	1
[K].yGFYTHVFR.[L]	N-Term(iTRAQ4plex)	1	1
[R].rQEcSIPVcGQDQVTVAmTPR.[S]	N-Term(iTRAQ4plex); C4(Carbam	1	1
[K].hQDFNSAVQLVENFcR.[N]	N-Term(iTRAQ4plex); C15(Carbar	1	1
[R].vTGWGNLk.[E]	N-Term(iTRAQ4plex); K8(iTRAQ4	1	1
[K].kPVAFSDYIHPVcLPDR.[E]	N-Term(iTRAQ4plex); K1(iTRAQ4	1	1
[K].kPVAFSDYIHPVcLPDR.[E]	N-Term(iTRAQ4plex); K1(iTRAQ4	1	1
[K].kPVAFSDYIHPVcLPDR.[E]	N-Term(iTRAQ4plex); K1(iTRAQ4	1	1
[K].kPVAFSDYIHPVcLPDR.[E]	N-Term(iTRAQ4plex); K1(iTRAQ4	1	1
[K].kPVAFSDYIHPVcLPDR.[E]	N-Term(iTRAQ4plex); K1(iTRAQ4	1	1
[K].hQDFNSAVQLVENFcR.[N]	N-Term(iTRAQ4plex); C15(Carbar	1	1
[K].sPQELLcGASLISDR.[W]	N-Term(iTRAQ4plex); C7(Carbam	1	1
[K].sPQELLcGASLISDR.[W]	N-Term(iTRAQ4plex); C7(Carbam	1	1
[K].hQDFNSAVQLVENFcR.[N]	N-Term(iTRAQ4plex); C15(Carbar	1	1
[K].HQDFNSAVQLVENFcR.[N]	C15(Carbamidomethyl)	1	1
[K].hQDFNSAVQLVENFcR.[N]	N-Term(iTRAQ4plex); C15(Carbar	1	1
[K].sPQELLcGASLISDR.[W]	N-Term(iTRAQ4plex); C7(Carbam	1	1
[K].hQDFNSAVQLVENFcR.[N]	N-Term(iTRAQ4plex); C15(Carbar	1	1
[R].kSPQELLcGASLISDR.[W]	N-Term(iTRAQ4plex); K1(iTRAQ4	1	1
[R].kSPQELLcGASLISDR.[W]	N-Term(iTRAQ4plex); K1(iTRAQ4	1	1
[R].kSPQELLcGASLISDR.[W]	N-Term(iTRAQ4plex); K1(iTRAQ4	1	1
[R].kSPQELLcGASLISDR.[W]	N-Term(iTRAQ4plex); K1(iTRAQ4	1	1
[R].kSPQELLcGASLISDR.[W]	N-Term(iTRAQ4plex); K1(iTRAQ4	1	1
[R].kSPQELLcGASLISDR.[W]	N-Term(iTRAQ4plex); K1(iTRAQ4	1	1
[R].kSPQELLcGASLISDR.[W]	N-Term(iTRAQ4plex); C8(Carbam	1	1
[R].kSPQELLcGASLISDR.[W]	N-Term(iTRAQ4plex); C8(Carbam	1	1
[R].kSPQELLcGASLISDR.[W]	K1(iTRAQ4plex); C8(Carbamidon	1	1

[R].kSPQELLcGASLISDR.[W]	K1(iTRAQ4plex); C8(Carbamidon	1	1
[R].tATSEYQTFFNPR.[T]	N-Term(iTRAQ4plex)	1	1
[R].tATSEYQTFFNPR.[T]	N-Term(iTRAQ4plex)	1	1
[R].tFGSGEADcGLRPLFEk.[K]	N-Term(iTRAQ4plex); C9(Carbam	1	1
[R].tFGSGEADcGLRPLFEk.[K]	N-Term(iTRAQ4plex); C9(Carbam	1	1
[R].TFGSGEADcGLRPLFEk.[K]	C9(Carbamidomethyl); K17(iTRA	1	1
[R].tFGSGEADcGLRPLFEk.[K]	N-Term(iTRAQ4plex); C9(Carbam	1	1
[R].tFGSGEADcGLRPLFEk.[K]	N-Term(iTRAQ4plex); C9(Carbam	1	1
[R].iTDNMFcAGYkPDEGkR.[G]	N-Term(iTRAQ4plex); C7(Carbam	1	1
[K].gQPSVLQVVNLPIVERPVck.[D]	N-Term(iTRAQ4plex); C19(Carbar	1	1
[K].gQPSVLQVVNLPIVERPVck.[D]	N-Term(iTRAQ4plex); C19(Carbar	1	1
[K].gQPSVLQVVNLPIVERPVck.[D]	N-Term(iTRAQ4plex); C19(Carbar	1	1
[R].gDAcEGDSGGPFVMk.[S]	N-Term(iTRAQ4plex); C4(Carbam	1	1
[R].gDAcEGDSGGPFVMk.[S]	N-Term(iTRAQ4plex); C4(Carbam	1	1
[R].gDAcEGDSGGPFVMk.[S]	N-Term(iTRAQ4plex); C4(Carbam	1	1
[R].GDAcEGDSGGPFVMk.[S]	C4(Carbamidomethyl); K15(iTRA	1	1
[R].wYQmGIVSWGEGcDR.[D]	N-Term(iTRAQ4plex); M4(Oxidat	1	1
[R].sEGSSVNLSPPLEQcVPDR.[G]	N-Term(iTRAQ4plex); C15(Carbar	1	1
[R].SEGSSVNLSPPLEQcVPDR.[G]	C15(Carbamidomethyl)	1	1
[R].ELLESYIDGR.[I]		1	1
[K].hQDFNSAVQLVENFcR.[N]	N-Term(iTRAQ4plex); C15(Carbar	1	1
[R].SEGSSVNLSPPLEQcVPDR.[G]	C15(Carbamidomethyl)	1	1
[R].sEGSSVNLSPPLEQcVPDR.[G]	N-Term(iTRAQ4plex); C15(Carbar	1	1
[R].sEGSSVNLSPPLEQcVPDR.[G]	N-Term(iTRAQ4plex); C15(Carbar	1	1
[R].SEGSSVNLSPPLEQcVPDR.[G]	C15(Carbamidomethyl)	1	1
[R].wYQMGIVSWGEGcDR.[D]	N-Term(iTRAQ4plex); C13(Carbar	1	1
[R].wYQMGIVSWGEGcDR.[D]	N-Term(iTRAQ4plex); C13(Carbar	1	1
[R].wYQMGIVSWGEGcDR.[D]	N-Term(iTRAQ4plex); C13(Carbar	1	1
[R].gDAcEGDSGGPFVmk.[S]	N-Term(iTRAQ4plex); C4(Carbam	1	1
[K].rGDAcEGDSGGPFVMk.[S]	N-Term(iTRAQ4plex); C5(Carbam	1	1
[K].rGDAcEGDSGGPFVMk.[S]	N-Term(iTRAQ4plex); C5(Carbam	1	1
[R].wVLTAAHcLLYPPWDk.[N]	N-Term(iTRAQ4plex); C8(Carbam	1	1
[R].gDAcEGDSGGPFVmk.[S]	N-Term(iTRAQ4plex); C4(Carbam	1	1
[R].wVLTAAHcLLYPPWDk.[N]	N-Term(iTRAQ4plex); C8(Carbam	1	1
[R].iVEGSDAEIGMSPWQVMLFR.[K]	N-Term(iTRAQ4plex)	1	1
[R].ETAASLLQAGYk.[G]	K12(iTRAQ4plex)	1	1
[K].kPVAFSDYIHPVcLPDR.[E]	N-Term(iTRAQ4plex); K1(iTRAQ4	1	1
[R].lAVTTHGLPcLAWASAQAk.[A]	N-Term(iTRAQ4plex); C10(Carbar	1	1
[R].lAVTTHGLPcLAWASAQAk.[A]	N-Term(iTRAQ4plex); C10(Carbar	1	1
[R].LAVTTHGLPcLAWASAQAk.[A]	C10(Carbamidomethyl); K19(iTR	1	1
[K].kPVAFSDYIHPVcLPDR.[E]	N-Term(iTRAQ4plex); K1(iTRAQ4	1	1
[R].eTAASLLQAGYk.[G]	N-Term(iTRAQ4plex); K12(iTRAQ	1	1
[R].rQEcSIPVcGQDQVTVAMTPR.[S]	N-Term(iTRAQ4plex); C4(Carbam	1	1
[R].VTGWGNLk.[E]	K8(iTRAQ4plex)	1	1
[R].sEGSSVNLSPPLEQcVPDR.[G]	N-Term(iTRAQ4plex); C15(Carbar	1	1
[R].vTGWGNLk.[E]	N-Term(iTRAQ4plex); K8(iTRAQ4	1	1
[R].TFGSGEADcGLRPLFEk.[K]	C9(Carbamidomethyl); K17(iTRA	1	1
[R].kSPQELLcGASLISDR.[W]	N-Term(iTRAQ4plex); K1(iTRAQ4	1	1
[K].gQPSVLQVVNLPIVERPVck.[D]	N-Term(iTRAQ4plex); C19(Carbar	1	1
[K].hQDFNSAVQLVENFcR.[N]	N-Term(iTRAQ4plex); C15(Carbar	1	1
[K].ETWTANVGk.[G]	K9(iTRAQ4plex)	1	1
[R].sGIEcQLWR.[S]	N-Term(iTRAQ4plex); C5(Carbam	1	1

[R].gDAcEGDSGGPFVmk.[S]	N-Term(iTRAQ4plex); C4(Carbam	1	1
[R].gDAcEGDSGGPFVmk.[S]	N-Term(iTRAQ4plex); C4(Carbam	1	1
[R].eLLESYIDGR.[I]	N-Term(iTRAQ4plex)	1	1
[K].eTWTANVGk.[G]	N-Term(iTRAQ4plex); K9(iTRAQ4	1	1
[R].wYQmGIVSWGEGcDR.[D]	N-Term(iTRAQ4plex); M4(Oxidat	1	1
[R].VTGWGNLk.[E]	K8(iTRAQ4plex)	1	1
[K].YGFYTHVFR.[L]		1	1
[K].lAAcLEGNcAEGLGTNYR.[G]	N-Term(iTRAQ4plex); C4(Carbam	1	1
[R].wYQMGIVSWGEGcDR.[D]	N-Term(iTRAQ4plex); C13(Carbar	1	1
[R].iTDNMFcAGYkPDEGk.[R]	N-Term(iTRAQ4plex); C7(Carbam	1	1
[R].vTGWGNLK.[E]	N-Term(iTRAQ4plex)	1	1
[R].vTGWGNLk.[E]	N-Term(iTRAQ4plex); K8(iTRAQ4	1	1
[K].yTAcETAR.[T]	N-Term(iTRAQ4plex); C4(Carbam	1	1
[R].eTAASLLQAGYk.[G]	N-Term(iTRAQ4plex); K12(iTRAQ	1	1
[R].sEGSSVNLSPPLEQcVPDR.[G]	N-Term(iTRAQ4plex); C15(Carbar	1	1
[R].VTGWGNLk.[E]	K8(iTRAQ4plex)	1	1
[R].vTGWGNLk.[E]	N-Term(iTRAQ4plex); K8(iTRAQ4	1	1
[K].kPVAFSDYIHPVcLPDR.[E]	N-Term(iTRAQ4plex); C13(Carbar	1	1
[K].kPVAFSDYIHPVcLPDR.[E]	K1(iTRAQ4plex); C13(Carbamido	1	1
[R].iTDNmFcAGYkPDEGk.[R]	N-Term(iTRAQ4plex); M5(Oxidat	1	1
[R].vTGWGNLk.[E]	N-Term(iTRAQ4plex); K8(iTRAQ4	1	1
[K].kPVAFSDYIHPVcLPDR.[E]	N-Term(iTRAQ4plex); K1(iTRAQ4	1	1
[R].sEGSSVNLSPPLEQcVPDR.[G]	N-Term(iTRAQ4plex); C15(Carbar	1	1
[R].vTGWGNLk.[E]	N-Term(iTRAQ4plex); K8(iTRAQ4	1	1
[R].iTDNMFcAGYkPDEGk.[R]	N-Term(iTRAQ4plex); C7(Carbam	1	1
[R].wVLTAAHcLLYPPWDk.[N]	N-Term(iTRAQ4plex); C8(Carbam	1	1
[R].eTAASLLQAGYk.[G]	N-Term(iTRAQ4plex); K12(iTRAQ	1	1
[R].eLLESYIDGR.[I]	N-Term(iTRAQ4plex)	1	1
[R].gDAcEGDSGGPFVMk.[S]	N-Term(iTRAQ4plex); C4(Carbam	1	1
[K].kPVAFSDYIHPVcLPDR.[E]	N-Term(iTRAQ4plex); K1(iTRAQ4	1	1
[R].kSPQELLcGASLISDR.[W]	N-Term(iTRAQ4plex); K1(iTRAQ4	1	1
[K].yTAcETAR.[T]	N-Term(iTRAQ4plex); C4(Carbam	1	1
[R].nPDSSTTGPWcYTTDPTVR.[R]	N-Term(iTRAQ4plex); C11(Carbar	1	1
[R].sVAVYGQYGGQPcVGNAFETQScEPTR.[G	N-Term(iTRAQ4plex); C13(Carbar	1	1
[R].sVAVYGQYGGQPcVGNAFETQScEPTR.[G	N-Term(iTRAQ4plex); C13(Carbar	1	1
[R].wLVGEMHcQk.[I]	N-Term(iTRAQ4plex); C8(Carbam	1	1
[K].acGAcPLWGk.[C]	N-Term(iTRAQ4plex); C2(Carbam	1	1
[K].mHVLHcQGR.[N]	N-Term(iTRAQ4plex); C6(Carbam	1	1
[K].mPYEcGPSLDVcAQDER.[S]	N-Term(iTRAQ4plex); C5(Carbam	1	1
[K].dGFVQDEGTMFPVGk.[N]	N-Term(iTRAQ4plex); K15(iTRAQ	1	1
[R].lIDQYGTHYLQSGSLGGEYR.[V]	N-Term(iTRAQ4plex)	1	1
[K].lTPLYELVk.[E]	N-Term(iTRAQ4plex); K9(iTRAQ4	1	1
[K].lTPLYELVk.[E]	N-Term(iTRAQ4plex); K9(iTRAQ4	1	1
[R].vLFYVDSEk.[L]	N-Term(iTRAQ4plex); K9(iTRAQ4	1	1
[R].wLVGEMHcQk.[I]	N-Term(iTRAQ4plex); C8(Carbam	1	1
[R].lIDQYGTHYLQSGSLGGEYR.[V]	N-Term(iTRAQ4plex)	1	1
[K].eLSHLPSLYDYSAYR.[R]	N-Term(iTRAQ4plex)	1	1
[R].dScTLPASAEk.[A]	N-Term(iTRAQ4plex); C3(Carbam	1	1
[R].dScTLPASAEk.[A]	N-Term(iTRAQ4plex); C3(Carbam	1	1
[R].sYTSHTNEIHk.[G]	N-Term(iTRAQ4plex); K11(iTRAQ	1	1
[K].aASGTQNNVLR.[G]	N-Term(iTRAQ4plex)	1	1
[K].aASGTQNNVLR.[G]	N-Term(iTRAQ4plex)	1	1

[K].dGFVQDEGTmFPVGk.[N]	N-Term(iTRAQ4plex); M10(Oxida	1	1
[R].lSGNVLSYTFQVk.[I]	N-Term(iTRAQ4plex); K13(iTRAQ	1	1
[R].ySAWAESVTNLPQVIk.[Q]	N-Term(iTRAQ4plex); K16(iTRAQ	1	1
[R].ySAWAESVTNLPQVIk.[Q]	N-Term(iTRAQ4plex); K16(iTRAQ	1	1
[K].vTVScSGGMSLEGPSAFLcGSSLk.[W]	N-Term(iTRAQ4plex); C5(Carbam	1	1
[K].iAcVLPVLMDGIQSHPQkPFYTVGEk.[V]	N-Term(iTRAQ4plex); C3(Carbam	1	1
[K].eQTMSEcEAGALR.[C]	N-Term(iTRAQ4plex); C7(Carbam	1	1
[K].eQTMSEcEAGALR.[C]	N-Term(iTRAQ4plex); C7(Carbam	1	1
[K].eQTMSEcEAGALR.[C]	N-Term(iTRAQ4plex); C7(Carbam	1	1
[K].eQTMSEcEAGALR.[C]	N-Term(iTRAQ4plex); C7(Carbam	1	1
[R].rYSAWAESVTNLPQVIk.[Q]	N-Term(iTRAQ4plex); K17(iTRAQ	1	1
[K].eQTMSEcEAGALR.[C]	N-Term(iTRAQ4plex); C7(Carbam	1	1
[R].wLVGEmHcQk.[I]	N-Term(iTRAQ4plex); M6(Oxidat	1	1
[K].mHVLHcQGR.[N]	N-Term(iTRAQ4plex); C6(Carbam	1	1
[K].eLSHLPSLYDYSAYR.[R]	N-Term(iTRAQ4plex)	1	1
[K].lTPLYELVk.[E]	N-Term(iTRAQ4plex); K9(iTRAQ4	1	1
[R].gcPTEEGcGER.[F]	N-Term(iTRAQ4plex); C2(Carbam	1	1
[K].eLENALk.[AN]	N-Term(iTRAQ4plex); K7(iTRAQ4	1	2
[K].iAcVLPVLMDGIQSHPQkPFYTVGEk.[V]	N-Term(iTRAQ4plex); C3(Carbam	1	1
[R].wLVGEMHcQk.[I]	N-Term(iTRAQ4plex); C8(Carbam	1	1
[R].YSAWAESVTNLPQVIk.[Q]	K16(iTRAQ4plex)	1	1
[R].VLFYVDSEk.[L]	K9(iTRAQ4plex)	1	1
[K].qNDFNSVEEk.[K]	N-Term(iTRAQ4plex); K10(iTRAQ	1	1
[K].eVPcASVk.[K]	N-Term(iTRAQ4plex); C4(Carbam	1	1
[R].iLPLTVck.[M]	N-Term(iTRAQ4plex); C7(Carbam	1	1
[K].fSSHGck.[E]	N-Term(iTRAQ4plex); C6(Carbam	1	1
[K].acGAcPLWGk.[C]	N-Term(iTRAQ4plex); C2(Carbam	1	1
[K].eQTmSEcEAGALR.[C]	N-Term(iTRAQ4plex); M4(Oxidat	1	1
[K].sSGWHFVVk.[F]	N-Term(iTRAQ4plex); K9(iTRAQ4	1	1
[K].eQTmSEcEAGALR.[C]	N-Term(iTRAQ4plex); M4(Oxidat	1	1
[K].sSGWHFVVk.[F]	N-Term(iTRAQ4plex); K9(iTRAQ4	1	1
[K].eLENALk.[AN]	N-Term(iTRAQ4plex); K7(iTRAQ4	1	2
[K].sSGWHFVVk.[F]	N-Term(iTRAQ4plex); K9(iTRAQ4	1	1
[R].dScTLPASAEk.[A]	N-Term(iTRAQ4plex); C3(Carbam	1	1
[K].eQTMSEcEAGALR.[C]	N-Term(iTRAQ4plex); C7(Carbam	1	1
[K].lQPLDFkENAEQSR.[A]	N-Term(iTRAQ4plex); K7(iTRAQ4	1	1
[K].LQPLDFKENAEQSR.[A]		1	1
[K].skLPGIVAEGR.[D]	N-Term(iTRAQ4plex); K2(iTRAQ4	1	1
[K].eQLQDMGLVDLFSPEk.[S]	N-Term(iTRAQ4plex); K16(iTRAQ	1	1
[K].eQLQDMGLVDLFSPEk.[S]	N-Term(iTRAQ4plex); K16(iTRAQ	1	1
[K].EQLQDMGLVDLFSPEk.[S]	K16(iTRAQ4plex)	1	1
[K].EQLQDMGLVDLFSPEk.[S]	K16(iTRAQ4plex)	1	1
[K].aTEDEGSEQk.[I]	N-Term(iTRAQ4plex); K10(iTRAQ	1	1
[K].kATEDEGSEQk.[I]	N-Term(iTRAQ4plex); K1(iTRAQ4	1	1
[K].eQLQDMGLVDLFSPEk.[S]	N-Term(iTRAQ4plex); K16(iTRAQ	1	1
[K].aNRPFLVFIR.[E]	N-Term(iTRAQ4plex)	1	1
[K].aNRPFLVFIR.[E]	N-Term(iTRAQ4plex)	1	1
[R].fATTFYQHLADSk.[N]	N-Term(iTRAQ4plex); K13(iTRAQ	1	1
[K].skLPGIVAEGR.[D]	N-Term(iTRAQ4plex); K2(iTRAQ4	1	1
[K].skLPGIVAEGR.[D]	N-Term(iTRAQ4plex); K2(iTRAQ4	1	1
[K].aTEDEGSEQk.[I]	N-Term(iTRAQ4plex); K10(iTRAQ	1	1
[K].sKLPGIVAEGR.[D]	N-Term(iTRAQ4plex)	1	1

[R].FATTFYQHLADSk.[N]	K13(iTRAQ4plex)	1	1
[R].FATTFYQHLADSk.[N]	K13(iTRAQ4plex)	1	1
[K].kATEDEGSEQk.[I]	N-Term(iTRAQ4plex); K1(iTRAQ4	1	1
[R].dIPMNPMcIYR.[S]	N-Term(iTRAQ4plex); C8(Carbam	1	1
[R].fATTFYQHLADSk.[N]	N-Term(iTRAQ4plex); K13(iTRAQ	1	1
[R].fATTFYQHLADSk.[N]	N-Term(iTRAQ4plex); K13(iTRAQ	1	1
[R].fATTFYQHLADSk.[N]	N-Term(iTRAQ4plex); K13(iTRAQ	1	1
[R].fATTFYQHLADSk.[N]	N-Term(iTRAQ4plex); K13(iTRAQ	1	1
[K].TSDQIHFFFAk.[L]	K11(iTRAQ4plex)	1	1
[K].tSDQIHFFFAk.[L]	N-Term(iTRAQ4plex); K11(iTRAQ	1	1
[K].tSDQIHFFFAk.[L]	N-Term(iTRAQ4plex); K11(iTRAQ	1	1
[K].tSDQIHFFFAk.[L]	N-Term(iTRAQ4plex); K11(iTRAQ	1	1
[K].tSDQIHFFFAk.[L]	N-Term(iTRAQ4plex); K11(iTRAQ	1	1
[K].tSDQIHFFFAk.[L]	N-Term(iTRAQ4plex); K11(iTRAQ	1	1
[K].aTEDEGSEQk.[I]	N-Term(iTRAQ4plex); K10(iTRAQ	1	1
[R].rVAEGTQVLELPFk.[G]	N-Term(iTRAQ4plex); K14(iTRAQ	1	1
[K].lPGIVAEGR.[D]	N-Term(iTRAQ4plex)	1	1
[K].eQLQDmGLVDLFSPEk.[S]	N-Term(iTRAQ4plex); M6(Oxidat	1	1
[K].eQLQDMGLVDLFSPEk.[S]	N-Term(iTRAQ4plex); K16(iTRAQ	1	1
[K].eQLQDMGLVDLFSPEk.[S]	N-Term(iTRAQ4plex); K16(iTRAQ	1	1
[R].vAEGTQVLELPFk.[G]	N-Term(iTRAQ4plex); K13(iTRAQ	1	1
[R].vAEGTQVLELPFk.[G]	N-Term(iTRAQ4plex); K13(iTRAQ	1	1
[K].gDDITMVLILPkPEk.[S]	N-Term(iTRAQ4plex); K12(iTRAQ	1	1
[K].gDDITMVLILPkPEk.[S]	N-Term(iTRAQ4plex); K12(iTRAQ	1	1
[K].gDDITMVLILPkPEk.[S]	N-Term(iTRAQ4plex); K12(iTRAQ	1	1
[K].gDDITMVLILPkPEk.[S]	N-Term(iTRAQ4plex); K12(iTRAQ	1	1
[R].dDLYVSDAFHk.[A]	N-Term(iTRAQ4plex); K11(iTRAQ	1	1
[R].dDLYVSDAFHk.[A]	N-Term(iTRAQ4plex); K11(iTRAQ	1	1
[R].dDLYVSDAFHk.[A]	N-Term(iTRAQ4plex); K11(iTRAQ	1	1
[K].aDGEScSASMmYQEGk.[F]	N-Term(iTRAQ4plex); C6(Carbam	1	1
[R].rVAEGTQVLELPFkGDDITMVLILPkPEk.[S	N-Term(iTRAQ4plex); K14(iTRAQ	1	1
[K].aDGEScSASmMYQEGk.[F]	N-Term(iTRAQ4plex); C6(Carbam	1	1
[K].aTEDEGSEQk.[I]	N-Term(iTRAQ4plex); K10(iTRAQ	1	1
[R].vAEGTQVLELPFkGDDITMVLILPkPEk.[S]	N-Term(iTRAQ4plex); K13(iTRAQ	1	1
[K].aTEDEGSEQk.[I]	N-Term(iTRAQ4plex); K10(iTRAQ	1	1
[R].eVPLNTIIFMGR.[V]	N-Term(iTRAQ4plex)	1	1
[R].eVPLNTIIFMGR.[V]	N-Term(iTRAQ4plex)	1	1
[K].aDGEScSASMMYQEGk.[F]	N-Term(iTRAQ4plex); C6(Carbam	1	1
[R].dDLYVSDAFHk.[A]	N-Term(iTRAQ4plex); K11(iTRAQ	1	1
[K].kATEDEGSEQk.[I]	N-Term(iTRAQ4plex); K1(iTRAQ4	1	1
[K].aNRPFLVFIR.[E]	N-Term(iTRAQ4plex)	1	1
[K].aFLEVNEEGSEAAASTAVVIAGR.[S]	N-Term(iTRAQ4plex)	1	1
[K].aTEDEGSEQk.[I]	N-Term(iTRAQ4plex); K10(iTRAQ	1	1
[K].kATEDEGSEQk.[I]	N-Term(iTRAQ4plex); K1(iTRAQ4	1	1
[K].aTEDEGSEQkIPEATNR.[R]	N-Term(iTRAQ4plex); K10(iTRAQ	1	1
[K].SkLPGIVAEGR.[D]	K2(iTRAQ4plex)	1	1
[K].aTEDEGSEQk.[I]	N-Term(iTRAQ4plex); K10(iTRAQ	1	1
[R].iEDGFSLk.[E]	N-Term(iTRAQ4plex); K8(iTRAQ4	1	1
[K].aDGEScSASmMYQEGk.[F]	N-Term(iTRAQ4plex); C6(Carbam	1	1
[K].aDGEScSASMmYQEGk.[F]	N-Term(iTRAQ4plex); C6(Carbam	1	1
[K].TSDQIHFFFAk.[L]	K11(iTRAQ4plex)	1	1
[K].aTEDEGSEQk.[I]	N-Term(iTRAQ4plex); K10(iTRAQ	1	1

[K].gDDITMVLILPkPEk.[S]	N-Term(iTRAQ4plex); K12(iTRAQ4	1	1
[K].aTEDEGSEQk.[I]	N-Term(iTRAQ4plex); K10(iTRAQ4	1	1
[K].TSDQIHFFFAk.[L]	K11(iTRAQ4plex)	1	1
[K].tSDQIHFFFAk.[L]	N-Term(iTRAQ4plex); K11(iTRAQ4	1	1
[R].fATTFYQHLADSk.[N]	N-Term(iTRAQ4plex); K13(iTRAQ4	1	1
[K].aTEDEGSEQk.[I]	N-Term(iTRAQ4plex); K10(iTRAQ4	1	1
[K].tSDQIHFFFAk.[L]	N-Term(iTRAQ4plex); K11(iTRAQ4	1	1
[R].iEDGFSLk.[E]	N-Term(iTRAQ4plex); K8(iTRAQ4	1	1
[K].tSDQIHFFFAk.[L]	N-Term(iTRAQ4plex); K11(iTRAQ4	1	1
[K].skLPGIVAEGR.[D]	N-Term(iTRAQ4plex); K2(iTRAQ4	1	1
[R].VAEGTQVLELPFk.[G]	K13(iTRAQ4plex)	1	1
[R].rVWELSk.[A]	N-Term(iTRAQ4plex); K7(iTRAQ4	1	1
[K].tSDQIHFFFAk.[L]	N-Term(iTRAQ4plex); K11(iTRAQ4	1	1
[R].IEDGFSLk.[E]	K8(iTRAQ4plex)	1	1
[K].fDTISEk.[T]	N-Term(iTRAQ4plex); K7(iTRAQ4	1	1
[K].tSDQIHFFFAk.[L]	N-Term(iTRAQ4plex); K11(iTRAQ4	1	1
[K].lPGIVAEGR.[D]	N-Term(iTRAQ4plex)	1	1
[K].eQLQDMGLVDLFSPEk.[S]	N-Term(iTRAQ4plex); K16(iTRAQ4	1	1
[R].fATTFYQHLADSk.[N]	N-Term(iTRAQ4plex); K13(iTRAQ4	1	1
[R].vAEGTQVLELPFk.[G]	N-Term(iTRAQ4plex); K13(iTRAQ4	1	1
[R].rVWELSkANSR.[F]	N-Term(iTRAQ4plex); K7(iTRAQ4	1	1
[K].fDTISEk.[T]	N-Term(iTRAQ4plex); K7(iTRAQ4	1	1
[K].kATEDEGSEQk.[I]	N-Term(iTRAQ4plex); K1(iTRAQ4	1	1
[K].aDGEScSASMMYQEGkFR.[Y]	N-Term(iTRAQ4plex); C6(Carbam	1	1
[R].fATTFYQHLADSk.[N]	N-Term(iTRAQ4plex); K13(iTRAQ4	1	1
[K].aDGEScSASMmYQEGk.[F]	N-Term(iTRAQ4plex); C6(Carbam	1	1
[K].aDGEScSASmMYQEGk.[F]	N-Term(iTRAQ4plex); C6(Carbam	1	1
[R].rVAEGTQVLELPFk.[G]	N-Term(iTRAQ4plex); K14(iTRAQ4	1	1
[K].eQLQDmGLVDLFSPEk.[S]	N-Term(iTRAQ4plex); M6(Oxidat	1	1
[R].EVPLNTIIFMGR.[V]		1	1
[K].sSkLVSANR.[L]	N-Term(iTRAQ4plex); K3(iTRAQ4	1	1
[R].dDLYVSDAFHk.[A]	N-Term(iTRAQ4plex); K11(iTRAQ4	1	1
[R].fATTFYQHLADSk.[N]	N-Term(iTRAQ4plex); K13(iTRAQ4	1	1
[K].fDTISEk.[T]	N-Term(iTRAQ4plex); K7(iTRAQ4	1	1
[K].aTEDEGSEQk.[I]	N-Term(iTRAQ4plex); K10(iTRAQ4	1	1
[K].aTEDEGSEQk.[I]	N-Term(iTRAQ4plex); K10(iTRAQ4	1	1
[K].aDGEScSASMMYQEGk.[F]	N-Term(iTRAQ4plex); C6(Carbam	1	1
[K].eQLQDmGLVDLFSPEk.[S]	N-Term(iTRAQ4plex); M6(Oxidat	1	1
[R].dIPMNPMcIYR.[S]	N-Term(iTRAQ4plex); C8(Carbam	1	1
[K].fDTISEk.[T]	N-Term(iTRAQ4plex); K7(iTRAQ4	1	1
[K].lQPLDFk.[E]	N-Term(iTRAQ4plex); K7(iTRAQ4	1	1
[R].rVWELSk.[A]	N-Term(iTRAQ4plex); K7(iTRAQ4	1	1
[K].sSkLVSANR.[L]	N-Term(iTRAQ4plex); K3(iTRAQ4	1	1
[K].tSDQIHFFFAk.[L]	N-Term(iTRAQ4plex); K11(iTRAQ4	1	1
[R].dDLYVSDAFHk.[A]	N-Term(iTRAQ4plex); K11(iTRAQ4	1	1
[R].rVWELSk.[A]	N-Term(iTRAQ4plex); K7(iTRAQ4	1	1
[K].aNRPFLVFIR.[E]	N-Term(iTRAQ4plex)	1	1
[K].lQPLDFk.[E]	N-Term(iTRAQ4plex); K7(iTRAQ4	1	1
[K].tSDQIHFFFAk.[L]	N-Term(iTRAQ4plex); K11(iTRAQ4	1	1
[R].dIPMNPMcIYR.[S]	N-Term(iTRAQ4plex); C8(Carbam	1	1
[K].lQPLDFk.[E]	N-Term(iTRAQ4plex); K7(iTRAQ4	1	1
[K].aDGEScSASMMYQEGkFR.[Y]	N-Term(iTRAQ4plex); C6(Carbam	1	1

[K].TLNIcEVGTIR.[C]	C5(Carbamidomethyl)	1	1
[R].vPANLENVGFEVQTAEDDLk.[T]	N-Term(iTRAQ4plex); K20(iTRAQ	1	1
[R].vPANLENVGFEVQTAEDDLk.[T]	N-Term(iTRAQ4plex); K20(iTRAQ	1	1
[R].vPANLENVGFEVQTAEDDLk.[T]	N-Term(iTRAQ4plex); K20(iTRAQ	1	1
[K].cLNNQQLHFLHIGScQDGR.[Q]	N-Term(iTRAQ4plex); C1(Carbam	1	1
[K].aLNHLPLEYNSALYSR.[I]	N-Term(iTRAQ4plex)	1	1
[K].cVcLLPPQcFk.[G]	N-Term(iTRAQ4plex); C1(Carbam	1	1
[K].aLNHLPLEYNSALYSR.[I]	N-Term(iTRAQ4plex)	1	1
[K].aLNHLPLEYNSALYSR.[I]	N-Term(iTRAQ4plex)	1	1
[K].cVcLLPPQcFk.[G]	N-Term(iTRAQ4plex); C1(Carbam	1	1
[K].tFSEWLESVk.[E]	N-Term(iTRAQ4plex); K10(iTRAQ	1	1
[K].tFSEWLESVk.[E]	N-Term(iTRAQ4plex); K10(iTRAQ	1	1
[K].eNPAVIDFELAPIVDLVR.[N]	N-Term(iTRAQ4plex)	1	1
[K].eNPAVIDFELAPIVDLVR.[N]	N-Term(iTRAQ4plex)	1	1
[R].cPINcLLGDFGPWSDcDPcIEk.[Q]	N-Term(iTRAQ4plex); C1(Carbam	1	1
[K].rSENINHNSAFk.[Q]	N-Term(iTRAQ4plex); K12(iTRAQ	1	1
[K].aLQEYAAk.[F]	N-Term(iTRAQ4plex); K8(iTRAQ4	1	1
[K].qAIQASHk.[K]	N-Term(iTRAQ4plex); K8(iTRAQ4	1	1
[K].rSENINHNSAFk.[Q]	N-Term(iTRAQ4plex); K12(iTRAQ	1	1
[K].rSENINHNSAFk.[Q]	N-Term(iTRAQ4plex); K12(iTRAQ	1	1
[K].yYQENFcEQIcSk.[Q]	N-Term(iTRAQ4plex); C7(Carbam	1	1
[R].iGESIELTcPk.[G]	N-Term(iTRAQ4plex); C9(Carbam	1	1
[K].qAIQASHk.[K]	N-Term(iTRAQ4plex); K8(iTRAQ4	1	1
[K].qAIQASHk.[K]	N-Term(iTRAQ4plex); K8(iTRAQ4	1	1
[R].gEVLDNSFTGGIck.[T]	N-Term(iTRAQ4plex); C13(Carbar	1	1
[R].gEVLDNSFTGGIck.[T]	N-Term(iTRAQ4plex); C13(Carbar	1	1
[K].gSSGLEEk.[T]	N-Term(iTRAQ4plex); K8(iTRAQ4	1	1
[R].sENINHNSAFk.[Q]	N-Term(iTRAQ4plex); K11(iTRAQ	1	1
[R].kmEILHPGk.[C]	N-Term(iTRAQ4plex); K1(iTRAQ4	1	1
[R].kmEILHPGk.[C]	N-Term(iTRAQ4plex); K1(iTRAQ4	1	1
[K].qAIQASHk.[K]	N-Term(iTRAQ4plex); K8(iTRAQ4	1	1
[K].aLNHLPLEYNSALYSR.[I]	N-Term(iTRAQ4plex)	1	1
[R].kMEILHPGk.[C]	N-Term(iTRAQ4plex); K1(iTRAQ4	1	1
[K].iEEADck.[N]	N-Term(iTRAQ4plex); C6(Carbam	1	1
[K].kEScGYDTcYDWEk.[C]	N-Term(iTRAQ4plex); K1(iTRAQ4	1	1
[K].cLNNQQLHFLHIGScQDGR.[Q]	N-Term(iTRAQ4plex); C1(Carbam	1	1
[K].tLNIcEVGTIR.[C]	N-Term(iTRAQ4plex); C5(Carbam	1	1
[K].eScGYDTcYDWEk.[C]	N-Term(iTRAQ4plex); C3(Carbam	1	1
[K].tLNIcEVGTIR.[C]	N-Term(iTRAQ4plex); C5(Carbam	1	1
[K].gFVVAGPSR.[Y]	N-Term(iTRAQ4plex)	1	1
[K].iEEADck.[N]	N-Term(iTRAQ4plex); C6(Carbam	1	1
[K].lkGHcQLGQk.[Q]	N-Term(iTRAQ4plex); K2(iTRAQ4	1	1
[K].mEILHPGk.[C]	N-Term(iTRAQ4plex); M1(Oxidat	1	1
[K].ALNHLPLEYNSALYSR.[I]		1	1
[K].dLHLSDVFLk.[A]	N-Term(iTRAQ4plex); K10(iTRAQ	1	1
[R].NIPcAVTk.[R]	C4(Carbamidomethyl); K8(iTRAC	1	1
[R].kMEILHPGk.[C]	N-Term(iTRAQ4plex); K1(iTRAQ4	1	1
[R].sEYGAALAWEk.[G]	N-Term(iTRAQ4plex); K11(iTRAQ	1	1
[K].lkGHcQLGQk.[Q]	N-Term(iTRAQ4plex); K2(iTRAQ4	1	1
[K].aLQEYAAk.[F]	N-Term(iTRAQ4plex); K8(iTRAQ4	1	1
[R].iGESIELTcPk.[G]	N-Term(iTRAQ4plex); C9(Carbam	1	1
[R].nIPcAVTk.[R]	N-Term(iTRAQ4plex); C4(Carbam	1	1

[K].gHcQLGQk.[Q]	N-Term(iTRAQ4plex); C3(Carbam	1	1
[K].tFSEWLESVk.[E]	N-Term(iTRAQ4plex); K10(iTRAQ	1	1
[K].gHcQLGQk.[Q]	N-Term(iTRAQ4plex); C3(Carbam	1	1
[K].gHcQLGQk.[Q]	N-Term(iTRAQ4plex); C3(Carbam	1	1
[K].tFSEWLESVk.[E]	N-Term(iTRAQ4plex); K10(iTRAQ	1	1
[R].sEYGAALAWEk.[G]	N-Term(iTRAQ4plex); K11(iTRAQ	1	1
[K].gFVVAGPSR.[Y]	N-Term(iTRAQ4plex)	1	1
[R].rPcFESLk.[A]	N-Term(iTRAQ4plex); C3(Carbam	1	1
[K].vMNHIcSk.[Q]	N-Term(iTRAQ4plex); C6(Carbam	1	1
[R].TINPAVDHcck.[T]	C9(Carbamidomethyl); C10(Carb	1	1
[K].eLISLVEDVSSNYDGccEGDVVQcIR.[D]	N-Term(iTRAQ4plex); C16(Carba	1	1
[K].lPNNVLQEk.[I]	N-Term(iTRAQ4plex); K9(iTRAQ4	1	1
[K].vMNHIcSk.[Q]	N-Term(iTRAQ4plex); C6(Carbam	1	1
[R].rHPDLSIPELLR.[I]	N-Term(iTRAQ4plex)	1	1
[K].eLISLVEDVSSNYDGccEGDVVQcIR.[D]	N-Term(iTRAQ4plex); C16(Carba	1	1
[K].eLISLVEDVSSNYDGccEGDVVQcIR.[D]	N-Term(iTRAQ4plex); C16(Carba	1	1
[R].rPcFESLk.[A]	N-Term(iTRAQ4plex); C3(Carbam	1	1
[R].rPcFESLk.[A]	N-Term(iTRAQ4plex); C3(Carbam	1	1
[K].HELTDEELQSLFTNFANVVDk.[C]	K21(iTRAQ4plex)	1	1
[K].SDVGFLPPFPTLDPEEk.[C]	K17(iTRAQ4plex)	1	1
[K].sDVGFLPPFPTLDPEEk.[C]	N-Term(iTRAQ4plex); K17(iTRAQ	1	1
[R].TINPAVDHcck.[T]	C9(Carbamidomethyl); C10(Carb	1	1
[R].hPDLSIPELLR.[I]	N-Term(iTRAQ4plex)	1	1
[R].rHPDLSIPELLR.[I]	N-Term(iTRAQ4plex)	1	1
[R].rHPDLSIPELLR.[I]	N-Term(iTRAQ4plex)	1	1
[R].rHPDLSIPELLR.[I]	N-Term(iTRAQ4plex)	1	1
[R].rHPDLSIPELLR.[I]	N-Term(iTRAQ4plex)	1	1
[R].rHPDLSIPELLR.[I]	N-Term(iTRAQ4plex)	1	1
[R].aIPVTQYLk.[A]	N-Term(iTRAQ4plex); K9(iTRAQ4	1	1
[R].aIPVTQYLk.[A]	N-Term(iTRAQ4plex); K9(iTRAQ4	1	1
[K].sDVGFLPPFPTLDPEEk.[C]	N-Term(iTRAQ4plex); K17(iTRAQ	1	1
[R].rHPDLSIPELLR.[I]	N-Term(iTRAQ4plex)	1	1
[R].rHPDLSIPELLR.[I]	N-Term(iTRAQ4plex)	1	1
[R].rHPDLSIPELLR.[I]	N-Term(iTRAQ4plex)	1	1
[R].rHPDLSIPELLR.[I]	N-Term(iTRAQ4plex)	1	1
[R].RHPDLSIPELLR.[I]		1	1
[K].hELTDEELQSLFTNFANVVDk.[C]	N-Term(iTRAQ4plex); K21(iTRAQ	1	1
[K].hELTDEELQSLFTNFANVVDk.[C]	N-Term(iTRAQ4plex); K21(iTRAQ	1	1
[K].icAMEGLPQk.[H]	N-Term(iTRAQ4plex); C2(Carbam	1	1
[K].TYVPPPFSQDLFTFHADMcQSQNEELQR.[	C19(Carbamidomethyl)	1	1
[K].tYVPPPFSQDLFTFHADMcQSQNEELQR.[	N-Term(iTRAQ4plex); C19(Carba	1	1
[K].kSDVGFLPPFPTLDPEEk.[C]	N-Term(iTRAQ4plex); K1(iTRAQ4	1	1
[K].kSDVGFLPPFPTLDPEEk.[C]	N-Term(iTRAQ4plex); K1(iTRAQ4	1	1
[K].LkHELTDEELQSLFTNFANVVDk.[C]	K2(iTRAQ4plex); K23(iTRAQ4ple	1	1
[K].lKHELTDEELQSLFTNFANVVDk.[C]	N-Term(iTRAQ4plex); K23(iTRAQ	1	1
[K].iAPQLSTEELVSLGEk.[M]	N-Term(iTRAQ4plex); K16(iTRAQ	1	1
[K].iAPQLSTEELVSLGEk.[M]	N-Term(iTRAQ4plex); K16(iTRAQ	1	1
[K].iAPQLSTEELVSLGEk.[M]	N-Term(iTRAQ4plex); K16(iTRAQ	1	1
[K].iAPQLSTEELVSLGEk.[M]	N-Term(iTRAQ4plex); K16(iTRAQ	1	1
[K].lkHELTDEELQSLFTNFANVVDk.[C]	N-Term(iTRAQ4plex); K2(iTRAQ4	1	1
[K].lkHELTDEELQSLFTNFANVVDk.[C]	N-Term(iTRAQ4plex); K2(iTRAQ4	1	1
[R].dADPDTFFAk.[F]	N-Term(iTRAQ4plex); K10(iTRAQ	1	1

[R].dADPDTFFAk.[F]	N-Term(iTRAQ4plex); K10(iTRAQ4	1	1
[K].fTDSENVcQER.[D]	N-Term(iTRAQ4plex); C8(Carbam	1	1
[K].fTDSENVcQER.[D]	N-Term(iTRAQ4plex); C8(Carbam	1	1
[R].TINPAVDHcck.[T]	C9(Carbamidomethyl); C10(Carb	1	1
[R].TINPAVDHcck.[T]	C9(Carbamidomethyl); C10(Carb	1	1
[R].tINPAVDHcck.[T]	N-Term(iTRAQ4plex); C9(Carbam	1	1
[R].tINPAVDHcck.[T]	N-Term(iTRAQ4plex); C9(Carbam	1	1
[K].vVHFIYIAILSQk.[F]	N-Term(iTRAQ4plex); K13(iTRAQ4	1	1
[R].eSLLNHFLYEVAR.[R]	N-Term(iTRAQ4plex)	1	1
[K].aESPEVcFNEESPk.[I]	N-Term(iTRAQ4plex); C7(Carbam	1	1
[R].eSLLNHFLYEVAR.[R]	N-Term(iTRAQ4plex)	1	1
[K].aESPEVcFNEESPk.[I]	N-Term(iTRAQ4plex); C7(Carbam	1	1
[R].eSLLNHFLYEVAR.[R]	N-Term(iTRAQ4plex)	1	1
[K].FTDSENVcQER.[D]	C8(Carbamidomethyl)	1	1
[K].FTDSENVcQER.[D]	C8(Carbamidomethyl)	1	1
[R].lcFFYNk.[K]	N-Term(iTRAQ4plex); C2(Carbam	1	1
[R].gQcIINSNk.[D]	N-Term(iTRAQ4plex); C3(Carbam	1	1
[K].FTDSENVcQER.[D]	C8(Carbamidomethyl)	1	1
[R].rHPDLSIPELLR.[I]	N-Term(iTRAQ4plex)	1	1
[R].tINPAVDHcck.[T]	N-Term(iTRAQ4plex); C9(Carbam	1	1
[R].TINPAVDHcck.[T]	C9(Carbamidomethyl); C10(Carb	1	1
[K].icAMEGLPQk.[H]	N-Term(iTRAQ4plex); C2(Carbam	1	1
[R].tINPAVDHcck.[T]	N-Term(iTRAQ4plex); C9(Carbam	1	1
[R].rLcFFYNk.[K]	N-Term(iTRAQ4plex); C3(Carbam	1	1
[K].qDSISSk.[I]	N-Term(iTRAQ4plex); K7(iTRAQ4	1	1
[R].lcFFYNk.[K]	N-Term(iTRAQ4plex); C2(Carbam	1	1
[K].hVcGALLk.[F]	N-Term(iTRAQ4plex); C3(Carbam	1	1
[R].fLVNLVk.[L]	N-Term(iTRAQ4plex); K7(iTRAQ4	1	1
[K].vMNHIcSk.[Q]	N-Term(iTRAQ4plex); C6(Carbam	1	1
[R].fLVNLVk.[L]	N-Term(iTRAQ4plex); K7(iTRAQ4	1	1
[K].vmNHIcSk.[Q]	N-Term(iTRAQ4plex); M2(Oxidat	1	1
[R].rPcFESLk.[A]	N-Term(iTRAQ4plex); C3(Carbam	1	1
[K].aFSSYQk.[H]	N-Term(iTRAQ4plex); K7(iTRAQ4	1	1
[R].gQcIINSNk.[D]	N-Term(iTRAQ4plex); C3(Carbam	1	1
[K].vMNHIcSk.[Q]	N-Term(iTRAQ4plex); C6(Carbam	1	1
[R].RHPDLSIPELLR.[I]		1	1
[R].dADPDTFFAk.[F]	N-Term(iTRAQ4plex); K10(iTRAQ4	1	1
[K].hFQNLGk.[D]	N-Term(iTRAQ4plex); K7(iTRAQ4	1	1
[K].hFQNLGk.[D]	N-Term(iTRAQ4plex); K7(iTRAQ4	1	1
[K].hVcGALLk.[F]	N-Term(iTRAQ4plex); C3(Carbam	1	1
[K].hVcGALLk.[F]	N-Term(iTRAQ4plex); C3(Carbam	1	1
[K].vMNHIcSk.[Q]	N-Term(iTRAQ4plex); C6(Carbam	1	1
[K].qDSISSk.[I]	N-Term(iTRAQ4plex); K7(iTRAQ4	1	1
[K].lPNNVLQEk.[I]	N-Term(iTRAQ4plex); K9(iTRAQ4	1	1
[R].aIPVTQYLk.[A]	N-Term(iTRAQ4plex); K9(iTRAQ4	1	1
[K].lPNNVLQEk.[I]	N-Term(iTRAQ4plex); K9(iTRAQ4	1	1
[K].vmNHIcSk.[Q]	N-Term(iTRAQ4plex); M2(Oxidat	1	1
[R].DADPDTFFAk.[F]	K10(iTRAQ4plex)	1	1
[K].hVcGALLk.[F]	N-Term(iTRAQ4plex); C3(Carbam	1	1
[K].hFQNLGk.[D]	N-Term(iTRAQ4plex); K7(iTRAQ4	1	1
[R].tINPAVDHcck.[T]	N-Term(iTRAQ4plex); C9(Carbam	1	1
[K].hFQNLGk.[D]	N-Term(iTRAQ4plex); K7(iTRAQ4	1	1

[R].dADPDTFFAk.[F]	N-Term(iTRAQ4plex); K10(iTRAQ4	1	1
[K].mVQQEck.[H]	N-Term(iTRAQ4plex); C6(Carbam	1	1
[K].hFQNLGk.[D]	N-Term(iTRAQ4plex); K7(iTRAQ4	1	1
[R].hPDLSIPELLR.[I]	N-Term(iTRAQ4plex)	1	1
[K].ELISLVEDVSSNYDGccEGDVVQcIR.[D]	C16(Carbamidomethyl); C17(Car	1	1
[K].HFQNLGk.[D]	K7(iTRAQ4plex)	1	1
[K].aESPEVcFNEESPk.[I]	N-Term(iTRAQ4plex); C7(Carbam	1	1
[R].rPcFESLk.[A]	N-Term(iTRAQ4plex); C3(Carbam	1	1
[K].qDSISSk.[I]	N-Term(iTRAQ4plex); K7(iTRAQ4	1	1
[K].VMNHIcSk.[Q]	C6(Carbamidomethyl); K8(iTRAC	1	1
[K].qDSISSk.[I]	N-Term(iTRAQ4plex); K7(iTRAQ4	1	1
[R].GQcIINSNk.[D]	C3(Carbamidomethyl); K9(iTRAC	1	1
[R].aIPVTQYLk.[A]	N-Term(iTRAQ4plex); K9(iTRAQ4	1	1
[K].tLPEcSk.[L]	N-Term(iTRAQ4plex); C5(Carbam	1	1
[K].vmNHIcSk.[Q]	N-Term(iTRAQ4plex); M2(Oxidat	1	1
[K].qDSISSk.[I]	N-Term(iTRAQ4plex); K7(iTRAQ4	1	1
[K].HFQNLGk.[D]	K7(iTRAQ4plex)	1	1
[R].DADPDTFFAk.[F]	K10(iTRAQ4plex)	1	1
[R].TINPAVDHcck.[T]	C9(Carbamidomethyl); C10(Carb	1	1
[R].lcFFYNk.[K]	N-Term(iTRAQ4plex); C2(Carbam	1	1
[K].tLPEcSk.[L]	N-Term(iTRAQ4plex); C5(Carbam	1	1
[K].vmNHIcSk.[Q]	N-Term(iTRAQ4plex); M2(Oxidat	1	1
[R].lcFFYNk.[K]	N-Term(iTRAQ4plex); C2(Carbam	1	1
[K].mVQQEck.[H]	N-Term(iTRAQ4plex); C6(Carbam	1	1
[K].aESPEVcFNEESPk.[I]	N-Term(iTRAQ4plex); C7(Carbam	1	1
[R].rHPDLSIPELLR.[I]	N-Term(iTRAQ4plex)	1	1
[K].hFQNLGk.[D]	N-Term(iTRAQ4plex); K7(iTRAQ4	1	1
[K].HFQNLGk.[D]	K7(iTRAQ4plex)	1	1
[R].dADPDTFFAk.[F]	N-Term(iTRAQ4plex); K10(iTRAQ4	1	1
[R].RHPDLSIPELLR.[I]		1	1
[R].rLcFFYNk.[K]	N-Term(iTRAQ4plex); C3(Carbam	1	1
[R].rHPDLSIPELLR.[I]	N-Term(iTRAQ4plex)	1	1
[K].tVGSDTFYSFk.[Y]	N-Term(iTRAQ4plex); K11(iTRAQ4	1	1
[K].tVGSDTFYSFk.[Y]	N-Term(iTRAQ4plex); K11(iTRAQ4	1	1
[K].tVGSDTFYSFk.[Y]	N-Term(iTRAQ4plex); K11(iTRAQ4	1	1
[K].TVGSDTFYSFk.[Y]	K11(iTRAQ4plex)	1	1
[K].TVGSDTFYSFk.[Y]	K11(iTRAQ4plex)	1	1
[K].rPPGFSPFR.[S]	N-Term(iTRAQ4plex)	1	1
[K].aVDAALk.[K]	N-Term(iTRAQ4plex); K7(iTRAQ4	1	1
[K].kYNSQNQSNNQFVLYR.[I]	N-Term(iTRAQ4plex); K1(iTRAQ4	1	1
[K].rPPGFSPFR.[S]	N-Term(iTRAQ4plex)	1	1
[K].rPPGFSPFR.[S]	N-Term(iTRAQ4plex)	1	1
[K].ENFLFLTPDck.[S]	C10(Carbamidomethyl); K11(iTR	1	1
[K].ENFLFLTPDck.[S]	C10(Carbamidomethyl); K11(iTR	1	1
[R].kHNLGHGHk.[H]	N-Term(iTRAQ4plex); K1(iTRAQ4	1	1
[K].yNSQNQSNNQFVLYR.[I]	N-Term(iTRAQ4plex)	1	1
[R].dIPTNSPELEETLTHTITk.[L]	N-Term(iTRAQ4plex); K19(iTRAQ4	1	1
[R].dIPTNSPELEETLTHTITk.[L]	N-Term(iTRAQ4plex); K19(iTRAQ4	1	1
[R].dIPTNSPELEETLTHTITk.[L]	N-Term(iTRAQ4plex); K19(iTRAQ4	1	1
[R].dIPTNSPELEETLTHTITk.[L]	N-Term(iTRAQ4plex); K19(iTRAQ4	1	1
[R].dIPTNSPELEETLTHTITk.[L]	N-Term(iTRAQ4plex); K19(iTRAQ4	1	1
[R].dIPTNSPELEETLTHTITk.[L]	N-Term(iTRAQ4plex); K19(iTRAQ4	1	1

[R].dIPTNSPELEETLTHTITk.[L]	N-Term(iTRAQ4plex); K19(iTRAQ4	1	1
[R].DIPTNSPELEETLTHTITk.[L]	K19(iTRAQ4plex)	1	1
[K].kYFIDFVAR.[E]	N-Term(iTRAQ4plex); K1(iTRAQ4	1	1
[K].kYFIDFVAR.[E]	N-Term(iTRAQ4plex); K1(iTRAQ4	1	1
[K].kYNSQNQSNNQFVLYR.[I]	N-Term(iTRAQ4plex); K1(iTRAQ4	1	1
[K].kYNSQNQSNNQFVLYR.[I]	N-Term(iTRAQ4plex); K1(iTRAQ4	1	1
[K].yNSQNQSNNQFVLYR.[I]	N-Term(iTRAQ4plex)	1	1
[K].yNSQNQSNNQFVLYR.[I]	N-Term(iTRAQ4plex)	1	1
[K].aVDAALk.[K]	N-Term(iTRAQ4plex); K7(iTRAQ4	1	1
[R].vQVVAGk.[K]	N-Term(iTRAQ4plex); K7(iTRAQ4	1	1
[R].qVVAGLNFR.[I]	N-Term(iTRAQ4plex)	1	1
[R].qVVAGLNFR.[I]	N-Term(iTRAQ4plex)	1	1
[K].rPPGFSPFR.[S]	N-Term(iTRAQ4plex)	1	1
[K].rPPGFSPFR.[S]	N-Term(iTRAQ4plex)	1	1
[K].rPPGFSPFR.[S]	N-Term(iTRAQ4plex)	1	1
[K].rPPGFSPFR.[S]	N-Term(iTRAQ4plex)	1	1
[R].iASFSQNcDIYPGk.[D]	N-Term(iTRAQ4plex); C8(Carbam	1	1
[R].iASFSQNcDIYPGk.[D]	N-Term(iTRAQ4plex); C8(Carbam	1	1
[R].iASFSQNcDIYPGk.[D]	N-Term(iTRAQ4plex); C8(Carbam	1	1
[K].aATGEcTATVGkR.[S]	N-Term(iTRAQ4plex); C6(Carbam	1	1
[R].iASFSQNcDIYPGk.[D]	N-Term(iTRAQ4plex); C8(Carbam	1	1
[K].tVGSDTFYSFk.[Y]	N-Term(iTRAQ4plex); K11(iTRAQ4	1	1
[K].tVGSDTFYSFk.[Y]	N-Term(iTRAQ4plex); K11(iTRAQ4	1	1
[R].iASFSQNcDIYPGk.[D]	N-Term(iTRAQ4plex); C8(Carbam	1	1
[K].lGQSLDcNAEVYVVPWEk.[K]	N-Term(iTRAQ4plex); C7(Carbam	1	1
[K].lGQSLDcNAEVYVVPWEk.[K]	N-Term(iTRAQ4plex); C7(Carbam	1	1
[K].lGQSLDcNAEVYVVPWEk.[K]	N-Term(iTRAQ4plex); C7(Carbam	1	1
[K].lGQSLDcNAEVYVVPWEk.[K]	N-Term(iTRAQ4plex); C7(Carbam	1	1
[K].fSVATQTcQITPAEGPVVTAQYDcLGcVHPI	N-Term(iTRAQ4plex); C8(Carbam	1	1
[K].kLGQSLDcNAEVYVVPWEk.[K]	N-Term(iTRAQ4plex); C8(Carbam	1	1
[K].kLGQSLDcNAEVYVVPWEk.[K]	K1(iTRAQ4plex); C8(Carbamidon	1	1
[K].kLGQSLDcNAEVYVVPWEk.[K]	N-Term(iTRAQ4plex); K1(iTRAQ4	1	1
[K].kLGQSLDcNAEVYVVPWEk.[K]	N-Term(iTRAQ4plex); K1(iTRAQ4	1	1
[K].eNFLFLTPDck.[S]	N-Term(iTRAQ4plex); C10(Carbar	1	1
[K].eNFLFLTPDck.[S]	N-Term(iTRAQ4plex); C10(Carbar	1	1
[K].eNFLFLTPDck.[S]	N-Term(iTRAQ4plex); C10(Carbar	1	1
[K].kIYPTVNcQPLGMISLMk.[R]	N-Term(iTRAQ4plex); K1(iTRAQ4	1	1
[K].aVDAALkk.[Y]	N-Term(iTRAQ4plex); K7(iTRAQ4	1	1
[K].aATGEcTATVGk.[R]	N-Term(iTRAQ4plex); C6(Carbam	1	1
[K].aATGEcTATVGk.[R]	N-Term(iTRAQ4plex); C6(Carbam	1	1
[K].lDDDLEHQGGHVLDHGHk.[H]	N-Term(iTRAQ4plex); K18(iTRAQ4	1	1
[K].aATGEcTATVGk.[R]	N-Term(iTRAQ4plex); C6(Carbam	1	1
[K].aATGEcTATVGk.[R]	N-Term(iTRAQ4plex); C6(Carbam	1	1
[K].aATGEcTATVGk.[R]	N-Term(iTRAQ4plex); C6(Carbam	1	1
[K].aATGEcTATVGk.[R]	N-Term(iTRAQ4plex); C6(Carbam	1	1
[K].aATGEcTATVGk.[R]	N-Term(iTRAQ4plex); C6(Carbam	1	1
[K].lDDDLEHQGGHVLDHGHk.[H]	N-Term(iTRAQ4plex); K18(iTRAQ4	1	1
[K].iYPTVNcQPLGMISLMk.[R]	N-Term(iTRAQ4plex); C7(Carbam	1	1
[K].aATGEcTATVGk.[R]	N-Term(iTRAQ4plex); C6(Carbam	1	1
[K].dFVQPPTk.[I]	N-Term(iTRAQ4plex); K8(iTRAQ4	1	1
[K].sVSEINPTTQMk.[E]	N-Term(iTRAQ4plex); K12(iTRAQ4	1	1
[K].eSNEELTEScETk.[K]	N-Term(iTRAQ4plex); C10(Carbar	1	1

[K].AATGEcTATVGk.[R]	C6(Carbamidomethyl); K12(iTRA	1	1
[K].fkLDDDLEHQGGHVLDHGHk.[H]	N-Term(iTRAQ4plex); K2(iTRAQ4	1	1
[K].aVDAALkk.[Y]	N-Term(iTRAQ4plex); K7(iTRAQ4	1	1
[R].qVVAGLNFR.[I]	N-Term(iTRAQ4plex)	1	1
[R].vQVVAGk.[K]	N-Term(iTRAQ4plex); K7(iTRAQ4	1	1
[R].qVVAGLNFR.[I]	N-Term(iTRAQ4plex)	1	1
[K].aATGEcTATVGk.[R]	N-Term(iTRAQ4plex); C6(Carbam	1	1
[K].tWQDcEYk.[D]	N-Term(iTRAQ4plex); C5(Carbam	1	1
[K].AATGEcTATVGk.[R]	C6(Carbamidomethyl); K12(iTRA	1	1
[R].qVVAGLNFR.[I]	N-Term(iTRAQ4plex)	1	1
[K].LGQSLDcNAEVYVVPWEk.[K]	C7(Carbamidomethyl); K18(iTRA	1	1
[R].vQVVAGk.[K]	N-Term(iTRAQ4plex); K7(iTRAQ4	1	1
[R].vQVVAGk.[K]	N-Term(iTRAQ4plex); K7(iTRAQ4	1	1
[K].dFVQPPTk.[I]	N-Term(iTRAQ4plex); K8(iTRAQ4	1	1
[R].kHNLGHGHk.[H]	N-Term(iTRAQ4plex); K1(iTRAQ4	1	1
[R].iASFSQNcDIYPGk.[D]	N-Term(iTRAQ4plex); C8(Carbam	1	1
[K].dFVQPPTk.[I]	N-Term(iTRAQ4plex); K8(iTRAQ4	1	1
[R].QVVAGLNFR.[I]		1	1
[K].TWQDcEYk.[D]	C5(Carbamidomethyl); K8(iTRAC	1	1
[K].ENFLFLTPDck.[S]	C10(Carbamidomethyl); K11(iTR	1	1
[R].qVVAGLNFR.[I]	N-Term(iTRAQ4plex)	1	1
[K].tWQDcEYk.[D]	N-Term(iTRAQ4plex); C5(Carbam	1	1
[R].vQVVAGk.[K]	N-Term(iTRAQ4plex); K7(iTRAQ4	1	1
[K].dFVQPPTk.[I]	N-Term(iTRAQ4plex); K8(iTRAQ4	1	1
[K].kLGQSLDcNAEVYVVPWEk.[K]	N-Term(iTRAQ4plex); K1(iTRAQ4	1	1
[K].lGQSLDcNAEVYVVPWEk.[K]	N-Term(iTRAQ4plex); C7(Carbam	1	1
[R].vQVVAGkk.[Y]	N-Term(iTRAQ4plex); K7(iTRAQ4	1	1
[K].kIYPTVNcQPLGMISLMk.[R]	N-Term(iTRAQ4plex); K1(iTRAQ4	1	1
[K].ENFLFLTPDck.[S]	C10(Carbamidomethyl); K11(iTR	1	1
[K].RPPGFSPFR.[S]		1	1
[R].vQVVAGk.[K]	N-Term(iTRAQ4plex); K7(iTRAQ4	1	1
[K].eNFLFLTPDck.[S]	N-Term(iTRAQ4plex); C10(Carbai	1	1
[K].tVGSDTFYSFk.[Y]	N-Term(iTRAQ4plex); K11(iTRAQ	1	1
[K].dFVQPPTk.[I]	N-Term(iTRAQ4plex); K8(iTRAQ4	1	1
[K].iYPTVNcQPLGMISLMk.[R]	N-Term(iTRAQ4plex); C7(Carbam	1	1
[K].rPPGFSPFR.[S]	N-Term(iTRAQ4plex)	1	1
[K].yNSQNQSNNQFVLYR.[I]	N-Term(iTRAQ4plex)	1	1
[K].YFIDFVAR.[E]		1	1
[K].kYFIDFVAR.[E]	N-Term(iTRAQ4plex); K1(iTRAQ4	1	1
[K].eNFLFLTPDck.[S]	N-Term(iTRAQ4plex); C10(Carbai	1	1
[R].rHDWGHEk.[Q]	N-Term(iTRAQ4plex); K8(iTRAQ4	1	1
[K].dFVQPPTk.[I]	N-Term(iTRAQ4plex); K8(iTRAQ4	1	1
[R].vQVVAGk.[K]	N-Term(iTRAQ4plex); K7(iTRAQ4	1	1
[R].iASFSQNcDIYPGk.[D]	N-Term(iTRAQ4plex); C8(Carbam	1	1
[K].lGQSLDcNAEVYVVPWEk.[K]	N-Term(iTRAQ4plex); C7(Carbam	1	1
[K].tVGSDTFYSFk.[Y]	N-Term(iTRAQ4plex); K11(iTRAQ	1	1
[K].ENFLFLTPDck.[S]	C10(Carbamidomethyl); K11(iTR	1	1
[K].lGQSLDcNAEVYVVPWEk.[K]	N-Term(iTRAQ4plex); C7(Carbam	1	1
[K].dFVQPPTk.[I]	N-Term(iTRAQ4plex); K8(iTRAQ4	1	1
[K].yFIDFVAR.[E]	N-Term(iTRAQ4plex)	1	1
[R].iASFSQNcDIYPGk.[D]	N-Term(iTRAQ4plex); C8(Carbam	1	1
[K].tVGSDTFYSFk.[Y]	N-Term(iTRAQ4plex); K11(iTRAQ	1	1

[R].QVVAGLNFR.[I]		1	1
[K].dFVQPPTk.[I]	N-Term(iTRAQ4plex); K8(iTRAQ4	1	1
[R].iASFSQNcDIYPGk.[D]	N-Term(iTRAQ4plex); C8(Carbam	1	1
[K].TVGSDTFYSFk.[Y]	K11(iTRAQ4plex)	1	1
[K].hNLGHGHk.[H]	N-Term(iTRAQ4plex); K8(iTRAQ4	1	1
[K].kYFIDFVAR.[E]	N-Term(iTRAQ4plex)	1	1
[K].kYFIDFVAR.[E]	K1(iTRAQ4plex)	1	1
[K].TVGSDTFYSFk.[Y]	K11(iTRAQ4plex)	1	1
[K].yFIDFVAR.[E]	N-Term(iTRAQ4plex)	1	1
[K].aVDAALk.[K]	N-Term(iTRAQ4plex); K7(iTRAQ4	1	1
[K].dFVQPPTk.[I]	N-Term(iTRAQ4plex); K8(iTRAQ4	1	1
[K].dFVQPPTk.[I]	N-Term(iTRAQ4plex); K8(iTRAQ4	1	1
[K].aVDAALk.[K]	N-Term(iTRAQ4plex); K7(iTRAQ4	1	1
[K].kYFIDFVAR.[E]	N-Term(iTRAQ4plex)	1	1
[K].kYFIDFVAR.[E]	K1(iTRAQ4plex)	1	1
[K].TWQDcEYk.[D]	C5(Carbamidomethyl); K8(iTRAC	1	1
[K].TWQDcEYk.[D]	C5(Carbamidomethyl); K8(iTRAC	1	1
[K].dFVQPPTk.[I]	N-Term(iTRAQ4plex); K8(iTRAQ4	1	1
[K].iYPTVNcQPLGMISLmk.[R]	N-Term(iTRAQ4plex); C7(Carbam	1	1
[R].iASFSQNcDIYPGk.[D]	N-Term(iTRAQ4plex); C8(Carbam	1	1
[K].tVGSDTFYSFk.[Y]	N-Term(iTRAQ4plex); K11(iTRAQ	1	1
[K].tVGSDTFYSFk.[Y]	N-Term(iTRAQ4plex); K11(iTRAQ	1	1
[K].TWQDcEYk.[D]	C5(Carbamidomethyl); K8(iTRAC	1	1
[K].iYPTVNcQPLGmISLMk.[R]	N-Term(iTRAQ4plex); C7(Carbam	1	1
[K].aVDAALk.[K]	N-Term(iTRAQ4plex); K7(iTRAQ4	1	1
[K].tWQDcEYk.[D]	N-Term(iTRAQ4plex); C5(Carbam	1	1
[R].vQVVAGk.[K]	N-Term(iTRAQ4plex); K7(iTRAQ4	1	1
[K].TVGSDTFYSFk.[Y]	K11(iTRAQ4plex)	1	1
[K].TVGSDTFYSFk.[Y]	K11(iTRAQ4plex)	1	1
[R].vQVVAGk.[K]	N-Term(iTRAQ4plex); K7(iTRAQ4	1	1
[K].kYNSQNQSNNQFVLYR.[I]	N-Term(iTRAQ4plex); K1(iTRAQ4	1	1
[K].TWQDcEYk.[D]	C5(Carbamidomethyl); K8(iTRAC	1	1
[K].kLGQSLDcNAEVYVVPWEk.[K]	N-Term(iTRAQ4plex); K1(iTRAQ4	1	1
[K].qTQVSVLPEGGETPLFk.[Q]	N-Term(iTRAQ4plex); K17(iTRAQ	1	1
[K].qTQVSVLPEGGETPLFk.[Q]	N-Term(iTRAQ4plex); K17(iTRAQ	1	1
[K].aGALNSNDAFVLk.[T]	N-Term(iTRAQ4plex); K13(iTRAQ	1	1
[K].aGALNSNDAFVLk.[T]	N-Term(iTRAQ4plex); K13(iTRAQ	1	1
[K].aGALNSNDAFVLk.[T]	N-Term(iTRAQ4plex); K13(iTRAQ	1	1
[R].lFAcSNk.[I]	N-Term(iTRAQ4plex); C4(Carbam	1	1
[R].aQPVQVAEGSEPDGFWEALGGk.[A]	N-Term(iTRAQ4plex); K22(iTRAQ	1	1
[R].aQPVQVAEGSEPDGFWEALGGk.[A]	N-Term(iTRAQ4plex); K22(iTRAQ	1	1
[R].aQPVQVAEGSEPDGFWEALGGk.[A]	N-Term(iTRAQ4plex); K22(iTRAQ	1	1
[R].aQPVQVAEGSEPDGFWEALGGk.[A]	N-Term(iTRAQ4plex); K22(iTRAQ	1	1
[K].kGGVASGFk.[H]	N-Term(iTRAQ4plex); K1(iTRAQ4	1	1
[R].aQPVQVAEGSEPDGFWEALGGk.[A]	N-Term(iTRAQ4plex); K22(iTRAQ	1	1
[R].aQPVQVAEGSEPDGFWEALGGk.[A]	N-Term(iTRAQ4plex); K22(iTRAQ	1	1
[R].aQPVQVAEGSEPDGFWEALGGk.[A]	N-Term(iTRAQ4plex); K22(iTRAQ	1	1
[R].aQPVQVAEGSEPDGFWEALGGk.[A]	N-Term(iTRAQ4plex); K22(iTRAQ	1	1
[R].aQPVQVAEGSEPDGFWEALGGk.[A]	N-Term(iTRAQ4plex); K22(iTRAQ	1	1
[R].aQPVQVAEGSEPDGFWEALGGk.[A]	N-Term(iTRAQ4plex); K22(iTRAQ	1	1
[R].AQPVQVAEGSEPDGFWEALGGk.[A]	K22(iTRAQ4plex)	1	1
[K].kGGVASGFk.[H]	N-Term(iTRAQ4plex); K1(iTRAQ4	1	1

[K].nWRDPDQTDGLGLSYLSSHIANVER.[V]	N-Term(iTRAQ4plex)	1	1
[K].nWRDPDQTDGLGLSYLSSHIANVER.[V]	N-Term(iTRAQ4plex)	1	1
[K].aGkEPGLQIWR.[V]	N-Term(iTRAQ4plex); K3(iTRAQ4	1	1
[K].hVVPNEVVVQR.[L]	N-Term(iTRAQ4plex)	1	1
[R].eVQGFESATFLGYFk.[S]	N-Term(iTRAQ4plex); K15(iTRAQ	1	1
[R].eVQGFESATFLGYFk.[S]	N-Term(iTRAQ4plex); K15(iTRAQ	1	1
[R].eVQGFESATFLGYFk.[S]	N-Term(iTRAQ4plex); K15(iTRAQ	1	1
[K].tPSAAYLWVGTGASEAEk.[T]	N-Term(iTRAQ4plex); K18(iTRAQ	1	1
[K].tPSAAYLWVGTGASEAEk.[T]	N-Term(iTRAQ4plex); K18(iTRAQ	1	1
[K].tPSAAYLWVGTGASEAEk.[T]	N-Term(iTRAQ4plex); K18(iTRAQ	1	1
[K].tPSAAYLWVGTGASEAEk.[T]	N-Term(iTRAQ4plex); K18(iTRAQ	1	1
[K].tPSAAYLWVGTGASEAEk.[T]	N-Term(iTRAQ4plex); K18(iTRAQ	1	1
[K].tPSAAYLWVGTGASEAEk.[T]	N-Term(iTRAQ4plex); K18(iTRAQ	1	1
[R].dPDQTDGLGLSYLSSHIANVER.[V]	N-Term(iTRAQ4plex)	1	1
[R].eVQGFESATFLGYFk.[S]	N-Term(iTRAQ4plex); K15(iTRAQ	1	1
[R].dPDQTDGLGLSYLSSHIANVER.[V]	N-Term(iTRAQ4plex)	1	1
[R].dPDQTDGLGLSYLSSHIANVER.[V]	N-Term(iTRAQ4plex)	1	1
[K].rYIETDPANR.[D]	N-Term(iTRAQ4plex)	1	1
[K].kMDAHPPR.[L]	N-Term(iTRAQ4plex); K1(iTRAQ4	1	1
[K].tEALTSAk.[R]	N-Term(iTRAQ4plex); K8(iTRAQ4	1	1
[K].sEDcFILDHGk.[D]	N-Term(iTRAQ4plex); C4(Carbam	1	1
[K].sEDcFILDHGk.[D]	N-Term(iTRAQ4plex); C4(Carbam	1	1
[K].dSQEEEkTEALTSAk.[R]	N-Term(iTRAQ4plex); K7(iTRAQ4	1	1
[K].SEDcFILDHGk.[D]	C4(Carbamidomethyl); K11(iTRA	1	1
[K].TASDFITk.[M]	K8(iTRAQ4plex)	1	1
[K].fDLVPVPTNLYGDFFTGDAYVILk.[T]	N-Term(iTRAQ4plex); K24(iTRAQ	1	1
[K].hVVPNEVVVQR.[L]	N-Term(iTRAQ4plex)	1	1
[R].gASQAGAPQGR.[V]	N-Term(iTRAQ4plex)	1	1
[R].yIETDPANR.[D]	N-Term(iTRAQ4plex)	1	1
[R].eGGQTAPASTR.[L]	N-Term(iTRAQ4plex)	1	1
[K].tASDFITk.[M]	N-Term(iTRAQ4plex); K8(iTRAQ4	1	1
[K].tASDFITk.[M]	N-Term(iTRAQ4plex); K8(iTRAQ4	1	1
[R].aQPVQVAEGSEPDGFWEALGGk.[A]	N-Term(iTRAQ4plex); K22(iTRAQ	1	1
[R].aQPVQVAEGSEPDGFWEALGGk.[A]	N-Term(iTRAQ4plex); K22(iTRAQ	1	1
[K].TGAQELLR.[V]		1	1
[K].sEDcFILDHGk.[D]	N-Term(iTRAQ4plex); C4(Carbam	1	1
[K].gGVASGFk.[H]	N-Term(iTRAQ4plex); K8(iTRAQ4	1	1
[R].aQPVQVAEGSEPDGFWEALGGk.[A]	N-Term(iTRAQ4plex); K22(iTRAQ	1	1
[R].eVQGFESATFLGYFk.[S]	N-Term(iTRAQ4plex); K15(iTRAQ	1	1
[K].SEDcFILDHGk.[D]	C4(Carbamidomethyl); K11(iTRA	1	1
[R].yIETDPANR.[D]	N-Term(iTRAQ4plex)	1	1
[K].hVVPNEVVVQR.[L]	N-Term(iTRAQ4plex)	1	1
[K].dSQEEEkTEALTSAk.[R]	N-Term(iTRAQ4plex); K7(iTRAQ4	1	1
[K].dSQEEEk.[T]	N-Term(iTRAQ4plex); K7(iTRAQ4	1	1
[K].kGGVASGFk.[H]	N-Term(iTRAQ4plex); K1(iTRAQ4	1	1
[K].TEALTSAk.[R]	K8(iTRAQ4plex)	1	1
[R].rTPITVVk.[Q]	N-Term(iTRAQ4plex); K8(iTRAQ4	1	1
[R].tPITVVk.[Q]	N-Term(iTRAQ4plex); K7(iTRAQ4	1	1
[K].sEDcFILDHGk.[D]	N-Term(iTRAQ4plex); C4(Carbam	1	1
[R].eGGQTAPASTR.[L]	N-Term(iTRAQ4plex)	1	1
[R].lkATQVSk.[G]	N-Term(iTRAQ4plex); K2(iTRAQ4	1	1
[K].tGAQELLR.[V]	N-Term(iTRAQ4plex)	1	1

[K].ePGLQIWR.[V]	N-Term(iTRAQ4plex)	1	1
[R].eGGQTAPASTR.[L]	N-Term(iTRAQ4plex)	1	1
[R].tPITVVk.[Q]	N-Term(iTRAQ4plex); K7(iTRAQ4	1	1
[K].aGkEPGLQIWR.[V]	N-Term(iTRAQ4plex); K3(iTRAQ4	1	1
[R].lFAcSNk.[I]	N-Term(iTRAQ4plex); C4(Carbam	1	1
[K].mDAHPPR.[L]	N-Term(iTRAQ4plex)	1	1
[K].tASDFITk.[M]	N-Term(iTRAQ4plex); K8(iTRAQ4	1	1
[K].ePGLQIWR.[V]	N-Term(iTRAQ4plex)	1	1
[R].aVEVLPk.[A]	N-Term(iTRAQ4plex); K7(iTRAQ4	1	1
[K].TEALTSAk.[R]	K8(iTRAQ4plex)	1	1
[R].aQPVQVAEGSEPDGFWEALGGk.[A]	N-Term(iTRAQ4plex); K22(iTRAQ	1	1
[R].tPITVVk.[Q]	N-Term(iTRAQ4plex); K7(iTRAQ4	1	1
[K].dSQEEEk.[T]	N-Term(iTRAQ4plex); K7(iTRAQ4	1	1
[R].dYVSQFEGSALGk.[Q]	N-Term(iTRAQ4plex); K13(iTRAQ	1	1
[R].dYVSQFEGSALGk.[Q]	N-Term(iTRAQ4plex); K13(iTRAQ	1	1
[R].dYVSQFEGSALGk.[Q]	N-Term(iTRAQ4plex); K13(iTRAQ	1	1
[R].AHVDALRTHLAPYSDELRQR.[L]		1	1
[K].kWQEEMELYR.[Q]	N-Term(iTRAQ4plex); K1(iTRAQ4	1	1
[K].kWQEEMELYR.[Q]	N-Term(iTRAQ4plex); K1(iTRAQ4	1	1
[K].kWQEEMELYR.[Q]	N-Term(iTRAQ4plex); K1(iTRAQ4	1	1
[R].AHVDALRTHLAPYSDELR.[Q]		1	1
[R].hFWQQDEPPQSPWDR.[V]	N-Term(iTRAQ4plex)	1	1
[R].hFWQQDEPPQSPWDR.[V]	N-Term(iTRAQ4plex)	1	1
[R].hFWQQDEPPQSPWDR.[V]	N-Term(iTRAQ4plex)	1	1
[R].dYVSQFEGSALGk.[Q]	N-Term(iTRAQ4plex); K13(iTRAQ	1	1
[R].hFWQQDEPPQSPWDR.[V]	N-Term(iTRAQ4plex)	1	1
[R].hFWQQDEPPQSPWDR.[V]	N-Term(iTRAQ4plex)	1	1
[R].hFWQQDEPPQSPWDR.[V]	N-Term(iTRAQ4plex)	1	1
[R].AHVDALRTHLAPYSDELR.[Q]		1	1
[R].hFWQQDEPPQSPWDR.[V]	N-Term(iTRAQ4plex)	1	1
[K].vQPYLDDFQk.[K]	N-Term(iTRAQ4plex); K10(iTRAQ	1	1
[K].vQPYLDDFQk.[K]	N-Term(iTRAQ4plex); K10(iTRAQ	1	1
[K].vQPYLDDFQk.[K]	N-Term(iTRAQ4plex); K10(iTRAQ	1	1
[R].HFWQQDEPPQSPWDR.[V]		1	1
[R].tHLAPYSDELR.[Q]	N-Term(iTRAQ4plex)	1	1
[K].aTEHLSTLSEk.[A]	N-Term(iTRAQ4plex); K11(iTRAQ	1	1
[R].tHLAPYSDELR.[Q]	N-Term(iTRAQ4plex)	1	1
[R].tHLAPYSDELR.[Q]	N-Term(iTRAQ4plex)	1	1
[K].akPALEDLR.[Q]	N-Term(iTRAQ4plex); K2(iTRAQ4	1	1
[R].QkVEPLRAELQEGAR.[Q]	K2(iTRAQ4plex)	1	1
[R].qKVEPLRAELQEGAR.[Q]	N-Term(iTRAQ4plex)	1	1
[R].THLAPYSDELR.[Q]		1	1
[K].DLATVYVDVLk.[D]	K11(iTRAQ4plex)	1	1
[R].eQLGPVTQEFWDNLEk.[E]	N-Term(iTRAQ4plex); K16(iTRAQ	1	1
[K].dLATVYVDVLk.[D]	N-Term(iTRAQ4plex); K11(iTRAQ	1	1
[K].dLATVYVDVLk.[D]	N-Term(iTRAQ4plex); K11(iTRAQ	1	1
[K].dLATVYVDVLk.[D]	N-Term(iTRAQ4plex); K11(iTRAQ	1	1
[K].dLATVYVDVLk.[D]	N-Term(iTRAQ4plex); K11(iTRAQ	1	1
[K].dLATVYVDVLk.[D]	N-Term(iTRAQ4plex); K11(iTRAQ	1	1
[R].eQLGPVTQEFWDNLEk.[E]	N-Term(iTRAQ4plex); K16(iTRAQ	1	1
[R].eQLGPVTQEFWDNLEk.[E]	N-Term(iTRAQ4plex); K16(iTRAQ	1	1
[R].eQLGPVTQEFWDNLEk.[E]	N-Term(iTRAQ4plex); K16(iTRAQ	1	1

[R].eQLGPVTQEFWDNLEk.[E]	N-Term(iTRAQ4plex); K16(iTRAQ	1	1
[R].eQLGPVTQEFWDNLEk.[E]	N-Term(iTRAQ4plex); K16(iTRAQ	1	1
[R].eQLGPVTQEFWDNLEK.[E]	N-Term(iTRAQ4plex)	1	1
[R].eQLGPVTQEFWDNLEK.[E]	N-Term(iTRAQ4plex)	1	1
[R].eQLGPVTQEFWDNLEk.[E]	N-Term(iTRAQ4plex); K16(iTRAQ	1	1
[R].eQLGPVTQEFWDNLEk.[E]	N-Term(iTRAQ4plex); K16(iTRAQ	1	1
[R].eQLGPVTQEFWDNLEk.[E]	N-Term(iTRAQ4plex); K16(iTRAQ	1	1
[R].eQLGPVTQEFWDNLEk.[E]	N-Term(iTRAQ4plex); K16(iTRAQ	1	1
[R].eQLGPVTQEFWDNLEk.[E]	N-Term(iTRAQ4plex); K16(iTRAQ	1	1
[R].eQLGPVTQEFWDNLEk.[E]	N-Term(iTRAQ4plex); K16(iTRAQ	1	1
[R].eQLGPVTQEFWDNLEk.[E]	N-Term(iTRAQ4plex); K16(iTRAQ	1	1
[R].eQLGPVTQEFWDNLEk.[E]	N-Term(iTRAQ4plex); K16(iTRAQ	1	1
[R].eQLGPVTQEFWDNLEk.[E]	N-Term(iTRAQ4plex); K16(iTRAQ	1	1
[R].eQLGPVTQEFWDNLEk.[E]	N-Term(iTRAQ4plex); K16(iTRAQ	1	1
[R].eQLGPVTQEFWDNLEk.[E]	N-Term(iTRAQ4plex); K16(iTRAQ	1	1
[R].eQLGPVTQEFWDNLEk.[E]	N-Term(iTRAQ4plex); K16(iTRAQ	1	1
[R].EQLGPVTQEFWDNLEk.[E]	K16(iTRAQ4plex)	1	1
[R].EQLGPVTQEFWDNLEk.[E]	K16(iTRAQ4plex)	1	1
[R].eQLGPVTQEFWDNLEk.[E]	N-Term(iTRAQ4plex); K16(iTRAQ	1	1
[R].eQLGPVTQEFWDNLEk.[E]	N-Term(iTRAQ4plex); K16(iTRAQ	1	1
[R].eQLGPVTQEFWDNLEk.[E]	N-Term(iTRAQ4plex); K16(iTRAQ	1	1
[R].eQLGPVTQEFWDNLEk.[E]	N-Term(iTRAQ4plex); K16(iTRAQ	1	1
[R].eQLGPVTQEFWDNLEk.[E]	N-Term(iTRAQ4plex); K16(iTRAQ	1	1
[R].eQLGPVTQEFWDNLEk.[E]	N-Term(iTRAQ4plex); K16(iTRAQ	1	1
[R].eQLGPVTQEFWDNLEk.[E]	N-Term(iTRAQ4plex); K16(iTRAQ	1	1
[R].eQLGPVTQEFWDNLEk.[E]	N-Term(iTRAQ4plex); K16(iTRAQ	1	1
[R].eQLGPVTQEFWDNLEk.[E]	N-Term(iTRAQ4plex); K16(iTRAQ	1	1
[R].eQLGPVTQEFWDNLEk.[E]	N-Term(iTRAQ4plex); K16(iTRAQ	1	1
[R].eQLGPVTQEFWDNLEk.[E]	N-Term(iTRAQ4plex); K16(iTRAQ	1	1
[R].eQLGPVTQEFWDNLEk.[E]	N-Term(iTRAQ4plex); K16(iTRAQ	1	1
[R].eQLGPVTQEFWDNLEk.[E]	N-Term(iTRAQ4plex); K16(iTRAQ	1	1
[R].eQLGPVTQEFWDNLEk.[E]	N-Term(iTRAQ4plex); K16(iTRAQ	1	1
[R].eQLGPVTQEFWDNLEk.[E]	N-Term(iTRAQ4plex); K16(iTRAQ	1	1
[R].eQLGPVTQEFWDNLEk.[E]	N-Term(iTRAQ4plex); K16(iTRAQ	1	1
[R].eQLGPVTQEFWDNLEk.[E]	N-Term(iTRAQ4plex); K16(iTRAQ	1	1
[R].eQLGPVTQEFWDNLEk.[E]	N-Term(iTRAQ4plex); K16(iTRAQ	1	1
[R].eQLGPVTQEFWDNLEk.[E]	N-Term(iTRAQ4plex); K16(iTRAQ	1	1
[R].eQLGPVTQEFWDNLEk.[E]	N-Term(iTRAQ4plex); K16(iTRAQ	1	1
[R].eQLGPVTQEFWDNLEk.[E]	N-Term(iTRAQ4plex); K16(iTRAQ	1	1
[R].eQLGPVTQEFWDNLEk.[E]	N-Term(iTRAQ4plex); K16(iTRAQ	1	1
[R].dYVSQFEGSALGk.[Q]	N-Term(iTRAQ4plex); K13(iTRAQ	1	1
[R].dYVSQFEGSALGk.[Q]	N-Term(iTRAQ4plex); K13(iTRAQ	1	1
[R].dYVSQFEGSALGk.[Q]	N-Term(iTRAQ4plex); K13(iTRAQ	1	1
[R].dYVSQFEGSALGk.[Q]	N-Term(iTRAQ4plex); K13(iTRAQ	1	1
[R].dYVSQFEGSALGk.[Q]	N-Term(iTRAQ4plex); K13(iTRAQ	1	1
[R].dYVSQFEGSALGk.[Q]	N-Term(iTRAQ4plex); K13(iTRAQ	1	1
[R].dYVSQFEGSALGk.[Q]	N-Term(iTRAQ4plex); K13(iTRAQ	1	1
[R].dYVSQFEGSALGk.[Q]	N-Term(iTRAQ4plex); K13(iTRAQ	1	1
[R].dYVSQFEGSALGk.[Q]	N-Term(iTRAQ4plex); K13(iTRAQ	1	1
[K].wQEEMELYR.[Q]	N-Term(iTRAQ4plex)	1	1
[K].wQEEMELYR.[Q]	N-Term(iTRAQ4plex)	1	1
[R].DYVSQFEGSALGk.[Q]	K13(iTRAQ4plex)	1	1

[R].dYVSQFEGSALGk.[Q]	N-Term(iTRAQ4plex); K13(iTRAQ	1	1
[R].dYVSQFEGSALGk.[Q]	N-Term(iTRAQ4plex); K13(iTRAQ	1	1
[R].DYVSQFEGSALGk.[Q]	K13(iTRAQ4plex)	1	1
[R].DYVSQFEGSALGk.[Q]	K13(iTRAQ4plex)	1	1
[R].eQLGPVTQEFWDNLEk.[E]	N-Term(iTRAQ4plex); K16(iTRAQ	1	1
[R].eQLGPVTQEFWDNLEk.[E]	N-Term(iTRAQ4plex); K16(iTRAQ	1	1
[R].lAEYHAk.[A]	N-Term(iTRAQ4plex); K7(iTRAQ4	1	1
[R].EQLGPVTQEFWDNLEk.[E]	K16(iTRAQ4plex)	1	1
[K].akPALEDLR.[Q]	N-Term(iTRAQ4plex); K2(iTRAQ4	1	1
[K].akPALEDLR.[Q]	N-Term(iTRAQ4plex); K2(iTRAQ4	1	1
[K].akPALEDLR.[Q]	N-Term(iTRAQ4plex); K2(iTRAQ4	1	1
[K].aTEHLSTLSEk.[A]	N-Term(iTRAQ4plex); K11(iTRAQ	1	1
[K].vSFLSALEEYTk.[K]	N-Term(iTRAQ4plex); K12(iTRAQ	1	1
[K].vSFLSALEEYTk.[K]	N-Term(iTRAQ4plex); K12(iTRAQ	1	1
[K].akPALEDLR.[Q]	N-Term(iTRAQ4plex); K2(iTRAQ4	1	1
[K].akPALEDLR.[Q]	N-Term(iTRAQ4plex); K2(iTRAQ4	1	1
[R].aELQEGAR.[Q]	N-Term(iTRAQ4plex)	1	1
[K].VSFLSALEEYTk.[K]	K12(iTRAQ4plex)	1	1
[R].tHLAPYSDELR.[Q]	N-Term(iTRAQ4plex)	1	1
[R].THLAPYSDELR.[Q]		1	1
[K].LLDNWDSVTSTFSk.[L]	K14(iTRAQ4plex)	1	1
[K].LLDNWDSVTSTFSk.[L]	K14(iTRAQ4plex)	1	1
[K].dLATVYVDVLk.[D]	N-Term(iTRAQ4plex); K11(iTRAQ	1	1
[K].dLATVYVDVLk.[D]	N-Term(iTRAQ4plex); K11(iTRAQ	1	1
[K].lLDNWDSVTSTFSk.[L]	N-Term(iTRAQ4plex); K14(iTRAQ	1	1
[K].lLDNWDSVTSTFSk.[L]	N-Term(iTRAQ4plex); K14(iTRAQ	1	1
[K].lLDNWDSVTSTFSk.[L]	N-Term(iTRAQ4plex); K14(iTRAQ	1	1
[R].vkDLATVYVDVLk.[D]	N-Term(iTRAQ4plex); K2(iTRAQ4	1	1
[R].vkDLATVYVDVLk.[D]	N-Term(iTRAQ4plex); K2(iTRAQ4	1	1
[R].vkDLATVYVDVLk.[D]	N-Term(iTRAQ4plex); K2(iTRAQ4	1	1
[R].vkDLATVYVDVLk.[D]	N-Term(iTRAQ4plex); K2(iTRAQ4	1	1
[R].vkDLATVYVDVLk.[D]	N-Term(iTRAQ4plex); K2(iTRAQ4	1	1
[R].vkDLATVYVDVLk.[D]	N-Term(iTRAQ4plex); K2(iTRAQ4	1	1
[K].lLDNWDSVTSTFSk.[L]	N-Term(iTRAQ4plex); K14(iTRAQ	1	1
[K].lLDNWDSVTSTFSk.[L]	N-Term(iTRAQ4plex); K14(iTRAQ	1	1
[K].lLDNWDSVTSTFSk.[L]	N-Term(iTRAQ4plex); K14(iTRAQ	1	1
[K].aTEHLSTLSEk.[A]	N-Term(iTRAQ4plex); K11(iTRAQ	1	1
[R].THLAPYSDELRQR.[L]		1	1
[R].THLAPYSDELRQR.[L]		1	1
[K].dLATVYVDVLk.[D]	N-Term(iTRAQ4plex); K11(iTRAQ	1	1
[K].vSFLSALEEYTk.[K]	N-Term(iTRAQ4plex); K12(iTRAQ	1	1
[K].vSFLSALEEYTk.[K]	N-Term(iTRAQ4plex); K12(iTRAQ	1	1
[K].lREQLGPVTQEFWDNLEk.[E]	N-Term(iTRAQ4plex); K18(iTRAQ	1	1
[K].vSFLSALEEYTk.[K]	N-Term(iTRAQ4plex); K12(iTRAQ	1	1
[K].vSFLSALEEYTk.[K]	N-Term(iTRAQ4plex); K12(iTRAQ	1	1
[K].lREQLGPVTQEFWDNLEk.[E]	N-Term(iTRAQ4plex); K18(iTRAQ	1	1
[K].lREQLGPVTQEFWDNLEk.[E]	N-Term(iTRAQ4plex); K18(iTRAQ	1	1
[K].vSFLSALEEYTk.[K]	N-Term(iTRAQ4plex); K12(iTRAQ	1	1
[K].vSFLSALEEYTk.[K]	N-Term(iTRAQ4plex); K12(iTRAQ	1	1
[K].VSFLSALEEYTk.[K]	K12(iTRAQ4plex)	1	1
[R].eQLGPVTQEFWDNLEk.[E]	N-Term(iTRAQ4plex); K16(iTRAQ	1	1
[K].vSFLSALEEYTk.[K]	N-Term(iTRAQ4plex); K12(iTRAQ	1	1

[K].vSFLSALEEYTk.[K]	N-Term(iTRAQ4plex); K12(iTRAQ4	1	1
[K].vSFLSALEEYTk.[K]	N-Term(iTRAQ4plex); K12(iTRAQ4	1	1
[R].vKDLATVYVDVLk.[D]	N-Term(iTRAQ4plex); K13(iTRAQ4	1	1
[R].VkDLATVYVDVLk.[D]	K2(iTRAQ4plex); K13(iTRAQ4plex	1	1
[R].eQLGPVTQEFWDNLEk.[E]	N-Term(iTRAQ4plex); K16(iTRAQ4	1	1
[K].dLATVYVDVLk.[D]	N-Term(iTRAQ4plex); K11(iTRAQ4	1	1
[K].dLATVYVDVLk.[D]	N-Term(iTRAQ4plex); K11(iTRAQ4	1	1
[K].dLATVYVDVLk.[D]	N-Term(iTRAQ4plex); K11(iTRAQ4	1	1
[K].VSFLSALEEYTk.[K]	K12(iTRAQ4plex)	1	1
[K].WQEEMELYR.[Q]		1	1
[R].THLAPYSDELR.[Q]		1	1
[R].THLAPYSDELR.[Q]		1	1
[R].THLAPYSDELR.[Q]		1	1
[R].THLAPYSDELR.[Q]		1	1
[R].THLAPYSDELR.[Q]		1	1
[R].qkLHELQEk.[L]	N-Term(iTRAQ4plex); K2(iTRAQ4	1	1
[R].qkLHELQEk.[L]	N-Term(iTRAQ4plex); K2(iTRAQ4	1	1
[R].vkDLATVYVDVLk.[D]	N-Term(iTRAQ4plex); K2(iTRAQ4	1	1
[K].lLDNWDSVTSTFSk.[L]	N-Term(iTRAQ4plex); K14(iTRAQ4	1	1
[K].lLDNWDSVTSTFSk.[L]	N-Term(iTRAQ4plex); K14(iTRAQ4	1	1
[R].QGLLPVLESFk.[V]	K11(iTRAQ4plex)	1	1
[K].aTEHLSTLSEk.[A]	N-Term(iTRAQ4plex); K11(iTRAQ4	1	1
[K].lLDNWDSVTSTFSk.[L]	N-Term(iTRAQ4plex); K14(iTRAQ4	1	1
[K].lLDNWDSVTSTFSk.[L]	N-Term(iTRAQ4plex); K14(iTRAQ4	1	1
[K].lLDNWDSVTSTFSk.[L]	N-Term(iTRAQ4plex); K14(iTRAQ4	1	1
[R].qGLLPVLESFk.[V]	N-Term(iTRAQ4plex); K11(iTRAQ4	1	1
[K].lLDNWDSVTSTFSk.[L]	N-Term(iTRAQ4plex); K14(iTRAQ4	1	1
[R].qGLLPVLESFk.[V]	N-Term(iTRAQ4plex); K11(iTRAQ4	1	1
[K].lLDNWDSVTSTFSk.[L]	N-Term(iTRAQ4plex); K14(iTRAQ4	1	1
[K].lLDNWDSVTSTFSk.[L]	N-Term(iTRAQ4plex); K14(iTRAQ4	1	1
[R].qGLLPVLESFk.[V]	N-Term(iTRAQ4plex); K11(iTRAQ4	1	1
[R].qGLLPVLESFk.[V]	N-Term(iTRAQ4plex); K11(iTRAQ4	1	1
[K].lLDNWDSVTSTFSk.[L]	N-Term(iTRAQ4plex); K14(iTRAQ4	1	1
[R].qGLLPVLESFk.[V]	N-Term(iTRAQ4plex); K11(iTRAQ4	1	1
[K].aTEHLSTLSEk.[A]	N-Term(iTRAQ4plex); K11(iTRAQ4	1	1
[K].aTEHLSTLSEk.[A]	N-Term(iTRAQ4plex); K11(iTRAQ4	1	1
[K].aTEHLSTLSEk.[A]	N-Term(iTRAQ4plex); K11(iTRAQ4	1	1
[K].lLDNWDSVTSTFSk.[L]	N-Term(iTRAQ4plex); K14(iTRAQ4	1	1
[K].lLDNWDSVTSTFSk.[L]	N-Term(iTRAQ4plex); K14(iTRAQ4	1	1
[R].qkLHELQEk.[L]	N-Term(iTRAQ4plex); K2(iTRAQ4	1	1
[K].aTEHLSTLSEk.[A]	N-Term(iTRAQ4plex); K11(iTRAQ4	1	1
[K].aKPALEDLR.[Q]	N-Term(iTRAQ4plex)	1	1
[K].AkPALEDLR.[Q]	K2(iTRAQ4plex)	1	1
[R].tHLAPYSDELR.[Q]	N-Term(iTRAQ4plex)	1	1
[R].THLAPYSDELR.[Q]		1	1
[R].tHLAPYSDELR.[Q]	N-Term(iTRAQ4plex)	1	1
[R].tHLAPYSDELR.[Q]	N-Term(iTRAQ4plex)	1	1
[R].THLAPYSDELR.[Q]		1	1
[R].THLAPYSDELR.[Q]		1	1
[R].THLAPYSDELR.[Q]		1	1
[R].tHLAPYSDELR.[Q]	N-Term(iTRAQ4plex)	1	1
[R].THLAPYSDELR.[Q]		1	1

[R].tHLAPYSDELR.[Q]	N-Term(iTRAQ4plex)	1	1
[R].tHLAPYSDELR.[Q]	N-Term(iTRAQ4plex)	1	1
[R].tHLAPYSDELR.[Q]	N-Term(iTRAQ4plex)	1	1
[R].tHLAPYSDELR.[Q]	N-Term(iTRAQ4plex)	1	1
[R].tHLAPYSDELR.[Q]	N-Term(iTRAQ4plex)	1	1
[R].tHLAPYSDELR.[Q]	N-Term(iTRAQ4plex)	1	1
[R].tHLAPYSDELR.[Q]	N-Term(iTRAQ4plex)	1	1
[K].aTEHLSTLSEk.[A]	N-Term(iTRAQ4plex); K11(iTRAQ4	1	1
[R].tHLAPYSDELR.[Q]	N-Term(iTRAQ4plex)	1	1
[K].kWQEEMELYR.[Q]	N-Term(iTRAQ4plex); K1(iTRAQ4	1	1
[K].kWQEEMELYR.[Q]	N-Term(iTRAQ4plex); K1(iTRAQ4	1	1
[K].kWQEEMELYR.[Q]	N-Term(iTRAQ4plex); K1(iTRAQ4	1	1
[K].kWQEEMELYR.[Q]	N-Term(iTRAQ4plex); K1(iTRAQ4	1	1
[K].VQPYLDDFQk.[K]	K10(iTRAQ4plex)	1	1
[K].kWQEEMELYR.[Q]	N-Term(iTRAQ4plex); K1(iTRAQ4	1	1
[K].kWQEEMELYR.[Q]	N-Term(iTRAQ4plex); K1(iTRAQ4	1	1
[K].kWQEEMELYR.[Q]	N-Term(iTRAQ4plex); K1(iTRAQ4	1	1
[K].lSPLGEEMR.[D]	N-Term(iTRAQ4plex)	1	1
[K].akPALEDLR.[Q]	N-Term(iTRAQ4plex); K2(iTRAQ4	1	1
[K].akPALEDLR.[Q]	N-Term(iTRAQ4plex); K2(iTRAQ4	1	1
[R].tHLAPYSDELR.[Q]	N-Term(iTRAQ4plex)	1	1
[K].akPALEDLR.[Q]	N-Term(iTRAQ4plex); K2(iTRAQ4	1	1
[K].akPALEDLR.[Q]	N-Term(iTRAQ4plex); K2(iTRAQ4	1	1
[R].tHLAPYSDELR.[Q]	N-Term(iTRAQ4plex)	1	1
[K].aTEHLSTLSEk.[A]	N-Term(iTRAQ4plex); K11(iTRAQ4	1	1
[R].qkVEPLRAELQEGAR.[Q]	N-Term(iTRAQ4plex); K2(iTRAQ4	1	1
[R].aELQEGAR.[Q]	N-Term(iTRAQ4plex)	1	1
[K].akPALEDLR.[Q]	N-Term(iTRAQ4plex); K2(iTRAQ4	1	1
[K].vQPYLDDFQk.[K]	N-Term(iTRAQ4plex); K10(iTRAQ4	1	1
[K].vQPYLDDFQk.[K]	N-Term(iTRAQ4plex); K10(iTRAQ4	1	1
[K].akPALEDLR.[Q]	N-Term(iTRAQ4plex); K2(iTRAQ4	1	1
[K].vQPYLDDFQk.[K]	N-Term(iTRAQ4plex); K10(iTRAQ4	1	1
[K].vQPYLDDFQk.[K]	N-Term(iTRAQ4plex); K10(iTRAQ4	1	1
[K].vQPYLDDFQk.[K]	N-Term(iTRAQ4plex); K10(iTRAQ4	1	1
[R].tHLAPYSDELR.[Q]	N-Term(iTRAQ4plex)	1	1
[K].vQPYLDDFQk.[K]	N-Term(iTRAQ4plex); K10(iTRAQ4	1	1
[K].vEPLRAELQEGAR.[Q]	N-Term(iTRAQ4plex)	1	1
[R].dYVSQFEGSALGk.[Q]	N-Term(iTRAQ4plex); K13(iTRAQ4	1	1
[R].tHLAPYSDELR.[Q]	N-Term(iTRAQ4plex)	1	1
[R].tHLAPYSDELR.[Q]	N-Term(iTRAQ4plex)	1	1
[R].tHLAPYSDELR.[Q]	N-Term(iTRAQ4plex)	1	1
[K].aTEHLSTLSEk.[A]	N-Term(iTRAQ4plex); K11(iTRAQ4	1	1
[K].aTEHLSTLSEk.[A]	N-Term(iTRAQ4plex); K11(iTRAQ4	1	1
[K].aTEHLSTLSEk.[A]	N-Term(iTRAQ4plex); K11(iTRAQ4	1	1
[K].aTEHLSTLSEk.[A]	N-Term(iTRAQ4plex); K11(iTRAQ4	1	1
[R].aELQEGAR.[Q]	N-Term(iTRAQ4plex)	1	1
[K].vQPYLDDFQk.[K]	N-Term(iTRAQ4plex); K10(iTRAQ4	1	1
[R].eQLGPVTQEFWDNLEK.[E]	N-Term(iTRAQ4plex)	1	1
[K].LSPLGEEMR.[D]		1	1
[R].THLAPYSDELR.[Q]		1	1
[K].aTEHLSTLSEk.[A]	N-Term(iTRAQ4plex); K11(iTRAQ4	1	1
[R].hFWQQDEPPQSPWDR.[V]	N-Term(iTRAQ4plex)	1	1

[K].aTEHLSTLSEk.[A]	N-Term(iTRAQ4plex); K11(iTRAQ4	1	1
[R].DYVSQFEGSALGk.[Q]	K13(iTRAQ4plex)	1	1
[R].qkLHELQEk.[L]	N-Term(iTRAQ4plex); K2(iTRAQ4	1	1
[K].wQEEMELYR.[Q]	N-Term(iTRAQ4plex)	1	1
[R].dYVSQFEGSALGk.[Q]	N-Term(iTRAQ4plex); K13(iTRAQ4	1	1
[R].THLAPYSDELR.[Q]		1	1
[K].wQEEMELYR.[Q]	N-Term(iTRAQ4plex)	1	1
[K].akPALEDLR.[Q]	N-Term(iTRAQ4plex); K2(iTRAQ4	1	1
[R].THLAPYSDELR.[Q]		1	1
[K].kWQEEMELYR.[Q]	N-Term(iTRAQ4plex)	1	1
[K].kWQEEMELYR.[Q]	K1(iTRAQ4plex)	1	1
[R].eQLGPVTQEFWDNLEk.[E]	N-Term(iTRAQ4plex); K16(iTRAQ4	1	1
[R].tHLAPYSDELR.[Q]	N-Term(iTRAQ4plex)	1	1
[R].LAEYHAk.[A]	K7(iTRAQ4plex)	1	1
[R].aELQEGAR.[Q]	N-Term(iTRAQ4plex)	1	1
[R].AHVDALRTHLAPYSDELRQR.[L]		1	1
[R].LAEYHAk.[A]	K7(iTRAQ4plex)	1	1
[K].wQEEMELYR.[Q]	N-Term(iTRAQ4plex)	1	1
[K].kWQEEmELYR.[Q]	N-Term(iTRAQ4plex); K1(iTRAQ4	1	1
[R].DYVSQFEGSALGk.[Q]	K13(iTRAQ4plex)	1	1
[K].lSPLGEEMR.[D]	N-Term(iTRAQ4plex)	1	1
[R].THLAPYSDELR.[Q]		1	1
[R].THLAPYSDELR.[Q]		1	1
[K].vEPLRAELQEGAR.[Q]	N-Term(iTRAQ4plex)	1	1
[R].tHLAPYSDELR.[Q]	N-Term(iTRAQ4plex)	1	1
[R].eQLGPVTQEFWDNLEk.[E]	N-Term(iTRAQ4plex); K16(iTRAQ4	1	1
[R].aELQEGAR.[Q]	N-Term(iTRAQ4plex)	1	1
[K].lLDNWDSVTSTFSk.[L]	N-Term(iTRAQ4plex); K14(iTRAQ4	1	1
[K].vQPYLDDFQk.[K]	N-Term(iTRAQ4plex); K10(iTRAQ4	1	1
[R].aELQEGAR.[Q]	N-Term(iTRAQ4plex)	1	1
[K].lLDNWDSVTSTFSk.[L]	N-Term(iTRAQ4plex); K14(iTRAQ4	1	1
[R].aHVDALRTHLAPYSDELR.[Q]	N-Term(iTRAQ4plex)	1	1
[R].eQLGPVTQEFWDNLEk.[E]	N-Term(iTRAQ4plex); K16(iTRAQ4	1	1
[K].wQEEMELYR.[Q]	N-Term(iTRAQ4plex)	1	1
[K].vQPYLDDFQK.[K]	N-Term(iTRAQ4plex)	1	1
[R].tHLAPYSDELRQR.[L]	N-Term(iTRAQ4plex)	1	1
[R].aELQEGAR.[Q]	N-Term(iTRAQ4plex)	1	1
[K].wQEEMELYR.[Q]	N-Term(iTRAQ4plex)	1	1
[K].akPALEDLR.[Q]	N-Term(iTRAQ4plex); K2(iTRAQ4	1	1
[R].qkLHELQEk.[L]	N-Term(iTRAQ4plex); K2(iTRAQ4	1	1
[R].dYVSQFEGSALGk.[Q]	N-Term(iTRAQ4plex); K13(iTRAQ4	1	1
[R].aELQEGAR.[Q]	N-Term(iTRAQ4plex)	1	1
[R].aELQEGAR.[Q]	N-Term(iTRAQ4plex)	1	1
[K].vQPYLDDFQk.[K]	N-Term(iTRAQ4plex); K10(iTRAQ4	1	1
[K].dLATVYVDVLk.[D]	N-Term(iTRAQ4plex); K11(iTRAQ4	1	1
[R].THLAPYSDELR.[Q]		1	1
[R].tHLAPYSDELR.[Q]	N-Term(iTRAQ4plex)	1	1
[K].vQPYLDDFQk.[K]	N-Term(iTRAQ4plex); K10(iTRAQ4	1	1
[R].aELQEGAR.[Q]	N-Term(iTRAQ4plex)	1	1
[R].tHLAPYSDELR.[Q]	N-Term(iTRAQ4plex)	1	1
[K].lSPLGEEMR.[D]	N-Term(iTRAQ4plex)	1	1
[R].aELQEGAR.[Q]	N-Term(iTRAQ4plex)	1	1

[R].ARAHVDALR.[T]		1	1
[K].lHELQEk.[L]	N-Term(iTRAQ4plex); K7(iTRAQ4	1	1
[R].DYVSQFEGSALGk.[Q]	K13(iTRAQ4plex)	1	1
[R].qKVEPLRAELQEGAR.[Q]	N-Term(iTRAQ4plex)	1	1
[R].tHLAPYSDELRQR.[L]	N-Term(iTRAQ4plex)	1	1
[K].lHELQEk.[L]	N-Term(iTRAQ4plex); K7(iTRAQ4	1	1
[R].eQLGPVTQEFWDNLEk.[E]	N-Term(iTRAQ4plex); K16(iTRAQ	1	1
[K].dLATVYVDVLk.[D]	N-Term(iTRAQ4plex); K11(iTRAQ	1	1
[R].eQLGPVTQEFWDNLEk.[E]	N-Term(iTRAQ4plex); K16(iTRAQ	1	1
[R].EQLGPVTQEFWDNLEk.[E]	K16(iTRAQ4plex)	1	1
[R].aELQEGAR.[Q]	N-Term(iTRAQ4plex)	1	1
[R].THLAPYSDELR.[Q]		1	1
[K].lLDNWDSVTSTFSk.[L]	N-Term(iTRAQ4plex); K14(iTRAQ	1	1
[R].lAEYHAk.[A]	N-Term(iTRAQ4plex); K7(iTRAQ4	1	1
[R].THLAPYSDELR.[Q]		1	1
[R].tHLAPYSDELR.[Q]	N-Term(iTRAQ4plex)	1	1
[R].HFWQQDEPPQSPWDR.[V]		1	1
[R].THLAPYSDELR.[Q]		1	1
[K].wQEEMELYR.[Q]	N-Term(iTRAQ4plex)	1	1
[R].lEALkENGGAR.[L]	N-Term(iTRAQ4plex); K5(iTRAQ4	1	1
[K].kWQEEMELYR.[Q]	N-Term(iTRAQ4plex); K1(iTRAQ4	1	1
[R].lAEYHAk.[A]	N-Term(iTRAQ4plex); K7(iTRAQ4	1	1
[R].qKLHELQEk.[L]	N-Term(iTRAQ4plex); K9(iTRAQ4	1	1
[K].LSPLGEEMR.[D]		1	1
[R].dYVSQFEGSALGk.[Q]	N-Term(iTRAQ4plex); K13(iTRAQ	1	1
[R].lAEYHAk.[A]	N-Term(iTRAQ4plex); K7(iTRAQ4	1	1
[K].kWQEEMELYR.[Q]	N-Term(iTRAQ4plex); K1(iTRAQ4	1	1
[K].LSPLGEEmR.[D]	M8(Oxidation)	1	1
[K].WQEEmELYR.[Q]	M5(Oxidation)	1	1
[K].lLDNWDSVTSTFSk.[L]	N-Term(iTRAQ4plex); K14(iTRAQ	1	1
[K].vSFLSALEEYTk.[K]	N-Term(iTRAQ4plex); K12(iTRAQ	1	1
[K].lSPLGEEMRDR.[A]	N-Term(iTRAQ4plex)	1	1
[R].eQLGPVTQEFWDNLEk.[E]	N-Term(iTRAQ4plex); K16(iTRAQ	1	1
[R].THLAPYSDELR.[Q]		1	1
[R].LEALkENGGAR.[L]	K5(iTRAQ4plex)	1	1
[R].eQLGPVTQEFWDNLEk.[E]	N-Term(iTRAQ4plex); K16(iTRAQ	1	1
[K].akPALEDLR.[Q]	N-Term(iTRAQ4plex); K2(iTRAQ4	1	1
[K].wQEEmELYR.[Q]	N-Term(iTRAQ4plex); M5(Oxidat	1	1
[R].eQLGPVTQEFWDNLEk.[E]	N-Term(iTRAQ4plex); K16(iTRAQ	1	1
[K].lLDNWDSVTSTFSk.[L]	N-Term(iTRAQ4plex); K14(iTRAQ	1	1
[K].LHELQEk.[L]	K7(iTRAQ4plex)	1	1
[R].THLAPYSDELR.[Q]		1	1
[K].lHELQEk.[L]	N-Term(iTRAQ4plex); K7(iTRAQ4	1	1
[K].aTEHLSTLSEk.[A]	N-Term(iTRAQ4plex); K11(iTRAQ	1	1
[R].tHLAPYSDELR.[Q]	N-Term(iTRAQ4plex)	1	1
[R].LAEYHAk.[A]	K7(iTRAQ4plex)	1	1
[K].wQEEMELYR.[Q]	N-Term(iTRAQ4plex)	1	1
[K].kWQEEMELYR.[Q]	N-Term(iTRAQ4plex); K1(iTRAQ4	1	1
[R].dYVSQFEGSALGk.[Q]	N-Term(iTRAQ4plex); K13(iTRAQ	1	1
[K].lHELQEk.[L]	N-Term(iTRAQ4plex); K7(iTRAQ4	1	1
[R].eQLGPVTQEFWDNLEk.[E]	N-Term(iTRAQ4plex); K16(iTRAQ	1	1
[R].EQLGPVTQEFWDNLEk.[E]	K16(iTRAQ4plex)	1	1

[R].dYVSQFEGSALGk.[Q]	N-Term(iTRAQ4plex); K13(iTRAQ4	1	1
[K].lHELQEk.[L]	N-Term(iTRAQ4plex); K7(iTRAQ4	1	1
[K].vQPYLDDFQk.[K]	N-Term(iTRAQ4plex); K10(iTRAQ	1	1
[K].lHELQEk.[L]	N-Term(iTRAQ4plex); K7(iTRAQ4	1	1
[R].tHLAPYSDELR.[Q]	N-Term(iTRAQ4plex)	1	1
[R].aELQEGAR.[Q]	N-Term(iTRAQ4plex)	1	1
[K].lHELQEk.[L]	N-Term(iTRAQ4plex); K7(iTRAQ4	1	1
[K].lHELQEk.[L]	N-Term(iTRAQ4plex); K7(iTRAQ4	1	1
[K].vSFLSALEEYTk.[K]	N-Term(iTRAQ4plex); K12(iTRAQ	1	1
[R].eQLGPVTQEFWDNLEK.[E]	N-Term(iTRAQ4plex)	1	1
[K].lLDNWDSVTSTFSk.[L]	N-Term(iTRAQ4plex); K14(iTRAQ	1	1
[K].lLDNWDSVTSTFSk.[L]	N-Term(iTRAQ4plex); K14(iTRAQ	1	1
[R].LAEYHAk.[A]	K7(iTRAQ4plex)	1	1
[K].ATEHLSTLSEk.[A]	K11(iTRAQ4plex)	1	1
[K].lLDNWDSVTSTFSk.[L]	N-Term(iTRAQ4plex); K14(iTRAQ	1	1
[R].qGLLPVLESFK.[V]	N-Term(iTRAQ4plex)	1	1
[K].vQPYLDDFQk.[K]	N-Term(iTRAQ4plex); K10(iTRAQ	1	1
[K].dLATVYVDVLKDSGR.[D]	N-Term(iTRAQ4plex)	1	1
[K].dLATVYVDVLK.[D]	N-Term(iTRAQ4plex)	1	1
[K].kWQEEmELYR.[Q]	N-Term(iTRAQ4plex); K1(iTRAQ4	1	1
[K].lLDNWDSVTSTFSk.[L]	N-Term(iTRAQ4plex); K14(iTRAQ	1	1
[K].lHELQEk.[L]	N-Term(iTRAQ4plex); K7(iTRAQ4	1	1
[K].vQPYLDDFQk.[K]	N-Term(iTRAQ4plex); K10(iTRAQ	1	1
[R].aELQEGAR.[Q]	N-Term(iTRAQ4plex)	1	1
[K].VSFLSALEEYTk.[K]	K12(iTRAQ4plex)	1	1
[R].aELQEGAR.[Q]	N-Term(iTRAQ4plex)	1	1
[R].THLAPYSDELRQR.[L]		1	1
[K].akPALEDLR.[Q]	N-Term(iTRAQ4plex); K2(iTRAQ4	1	1
[R].AELQEGAR.[Q]		1	1
[K].lLDNWDSVTSTFSk.[L]	N-Term(iTRAQ4plex); K14(iTRAQ	1	1
[R].aELQEGAR.[Q]	N-Term(iTRAQ4plex)	1	1
[K].lLDNWDSVTSTFSk.[L]	N-Term(iTRAQ4plex); K14(iTRAQ	1	1
[R].qkLHELQEk.[L]	N-Term(iTRAQ4plex); K2(iTRAQ4	1	1
[R].THLAPYSDELR.[Q]		1	1
[R].THLAPYSDELR.[Q]		1	1
[R].lEALkENGGAR.[L]	N-Term(iTRAQ4plex); K5(iTRAQ4	1	1
[R].eQLGPVTQEFWDNLEk.[E]	N-Term(iTRAQ4plex); K16(iTRAQ	1	1
[R].EQLGPVTQEFWDNLEk.[E]	K16(iTRAQ4plex)	1	1
[R].dYVSQFEGSALGk.[Q]	N-Term(iTRAQ4plex); K13(iTRAQ	1	1
[K].lHELQEk.[L]	N-Term(iTRAQ4plex); K7(iTRAQ4	1	1
[R].dYVSQFEGSALGk.[Q]	N-Term(iTRAQ4plex); K13(iTRAQ	1	1
[K].akPALEDLR.[Q]	N-Term(iTRAQ4plex); K2(iTRAQ4	1	1
[R].AELQEGAR.[Q]		1	1
[K].vQPYLDDFQk.[K]	N-Term(iTRAQ4plex); K10(iTRAQ	1	1
[K].lHELQEk.[L]	N-Term(iTRAQ4plex); K7(iTRAQ4	1	1
[K].lHELQEk.[L]	N-Term(iTRAQ4plex); K7(iTRAQ4	1	1
[K].kWQEEMELYR.[Q]	N-Term(iTRAQ4plex); K1(iTRAQ4	1	1
[R].QGLLPVLESFk.[V]	K11(iTRAQ4plex)	1	1
[R].qKLHELQEk.[L]	N-Term(iTRAQ4plex); K9(iTRAQ4	1	1
[R].QkLHELQEk.[L]	K2(iTRAQ4plex); K9(iTRAQ4plex)	1	1
[K].lSPLGEEMR.[D]	N-Term(iTRAQ4plex)	1	1
[K].vQPYLDDFQk.[K]	N-Term(iTRAQ4plex); K10(iTRAQ	1	1

[R].lAEYHAk.[A]	N-Term(iTRAQ4plex); K7(iTRAQ4	1	1
[R].VkDLATVYVDVLk.[D]	K2(iTRAQ4plex); K13(iTRAQ4ple›	1	1
[R].vKDLATVYVDVLk.[D]	N-Term(iTRAQ4plex); K13(iTRAQ	1	1
[R].LAEYHAk.[A]	K7(iTRAQ4plex)	1	1
[K].dLEEVkAk.[V]	N-Term(iTRAQ4plex); K6(iTRAQ4	1	1
[R].THLAPYSDELR.[Q]		1	1
[R].dYVSQFEGSALGk.[Q]	N-Term(iTRAQ4plex); K13(iTRAQ	1	1
[R].qGLLPVLESFk.[V]	N-Term(iTRAQ4plex); K11(iTRAQ	1	1
[R].THLAPYSDELR.[Q]		1	1
[K].vQPYLDDFQk.[K]	N-Term(iTRAQ4plex); K10(iTRAQ	1	1
[K].vSFLSALEEYTk.[K]	N-Term(iTRAQ4plex); K12(iTRAQ	1	1
[K].aKVQPYLDDFQk.[K]	N-Term(iTRAQ4plex); K12(iTRAQ	1	1
[K].AkVQPYLDDFQk.[K]	K2(iTRAQ4plex); K12(iTRAQ4ple›	1	1
[K].VSFLSALEEYTk.[K]	K12(iTRAQ4plex)	1	1
[K].lHELQEk.[L]	N-Term(iTRAQ4plex); K7(iTRAQ4	1	1
[R].dYVSQFEGSALGk.[Q]	N-Term(iTRAQ4plex); K13(iTRAQ	1	1
[R].qkLHELQEk.[L]	N-Term(iTRAQ4plex); K2(iTRAQ4	1	1
[R].qGLLPVLESFk.[V]	N-Term(iTRAQ4plex); K11(iTRAQ	1	1
[K].DLATVYVDVLk.[D]	K11(iTRAQ4plex)	1	1
[R].lAEYHAk.[A]	N-Term(iTRAQ4plex); K7(iTRAQ4	1	1
[R].dYVSQFEGSALGk.[Q]	N-Term(iTRAQ4plex); K13(iTRAQ	1	1
[K].lHELQEk.[L]	N-Term(iTRAQ4plex); K7(iTRAQ4	1	1
[K].LLDNWDSVTSTFSk.[L]	K14(iTRAQ4plex)	1	1
[R].LEALkENGGAR.[L]	K5(iTRAQ4plex)	1	1
[K].VQPYLDDFQk.[K]	K10(iTRAQ4plex)	1	1
[R].EQLGPVTQEFWDNLEk.[E]	K16(iTRAQ4plex)	1	1
[K].vSFLSALEEYTK.[K]	N-Term(iTRAQ4plex)	1	1
[R].qkLHELQEk.[L]	N-Term(iTRAQ4plex); K2(iTRAQ4	1	1
[R].aELQEGAR.[Q]	N-Term(iTRAQ4plex)	1	1
[R].dYVSQFEGSALGk.[Q]	N-Term(iTRAQ4plex); K13(iTRAQ	1	1
[K].lHELQEk.[L]	N-Term(iTRAQ4plex); K7(iTRAQ4	1	1
[K].vQPYLDDFQk.[K]	N-Term(iTRAQ4plex); K10(iTRAQ	1	1
[K].lHELQEk.[L]	N-Term(iTRAQ4plex); K7(iTRAQ4	1	1
[K].lLDNWDSVTSTFSk.[L]	N-Term(iTRAQ4plex); K14(iTRAQ	1	1
[R].dYVSQFEGSALGk.[Q]	N-Term(iTRAQ4plex); K13(iTRAQ	1	1
[R].AHVDALRTHLAPYSDELRQR.[L]		1	1
[R].qkLHELQEk.[L]	N-Term(iTRAQ4plex); K2(iTRAQ4	1	1
[K].LSPLGEEMR.[D]		1	1
[K].kWQEEMELYR.[Q]	N-Term(iTRAQ4plex); K1(iTRAQ4	1	1
[K].vQPYLDDFQk.[K]	N-Term(iTRAQ4plex); K10(iTRAQ	1	1
[R].eQLGPVTQEFWDNLEk.[E]	N-Term(iTRAQ4plex); K16(iTRAQ	1	1
[K].aKPALEDLR.[Q]	N-Term(iTRAQ4plex)	1	1
[K].AkPALEDLR.[Q]	K2(iTRAQ4plex)	1	1
[R].LAEYHAk.[A]	K7(iTRAQ4plex)	1	1
[R].LAEYHAk.[A]	K7(iTRAQ4plex)	1	1
[R].QGLLPVLESFk.[V]	K11(iTRAQ4plex)	1	1
[R].THLAPYSDELR.[Q]		1	1
[K].lHELQEk.[L]	N-Term(iTRAQ4plex); K7(iTRAQ4	1	1
[R].DYVSQFEGSALGk.[Q]	K13(iTRAQ4plex)	1	1
[K].vQPYLDDFQk.[K]	N-Term(iTRAQ4plex); K10(iTRAQ	1	1
[R].THLAPYSDELR.[Q]		1	1
[K].ATEHLSTLSEk.[A]	K11(iTRAQ4plex)	1	1

[R].dYVSQFEGSALGk.[Q]	N-Term(iTRAQ4plex); K13(iTRAQ	1	1
[K].akPALEDLR.[Q]	N-Term(iTRAQ4plex); K2(iTRAQ4	1	1
[R].eQLGPVTQEFWDNLEk.[E]	N-Term(iTRAQ4plex); K16(iTRAQ	1	1
[R].hFWQQDEPPQSPWDR.[V]	N-Term(iTRAQ4plex)	1	1
[R].aELQEGAR.[Q]	N-Term(iTRAQ4plex)	1	1
[K].VSFLSALEEYTk.[K]	K12(iTRAQ4plex)	1	1
[R].THLAPYSDELR.[Q]		1	1
[K].wQEEmELYR.[Q]	N-Term(iTRAQ4plex); M5(Oxidat	1	1
[R].THLAPYSDELR.[Q]		1	1
[K].LHELQEk.[L]	K7(iTRAQ4plex)	1	1
[R].hFWQQDEPPQSPWDR.[V]	N-Term(iTRAQ4plex)	1	1
[R].RTHLPEVFLSk.[V]	K11(iTRAQ4plex)	1	1
[R].rTHLPEVFLSk.[V]	N-Term(iTRAQ4plex); K11(iTRAQ	1	1
[R].rTHLPEVFLSk.[V]	N-Term(iTRAQ4plex); K11(iTRAQ	1	1
[R].RTHLPEVFLSk.[V]	K11(iTRAQ4plex)	1	1
[K].HLSLLTTLSNR.[V]		1	1
[K].hLSLLTTLSNR.[V]	N-Term(iTRAQ4plex)	1	1
[R].tHLPEVFLSk.[V]	N-Term(iTRAQ4plex); K10(iTRAQ	1	1
[K].hLSLLTTLSNR.[V]	N-Term(iTRAQ4plex)	1	1
[R].vcSQYAAYGEk.[K]	N-Term(iTRAQ4plex); C2(Carbam	1	1
[K].kELSSFIDk.[G]	N-Term(iTRAQ4plex); K1(iTRAQ4	1	1
[K].hQPQEFPTYVEPTNDEIcEAFR.[K]	N-Term(iTRAQ4plex); C18(Carbar	1	1
[K].hQPQEFPTYVEPTNDEIcEAFR.[K]	N-Term(iTRAQ4plex); C18(Carbar	1	1
[R].sDFASNccSINSPPLYcDSEIDAELk.[N]	N-Term(iTRAQ4plex); C7(Carbam	1	1
[R].sDFASNccSINSPPLYcDSEIDAELk.[N]	N-Term(iTRAQ4plex); C7(Carbam	1	1
[K].dVcDPGNTk.[V]	N-Term(iTRAQ4plex); C3(Carbam	1	1
[R].sDFASNccSINSPPLYcDSEIDAELk.[N]	N-Term(iTRAQ4plex); C7(Carbam	1	1
[R].sDFASNccSINSPPLYcDSEIDAELk.[N]	N-Term(iTRAQ4plex); C7(Carbam	1	1
[R].sDFASNccSINSPPLYcDSEIDAELk.[N]	N-Term(iTRAQ4plex); C7(Carbam	1	1
[R].SDFASNccSINSPPLYcDSEIDAELk.[N]	C7(Carbamidomethyl); C8(Carba	1	1
[R].sDFASNccSINSPPLYcDSEIDAELk.[N]	N-Term(iTRAQ4plex); C7(Carbam	1	1
[R].sDFASNccSINSPPLYcDSEIDAELk.[N]	N-Term(iTRAQ4plex); C7(Carbam	1	1
[R].sDFASNccSINSPPLYcDSEIDAELk.[N]	N-Term(iTRAQ4plex); C7(Carbam	1	1
[R].sDFASNccSINSPPLYcDSEIDAELk.[N]	N-Term(iTRAQ4plex); C7(Carbam	1	1
[R].sDFASNccSINSPPLYcDSEIDAELk.[N]	N-Term(iTRAQ4plex); C7(Carbam	1	1
[R].sDFASNccSINSPPLYcDSEIDAELk.[N]	N-Term(iTRAQ4plex); C7(Carbam	1	1
[K].hQPQEFPTYVEPTNDEIcEAFR.[K]	N-Term(iTRAQ4plex); C18(Carbar	1	1
[R].sDFASNccSINSPPLYcDSEIDAELk.[N]	N-Term(iTRAQ4plex); C7(Carbam	1	1
[R].sDFASNccSINSPPLYcDSEIDAELk.[N]	N-Term(iTRAQ4plex); C7(Carbam	1	1
[K].HQPQEFPTYVEPTNDEIcEAFR.[K]	C18(Carbamidomethyl)	1	1
[K].HQPQEFPTYVEPTNDEIcEAFR.[K]	C18(Carbamidomethyl)	1	1
[K].HQPQEFPTYVEPTNDEIcEAFR.[K]	C18(Carbamidomethyl)	1	1
[K].HQPQEFPTYVEPTNDEIcEAFR.[K]	C18(Carbamidomethyl)	1	1
[K].hQPQEFPTYVEPTNDEIcEAFR.[K]	N-Term(iTRAQ4plex); C18(Carbar	1	1
[K].HQPQEFPTYVEPTNDEIcEAFR.[K]	C18(Carbamidomethyl)	1	1
[K].hQPQEFPTYVEPTNDEIcEAFR.[K]	N-Term(iTRAQ4plex); C18(Carbar	1	1
[K].hQPQEFPTYVEPTNDEIcEAFR.[K]	N-Term(iTRAQ4plex); C18(Carbar	1	1
[K].hQPQEFPTYVEPTNDEIcEAFR.[K]	N-Term(iTRAQ4plex); C18(Carbar	1	1
[R].sDFASNccSINSPPLYcDSEIDAELk.[N]	N-Term(iTRAQ4plex); C7(Carbam	1	1
[R].sDFASNccSINSPPLYcDSEIDAELk.[N]	N-Term(iTRAQ4plex); C7(Carbam	1	1
[R].sDFASNccSINSPPLYcDSEIDAELk.[N]	N-Term(iTRAQ4plex); C7(Carbam	1	1
[K].hQPQEFPTYVEPTNDEIcEAFR.[K]	N-Term(iTRAQ4plex); C18(Carbar	1	1

[K].hQPQEFPTYVEPTNDEIcEAFR.[K]	N-Term(iTRAQ4plex); C18(Carbar	1	1
[K].gQELcADYSENTFTEYk.[K]	N-Term(iTRAQ4plex); C5(Carbam	1	1
[K].gQELcADYSENTFTEYk.[K]	N-Term(iTRAQ4plex); C5(Carbam	1	1
[K].gQELcADYSENTFTEYk.[K]	N-Term(iTRAQ4plex); C5(Carbam	1	1
[K].hQPQEFPTYVEPTNDEIcEAFR.[K]	N-Term(iTRAQ4plex); C18(Carbar	1	1
[K].hQPQEFPTYVEPTNDEIcEAFR.[K]	N-Term(iTRAQ4plex); C18(Carbar	1	1
[K].hQPQEFPTYVEPTNDEIcEAFR.[K]	N-Term(iTRAQ4plex); C18(Carbar	1	1
[R].tHLPEVFLSk.[V]	N-Term(iTRAQ4plex); K10(iTRAQ	1	1
[K].hLSLLTTLSNR.[V]	N-Term(iTRAQ4plex)	1	1
[K].hLSLLTTLSNR.[V]	N-Term(iTRAQ4plex)	1	1
[K].hLSLLTTLSNR.[V]	N-Term(iTRAQ4plex)	1	1
[K].hLSLLTTLSNR.[V]	N-Term(iTRAQ4plex)	1	1
[K].dVcDPGNTk.[V]	N-Term(iTRAQ4plex); C3(Carbam	1	1
[K].kELSSFIDk.[G]	N-Term(iTRAQ4plex); K1(iTRAQ4	1	1
[K].kELSSFIDk.[G]	N-Term(iTRAQ4plex); K1(iTRAQ4	1	1
[R].rTHLPEVFLSk.[V]	N-Term(iTRAQ4plex); K11(iTRAQ	1	1
[R].RTHLPEVFLSk.[V]	K11(iTRAQ4plex)	1	1
[R].rTHLPEVFLSk.[V]	N-Term(iTRAQ4plex); K11(iTRAQ	1	1
[R].rTHLPEVFLSk.[V]	N-Term(iTRAQ4plex); K11(iTRAQ	1	1
[R].RTHLPEVFLSk.[V]	K11(iTRAQ4plex)	1	1
[R].rTHLPEVFLSk.[V]	N-Term(iTRAQ4plex); K11(iTRAQ	1	1
[R].rTHLPEVFLSk.[V]	N-Term(iTRAQ4plex); K11(iTRAQ	1	1
[R].RTHLPEVFLSk.[V]	K11(iTRAQ4plex)	1	1
[R].RTHLPEVFLSk.[V]	K11(iTRAQ4plex)	1	1
[R].rTHLPEVFLSk.[V]	N-Term(iTRAQ4plex); K11(iTRAQ	1	1
[R].tHLPEVFLSK.[V]	N-Term(iTRAQ4plex)	1	1
[K].lcMAALk.[H]	N-Term(iTRAQ4plex); C2(Carbam	1	1
[R].rTHLPEVFLSk.[V]	N-Term(iTRAQ4plex); K11(iTRAQ	1	1
[R].rTHLPEVFLSk.[V]	N-Term(iTRAQ4plex); K11(iTRAQ	1	1
[R].tHLPEVFLSk.[V]	N-Term(iTRAQ4plex); K10(iTRAQ	1	1
[R].tHLPEVFLSk.[V]	N-Term(iTRAQ4plex); K10(iTRAQ	1	1
[R].tHLPEVFLSk.[V]	N-Term(iTRAQ4plex); K10(iTRAQ	1	1
[R].tHLPEVFLSk.[V]	N-Term(iTRAQ4plex); K10(iTRAQ	1	1
[R].tHLPEVFLSk.[V]	N-Term(iTRAQ4plex); K10(iTRAQ	1	1
[R].tHLPEVFLSk.[V]	N-Term(iTRAQ4plex); K10(iTRAQ	1	1
[R].tHLPEVFLSk.[V]	N-Term(iTRAQ4plex); K10(iTRAQ	1	1
[K].eDFTSLSLVLYSR.[K]	N-Term(iTRAQ4plex)	1	1
[R].kLcMAALk.[H]	N-Term(iTRAQ4plex); K1(iTRAQ4	1	1
[R].kLcMAALk.[H]	N-Term(iTRAQ4plex); K1(iTRAQ4	1	1
[K].eDFTSLSLVLYSR.[K]	N-Term(iTRAQ4plex)	1	1
[K].eDFTSLSLVLYSR.[K]	N-Term(iTRAQ4plex)	1	1
[R].kFPSGTFEQVSQLVk.[E]	N-Term(iTRAQ4plex); K1(iTRAQ4	1	1
[R].kFPSGTFEQVSQLVk.[E]	N-Term(iTRAQ4plex); K1(iTRAQ4	1	1
[K].VPTADLEDVLPLAEDITNILSk.[C]	K22(iTRAQ4plex)	1	1
[K].vPTADLEDVLPLAEDITNILSk.[C]	N-Term(iTRAQ4plex); K22(iTRAQ	1	1
[R].RTHLPEVFLSk.[V]	K11(iTRAQ4plex)	1	1
[R].VcSQYAAYGEk.[K]	C2(Carbamidomethyl); K11(iTRA	1	1
[R].tHLPEVFLSk.[V]	N-Term(iTRAQ4plex); K10(iTRAQ	1	1
[K].sLGEccDVEDSTTcFNAk.[G]	N-Term(iTRAQ4plex); C5(Carbam	1	1
[K].sLGEccDVEDSTTcFNAk.[G]	N-Term(iTRAQ4plex); C5(Carbam	1	1
[K].sLGEccDVEDSTTcFNAk.[G]	N-Term(iTRAQ4plex); C5(Carbam	1	1
[K].sLGEccDVEDSTTcFNAk.[G]	N-Term(iTRAQ4plex); C5(Carbam	1	1

[K].sLGEccDVEDSTTcFNAk.[G]	N-Term(iTRAQ4plex); C5(Carbam	1	1
[K].sLGEccDVEDSTTcFNAk.[G]	N-Term(iTRAQ4plex); C5(Carbam	1	1
[K].fPSGTFEQVSQLVk.[E]	N-Term(iTRAQ4plex); K14(iTRAQ4	1	1
[K].gQELcADYSENTFTEYk.[K]	N-Term(iTRAQ4plex); C5(Carbam	1	1
[K].gQELcADYSENTFTEYk.[K]	N-Term(iTRAQ4plex); C5(Carbam	1	1
[K].gQELcADYSENTFTEYk.[K]	N-Term(iTRAQ4plex); C5(Carbam	1	1
[K].gQELcADYSENTFTEYk.[K]	N-Term(iTRAQ4plex); C5(Carbam	1	1
[K].gQELcADYSENTFTEYk.[K]	N-Term(iTRAQ4plex); C5(Carbam	1	1
[K].gQELcADYSENTFTEYk.[K]	N-Term(iTRAQ4plex); C5(Carbam	1	1
[K].gQELcADYSENTFTEYk.[K]	N-Term(iTRAQ4plex); C5(Carbam	1	1
[K].gQELcADYSENTFTEYk.[K]	N-Term(iTRAQ4plex); C5(Carbam	1	1
[K].gQELcADYSENTFTEYk.[K]	N-Term(iTRAQ4plex); C5(Carbam	1	1
[K].gQELcADYSENTFTEYk.[K]	N-Term(iTRAQ4plex); C5(Carbam	1	1
[K].gQELcADYSENTFTEYk.[K]	N-Term(iTRAQ4plex); C5(Carbam	1	1
[K].gQELcADYSENTFTEYk.[K]	N-Term(iTRAQ4plex); C5(Carbam	1	1
[K].eVVSLTEAccAEGADPDcYDTR.[T]	N-Term(iTRAQ4plex); C9(Carbam	1	1
[K].gQELcADYSENTFTEYk.[K]	N-Term(iTRAQ4plex); C5(Carbam	1	1
[K].eVVSLTEAccAEGADPDcYDTR.[T]	N-Term(iTRAQ4plex); C9(Carbam	1	1
[K].gQELcADYSENTFTEYk.[K]	N-Term(iTRAQ4plex); C5(Carbam	1	1
[K].eFSHLGkEDFTSLSLVLYSR.[K]	N-Term(iTRAQ4plex); K7(iTRAQ4	1	1
[K].eVVSLTEAccAEGADPDcYDTR.[T]	N-Term(iTRAQ4plex); C9(Carbam	1	1
[K].eVVSLTEAccAEGADPDcYDTR.[T]	N-Term(iTRAQ4plex); C9(Carbam	1	1
[K].eVVSLTEAccAEGADPDcYDTR.[T]	N-Term(iTRAQ4plex); C9(Carbam	1	1
[K].sYLSMVGSccTSASPTVcFLk.[E]	N-Term(iTRAQ4plex); C9(Carbam	1	1
[K].sYLSMVGSccTSASPTVcFLk.[E]	N-Term(iTRAQ4plex); C9(Carbam	1	1
[K].eLPEHTVk.[L]	N-Term(iTRAQ4plex); K8(iTRAQ4	1	1
[K].eLPEHTVk.[L]	N-Term(iTRAQ4plex); K8(iTRAQ4	1	1
[RK].tSALSAk.[SK]	N-Term(iTRAQ4plex); K7(iTRAQ4	1	2
[K].SYLSMVGSccTSASPTVcFLk.[E]	C9(Carbamidomethyl); C10(Carb	1	1
[K].ScESNSPFPVHPGTAEccTk.[E]	C2(Carbamidomethyl); C17(Carb	1	1
[K].scESNSPFPVHPGTAEccTk.[E]	N-Term(iTRAQ4plex); C2(Carbam	1	1
[K].ScESNSPFPVHPGTAEccTk.[E]	C2(Carbamidomethyl); C17(Carb	1	1
[K].sYLSMVGSccTSASPTVcFLk.[E]	N-Term(iTRAQ4plex); C9(Carbam	1	1
[K].sYLSMVGSccTSASPTVcFLk.[E]	N-Term(iTRAQ4plex); C9(Carbam	1	1
[R].vcSQYAAYGEk.[K]	N-Term(iTRAQ4plex); C2(Carbam	1	1
[R].vcSQYAAYGEk.[K]	N-Term(iTRAQ4plex); C2(Carbam	1	1
[K].fEDccQEk.[T]	N-Term(iTRAQ4plex); C4(Carbam	1	1
[R].kFPSGTFEQVSQLVk.[E]	N-Term(iTRAQ4plex); K1(iTRAQ4	1	1
[R].kFPSGTFEQVSQLVk.[E]	N-Term(iTRAQ4plex); K1(iTRAQ4	1	1
[R].kFPSGTFEQVSQLVk.[E]	N-Term(iTRAQ4plex); K15(iTRAQ4	1	1
[K].eLSSFIDk.[G]	N-Term(iTRAQ4plex); K8(iTRAQ4	1	1
[K].eLSSFIDk.[G]	N-Term(iTRAQ4plex); K8(iTRAQ4	1	1
[R].kFPSGTFEQVSQLVk.[E]	K1(iTRAQ4plex); K15(iTRAQ4plex	1	1
[R].kFPSGTFEQVSQLVk.[E]	N-Term(iTRAQ4plex); K15(iTRAQ4	1	1
[R].kFPSGTFEQVSQLVk.[E]	K1(iTRAQ4plex); K15(iTRAQ4plex	1	1
[R].kFPSGTFEQVSQLVk.[E]	N-Term(iTRAQ4plex); K1(iTRAQ4	1	1
[R].kFPSGTFEQVSQLVk.[E]	N-Term(iTRAQ4plex); K1(iTRAQ4	1	1
[K].dVcDPGNTk.[V]	N-Term(iTRAQ4plex); C3(Carbam	1	1
[K].eYANQFMWEYSTNYGQAPLSLLVSYTk.[S]	N-Term(iTRAQ4plex); K27(iTRAQ4	1	1
[R].kFPSGTFEQVSQLVk.[E]	N-Term(iTRAQ4plex); K1(iTRAQ4	1	1
[R].kFPSGTFEQVSQLVk.[E]	N-Term(iTRAQ4plex); K1(iTRAQ4	1	1
[K].tAMDVFVcTYFMPAAQLPELPDVELPTNk.	N-Term(iTRAQ4plex); C8(Carbam	1	1

[K].eLSSFIDk.[G]	N-Term(iTRAQ4plex); K8(iTRAQ4	1	1
[R].kFPSGTFEQVSQLVk.[E]	K1(iTRAQ4plex); K15(iTRAQ4ple	1	1
[R].kFPSGTFEQVSQLVk.[E]	N-Term(iTRAQ4plex); K1(iTRAQ4	1	1
[R].kFPSGTFEQVSQLVk.[E]	N-Term(iTRAQ4plex); K15(iTRAQ	1	1
[K].ccESASEDcMAk.[E]	N-Term(iTRAQ4plex); C1(Carbam	1	1
[K].eLSSFIDk.[G]	N-Term(iTRAQ4plex); K8(iTRAQ4	1	1
[K].ccESASEDcMAk.[E]	N-Term(iTRAQ4plex); C1(Carbam	1	1
[K].lcDNLSTk.[N]	N-Term(iTRAQ4plex); C2(Carbam	1	1
[R].vcSQYAAYGEk.[K]	N-Term(iTRAQ4plex); C2(Carbam	1	1
[K].scESNSPFPVHPGTAEccTk.[E]	N-Term(iTRAQ4plex); C2(Carbam	1	1
[K].scESNSPFPVHPGTAEccTk.[E]	N-Term(iTRAQ4plex); C2(Carbam	1	1
[K].scESNSPFPVHPGTAEccTk.[E]	N-Term(iTRAQ4plex); C2(Carbam	1	1
[K].dVcDPGNTk.[V]	N-Term(iTRAQ4plex); C3(Carbam	1	1
[R].vcSQYAAYGEk.[K]	N-Term(iTRAQ4plex); C2(Carbam	1	1
[R].vcSQYAAYGEk.[K]	N-Term(iTRAQ4plex); C2(Carbam	1	1
[K].ccESASEDcMAk.[E]	C1(Carbamidomethyl); C2(Carba	1	1
[R].SDFASNccSINSPPLYcDSEIDAELk.[N]	C7(Carbamidomethyl); C8(Carba	1	1
[R].RTHLPEVFLSk.[V]	K11(iTRAQ4plex)	1	1
[K].kELSSFIDk.[G]	N-Term(iTRAQ4plex); K1(iTRAQ4	1	1
[K].eLPEHTVk.[L]	N-Term(iTRAQ4plex); K8(iTRAQ4	1	1
[K].GQELcADYSENTFTEYk.[K]	C5(Carbamidomethyl); K17(iTRA	1	1
[K].hLSLLTTLSNR.[V]	N-Term(iTRAQ4plex)	1	1
[R].sDFASNccSINSPPLYcDSEIDAELk.[N]	N-Term(iTRAQ4plex); C7(Carbam	1	1
[K].HLSLLTTLSNR.[V]		1	1
[K].hLSLLTTLSNR.[V]	N-Term(iTRAQ4plex)	1	1
[RK].tSALSAk.[SK]	N-Term(iTRAQ4plex); K7(iTRAQ4	1	2
[K].fEDccQEk.[T]	N-Term(iTRAQ4plex); C4(Carbam	1	1
[RK].tSALSAk.[SK]	N-Term(iTRAQ4plex); K7(iTRAQ4	1	2
[K].dVcDPGNTk.[V]	N-Term(iTRAQ4plex); C3(Carbam	1	1
[R].tHLPEVFLSk.[V]	N-Term(iTRAQ4plex); K10(iTRAQ	1	1
[K].lcDNLSTk.[N]	N-Term(iTRAQ4plex); C2(Carbam	1	1
[R].sDFASNccSINSPPLYcDSEIDAELk.[N]	N-Term(iTRAQ4plex); C7(Carbam	1	1
[K].LcDNLSTk.[N]	C2(Carbamidomethyl); K8(iTRAC	1	1
[K].fEDccQEk.[T]	N-Term(iTRAQ4plex); C4(Carbam	1	1
[R].THLPEVFLSk.[V]	K10(iTRAQ4plex)	1	1
[K].eFSHLGkEDFTSLSLVLYSR.[K]	N-Term(iTRAQ4plex); K7(iTRAQ4	1	1
[R].sDFASNccSINSPPLYcDSEIDAELk.[N]	N-Term(iTRAQ4plex); C7(Carbam	1	1
[R].sDFASNccSINSPPLYcDSEIDAELk.[N]	N-Term(iTRAQ4plex); C7(Carbam	1	1
[K].dVcDPGNTk.[V]	N-Term(iTRAQ4plex); C3(Carbam	1	1
[K].eLPEHTVk.[L]	N-Term(iTRAQ4plex); K8(iTRAQ4	1	1
[K].LcDNLSTk.[N]	C2(Carbamidomethyl); K8(iTRAC	1	1
[R].VcSQYAAYGEk.[K]	C2(Carbamidomethyl); K11(iTRA	1	1
[K].lcDNLSTk.[N]	N-Term(iTRAQ4plex); C2(Carbam	1	1
[R].RTHLPEVFLSk.[V]	K11(iTRAQ4plex)	1	1
[RK].tSALSAk.[SK]	N-Term(iTRAQ4plex); K7(iTRAQ4	1	2
[K].gQELcADYSENTFTEYk.[K]	N-Term(iTRAQ4plex); C5(Carbam	1	1
[K].LcDNLSTk.[N]	C2(Carbamidomethyl); K8(iTRAC	1	1
[K].fEDccQEk.[T]	N-Term(iTRAQ4plex); C4(Carbam	1	1
[K].fEDccQEk.[T]	N-Term(iTRAQ4plex); C4(Carbam	1	1
[K].HLSLLTTLSNR.[V]		1	1
[R].vcSQYAAYGEk.[K]	N-Term(iTRAQ4plex); C2(Carbam	1	1
[K].lcMAALk.[H]	N-Term(iTRAQ4plex); C2(Carbam	1	1

[R].THLPEVFLSk.[V]	K10(iTRAQ4plex)	1	1
[K].akLPDATPk.[E]	N-Term(iTRAQ4plex); K2(iTRAQ4	1	1
[R].kLcMAALk.[H]	N-Term(iTRAQ4plex); K1(iTRAQ4	1	1
[RK].tSALSAk.[SK]	N-Term(iTRAQ4plex); K7(iTRAQ4	1	2
[K].HLSLLTTLSNR.[V]		1	1
[RK].tSALSAk.[SK]	N-Term(iTRAQ4plex); K7(iTRAQ4	1	2
[K].eLPEHTVk.[L]	N-Term(iTRAQ4plex); K8(iTRAQ4	1	1
[R].THLPEVFLSk.[V]	K10(iTRAQ4plex)	1	1
[K].eLPEHTVk.[L]	N-Term(iTRAQ4plex); K8(iTRAQ4	1	1
[R].sDFASNccSINSPPLYcDSEIDAELk.[N]	N-Term(iTRAQ4plex); C7(Carbam	1	1
[K].vLEPTLk.[S]	N-Term(iTRAQ4plex); K7(iTRAQ4	1	1
[K].gQELcADYSENTFTEYk.[K]	N-Term(iTRAQ4plex); C5(Carbam	1	1
[R].RTHLPEVFLSk.[V]	K11(iTRAQ4plex)	1	1
[K].eLPEHTVk.[L]	N-Term(iTRAQ4plex); K8(iTRAQ4	1	1
[R].rTHLPEVFLSk.[V]	N-Term(iTRAQ4plex); K11(iTRAQ	1	1
[K].fPSGTFEQVSQLVk.[E]	N-Term(iTRAQ4plex); K14(iTRAQ	1	1
[K].gQELcADYSENTFTEYk.[K]	N-Term(iTRAQ4plex); C5(Carbam	1	1
[K].kELSSFIDk.[G]	N-Term(iTRAQ4plex); K1(iTRAQ4	1	1
[K].lcDNLSTk.[N]	N-Term(iTRAQ4plex); C2(Carbam	1	1
[RK].tSALSAk.[SK]	N-Term(iTRAQ4plex); K7(iTRAQ4	1	2
[R].kLcMAALk.[H]	N-Term(iTRAQ4plex); K1(iTRAQ4	1	1
[K].sLGEccDVEDSTTcFNAk.[G]	N-Term(iTRAQ4plex); C5(Carbam	1	1
[K].eLSSFIDk.[G]	N-Term(iTRAQ4plex); K8(iTRAQ4	1	1
[K].eVVSLTEAccAEGADPDcYDTR.[T]	N-Term(iTRAQ4plex); C9(Carbam	1	1
[K].lPDATPk.[E]	N-Term(iTRAQ4plex); K7(iTRAQ4	1	1
[K].fEDccQEk.[T]	N-Term(iTRAQ4plex); C4(Carbam	1	1
[K].eLPEHTVk.[L]	N-Term(iTRAQ4plex); K8(iTRAQ4	1	1
[K].gQELcADYSENTFTEYk.[K]	N-Term(iTRAQ4plex); C5(Carbam	1	1
[K].LcDNLSTk.[N]	C2(Carbamidomethyl); K8(iTRAQ	1	1
[K].eVVSLTEAccAEGADPDcYDTR.[T]	N-Term(iTRAQ4plex); C9(Carbam	1	1
[R].tHLPEVFLSk.[V]	N-Term(iTRAQ4plex); K10(iTRAQ	1	1
[R].THLPEVFLSk.[V]	K10(iTRAQ4plex)	1	1
[R].kLcmAALk.[H]	N-Term(iTRAQ4plex); K1(iTRAQ4	1	1
[R].vcSQYAAYGEkkSR.[L]	N-Term(iTRAQ4plex); C2(Carbam	1	1
[K].LPDATPk.[E]	K7(iTRAQ4plex)	1	1
[K].sLGEccDVEDSTTcFNAk.[G]	N-Term(iTRAQ4plex); C5(Carbam	1	1
[K].vPTADLEDVLPLAEDITNILSk.[C]	N-Term(iTRAQ4plex); K22(iTRAQ	1	1
[K].ELSSFIDk.[G]	K8(iTRAQ4plex)	1	1
[K].gQELcADYSENTFTEYk.[K]	N-Term(iTRAQ4plex); C5(Carbam	1	1
[R].sDFASNccSINSPPLYcDSEIDAELk.[N]	N-Term(iTRAQ4plex); C7(Carbam	1	1
[K].LcDNLSTk.[N]	C2(Carbamidomethyl); K8(iTRAQ	1	1
[R].kLcmAALk.[H]	N-Term(iTRAQ4plex); K1(iTRAQ4	1	1
[K].kELSSFIDk.[G]	N-Term(iTRAQ4plex); K1(iTRAQ4	1	1
[RK].tSALSAk.[SK]	N-Term(iTRAQ4plex); K7(iTRAQ4	1	2
[K].eVVSLTEAccAEGADPDcYDTR.[T]	N-Term(iTRAQ4plex); C9(Carbam	1	1
[RK].TSALSAk.[SK]	K7(iTRAQ4plex)	1	2
[K].sLGEccDVEDSTTcFNAk.[G]	N-Term(iTRAQ4plex); C5(Carbam	1	1
[R].kLcMAALk.[H]	K1(iTRAQ4plex); C3(Carbamidom	1	1
[R].kLcMAALk.[H]	N-Term(iTRAQ4plex); C3(Carbam	1	1
[K].gQELcADYSENTFTEYk.[K]	N-Term(iTRAQ4plex); C5(Carbam	1	1
[K].lPDATPk.[E]	N-Term(iTRAQ4plex); K7(iTRAQ4	1	1
[K].HLSLLTTLSNR.[V]		1	1

[R].vcSQYAAYGEk.[K]	N-Term(iTRAQ4plex); C2(Carbam	1	1
[R].vcSQYAAYGEk.[K]	N-Term(iTRAQ4plex); C2(Carbam	1	1
[K].hQPQEFPTYVEPTNDEIcEAFR.[K]	N-Term(iTRAQ4plex); C18(Carbar	1	1
[K].ccESASEDcMAk.[E]	N-Term(iTRAQ4plex); C1(Carbam	1	1
[K].eLSSFIDk.[G]	N-Term(iTRAQ4plex); K8(iTRAQ4	1	1
[K].vLEPTLk.[S]	N-Term(iTRAQ4plex); K7(iTRAQ4	1	1
[R].sDFASNccSINSPPLYcDSEIDAELk.[N]	N-Term(iTRAQ4plex); C7(Carbam	1	1
[K].dVcDPGNTk.[V]	N-Term(iTRAQ4plex); C3(Carbam	1	1
[RK].tSALSAk.[SK]	N-Term(iTRAQ4plex); K7(iTRAQ4	1	2
[K].ccESASEDcmAk.[E]	N-Term(iTRAQ4plex); C1(Carbam	1	1
[R].kLcmAALk.[H]	N-Term(iTRAQ4plex); K1(iTRAQ4	1	1
[R].vcSQYAAYGEk.[K]	N-Term(iTRAQ4plex); C2(Carbam	1	1
[K].vLEPTLk.[S]	N-Term(iTRAQ4plex); K7(iTRAQ4	1	1
[R].kFPSGTFEQVSQLVk.[E]	N-Term(iTRAQ4plex); K1(iTRAQ4	1	1
[K].eLPEHTVk.[L]	N-Term(iTRAQ4plex); K8(iTRAQ4	1	1
[K].LcDNLSTk.[N]	C2(Carbamidomethyl); K8(iTRAC	1	1
[K].LcDNLSTk.[N]	C2(Carbamidomethyl); K8(iTRAC	1	1
[K].eLSSFIDk.[G]	N-Term(iTRAQ4plex); K8(iTRAQ4	1	1
[K].vLEPTLk.[S]	N-Term(iTRAQ4plex); K7(iTRAQ4	1	1
[R].kFPSGTFEQVSQLVk.[E]	N-Term(iTRAQ4plex); K1(iTRAQ4	1	1
[K].eLPEHTVk.[L]	N-Term(iTRAQ4plex); K8(iTRAQ4	1	1
[K].LcDNLSTk.[N]	C2(Carbamidomethyl); K8(iTRAC	1	1
[K].dVcDPGNTk.[V]	N-Term(iTRAQ4plex); C3(Carbam	1	1
[R].kLcmAALk.[H]	N-Term(iTRAQ4plex); C3(Carbam	1	1
[R].kLcmAALk.[H]	K1(iTRAQ4plex); C3(Carbamidon	1	1
[K].LcDNLSTk.[N]	C2(Carbamidomethyl); K8(iTRAC	1	1
[K].hLSLLTTLSNR.[V]	N-Term(iTRAQ4plex)	1	1
[K].vPTADLEDVLPLAEDITNILSk.[C]	N-Term(iTRAQ4plex); K22(iTRAQ	1	1
[K].eVVSLTEAccAEGADPDcYDTR.[T]	N-Term(iTRAQ4plex); C9(Carbam	1	1
[R].THLPEVFLSk.[V]	K10(iTRAQ4plex)	1	1
[K].ccESASEDcMAk.[E]	C1(Carbamidomethyl); C2(Carba	1	1
[R].vcSQYAAYGEk.[K]	N-Term(iTRAQ4plex); C2(Carbam	1	1
[K].LcDNLSTk.[N]	C2(Carbamidomethyl); K8(iTRAC	1	1
[K].fEDccQEk.[T]	N-Term(iTRAQ4plex); C4(Carbam	1	1
[R].RTHLPEVFLSk.[V]	K11(iTRAQ4plex)	1	1
[R].sDFASNccSINSPPLYcDSEIDAELk.[N]	N-Term(iTRAQ4plex); C7(Carbam	1	1
[K].hLSLLTTLSNR.[V]	N-Term(iTRAQ4plex)	1	1
[R].kLcMAALk.[H]	N-Term(iTRAQ4plex); K1(iTRAQ4	1	1
[K].gQELcADYSENTFTEYk.[K]	N-Term(iTRAQ4plex); C5(Carbam	1	1
[RK].TSALSAk.[SK]	K7(iTRAQ4plex)	1	2
[K].eVVSLTEAccAEGADPDcYDTR.[T]	N-Term(iTRAQ4plex); C9(Carbam	1	1
[K].hLSLLTTLSNR.[V]	N-Term(iTRAQ4plex)	1	1
[K].SYLSMVGSccTSASPTVcFLk.[E]	C9(Carbamidomethyl); C10(Carb	1	1
[K].LcDNLSTk.[N]	C2(Carbamidomethyl); K8(iTRAC	1	1
[R].sDFASNccSINSPPLYcDSEIDAELk.[N]	N-Term(iTRAQ4plex); C7(Carbam	1	1
[K].lcMAALk.[H]	N-Term(iTRAQ4plex); C2(Carbam	1	1
[K].kELSSFIDk.[G]	N-Term(iTRAQ4plex); K1(iTRAQ4	1	1
[R].kFPSGTFEQVSQLVk.[E]	N-Term(iTRAQ4plex); K1(iTRAQ4	1	1
[K].GQELcADYSENTFTEYk.[K]	C5(Carbamidomethyl); K17(iTRA	1	1
[R].THLPEVFLSk.[V]	K10(iTRAQ4plex)	1	1
[K].ccESASEDcMAk.[E]	N-Term(iTRAQ4plex); C1(Carbam	1	1
[K].fEDccQEk.[T]	N-Term(iTRAQ4plex); C4(Carbam	1	1

[K].LcDNLSTk.[N]	C2(Carbamidomethyl); K8(iTRAC	1	1
[K].eVVSLTEAccAEGADPDcYDTR.[T]	N-Term(iTRAQ4plex); C9(Carbam	1	1
[K].aLVQQMEQLR.[Q]	N-Term(iTRAQ4plex)	1	1
[K].aLVQQMEQLR.[Q]	N-Term(iTRAQ4plex)	1	1
[K].aLVQQMEQLR.[Q]	N-Term(iTRAQ4plex)	1	1
[K].aLVQQMEQLR.[Q]	N-Term(iTRAQ4plex)	1	1
[R].lLPHANEVSQk.[I]	N-Term(iTRAQ4plex); K11(iTRAQ	1	1
[R].TQVNTQAEQLR.[R]		1	1
[K].lNHQLEGLTFQMk.[K]	N-Term(iTRAQ4plex); K13(iTRAQ	1	1
[K].lVPFATELHER.[L]	N-Term(iTRAQ4plex)	1	1
[K].tLSLPELEQQQEQQQEQQQEQVQMLAPLE	N-Term(iTRAQ4plex)	1	1
[K].tLSLPELEQQQEQQQEQQQEQVQMLAPLE	N-Term(iTRAQ4plex)	1	1
[K].tLSLPELEQQQEQQQEQQQEQVQmLAPLE	N-Term(iTRAQ4plex); M24(Oxida	1	1
[R].lLPHANEVSQk.[I]	N-Term(iTRAQ4plex); K11(iTRAQ	1	1
[R].lLPHANEVSQk.[I]	N-Term(iTRAQ4plex); K11(iTRAQ	1	1
[K].aLVQQmEQLR.[Q]	N-Term(iTRAQ4plex); M6(Oxidat	1	1
[R].lLPHANEVSQk.[I]	N-Term(iTRAQ4plex); K11(iTRAQ	1	1
[R].lLPHANEVSQk.[I]	N-Term(iTRAQ4plex); K11(iTRAQ	1	1
[K].kLVPFATELHER.[L]	N-Term(iTRAQ4plex); K1(iTRAQ4	1	1
[K].kLVPFATELHER.[L]	N-Term(iTRAQ4plex); K1(iTRAQ4	1	1
[K].kLVPFATELHER.[L]	N-Term(iTRAQ4plex); K1(iTRAQ4	1	1
[K].kLVPFATELHER.[L]	N-Term(iTRAQ4plex); K1(iTRAQ4	1	1
[R].TQVNTQAEQLR.[R]		1	1
[K].sLAELGGHLDQQVEEFRR.[R]	N-Term(iTRAQ4plex)	1	1
[K].lVPFATELHER.[L]	N-Term(iTRAQ4plex)	1	1
[K].lVPFATELHER.[L]	N-Term(iTRAQ4plex)	1	1
[K].LNHQLEGLTFQMk.[K]	K13(iTRAQ4plex)	1	1
[K].lVPFATELHER.[L]	N-Term(iTRAQ4plex)	1	1
[K].lVPFATELHER.[L]	N-Term(iTRAQ4plex)	1	1
[K].lNHQLEGLTFQMk.[K]	N-Term(iTRAQ4plex); K13(iTRAQ	1	1
[K].lGPHAGDVEGHLSFLEk.[D]	N-Term(iTRAQ4plex); K17(iTRAQ	1	1
[K].lGPHAGDVEGHLSFLEk.[D]	N-Term(iTRAQ4plex); K17(iTRAQ	1	1
[K].lGPHAGDVEGHLSFLEk.[D]	N-Term(iTRAQ4plex); K17(iTRAQ	1	1
[K].LNHQLEGLTFQMk.[K]	K13(iTRAQ4plex)	1	1
[K].kLVPFATELHER.[L]	N-Term(iTRAQ4plex); K1(iTRAQ4	1	1
[R].ISASAEELR.[Q]		1	1
[K].LVPFATELHER.[L]		1	1
[K].aLVQQMEQLR.[Q]	N-Term(iTRAQ4plex)	1	1
[R].TQVNTQAEQLR.[R]		1	1
[R].ISASAEELRQR.[L]		1	1
[K].sLAELGGHLDQQVEEFR.[R]	N-Term(iTRAQ4plex)	1	1
[K].sLAELGGHLDQQVEEFR.[R]	N-Term(iTRAQ4plex)	1	1
[K].sLAELGGHLDQQVEEFR.[R]	N-Term(iTRAQ4plex)	1	1
[R].tQVNTQAEQLR.[R]	N-Term(iTRAQ4plex)	1	1
[R].tQVNTQAEQLR.[R]	N-Term(iTRAQ4plex)	1	1
[R].tQVNTQAEQLR.[R]	N-Term(iTRAQ4plex)	1	1
[R].tQVNTQAEQLR.[R]	N-Term(iTRAQ4plex)	1	1
[K].IDQTVEELR.[R]		1	1
[K].iDQNVEELk.[G]	N-Term(iTRAQ4plex); K9(iTRAQ4	1	1
[K].iDQNVEELk.[G]	N-Term(iTRAQ4plex); K9(iTRAQ4	1	1
[K].iDQNVEELk.[G]	N-Term(iTRAQ4plex); K9(iTRAQ4	1	1
[K].iDQTVEELR.[R]	N-Term(iTRAQ4plex)	1	1

[K].eAVEHLQk.[S]	N-Term(iTRAQ4plex); K8(iTRAQ4	1	1
[K].eAVEHLQk.[S]	N-Term(iTRAQ4plex); K8(iTRAQ4	1	1
[K].eAVEHLQk.[S]	N-Term(iTRAQ4plex); K8(iTRAQ4	1	1
[K].eAVEHLQk.[S]	N-Term(iTRAQ4plex); K8(iTRAQ4	1	1
[R].gNTEGLQk.[S]	N-Term(iTRAQ4plex); K8(iTRAQ4	1	1
[R].rVEPYGENFNk.[A]	N-Term(iTRAQ4plex); K11(iTRAQ	1	1
[R].rVEPYGENFNk.[A]	N-Term(iTRAQ4plex); K11(iTRAQ	1	1
[R].rVEPYGENFNk.[A]	N-Term(iTRAQ4plex); K11(iTRAQ	1	1
[K].kNAEELk.[A]	N-Term(iTRAQ4plex); K1(iTRAQ4	1	1
[R].LAPLAEDVR.[G]		1	1
[R].iSASAEELR.[Q]	N-Term(iTRAQ4plex)	1	1
[R].sLAPYAQDTQEk.[L]	N-Term(iTRAQ4plex); K12(iTRAQ	1	1
[R].sLAPYAQDTQEk.[L]	N-Term(iTRAQ4plex); K12(iTRAQ	1	1
[R].sLAPYAQDTQEk.[L]	N-Term(iTRAQ4plex); K12(iTRAQ	1	1
[K].lGEVNTYAGDLQk.[K]	N-Term(iTRAQ4plex); K13(iTRAQ	1	1
[R].TQVNTQAEQLR.[R]		1	1
[R].TQVNTQAEQLR.[R]		1	1
[R].eNADSLQASLRPHADELk.[A]	N-Term(iTRAQ4plex); K18(iTRAQ	1	1
[R].TQVNTQAEQLR.[R]		1	1
[R].eNADSLQASLRPHADELk.[A]	N-Term(iTRAQ4plex); K18(iTRAQ	1	1
[K].lGEVNTYAGDLQk.[K]	N-Term(iTRAQ4plex); K13(iTRAQ	1	1
[R].lTPYADEFk.[V]	N-Term(iTRAQ4plex); K9(iTRAQ4	1	1
[R].dkVNSFFSTFk.[E]	N-Term(iTRAQ4plex); K2(iTRAQ4	1	1
[R].ISASAEELR.[Q]		1	1
[R].dkVNSFFSTFk.[E]	N-Term(iTRAQ4plex); K2(iTRAQ4	1	1
[R].lEPYADQLR.[T]	N-Term(iTRAQ4plex)	1	1
[R].lAPLAEDVR.[G]	N-Term(iTRAQ4plex)	1	1
[K].lNHQLEGLTFQMk.[K]	N-Term(iTRAQ4plex); K13(iTRAQ	1	1
[K].lNHQLEGLTFQMk.[K]	N-Term(iTRAQ4plex); K13(iTRAQ	1	1
[R].rVEPYGENFNk.[A]	N-Term(iTRAQ4plex); K11(iTRAQ	1	1
[K].iDQNVEELkGR.[L]	N-Term(iTRAQ4plex); K9(iTRAQ4	1	1
[R].sLAPYAQDTQEk.[L]	N-Term(iTRAQ4plex); K12(iTRAQ	1	1
[K].eAVEHLQk.[S]	N-Term(iTRAQ4plex); K8(iTRAQ4	1	1
[K].eAVEHLQk.[S]	N-Term(iTRAQ4plex); K8(iTRAQ4	1	1
[K].lGEVNTYAGDLQk.[K]	N-Term(iTRAQ4plex); K13(iTRAQ	1	1
[K].LNHQLEGLTFQmk.[K]	M12(Oxidation); K13(iTRAQ4ple	1	1
[K].lNHQLEGLTFQmk.[K]	N-Term(iTRAQ4plex); M12(Oxida	1	1
[K].ALVQQMEQLR.[Q]		1	1
[R].iSASAEELR.[Q]	N-Term(iTRAQ4plex)	1	1
[R].eNADSLQASLRPHADELk.[A]	N-Term(iTRAQ4plex); K18(iTRAQ	1	1
[R].LAPLAEDVR.[G]		1	1
[R].gNTEGLQk.[S]	N-Term(iTRAQ4plex); K8(iTRAQ4	1	1
[K].eAVEHLQk.[S]	N-Term(iTRAQ4plex); K8(iTRAQ4	1	1
[K].sELTQQLNALFQDk.[L]	N-Term(iTRAQ4plex); K14(iTRAQ	1	1
[R].lLPHANEVSQk.[I]	N-Term(iTRAQ4plex); K11(iTRAQ	1	1
[R].LLPHANEVSQk.[I]	K11(iTRAQ4plex)	1	1
[R].rVEPYGENFNk.[A]	N-Term(iTRAQ4plex); K11(iTRAQ	1	1
[K].lGPHAGDVEGHLSFLEk.[D]	N-Term(iTRAQ4plex); K17(iTRAQ	1	1
[R].rVEPYGENFNk.[A]	N-Term(iTRAQ4plex); K11(iTRAQ	1	1
[K].vNSFFSTFk.[E]	N-Term(iTRAQ4plex); K9(iTRAQ4	1	1
[R].gNTEGLQk.[S]	N-Term(iTRAQ4plex); K8(iTRAQ4	1	1
[K].eAVEHLQk.[S]	N-Term(iTRAQ4plex); K8(iTRAQ4	1	1

[K].sELTQQLNALFQDk.[L]	N-Term(iTRAQ4plex); K14(iTRAQ	1	1
[R].ISASAEELR.[Q]		1	1
[K].aLVQQmEQLR.[Q]	N-Term(iTRAQ4plex); M6(Oxidat	1	1
[R].iSASAEELR.[Q]	N-Term(iTRAQ4plex)	1	1
[R].gNTEGLQk.[S]	N-Term(iTRAQ4plex); K8(iTRAQ4	1	1
[R].gNTEGLQk.[S]	N-Term(iTRAQ4plex); K8(iTRAQ4	1	1
[R].LLPHANEVSQk.[I]	K11(iTRAQ4plex)	1	1
[K].lVPFATELHER.[L]	N-Term(iTRAQ4plex)	1	1
[R].TQVNTQAEQLR.[R]		1	1
[K].iDQTVEELR.[R]	N-Term(iTRAQ4plex)	1	1
[K].IDQNVEELk.[G]	K9(iTRAQ4plex)	1	1
[R].RQLTPYAQR.[M]		1	1
[K].aLVQQmEQLR.[Q]	N-Term(iTRAQ4plex); M6(Oxidat	1	1
[K].iDQNVEELk.[G]	N-Term(iTRAQ4plex); K9(iTRAQ4	1	1
[R].LTPYADEFk.[V]	K9(iTRAQ4plex)	1	1
[R].lEPYADQLR.[T]	N-Term(iTRAQ4plex)	1	1
[K].lkEEIGk.[LE]	N-Term(iTRAQ4plex); K2(iTRAQ4	1	1
[K].kLVPFATELHER.[L]	N-Term(iTRAQ4plex); K1(iTRAQ4	1	1
[R].TQVNTQAEQLR.[R]		1	1
[K].EAVEHLQk.[S]	K8(iTRAQ4plex)	1	1
[K].vNSFFSTFk.[E]	N-Term(iTRAQ4plex); K9(iTRAQ4	1	1
[R].dkVNSFFSTFk.[E]	N-Term(iTRAQ4plex); K2(iTRAQ4	1	1
[R].gNTEGLQk.[S]	N-Term(iTRAQ4plex); K8(iTRAQ4	1	1
[K].lNHQLEGLTFQmKk.[N]	N-Term(iTRAQ4plex); M12(Oxida	1	1
[K].LVPFATELHER.[L]		1	1
[K].sLAELGGHLDQQVEEFR.[R]	N-Term(iTRAQ4plex)	1	1
[R].LEPYADQLR.[T]		1	1
[R].vEPYGENFNk.[A]	N-Term(iTRAQ4plex); K10(iTRAQ	1	1
[K].SLAELGGHLDQQVEEFR.[R]		1	1
[K].aLVQQMEQLR.[Q]	N-Term(iTRAQ4plex)	1	1
[R].LTPYADEFk.[V]	K9(iTRAQ4plex)	1	1
[R].lAPLAEDVR.[G]	N-Term(iTRAQ4plex)	1	1
[K].lkEEIGk.[LE]	N-Term(iTRAQ4plex); K2(iTRAQ4	1	1
[R].tQVNTQAEQLR.[R]	N-Term(iTRAQ4plex)	1	1
[R].ISASAEELRQR.[L]		1	1
[R].iSASAEELR.[Q]	N-Term(iTRAQ4plex)	1	1
[R].lTPYADEFk.[V]	N-Term(iTRAQ4plex); K9(iTRAQ4	1	1
[R].lLPHANEVSQk.[I]	N-Term(iTRAQ4plex); K11(iTRAQ	1	1
[K].iDQTVEELR.[R]	N-Term(iTRAQ4plex)	1	1
[K].lVPFATELHER.[L]	N-Term(iTRAQ4plex)	1	1
[R].rVEPYGENFNk.[A]	N-Term(iTRAQ4plex); K11(iTRAQ	1	1
[K].lkEEIGk.[LE]	N-Term(iTRAQ4plex); K2(iTRAQ4	1	1
[K].lNHQLEGLTFQMk.[K]	N-Term(iTRAQ4plex); K13(iTRAQ	1	1
[R].LEPYADQLR.[T]		1	1
[R].ISASAEELR.[Q]		1	1
[R].vEPYGENFNk.[A]	N-Term(iTRAQ4plex); K10(iTRAQ	1	1
[K].sVGSQWASPENPcLINEcVR.[V]	N-Term(iTRAQ4plex); C13(Carbar	1	1
[K].sVGSQWASPENPcLINEcVR.[V]	N-Term(iTRAQ4plex); C13(Carbar	1	1
[K].aFVLSSVDELEQQR.[D]	N-Term(iTRAQ4plex)	1	1
[K].aFVLSSVDELEQQR.[D]	N-Term(iTRAQ4plex)	1	1
[R].iLAGPAGDSNVVk.[L]	N-Term(iTRAQ4plex); K13(iTRAQ	1	1
[R].lPGDIQVVPIGVGPNANVQELER.[I]	N-Term(iTRAQ4plex)	1	1

[K].aVVILVTDVSVDSVDAAADAAR.[S]	N-Term(iTRAQ4plex)	1	1
[R].icMDEDGNEk.[R]	N-Term(iTRAQ4plex); C2(Carbam	1	1
[R].gLRPScPNSQSPVk.[V]	N-Term(iTRAQ4plex); C6(Carbam	1	1
[R].vSPPScPPHR.[L]	N-Term(iTRAQ4plex); C6(Carbam	1	1
[K].qHSDPcALNPR.[M]	N-Term(iTRAQ4plex); C6(Carbam	1	1
[K].eFMEEVIQR.[M]	N-Term(iTRAQ4plex)	1	1
[K].eFMEEVIQR.[M]	N-Term(iTRAQ4plex)	1	1
[R].aEGLEcTk.[T]	N-Term(iTRAQ4plex); C6(Carbam	1	1
[R].yIILLLGk.[A]	N-Term(iTRAQ4plex); K8(iTRAQ4	1	1
[K].yAGSQVASTSEVLk.[Y]	N-Term(iTRAQ4plex); K14(iTRAQ	1	1
[R].skEFMEEVIQR.[M]	N-Term(iTRAQ4plex); K2(iTRAQ4	1	1
[K].aHLLSLVDVMQR.[E]	N-Term(iTRAQ4plex)	1	1
[R].gDSQSSWk.[S]	N-Term(iTRAQ4plex); K8(iTRAQ4	1	1
[K].eYAPGETVk.[I]	N-Term(iTRAQ4plex); K9(iTRAQ4	1	1
[R].cPcFHQGk.[E]	N-Term(iTRAQ4plex); C1(Carbam	1	1
[K].aLSVVWDR.[H]	N-Term(iTRAQ4plex)	1	1
[R].dETLQDGcDTHFck.[V]	N-Term(iTRAQ4plex); C8(Carbam	1	1
[R].vkEEVFIQQR.[N]	N-Term(iTRAQ4plex); K2(iTRAQ4	1	1
[R].sLScRPPMVk.[L]	N-Term(iTRAQ4plex); C4(Carbam	1	1
[K].tTcNPcPLGYk.[E]	N-Term(iTRAQ4plex); C3(Carbam	1	1
[R].eQAPNLVYMVTGNPASDEIk.[R]	N-Term(iTRAQ4plex); K20(iTRAQ	1	1
[K].gcSHPSVk.[C]	N-Term(iTRAQ4plex); C2(Carbam	1	1
[K].rPGDVWTLPDQcHTVTcQPDGQTLLk.[S]	N-Term(iTRAQ4plex); C12(Carbar	1	1
[R].sLScRPPMVk.[L]	N-Term(iTRAQ4plex); C4(Carbam	1	1
[R].aEGLEcTk.[T]	N-Term(iTRAQ4plex); C6(Carbam	1	1
[R].gGQIMTLk.[R]	N-Term(iTRAQ4plex); K8(iTRAQ4	1	1
[R].lLDLVFLLDGSSR.[L]	N-Term(iTRAQ4plex)	1	1
[R].tNTGLALR.[Y]	N-Term(iTRAQ4plex)	1	1
[K].aPTcGLcEVAR.[L]	N-Term(iTRAQ4plex); C4(Carbam	1	1
[K].gLWEQcQLLk.[S]	N-Term(iTRAQ4plex); C6(Carbam	1	1
[R].sGFTYVLHEGEccGR.[C]	N-Term(iTRAQ4plex); C12(Carbar	1	1
[K].lSGEAYGFVAR.[I]	N-Term(iTRAQ4plex)	1	1
[R].YGLVTYATYPk.[I]	K11(iTRAQ4plex)	1	1
[R].dLLYIGk.[D]	N-Term(iTRAQ4plex); K7(iTRAQ4	1	1
[K].vASYGVkPR.[Y]	N-Term(iTRAQ4plex); K7(iTRAQ4	1	1
[K].vkDISEVVTPR.[F]	N-Term(iTRAQ4plex); K2(iTRAQ4	1	1
[K].eAGIPEFYDYDVALIk.[L]	N-Term(iTRAQ4plex); K16(iTRAQ	1	1
[K].eAGIPEFYDYDVALIk.[L]	N-Term(iTRAQ4plex); K16(iTRAQ	1	1
[K].eAGIPEFYDYDVALIk.[L]	N-Term(iTRAQ4plex); K16(iTRAQ	1	1
[K].kDNEQHVFk.[V]	N-Term(iTRAQ4plex); K1(iTRAQ4	1	1
[R].sTGSWSTLk.[T]	N-Term(iTRAQ4plex); K9(iTRAQ4	1	1
[R].sTGSWSTLk.[T]	N-Term(iTRAQ4plex); K9(iTRAQ4	1	1
[K].vASYGVkPR.[Y]	N-Term(iTRAQ4plex); K7(iTRAQ4	1	1
[K].eAGIPEFYDYDVALIk.[L]	N-Term(iTRAQ4plex); K16(iTRAQ	1	1
[K].vASYGVkPR.[Y]	N-Term(iTRAQ4plex); K7(iTRAQ4	1	1
[K].vASYGVkPR.[Y]	N-Term(iTRAQ4plex); K7(iTRAQ4	1	1
[K].kcLVNLIEk.[V]	N-Term(iTRAQ4plex); K1(iTRAQ4	1	1
[K].yGQTIRPIcLPcTEGTTR.[A]	N-Term(iTRAQ4plex); C9(Carbam	1	1
[K].vASYGVkPR.[Y]	N-Term(iTRAQ4plex); K7(iTRAQ4	1	1
[K].iSVIRPSk.[G]	N-Term(iTRAQ4plex); K8(iTRAQ4	1	1
[K].iSVIRPSk.[G]	N-Term(iTRAQ4plex); K8(iTRAQ4	1	1
[K].iSVIRPSk.[G]	N-Term(iTRAQ4plex); K8(iTRAQ4	1	1

[K].iSVIRPSk.[G]	N-Term(iTRAQ4plex); K8(iTRAQ4	1	1
[K].iSVIRPSk.[G]	N-Term(iTRAQ4plex); K8(iTRAQ4	1	1
[K].kDNEQHVFk.[V]	N-Term(iTRAQ4plex); K1(iTRAQ4	1	1
[R].fLcTGGVSPYADPNTcR.[G]	N-Term(iTRAQ4plex); C3(Carbam	1	1
[R].fLcTGGVSPYADPNTcR.[G]	N-Term(iTRAQ4plex); C3(Carbam	1	1
[R].fLcTGGVSPYADPNTcR.[G]	N-Term(iTRAQ4plex); C3(Carbam	1	1
[R].fLcTGGVSPYADPNTcR.[G]	N-Term(iTRAQ4plex); C3(Carbam	1	1
[K].cLVNLIEk.[V]	N-Term(iTRAQ4plex); C1(Carbam	1	1
[R].yGLVTYATYPk.[I]	N-Term(iTRAQ4plex); K11(iTRAQ	1	1
[R].yGLVTYATYPk.[I]	N-Term(iTRAQ4plex); K11(iTRAQ	1	1
[K].vkDISEVVTPR.[F]	N-Term(iTRAQ4plex); K2(iTRAQ4	1	1
[R].gDSGGPLIVHkR.[S]	N-Term(iTRAQ4plex); K11(iTRAQ	1	1
[K].vASYGVkPR.[Y]	N-Term(iTRAQ4plex); K7(iTRAQ4	1	1
[K].gHEScMGAVVSEYFVLTAAHcFTVDDk.[E]	N-Term(iTRAQ4plex); C5(Carbam	1	1
[K].kEAGIPEFYDYDVALIk.[L]	N-Term(iTRAQ4plex); K1(iTRAQ4	1	1
[K].kEAGIPEFYDYDVALIk.[L]	N-Term(iTRAQ4plex); K1(iTRAQ4	1	1
[K].kEAGIPEFYDYDVALIk.[L]	N-Term(iTRAQ4plex); K1(iTRAQ4	1	1
[R].lLQEGQALEYVcPSGFYPYPVQTR.[T]	N-Term(iTRAQ4plex); C12(Carba	1	1
[K].QLNEINYEDHk.[L]	K11(iTRAQ4plex)	1	1
[K].eELLPAQDIk.[A]	N-Term(iTRAQ4plex); K10(iTRAQ	1	1
[K].eELLPAQDIk.[A]	N-Term(iTRAQ4plex); K10(iTRAQ	1	1
[R].HVIILMTDGLHNMGGDPITVIDEIR.[D]		1	1
[R].hVIILMTDGLHNMGGDPITVIDEIR.[D]	N-Term(iTRAQ4plex)	1	1
[R].hVIILMTDGLHNMGGDPITVIDEIR.[D]	N-Term(iTRAQ4plex)	1	1
[K].vSEADSSNADWVTk.[Q]	N-Term(iTRAQ4plex); K14(iTRAQ	1	1
[K].vSEADSSNADWVTk.[Q]	N-Term(iTRAQ4plex); K14(iTRAQ	1	1
[K].vSEADSSNADWVTk.[Q]	N-Term(iTRAQ4plex); K14(iTRAQ	1	1
[K].aLFVSEEEk.[K]	N-Term(iTRAQ4plex); K9(iTRAQ4	1	1
[K].aLFVSEEEk.[K]	N-Term(iTRAQ4plex); K9(iTRAQ4	1	1
[R].dAQYAPGYDk.[V]	N-Term(iTRAQ4plex); K10(iTRAQ	1	1
[R].dAQYAPGYDk.[V]	N-Term(iTRAQ4plex); K10(iTRAQ	1	1
[K].kDNEQHVFk.[V]	N-Term(iTRAQ4plex); K1(iTRAQ4	1	1
[K].kDNEQHVFk.[V]	N-Term(iTRAQ4plex); K1(iTRAQ4	1	1
[R].lLQEGQALEYVcPSGFYPYPVQTR.[T]	N-Term(iTRAQ4plex); C12(Carba	1	1
[R].gDSGGPLIVHkR.[S]	N-Term(iTRAQ4plex); K11(iTRAQ	1	1
[R].LEDSVTYHcSR.[G]	C9(Carbamidomethyl)	1	1
[R].hVIILMTDGLHNMGGDPITVIDEIR.[D]	N-Term(iTRAQ4plex)	1	1
[R].HVIILMTDGLHNMGGDPITVIDEIR.[D]		1	1
[R].lLQEGQALEYVcPSGFYPYPVQTR.[T]	N-Term(iTRAQ4plex); C12(Carba	1	1
[K].ekLQDEDLGFL.[-]	N-Term(iTRAQ4plex); K2(iTRAQ4	1	1
[K].ekLQDEDLGFL.[-]	N-Term(iTRAQ4plex); K2(iTRAQ4	1	1
[K].qLNEINYEDHk.[L]	N-Term(iTRAQ4plex); K11(iTRAQ	1	1
[K].qLNEINYEDHk.[L]	N-Term(iTRAQ4plex); K11(iTRAQ	1	1
[K].qLNEINYEDHk.[L]	N-Term(iTRAQ4plex); K11(iTRAQ	1	1
[R].GDSGGPLIVHk.[R]	K11(iTRAQ4plex)	1	1
[K].kDNEQHVFk.[V]	N-Term(iTRAQ4plex); K1(iTRAQ4	1	1
[R].lEDSVTYHcSR.[G]	N-Term(iTRAQ4plex); C9(Carbam	1	1
[K].aLFVSEEEkk.[L]	N-Term(iTRAQ4plex); K9(iTRAQ4	1	1
[K].dISEVVTPR.[F]	N-Term(iTRAQ4plex)	1	1
[R].DFHINLFQVLPWLk.[E]	K14(iTRAQ4plex)	1	1
[R].DFHINLFQVLPWLk.[E]	K14(iTRAQ4plex)	1	1
[R].eDYLDVYVFGVGPLVNQVNINALASk.[K]	N-Term(iTRAQ4plex); K26(iTRAQ	1	1

[R].eDYLDVYVFGVGPLVNQVNINALASk.[K]	N-Term(iTRAQ4plex); K26(iTRAQ	1	1
[R].gDSGGPLIVHk.[R]	N-Term(iTRAQ4plex); K11(iTRAQ	1	1
[R].gDSGGPLIVHk.[R]	N-Term(iTRAQ4plex); K11(iTRAQ	1	1
[R].lPPTTTcQQQk.[E]	N-Term(iTRAQ4plex); C7(Carbam	1	1
[R].lPPTTTcQQQk.[E]	N-Term(iTRAQ4plex); C7(Carbam	1	1
[R].gDSGGPLIVHk.[R]	N-Term(iTRAQ4plex); K11(iTRAQ	1	1
[R].lPPTTTcQQQk.[E]	N-Term(iTRAQ4plex); C7(Carbam	1	1
[R].lEDSVTYHcSR.[G]	N-Term(iTRAQ4plex); C9(Carbam	1	1
[R].DAQYAPGYDk.[V]	K10(iTRAQ4plex)	1	1
[R].fIQVGVISWGVVDVck.[N]	N-Term(iTRAQ4plex); C15(Carbar	1	1
[R].fIQVGVISWGVVDVck.[N]	N-Term(iTRAQ4plex); C15(Carbar	1	1
[R].LPPTTTcQQQk.[E]	C7(Carbamidomethyl); K11(iTRA	1	1
[K].gHEScMGAVVSEYFVLTAAHcFTVDDk.[E]	N-Term(iTRAQ4plex); C5(Carbam	1	1
[K].yGQTIRPIcLPcTEGTTR.[A]	N-Term(iTRAQ4plex); C9(Carbam	1	1
[K].kDNEQHVFk.[V]	N-Term(iTRAQ4plex); K1(iTRAQ4	1	1
[R].fLcTGGVSPYADPNTcR.[G]	N-Term(iTRAQ4plex); C3(Carbam	1	1
[K].dISEVVTPR.[F]	N-Term(iTRAQ4plex)	1	1
[K].lQDEDLGFL.[-]	N-Term(iTRAQ4plex)	1	1
[K].EELLPAQDIk.[A]	K10(iTRAQ4plex)	1	1
[K].eAGIPEFYDYDVALIk.[L]	N-Term(iTRAQ4plex); K16(iTRAQ	1	1
[K].ekLQDEDLGFL.[-]	N-Term(iTRAQ4plex); K2(iTRAQ4	1	1
[K].iSVIRPSk.[G]	N-Term(iTRAQ4plex); K8(iTRAQ4	1	1
[R].lPPTTTcQQQk.[E]	N-Term(iTRAQ4plex); C7(Carbam	1	1
[K].dISEVVTPR.[F]	N-Term(iTRAQ4plex)	1	1
[K].dISEVVTPR.[F]	N-Term(iTRAQ4plex)	1	1
[K].eELLPAQDIk.[A]	N-Term(iTRAQ4plex); K10(iTRAQ	1	1
[R].dAQYAPGYDk.[V]	N-Term(iTRAQ4plex); K10(iTRAQ	1	1
[K].aLFVSEEEk.[K]	N-Term(iTRAQ4plex); K9(iTRAQ4	1	1
[K].yGQTIRPIcLPcTEGTTR.[A]	N-Term(iTRAQ4plex); C9(Carbam	1	1
[R].dAQYAPGYDk.[V]	N-Term(iTRAQ4plex); K10(iTRAQ	1	1
[R].fIQVGVISWGVVDVck.[N]	N-Term(iTRAQ4plex); C15(Carbar	1	1
[K].kDNEQHVFk.[V]	N-Term(iTRAQ4plex); K1(iTRAQ4	1	1
[K].dNEQHVFk.[V]	N-Term(iTRAQ4plex); K8(iTRAQ4	1	1
[R].dLLYIGk.[D]	N-Term(iTRAQ4plex); K7(iTRAQ4	1	1
[R].gDSGGPLIVHkR.[S]	N-Term(iTRAQ4plex); K11(iTRAQ	1	1
[K].lQDEDLGFL.[-]	N-Term(iTRAQ4plex)	1	1
[R].FLcTGGVSPYADPNTcR.[G]	C3(Carbamidomethyl); C16(Carb	1	1
[R].dAQYAPGYDk.[V]	N-Term(iTRAQ4plex); K10(iTRAQ	1	1
[K].eELLPAQDIk.[A]	N-Term(iTRAQ4plex); K10(iTRAQ	1	1
[R].dLLYIGk.[D]	N-Term(iTRAQ4plex); K7(iTRAQ4	1	1
[K].vASYGVkPR.[Y]	N-Term(iTRAQ4plex); K7(iTRAQ4	1	1
[R].sTGSWSTLk.[T]	N-Term(iTRAQ4plex); K9(iTRAQ4	1	1
[K].kDNEQHVFk.[V]	N-Term(iTRAQ4plex); K1(iTRAQ4	1	1
[R].lEDSVTYHcSR.[G]	N-Term(iTRAQ4plex); C9(Carbam	1	1
[K].kDNEQHVFk.[V]	N-Term(iTRAQ4plex); K1(iTRAQ4	1	1
[K].kDNEQHVFk.[V]	N-Term(iTRAQ4plex); K1(iTRAQ4	1	1
[K].yGQTIRPIcLPcTEGTTR.[A]	N-Term(iTRAQ4plex); C9(Carbam	1	1
[K].dNEQHVFk.[V]	N-Term(iTRAQ4plex); K8(iTRAQ4	1	1
[R].yGLVTYATYPk.[I]	N-Term(iTRAQ4plex); K11(iTRAQ	1	1
[K].vSVGGEk.[R]	N-Term(iTRAQ4plex); K7(iTRAQ4	1	1
[K].kDNEQHVFk.[V]	N-Term(iTRAQ4plex); K1(iTRAQ4	1	1
[K].ISVIRPSk.[G]	K8(iTRAQ4plex)	1	1

[K].dNEQHVFk.[V]	N-Term(iTRAQ4plex); K8(iTRAQ4	1	1
[R].lPPTTTcQQQk.[E]	N-Term(iTRAQ4plex); C7(Carbam	1	1
[K].eAGIPEFYDYDVALIk.[L]	N-Term(iTRAQ4plex); K16(iTRAQ	1	1
[R].yGLVTYATYPk.[I]	N-Term(iTRAQ4plex); K11(iTRAQ	1	1
[K].eAGIPEFYDYDVALIk.[L]	N-Term(iTRAQ4plex); K16(iTRAQ	1	1
[R].dLLYIGk.[D]	N-Term(iTRAQ4plex); K7(iTRAQ4	1	1
[K].kDNEQHVFk.[V]	N-Term(iTRAQ4plex); K1(iTRAQ4	1	1
[K].vSEADSSNADWVTk.[Q]	N-Term(iTRAQ4plex); K14(iTRAQ	1	1
[K].vSVGGEk.[R]	N-Term(iTRAQ4plex); K7(iTRAQ4	1	1
[K].iSVIRPSk.[G]	N-Term(iTRAQ4plex); K8(iTRAQ4	1	1
[R].STGSWSTLk.[T]	K9(iTRAQ4plex)	1	1
[R].kGTDYHk.[Q]	N-Term(iTRAQ4plex); K1(iTRAQ4	1	1
[R].dLLYIGk.[D]	N-Term(iTRAQ4plex); K7(iTRAQ4	1	1
[K].VASYGVkPR.[Y]	K7(iTRAQ4plex)	1	1
[R].gDSGGPLIVHk.[R]	N-Term(iTRAQ4plex); K11(iTRAQ	1	1
[R].lPPTTTcQQQk.[E]	N-Term(iTRAQ4plex); C7(Carbam	1	1
[K].vSVGGEk.[R]	N-Term(iTRAQ4plex); K7(iTRAQ4	1	1
[K].kDNEQHVFk.[V]	N-Term(iTRAQ4plex); K9(iTRAQ4	1	1
[K].kDNEQHVFk.[V]	K1(iTRAQ4plex); K9(iTRAQ4plex)	1	1
[R].lPPTTTcQQQk.[E]	N-Term(iTRAQ4plex); C7(Carbam	1	1
[K].vASYGVkPR.[Y]	N-Term(iTRAQ4plex); K7(iTRAQ4	1	1
[K].cLVNLIEk.[V]	C1(Carbamidomethyl); K8(iTRAQ	1	1
[R].lPPTTTcQQQk.[E]	N-Term(iTRAQ4plex); C7(Carbam	1	1
[R].dLLYIGk.[D]	N-Term(iTRAQ4plex); K7(iTRAQ4	1	1
[K].qLNEINYEDHk.[L]	N-Term(iTRAQ4plex); K11(iTRAQ	1	1
[K].dISEVVTPR.[F]	N-Term(iTRAQ4plex)	1	1
[K].cLVNLIEk.[V]	N-Term(iTRAQ4plex); C1(Carbam	1	1
[K].aLFVSEEEk.[K]	N-Term(iTRAQ4plex); K9(iTRAQ4	1	1
[K].qLNEINYEDHk.[L]	N-Term(iTRAQ4plex); K11(iTRAQ	1	1
[R].dLLYIGk.[D]	N-Term(iTRAQ4plex); K7(iTRAQ4	1	1
[K].ekLQDEDLGFL.[-]	N-Term(iTRAQ4plex); K2(iTRAQ4	1	1
[K].iSVIRPSk.[G]	N-Term(iTRAQ4plex); K8(iTRAQ4	1	1
[R].lPPTTTcQQQk.[E]	N-Term(iTRAQ4plex); C7(Carbam	1	1
[K].dISEVVTPR.[F]	N-Term(iTRAQ4plex)	1	1
[R].gDSGGPLIVHk.[R]	N-Term(iTRAQ4plex); K11(iTRAQ	1	1
[R].LPPTTTcQQQk.[E]	C7(Carbamidomethyl); K11(iTRA	1	1
[R].lPPTTTcQQQk.[E]	N-Term(iTRAQ4plex); C7(Carbam	1	1
[K].dISEVVTPR.[F]	N-Term(iTRAQ4plex)	1	1
[K].eELLPAQDIk.[A]	N-Term(iTRAQ4plex); K10(iTRAQ	1	1
[K].aLFVSEEEk.[K]	N-Term(iTRAQ4plex); K9(iTRAQ4	1	1
[K].vkDISEVVTPR.[F]	N-Term(iTRAQ4plex); K2(iTRAQ4	1	1
[K].vSVGGEk.[R]	N-Term(iTRAQ4plex); K7(iTRAQ4	1	1
[K].dISEVVTPR.[F]	N-Term(iTRAQ4plex)	1	1
[K].vSEADSSNADWVTk.[Q]	N-Term(iTRAQ4plex); K14(iTRAQ	1	1
[K].TGLLLLSDPDk.[V]	K11(iTRAQ4plex)	1	1
[K].TGLLLLSDPDk.[V]	K11(iTRAQ4plex)	1	1
[R].aNTVQEATFQMELPk.[K]	N-Term(iTRAQ4plex); K15(iTRAQ	1	1
[R].aNTVQEATFQMELPk.[K]	N-Term(iTRAQ4plex); K15(iTRAQ	1	1
[R].aNTVQEATFQMELPk.[K]	N-Term(iTRAQ4plex); K15(iTRAQ	1	1
[R].aNTVQEATFQmELPk.[K]	N-Term(iTRAQ4plex); M11(Oxida	1	1
[R].fkPTLSQQQk.[S]	N-Term(iTRAQ4plex); K2(iTRAQ4	1	1
[R].nVHSGSTFFk.[Y]	N-Term(iTRAQ4plex); K10(iTRAQ	1	1

[R].fkPTLSQQQk.[S]	N-Term(iTRAQ4plex); K2(iTRAQ4	1	1
[R].fKPTLSQQQk.[S]	N-Term(iTRAQ4plex); K10(iTRAQ	1	1
[R].FkPTLSQQQk.[S]	K2(iTRAQ4plex); K10(iTRAQ4ple	1	1
[R].mNFRPGVLSSR.[Q]	N-Term(iTRAQ4plex)	1	1
[R].mNFRPGVLSSR.[Q]	N-Term(iTRAQ4plex)	1	1
[R].nVHSGSTFFk.[Y]	N-Term(iTRAQ4plex); K10(iTRAQ	1	1
[R].nVHSGSTFFk.[Y]	N-Term(iTRAQ4plex); K10(iTRAQ	1	1
[R].fkPTLSQQQk.[S]	N-Term(iTRAQ4plex); K2(iTRAQ4	1	1
[R].fkPTLSQQQk.[S]	N-Term(iTRAQ4plex); K2(iTRAQ4	1	1
[K].vRPQQLVk.[H]	N-Term(iTRAQ4plex); K8(iTRAQ4	1	1
[K].vRPQQLVk.[H]	N-Term(iTRAQ4plex); K8(iTRAQ4	1	1
[K].vRPQQLVk.[H]	N-Term(iTRAQ4plex); K8(iTRAQ4	1	1
[K].iPkPEASFSPR.[R]	N-Term(iTRAQ4plex); K3(iTRAQ4	1	1
[R].mNFRPGVLSSR.[Q]	N-Term(iTRAQ4plex)	1	1
[K].nPLVWVHASPEHVVVTR.[N]	N-Term(iTRAQ4plex)	1	1
[K].nPLVWVHASPEHVVVTR.[N]	N-Term(iTRAQ4plex)	1	1
[R].qGPVNLLSDPEQGVEVTGQYER.[E]	N-Term(iTRAQ4plex)	1	1
[R].qGPVNLLSDPEQGVEVTGQYER.[E]	N-Term(iTRAQ4plex)	1	1
[R].qGPVNLLSDPEQGVEVTGQYER.[E]	N-Term(iTRAQ4plex)	1	1
[K].nPLVWVHASPEHVVVTR.[N]	N-Term(iTRAQ4plex)	1	1
[K].nPLVWVHASPEHVVVTR.[N]	N-Term(iTRAQ4plex)	1	1
[R].QGPVNLLSDPEQGVEVTGQYER.[E]		1	1
[K].nPLVWVHASPEHVVVTR.[N]	N-Term(iTRAQ4plex)	1	1
[K].nPLVWVHASPEHVVVTR.[N]	N-Term(iTRAQ4plex)	1	1
[K].sPEQQETVLDGNLIIR.[Y]	N-Term(iTRAQ4plex)	1	1
[K].sPEQQETVLDGNLIIR.[Y]	N-Term(iTRAQ4plex)	1	1
[K].sPEQQETVLDGNLIIR.[Y]	N-Term(iTRAQ4plex)	1	1
[R].gPDVLTATVSGk.[L]	N-Term(iTRAQ4plex); K12(iTRAQ	1	1
[R].gPDVLTATVSGk.[L]	N-Term(iTRAQ4plex); K12(iTRAQ	1	1
[R].gPDVLTATVSGk.[L]	N-Term(iTRAQ4plex); K12(iTRAQ	1	1
[K].yIFHNFMER.[L]	N-Term(iTRAQ4plex)	1	1
[K].yIFHNFMER.[L]	N-Term(iTRAQ4plex)	1	1
[K].yIFHNFMER.[L]	N-Term(iTRAQ4plex)	1	1
[R].QGPVNLLSDPEQGVEVTGQYER.[E]		1	1
[R].fKPTLSQQQk.[S]	N-Term(iTRAQ4plex); K10(iTRAQ	1	1
[R].FkPTLSQQQk.[S]	K2(iTRAQ4plex); K10(iTRAQ4ple	1	1
[R].FkPTLSQQQk.[S]	K2(iTRAQ4plex); K10(iTRAQ4ple	1	1
[R].fKPTLSQQQk.[S]	N-Term(iTRAQ4plex); K10(iTRAQ	1	1
[R].nVHSGSTFFk.[Y]	N-Term(iTRAQ4plex); K10(iTRAQ	1	1
[R].nVHSGSTFFk.[Y]	N-Term(iTRAQ4plex); K10(iTRAQ	1	1
[K].vRPQQLVk.[H]	N-Term(iTRAQ4plex); K8(iTRAQ4	1	1
[R].qLGLPGPPDVPDHAAYHPFR.[R]	N-Term(iTRAQ4plex)	1	1
[R].qLGLPGPPDVPDHAAYHPFR.[R]	N-Term(iTRAQ4plex)	1	1
[R].qLGLPGPPDVPDHAAYHPFR.[R]	N-Term(iTRAQ4plex)	1	1
[R].qLGLPGPPDVPDHAAYHPFR.[R]	N-Term(iTRAQ4plex)	1	1
[R].qLGLPGPPDVPDHAAYHPFR.[R]	N-Term(iTRAQ4plex)	1	1
[R].qLGLPGPPDVPDHAAYHPFR.[R]	N-Term(iTRAQ4plex)	1	1
[R].qLGLPGPPDVPDHAAYHPFR.[R]	N-Term(iTRAQ4plex)	1	1
[R].QLGLPGPPDVPDHAAYHPFR.[R]		1	1
[R].nMEQFQVSVSVAPNAk.[I]	N-Term(iTRAQ4plex); K16(iTRAQ	1	1
[K].nVVFVIDk.[S]	N-Term(iTRAQ4plex); K8(iTRAQ4	1	1
[K].tGLLLLSDPDk.[V]	N-Term(iTRAQ4plex); K11(iTRAQ	1	1

[K].iTFELVYEELLk.[R]	N-Term(iTRAQ4plex); K12(iTRAQ	1	1
[R].nMEQFQVSVSVAPNAk.[I]	N-Term(iTRAQ4plex); K16(iTRAQ	1	1
[R].nMEQFQVSVSVAPNAk.[I]	N-Term(iTRAQ4plex); K16(iTRAQ	1	1
[K].tGLLLLSDPDk.[V]	N-Term(iTRAQ4plex); K11(iTRAQ	1	1
[K].aEAQAQYSAAVAk.[G]	N-Term(iTRAQ4plex); K13(iTRAQ	1	1
[R].rLDYQEGPPGVEIScWSVEL.[-]	N-Term(iTRAQ4plex); C15(Carbar	1	1
[R].rLDYQEGPPGVEIScWSVEL.[-]	N-Term(iTRAQ4plex); C15(Carbar	1	1
[R].LGVYELLLk.[V]	K9(iTRAQ4plex)	1	1
[R].dQFNLIVFSTEATQWRPSLVPASAENVNk.	N-Term(iTRAQ4plex); K29(iTRAQ	1	1
[R].dQFNLIVFSTEATQWRPSLVPASAENVNk.	N-Term(iTRAQ4plex); K29(iTRAQ	1	1
[K].aGFSWIEVTFk.[N]	N-Term(iTRAQ4plex); K11(iTRAQ	1	1
[K].aEAQAQYSAAVAk.[G]	N-Term(iTRAQ4plex); K13(iTRAQ	1	1
[K].aGFSWIEVTFk.[N]	N-Term(iTRAQ4plex); K11(iTRAQ	1	1
[K].aGFSWIEVTFk.[N]	N-Term(iTRAQ4plex); K11(iTRAQ	1	1
[K].aGFSWIEVTFk.[N]	N-Term(iTRAQ4plex); K11(iTRAQ	1	1
[K].aEAQAQYSAAVAk.[G]	N-Term(iTRAQ4plex); K13(iTRAQ	1	1
[K].ekAEAQAQYSAAVAk.[G]	N-Term(iTRAQ4plex); K2(iTRAQ4	1	1
[K].ekAEAQAQYSAAVAk.[G]	N-Term(iTRAQ4plex); K2(iTRAQ4	1	1
[K].wkETLFSVMPGLk.[M]	N-Term(iTRAQ4plex); K2(iTRAQ4	1	1
[K].aEAQAQYSAAVAk.[G]	N-Term(iTRAQ4plex); K13(iTRAQ	1	1
[K].aEAQAQYSAAVAk.[G]	N-Term(iTRAQ4plex); K13(iTRAQ	1	1
[K].aEAQAQYSAAVAk.[G]	N-Term(iTRAQ4plex); K13(iTRAQ	1	1
[K].ekAEAQAQYSAAVAk.[G]	N-Term(iTRAQ4plex); K2(iTRAQ4	1	1
[K].ekAEAQAQYSAAVAk.[G]	N-Term(iTRAQ4plex); K2(iTRAQ4	1	1
[K].aEAQAQYSAAVAk.[G]	N-Term(iTRAQ4plex); K13(iTRAQ	1	1
[K].aEAQAQYSAAVAk.[G]	N-Term(iTRAQ4plex); K13(iTRAQ	1	1
[K].aGFSWIEVTFk.[N]	N-Term(iTRAQ4plex); K11(iTRAQ	1	1
[R].lGVYELLLk.[V]	N-Term(iTRAQ4plex); K9(iTRAQ4	1	1
[K].gSEMVVAGk.[L]	N-Term(iTRAQ4plex); K9(iTRAQ4	1	1
[K].gSEMVVAGk.[L]	N-Term(iTRAQ4plex); K9(iTRAQ4	1	1
[R].fSSHVGGTLGQFYQEVLWGSPAASDDGR.	N-Term(iTRAQ4plex)	1	1
[R].fSSHVGGTLGQFYQEVLWGSPAASDDGR.	N-Term(iTRAQ4plex)	1	1
[K].eTLFSVMPGLk.[M]	N-Term(iTRAQ4plex); K11(iTRAQ	1	1
[K].eTLFSVMPGLk.[M]	N-Term(iTRAQ4plex); K11(iTRAQ	1	1
[K].aEAQAQYSAAVAk.[G]	N-Term(iTRAQ4plex); K13(iTRAQ	1	1
[K].aEAQAQYSAAVAk.[G]	N-Term(iTRAQ4plex); K13(iTRAQ	1	1
[R].fSSHVGGTLGQFYQEVLWGSPAASDDGR.	N-Term(iTRAQ4plex)	1	1
[K].tGLLLLSDPDkVTIGLLFWDGR.[G]	N-Term(iTRAQ4plex); K11(iTRAQ	1	1
[R].lPEGSVSLIILLTDGDPTVGETNPR.[S]	N-Term(iTRAQ4plex)	1	1
[R].lPEGSVSLIILLTDGDPTVGETNPR.[S]	N-Term(iTRAQ4plex)	1	1
[K].aEAQAQYSAAVAk.[G]	N-Term(iTRAQ4plex); K13(iTRAQ	1	1
[R].lWAYLTIQQLLEQTVSASDADQQALR.[N]	N-Term(iTRAQ4plex)	1	1
[R].vQGNDHSATR.[E]	N-Term(iTRAQ4plex)	1	1
[R].lWAYLTIQQLLEQTVSASDADQQALR.[N]	N-Term(iTRAQ4plex)	1	1
[R].fSSHVGGTLGQFYQEVLWGSPAASDDGR.	N-Term(iTRAQ4plex)	1	1
[K].nVVFVIDk.[S]	N-Term(iTRAQ4plex); K8(iTRAQ4	1	1
[K].yIFHNFMER.[L]	N-Term(iTRAQ4plex)	1	1
[R].nVHSGSTFFk.[Y]	N-Term(iTRAQ4plex); K10(iTRAQ	1	1
[K].vRPQQLVk.[H]	N-Term(iTRAQ4plex); K8(iTRAQ4	1	1
[R].fKPTLSQQQk.[S]	N-Term(iTRAQ4plex); K10(iTRAQ	1	1
[R].nVHSGSTFFk.[Y]	N-Term(iTRAQ4plex); K10(iTRAQ	1	1
[K].aGFSWIEVTFk.[N]	N-Term(iTRAQ4plex); K11(iTRAQ	1	1

[K].nPLVWVHASPEHVVVTR.[N]	N-Term(iTRAQ4plex)	1	1
[R].MNFRPGVLSSR.[Q]		1	1
[K].NVVFVIDk.[S]	K8(iTRAQ4plex)	1	1
[K].tGLLLLSDPDk.[V]	N-Term(iTRAQ4plex); K11(iTRAQ	1	1
[K].iLDDLSPR.[D]	N-Term(iTRAQ4plex)	1	1
[R].FkPTLSQQQk.[S]	K2(iTRAQ4plex); K10(iTRAQ4ple	1	1
[K].eTLFSVMPGLk.[M]	N-Term(iTRAQ4plex); K11(iTRAQ	1	1
[R].mNFRPGVLSSR.[Q]	N-Term(iTRAQ4plex)	1	1
[R].fkPTLSQQQk.[S]	N-Term(iTRAQ4plex); K2(iTRAQ4	1	1
[K].gSEMVVAGk.[L]	N-Term(iTRAQ4plex); K9(iTRAQ4	1	1
[K].iTFELVYEELLk.[R]	N-Term(iTRAQ4plex); K12(iTRAQ	1	1
[K].gSEmVVAGk.[L]	N-Term(iTRAQ4plex); M4(Oxidat	1	1
[K].NPLVWVHASPEHVVVTR.[N]		1	1
[K].yIFHNFMER.[L]	N-Term(iTRAQ4plex)	1	1
[K].eTLFSVmPGLk.[M]	N-Term(iTRAQ4plex); M7(Oxidat	1	1
[R].mNFRPGVLSSR.[Q]	N-Term(iTRAQ4plex); M1(Oxidat	1	1
[K].TGLLLLSDPDk.[V]	K11(iTRAQ4plex)	1	1
[R].FAHTVVTSR.[V]		1	1
[K].IPkPEASFSPR.[R]	K3(iTRAQ4plex)	1	1
[R].gPDVLTATVSGk.[L]	N-Term(iTRAQ4plex); K12(iTRAQ	1	1
[K].eTLFSVMPGLk.[M]	N-Term(iTRAQ4plex); K11(iTRAQ	1	1
[K].NVVFVIDk.[S]	K8(iTRAQ4plex)	1	1
[R].fkPTLSQQQk.[S]	N-Term(iTRAQ4plex); K2(iTRAQ4	1	1
[R].fKPTLSQQQk.[S]	N-Term(iTRAQ4plex); K10(iTRAQ	1	1
[K].TGLLLLSDPDk.[V]	K11(iTRAQ4plex)	1	1
[K].sPEQQETVLDGNLIIR.[Y]	N-Term(iTRAQ4plex)	1	1
[K].gkSAGLVk.[A]	N-Term(iTRAQ4plex); K2(iTRAQ4	1	1
[K].aEAQAQYSAAVAk.[G]	N-Term(iTRAQ4plex); K13(iTRAQ	1	1
[R].mNFRPGVLSSR.[Q]	N-Term(iTRAQ4plex)	1	1
[K].AEAQAQYSAAVAk.[G]	K13(iTRAQ4plex)	1	1
[K].gSEmVVAGk.[L]	N-Term(iTRAQ4plex); M4(Oxidat	1	1
[R].qLGLPGPPDVPDHAAYHPFR.[R]	N-Term(iTRAQ4plex)	1	1
[K].sAGLVkATGR.[N]	N-Term(iTRAQ4plex); K6(iTRAQ4	1	1
[K].NPLVWVHASPEHVVVTR.[N]		1	1
[R].FAHTVVTSR.[V]		1	1
[K].tGLLLLSDPDk.[V]	N-Term(iTRAQ4plex); K11(iTRAQ	1	1
[R].lGVYELLLk.[V]	N-Term(iTRAQ4plex); K9(iTRAQ4	1	1
[K].vRPQQLVk.[H]	N-Term(iTRAQ4plex); K8(iTRAQ4	1	1
[K].iLDDLSPR.[D]	N-Term(iTRAQ4plex)	1	1
[K].nVVFVIDk.[S]	N-Term(iTRAQ4plex); K8(iTRAQ4	1	1
[R].qGPVNLLSDPEQGVEVTGQYER.[E]	N-Term(iTRAQ4plex)	1	1
[R].mNFRPGVLSSR.[Q]	N-Term(iTRAQ4plex)	1	1
[R].FAHTVVTSR.[V]		1	1
[K].eTLFSVMPGLk.[M]	N-Term(iTRAQ4plex); K11(iTRAQ	1	1
[K].iPkPEASFSPR.[R]	N-Term(iTRAQ4plex); K3(iTRAQ4	1	1
[K].yIFHNFmER.[L]	N-Term(iTRAQ4plex); M7(Oxidat	1	1
[R].fAHTVVTSR.[V]	N-Term(iTRAQ4plex)	1	1
[K].iPkPEASFSPR.[R]	N-Term(iTRAQ4plex); K3(iTRAQ4	1	1
[K].ILDDLSPR.[D]		1	1
[K].aEAQAQYSAAVAk.[G]	N-Term(iTRAQ4plex); K13(iTRAQ	1	1
[R].fkPTLSQQQk.[S]	N-Term(iTRAQ4plex); K2(iTRAQ4	1	1
[R].fSSHVGGTLGQFYQEVLWGSPAASDDGR.	N-Term(iTRAQ4plex)	1	1

[K].eTLFSVmPGLk.[M]	N-Term(iTRAQ4plex); M7(Oxidat	1	1
[R].fAHTVVTSR.[V]	N-Term(iTRAQ4plex)	1	1
[R].qLGLPGPPDVPDHAAYHPFR.[R]	N-Term(iTRAQ4plex)	1	1
[K].aGFSWIEVTFk.[N]	N-Term(iTRAQ4plex); K11(iTRAQ	1	1
[R].fAHTVVTSR.[V]	N-Term(iTRAQ4plex)	1	1
[K].iLDDLSPR.[D]	N-Term(iTRAQ4plex)	1	1
[K].aEAQAQYSAAVAk.[G]	N-Term(iTRAQ4plex); K13(iTRAQ	1	1
[K].nPLVWVHASPEHVVVTR.[N]	N-Term(iTRAQ4plex)	1	1
[R].nmEQFQVSVSVAPNAk.[I]	N-Term(iTRAQ4plex); M2(Oxidat	1	1
[K].yYLQGAk.[I]	N-Term(iTRAQ4plex); K7(iTRAQ4	1	1
[K].YIFHNFMER.[L]		1	1
[K].yIFHNFMER.[L]	N-Term(iTRAQ4plex)	1	1
[K].yYLQGAk.[I]	N-Term(iTRAQ4plex); K7(iTRAQ4	1	1
[R].MNFRPGVLSSR.[Q]		1	1
[K].TGLLLLSDPDk.[V]	K11(iTRAQ4plex)	1	1
[R].lGVYELLLk.[V]	N-Term(iTRAQ4plex); K9(iTRAQ4	1	1
[R].rLGVYELLLk.[V]	N-Term(iTRAQ4plex); K10(iTRAQ	1	1
[R].fkPTLSQQQk.[S]	N-Term(iTRAQ4plex); K2(iTRAQ4	1	1
[K].eTLFSVMPGLk.[M]	N-Term(iTRAQ4plex); K11(iTRAQ	1	1
[K].ILDDLSPR.[D]		1	1
[R].fkPTLSQQQk.[S]	N-Term(iTRAQ4plex); K2(iTRAQ4	1	1
[K].iLDDLSPR.[D]	N-Term(iTRAQ4plex)	1	1
[R].nMEQFQVSVSVAPNAk.[I]	N-Term(iTRAQ4plex); K16(iTRAQ	1	1
[R].fkPTLSQQQk.[S]	N-Term(iTRAQ4plex); K2(iTRAQ4	1	1
[K].VRPQQLVk.[H]	K8(iTRAQ4plex)	1	1
[R].mNFRPGVLSSR.[Q]	N-Term(iTRAQ4plex)	1	1
[R].mNFRPGVLSSR.[Q]	N-Term(iTRAQ4plex); M1(Oxidat	1	1
[K].yIFHNFmER.[L]	N-Term(iTRAQ4plex); M7(Oxidat	1	1
[R].fkPTLSQQQk.[S]	N-Term(iTRAQ4plex); K2(iTRAQ4	1	1
[R].fAHTVVTSR.[V]	N-Term(iTRAQ4plex)	1	1
[R].gPDVLTATVSGk.[L]	N-Term(iTRAQ4plex); K12(iTRAQ	1	1
[R].FAHTVVTSR.[V]		1	1
[K].yIFHNFmER.[L]	N-Term(iTRAQ4plex); M7(Oxidat	1	1
[K].vRPQQLVk.[H]	N-Term(iTRAQ4plex); K8(iTRAQ4	1	1
[K].vRPQQLVk.[H]	N-Term(iTRAQ4plex); K8(iTRAQ4	1	1
[R].fAHTVVTSR.[V]	N-Term(iTRAQ4plex)	1	1
[K].aEAQAQYSAAVAk.[G]	N-Term(iTRAQ4plex); K13(iTRAQ	1	1
[K].yIFHNFmER.[L]	N-Term(iTRAQ4plex); M7(Oxidat	1	1
[R].mNFRPGVLSSR.[Q]	N-Term(iTRAQ4plex); M1(Oxidat	1	1
[K].aGFSWIEVTFk.[N]	N-Term(iTRAQ4plex); K11(iTRAQ	1	1
[R].mNFRPGVLSSR.[Q]	N-Term(iTRAQ4plex); M1(Oxidat	1	1
[R].fkPTLSQQQK.[S]	N-Term(iTRAQ4plex); K2(iTRAQ4	1	1
[R].fAHTVVTSR.[V]	N-Term(iTRAQ4plex)	1	1
[K].YIFHNFMER.[L]		1	1
[R].FAHTVVTSR.[V]		1	1
[K].sPEQQETVLDGNLIIR.[Y]	N-Term(iTRAQ4plex)	1	1
[K].AEAQAQYSAAVAk.[G]	K13(iTRAQ4plex)	1	1
[K].nVVFVIDk.[S]	N-Term(iTRAQ4plex); K8(iTRAQ4	1	1
[K].iLDDLSPR.[D]	N-Term(iTRAQ4plex)	1	1
[R].eIMENYNIALR.[W]	N-Term(iTRAQ4plex)	2	2
[R].eIMENYNIALR.[W]	N-Term(iTRAQ4plex)	2	2
[K].sSNLIILEEHLk.[N]	N-Term(iTRAQ4plex); K12(iTRAQ	1	1

[K].sSNLIILEEHLk.[N]	N-Term(iTRAQ4plex); K12(iTRAQ4	1	1
[K].sSNLIILEEHLk.[N]	N-Term(iTRAQ4plex); K12(iTRAQ4	1	1
[K].sSNLIILEEHLk.[N]	N-Term(iTRAQ4plex); K12(iTRAQ4	1	1
[K].sSNLIILEEHLk.[N]	N-Term(iTRAQ4plex); K12(iTRAQ4	1	1
[K].wSSPPQcEGLPck.[S]	N-Term(iTRAQ4plex); C7(Carbam	1	1
[K].wSHPPScIk.[T]	N-Term(iTRAQ4plex); C7(Carbam	1	1
[K].wSHPPScIk.[T]	N-Term(iTRAQ4plex); C7(Carbam	1	1
[K].wSHPPScIk.[T]	N-Term(iTRAQ4plex); C7(Carbam	1	1
[K].wSHPPScIk.[T]	N-Term(iTRAQ4plex); C7(Carbam	1	1
[R].nTEILTGSWSDQTYPEGTQAIYk.[C]	N-Term(iTRAQ4plex); K23(iTRAQ4	1	1
[R].nTEILTGSWSDQTYPEGTQAIYk.[C]	N-Term(iTRAQ4plex); K23(iTRAQ4	1	1
[R].nTEILTGSWSDQTYPEGTQAIYk.[C]	N-Term(iTRAQ4plex); K23(iTRAQ4	1	1
[R].ecDTDGWTNDIPIcEVVk.[C]	N-Term(iTRAQ4plex); C2(Carbam	1	1
[R].ecDTDGWTNDIPIcEVVk.[C]	N-Term(iTRAQ4plex); C2(Carbam	1	1
[K].cTSTGWIPAPR.[C]	C1(Carbamidomethyl)	1	1
[K].iDVHLVPDR.[K]	N-Term(iTRAQ4plex)	1	1
[K].iDVHLVPDR.[K]	N-Term(iTRAQ4plex)	1	1
[K].sIDVAcHPGYALPk.[A]	N-Term(iTRAQ4plex); C6(Carbam	1	1
[K].sIDVAcHPGYALPk.[A]	N-Term(iTRAQ4plex); C6(Carbam	1	1
[K].sIDVAcHPGYALPk.[A]	N-Term(iTRAQ4plex); C6(Carbam	1	1
[K].vSVLcQENYLIQEGEEITck.[D]	N-Term(iTRAQ4plex); C5(Carbam	1	1
[K].vSVLcQENYLIQEGEEITck.[D]	N-Term(iTRAQ4plex); C5(Carbam	1	1
[K].vSVLcQENYLIQEGEEITck.[D]	N-Term(iTRAQ4plex); C5(Carbam	1	1
[K].vSVLcQENYLIQEGEEITck.[D]	N-Term(iTRAQ4plex); C5(Carbam	1	1
[R].kGEWVALNPLR.[K]	N-Term(iTRAQ4plex); K1(iTRAQ4	1	1
[R].kGEWVALNPLR.[K]	N-Term(iTRAQ4plex); K1(iTRAQ4	1	1
[R].kGEWVALNPLR.[K]	N-Term(iTRAQ4plex); K1(iTRAQ4	1	1
[R].rPYFPVAVGk.[Y]	N-Term(iTRAQ4plex); K10(iTRAQ4	2	2
[R].rPYFPVAVGk.[Y]	N-Term(iTRAQ4plex); K10(iTRAQ4	2	2
[R].rPYFPVAVGk.[Y]	N-Term(iTRAQ4plex); K10(iTRAQ4	2	2
[R].rPYFPVAVGk.[Y]	N-Term(iTRAQ4plex); K10(iTRAQ4	2	2
[K].wSHPPScIk.[T]	N-Term(iTRAQ4plex); C7(Carbam	1	1
[R].wQSIPLcVEk.[I]	N-Term(iTRAQ4plex); C7(Carbam	1	1
[R].wQSIPLcVEk.[I]	N-Term(iTRAQ4plex); C7(Carbam	1	1
[R].wQSIPLcVEk.[I]	N-Term(iTRAQ4plex); C7(Carbam	1	1
[K].scDIPVFMNAR.[T]	N-Term(iTRAQ4plex); C2(Carbam	1	1
[K].cTSTGWIPAPR.[C]	N-Term(iTRAQ4plex); C1(Carbam	1	1
[K].cTSTGWIPAPR.[C]	N-Term(iTRAQ4plex); C1(Carbam	1	1
[K].tDcLSLPSFENAIPMGEk.[K]	N-Term(iTRAQ4plex); C3(Carbam	1	1
[K].aVYTcNEGYQLLGEINYR.[E]	N-Term(iTRAQ4plex); C5(Carbam	1	1
[K].iEGDEEMHcSDDGFWSk.[E]	N-Term(iTRAQ4plex); C9(Carbam	1	1
[K].eEYGHSEVVEYYcNPR.[F]	N-Term(iTRAQ4plex); C13(Carbar	1	1
[R].gDAVcTESGWRPLPScEEk.[S]	N-Term(iTRAQ4plex); C5(Carbam	1	1
[R].gDAVcTESGWRPLPScEEk.[S]	N-Term(iTRAQ4plex); C5(Carbam	1	1
[R].sITcIHGVWTQLPQcVAIDk.[L]	N-Term(iTRAQ4plex); C4(Carbam	1	1
[K].tkEEYGHSEVVEYYcNPR.[F]	N-Term(iTRAQ4plex); K2(iTRAQ4	1	1
[K].hGGLYHENMR.[R]	N-Term(iTRAQ4plex)	1	1
[R].tGESVEFVck.[R]	N-Term(iTRAQ4plex); C9(Carbam	1	1
[R].tGESVEFVck.[R]	N-Term(iTRAQ4plex); C9(Carbam	1	1
[K].tkEEYGHSEVVEYYcNPR.[F]	N-Term(iTRAQ4plex); K2(iTRAQ4	1	1
[K].dGWSAQPTcIk.[S]	N-Term(iTRAQ4plex); C9(Carbam	1	1
[K].dGWSAQPTcIk.[S]	N-Term(iTRAQ4plex); C9(Carbam	1	1

[K].aGEQVTYTcATYYk.[M]	N-Term(iTRAQ4plex); C9(Carbam	1	1
[R].TGDEITYQcR.[N]	C9(Carbamidomethyl)	1	1
[R].TGDEITYQcR.[N]	C9(Carbamidomethyl)	1	1
[R].tGDEITYQcR.[N]	N-Term(iTRAQ4plex); C9(Carbam	1	1
[R].tGDEITYQcR.[N]	N-Term(iTRAQ4plex); C9(Carbam	1	1
[R].sSQESYAHGTk.[L]	N-Term(iTRAQ4plex); K11(iTRAQ	1	1
[R].sSQESYAHGTk.[L]	N-Term(iTRAQ4plex); K11(iTRAQ	1	1
[K].lGYVTADGETSGSITcGk.[D]	N-Term(iTRAQ4plex); C16(Carbar	1	1
[R].gDAVcTESGWRPLPScEEk.[S]	N-Term(iTRAQ4plex); C5(Carbam	1	1
[R].sSQESYAHGTk.[L]	N-Term(iTRAQ4plex); K11(iTRAQ	1	1
[R].dTScVNPPTVQNAYIVSR.[Q]	N-Term(iTRAQ4plex); C4(Carbam	1	1
[R].sSQESYAHGTk.[L]	N-Term(iTRAQ4plex); K11(iTRAQ	1	1
[K].vSVLcQENYLIQEGEEITck.[D]	N-Term(iTRAQ4plex); C5(Carbam	1	1
[R].TGESVEFVck.[R]	C9(Carbamidomethyl); K10(iTRA	1	1
[K].nDFTWFk.[L]	N-Term(iTRAQ4plex); K7(iTRAQ4	1	1
[R].sSQESYAHGTk.[L]	N-Term(iTRAQ4plex); K11(iTRAQ	1	1
[R].cTLkPcDYPDIk.[H]	N-Term(iTRAQ4plex); C1(Carbam	1	1
[K].lSYTcEGGFR.[I]	N-Term(iTRAQ4plex); C5(Carbam	1	1
[R].sLGNVImVcR.[K]	N-Term(iTRAQ4plex); M7(Oxidat	1	1
[R].tGDEITYQcR.[N]	N-Term(iTRAQ4plex); C9(Carbam	1	1
[R].EIMENYNIALR.[W]		2	2
[K].IVSSAMEPDR.[E]		1	1
[K].lGYVTADGETSGSITcGk.[D]	N-Term(iTRAQ4plex); C16(Carbar	1	1
[K].wSHPPScIk.[T]	N-Term(iTRAQ4plex); C7(Carbam	1	1
[R].sSQESYAHGTk.[L]	N-Term(iTRAQ4plex); K11(iTRAQ	1	1
[K].vSVLcQENYLIQEGEEITck.[D]	N-Term(iTRAQ4plex); C5(Carbam	1	1
[R].fVcNSGYk.[I]	N-Term(iTRAQ4plex); C3(Carbam	1	1
[K].wSHPPScIk.[T]	N-Term(iTRAQ4plex); C7(Carbam	1	1
[K].iVSSAMEPDR.[E]	N-Term(iTRAQ4plex)	1	1
[K].nDFTWFk.[L]	N-Term(iTRAQ4plex); K7(iTRAQ4	1	1
[R].cTLkPcDYPDIk.[H]	N-Term(iTRAQ4plex); C1(Carbam	1	1
[K].aGEQVTYTcATYYk.[M]	N-Term(iTRAQ4plex); C9(Carbam	1	1
[K].LSYTcEGGFR.[I]	C5(Carbamidomethyl)	1	1
[K].eFDHNSNIR.[Y]	N-Term(iTRAQ4plex)	1	1
[K].iDVHLVPDR.[K]	N-Term(iTRAQ4plex)	1	1
[R].sLGNVIMVcR.[K]	N-Term(iTRAQ4plex); C9(Carbam	1	1
[R].kGEWVALNPLR.[K]	N-Term(iTRAQ4plex)	1	1
[R].kGEWVALNPLR.[K]	K1(iTRAQ4plex)	1	1
[K].scDIPVFMNAR.[T]	N-Term(iTRAQ4plex); C2(Carbam	1	1
[K].lSYTcEGGFR.[I]	N-Term(iTRAQ4plex); C5(Carbam	1	1
[R].fVcNSGYk.[I]	N-Term(iTRAQ4plex); C3(Carbam	1	1
[K].eEYGHSEVVEYYcNPR.[F]	N-Term(iTRAQ4plex); C13(Carbar	1	1
[R].sLGNVIMVcR.[K]	N-Term(iTRAQ4plex); C9(Carbam	1	1
[R].gDAVcTESGWRPLPScEEk.[S]	N-Term(iTRAQ4plex); C5(Carbam	1	1
[R].nTEILTGSWSDQTYPEGTQAIYk.[C]	N-Term(iTRAQ4plex); K23(iTRAQ	1	1
[R].tGESVEFVck.[R]	N-Term(iTRAQ4plex); C9(Carbam	1	1
[K].hGGLYHENmR.[R]	N-Term(iTRAQ4plex); M9(Oxidat	1	1
[K].eFDHNSNIR.[Y]	N-Term(iTRAQ4plex)	1	1
[R].eImENYNIALR.[W]	N-Term(iTRAQ4plex); M3(Oxidat	2	2
[K].cLHPcVISR.[E]	N-Term(iTRAQ4plex); C1(Carbam	2	2
[K].vSVLcQENYLIQEGEEITck.[D]	N-Term(iTRAQ4plex); C5(Carbam	1	1
[R].nTEILTGSWSDQTYPEGTQAIYk.[C]	N-Term(iTRAQ4plex); K23(iTRAQ	1	1

[R].FVcNSGYk.[I]	C3(Carbamidomethyl); K8(iTRAC	1	1
[K].iVSSAMEPDREYHFGQAVR.[F]	N-Term(iTRAQ4plex)	1	1
[K].cVEISck.[S]	N-Term(iTRAQ4plex); C1(Carbam	1	1
[R].SITcIHGVWTQLPQcVAIDk.[L]	C4(Carbamidomethyl); C15(Carb	1	1
[R].SSQESYAHGTk.[L]	K11(iTRAQ4plex)	1	1
[R].sITcIHGVWTQLPQcVAIDk.[L]	N-Term(iTRAQ4plex); C4(Carbam	1	1
[R].kGEWVALNPLR.[K]	N-Term(iTRAQ4plex); K1(iTRAQ4	1	1
[R].wTGRPTcR.[D]	N-Term(iTRAQ4plex); C7(Carbam	1	1
[R].wTGRPTcR.[D]	N-Term(iTRAQ4plex); C7(Carbam	1	1
[R].TGDEITYQcR.[N]	C9(Carbamidomethyl)	1	1
[K].wSSPPQcEGLPck.[S]	N-Term(iTRAQ4plex); C7(Carbam	1	1
[K].hGGLYHENmR.[R]	N-Term(iTRAQ4plex); M9(Oxidat	1	1
[R].ecDTDGWTNDIPIcEVVk.[C]	N-Term(iTRAQ4plex); C2(Carbam	1	1
[K].vSVLcQENYLIQEGEEITck.[D]	N-Term(iTRAQ4plex); C5(Carbam	1	1
[K].tkEEYGHSEVVEYYcNPR.[F]	N-Term(iTRAQ4plex); K2(iTRAQ4	1	1
[R].eIMENYNIALR.[W]	N-Term(iTRAQ4plex)	2	2
[K].sSNLIILEEHLk.[N]	N-Term(iTRAQ4plex); K12(iTRAQ	1	1
[K].sSNLIILEEHLk.[N]	N-Term(iTRAQ4plex); K12(iTRAQ	1	1
[R].fDEFFSEGcAPGSk.[K]	N-Term(iTRAQ4plex); C9(Carbam	1	1
[R].fDEFFSEGcAPGSk.[K]	N-Term(iTRAQ4plex); C9(Carbam	1	1
[R].fDEFFSEGcAPGSk.[K]	N-Term(iTRAQ4plex); C9(Carbam	1	1
[K].wcALSHHER.[L]	N-Term(iTRAQ4plex); C2(Carbam	1	1
[R].dQYELLcLDNTR.[K]	N-Term(iTRAQ4plex); C7(Carbam	1	1
[K].dYELLcLDGTR.[K]	N-Term(iTRAQ4plex); C6(Carbam	1	1
[K].kDSGFQmNQLR.[G]	N-Term(iTRAQ4plex); K1(iTRAQ4	1	1
[K].wcALSHHER.[L]	N-Term(iTRAQ4plex); C2(Carbam	1	1
[K].dcHLAQVPSHTVVAR.[S]	N-Term(iTRAQ4plex); C2(Carbam	1	1
[K].kDSGFQMNQLR.[G]	N-Term(iTRAQ4plex); K1(iTRAQ4	1	1
[K].hSTIFENLANk.[A]	N-Term(iTRAQ4plex); K11(iTRAQ	1	1
[K].hSTIFENLANk.[A]	N-Term(iTRAQ4plex); K11(iTRAQ	1	1
[K].mYLGYEYVTAIR.[N]	N-Term(iTRAQ4plex)	1	1
[K].mYLGYEYVTAIR.[N]	N-Term(iTRAQ4plex)	1	1
[K].cSTSSLLEAcTFR.[R]	N-Term(iTRAQ4plex); C1(Carbam	1	1
[K].eFQLFSSPHGk.[D]	N-Term(iTRAQ4plex); K11(iTRAQ	1	1
[K].eFQLFSSPHGk.[D]	N-Term(iTRAQ4plex); K11(iTRAQ	1	1
[K].hSTIFENLANk.[A]	N-Term(iTRAQ4plex); K11(iTRAQ	1	1
[K].eDPQTFYYAVAVVk.[K]	N-Term(iTRAQ4plex); K14(iTRAQ	1	1
[K].eDPQTFYYAVAVVk.[K]	N-Term(iTRAQ4plex); K14(iTRAQ	1	1
[K].cDEWSVNSVGk.[I]	N-Term(iTRAQ4plex); C1(Carbam	1	1
[R].dDTVcLAk.[L]	N-Term(iTRAQ4plex); C5(Carbam	1	1
[R].wcAVSEHEATk.[C]	N-Term(iTRAQ4plex); C2(Carbam	1	1
[R].tAGWNIPMGLLYNk.[I]	N-Term(iTRAQ4plex); K14(iTRAQ	1	1
[R].tAGWNIPMGLLYNk.[I]	N-Term(iTRAQ4plex); K14(iTRAQ	1	1
[K].dGAGDVAFVk.[H]	N-Term(iTRAQ4plex); K10(iTRAQ	1	1
[K].yLGEEYVk.[A]	N-Term(iTRAQ4plex); K8(iTRAQ4	1	1
[K].aDRDQYELLcLDNTR.[K]	N-Term(iTRAQ4plex); C10(Carbar	1	1
[K].eDLIWELLNQAQEHFGk.[D]	N-Term(iTRAQ4plex); K17(iTRAQ	1	1
[K].aDRDQYELLcLDNTR.[K]	N-Term(iTRAQ4plex); C10(Carbar	1	1
[K].sVIPSDGPSVAcVk.[K]	N-Term(iTRAQ4plex); C12(Carbar	1	1
[K].eGYYGYTGAFR.[C]	N-Term(iTRAQ4plex)	1	1
[K].sASDLTWDNLk.[G]	N-Term(iTRAQ4plex); K11(iTRAQ	1	1
[K].sASDLTWDNLk.[G]	N-Term(iTRAQ4plex); K11(iTRAQ	1	1

[R].kPVEEYANcHLAR.[A]	N-Term(iTRAQ4plex); K1(iTRAQ4	1	1
[K].dcHLAQVPSHTVVAR.[S]	N-Term(iTRAQ4plex); C2(Carbam	1	1
[K].dcHLAQVPSHTVVAR.[S]	N-Term(iTRAQ4plex); C2(Carbam	1	1
[R].wcAVSEHEATk.[C]	N-Term(iTRAQ4plex); C2(Carbam	1	1
[R].kPVEEYANcHLAR.[A]	N-Term(iTRAQ4plex); K1(iTRAQ4	1	1
[R].kPVEEYANcHLAR.[A]	N-Term(iTRAQ4plex); K1(iTRAQ4	1	1
[R].eGTcPEAPTDEckPVk.[W]	N-Term(iTRAQ4plex); C4(Carbam	1	1
[R].eGTcPEAPTDEckPVk.[W]	N-Term(iTRAQ4plex); C4(Carbam	1	1
[R].eGTcPEAPTDEckPVk.[W]	N-Term(iTRAQ4plex); C4(Carbam	1	1
[R].eGTcPEAPTDEckPVk.[W]	N-Term(iTRAQ4plex); C4(Carbam	1	1
[R].eGTcPEAPTDEckPVk.[W]	N-Term(iTRAQ4plex); C4(Carbam	1	1
[R].eGTcPEAPTDEckPVk.[W]	N-Term(iTRAQ4plex); C4(Carbam	1	1
[R].eGTcPEAPTDEckPVk.[W]	N-Term(iTRAQ4plex); C4(Carbam	1	1
[K].dSAHGFLk.[V]	N-Term(iTRAQ4plex); K8(iTRAQ4	1	1
[R].eGTcPEAPTDEckPVk.[W]	N-Term(iTRAQ4plex); C4(Carbam	1	1
[R].eGTcPEAPTDEckPVk.[W]	N-Term(iTRAQ4plex); C4(Carbam	1	1
[R].eGTcPEAPTDEckPVk.[W]	N-Term(iTRAQ4plex); C4(Carbam	1	1
[R].sAGWNIPIGLLYcDLPEPR.[K]	N-Term(iTRAQ4plex); C13(Carbar	1	1
[R].sAGWNIPIGLLYcDLPEPR.[K]	N-Term(iTRAQ4plex); C13(Carbar	1	1
[R].eGTcPEAPTDEckPVk.[W]	N-Term(iTRAQ4plex); C4(Carbam	1	1
[K].hQTVPQNTGGk.[N]	N-Term(iTRAQ4plex); K11(iTRAQ	1	1
[K].dcHLAQVPSHTVVAR.[S]	N-Term(iTRAQ4plex); C2(Carbam	1	1
[R].eGTcPEAPTDEckPVk.[W]	N-Term(iTRAQ4plex); C4(Carbam	1	1
[R].eGTcPEAPTDEckPVk.[W]	N-Term(iTRAQ4plex); C4(Carbam	1	1
[K].hQTVPQNTGGk.[N]	N-Term(iTRAQ4plex); K11(iTRAQ	1	1
[R].eGTcPEAPTDEckPVk.[W]	N-Term(iTRAQ4plex); C4(Carbam	1	1
[K].dkEAcVHk.[I]	N-Term(iTRAQ4plex); K2(iTRAQ4	1	1
[K].hQTVPQNTGGk.[N]	N-Term(iTRAQ4plex); K11(iTRAQ	1	1
[K].wcALSHHER.[L]	N-Term(iTRAQ4plex); C2(Carbam	1	1
[R].fDEFFSEGcAPGSk.[K]	N-Term(iTRAQ4plex); C9(Carbam	1	1
[K].WcALSHHER.[L]	C2(Carbamidomethyl)	1	1
[K].wcALSHHER.[L]	N-Term(iTRAQ4plex); C2(Carbam	1	1
[K].aSYLDcIR.[A]	N-Term(iTRAQ4plex); C6(Carbam	1	1
[K].hQTVPQNTGGk.[N]	N-Term(iTRAQ4plex); K11(iTRAQ	1	1
[R].aPNHAVVTR.[K]	N-Term(iTRAQ4plex)	1	1
[K].hQTVPQNTGGk.[N]	N-Term(iTRAQ4plex); K11(iTRAQ	1	1
[K].hSTIFENLANk.[A]	N-Term(iTRAQ4plex); K11(iTRAQ	1	1
[K].scHTGLGR.[S]	N-Term(iTRAQ4plex); C2(Carbam	1	1
[R].DQYELLcLDNTR.[K]	C7(Carbamidomethyl)	1	1
[R].wcAVSEHEATk.[C]	N-Term(iTRAQ4plex); C2(Carbam	1	1
[K].sVIPSDGPSVAcVk.[K]	N-Term(iTRAQ4plex); C12(Carbar	1	1
[R].dDTVcLAk.[L]	N-Term(iTRAQ4plex); C5(Carbam	1	1
[K].kDSSLck.[L]	N-Term(iTRAQ4plex); K1(iTRAQ4	1	1
[K].hQTVPQNTGGk.[N]	N-Term(iTRAQ4plex); K11(iTRAQ	1	1
[K].HSTIFENLANk.[A]	K11(iTRAQ4plex)	1	1
[K].kSASDLTWDNLk.[G]	N-Term(iTRAQ4plex); K1(iTRAQ4	1	1
[K].DGAGDVAFVk.[H]	K10(iTRAQ4plex)	1	1
[K].yLGEEYVk.[A]	N-Term(iTRAQ4plex); K8(iTRAQ4	1	1
[K].eFQLFSSPHGk.[D]	N-Term(iTRAQ4plex); K11(iTRAQ	1	1
[K].kASYLDcIR.[A]	N-Term(iTRAQ4plex); K1(iTRAQ4	1	1
[R].dDTVcLAk.[L]	N-Term(iTRAQ4plex); C5(Carbam	1	1
[K].iEcVSAETTEDcIAk.[I]	N-Term(iTRAQ4plex); C3(Carbam	1	1

[R].dDTVcLAk.[L]	N-Term(iTRAQ4plex); C5(Carbam	1	1
[K].MYLGYEYVTAIR.[N]		1	1
[AK].gDVAFVk.[H]	N-Term(iTRAQ4plex); K7(iTRAQ4	1	1
[K].DYELLcLDGTR.[K]	C6(Carbamidomethyl)	1	1
[R].DDTVcLAk.[L]	C5(Carbamidomethyl); K8(iTRAC	1	1
[K].mYLGYEYVTAIR.[N]	N-Term(iTRAQ4plex)	1	1
[K].dSGFQMNQLR.[G]	N-Term(iTRAQ4plex)	1	1
[AK].gDVAFVk.[H]	N-Term(iTRAQ4plex); K7(iTRAQ4	1	1
[K].iEcVSAETTEDcIAk.[I]	N-Term(iTRAQ4plex); C3(Carbam	1	1
[K].hQTVPQNTGGk.[N]	N-Term(iTRAQ4plex); K11(iTRAQ	1	1
[K].hSTIFENLANk.[A]	N-Term(iTRAQ4plex); K11(iTRAQ	1	1
[K].aDRDQYELLcLDNTR.[K]	N-Term(iTRAQ4plex); C10(Carbar	1	1
[R].EGTcPEAPTDEckPVk.[W]	C4(Carbamidomethyl); C12(Carb	1	1
[AK].gDVAFVk.[H]	N-Term(iTRAQ4plex); K7(iTRAQ4	1	1
[R].wcAVSEHEATk.[C]	N-Term(iTRAQ4plex); C2(Carbam	1	1
[K].dGAGDVAFVk.[H]	N-Term(iTRAQ4plex); K10(iTRAQ	1	1
[R].sSPVVIDASTAIDAPSNLR.[F]	N-Term(iTRAQ4plex)	1	1
[R].eESPLLIGQQSTVSDVPR.[D]	N-Term(iTRAQ4plex)	1	1
[R].dLQFVEVTDVk.[V]	N-Term(iTRAQ4plex); K11(iTRAQ	1	1
[R].vPGTSTSATLTGLTR.[G]	N-Term(iTRAQ4plex)	1	1
[K].eINLAPDSSSVVVSGLMVATk.[Y]	N-Term(iTRAQ4plex); K21(iTRAQ	1	1
[R].wSRPQAPITGYR.[I]	N-Term(iTRAQ4plex)	1	1
[R].wSRPQAPITGYR.[I]	N-Term(iTRAQ4plex)	1	1
[K].iYLYTLNDNAR.[S]	N-Term(iTRAQ4plex)	1	1
[R].tNTNVNcPIEcFMPLDVQADR.[E]	N-Term(iTRAQ4plex); C7(Carbam	1	1
[R].gFNcESkPEAEETcFDk.[Y]	N-Term(iTRAQ4plex); C4(Carbam	1	1
[R].rPGGEPSPEGTTGQSYNQYSQR.[Y]	N-Term(iTRAQ4plex)	1	1
[K].vVTPLSPPTNLHLEANPDTGVLTVSWER.[S	N-Term(iTRAQ4plex)	1	1
[R].eSkPLTAQQTTk.[L]	N-Term(iTRAQ4plex); K3(iTRAQ4	1	1
[R].qDGHLWcSTTSNYEQDQk.[Y]	N-Term(iTRAQ4plex); C7(Carbam	1	1
[K].vTIMWTPPESAVTGYR.[V]	N-Term(iTRAQ4plex)	1	1
[R].qDGHLWcSTTSNYEQDQk.[Y]	N-Term(iTRAQ4plex); C7(Carbam	1	1
[K].cDPHEATcYDDGk.[T]	N-Term(iTRAQ4plex); C1(Carbam	1	1
[K].hYQINQQWER.[T]	N-Term(iTRAQ4plex)	1	1
[R].qYNVGPSVSk.[Y]	N-Term(iTRAQ4plex); K10(iTRAQ	1	1
[K].tYHVGEQWQk.[E]	N-Term(iTRAQ4plex); K10(iTRAQ	1	1
[R].sSPVVIDASTAIDAPSNLR.[F]	N-Term(iTRAQ4plex)	1	1
[R].gDSPASSkPISINYR.[T]	N-Term(iTRAQ4plex); K8(iTRAQ4	1	1
[R].cHEGGQSYk.[I]	N-Term(iTRAQ4plex); C1(Carbam	1	1
[R].dAPIVNk.[V]	N-Term(iTRAQ4plex); K7(iTRAQ4	1	1
[K].dSMIWDcTcIGAGR.[G]	N-Term(iTRAQ4plex); C7(Carbam	1	1
[R].iTYGETGGNSPVQEFTVPGSk.[S]	N-Term(iTRAQ4plex); K21(iTRAQ	1	1
[K].yEVSVYALk.[D]	N-Term(iTRAQ4plex); K9(iTRAQ4	1	1
[K].gEWTckPIAEk.[C]	N-Term(iTRAQ4plex); C5(Carbam	1	1
[R].nTFAEVTGLSPGVTYYFk.[V]	N-Term(iTRAQ4plex); K18(iTRAQ	1	1
[K].lLcQcLGFGSGHFR.[C]	N-Term(iTRAQ4plex); C3(Carbam	1	1
[R].dAPIVNk.[V]	N-Term(iTRAQ4plex); K7(iTRAQ4	1	1
[K].yEkPGSPPR.[E]	N-Term(iTRAQ4plex); K3(iTRAQ4	1	1
[R].rPGGEPSPEGTTGQSYNQYSQR.[Y]	N-Term(iTRAQ4plex)	1	1
[R].iScTIANR.[C]	N-Term(iTRAQ4plex); C3(Carbam	1	1
[K].sTATISGLkPGVDYTITVYAVTGR.[G]	N-Term(iTRAQ4plex); K9(iTRAQ4	1	1
[R].gDSPASSkPISINYR.[T]	N-Term(iTRAQ4plex); K8(iTRAQ4	1	1

[K].gEWTckPIAEk.[C]	N-Term(iTRAQ4plex); C5(Carbam	1	1
[R].FTNIGPDTMR.[V]		1	1
[K].vFAVSHGR.[E]	N-Term(iTRAQ4plex)	1	1
[K].ySFcTDHTVLVQTR.[G]	N-Term(iTRAQ4plex); C4(Carbam	1	1
[K].wcGTTQNYDADQk.[F]	N-Term(iTRAQ4plex); C2(Carbam	1	1
[R].cHEGGQSYk.[I]	N-Term(iTRAQ4plex); C1(Carbam	1	1
[K].ySFcTDHTVLVQTR.[G]	N-Term(iTRAQ4plex); C4(Carbam	1	1
[K].iYLYTLNDNAR.[S]	N-Term(iTRAQ4plex)	1	1
[K].vFAVSHGR.[E]	N-Term(iTRAQ4plex)	1	1
[R].qYNVGPSVSkYPLR.[N]	N-Term(iTRAQ4plex); K10(iTRAQ4	1	1
[R].sTTPDITGYR.[I]	N-Term(iTRAQ4plex)	1	1
[R].iTYGETGGNSPVQEFTVPGSk.[S]	N-Term(iTRAQ4plex); K21(iTRAQ4	1	1
[R].dLQFVEVTDVk.[V]	N-Term(iTRAQ4plex); K11(iTRAQ4	1	1
[K].hYQINQQWER.[T]	N-Term(iTRAQ4plex)	1	1
[K].eINLAPDSSSVVVSGLMVATk.[Y]	N-Term(iTRAQ4plex); K21(iTRAQ4	1	1
[R].IScTIANR.[C]	C3(Carbamidomethyl)	1	1
[R].ESkPLTAQQTTk.[L]	K3(iTRAQ4plex); K12(iTRAQ4plex	1	1
[R].eSKPLTAQQTTk.[L]	N-Term(iTRAQ4plex); K12(iTRAQ4	1	1
[R].gEWTcIAYSQLR.[D]	N-Term(iTRAQ4plex); C5(Carbam	1	1
[K].ySFcTDHTVLVQTR.[G]	N-Term(iTRAQ4plex); C4(Carbam	1	1
[R].iScTIANR.[C]	N-Term(iTRAQ4plex); C3(Carbam	1	1
[R].aEDHFSVIDFNQNIR.[T]	N-Term(iTRAQ4plex)	1	1
[R].aEDHFSVIDFNQNIR.[T]	N-Term(iTRAQ4plex)	1	1
[R].aEDHFSVIDFNQNIR.[T]	N-Term(iTRAQ4plex)	1	1
[R].aEDHFSVIDFNQNIR.[T]	N-Term(iTRAQ4plex)	1	1
[R].aEDHFSVIDFNQNIR.[T]	N-Term(iTRAQ4plex)	1	1
[R].aEDHFSVIDFNQNIR.[T]	N-Term(iTRAQ4plex)	1	1
[R].aEDHFSVIDFNQNIR.[T]	N-Term(iTRAQ4plex)	1	1
[R].mLADAPPQDPSccSGALYYGSk.[V]	N-Term(iTRAQ4plex); C12(Carbar	1	1
[R].mLADAPPQDPSccSGALYYGSk.[V]	N-Term(iTRAQ4plex); C12(Carbar	1	1
[R].mLADAPPQDPSccSGALYYGSk.[V]	N-Term(iTRAQ4plex); C12(Carbar	1	1
[K].kFYNQVSTPLLR.[N]	N-Term(iTRAQ4plex)	1	1
[K].IQPSGGTNINEALLR.[A]		1	1
[K].kFYNQVSTPLLR.[N]	K1(iTRAQ4plex)	1	1
[R].SLAPTAAAk.[R]	K9(iTRAQ4plex)	1	1
[K].aHVSFkPTVAQQR.[I]	N-Term(iTRAQ4plex); K6(iTRAQ4	1	1
[R].eTAVDGELVVLYDVk.[R]	N-Term(iTRAQ4plex); K15(iTRAQ4	1	1
[R].eTAVDGELVVLYDVk.[R]	N-Term(iTRAQ4plex); K15(iTRAQ4	1	1
[R].eTAVDGELVVLYDVk.[R]	N-Term(iTRAQ4plex); K15(iTRAQ4	1	1
[R].eTAVDGELVVLYDVk.[R]	N-Term(iTRAQ4plex); K15(iTRAQ4	1	1
[R].eTAVDGELVVLYDVk.[R]	N-Term(iTRAQ4plex); K15(iTRAQ4	1	1
[R].eTAVDGELVVLYDVk.[R]	N-Term(iTRAQ4plex); K15(iTRAQ4	1	1
[R].eTAVDGELVVLYDVk.[R]	N-Term(iTRAQ4plex); K15(iTRAQ4	1	1
[R].eTAVDGELVVLYDVk.[R]	N-Term(iTRAQ4plex); K15(iTRAQ4	1	1
[R].eTAVDGELVVLYDVk.[R]	N-Term(iTRAQ4plex); K15(iTRAQ4	1	1
[R].eTAVDGELVVLYDVk.[R]	N-Term(iTRAQ4plex); K15(iTRAQ4	1	1
[R].eTAVDGELVVLYDVk.[R]	N-Term(iTRAQ4plex); K15(iTRAQ4	1	1
[R].eTAVDGELVVLYDVk.[R]	N-Term(iTRAQ4plex); K15(iTRAQ4	1	1
[R].eTAVDGELVVLYDVk.[R]	N-Term(iTRAQ4plex); K15(iTRAQ4	1	1
[R].eTAVDGELVVLYDVk.[R]	N-Term(iTRAQ4plex); K15(iTRAQ4	1	1
[R].eTAVDGELVVLYDVk.[R]	N-Term(iTRAQ4plex); K15(iTRAQ4	1	1
[R].eTAVDGELVVLYDVk.[R]	N-Term(iTRAQ4plex); K15(iTRAQ4	1	1

[R].eTAVDGELVVLYDVk.[R]	N-Term(iTRAQ4plex); K15(iTRAQ	1	1
[R].eTAVDGELVVLYDVk.[R]	N-Term(iTRAQ4plex); K15(iTRAQ	1	1
[R].eTAVDGELVVLYDVk.[R]	N-Term(iTRAQ4plex); K15(iTRAQ	1	1
[R].eTAVDGELVVLYDVk.[R]	N-Term(iTRAQ4plex); K15(iTRAQ	1	1
[R].eTAVDGELVVLYDVk.[R]	N-Term(iTRAQ4plex); K15(iTRAQ	1	1
[R].eTAVDGELVVLYDVk.[R]	N-Term(iTRAQ4plex); K15(iTRAQ	1	1
[R].eTAVDGELVVLYDVk.[R]	N-Term(iTRAQ4plex); K15(iTRAQ	1	1
[R].eTAVDGELVVLYDVk.[R]	N-Term(iTRAQ4plex); K15(iTRAQ	1	1
[R].eTAVDGELVVLYDVk.[R]	N-Term(iTRAQ4plex); K15(iTRAQ	1	1
[R].eTAVDGELVVLYDVk.[R]	N-Term(iTRAQ4plex); K15(iTRAQ	1	1
[R].eTAVDGELVVLYDVk.[R]	N-Term(iTRAQ4plex); K15(iTRAQ	1	1
[R].eTAVDGELVVLYDVk.[R]	N-Term(iTRAQ4plex); K15(iTRAQ	1	1
[R].eTAVDGELVVLYDVk.[R]	N-Term(iTRAQ4plex); K15(iTRAQ	1	1
[R].eTAVDGELVVLYDVk.[R]	N-Term(iTRAQ4plex); K15(iTRAQ	1	1
[R].eTAVDGELVVLYDVk.[R]	N-Term(iTRAQ4plex); K15(iTRAQ	1	1
[R].eTAVDGELVVLYDVk.[R]	N-Term(iTRAQ4plex); K15(iTRAQ	1	1
[R].mLADAPPQDPSccSGALYYGSk.[V]	N-Term(iTRAQ4plex); C12(Carbar	1	1
[R].mLADAPPQDPSccSGALYYGSk.[V]	N-Term(iTRAQ4plex); C12(Carbar	1	1
[R].mLADAPPQDPSccSGALYYGSk.[V]	N-Term(iTRAQ4plex); C12(Carbar	1	1
[R].mLADAPPQDPSccSGALYYGSk.[V]	N-Term(iTRAQ4plex); C12(Carbar	1	1
[K].kFYNQVSTPLLR.[N]	N-Term(iTRAQ4plex); K1(iTRAQ4	1	1
[R].mATTMIQSk.[V]	N-Term(iTRAQ4plex); K9(iTRAQ4	1	1
[R].lSNENHGIAQR.[I]	N-Term(iTRAQ4plex)	1	1
[R].lSNENHGIAQR.[I]	N-Term(iTRAQ4plex)	1	1
[R].eTAVDGELVVLYDVk.[R]	N-Term(iTRAQ4plex); K15(iTRAQ	1	1
[R].eTAVDGELVVLYDVk.[R]	N-Term(iTRAQ4plex); K15(iTRAQ	1	1
[R].kLGSYEHR.[I]	N-Term(iTRAQ4plex); K1(iTRAQ4	1	1
[R].sLPGESEEMMEEVDQVTLYSYk.[V]	N-Term(iTRAQ4plex); K22(iTRAQ	1	1
[R].sLPGESEEMMEEVDQVTLYSYk.[V]	N-Term(iTRAQ4plex); K22(iTRAQ	1	1
[R].mATTMIQSk.[V]	N-Term(iTRAQ4plex); M1(Oxidat	1	1
[R].mATTmIQSk.[V]	N-Term(iTRAQ4plex); M5(Oxidat	1	1
[K].aHVSFkPTVAQQR.[I]	N-Term(iTRAQ4plex); K6(iTRAQ4	1	1
[K].aHVSFkPTVAQQR.[I]	N-Term(iTRAQ4plex); K6(iTRAQ4	1	1
[K].aHVSFkPTVAQQR.[I]	N-Term(iTRAQ4plex); K6(iTRAQ4	1	1
[K].aHVSFkPTVAQQR.[I]	N-Term(iTRAQ4plex); K6(iTRAQ4	1	1
[K].aHVSFkPTVAQQR.[I]	N-Term(iTRAQ4plex); K6(iTRAQ4	1	1
[R].FLHVPDTFEGHFDGVPVISk.[G]	K20(iTRAQ4plex)	1	1
[K].vVNNSPQPQNVVFDVQIPk.[G]	N-Term(iTRAQ4plex); K19(iTRAQ	1	1
[K].vVNNSPQPQNVVFDVQIPk.[G]	N-Term(iTRAQ4plex); K19(iTRAQ	1	1
[K].vVNNSPQPQNVVFDVQIPk.[G]	N-Term(iTRAQ4plex); K19(iTRAQ	1	1
[R].fLHVPDTFEGHFDGVPVISk.[G]	N-Term(iTRAQ4plex); K20(iTRAQ	1	1
[R].nVQFNYPHTSVTDVTQNNFHNYFGGSEIV	N-Term(iTRAQ4plex); K33(iTRAQ	1	1
[R].fLHVPDTFEGHFDGVPVISk.[G]	N-Term(iTRAQ4plex); K20(iTRAQ	1	1
[K].iQPSGGTNINEALLR.[A]	N-Term(iTRAQ4plex)	1	1
[K].iQPSGGTNINEALLR.[A]	N-Term(iTRAQ4plex)	1	1
[R].LSNENHGIAQR.[I]		1	1
[R].LSNENHGIAQR.[I]		1	1
[R].aEDHFSVIDFNQNIR.[T]	N-Term(iTRAQ4plex)	1	1
[K].AHVSFkPTVAQQR.[I]	K6(iTRAQ4plex)	1	1
[K].vQFELHYQEVk.[W]	N-Term(iTRAQ4plex); K11(iTRAQ	1	1
[R].sLAPTAAAkR.[R]	N-Term(iTRAQ4plex); K9(iTRAQ4	1	1
[K].vQFELHYQEVk.[W]	N-Term(iTRAQ4plex); K11(iTRAQ	1	1

[R].SLAPTAAAk.[R]	K9(iTRAQ4plex)	1	1
[K].vQFELHYQEVk.[W]	N-Term(iTRAQ4plex); K11(iTRAQ	1	1
[K].vQFELHYQEVk.[W]	N-Term(iTRAQ4plex); K11(iTRAQ	1	1
[K].vQFELHYQEVk.[W]	N-Term(iTRAQ4plex); K11(iTRAQ	1	1
[K].vQFELHYQEVk.[W]	N-Term(iTRAQ4plex); K11(iTRAQ	1	1
[K].vQFELHYQEVk.[W]	N-Term(iTRAQ4plex); K11(iTRAQ	1	1
[K].vQFELHYQEVk.[W]	N-Term(iTRAQ4plex); K11(iTRAQ	1	1
[K].hLEVDVWVIEPQGLR.[F]	N-Term(iTRAQ4plex)	1	1
[K].hLEVDVWVIEPQGLR.[F]	N-Term(iTRAQ4plex)	1	1
[K].hLEVDVWVIEPQGLR.[F]	N-Term(iTRAQ4plex)	1	1
[K].tILDDLRAEDHFSVIDFNQNIR.[T]	N-Term(iTRAQ4plex)	1	1
[K].hLEVDVWVIEPQGLR.[F]	N-Term(iTRAQ4plex)	1	1
[R].sILQMSLDHHIVTPLTSLVIENEAGDER.[M]	N-Term(iTRAQ4plex)	1	1
[R].SSALDMENFR.[T]		1	1
[R].kLGSYEHR.[I]	N-Term(iTRAQ4plex); K1(iTRAQ4	1	1
[K].dkHADPDFTR.[K]	N-Term(iTRAQ4plex); K2(iTRAQ4	1	1
[R].kLWAYLTINQLLAER.[S]	N-Term(iTRAQ4plex); K1(iTRAQ4	1	1
[R].kLWAYLTINQLLAER.[S]	N-Term(iTRAQ4plex); K1(iTRAQ4	1	1
[R].sLAPTAAAk.[R]	N-Term(iTRAQ4plex); K9(iTRAQ4	1	1
[R].sLAPTAAAk.[R]	N-Term(iTRAQ4plex); K9(iTRAQ4	1	1
[R].sLAPTAAAk.[R]	N-Term(iTRAQ4plex); K9(iTRAQ4	1	1
[R].fLHVPDTFEGHFDGVPVISk.[G]	N-Term(iTRAQ4plex); K20(iTRAQ	1	1
[R].fLHVPDTFEGHFDGVPVISk.[G]	N-Term(iTRAQ4plex); K20(iTRAQ	1	1
[R].fLHVPDTFEGHFDGVPVISk.[G]	N-Term(iTRAQ4plex); K20(iTRAQ	1	1
[R].fLHVPDTFEGHFDGVPVISk.[G]	N-Term(iTRAQ4plex); K20(iTRAQ	1	1
[R].fLHVPDTFEGHFDGVPVISk.[G]	N-Term(iTRAQ4plex); K20(iTRAQ	1	1
[R].fLHVPDTFEGHFDGVPVISk.[G]	N-Term(iTRAQ4plex); K20(iTRAQ	1	1
[K].nILFVIDVSGSMWGVk.[M]	N-Term(iTRAQ4plex); K16(iTRAQ	1	1
[K].nILFVIDVSGSMWGVk.[M]	N-Term(iTRAQ4plex); K16(iTRAQ	1	1
[R].tEVNVLPGAk.[V]	N-Term(iTRAQ4plex); K10(iTRAQ	1	1
[R].tEVNVLPGAk.[V]	N-Term(iTRAQ4plex); K10(iTRAQ	1	1
[R].tEVNVLPGAk.[V]	N-Term(iTRAQ4plex); K10(iTRAQ	1	1
[K].lWAYLTINQLLAER.[S]	N-Term(iTRAQ4plex)	1	1
[K].lWAYLTINQLLAER.[S]	N-Term(iTRAQ4plex)	1	1
[R].sSALDMENFR.[T]	N-Term(iTRAQ4plex)	1	1
[R].sSALDMENFR.[T]	N-Term(iTRAQ4plex)	1	1
[K].fYNQVSTPLLR.[N]	N-Term(iTRAQ4plex)	1	1
[K].fYNQVSTPLLR.[N]	N-Term(iTRAQ4plex)	1	1
[K].fYNQVSTPLLR.[N]	N-Term(iTRAQ4plex)	1	1
[K].lDQIESVITATSANTQLVLETLAQMDDLQD	N-Term(iTRAQ4plex); K34(iTRAQ	1	1
[R].iYGNQDTSSQLk.[K]	N-Term(iTRAQ4plex); K12(iTRAQ	1	1
[R].iYGNQDTSSQLk.[K]	N-Term(iTRAQ4plex); K12(iTRAQ	1	1
[K].lDQIESVITATSANTQLVLETLAQMDDLQD	N-Term(iTRAQ4plex); K34(iTRAQ	1	1
[R].mLADAPPQDPSccSGALYYGSk.[V]	N-Term(iTRAQ4plex); M1(Oxidat	1	1
[R].LSNENHGIAQR.[I]		1	1
[R].nDLISATk.[T]	N-Term(iTRAQ4plex); K8(iTRAQ4	1	1
[K].aHVSFkPTVAQQR.[I]	N-Term(iTRAQ4plex); K6(iTRAQ4	1	1
[R].lSNENHGIAQR.[I]	N-Term(iTRAQ4plex)	1	1
[R].eTAVDGELVVLYDVk.[R]	N-Term(iTRAQ4plex); K15(iTRAQ	1	1
[K].aHVSFkPTVAQQR.[I]	N-Term(iTRAQ4plex); K6(iTRAQ4	1	1
[R].sLAPTAAAk.[R]	N-Term(iTRAQ4plex); K9(iTRAQ4	1	1
[R].sLAPTAAAk.[R]	N-Term(iTRAQ4plex); K9(iTRAQ4	1	1

[R].FLHVPDTFEGHFDGVPVISk.[G]	K20(iTRAQ4plex)	1	1
[R].aEDHFSVIDFNQNIR.[T]	N-Term(iTRAQ4plex)	1	1
[R].FLHVPDTFEGHFDGVPVISk.[G]	K20(iTRAQ4plex)	1	1
[K].aHVSFkPTVAQQR.[I]	N-Term(iTRAQ4plex); K6(iTRAQ4	1	1
[R].tWRNDLISATk.[T]	N-Term(iTRAQ4plex); K11(iTRAQ	1	1
[R].AEDHFSVIDFNQNIR.[T]		1	1
[R].lSNENHGIAQR.[I]	N-Term(iTRAQ4plex)	1	1
[K].hLEVDVWVIEPQGLR.[F]	N-Term(iTRAQ4plex)	1	1
[R].eTAVDGELVVLYDVk.[R]	N-Term(iTRAQ4plex); K15(iTRAQ	1	1
[R].eTAVDGELVVLYDVk.[R]	N-Term(iTRAQ4plex); K15(iTRAQ	1	1
[R].aEDHFSVIDFNQNIR.[T]	N-Term(iTRAQ4plex)	1	1
[K].AHVSFkPTVAQQR.[I]	K6(iTRAQ4plex)	1	1
[R].TEVNVLPGAk.[V]	K10(iTRAQ4plex)	1	1
[K].tQVADAk.[R]	N-Term(iTRAQ4plex); K7(iTRAQ4	1	1
[R].lSNENHGIAQR.[I]	N-Term(iTRAQ4plex)	1	1
[K].fQLVAENR.[R]	N-Term(iTRAQ4plex)	1	1
[R].nDLISATk.[T]	N-Term(iTRAQ4plex); K8(iTRAQ4	1	1
[R].sSALDmENFR.[T]	N-Term(iTRAQ4plex); M6(Oxidat	1	1
[K].aHVSFkPTVAQQR.[I]	N-Term(iTRAQ4plex); K6(iTRAQ4	1	1
[K].HADPDFTR.[K]		1	1
[K].FYNQVSTPLLR.[N]		1	1
[K].kFYNQVSTPLLR.[N]	N-Term(iTRAQ4plex); K1(iTRAQ4	1	1
[K].tQVADAk.[R]	N-Term(iTRAQ4plex); K7(iTRAQ4	1	1
[R].FLHVPDTFEGHFDGVPVISk.[G]	K20(iTRAQ4plex)	1	1
[R].mLADAPPQDPSccSGALYYGSk.[V]	N-Term(iTRAQ4plex); C12(Carbar	1	1
[R].mLADAPPQDPSccSGALYYGSk.[V]	N-Term(iTRAQ4plex); M1(Oxidat	1	1
[R].SSALDmENFR.[T]	M6(Oxidation)	1	1
[R].sSIkEKTVGR.[A]	N-Term(iTRAQ4plex); K4(iTRAQ4	1	1
[R].SLAPTAAAkR.[R]	K9(iTRAQ4plex)	1	1
[K].aHVSFkPTVAQQR.[I]	N-Term(iTRAQ4plex); K6(iTRAQ4	1	1
[K].tILDDLR.[A]	N-Term(iTRAQ4plex)	1	1
[R].eTAVDGELVVLYDVk.[R]	N-Term(iTRAQ4plex); K15(iTRAQ	1	1
[K].rLSNENHGIAQR.[I]	N-Term(iTRAQ4plex)	1	1
[R].mATTMIQSk.[V]	N-Term(iTRAQ4plex); K9(iTRAQ4	1	1
[K].qTVEAMk.[T]	N-Term(iTRAQ4plex); K7(iTRAQ4	1	1
[K].HADPDFTR.[K]		1	1
[K].AHVSFkPTVAQQR.[I]	K6(iTRAQ4plex)	1	1
[K].hLEVDVWVIEPQGLR.[F]	N-Term(iTRAQ4plex)	1	1
[K].qTVEAMk.[T]	N-Term(iTRAQ4plex); K7(iTRAQ4	1	1
[R].nVQFNYPHTSVTDVTQNNFHNYFGGSEIV	N-Term(iTRAQ4plex); K33(iTRAQ	1	1
[K].qTVEAMk.[T]	N-Term(iTRAQ4plex); K7(iTRAQ4	1	1
[K].VQSTITSR.[M]		1	1
[R].nDLISATk.[T]	N-Term(iTRAQ4plex); K8(iTRAQ4	1	1
[R].FLHVPDTFEGHFDGVPVISk.[G]	K20(iTRAQ4plex)	1	1
[R].sLAPTAAAk.[R]	N-Term(iTRAQ4plex); K9(iTRAQ4	1	1
[R].mATTmIQSk.[V]	N-Term(iTRAQ4plex); M5(Oxidat	1	1
[K].tQVADAk.[R]	N-Term(iTRAQ4plex); K7(iTRAQ4	1	1
[R].LSNENHGIAQR.[I]		1	1
[R].IYGNQDTSSQLk.[K]	K12(iTRAQ4plex)	1	1
[K].vQSTITSR.[M]	N-Term(iTRAQ4plex)	1	1
[K].vQSTITSR.[M]	N-Term(iTRAQ4plex)	1	1
[R].fLHVPDTFEGHFDGVPVISk.[G]	N-Term(iTRAQ4plex); K20(iTRAQ	1	1

[R].eTAVDGELVVLYDVk.[R]	N-Term(iTRAQ4plex); K15(iTRAQ4	1	1
[K].AHVSFkPTVAQQR.[I]	K6(iTRAQ4plex)	1	1
[K].tILDDLR.[A]	N-Term(iTRAQ4plex)	1	1
[K].qTVEAMk.[T]	N-Term(iTRAQ4plex); K7(iTRAQ4	1	1
[K].vVNNSPQPQNVVFDVQIPk.[G]	N-Term(iTRAQ4plex); K19(iTRAQ4	1	1
[K].vVNNSPQPQNVVFDVQIPk.[G]	N-Term(iTRAQ4plex); K19(iTRAQ4	1	1
[R].iYGNQDTSSQLk.[K]	N-Term(iTRAQ4plex); K12(iTRAQ4	1	1
[R].mLADAPPQDPSccSGALYYGSk.[V]	N-Term(iTRAQ4plex); C12(Carbar	1	1
[K].qTVEAMk.[T]	N-Term(iTRAQ4plex); K7(iTRAQ4	1	1
[R].LSNENHGIAQR.[I]		1	1
[K].FYNQVSTPLLR.[N]		1	1
[R].SLAPTAAAk.[R]	K9(iTRAQ4plex)	1	1
[R].nDLISATk.[T]	N-Term(iTRAQ4plex); K8(iTRAQ4	1	1
[K].aHVSFkPTVAQQR.[I]	N-Term(iTRAQ4plex); K6(iTRAQ4	1	1
[K].tQVADAk.[R]	N-Term(iTRAQ4plex); K7(iTRAQ4	1	1
[R].nDLISATk.[T]	N-Term(iTRAQ4plex); K8(iTRAQ4	1	1
[R].LSNENHGIAQR.[I]		1	1
[R].aEDHFSVIDFNQNIR.[T]	N-Term(iTRAQ4plex)	1	1
[K].aHVSFkPTVAQQR.[I]	N-Term(iTRAQ4plex); K6(iTRAQ4	1	1
[K].kFYNQVSTPLLR.[N]	N-Term(iTRAQ4plex); K1(iTRAQ4	1	1
[R].SLAPTAAAk.[R]	K9(iTRAQ4plex)	1	1
[R].fLHVPDTFEGHFDGVPVISk.[G]	N-Term(iTRAQ4plex); K20(iTRAQ4	1	1
[K].qTVEAMk.[T]	N-Term(iTRAQ4plex); K7(iTRAQ4	1	1
[R].mATTMIQSk.[V]	N-Term(iTRAQ4plex); M1(Oxidat	1	1
[R].FLHVPDTFEGHFDGVPVISk.[G]	K20(iTRAQ4plex)	1	1
[K].tILDDLR.[A]	N-Term(iTRAQ4plex)	1	1
[K].dkHADPDFTR.[K]	N-Term(iTRAQ4plex); K2(iTRAQ4	1	1
[R].mLADAPPQDPSccSGALYYGSk.[V]	N-Term(iTRAQ4plex); M1(Oxidat	1	1
[R].nVQFNYPHTSVTDVTQNNFHNYFGGSEIV	N-Term(iTRAQ4plex); K33(iTRAQ4	1	1
[K].aHVSFkPTVAQQR.[I]	N-Term(iTRAQ4plex); K6(iTRAQ4	1	1
[R].QSEDSTFYLGER.[T]		1	1
[K].aLYLQYTDETFR.[T]	N-Term(iTRAQ4plex)	1	1
[K].ALYLQYTDETFR.[T]		1	1
[R].kAEEEHLGILGPQLHADVGDk.[V]	N-Term(iTRAQ4plex); K1(iTRAQ4	1	1
[K].vDkDNEDFQESNR.[M]	N-Term(iTRAQ4plex); K3(iTRAQ4	1	1
[R].iYHSHIDAPk.[D]	N-Term(iTRAQ4plex); K10(iTRAQ4	1	1
[R].sGAGTEDSAcIPWAYYSTVDQVk.[D]	N-Term(iTRAQ4plex); C10(Carbar	1	1
[R].sGAGTEDSAcIPWAYYSTVDQVk.[D]	N-Term(iTRAQ4plex); C10(Carbar	1	1
[R].sGAGTEDSAcIPWAYYSTVDQVk.[D]	N-Term(iTRAQ4plex); C10(Carbar	1	1
[R].sGAGTEDSAcIPWAYYSTVDQVk.[D]	N-Term(iTRAQ4plex); C10(Carbar	1	1
[R].sGAGTEDSAcIPWAYYSTVDQVk.[D]	N-Term(iTRAQ4plex); C10(Carbar	1	1
[R].sGAGTEDSAcIPWAYYSTVDQVk.[D]	N-Term(iTRAQ4plex); C10(Carbar	1	1
[R].sGAGTEDSAcIPWAYYSTVDQVk.[D]	N-Term(iTRAQ4plex); C10(Carbar	1	1
[R].sGAGTEDSAcIPWAYYSTVDQVk.[D]	N-Term(iTRAQ4plex); C10(Carbar	1	1
[R].IYHSHIDAPk.[D]	K10(iTRAQ4plex)	1	1
[R].IYHSHIDAPk.[D]	K10(iTRAQ4plex)	1	1
[R].SGAGTEDSAcIPWAYYSTVDQVk.[D]	C10(Carbamidomethyl); K23(iTR	1	1
[K].vYPGEQYTYmLLATEEQSPGEGDGNcVTR.	N-Term(iTRAQ4plex); M10(Oxida	1	1
[R].iYHSHIDAPk.[D]	N-Term(iTRAQ4plex); K10(iTRAQ4	1	1
[R].IYHSHIDAPk.[D]	K10(iTRAQ4plex)	1	1
[R].IYHSHIDAPk.[D]	K10(iTRAQ4plex)	1	1
[R].iYHSHIDAPk.[D]	N-Term(iTRAQ4plex); K10(iTRAQ4	1	1

[R].iYHSHIDAPk.[D]	N-Term(iTRAQ4plex); K10(iTRAQ	1	1
[R].iYHSHIDAPk.[D]	N-Term(iTRAQ4plex); K10(iTRAQ	1	1
[K].vDkDNEDFQESNR.[M]	N-Term(iTRAQ4plex); K3(iTRAQ4	1	1
[K].dVDkEFYLFPTVFDENESLLLEDNIR.[M]	N-Term(iTRAQ4plex); K4(iTRAQ4	1	1
[K].dVDkEFYLFPTVFDENESLLLEDNIR.[M]	N-Term(iTRAQ4plex); K4(iTRAQ4	1	1
[R].IYHSHIDAPk.[D]	K10(iTRAQ4plex)	1	1
[K].aGLQAFFQVQEcNk.[S]	N-Term(iTRAQ4plex); C12(Carbar	1	1
[K].aGLQAFFQVQEcNk.[S]	N-Term(iTRAQ4plex); C12(Carbar	1	1
[R].gPEEEHLGILGPVIWAEVGDTIR.[V]	N-Term(iTRAQ4plex)	1	1
[R].IYHSHIDAPk.[D]	K10(iTRAQ4plex)	1	1
[K].vDkDNEDFQESNR.[M]	N-Term(iTRAQ4plex); K3(iTRAQ4	1	1
[K].hYYIGIIETTWDYASDHGEk.[K]	N-Term(iTRAQ4plex); K20(iTRAQ	1	1
[K].hYYIGIIETTWDYASDHGEk.[K]	N-Term(iTRAQ4plex); K20(iTRAQ	1	1
[K].hYYIGIIETTWDYASDHGEk.[K]	N-Term(iTRAQ4plex); K20(iTRAQ	1	1
[K].hYYIGIIETTWDYASDHGEk.[K]	N-Term(iTRAQ4plex); K20(iTRAQ	1	1
[K].hYYIGIIETTWDYASDHGEk.[K]	N-Term(iTRAQ4plex); K20(iTRAQ	1	1
[K].hYYIGIIETTWDYASDHGEk.[K]	N-Term(iTRAQ4plex); K20(iTRAQ	1	1
[R].tYYIAAVEVEWDYSPQR.[E]	N-Term(iTRAQ4plex)	1	1
[R].tYYIAAVEVEWDYSPQR.[E]	N-Term(iTRAQ4plex)	1	1
[K].vYPGEQYTYMLLATEEQSPGEGDGNcVTR.	N-Term(iTRAQ4plex); C26(Carbar	1	1
[K].vYPGEQYTYMLLATEEQSPGEGDGNcVTR.	N-Term(iTRAQ4plex); C26(Carbar	1	1
[K].vYPGEQYTYMLLATEEQSPGEGDGNcVTR.	N-Term(iTRAQ4plex); C26(Carbar	1	1
[K].eRGPEEEHLGILGPVIWAEVGDTIR.[V]	N-Term(iTRAQ4plex)	1	1
[R].gPEEEHLGILGPVIWAEVGDTIR.[V]	N-Term(iTRAQ4plex)	1	1
[R].qkDVDkEFYLFPTVFDENESLLLEDNIR.[M]	N-Term(iTRAQ4plex); K2(iTRAQ4	1	1
[R].sVPPSASHVAPTETFTYEWTVPk.[E]	N-Term(iTRAQ4plex); K23(iTRAQ	1	1
[R].sVPPSASHVAPTETFTYEWTVPk.[E]	N-Term(iTRAQ4plex); K23(iTRAQ	1	1
[K].aLYLQYTDETFR.[T]	N-Term(iTRAQ4plex)	1	1
[K].aLYLQYTDETFR.[T]	N-Term(iTRAQ4plex)	1	1
[R].sVPPSASHVAPTETFTYEWTVPk.[E]	N-Term(iTRAQ4plex); K23(iTRAQ	1	1
[R].sVPPSASHVAPTETFTYEWTVPk.[E]	N-Term(iTRAQ4plex); K23(iTRAQ	1	1
[R].sVPPSASHVAPTETFTYEWTVPk.[E]	N-Term(iTRAQ4plex); K23(iTRAQ	1	1
[K].gAYPLSIEPIGVR.[F]	N-Term(iTRAQ4plex)	1	1
[K].aLYLQYTDETFR.[T]	N-Term(iTRAQ4plex)	1	1
[R].sVPPSASHVAPTETFTYEWTVPk.[E]	N-Term(iTRAQ4plex); K23(iTRAQ	1	1
[R].sVPPSASHVAPTETFTYEWTVPk.[E]	N-Term(iTRAQ4plex); K23(iTRAQ	1	1
[R].sVPPSASHVAPTETFTYEWTVPk.[E]	N-Term(iTRAQ4plex); K23(iTRAQ	1	1
[R].sVPPSASHVAPTETFTYEWTVPk.[E]	N-Term(iTRAQ4plex); K23(iTRAQ	1	1
[K].gAYPLSIEPIGVR.[F]	N-Term(iTRAQ4plex)	1	1
[K].dIFTGLIGPmk.[I]	N-Term(iTRAQ4plex); M10(Oxida	1	1
[K].eVGPTNADPVcLAk.[M]	N-Term(iTRAQ4plex); C11(Carbar	1	1
[K].eVGPTNADPVcLAk.[M]	N-Term(iTRAQ4plex); C11(Carbar	1	1
[K].eVGPTNADPVcLAk.[M]	N-Term(iTRAQ4plex); C11(Carbar	1	1
[K].dIFTGLIGPmk.[I]	N-Term(iTRAQ4plex); M10(Oxida	1	1
[R].mFTTAPDQVDkEDEDFQESNk.[M]	N-Term(iTRAQ4plex); K11(iTRAQ	1	1
[R].mFTTAPDQVDkEDEDFQESNk.[M]	N-Term(iTRAQ4plex); K11(iTRAQ	1	1
[K].wYLFGMGNEVDVHAAFFHGQALTNk.[N]	N-Term(iTRAQ4plex); K25(iTRAQ	1	1
[R].mFTTAPDQVDkEDEDFQESNk.[M]	N-Term(iTRAQ4plex); K11(iTRAQ	1	1
[R].mFTTAPDQVDkEDEDFQESNk.[M]	N-Term(iTRAQ4plex); K11(iTRAQ	1	1
[R].mFTTAPDQVDkEDEDFQESNk.[M]	N-Term(iTRAQ4plex); K11(iTRAQ	1	1
[R].mFTTAPDQVDkEDEDFQESNk.[M]	N-Term(iTRAQ4plex); K11(iTRAQ	1	1
[K].eVGPTNADPVcLAk.[M]	N-Term(iTRAQ4plex); C11(Carbar	1	1

[R].eYTDASFTNR.[K]	N-Term(iTRAQ4plex)	1	1
[K].dIASGLIGPLIIck.[K]	N-Term(iTRAQ4plex); C13(Carbar	1	1
[R].eYTDASFTNR.[K]	N-Term(iTRAQ4plex)	1	1
[K].dIASGLIGPLIIck.[K]	N-Term(iTRAQ4plex); C13(Carbar	1	1
[R].eYTDASFTNR.[K]	N-Term(iTRAQ4plex)	1	1
[K].wYLFGMGNEVDVHAAFFHGQALTNk.[N]	N-Term(iTRAQ4plex); K25(iTRAQ	1	1
[R].mFTTAPDQVDkEDEDFQESNk.[M]	N-Term(iTRAQ4plex); M1(Oxidat	1	1
[R].mFTTAPDQVDkEDEDFQESNk.[M]	N-Term(iTRAQ4plex); M1(Oxidat	1	1
[R].mFTTAPDQVDkEDEDFQESNk.[M]	N-Term(iTRAQ4plex); M1(Oxidat	1	1
[R].mFTTAPDQVDkEDEDFQESNk.[M]	N-Term(iTRAQ4plex); M1(Oxidat	1	1
[R].IYHSHIDAPk.[D]	K10(iTRAQ4plex)	1	1
[R].IYHSHIDAPk.[D]	K10(iTRAQ4plex)	1	1
[R].TTIEkPVWLGFLGPIIk.[A]	K5(iTRAQ4plex); K17(iTRAQ4ple:	1	1
[R].tTIEKPVWLGFLGPIIk.[A]	N-Term(iTRAQ4plex); K17(iTRAQ	1	1
[K].kDSLDkEk.[E]	N-Term(iTRAQ4plex); K1(iTRAQ4	1	1
[R].TTIEkPVWLGFLGPIIk.[A]	K5(iTRAQ4plex); K17(iTRAQ4ple:	1	1
[R].iYHSHIDAPk.[D]	N-Term(iTRAQ4plex); K10(iTRAQ	1	1
[R].tTIEkPVWLGFLGPIIk.[A]	N-Term(iTRAQ4plex); K5(iTRAQ4	1	1
[R].tTIEkPVWLGFLGPIIk.[A]	N-Term(iTRAQ4plex); K5(iTRAQ4	1	1
[R].tTIEkPVWLGFLGPIIk.[A]	N-Term(iTRAQ4plex); K5(iTRAQ4	1	1
[R].dTANLFPQTSLTLHMWPDTEGTFNVEcLT	N-Term(iTRAQ4plex); C27(Carbar	1	1
[R].dTANLFPQTSLTLHMWPDTEGTFNVEcLT	N-Term(iTRAQ4plex); C27(Carbar	1	1
[K].dIFTGLIGPMk.[I]	N-Term(iTRAQ4plex); K11(iTRAQ	1	1
[K].dIFTGLIGPMk.[I]	N-Term(iTRAQ4plex); K11(iTRAQ	1	1
[K].nNEGTYYSPNYNPQSR.[S]	N-Term(iTRAQ4plex)	1	1
[K].nNEGTYYSPNYNPQSR.[S]	N-Term(iTRAQ4plex)	1	1
[K].nNEGTYYSPNYNPQSR.[S]	N-Term(iTRAQ4plex)	1	1
[K].NNEGTYYSPNYNPQSR.[S]		1	1
[K].vNkDDEEFIESNk.[M]	N-Term(iTRAQ4plex); K3(iTRAQ4	1	1
[K].vNkDDEEFIESNk.[M]	N-Term(iTRAQ4plex); K3(iTRAQ4	1	1
[K].vNkDDEEFIESNk.[M]	N-Term(iTRAQ4plex); K3(iTRAQ4	1	1
[K].vNkDDEEFIESNk.[M]	N-Term(iTRAQ4plex); K3(iTRAQ4	1	1
[K].vNkDDEEFIESNk.[M]	N-Term(iTRAQ4plex); K3(iTRAQ4	1	1
[K].vNkDDEEFIESNk.[M]	N-Term(iTRAQ4plex); K3(iTRAQ4	1	1
[K].vNkDDEEFIESNk.[M]	N-Term(iTRAQ4plex); K3(iTRAQ4	1	1
[K].vNkDDEEFIESNk.[M]	N-Term(iTRAQ4plex); K3(iTRAQ4	1	1
[K].dIASGLIGPLIIck.[K]	N-Term(iTRAQ4plex); C13(Carbar	1	1
[K].dIASGLIGPLIIck.[K]	N-Term(iTRAQ4plex); C13(Carbar	1	1
[K].vNkDDEEFIESNk.[M]	N-Term(iTRAQ4plex); K3(iTRAQ4	1	1
[K].vNkDDEEFIESNk.[M]	N-Term(iTRAQ4plex); K3(iTRAQ4	1	1
[K].vNkDDEEFIESNk.[M]	N-Term(iTRAQ4plex); K3(iTRAQ4	1	1
[K].vNkDDEEFIESNk.[M]	N-Term(iTRAQ4plex); K3(iTRAQ4	1	1
[R].mFTTAPDQVDkEDEDFQESNk.[M]	N-Term(iTRAQ4plex); M1(Oxidat	1	1
[R].mFTTAPDQVDkEDEDFQESNk.[M]	N-Term(iTRAQ4plex); M1(Oxidat	1	1
[R].tPGIWLLHcHVTDHIHAGMETTYTVLQNE	N-Term(iTRAQ4plex); C9(Carbam	1	1
[K].vNkDDEEFIESNk.[M]	N-Term(iTRAQ4plex); K3(iTRAQ4	1	1
[K].vNkDDEEFIESNk.[M]	N-Term(iTRAQ4plex); K3(iTRAQ4	1	1
[K].vNkDDEEFIESNk.[M]	N-Term(iTRAQ4plex); K3(iTRAQ4	1	1
[K].vNkDDEEFIESNk.[M]	N-Term(iTRAQ4plex); K3(iTRAQ4	1	1
[K].vNkDDEEFIESNk.[M]	N-Term(iTRAQ4plex); K3(iTRAQ4	1	1
[R].rQSEDSTFYLGER.[T]	N-Term(iTRAQ4plex)	1	1
[K].wYLFGMGNEVDVHAAFFHGQALTNk.[N]	N-Term(iTRAQ4plex); K25(iTRAQ	1	1

[K].vNkDDEEFIESNk.[M]	N-Term(iTRAQ4plex); K3(iTRAQ4	1	1
[K].vNkDDEEFIESNk.[M]	N-Term(iTRAQ4plex); K3(iTRAQ4	1	1
[K].vNkDDEEFIESNk.[M]	N-Term(iTRAQ4plex); K3(iTRAQ4	1	1
[R].rQSEDSTFYLGER.[T]	N-Term(iTRAQ4plex)	1	1
[K].vNkDDEEFIESNk.[M]	N-Term(iTRAQ4plex); K3(iTRAQ4	1	1
[R].rQSEDSTFYLGER.[T]	N-Term(iTRAQ4plex)	1	1
[K].vNkDDEEFIESNk.[M]	N-Term(iTRAQ4plex); K3(iTRAQ4	1	1
[K].vNkDDEEFIESNk.[M]	N-Term(iTRAQ4plex); K3(iTRAQ4	1	1
[K].vNkDDEEFIESNk.[M]	N-Term(iTRAQ4plex); K3(iTRAQ4	1	1
[R].rQSEDSTFYLGER.[T]	N-Term(iTRAQ4plex)	1	1
[K].vNkDDEEFIESNk.[M]	N-Term(iTRAQ4plex); K3(iTRAQ4	1	1
[R].QSEDSTFYLGER.[T]		1	1
[R].qSEDSTFYLGER.[T]	N-Term(iTRAQ4plex)	1	1
[R].qSEDSTFYLGER.[T]	N-Term(iTRAQ4plex)	1	1
[R].qSEDSTFYLGER.[T]	N-Term(iTRAQ4plex)	1	1
[R].qSEDSTFYLGER.[T]	N-Term(iTRAQ4plex)	1	1
[K].tYcSEPEk.[V]	N-Term(iTRAQ4plex); C3(Carbam	1	1
[R].gVYSSDVFDIFPGTYQTLEmFPR.[T]	N-Term(iTRAQ4plex); M20(Oxida	1	1
[K].mYYSAVDPTk.[D]	N-Term(iTRAQ4plex); K10(iTRAQ	1	1
[K].gEFYIGSk.[Y]	N-Term(iTRAQ4plex); K8(iTRAQ4	1	1
[K].aEEEHLGILGPQLHADVGDk.[V]	N-Term(iTRAQ4plex); K20(iTRAQ	1	1
[K].aEEEHLGILGPQLHADVGDk.[V]	N-Term(iTRAQ4plex); K20(iTRAQ	1	1
[K].vDkDNEDFQESNR.[M]	N-Term(iTRAQ4plex); K3(iTRAQ4	1	1
[K].vDkDNEDFQESNR.[M]	N-Term(iTRAQ4plex); K3(iTRAQ4	1	1
[K].vDkDNEDFQESNR.[M]	N-Term(iTRAQ4plex); K3(iTRAQ4	1	1
[K].vDkDNEDFQESNR.[M]	N-Term(iTRAQ4plex); K3(iTRAQ4	1	1
[K].vDkDNEDFQESNR.[M]	N-Term(iTRAQ4plex); K3(iTRAQ4	1	1
[K].vDkDNEDFQESNR.[M]	N-Term(iTRAQ4plex); K3(iTRAQ4	1	1
[R].kAEEEHLGILGPQLHADVGDk.[V]	N-Term(iTRAQ4plex); K21(iTRAQ	1	1
[K].dDEEFIESNk.[M]	N-Term(iTRAQ4plex); K10(iTRAQ	1	1
[R].kAEEEHLGILGPQLHADVGDk.[V]	K1(iTRAQ4plex); K21(iTRAQ4ple	1	1
[R].kAEEEHLGILGPQLHADVGDk.[V]	N-Term(iTRAQ4plex); K1(iTRAQ4	1	1
[R].kAEEEHLGILGPQLHADVGDk.[V]	N-Term(iTRAQ4plex); K1(iTRAQ4	1	1
[R].iDTINLFPATLFDAYMVAQNPGEWMLScC	N-Term(iTRAQ4plex); C28(Carba	1	1
[R].kAEEEHLGILGPQLHADVGDk.[V]	N-Term(iTRAQ4plex); K1(iTRAQ4	1	1
[R].kAEEEHLGILGPQLHADVGDk.[V]	N-Term(iTRAQ4plex); K1(iTRAQ4	1	1
[R].kAEEEHLGILGPQLHADVGDk.[V]	N-Term(iTRAQ4plex); K1(iTRAQ4	1	1
[R].kAEEEHLGILGPQLHADVGDk.[V]	N-Term(iTRAQ4plex); K1(iTRAQ4	1	1
[R].kAEEEHLGILGPQLHADVGDk.[V]	N-Term(iTRAQ4plex); K1(iTRAQ4	1	1
[R].kAEEEHLGILGPQLHADVGDk.[V]	N-Term(iTRAQ4plex); K1(iTRAQ4	1	1
[R].kAEEEHLGILGPQLHADVGDk.[V]	N-Term(iTRAQ4plex); K1(iTRAQ4	1	1
[R].kAEEEHLGILGPQLHADVGDk.[V]	N-Term(iTRAQ4plex); K1(iTRAQ4	1	1
[R].kAEEEHLGILGPQLHADVGDk.[V]	N-Term(iTRAQ4plex); K1(iTRAQ4	1	1
[R].gVYSSDVFDIFPGTYQTLEMFPR.[T]	N-Term(iTRAQ4plex)	1	1
[R].gVYSSDVFDIFPGTYQTLEMFPR.[T]	N-Term(iTRAQ4plex)	1	1
[R].gVYSSDVFDIFPGTYQTLEMFPR.[T]	N-Term(iTRAQ4plex)	1	1
[R].hYYIAAEEIIWNYAPSGIDIFTk.[E]	N-Term(iTRAQ4plex); K23(iTRAQ	1	1
[K].VNkDDEEFIESNk.[M]	K3(iTRAQ4plex); K13(iTRAQ4ple	1	1
[K].vNKDDEEFIESNk.[M]	N-Term(iTRAQ4plex); K13(iTRAQ	1	1
[K].VNkDDEEFIESNk.[M]	K3(iTRAQ4plex); K13(iTRAQ4ple	1	1
[K].dLYSGLIGPLIVcR.[R]	N-Term(iTRAQ4plex); C13(Carba	1	1
[R].hYYIAAEEIIWNYAPSGIDIFTk.[E]	N-Term(iTRAQ4plex); K23(iTRAQ	1	1

[K].aGLQAFFQVQEcNk.[S]	N-Term(iTRAQ4plex); C12(Carbar	1	1
[K].aGLQAFFQVQEcNk.[S]	N-Term(iTRAQ4plex); C12(Carbar	1	1
[K].mYYSAVDPTk.[D]	N-Term(iTRAQ4plex); M1(Oxidat	1	1
[K].DIASGLIGPLIIck.[K]	C13(Carbamidomethyl); K14(iTR	1	1
[K].gEFYIGSk.[Y]	N-Term(iTRAQ4plex); K8(iTRAQ4	1	1
[R].kAEEEHLGILGPQLHADVGDk.[V]	N-Term(iTRAQ4plex); K1(iTRAQ4	1	1
[R].tPGIWLLHcHVTDHIHAGMETTYTVLQNE	N-Term(iTRAQ4plex); C9(Carbam	1	1
[K].vDkDNEDFQESNR.[M]	N-Term(iTRAQ4plex); K3(iTRAQ4	1	1
[K].VNkDDEEFIESNk.[M]	K3(iTRAQ4plex); K13(iTRAQ4ple	1	1
[R].MFTTAPDQVDkEDEDFQESNk.[M]	K11(iTRAQ4plex); K21(iTRAQ4pl	1	1
[R].sVPPSASHVAPTETFTYEWTVPk.[E]	N-Term(iTRAQ4plex); K23(iTRAQ	1	1
[K].VDkDNEDFQESNR.[M]	K3(iTRAQ4plex)	1	1
[R].eYTDASFTNR.[K]	N-Term(iTRAQ4plex)	1	1
[K].vDkDNEDFQESNR.[M]	N-Term(iTRAQ4plex); K3(iTRAQ4	1	1
[K].vDkDNEDFQESNR.[M]	N-Term(iTRAQ4plex); K3(iTRAQ4	1	1
[R].EYTDASFTNR.[K]		1	1
[K].vNKDDEEFIESNk.[M]	N-Term(iTRAQ4plex); K13(iTRAQ	1	1
[K].dIASGLIGPLIIck.[K]	N-Term(iTRAQ4plex); C13(Carbar	1	1
[K].dNEDFQESNR.[M]	N-Term(iTRAQ4plex)	1	1
[K].EVGPTNADPVcLAk.[M]	C11(Carbamidomethyl); K14(iTR	1	1
[R].qSEDSTFYLGER.[T]	N-Term(iTRAQ4plex)	1	1
[R].kAEEEHLGILGPQLHADVGDk.[V]	N-Term(iTRAQ4plex); K1(iTRAQ4	1	1
[K].tYSDHPEk.[V]	N-Term(iTRAQ4plex); K8(iTRAQ4	1	1
[K].gEFYIGSk.[Y]	N-Term(iTRAQ4plex); K8(iTRAQ4	1	1
[K].aLYLQYTDETFR.[T]	N-Term(iTRAQ4plex)	1	1
[K].mYYSAVDPTk.[D]	N-Term(iTRAQ4plex); K10(iTRAQ	1	1
[K].gAYPLSIEPIGVR.[F]	N-Term(iTRAQ4plex)	1	1
[R].QSEDSTFYLGER.[T]		1	1
[K].mYYSAVDPTk.[D]	N-Term(iTRAQ4plex); M1(Oxidat	1	1
[K].aLYLQYTDETFR.[T]	N-Term(iTRAQ4plex)	1	1
[R].rPYLkVFNPR.[R]	N-Term(iTRAQ4plex); K5(iTRAQ4	1	1
[K].tYSDHPEk.[V]	N-Term(iTRAQ4plex); K8(iTRAQ4	1	1
[R].mFTTAPDQVDkEDEDFQESNk.[M]	N-Term(iTRAQ4plex); M1(Oxidat	1	1
[R].EYTDASFTNR.[K]		1	1
[K].gEFYIGSk.[Y]	N-Term(iTRAQ4plex); K8(iTRAQ4	1	1
[R].QYTDSTFR.[V]		1	1
[R].sVPPSASHVAPTETFTYEWTVPk.[E]	N-Term(iTRAQ4plex); K23(iTRAQ	1	1
[K].vDkDNEDFQESNR.[M]	N-Term(iTRAQ4plex); K3(iTRAQ4	1	1
[R].qSEDSTFYLGER.[T]	N-Term(iTRAQ4plex)	1	1
[K].dIFTGLIGPMk.[I]	N-Term(iTRAQ4plex); K11(iTRAQ	1	1
[R].iYHSHIDAPk.[D]	N-Term(iTRAQ4plex); K10(iTRAQ	1	1
[K].dIFTGLIGPmk.[I]	N-Term(iTRAQ4plex); M10(Oxida	1	1
[R].eYTDASFTNR.[K]	N-Term(iTRAQ4plex)	1	1
[R].eYTDASFTNR.[K]	N-Term(iTRAQ4plex)	1	1
[K].vNkDDEEFIESNk.[M]	N-Term(iTRAQ4plex); K3(iTRAQ4	1	1
[K].mYYSAVDPTk.[D]	N-Term(iTRAQ4plex); K10(iTRAQ	1	1
[K].vNkDDEEFIESNk.[M]	N-Term(iTRAQ4plex); K3(iTRAQ4	1	1
[K].tYcSEPEk.[V]	N-Term(iTRAQ4plex); C3(Carbam	1	1
[R].eYTDASFTNR.[K]	N-Term(iTRAQ4plex)	1	1
[K].vNkDDEEFIESNk.[M]	N-Term(iTRAQ4plex); K3(iTRAQ4	1	1
[R].kAEEEHLGILGPQLHADVGDk.[V]	N-Term(iTRAQ4plex); K1(iTRAQ4	1	1
[R].iYHSHIDAPk.[D]	N-Term(iTRAQ4plex); K10(iTRAQ	1	1

[K].mYYSAVDPTk.[D]	N-Term(iTRAQ4plex); K10(iTRAQ4	1	1
[K].VNkDDEEFIESNk.[M]	K3(iTRAQ4plex); K13(iTRAQ4plex	1	1
[R].kAEEEHLGILGPQLHADVGDk.[V]	N-Term(iTRAQ4plex); K1(iTRAQ4	1	1
[R].mFTTAPDQVDkEDEDFQESNk.[M]	N-Term(iTRAQ4plex); K11(iTRAQ4	1	1
[K].vNKDDEEFIESNk.[M]	N-Term(iTRAQ4plex); K13(iTRAQ4	1	1
[K].eVGPTNADPVcLAk.[M]	N-Term(iTRAQ4plex); C11(Carbam	1	1
[K].VNkDDEEFIESNk.[M]	K3(iTRAQ4plex); K13(iTRAQ4plex	1	1
[R].eYTDASFTNR.[K]	N-Term(iTRAQ4plex)	1	1
[K].dNEDFQESNR.[M]	N-Term(iTRAQ4plex)	1	1
[R].sVPPSASHVAPTETFTYEWTVPk.[E]	N-Term(iTRAQ4plex); K23(iTRAQ4	1	1
[R].sGAGTEDSAcIPWAYYSTVDQVk.[D]	N-Term(iTRAQ4plex); C10(Carbam	1	1
[K].eVGPTNADPVcLAk.[M]	N-Term(iTRAQ4plex); C11(Carbam	1	1
[R].sVPPSASHVAPTETFTYEWTVPk.[E]	N-Term(iTRAQ4plex); K23(iTRAQ4	1	1
[K].gEFYIGSk.[Y]	N-Term(iTRAQ4plex); K8(iTRAQ4	1	1
[K].dDEEFIESNk.[M]	N-Term(iTRAQ4plex); K10(iTRAQ4	1	1
[K].vNkDDEEFIESNk.[M]	N-Term(iTRAQ4plex); K3(iTRAQ4	1	1
[K].tYSDHPEk.[V]	N-Term(iTRAQ4plex); K8(iTRAQ4	1	1
[R].kAEEEHLGILGPQLHADVGDk.[V]	N-Term(iTRAQ4plex); K1(iTRAQ4	1	1
[K].vNkDDEEFIESNk.[M]	N-Term(iTRAQ4plex); K3(iTRAQ4	1	1
[K].tYSDHPEk.[V]	N-Term(iTRAQ4plex); K8(iTRAQ4	1	1
[R].QYTDSTFR.[V]		1	1
[K].vNkDDEEFIESNk.[M]	N-Term(iTRAQ4plex); K3(iTRAQ4	1	1
[R].QYTDSTFR.[V]		1	1
[K].dNEDFQESNR.[M]	N-Term(iTRAQ4plex)	1	1
[K].gEFYIGSk.[Y]	N-Term(iTRAQ4plex); K8(iTRAQ4	1	1
[K].tYcSEPEk.[V]	N-Term(iTRAQ4plex); C3(Carbam	1	1
[K].GAYPLSIEPIGVR.[F]		1	1
[K].tYSDHPEk.[V]	N-Term(iTRAQ4plex); K8(iTRAQ4	1	1
[K].dIFTGLIGPmk.[I]	N-Term(iTRAQ4plex); M10(Oxida	1	1
[K].vNkDDEEFIESNk.[M]	N-Term(iTRAQ4plex); K3(iTRAQ4	1	1
[K].vDkDNEDFQESNR.[M]	N-Term(iTRAQ4plex); K3(iTRAQ4	1	1
[R].QSEDSTFYLGER.[T]		1	1
[R].qSEDSTFYLGER.[T]	N-Term(iTRAQ4plex)	1	1
[K].vNkDDEEFIESNk.[M]	N-Term(iTRAQ4plex); K3(iTRAQ4	1	1
[R].qSEDSTFYLGER.[T]	N-Term(iTRAQ4plex)	1	1
[K].GAYPLSIEPIGVR.[F]		1	1
[K].vDkDNEDFQESNR.[M]	N-Term(iTRAQ4plex); K3(iTRAQ4	1	1
[R].sVPPSASHVAPTETFTYEWTVPk.[E]	N-Term(iTRAQ4plex); K23(iTRAQ4	1	1
[K].tYSDHPEk.[V]	N-Term(iTRAQ4plex); K8(iTRAQ4	1	1
[R].sVPPSASHVAPTETFTYEWTVPk.[E]	N-Term(iTRAQ4plex); K23(iTRAQ4	1	1
[K].dDEEFIESNk.[M]	N-Term(iTRAQ4plex); K10(iTRAQ4	1	1
[K].wYLFGMGNEVDVHAAFFHGQALTNk.[N]	N-Term(iTRAQ4plex); K25(iTRAQ4	1	1
[K].mYYSAVDPTk.[D]	N-Term(iTRAQ4plex); M1(Oxidat	1	1
[R].eYTDASFTNR.[K]	N-Term(iTRAQ4plex)	1	1
[R].IYHSHIDAPk.[D]	K10(iTRAQ4plex)	1	1
[R].rQSEDSTFYLGER.[T]	N-Term(iTRAQ4plex)	1	1
[K].mYYSAVDPTk.[D]	N-Term(iTRAQ4plex); K10(iTRAQ4	1	1
[R].kLYDYcDVPQcAAPSFDcGkPQVEPk.[K]	N-Term(iTRAQ4plex); K1(iTRAQ4	1	1
[R].kLYDYcDVPQcAAPSFDcGkPQVEPk.[K]	N-Term(iTRAQ4plex); K1(iTRAQ4	1	1
[R].kLYDYcDVPQcAAPSFDcGkPQVEPk.[K]	N-Term(iTRAQ4plex); K1(iTRAQ4	1	1
[R].kLYDYcDVPQcAAPSFDcGkPQVEPk.[K]	N-Term(iTRAQ4plex); K1(iTRAQ4	1	1
[R].vQSTELcAGHLAGGTDScQGDSGGPLVcF	N-Term(iTRAQ4plex); C7(Carbam	1	1

[R].vQSTELcAGHLAGGTDScQGDSGGPLVcF	N-Term(iTRAQ4plex); C7(Carbam	1	1
[K].lYDYcDVPQcAAPSFDcGkPQVEPk.[K]	N-Term(iTRAQ4plex); C5(Carbam	1	1
[K].lYDYcDVPQcAAPSFDcGkPQVEPk.[K]	N-Term(iTRAQ4plex); C5(Carbam	1	1
[K].lYDYcDVPQcAAPSFDcGkPQVEPk.[K]	N-Term(iTRAQ4plex); C5(Carbam	1	1
[K].lYDYcDVPQcAAPSFDcGkPQVEPk.[K]	N-Term(iTRAQ4plex); C5(Carbam	1	1
[R].vQSTELcAGHLAGGTDScQGDSGGPLVcF	N-Term(iTRAQ4plex); C7(Carbam	1	1
[K].rWELcDIPR.[C]	N-Term(iTRAQ4plex); C5(Carbam	1	1
[K].rWELcDIPR.[C]	N-Term(iTRAQ4plex); C5(Carbam	1	1
[K].kcQSWSSMTPHR.[H]	N-Term(iTRAQ4plex); K1(iTRAQ4	1	1
[K].wSSTSPHRPR.[F]	N-Term(iTRAQ4plex)	1	1
[K].cQSWSSmTPHR.[H]	N-Term(iTRAQ4plex); C1(Carbam	1	1
[R].hSIFTPETNPR.[A]	N-Term(iTRAQ4plex)	1	1
[K].cQSWSSMTPHR.[H]	N-Term(iTRAQ4plex); C1(Carbam	1	1
[R].hSIFTPETNPR.[A]	N-Term(iTRAQ4plex)	1	1
[K].cQSWSSMTPHR.[H]	N-Term(iTRAQ4plex); C1(Carbam	1	1
[R].hSIFTPETNPR.[A]	N-Term(iTRAQ4plex)	1	1
[R].hSIFTPETNPR.[A]	N-Term(iTRAQ4plex)	1	1
[K].kVYLSEck.[T]	N-Term(iTRAQ4plex); K1(iTRAQ4	1	1
[R].eLRPWcFTTDPNkR.[W]	N-Term(iTRAQ4plex); C6(Carbam	1	1
[R].eLRPWcFTTDPNkR.[W]	N-Term(iTRAQ4plex); C6(Carbam	1	1
[K].vIPAcLPSPNYVVADR.[T]	N-Term(iTRAQ4plex); C5(Carbam	1	1
[K].vIPAcLPSPNYVVADR.[T]	N-Term(iTRAQ4plex); C5(Carbam	1	1
[K].tMSGLEcQAWDSQSPHAHGYIPSk.[F]	N-Term(iTRAQ4plex); C7(Carbam	1	1
[K].vILGAHQEVNLEPHVQEIEVSR.[L]	N-Term(iTRAQ4plex)	1	1
[K].vILGAHQEVNLEPHVQEIEVSR.[L]	N-Term(iTRAQ4plex)	1	1
[K].vILGAHQEVNLEPHVQEIEVSR.[L]	N-Term(iTRAQ4plex)	1	1
[K].vILGAHQEVNLEPHVQEIEVSR.[L]	N-Term(iTRAQ4plex)	1	1
[K].gPWcFTTDPSVR.[W]	N-Term(iTRAQ4plex); C4(Carbam	1	1
[K].vILGAHQEVNLEPHVQEIEVSR.[L]	N-Term(iTRAQ4plex)	1	1
[R].eLRPWcFTTDPNk.[R]	N-Term(iTRAQ4plex); C6(Carbam	1	1
[R].TEcFITGWGETQGTFGAGLLk.[E]	C3(Carbamidomethyl); K21(iTRA	1	1
[R].tEcFITGWGETQGTFGAGLLk.[E]	N-Term(iTRAQ4plex); C3(Carbam	1	1
[R].fGmHFcGGTLISPEWVLTAAHcLEk.[S]	N-Term(iTRAQ4plex); M3(Oxidat	1	1
[K].eQQcVImAENR.[K]	N-Term(iTRAQ4plex); C4(Carbam	1	2
[R].nPDNDPQGPWcYTTDPEk.[R]	N-Term(iTRAQ4plex); C11(Carbar	1	1
[K].eQQcVImAENR.[K]	N-Term(iTRAQ4plex); C4(Carbam	1	2
[K].eQQcVImAENR.[K]	N-Term(iTRAQ4plex); C4(Carbam	1	2
[R].nPDNDPQGPWcYTTDPEk.[R]	N-Term(iTRAQ4plex); C11(Carbar	1	1
[K].eQQcVIMAENR.[K]	N-Term(iTRAQ4plex); C4(Carbam	1	2
[K].eQQcVIMAENR.[K]	N-Term(iTRAQ4plex); C4(Carbam	1	2
[K].EAQLPVIENk.[V]	K10(iTRAQ4plex)	1	1
[K].eQQcVIMAENR.[K]	N-Term(iTRAQ4plex); C4(Carbam	1	2
[R].nPDNDPQGPWcYTTDPEkR.[Y]	N-Term(iTRAQ4plex); C11(Carbar	1	1
[R].tEcFITGWGETQGTFGAGLLk.[E]	N-Term(iTRAQ4plex); C3(Carbam	1	1
[R].nPDNDPQGPWcYTTDPEkR.[Y]	N-Term(iTRAQ4plex); C11(Carbar	1	1
[R].fVTWIEGVMR.[N]	N-Term(iTRAQ4plex)	1	1
[K].eAQLPVIENk.[V]	N-Term(iTRAQ4plex); K10(iTRAQ	1	1
[K].eAQLPVIENk.[V]	N-Term(iTRAQ4plex); K10(iTRAQ	1	1
[K].kVYLSEck.[T]	N-Term(iTRAQ4plex); K1(iTRAQ4	1	1
[R].fGMHFcGGTLISPEWVLTAAHcLEk.[S]	N-Term(iTRAQ4plex); C6(Carbam	1	1
[R].fGMHFcGGTLISPEWVLTAAHcLEk.[S]	N-Term(iTRAQ4plex); C6(Carbam	1	1
[R].tEcFITGWGETQGTFGAGLLk.[E]	N-Term(iTRAQ4plex); C3(Carbam	1	1

[R].tEcFITGWGETQGTFGAGLLk.[E]	N-Term(iTRAQ4plex); C3(Carbam	1	1
[K].QLGAGSIEEcAAk.[C]	C10(Carbamidomethyl); K13(iTR	1	1
[K].qLGAGSIEEcAAk.[C]	N-Term(iTRAQ4plex); C10(Carbar	1	1
[K].qLGAGSIEEcAAk.[C]	N-Term(iTRAQ4plex); C10(Carbar	1	1
[K].qLGAGSIEEcAAk.[C]	N-Term(iTRAQ4plex); C10(Carbar	1	1
[R].NPDNDPQGPWcYTTDPEk.[R]	C11(Carbamidomethyl); K18(iTR	1	1
[K].lSSPAVITDk.[V]	N-Term(iTRAQ4plex); K10(iTRAQ	1	1
[R].nPDGDVGGPWcYTTNPR.[K]	N-Term(iTRAQ4plex); C11(Carbar	1	1
[R].nPDGDVGGPWcYTTNPR.[K]	N-Term(iTRAQ4plex); C11(Carbar	1	1
[R].nPDGDVGGPWcYTTNPR.[K]	N-Term(iTRAQ4plex); C11(Carbar	1	1
[R].cTTPPPSSGPTYQcLk.[G]	N-Term(iTRAQ4plex); C1(Carbam	1	1
[R].fSPATHPSEGLEENYcR.[N]	N-Term(iTRAQ4plex); C16(Carbar	1	1
[R].aTTVTGTPcQDWAAQEPHR.[H]	N-Term(iTRAQ4plex); C9(Carbam	1	1
[R].fSPATHPSEGLEENYcR.[N]	N-Term(iTRAQ4plex); C16(Carbar	1	1
[R].aTTVTGTPcQDWAAQEPHR.[H]	N-Term(iTRAQ4plex); C9(Carbam	1	1
[R].aTTVTGTPcQDWAAQEPHR.[H]	N-Term(iTRAQ4plex); C9(Carbam	1	1
[R].gTSSTTTTGk.[K]	N-Term(iTRAQ4plex); K10(iTRAQ	1	1
[K].cEEDEEFTcR.[A]	N-Term(iTRAQ4plex); C1(Carbam	1	1
[K].eAQLPVIENk.[V]	N-Term(iTRAQ4plex); K10(iTRAQ	1	1
[R].vVGGcVAHPHSWPWQVSLR.[T]	N-Term(iTRAQ4plex); C5(Carbam	1	1
[R].vVGGcVAHPHSWPWQVSLR.[T]	N-Term(iTRAQ4plex); C5(Carbam	1	1
[R].vVGGcVAHPHSWPWQVSLR.[T]	N-Term(iTRAQ4plex); C5(Carbam	1	1
[K].cEEDEEFTcR.[A]	N-Term(iTRAQ4plex); C1(Carbam	1	1
[R].nPDADkGPWcFTTDPSVR.[W]	N-Term(iTRAQ4plex); K6(iTRAQ4	1	1
[K].kQLGAGSIEEcAAk.[C]	N-Term(iTRAQ4plex); K1(iTRAQ4	1	1
[K].LSSPAVITDk.[V]	K10(iTRAQ4plex)	1	1
[R].cTTPPPSSGPTYQcLk.[G]	N-Term(iTRAQ4plex); C1(Carbam	1	1
[K].cEEDEEFTcR.[A]	N-Term(iTRAQ4plex); C1(Carbam	1	1
[R].aPWcHTTNSQVR.[W]	N-Term(iTRAQ4plex); C4(Carbam	1	1
[R].aPWcHTTNSQVR.[W]	N-Term(iTRAQ4plex); C4(Carbam	1	1
[R].WEYcNLk.[K]	C4(Carbamidomethyl); K7(iTRAC	1	1
[K].rAPWcHTTNSQVR.[W]	N-Term(iTRAQ4plex); C5(Carbam	1	1
[R].wELcDIPR.[C]	N-Term(iTRAQ4plex); C4(Carbam	1	1
[K].vILGAHQEVNLEPHVQEIEVSR.[L]	N-Term(iTRAQ4plex)	1	1
[K].eQQcVImAENR.[K]	N-Term(iTRAQ4plex); C4(Carbam	1	2
[R].aFQYHSk.[E]	N-Term(iTRAQ4plex); K7(iTRAQ4	1	3
[K].RAPWcHTTNSQVR.[W]	C5(Carbamidomethyl)	1	1
[K].EQQcVIMAENR.[K]	C4(Carbamidomethyl)	1	2
[R].wELcDIPR.[C]	N-Term(iTRAQ4plex); C4(Carbam	1	1
[R].tEcFITGWGETQGTFGAGLLk.[E]	N-Term(iTRAQ4plex); C3(Carbam	1	1
[K].lSSPAVITDk.[V]	N-Term(iTRAQ4plex); K10(iTRAQ	1	1
[K].nLDENYcR.[N]	N-Term(iTRAQ4plex); C7(Carbam	1	1
[K].nLDENYcR.[N]	N-Term(iTRAQ4plex); C7(Carbam	1	1
[R].mRDVVLFEk.[-K]	N-Term(iTRAQ4plex); K9(iTRAQ4	1	2
[R].vVGGcVAHPHSWPWQVSLR.[T]	N-Term(iTRAQ4plex); C5(Carbam	1	1
[R].kDIALLk.[L]	N-Term(iTRAQ4plex); K1(iTRAQ4	1	1
[R].tPENFPck.[N]	N-Term(iTRAQ4plex); C7(Carbam	1	1
[R].vVGGcVAHPHSWPWQVSLR.[T]	N-Term(iTRAQ4plex); C5(Carbam	1	1
[K].nLDENYcR.[N]	N-Term(iTRAQ4plex); C7(Carbam	1	1
[R].aFQYHSk.[E]	N-Term(iTRAQ4plex); K7(iTRAQ4	1	3
[R].aPWcHTTNSQVR.[W]	N-Term(iTRAQ4plex); C4(Carbam	1	1
[K].rAPWcHTTNSQVR.[W]	N-Term(iTRAQ4plex); C5(Carbam	1	1

[R].hSIFTPETNPR.[A]	N-Term(iTRAQ4plex)	1	1
[R].hSIFTPETNPR.[A]	N-Term(iTRAQ4plex)	1	1
[R].nPDNDPQGPWcYTTDPEk.[R]	N-Term(iTRAQ4plex); C11(Carbar	1	1
[R].wELcDIPR.[C]	N-Term(iTRAQ4plex); C4(Carbam	1	1
[R].NPDNDPQGPWcYTTDPEk.[R]	C11(Carbamidomethyl); K18(iTR	1	1
[K].nLDENYcR.[N]	N-Term(iTRAQ4plex); C7(Carbam	1	1
[R].nPDNDPQGPWcYTTDPEk.[R]	N-Term(iTRAQ4plex); C11(Carbar	1	1
[K].cEEDEEFTcR.[A]	N-Term(iTRAQ4plex); C1(Carbam	1	1
[R].wEYcNLk.[K]	N-Term(iTRAQ4plex); C4(Carbam	1	1
[R].hSIFTPETNPR.[A]	N-Term(iTRAQ4plex)	1	1
[K].cEEDEEFTcR.[A]	N-Term(iTRAQ4plex); C1(Carbam	1	1
[K].QLGAGSIEEcAAk.[C]	C10(Carbamidomethyl); K13(iTR	1	1
[R].dVVLFEk.[-K]	N-Term(iTRAQ4plex); K7(iTRAQ4	1	2
[K].cEEDEEFTcR.[A]	C1(Carbamidomethyl); C9(Carba	1	1
[K].kcQSWSSMTPHR.[H]	N-Term(iTRAQ4plex); K1(iTRAQ4	1	1
[R].dVVLFEk.[-K]	N-Term(iTRAQ4plex); K7(iTRAQ4	1	2
[R].aFQYHSk.[E]	N-Term(iTRAQ4plex); K7(iTRAQ4	1	3
[R].aPWcHTTNSQVR.[W]	N-Term(iTRAQ4plex); C4(Carbam	1	1
[K].eAQLPVIENk.[V]	N-Term(iTRAQ4plex); K10(iTRAQ	1	1
[R].wELcDIPR.[C]	N-Term(iTRAQ4plex); C4(Carbam	1	1
[K].EQQcVIMAENR.[K]	C4(Carbamidomethyl)	1	2
[R].nPDNDPQGPWcYTTDPEkR.[Y]	N-Term(iTRAQ4plex); C11(Carbar	1	1
[K].nLDENYcR.[N]	N-Term(iTRAQ4plex); C7(Carbam	1	1
[K].rWELcDIPR.[C]	N-Term(iTRAQ4plex); C5(Carbam	1	1
[R].wEYcNLk.[K]	N-Term(iTRAQ4plex); C4(Carbam	1	1
[K].nGITcQk.[W]	N-Term(iTRAQ4plex); C5(Carbam	1	1
[K].eQQcVImAENR.[K]	N-Term(iTRAQ4plex); C4(Carbam	1	2
[K].NLDENYcR.[N]	C7(Carbamidomethyl)	1	1
[R].HSIFTPETNPR.[A]		1	1
[R].aFQYHSk.[E]	N-Term(iTRAQ4plex); K7(iTRAQ4	1	3
[K].wSSTSPHRPR.[F]	N-Term(iTRAQ4plex)	1	1
[R].fVTWIEGVMR.[N]	N-Term(iTRAQ4plex)	1	1
[R].eLRPWcFTTDPNk.[R]	N-Term(iTRAQ4plex); C6(Carbam	1	1
[R].nPDNDPQGPWcYTTDPEk.[R]	N-Term(iTRAQ4plex); C11(Carbar	1	1
[K].eQQcVImAENR.[K]	N-Term(iTRAQ4plex); C4(Carbam	1	2
[R].VVGGcVAHPHSWPWQVSLR.[T]	C5(Carbamidomethyl)	1	1
[R].hSIFTPETNPR.[A]	N-Term(iTRAQ4plex)	1	1
[R].lFLEPTRk.[D]	N-Term(iTRAQ4plex); K8(iTRAQ4	1	1
[R].WELcDIPR.[C]	C4(Carbamidomethyl)	1	1
[R].tPENFPck.[N]	N-Term(iTRAQ4plex); C7(Carbam	1	1
[K].tMSGLEcQAWDSQSPHAHGYIPSk.[F]	N-Term(iTRAQ4plex); C7(Carbam	1	1
[R].nPDNDPQGPWcYTTDPEk.[R]	N-Term(iTRAQ4plex); C11(Carbar	1	1
[K].nLDENYcR.[N]	N-Term(iTRAQ4plex); C7(Carbam	1	1
[K].NLDENYcR.[N]	C7(Carbamidomethyl)	1	1
[K].lSSPAVITDk.[V]	N-Term(iTRAQ4plex); K10(iTRAQ	1	1
[K].lSSPAVITDk.[V]	N-Term(iTRAQ4plex); K10(iTRAQ	1	1
[K].cEEDEEFTcR.[A]	N-Term(iTRAQ4plex); C1(Carbam	1	1
[R].aFQYHSk.[E]	N-Term(iTRAQ4plex); K7(iTRAQ4	1	3
[R].nPDNDPQGPWcYTTDPEk.[R]	N-Term(iTRAQ4plex); C11(Carbar	1	1
[K].kVYLSEck.[T]	N-Term(iTRAQ4plex); K1(iTRAQ4	1	1
[R].tPENFPck.[N]	N-Term(iTRAQ4plex); C7(Carbam	1	1
[R].nPDADkGPWcFTTDPSVR.[W]	N-Term(iTRAQ4plex); K6(iTRAQ4	1	1

[K].nLDENYcR.[N]	N-Term(iTRAQ4plex); C7(Carbam	1	1
[R].rPcFSALEVDETYVPk.[E]	N-Term(iTRAQ4plex); C3(Carbam	1	1
[R].rPcFSALEVDETYVPk.[E]	N-Term(iTRAQ4plex); C3(Carbam	1	1
[K].aVmDDFAAFVEk.[C]	N-Term(iTRAQ4plex); M3(Oxidat	1	1
[K].aVmDDFAAFVEk.[C]	N-Term(iTRAQ4plex); M3(Oxidat	1	1
[K].aVmDDFAAFVEk.[C]	N-Term(iTRAQ4plex); M3(Oxidat	1	1
[K].qNcELFEQLGEYk.[F]	N-Term(iTRAQ4plex); C3(Carbam	1	1
[K].aVmDDFAAFVEk.[C]	N-Term(iTRAQ4plex); M3(Oxidat	1	1
[K].aVmDDFAAFVEk.[C]	N-Term(iTRAQ4plex); M3(Oxidat	1	1
[R].yTKkVPQVSTPTLVEVSR.[N]	N-Term(iTRAQ4plex); K4(iTRAQ4	1	1
[R].yTkKVPQVSTPTLVEVSR.[N]	N-Term(iTRAQ4plex); K3(iTRAQ4	1	1
[R].YTkkVPQVSTPTLVEVSR.[N]	K3(iTRAQ4plex); K4(iTRAQ4plex)	1	1
[K].qTALVELVk.[H]	N-Term(iTRAQ4plex); K9(iTRAQ4	1	1
[K].vHTEccHGDLLEcADDR.[A]	N-Term(iTRAQ4plex); C5(Carbam	1	1
[K].tcVADESAENcDk.[S]	N-Term(iTRAQ4plex); C2(Carbam	1	1
[R].lkcASLQk.[F]	N-Term(iTRAQ4plex); K2(iTRAQ4	1	1
[K].TcVADESAENcDk.[S]	C2(Carbamidomethyl); C11(Carb	1	1
[K].aDDkETcFAEEGk.[K]	N-Term(iTRAQ4plex); K4(iTRAQ4	1	1
[K].kYLYEIAR.[R]	N-Term(iTRAQ4plex); K1(iTRAQ4	1	1
[K].aVmDDFAAFVEk.[C]	N-Term(iTRAQ4plex); M3(Oxidat	1	1
[K].aVmDDFAAFVEk.[C]	N-Term(iTRAQ4plex); M3(Oxidat	1	1
[K].aVmDDFAAFVEk.[C]	N-Term(iTRAQ4plex); M3(Oxidat	1	1
[K].aVmDDFAAFVEk.[C]	N-Term(iTRAQ4plex); M3(Oxidat	1	1
[K].aVmDDFAAFVEk.[C]	N-Term(iTRAQ4plex); M3(Oxidat	1	1
[K].aVMDDFAAFVEk.[C]	N-Term(iTRAQ4plex); K12(iTRAQ	1	1
[K].sHcIAEVENDEMPADLPSLAADFVESk.[D]	N-Term(iTRAQ4plex); C3(Carbam	1	1
[K].aVMDDFAAFVEk.[C]	N-Term(iTRAQ4plex); K12(iTRAQ	1	1
[K].aVMDDFAAFVEk.[C]	N-Term(iTRAQ4plex); K12(iTRAQ	1	1
[K].aVMDDFAAFVEk.[C]	N-Term(iTRAQ4plex); K12(iTRAQ	1	1
[K].aVMDDFAAFVEk.[C]	N-Term(iTRAQ4plex); K12(iTRAQ	1	1
[K].aVMDDFAAFVEk.[C]	N-Term(iTRAQ4plex); K12(iTRAQ	1	1
[K].aVMDDFAAFVEk.[C]	N-Term(iTRAQ4plex); K12(iTRAQ	1	1
[K].aVMDDFAAFVEk.[C]	N-Term(iTRAQ4plex); K12(iTRAQ	1	1
[K].aVMDDFAAFVEk.[C]	N-Term(iTRAQ4plex); K12(iTRAQ	1	1
[K].aVMDDFAAFVEk.[C]	N-Term(iTRAQ4plex); K12(iTRAQ	1	1
[R].rHPDYSVVLLLR.[L]	N-Term(iTRAQ4plex)	1	1
[R].RHPDYSVVLLLR.[L]		1	1
[R].rHPDYSVVLLLR.[L]	N-Term(iTRAQ4plex)	1	1
[R].rHPDYSVVLLLR.[L]	N-Term(iTRAQ4plex)	1	1
[R].rHPDYSVVLLLR.[L]	N-Term(iTRAQ4plex)	1	1
[R].rHPDYSVVLLLR.[L]	N-Term(iTRAQ4plex)	1	1
[R].rHPDYSVVLLLR.[L]	N-Term(iTRAQ4plex)	1	1
[R].rHPDYSVVLLLR.[L]	N-Term(iTRAQ4plex)	1	1
[R].rPcFSALEVDETYVPk.[E]	N-Term(iTRAQ4plex); C3(Carbam	1	1
[R].rPcFSALEVDETYVPk.[E]	N-Term(iTRAQ4plex); C3(Carbam	1	1
[R].rPcFSALEVDETYVPk.[E]	N-Term(iTRAQ4plex); C3(Carbam	1	1
[R].rPcFSALEVDETYVPk.[E]	N-Term(iTRAQ4plex); C3(Carbam	1	1
[R].RPcFSALEVDETYVPk.[E]	C3(Carbamidomethyl); K16(iTRA	1	1
[R].rPcFSALEVDETYVPk.[E]	N-Term(iTRAQ4plex); C3(Carbam	1	1
[R].rPcFSALEVDETYVPk.[E]	N-Term(iTRAQ4plex); C3(Carbam	1	1
[R].rPcFSALEVDETYVPk.[E]	N-Term(iTRAQ4plex); C3(Carbam	1	1
[R].rPcFSALEVDETYVPk.[E]	N-Term(iTRAQ4plex); C3(Carbam	1	1

[K].lVNEVTEFAk.[T]	N-Term(iTRAQ4plex); K10(iTRAQ4	1	1
[K].lVNEVTEFAk.[T]	N-Term(iTRAQ4plex); K10(iTRAQ4	1	1
[K].lVNEVTEFAk.[T]	N-Term(iTRAQ4plex); K10(iTRAQ4	1	1
[K].lVNEVTEFAk.[T]	N-Term(iTRAQ4plex); K10(iTRAQ4	1	1
[K].aVmDDFAAFVEk.[C]	N-Term(iTRAQ4plex); M3(Oxidat	1	1
[K].qNcELFEQLGEYk.[F]	N-Term(iTRAQ4plex); C3(Carbam	1	1
[K].qNcELFEQLGEYk.[F]	N-Term(iTRAQ4plex); C3(Carbam	1	1
[K].qNcELFEQLGEYk.[F]	N-Term(iTRAQ4plex); C3(Carbam	1	1
[R].RHPDYSVVLLLR.[L]		1	1
[K].kVPQVSTPTLVEVSR.[N]	N-Term(iTRAQ4plex); K1(iTRAQ4	1	1
[K].kQTALVELVk.[H]	N-Term(iTRAQ4plex); K10(iTRAQ4	1	1
[K].kQTALVELVk.[H]	K1(iTRAQ4plex); K10(iTRAQ4plex	1	1
[K].kVPQVSTPTLVEVSR.[N]	N-Term(iTRAQ4plex); K1(iTRAQ4	1	1
[K].tcVADESAENcDk.[S]	N-Term(iTRAQ4plex); C2(Carbam	1	1
[K].sHcIAEVENDEMPADLPSLAADFVESk.[D]	N-Term(iTRAQ4plex); C3(Carbam	1	1
[K].sHcIAEVENDEMPADLPSLAADFVESk.[D]	N-Term(iTRAQ4plex); C3(Carbam	1	1
[R].rHPDYSVVLLLR.[L]	N-Term(iTRAQ4plex)	1	1
[K].sHcIAEVENDEmPADLPSLAADFVESk.[D]	N-Term(iTRAQ4plex); C3(Carbam	1	1
[K].sHcIAEVENDEmPADLPSLAADFVESk.[D]	N-Term(iTRAQ4plex); C3(Carbam	1	1
[K].sHcIAEVENDEmPADLPSLAADFVESk.[D]	N-Term(iTRAQ4plex); C3(Carbam	1	1
[K].vHTEccHGDLLEcADDR.[A]	N-Term(iTRAQ4plex); C5(Carbam	1	1
[K].kVPQVSTPTLVEVSR.[N]	N-Term(iTRAQ4plex); K1(iTRAQ4	1	1
[K].kVPQVSTPTLVEVSR.[N]	N-Term(iTRAQ4plex); K1(iTRAQ4	1	1
[K].kVPQVSTPTLVEVSR.[N]	N-Term(iTRAQ4plex); K1(iTRAQ4	1	1
[K].kVPQVSTPTLVEVSR.[N]	N-Term(iTRAQ4plex); K1(iTRAQ4	1	1
[K].kVPQVSTPTLVEVSR.[N]	N-Term(iTRAQ4plex); K1(iTRAQ4	1	1
[K].kVPQVSTPTLVEVSR.[N]	N-Term(iTRAQ4plex); K1(iTRAQ4	1	1
[K].kVPQVSTPTLVEVSR.[N]	N-Term(iTRAQ4plex); K1(iTRAQ4	1	1
[K].tcVADESAENcDk.[S]	N-Term(iTRAQ4plex); C2(Carbam	1	1
[K].tcVADESAENcDk.[S]	N-Term(iTRAQ4plex); C2(Carbam	1	1
[K].vHTEccHGDLLEcADDR.[A]	N-Term(iTRAQ4plex); C5(Carbam	1	1
[K].sLHTLFGDk.[L]	N-Term(iTRAQ4plex); K9(iTRAQ4	1	1
[K].sLHTLFGDk.[L]	N-Term(iTRAQ4plex); K9(iTRAQ4	1	1
[K].sLHTLFGDk.[L]	N-Term(iTRAQ4plex); K9(iTRAQ4	1	1
[K].sLHTLFGDk.[L]	N-Term(iTRAQ4plex); K9(iTRAQ4	1	1
[K].sLHTLFGDk.[L]	N-Term(iTRAQ4plex); K9(iTRAQ4	1	1
[K].sLHTLFGDk.[L]	N-Term(iTRAQ4plex); K9(iTRAQ4	1	1
[K].sLHTLFGDk.[L]	N-Term(iTRAQ4plex); K9(iTRAQ4	1	1
[K].sLHTLFGDk.[L]	N-Term(iTRAQ4plex); K9(iTRAQ4	1	1
[R].hPDYSVVLLLR.[L]	N-Term(iTRAQ4plex)	1	1
[K].vPQVSTPTLVEVSR.[N]	N-Term(iTRAQ4plex)	1	1
[R].rPcFSALEVDETYVPk.[E]	N-Term(iTRAQ4plex); C3(Carbam	1	1
[K].vPQVSTPTLVEVSR.[N]	N-Term(iTRAQ4plex)	1	1
[K].kVPQVSTPTLVEVSR.[N]	K1(iTRAQ4plex)	1	1
[K].kVPQVSTPTLVEVSR.[N]	N-Term(iTRAQ4plex); K1(iTRAQ4	1	1
[K].kQTALVELVk.[H]	N-Term(iTRAQ4plex); K1(iTRAQ4	1	1
[K].kQTALVELVk.[H]	N-Term(iTRAQ4plex); K1(iTRAQ4	1	1
[K].kQTALVELVk.[H]	N-Term(iTRAQ4plex); K1(iTRAQ4	1	1
[K].kQTALVELVk.[H]	N-Term(iTRAQ4plex); K1(iTRAQ4	1	1
[R].YkAAFTEccQAADk.[A]	K2(iTRAQ4plex); C8(Carbamidon	1	1
[R].yKAAFTEccQAADk.[A]	N-Term(iTRAQ4plex); C8(Carbam	1	1
[K].kVPQVSTPTLVEVSR.[N]	N-Term(iTRAQ4plex)	1	1

[K].tcVADESAENcDk.[S]	N-Term(iTRAQ4plex); C2(Carbam	1	1
[K].kVPQVSTPTLVEVSR.[N]	N-Term(iTRAQ4plex); K1(iTRAQ4	1	1
[K].kVPQVSTPTLVEVSR.[N]	N-Term(iTRAQ4plex); K1(iTRAQ4	1	1
[K].kVPQVSTPTLVEVSR.[N]	N-Term(iTRAQ4plex); K1(iTRAQ4	1	1
[K].kVPQVSTPTLVEVSR.[N]	N-Term(iTRAQ4plex); K1(iTRAQ4	1	1
[R].lVRPEVDVMcTAFHDNEETFLk.[K]	N-Term(iTRAQ4plex); C10(Carbar	1	1
[R].lVRPEVDVMcTAFHDNEETFLk.[K]	N-Term(iTRAQ4plex); C10(Carbar	1	1
[R].lVRPEVDVMcTAFHDNEETFLk.[K]	N-Term(iTRAQ4plex); C10(Carbar	1	1
[R].lVRPEVDVMcTAFHDNEETFLk.[K]	N-Term(iTRAQ4plex); C10(Carbar	1	1
[K].vFDEFKPLVEEPQNLIk.[Q]	N-Term(iTRAQ4plex); K17(iTRAQ4	1	1
[K].VFDEFkPLVEEPQNLIk.[Q]	K6(iTRAQ4plex); K17(iTRAQ4plex	1	1
[R].lVRPEVDVMcTAFHDNEETFLk.[K]	N-Term(iTRAQ4plex); C10(Carbar	1	1
[R].lVRPEVDVMcTAFHDNEETFLk.[K]	N-Term(iTRAQ4plex); C10(Carbar	1	1
[R].lVRPEVDVMcTAFHDNEETFLk.[K]	N-Term(iTRAQ4plex); C10(Carbar	1	1
[R].lVRPEVDVMcTAFHDNEETFLk.[K]	N-Term(iTRAQ4plex); C10(Carbar	1	1
[R].lVRPEVDVMcTAFHDNEETFLk.[K]	N-Term(iTRAQ4plex); C10(Carbar	1	1
[R].lVRPEVDVMcTAFHDNEETFLk.[K]	N-Term(iTRAQ4plex); C10(Carbar	1	1
[R].lVRPEVDVMcTAFHDNEETFLk.[K]	N-Term(iTRAQ4plex); C10(Carbar	1	1
[R].lVRPEVDVMcTAFHDNEETFLk.[K]	N-Term(iTRAQ4plex); C10(Carbar	1	1
[K].vFDEFkPLVEEPQNLIk.[Q]	N-Term(iTRAQ4plex); K6(iTRAQ4	1	1
[K].vFDEFkPLVEEPQNLIk.[Q]	N-Term(iTRAQ4plex); K6(iTRAQ4	1	1
[K].vFDEFkPLVEEPQNLIk.[Q]	N-Term(iTRAQ4plex); K6(iTRAQ4	1	1
[K].vFDEFkPLVEEPQNLIk.[Q]	N-Term(iTRAQ4plex); K6(iTRAQ4	1	1
[K].vFDEFkPLVEEPQNLIk.[Q]	N-Term(iTRAQ4plex); K6(iTRAQ4	1	1
[K].vFDEFkPLVEEPQNLIk.[Q]	N-Term(iTRAQ4plex); K6(iTRAQ4	1	1
[K].vFDEFkPLVEEPQNLIk.[Q]	N-Term(iTRAQ4plex); K6(iTRAQ4	1	1
[R].lVRPEVDVmcTAFHDNEETFLk.[K]	N-Term(iTRAQ4plex); M9(Oxidat	1	1
[K].eFNAETFTFHADIcTLSEk.[E]	N-Term(iTRAQ4plex); C14(Carbar	1	1
[K].eFNAETFTFHADIcTLSEk.[E]	N-Term(iTRAQ4plex); C14(Carbar	1	1
[K].eFNAETFTFHADIcTLSEk.[E]	N-Term(iTRAQ4plex); C14(Carbar	1	1
[R].LVRPEVDVMcTAFHDNEETFLk.[K]	C10(Carbamidomethyl); K22(iTR	1	1
[K].eFNAETFTFHADIcTLSEk.[E]	N-Term(iTRAQ4plex); C14(Carbar	1	1
[K].eFNAETFTFHADIcTLSEk.[E]	N-Term(iTRAQ4plex); C14(Carbar	1	1
[K].eFNAETFTFHADIcTLSEk.[E]	N-Term(iTRAQ4plex); C14(Carbar	1	1
[R].hPYFYAPELLFFAk.[R]	N-Term(iTRAQ4plex); K14(iTRAQ4	1	1
[K].aDDkETcFAEEGkk.[L]	N-Term(iTRAQ4plex); K4(iTRAQ4	1	1
[K].AAFTEccQAADk.[A]	C6(Carbamidomethyl); C7(Carba	1	1
[R].hPYFYAPELLFFAk.[R]	N-Term(iTRAQ4plex); K14(iTRAQ4	1	1
[K].VHTEccHGDLLEcADDR.[A]	C5(Carbamidomethyl); C6(Carba	1	1
[K].VHTEccHGDLLEcADDR.[A]	C5(Carbamidomethyl); C6(Carba	1	1
[K].VHTEccHGDLLEcADDR.[A]	C5(Carbamidomethyl); C6(Carba	1	1
[K].lDELRDEGk.[A]	N-Term(iTRAQ4plex); K9(iTRAQ4	1	1
[K].tYETTLEk.[C]	N-Term(iTRAQ4plex); K8(iTRAQ4	1	1
[R].nEcFLQHk.[D]	N-Term(iTRAQ4plex); C3(Carbam	1	1
[K].ccAAADPHEcYAk.[V]	N-Term(iTRAQ4plex); C1(Carbam	1	1
[K].ccAAADPHEcYAk.[V]	N-Term(iTRAQ4plex); C1(Carbam	1	1
[K].ccAAADPHEcYAk.[V]	N-Term(iTRAQ4plex); C1(Carbam	1	1
[K].ccTESLVNR.[R]	N-Term(iTRAQ4plex); C1(Carbam	1	1
[K].tcVADESAENcDk.[S]	N-Term(iTRAQ4plex); C2(Carbam	1	1
[K].aAFTEccQAADk.[A]	N-Term(iTRAQ4plex); C6(Carbam	1	1
[K].aAFTEccQAADk.[A]	N-Term(iTRAQ4plex); C6(Carbam	1	1
[K].eccEkPLLEk.[S]	N-Term(iTRAQ4plex); C2(Carbam	1	1

[K].lVAASQAALGL.[-]	N-Term(iTRAQ4plex)	1	1
[R].rHPYFYAPELLFFAk.[R]	N-Term(iTRAQ4plex); K15(iTRAQ	1	1
[R].rHPYFYAPELLFFAk.[R]	N-Term(iTRAQ4plex); K15(iTRAQ	1	1
[R].rHPYFYAPELLFFAk.[R]	N-Term(iTRAQ4plex); K15(iTRAQ	1	1
[R].nEcFLQHk.[D]	N-Term(iTRAQ4plex); C3(Carbam	1	1
[R].rHPYFYAPELLFFAk.[R]	N-Term(iTRAQ4plex); K15(iTRAQ	1	1
[R].rHPYFYAPELLFFAk.[R]	N-Term(iTRAQ4plex); K15(iTRAQ	1	1
[R].RHPYFYAPELLFFAk.[R]	K15(iTRAQ4plex)	1	1
[K].yIcENQDSISSk.[L]	N-Term(iTRAQ4plex); C3(Carbam	1	1
[K].yIcENQDSISSk.[L]	N-Term(iTRAQ4plex); C3(Carbam	1	1
[K].yIcENQDSISSk.[L]	N-Term(iTRAQ4plex); C3(Carbam	1	1
[K].dLGEENFk.[A]	N-Term(iTRAQ4plex); K8(iTRAQ4	1	1
[K].dLGEENFk.[A]	N-Term(iTRAQ4plex); K8(iTRAQ4	1	1
[K].yIcENQDSISSk.[L]	N-Term(iTRAQ4plex); C3(Carbam	1	1
[K].vHTEccHGDLLEcADDR.[A]	N-Term(iTRAQ4plex); C5(Carbam	1	1
[K].vHTEccHGDLLEcADDR.[A]	N-Term(iTRAQ4plex); C5(Carbam	1	1
[K].vHTEccHGDLLEcADDR.[A]	N-Term(iTRAQ4plex); C5(Carbam	1	1
[K].vHTEccHGDLLEcADDR.[A]	N-Term(iTRAQ4plex); C5(Carbam	1	1
[K].vHTEccHGDLLEcADDR.[A]	N-Term(iTRAQ4plex); C5(Carbam	1	1
[K].lVAASQAALGL.[-]	N-Term(iTRAQ4plex)	1	1
[K].vHTEccHGDLLEcADDR.[A]	N-Term(iTRAQ4plex); C5(Carbam	1	1
[K].aEFAEVSk.[L]	N-Term(iTRAQ4plex); K8(iTRAQ4	1	1
[K].vHTEccHGDLLEcADDR.[A]	N-Term(iTRAQ4plex); C5(Carbam	1	1
[K].vHTEccHGDLLEcADDR.[A]	N-Term(iTRAQ4plex); C5(Carbam	1	1
[K].lVAASQAALGL.[-]	N-Term(iTRAQ4plex)	1	1
[K].vHTEccHGDLLEcADDR.[A]	N-Term(iTRAQ4plex); C5(Carbam	1	1
[K].tcVADESAENcDk.[S]	N-Term(iTRAQ4plex); C2(Carbam	1	1
[R].fkDLGEENFk.[A]	N-Term(iTRAQ4plex); K2(iTRAQ4	1	1
[K].tcVADESAENcDk.[S]	N-Term(iTRAQ4plex); C2(Carbam	1	1
[K].ccAAADPHEcYAk.[V]	C1(Carbamidomethyl); C2(Carba	1	1
[K].tcVADESAENcDk.[S]	N-Term(iTRAQ4plex); C2(Carbam	1	1
[K].tcVADESAENcDk.[S]	N-Term(iTRAQ4plex); C2(Carbam	1	1
[R].fkDLGEENFk.[A]	N-Term(iTRAQ4plex); K2(iTRAQ4	1	1
[K].ccTESLVNR.[R]	C1(Carbamidomethyl); C2(Carba	1	1
[K].eTcFAEEGkk.[L]	N-Term(iTRAQ4plex); C3(Carbam	1	1
[K].eTcFAEEGk.[K]	N-Term(iTRAQ4plex); C3(Carbam	1	1
[K].eTcFAEEGk.[K]	N-Term(iTRAQ4plex); C3(Carbam	1	1
[K].eTcFAEEGk.[K]	N-Term(iTRAQ4plex); C3(Carbam	1	1
[R].eTYGEmADccAk.[Q]	N-Term(iTRAQ4plex); M6(Oxidat	1	1
[K].TcVADESAENcDk.[S]	C2(Carbamidomethyl); C11(Carb	1	1
[R].eTYGEmADccAk.[Q]	N-Term(iTRAQ4plex); M6(Oxidat	1	1
[R].eTYGEmADccAk.[Q]	N-Term(iTRAQ4plex); M6(Oxidat	1	1
[K].eTcFAEEGk.[K]	N-Term(iTRAQ4plex); C3(Carbam	1	1
[K].kLVAASQAALGL.[-]	N-Term(iTRAQ4plex); K1(iTRAQ4	1	1
[K].tcVADESAENcDk.[S]	N-Term(iTRAQ4plex); C2(Carbam	1	1
[K].tcVADESAENcDk.[S]	N-Term(iTRAQ4plex); C2(Carbam	1	1
[K].dVFLGMFLYEYAR.[R]	N-Term(iTRAQ4plex)	1	1
[K].tcVADESAENcDk.[S]	N-Term(iTRAQ4plex); C2(Carbam	1	1
[R].mPcAEDYLSVVLNQLcVLHEk.[T]	N-Term(iTRAQ4plex); M1(Oxidat	1	1
[K].LkEccEkPLLEk.[S]	K2(iTRAQ4plex); C4(Carbamidon	1	1
[K].aDDkETcFAEEGk.[K]	N-Term(iTRAQ4plex); K4(iTRAQ4	1	1
[R].mPcAEDYLSVVLNQLcVLHEk.[T]	N-Term(iTRAQ4plex); C3(Carbam	1	1

[R].mPcAEDYLSVVLNQLcVLHEk.[T]	N-Term(iTRAQ4plex); C3(Carbam	1	1
[K].aDDkETcFAEEGk.[K]	N-Term(iTRAQ4plex); K4(iTRAQ4	1	1
[K].aDDkETcFAEEGk.[K]	N-Term(iTRAQ4plex); K4(iTRAQ4	1	1
[K].aDDkETcFAEEGk.[K]	N-Term(iTRAQ4plex); K4(iTRAQ4	1	1
[K].aDDkETcFAEEGk.[K]	N-Term(iTRAQ4plex); K4(iTRAQ4	1	1
[K].aDDkETcFAEEGk.[K]	N-Term(iTRAQ4plex); K4(iTRAQ4	1	1
[K].aDDkETcFAEEGk.[K]	N-Term(iTRAQ4plex); K4(iTRAQ4	1	1
[R].hPDYSVVLLLR.[L]	N-Term(iTRAQ4plex)	1	1
[K].aDDkETcFAEEGk.[K]	N-Term(iTRAQ4plex); K4(iTRAQ4	1	1
[R].eTYGEMADccAk.[Q]	N-Term(iTRAQ4plex); C9(Carbam	1	1
[R].eTYGEMADccAk.[Q]	N-Term(iTRAQ4plex); C9(Carbam	1	1
[R].eTYGEMADccAk.[Q]	N-Term(iTRAQ4plex); C9(Carbam	1	1
[R].eTYGEMADccAk.[Q]	N-Term(iTRAQ4plex); C9(Carbam	1	1
[R].eTYGEMADccAk.[Q]	N-Term(iTRAQ4plex); C9(Carbam	1	1
[R].eTYGEMADccAk.[Q]	N-Term(iTRAQ4plex); C9(Carbam	1	1
[R].eTYGEMADccAk.[Q]	N-Term(iTRAQ4plex); C9(Carbam	1	1
[K].aDDkETcFAEEGk.[K]	N-Term(iTRAQ4plex); K4(iTRAQ4	1	1
[K].aDDkETcFAEEGk.[K]	N-Term(iTRAQ4plex); K4(iTRAQ4	1	1
[K].aDDkETcFAEEGk.[K]	N-Term(iTRAQ4plex); K4(iTRAQ4	1	1
[R].ETYGEMADccAk.[Q]	C9(Carbamidomethyl); C10(Carb	1	1
[K].aDDkETcFAEEGk.[K]	N-Term(iTRAQ4plex); K4(iTRAQ4	1	1
[K].aDDkETcFAEEGk.[K]	N-Term(iTRAQ4plex); K4(iTRAQ4	1	1
[R].eTYGEMADccAk.[Q]	N-Term(iTRAQ4plex); C9(Carbam	1	1
[K].eccEkPLLEk.[S]	N-Term(iTRAQ4plex); C2(Carbam	1	1
[R].RHPDYSVVLLLR.[L]		1	1
[K].aDDkETcFAEEGk.[K]	N-Term(iTRAQ4plex); K4(iTRAQ4	1	1
[K].lVNEVTEFAk.[T]	N-Term(iTRAQ4plex); K10(iTRAQ	1	1
[K].lVTDLTk.[V]	N-Term(iTRAQ4plex); K7(iTRAQ4	1	1
[K].eTcFAEEGk.[K]	N-Term(iTRAQ4plex); C3(Carbam	1	1
[K].aVmDDFAAFVEk.[C]	N-Term(iTRAQ4plex); M3(Oxidat	1	1
[K].lVTDLTk.[V]	N-Term(iTRAQ4plex); K7(iTRAQ4	1	1
[K].AEFAEVSk.[L]	K8(iTRAQ4plex)	1	1
[R].nEcFLQHk.[D]	N-Term(iTRAQ4plex); C3(Carbam	1	1
[K].kYLYEIAR.[R]	N-Term(iTRAQ4plex); K1(iTRAQ4	1	1
[K].AVMDDFAAFVEk.[C]	K12(iTRAQ4plex)	1	1
[K].lVTDLTk.[V]	N-Term(iTRAQ4plex); K7(iTRAQ4	1	1
[K].ccAAADPHEcYAk.[V]	C1(Carbamidomethyl); C2(Carba	1	1
[K].sLHTLFGDk.[L]	N-Term(iTRAQ4plex); K9(iTRAQ4	1	1
[K].tcVADESAENcDk.[S]	N-Term(iTRAQ4plex); C2(Carbam	1	1
[K].tYETTLEk.[C]	N-Term(iTRAQ4plex); K8(iTRAQ4	1	1
[K].eTcFAEEGk.[K]	N-Term(iTRAQ4plex); C3(Carbam	1	1
[K].lDELRDEGk.[A]	N-Term(iTRAQ4plex); K9(iTRAQ4	1	1
[K].AEFAEVSk.[L]	K8(iTRAQ4plex)	1	1
[K].aAFTEccQAADk.[A]	N-Term(iTRAQ4plex); C6(Carbam	1	1
[K].aVmDDFAAFVEk.[C]	N-Term(iTRAQ4plex); M3(Oxidat	1	1
[K].sHcIAEVENDEmPADLPSLAADFVESk.[D]	N-Term(iTRAQ4plex); C3(Carbam	1	1
[R].rHPDYSVVLLLR.[L]	N-Term(iTRAQ4plex)	1	1
[K].aEFAEVSk.[L]	N-Term(iTRAQ4plex); K8(iTRAQ4	1	1
[K].lVTDLTk.[V]	N-Term(iTRAQ4plex); K7(iTRAQ4	1	1
[K].vHTEccHGDLLEcADDR.[A]	N-Term(iTRAQ4plex); C5(Carbam	1	1
[K].aAFTEccQAADk.[A]	N-Term(iTRAQ4plex); C6(Carbam	1	1
[R].rHPYFYAPELLFFAk.[R]	N-Term(iTRAQ4plex); K15(iTRAQ	1	1

[K].TcVADESAENcDk.[S]	C2(Carbamidomethyl); C11(Carb	1	1
[R].aFKAWAVAR.[L]	N-Term(iTRAQ4plex)	1	1
[R].nEcFLQHk.[D]	N-Term(iTRAQ4plex); C3(Carbam	1	1
[K].sLHTLFGDk.[L]	N-Term(iTRAQ4plex); K9(iTRAQ4	1	1
[K].qTALVELVk.[H]	N-Term(iTRAQ4plex); K9(iTRAQ4	1	1
[R].NEcFLQHk.[D]	C3(Carbamidomethyl); K8(iTRAC	1	1
[K].lVTDLTk.[V]	N-Term(iTRAQ4plex); K7(iTRAQ4	1	1
[K].ADDkETcFAEEGk.[K]	K4(iTRAQ4plex); C7(Carbamidon	1	1
[K].ADDkETcFAEEGk.[K]	K4(iTRAQ4plex); C7(Carbamidon	1	1
[K].kQTALVELVk.[H]	N-Term(iTRAQ4plex); K1(iTRAQ4	1	1
[R].eTYGEmADccAk.[Q]	N-Term(iTRAQ4plex); M6(Oxidat	1	1
[R].nEcFLQHk.[D]	N-Term(iTRAQ4plex); C3(Carbam	1	1
[K].fQNALLVR.[Y]	N-Term(iTRAQ4plex)	1	1
[K].eFNAETFTFHADIcTLSEk.[E]	N-Term(iTRAQ4plex); C14(Carbar	1	1
[K].aDDKETcFAEEGk.[K]	N-Term(iTRAQ4plex); C7(Carbam	1	1
[K].qTALVELVk.[H]	N-Term(iTRAQ4plex); K9(iTRAQ4	1	1
[K].dDNPNLPR.[L]	N-Term(iTRAQ4plex)	1	1
[K].lVTDLTk.[V]	N-Term(iTRAQ4plex); K7(iTRAQ4	1	1
[K].dDNPNLPR.[L]	N-Term(iTRAQ4plex)	1	1
[K].aDDkETcFAEEGk.[K]	N-Term(iTRAQ4plex); K4(iTRAQ4	1	1
[K].eFNAETFTFHADIcTLSEk.[E]	N-Term(iTRAQ4plex); C14(Carbar	1	1
[K].aVMDDFAAFVEk.[C]	N-Term(iTRAQ4plex); K12(iTRAQ	1	1
[K].aDDkETcFAEEGk.[K]	N-Term(iTRAQ4plex); K4(iTRAQ4	1	1
[K].yIcENQDSISSk.[L]	N-Term(iTRAQ4plex); C3(Carbam	1	1
[K].lVTDLTk.[V]	N-Term(iTRAQ4plex); K7(iTRAQ4	1	1
[K].dLGEENFk.[A]	N-Term(iTRAQ4plex); K8(iTRAQ4	1	1
[K].lcTVATLR.[E]	N-Term(iTRAQ4plex); C2(Carbam	1	1
[K].sLHTLFGDk.[L]	N-Term(iTRAQ4plex); K9(iTRAQ4	1	1
[K].lcTVATLR.[E]	N-Term(iTRAQ4plex); C2(Carbam	1	1
[K].ADDkETcFAEEGk.[K]	K4(iTRAQ4plex); C7(Carbamidon	1	1
[R].rHPDYSVVLLLR.[L]	N-Term(iTRAQ4plex)	1	1
[K].aVmDDFAAFVEk.[C]	N-Term(iTRAQ4plex); M3(Oxidat	1	1
[K].ccTESLVNR.[R]	N-Term(iTRAQ4plex); C1(Carbam	1	1
[K].aAFTEccQAADk.[A]	N-Term(iTRAQ4plex); C6(Carbam	1	1
[R].lVRPEVDVMcTAFHDNEETFLk.[K]	N-Term(iTRAQ4plex); C10(Carbar	1	1
[K].VHTEccHGDLLEcADDR.[A]	C5(Carbamidomethyl); C6(Carba	1	1
[K].lVTDLTk.[V]	N-Term(iTRAQ4plex); K7(iTRAQ4	1	1
[K].aVMDDFAAFVEk.[C]	N-Term(iTRAQ4plex); K12(iTRAQ	1	1
[K].aDDkETcFAEEGk.[K]	N-Term(iTRAQ4plex); K4(iTRAQ4	1	1
[K].kLVAASQAALGL.[-]	N-Term(iTRAQ4plex); K1(iTRAQ4	1	1
[K].tcVADESAENcDk.[S]	N-Term(iTRAQ4plex); C2(Carbam	1	1
[K].sLHTLFGDk.[L]	N-Term(iTRAQ4plex); K9(iTRAQ4	1	1
[R].rHPDYSVVLLLR.[L]	N-Term(iTRAQ4plex)	1	1
[K].lVTDLTk.[V]	N-Term(iTRAQ4plex); K7(iTRAQ4	1	1
[K].dDNPNLPR.[L]	N-Term(iTRAQ4plex)	1	1
[K].aDDkETcFAEEGkk.[L]	N-Term(iTRAQ4plex); K4(iTRAQ4	1	1
[K].aDDKETcFAEEGk.[K]	N-Term(iTRAQ4plex); C7(Carbam	1	1
[K].vFDEFkPLVEEPQNLIk.[Q]	N-Term(iTRAQ4plex); K6(iTRAQ4	1	1
[R].rHPDYSVVLLLR.[L]	N-Term(iTRAQ4plex)	1	1
[R].nEcFLQHk.[D]	N-Term(iTRAQ4plex); C3(Carbam	1	1
[K].TYETTLEk.[C]	K8(iTRAQ4plex)	1	1
[K].vFDEFKPLVEEPQNLIk.[Q]	N-Term(iTRAQ4plex); K17(iTRAQ	1	1

[K].VHTEccHGDLLEcADDR.[A]	C5(Carbamidomethyl); C6(Carba	1	1
[K].qNcELFEQLGEYk.[F]	N-Term(iTRAQ4plex); C3(Carbam	1	1
[K].lcTVATLR.[E]	N-Term(iTRAQ4plex); C2(Carbam	1	1
[R].rPcFSALEVDETYVPk.[E]	N-Term(iTRAQ4plex); C3(Carbam	1	1
[K].TYETTLEk.[C]	K8(iTRAQ4plex)	1	1
[K].aVmDDFAAFVEk.[C]	N-Term(iTRAQ4plex); M3(Oxidat	1	1
[R].eTYGEmADccAk.[Q]	N-Term(iTRAQ4plex); M6(Oxidat	1	1
[R].eTYGEmADccAk.[Q]	N-Term(iTRAQ4plex); M6(Oxidat	1	1
[K].lDELRDEGk.[A]	N-Term(iTRAQ4plex); K9(iTRAQ4	1	1
[R].eTYGEmADccAk.[Q]	N-Term(iTRAQ4plex); M6(Oxidat	1	1
[K].lVTDLTk.[V]	N-Term(iTRAQ4plex); K7(iTRAQ4	1	1
[R].ETYGEMADccAk.[Q]	C9(Carbamidomethyl); C10(Carb	1	1
[R].eTYGEmADccAk.[Q]	N-Term(iTRAQ4plex); M6(Oxidat	1	1
[K].aVmDDFAAFVEk.[C]	N-Term(iTRAQ4plex); M3(Oxidat	1	1
[K].tcVADESAENcDk.[S]	N-Term(iTRAQ4plex); C2(Carbam	1	1
[K].ccTESLVNR.[R]	N-Term(iTRAQ4plex); C1(Carbam	1	1
[K].TYETTLEk.[C]	K8(iTRAQ4plex)	1	1
[K].aDDkETcFAEEGk.[K]	N-Term(iTRAQ4plex); K4(iTRAQ4	1	1
[K].dDNPNLPR.[L]	N-Term(iTRAQ4plex)	1	1
[K].aDDkETcFAEEGk.[K]	N-Term(iTRAQ4plex); K4(iTRAQ4	1	1
[K].aDDkETcFAEEGk.[K]	N-Term(iTRAQ4plex); K4(iTRAQ4	1	1
[K].aDDkETcFAEEGk.[K]	N-Term(iTRAQ4plex); K4(iTRAQ4	1	1
[K].aDDkETcFAEEGk.[K]	N-Term(iTRAQ4plex); K4(iTRAQ4	1	1
[K].YIcENQDSISSk.[L]	C3(Carbamidomethyl); K12(iTRA	1	1
[R].aFkAWAVAR.[L]	N-Term(iTRAQ4plex); K3(iTRAQ4	1	1
[K].sLHTLFGDk.[L]	N-Term(iTRAQ4plex); K9(iTRAQ4	1	1
[K].dLGEENFk.[A]	N-Term(iTRAQ4plex); K8(iTRAQ4	1	1
[K].ADDkETcFAEEGkk.[L]	K4(iTRAQ4plex); C7(Carbamidon	1	1
[K].LVNEVTEFAk.[T]	K10(iTRAQ4plex)	1	1
[K].FQNALLVR.[Y]		1	1
[K].eFNAETFTFHADIcTLSEk.[E]	N-Term(iTRAQ4plex); C14(Carbar	1	1
[K].dLGEENFk.[A]	N-Term(iTRAQ4plex); K8(iTRAQ4	1	1
[K].eFNAETFTFHADIcTLSEk.[E]	N-Term(iTRAQ4plex); C14(Carbar	1	1
[K].lDELRDEGk.[A]	N-Term(iTRAQ4plex); K9(iTRAQ4	1	1
[R].LSQRFPkAEFAEVSk.[L]	K7(iTRAQ4plex); K15(iTRAQ4plex	1	1
[K].eFNAETFTFHADIcTLSEk.[E]	N-Term(iTRAQ4plex); C14(Carbar	1	1
[K].LVAASQAALGL.[-]		1	1
[K].dDNPNLPR.[L]	N-Term(iTRAQ4plex)	1	1
[K].aDDkETcFAEEGk.[K]	N-Term(iTRAQ4plex); K4(iTRAQ4	1	1
[R].NEcFLQHk.[D]	C3(Carbamidomethyl); K8(iTRAC	1	1
[K].DLGEENFk.[A]	K8(iTRAQ4plex)	1	1
[K].TYETTLEk.[C]	K8(iTRAQ4plex)	1	1
[R].rPcFSALEVDETYVPk.[E]	N-Term(iTRAQ4plex); C3(Carbam	1	1
[K].kQTALVELVk.[H]	N-Term(iTRAQ4plex); K1(iTRAQ4	1	1
[R].nEcFLQHk.[D]	N-Term(iTRAQ4plex); C3(Carbam	1	1
[K].vHTEccHGDLLEcADDR.[A]	N-Term(iTRAQ4plex); C5(Carbam	1	1
[K].eccEKPLLEk.[S]	N-Term(iTRAQ4plex); C2(Carbam	1	1
[R].lVRPEVDVMcTAFHDNEETFLk.[K]	N-Term(iTRAQ4plex); C10(Carbar	1	1
[K].vFDEFkPLVEEPQNLIk.[Q]	N-Term(iTRAQ4plex); K6(iTRAQ4	1	1
[K].eTcFAEEGk.[K]	N-Term(iTRAQ4plex); C3(Carbam	1	1
[K].aAFTEccQAADk.[A]	N-Term(iTRAQ4plex); C6(Carbam	1	1
[K].vFDEFkPLVEEPQNLIk.[Q]	N-Term(iTRAQ4plex); K6(iTRAQ4	1	1

[K].aDDkETcFAEEGkk.[L]	N-Term(iTRAQ4plex); K4(iTRAQ4	1	1
[R].lVRPEVDVMcTAFHDNEETFLk.[K]	N-Term(iTRAQ4plex); C10(Carbar	1	1
[K].eFNAETFTFHADIcTLSEk.[E]	N-Term(iTRAQ4plex); C14(Carbar	1	1
[K].LVTDLTk.[V]	K7(iTRAQ4plex)	1	1
[K].sLHTLFGDk.[L]	N-Term(iTRAQ4plex); K9(iTRAQ4	1	1
[K].ETcFAEEGk.[K]	C3(Carbamidomethyl); K9(iTRAC	1	1
[K].aEFAEVSk.[L]	N-Term(iTRAQ4plex); K8(iTRAQ4	1	1
[K].aVMDDFAAFVEk.[C]	N-Term(iTRAQ4plex); K12(iTRAQ	1	1
[K].vHTEccHGDLLEcADDR.[A]	N-Term(iTRAQ4plex); C5(Carbam	1	1
[K].aDDkETcFAEEGk.[K]	N-Term(iTRAQ4plex); K4(iTRAQ4	1	1
[K].vHTEccHGDLLEcADDR.[A]	N-Term(iTRAQ4plex); C5(Carbam	1	1
[R].eTYGEmADccAk.[Q]	N-Term(iTRAQ4plex); M6(Oxidat	1	1
[K].vFDEFKPLVEEPQNLIk.[Q]	N-Term(iTRAQ4plex); K17(iTRAQ	1	1
[K].SLHTLFGDk.[L]	K9(iTRAQ4plex)	1	1
[K].eFNAETFTFHADIcTLSEk.[E]	N-Term(iTRAQ4plex); C14(Carbar	1	1
[K].eTcFAEEGk.[K]	N-Term(iTRAQ4plex); C3(Carbam	1	1
[K].aDDKETcFAEEGkk.[L]	N-Term(iTRAQ4plex); C7(Carbam	1	1
[K].LVNEVTEFAk.[T]	K10(iTRAQ4plex)	1	1
[K].QTALVELVk.[H]	K9(iTRAQ4plex)	1	1
[K].FQNALLVR.[Y]		1	1
[K].VHTEccHGDLLEcADDR.[A]	C5(Carbamidomethyl); C6(Carba	1	1
[K].aVMDDFAAFVEK.[C]	N-Term(iTRAQ4plex)	1	1
[K].aDDkETcFAEEGkk.[L]	N-Term(iTRAQ4plex); K4(iTRAQ4	1	1
[K].sLHTLFGDk.[L]	N-Term(iTRAQ4plex); K9(iTRAQ4	1	1
[K].dDNPNLPR.[L]	N-Term(iTRAQ4plex)	1	1
[K].vFDEFkPLVEEPQNLIk.[Q]	N-Term(iTRAQ4plex); K6(iTRAQ4	1	1
[K].TYETTLEk.[C]	K8(iTRAQ4plex)	1	1
[K].tcVADESAENcDk.[S]	N-Term(iTRAQ4plex); C2(Carbam	1	1
[K].sLHTLFGDK.[L]	N-Term(iTRAQ4plex)	1	1
[K].kVPQVSTPTLVEVSR.[N]	N-Term(iTRAQ4plex); K1(iTRAQ4	1	1
[K].tYETTLEk.[C]	N-Term(iTRAQ4plex); K8(iTRAQ4	1	1
[K].kQTALVELVk.[H]	N-Term(iTRAQ4plex); K1(iTRAQ4	1	1
[K].lcTVATLR.[E]	N-Term(iTRAQ4plex); C2(Carbam	1	1
[K].qNcELFEQLGEYk.[F]	N-Term(iTRAQ4plex); C3(Carbam	1	1
[K].aEFAEVSk.[L]	N-Term(iTRAQ4plex); K8(iTRAQ4	1	1
[R].LVRPEVDVMcTAFHDNEETFLk.[K]	C10(Carbamidomethyl); K22(iTR	1	1
[K].aDDkETcFAEEGk.[K]	N-Term(iTRAQ4plex); K4(iTRAQ4	1	1
[R].eTYGEMADccAk.[Q]	N-Term(iTRAQ4plex); C9(Carbam	1	1
[K].fQNALLVR.[Y]	N-Term(iTRAQ4plex)	1	1
[K].FQNALLVR.[Y]		1	1
[K].vPQVSTPTLVEVSR.[N]	N-Term(iTRAQ4plex)	1	1
[K].dLGEENFk.[A]	N-Term(iTRAQ4plex); K8(iTRAQ4	1	1
[K].aDDkETcFAEEGkk.[L]	N-Term(iTRAQ4plex); K4(iTRAQ4	1	1
[R].eTYGEmADccAk.[Q]	N-Term(iTRAQ4plex); M6(Oxidat	1	1
[R].eTYGEmADccAk.[Q]	N-Term(iTRAQ4plex); M6(Oxidat	1	1
[K].kVPQVSTPTLVEVSR.[N]	N-Term(iTRAQ4plex); K1(iTRAQ4	1	1
[K].eFNAETFTFHADIcTLSEk.[E]	N-Term(iTRAQ4plex); C14(Carbar	1	1
[R].nEcFLQHk.[D]	N-Term(iTRAQ4plex); C3(Carbam	1	1
[K].eFNAETFTFHADIcTLSEk.[E]	N-Term(iTRAQ4plex); C14(Carbar	1	1
[R].YTkkVPQVSTPTLVEVSR.[N]	K3(iTRAQ4plex); K4(iTRAQ4plex)	1	1
[R].yTkKVPQVSTPTLVEVSR.[N]	N-Term(iTRAQ4plex); K3(iTRAQ4	1	1
[R].yTKkVPQVSTPTLVEVSR.[N]	N-Term(iTRAQ4plex); K4(iTRAQ4	1	1

[R].hPDYSVVLLLR.[L]	N-Term(iTRAQ4plex)	1	1
[K].vFDEFkPLVEEPQNLIk.[Q]	N-Term(iTRAQ4plex); K6(iTRAQ4	1	1
[K].eFNAETFTFHADIcTLSEk.[E]	N-Term(iTRAQ4plex); C14(Carbar	1	1
[K].TYETTLEk.[C]	K8(iTRAQ4plex)	1	1
[K].qTALVELVk.[H]	N-Term(iTRAQ4plex); K9(iTRAQ4	1	1
[K].aVMDDFAAFVEk.[C]	N-Term(iTRAQ4plex); K12(iTRAQ	1	1
[K].lcTVATLR.[E]	N-Term(iTRAQ4plex); C2(Carbam	1	1
[K].lcTVATLR.[E]	N-Term(iTRAQ4plex); C2(Carbam	1	1
[R].eTYGEmADccAk.[Q]	N-Term(iTRAQ4plex); M6(Oxidat	1	1
[K].aDDkETcFAEEGk.[K]	N-Term(iTRAQ4plex); K4(iTRAQ4	1	1
[K].SLHTLFGDk.[L]	K9(iTRAQ4plex)	1	1
[K].SLHTLFGDk.[L]	K9(iTRAQ4plex)	1	1
[R].rPcFSALEVDETYVPk.[E]	N-Term(iTRAQ4plex); C3(Carbam	1	1
[R].lVRPEVDVMcTAFHDNEETFLk.[K]	N-Term(iTRAQ4plex); C10(Carbar	1	1
[K].eFNAETFTFHADIcTLSEk.[E]	N-Term(iTRAQ4plex); C14(Carbar	1	1
[K].vHTEccHGDLLEcADDR.[A]	N-Term(iTRAQ4plex); C5(Carbam	1	1
[K].ccTESLVNR.[R]	N-Term(iTRAQ4plex); C1(Carbam	1	1
[R].eTYGEMADccAk.[Q]	N-Term(iTRAQ4plex); C9(Carbam	1	1
[R].eTYGEmADccAk.[Q]	N-Term(iTRAQ4plex); M6(Oxidat	1	1
[K].dDNPNLPR.[L]	N-Term(iTRAQ4plex)	1	1
[K].aVMDDFAAFVEk.[C]	N-Term(iTRAQ4plex); K12(iTRAQ	1	1
[K].FQNALLVR.[Y]		1	1
[K].AAFTEccQAADk.[A]	C6(Carbamidomethyl); C7(Carba	1	1
[K].dINYVNPVIk.[W]	N-Term(iTRAQ4plex); K10(iTRAQ	1	1
[K].aFTEccVVASQLR.[A]	N-Term(iTRAQ4plex); C5(Carbam	1	1
[K].aFTEccVVASQLR.[A]	N-Term(iTRAQ4plex); C5(Carbam	1	1
[K].aFTEccVVASQLR.[A]	N-Term(iTRAQ4plex); C5(Carbam	1	1
[R].iVAcASYkPSR.[E]	N-Term(iTRAQ4plex); C4(Carbam	1	1
[R].iVAcASYkPSR.[E]	N-Term(iTRAQ4plex); C4(Carbam	1	1
[K].qcTMFYSTSNIk.[I]	N-Term(iTRAQ4plex); C2(Carbam	1	1
[K].lQGTLPVEAR.[E]	N-Term(iTRAQ4plex)	1	1
[K].lQGTLPVEAR.[E]	N-Term(iTRAQ4plex)	1	1
[R].IVAcASYkPSR.[E]	C4(Carbamidomethyl); K8(iTRAC	1	1
[K].iTHYNYLILSk.[G]	N-Term(iTRAQ4plex); K11(iTRAQ	1	1
[K].iTHYNYLILSk.[G]	N-Term(iTRAQ4plex); K11(iTRAQ	1	1
[K].tLLPVSkPEIR.[S]	N-Term(iTRAQ4plex); K7(iTRAQ4	1	1
[K].tLLPVSkPEIR.[S]	N-Term(iTRAQ4plex); K7(iTRAQ4	1	1
[K].tLLPVSkPEIR.[S]	N-Term(iTRAQ4plex); K7(iTRAQ4	1	1
[K].dSLDQLVGGVPVTLNAQTIDVNQETSDLD	N-Term(iTRAQ4plex); K32(iTRAQ	1	1
[K].eGMLSIMSYR.[N]	N-Term(iTRAQ4plex)	1	1
[K].aFDIcPLVk.[I]	N-Term(iTRAQ4plex); C5(Carbam	1	1
[K].aTLLDIYk.[T]	N-Term(iTRAQ4plex); K8(iTRAQ4	1	1
[K].aLVEGVDQLFTDYQIk.[D]	N-Term(iTRAQ4plex); K16(iTRAQ	1	1
[K].aLVEGVDQLFTDYQIk.[D]	N-Term(iTRAQ4plex); K16(iTRAQ	1	1
[K].aLVEGVDQLFTDYQIk.[D]	N-Term(iTRAQ4plex); K16(iTRAQ	1	1
[K].qLPGGQNPVSYVYLEVVSk.[H]	N-Term(iTRAQ4plex); K19(iTRAQ	1	1
[K].qLPGGQNPVSYVYLEVVSk.[H]	N-Term(iTRAQ4plex); K19(iTRAQ	1	1
[K].eNSQYQPIk.[L]	N-Term(iTRAQ4plex); K9(iTRAQ4	1	1
[K].IDTQDIEASHYR.[G]		1	1
[R].eSYSGVTLDPR.[G]	N-Term(iTRAQ4plex)	1	1
[R].sYFPESWLWEVHLVPR.[R]	N-Term(iTRAQ4plex)	1	1
[R].sYFPESWLWEVHLVPR.[R]	N-Term(iTRAQ4plex)	1	1

[K].lNLVATPLFLkPGIPYPIk.[V]	N-Term(iTRAQ4plex); K11(iTRAQ	1	1
[K].lNLVATPLFLkPGIPYPIk.[V]	N-Term(iTRAQ4plex); K11(iTRAQ	1	1
[K].kccYDGAcVNNDETcEQR.[A]	N-Term(iTRAQ4plex); K1(iTRAQ4	1	1
[K].kIEEIAAk.[Y]	N-Term(iTRAQ4plex); K1(iTRAQ4	1	1
[K].dGHVILQLNSIPSSDFLcVR.[F]	N-Term(iTRAQ4plex); C18(Carbar	1	1
[K].fSDASYQSINIPVTQNMVPSSR.[L]	N-Term(iTRAQ4plex)	1	1
[K].iDTQDIEASHYR.[G]	N-Term(iTRAQ4plex)	1	1
[R].vYSLNDDLkPAk.[R]	N-Term(iTRAQ4plex); K9(iTRAQ4	1	1
[K].fQNSAILTIQPk.[Q]	N-Term(iTRAQ4plex); K12(iTRAQ	1	1
[K].tSTSEEVcSFYLk.[I]	N-Term(iTRAQ4plex); C8(Carbam	1	1
[R].yIYPLDSLTWIEYWPR.[D]	N-Term(iTRAQ4plex)	1	1
[R].yIYPLDSLTWIEYWPR.[D]	N-Term(iTRAQ4plex)	1	1
[R].vDDGVASFVLNLPSGVTVLEFNVk.[T]	N-Term(iTRAQ4plex); K24(iTRAQ	1	1
[K].TDAPDLPEENQAR.[E]		1	1
[K].ccYDGAcVNNDETcEQR.[A]	C1(Carbamidomethyl); C2(Carba	1	1
[K].gLLVGEILSAVLSQEGINILTHLPk.[G]	N-Term(iTRAQ4plex); K25(iTRAQ	1	1
[K].aLLVGEHLNIIVTPk.[S]	N-Term(iTRAQ4plex); K15(iTRAQ	1	1
[K].gLLVGEILSAVLSQEGINILTHLPk.[G]	N-Term(iTRAQ4plex); K25(iTRAQ	1	1
[K].kIEEIAAk.[Y]	N-Term(iTRAQ4plex); K1(iTRAQ4	1	1
[K].aLLVGEHLNIIVTPk.[S]	N-Term(iTRAQ4plex); K15(iTRAQ	1	1
[K].aLLVGEHLNIIVTPk.[S]	N-Term(iTRAQ4plex); K15(iTRAQ	1	1
[K].ccYDGAcVNNDETcEQR.[A]	N-Term(iTRAQ4plex); C1(Carbam	1	1
[R].vFQFLEk.[S]	N-Term(iTRAQ4plex); K7(iTRAQ4	1	1
[K].tDAPDLPEENQAR.[E]	N-Term(iTRAQ4plex)	1	1
[K].tDAPDLPEENQAR.[E]	N-Term(iTRAQ4plex)	1	1
[K].dINYVNPVIk.[W]	N-Term(iTRAQ4plex); K10(iTRAQ	1	1
[K].dINYVNPVIk.[W]	N-Term(iTRAQ4plex); K10(iTRAQ	1	1
[R].lSmDIDVSYk.[H]	N-Term(iTRAQ4plex); M3(Oxidat	1	1
[K].dSSVPNTGTAR.[M]	N-Term(iTRAQ4plex)	1	1
[R].qYLIMGk.[E]	N-Term(iTRAQ4plex); K7(iTRAQ4	1	1
[K].eGMLSIMSYR.[N]	N-Term(iTRAQ4plex)	1	1
[K].dSSVPNTGTAR.[M]	N-Term(iTRAQ4plex)	1	1
[K].TLLPVSkPEIR.[S]	K7(iTRAQ4plex)	1	1
[R].eSYSGVTLDPR.[G]	N-Term(iTRAQ4plex)	1	1
[K].yVLSPYk.[L]	N-Term(iTRAQ4plex); K7(iTRAQ4	1	1
[K].vTcTNAELVk.[G]	N-Term(iTRAQ4plex); C3(Carbam	1	1
[K].dSEITFIk.[K]	N-Term(iTRAQ4plex); K8(iTRAQ4	1	1
[R].vYSLNDDLkPAk.[R]	N-Term(iTRAQ4plex); K9(iTRAQ4	1	1
[K].aFTEccVVASQLR.[A]	N-Term(iTRAQ4plex); C5(Carbam	1	1
[R].vYSLNDDLkPAkR.[E]	N-Term(iTRAQ4plex); K9(iTRAQ4	1	1
[K].TGEAVAEk.[D]	K8(iTRAQ4plex)	1	1
[K].dSSVPNTGTAR.[M]	N-Term(iTRAQ4plex)	1	1
[R].kAFDIcPLVk.[I]	N-Term(iTRAQ4plex); K1(iTRAQ4	1	1
[R].IVAcASYkPSR.[E]	C4(Carbamidomethyl); K8(iTRAC	1	1
[K].vSITSITVENVFVk.[Y]	N-Term(iTRAQ4plex); K14(iTRAQ	1	1
[K].ITHYNYLILSk.[G]	K11(iTRAQ4plex)	1	1
[K].vcEGAAck.[C]	N-Term(iTRAQ4plex); C2(Carbam	1	1
[K].vcEGAAck.[C]	N-Term(iTRAQ4plex); C2(Carbam	1	1
[K].iDTALIk.[A]	N-Term(iTRAQ4plex); K7(iTRAQ4	1	1
[R].iVAcASYkPSR.[E]	N-Term(iTRAQ4plex); C4(Carbam	1	1
[R].kAFDIcPLVk.[I]	N-Term(iTRAQ4plex); C6(Carbam	1	1
[R].kAFDIcPLVk.[I]	K1(iTRAQ4plex); C6(Carbamidon	1	1

[K].ccYDGAcVNNDETcEQR.[A]	N-Term(iTRAQ4plex); C1(Carbam	1	1
[K].eNSQYQPIk.[L]	N-Term(iTRAQ4plex); K9(iTRAQ4	1	1
[K].tLLPVSkPEIR.[S]	N-Term(iTRAQ4plex); K7(iTRAQ4	1	1
[K].IDTALIk.[A]	K7(iTRAQ4plex)	1	1
[K].tGEAVAEk.[D]	N-Term(iTRAQ4plex); K8(iTRAQ4	1	1
[K].tDAPDLPEENQAR.[E]	N-Term(iTRAQ4plex)	1	1
[K].TDAPDLPEENQAR.[E]		1	1
[K].ITHYNYLILSk.[G]	K11(iTRAQ4plex)	1	1
[R].VYSLNDDLkPAkR.[E]	K9(iTRAQ4plex); K12(iTRAQ4plex	1	1
[K].tGEAVAEk.[D]	N-Term(iTRAQ4plex); K8(iTRAQ4	1	1
[R].iPLDLVPk.[T]	N-Term(iTRAQ4plex); K8(iTRAQ4	1	1
[R].VYSLNDDLkPAkR.[E]	K9(iTRAQ4plex); K12(iTRAQ4plex	1	1
[R].vFQFLEk.[S]	N-Term(iTRAQ4plex); K7(iTRAQ4	1	1
[R].qYLIMGk.[E]	N-Term(iTRAQ4plex); K7(iTRAQ4	1	1
[R].iVAcASYkPSR.[E]	N-Term(iTRAQ4plex); C4(Carbam	1	1
[K].tDAPDLPEENQAR.[E]	N-Term(iTRAQ4plex)	1	1
[R].sIVSALk.[R]	N-Term(iTRAQ4plex); K7(iTRAQ4	1	1
[R].kAFDIcPLVk.[I]	N-Term(iTRAQ4plex); K1(iTRAQ4	1	1
[K].ITHYNYLILSk.[G]	K11(iTRAQ4plex)	1	1
[K].iDTQDIEASHYR.[G]	N-Term(iTRAQ4plex)	1	1
[K].IEEIAAk.[Y]	K7(iTRAQ4plex)	1	1
[K].fQNSAILTIQPk.[Q]	N-Term(iTRAQ4plex); K12(iTRAQ4	1	1
[R].sIVSALk.[R]	N-Term(iTRAQ4plex); K7(iTRAQ4	1	1
[K].iDTALIk.[A]	N-Term(iTRAQ4plex); K7(iTRAQ4	1	1
[K].iTHYNYLILSk.[G]	N-Term(iTRAQ4plex); K11(iTRAQ4	1	1
[R].kQTAckPEIAYAYk.[V]	N-Term(iTRAQ4plex); K1(iTRAQ4	1	1
[K].TDAPDLPEENQAR.[E]		1	1
[R].eDLkDDQk.[E]	N-Term(iTRAQ4plex); K4(iTRAQ4	1	1
[R].gYGNSDYk.[R]	N-Term(iTRAQ4plex); K8(iTRAQ4	1	1
[R].yGMWTIk.[A]	N-Term(iTRAQ4plex); K7(iTRAQ4	1	1
[R].VFQFLEk.[S]	K7(iTRAQ4plex)	1	1
[K].yVLSPYk.[L]	N-Term(iTRAQ4plex); K7(iTRAQ4	1	1
[R].sIVSALk.[R]	N-Term(iTRAQ4plex); K7(iTRAQ4	1	1
[R].iPLDLVPk.[T]	N-Term(iTRAQ4plex); K8(iTRAQ4	1	1
[K].iTHYNYLILSk.[G]	N-Term(iTRAQ4plex); K11(iTRAQ4	1	1
[K].iEEIAAk.[Y]	N-Term(iTRAQ4plex); K7(iTRAQ4	1	1
[K].dSSVPNTGTAR.[M]	N-Term(iTRAQ4plex)	1	1
[K].nFEITIk.[A]	N-Term(iTRAQ4plex); K7(iTRAQ4	1	1
[K].TDAPDLPEENQAR.[E]		1	1
[K].nFEITIk.[A]	N-Term(iTRAQ4plex); K7(iTRAQ4	1	1
[K].aTLLDIYk.[T]	N-Term(iTRAQ4plex); K8(iTRAQ4	1	1
[K].iDTQDIEASHYR.[G]	N-Term(iTRAQ4plex)	1	1
[K].ITHYNYLILSk.[G]	K11(iTRAQ4plex)	1	1
[K].tGEAVAEk.[D]	N-Term(iTRAQ4plex); K8(iTRAQ4	1	1
[K].dSEITFIk.[K]	N-Term(iTRAQ4plex); K8(iTRAQ4	1	1
[K].tDAPDLPEENQAR.[E]	N-Term(iTRAQ4plex)	1	1
[K].yVLSPYk.[L]	N-Term(iTRAQ4plex); K7(iTRAQ4	1	1
[K].aAQVTIQSSGTFSSk.[F]	N-Term(iTRAQ4plex); K15(iTRAQ4	1	1
[R].TEHPFTVEEFVLPk.[F]	K14(iTRAQ4plex)	1	1
[K].mcPQLQQYEmHGPEGLR.[V]	N-Term(iTRAQ4plex); C2(Carbam	1	1
[K].mcPQLQQYEMHGPEGLR.[V]	N-Term(iTRAQ4plex); M1(Oxidat	1	1
[K].mcPQLQQYEMHGPEGLR.[V]	N-Term(iTRAQ4plex); C2(Carbam	1	1

[K].mcPQLQQYEMHGPEGLR.[V]	N-Term(iTRAQ4plex); C2(Carbam	1	1
[K].mcPQLQQYEMHGPEGLR.[V]	N-Term(iTRAQ4plex); C2(Carbam	1	1
[K].mcPQLQQYEMHGPEGLR.[V]	N-Term(iTRAQ4plex); C2(Carbam	1	1
[K].lHTEAQIQEEGTVVELTGR.[Q]	N-Term(iTRAQ4plex)	1	1
[R].vGFYESDVMGR.[G]	N-Term(iTRAQ4plex)	1	1
[K].aAQVTIQSSGTFSSk.[F]	N-Term(iTRAQ4plex); K15(iTRAQ	1	1
[K].aAQVTIQSSGTFSSk.[F]	N-Term(iTRAQ4plex); K15(iTRAQ	1	1
[R].SSGSLLNNAIk.[G]	K11(iTRAQ4plex)	2	2
[R].lVHVEEPHTETVR.[K]	N-Term(iTRAQ4plex)	1	1
[K].aAQVTIQSSGTFSSk.[F]	N-Term(iTRAQ4plex); K15(iTRAQ	1	1
[K].aAQVTIQSSGTFSSk.[F]	N-Term(iTRAQ4plex); K15(iTRAQ	1	1
[R].VTAAPQSVcALR.[A]	C9(Carbamidomethyl)	1	1
[K].aAQVTIQSSGTFSSk.[F]	N-Term(iTRAQ4plex); K15(iTRAQ	1	1
[K].vYDYYETDEFAIAEYNAPcSk.[D]	N-Term(iTRAQ4plex); C19(Carbar	1	1
[K].aAQVTIQSSGTFSSk.[F]	N-Term(iTRAQ4plex); K15(iTRAQ	1	1
[K].aAQVTIQSSGTFSSk.[F]	N-Term(iTRAQ4plex); K15(iTRAQ	1	1
[K].aAQVTIQSSGTFSSk.[F]	N-Term(iTRAQ4plex); K15(iTRAQ	1	1
[K].aAQVTIQSSGTFSSk.[F]	N-Term(iTRAQ4plex); K15(iTRAQ	1	1
[K].aAQVTIQSSGTFSSk.[F]	N-Term(iTRAQ4plex); K15(iTRAQ	1	1
[K].EEFPFALGVQTLPQTcDEPk.[A]	C16(Carbamidomethyl); K20(iTR	1	1
[K].vYDYYETDEFAIAEYNAPcSk.[D]	N-Term(iTRAQ4plex); C19(Carbar	1	1
[K].vYDYYETDEFAIAEYNAPcSk.[D]	N-Term(iTRAQ4plex); C19(Carbar	1	1
[K].vYDYYETDEFAIAEYNAPcSk.[D]	N-Term(iTRAQ4plex); C19(Carbar	1	1
[K].vYDYYETDEFAIAEYNAPcSk.[D]	N-Term(iTRAQ4plex); C19(Carbar	1	1
[K].vYDYYETDEFAIAEYNAPcSk.[D]	N-Term(iTRAQ4plex); C19(Carbar	1	1
[K].vYDYYETDEFAIAEYNAPcSk.[D]	N-Term(iTRAQ4plex); C19(Carbar	1	1
[K].lHTEAQIQEEGTVVELTGR.[Q]	N-Term(iTRAQ4plex)	1	1
[K].dTVIkPLLVEPEGLEk.[E]	N-Term(iTRAQ4plex); K5(iTRAQ4	1	1
[K].dTVIkPLLVEPEGLEk.[E]	N-Term(iTRAQ4plex); K5(iTRAQ4	1	1
[K].aAQVTIQSSGTFSSk.[F]	N-Term(iTRAQ4plex); K15(iTRAQ	1	1
[R].vTAAPQSVcALR.[A]	N-Term(iTRAQ4plex); C9(Carbam	1	1
[R].vTAAPQSVcALR.[A]	N-Term(iTRAQ4plex); C9(Carbam	1	1
[K].aAQVTIQSSGTFSSk.[F]	N-Term(iTRAQ4plex); K15(iTRAQ	1	1
[K].aAQVTIQSSGTFSSk.[F]	N-Term(iTRAQ4plex); K15(iTRAQ	1	1
[K].aAQVTIQSSGTFSSk.[F]	N-Term(iTRAQ4plex); K15(iTRAQ	1	1
[K].aAQVTIQSSGTFSSk.[F]	N-Term(iTRAQ4plex); K15(iTRAQ	1	1
[K].aAQVTIQSSGTFSSk.[F]	N-Term(iTRAQ4plex); K15(iTRAQ	1	1
[K].aAQVTIQSSGTFSSk.[F]	N-Term(iTRAQ4plex); K15(iTRAQ	1	1
[K].aAQVTIQSSGTFSSk.[F]	N-Term(iTRAQ4plex); K15(iTRAQ	1	1
[K].gHFSISIPVk.[S]	N-Term(iTRAQ4plex); K10(iTRAQ	1	1
[K].aAQVTIQSSGTFSSk.[F]	N-Term(iTRAQ4plex); K15(iTRAQ	1	1
[K].aAQVTIQSSGTFSSk.[F]	N-Term(iTRAQ4plex); K15(iTRAQ	1	1
[K].aAQVTIQSSGTFSSk.[F]	N-Term(iTRAQ4plex); K15(iTRAQ	1	1
[K].aIGYLNTGYQR.[Q]	N-Term(iTRAQ4plex)	1	1
[K].aIGYLNTGYQR.[Q]	N-Term(iTRAQ4plex)	1	1
[K].aIGYLNTGYQR.[Q]	N-Term(iTRAQ4plex)	1	1
[K].vDLSFSPSQSLPASHAHLR.[V]	N-Term(iTRAQ4plex)	1	1
[K].mVSGFIPLkPTVk.[M]	N-Term(iTRAQ4plex); M1(Oxidat	2	2
[K].mVSGFIPLkPTVk.[M]	N-Term(iTRAQ4plex); M1(Oxidat	2	2
[K].vDLSFSPSQSLPASHAHLR.[V]	N-Term(iTRAQ4plex)	1	1
[K].sIYkPGQTVk.[F]	N-Term(iTRAQ4plex); K4(iTRAQ4	1	1
[K].sIYkPGQTVk.[F]	N-Term(iTRAQ4plex); K4(iTRAQ4	1	1

[K].fSGQLNSHGcFYQQVk.[T]	N-Term(iTRAQ4plex); C10(Carbar	1	1
[R].vSVQLEASPAFLAVPVEk.[E]	N-Term(iTRAQ4plex); K18(iTRAQ	1	1
[R].vSVQLEASPAFLAVPVEk.[E]	N-Term(iTRAQ4plex); K18(iTRAQ	1	1
[K].aIGYLNTGYQR.[Q]	N-Term(iTRAQ4plex)	1	1
[K].gHFSISIPVk.[S]	N-Term(iTRAQ4plex); K10(iTRAQ	1	1
[K].gHFSISIPVk.[S]	N-Term(iTRAQ4plex); K10(iTRAQ	1	1
[K].aAQVTIQSSGTFSSk.[F]	N-Term(iTRAQ4plex); K15(iTRAQ	1	1
[K].aAQVTIQSSGTFSSk.[F]	N-Term(iTRAQ4plex); K15(iTRAQ	1	1
[K].aAQVTIQSSGTFSSk.[F]	N-Term(iTRAQ4plex); K15(iTRAQ	1	1
[R].nALFcLESAWk.[T]	N-Term(iTRAQ4plex); C5(Carbam	1	1
[K].vDLSFSPSQSLPASHAHLR.[V]	N-Term(iTRAQ4plex)	1	1
[K].vTGEGcVYLQTSLk.[Y]	N-Term(iTRAQ4plex); C6(Carbam	1	1
[K].qFSFPLSSEPFQGSYk.[V]	N-Term(iTRAQ4plex); K16(iTRAQ	1	1
[R].nALFcLESAWk.[T]	N-Term(iTRAQ4plex); C5(Carbam	1	1
[R].nALFcLESAWk.[T]	N-Term(iTRAQ4plex); C5(Carbam	1	1
[K].aTVLNYLPk.[C]	N-Term(iTRAQ4plex); K9(iTRAQ4	2	2
[K].qQNAQGGFSSTQDTVVALHALSk.[Y]	N-Term(iTRAQ4plex); K23(iTRAQ	1	1
[K].fEVQVTVPk.[I]	N-Term(iTRAQ4plex); K9(iTRAQ4	1	1
[K].vTGEGcVYLQTSLk.[Y]	N-Term(iTRAQ4plex); C6(Carbam	1	1
[K].vTGEGcVYLQTSLk.[Y]	N-Term(iTRAQ4plex); C6(Carbam	1	1
[K].vTGEGcVYLQTSLk.[Y]	N-Term(iTRAQ4plex); C6(Carbam	1	1
[K].vTGEGcVYLQTSLk.[Y]	N-Term(iTRAQ4plex); C6(Carbam	1	1
[K].vTGEGcVYLQTSLk.[Y]	N-Term(iTRAQ4plex); C6(Carbam	1	1
[K].vTGEGcVYLQTSLk.[Y]	N-Term(iTRAQ4plex); C6(Carbam	1	1
[R].kDTVIkPLLVEPEGLEk.[E]	N-Term(iTRAQ4plex); K1(iTRAQ4	1	1
[K].dMYSFLEDMGLk.[A]	N-Term(iTRAQ4plex); K12(iTRAQ	1	1
[K].dMYSFLEDMGLk.[A]	N-Term(iTRAQ4plex); K12(iTRAQ	1	1
[K].qQNAQGGFSSTQDTVVALHALSk.[Y]	N-Term(iTRAQ4plex); K23(iTRAQ	1	1
[K].qQNAQGGFSSTQDTVVALHALSk.[Y]	N-Term(iTRAQ4plex); K23(iTRAQ	1	1
[R].kDTVIkPLLVEPEGLEk.[E]	N-Term(iTRAQ4plex); K1(iTRAQ4	1	1
[K].aIGYLNTGYQR.[Q]	N-Term(iTRAQ4plex)	1	1
[R].LVHVEEPHTETVR.[K]		1	1
[R].lVHVEEPHTETVR.[K]	N-Term(iTRAQ4plex)	1	1
[R].qTVSWAVTPk.[S]	N-Term(iTRAQ4plex); K10(iTRAQ	1	1
[R].qTVSWAVTPk.[S]	N-Term(iTRAQ4plex); K10(iTRAQ	1	1
[R].tEVSSNHVLIYLDk.[V]	N-Term(iTRAQ4plex); K14(iTRAQ	1	1
[R].tEVSSNHVLIYLDk.[V]	N-Term(iTRAQ4plex); K14(iTRAQ	1	1
[K].eQAPHcIcANGR.[Q]	N-Term(iTRAQ4plex); C6(Carbam	1	1
[K].eQAPHcIcANGR.[Q]	N-Term(iTRAQ4plex); C6(Carbam	1	1
[R].tEVSSNHVLIYLDk.[V]	N-Term(iTRAQ4plex); K14(iTRAQ	1	1
[R].tEVSSNHVLIYLDk.[V]	N-Term(iTRAQ4plex); K14(iTRAQ	1	1
[K].yNILPEkEEFPFALGVQTLPQTcDEPk.[A]	N-Term(iTRAQ4plex); K7(iTRAQ4	1	1
[K].yNILPEkEEFPFALGVQTLPQTcDEPk.[A]	N-Term(iTRAQ4plex); K7(iTRAQ4	1	1
[K].eTTFNSLLcPSGGEVSEELSLk.[L]	N-Term(iTRAQ4plex); C9(Carbam	1	1
[K].eEFPFALGVQTLPQTcDEPk.[A]	N-Term(iTRAQ4plex); C16(Carbar	1	1
[K].eTTFNSLLcPSGGEVSEELSLk.[L]	N-Term(iTRAQ4plex); C9(Carbam	1	1
[K].eTTFNSLLcPSGGEVSEELSLk.[L]	N-Term(iTRAQ4plex); C9(Carbam	1	1
[K].eTTFNSLLcPSGGEVSEELSLk.[L]	N-Term(iTRAQ4plex); C9(Carbam	1	1
[K].eTTFNSLLcPSGGEVSEELSLk.[L]	N-Term(iTRAQ4plex); C9(Carbam	1	1
[K].eTTFNSLLcPSGGEVSEELSLk.[L]	N-Term(iTRAQ4plex); C9(Carbam	1	1
[R].tEHPFTVEEFVLPk.[F]	N-Term(iTRAQ4plex); K14(iTRAQ	1	1
[R].tEHPFTVEEFVLPk.[F]	N-Term(iTRAQ4plex); K14(iTRAQ	1	1

[R].sLFTDLEAENDVLHcVAFAVPk.[S]	N-Term(iTRAQ4plex); C15(Carbar	1	1
[R].sLFTDLEAENDVLHcVAFAVPk.[S]	N-Term(iTRAQ4plex); C15(Carbar	1	1
[K].sSSNEEVMFLTVQVk.[G]	N-Term(iTRAQ4plex); K15(iTRAQ	1	1
[R].aVDQSVLLMkPDAELSASSVYNLLPEk.[D]	N-Term(iTRAQ4plex); K10(iTRAQ	1	1
[R].aVDQSVLLMkPDAELSASSVYNLLPEk.[D]	N-Term(iTRAQ4plex); K10(iTRAQ	1	1
[K].kLSFYYLIMAk.[G]	N-Term(iTRAQ4plex); K1(iTRAQ4	1	1
[K].nEDSLVFVQTDk.[S]	N-Term(iTRAQ4plex); K12(iTRAQ	1	1
[R].iAQWQSFQLEGGLk.[Q]	N-Term(iTRAQ4plex); K14(iTRAQ	1	1
[K].lPPNVVEESAR.[A]	N-Term(iTRAQ4plex)	1	1
[R].lLLQQVSLPELPGEYSmk.[V]	N-Term(iTRAQ4plex); M17(Oxida	1	1
[K].lPPNVVEESAR.[A]	N-Term(iTRAQ4plex)	1	1
[R].kYSDASDcHGEDSQAFcEk.[F]	N-Term(iTRAQ4plex); K1(iTRAQ4	1	1
[K].lPPNVVEESAR.[A]	N-Term(iTRAQ4plex)	1	1
[R].kYSDASDcHGEDSQAFcEk.[F]	N-Term(iTRAQ4plex); K1(iTRAQ4	1	1
[K].lSFYYLIMAk.[G]	N-Term(iTRAQ4plex); K10(iTRAQ	1	1
[K].tAQEGDHGSHVYTk.[A]	N-Term(iTRAQ4plex); K14(iTRAQ	1	1
[K].tAQEGDHGSHVYTk.[A]	N-Term(iTRAQ4plex); K14(iTRAQ	1	1
[K].eQAPHcIcANGR.[Q]	N-Term(iTRAQ4plex); C6(Carbam	1	1
[R].lLLQQVSLPELPGEYSMk.[V]	N-Term(iTRAQ4plex); K18(iTRAQ	1	1
[K].eQAPHcIcANGR.[Q]	N-Term(iTRAQ4plex); C6(Carbam	1	1
[K].eQAPHcIcANGR.[Q]	N-Term(iTRAQ4plex); C6(Carbam	1	1
[R].lLLQQVSLPELPGEYSMk.[V]	N-Term(iTRAQ4plex); K18(iTRAQ	1	1
[K].dNSVHWERPQkPk.[A]	N-Term(iTRAQ4plex); K11(iTRAQ	1	1
[K].aGAFcLSEDAGLGISSTASLR.[A]	N-Term(iTRAQ4plex); C5(Carbam	2	2
[K].hYDGSYSTFGER.[Y]	N-Term(iTRAQ4plex)	1	1
[K].aGAFcLSEDAGLGISSTASLR.[A]	N-Term(iTRAQ4plex); C5(Carbam	2	2
[K].hYDGSYSTFGER.[Y]	N-Term(iTRAQ4plex)	1	1
[R].lVHVEEPHTETVR.[K]	N-Term(iTRAQ4plex)	1	1
[R].lVHVEEPHTETVR.[K]	N-Term(iTRAQ4plex)	1	1
[R].VSVQLEASPAFLAVPVEk.[E]	K18(iTRAQ4plex)	1	1
[R].LVHVEEPHTETVR.[K]		1	1
[K].YDVENcLANk.[V]	C6(Carbamidomethyl); K10(iTRA	1	1
[K].yDVENcLANk.[V]	N-Term(iTRAQ4plex); C6(Carbam	1	1
[K].lPPNVVEESAR.[A]	N-Term(iTRAQ4plex)	1	1
[K].lPPNVVEESAR.[A]	N-Term(iTRAQ4plex)	1	1
[R].sASNMAIVDVk.[M]	N-Term(iTRAQ4plex); K11(iTRAQ	1	1
[K].sLNEEAVk.[K]	N-Term(iTRAQ4plex); K8(iTRAQ4	1	1
[K].sLNEEAVk.[K]	N-Term(iTRAQ4plex); K8(iTRAQ4	1	1
[K].sLNEEAVk.[K]	N-Term(iTRAQ4plex); K8(iTRAQ4	1	1
[K].sLNEEAVk.[K]	N-Term(iTRAQ4plex); K8(iTRAQ4	1	1
[K].aGAFcLSEDAGLGISSTASLR.[A]	N-Term(iTRAQ4plex); C5(Carbam	2	2
[K].mVSGFIPLkPTVk.[M]	N-Term(iTRAQ4plex); K9(iTRAQ4	2	2
[K].aGAFcLSEDAGLGISSTASLR.[A]	N-Term(iTRAQ4plex); C5(Carbam	2	2
[K].mVSGFIPLkPTVk.[M]	N-Term(iTRAQ4plex); K9(iTRAQ4	2	2
[R].hNVYINGITYTPVSSTNEk.[D]	N-Term(iTRAQ4plex); K19(iTRAQ	1	1
[K].vSNQTLSLFFTVLQDVPVR.[D]	N-Term(iTRAQ4plex)	1	1
[K].vSNQTLSLFFTVLQDVPVR.[D]	N-Term(iTRAQ4plex)	1	1
[K].lPPNVVEESAR.[A]	N-Term(iTRAQ4plex)	1	1
[K].aPVGHFYEPQAPSAEVEMTSYVLLAYLTAQ	N-Term(iTRAQ4plex); K46(iTRAQ	1	1
[K].lPPNVVEESAR.[A]	N-Term(iTRAQ4plex)	1	1
[K].lPPNVVEESAR.[A]	N-Term(iTRAQ4plex)	1	1
[K].lPPNVVEESAR.[A]	N-Term(iTRAQ4plex)	1	1

[K].lPPNVVEESAR.[A]	N-Term(iTRAQ4plex)	1	1
[R].aFQPFFVELTMPYSVIR.[G]	N-Term(iTRAQ4plex)	2	2
[R].aFQPFFVELTMPYSVIR.[G]	N-Term(iTRAQ4plex)	2	2
[R].vGFYESDVMGR.[G]	N-Term(iTRAQ4plex)	1	1
[K].lHTEAQIQEEGTVVELTGR.[Q]	N-Term(iTRAQ4plex)	1	1
[K].lHTEAQIQEEGTVVELTGR.[Q]	N-Term(iTRAQ4plex)	1	1
[R].vVSMDENFHPLNELIPLVYIQDPk.[G]	N-Term(iTRAQ4plex); K24(iTRAQ	1	1
[K].gGVEDEVTLSAYITIALLEIPLTVTHPVVR.[N	N-Term(iTRAQ4plex)	1	1
[K].lHTEAQIQEEGTVVELTGR.[Q]	N-Term(iTRAQ4plex)	1	1
[K].aGAFcLSEDAGLGISSTASLR.[A]	N-Term(iTRAQ4plex); C5(Carbam	2	2
[K].lHTEAQIQEEGTVVELTGR.[Q]	N-Term(iTRAQ4plex)	1	1
[K].hYDGSYSTFGER.[Y]	N-Term(iTRAQ4plex)	1	1
[K].tAQEGDHGSHVYTk.[A]	N-Term(iTRAQ4plex); K14(iTRAQ	1	1
[K].tAQEGDHGSHVYTk.[A]	N-Term(iTRAQ4plex); K14(iTRAQ	1	1
[K].sIYkPGQTVk.[F]	N-Term(iTRAQ4plex); K4(iTRAQ4	1	1
[K].tAQEGDHGSHVYTk.[A]	N-Term(iTRAQ4plex); K14(iTRAQ	1	1
[R].lLIYAVLPTGDVIGDSAk.[Y]	N-Term(iTRAQ4plex); K18(iTRAQ	1	1
[K].ySDASDcHGEDSQAFcEk.[F]	N-Term(iTRAQ4plex); C7(Carbam	1	1
[K].ySDASDcHGEDSQAFcEk.[F]	N-Term(iTRAQ4plex); C7(Carbam	1	1
[K].ySDASDcHGEDSQAFcEk.[F]	N-Term(iTRAQ4plex); C7(Carbam	1	1
[R].lLIYAVLPTGDVIGDSAk.[Y]	N-Term(iTRAQ4plex); K18(iTRAQ	1	1
[R].vVSMDENFHPLNELIPLVYIQDPk.[G]	N-Term(iTRAQ4plex); K24(iTRAQ	1	1
[R].lLIYAVLPTGDVIGDSAk.[Y]	N-Term(iTRAQ4plex); K18(iTRAQ	1	1
[K].vTGEGcVYLQTSLk.[Y]	N-Term(iTRAQ4plex); C6(Carbam	1	1
[K].lPPNVVEESAR.[A]	N-Term(iTRAQ4plex)	1	1
[R].tEVSSNHVLIYLDk.[V]	N-Term(iTRAQ4plex); K14(iTRAQ	1	1
[R].qTVSWAVTPk.[S]	N-Term(iTRAQ4plex); K10(iTRAQ	1	1
[K].EQAPHcIcANGR.[Q]	C6(Carbamidomethyl); C8(Carba	1	1
[R].qTVSWAVTPk.[S]	N-Term(iTRAQ4plex); K10(iTRAQ	1	1
[K].aIGYLNTGYQR.[Q]	N-Term(iTRAQ4plex)	1	1
[R].sSGSLLNNAIk.[G]	N-Term(iTRAQ4plex); K11(iTRAQ	2	2
[R].sASNMAIVDVk.[M]	N-Term(iTRAQ4plex); K11(iTRAQ	1	1
[R].aVDQSVLLMKPDAELSASSVYNLLPEk.[D]	N-Term(iTRAQ4plex); K27(iTRAQ	1	1
[K].lPPNVVEESAR.[A]	N-Term(iTRAQ4plex)	1	1
[K].lHTEAQIQEEGTVVELTGR.[Q]	N-Term(iTRAQ4plex)	1	1
[R].tGTHGLLVk.[Q]	N-Term(iTRAQ4plex); K9(iTRAQ4	1	1
[K].sDIAPVAR.[L]	N-Term(iTRAQ4plex)	1	1
[R].vGFYESDVMGR.[G]	N-Term(iTRAQ4plex)	1	1
[R].tGTHGLLVk.[Q]	N-Term(iTRAQ4plex); K9(iTRAQ4	1	1
[R].tGTHGLLVk.[Q]	N-Term(iTRAQ4plex); K9(iTRAQ4	1	1
[K].aAQVTIQSSGTFSSk.[F]	N-Term(iTRAQ4plex); K15(iTRAQ	1	1
[K].lPPNVVEESAR.[A]	N-Term(iTRAQ4plex)	1	1
[R].lVHVEEPHTETVRk.[Y]	N-Term(iTRAQ4plex); K14(iTRAQ	1	1
[K].vTGEGcVYLQTSLk.[Y]	N-Term(iTRAQ4plex); C6(Carbam	1	1
[R].tGTHGLLVk.[Q]	N-Term(iTRAQ4plex); K9(iTRAQ4	1	1
[K].fSGQLNSHGcFYQQVk.[T]	N-Term(iTRAQ4plex); C10(Carbar	1	1
[K].YNILPEkEEFPFALGVQTLPQTcDEPk.[A]	K7(iTRAQ4plex); C23(Carbamido	1	1
[R].lVHVEEPHTETVR.[K]	N-Term(iTRAQ4plex)	1	1
[R].sPcYGYQWVSEEHEEAHHTAYLVFSPSk.[S	N-Term(iTRAQ4plex); C3(Carbam	1	1
[K].qFSFPLSSEPFQGSYk.[V]	N-Term(iTRAQ4plex); K16(iTRAQ	1	1
[K].sLNEEAVk.[K]	N-Term(iTRAQ4plex); K8(iTRAQ4	1	1
[K].eQAPHcIcANGR.[Q]	N-Term(iTRAQ4plex); C6(Carbam	1	1

[K].eQAPHcIcANGR.[Q]	N-Term(iTRAQ4plex); C6(Carbam	1	1
[R].qTVSWAVTPk.[S]	N-Term(iTRAQ4plex); K10(iTRAQ	1	1
[R].kYSDASDcHGEDSQAFcEk.[F]	K1(iTRAQ4plex); C8(Carbamidon	1	1
[R].kYSDASDcHGEDSQAFcEk.[F]	N-Term(iTRAQ4plex); C8(Carbam	1	1
[K].aTVLNYLPk.[C]	N-Term(iTRAQ4plex); K9(iTRAQ4	2	2
[K].sLNEEAVk.[K]	N-Term(iTRAQ4plex); K8(iTRAQ4	1	1
[K].SIYkPGQTVk.[F]	K4(iTRAQ4plex); K10(iTRAQ4ple	1	1
[R].tGTHGLLVk.[Q]	N-Term(iTRAQ4plex); K9(iTRAQ4	1	1
[K].eTTFNSLLcPSGGEVSEELSLk.[L]	N-Term(iTRAQ4plex); C9(Carbam	1	1
[K].hYDGSYSTFGER.[Y]	N-Term(iTRAQ4plex)	1	1
[K].ySDASDcHGEDSQAFcEk.[F]	N-Term(iTRAQ4plex); C7(Carbam	1	1
[R].dLkPAIVk.[V]	N-Term(iTRAQ4plex); K3(iTRAQ4	1	1
[K].lHTEAQIQEEGTVVELTGR.[Q]	N-Term(iTRAQ4plex)	1	1
[K].lPPNVVEESAR.[A]	N-Term(iTRAQ4plex)	1	1
[K].aAQVTIQSSGTFSSk.[F]	N-Term(iTRAQ4plex); K15(iTRAQ	1	1
[R].lVHVEEPHTETVR.[K]	N-Term(iTRAQ4plex)	1	1
[K].lPPNVVEESAR.[A]	N-Term(iTRAQ4plex)	1	1
[R].sSGSLLNNAIk.[G]	N-Term(iTRAQ4plex); K11(iTRAQ	2	2
[R].dLkPAIVk.[V]	N-Term(iTRAQ4plex); K3(iTRAQ4	1	1
[K].AIGYLNTGYQR.[Q]		1	1
[K].aAQVTIQSSGTFSSk.[F]	N-Term(iTRAQ4plex); K15(iTRAQ	1	1
[R].gEAFTLk.[A]	N-Term(iTRAQ4plex); K7(iTRAQ4	1	1
[R].dLkPAIVk.[V]	N-Term(iTRAQ4plex); K3(iTRAQ4	1	1
[K].ySDASDcHGEDSQAFcEk.[F]	N-Term(iTRAQ4plex); C7(Carbam	1	1
[K].mVSGFIPLkPTVk.[M]	N-Term(iTRAQ4plex); K9(iTRAQ4	2	2
[K].mcPQLQQYEmHGPEGLR.[V]	N-Term(iTRAQ4plex); C2(Carbam	1	1
[K].lHTEAQIQEEGTVVELTGR.[Q]	N-Term(iTRAQ4plex)	1	1
[K].aAQVTIQSSGTFSSk.[F]	N-Term(iTRAQ4plex); K15(iTRAQ	1	1
[R].sSGSLLNNAIk.[G]	N-Term(iTRAQ4plex); K11(iTRAQ	2	2
[R].TGTHGLLVk.[Q]	K9(iTRAQ4plex)	1	1
[K].sLNEEAVk.[K]	N-Term(iTRAQ4plex); K8(iTRAQ4	1	1
[R].tGTHGLLVk.[Q]	N-Term(iTRAQ4plex); K9(iTRAQ4	1	1
[R].nALFcLESAWk.[T]	N-Term(iTRAQ4plex); C5(Carbam	1	1
[R].kDTVIkPLLVEPEGLEk.[E]	N-Term(iTRAQ4plex); K1(iTRAQ4	1	1
[K].lPPNVVEESAR.[A]	N-Term(iTRAQ4plex)	1	1
[K].sIYkPGQTVk.[F]	N-Term(iTRAQ4plex); K4(iTRAQ4	1	1
[R].tEHPFTVEEFVLPk.[F]	N-Term(iTRAQ4plex); K14(iTRAQ	1	1
[K].aAQVTIQSSGTFSSk.[F]	N-Term(iTRAQ4plex); K15(iTRAQ	1	1
[K].lPPNVVEESAR.[A]	N-Term(iTRAQ4plex)	1	1
[K].sSSNEEVMFLTVQVk.[G]	N-Term(iTRAQ4plex); K15(iTRAQ	1	1
[K].mcPQLQQYEMHGPEGLR.[V]	N-Term(iTRAQ4plex); M1(Oxidat	1	1
[R].sSGSLLNNAIk.[G]	N-Term(iTRAQ4plex); K11(iTRAQ	2	2
[K].aAQVTIQSSGTFSSk.[F]	N-Term(iTRAQ4plex); K15(iTRAQ	1	1
[K].vYDYYETDEFAIAEYNAPcSk.[D]	N-Term(iTRAQ4plex); C19(Carbar	1	1
[R].tEVSSNHVLIYLDk.[V]	N-Term(iTRAQ4plex); K14(iTRAQ	1	1
[R].vGFYESDVMGR.[G]	N-Term(iTRAQ4plex)	1	1
[R].vTAAPQSVcALR.[A]	N-Term(iTRAQ4plex); C9(Carbam	1	1
[K].yDVENcLANk.[V]	N-Term(iTRAQ4plex); C6(Carbam	1	1
[K].aTVLNYLPk.[C]	N-Term(iTRAQ4plex); K9(iTRAQ4	2	2
[K].lPPNVVEESAR.[A]	N-Term(iTRAQ4plex)	1	1
[R].iAQWQSFQLEGGLk.[Q]	N-Term(iTRAQ4plex); K14(iTRAQ	1	1
[K].yDVENcLANk.[V]	N-Term(iTRAQ4plex); C6(Carbam	1	1

[K].lHTEAQIQEEGTVVELTGR.[Q]	N-Term(iTRAQ4plex)	1	1
[R].dLkPAIVk.[V]	N-Term(iTRAQ4plex); K3(iTRAQ4	1	1
[K].LPPNVVEESAR.[A]		1	1
[K].eEFPFALGVQTLPQTcDEPk.[A]	N-Term(iTRAQ4plex); C16(Carbar	1	1
[K].GHFSISIPVk.[S]	K10(iTRAQ4plex)	1	1
[K].fSGQLNSHGcFYQQVk.[T]	N-Term(iTRAQ4plex); C10(Carbar	1	1
[R].sASNMAIVDVk.[M]	N-Term(iTRAQ4plex); K11(iTRAQ	1	1
[K].sDIAPVAR.[L]	N-Term(iTRAQ4plex)	1	1
[R].tEVSSNHVLIYLDk.[V]	N-Term(iTRAQ4plex); K14(iTRAQ	1	1
[K].tAQEGDHGSHVYTk.[A]	N-Term(iTRAQ4plex); K14(iTRAQ	1	1
[K].lHTEAQIQEEGTVVELTGR.[Q]	N-Term(iTRAQ4plex)	1	1
[R].SASNMAIVDVk.[M]	K11(iTRAQ4plex)	1	1
[K].ySDASDcHGEDSQAFcEk.[F]	N-Term(iTRAQ4plex); C7(Carbam	1	1
[K].aTVLNYLPk.[C]	N-Term(iTRAQ4plex); K9(iTRAQ4	2	2
[K].SLNEEAVk.[K]	K8(iTRAQ4plex)	1	1
[K].fQVDNNNR.[L]	N-Term(iTRAQ4plex)	1	1
[K].vTGEGcVYLQTSLk.[Y]	N-Term(iTRAQ4plex); C6(Carbam	1	1
[K].aAQVTIQSSGTFSSk.[F]	N-Term(iTRAQ4plex); K15(iTRAQ	1	1
[K].ySDASDcHGEDSQAFcEk.[F]	N-Term(iTRAQ4plex); C7(Carbam	1	1
[K].TAQEGDHGSHVYTk.[A]	K14(iTRAQ4plex)	1	1
[K].lPPNVVEESAR.[A]	N-Term(iTRAQ4plex)	1	1
[K].vDLSFSPSQSLPASHAHLR.[V]	N-Term(iTRAQ4plex)	1	1
[R].tEHPFTVEEFVLPk.[F]	N-Term(iTRAQ4plex); K14(iTRAQ	1	1
[K].sLNEEAVk.[K]	N-Term(iTRAQ4plex); K8(iTRAQ4	1	1
[K].EQAPHcIcANGR.[Q]	C6(Carbamidomethyl); C8(Carba	1	1
[R].tEVSSNHVLIYLDk.[V]	N-Term(iTRAQ4plex); K14(iTRAQ	1	1
[K].sIYkPGQTVk.[F]	N-Term(iTRAQ4plex); K4(iTRAQ4	1	1
[K].qQNAQGGFSSTQDTVVALHALSk.[Y]	N-Term(iTRAQ4plex); K23(iTRAQ	1	1
[K].aAQVTIQSSGTFSSk.[F]	N-Term(iTRAQ4plex); K15(iTRAQ	1	1
[K].vTGEGcVYLQTSLk.[Y]	N-Term(iTRAQ4plex); C6(Carbam	1	1
[K].fSGQLNSHGcFYQQVk.[T]	N-Term(iTRAQ4plex); C10(Carbar	1	1
[K].LPPNVVEESAR.[A]		1	1
[R].LVHVEEPHTETVR.[K]		1	1
[K].dTVIkPLLVEPEGLEk.[E]	N-Term(iTRAQ4plex); K5(iTRAQ4	1	1
[K].vDLSFSPSQSLPASHAHLR.[V]	N-Term(iTRAQ4plex)	1	1
[K].hYDGSYSTFGER.[Y]	N-Term(iTRAQ4plex)	1	1
[K].fQVDNNNR.[L]	N-Term(iTRAQ4plex)	1	1
[K].sSSNEEVMFLTVQVk.[G]	N-Term(iTRAQ4plex); K15(iTRAQ	1	1
[R].vGFYESDVMGR.[G]	N-Term(iTRAQ4plex)	1	1
[R].sASNMAIVDVk.[M]	N-Term(iTRAQ4plex); K11(iTRAQ	1	1
[K].lHTEAQIQEEGTVVELTGR.[Q]	N-Term(iTRAQ4plex)	1	1
[K].mcPQLQQYEmHGPEGLR.[V]	N-Term(iTRAQ4plex); C2(Carbam	1	1
[K].nEDSLVFVQTDk.[S]	N-Term(iTRAQ4plex); K12(iTRAQ	1	1
[K].gQGTLSVVTMYHAk.[A]	N-Term(iTRAQ4plex); K14(iTRAQ	1	1
[K].gLEVTITAR.[F]	N-Term(iTRAQ4plex)	1	1
[R].tVmVNIENPEGIPVk.[Q]	N-Term(iTRAQ4plex); M3(Oxidat	1	1
[R].tVmVNIENPEGIPVk.[Q]	N-Term(iTRAQ4plex); M3(Oxidat	1	1
[K].aGDFLEANYmNLQR.[S]	N-Term(iTRAQ4plex); M10(Oxida	1	1
[R].tVmVNIENPEGIPVk.[Q]	N-Term(iTRAQ4plex); M3(Oxidat	1	1
[K].lSINTHPSQkPLSITVR.[T]	N-Term(iTRAQ4plex); K10(iTRAQ	1	1
[R].tVmVNIENPEGIPVk.[Q]	N-Term(iTRAQ4plex); M3(Oxidat	1	1
[K].aGDFLEANYmNLQR.[S]	N-Term(iTRAQ4plex); M10(Oxida	1	1

[K].LSINTHPSQkPLSITVR.[T]	K10(iTRAQ4plex)	1	1
[K].LSINTHPSQkPLSITVR.[T]	K10(iTRAQ4plex)	1	1
[K].lSINTHPSQkPLSITVR.[T]	N-Term(iTRAQ4plex); K10(iTRAQ4	1	1
[R].tVmVNIENPEGIPVk.[Q]	N-Term(iTRAQ4plex); M3(Oxidat	1	1
[K].AGDFLEANYMNLQR.[S]		1	1
[R].vPVAVQGEDTVQSLTQGDGVAK.[L]	N-Term(iTRAQ4plex)	1	1
[R].vPVAVQGEDTVQSLTQGDGVAk.[L]	N-Term(iTRAQ4plex); K22(iTRAQ4	1	1
[K].AGDFLEANYMNLQR.[S]		1	1
[K].dAPDHQELNLDVSLQLPSR.[S]	N-Term(iTRAQ4plex)	1	1
[K].dFDFVPPVVR.[W]	N-Term(iTRAQ4plex)	1	1
[R].iLLQGTPVAQmTEDAVDAER.[L]	N-Term(iTRAQ4plex); M11(Oxida	1	1
[R].iLLQGTPVAQmTEDAVDAER.[L]	N-Term(iTRAQ4plex); M11(Oxida	1	1
[R].iLLQGTPVAQmTEDAVDAER.[L]	N-Term(iTRAQ4plex); M11(Oxida	1	1
[R].eVVADSVWVDVk.[D]	N-Term(iTRAQ4plex); K12(iTRAQ4	1	1
[R].eVVADSVWVDVk.[D]	N-Term(iTRAQ4plex); K12(iTRAQ4	1	1
[K].qkPDGVFQEDAPVIHQEMIGGLR.[N]	N-Term(iTRAQ4plex); K2(iTRAQ4	1	1
[R].tVMVNIENPEGIPVk.[Q]	N-Term(iTRAQ4plex); K15(iTRAQ4	1	1
[R].tVMVNIENPEGIPVk.[Q]	N-Term(iTRAQ4plex); K15(iTRAQ4	1	1
[R].tVMVNIENPEGIPVk.[Q]	N-Term(iTRAQ4plex); K15(iTRAQ4	1	1
[R].tVMVNIENPEGIPVk.[Q]	N-Term(iTRAQ4plex); K15(iTRAQ4	1	1
[K].qKPDGVFQEDAPVIHQEMIGGLR.[N]	N-Term(iTRAQ4plex)	1	1
[K].QkPDGVFQEDAPVIHQEMIGGLR.[N]	K2(iTRAQ4plex)	1	1
[K].qKPDGVFQEDAPVIHQEMIGGLR.[N]	N-Term(iTRAQ4plex)	1	1
[K].QkPDGVFQEDAPVIHQEMIGGLR.[N]	K2(iTRAQ4plex)	1	1
[R].tVMVNIENPEGIPVk.[Q]	N-Term(iTRAQ4plex); K15(iTRAQ4	1	1
[K].qkPDGVFQEDAPVIHQEMIGGLR.[N]	N-Term(iTRAQ4plex); K2(iTRAQ4	1	1
[K].qkPDGVFQEDAPVIHQEMIGGLR.[N]	N-Term(iTRAQ4plex); K2(iTRAQ4	1	1
[K].qKPDGVFQEDAPVIHQEMIGGLR.[N]	N-Term(iTRAQ4plex)	1	1
[K].QkPDGVFQEDAPVIHQEMIGGLR.[N]	K2(iTRAQ4plex)	1	1
[K].qkPDGVFQEDAPVIHQEMIGGLR.[N]	N-Term(iTRAQ4plex); K2(iTRAQ4	1	1
[K].qkPDGVFQEDAPVIHQEmIGGLR.[N]	N-Term(iTRAQ4plex); K2(iTRAQ4	1	1
[K].TIYTPGSTVLYR.[I]		1	1
[K].TIYTPGSTVLYR.[I]		1	1
[K].lSINTHPSQkPLSITVR.[T]	N-Term(iTRAQ4plex); K10(iTRAQ4	1	1
[K].lSINTHPSQkPLSITVR.[T]	N-Term(iTRAQ4plex); K10(iTRAQ4	1	1
[K].lSINTHPSQkPLSITVR.[T]	N-Term(iTRAQ4plex); K10(iTRAQ4	1	1
[K].lSINTHPSQkPLSITVR.[T]	N-Term(iTRAQ4plex); K10(iTRAQ4	1	1
[K].lSINTHPSQkPLSITVR.[T]	N-Term(iTRAQ4plex); K10(iTRAQ4	1	1
[R].IHWESASLLR.[S]		1	1
[K].rPQDAkNTMILEIcTR.[Y]	N-Term(iTRAQ4plex); K6(iTRAQ4	1	1
[K].rPQDAkNTMILEIcTR.[Y]	N-Term(iTRAQ4plex); K6(iTRAQ4	1	1
[K].vYAYYNLEEScTR.[F]	N-Term(iTRAQ4plex); C11(Carbar	1	1
[R].IFTVNHkLLPVGR.[T]	K7(iTRAQ4plex)	1	1
[K].vYAYYNLEEScTR.[F]	N-Term(iTRAQ4plex); C11(Carbar	1	1
[K].rPQDAkNTMILEIcTR.[Y]	N-Term(iTRAQ4plex); K6(iTRAQ4	1	1
[K].lSINTHPSQkPLSITVR.[T]	N-Term(iTRAQ4plex); K10(iTRAQ4	1	1
[K].vYAYYNLEEScTR.[F]	N-Term(iTRAQ4plex); C11(Carbar	1	1
[K].vYAYYNLEEScTR.[F]	N-Term(iTRAQ4plex); C11(Carbar	1	1
[K].VSHSEDDcLAFk.[V]	C8(Carbamidomethyl); K12(iTRA	1	1
[R].kVLLDGVQNPR.[A]	N-Term(iTRAQ4plex); K1(iTRAQ4	1	1
[K].kGYTQQLAFR.[Q]	N-Term(iTRAQ4plex); K1(iTRAQ4	1	1
[K].kGYTQQLAFR.[Q]	N-Term(iTRAQ4plex); K1(iTRAQ4	1	1

[K].kGYTQQLAFR.[Q]	N-Term(iTRAQ4plex); K1(iTRAQ4	1	1
[K].kGYTQQLAFR.[Q]	N-Term(iTRAQ4plex); K1(iTRAQ4	1	1
[K].kGYTQQLAFR.[Q]	N-Term(iTRAQ4plex); K1(iTRAQ4	1	1
[K].kQELSEAEQATR.[T]	N-Term(iTRAQ4plex); K1(iTRAQ4	1	1
[R].hQQTVTIPPk.[S]	N-Term(iTRAQ4plex); K10(iTRAQ	1	1
[K].sGSDEVQVGQQR.[T]	N-Term(iTRAQ4plex)	1	1
[K].VTIkPAPETEk.[R]	K4(iTRAQ4plex); K11(iTRAQ4plex	1	1
[R].hQQTVTIPPk.[S]	N-Term(iTRAQ4plex); K10(iTRAQ	1	1
[K].aAVYHHFISDGVR.[K]	N-Term(iTRAQ4plex)	1	1
[R].hQQTVTIPPk.[S]	N-Term(iTRAQ4plex); K10(iTRAQ	1	1
[R].kVLLDGVQNPR.[A]	N-Term(iTRAQ4plex); K1(iTRAQ4	1	1
[K].sLkVVPEGIR.[M]	N-Term(iTRAQ4plex); K3(iTRAQ4	1	1
[K].gQGTLSVVTmYHAk.[A]	N-Term(iTRAQ4plex); M10(Oxida	1	1
[K].gQGTLSVVTMYHAk.[A]	N-Term(iTRAQ4plex); K14(iTRAQ	1	1
[R].kSLKVVPEGIR.[M]	N-Term(iTRAQ4plex); K1(iTRAQ4	1	1
[R].kSLKVVPEGIR.[M]	N-Term(iTRAQ4plex); K1(iTRAQ4	1	1
[R].fYHPEkEDGk.[L]	N-Term(iTRAQ4plex); K6(iTRAQ4	1	1
[K].aAVYHHFISDGVR.[K]	N-Term(iTRAQ4plex)	1	1
[K].aAVYHHFISDGVR.[K]	N-Term(iTRAQ4plex)	1	1
[K].aAVYHHFISDGVR.[K]	N-Term(iTRAQ4plex)	1	1
[R].kVLLDGVQNPR.[A]	N-Term(iTRAQ4plex); K1(iTRAQ4	1	1
[K].aAVYHHFISDGVR.[K]	N-Term(iTRAQ4plex)	1	1
[K].aAVYHHFISDGVR.[K]	N-Term(iTRAQ4plex)	1	1
[K].aAVYHHFISDGVR.[K]	N-Term(iTRAQ4plex)	1	1
[K].aAVYHHFISDGVR.[K]	N-Term(iTRAQ4plex)	1	1
[K].aAVYHHFISDGVR.[K]	N-Term(iTRAQ4plex)	1	1
[K].aAVYHHFISDGVR.[K]	N-Term(iTRAQ4plex)	1	1
[R].lESEETMVLEAHDAQGDVPVTVTVHDFPG	N-Term(iTRAQ4plex); K30(iTRAQ	1	1
[R].ILLQGTPVAQMTEDAVDAER.[L]		1	1
[R].ILLQGTPVAQMTEDAVDAER.[L]		1	1
[R].ILLQGTPVAQMTEDAVDAER.[L]		1	1
[R].ILLQGTPVAQMTEDAVDAER.[L]		1	1
[R].aYYENSPQQVFSTEFEVk.[E]	N-Term(iTRAQ4plex); K18(iTRAQ	1	1
[R].aYYENSPQQVFSTEFEVk.[E]	N-Term(iTRAQ4plex); K18(iTRAQ	1	1
[K].lSINTHPSQkPLSITVR.[T]	N-Term(iTRAQ4plex); K10(iTRAQ	1	1
[R].ILLQGTPVAQMTEDAVDAER.[L]		1	1
[K].eYVLPSFEVIVEPTEk.[F]	N-Term(iTRAQ4plex); K16(iTRAQ	1	1
[K].eYVLPSFEVIVEPTEk.[F]	N-Term(iTRAQ4plex); K16(iTRAQ	1	1
[K].lSINTHPSQkPLSITVR.[T]	N-Term(iTRAQ4plex); K10(iTRAQ	1	1
[R].lESEETMVLEAHDAQGDVPVTVTVHDFPG	N-Term(iTRAQ4plex); K30(iTRAQ	1	1
[R].lESEETMVLEAHDAQGDVPVTVTVHDFPG	N-Term(iTRAQ4plex); K30(iTRAQ	1	1
[R].lESEETMVLEAHDAQGDVPVTVTVHDFPG	N-Term(iTRAQ4plex); K30(iTRAQ	1	1
[R].lESEETMVLEAHDAQGDVPVTVTVHDFPG	N-Term(iTRAQ4plex); K30(iTRAQ	1	1
[R].lESEETMVLEAHDAQGDVPVTVTVHDFPG	N-Term(iTRAQ4plex); K30(iTRAQ	1	1
[R].lESEETMVLEAHDAQGDVPVTVTVHDFPG	N-Term(iTRAQ4plex); K30(iTRAQ	1	1
[R].lESEETMVLEAHDAQGDVPVTVTVHDFPG	N-Term(iTRAQ4plex); K30(iTRAQ	1	1
[R].lESEETMVLEAHDAQGDVPVTVTVHDFPG	N-Term(iTRAQ4plex); K30(iTRAQ	1	1
[R].lESEETMVLEAHDAQGDVPVTVTVHDFPG	N-Term(iTRAQ4plex); K30(iTRAQ	1	1
[R].lESEETMVLEAHDAQGDVPVTVTVHDFPG	N-Term(iTRAQ4plex); K30(iTRAQ	1	1
[R].aYYENSPQQVFSTEFEVk.[E]	N-Term(iTRAQ4plex); K18(iTRAQ	1	1
[R].aYYENSPQQVFSTEFEVk.[E]	N-Term(iTRAQ4plex); K18(iTRAQ	1	1
[R].lESEETmVLEAHDAQGDVPVTVTVHDFPG	N-Term(iTRAQ4plex); M7(Oxidat	1	1

[R].aYYENSPQQVFSTEFEVk.[E]	N-Term(iTRAQ4plex); K18(iTRAQ	1	1
[R].aYYENSPQQVFSTEFEVk.[E]	N-Term(iTRAQ4plex); K18(iTRAQ	1	1
[R].aYYENSPQQVFSTEFEVk.[E]	N-Term(iTRAQ4plex); K18(iTRAQ	1	1
[R].aYYENSPQQVFSTEFEVk.[E]	N-Term(iTRAQ4plex); K18(iTRAQ	1	1
[R].aYYENSPQQVFSTEFEVk.[E]	N-Term(iTRAQ4plex); K18(iTRAQ	1	1
[R].aYYENSPQQVFSTEFEVk.[E]	N-Term(iTRAQ4plex); K18(iTRAQ	1	1
[R].aYYENSPQQVFSTEFEVk.[E]	N-Term(iTRAQ4plex); K18(iTRAQ	1	1
[R].aYYENSPQQVFSTEFEVk.[E]	N-Term(iTRAQ4plex); K18(iTRAQ	1	1
[R].AYYENSPQQVFSTEFEVk.[E]	K18(iTRAQ4plex)	1	1
[R].lESEETMVLEAHDAQGDVPVTVTVHDFPG	N-Term(iTRAQ4plex); K30(iTRAQ	1	1
[R].lESEETMVLEAHDAQGDVPVTVTVHDFPG	N-Term(iTRAQ4plex); K30(iTRAQ	1	1
[R].lESEETMVLEAHDAQGDVPVTVTVHDFPG	N-Term(iTRAQ4plex); K30(iTRAQ	1	1
[K].vHQYFNVELIQPGAVk.[V]	N-Term(iTRAQ4plex); K16(iTRAQ	1	1
[R].lESEETMVLEAHDAQGDVPVTVTVHDFPG	N-Term(iTRAQ4plex); K30(iTRAQ	1	1
[R].lESEETMVLEAHDAQGDVPVTVTVHDFPG	N-Term(iTRAQ4plex); K30(iTRAQ	1	1
[R].lESEETMVLEAHDAQGDVPVTVTVHDFPG	N-Term(iTRAQ4plex); K30(iTRAQ	1	1
[R].lESEETMVLEAHDAQGDVPVTVTVHDFPG	N-Term(iTRAQ4plex); K30(iTRAQ	1	1
[R].lESEETMVLEAHDAQGDVPVTVTVHDFPG	N-Term(iTRAQ4plex); K30(iTRAQ	1	1
[R].lESEETMVLEAHDAQGDVPVTVTVHDFPG	N-Term(iTRAQ4plex); K30(iTRAQ	1	1
[R].lESEETMVLEAHDAQGDVPVTVTVHDFPG	N-Term(iTRAQ4plex); K30(iTRAQ	1	1
[R].lESEETMVLEAHDAQGDVPVTVTVHDFPG	N-Term(iTRAQ4plex); K30(iTRAQ	1	1
[R].lESEETMVLEAHDAQGDVPVTVTVHDFPG	N-Term(iTRAQ4plex); K30(iTRAQ	1	1
[R].iPIEDGSGEVVLSR.[K]	N-Term(iTRAQ4plex)	1	1
[R].iPIEDGSGEVVLSR.[K]	N-Term(iTRAQ4plex)	1	1
[R].iPIEDGSGEVVLSR.[K]	N-Term(iTRAQ4plex)	1	1
[R].tVmVNIENPEGIPVk.[Q]	N-Term(iTRAQ4plex); M3(Oxidat	1	1
[R].eVVADSVWVDVk.[D]	N-Term(iTRAQ4plex); K12(iTRAQ	1	1
[R].VPVAVQGEDTVQSLTQGDGVAk.[L]	K22(iTRAQ4plex)	1	1
[R].VPVAVQGEDTVQSLTQGDGVAk.[L]	K22(iTRAQ4plex)	1	1
[R].iPIEDGSGEVVLSR.[K]	N-Term(iTRAQ4plex)	1	1
[R].vPVAVQGEDTVQSLTQGDGVAk.[L]	N-Term(iTRAQ4plex); K22(iTRAQ	1	1
[R].vPVAVQGEDTVQSLTQGDGVAk.[L]	N-Term(iTRAQ4plex); K22(iTRAQ	1	1
[R].vPVAVQGEDTVQSLTQGDGVAk.[L]	N-Term(iTRAQ4plex); K22(iTRAQ	1	1
[R].vPVAVQGEDTVQSLTQGDGVAk.[L]	N-Term(iTRAQ4plex); K22(iTRAQ	1	1
[R].vPVAVQGEDTVQSLTQGDGVAk.[L]	N-Term(iTRAQ4plex); K22(iTRAQ	1	1
[K].dAPDHQELNLDVSLQLPSR.[S]	N-Term(iTRAQ4plex)	1	1
[K].dAPDHQELNLDVSLQLPSR.[S]	N-Term(iTRAQ4plex)	1	1
[R].eVVADSVWVDVk.[D]	N-Term(iTRAQ4plex); K12(iTRAQ	1	1
[R].eVVADSVWVDVk.[D]	N-Term(iTRAQ4plex); K12(iTRAQ	1	1
[R].eVVADSVWVDVk.[D]	N-Term(iTRAQ4plex); K12(iTRAQ	1	1
[R].eVVADSVWVDVk.[D]	N-Term(iTRAQ4plex); K12(iTRAQ	1	1
[R].TVMVNIENPEGIPVk.[Q]	K15(iTRAQ4plex)	1	1
[R].eVVADSVWVDVk.[D]	N-Term(iTRAQ4plex); K12(iTRAQ	1	1
[R].eVVADSVWVDVk.[D]	N-Term(iTRAQ4plex); K12(iTRAQ	1	1
[R].eVVADSVWVDVk.[D]	N-Term(iTRAQ4plex); K12(iTRAQ	1	1
[R].eVVADSVWVDVk.[D]	N-Term(iTRAQ4plex); K12(iTRAQ	1	1
[R].eVVADSVWVDVk.[D]	N-Term(iTRAQ4plex); K12(iTRAQ	1	1
[R].eVVADSVWVDVk.[D]	N-Term(iTRAQ4plex); K12(iTRAQ	1	1
[R].eVVADSVWVDVk.[D]	N-Term(iTRAQ4plex); K12(iTRAQ	1	1
[R].eVVADSVWVDVk.[D]	N-Term(iTRAQ4plex); K12(iTRAQ	1	1
[R].eVVADSVWVDVk.[D]	N-Term(iTRAQ4plex); K12(iTRAQ	1	1
[R].eVVADSVWVDVk.[D]	N-Term(iTRAQ4plex); K12(iTRAQ	1	1

[K].vLLDGVQNPR.[A]	N-Term(iTRAQ4plex)	1	1
[K].gYTQQLAFR.[Q]	N-Term(iTRAQ4plex)	1	1
[R].VVLVAVDk.[G]	K8(iTRAQ4plex)	1	1
[K].rQGALELIk.[K]	N-Term(iTRAQ4plex); K9(iTRAQ4	1	1
[R].iHWESASLLR.[S]	N-Term(iTRAQ4plex)	1	1
[K].rQGALELIk.[K]	N-Term(iTRAQ4plex); K9(iTRAQ4	1	1
[R].iHWESASLLR.[S]	N-Term(iTRAQ4plex)	1	1
[R].qPVPGQQMTLk.[I]	N-Term(iTRAQ4plex); K11(iTRAQ	1	1
[R].qPVPGQQMTLk.[I]	N-Term(iTRAQ4plex); K11(iTRAQ	1	1
[K].gQGTLSVVTmYHAk.[A]	N-Term(iTRAQ4plex); M10(Oxida	1	1
[R].qPVPGQQMTLk.[I]	N-Term(iTRAQ4plex); K11(iTRAQ	1	1
[R].qPVPGQQMTLk.[I]	N-Term(iTRAQ4plex); K11(iTRAQ	1	1
[K].gQGTLSVVTmYHAk.[A]	N-Term(iTRAQ4plex); M10(Oxida	1	1
[K].aAVYHHFISDGVR.[K]	N-Term(iTRAQ4plex)	1	1
[K].gYTQQLAFR.[Q]	N-Term(iTRAQ4plex)	1	1
[R].vVLVAVDk.[G]	N-Term(iTRAQ4plex); K8(iTRAQ4	1	1
[K].lSINTHPSQkPLSITVR.[T]	N-Term(iTRAQ4plex); K10(iTRAQ	1	1
[K].lSINTHPSQkPLSITVR.[T]	N-Term(iTRAQ4plex); K10(iTRAQ	1	1
[K].lSINTHPSQkPLSITVR.[T]	N-Term(iTRAQ4plex); K10(iTRAQ	1	1
[K].gLEVTITAR.[F]	N-Term(iTRAQ4plex)	1	1
[K].gLEVTITAR.[F]	N-Term(iTRAQ4plex)	1	1
[K].vLLDGVQNPR.[A]	N-Term(iTRAQ4plex)	1	1
[K].gLEVTITAR.[F]	N-Term(iTRAQ4plex)	1	1
[K].gLEVTITAR.[F]	N-Term(iTRAQ4plex)	1	1
[R].iHWESASLLR.[S]	N-Term(iTRAQ4plex)	1	1
[R].iHWESASLLR.[S]	N-Term(iTRAQ4plex)	1	1
[R].rHQQTVTIPPk.[S]	N-Term(iTRAQ4plex); K11(iTRAQ	1	1
[R].iHWESASLLR.[S]	N-Term(iTRAQ4plex)	1	1
[R].rHQQTVTIPPk.[S]	N-Term(iTRAQ4plex); K11(iTRAQ	1	1
[R].rHQQTVTIPPk.[S]	N-Term(iTRAQ4plex); K11(iTRAQ	1	1
[R].rHQQTVTIPPk.[S]	N-Term(iTRAQ4plex); K11(iTRAQ	1	1
[K].sGSDEVQVGQQR.[T]	N-Term(iTRAQ4plex)	1	1
[R].rHQQTVTIPPk.[S]	N-Term(iTRAQ4plex); K11(iTRAQ	1	1
[R].iHWESASLLR.[S]	N-Term(iTRAQ4plex)	1	1
[R].iHWESASLLR.[S]	N-Term(iTRAQ4plex)	1	1
[R].iHWESASLLR.[S]	N-Term(iTRAQ4plex)	1	1
[K].kLVLSSEk.[T]	N-Term(iTRAQ4plex); K1(iTRAQ4	1	1
[R].hQQTVTIPPk.[S]	N-Term(iTRAQ4plex); K10(iTRAQ	1	1
[R].hQQTVTIPPk.[S]	N-Term(iTRAQ4plex); K10(iTRAQ	1	1
[K].kQELSEAEQATR.[T]	N-Term(iTRAQ4plex); K1(iTRAQ4	1	1
[R].hQQTVTIPPk.[S]	N-Term(iTRAQ4plex); K10(iTRAQ	1	1
[R].rHQQTVTIPPk.[S]	N-Term(iTRAQ4plex); K11(iTRAQ	1	1
[R].hQQTVTIPPk.[S]	N-Term(iTRAQ4plex); K10(iTRAQ	1	1
[R].hQQTVTIPPk.[S]	N-Term(iTRAQ4plex); K10(iTRAQ	1	1
[R].hQQTVTIPPk.[S]	N-Term(iTRAQ4plex); K10(iTRAQ	1	1
[R].hQQTVTIPPk.[S]	N-Term(iTRAQ4plex); K10(iTRAQ	1	1
[R].hQQTVTIPPk.[S]	N-Term(iTRAQ4plex); K10(iTRAQ	1	1
[K].kLVLSSEk.[T]	N-Term(iTRAQ4plex); K1(iTRAQ4	1	1
[K].kVEGTAFVIFGIQDGEQR.[I]	N-Term(iTRAQ4plex); K1(iTRAQ4	1	1
[K].dYAGVFSDAGLTFTSSSGQQTAQR.[A]	N-Term(iTRAQ4plex)	1	1
[K].fVTVQATFGTQVVEk.[V]	N-Term(iTRAQ4plex); K15(iTRAQ	1	1
[K].kVEGTAFVIFGIQDGEQR.[I]	N-Term(iTRAQ4plex); K1(iTRAQ4	1	1

[R].vVLVAVDk.[G]	N-Term(iTRAQ4plex); K8(iTRAQ4	1	1
[K].kVEGTAFVIFGIQDGEQR.[I]	N-Term(iTRAQ4plex); K1(iTRAQ4	1	1
[K].dYAGVFSDAGLTFTSSSGQQTAQR.[A]	N-Term(iTRAQ4plex)	1	1
[R].iSLPESLkR.[I]	N-Term(iTRAQ4plex); K8(iTRAQ4	1	1
[K].kVEGTAFVIFGIQDGEQR.[I]	N-Term(iTRAQ4plex); K1(iTRAQ4	1	1
[K].kVEGTAFVIFGIQDGEQR.[I]	N-Term(iTRAQ4plex); K1(iTRAQ4	1	1
[K].dYAGVFSDAGLTFTSSSGQQTAQR.[A]	N-Term(iTRAQ4plex)	1	1
[K].gQGTLSVVTMYHAk.[A]	N-Term(iTRAQ4plex); K14(iTRAQ	1	1
[K].gQGTLSVVTMYHAk.[A]	N-Term(iTRAQ4plex); K14(iTRAQ	1	1
[K].kVEGTAFVIFGIQDGEQR.[I]	N-Term(iTRAQ4plex); K1(iTRAQ4	1	1
[R].iHWESASLLR.[S]	N-Term(iTRAQ4plex)	1	1
[K].kVEGTAFVIFGIQDGEQR.[I]	N-Term(iTRAQ4plex); K1(iTRAQ4	1	1
[K].aAVYHHFISDGVR.[K]	N-Term(iTRAQ4plex)	1	1
[K].kVEGTAFVIFGIQDGEQR.[I]	N-Term(iTRAQ4plex); K1(iTRAQ4	1	1
[R].iHWESASLLR.[S]	N-Term(iTRAQ4plex)	1	1
[K].aAVYHHFISDGVR.[K]	N-Term(iTRAQ4plex)	1	1
[K].kVEGTAFVIFGIQDGEQR.[I]	N-Term(iTRAQ4plex); K1(iTRAQ4	1	1
[K].kVEGTAFVIFGIQDGEQR.[I]	N-Term(iTRAQ4plex); K1(iTRAQ4	1	1
[K].dYAGVFSDAGLTFTSSSGQQTAQR.[A]	N-Term(iTRAQ4plex)	1	1
[K].kVEGTAFVIFGIQDGEQR.[I]	N-Term(iTRAQ4plex); K1(iTRAQ4	1	1
[K].kVEGTAFVIFGIQDGEQR.[I]	N-Term(iTRAQ4plex); K1(iTRAQ4	1	1
[K].kVEGTAFVIFGIQDGEQR.[I]	N-Term(iTRAQ4plex); K1(iTRAQ4	1	1
[K].vEGTAFVIFGIQDGEQR.[I]	N-Term(iTRAQ4plex)	1	1
[K].fVTVQATFGTQVVEk.[V]	N-Term(iTRAQ4plex); K15(iTRAQ	1	1
[K].kVEGTAFVIFGIQDGEQR.[I]	N-Term(iTRAQ4plex); K1(iTRAQ4	1	1
[K].vEGTAFVIFGIQDGEQR.[I]	N-Term(iTRAQ4plex)	1	1
[K].dYAGVFSDAGLTFTSSSGQQTAQR.[A]	N-Term(iTRAQ4plex)	1	1
[K].fVTVQATFGTQVVEk.[V]	N-Term(iTRAQ4plex); K15(iTRAQ	1	1
[K].vEGTAFVIFGIQDGEQR.[I]	N-Term(iTRAQ4plex)	1	1
[K].fVTVQATFGTQVVEk.[V]	N-Term(iTRAQ4plex); K15(iTRAQ	1	1
[K].dYAGVFSDAGLTFTSSSGQQTAQR.[A]	N-Term(iTRAQ4plex)	1	1
[K].kVEGTAFVIFGIQDGEQR.[I]	N-Term(iTRAQ4plex); K1(iTRAQ4	1	1
[R].sEFPESWLWNVEDLk.[E]	N-Term(iTRAQ4plex); K15(iTRAQ	1	1
[K].dSITTWEILAVSMSDk.[K]	N-Term(iTRAQ4plex); K16(iTRAQ	1	1
[K].dSITTWEILAVSMSDk.[K]	N-Term(iTRAQ4plex); K16(iTRAQ	1	1
[R].SEFPESWLWNVEDLk.[E]	K15(iTRAQ4plex)	1	1
[R].sEFPESWLWNVEDLk.[E]	N-Term(iTRAQ4plex); K15(iTRAQ	1	1
[R].gDQDATMSILDISmMTGFAPDTDDLk.[Q]	N-Term(iTRAQ4plex); M14(Oxida	1	1
[K].fVTVQATFGTQVVEk.[V]	N-Term(iTRAQ4plex); K15(iTRAQ	1	1
[K].dYAGVFSDAGLTFTSSSGQQTAQR.[A]	N-Term(iTRAQ4plex)	1	1
[R].gDQDATMSILDISMMTGFAPDTDDLk.[Q]	N-Term(iTRAQ4plex); K26(iTRAQ	1	1
[K].QcQDLGAFTESMVVFGcPN.[-]	C2(Carbamidomethyl); C17(Carb	1	1
[K].qcQDLGAFTESMVVFGcPN.[-]	N-Term(iTRAQ4plex); C2(Carbam	1	1
[K].qcQDLGAFTESMVVFGcPN.[-]	N-Term(iTRAQ4plex); C2(Carbam	1	1
[K].DSITTWEILAVSMSDk.[K]	K16(iTRAQ4plex)	1	1
[R].gDQDATMSILDISMmTGFAPDTDDLk.[Q]	N-Term(iTRAQ4plex); M15(Oxida	1	1
[R].gDQDATMSILDISmMTGFAPDTDDLk.[Q]	N-Term(iTRAQ4plex); M14(Oxida	1	1
[R].gDQDATMSILDISMmTGFAPDTDDLk.[Q]	N-Term(iTRAQ4plex); M15(Oxida	1	1
[K].kVEGTAFVIFGIQDGEQR.[I]	N-Term(iTRAQ4plex); K1(iTRAQ4	1	1
[K].vEGTAFVIFGIQDGEQR.[I]	N-Term(iTRAQ4plex)	1	1
[K].kVEGTAFVIFGIQDGEQR.[I]	N-Term(iTRAQ4plex); K1(iTRAQ4	1	1
[K].vEGTAFVIFGIQDGEQR.[I]	N-Term(iTRAQ4plex)	1	1

[K].kVEGTAFVIFGIQDGEQR.[I]	N-Term(iTRAQ4plex); K1(iTRAQ4	1	1
[K].gQGTLSVVTmYHAk.[A]	N-Term(iTRAQ4plex); M10(Oxida	1	1
[K].aAVYHHFISDGVR.[K]	N-Term(iTRAQ4plex)	1	1
[K].aAVYHHFISDGVR.[K]	N-Term(iTRAQ4plex)	1	1
[K].vEGTAFVIFGIQDGEQR.[I]	N-Term(iTRAQ4plex)	1	1
[K].aAVYHHFISDGVR.[K]	N-Term(iTRAQ4plex)	1	1
[K].kVEGTAFVIFGIQDGEQR.[I]	N-Term(iTRAQ4plex); K1(iTRAQ4	1	1
[K].dYAGVFSDAGLTFTSSSGQQTAQR.[A]	N-Term(iTRAQ4plex)	1	1
[K].kVEGTAFVIFGIQDGEQR.[I]	N-Term(iTRAQ4plex); K1(iTRAQ4	1	1
[K].dYAGVFSDAGLTFTSSSGQQTAQR.[A]	N-Term(iTRAQ4plex)	1	1
[K].aAVYHHFISDGVR.[K]	N-Term(iTRAQ4plex)	1	1
[K].kVEGTAFVIFGIQDGEQR.[I]	N-Term(iTRAQ4plex); K1(iTRAQ4	1	1
[K].vEGTAFVIFGIQDGEQR.[I]	N-Term(iTRAQ4plex)	1	1
[K].dYAGVFSDAGLTFTSSSGQQTAQR.[A]	N-Term(iTRAQ4plex)	1	1
[K].aAVYHHFISDGVR.[K]	N-Term(iTRAQ4plex)	1	1
[K].aAVYHHFISDGVR.[K]	N-Term(iTRAQ4plex)	1	1
[K].gVFVLNkk.[N]	N-Term(iTRAQ4plex); K7(iTRAQ4	1	1
[K].aAVYHHFISDGVR.[K]	N-Term(iTRAQ4plex)	1	1
[R].lkGPLLNk.[F]	N-Term(iTRAQ4plex); K2(iTRAQ4	1	1
[K].kVEGTAFVIFGIQDGEQR.[I]	N-Term(iTRAQ4plex); K1(iTRAQ4	1	1
[K].kGYTQQLAFR.[Q]	N-Term(iTRAQ4plex); K1(iTRAQ4	1	1
[K].vEGTAFVIFGIQDGEQR.[I]	N-Term(iTRAQ4plex)	1	1
[K].dYAGVFSDAGLTFTSSSGQQTAQR.[A]	N-Term(iTRAQ4plex)	1	1
[K].aAVYHHFISDGVR.[K]	N-Term(iTRAQ4plex)	1	1
[K].aAVYHHFISDGVR.[K]	N-Term(iTRAQ4plex)	1	1
[K].aAVYHHFISDGVR.[K]	N-Term(iTRAQ4plex)	1	1
[K].aAVYHHFISDGVR.[K]	N-Term(iTRAQ4plex)	1	1
[K].aAVYHHFISDGVR.[K]	N-Term(iTRAQ4plex)	1	1
[K].aAVYHHFISDGVR.[K]	N-Term(iTRAQ4plex)	1	1
[K].vEGTAFVIFGIQDGEQR.[I]	N-Term(iTRAQ4plex)	1	1
[K].aAVYHHFISDGVR.[K]	N-Term(iTRAQ4plex)	1	1
[K].aAVYHHFISDGVR.[K]	N-Term(iTRAQ4plex)	1	1
[K].aAVYHHFISDGVR.[K]	N-Term(iTRAQ4plex)	1	1
[K].aAVYHHFISDGVR.[K]	N-Term(iTRAQ4plex)	1	1
[K].aAVYHHFISDGVR.[K]	N-Term(iTRAQ4plex)	1	1
[K].aAVYHHFISDGVR.[K]	N-Term(iTRAQ4plex)	1	1
[K].aAVYHHFISDGVR.[K]	N-Term(iTRAQ4plex)	1	1
[K].kVEGTAFVIFGIQDGEQR.[I]	N-Term(iTRAQ4plex); K1(iTRAQ4	1	1
[K].aAVYHHFISDGVR.[K]	N-Term(iTRAQ4plex)	1	1
[K].kVEGTAFVIFGIQDGEQR.[I]	N-Term(iTRAQ4plex); K1(iTRAQ4	1	1
[K].fVTVQATFGTQVVEk.[V]	N-Term(iTRAQ4plex); K15(iTRAQ4	1	1
[K].kVEGTAFVIFGIQDGEQR.[I]	N-Term(iTRAQ4plex); K1(iTRAQ4	1	1
[K].dYAGVFSDAGLTFTSSSGQQTAQR.[A]	N-Term(iTRAQ4plex)	1	1
[K].vFLDccNYITELR.[R]	N-Term(iTRAQ4plex); C5(Carbam	1	1
[R].nTLIIYLDk.[V]	N-Term(iTRAQ4plex); K9(iTRAQ4	1	1
[K].vFLDccNYITELR.[R]	N-Term(iTRAQ4plex); C5(Carbam	1	1
[K].kVEGTAFVIFGIQDGEQR.[I]	N-Term(iTRAQ4plex); K1(iTRAQ4	1	1
[K].fVTVQATFGTQVVEk.[V]	N-Term(iTRAQ4plex); K15(iTRAQ4	1	1
[K].kVEGTAFVIFGIQDGEQR.[I]	N-Term(iTRAQ4plex); K1(iTRAQ4	1	1
[K].fVTVQATFGTQVVEk.[V]	N-Term(iTRAQ4plex); K15(iTRAQ4	1	1
[K].fVTVQATFGTQVVEk.[V]	N-Term(iTRAQ4plex); K15(iTRAQ4	1	1
[K].kVEGTAFVIFGIQDGEQR.[I]	N-Term(iTRAQ4plex); K1(iTRAQ4	1	1

[K].vFLDccNYITELR.[R]	N-Term(iTRAQ4plex); C5(Carbam	1	1
[K].kVEGTAFVIFGIQDGEQR.[I]	N-Term(iTRAQ4plex); K1(iTRAQ4	1	1
[K].kVEGTAFVIFGIQDGEQR.[I]	N-Term(iTRAQ4plex); K1(iTRAQ4	1	1
[K].vFLDccNYITELR.[R]	N-Term(iTRAQ4plex); C5(Carbam	1	1
[K].dYAGVFSDAGLTFTSSSGQQTAQR.[A]	N-Term(iTRAQ4plex)	1	1
[K].dYAGVFSDAGLTFTSSSGQQTAQR.[A]	N-Term(iTRAQ4plex)	1	1
[K].dYAGVFSDAGLTFTSSSGQQTAQR.[A]	N-Term(iTRAQ4plex)	1	1
[K].dYAGVFSDAGLTFTSSSGQQTAQR.[A]	N-Term(iTRAQ4plex)	1	1
[K].fVTVQATFGTQVVEk.[V]	N-Term(iTRAQ4plex); K15(iTRAQ	1	1
[K].kVFLDccNYITELR.[R]	N-Term(iTRAQ4plex); K1(iTRAQ4	1	1
[K].VHQYFNVELIQPGAVk.[V]	K16(iTRAQ4plex)	1	1
[K].kVFLDccNYITELR.[R]	N-Term(iTRAQ4plex); K1(iTRAQ4	1	1
[K].kVFLDccNYITELR.[R]	N-Term(iTRAQ4plex); K1(iTRAQ4	1	1
[K].dYAGVFSDAGLTFTSSSGQQTAQR.[A]	N-Term(iTRAQ4plex)	1	1
[K].kVFLDccNYITELR.[R]	N-Term(iTRAQ4plex); K1(iTRAQ4	1	1
[K].dYAGVFSDAGLTFTSSSGQQTAQR.[A]	N-Term(iTRAQ4plex)	1	1
[K].kVFLDccNYITELR.[R]	N-Term(iTRAQ4plex); K1(iTRAQ4	1	1
[K].vFLDccNYITELR.[R]	N-Term(iTRAQ4plex); C5(Carbam	1	1
[K].kVEGTAFVIFGIQDGEQR.[I]	N-Term(iTRAQ4plex); K1(iTRAQ4	1	1
[K].fVTVQATFGTQVVEk.[V]	N-Term(iTRAQ4plex); K15(iTRAQ	1	1
[K].fVTVQATFGTQVVEk.[V]	N-Term(iTRAQ4plex); K15(iTRAQ	1	1
[K].kVEGTAFVIFGIQDGEQR.[I]	N-Term(iTRAQ4plex); K1(iTRAQ4	1	1
[K].fVTVQATFGTQVVEk.[V]	N-Term(iTRAQ4plex); K15(iTRAQ	1	1
[K].kVEGTAFVIFGIQDGEQR.[I]	N-Term(iTRAQ4plex); K1(iTRAQ4	1	1
[K].kVEGTAFVIFGIQDGEQR.[I]	N-Term(iTRAQ4plex); K1(iTRAQ4	1	1
[K].fVTVQATFGTQVVEk.[V]	N-Term(iTRAQ4plex); K15(iTRAQ	1	1
[K].kVEGTAFVIFGIQDGEQR.[I]	N-Term(iTRAQ4plex); K1(iTRAQ4	1	1
[K].kVEGTAFVIFGIQDGEQR.[I]	N-Term(iTRAQ4plex); K1(iTRAQ4	1	1
[K].fVTVQATFGTQVVEk.[V]	N-Term(iTRAQ4plex); K15(iTRAQ	1	1
[K].kVEGTAFVIFGIQDGEQR.[I]	N-Term(iTRAQ4plex); K1(iTRAQ4	1	1
[K].fVTVQATFGTQVVEk.[V]	N-Term(iTRAQ4plex); K15(iTRAQ	1	1
[K].kVEGTAFVIFGIQDGEQR.[I]	N-Term(iTRAQ4plex); K1(iTRAQ4	1	1
[K].kVEGTAFVIFGIQDGEQR.[I]	N-Term(iTRAQ4plex); K1(iTRAQ4	1	1
[K].kVEGTAFVIFGIQDGEQR.[I]	N-Term(iTRAQ4plex); K1(iTRAQ4	1	1
[K].fVTVQATFGTQVVEk.[V]	N-Term(iTRAQ4plex); K15(iTRAQ	1	1
[K].kVEGTAFVIFGIQDGEQR.[I]	N-Term(iTRAQ4plex); K1(iTRAQ4	1	1
[K].fVTVQATFGTQVVEk.[V]	N-Term(iTRAQ4plex); K15(iTRAQ	1	1
[K].kVEGTAFVIFGIQDGEQR.[I]	N-Term(iTRAQ4plex); K1(iTRAQ4	1	1
[K].kVEGTAFVIFGIQDGEQR.[I]	N-Term(iTRAQ4plex); K1(iTRAQ4	1	1
[K].fVTVQATFGTQVVEk.[V]	N-Term(iTRAQ4plex); K15(iTRAQ	1	1
[K].fVTVQATFGTQVVEk.[V]	N-Term(iTRAQ4plex); K15(iTRAQ	1	1
[K].kVEGTAFVIFGIQDGEQR.[I]	N-Term(iTRAQ4plex); K1(iTRAQ4	1	1
[K].fVTVQATFGTQVVEk.[V]	N-Term(iTRAQ4plex); K15(iTRAQ	1	1
[K].fVTVQATFGTQVVEk.[V]	N-Term(iTRAQ4plex); K15(iTRAQ	1	1
[K].kVEGTAFVIFGIQDGEQR.[I]	N-Term(iTRAQ4plex); K1(iTRAQ4	1	1
[K].fVTVQATFGTQVVEk.[V]	N-Term(iTRAQ4plex); K15(iTRAQ	1	1
[K].fVTVQATFGTQVVEk.[V]	N-Term(iTRAQ4plex); K15(iTRAQ	1	1
[K].fVTVQATFGTQVVEk.[V]	N-Term(iTRAQ4plex); K15(iTRAQ	1	1
[K].fVTVQATFGTQVVEk.[V]	N-Term(iTRAQ4plex); K15(iTRAQ	1	1
[K].kVEGTAFVIFGIQDGEQR.[I]	N-Term(iTRAQ4plex); K1(iTRAQ4	1	1
[K].fVTVQATFGTQVVEk.[V]	N-Term(iTRAQ4plex); K15(iTRAQ	1	1
[R].sGIPIVTSPYQIHFTk.[T]	N-Term(iTRAQ4plex); K16(iTRAQ	1	1

[K].kVEGTAFVIFGIQDGEQR.[I]	N-Term(iTRAQ4plex); K1(iTRAQ4	1	1
[K].kVEGTAFVIFGIQDGEQR.[I]	N-Term(iTRAQ4plex); K1(iTRAQ4	1	1
[K].kVEGTAFVIFGIQDGEQR.[I]	N-Term(iTRAQ4plex); K1(iTRAQ4	1	1
[K].fVTVQATFGTQVVEk.[V]	N-Term(iTRAQ4plex); K15(iTRAQ4	1	1
[K].kVEGTAFVIFGIQDGEQR.[I]	N-Term(iTRAQ4plex); K1(iTRAQ4	1	1
[K].kVEGTAFVIFGIQDGEQR.[I]	N-Term(iTRAQ4plex); K1(iTRAQ4	1	1
[K].fVTVQATFGTQVVEk.[V]	N-Term(iTRAQ4plex); K15(iTRAQ4	1	1
[K].kVEGTAFVIFGIQDGEQR.[I]	N-Term(iTRAQ4plex); K1(iTRAQ4	1	1
[K].kVEGTAFVIFGIQDGEQR.[I]	N-Term(iTRAQ4plex); K1(iTRAQ4	1	1
[K].fVTVQATFGTQVVEk.[V]	N-Term(iTRAQ4plex); K15(iTRAQ4	1	1
[K].kVEGTAFVIFGIQDGEQR.[I]	N-Term(iTRAQ4plex); K1(iTRAQ4	1	1
[K].fVTVQATFGTQVVEk.[V]	N-Term(iTRAQ4plex); K15(iTRAQ4	1	1
[K].kVEGTAFVIFGIQDGEQR.[I]	N-Term(iTRAQ4plex); K1(iTRAQ4	1	1
[K].kVEGTAFVIFGIQDGEQR.[I]	N-Term(iTRAQ4plex); K1(iTRAQ4	1	1
[K].fVTVQATFGTQVVEk.[V]	N-Term(iTRAQ4plex); K15(iTRAQ4	1	1
[K].kVEGTAFVIFGIQDGEQR.[I]	N-Term(iTRAQ4plex); K1(iTRAQ4	1	1
[K].kVEGTAFVIFGIQDGEQR.[I]	N-Term(iTRAQ4plex); K1(iTRAQ4	1	1
[K].kVEGTAFVIFGIQDGEQR.[I]	N-Term(iTRAQ4plex); K1(iTRAQ4	1	1
[K].fVTVQATFGTQVVEk.[V]	N-Term(iTRAQ4plex); K15(iTRAQ4	1	1
[K].dYAGVFSDAGLTFTSSSGQQTAQR.[A]	N-Term(iTRAQ4plex)	1	1
[R].qPSSAFAAFVk.[R]	N-Term(iTRAQ4plex); K11(iTRAQ4	1	1
[R].qPSSAFAAFVk.[R]	N-Term(iTRAQ4plex); K11(iTRAQ4	1	1
[R].qPSSAFAAFVk.[R]	N-Term(iTRAQ4plex); K11(iTRAQ4	1	1
[R].qPSSAFAAFVk.[R]	N-Term(iTRAQ4plex); K11(iTRAQ4	1	1
[K].kVEGTAFVIFGIQDGEQR.[I]	N-Term(iTRAQ4plex); K1(iTRAQ4	1	1
[K].kVEGTAFVIFGIQDGEQR.[I]	N-Term(iTRAQ4plex); K1(iTRAQ4	1	1
[K].kVEGTAFVIFGIQDGEQR.[I]	N-Term(iTRAQ4plex); K1(iTRAQ4	1	1
[R].iSLPESLk.[R]	N-Term(iTRAQ4plex); K8(iTRAQ4	1	1
[R].qPSSAFAAFVk.[R]	N-Term(iTRAQ4plex); K11(iTRAQ4	1	1
[K].fYYIYNEk.[G]	N-Term(iTRAQ4plex); K8(iTRAQ4	1	1
[K].tIYTPGSTVLYR.[I]	N-Term(iTRAQ4plex)	1	1
[K].kVEGTAFVIFGIQDGEQR.[I]	N-Term(iTRAQ4plex); K1(iTRAQ4	1	1
[K].fVTVQATFGTQVVEk.[V]	N-Term(iTRAQ4plex); K15(iTRAQ4	1	1
[K].dYAGVFSDAGLTFTSSSGQQTAQR.[A]	N-Term(iTRAQ4plex)	1	1
[K].dYAGVFSDAGLTFTSSSGQQTAQR.[A]	N-Term(iTRAQ4plex)	1	1
[K].kVEGTAFVIFGIQDGEQR.[I]	N-Term(iTRAQ4plex); K1(iTRAQ4	1	1
[K].dYAGVFSDAGLTFTSSSGQQTAQR.[A]	N-Term(iTRAQ4plex)	1	1
[K].kVEGTAFVIFGIQDGEQR.[I]	N-Term(iTRAQ4plex); K1(iTRAQ4	1	1
[K].dYAGVFSDAGLTFTSSSGQQTAQR.[A]	N-Term(iTRAQ4plex)	1	1
[R].yYTYLImNk.[G]	N-Term(iTRAQ4plex); M7(Oxidati	1	1
[K].dYAGVFSDAGLTFTSSSGQQTAQR.[A]	N-Term(iTRAQ4plex)	1	1
[K].kVEGTAFVIFGIQDGEQR.[I]	N-Term(iTRAQ4plex); K1(iTRAQ4	1	1
[R].YYTYLIMNk.[G]	K9(iTRAQ4plex)	1	1
[K].dYAGVFSDAGLTFTSSSGQQTAQR.[A]	N-Term(iTRAQ4plex)	1	1
[K].kVEGTAFVIFGIQDGEQR.[I]	N-Term(iTRAQ4plex); K1(iTRAQ4	1	1
[K].kVEGTAFVIFGIQDGEQR.[I]	N-Term(iTRAQ4plex); K1(iTRAQ4	1	1
[R].VELLHNPAFcSLATTk.[R]	C10(Carbamidomethyl); K16(iTR	1	1
[K].dYAGVFSDAGLTFTSSSGQQTAQR.[A]	N-Term(iTRAQ4plex)	1	1
[K].kVEGTAFVIFGIQDGEQR.[I]	N-Term(iTRAQ4plex); K1(iTRAQ4	1	1
[K].vQLSNDFDEYIMAIEQTIk.[S]	N-Term(iTRAQ4plex); K19(iTRAQ4	1	1
[K].nTMILEIcTR.[Y]	N-Term(iTRAQ4plex); C8(Carbam	1	1
[R].gDQDATMSILDISMMTGFAPDTDDLk.[Q]	N-Term(iTRAQ4plex); K26(iTRAQ4	1	1

[K].vQLSNDFDEYImAIEQTIk.[S]	N-Term(iTRAQ4plex); M12(Oxida	1	1
[K].qcQDLGAFTESMVVFGcPN.[-]	N-Term(iTRAQ4plex); C2(Carbam	1	1
[K].qcQDLGAFTESMVVFGcPN.[-]	N-Term(iTRAQ4plex); C2(Carbam	1	1
[K].vQLSNDFDEYIMAIEQTIk.[S]	N-Term(iTRAQ4plex); K19(iTRAQ4	1	1
[K].tIYTPGSTVLYR.[I]	N-Term(iTRAQ4plex)	1	1
[K].tIYTPGSTVLYR.[I]	N-Term(iTRAQ4plex)	1	1
[K].tIYTPGSTVLYR.[I]	N-Term(iTRAQ4plex)	1	1
[K].fYYIYNEk.[G]	N-Term(iTRAQ4plex); K8(iTRAQ4	1	1
[K].nTMILEIcTR.[Y]	N-Term(iTRAQ4plex); C8(Carbam	1	1
[K].nTMILEIcTR.[Y]	N-Term(iTRAQ4plex); C8(Carbam	1	1
[K].nTMILEIcTR.[Y]	N-Term(iTRAQ4plex); C8(Carbam	1	1
[K].nTMILEIcTR.[Y]	N-Term(iTRAQ4plex); C8(Carbam	1	1
[K].qkPDGVFQEDAPVIHQEmIGGLR.[N]	N-Term(iTRAQ4plex); K2(iTRAQ4	1	1
[K].qkPDGVFQEDAPVIHQEMIGGLR.[N]	N-Term(iTRAQ4plex); K2(iTRAQ4	1	1
[R].yRGDQDATMSILDISMMTGFAPDTDDLk.	N-Term(iTRAQ4plex); K28(iTRAQ4	1	1
[K].dSITTWEILAVSMSDk.[K]	N-Term(iTRAQ4plex); K16(iTRAQ4	1	1
[K].qkPDGVFQEDAPVIHQEMIGGLR.[N]	N-Term(iTRAQ4plex); K2(iTRAQ4	1	1
[K].qkPDGVFQEDAPVIHQEMIGGLR.[N]	N-Term(iTRAQ4plex); K2(iTRAQ4	1	1
[K].qkPDGVFQEDAPVIHQEMIGGLR.[N]	N-Term(iTRAQ4plex); K2(iTRAQ4	1	1
[R].NTLIIYLDk.[V]	K9(iTRAQ4plex)	1	1
[R].fYHPEkEDGk.[L]	N-Term(iTRAQ4plex); K6(iTRAQ4	1	1
[K].aGDFLEANYmNLQR.[S]	N-Term(iTRAQ4plex); M10(Oxida	1	1
[K].aGDFLEANYmNLQR.[S]	N-Term(iTRAQ4plex); M10(Oxida	1	1
[R].tKkQELSEAEQATR.[T]	N-Term(iTRAQ4plex); K3(iTRAQ4	1	1
[R].tkKQELSEAEQATR.[T]	N-Term(iTRAQ4plex); K2(iTRAQ4	1	1
[K].gQGTLSVVTMYHAk.[A]	N-Term(iTRAQ4plex); K14(iTRAQ4	1	1
[R].tKkQELSEAEQATR.[T]	N-Term(iTRAQ4plex); K3(iTRAQ4	1	1
[R].TkkQELSEAEQATR.[T]	K2(iTRAQ4plex); K3(iTRAQ4plex)	1	1
[K].gQGTLSVVTMYHAk.[A]	N-Term(iTRAQ4plex); K14(iTRAQ4	1	1
[R].TkkQELSEAEQATR.[T]	K2(iTRAQ4plex); K3(iTRAQ4plex)	1	1
[K].gQGTLSVVTMYHAk.[A]	N-Term(iTRAQ4plex); K14(iTRAQ4	1	1
[K].gQGTLSVVTMYHAk.[A]	N-Term(iTRAQ4plex); K14(iTRAQ4	1	1
[R].TkkQELSEAEQATR.[T]	K2(iTRAQ4plex); K3(iTRAQ4plex)	1	1
[R].fISLGEAck.[K]	N-Term(iTRAQ4plex); C8(Carbam	1	1
[R].tkKQELSEAEQATR.[T]	N-Term(iTRAQ4plex); K2(iTRAQ4	1	1
[R].tkkQELSEAEQATR.[T]	N-Term(iTRAQ4plex); K2(iTRAQ4	1	1
[R].FISLGEAck.[K]	C8(Carbamidomethyl); K9(iTRAQ	1	1
[K].gYTQQLAFR.[Q]	N-Term(iTRAQ4plex)	1	1
[K].vLLDGVQNPR.[A]	N-Term(iTRAQ4plex)	1	1
[K].vLLDGVQNPR.[A]	N-Term(iTRAQ4plex)	1	1
[K].vLLDGVQNPR.[A]	N-Term(iTRAQ4plex)	1	1
[R].fYHPEkEDGk.[L]	N-Term(iTRAQ4plex); K6(iTRAQ4	1	1
[R].fISLGEAck.[K]	N-Term(iTRAQ4plex); C8(Carbam	1	1
[K].SGSDEVQVGQQR.[T]		1	1
[R].fISLGEAck.[K]	N-Term(iTRAQ4plex); C8(Carbam	1	1
[R].vVLVAVDk.[G]	N-Term(iTRAQ4plex); K8(iTRAQ4	1	1
[R].vVLVAVDk.[G]	N-Term(iTRAQ4plex); K8(iTRAQ4	1	1
[R].vVLVAVDk.[G]	N-Term(iTRAQ4plex); K8(iTRAQ4	1	1
[K].rIPIEDGSGEVVLSR.[K]	N-Term(iTRAQ4plex)	1	1
[R].vVLVAVDk.[G]	N-Term(iTRAQ4plex); K8(iTRAQ4	1	1
[R].vVLVAVDk.[G]	N-Term(iTRAQ4plex); K8(iTRAQ4	1	1
[R].fISLGEAck.[K]	N-Term(iTRAQ4plex); C8(Carbam	1	1

[R].vVLVAVDk.[G]	N-Term(iTRAQ4plex); K8(iTRAQ4	1	1
[R].vVLVAVDk.[G]	N-Term(iTRAQ4plex); K8(iTRAQ4	1	1
[R].vVLVAVDk.[G]	N-Term(iTRAQ4plex); K8(iTRAQ4	1	1
[K].qkPDGVFQEDAPVIHQEMIGGLR.[N]	N-Term(iTRAQ4plex); K2(iTRAQ4	1	1
[K].qkPDGVFQEDAPVIHQEMIGGLR.[N]	N-Term(iTRAQ4plex); K2(iTRAQ4	1	1
[K].qkPDGVFQEDAPVIHQEMIGGLR.[N]	N-Term(iTRAQ4plex); K2(iTRAQ4	1	1
[K].dYAGVFSDAGLTFTSSSGQQTAQR.[A]	N-Term(iTRAQ4plex)	1	1
[K].kVEGTAFVIFGIQDGEQR.[I]	N-Term(iTRAQ4plex); K1(iTRAQ4	1	1
[K].kVEGTAFVIFGIQDGEQR.[I]	N-Term(iTRAQ4plex); K1(iTRAQ4	1	1
[K].kVEGTAFVIFGIQDGEQR.[I]	N-Term(iTRAQ4plex); K1(iTRAQ4	1	1
[K].dYAGVFSDAGLTFTSSSGQQTAQR.[A]	N-Term(iTRAQ4plex)	1	1
[K].kVEGTAFVIFGIQDGEQR.[I]	N-Term(iTRAQ4plex); K1(iTRAQ4	1	1
[K].fVTVQATFGTQVVEk.[V]	N-Term(iTRAQ4plex); K15(iTRAQ	1	1
[K].dSITTWEILAVSmSDk.[K]	N-Term(iTRAQ4plex); M13(Oxida	1	1
[K].dSITTWEILAVSMSDkk.[G]	N-Term(iTRAQ4plex); K16(iTRAQ	1	1
[R].iLLQGTPVAQMTEDAVDAER.[L]	N-Term(iTRAQ4plex)	1	1
[K].dSITTWEILAVSmSDk.[K]	N-Term(iTRAQ4plex); M13(Oxida	1	1
[R].sEFPESWLWNVEDLkEPPk.[N]	N-Term(iTRAQ4plex); K15(iTRAQ	1	1
[R].sEFPESWLWNVEDLkEPPk.[N]	N-Term(iTRAQ4plex); K15(iTRAQ	1	1
[K].DYAGVFSDAGLTFTSSSGQQTAQR.[A]		1	1
[R].sEFPESWLWNVEDLkEPPk.[N]	N-Term(iTRAQ4plex); K15(iTRAQ	1	1
[K].dYAGVFSDAGLTFTSSSGQQTAQR.[A]	N-Term(iTRAQ4plex)	1	1
[K].kVEGTAFVIFGIQDGEQR.[I]	N-Term(iTRAQ4plex); K1(iTRAQ4	1	1
[K].kVEGTAFVIFGIQDGEQR.[I]	N-Term(iTRAQ4plex); K1(iTRAQ4	1	1
[R].iLLQGTPVAQMTEDAVDAER.[L]	N-Term(iTRAQ4plex)	1	1
[R].iLLQGTPVAQMTEDAVDAER.[L]	N-Term(iTRAQ4plex)	1	1
[R].iLLQGTPVAQMTEDAVDAER.[L]	N-Term(iTRAQ4plex)	1	1
[K].kVEGTAFVIFGIQDGEQR.[I]	N-Term(iTRAQ4plex); K1(iTRAQ4	1	1
[K].kVEGTAFVIFGIQDGEQR.[I]	N-Term(iTRAQ4plex); K1(iTRAQ4	1	1
[K].vEGTAFVIFGIQDGEQR.[I]	N-Term(iTRAQ4plex)	1	1
[K].eYVLPSFEVIVEPTEk.[F]	N-Term(iTRAQ4plex); K16(iTRAQ	1	1
[K].fVTVQATFGTQVVEk.[V]	N-Term(iTRAQ4plex); K15(iTRAQ	1	1
[R].iLLQGTPVAQMTEDAVDAER.[L]	N-Term(iTRAQ4plex)	1	1
[R].iLLQGTPVAQMTEDAVDAER.[L]	N-Term(iTRAQ4plex)	1	1
[R].iLLQGTPVAQMTEDAVDAER.[L]	N-Term(iTRAQ4plex)	1	1
[R].iLLQGTPVAQmTEDAVDAER.[L]	N-Term(iTRAQ4plex); M11(Oxida	1	1
[R].iLLQGTPVAQmTEDAVDAER.[L]	N-Term(iTRAQ4plex); M11(Oxida	1	1
[R].nTLIIYLDk.[V]	N-Term(iTRAQ4plex); K9(iTRAQ4	1	1
[K].qkPDGVFQEDAPVIHQEMIGGLR.[N]	N-Term(iTRAQ4plex); K2(iTRAQ4	1	1
[K].qkPDGVFQEDAPVIHQEMIGGLR.[N]	N-Term(iTRAQ4plex); K2(iTRAQ4	1	1
[K].qkPDGVFQEDAPVIHQEMIGGLR.[N]	N-Term(iTRAQ4plex); K2(iTRAQ4	1	1
[R].nTLIIYLDk.[V]	N-Term(iTRAQ4plex); K9(iTRAQ4	1	1
[R].nTLIIYLDk.[V]	N-Term(iTRAQ4plex); K9(iTRAQ4	1	1
[R].nTLIIYLDk.[V]	N-Term(iTRAQ4plex); K9(iTRAQ4	1	1
[K].dYAGVFSDAGLTFTSSSGQQTAQR.[A]	N-Term(iTRAQ4plex)	1	1
[K].dFDFVPPVVR.[W]	N-Term(iTRAQ4plex)	1	1
[K].aGDFLEANYMNLQR.[S]	N-Term(iTRAQ4plex)	1	1
[K].VFLDccNYITELR.[R]	C5(Carbamidomethyl); C6(Carba	1	1
[K].dYAGVFSDAGLTFTSSSGQQTAQR.[A]	N-Term(iTRAQ4plex)	1	1
[K].kVEGTAFVIFGIQDGEQR.[I]	N-Term(iTRAQ4plex); K1(iTRAQ4	1	1
[K].aGDFLEANYMNLQR.[S]	N-Term(iTRAQ4plex)	1	1
[K].aGDFLEANYMNLQR.[S]	N-Term(iTRAQ4plex)	1	1

[K].kVEGTAFVIFGIQDGEQR.[I]	N-Term(iTRAQ4plex); K1(iTRAQ4	1	1
[K].kQELSEAEQATR.[T]	N-Term(iTRAQ4plex); K1(iTRAQ4	1	1
[K].sLYVSATVILHSGSDMVQAER.[S]	N-Term(iTRAQ4plex)	1	1
[K].kQELSEAEQATR.[T]	N-Term(iTRAQ4plex); K1(iTRAQ4	1	1
[R].tELRPGETLNVNFLLR.[M]	N-Term(iTRAQ4plex)	1	1
[R].eGVQkEDIPPADLSDQVPDTESETR.[I]	N-Term(iTRAQ4plex); K5(iTRAQ4	1	1
[K].kQELSEAEQATR.[T]	N-Term(iTRAQ4plex); K1(iTRAQ4	1	1
[R].aPSTWLTAYVVk.[V]	N-Term(iTRAQ4plex); K12(iTRAQ4	1	1
[R].eGVQkEDIPPADLSDQVPDTESETR.[I]	N-Term(iTRAQ4plex); K5(iTRAQ4	1	1
[R].eGVQkEDIPPADLSDQVPDTESETR.[I]	N-Term(iTRAQ4plex); K5(iTRAQ4	1	1
[R].eGVQkEDIPPADLSDQVPDTESETR.[I]	N-Term(iTRAQ4plex); K5(iTRAQ4	1	1
[R].eGVQkEDIPPADLSDQVPDTESETR.[I]	N-Term(iTRAQ4plex); K5(iTRAQ4	1	1
[K].kQELSEAEQATR.[T]	N-Term(iTRAQ4plex); K1(iTRAQ4	1	1
[R].lVAYYTLIGASGQR.[E]	N-Term(iTRAQ4plex)	1	1
[R].lVAYYTLIGASGQR.[E]	N-Term(iTRAQ4plex)	1	1
[R].lVAYYTLIGASGQR.[E]	N-Term(iTRAQ4plex)	1	1
[K].dTWVEHWPEEDEcQDEENQk.[Q]	N-Term(iTRAQ4plex); C13(Carbar	1	1
[R].eGVQkEDIPPADLSDQVPDTESETR.[I]	N-Term(iTRAQ4plex); K5(iTRAQ4	1	1
[R].lVAYYTLIGASGQR.[E]	N-Term(iTRAQ4plex)	1	1
[K].dTWVEHWPEEDEcQDEENQk.[Q]	N-Term(iTRAQ4plex); C13(Carbar	1	1
[K].dTWVEHWPEEDEcQDEENQk.[Q]	N-Term(iTRAQ4plex); C13(Carbar	1	1
[R].aPSTWLTAYVVk.[V]	N-Term(iTRAQ4plex); K12(iTRAQ4	1	1
[R].eGVQkEDIPPADLSDQVPDTESETR.[I]	N-Term(iTRAQ4plex); K5(iTRAQ4	1	1
[K].yFkPGMPFDLMVFVTNPDGSPAYR.[V]	N-Term(iTRAQ4plex); K3(iTRAQ4	1	1
[R].lVAYYTLIGASGQR.[E]	N-Term(iTRAQ4plex)	1	1
[R].eGVQkEDIPPADLSDQVPDTESETR.[I]	N-Term(iTRAQ4plex); K5(iTRAQ4	1	1
[K].kQELSEAEQATR.[T]	N-Term(iTRAQ4plex); K1(iTRAQ4	1	1
[K].kQELSEAEQATR.[T]	N-Term(iTRAQ4plex); K1(iTRAQ4	1	1
[R].eGVQkEDIPPADLSDQVPDTESETR.[I]	N-Term(iTRAQ4plex); K5(iTRAQ4	1	1
[K].kQELSEAEQATR.[T]	N-Term(iTRAQ4plex); K1(iTRAQ4	1	1
[R].eGVQkEDIPPADLSDQVPDTESETR.[I]	N-Term(iTRAQ4plex); K5(iTRAQ4	1	1
[K].kQELSEAEQATR.[T]	N-Term(iTRAQ4plex); K1(iTRAQ4	1	1
[K].kQELSEAEQATR.[T]	N-Term(iTRAQ4plex); K1(iTRAQ4	1	1
[R].eGVQkEDIPPADLSDQVPDTESETR.[I]	N-Term(iTRAQ4plex); K5(iTRAQ4	1	1
[R].IPIEDGSGEVVLSR.[K]		1	1
[K].kQELSEAEQATR.[T]	N-Term(iTRAQ4plex); K1(iTRAQ4	1	1
[R].eGVQkEDIPPADLSDQVPDTESETR.[I]	N-Term(iTRAQ4plex); K5(iTRAQ4	1	1
[R].eGVQkEDIPPADLSDQVPDTESETR.[I]	N-Term(iTRAQ4plex); K5(iTRAQ4	1	1
[R].eGVQkEDIPPADLSDQVPDTESETR.[I]	N-Term(iTRAQ4plex); K5(iTRAQ4	1	1
[R].eGVQkEDIPPADLSDQVPDTESETR.[I]	N-Term(iTRAQ4plex); K5(iTRAQ4	1	1
[K].kQELSEAEQATR.[T]	N-Term(iTRAQ4plex); K1(iTRAQ4	1	1
[K].kQELSEAEQATR.[T]	N-Term(iTRAQ4plex); K1(iTRAQ4	1	1
[R].eGVQkEDIPPADLSDQVPDTESETR.[I]	N-Term(iTRAQ4plex); K5(iTRAQ4	1	1
[K].kQELSEAEQATR.[T]	N-Term(iTRAQ4plex); K1(iTRAQ4	1	1
[K].kQELSEAEQATR.[T]	N-Term(iTRAQ4plex); K1(iTRAQ4	1	1
[K].kHYLMWGLSSDFWGEkPNLSYIIGk.[D]	K1(iTRAQ4plex); K16(iTRAQ4plex	1	1
[K].sLYVSATVILHSGSDMVQAER.[S]	N-Term(iTRAQ4plex)	1	1
[K].sLYVSATVILHSGSDMVQAER.[S]	N-Term(iTRAQ4plex)	1	1
[K].kHYLMWGLSSDFWGEkPNLSYIIGk.[D]	N-Term(iTRAQ4plex); K16(iTRAQ4	1	1
[K].sLYVSATVILHSGSDMVQAER.[S]	N-Term(iTRAQ4plex)	1	1
[K].acEPGVDYVYk.[T]	N-Term(iTRAQ4plex); C2(Carbam	1	1
[K].acEPGVDYVYk.[T]	N-Term(iTRAQ4plex); C2(Carbam	1	1

[K].acEPGVDYVYk.[T]	N-Term(iTRAQ4plex); C2(Carbam	1	1
[K].acEPGVDYVYk.[T]	N-Term(iTRAQ4plex); C2(Carbam	1	1
[K].sLYVSATVILHSGSDMVQAER.[S]	N-Term(iTRAQ4plex)	1	1
[K].fVTVQATFGTQVVEk.[V]	N-Term(iTRAQ4plex); K15(iTRAQ	1	1
[K].sLYVSATVILHSGSDMVQAER.[S]	N-Term(iTRAQ4plex)	1	1
[R].tELRPGETLNVNFLLR.[M]	N-Term(iTRAQ4plex)	1	1
[K].sLYVSATVILHSGSDMVQAER.[S]	N-Term(iTRAQ4plex)	1	1
[K].kHYLMWGLSSDFWGEkPNLSYIIGk.[D]	N-Term(iTRAQ4plex); K1(iTRAQ4	1	1
[K].kVEGTAFVIFGIQDGEQR.[I]	N-Term(iTRAQ4plex); K1(iTRAQ4	1	1
[K].fVTVQATFGTQVVEk.[V]	N-Term(iTRAQ4plex); K15(iTRAQ	1	1
[R].tMQALPYSTVGNSNNYLHLSVLR.[T]	N-Term(iTRAQ4plex)	1	1
[K].sLYVSATVILHSGSDMVQAER.[S]	N-Term(iTRAQ4plex)	1	1
[R].tMQALPYSTVGNSNNYLHLSVLR.[T]	N-Term(iTRAQ4plex)	1	1
[K].sLYVSATVILHSGSDMVQAER.[S]	N-Term(iTRAQ4plex)	1	1
[R].tELRPGETLNVNFLLR.[M]	N-Term(iTRAQ4plex)	1	1
[K].kVEGTAFVIFGIQDGEQR.[I]	N-Term(iTRAQ4plex); K1(iTRAQ4	1	1
[K].akDQLTcNk.[F]	N-Term(iTRAQ4plex); K2(iTRAQ4	1	1
[K].sGSDEVQVGQQR.[T]	N-Term(iTRAQ4plex)	1	1
[K].fVTVQATFGTQVVEk.[V]	N-Term(iTRAQ4plex); K15(iTRAQ	1	1
[R].tELRPGETLNVNFLLR.[M]	N-Term(iTRAQ4plex)	1	1
[R].rHQQTVTIPPk.[S]	N-Term(iTRAQ4plex); K11(iTRAQ	1	1
[R].rHQQTVTIPPk.[S]	N-Term(iTRAQ4plex); K11(iTRAQ	1	1
[R].TELRPGETLNVNFLLR.[M]		1	1
[K].aGDFLEANYMNLQR.[S]	N-Term(iTRAQ4plex)	1	1
[R].rHQQTVTIPPk.[S]	N-Term(iTRAQ4plex); K11(iTRAQ	1	1
[K].fVTVQATFGTQVVEk.[V]	N-Term(iTRAQ4plex); K15(iTRAQ	1	1
[R].hQQTVTIPPk.[S]	N-Term(iTRAQ4plex); K10(iTRAQ	1	1
[K].vVLVSLQSGYLFIQTDk.[T]	N-Term(iTRAQ4plex); K17(iTRAQ	1	1
[K].kVEGTAFVIFGIQDGEQR.[I]	N-Term(iTRAQ4plex); K1(iTRAQ4	1	1
[R].hQQTVTIPPk.[S]	N-Term(iTRAQ4plex); K10(iTRAQ	1	1
[R].hQQTVTIPPk.[S]	N-Term(iTRAQ4plex); K10(iTRAQ	1	1
[K].vVLVSLQSGYLFIQTDk.[T]	N-Term(iTRAQ4plex); K17(iTRAQ	1	1
[R].rHQQTVTIPPk.[S]	N-Term(iTRAQ4plex); K11(iTRAQ	1	1
[R].rHQQTVTIPPk.[S]	N-Term(iTRAQ4plex); K11(iTRAQ	1	1
[R].rHQQTVTIPPk.[S]	N-Term(iTRAQ4plex); K11(iTRAQ	1	1
[K].DIcEEQVNSLPGSITk.[A]	C3(Carbamidomethyl); K16(iTRA	1	1
[K].eDIPPADLSDQVPDTESETR.[I]	N-Term(iTRAQ4plex)	1	1
[K].eDIPPADLSDQVPDTESETR.[I]	N-Term(iTRAQ4plex)	1	1
[R].sYTVAIAGYALAQMGR.[L]	N-Term(iTRAQ4plex)	1	1
[R].sYTVAIAGYALAQMGR.[L]	N-Term(iTRAQ4plex)	1	1
[R].tELRPGETLNVNFLLR.[M]	N-Term(iTRAQ4plex)	1	1
[K].hYLMWGLSSDFWGEkPNLSYIIGk.[D]	N-Term(iTRAQ4plex); K15(iTRAQ	1	1
[R].tELRPGETLNVNFLLR.[M]	N-Term(iTRAQ4plex)	1	1
[R].TELRPGETLNVNFLLR.[M]		1	1
[K].ADIGcTPGSGk.[D]	C5(Carbamidomethyl); K11(iTRA	1	1
[R].tELRPGETLNVNFLLR.[M]	N-Term(iTRAQ4plex)	1	1
[R].tELRPGETLNVNFLLR.[M]	N-Term(iTRAQ4plex)	1	1
[R].TELRPGETLNVNFLLR.[M]		1	1
[K].kQELSEAEQATR.[T]	K1(iTRAQ4plex)	1	1
[K].kQELSEAEQATR.[T]	N-Term(iTRAQ4plex)	1	1
[K].yFkPGMPFDLmVFVTNPDGSPAYR.[V]	N-Term(iTRAQ4plex); K3(iTRAQ4	1	1
[K].hYLMWGLSSDFWGEkPNLSYIIGk.[D]	N-Term(iTRAQ4plex); K15(iTRAQ	1	1

[R].tELRPGETLNVNFLLR.[M]	N-Term(iTRAQ4plex)	1	1
[K].fVTVQATFGTQVVEk.[V]	N-Term(iTRAQ4plex); K15(iTRAQ	1	1
[K].kVEGTAFVIFGIQDGEQR.[I]	N-Term(iTRAQ4plex); K1(iTRAQ4	1	1
[R].sNLDEDIIAEENIVSR.[S]	N-Term(iTRAQ4plex)	1	1
[R].sNLDEDIIAEENIVSR.[S]	N-Term(iTRAQ4plex)	1	1
[R].SNLDEDIIAEENIVSR.[S]		1	1
[R].SNLDEDIIAEENIVSR.[S]		1	1
[K].eDIPPADLSDQVPDTESETR.[I]	N-Term(iTRAQ4plex)	1	1
[K].eDIPPADLSDQVPDTESETR.[I]	N-Term(iTRAQ4plex)	1	1
[K].eDIPPADLSDQVPDTESETR.[I]	N-Term(iTRAQ4plex)	1	1
[K].eDIPPADLSDQVPDTESETR.[I]	N-Term(iTRAQ4plex)	1	1
[R].tkkQELSEAEQATR.[T]	N-Term(iTRAQ4plex); K2(iTRAQ4	1	1
[K].eDIPPADLSDQVPDTESETR.[I]	N-Term(iTRAQ4plex)	1	1
[K].sSLSVPYVIVPLk.[T]	N-Term(iTRAQ4plex); K13(iTRAQ	1	1
[K].eDIPPADLSDQVPDTESETR.[I]	N-Term(iTRAQ4plex)	1	1
[K].eDIPPADLSDQVPDTESETR.[I]	N-Term(iTRAQ4plex)	1	1
[K].sLYVSATVILHSGSDMVQAER.[S]	N-Term(iTRAQ4plex)	1	1
[K].eDIPPADLSDQVPDTESETR.[I]	N-Term(iTRAQ4plex)	1	1
[K].eDIPPADLSDQVPDTESETR.[I]	N-Term(iTRAQ4plex)	1	1
[K].EDIPPADLSDQVPDTESETR.[I]		1	1
[K].eDIPPADLSDQVPDTESETR.[I]	N-Term(iTRAQ4plex)	1	1
[K].DTWVEHWPEEDEcQDEENQk.[Q]	C13(Carbamidomethyl); K20(iTR	1	1
[K].eDIPPADLSDQVPDTESETR.[I]	N-Term(iTRAQ4plex)	1	1
[K].dTWVEHWPEEDEcQDEENQk.[Q]	N-Term(iTRAQ4plex); C13(Carbai	1	1
[K].dTWVEHWPEEDEcQDEENQk.[Q]	N-Term(iTRAQ4plex); C13(Carbai	1	1
[K].eDIPPADLSDQVPDTESETR.[I]	N-Term(iTRAQ4plex)	1	1
[K].DTWVEHWPEEDEcQDEENQk.[Q]	C13(Carbamidomethyl); K20(iTR	1	1
[K].dTWVEHWPEEDEcQDEENQk.[Q]	N-Term(iTRAQ4plex); C13(Carbai	1	1
[K].eDIPPADLSDQVPDTESETR.[I]	N-Term(iTRAQ4plex)	1	1
[K].dTWVEHWPEEDEcQDEENQk.[Q]	N-Term(iTRAQ4plex); C13(Carbai	1	1
[K].eDIPPADLSDQVPDTESETR.[I]	N-Term(iTRAQ4plex)	1	1
[K].eDIPPADLSDQVPDTESETR.[I]	N-Term(iTRAQ4plex)	1	1
[K].EDIPPADLSDQVPDTESETR.[I]		1	1
[K].eDIPPADLSDQVPDTESETR.[I]	N-Term(iTRAQ4plex)	1	1
[K].eDIPPADLSDQVPDTESETR.[I]	N-Term(iTRAQ4plex)	1	1
[K].eDIPPADLSDQVPDTESETR.[I]	N-Term(iTRAQ4plex)	1	1
[K].VSHSEDDcLAFk.[V]	C8(Carbamidomethyl); K12(iTRA	1	1
[K].eDIPPADLSDQVPDTESETR.[I]	N-Term(iTRAQ4plex)	1	1
[K].EDIPPADLSDQVPDTESETR.[I]		1	1
[K].eDIPPADLSDQVPDTESETR.[I]	N-Term(iTRAQ4plex)	1	1
[K].eDIPPADLSDQVPDTESETR.[I]	N-Term(iTRAQ4plex)	1	1
[K].EDIPPADLSDQVPDTESETR.[I]		1	1
[K].eDIPPADLSDQVPDTESETR.[I]	N-Term(iTRAQ4plex)	1	1
[K].EDIPPADLSDQVPDTESETR.[I]		1	1
[K].eDIPPADLSDQVPDTESETR.[I]	N-Term(iTRAQ4plex)	1	1
[K].VSHSEDDcLAFk.[V]	C8(Carbamidomethyl); K12(iTRA	1	1
[K].EDIPPADLSDQVPDTESETR.[I]		1	1
[K].eDIPPADLSDQVPDTESETR.[I]	N-Term(iTRAQ4plex)	1	1
[K].EDIPPADLSDQVPDTESETR.[I]		1	1
[K].eDIPPADLSDQVPDTESETR.[I]	N-Term(iTRAQ4plex)	1	1
[K].dTWVEHWPEEDEcQDEENQk.[Q]	N-Term(iTRAQ4plex); C13(Carbai	1	1
[K].VSHSEDDcLAFk.[V]	C8(Carbamidomethyl); K12(iTRA	1	1

[K].VTIkPAPETEk.[R]	K4(iTRAQ4plex); K11(iTRAQ4plex	1	1
[K].aDIGcTPGSGk.[D]	N-Term(iTRAQ4plex); C5(Carbam	1	1
[R].fYHPEkEDGk.[L]	N-Term(iTRAQ4plex); K6(iTRAQ4	1	1
[K].aDIGcTPGSGk.[D]	N-Term(iTRAQ4plex); C5(Carbam	1	1
[R].sYTVAIAGYALAQMGR.[L]	N-Term(iTRAQ4plex)	1	1
[R].fYHPEkEDGk.[L]	N-Term(iTRAQ4plex); K6(iTRAQ4	1	1
[K].sLYVSATVILHSGSDMVQAER.[S]	N-Term(iTRAQ4plex)	1	1
[R].sYTVAIAGYALAQMGR.[L]	N-Term(iTRAQ4plex)	1	1
[R].fYHPEkEDGk.[L]	N-Term(iTRAQ4plex); K6(iTRAQ4	1	1
[K].aDIGcTPGSGk.[D]	N-Term(iTRAQ4plex); C5(Carbam	1	1
[K].aDIGcTPGSGk.[D]	N-Term(iTRAQ4plex); C5(Carbam	1	1
[K].sSLSVPYVIVPLk.[T]	N-Term(iTRAQ4plex); K13(iTRAQ	1	1
[K].akDQLTcNk.[F]	N-Term(iTRAQ4plex); K2(iTRAQ4	1	1
[R].aELQcPQPAAR.[R]	N-Term(iTRAQ4plex); C5(Carbam	1	1
[R].aELQcPQPAAR.[R]	N-Term(iTRAQ4plex); C5(Carbam	1	1
[R].sYTVAIAGYALAQMGR.[L]	N-Term(iTRAQ4plex)	1	1
[R].sYTVAIAGYALAQMGR.[L]	N-Term(iTRAQ4plex)	1	1
[R].sYTVAIAGYALAQMGR.[L]	N-Term(iTRAQ4plex)	1	1
[K].sSLSVPYVIVPLk.[T]	N-Term(iTRAQ4plex); K13(iTRAQ	1	1
[K].VTIkPAPETEk.[R]	K4(iTRAQ4plex); K11(iTRAQ4plex	1	1
[K].DTWVEHWPEEDEcQDEENQk.[Q]	C13(Carbamidomethyl); K20(iTR	1	1
[K].eNEGFTVTAEGk.[G]	N-Term(iTRAQ4plex); K12(iTRAQ	1	1
[K].eNEGFTVTAEGk.[G]	N-Term(iTRAQ4plex); K12(iTRAQ	1	1
[K].eNEGFTVTAEGk.[G]	N-Term(iTRAQ4plex); K12(iTRAQ	1	1
[R].cAEENcFIQk.[S]	N-Term(iTRAQ4plex); C1(Carbam	1	1
[R].cAEENcFIQk.[S]	N-Term(iTRAQ4plex); C1(Carbam	1	1
[K].eNEGFTVTAEGk.[G]	N-Term(iTRAQ4plex); K12(iTRAQ	1	1
[K].eNEGFTVTAEGk.[G]	N-Term(iTRAQ4plex); K12(iTRAQ	1	1
[K].sLYVSATVILHSGSDMVQAER.[S]	N-Term(iTRAQ4plex)	1	1
[K].eNEGFTVTAEGk.[G]	N-Term(iTRAQ4plex); K12(iTRAQ	1	1
[K].eNEGFTVTAEGk.[G]	N-Term(iTRAQ4plex); K12(iTRAQ	1	1
[K].tGLQEVEVk.[A]	N-Term(iTRAQ4plex); K9(iTRAQ4	1	1
[K].tGLQEVEVk.[A]	N-Term(iTRAQ4plex); K9(iTRAQ4	1	1
[K].vSHSEDDcLAFk.[V]	N-Term(iTRAQ4plex); C8(Carbam	1	1
[K].vSHSEDDcLAFk.[V]	N-Term(iTRAQ4plex); C8(Carbam	1	1
[K].vSHSEDDcLAFk.[V]	N-Term(iTRAQ4plex); C8(Carbam	1	1
[K].vSHSEDDcLAFk.[V]	N-Term(iTRAQ4plex); C8(Carbam	1	1
[K].eNEGFTVTAEGk.[G]	N-Term(iTRAQ4plex); K12(iTRAQ	1	1
[K].eNEGFTVTAEGk.[G]	N-Term(iTRAQ4plex); K12(iTRAQ	1	1
[R].cAEENcFIQk.[S]	N-Term(iTRAQ4plex); C1(Carbam	1	1
[K].tGLQEVEVk.[A]	N-Term(iTRAQ4plex); K9(iTRAQ4	1	1
[K].tGLQEVEVk.[A]	N-Term(iTRAQ4plex); K9(iTRAQ4	1	1
[K].dYAGVFSDAGLTFTSSSGQQTAQR.[A]	N-Term(iTRAQ4plex)	1	1
[K].sLYVSATVILHSGSDMVQAER.[S]	N-Term(iTRAQ4plex)	1	1
[R].sEETkENEGFTVTAEGk.[G]	N-Term(iTRAQ4plex); K5(iTRAQ4	1	1
[R].sEETkENEGFTVTAEGk.[G]	N-Term(iTRAQ4plex); K5(iTRAQ4	1	1
[K].sLYVSATVILHSGSDMVQAER.[S]	N-Term(iTRAQ4plex)	1	1
[K].ENEGFTVTAEGk.[G]	K12(iTRAQ4plex)	1	1
[K].eNEGFTVTAEGk.[G]	N-Term(iTRAQ4plex); K12(iTRAQ	1	1
[K].eNEGFTVTAEGk.[G]	N-Term(iTRAQ4plex); K12(iTRAQ	1	1
[K].fVTVQATFGTQVVEk.[V]	N-Term(iTRAQ4plex); K15(iTRAQ	1	1
[K].vSHSEDDcLAFk.[V]	N-Term(iTRAQ4plex); C8(Carbam	1	1

[K].dTWVEHWPEEDEcQDEENQk.[Q]	N-Term(iTRAQ4plex); C13(Carbar	1	1
[K].dTWVEHWPEEDEcQDEENQk.[Q]	N-Term(iTRAQ4plex); C13(Carbar	1	1
[K].dTWVEHWPEEDEcQDEENQk.[Q]	N-Term(iTRAQ4plex); C13(Carbar	1	1
[K].dTWVEHWPEEDEcQDEENQk.[Q]	N-Term(iTRAQ4plex); C13(Carbar	1	1
[K].eDIPPADLSDQVPDTESETR.[I]	N-Term(iTRAQ4plex)	1	1
[K].dTWVEHWPEEDEcQDEENQk.[Q]	N-Term(iTRAQ4plex); C13(Carbar	1	1
[K].eDIPPADLSDQVPDTESETR.[I]	N-Term(iTRAQ4plex)	1	1
[K].eDIPPADLSDQVPDTESETR.[I]	N-Term(iTRAQ4plex)	1	1
[K].vSHSEDDcLAFk.[V]	N-Term(iTRAQ4plex); C8(Carbam	1	1
[K].AcEPGVDYVYk.[T]	C2(Carbamidomethyl); K11(iTRA	1	1
[K].AcEPGVDYVYk.[T]	C2(Carbamidomethyl); K11(iTRA	1	1
[K].vSHSEDDcLAFk.[V]	N-Term(iTRAQ4plex); C8(Carbam	1	1
[K].vSHSEDDcLAFk.[V]	N-Term(iTRAQ4plex); C8(Carbam	1	1
[R].cAEENcFIQk.[S]	N-Term(iTRAQ4plex); C1(Carbam	1	1
[K].vSHSEDDcLAFk.[V]	N-Term(iTRAQ4plex); C8(Carbam	1	1
[K].vSHSEDDcLAFk.[V]	N-Term(iTRAQ4plex); C8(Carbam	1	1
[K].sLYVSATVILHSGSDMVQAER.[S]	N-Term(iTRAQ4plex)	1	1
[K].sDDkVTLEER.[L]	N-Term(iTRAQ4plex); K4(iTRAQ4	1	1
[K].VHQYFNVELIQPGAVk.[V]	K16(iTRAQ4plex)	1	1
[R].vELLHNPAFcSLATTk.[R]	N-Term(iTRAQ4plex); C10(Carbar	1	1
[K].qLYNVEATSYALLALLQLkDFDFVPPVVR.[\	N-Term(iTRAQ4plex); K19(iTRAQ	1	1
[K].sDDkVTLEER.[L]	N-Term(iTRAQ4plex); K4(iTRAQ4	1	1
[K].VHQYFNVELIQPGAVk.[V]	K16(iTRAQ4plex)	1	1
[K].sDDkVTLEER.[L]	N-Term(iTRAQ4plex); K4(iTRAQ4	1	1
[K].sDDkVTLEER.[L]	N-Term(iTRAQ4plex); K4(iTRAQ4	1	1
[K].sDDkVTLEER.[L]	N-Term(iTRAQ4plex); K4(iTRAQ4	1	1
[R].vELLHNPAFcSLATTk.[R]	N-Term(iTRAQ4plex); C10(Carbar	1	1
[K].dScVGSLVVk.[S]	N-Term(iTRAQ4plex); C3(Carbam	1	1
[K].dScVGSLVVk.[S]	N-Term(iTRAQ4plex); C3(Carbam	1	1
[K].kQELSEAEQATR.[T]	K1(iTRAQ4plex)	1	1
[K].kQELSEAEQATR.[T]	N-Term(iTRAQ4plex)	1	1
[K].kQELSEAEQATR.[T]	K1(iTRAQ4plex)	1	1
[K].kQELSEAEQATR.[T]	N-Term(iTRAQ4plex)	1	1
[K].kQELSEAEQATR.[T]	K1(iTRAQ4plex)	1	1
[K].gLEVTITAR.[F]	N-Term(iTRAQ4plex)	1	1
[K].VHQYFNVELIQPGAVk.[V]	K16(iTRAQ4plex)	1	1
[K].VHQYFNVELIQPGAVk.[V]	K16(iTRAQ4plex)	1	1
[K].kQELSEAEQATR.[T]	N-Term(iTRAQ4plex)	1	1
[K].kQELSEAEQATR.[T]	N-Term(iTRAQ4plex)	1	1
[K].kQELSEAEQATR.[T]	K1(iTRAQ4plex)	1	1
[K].kQELSEAEQATR.[T]	N-Term(iTRAQ4plex)	1	1
[K].kQELSEAEQATR.[T]	K1(iTRAQ4plex)	1	1
[K].kQELSEAEQATR.[T]	N-Term(iTRAQ4plex)	1	1
[K].sGSDEVQVGQQR.[T]	N-Term(iTRAQ4plex)	1	1
[K].sGSDEVQVGQQR.[T]	N-Term(iTRAQ4plex)	1	1
[R].vELLHNPAFcSLATTk.[R]	N-Term(iTRAQ4plex); C10(Carbar	1	1
[R].vELLHNPAFcSLATTk.[R]	N-Term(iTRAQ4plex); C10(Carbar	1	1
[R].vELLHNPAFcSLATTk.[R]	N-Term(iTRAQ4plex); C10(Carbar	1	1
[K].vYAYYNLEEScTR.[F]	N-Term(iTRAQ4plex); C11(Carbar	1	1
[K].vYAYYNLEEScTR.[F]	N-Term(iTRAQ4plex); C11(Carbar	1	1
[R].vELLHNPAFcSLATTk.[R]	N-Term(iTRAQ4plex); C10(Carbar	1	1
[R].vELLHNPAFcSLATTk.[R]	N-Term(iTRAQ4plex); C10(Carbar	1	1

[K].sDDkVTLEER.[L]	N-Term(iTRAQ4plex); K4(iTRAQ4	1	1
[K].sDDkVTLEER.[L]	N-Term(iTRAQ4plex); K4(iTRAQ4	1	1
[R].vELLHNPAFcSLATTk.[R]	N-Term(iTRAQ4plex); C10(Carbar	1	1
[K].sGSDEVQVGQQR.[T]	N-Term(iTRAQ4plex)	1	1
[K].sGSDEVQVGQQR.[T]	N-Term(iTRAQ4plex)	1	1
[K].sGSDEVQVGQQR.[T]	N-Term(iTRAQ4plex)	1	1
[K].kQELSEAEQATR.[T]	N-Term(iTRAQ4plex)	1	1
[K].rIPIEDGSGEVVLSR.[K]	N-Term(iTRAQ4plex)	1	1
[R].sYTVAIAGYALAQMGR.[L]	N-Term(iTRAQ4plex)	1	1
[K].vFSLAVNLIAIDSQVLcGAVk.[W]	N-Term(iTRAQ4plex); C17(Carbar	1	1
[K].VYAYYNLEEScTR.[F]	C11(Carbamidomethyl)	1	1
[K].qDSLSSQNQLGVLPLSWDIPELVNMGQW	N-Term(iTRAQ4plex); K29(iTRAQ	1	1
[K].kVEGTAFVIFGIQDGEQR.[I]	N-Term(iTRAQ4plex); K1(iTRAQ4	1	1
[K].rIPIEDGSGEVVLSR.[K]	N-Term(iTRAQ4plex)	1	1
[K].sLYVSATVILHSGSDMVQAER.[S]	N-Term(iTRAQ4plex)	1	1
[K].qDSLSSQNQLGVLPLSWDIPELVNMGQW	N-Term(iTRAQ4plex); K29(iTRAQ	1	1
[K].vFSLAVNLIAIDSQVLcGAVk.[W]	N-Term(iTRAQ4plex); C17(Carbar	1	1
[K].qDSLSSQNQLGVLPLSWDIPELVNMGQW	N-Term(iTRAQ4plex); K29(iTRAQ	1	1
[K].aAVYHHFISDGVR.[K]	N-Term(iTRAQ4plex)	1	1
[K].rIPIEDGSGEVVLSR.[K]	N-Term(iTRAQ4plex)	1	1
[K].rIPIEDGSGEVVLSR.[K]	N-Term(iTRAQ4plex)	1	1
[K].rIPIEDGSGEVVLSR.[K]	N-Term(iTRAQ4plex)	1	1
[K].rIPIEDGSGEVVLSR.[K]	N-Term(iTRAQ4plex)	1	1
[K].rAPSTWLTAYVVk.[V]	N-Term(iTRAQ4plex); K13(iTRAQ	1	1
[K].rIPIEDGSGEVVLSR.[K]	N-Term(iTRAQ4plex)	1	1
[K].vFSLAVNLIAIDSQVLcGAVk.[W]	N-Term(iTRAQ4plex); C17(Carbar	1	1
[K].vRVELLHNPAFcSLATTk.[R]	N-Term(iTRAQ4plex); C12(Carbar	1	1
[K].sGSDEVQVGQQR.[T]	N-Term(iTRAQ4plex)	1	1
[K].gLEVTITAR.[F]	N-Term(iTRAQ4plex)	1	1
[R].ePGQDLVVLPLSITTDFIPSFR.[L]	N-Term(iTRAQ4plex)	1	1
[R].vELLHNPAFcSLATTK.[R]	N-Term(iTRAQ4plex); C10(Carbar	1	1
[R].ePGQDLVVLPLSITTDFIPSFR.[L]	N-Term(iTRAQ4plex)	1	1
[K].sGSDEVQVGQQR.[T]	N-Term(iTRAQ4plex)	1	1
[K].gLEVTITAR.[F]	N-Term(iTRAQ4plex)	1	1
[K].kQELSEAEQATR.[T]	K1(iTRAQ4plex)	1	1
[K].gLEVTITAR.[F]	N-Term(iTRAQ4plex)	1	1
[R].VELLHNPAFcSLATTk.[R]	C10(Carbamidomethyl); K16(iTR	1	1
[K].gLEVTITAR.[F]	N-Term(iTRAQ4plex)	1	1
[K].vHQYFNVELIQPGAVk.[V]	N-Term(iTRAQ4plex); K16(iTRAQ	1	1
[R].vELLHNPAFcSLATTk.[R]	N-Term(iTRAQ4plex); C10(Carbar	1	1
[R].vELLHNPAFcSLATTk.[R]	N-Term(iTRAQ4plex); C10(Carbar	1	1
[R].qGALELIk.[K]	N-Term(iTRAQ4plex); K8(iTRAQ4	1	1
[K].dMALTAFVLISLQEAk.[D]	N-Term(iTRAQ4plex); K16(iTRAQ	1	1
[K].dMALTAFVLISLQEAk.[D]	N-Term(iTRAQ4plex); K16(iTRAQ	1	1
[K].gIcVADPFEVTVMQDFFIDLR.[L]	N-Term(iTRAQ4plex); C3(Carbam	1	1
[K].QELSEAEQATR.[T]		1	1
[K].dIcEEQVNSLPGSITk.[A]	N-Term(iTRAQ4plex); C3(Carbam	1	1
[K].VLLDGVQNPR.[A]		1	1
[R].hQQTVTIPPk.[S]	N-Term(iTRAQ4plex); K10(iTRAQ	1	1
[R].SGIPIVTSPYQIHFTk.[T]	K16(iTRAQ4plex)	1	1
[K].fVTVQATFGTQVVEk.[V]	N-Term(iTRAQ4plex); K15(iTRAQ	1	1
[R].yYTYLIMNk.[G]	N-Term(iTRAQ4plex); K9(iTRAQ4	1	1

[R].hQQTVTIPPk.[S]	N-Term(iTRAQ4plex); K10(iTRAQ	1	1
[K].vSHSEDDcLAFk.[V]	N-Term(iTRAQ4plex); C8(Carbam	1	1
[K].vYAYYNLEEScTR.[F]	N-Term(iTRAQ4plex); C11(Carbar	1	1
[K].dIcEEQVNSLPGSITk.[A]	N-Term(iTRAQ4plex); C3(Carbam	1	1
[K].yFkPGMPFDLMVFVTNPDGSPAYR.[V]	N-Term(iTRAQ4plex); K3(iTRAQ4	1	1
[K].dIcEEQVNSLPGSITk.[A]	N-Term(iTRAQ4plex); C3(Carbam	1	1
[K].eDIPPADLSDQVPDTESETR.[I]	N-Term(iTRAQ4plex)	1	1
[K].EDIPPADLSDQVPDTESETR.[I]		1	1
[K].vTIkPAPETEk.[R]	N-Term(iTRAQ4plex); K4(iTRAQ4	1	1
[K].eDIPPADLSDQVPDTESETR.[I]	N-Term(iTRAQ4plex)	1	1
[R].sGIPIVTSPYQIHFTk.[T]	N-Term(iTRAQ4plex); K16(iTRAQ	1	1
[K].eDIPPADLSDQVPDTESETR.[I]	N-Term(iTRAQ4plex)	1	1
[K].eDIPPADLSDQVPDTESETR.[I]	N-Term(iTRAQ4plex)	1	1
[K].EDIPPADLSDQVPDTESETR.[I]		1	1
[K].eDIPPADLSDQVPDTESETR.[I]	N-Term(iTRAQ4plex)	1	1
[K].eDIPPADLSDQVPDTESETR.[I]	N-Term(iTRAQ4plex)	1	1
[K].EDIPPADLSDQVPDTESETR.[I]		1	1
[K].eDIPPADLSDQVPDTESETR.[I]	N-Term(iTRAQ4plex)	1	1
[K].eDIPPADLSDQVPDTESETR.[I]	N-Term(iTRAQ4plex)	1	1
[K].eDIPPADLSDQVPDTESETR.[I]	N-Term(iTRAQ4plex)	1	1
[K].eDIPPADLSDQVPDTESETR.[I]	N-Term(iTRAQ4plex)	1	1
[K].eDIPPADLSDQVPDTESETR.[I]	N-Term(iTRAQ4plex)	1	1
[K].eDIPPADLSDQVPDTESETR.[I]	N-Term(iTRAQ4plex)	1	1
[K].eDIPPADLSDQVPDTESETR.[I]	N-Term(iTRAQ4plex)	1	1
[K].fVTVQATFGTQVVEk.[V]	N-Term(iTRAQ4plex); K15(iTRAQ	1	1
[K].yFkPGMPFDLMVFVTNPDGSPAYR.[V]	N-Term(iTRAQ4plex); K3(iTRAQ4	1	1
[K].eDIPPADLSDQVPDTESETR.[I]	N-Term(iTRAQ4plex)	1	1
[R].YYTYLIMNk.[G]	K9(iTRAQ4plex)	1	1
[K].eDIPPADLSDQVPDTESETR.[I]	N-Term(iTRAQ4plex)	1	1
[K].eDIPPADLSDQVPDTESETR.[I]	N-Term(iTRAQ4plex)	1	1
[K].vTIkPAPETEk.[R]	N-Term(iTRAQ4plex); K4(iTRAQ4	1	1
[K].EDIPPADLSDQVPDTESETR.[I]		1	1
[K].eDIPPADLSDQVPDTESETR.[I]	N-Term(iTRAQ4plex)	1	1
[K].eDIPPADLSDQVPDTESETR.[I]	N-Term(iTRAQ4plex)	1	1
[K].eDIPPADLSDQVPDTESETR.[I]	N-Term(iTRAQ4plex)	1	1
[K].EDIPPADLSDQVPDTESETR.[I]		1	1
[K].eDIPPADLSDQVPDTESETR.[I]	N-Term(iTRAQ4plex)	1	1
[K].vTIkPAPETEk.[R]	N-Term(iTRAQ4plex); K4(iTRAQ4	1	1
[K].kVEGTAFVIFGIQDGEQR.[I]	N-Term(iTRAQ4plex); K1(iTRAQ4	1	1
[K].dIcEEQVNSLPGSITk.[A]	N-Term(iTRAQ4plex); C3(Carbam	1	1
[K].eDIPPADLSDQVPDTESETR.[I]	N-Term(iTRAQ4plex)	1	1
[R].SGIPIVTSPYQIHFTk.[T]	K16(iTRAQ4plex)	1	1
[K].eDIPPADLSDQVPDTESETR.[I]	N-Term(iTRAQ4plex)	1	1
[K].eDIPPADLSDQVPDTESETR.[I]	N-Term(iTRAQ4plex)	1	1
[R].sGIPIVTSPYQIHFTk.[T]	N-Term(iTRAQ4plex); K16(iTRAQ	1	1
[K].fVTVQATFGTQVVEk.[V]	N-Term(iTRAQ4plex); K15(iTRAQ	1	1
[R].sGIPIVTSPYQIHFTk.[T]	N-Term(iTRAQ4plex); K16(iTRAQ	1	1
[K].dIcEEQVNSLPGSITk.[A]	N-Term(iTRAQ4plex); C3(Carbam	1	1
[R].sYTVAIAGYALAQMGR.[L]	N-Term(iTRAQ4plex)	1	1
[K].fVTVQATFGTQVVEk.[V]	N-Term(iTRAQ4plex); K15(iTRAQ	1	1
[K].EDIPPADLSDQVPDTESETR.[I]		1	1
[K].eDIPPADLSDQVPDTESETR.[I]	N-Term(iTRAQ4plex)	1	1

[K].EDIPPADLSDQVPDTESETR.[I]		1	1
[K].eDIPPADLSDQVPDTESETR.[I]	N-Term(iTRAQ4plex)	1	1
[K].EDIPPADLSDQVPDTESETR.[I]		1	1
[K].vTIkPAPETEk.[R]	N-Term(iTRAQ4plex); K4(iTRAQ4	1	1
[K].eDIPPADLSDQVPDTESETR.[I]	N-Term(iTRAQ4plex)	1	1
[K].dIcEEQVNSLPGSITk.[A]	N-Term(iTRAQ4plex); C3(Carbam	1	1
[K].eDIPPADLSDQVPDTESETR.[I]	N-Term(iTRAQ4plex)	1	1
[K].eDIPPADLSDQVPDTESETR.[I]	N-Term(iTRAQ4plex)	1	1
[K].eDIPPADLSDQVPDTESETR.[I]	N-Term(iTRAQ4plex)	1	1
[K].eDIPPADLSDQVPDTESETR.[I]	N-Term(iTRAQ4plex)	1	1
[K].eDIPPADLSDQVPDTESETR.[I]	N-Term(iTRAQ4plex)	1	1
[K].fVTVQATFGTQVVEk.[V]	N-Term(iTRAQ4plex); K15(iTRAQ	1	1
[K].vHQYFNVELIQPGAVk.[V]	N-Term(iTRAQ4plex); K16(iTRAQ	1	1
[K].vYAYYNLEEScTR.[F]	N-Term(iTRAQ4plex); C11(Carbar	1	1
[K].vHQYFNVELIQPGAVk.[V]	N-Term(iTRAQ4plex); K16(iTRAQ	1	1
[R].hQQTVTIPPk.[S]	N-Term(iTRAQ4plex); K10(iTRAQ	1	1
[K].vHQYFNVELIQPGAVk.[V]	N-Term(iTRAQ4plex); K16(iTRAQ	1	1
[K].vHQYFNVELIQPGAVk.[V]	N-Term(iTRAQ4plex); K16(iTRAQ	1	1
[R].LVAYYTLIGASGQR.[E]		1	1
[K].kVEGTAFVIFGIQDGEQR.[I]	N-Term(iTRAQ4plex); K1(iTRAQ4	1	1
[K].qELSEAEQATR.[T]	N-Term(iTRAQ4plex)	1	1
[K].qELSEAEQATR.[T]	N-Term(iTRAQ4plex)	1	1
[K].qELSEAEQATR.[T]	N-Term(iTRAQ4plex)	1	1
[K].vHQYFNVELIQPGAVk.[V]	N-Term(iTRAQ4plex); K16(iTRAQ	1	1
[K].vHQYFNVELIQPGAVk.[V]	N-Term(iTRAQ4plex); K16(iTRAQ	1	1
[K].qELSEAEQATR.[T]	N-Term(iTRAQ4plex)	1	1
[K].qELSEAEQATR.[T]	N-Term(iTRAQ4plex)	1	1
[R].LVAYYTLIGASGQR.[E]		1	1
[R].qPVPGQQMTLk.[I]	N-Term(iTRAQ4plex); K11(iTRAQ	1	1
[K].kVEGTAFVIFGIQDGEQR.[I]	N-Term(iTRAQ4plex); K1(iTRAQ4	1	1
[R].vELLHNPAFcSLATTk.[R]	N-Term(iTRAQ4plex); C10(Carbar	1	1
[R].hQQTVTIPPk.[S]	N-Term(iTRAQ4plex); K10(iTRAQ	1	1
[K].vYAYYNLEEScTR.[F]	N-Term(iTRAQ4plex); C11(Carbar	1	1
[K].fVTVQATFGTQVVEk.[V]	N-Term(iTRAQ4plex); K15(iTRAQ	1	1
[R].yYGGGYGSTQATFMVFQALAQYQk.[D]	N-Term(iTRAQ4plex); K24(iTRAQ	1	1
[R].hQQTVTIPPk.[S]	N-Term(iTRAQ4plex); K10(iTRAQ	1	1
[K].dIcEEQVNSLPGSITk.[A]	N-Term(iTRAQ4plex); C3(Carbam	1	1
[K].vYAYYNLEEScTR.[F]	N-Term(iTRAQ4plex); C11(Carbar	1	1
[K].fVTVQATFGTQVVEk.[V]	N-Term(iTRAQ4plex); K15(iTRAQ	1	1
[K].vYAYYNLEEScTR.[F]	N-Term(iTRAQ4plex); C11(Carbar	1	1
[K].RAPSTWLTAYVVk.[V]	K13(iTRAQ4plex)	1	1
[R].hQQTVTIPPk.[S]	N-Term(iTRAQ4plex); K10(iTRAQ	1	1
[K].vYAYYNLEEScTR.[F]	N-Term(iTRAQ4plex); C11(Carbar	1	1
[K].dYAGVFSDAGLTFTSSSGQQTAQR.[A]	N-Term(iTRAQ4plex)	1	1
[K].aDIGcTPGSGk.[D]	N-Term(iTRAQ4plex); C5(Carbam	1	1
[R].lkGPLLNk.[F]	N-Term(iTRAQ4plex); K2(iTRAQ4	1	1
[R].sGIPIVTSPYQIHFTk.[T]	N-Term(iTRAQ4plex); K16(iTRAQ	1	1
[R].SGIPIVTSPYQIHFTk.[T]	K16(iTRAQ4plex)	1	1
[K].aGDFLEANYmNLQR.[S]	N-Term(iTRAQ4plex); M10(Oxida	1	1
[R].qGALELIk.[K]	N-Term(iTRAQ4plex); K8(iTRAQ4	1	1
[R].yYTYLImNk.[G]	N-Term(iTRAQ4plex); M7(Oxidat	1	1
[K].aAVYHHFISDGVR.[K]	N-Term(iTRAQ4plex)	1	1

[K].kVEGTAFVIFGIQDGEQR.[I]	N-Term(iTRAQ4plex); K1(iTRAQ4	1	1
[K].dQLTcNkFDLk.[V]	N-Term(iTRAQ4plex); C5(Carbam	1	1
[R].vVLVAVDk.[G]	N-Term(iTRAQ4plex); K8(iTRAQ4	1	1
[R].vELLHNPAFcSLATTk.[R]	N-Term(iTRAQ4plex); C10(Carbar	1	1
[R].tFISPIk.[C]	N-Term(iTRAQ4plex); K7(iTRAQ4	1	1
[R].vPVAVQGEDTVQSLTQGDGVAk.[L]	N-Term(iTRAQ4plex); K22(iTRAQ	1	1
[R].iHWESASLLR.[S]	N-Term(iTRAQ4plex)	1	1
[R].lESEETMVLEAHDAQGDVPVTVTVHDFPG	N-Term(iTRAQ4plex); K30(iTRAQ	1	1
[R].eALkLEEk.[K]	N-Term(iTRAQ4plex); K4(iTRAQ4	1	1
[K].EDIPPADLSDQVPDTESETR.[I]		1	1
[K].aAVYHHFISDGVR.[K]	N-Term(iTRAQ4plex)	1	1
[K].fVTVQATFGTQVVEk.[V]	N-Term(iTRAQ4plex); K15(iTRAQ	1	1
[K].gYTQQLAFR.[Q]	N-Term(iTRAQ4plex)	1	1
[R].TVmVNIENPEGIPVk.[Q]	M3(Oxidation); K15(iTRAQ4plex)	1	1
[K].vTIkPAPETEk.[R]	N-Term(iTRAQ4plex); K4(iTRAQ4	1	1
[R].fYHPEkEDGk.[L]	N-Term(iTRAQ4plex); K6(iTRAQ4	1	1
[K].lMNIFLk.[D]	N-Term(iTRAQ4plex); K7(iTRAQ4	1	1
[K].eNEGFTVTAEGk.[G]	N-Term(iTRAQ4plex); K12(iTRAQ	1	1
[R].lVAYYTLIGASGQR.[E]	N-Term(iTRAQ4plex)	1	1
[K].AcEPGVDYVYk.[T]	C2(Carbamidomethyl); K11(iTRA	1	1
[K].sDDkVTLEER.[L]	N-Term(iTRAQ4plex); K4(iTRAQ4	1	1
[K].aAVYHHFISDGVR.[K]	N-Term(iTRAQ4plex)	1	1
[K].gYTQQLAFR.[Q]	N-Term(iTRAQ4plex)	1	1
[K].fVTVQATFGTQVVEk.[V]	N-Term(iTRAQ4plex); K15(iTRAQ	1	1
[R].iSLPESLk.[R]	N-Term(iTRAQ4plex); K8(iTRAQ4	1	1
[K].eDIPPADLSDQVPDTESETR.[I]	N-Term(iTRAQ4plex)	1	1
[K].TIYTPGSTVLYR.[I]		1	1
[K].vHQYFNVELIQPGAVk.[V]	N-Term(iTRAQ4plex); K16(iTRAQ	1	1
[K].aGDFLEANYmNLQR.[S]	N-Term(iTRAQ4plex); M10(Oxida	1	1
[R].tFISPIkcR.[E]	N-Term(iTRAQ4plex); K7(iTRAQ4	1	1
[K].ADIGcTPGSGk.[D]	C5(Carbamidomethyl); K11(iTRA	1	1
[K].rIPIEDGSGEVVLSRk.[V]	N-Term(iTRAQ4plex); K16(iTRAQ	1	1
[K].gYTQQLAFR.[Q]	N-Term(iTRAQ4plex)	1	1
[K].rQGALELIkk.[G]	N-Term(iTRAQ4plex); K9(iTRAQ4	1	1
[R].qGALELIk.[K]	N-Term(iTRAQ4plex); K8(iTRAQ4	1	1
[R].vVLVAVDk.[G]	N-Term(iTRAQ4plex); K8(iTRAQ4	1	1
[K].aAVYHHFISDGVR.[K]	N-Term(iTRAQ4plex)	1	1
[K].sLYVSATVILHSGSDMVQAER.[S]	N-Term(iTRAQ4plex)	1	1
[R].lVAYYTLIGASGQR.[E]	N-Term(iTRAQ4plex)	1	1
[R].tkkQELSEAEQATR.[T]	N-Term(iTRAQ4plex); K2(iTRAQ4	1	1
[K].kVEGTAFVIFGIQDGEQR.[I]	N-Term(iTRAQ4plex); K1(iTRAQ4	1	1
[K].fVTVQATFGTQVVEk.[V]	N-Term(iTRAQ4plex); K15(iTRAQ	1	1
[R].fYHPEkEDGk.[L]	N-Term(iTRAQ4plex); K6(iTRAQ4	1	1
[K].vEGTAFVIFGIQDGEQR.[I]	N-Term(iTRAQ4plex)	1	1
[K].aGDFLEANYMNLQR.[S]	N-Term(iTRAQ4plex)	1	1
[K].dFDFVPPVVR.[W]	N-Term(iTRAQ4plex)	1	1
[R].sYTVAIAGYALAQMGR.[L]	N-Term(iTRAQ4plex)	1	1
[R].vELLHNPAFcSLATTk.[R]	N-Term(iTRAQ4plex); C10(Carbar	1	1
[K].fVTVQATFGTQVVEk.[V]	N-Term(iTRAQ4plex); K15(iTRAQ	1	1
[R].sGIPIVTSPYQIHFTk.[T]	N-Term(iTRAQ4plex); K16(iTRAQ	1	1
[K].eDIPPADLSDQVPDTESETR.[I]	N-Term(iTRAQ4plex)	1	1
[K].kVEGTAFVIFGIQDGEQR.[I]	N-Term(iTRAQ4plex); K1(iTRAQ4	1	1

[R].nTLIIYLDk.[V]	N-Term(iTRAQ4plex); K9(iTRAQ4	1	1
[K].kVEGTAFVIFGIQDGEQR.[I]	N-Term(iTRAQ4plex); K1(iTRAQ4	1	1
[R].fISLGEAck.[K]	N-Term(iTRAQ4plex); C8(Carbam	1	1
[K].dYAGVFSDAGLTFTSSSGQQTAQR.[A]	N-Term(iTRAQ4plex)	1	1
[K].dIcEEQVNSLPGSITk.[A]	N-Term(iTRAQ4plex); C3(Carbam	1	1
[R].eGVQkEDIPPADLSDQVPDTESETR.[I]	N-Term(iTRAQ4plex); K5(iTRAQ4	1	1
[K].vYAYYNLEEScTR.[F]	N-Term(iTRAQ4plex); C11(Carbar	1	1
[R].rHQQTVTIPPk.[S]	N-Term(iTRAQ4plex); K11(iTRAQ	1	1
[K].fVTVQATFGTQVVEk.[V]	N-Term(iTRAQ4plex); K15(iTRAQ	1	1
[R].vPVAVQGEDTVQSLTQGDGVAk.[L]	N-Term(iTRAQ4plex); K22(iTRAQ	1	1
[K].aDIGcTPGSGk.[D]	N-Term(iTRAQ4plex); C5(Carbam	1	1
[K].rAPSTWLTAYVVk.[V]	N-Term(iTRAQ4plex); K13(iTRAQ	1	1
[K].sLYVSATVILHSGSDMVQAER.[S]	N-Term(iTRAQ4plex)	1	1
[R].yYTYLIMNk.[G]	N-Term(iTRAQ4plex); K9(iTRAQ4	1	1
[R].lESEETMVLEAHDAQGDVPVTVTVHDFPG	N-Term(iTRAQ4plex); K30(iTRAQ	1	1
[R].lESEETmVLEAHDAQGDVPVTVTVHDFPG	N-Term(iTRAQ4plex); M7(Oxidat	1	1
[K].lVLSSEk.[TL]	N-Term(iTRAQ4plex); K7(iTRAQ4	1	2
[K].DFDFVPPVVR.[W]		1	1
[R].iSLPESLk.[R]	N-Term(iTRAQ4plex); K8(iTRAQ4	1	1
[R].NTLIIYLDk.[V]	K9(iTRAQ4plex)	1	1
[K].eNEGFTVTAEGk.[G]	N-Term(iTRAQ4plex); K12(iTRAQ	1	1
[K].gQGTLSVVTmYHAk.[A]	N-Term(iTRAQ4plex); M10(Oxida	1	1
[R].iPIEDGSGEVVLSR.[K]	N-Term(iTRAQ4plex)	1	1
[K].eDIPPADLSDQVPDTESETR.[I]	N-Term(iTRAQ4plex)	1	1
[K].sDDkVTLEER.[L]	N-Term(iTRAQ4plex); K4(iTRAQ4	1	1
[K].kVEGTAFVIFGIQDGEQR.[I]	N-Term(iTRAQ4plex); K1(iTRAQ4	1	1
[R].iSLPESLk.[R]	N-Term(iTRAQ4plex); K8(iTRAQ4	1	1
[K].vLLDGVQNPR.[A]	N-Term(iTRAQ4plex)	1	1
[R].iPIEDGSGEVVLSR.[K]	N-Term(iTRAQ4plex)	1	1
[K].dYAGVFSDAGLTFTSSSGQQTAQR.[A]	N-Term(iTRAQ4plex)	1	1
[R].eVVADSVWVDVk.[D]	N-Term(iTRAQ4plex); K12(iTRAQ	1	1
[R].lESEETmVLEAHDAQGDVPVTVTVHDFPG	N-Term(iTRAQ4plex); M7(Oxidat	1	1
[K].dScVGSLVVk.[S]	N-Term(iTRAQ4plex); C3(Carbam	1	1
[K].dSITTWEILAVSmSDk.[K]	N-Term(iTRAQ4plex); M13(Oxida	1	1
[R].iSLPESLkR.[I]	N-Term(iTRAQ4plex); K8(iTRAQ4	1	1
[K].eDIPPADLSDQVPDTESETR.[I]	N-Term(iTRAQ4plex)	1	1
[K].aAVYHHFISDGVR.[K]	N-Term(iTRAQ4plex)	1	1
[R].vELLHNPAFcSLATTk.[R]	N-Term(iTRAQ4plex); C10(Carbar	1	1
[R].nTLIIYLDk.[V]	N-Term(iTRAQ4plex); K9(iTRAQ4	1	1
[K].RIPIEDGSGEVVLSR.[K]		1	1
[K].gLEVTITAR.[F]	N-Term(iTRAQ4plex)	1	1
[R].iHWESASLLR.[S]	N-Term(iTRAQ4plex)	1	1
[K].sDDkVTLEER.[L]	N-Term(iTRAQ4plex); K4(iTRAQ4	1	1
[R].fISLGEAcKk.[V]	N-Term(iTRAQ4plex); C8(Carbam	1	1
[R].IFTVNHk.[L]	K7(iTRAQ4plex)	1	1
[K].fVTVQATFGTQVVEk.[V]	N-Term(iTRAQ4plex); K15(iTRAQ	1	1
[R].FYHPEkEDGk.[L]	K6(iTRAQ4plex); K10(iTRAQ4ple:	1	1
[R].fYHPEkEDGk.[L]	N-Term(iTRAQ4plex); K6(iTRAQ4	1	1
[K].dYAGVFSDAGLTFTSSSGQQTAQR.[A]	N-Term(iTRAQ4plex)	1	1
[R].tVmVNIENPEGIPVk.[Q]	N-Term(iTRAQ4plex); M3(Oxidat	1	1
[K].tIYTPGSTVLYR.[I]	N-Term(iTRAQ4plex)	1	1
[K].sDDkVTLEER.[L]	N-Term(iTRAQ4plex); K4(iTRAQ4	1	1

[R].iFTVNHk.[L]	N-Term(iTRAQ4plex); K7(iTRAQ4	1	1
[R].iSLPESLk.[R]	N-Term(iTRAQ4plex); K8(iTRAQ4	1	1
[K].dIcEEQVNSLPGSITk.[A]	N-Term(iTRAQ4plex); C3(Carbam	1	1
[R].HQQTVTIPPk.[S]	K10(iTRAQ4plex)	1	1
[R].tFISPIk.[C]	N-Term(iTRAQ4plex); K7(iTRAQ4	1	1
[K].vHQYFNVELIQPGAVk.[V]	N-Term(iTRAQ4plex); K16(iTRAQ	1	1
[R].eALkLEEk.[K]	N-Term(iTRAQ4plex); K4(iTRAQ4	1	1
[K].lSINTHPSQkPLSITVR.[T]	N-Term(iTRAQ4plex); K10(iTRAQ	1	1
[R].qGALELIk.[K]	N-Term(iTRAQ4plex); K8(iTRAQ4	1	1
[K].fVTVQATFGTQVVEk.[V]	N-Term(iTRAQ4plex); K15(iTRAQ	1	1
[K].vEGTAFVIFGIQDGEQR.[I]	N-Term(iTRAQ4plex)	1	1
[K].lVLSSEk.[TL]	N-Term(iTRAQ4plex); K7(iTRAQ4	1	2
[R].sGIPIVTSPYQIHFTk.[T]	N-Term(iTRAQ4plex); K16(iTRAQ	1	1
[R].sGIPIVTSPYQIHFTk.[T]	N-Term(iTRAQ4plex); K16(iTRAQ	1	1
[K].ENEGFTVTAEGk.[G]	K12(iTRAQ4plex)	1	1
[K].fYYIYNEk.[G]	N-Term(iTRAQ4plex); K8(iTRAQ4	1	1
[R].IHWESASLLR.[S]		1	1
[R].NTLIIYLDk.[V]	K9(iTRAQ4plex)	1	1
[K].kVEGTAFVIFGIQDGEQR.[I]	N-Term(iTRAQ4plex); K1(iTRAQ4	1	1
[K].DTWVEHWPEEDEcQDEENQk.[Q]	C13(Carbamidomethyl); K20(iTR	1	1
[R].TVMVNIENPEGIPVk.[Q]	K15(iTRAQ4plex)	1	1
[R].eVVADSVWVDVk.[D]	N-Term(iTRAQ4plex); K12(iTRAQ	1	1
[R].hQQTVTIPPk.[S]	N-Term(iTRAQ4plex); K10(iTRAQ	1	1
[K].fVTVQATFGTQVVEk.[V]	N-Term(iTRAQ4plex); K15(iTRAQ	1	1
[R].tFISPIk.[C]	N-Term(iTRAQ4plex); K7(iTRAQ4	1	1
[K].aDIGcTPGSGK.[D]	N-Term(iTRAQ4plex); C5(Carbam	1	1
[K].ADIGcTPGSGk.[D]	C5(Carbamidomethyl); K11(iTRA	1	1
[R].aEDLVGk.[S]	N-Term(iTRAQ4plex); K7(iTRAQ4	1	1
[R].aSHLGLAR.[S]	N-Term(iTRAQ4plex)	1	1
[K].vLLDGVQNPR.[A]	N-Term(iTRAQ4plex)	1	1
[R].iSLPESLkRIPIEDGSGEVVLSR.[K]	N-Term(iTRAQ4plex); K8(iTRAQ4	1	1
[R].sVQLTEk.[R]	N-Term(iTRAQ4plex); K7(iTRAQ4	1	1
[R].yYTYLIMNk.[G]	N-Term(iTRAQ4plex); K9(iTRAQ4	1	1
[R].fISLGEAck.[K]	N-Term(iTRAQ4plex); C8(Carbam	1	1
[R].fISLGEAck.[K]	N-Term(iTRAQ4plex); C8(Carbam	1	1
[K].vEGTAFVIFGIQDGEQR.[I]	N-Term(iTRAQ4plex)	1	1
[K].akDQLTcNk.[F]	N-Term(iTRAQ4plex); K2(iTRAQ4	1	1
[R].iPIEDGSGEVVLSR.[K]	N-Term(iTRAQ4plex)	1	1
[R].vVLVAVDk.[G]	N-Term(iTRAQ4plex); K8(iTRAQ4	1	1
[K].sDDkVTLEER.[L]	N-Term(iTRAQ4plex); K4(iTRAQ4	1	1
[K].AAVYHHFISDGVR.[K]		1	1
[R].fYHPEkEDGk.[L]	N-Term(iTRAQ4plex); K6(iTRAQ4	1	1
[R].kVLLDGVQNPR.[A]	N-Term(iTRAQ4plex); K1(iTRAQ4	1	1
[R].lESEETMVLEAHDAQGDVPVTVTVHDFPG	N-Term(iTRAQ4plex); K30(iTRAQ	1	1
[R].aYYENSPQQVFSTEFEVk.[E]	N-Term(iTRAQ4plex); K18(iTRAQ	1	1
[K].fVTVQATFGTQVVEk.[V]	N-Term(iTRAQ4plex); K15(iTRAQ	1	1
[K].DFDFVPPVVR.[W]		1	1
[K].kVFLDccNYITELR.[R]	K1(iTRAQ4plex); C6(Carbamidon	1	1
[R].aYYENSPQQVFSTEFEVk.[E]	N-Term(iTRAQ4plex); K18(iTRAQ	1	1
[K].dFDFVPPVVR.[W]	N-Term(iTRAQ4plex)	1	1
[K].kVFLDccNYITELR.[R]	N-Term(iTRAQ4plex); C6(Carbam	1	1
[R].nTLIIYLDk.[V]	N-Term(iTRAQ4plex); K9(iTRAQ4	1	1

[R].iFTVNHk.[L]	N-Term(iTRAQ4plex); K7(iTRAQ4	1	1
[R].lESEETMVLEAHDAQGDVPVTVTVHDFPG	N-Term(iTRAQ4plex); K30(iTRAQ	1	1
[K].gVFVLNkk.[N]	N-Term(iTRAQ4plex); K7(iTRAQ4	1	1
[K].nTmILEIcTR.[Y]	N-Term(iTRAQ4plex); M3(Oxidat	1	1
[K].akDQLTcNk.[F]	N-Term(iTRAQ4plex); K2(iTRAQ4	1	1
[R].aSHLGLAR.[S]	N-Term(iTRAQ4plex)	1	1
[R].kVLLDGVQNPR.[A]	N-Term(iTRAQ4plex); K1(iTRAQ4	1	1
[K].dYAGVFSDAGLTFTSSSGQQTAQR.[A]	N-Term(iTRAQ4plex)	1	1
[R].tFISPIk.[C]	N-Term(iTRAQ4plex); K7(iTRAQ4	1	1
[R].IFTVNHkLLPVGR.[T]	K7(iTRAQ4plex)	1	1
[R].iPIEDGSGEVVLSR.[K]	N-Term(iTRAQ4plex)	1	1
[K].tIYTPGSTVLYR.[I]	N-Term(iTRAQ4plex)	1	1
[R].iSLPESLk.[R]	N-Term(iTRAQ4plex); K8(iTRAQ4	1	1
[R].qGALELIk.[K]	N-Term(iTRAQ4plex); K8(iTRAQ4	1	1
[R].sVQLTEk.[R]	N-Term(iTRAQ4plex); K7(iTRAQ4	1	1
[K].vLLDGVQNPR.[A]	N-Term(iTRAQ4plex)	1	1
[R].fISLGEAck.[K]	N-Term(iTRAQ4plex); C8(Carbam	1	1
[K].EDIPPADLSDQVPDTESETR.[I]		1	1
[R].qPVPGQQmTLk.[I]	N-Term(iTRAQ4plex); M8(Oxidat	1	1
[R].fISLGEAck.[K]	N-Term(iTRAQ4plex); C8(Carbam	1	1
[K].dYAGVFSDAGLTFTSSSGQQTAQR.[A]	N-Term(iTRAQ4plex)	1	1
[K].lSINTHPSQkPLSITVR.[T]	N-Term(iTRAQ4plex); K10(iTRAQ	1	1
[R].hQQTVTIPPk.[S]	N-Term(iTRAQ4plex); K10(iTRAQ	1	1
[K].kQELSEAEQATR.[T]	N-Term(iTRAQ4plex); K1(iTRAQ4	1	1
[K].dFDFVPPVVR.[W]	N-Term(iTRAQ4plex)	1	1
[K].SGSDEVQVGQQR.[T]		1	1
[K].tIYTPGSTVLYR.[I]	N-Term(iTRAQ4plex)	1	1
[R].kVLLDGVQNPR.[A]	N-Term(iTRAQ4plex); K1(iTRAQ4	1	1
[R].LVAYYTLIGASGQR.[E]		1	1
[R].yYTYLIMNk.[G]	N-Term(iTRAQ4plex); K9(iTRAQ4	1	1
[R].fYHPEkEDGk.[L]	N-Term(iTRAQ4plex); K6(iTRAQ4	1	1
[R].sEFPESWLWNVEDLkEPPkNGISTk.[L]	N-Term(iTRAQ4plex); K15(iTRAQ	1	1
[K].dYAGVFSDAGLTFTSSSGQQTAQR.[A]	N-Term(iTRAQ4plex)	1	1
[R].tVMVNIENPEGIPVk.[Q]	N-Term(iTRAQ4plex); K15(iTRAQ	1	1
[R].VVLVAVDk.[G]	K8(iTRAQ4plex)	1	1
[K].ADIGcTPGSGk.[D]	C5(Carbamidomethyl); K11(iTRA	1	1
[K].sDDkVTLEER.[L]	N-Term(iTRAQ4plex); K4(iTRAQ4	1	1
[K].dAPDHQELNLDVSLQLPSR.[S]	N-Term(iTRAQ4plex)	1	1
[R].IHWESASLLR.[S]		1	1
[R].sEFPESWLWNVEDLkEPPk.[N]	N-Term(iTRAQ4plex); K15(iTRAQ	1	1
[K].DFDFVPPVVR.[W]		1	1
[K].vLLDGVQNPR.[A]	N-Term(iTRAQ4plex)	1	1
[K].dFDFVPPVVR.[W]	N-Term(iTRAQ4plex)	1	1
[R].aEDLVGk.[S]	N-Term(iTRAQ4plex); K7(iTRAQ4	1	1
[K].DFDFVPPVVR.[W]		1	1
[R].yYTYLIMNk.[G]	N-Term(iTRAQ4plex); K9(iTRAQ4	1	1
[K].eDIPPADLSDQVPDTESETR.[I]	N-Term(iTRAQ4plex)	1	1
[K].fVTVQATFGTQVVEk.[V]	N-Term(iTRAQ4plex); K15(iTRAQ	1	1
[K].gQGTLSVVTmYHAk.[A]	N-Term(iTRAQ4plex); M10(Oxida	1	1
[R].iFTVNHk.[L]	N-Term(iTRAQ4plex); K7(iTRAQ4	1	1
[K].TGLQEVEVk.[A]	K9(iTRAQ4plex)	1	1
[K].dQLTcNk.[F]	N-Term(iTRAQ4plex); C5(Carbam	1	1

[K].VLLDGVQNPR.[A]		1	1
[R].VELLHNPAFcSLATTk.[R]	C10(Carbamidomethyl); K16(iTR	1	1
[K].rIPIEDGSGEVVLSR.[K]	N-Term(iTRAQ4plex)	1	1
[K].lMNIFLk.[D]	N-Term(iTRAQ4plex); K7(iTRAQ4	1	1
[R].NTLIIYLDk.[V]	K9(iTRAQ4plex)	1	1
[R].LVAYYTLIGASGQR.[E]		1	1
[K].gQGTLSVVTmYHAk.[A]	N-Term(iTRAQ4plex); M10(Oxida	1	1
[K].kQELSEAEQATR.[T]	N-Term(iTRAQ4plex); K1(iTRAQ4	1	1
[R].iLLQGTPVAQmTEDAVDAER.[L]	N-Term(iTRAQ4plex); M11(Oxida	1	1
[R].eALkLEEk.[K]	N-Term(iTRAQ4plex); K4(iTRAQ4	1	1
[K].kVEGTAFVIFGIQDGEQR.[I]	N-Term(iTRAQ4plex); K1(iTRAQ4	1	1
[K].fVTVQATFGTQVVEk.[V]	N-Term(iTRAQ4plex); K15(iTRAQ	1	1
[R].tkkQELSEAEQATR.[T]	N-Term(iTRAQ4plex); K2(iTRAQ4	1	1
[R].iSLPESLk.[R]	N-Term(iTRAQ4plex); K8(iTRAQ4	1	1
[K].aDIGcTPGSGk.[D]	N-Term(iTRAQ4plex); C5(Carbam	1	1
[R].fYHPEkEDGk.[L]	N-Term(iTRAQ4plex); K6(iTRAQ4	1	1
[R].lESEETMVLEAHDAQGDVPVTVTVHDFPG	N-Term(iTRAQ4plex); K30(iTRAQ	1	1
[K].lVLSSEk.[TL]	N-Term(iTRAQ4plex); K7(iTRAQ4	1	2
[R].tFISPIk.[C]	N-Term(iTRAQ4plex); K7(iTRAQ4	1	1
[R].lESEETMVLEAHDAQGDVPVTVTVHDFPG	N-Term(iTRAQ4plex); K30(iTRAQ	1	1
[R].iHWESASLLR.[S]	N-Term(iTRAQ4plex)	1	1
[K].sLYVSATVILHSGSDMVQAER.[S]	N-Term(iTRAQ4plex)	1	1
[K].EDIPPADLSDQVPDTESETR.[I]		1	1
[R].tELRPGETLNVNFLLR.[M]	N-Term(iTRAQ4plex)	1	1
[R].tVmVNIENPEGIPVk.[Q]	N-Term(iTRAQ4plex); M3(Oxidat	1	1
[K].gVFVLNk.[K]	N-Term(iTRAQ4plex); K7(iTRAQ4	1	1
[R].HQQTVTIPPk.[S]	K10(iTRAQ4plex)	1	1
[R].IFTVNHkLLPVGR.[T]	K7(iTRAQ4plex)	1	1
[R].kVLLDGVQNPR.[A]	N-Term(iTRAQ4plex); K1(iTRAQ4	1	1
[R].sVQLTEk.[R]	N-Term(iTRAQ4plex); K7(iTRAQ4	1	1
[R].qGALELIk.[K]	N-Term(iTRAQ4plex); K8(iTRAQ4	1	1
[K].EDIPPADLSDQVPDTESETR.[I]		1	1
[R].FISLGEAck.[K]	C8(Carbamidomethyl); K9(iTRAQ	1	1
[K].aAVYHHFISDGVR.[K]	N-Term(iTRAQ4plex)	1	1
[K].aKDQLTcNk.[F]	N-Term(iTRAQ4plex); C7(Carbam	1	1
[R].vELLHNPAFcSLATTKR.[R]	N-Term(iTRAQ4plex); C10(Carbar	1	1
[R].IHWESASLLR.[S]		1	1
[K].acEPGVDYVYk.[T]	N-Term(iTRAQ4plex); C2(Carbam	1	1
[R].aEDLVGk.[S]	N-Term(iTRAQ4plex); K7(iTRAQ4	1	1
[K].eDIPPADLSDQVPDTESETR.[I]	N-Term(iTRAQ4plex)	1	1
[R].sVQLTEk.[R]	N-Term(iTRAQ4plex); K7(iTRAQ4	1	1
[R].fISLGEAck.[K]	N-Term(iTRAQ4plex); C8(Carbam	1	1
[K].IWDVVEk.[A]	K7(iTRAQ4plex)	1	1
[K].fYYIYNEk.[G]	N-Term(iTRAQ4plex); K8(iTRAQ4	1	1
[R].aYYENSPQQVFSTEFEVk.[E]	N-Term(iTRAQ4plex); K18(iTRAQ	1	1
[R].tVMVNIENPEGIPVk.[Q]	N-Term(iTRAQ4plex); K15(iTRAQ	1	1
[K].eDIPPADLSDQVPDTESETR.[I]	N-Term(iTRAQ4plex)	1	1
[K].EDIPPADLSDQVPDTESETR.[I]		1	1
[R].lDkAcEPGVDYVYkTR.[L]	N-Term(iTRAQ4plex); K3(iTRAQ4	1	1
[R].VELLHNPAFcSLATTk.[R]	C10(Carbamidomethyl); K16(iTR	1	1
[K].TGLQEVEVk.[A]	K9(iTRAQ4plex)	1	1
[R].HQQTVTIPPk.[S]	K10(iTRAQ4plex)	1	1

[K].lVLSSEk.[TL]	N-Term(iTRAQ4plex); K7(iTRAQ4	1	2
[K].aDIGcTPGSGk.[D]	N-Term(iTRAQ4plex); C5(Carbam	1	1
[K].aDIGcTPGSGk.[D]	N-Term(iTRAQ4plex); C5(Carbam	1	1
[R].fYHPEkEDGk.[L]	N-Term(iTRAQ4plex); K6(iTRAQ4	1	1
[K].lVLSSEk.[TL]	N-Term(iTRAQ4plex); K7(iTRAQ4	1	2
[R].fYHPEkEDGk.[L]	N-Term(iTRAQ4plex); K6(iTRAQ4	1	1
[K].fVTVQATFGTQVVEk.[V]	N-Term(iTRAQ4plex); K15(iTRAQ	1	1
[R].iFTVNHk.[L]	N-Term(iTRAQ4plex); K7(iTRAQ4	1	1
[R].yYTYLIMNk.[G]	N-Term(iTRAQ4plex); K9(iTRAQ4	1	1
[K].sGSDEVQVGQQR.[T]	N-Term(iTRAQ4plex)	1	1
[K].gVFVLNk.[K]	N-Term(iTRAQ4plex); K7(iTRAQ4	1	1
[K].lVLSSEk.[TL]	N-Term(iTRAQ4plex); K7(iTRAQ4	1	2
[R].sEETkENEGFTVTAEGk.[G]	N-Term(iTRAQ4plex); K5(iTRAQ4	1	1
[R].ISLPESLk.[R]	K8(iTRAQ4plex)	1	1
[K].kLVLSSEk.[T]	N-Term(iTRAQ4plex); K1(iTRAQ4	1	1
[K].gQGTLSVVTmYHAk.[A]	N-Term(iTRAQ4plex); M10(Oxida	1	1
[R].VVLVAVDk.[G]	K8(iTRAQ4plex)	1	1
[R].ISLPESLk.[R]	K8(iTRAQ4plex)	1	1
[K].dTWVEHWPEEDEcQDEENQk.[Q]	N-Term(iTRAQ4plex); C13(Carbar	1	1
[R].tVMVNIENPEGIPVk.[Q]	N-Term(iTRAQ4plex); K15(iTRAQ	1	1
[K].fVTVQATFGTQVVEk.[V]	N-Term(iTRAQ4plex); K15(iTRAQ	1	1
[K].vTIkPAPETEk.[R]	N-Term(iTRAQ4plex); K4(iTRAQ4	1	1
[K].sGSDEVQVGQQR.[T]	N-Term(iTRAQ4plex)	1	1
[R].sEFPESWLWNVEDLkEPPkNGISTk.[L]	N-Term(iTRAQ4plex); K15(iTRAQ	1	1
[K].iWDVVEk.[A]	N-Term(iTRAQ4plex); K7(iTRAQ4	1	1
[R].hQQTVTIPPk.[S]	N-Term(iTRAQ4plex); K10(iTRAQ	1	1
[R].TkkQELSEAEQATR.[T]	K2(iTRAQ4plex); K3(iTRAQ4plex)	1	1
[K].sGSDEVQVGQQR.[T]	N-Term(iTRAQ4plex)	1	1
[K].dQLTcNk.[F]	N-Term(iTRAQ4plex); C5(Carbam	1	1
[R].iSLPESLk.[R]	N-Term(iTRAQ4plex); K8(iTRAQ4	1	1
[K].tGLQEVEVk.[A]	N-Term(iTRAQ4plex); K9(iTRAQ4	1	1
[R].VPVAVQGEDTVQSLTQGDGVAk.[L]	K22(iTRAQ4plex)	1	1
[K].gVFVLNk.[K]	N-Term(iTRAQ4plex); K7(iTRAQ4	1	1
[R].iFTVNHk.[L]	N-Term(iTRAQ4plex); K7(iTRAQ4	1	1
[R].sVQLTEk.[R]	N-Term(iTRAQ4plex); K7(iTRAQ4	1	1
[K].LVLSSEk.[TL]	K7(iTRAQ4plex)	1	2
[K].kVEGTAFVIFGIQDGEQR.[I]	N-Term(iTRAQ4plex); K1(iTRAQ4	1	1
[K].vLLDGVQNPR.[A]	N-Term(iTRAQ4plex)	1	1
[R].qPSSAFAAFVk.[R]	N-Term(iTRAQ4plex); K11(iTRAQ	1	1
[R].vPVAVQGEDTVQSLTQGDGVAk.[L]	N-Term(iTRAQ4plex); K22(iTRAQ	1	1
[K].akDQLTcNk.[F]	N-Term(iTRAQ4plex); K2(iTRAQ4	1	1
[K].kVEGTAFVIFGIQDGEQR.[I]	N-Term(iTRAQ4plex); K1(iTRAQ4	1	1
[K].iWDVVEk.[A]	N-Term(iTRAQ4plex); K7(iTRAQ4	1	1
[K].iWDVVEk.[A]	N-Term(iTRAQ4plex); K7(iTRAQ4	1	1
[K].gLEVTITAR.[F]	N-Term(iTRAQ4plex)	1	1
[R].lVAYYTLIGASGQR.[E]	N-Term(iTRAQ4plex)	1	1
[R].fYHPEkEDGk.[L]	N-Term(iTRAQ4plex); K6(iTRAQ4	1	1
[K].nTmILEIcTR.[Y]	N-Term(iTRAQ4plex); M3(Oxidat	1	1
[R].IHWESASLLR.[S]		1	1
[R].lESEETMVLEAHDAQGDVPVTVTVHDFPG	N-Term(iTRAQ4plex); K30(iTRAQ	1	1
[K].dScVGSLVVk.[S]	N-Term(iTRAQ4plex); C3(Carbam	1	1
[R].yYTYLImNk.[G]	N-Term(iTRAQ4plex); M7(Oxidat	1	1

[R].vPVAVQGEDTVQSLTQGDGVAk.[L]	N-Term(iTRAQ4plex); K22(iTRAQ4	1	1
[K].AcEPGVDYVYkTR.[L]	C2(Carbamidomethyl); K11(iTRA	1	1
[K].eNEGFTVTAEGk.[G]	N-Term(iTRAQ4plex); K12(iTRAQ4	1	1
[K].DFDFVPPVVR.[W]		1	1
[K].qcQDLGAFTESmVVFGcPN.[-]	N-Term(iTRAQ4plex); C2(Carbam	1	1
[K].dFDFVPPVVR.[W]	N-Term(iTRAQ4plex)	1	1
[K].fVTVQATFGTQVVEk.[V]	N-Term(iTRAQ4plex); K15(iTRAQ4	1	1
[K].lmNIFLk.[D]	N-Term(iTRAQ4plex); M2(Oxidat	1	1
[K].lSINTHPSQKPLSITVR.[T]	N-Term(iTRAQ4plex)	1	1
[K].dYAGVFSDAGLTFTSSSGQQTAQR.[A]	N-Term(iTRAQ4plex)	1	1
[K].fVTVQATFGTQVVEk.[V]	N-Term(iTRAQ4plex); K15(iTRAQ4	1	1
[K].sLYVSATVILHSGSDMVQAER.[S]	N-Term(iTRAQ4plex)	1	1
[R].lESEETMVLEAHDAQGDVPVTVTVHDFPG	N-Term(iTRAQ4plex); K30(iTRAQ4	1	1
[K].kGYTQQLAFR.[Q]	N-Term(iTRAQ4plex); K1(iTRAQ4	1	1
[K].sDDkVTLEER.[L]	N-Term(iTRAQ4plex); K4(iTRAQ4	1	1
[K].gVFVLNk.[K]	N-Term(iTRAQ4plex); K7(iTRAQ4	1	1
[K].gLEVTITAR.[F]	N-Term(iTRAQ4plex)	1	1
[K].sDDkVTLEER.[L]	N-Term(iTRAQ4plex); K4(iTRAQ4	1	1
[K].TGLQEVEVk.[A]	K9(iTRAQ4plex)	1	1
[K].iWDVVEk.[A]	N-Term(iTRAQ4plex); K7(iTRAQ4	1	1
[R].tFISPIk.[C]	N-Term(iTRAQ4plex); K7(iTRAQ4	1	1
[K].lVLSSEk.[TL]	N-Term(iTRAQ4plex); K7(iTRAQ4	1	2
[K].nTMILEIcTR.[Y]	N-Term(iTRAQ4plex); C8(Carbam	1	1
[K].fYYIYNEk.[G]	N-Term(iTRAQ4plex); K8(iTRAQ4	1	1
[K].AcEPGVDYVYk.[T]	C2(Carbamidomethyl); K11(iTRA	1	1
[K].vYAYYNLEEScTR.[F]	N-Term(iTRAQ4plex); C11(Carbar	1	1
[K].nTmILEIcTR.[Y]	N-Term(iTRAQ4plex); M3(Oxidat	1	1
[K].acEPGVDYVYkTR.[L]	N-Term(iTRAQ4plex); C2(Carbam	1	1
[R].iFTVNHk.[L]	N-Term(iTRAQ4plex); K7(iTRAQ4	1	1
[R].iFTVNHk.[L]	N-Term(iTRAQ4plex); K7(iTRAQ4	1	1
[K].rAPSTWLTAYVVk.[V]	N-Term(iTRAQ4plex); K13(iTRAQ4	1	1
[R].iHWESASLLR.[S]	N-Term(iTRAQ4plex)	1	1
[R].TVMVNIENPEGIPVk.[Q]	K15(iTRAQ4plex)	1	1
[K].NTMILEIcTR.[Y]	C8(Carbamidomethyl)	1	1
[R].eVVADSVWVDVk.[D]	N-Term(iTRAQ4plex); K12(iTRAQ4	1	1
[K].iWDVVEk.[A]	N-Term(iTRAQ4plex); K7(iTRAQ4	1	1
[K].dQLTcNk.[F]	N-Term(iTRAQ4plex); C5(Carbam	1	1
[K].dIcEEQVNSLPGSITk.[A]	N-Term(iTRAQ4plex); C3(Carbam	1	1
[K].rQGALELIk.[K]	N-Term(iTRAQ4plex); K9(iTRAQ4	1	1
[R].yYTYLImNk.[G]	N-Term(iTRAQ4plex); M7(Oxidat	1	1
[R].YYTYLIMNk.[G]	K9(iTRAQ4plex)	1	1
[K].EDIPPADLSDQVPDTESETR.[I]		1	1
[R].iSLPESLk.[R]	N-Term(iTRAQ4plex); K8(iTRAQ4	1	1
[R].HQQTVTIPPk.[S]	K10(iTRAQ4plex)	1	1
[K].aDIGcTPGSGk.[D]	N-Term(iTRAQ4plex); C5(Carbam	1	1
[K].kVFLDccNYITELR.[R]	N-Term(iTRAQ4plex); K1(iTRAQ4	1	1
[R].iSLPESLk.[R]	N-Term(iTRAQ4plex); K8(iTRAQ4	1	1
[K].lSINTHPSQkPLSITVR.[T]	N-Term(iTRAQ4plex); K10(iTRAQ4	1	1
[K].fYYIYNEk.[G]	N-Term(iTRAQ4plex); K8(iTRAQ4	1	1
[R].nTLIIYLDk.[V]	N-Term(iTRAQ4plex); K9(iTRAQ4	1	1
[K].vLLDGVQNPR.[A]	N-Term(iTRAQ4plex)	1	1
[R].vVLVAVDk.[G]	N-Term(iTRAQ4plex); K8(iTRAQ4	1	1

[K].iWDVVEk.[A]	N-Term(iTRAQ4plex); K7(iTRAQ4	1	1
[K].ENEGFTVTAEGk.[G]	K12(iTRAQ4plex)	1	1
[K].GYTQQLAFR.[Q]		1	1
[R].sVQLTEk.[R]	N-Term(iTRAQ4plex); K7(iTRAQ4	1	1
[K].kVEGTAFVIFGIQDGEQR.[I]	N-Term(iTRAQ4plex); K1(iTRAQ4	1	1
[R].aEDLVGk.[S]	N-Term(iTRAQ4plex); K7(iTRAQ4	1	1
[K].sLYVSATVILHSGSDMVQAER.[S]	N-Term(iTRAQ4plex)	1	1
[R].sEFPESWLWNVEDLkEPPkNGISTk.[L]	N-Term(iTRAQ4plex); K15(iTRAQ	1	1
[K].akDQLTcNk.[F]	N-Term(iTRAQ4plex); K2(iTRAQ4	1	1
[K].LSINTHPSQkPLSITVR.[T]	K10(iTRAQ4plex)	1	1
[K].gVFVLNk.[K]	N-Term(iTRAQ4plex); K7(iTRAQ4	1	1
[K].gLEVTITAR.[F]	N-Term(iTRAQ4plex)	1	1
[R].qPVPGQQMTLkIEGDHGAR.[V]	N-Term(iTRAQ4plex); K11(iTRAQ	1	1
[K].kGYTQQLAFR.[Q]	N-Term(iTRAQ4plex); K1(iTRAQ4	1	1
[K].tGLQEVEVK.[A]	N-Term(iTRAQ4plex)	1	1
[K].qcQDLGAFTESmVVFGcPN.[-]	N-Term(iTRAQ4plex); C2(Carbam	1	1
[R].LESEETMVLEAHDAQGDVPVTVTVHDFPG	K30(iTRAQ4plex)	1	1
[K].acEPGVDYVYk.[T]	N-Term(iTRAQ4plex); C2(Carbam	1	1
[R].nTLIIYLDk.[V]	N-Term(iTRAQ4plex); K9(iTRAQ4	1	1
[R].iLLQGTPVAQmTEDAVDAER.[L]	N-Term(iTRAQ4plex); M11(Oxida	1	1
[R].fISLGEAckk.[V]	N-Term(iTRAQ4plex); C8(Carbam	1	1
[R].iLLQGTPVAQmTEDAVDAER.[L]	N-Term(iTRAQ4plex); M11(Oxida	1	1
[K].kGYTQQLAFR.[Q]	N-Term(iTRAQ4plex); K1(iTRAQ4	1	1
[K].vHQYFNVELIQPGAVk.[V]	N-Term(iTRAQ4plex); K16(iTRAQ	1	1
[K].IWDVVEk.[A]	K7(iTRAQ4plex)	1	1
[R].ISLPESLkR.[I]	K8(iTRAQ4plex)	1	1
[K].GQGTLSVVTMYHAk.[A]	K14(iTRAQ4plex)	1	1
[K].VTIkPAPETEk.[R]	K4(iTRAQ4plex); K11(iTRAQ4ple	1	1
[K].eDIPPADLSDQVPDTESETR.[I]	N-Term(iTRAQ4plex)	1	1
[K].EDIPPADLSDQVPDTESETR.[I]		1	1
[K].TGLQEVEVk.[A]	K9(iTRAQ4plex)	1	1
[K].TGLQEVEVk.[A]	K9(iTRAQ4plex)	1	1
[R].lESEETMVLEAHDAQGDVPVTVTVHDFPG	N-Term(iTRAQ4plex); K30(iTRAQ	1	1
[R].kVLLDGVQNPR.[A]	N-Term(iTRAQ4plex)	1	1
[R].qGALELIk.[K]	N-Term(iTRAQ4plex); K8(iTRAQ4	1	1
[K].lVLSSEk.[TL]	N-Term(iTRAQ4plex); K7(iTRAQ4	1	2
[R].lVAYYTLIGASGQR.[E]	N-Term(iTRAQ4plex)	1	1
[K].EDIPPADLSDQVPDTESETR.[I]		1	1
[K].kVEGTAFVIFGIQDGEQR.[I]	N-Term(iTRAQ4plex); K1(iTRAQ4	1	1
[K].acEPGVDYVYk.[T]	N-Term(iTRAQ4plex); C2(Carbam	1	1
[R].kVLLDGVQNPR.[A]	K1(iTRAQ4plex)	1	1
[R].tFISPIk.[C]	N-Term(iTRAQ4plex); K7(iTRAQ4	1	1
[K].iWDVVEk.[A]	N-Term(iTRAQ4plex); K7(iTRAQ4	1	1
[R].vVLVAVDk.[G]	N-Term(iTRAQ4plex); K8(iTRAQ4	1	1
[K].dQLTcNk.[F]	N-Term(iTRAQ4plex); C5(Carbam	1	1
[R].QPSSAFAAFVk.[R]	K11(iTRAQ4plex)	1	1
[K].lSINTHPSQkPLSITVR.[T]	N-Term(iTRAQ4plex); K10(iTRAQ	1	1
[R].sVQLTEk.[R]	N-Term(iTRAQ4plex); K7(iTRAQ4	1	1
[R].sNLDEDIIAEENIVSR.[S]	N-Term(iTRAQ4plex)	1	1
[K].sLYVSATVILHSGSDMVQAER.[S]	N-Term(iTRAQ4plex)	1	1
[R].YYTYLImNk.[G]	M7(Oxidation); K9(iTRAQ4plex)	1	1
[R].LkGPLLNk.[F]	K2(iTRAQ4plex); K8(iTRAQ4plex)	1	1

[K].DIcEEQVNSLPGSITk.[A]	C3(Carbamidomethyl); K16(iTRA	1	1
[K].kVEGTAFVIFGIQDGEQR.[I]	N-Term(iTRAQ4plex); K1(iTRAQ4	1	1
[K].fYYIYNEk.[G]	N-Term(iTRAQ4plex); K8(iTRAQ4	1	1
[K].EDIPPADLSDQVPDTESETR.[I]		1	1
[K].vTIkPAPETEk.[R]	N-Term(iTRAQ4plex); K4(iTRAQ4	1	1
[K].sSLSVPYVIVPLK.[T]	N-Term(iTRAQ4plex)	1	1
[R].qPVPGQQmTLk.[I]	N-Term(iTRAQ4plex); M8(Oxidat	1	1
[R].tELRPGETLNVNFLLR.[M]	N-Term(iTRAQ4plex)	1	1
[K].dQLTcNk.[F]	N-Term(iTRAQ4plex); C5(Carbam	1	1
[R].iSLPESLk.[R]	N-Term(iTRAQ4plex); K8(iTRAQ4	1	1
[R].iFTVNHk.[L]	N-Term(iTRAQ4plex); K7(iTRAQ4	1	1
[K].dFDFVPPVVR.[W]	N-Term(iTRAQ4plex)	1	1
[R].hQQTVTIPPk.[S]	N-Term(iTRAQ4plex); K10(iTRAQ	1	1
[K].nkLTQSk.[IV]	N-Term(iTRAQ4plex); K2(iTRAQ4	1	2
[K].dYAGVFSDAGLTFTSSSGQQTAQR.[A]	N-Term(iTRAQ4plex)	1	1
[K].FYYIYNEk.[G]	K8(iTRAQ4plex)	1	1
[K].GYTQQLAFR.[Q]		1	1
[K].gVFVLNk.[K]	N-Term(iTRAQ4plex); K7(iTRAQ4	1	1
[R].FYHPEkEDGk.[L]	K6(iTRAQ4plex); K10(iTRAQ4plex	1	1
[K].gVFVLNk.[K]	N-Term(iTRAQ4plex); K7(iTRAQ4	1	1
[K].fVTVQATFGTQVVEk.[V]	N-Term(iTRAQ4plex); K15(iTRAQ	1	1
[K].sDDkVTLEER.[L]	N-Term(iTRAQ4plex); K4(iTRAQ4	1	1
[K].kQELSEAEQATR.[T]	N-Term(iTRAQ4plex); K1(iTRAQ4	1	1
[K].qkPDGVFQEDAPVIHQEMIGGLR.[N]	N-Term(iTRAQ4plex); K2(iTRAQ4	1	1
[R].VVLVAVDk.[G]	K8(iTRAQ4plex)	1	1
[R].VVLVAVDk.[G]	K8(iTRAQ4plex)	1	1
[K].dYAGVFSDAGLTFTSSSGQQTAQR.[A]	N-Term(iTRAQ4plex)	1	1
[R].YYTYLImNk.[G]	M7(Oxidation); K9(iTRAQ4plex)	1	1
[R].IHWESASLLR.[S]		1	1
[K].fVTVQATFGTQVVEk.[V]	N-Term(iTRAQ4plex); K15(iTRAQ	1	1
[R].sNLDEDIIAEENIVSR.[S]	N-Term(iTRAQ4plex)	1	1
[R].sVQLTEk.[R]	N-Term(iTRAQ4plex); K7(iTRAQ4	1	1
[R].eVVADSVWVDVk.[D]	N-Term(iTRAQ4plex); K12(iTRAQ	1	1
[K].lSINTHPSQkPLSITVR.[T]	N-Term(iTRAQ4plex); K10(iTRAQ	1	1
[R].yYTYLIMNk.[G]	N-Term(iTRAQ4plex); K9(iTRAQ4	1	1
[K].dTWVEHWPEEDEcQDEENQk.[Q]	N-Term(iTRAQ4plex); C13(Carbar	1	1
[K].TGLQEVEVk.[A]	K9(iTRAQ4plex)	1	1
[K].kVEGTAFVIFGIQDGEQR.[I]	N-Term(iTRAQ4plex); K1(iTRAQ4	1	1
[R].tVMVNIENPEGIPVk.[Q]	N-Term(iTRAQ4plex); K15(iTRAQ	1	1
[K].dAPDHQELNLDVSLQLPSR.[S]	N-Term(iTRAQ4plex)	1	1
[K].nTMILEIcTR.[Y]	N-Term(iTRAQ4plex); C8(Carbam	1	1
[K].vFLDccNYITELR.[R]	N-Term(iTRAQ4plex); C5(Carbam	1	1
[R].HQQTVTIPPk.[S]	K10(iTRAQ4plex)	1	1
[R].kVLLDGVQNPR.[A]	N-Term(iTRAQ4plex); K1(iTRAQ4	1	1
[K].EDIPPADLSDQVPDTESETR.[I]		1	1
[K].dQLTcNk.[F]	N-Term(iTRAQ4plex); C5(Carbam	1	1
[R].SVQLTEKR.[M]		1	1
[R].sVQLTEk.[R]	N-Term(iTRAQ4plex); K7(iTRAQ4	1	1
[K].acEPGVDYVYkTR.[L]	N-Term(iTRAQ4plex); C2(Carbam	1	1
[K].lSINTHPSQkPLSITVR.[T]	N-Term(iTRAQ4plex); K10(iTRAQ	1	1
[R].sYTVAIAGYALAQMGR.[L]	N-Term(iTRAQ4plex)	1	1
[K].kQELSEAEQATR.[T]	N-Term(iTRAQ4plex); K1(iTRAQ4	1	1

[R].ILLQGTPVAQMTEDAVDAER.[L]		1	1
[K].lVLSSEk.[TL]	N-Term(iTRAQ4plex); K7(iTRAQ4	1	2
[R].yYTYLImNk.[G]	N-Term(iTRAQ4plex); M7(Oxidat	1	1
[R].lVAYYTLIGASGQR.[E]	N-Term(iTRAQ4plex)	1	1
[K].LMNIFLk.[D]	K7(iTRAQ4plex)	1	1
[R].TVMVNIENPEGIPVk.[Q]	K15(iTRAQ4plex)	1	1
[K].iEGDHGAR.[V]	N-Term(iTRAQ4plex)	1	1
[R].cAEENcFIQk.[S]	C1(Carbamidomethyl); C6(Carba	1	1
[R].IFTVNHk.[L]	K7(iTRAQ4plex)	1	1
[K].aGDFLEANYMNLQR.[S]	N-Term(iTRAQ4plex)	1	1
[R].kVLLDGVQNPR.[A]	N-Term(iTRAQ4plex)	1	1
[K].gYTQQLAFR.[Q]	N-Term(iTRAQ4plex)	1	1
[R].kVLLDGVQNPR.[A]	K1(iTRAQ4plex)	1	1
[K].lMNIFLk.[D]	N-Term(iTRAQ4plex); K7(iTRAQ4	1	1
[K].iWDVVEk.[A]	N-Term(iTRAQ4plex); K7(iTRAQ4	1	1
[K].sGSDEVQVGQQR.[T]	N-Term(iTRAQ4plex)	1	1
[K].kVEGTAFVIFGIQDGEQR.[I]	N-Term(iTRAQ4plex); K1(iTRAQ4	1	1
[K].IWDVVEk.[A]	K7(iTRAQ4plex)	1	1
[R].IHWESASLLR.[S]		1	1
[K].gLEVTITAR.[F]	N-Term(iTRAQ4plex)	1	1
[R].iFTVNHk.[L]	N-Term(iTRAQ4plex); K7(iTRAQ4	1	1
[R].qGALELIk.[K]	N-Term(iTRAQ4plex); K8(iTRAQ4	1	1
[K].gLEVTITAR.[F]	N-Term(iTRAQ4plex)	1	1
[R].sEFPESWLWNVEDLkEPPk.[N]	N-Term(iTRAQ4plex); K15(iTRAQ	1	1
[K].EDIPPADLSDQVPDTESETR.[I]		1	1
[R].LESEETMVLEAHDAQGDVPVTVTVHDFPG	K30(iTRAQ4plex)	1	1
[K].aAVYHHFISDGVR.[K]	N-Term(iTRAQ4plex)	1	1
[R].RHQQTVTIPPk.[S]	K11(iTRAQ4plex)	1	1
[K].gLEVTITAR.[F]	N-Term(iTRAQ4plex)	1	1
[K].kVFLDccNYITELR.[R]	N-Term(iTRAQ4plex); K1(iTRAQ4	1	1
[R].iFTVNHk.[L]	N-Term(iTRAQ4plex); K7(iTRAQ4	1	1
[K].acEPGVDYVYk.[T]	N-Term(iTRAQ4plex); C2(Carbam	1	1
[K].aAVYHHFISDGVR.[K]	N-Term(iTRAQ4plex)	1	1
[R].eVVADSVWVDVk.[D]	N-Term(iTRAQ4plex); K12(iTRAQ	1	1
[R].kVLLDGVQNPR.[A]	N-Term(iTRAQ4plex); K1(iTRAQ4	1	1
[K].eDIPPADLSDQVPDTESETR.[I]	N-Term(iTRAQ4plex)	1	1
[R].IHWESASLLR.[S]		1	1
[R].sVQLTEk.[R]	N-Term(iTRAQ4plex); K7(iTRAQ4	1	1
[K].kLVLSSEk.[T]	K1(iTRAQ4plex); K8(iTRAQ4plex)	1	1
[K].aGDFLEANYMNLQR.[S]	N-Term(iTRAQ4plex)	1	1
[K].kLVLSSEk.[T]	N-Term(iTRAQ4plex); K8(iTRAQ4	1	1
[R].aEDLVGk.[S]	N-Term(iTRAQ4plex); K7(iTRAQ4	1	1
[K].dFDFVPPVVR.[W]	N-Term(iTRAQ4plex)	1	1
[K].aAVYHHFISDGVR.[K]	N-Term(iTRAQ4plex)	1	1
[R].TELRPGETLNVNFLLR.[M]		1	1
[K].iWDVVEk.[A]	N-Term(iTRAQ4plex); K7(iTRAQ4	1	1
[R].lESEETMVLEAHDAQGDVPVTVTVHDFPG	N-Term(iTRAQ4plex); K30(iTRAQ	1	1
[K].fVTVQATFGTQVVEk.[V]	N-Term(iTRAQ4plex); K15(iTRAQ	1	1
[K].fVTVQATFGTQVVEk.[V]	N-Term(iTRAQ4plex); K15(iTRAQ	1	1
[R].ISLPESLk.[R]	K8(iTRAQ4plex)	1	1
[K].vRVELLHNPAFcSLATTk.[R]	N-Term(iTRAQ4plex); C12(Carbar	1	1
[K].ADIGcTPGSGk.[D]	C5(Carbamidomethyl); K11(iTRA	1	1

[K].vSHSEDDcLAFk.[V]	N-Term(iTRAQ4plex); C8(Carbam	1	1
[K].GQGTLSVVTMYHAk.[A]	K14(iTRAQ4plex)	1	1
[R].tVmVNIENPEGIPVk.[Q]	N-Term(iTRAQ4plex); M3(Oxidat	1	1
[K].aGDFLEANYMNLQR.[S]	N-Term(iTRAQ4plex)	1	1
[R].eGVQkEDIPPADLSDQVPDTESETR.[I]	N-Term(iTRAQ4plex); K5(iTRAQ4	1	1
[R].cAEENcFIQk.[S]	C1(Carbamidomethyl); C6(Carba	1	1
[K].rIPIEDGSGEVVLSR.[K]	N-Term(iTRAQ4plex)	1	1
[K].fVTVQATFGTQVVEk.[V]	N-Term(iTRAQ4plex); K15(iTRAQ	1	1
[K].dFDFVPPVVR.[W]	N-Term(iTRAQ4plex)	1	1
[R].lVAYYTLIGASGQR.[E]	N-Term(iTRAQ4plex)	1	1
[K].NTMILEIcTR.[Y]	C8(Carbamidomethyl)	1	1
[R].aSHLGLAR.[S]	N-Term(iTRAQ4plex)	1	1
[K].dQLTcNk.[F]	N-Term(iTRAQ4plex); C5(Carbam	1	1
[R].eALkLEEk.[K]	N-Term(iTRAQ4plex); K4(iTRAQ4	1	1
[R].fISLGEAck.[K]	N-Term(iTRAQ4plex); C8(Carbam	1	1
[K].eNEGFTVTAEGk.[G]	N-Term(iTRAQ4plex); K12(iTRAQ	1	1
[R].vELLHNPAFcSLATTk.[R]	N-Term(iTRAQ4plex); C10(Carbar	1	1
[R].eVVADSVWVDVk.[D]	N-Term(iTRAQ4plex); K12(iTRAQ	1	1
[R].tKkQELSEAEQATR.[T]	N-Term(iTRAQ4plex); K3(iTRAQ4	1	1
[K].fYYIYNEk.[G]	N-Term(iTRAQ4plex); K8(iTRAQ4	1	1
[R].tkKQELSEAEQATR.[T]	N-Term(iTRAQ4plex); K2(iTRAQ4	1	1
[K].kQELSEAEQATR.[T]	N-Term(iTRAQ4plex); K1(iTRAQ4	1	1
[K].iWDVVEk.[A]	N-Term(iTRAQ4plex); K7(iTRAQ4	1	1
[K].FYYIYNEk.[G]	K8(iTRAQ4plex)	1	1
[K].EDIPPADLSDQVPDTESETR.[I]		1	1
[K].vLLDGVQNPR.[A]	N-Term(iTRAQ4plex)	1	1
[K].gVFVLNk.[K]	N-Term(iTRAQ4plex); K7(iTRAQ4	1	1
[K].akDQLTcNk.[F]	N-Term(iTRAQ4plex); K2(iTRAQ4	1	1
[R].aEDLVGk.[S]	N-Term(iTRAQ4plex); K7(iTRAQ4	1	1
[K].SSLSVPYVIVPLk.[T]	K13(iTRAQ4plex)	1	1
[K].dYAGVFSDAGLTFTSSSGQQTAQR.[A]	N-Term(iTRAQ4plex)	1	1
[K].tGLQEVEVk.[A]	N-Term(iTRAQ4plex); K9(iTRAQ4	1	1
[R].vVLVAVDk.[G]	N-Term(iTRAQ4plex); K8(iTRAQ4	1	1
[R].vELLHNPAFcSLATTk.[R]	N-Term(iTRAQ4plex); C10(Carbar	1	1
[K].dAPDHQELNLDVSLQLPSR.[S]	N-Term(iTRAQ4plex)	1	1
[K].kQELSEAEQATR.[T]	N-Term(iTRAQ4plex); K1(iTRAQ4	1	1
[K].lMNIFLk.[D]	N-Term(iTRAQ4plex); K7(iTRAQ4	1	1
[R].aEDLVGk.[S]	N-Term(iTRAQ4plex); K7(iTRAQ4	1	1
[K].TGLQEVEVk.[A]	K9(iTRAQ4plex)	1	1
[K].kQELSEAEQATR.[T]	N-Term(iTRAQ4plex)	1	1
[R].lkGPLLNk.[F]	N-Term(iTRAQ4plex); K2(iTRAQ4	1	1
[K].kQELSEAEQATR.[T]	K1(iTRAQ4plex)	1	1
[R].qPVPGQQmTLk.[I]	N-Term(iTRAQ4plex); M8(Oxidat	1	1
[R].eGVQkEDIPPADLSDQVPDTESETR.[I]	N-Term(iTRAQ4plex); K5(iTRAQ4	1	1
[R].sYTVAIAGYALAQMGR.[L]	N-Term(iTRAQ4plex)	1	1
[R].VVLVAVDk.[G]	K8(iTRAQ4plex)	1	1
[R].qPVPGQQmTLk.[I]	N-Term(iTRAQ4plex); M8(Oxidat	1	1
[R].lESEETMVLEAHDAQGDVPVTVTVHDFPG	N-Term(iTRAQ4plex); K30(iTRAQ	1	1
[K].VSHSEDDcLAFk.[V]	C8(Carbamidomethyl); K12(iTRA	1	1
[K].gYTQQLAFR.[Q]	N-Term(iTRAQ4plex)	1	1
[R].iSLPESLk.[R]	N-Term(iTRAQ4plex); K8(iTRAQ4	1	1
[R].sGIPIVTSPYQIHFTk.[T]	N-Term(iTRAQ4plex); K16(iTRAQ	1	1

[K].VHQYFNVELIQPGAVk.[V]	K16(iTRAQ4plex)	1	1
[K].vLLDGVQNPR.[A]	N-Term(iTRAQ4plex)	1	1
[K].LMNIFLk.[D]	K7(iTRAQ4plex)	1	1
[K].vFLDccNYITELR.[R]	N-Term(iTRAQ4plex); C5(Carbam	1	1
[K].vLLDGVQNPR.[A]	N-Term(iTRAQ4plex)	1	1
[R].sYTVAIAGYALAQMGR.[L]	N-Term(iTRAQ4plex)	1	1
[R].yYTYLImNk.[G]	N-Term(iTRAQ4plex); M7(Oxidat	1	1
[K].ADIGcTPGSGk.[D]	C5(Carbamidomethyl); K11(iTRA	1	1
[R].eGVQkEDIPPADLSDQVPDTESETR.[I]	N-Term(iTRAQ4plex); K5(iTRAQ4	1	1
[K].aAVYHHFISDGVR.[K]	N-Term(iTRAQ4plex)	1	1
[R].iHWESASLLR.[S]	N-Term(iTRAQ4plex)	1	1
[K].lmNIFLk.[D]	N-Term(iTRAQ4plex); M2(Oxidat	1	1
[K].sDDkVTLEER.[L]	N-Term(iTRAQ4plex); K4(iTRAQ4	1	1
[K].kVEGTAFVIFGIQDGEQR.[I]	N-Term(iTRAQ4plex); K1(iTRAQ4	1	1
[R].sVQLTEk.[R]	N-Term(iTRAQ4plex); K7(iTRAQ4	1	1
[K].kQELSEAEQATR.[T]	K1(iTRAQ4plex)	1	1
[K].kQELSEAEQATR.[T]	N-Term(iTRAQ4plex)	1	1
[R].aYYENSPQQVFSTEFEVk.[E]	N-Term(iTRAQ4plex); K18(iTRAQ	1	1
[R].eVVADSVWVDVk.[D]	N-Term(iTRAQ4plex); K12(iTRAQ	1	1
[R].iFTVNHk.[L]	N-Term(iTRAQ4plex); K7(iTRAQ4	1	1
[K].fVTVQATFGTQVVEk.[V]	N-Term(iTRAQ4plex); K15(iTRAQ	1	1
[K].fYYIYNEk.[G]	N-Term(iTRAQ4plex); K8(iTRAQ4	1	1
[K].AcEPGVDYVYk.[T]	C2(Carbamidomethyl); K11(iTRA	1	1
[R].RHQQTVTIPPk.[S]	K11(iTRAQ4plex)	1	1
[K].gQGTLSVVTmYHAk.[A]	N-Term(iTRAQ4plex); M10(Oxida	1	1
[R].aEDLVGk.[S]	N-Term(iTRAQ4plex); K7(iTRAQ4	1	1
[K].vEGTAFVIFGIQDGEQR.[I]	N-Term(iTRAQ4plex)	1	1
[R].yYTYLImNk.[G]	N-Term(iTRAQ4plex); M7(Oxidat	1	1
[K].EDIPPADLSDQVPDTESETR.[I]		1	1
[R].FYHPEkEDGk.[L]	K6(iTRAQ4plex); K10(iTRAQ4ple	1	1
[K].VSHSEDDcLAFk.[V]	C8(Carbamidomethyl); K12(iTRA	1	1
[K].vLLDGVQNPR.[A]	N-Term(iTRAQ4plex)	1	1
[R].iLLQGTPVAQMTEDAVDAER.[L]	N-Term(iTRAQ4plex)	1	1
[K].aDIGcTPGSGk.[D]	N-Term(iTRAQ4plex); C5(Carbam	1	1
[K].kGYTQQLAFR.[Q]	N-Term(iTRAQ4plex); K1(iTRAQ4	1	1
[K].vEGTAFVIFGIQDGEQR.[I]	N-Term(iTRAQ4plex)	1	1
[K].vSHSEDDcLAFk.[V]	N-Term(iTRAQ4plex); C8(Carbam	1	1
[R].iFTVNHk.[L]	N-Term(iTRAQ4plex); K7(iTRAQ4	1	1
[K].EDIPPADLSDQVPDTESETR.[I]		1	1
[K].vLLDGVQNPR.[A]	N-Term(iTRAQ4plex)	1	1
[R].aEDLVGk.[S]	N-Term(iTRAQ4plex); K7(iTRAQ4	1	1
[R].qPVPGQQMTLk.[I]	N-Term(iTRAQ4plex); K11(iTRAQ	1	1
[R].lESEETMVLEAHDAQGDVPVTVTVHDFPG	N-Term(iTRAQ4plex); K30(iTRAQ	1	1
[K].dFDFVPPVVR.[W]	N-Term(iTRAQ4plex)	1	1
[K].vSHSEDDcLAFk.[V]	N-Term(iTRAQ4plex); C8(Carbam	1	1
[K].fYYIYNEk.[G]	N-Term(iTRAQ4plex); K8(iTRAQ4	1	1
[K].dQLTcNk.[F]	N-Term(iTRAQ4plex); C5(Carbam	1	1
[K].sLYVSATVILHSGSDMVQAER.[S]	N-Term(iTRAQ4plex)	1	1
[R].aSHLGLAR.[S]	N-Term(iTRAQ4plex)	1	1
[R].tVmVNIENPEGIPVk.[Q]	N-Term(iTRAQ4plex); M3(Oxidat	1	1
[K].sDDkVTLEER.[L]	N-Term(iTRAQ4plex); K4(iTRAQ4	1	1
[K].TIYTPGSTVLYR.[I]		1	1

[R].kSLkVVPEGIR.[M]	N-Term(iTRAQ4plex); K4(iTRAQ4	1	1
[R].kSLkVVPEGIR.[M]	K1(iTRAQ4plex); K4(iTRAQ4plex)	1	1
[K].dYAGVFSDAGLTFTSSSGQQTAQR.[A]	N-Term(iTRAQ4plex)	1	1
[K].kVEGTAFVIFGIQDGEQR.[I]	N-Term(iTRAQ4plex); K1(iTRAQ4	1	1
[K].ENEGFTVTAEGk.[G]	K12(iTRAQ4plex)	1	1
[K].kVEGTAFVIFGIQDGEQR.[I]	N-Term(iTRAQ4plex); K1(iTRAQ4	1	1
[R].tFISPIk.[C]	N-Term(iTRAQ4plex); K7(iTRAQ4	1	1
[K].DIcEEQVNSLPGSITk.[A]	C3(Carbamidomethyl); K16(iTRA	1	1
[R].TELRPGETLNVNFLLR.[M]		1	1
[K].iEGDHGAR.[V]	N-Term(iTRAQ4plex)	1	1
[R].RHQQTVTIPPk.[S]	K11(iTRAQ4plex)	1	1
[K].EDIPPADLSDQVPDTESETR.[I]		1	1
[R].yYTYLImNk.[G]	N-Term(iTRAQ4plex); M7(Oxidat	1	1
[R].sGIPIVTSPYQIHFTk.[T]	N-Term(iTRAQ4plex); K16(iTRAQ	1	1
[R].iLLQGTPVAQmTEDAVDAER.[L]	N-Term(iTRAQ4plex); M11(Oxida	1	1
[K].vHQYFNVELIQPGAVk.[V]	N-Term(iTRAQ4plex); K16(iTRAQ	1	1
[K].vYAYYNLEEScTR.[F]	N-Term(iTRAQ4plex); C11(Carbar	1	1
[R].qGALELIk.[K]	N-Term(iTRAQ4plex); K8(iTRAQ4	1	1
[K].fVTVQATFGTQVVEk.[V]	N-Term(iTRAQ4plex); K15(iTRAQ	1	1
[K].kQELSEAEQATR.[T]	N-Term(iTRAQ4plex)	1	1
[R].LkGPLLNk.[F]	K2(iTRAQ4plex); K8(iTRAQ4plex)	1	1
[R].lkGPLLNk.[F]	N-Term(iTRAQ4plex); K2(iTRAQ4	1	1
[R].qPVPGQQMTLk.[I]	N-Term(iTRAQ4plex); K11(iTRAQ	1	1
[K].DFDFVPPVVR.[W]		1	1
[R].sEFPESWLWNVEDLkEPPk.[N]	N-Term(iTRAQ4plex); K15(iTRAQ	1	1
[K].vSHSEDDcLAFk.[V]	N-Term(iTRAQ4plex); C8(Carbam	1	1
[R].sVQLTEk.[R]	N-Term(iTRAQ4plex); K7(iTRAQ4	1	1
[K].lVLSSEk.[TL]	N-Term(iTRAQ4plex); K7(iTRAQ4	1	2
[K].VTIkPAPETEk.[R]	K4(iTRAQ4plex); K11(iTRAQ4ple	1	1
[R].aEDLVGk.[S]	N-Term(iTRAQ4plex); K7(iTRAQ4	1	1
[R].hQQTVTIPPk.[S]	N-Term(iTRAQ4plex); K10(iTRAQ	1	1
[R].sYTVAIAGYALAQMGR.[L]	N-Term(iTRAQ4plex)	1	1
[K].dQLTcNk.[F]	N-Term(iTRAQ4plex); C5(Carbam	1	1
[K].rPQDAkNTMILEIcTR.[Y]	N-Term(iTRAQ4plex); K6(iTRAQ4	1	1
[K].TIYTPGSTVLYR.[I]		1	1
[K].acEPGVDYVYk.[T]	N-Term(iTRAQ4plex); C2(Carbam	1	1
[K].ADIGcTPGSGk.[D]	C5(Carbamidomethyl); K11(iTRA	1	1
[R].YYTYLIMNk.[G]	K9(iTRAQ4plex)	1	1
[K].LLSGGNTLHLVSTTk.[T]	K15(iTRAQ4plex)	1	1
[R].eELcTMFIR.[E]	N-Term(iTRAQ4plex); C4(Carbam	1	1
[R].NLQNNAEWVYQGAIR.[Q]		1	1
[K].vLVDHFGYTk.[D]	N-Term(iTRAQ4plex); K10(iTRAQ	1	1
[K].LLSGGNTLHLVSTTk.[T]	K15(iTRAQ4plex)	1	1
[K].tEVIPPLIENR.[Q]	N-Term(iTRAQ4plex)	1	1
[K].tEVIPPLIENR.[Q]	N-Term(iTRAQ4plex)	1	1
[K].tEVIPPLIENR.[Q]	N-Term(iTRAQ4plex)	1	1
[K].tEVIPPLIENR.[Q]	N-Term(iTRAQ4plex)	1	1
[K].tEVIPPLIENR.[Q]	N-Term(iTRAQ4plex)	1	1
[K].dNVFDGLVR.[V]	N-Term(iTRAQ4plex)	1	1
[K].fPEVDVLTk.[Y]	N-Term(iTRAQ4plex); K9(iTRAQ4	1	1
[K].ESQLPTVMDFR.[K]		1	1
[K].eSQLPTVMDFR.[K]	N-Term(iTRAQ4plex)	1	1

[K].eSQLPTVMDFR.[K]	N-Term(iTRAQ4plex)	1	1
[K].eSQLPTVMDFR.[K]	N-Term(iTRAQ4plex)	1	1
[R].tEHGSEMLFFGNAIEGk.[S]	N-Term(iTRAQ4plex); K17(iTRAQ	1	1
[R].tEHGSEMLFFGNAIEGk.[S]	N-Term(iTRAQ4plex); K17(iTRAQ	1	1
[K].lDFSSQADLR.[N]	N-Term(iTRAQ4plex)	1	1
[K].eSQLPTVmDFR.[K]	N-Term(iTRAQ4plex); M8(Oxidat	1	1
[R].aTLYALSHAVNNYHk.[T]	N-Term(iTRAQ4plex); K15(iTRAQ	1	1
[K].qVFLYPEk.[D]	N-Term(iTRAQ4plex); K8(iTRAQ4	1	1
[R].nNALDFVTk.[S]	N-Term(iTRAQ4plex); K9(iTRAQ4	1	1
[R].nNALDFVTk.[S]	N-Term(iTRAQ4plex); K9(iTRAQ4	1	1
[R].aTLYALSHAVNNYHk.[T]	N-Term(iTRAQ4plex); K15(iTRAQ	1	1
[R].aTLYALSHAVNNYHk.[T]	N-Term(iTRAQ4plex); K15(iTRAQ	1	1
[K].hINIDQFVR.[K]	N-Term(iTRAQ4plex)	1	1
[K].dFSLWEk.[T]	N-Term(iTRAQ4plex); K7(iTRAQ4	1	1
[K].IAELSATAQEIIk.[S]	K13(iTRAQ4plex)	1	1
[K].nTLELSNGVIVk.[I]	N-Term(iTRAQ4plex); K12(iTRAQ	1	1
[K].sPAFTDLHLR.[Y]	N-Term(iTRAQ4plex)	1	1
[K].aTGVLYDYVNk.[Y]	N-Term(iTRAQ4plex); K11(iTRAQ	1	1
[K].aTGVLYDYVNk.[Y]	N-Term(iTRAQ4plex); K11(iTRAQ	1	1
[R].vSTAFVYTk.[N]	N-Term(iTRAQ4plex); K9(iTRAQ4	1	1
[K].nSEEFAAAMSR.[Y]	N-Term(iTRAQ4plex)	1	1
[K].kIISDYHQQFR.[Y]	N-Term(iTRAQ4plex); K1(iTRAQ4	1	1
[K].nSEEFAAAMSR.[Y]	N-Term(iTRAQ4plex)	1	1
[R].kYTYNYEAESSSGVPGTADSR.[S]	N-Term(iTRAQ4plex); K1(iTRAQ4	1	1
[K].iISDYHQQFR.[Y]	N-Term(iTRAQ4plex)	1	1
[K].lHVAGNLk.[G]	N-Term(iTRAQ4plex); K8(iTRAQ4	1	1
[R].sEYQADYESLR.[F]	N-Term(iTRAQ4plex)	1	1
[R].IYSLWEHSTk.[N]	K10(iTRAQ4plex)	1	1
[K].cVQSTkPSLMIQk.[A]	N-Term(iTRAQ4plex); C1(Carbam	1	1
[K].cVQSTkPSLMIQk.[A]	N-Term(iTRAQ4plex); C1(Carbam	1	1
[K].iISDYHQQFR.[Y]	N-Term(iTRAQ4plex)	1	1
[K].iISDYHQQFR.[Y]	N-Term(iTRAQ4plex)	1	1
[K].iISDYHQQFR.[Y]	N-Term(iTRAQ4plex)	1	1
[K].lVELAHQYk.[L]	N-Term(iTRAQ4plex); K9(iTRAQ4	1	1
[K].LATALSLSNk.[F]	K10(iTRAQ4plex)	1	1
[K].iISDYHQQFR.[Y]	N-Term(iTRAQ4plex)	1	1
[R].kYTYNYEAESSSGVPGTADSR.[S]	K1(iTRAQ4plex)	1	1
[R].kYTYNYEAESSSGVPGTADSR.[S]	N-Term(iTRAQ4plex)	1	1
[K].yHWEHTGLTLR.[E]	N-Term(iTRAQ4plex)	1	1
[K].sVGFHLPSR.[E]	N-Term(iTRAQ4plex)	1	1
[K].sVGFHLPSR.[E]	N-Term(iTRAQ4plex)	1	1
[R].yEVDQQIQVLMDk.[L]	N-Term(iTRAQ4plex); K13(iTRAQ	1	1
[R].yEVDQQIQVLMDk.[L]	N-Term(iTRAQ4plex); K13(iTRAQ	1	1
[R].yEDGTLSLTSTSDLQSGIIk.[N]	N-Term(iTRAQ4plex); K20(iTRAQ	1	1
[K].IDDIWNLEVk.[E]	K10(iTRAQ4plex)	1	1
[K].yHWEHTGLTLR.[E]	N-Term(iTRAQ4plex)	1	1
[K].yHWEHTGLTLR.[E]	N-Term(iTRAQ4plex)	1	1
[K].dkDQEVLLQTFLDDASPGDkR.[L]	N-Term(iTRAQ4plex); K2(iTRAQ4	1	1
[K].dkDQEVLLQTFLDDASPGDkR.[L]	N-Term(iTRAQ4plex); K2(iTRAQ4	1	1
[K].dkDQEVLLQTFLDDASPGDkR.[L]	N-Term(iTRAQ4plex); K2(iTRAQ4	1	1
[K].dkDQEVLLQTFLDDASPGDkR.[L]	N-Term(iTRAQ4plex); K2(iTRAQ4	1	1
[K].lQDFSDQLSDYYEk.[F]	N-Term(iTRAQ4plex); K14(iTRAQ	1	1

[R].iTENDIQIALDDAk.[I]	N-Term(iTRAQ4plex); K14(iTRAQ4	1	1
[K].lQDFSDQLSDYYEk.[F]	N-Term(iTRAQ4plex); K14(iTRAQ4	1	1
[K].lQDFSDQLSDYYEk.[F]	N-Term(iTRAQ4plex); K14(iTRAQ4	1	1
[R].iTENDIQIALDDAk.[I]	N-Term(iTRAQ4plex); K14(iTRAQ4	1	1
[R].iTENDIQIALDDAk.[I]	N-Term(iTRAQ4plex); K14(iTRAQ4	1	1
[R].iTENDIQIALDDAk.[I]	N-Term(iTRAQ4plex); K14(iTRAQ4	1	1
[K].dkDQEVLLQTFLDDASPGDk.[R]	N-Term(iTRAQ4plex); K2(iTRAQ4	1	1
[K].dkDQEVLLQTFLDDASPGDk.[R]	N-Term(iTRAQ4plex); K2(iTRAQ4	1	1
[K].dkDQEVLLQTFLDDASPGDk.[R]	N-Term(iTRAQ4plex); K2(iTRAQ4	1	1
[K].dkDQEVLLQTFLDDASPGDk.[R]	N-Term(iTRAQ4plex); K2(iTRAQ4	1	1
[K].dkDQEVLLQTFLDDASPGDk.[R]	N-Term(iTRAQ4plex); K2(iTRAQ4	1	1
[K].dkDQEVLLQTFLDDASPGDk.[R]	N-Term(iTRAQ4plex); K2(iTRAQ4	1	1
[K].dkDQEVLLQTFLDDASPGDk.[R]	N-Term(iTRAQ4plex); K2(iTRAQ4	1	1
[K].dkDQEVLLQTFLDDASPGDk.[R]	N-Term(iTRAQ4plex); K2(iTRAQ4	1	1
[K].dkDQEVLLQTFLDDASPGDk.[R]	N-Term(iTRAQ4plex); K2(iTRAQ4	1	1
[K].dkDQEVLLQTFLDDASPGDk.[R]	N-Term(iTRAQ4plex); K2(iTRAQ4	1	1
[K].dEPTYILNIk.[R]	N-Term(iTRAQ4plex); K10(iTRAQ4	1	1
[R].eYSGTIASEANTYLNSk.[S]	N-Term(iTRAQ4plex); K17(iTRAQ4	1	1
[K].FPEVDVLTk.[Y]	K9(iTRAQ4plex)	1	1
[K].mTSNFPVDLSDYPk.[S]	N-Term(iTRAQ4plex); M1(Oxidat	1	1
[K].lALWGEHTGQLYSk.[F]	N-Term(iTRAQ4plex); K14(iTRAQ4	1	1
[K].lTISEQNIQR.[A]	N-Term(iTRAQ4plex)	1	1
[K].lTISEQNIQR.[A]	N-Term(iTRAQ4plex)	1	1
[K].lATALSLSNk.[F]	N-Term(iTRAQ4plex); K10(iTRAQ4	1	1
[K].lATALSLSNk.[F]	N-Term(iTRAQ4plex); K10(iTRAQ4	1	1
[K].lATALSLSNk.[F]	N-Term(iTRAQ4plex); K10(iTRAQ4	1	1
[K].iNNQLTLDSNTk.[Y]	N-Term(iTRAQ4plex); K12(iTRAQ4	1	1
[K].lATALSLSNk.[F]	N-Term(iTRAQ4plex); K10(iTRAQ4	1	1
[K].skPTVSSSMEFk.[Y]	N-Term(iTRAQ4plex); K2(iTRAQ4	1	1
[K].skPTVSSSMEFk.[Y]	N-Term(iTRAQ4plex); K2(iTRAQ4	1	1
[K].hIYAISSAALSASYk.[A]	N-Term(iTRAQ4plex); K15(iTRAQ4	1	1
[K].mGLAFESTk.[S]	N-Term(iTRAQ4plex); K9(iTRAQ4	1	1
[K].mGLAFESTk.[S]	N-Term(iTRAQ4plex); K9(iTRAQ4	1	1
[R].iGQDGISTSATTNLk.[C]	N-Term(iTRAQ4plex); K15(iTRAQ4	1	1
[K].aHLDIAGSLEGHLR.[F]	N-Term(iTRAQ4plex)	1	1
[K].qGFFPDSVNk.[A]	N-Term(iTRAQ4plex); K10(iTRAQ4	1	1
[K].aHLDIAGSLEGHLR.[F]	N-Term(iTRAQ4plex)	1	1
[K].SPAFTDLHLR.[Y]		1	1
[K].SPAFTDLHLR.[Y]		1	1
[R].kHVAEAIck.[E]	N-Term(iTRAQ4plex); K1(iTRAQ4	1	1
[K].dQEVLLQTFLDDASPGDk.[R]	N-Term(iTRAQ4plex); K18(iTRAQ4	1	1
[K].lLSGGNTLHLVSTTk.[T]	N-Term(iTRAQ4plex); K15(iTRAQ4	1	1
[K].IISDYHQQFR.[Y]		1	1
[K].lLSGGNTLHLVSTTk.[T]	N-Term(iTRAQ4plex); K15(iTRAQ4	1	1
[K].nkYGMVAQVTQTLk.[L]	N-Term(iTRAQ4plex); K2(iTRAQ4	1	1
[K].fVTQAEGAk.[Q]	N-Term(iTRAQ4plex); K9(iTRAQ4	1	1
[R].fQFPGkPGIYTR.[E]	N-Term(iTRAQ4plex); K6(iTRAQ4	1	1
[K].lLSGGNTLHLVSTTk.[T]	N-Term(iTRAQ4plex); K15(iTRAQ4	1	1
[K].kLTISEQNIQR.[A]	N-Term(iTRAQ4plex); K1(iTRAQ4	1	1
[K].kLTISEQNIQR.[A]	N-Term(iTRAQ4plex); K1(iTRAQ4	1	1
[K].kLTISEQNIQR.[A]	N-Term(iTRAQ4plex); K1(iTRAQ4	1	1
[K].lLSGGNTLHLVSTTk.[T]	N-Term(iTRAQ4plex); K15(iTRAQ4	1	1

[R].kSISAALEHk.[V]	N-Term(iTRAQ4plex); K1(iTRAQ4	1	1
[R].fQFPGkPGIYTR.[E]	N-Term(iTRAQ4plex); K6(iTRAQ4	1	1
[R].fQFPGkPGIYTR.[E]	N-Term(iTRAQ4plex); K6(iTRAQ4	1	1
[K].lLSGGNTLHLVSTTk.[T]	N-Term(iTRAQ4plex); K15(iTRAQ	1	1
[R].TSSFALNLPTLPEVk.[F]	K15(iTRAQ4plex)	1	1
[K].hINIDQFVR.[K]	N-Term(iTRAQ4plex)	1	1
[K].hINIDQFVR.[K]	N-Term(iTRAQ4plex)	1	1
[K].hINIDQFVR.[K]	N-Term(iTRAQ4plex)	1	1
[K].yHWEHTGLTLR.[E]	N-Term(iTRAQ4plex)	1	1
[K].yHWEHTGLTLR.[E]	N-Term(iTRAQ4plex)	1	1
[K].yHWEHTGLTLR.[E]	N-Term(iTRAQ4plex)	1	1
[R].tLADLTLLDSPIk.[V]	N-Term(iTRAQ4plex); K13(iTRAQ	1	1
[K].yHWEHTGLTLR.[E]	N-Term(iTRAQ4plex)	1	1
[K].LLSGGNTLHLVSTTk.[T]	K15(iTRAQ4plex)	1	1
[R].sEILAHWSPAk.[L]	N-Term(iTRAQ4plex); K11(iTRAQ	1	1
[K].vLVDHFGYTk.[D]	N-Term(iTRAQ4plex); K10(iTRAQ	1	1
[K].yHWEHTGLTLR.[E]	N-Term(iTRAQ4plex)	1	1
[K].yHWEHTGLTLR.[E]	N-Term(iTRAQ4plex)	1	1
[K].yHWEHTGLTLR.[E]	N-Term(iTRAQ4plex)	1	1
[K].lLLQmDSSATAYGSTVSkR.[V]	N-Term(iTRAQ4plex); M5(Oxidat	1	1
[K].wNFYYSPQSSPDkk.[L]	N-Term(iTRAQ4plex); K13(iTRAQ	1	1
[K].aHLDIAGSLEGHLR.[F]	N-Term(iTRAQ4plex)	1	1
[K].wNFYYSPQSSPDkk.[L]	N-Term(iTRAQ4plex); K13(iTRAQ	1	1
[K].aHLDIAGSLEGHLR.[F]	N-Term(iTRAQ4plex)	1	1
[K].aHLDIAGSLEGHLR.[F]	N-Term(iTRAQ4plex)	1	1
[K].aHLDIAGSLEGHLR.[F]	N-Term(iTRAQ4plex)	1	1
[K].aHLDIAGSLEGHLR.[F]	N-Term(iTRAQ4plex)	1	1
[K].lPQQANDYLNSFNWER.[Q]	N-Term(iTRAQ4plex)	1	1
[R].gIISALLVPPETEEAk.[Q]	N-Term(iTRAQ4plex); K16(iTRAQ	1	1
[R].gIISALLVPPETEEAk.[Q]	N-Term(iTRAQ4plex); K16(iTRAQ	1	1
[R].gIISALLVPPETEEAk.[Q]	N-Term(iTRAQ4plex); K16(iTRAQ	1	1
[K].wNFYYSPQSSPDkk.[L]	N-Term(iTRAQ4plex); K13(iTRAQ	1	1
[K].aHLDIAGSLEGHLR.[F]	N-Term(iTRAQ4plex)	1	1
[K].wNFYYSPQSSPDkk.[L]	N-Term(iTRAQ4plex); K13(iTRAQ	1	1
[R].iYSLWEHSTk.[N]	N-Term(iTRAQ4plex); K10(iTRAQ	1	1
[K].yGmVAQVTQTLk.[L]	N-Term(iTRAQ4plex); M3(Oxidat	1	1
[K].yGmVAQVTQTLk.[L]	N-Term(iTRAQ4plex); M3(Oxidat	1	1
[R].iYSLWEHSTk.[N]	N-Term(iTRAQ4plex); K10(iTRAQ	1	1
[R].iYSLWEHSTk.[N]	N-Term(iTRAQ4plex); K10(iTRAQ	1	1
[R].iYSLWEHSTk.[N]	N-Term(iTRAQ4plex); K10(iTRAQ	1	1
[K].lATALSLSNk.[F]	N-Term(iTRAQ4plex); K10(iTRAQ	1	1
[K].kLQSTTVMNPYMk.[L]	N-Term(iTRAQ4plex); K1(iTRAQ4	1	1
[K].GFEPTLEALFGk.[Q]	K12(iTRAQ4plex)	1	1
[K].aQNLYQELLTQEGQASFQGLk.[D]	N-Term(iTRAQ4plex); K21(iTRAQ	1	1
[K].kIISDYHQQFR.[Y]	N-Term(iTRAQ4plex); K1(iTRAQ4	1	1
[K].rHIQNIDIQHLAGk.[L]	N-Term(iTRAQ4plex); K14(iTRAQ	1	1
[K].iISDYHQQFR.[Y]	N-Term(iTRAQ4plex)	1	1
[K].iISDYHQQFR.[Y]	N-Term(iTRAQ4plex)	1	1
[K].sVGFHLPSR.[E]	N-Term(iTRAQ4plex)	1	1
[K].sVGFHLPSR.[E]	N-Term(iTRAQ4plex)	1	1
[K].vSALLTPAEQTGTWk.[L]	N-Term(iTRAQ4plex); K15(iTRAQ	1	1
[K].vSALLTPAEQTGTWk.[L]	N-Term(iTRAQ4plex); K15(iTRAQ	1	1

[K].eQHLFLPFSYk.[N]	N-Term(iTRAQ4plex); K11(iTRAQ4	1	1
[K].iVQILPWEQNEQVk.[N]	N-Term(iTRAQ4plex); K14(iTRAQ4	1	1
[R].qTIIVVLENVQR.[N]	N-Term(iTRAQ4plex)	1	1
[R].qTIIVVLENVQR.[N]	N-Term(iTRAQ4plex)	1	1
[R].qTIIVVLENVQR.[N]	N-Term(iTRAQ4plex)	1	1
[K].iVQILPWEQNEQVk.[N]	N-Term(iTRAQ4plex); K14(iTRAQ4	1	1
[K].iVQILPWEQNEQVk.[N]	N-Term(iTRAQ4plex); K14(iTRAQ4	1	1
[K].lLLQMDSSATAYGSTVSk.[R]	N-Term(iTRAQ4plex); K18(iTRAQ4	1	1
[K].lLLQMDSSATAYGSTVSk.[R]	N-Term(iTRAQ4plex); K18(iTRAQ4	1	1
[R].IPSVQINFk.[D]	K9(iTRAQ4plex)	1	1
[K].iTEVALMGHLScDTk.[E]	N-Term(iTRAQ4plex); C12(Carbar	1	1
[R].eVGTVLSQVYSk.[V]	N-Term(iTRAQ4plex); K12(iTRAQ4	1	1
[K].yGMVAQVTQTLk.[L]	N-Term(iTRAQ4plex); K12(iTRAQ4	1	1
[K].yGMVAQVTQTLk.[L]	N-Term(iTRAQ4plex); K12(iTRAQ4	1	1
[K].yGMVAQVTQTLk.[L]	N-Term(iTRAQ4plex); K12(iTRAQ4	1	1
[K].yGMVAQVTQTLk.[L]	N-Term(iTRAQ4plex); K12(iTRAQ4	1	1
[K].sVSLPSLDPASAk.[I]	N-Term(iTRAQ4plex); K13(iTRAQ4	1	1
[K].sVSLPSLDPASAk.[I]	N-Term(iTRAQ4plex); K13(iTRAQ4	1	1
[R].eVGTVLSQVYSk.[V]	N-Term(iTRAQ4plex); K12(iTRAQ4	1	1
[K].sVSLPSLDPASAk.[I]	N-Term(iTRAQ4plex); K13(iTRAQ4	1	1
[K].sVSLPSLDPASAk.[I]	N-Term(iTRAQ4plex); K13(iTRAQ4	1	1
[R].eVGTVLSQVYSk.[V]	N-Term(iTRAQ4plex); K12(iTRAQ4	1	1
[K].aTGVLYDYVNk.[Y]	N-Term(iTRAQ4plex); K11(iTRAQ4	1	1
[K].nIILPVYDk.[S]	N-Term(iTRAQ4plex); K9(iTRAQ4	1	1
[R].vIGNmGQTMEQLTPELk.[S]	N-Term(iTRAQ4plex); M5(Oxidat	1	1
[K].nIILPVYDk.[S]	N-Term(iTRAQ4plex); K9(iTRAQ4	1	1
[R].tIDQmLNSELQWPVPDIYLR.[D]	N-Term(iTRAQ4plex); M5(Oxidat	1	1
[R].nLQNNAEWVYQGAIR.[Q]	N-Term(iTRAQ4plex)	1	1
[R].nLQNNAEWVYQGAIR.[Q]	N-Term(iTRAQ4plex)	1	1
[K].wNFYYSPQSSPDk.[K]	N-Term(iTRAQ4plex); K13(iTRAQ4	1	1
[K].wNFYYSPQSSPDk.[K]	N-Term(iTRAQ4plex); K13(iTRAQ4	1	1
[R].nLQNNAEWVYQGAIR.[Q]	N-Term(iTRAQ4plex)	1	1
[K].wNFYYSPQSSPDk.[K]	N-Term(iTRAQ4plex); K13(iTRAQ4	1	1
[R].sEILAHWSPAk.[L]	N-Term(iTRAQ4plex); K11(iTRAQ4	1	1
[K].vLVDHFGYTk.[D]	N-Term(iTRAQ4plex); K10(iTRAQ4	1	1
[K].sPAFTDLHLR.[Y]	N-Term(iTRAQ4plex)	1	1
[K].sPAFTDLHLR.[Y]	N-Term(iTRAQ4plex)	1	1
[K].sPAFTDLHLR.[Y]	N-Term(iTRAQ4plex)	1	1
[K].sPAFTDLHLR.[Y]	N-Term(iTRAQ4plex)	1	1
[R].vSTAFVYTk.[N]	N-Term(iTRAQ4plex); K9(iTRAQ4	1	1
[K].mGLAFESTk.[S]	N-Term(iTRAQ4plex); K9(iTRAQ4	1	1
[K].lDVTTSIGR.[R]	N-Term(iTRAQ4plex)	1	1
[R].hIQNIDIQHLAGk.[L]	N-Term(iTRAQ4plex); K13(iTRAQ4	1	1
[R].hIQNIDIQHLAGk.[L]	N-Term(iTRAQ4plex); K13(iTRAQ4	1	1
[K].lDVTTSIGR.[R]	N-Term(iTRAQ4plex)	1	1
[R].hIQNIDIQHLAGk.[L]	N-Term(iTRAQ4plex); K13(iTRAQ4	1	1
[K].IISDYHQQFR.[Y]		1	1
[R].hIQNIDIQHLAGk.[L]	N-Term(iTRAQ4plex); K13(iTRAQ4	1	1
[R].sEILAHWSPAk.[L]	N-Term(iTRAQ4plex); K11(iTRAQ4	1	1
[K].vLVDHFGYTk.[D]	N-Term(iTRAQ4plex); K10(iTRAQ4	1	1
[K].aHLDIAGSLEGHLR.[F]	N-Term(iTRAQ4plex)	1	1
[K].aHLDIAGSLEGHLR.[F]	N-Term(iTRAQ4plex)	1	1

[K].lQSTTVMNPYMk.[L]	N-Term(iTRAQ4plex); K12(iTRAQ	1	1
[R].SPSQADINk.[I]	K9(iTRAQ4plex)	1	1
[K].yGmVAQVTQTLk.[L]	N-Term(iTRAQ4plex); M3(Oxidat	1	1
[R].lTLDIQNk.[K]	N-Term(iTRAQ4plex); K8(iTRAQ4	1	1
[K].sPAFTDLHLR.[Y]	N-Term(iTRAQ4plex)	1	1
[K].yGmVAQVTQTLk.[L]	N-Term(iTRAQ4plex); M3(Oxidat	1	1
[K].aHLDIAGSLEGHLR.[F]	N-Term(iTRAQ4plex)	1	1
[K].iEGNLIFDPNNYLPk.[E]	N-Term(iTRAQ4plex); K15(iTRAQ	1	1
[K].iEGNLIFDPNNYLPk.[E]	N-Term(iTRAQ4plex); K15(iTRAQ	1	1
[R].gIISALLVPPETEEAk.[Q]	N-Term(iTRAQ4plex); K16(iTRAQ	1	1
[R].gIISALLVPPETEEAk.[Q]	N-Term(iTRAQ4plex); K16(iTRAQ	1	1
[K].nLTDFAEQYSIQDWAk.[R]	N-Term(iTRAQ4plex); K16(iTRAQ	1	1
[R].lELELRPTGEIEQYSVSATYELQR.[E]	N-Term(iTRAQ4plex)	1	1
[K].aQNLYQELLTQEGQASFQGLk.[D]	N-Term(iTRAQ4plex); K21(iTRAQ	1	1
[K].iDDIWNLEVk.[E]	N-Term(iTRAQ4plex); K10(iTRAQ	1	1
[K].iDDIWNLEVk.[E]	N-Term(iTRAQ4plex); K10(iTRAQ	1	1
[K].iDDIWNLEVk.[E]	N-Term(iTRAQ4plex); K10(iTRAQ	1	1
[K].qVFLYPEkDEPTYILNIk.[R]	N-Term(iTRAQ4plex); K8(iTRAQ4	1	1
[R].vIGNMGQTMEQLTPELk.[S]	N-Term(iTRAQ4plex); K17(iTRAQ	1	1
[K].qVFLYPEkDEPTYILNIk.[R]	N-Term(iTRAQ4plex); K8(iTRAQ4	1	1
[K].qVFLYPEkDEPTYILNIk.[R]	N-Term(iTRAQ4plex); K8(iTRAQ4	1	1
[K].qVFLYPEkDEPTYILNIk.[R]	N-Term(iTRAQ4plex); K8(iTRAQ4	1	1
[K].iDDIWNLEVk.[E]	N-Term(iTRAQ4plex); K10(iTRAQ	1	1
[K].iDDIWNLEVk.[E]	N-Term(iTRAQ4plex); K10(iTRAQ	1	1
[R].lELELRPTGEIEQYSVSATYELQR.[E]	N-Term(iTRAQ4plex)	1	1
[K].iDDIWNLEVkENFAGEATLQR.[I]	N-Term(iTRAQ4plex); K10(iTRAQ	1	1
[K].aQNLYQELLTQEGQASFQGLk.[D]	N-Term(iTRAQ4plex); K21(iTRAQ	1	1
[K].VNWEEEAASGLLTSLk.[D]	K16(iTRAQ4plex)	1	1
[K].VNWEEEAASGLLTSLk.[D]	K16(iTRAQ4plex)	1	1
[K].vNWEEEAASGLLTSLk.[D]	N-Term(iTRAQ4plex); K16(iTRAQ	1	1
[R].eEYFDPSIVGWTVk.[Y]	N-Term(iTRAQ4plex); K14(iTRAQ	1	1
[K].aQNLYQELLTQEGQASFQGLk.[D]	N-Term(iTRAQ4plex); K21(iTRAQ	1	1
[R].lELELRPTGEIEQYSVSATYELQR.[E]	N-Term(iTRAQ4plex)	1	1
[K].nLTDFAEQYSIQDWAk.[R]	N-Term(iTRAQ4plex); K16(iTRAQ	1	1
[R].lELELRPTGEIEQYSVSATYELQR.[E]	N-Term(iTRAQ4plex)	1	1
[K].iEGNLIFDPNNYLPk.[E]	N-Term(iTRAQ4plex); K15(iTRAQ	1	1
[K].nLTDFAEQYSIQDWAk.[R]	N-Term(iTRAQ4plex); K16(iTRAQ	1	1
[K].aQNLYQELLTQEGQASFQGLk.[D]	N-Term(iTRAQ4plex); K21(iTRAQ	1	1
[R].iLGEELGFASLHDLQLLGk.[L]	N-Term(iTRAQ4plex); K19(iTRAQ	1	1
[K].gFEPTLEALFGk.[Q]	N-Term(iTRAQ4plex); K12(iTRAQ	1	1
[K].gFEPTLEALFGk.[Q]	N-Term(iTRAQ4plex); K12(iTRAQ	1	1
[K].vNWEEEAASGLLTSLk.[D]	N-Term(iTRAQ4plex); K16(iTRAQ	1	1
[K].fPEVDVLTk.[Y]	N-Term(iTRAQ4plex); K9(iTRAQ4	1	1
[R].nIQEYLSILTDPDGk.[G]	N-Term(iTRAQ4plex); K15(iTRAQ	1	1
[R].nIQEYLSILTDPDGk.[G]	N-Term(iTRAQ4plex); K15(iTRAQ	1	1
[K].iEFEWNTGTNVDTk.[K]	N-Term(iTRAQ4plex); K14(iTRAQ	1	1
[K].NFVASHIANILNSEELDIQDLk.[K]	K22(iTRAQ4plex)	1	1
[K].aTVAVYLESLQDTk.[I]	N-Term(iTRAQ4plex); K14(iTRAQ	1	1
[K].sVSDGIAALDLNAVANk.[I]	N-Term(iTRAQ4plex); K17(iTRAQ	1	1
[K].aDSVVDLLSYNVQGSGETTYDHk.[N]	N-Term(iTRAQ4plex); K23(iTRAQ	1	1
[K].aLVEQGFTVPEIk.[T]	N-Term(iTRAQ4plex); K13(iTRAQ	1	1
[K].aLVEQGFTVPEIk.[T]	N-Term(iTRAQ4plex); K13(iTRAQ	1	1

[K].iAELSATAQEIIk.[S]	N-Term(iTRAQ4plex); K13(iTRAQ4	1	1
[K].iAELSATAQEIIk.[S]	N-Term(iTRAQ4plex); K13(iTRAQ4	1	1
[K].iAELSATAQEIIk.[S]	N-Term(iTRAQ4plex); K13(iTRAQ4	1	1
[K].sVSDGIAALDLNAVANk.[I]	N-Term(iTRAQ4plex); K17(iTRAQ4	1	1
[K].sVSDGIAALDLNAVANk.[I]	N-Term(iTRAQ4plex); K17(iTRAQ4	1	1
[K].mTSNFPVDLSDYPk.[S]	N-Term(iTRAQ4plex); K14(iTRAQ4	1	1
[K].KmTSNFPVDLSDYPk.[S]	M2(Oxidation); K15(iTRAQ4plex)	1	1
[K].iAELSATAQEIIk.[S]	N-Term(iTRAQ4plex); K13(iTRAQ4	1	1
[K].sVSDGIAALDLNAVANk.[I]	N-Term(iTRAQ4plex); K17(iTRAQ4	1	1
[K].yEGLQEWEGk.[A]	N-Term(iTRAQ4plex); K10(iTRAQ4	1	1
[K].yEGLQEWEGk.[A]	N-Term(iTRAQ4plex); K10(iTRAQ4	1	1
[K].tQFNNNEYSQDLDAYNTk.[D]	N-Term(iTRAQ4plex); K18(iTRAQ4	1	1
[K].eVYGFNPEGk.[A]	N-Term(iTRAQ4plex); K10(iTRAQ4	1	1
[K].yTYNYEAESSSGVPGTADSR.[S]	N-Term(iTRAQ4plex)	1	1
[K].gNVATEISTER.[D]	N-Term(iTRAQ4plex)	1	1
[R].sSVkLQGTSk.[I]	N-Term(iTRAQ4plex); K4(iTRAQ4	1	1
[R].kHVAEAIck.[E]	N-Term(iTRAQ4plex); K1(iTRAQ4	1	1
[R].iLGEELGFASLHDLQLLGk.[L]	N-Term(iTRAQ4plex); K19(iTRAQ4	1	1
[R].kHVAEAIck.[E]	N-Term(iTRAQ4plex); K1(iTRAQ4	1	1
[R].iLGEELGFASLHDLQLLGk.[L]	N-Term(iTRAQ4plex); K19(iTRAQ4	1	1
[R].kHVAEAIck.[E]	N-Term(iTRAQ4plex); C8(Carbam	1	1
[R].kHVAEAIck.[E]	K1(iTRAQ4plex); C8(Carbamidon	1	1
[R].kGNVATEISTER.[D]	N-Term(iTRAQ4plex); K1(iTRAQ4	1	1
[K].nFVASHIANILNSEELDIQDLk.[K]	N-Term(iTRAQ4plex); K22(iTRAQ4	1	1
[K].nFVASHIANILNSEELDIQDLk.[K]	N-Term(iTRAQ4plex); K22(iTRAQ4	1	1
[K].lEVLNFDFQANAQLSNPk.[I]	N-Term(iTRAQ4plex); K18(iTRAQ4	1	1
[K].gISTSAASPAVGTVGmDmDEDDDFSk.[W	N-Term(iTRAQ4plex); M16(Oxida	1	1
[K].gISTSAASPAVGTVGmDmDEDDDFSk.[W	N-Term(iTRAQ4plex); M16(Oxida	1	1
[K].gISTSAASPAVGTVGmDMDEDDDFSk.[W	N-Term(iTRAQ4plex); M16(Oxida	1	1
[K].gISTSAASPAVGTVGMDmDEDDDFSk.[W	N-Term(iTRAQ4plex); M18(Oxida	1	1
[R].sPSQADINk.[I]	N-Term(iTRAQ4plex); K9(iTRAQ4	1	1
[K].tILGTmPAFEVSLQALQk.[A]	N-Term(iTRAQ4plex); M6(Oxidat	1	1
[K].iEIPLPFGGk.[S]	N-Term(iTRAQ4plex); K10(iTRAQ4	1	1
[K].lIVAMSSWLQk.[A]	N-Term(iTRAQ4plex); K11(iTRAQ4	1	1
[K].iEIPLPFGGk.[S]	N-Term(iTRAQ4plex); K10(iTRAQ4	1	1
[K].fDHTNSLNIAGLSLDFSSk.[L]	N-Term(iTRAQ4plex); K19(iTRAQ4	1	1
[K].iEIPLPFGGk.[S]	N-Term(iTRAQ4plex); K10(iTRAQ4	1	1
[K].aVSMPSFSILGSDVR.[V]	N-Term(iTRAQ4plex)	1	1
[R].lAAYLMLMR.[S]	N-Term(iTRAQ4plex)	1	1
[R].lAAYLMLMR.[S]	N-Term(iTRAQ4plex)	1	1
[R].kGNVATEISTER.[D]	N-Term(iTRAQ4plex); K1(iTRAQ4	1	1
[K].nmEVSVATTTk.[A]	N-Term(iTRAQ4plex); M2(Oxidat	1	1
[K].sLDEHYHIR.[V]	N-Term(iTRAQ4plex)	1	1
[R].kGNVATEISTER.[D]	N-Term(iTRAQ4plex); K1(iTRAQ4	1	1
[R].kGNVATEISTER.[D]	N-Term(iTRAQ4plex); K1(iTRAQ4	1	1
[R].kGNVATEISTER.[D]	N-Term(iTRAQ4plex); K1(iTRAQ4	1	1
[R].kGNVATEISTER.[D]	N-Term(iTRAQ4plex); K1(iTRAQ4	1	1
[K].aVSMPSFSILGSDVR.[V]	N-Term(iTRAQ4plex)	1	1
[R].tSSFALNLPTLPEVk.[F]	N-Term(iTRAQ4plex); K15(iTRAQ4	1	1
[K].lPQQANDYLNSFNWER.[Q]	N-Term(iTRAQ4plex)	1	1
[K].sNTVASLHTEk.[N]	N-Term(iTRAQ4plex); K11(iTRAQ4	1	1
[R].tSSFALNLPTLPEVk.[F]	N-Term(iTRAQ4plex); K15(iTRAQ4	1	1

[K].lPQQANDYLNSFNWER.[Q]	N-Term(iTRAQ4plex)	1	1
[K].qHIEAIDVR.[V]	N-Term(iTRAQ4plex)	1	1
[K].hVAEAIck.[E]	N-Term(iTRAQ4plex); C7(Carbam	1	1
[K].qHIEAIDVR.[V]	N-Term(iTRAQ4plex)	1	1
[R].tLQGIPQMIGEVIR.[K]	N-Term(iTRAQ4plex)	1	1
[R].tLQGIPQMIGEVIR.[K]	N-Term(iTRAQ4plex)	1	1
[R].tLQGIPQMIGEVIR.[K]	N-Term(iTRAQ4plex)	1	1
[R].sEYQADYESLR.[F]	N-Term(iTRAQ4plex)	1	1
[R].tLQGIPQMIGEVIR.[K]	N-Term(iTRAQ4plex)	1	1
[K].aASGTTGTYQEWk.[D]	N-Term(iTRAQ4plex); K13(iTRAQ	1	1
[K].eNFAGEATLQR.[I]	N-Term(iTRAQ4plex)	1	1
[K].aASGTTGTYQEWk.[D]	N-Term(iTRAQ4plex); K13(iTRAQ	1	1
[R].SEYQADYESLR.[F]		1	1
[R].kYTYNYEAESSSGVPGTADSR.[S]	N-Term(iTRAQ4plex)	1	1
[R].kYTYNYEAESSSGVPGTADSR.[S]	K1(iTRAQ4plex)	1	1
[K].dSYDLHDLk.[I]	N-Term(iTRAQ4plex); K9(iTRAQ4	1	1
[K].eNFAGEATLQR.[I]	N-Term(iTRAQ4plex)	1	1
[K].nSEEFAAAMSR.[Y]	N-Term(iTRAQ4plex)	1	1
[K].eNFAGEATLQR.[I]	N-Term(iTRAQ4plex)	1	1
[K].tILGTmPAFEVSLQALQk.[A]	N-Term(iTRAQ4plex); M6(Oxidat	1	1
[R].lPYTIITTPPLk.[D]	N-Term(iTRAQ4plex); K12(iTRAQ	1	1
[R].sEYQADYESLR.[F]	N-Term(iTRAQ4plex)	1	1
[R].sEYQADYESLR.[F]	N-Term(iTRAQ4plex)	1	1
[K].iEDGTLASk.[T]	N-Term(iTRAQ4plex); K9(iTRAQ4	1	1
[K].tIHDLHLFIENIDFNk.[S]	N-Term(iTRAQ4plex); K16(iTRAQ	1	1
[K].tIHDLHLFIENIDFNk.[S]	N-Term(iTRAQ4plex); K16(iTRAQ	1	1
[K].tIHDLHLFIENIDFNk.[S]	N-Term(iTRAQ4plex); K16(iTRAQ	1	1
[K].tIHDLHLFIENIDFNk.[S]	N-Term(iTRAQ4plex); K16(iTRAQ	1	1
[R].hSITNPLAVLcEFISQSIk.[S]	N-Term(iTRAQ4plex); C11(Carbar	1	1
[R].hSITNPLAVLcEFISQSIk.[S]	N-Term(iTRAQ4plex); C11(Carbar	1	1
[K].yENYELTLk.[S]	N-Term(iTRAQ4plex); K9(iTRAQ4	1	1
[R].eSDEETQIk.[V]	N-Term(iTRAQ4plex); K9(iTRAQ4	1	1
[K].hLIDSLIDFLNFPR.[F]	N-Term(iTRAQ4plex)	1	1
[R].eSDEETQIk.[V]	N-Term(iTRAQ4plex); K9(iTRAQ4	1	1
[K].hLIDSLIDFLNFPR.[F]	N-Term(iTRAQ4plex)	1	1
[R].qSMTLSSEVQIPDFDVDLGTILR.[V]	N-Term(iTRAQ4plex)	1	1
[K].IADFELPTIIVPEQTIEIPSIk.[F]	K22(iTRAQ4plex)	1	1
[R].iHSGSFQSQVELSNDQEk.[A]	N-Term(iTRAQ4plex); K18(iTRAQ	1	1
[R].iHSGSFQSQVELSNDQEk.[A]	N-Term(iTRAQ4plex); K18(iTRAQ	1	1
[R].kYTYNYEAESSSGVPGTADSR.[S]	N-Term(iTRAQ4plex); K1(iTRAQ4	1	1
[R].kYTYNYEAESSSGVPGTADSR.[S]	N-Term(iTRAQ4plex); K1(iTRAQ4	1	1
[R].iHSGSFQSQVELSNDQEk.[A]	N-Term(iTRAQ4plex); K18(iTRAQ	1	1
[K].nQDVHSINLPFFETLQEYFER.[N]	N-Term(iTRAQ4plex)	1	1
[R].iHSGSFQSQVELSNDQEk.[A]	N-Term(iTRAQ4plex); K18(iTRAQ	1	1
[K].tTLTAFGFASADLIEIGLEGk.[G]	N-Term(iTRAQ4plex); K21(iTRAQ	1	1
[K].ySQPEDSLIPFFEITVPESQLTVSQFTLPk.[S]	N-Term(iTRAQ4plex); K30(iTRAQ	1	1
[K].ySQPEDSLIPFFEITVPESQLTVSQFTLPk.[S]	N-Term(iTRAQ4plex); K30(iTRAQ	1	1
[K].sGSSTASWIQNVDTk.[Y]	N-Term(iTRAQ4plex); K15(iTRAQ	1	1
[R].tIDQMLNSELQWPVPDIYLR.[D]	N-Term(iTRAQ4plex)	1	1
[K].iADFELPTIIVPEQTIEIPSIk.[F]	N-Term(iTRAQ4plex); K22(iTRAQ	1	1
[K].iADFELPTIIVPEQTIEIPSIk.[F]	N-Term(iTRAQ4plex); K22(iTRAQ	1	1
[K].iADFELPTIIVPEQTIEIPSIk.[F]	N-Term(iTRAQ4plex); K22(iTRAQ	1	1

[K].aSGSLPYTQTLQDHLNSLk.[E]	N-Term(iTRAQ4plex); K19(iTRAQ4	1	1
[K].yGMVAQVTQTLk.[L]	N-Term(iTRAQ4plex); K12(iTRAQ4	1	1
[K].aSGSLPYTQTLQDHLNSLk.[E]	N-Term(iTRAQ4plex); K19(iTRAQ4	1	1
[K].aTFQTPDFIVPLTDLR.[I]	N-Term(iTRAQ4plex)	1	1
[R].vPSYTLILPSLELPVLHVPR.[N]	N-Term(iTRAQ4plex)	1	1
[R].dFSAEYEEDGk.[Y]	N-Term(iTRAQ4plex); K11(iTRAQ4	1	1
[R].dFSAEYEEDGk.[Y]	N-Term(iTRAQ4plex); K11(iTRAQ4	1	1
[R].dFSAEYEEDGk.[Y]	N-Term(iTRAQ4plex); K11(iTRAQ4	1	1
[K].sFDYHQFVDETNDk.[I]	N-Term(iTRAQ4plex); K14(iTRAQ4	1	1
[K].aTFQTPDFIVPLTDLR.[I]	N-Term(iTRAQ4plex)	1	1
[R].vHANPLLIDVVTYLVALIPEPSAQQLR.[E]	N-Term(iTRAQ4plex)	1	1
[R].vHANPLLIDVVTYLVALIPEPSAQQLR.[E]	N-Term(iTRAQ4plex)	1	1
[K].tILGTMPAFEVSLQALQk.[A]	N-Term(iTRAQ4plex); K18(iTRAQ4	1	1
[R].tGISPLALIk.[G]	N-Term(iTRAQ4plex); K10(iTRAQ4	1	1
[K].tILGTMPAFEVSLQALQk.[A]	N-Term(iTRAQ4plex); K18(iTRAQ4	1	1
[R].dFSAEYEEDGk.[Y]	N-Term(iTRAQ4plex); K11(iTRAQ4	1	1
[K].vELEVPQLcSFILk.[T]	N-Term(iTRAQ4plex); C9(Carbam	1	1
[K].vPLLLSEPINIIDALEMR.[D]	N-Term(iTRAQ4plex)	1	1
[K].nSEEFAAAMSR.[Y]	N-Term(iTRAQ4plex)	1	1
[R].kYTYNYEAESSSGVPGTADSR.[S]	N-Term(iTRAQ4plex); K1(iTRAQ4	1	1
[R].kYTYNYEAESSSGVPGTADSR.[S]	N-Term(iTRAQ4plex); K1(iTRAQ4	1	1
[K].fSVPAGIVIPSFQALTAR.[F]	N-Term(iTRAQ4plex)	1	1
[K].vPLLLSEPINIIDALEMR.[D]	N-Term(iTRAQ4plex)	1	1
[K].lNDLNSVLVMPTFHVPFTDLQVPSck.[L]	N-Term(iTRAQ4plex); C25(Carbar	1	1
[K].dkIGVELTGR.[T]	N-Term(iTRAQ4plex); K2(iTRAQ4	1	1
[K].lSLESLTSYFSIESSTk.[G]	N-Term(iTRAQ4plex); K17(iTRAQ4	1	1
[K].lSLESLTSYFSIESSTk.[G]	N-Term(iTRAQ4plex); K17(iTRAQ4	1	1
[R].vPSYTLILPSLELPVLHVPR.[N]	N-Term(iTRAQ4plex)	1	1
[K].vQGVEFSHR.[L]	N-Term(iTRAQ4plex)	1	1
[K].eFNLQNMGLPDFHIPENLFLk.[S]	N-Term(iTRAQ4plex); K21(iTRAQ4	1	1
[K].sPAFTDLHLR.[Y]	N-Term(iTRAQ4plex)	1	1
[K].eSQLPTVMDFR.[K]	N-Term(iTRAQ4plex)	1	1
[K].sVSLPSLDPASAk.[I]	N-Term(iTRAQ4plex); K13(iTRAQ4	1	1
[K].fPEVDVLTk.[Y]	N-Term(iTRAQ4plex); K9(iTRAQ4	1	1
[K].gMALFGEGk.[A]	N-Term(iTRAQ4plex); K9(iTRAQ4	1	1
[R].kGNVATEISTER.[D]	N-Term(iTRAQ4plex); K1(iTRAQ4	1	1
[R].lELELRPTGEIEQYSVSATYELQR.[E]	N-Term(iTRAQ4plex)	1	1
[K].lEIQSQVDSQHVGHSVLTAk.[G]	N-Term(iTRAQ4plex); K20(iTRAQ4	1	1
[K].aHLDIAGSLEGHLR.[F]	N-Term(iTRAQ4plex)	1	1
[R].kSISAALEHk.[V]	N-Term(iTRAQ4plex); K1(iTRAQ4	1	1
[R].mYQMDIQQELQR.[Y]	N-Term(iTRAQ4plex)	1	1
[K].lDVTTSIGR.[R]	N-Term(iTRAQ4plex)	1	1
[K].lAELSATAQEIIk.[S]	K13(iTRAQ4plex)	1	1
[K].yHWEHTGLTLR.[E]	N-Term(iTRAQ4plex)	1	1
[K].nIILPVYDk.[S]	N-Term(iTRAQ4plex); K9(iTRAQ4	1	1
[R].TLQGIPQMIGEVIR.[K]		1	1
[K].lDVTTSIGR.[R]	N-Term(iTRAQ4plex)	1	1
[R].iHSGSFQSQVELSNDQEk.[A]	N-Term(iTRAQ4plex); K18(iTRAQ4	1	1
[K].aGHIAWTSSGk.[G]	N-Term(iTRAQ4plex); K11(iTRAQ4	1	1
[R].QTIIVVLENVQR.[N]		1	1
[K].hIYAISSAALSASYk.[A]	N-Term(iTRAQ4plex); K15(iTRAQ4	1	1
[K].yHWEHTGLTLR.[E]	N-Term(iTRAQ4plex)	1	1

[K].sVGFHLPSR.[E]	N-Term(iTRAQ4plex)	1	1
[K].eNFAGEATLQR.[I]	N-Term(iTRAQ4plex)	1	1
[K].kMGLAFESTk.[S]	N-Term(iTRAQ4plex); K1(iTRAQ4	1	1
[K].qVFLYPEk.[D]	N-Term(iTRAQ4plex); K8(iTRAQ4	1	1
[R].sEYQADYESLR.[F]	N-Term(iTRAQ4plex)	1	1
[R].aLVDTLk.[F]	N-Term(iTRAQ4plex); K7(iTRAQ4	1	1
[K].gMTRPLSTLISSSQScQYTLDAk.[R]	N-Term(iTRAQ4plex); C16(Carbar	1	1
[R].qTIIVVLENVQR.[N]	N-Term(iTRAQ4plex)	1	1
[K].hINIDQFVR.[K]	N-Term(iTRAQ4plex)	1	1
[K].iAIANIIDEIIEk.[L]	N-Term(iTRAQ4plex); K13(iTRAQ	1	1
[K].iAELSATAQEIIk.[S]	N-Term(iTRAQ4plex); K13(iTRAQ	1	1
[K].FSVPAGIVIPSFQALTAR.[F]		1	1
[K].lATALSLSNk.[F]	N-Term(iTRAQ4plex); K10(iTRAQ	1	1
[R].kYTYNYEAESSSGVPGTADSR.[S]	N-Term(iTRAQ4plex); K1(iTRAQ4	1	1
[K].aTGVLYDYVNk.[Y]	N-Term(iTRAQ4plex); K11(iTRAQ	1	1
[K].dFSLWEk.[T]	N-Term(iTRAQ4plex); K7(iTRAQ4	1	1
[R].nNALDFVTk.[S]	N-Term(iTRAQ4plex); K9(iTRAQ4	1	1
[K].sVGFHLPSR.[E]	N-Term(iTRAQ4plex)	1	1
[K].nMEVSVATTTk.[A]	N-Term(iTRAQ4plex); K11(iTRAQ	1	1
[K].lDFSSQADLR.[N]	N-Term(iTRAQ4plex)	1	1
[K].lALWGEHTGQLYSk.[F]	N-Term(iTRAQ4plex); K14(iTRAQ	1	1
[K].eVYGFNPEGk.[A]	N-Term(iTRAQ4plex); K10(iTRAQ	1	1
[K].fLDMLIk.[K]	N-Term(iTRAQ4plex); K7(iTRAQ4	1	1
[K].fDHTNSLNIAGLSLDFSSk.[L]	N-Term(iTRAQ4plex); K19(iTRAQ	1	1
[R].aLVDTLk.[F]	N-Term(iTRAQ4plex); K7(iTRAQ4	1	1
[R].vLLDQLGTTISFER.[I]	N-Term(iTRAQ4plex)	1	1
[K].iGVELTGR.[T]	N-Term(iTRAQ4plex)	1	1
[K].TEVIPPLIENR.[Q]		1	1
[R].eSDEETQIk.[V]	N-Term(iTRAQ4plex); K9(iTRAQ4	1	1
[R].lELELRPTGEIEQYSVSATYELQR.[E]	N-Term(iTRAQ4plex)	1	1
[K].qVFLYPEkDEPTYILNIk.[R]	N-Term(iTRAQ4plex); K8(iTRAQ4	1	1
[R].FQFPGkPGIYTR.[E]	K6(iTRAQ4plex)	1	1
[R].dFSAEYEEDGk.[Y]	N-Term(iTRAQ4plex); K11(iTRAQ	1	1
[K].eVYGFNPEGk.[A]	N-Term(iTRAQ4plex); K10(iTRAQ	1	1
[K].NSEEFAAAMSR.[Y]		1	1
[K].ENFAGEATLQR.[I]		1	1
[K].fRETLEDTR.[D]	N-Term(iTRAQ4plex)	1	1
[K].sPAFTDLHLR.[Y]	N-Term(iTRAQ4plex)	1	1
[K].IEDGTLASk.[T]	K9(iTRAQ4plex)	1	1
[K].lTISEQNIQR.[A]	N-Term(iTRAQ4plex)	1	1
[K].EVYGFNPEGk.[A]	K10(iTRAQ4plex)	1	1
[K].nSEEFAAAMSR.[Y]	N-Term(iTRAQ4plex)	1	1
[K].vSALLTPAEQTGTWk.[L]	N-Term(iTRAQ4plex); K15(iTRAQ	1	1
[R].VNDESTEGkTSYR.[L]	K9(iTRAQ4plex)	1	1
[R].kGNVATEISTER.[D]	N-Term(iTRAQ4plex); K1(iTRAQ4	1	1
[K].FPEVDVLTk.[Y]	K9(iTRAQ4plex)	1	1
[K].nSEEFAAAmSR.[Y]	N-Term(iTRAQ4plex); M9(Oxidat	1	1
[K].iAELSATAQEIIk.[S]	N-Term(iTRAQ4plex); K13(iTRAQ	1	1
[K].fPEVDVLTk.[Y]	N-Term(iTRAQ4plex); K9(iTRAQ4	1	1
[K].ATGVLYDYVNk.[Y]	K11(iTRAQ4plex)	1	1
[K].nSLkIEIPLPFGGk.[S]	N-Term(iTRAQ4plex); K4(iTRAQ4	1	1
[R].hIQNIDIQHLAGk.[L]	N-Term(iTRAQ4plex); K13(iTRAQ	1	1

[K].aHLDIAGSLEGHLR.[F]	N-Term(iTRAQ4plex)	1	1
[R].tLADLTLLDSPIk.[V]	N-Term(iTRAQ4plex); K13(iTRAQ4	1	1
[K].tEVIPPLIENR.[Q]	N-Term(iTRAQ4plex)	1	1
[R].gIISALLVPPETEEAk.[Q]	N-Term(iTRAQ4plex); K16(iTRAQ4	1	1
[R].qTIIVVLENVQR.[N]	N-Term(iTRAQ4plex)	1	1
[K].hINIDQFVR.[K]	N-Term(iTRAQ4plex)	1	1
[R].kGNVATEISTER.[D]	N-Term(iTRAQ4plex); K1(iTRAQ4	1	1
[K].kLTISEQNIQR.[A]	N-Term(iTRAQ4plex); K1(iTRAQ4	1	1
[R].VTQEFHMk.[V]	K8(iTRAQ4plex)	1	1
[K].ATGVLYDYVNk.[Y]	K11(iTRAQ4plex)	1	1
[R].TPALHFk.[S]	K7(iTRAQ4plex)	1	1
[K].lSNVLQQVk.[I]	N-Term(iTRAQ4plex); K9(iTRAQ4	1	1
[K].aLYWVNGQVPDGVSk.[V]	N-Term(iTRAQ4plex); K15(iTRAQ4	1	1
[R].lPYTIITTPPLk.[D]	N-Term(iTRAQ4plex); K12(iTRAQ4	1	1
[K].eVYGFNPEGk.[A]	N-Term(iTRAQ4plex); K10(iTRAQ4	1	1
[R].vNQNLVYESGSLNFSk.[L]	N-Term(iTRAQ4plex); K16(iTRAQ4	1	1
[K].lFLEETk.[A]	N-Term(iTRAQ4plex); K7(iTRAQ4	1	1
[K].aHLDIAGSLEGHLR.[F]	N-Term(iTRAQ4plex)	1	1
[K].sQAIATk.[K]	N-Term(iTRAQ4plex); K7(iTRAQ4	1	1
[K].LDVTTSIGR.[R]		1	1
[K].eSQLPTVmDFR.[K]	N-Term(iTRAQ4plex); M8(Oxidat	1	1
[R].eFQVPTFTIPk.[L]	N-Term(iTRAQ4plex); K11(iTRAQ4	1	1
[R].qIDDIDVR.[F]	N-Term(iTRAQ4plex)	1	1
[K].dNVFDGLVR.[V]	N-Term(iTRAQ4plex)	1	1
[K].sLWDFLk.[L]	N-Term(iTRAQ4plex); K7(iTRAQ4	1	1
[K].fVTQAEGAk.[Q]	N-Term(iTRAQ4plex); K9(iTRAQ4	1	1
[K].yNALDLTNNGk.[L]	N-Term(iTRAQ4plex); K11(iTRAQ4	1	1
[K].lAIPEGk.[Q]	N-Term(iTRAQ4plex); K7(iTRAQ4	1	1
[K].qGFFPDSVNk.[A]	N-Term(iTRAQ4plex); K10(iTRAQ4	1	1
[K].iEFEWNTGTNVDTk.[K]	N-Term(iTRAQ4plex); K14(iTRAQ4	1	1
[R].dAVEkPQEFTIVAFVk.[Y]	N-Term(iTRAQ4plex); K5(iTRAQ4	1	1
[K].nMEVSVATTTk.[A]	N-Term(iTRAQ4plex); K11(iTRAQ4	1	1
[K].lLLQmDSSATAYGSTVSk.[R]	N-Term(iTRAQ4plex); M5(Oxidat	1	1
[K].YHWEHTGLTLR.[E]		1	1
[K].lLSGGNTLHLVSTTk.[T]	N-Term(iTRAQ4plex); K15(iTRAQ4	1	1
[R].iYSLWEHSTk.[N]	N-Term(iTRAQ4plex); K10(iTRAQ4	1	1
[K].gTYGLScQR.[D]	N-Term(iTRAQ4plex); C7(Carbam	1	1
[R].ITENDIQIALDDAk.[I]	K14(iTRAQ4plex)	1	1
[K].sLHMYANR.[L]	N-Term(iTRAQ4plex)	1	1
[K].gAYQNNEIk.[H]	N-Term(iTRAQ4plex); K9(iTRAQ4	1	1
[K].sLHMYANR.[L]	N-Term(iTRAQ4plex)	1	1
[K].sLHMYANR.[L]	N-Term(iTRAQ4plex)	1	1
[K].sISAALEHk.[V]	N-Term(iTRAQ4plex); K9(iTRAQ4	1	1
[K].aTGVLYDYVNk.[Y]	N-Term(iTRAQ4plex); K11(iTRAQ4	1	1
[K].FVTQAEGAk.[Q]	K9(iTRAQ4plex)	1	1
[R].iGQDGISTSATTNLk.[C]	N-Term(iTRAQ4plex); K15(iTRAQ4	1	1
[K].YHWEHTGLTLR.[E]		1	1
[K].tSQcTLk.[E]	N-Term(iTRAQ4plex); C4(Carbam	1	1
[K].iVQILPWEQNEQVk.[N]	N-Term(iTRAQ4plex); K14(iTRAQ4	1	1
[K].mGLAFESTk.[S]	N-Term(iTRAQ4plex); K9(iTRAQ4	1	1
[K].lSLPDFk.[E]	N-Term(iTRAQ4plex); K7(iTRAQ4	1	1
[K].sPAFTDLHLR.[Y]	N-Term(iTRAQ4plex)	1	1

[K].gMALFGEGk.[A]	N-Term(iTRAQ4plex); K9(iTRAQ4	1	1
[R].mNFkQELNGNTk.[S]	N-Term(iTRAQ4plex); K4(iTRAQ4	1	1
[K].iNPLALk.[E]	N-Term(iTRAQ4plex); K7(iTRAQ4	1	1
[K].tEVIPPLIENR.[Q]	N-Term(iTRAQ4plex)	1	1
[R].NLQNNAEWVYQGAIR.[Q]		1	1
[K].sVGFHLPSR.[E]	N-Term(iTRAQ4plex)	1	1
[K].dEPTYILNIk.[R]	N-Term(iTRAQ4plex); K10(iTRAQ	1	1
[K].dFSLWEk.[T]	N-Term(iTRAQ4plex); K7(iTRAQ4	1	1
[K].kMTSNFPVDLSDYPk.[S]	N-Term(iTRAQ4plex); K1(iTRAQ4	1	1
[R].iPSVQINFk.[D]	N-Term(iTRAQ4plex); K9(iTRAQ4	1	1
[R].iLGEELGFASLHDLQLLGk.[L]	N-Term(iTRAQ4plex); K19(iTRAQ	1	1
[K].vNWEEEAASGLLTSLk.[D]	N-Term(iTRAQ4plex); K16(iTRAQ	1	1
[K].dNVFDGLVR.[V]	N-Term(iTRAQ4plex)	1	1
[K].iEFEWNTGTNVDTk.[K]	N-Term(iTRAQ4plex); K14(iTRAQ	1	1
[R].eFQVPTFTIPk.[L]	N-Term(iTRAQ4plex); K11(iTRAQ	1	1
[R].kYTYNYEAESSSGVPGTADSR.[S]	N-Term(iTRAQ4plex); K1(iTRAQ4	1	1
[K].tSQcTLk.[E]	N-Term(iTRAQ4plex); C4(Carbam	1	1
[K].ATGVLYDYVNk.[Y]	K11(iTRAQ4plex)	1	1
[K].iEFEWNTGTNVDTk.[K]	N-Term(iTRAQ4plex); K14(iTRAQ	1	1
[K].gNVATEISTER.[D]	N-Term(iTRAQ4plex)	1	1
[K].nFVASHIANILNSEELDIQDLk.[K]	N-Term(iTRAQ4plex); K22(iTRAQ	1	1
[R].eSDEETQIk.[V]	N-Term(iTRAQ4plex); K9(iTRAQ4	1	1
[R].qIDDIDVR.[F]	N-Term(iTRAQ4plex)	1	1
[K].TFIEDVNk.[F]	K8(iTRAQ4plex)	1	1
[K].SVSLPSLDPASAk.[I]	K13(iTRAQ4plex)	1	1
[K].qAEAVLk.[T]	N-Term(iTRAQ4plex); K7(iTRAQ4	1	1
[K].lIVAmSSWLQk.[A]	N-Term(iTRAQ4plex); M5(Oxidat	1	1
[K].nSEEFAAAMSR.[Y]	N-Term(iTRAQ4plex)	1	1
[K].kIISDYHQQFR.[Y]	N-Term(iTRAQ4plex); K1(iTRAQ4	1	1
[R].lTLDIQNkk.[I]	N-Term(iTRAQ4plex); K8(iTRAQ4	1	1
[K].tkNSEEFAAAMSR.[Y]	N-Term(iTRAQ4plex); K2(iTRAQ4	1	1
[R].NNALDFVTk.[S]	K9(iTRAQ4plex)	1	1
[R].QIDDIDVR.[F]		1	1
[K].yHWEHTGLTLR.[E]	N-Term(iTRAQ4plex)	1	1
[R].tLADLTLLDSPIk.[V]	N-Term(iTRAQ4plex); K13(iTRAQ	1	1
[K].aEPLAFTFSHDYk.[G]	N-Term(iTRAQ4plex); K13(iTRAQ	1	1
[R].eSDEETQIk.[V]	N-Term(iTRAQ4plex); K9(iTRAQ4	1	1
[K].IVQILPWEQNEQVk.[N]	K14(iTRAQ4plex)	1	1
[K].lIDVISMYR.[E]	N-Term(iTRAQ4plex)	1	1
[K].yHWEHTGLTLR.[E]	N-Term(iTRAQ4plex)	1	1
[R].eELcTMFIR.[E]	N-Term(iTRAQ4plex); C4(Carbam	1	1
[K].IISDYHQQFR.[Y]		1	1
[K].qGFFPDSVNk.[A]	N-Term(iTRAQ4plex); K10(iTRAQ	1	1
[R].fQFPGkPGIYTR.[E]	N-Term(iTRAQ4plex); K6(iTRAQ4	1	1
[K].lTISEQNIQR.[A]	N-Term(iTRAQ4plex)	1	1
[K].AHLDIAGSLEGHLR.[F]		1	1
[K].sLHMYANR.[L]	N-Term(iTRAQ4plex)	1	1
[K].sVGFHLPSR.[E]	N-Term(iTRAQ4plex)	1	1
[R].mYQmDIQQELQR.[Y]	N-Term(iTRAQ4plex); M1(Oxidat	1	1
[K].nSLkIEIPLPFGGk.[S]	N-Term(iTRAQ4plex); K4(iTRAQ4	1	1
[K].yENYELTLk.[S]	N-Term(iTRAQ4plex); K9(iTRAQ4	1	1
[R].LNGEIQALELPQk.[A]	K13(iTRAQ4plex)	1	1

[R].ITLPDFR.[L]		1	1
[R].TGISPLALIk.[G]	K10(iTRAQ4plex)	1	1
[K].eSQLPTVmDFR.[K]	N-Term(iTRAQ4plex); M8(Oxidat	1	1
[K].eNLcLNLHk.[F]	N-Term(iTRAQ4plex); C4(Carbam	1	1
[R].VSSFYAk.[G]	K7(iTRAQ4plex)	1	1
[K].sHDELPR.[T]	N-Term(iTRAQ4plex)	1	1
[K].gAYQNNEIk.[H]	N-Term(iTRAQ4plex); K9(iTRAQ4	1	1
[R].vIGNmGQTMEQLTPELk.[S]	N-Term(iTRAQ4plex); M5(Oxidat	1	1
[K].gFEPTLEALFGk.[Q]	N-Term(iTRAQ4plex); K12(iTRAQ	1	1
[R].lELELRPTGEIEQYSVSATYELQR.[E]	N-Term(iTRAQ4plex)	1	1
[K].kLTISEQNIQR.[A]	N-Term(iTRAQ4plex)	1	1
[K].kLTISEQNIQR.[A]	K1(iTRAQ4plex)	1	1
[K].lLLQMDSSATAYGSTVSk.[R]	N-Term(iTRAQ4plex); K18(iTRAQ	1	1
[K].FVTQAEGAk.[Q]	K9(iTRAQ4plex)	1	1
[R].SPSQADINk.[I]	K9(iTRAQ4plex)	1	1
[R].HIQNIDIQHLAGk.[L]	K13(iTRAQ4plex)	1	1
[K].lFLEETk.[A]	N-Term(iTRAQ4plex); K7(iTRAQ4	1	1
[K].nLLVALk.[D]	N-Term(iTRAQ4plex); K7(iTRAQ4	1	1
[K].sLHmYANR.[L]	N-Term(iTRAQ4plex); M4(Oxidat	1	1
[K].qSFDLSVk.[A]	N-Term(iTRAQ4plex); K8(iTRAQ4	1	1
[K].iEGNLIFDPNNYLPk.[E]	N-Term(iTRAQ4plex); K15(iTRAQ	1	1
[K].iNPLALk.[E]	N-Term(iTRAQ4plex); K7(iTRAQ4	1	1
[R].aTLYALSHAVNNYHk.[T]	N-Term(iTRAQ4plex); K15(iTRAQ	1	1
[R].sPSQADINk.[I]	N-Term(iTRAQ4plex); K9(iTRAQ4	1	1
[R].vPQTDMTFR.[H]	N-Term(iTRAQ4plex)	1	1
[R].lELELRPTGEIEQYSVSATYELQR.[E]	N-Term(iTRAQ4plex)	1	1
[K].tGLkEFLk.[T]	N-Term(iTRAQ4plex); K4(iTRAQ4	1	1
[K].sLWDFLk.[L]	N-Term(iTRAQ4plex); K7(iTRAQ4	1	1
[K].sHDELPR.[T]	N-Term(iTRAQ4plex)	1	1
[K].FLDSNIk.[F]	K7(iTRAQ4plex)	1	1
[K].wNFYYSPQSSPDkk.[L]	N-Term(iTRAQ4plex); K13(iTRAQ	1	1
[K].cVQSTkPSLMIQk.[A]	C1(Carbamidomethyl); K6(iTRAC	1	1
[K].aHLDIAGSLEGHLR.[F]	N-Term(iTRAQ4plex)	1	1
[K].sLHMYANR.[L]	N-Term(iTRAQ4plex)	1	1
[K].SVGFHLPSR.[E]		1	1
[R].sPSQADINk.[I]	N-Term(iTRAQ4plex); K9(iTRAQ4	1	1
[K].SGSSTASWIQNVDTk.[Y]	K15(iTRAQ4plex)	1	1
[K].iAIANIIDEIIEk.[L]	N-Term(iTRAQ4plex); K13(iTRAQ	1	1
[K].IEDGTLASk.[T]	K9(iTRAQ4plex)	1	1
[R].eIFNMAR.[D]	N-Term(iTRAQ4plex)	1	1
[K].mGLAFESTk.[S]	N-Term(iTRAQ4plex); K9(iTRAQ4	1	1
[K].mTSNFPVDLSDYPk.[S]	N-Term(iTRAQ4plex); K14(iTRAQ	1	1
[K].yEGLQEWEGk.[A]	N-Term(iTRAQ4plex); K10(iTRAQ	1	1
[R].vkYTLNk.[N]	N-Term(iTRAQ4plex); K2(iTRAQ4	1	1
[K].yHWEHTGLTLR.[E]	N-Term(iTRAQ4plex)	1	1
[K].qAEAVLk.[T]	N-Term(iTRAQ4plex); K7(iTRAQ4	1	1
[R].SEILAHWSPAk.[L]	K11(iTRAQ4plex)	1	1
[K].mGLAFESTk.[S]	N-Term(iTRAQ4plex); K9(iTRAQ4	1	1
[K].eQHLFLPFSYk.[N]	N-Term(iTRAQ4plex); K11(iTRAQ	1	1
[K].sQAIATk.[K]	N-Term(iTRAQ4plex); K7(iTRAQ4	1	1
[K].eSQLPTVMDFR.[K]	N-Term(iTRAQ4plex)	1	1
[K].sLHmYANR.[L]	N-Term(iTRAQ4plex); M4(Oxidat	1	1

[K].iTEVALMGHLScDTk.[E]	N-Term(iTRAQ4plex); C12(Carbar	1	1
[K].MTSNFPVDLSDYPk.[S]	K14(iTRAQ4plex)	1	1
[K].lkQHIEAIDVR.[V]	N-Term(iTRAQ4plex); K2(iTRAQ4	1	1
[K].NIILPVYDk.[S]	K9(iTRAQ4plex)	1	1
[R].eFQVPTFTIPk.[L]	N-Term(iTRAQ4plex); K11(iTRAQ	1	1
[K].VEDIPLAR.[I]		1	1
[R].QIDDIDVR.[F]		1	1
[R].vAWHYDEEk.[I]	N-Term(iTRAQ4plex); K9(iTRAQ4	1	1
[R].kGNVATEISTER.[D]	N-Term(iTRAQ4plex)	1	1
[K].aVSmPSFSILGSDVR.[V]	N-Term(iTRAQ4plex); M4(Oxidat	1	1
[R].kGNVATEISTER.[D]	K1(iTRAQ4plex)	1	1
[R].eSDEETQIk.[V]	N-Term(iTRAQ4plex); K9(iTRAQ4	1	1
[K].aTVAVYLESLQDTk.[I]	N-Term(iTRAQ4plex); K14(iTRAQ	1	1
[R].mYQMDIQQELQR.[Y]	N-Term(iTRAQ4plex)	1	1
[K].mGLAFESTk.[S]	N-Term(iTRAQ4plex); M1(Oxidat	1	1
[K].iISDYHQQFR.[Y]	N-Term(iTRAQ4plex)	1	1
[K].qTEATMTFk.[Y]	N-Term(iTRAQ4plex); K9(iTRAQ4	1	1
[K].nPNGYSFSIPVk.[V]	N-Term(iTRAQ4plex); K12(iTRAQ	1	1
[K].wNFYYSPQSSPDkk.[L]	N-Term(iTRAQ4plex); K13(iTRAQ	1	1
[R].vSTAFVYTk.[N]	N-Term(iTRAQ4plex); K9(iTRAQ4	1	1
[K].aTVAVYLESLQDTk.[I]	N-Term(iTRAQ4plex); K14(iTRAQ	1	1
[K].iDDIWNLEVk.[E]	N-Term(iTRAQ4plex); K10(iTRAQ	1	1
[R].TLADLTLLDSPIk.[V]	K13(iTRAQ4plex)	1	1
[K].NSEEFAAAMSR.[Y]		1	1
[K].ALVEQGFTVPEIk.[T]	K13(iTRAQ4plex)	1	1
[K].dNVFDGLVR.[V]	N-Term(iTRAQ4plex)	1	1
[K].mGLAFESTk.[S]	N-Term(iTRAQ4plex); K9(iTRAQ4	1	1
[K].MGLAFESTk.[S]	K9(iTRAQ4plex)	1	1
[K].aHLDIAGSLEGHLR.[F]	N-Term(iTRAQ4plex)	1	1
[R].eELcTMFIR.[E]	N-Term(iTRAQ4plex); C4(Carbam	1	1
[K].aTGVLYDYVNk.[Y]	N-Term(iTRAQ4plex); K11(iTRAQ	1	1
[K].sLHMYANR.[L]	N-Term(iTRAQ4plex)	1	1
[K].fPEVDVLTk.[Y]	N-Term(iTRAQ4plex); K9(iTRAQ4	1	1
[R].nLQNNAEWVYQGAIR.[Q]	N-Term(iTRAQ4plex)	1	1
[R].dFSAEYEEDGk.[Y]	N-Term(iTRAQ4plex); K11(iTRAQ	1	1
[K].lLSGGNTLHLVSTTk.[T]	N-Term(iTRAQ4plex); K15(iTRAQ	1	1
[K].aTGVLYDYVNk.[Y]	N-Term(iTRAQ4plex); K11(iTRAQ	1	1
[K].mTSNFPVDLSDYPk.[S]	N-Term(iTRAQ4plex); M1(Oxidat	1	1
[K].tEVIPPLIENR.[Q]	N-Term(iTRAQ4plex)	1	1
[R].rNLQNNAEWVYQGAIR.[Q]	N-Term(iTRAQ4plex)	1	1
[K].sLHmYANR.[L]	N-Term(iTRAQ4plex); M4(Oxidat	1	1
[K].sQAIATk.[K]	N-Term(iTRAQ4plex); K7(iTRAQ4	1	1
[K].aSGSLPYTQTLQDHLNSLk.[E]	N-Term(iTRAQ4plex); K19(iTRAQ	1	1
[R].eVGTVLSQVYSk.[V]	N-Term(iTRAQ4plex); K12(iTRAQ	1	1
[R].dFSAEYEEDGkYEGLQEWEGk.[A]	N-Term(iTRAQ4plex); K11(iTRAQ	1	1
[K].lkQHIEAIDVR.[V]	N-Term(iTRAQ4plex); K2(iTRAQ4	1	1
[K].fPEVDVLTk.[Y]	N-Term(iTRAQ4plex); K9(iTRAQ4	1	1
[R].FFGEGTk.[K]	K7(iTRAQ4plex)	1	1
[R].eSDEETQIk.[V]	N-Term(iTRAQ4plex); K9(iTRAQ4	1	1
[R].iYSLWEHSTk.[N]	N-Term(iTRAQ4plex); K10(iTRAQ	1	1
[K].qTEATmTFk.[Y]	N-Term(iTRAQ4plex); M6(Oxidat	1	1
[K].nLTDFAEQYSIQDWAk.[R]	N-Term(iTRAQ4plex); K16(iTRAQ	1	1

[R].dAVEkPQEFTIVAFVk.[Y]	N-Term(iTRAQ4plex); K5(iTRAQ4	1	1
[RK].aAIQALR.[RK]	N-Term(iTRAQ4plex)	1	1
[K].iEGNLIFDPNNYLPk.[E]	N-Term(iTRAQ4plex); K15(iTRAQ	1	1
[K].sHDELPR.[T]	N-Term(iTRAQ4plex)	1	1
[K].lIVAMSSWLQk.[A]	N-Term(iTRAQ4plex); K11(iTRAQ	1	1
[K].aVSMPSFSILGSDVR.[V]	N-Term(iTRAQ4plex)	1	1
[K].qTEATmTFk.[Y]	N-Term(iTRAQ4plex); M6(Oxidat	1	1
[R].TGISPLALIk.[G]	K10(iTRAQ4plex)	1	1
[K].lQSTTVMNPYMk.[L]	N-Term(iTRAQ4plex); K12(iTRAQ	1	1
[K].sHDELPR.[T]	N-Term(iTRAQ4plex)	1	1
[K].dNVFDGLVR.[V]	N-Term(iTRAQ4plex)	1	1
[R].eSDEETQIk.[V]	N-Term(iTRAQ4plex); K9(iTRAQ4	1	1
[R].VRESDEETQIk.[V]	K11(iTRAQ4plex)	1	1
[R].vPQTDMTFR.[H]	N-Term(iTRAQ4plex)	1	1
[K].dkAQNLYQELLTQEGQASFQGLk.[D]	N-Term(iTRAQ4plex); K2(iTRAQ4	1	1
[K].vEDIPLAR.[I]	N-Term(iTRAQ4plex)	1	1
[K].fVTQAEGAk.[Q]	N-Term(iTRAQ4plex); K9(iTRAQ4	1	1
[K].sVGFHLPSR.[E]	N-Term(iTRAQ4plex)	1	1
[K].hVAEAIck.[E]	N-Term(iTRAQ4plex); C7(Carbam	1	1
[K].yHWEHTGLTLR.[E]	N-Term(iTRAQ4plex)	1	1
[R].sPSQADINk.[I]	N-Term(iTRAQ4plex); K9(iTRAQ4	1	1
[R].eSDEETQIk.[V]	N-Term(iTRAQ4plex); K9(iTRAQ4	1	1
[R].kGNVATEISTER.[D]	K1(iTRAQ4plex)	1	1
[R].kGNVATEISTER.[D]	N-Term(iTRAQ4plex)	1	1
[K].sVGFHLPSR.[E]	N-Term(iTRAQ4plex)	1	1
[K].iAELSATAQEIIk.[S]	N-Term(iTRAQ4plex); K13(iTRAQ	1	1
[K].fPEVDVLTk.[Y]	N-Term(iTRAQ4plex); K9(iTRAQ4	1	1
[K].lSLPDFk.[E]	N-Term(iTRAQ4plex); K7(iTRAQ4	1	1
[K].iAELSATAQEIIk.[S]	N-Term(iTRAQ4plex); K13(iTRAQ	1	1
[K].sLWDFLk.[L]	N-Term(iTRAQ4plex); K7(iTRAQ4	1	1
[K].eNFAGEATLQR.[I]	N-Term(iTRAQ4plex)	1	1
[K].sLHmYANR.[L]	N-Term(iTRAQ4plex); M4(Oxidat	1	1
[K].fPEVDVLTk.[Y]	N-Term(iTRAQ4plex); K9(iTRAQ4	1	1
[K].nMEVSVATTTk.[A]	N-Term(iTRAQ4plex); K11(iTRAQ	1	1
[R].eSDEETQIk.[V]	N-Term(iTRAQ4plex); K9(iTRAQ4	1	1
[K].skEVPEAR.[A]	N-Term(iTRAQ4plex); K2(iTRAQ4	1	1
[K].sLHMYANR.[L]	N-Term(iTRAQ4plex)	1	1
[R].vSTAFVYTk.[N]	N-Term(iTRAQ4plex); K9(iTRAQ4	1	1
[K].iVQILPWEQNEQVk.[N]	N-Term(iTRAQ4plex); K14(iTRAQ	1	1
[K].lLSGGNTLHLVSTTk.[T]	N-Term(iTRAQ4plex); K15(iTRAQ	1	1
[R].eELcTMFIR.[E]	N-Term(iTRAQ4plex); C4(Carbam	1	1
[R].kYTYNYEAESSSGVPGTADSR.[S]	N-Term(iTRAQ4plex); K1(iTRAQ4	1	1
[K].aHLDIAGSLEGHLR.[F]	N-Term(iTRAQ4plex)	1	1
[K].HINIDQFVR.[K]		1	1
[R].eFQVPTFTIPk.[L]	N-Term(iTRAQ4plex); K11(iTRAQ	1	1
[K].dkDQEVLLQTFLDDASPGDk.[R]	N-Term(iTRAQ4plex); K2(iTRAQ4	1	1
[R].kGNVATEISTER.[D]	N-Term(iTRAQ4plex)	1	1
[R].kGNVATEISTER.[D]	K1(iTRAQ4plex)	1	1
[K].sGSSTASWIQNVDTk.[Y]	N-Term(iTRAQ4plex); K15(iTRAQ	1	1
[K].iISDYHQQFR.[Y]	N-Term(iTRAQ4plex)	1	1
[K].aHLDIAGSLEGHLR.[F]	N-Term(iTRAQ4plex)	1	1
[R].mYQMDIQQELQR.[Y]	N-Term(iTRAQ4plex)	1	1

[K].qAEAVLk.[T]	N-Term(iTRAQ4plex); K7(iTRAQ4	1	1
[R].hIQNIDIQHLAGk.[L]	N-Term(iTRAQ4plex); K13(iTRAQ4	1	1
[R].eSDEETQIk.[V]	N-Term(iTRAQ4plex); K9(iTRAQ4	1	1
[K].tSQcTLk.[E]	N-Term(iTRAQ4plex); C4(Carbam	1	1
[R].eELcTMFIR.[E]	N-Term(iTRAQ4plex); C4(Carbam	1	1
[K].iEDGTLASk.[T]	N-Term(iTRAQ4plex); K9(iTRAQ4	1	1
[R].sPSQADINk.[I]	N-Term(iTRAQ4plex); K9(iTRAQ4	1	1
[K].kLQSTTVMNPYMk.[L]	N-Term(iTRAQ4plex); K1(iTRAQ4	1	1
[K].nmEVSVATTTk.[A]	N-Term(iTRAQ4plex); M2(Oxidat	1	1
[K].sQAIATk.[K]	N-Term(iTRAQ4plex); K7(iTRAQ4	1	1
[K].sLHmYANR.[L]	N-Term(iTRAQ4plex); M4(Oxidat	1	1
[R].iPSVQINFk.[D]	N-Term(iTRAQ4plex); K9(iTRAQ4	1	1
[K].tSQcTLk.[E]	N-Term(iTRAQ4plex); C4(Carbam	1	1
[K].iVQILPWEQNEQVk.[N]	N-Term(iTRAQ4plex); K14(iTRAQ4	1	1
[K].kLTISEQNIQR.[A]	N-Term(iTRAQ4plex); K1(iTRAQ4	1	1
[K].tQFNNNEYSQDLDAYNTk.[D]	N-Term(iTRAQ4plex); K18(iTRAQ4	1	1
[K].eSQLPTVmDFR.[K]	N-Term(iTRAQ4plex); M8(Oxidat	1	1
[K].kIISDYHQQFR.[Y]	N-Term(iTRAQ4plex); K1(iTRAQ4	1	1
[K].lHVAGNLk.[G]	N-Term(iTRAQ4plex); K8(iTRAQ4	1	1
[R].lTLDIQNk.[K]	N-Term(iTRAQ4plex); K8(iTRAQ4	1	1
[R].kYTYNYEAESSSGVPGTADSR.[S]	N-Term(iTRAQ4plex); K1(iTRAQ4	1	1
[K].dSYDLHDLk.[I]	N-Term(iTRAQ4plex); K9(iTRAQ4	1	1
[K].hINIDQFVR.[K]	N-Term(iTRAQ4plex)	1	1
[K].qVFLYPEk.[D]	N-Term(iTRAQ4plex); K8(iTRAQ4	1	1
[R].kYTYNYEAESSSGVPGTADSR.[S]	K1(iTRAQ4plex)	1	1
[R].kYTYNYEAESSSGVPGTADSR.[S]	N-Term(iTRAQ4plex)	1	1
[K].aHLDIAGSLEGHLR.[F]	N-Term(iTRAQ4plex)	1	1
[K].mTSNFPVDLSDYPk.[S]	N-Term(iTRAQ4plex); M1(Oxidat	1	1
[K].dkDQEVLLQTFLDDASPGDk.[R]	N-Term(iTRAQ4plex); K2(iTRAQ4	1	1
[R].sPSQADINk.[I]	N-Term(iTRAQ4plex); K9(iTRAQ4	1	1
[K].lSNVLQQVk.[I]	N-Term(iTRAQ4plex); K9(iTRAQ4	1	1
[R].vIGNMGQTmEQLTPELk.[S]	N-Term(iTRAQ4plex); M9(Oxidat	1	1
[K].tSQcTLk.[E]	N-Term(iTRAQ4plex); C4(Carbam	1	1
[R].lTLDIQNk.[K]	N-Term(iTRAQ4plex); K8(iTRAQ4	1	1
[K].nFVASHIANILNSEELDIQDLk.[K]	N-Term(iTRAQ4plex); K22(iTRAQ4	1	1
[R].eVGTVLSQVYSk.[V]	N-Term(iTRAQ4plex); K12(iTRAQ4	1	1
[K].sQAIATk.[K]	N-Term(iTRAQ4plex); K7(iTRAQ4	1	1
[R].QIDDIDVR.[F]		1	1
[K].sLHMYANR.[L]	N-Term(iTRAQ4plex)	1	1
[R].lELELRPTGEIEQYSVSATYELQR.[E]	N-Term(iTRAQ4plex)	1	1

Protein Acc	# Missed Cle	DeltaScore	m/z [Da]	MH+ [Da]	DeltaM [ppm	Deltam/z [D	Isolation In
P02042; P6	0	1	424.2276	1270.668	1.66	0.0007	6.509261
P02042; P6	0	0.9136	479.9645	1437.879	0.68	0.00033	15.47896
P02042; P6	0	0.883	719.4435	1437.88	1.26	0.00091	7.142306
P02042; P6	0	0.825	479.9647	1437.88	1.06	0.00051	34.55009
P02042; P6	0	0.9794	647.3928	1293.778	1.79	0.00116	26.37509
P02042; P6	0	0.5	414.2438	1240.717	2.42	0.001	30.8405
P02100; P0	0	0.9348	709.9196	1418.832	2.96	0.0021	14.76605
P02100; P0	0	0.9286	709.9183	1418.829	1.16	0.00082	31.45067
P02042; P6	0	0.9892	653.3716	1958.1	2.79	0.00182	13.43204
P02042; P6	0	1	424.2276	1270.668	1.8	0.00076	0
P02042	0	0.9767	416.584	1247.737	1.84	0.00076	9.137834
P02042; P6	0	1	635.8383	1270.669	2.51	0.0016	11.28336
P02042; P6	0	1	431.931	1293.778	1.94	0.00084	37.24517
P02100; P0	0	1	637.8677	1274.728	1.97	0.00125	41.66899
P02042	0	1	700.8832	1400.759	-1.66	-0.00116	29.07772
B9A064; P0	0	1	729.7221	2187.152	4.67	0.0034	7.072478
B9A064; P0	0	1	678.0274	2032.068	2.32	0.00157	15.40447
B9A064; P0	0	1	678.0273	2032.067	2.23	0.00151	14.60128
B9A064; P0	0	1	777.7546	2331.249	2.46	0.00191	7.245492
B9A064; P0	0	1	777.755	2331.25	3.01	0.00234	20.78843
B9A064; P0	0	0.962	667.3279	1999.969	2.89	0.00192	11.1451
B9A064; P0	0	0.9583	667.3274	1999.968	2.15	0.00144	9.356668
B9A064; P0	0	1	729.7219	2187.151	4.5	0.00328	10.86865
B9A064; P0	0	0.9375	729.7202	2187.146	2.07	0.00151	0.969161
B9A064; P0	0	1	500.7472	1999.967	1.75	0.00088	32.04743
B9A064	0	0.7949	565.3661	1129.725	5.84	0.0033	10.01449
B9A064	0	0.7568	565.3661	1129.725	5.84	0.0033	0
B9A064	0	0.8947	377.2455	1129.722	3.26	0.00123	9.129904
B9A064; P0	0	1	667.3258	1999.963	-0.23	-0.00015	17.3943
B9A064; P0	0	0.8571	678.0297	2032.075	5.74	0.00389	0
Q02325; P0	0	1	513.2464	1537.725	1.77	0.00091	8.781481
Q02325; P0	0	1	769.3662	1537.725	2.02	0.00155	3.467448
Q02325; P0	0	1	513.2459	1537.723	0.82	0.00042	28.60907
Q02325; P0	0	0.9351	761.3681	1521.729	1.26	0.00096	3.723963
Q02325; P0	0	0.875	507.9149	1521.73	1.98	0.001	3.228438
Q02325; P0	0	0.8372	507.9146	1521.729	1.43	0.00073	11.80114
Q02325; P0	0	1	513.2466	1537.725	2.13	0.00109	6.229238
Q02325; Q1	0	0.8	584.823	1168.639	2.92	0.00171	12.57676
Q02325; P0	0	0.8824	689.3164	1377.626	0.43	0.0003	0
Q02325; Q1	0	0.5833	390.2174	1168.638	2.1	0.00082	27.11907
Q02325; Q1	0	0.8182	584.8233	1168.639	3.44	0.00201	7.360862
Q02325; P0	0	0.9756	689.3164	1377.625	0.34	0.00023	17.78435
Q02325; P0	0	1	513.2463	1537.724	1.65	0.00085	12.06906
Q02325; Q1	0	0.5833	390.2172	1168.637	1.55	0.00061	10.91142
Q02325; P0	0	1	513.2466	1537.725	2.25	0.00115	59.04612
Q02325; Q1	0	0.44	390.2168	1168.636	0.38	0.00015	14.30095
P0DJI8	0	1	774.6933	2322.065	-0.1	-0.00008	11.12954
P0DJI8	0	1	774.695	2322.07	2.03	0.00157	4.701438
P0DJI9; P0D	0	0.9733	847.921	1694.835	3.12	0.00264	12.78635
P0DJI8	0	1	774.6944	2322.069	1.32	0.00102	0
P35443; P4	0	0.975	675.0043	2022.998	3.06	0.00206	4.581845

P35443; P4	0	1	461.7607	1844.021	0.61	0.00028	43.29137
P35443	0	0.9821	708.9905	2124.957	2.77	0.00196	3.831197
P35443	0	0.7097	583.9708	1749.898	6.37	0.00372	23.27845
P35443; P4	0	0.8095	657.4075	1313.808	3.48	0.00228	6.450108
P02771	1	1	383.9939	1532.954	2.02	0.00077	0
P02771	1	1	511.6558	1532.953	1.49	0.00076	11.12538
P02771	1	1	511.6551	1532.951	0.18	0.00009	13.27279
P02771	0	1	459.6222	1376.852	1.82	0.00083	30.62461
P36980; Q0	0	0.8036	492.2609	1474.768	1.51	0.00074	4.42155
P36980; Q0	0	1	540.294	1618.867	-0.26	-0.00014	18.41137
P36980; Q0	0	1	540.2943	1618.868	0.2	0.00011	29.04418
P36980	0	0.3617	512.3002	1023.593	0.61	0.00031	3.62429
P36980	0	1	588.6351	1763.891	1.82	0.00107	40.7585
Q9UJJ9	0	1	1021.808	3063.408	3.76	0.00384	5.008028
Q9UJJ9	0	0.8182	532.8058	1064.604	2.88	0.00153	31.97983
P08294	0	1	492.9486	1476.831	2.19	0.00108	8.144977
P08294	0	1	540.9828	1620.934	2.23	0.0012	14.37619
P08294	0	1	494.6007	1481.787	3.05	0.00151	0
P08294	0	0.8462	494.6	1481.785	1.75	0.00087	5.127842
P41222	0	1	733.3936	2198.166	3.55	0.0026	33.7404
P41222	1	1	447.5964	1340.775	-0.49	-0.00022	47.1739
Q15166	0	0.8654	439.2838	1315.837	2.05	0.0009	9.680024
Q15166	0	0.8333	556.3351	1111.663	2.6	0.00144	14.96833
Q15166	0	1	391.2498	1171.735	2.45	0.00096	43.53766
Q15166	0	0.6552	658.4205	1315.834	-0.16	-0.00011	21.87804
Q15166	0	1	439.2833	1315.835	0.94	0.00041	33.67272
P04070	0	0.9677	585.3236	1753.956	3.06	0.00179	45.92674
P04070	1	0.5405	630.0246	1888.059	3.27	0.00206	20.702
P04070	1	0.6562	630.0234	1888.056	1.33	0.00084	10.52519
P04070	1	0.5429	630.0241	1888.058	2.4	0.00151	46.83348
P04070	0	1	585.3239	1753.957	3.58	0.0021	6.390798
P04070	1	0.84	630.0242	1888.058	2.69	0.00169	25.22333
P04070	1	0.6957	630.0237	1888.057	1.92	0.00121	18.22969
P04070	0	0.2105	439.2436	1753.952	0.86	0.00038	30.03496
P61769	0	0.9104	705.9202	1410.833	1.66	0.00117	0
P61769	0	1	479.5921	1436.762	-0.36	-0.00017	32.90707
P61769	0	0.9636	633.8691	1266.731	1.86	0.00118	0
P00918	0	1	472.9681	1416.89	-0.05	-0.00002	29.17554
P00918	0	0.7692	638.3926	1275.778	1.4	0.00089	0
Q9BXR6; Q(	0	1	598.6377	1793.899	0.19	0.00011	33.30711
Q9BXR6	0	0.8824	542.0378	2165.129	1.38	0.00075	12.74582
Q9BXR6	0	0.8571	501.8317	1002.656	1.2	0.0006	70.55062
Q76LX8	0	0.7742	427.9241	1281.758	2.38	0.00102	15.54964
Q76LX8	0	1	536.3162	1606.934	-3.48	-0.00186	41.31217
P33151	0	1	652.7131	1956.125	2.98	0.00194	18.02342
P33151	0	0.9118	522.6035	1565.796	2.06	0.00108	20.3293
Q13740	0	1	638.6848	1914.04	1.76	0.00112	38.91248
Q13740	0	1	544.2796	1630.824	2.45	0.00133	0
P63267; P6	0	1	487.2637	1459.777	0.94	0.00046	46.31744
A5A3E0; P6	0	0.8636	645.6713	1934.999	2.72	0.00175	55.86254
P08709	0	0.6364	527.9681	1581.89	1.13	0.0006	10.08972
P08709	0	1	874.8304	2622.477	1.16	0.00102	42.82035

P00441	0	0.5	505.279	1513.822	0.7	0.00035	69.69789
P00441	0	0.9524	573.3288	1145.65	2.99	0.00171	12.17622
Q99784	0	0.7576	386.5692	1157.693	1.76	0.00068	18.11504
Q99784	0	0.9655	473.2899	1417.855	3.35	0.00159	14.13693
A5A3E0; Q9	0	1	554.2729	1660.804	-0.37	-0.0002	38.75434
A5A3E0; Q9	0	1	505.9223	1515.752	2.11	0.00107	29.73994
A5A3E0; Q9	0	1	554.2729	1660.804	-0.37	-0.0002	38.75434
A5A3E0; Q9	0	1	505.9223	1515.752	2.11	0.00107	29.73994
P11021	0	0.9608	523.8276	1046.648	3.05	0.0016	5.012991
P11021	0	0.5405	423.9038	1269.697	3	0.00127	0
P02452	0	0.9	877.9328	1754.858	2.3	0.00202	8.353262
P02458; P0	0	0.4286	492.2642	1474.778	4.5	0.00221	0
P02458; P0	0	0.7143	492.264	1474.777	4	0.00197	20.83963
P02458; P0	0	1	492.2633	1474.775	2.57	0.00126	48.95316
P05062	0	0.9615	510.6231	1529.855	1.44	0.00073	58.29253
P05062	0	1	762.726	2286.163	0.28	0.00021	51.26673
P22105; Q1	0	1	584.3294	1750.974	3.98	0.00232	6.555482
P22105; Q1	0	0.6667	621.864	1242.721	2.59	0.00161	50.187
P06702	0	0.4167	420.5719	1259.701	1.99	0.00084	22.3853
P06702	1	1	479.9658	1437.883	2.76	0.00132	7.225609
P23470	0	0.75	523.3655	1045.724	1.29	0.00068	42.90685
P23470	0	1	481.9292	1443.773	2.84	0.00137	5.758438
P13598	1	0.5909	406.2717	1216.8	1.27	0.00051	61.84037
P13598	0	0.8333	623.4011	1868.189	3.4	0.00212	45.99354
Q9UNN8	0	1	396.5589	1187.662	-0.09	-0.00003	47.25684
Q9UNN8	0	1	484.6039	1451.797	1.47	0.00071	5.991768
P05109	0	0.9773	555.814	1110.621	1.86	0.00103	5.130662
P05109	0	0.7027	576.3337	1151.66	1.45	0.00084	25.93389
Q9Y4L1	0	0.6296	647.8856	1294.764	2.37	0.00153	40.0595
Q9Y4L1	1	1	486.2835	1456.836	1.44	0.0007	19.7084
P0C0L4; P0	0	0.88	814.4339	1627.861	2.17	0.00177	11.91346
P0C0L4; P0	0	0.72	543.2917	1627.861	2.17	0.00117	2.514847
P0C0L4; P0	0	0.9589	814.4331	1627.859	1.2	0.00098	6.002271
P0C0L4; P0	0	0.7451	543.2906	1627.857	0.14	0.00008	11.02971
P0C0L4; P0	0	0.9467	543.2921	1627.862	2.84	0.00154	8.938625
P0C0L4; P0	0	0.1429	440.0023	1756.987	0.38	0.00017	13.3462
P0C0L4; P0	1	0.8929	790.0978	2368.279	1.16	0.00092	9.614325
P0C0L4; P0	0	0.8214	543.2907	1627.857	0.25	0.00014	0
P0C0L4; P0	0	0.9559	610.679	1830.022	0.67	0.00041	8.518244
P0C0L4; P0	0	0.8901	915.5165	1830.026	2.58	0.00236	6.086547
P0C0L4; P0	0	0.8864	458.2622	1830.027	3.16	0.00145	2.335641
P0C0L4; P0	0	1	610.6807	1830.027	3.47	0.00212	17.67647
P0C0L4; P0	0	1	610.6804	1830.027	3.07	0.00187	9.608068
P0C0L4; P0	0	1	636.3515	1271.696	3.9	0.00248	14.22901
P0C0L4; P0	0	0.8824	459.9129	1377.724	1.79	0.00082	0
P0C0L4; P0	0	0.7368	557.8151	1114.623	1.21	0.00068	0
P0C0L4; P0	0	0.9608	636.3503	1271.693	1.98	0.00126	7.495775
P0C0L4; P0	1	0.7333	592.8256	2368.281	1.96	0.00116	2.025505
P0C0L4; P0	1	0.88	790.0981	2368.28	1.55	0.00122	4.007072
P0C0L4; P0	1	0.7407	592.8265	2368.284	3.5	0.00207	65.63628
P0C0L4; P0	1	0.963	790.0977	2368.279	1.09	0.00086	5.731484
P0C0L4; P0	1	0.9706	790.0981	2368.28	1.63	0.00129	6.935635

P0C0L4; P0	1	1	571.8116	2284.225	7.79	0.00445	20.25506
P0C0L4; P0	1	0.9118	762.0757	2284.213	2.61	0.00199	0
P0C0L4; P0	0	1	710.0423	2128.112	3.17	0.00225	84.79305
P0C0L4; P0	0	0.8197	615.872	1230.737	3.21	0.00198	13.4027
P0C0L4; P0	0	1	542.2908	1083.574	1.49	0.00081	1.305774
P0C0L4; P0	1	0.8049	470.6024	1409.793	3.44	0.00162	17.80892
P0C0L4; P0	0	0.7	481.956	1443.854	4.79	0.00231	13.23638
P0C0L4; P0	0	0.7015	662.3807	1323.754	2.28	0.00151	3.729706
P0C0L4; P0	0	0.6579	524.311	1570.918	1.83	0.00096	6.544328
P0C0L4; P0	1	0.9074	490.2613	1468.769	1.87	0.00091	25.50551
P0C0L4; P0	1	0.9074	490.2613	1468.769	1.87	0.00091	25.50551
P0C0L4; P0	1	1	490.2612	1468.769	1.8	0.00088	2.070895
P0C0L4; P0	1	1	490.2612	1468.769	1.8	0.00088	2.070895
P0C0L4; P0	0	1	904.4314	2711.28	2.11	0.00191	1.925776
P0C0L4; P0	0	0.8333	399.5598	1196.665	2.57	0.00103	10.67162
P0C0L4; P0	0	0.697	481.9547	1443.85	2.13	0.00102	11.48204
P0C0L4; P0	0	1	899.0993	2695.283	1.62	0.00145	1.309225
P0C0L4; P0	0	1	899.0989	2695.282	1.21	0.00108	2.199146
P0C0L4; P0	0	1	851.0645	2551.179	0.73	0.00062	0
P0C0L4; P0	0	0.9524	862.9702	1724.933	1.32	0.00114	7.43602
P0C0L4; P0	0	0.9444	575.6502	1724.936	2.9	0.00167	28.3975
P0C0L4; P0	0	0.9298	575.65	1724.936	2.69	0.00155	8.106833
P0C0L4; P0	0	0.962	862.971	1724.935	2.24	0.00193	1.706079
P0C0L4; P0	0	0.9268	575.6498	1724.935	2.37	0.00136	11.78024
P0C0L4; P0	0	0.8571	862.9707	1724.934	1.89	0.00163	10.14675
P0C0L4; P0	0	0.9815	575.6504	1724.937	3.33	0.00191	8.231376
P0C0L4; P0	0	0.8571	590.9779	1770.919	2.76	0.00163	31.60502
P0C0L4; P0	0	0.9104	590.9768	1770.916	0.8	0.00047	3.83896
P0C0L4; P0	0	0.8462	885.9625	1770.918	1.91	0.00169	3.769225
P0C0L4; P0	0	0.92	590.9765	1770.915	0.38	0.00023	4.631679
P0C0L4; P0	0	0.5714	468.5796	1403.724	0.71	0.00033	27.22315
P0C0L4; P0	0	0.987	702.3669	1403.726	2.39	0.00168	6.447455
P0C0L4; P0	0	0.9189	459.9125	1377.723	0.72	0.00033	9.700747
P0C0L4; P0	0	0.7647	599.3157	1197.624	0.75	0.00045	4.685958
P0C0L4; P0	0	0.9167	459.9125	1377.723	0.72	0.00033	3.849536
P0C0L4; P0	0	0.8649	459.9129	1377.724	1.79	0.00082	3.418623
P0C0L4; P0	1	1	490.2617	1468.771	2.74	0.00134	68.65987
P0C0L4; P0	0	0.9831	458.9499	1374.835	0.21	0.0001	8.918069
P0C0L4; P0	0	0.7882	687.9224	1374.838	2.07	0.00142	6.157514
P0C0L4; P0	1	1	490.2617	1468.771	2.74	0.00134	68.65987
P0C0L4; P0	0	1	458.9507	1374.838	2.14	0.00098	8.437518
P0C0L4; P0	0	0.9808	662.0079	1984.009	2.79	0.00184	19.66835
P0C0L4; P0	0	1	572.7972	2288.167	2.69	0.00154	16.94717
P0C0L4; P0	0	0.8723	722.4288	1443.85	2.62	0.00189	8.394596
P0C0L4; P0	0	1	481.955	1443.85	2.64	0.00127	18.88451
P0C0L4; P0	0	0.8706	790.413	2369.224	1.66	0.00131	7.247535
P0C0L4; P0	1	1	538.2988	1612.882	8.3	0.00446	28.51182
P0C0L4; P0	0	0.6596	576.7955	2304.16	2.01	0.00116	5.662117
P0C0L4; P0	1	1	571.8084	2284.212	2.23	0.00128	2.234328
P0C0L4; P0	1	1	762.0748	2284.21	1.33	0.00101	2.679379
P0C0L4; P0	1	1	571.808	2284.21	1.59	0.00091	2.213109
P0C0L4; P0	1	1	762.075	2284.21	1.65	0.00126	2.43145

P0C0L4; P0	0	1	634.368	1901.09	0.45	0.00029	7.470258
P0C0L4; P0	0	1	476.0281	1901.091	0.99	0.00047	7.908507
P0C0L4; P0	1	0.9792	762.0773	2284.217	4.7	0.00358	0
P0C0L4; P0	1	1	571.8112	2284.223	7.04	0.00402	14.00352
P0C0L4; P0	0	0.8525	409.8971	1227.677	1.47	0.0006	10.92853
P0C0L4; P0	0	0.8636	614.3425	1227.678	2.4	0.00147	3.654341
P0C0L4; P0	0	0.8621	409.8967	1227.676	0.65	0.00027	5.003325
P0C0L4; P0	0	1	1126.59	2252.174	2.53	0.00285	1.71125
P0C0L4; P0	0	1	751.3956	2252.172	1.88	0.00141	2.23579
P0C0L4; P0	0	1	1215.597	3644.776	1.73	0.0021	0
P0C0L4; P0	0	1	911.949	3644.774	1.14	0.00104	0.705887
P0C0L4; P0	0	0.7143	399.5596	1196.664	2.19	0.00087	8.869975
P0C0L4; P0	0	0.5517	524.3106	1570.917	1.13	0.00059	53.46223
P0C0L4; P0	0	0.9231	698.9091	1396.811	2.23	0.00156	6.511384
P0C0L4; P0	0	0.9855	617.0257	1849.063	2.77	0.00171	25.65826
P0C0L4; P0	0	1	617.0252	1849.061	1.88	0.00116	47.37478
P0C0L4; P0	0	0.94	733.3654	2198.082	-1.93	-0.00141	65.47301
P0C0L4; P0	0	1	695.02	2083.046	1.08	0.00075	0
P0C0L4; P0	0	1	617.025	1849.06	1.58	0.00097	19.06164
P0C0L4; P0	0	1	617.0253	1849.061	2.08	0.00128	6.541273
P0C0L4; P0	0	1	706.8581	2824.411	1.52	0.00107	3.352633
P0C0L4; P0	0	1	763.3923	2288.162	0.74	0.00057	4.858709
P0C0L4; P0	0	1	572.7972	2288.167	2.69	0.00154	3.311056
P0C0L4; P0	0	1	763.3929	2288.164	1.46	0.00112	3.275803
P0C0L4; P0	0	1	572.7966	2288.165	1.73	0.00099	1.890797
P0C0L4; P0	0	1	942.143	2824.414	2.91	0.00274	1.905259
P0C0L4; P0	0	1	942.1425	2824.413	2.32	0.00219	2.277306
P0C0L4; P0	0	1	572.7976	2288.168	3.33	0.0019	3.885201
P0C0L4; P0	0	1	706.8582	2824.411	1.6	0.00113	2.626854
P0C0L4; P0	1	0.9091	418.0074	1669.008	1.63	0.00068	17.1066
P0C0L4; P0	0	1	572.7955	2288.16	-0.3	-0.00017	0
P0C0L4; P0	1	1	557.0079	1669.009	2.51	0.0014	10.70534
P0C0L4; P0	0	1	710.0408	2128.108	1.02	0.00072	1.937721
P0C0L4; P0	1	0.8864	418.006	1669.002	-1.66	-0.00069	8.256634
P0C0L4; P0	1	0.9737	557.0076	1669.008	2.07	0.00115	13.86559
P0C0L4; P0	0	1	532.7825	2128.108	1.15	0.00061	25.97721
P0C0L4; P0	0	0.9375	710.0416	2128.11	2.22	0.00158	12.2241
P0C0L4; P0	0	0.8421	679.3927	1357.778	2.88	0.00195	12.50909
P0C0L4; P0	0	0.5957	453.2638	1357.777	1.99	0.0009	14.11089
P0C0L4; P0	0	0.86	629.8673	1258.727	2.91	0.00183	22.45838
P0C0L4; P0	0	0.8197	453.2637	1357.777	1.72	0.00078	6.476308
P0C0L4; P0	0	0.9286	679.392	1357.777	1.89	0.00128	4.755651
P0C0L4; P0	1	0.9787	557.0079	1669.009	2.51	0.0014	11.11647
P0C0L4; P0	0	1	710.0415	2128.11	2.05	0.00145	0
P0C0L4; P0	0	1	706.8581	2824.411	1.52	0.00107	1.25589
P0C0L4; P0	0	0.9821	463.0205	1849.06	1.44	0.00067	8.049833
P0C0L4; P0	0	0.8714	925.0349	1849.062	2.71	0.00251	5.728807
P0C0L4; P0	0	0.9275	617.0245	1849.059	0.89	0.00055	9.125571
P0C0L4; P0	0	1	463.0206	1849.061	1.77	0.00082	10.18817
P0C0L4; P0	0	1	925.0341	1849.061	1.85	0.00171	0
P0C0L4; P0	0	1	617.025	1849.06	1.58	0.00097	7.924266
P0C0L4; P0	0	0.902	617.0258	1849.063	2.97	0.00183	39.13517

P0C0L4; P0	0	0.8367	568.9914	1704.96	2.51	0.00143	6.909508
P0C0L4; P0	0	0.3689	1063.531	3188.578	1.4	0.00148	3.788276
P0C0L4; P0	0	0.3882	797.8998	3188.577	1.03	0.00082	3.799753
P0C0L5	0	1	797.3952	1593.783	1.24	0.00099	1.954859
P0C0L4; P0	0	1	710.0415	2128.11	2.05	0.00145	1.810176
P0C0L4; P0	0	0.7188	399.5593	1196.663	1.5	0.0006	23.17154
P0C0L5	0	0.8298	531.9325	1593.783	1.12	0.00059	44.23657
P0C0L4; P0	0	1	670.8411	1340.675	2.57	0.00172	4.709168
P0C0L4; P0	0	0.8298	629.8673	1258.727	3	0.00189	8.2719
P0C0L4; P0	0	0.8958	629.8668	1258.726	2.23	0.0014	32.32337
P0C0L4; P0	0	1	911.9504	3644.78	2.74	0.0025	5.921183
P0C0L4; P0	0	1	1215.599	3644.782	3.43	0.00417	0
P0C0L4; P0	0	0.9912	876.4736	2627.406	2.03	0.00178	2.412186
P0C0L4; P0	0	1	974.8275	2922.468	2.69	0.00262	0
P0C0L4; P0	0	1	926.7924	2778.363	1.6	0.00148	26.17738
P0C0L4; P0	0	1	1068.863	3204.576	2.1	0.00224	6.34409
P0C0L4; P0	0	1	733.3691	2198.093	3.24	0.00237	42.34372
P0C0L4; P0	0	1	1242.156	2483.305	2.4	0.00298	0
P0C0L4; P0	0	1	1314.207	2627.406	1.99	0.00261	0
P0C0L4; P0	0	0.9184	685.3339	2053.987	1.72	0.00117	2.506288
P0C0L4; P0	1	1	538.2955	1612.872	2.05	0.0011	19.50909
P0C0L4; P0	1	1	538.2947	1612.87	0.69	0.00037	38.42788
P0C0L4; P0	0	0.8095	409.8974	1227.678	2.29	0.00094	12.2222
P0C0L4; P0	0	0.7937	554.836	1108.665	3.85	0.00213	9.636339
P0C0L4; P0	1	0.9773	538.295	1612.871	1.26	0.00068	15.72817
P0C0L4; P0	1	0.9898	538.2946	1612.869	0.46	0.00025	8.549582
P0C0L4; P0	1	1	538.2954	1612.872	1.94	0.00104	9.702331
P0C0L4; P0	0	0.8	560.2935	1119.58	3.15	0.00177	11.31302
P0C0L4; P0	0	0.9333	610.6798	1830.025	1.97	0.0012	44.52637
P0C0L4; P0	1	0.9677	563.6411	1688.909	1.81	0.00102	4.968037
P0C0L4; P0	0	1	1209.052	2417.098	-0.21	-0.00025	0
P0C0L4; P0	0	1	806.3714	2417.1	0.59	0.00048	3.273516
P0C0L5	0	0.9059	526.6008	1577.788	1.08	0.00057	5.473201
P0C0L5	0	0.9451	789.3973	1577.787	0.66	0.00052	2.945702
P0C0L4; P0	0	1	733.3657	2198.083	-1.43	-0.00105	36.81449
P0C0L4; P0	0	1	733.3678	2198.089	1.4	0.00103	14.15553
P0C0L4; P0	0	0.9783	733.3682	2198.09	1.9	0.00139	11.64252
P0C0L4; P0	0	1	733.3679	2198.089	1.49	0.00109	8.001216
P0C0L4; P0	0	1	629.9674	1887.888	1.43	0.0009	4.39021
P0C0L4; P0	0	0.9048	944.447	1887.887	0.89	0.00084	3.799334
P0C0L4; P0	0	0.9672	629.9689	1887.892	3.86	0.00243	21.66053
P0C0L4; P0	0	1	723.0315	2167.08	1.69	0.00122	1.818813
P0C0L5	1	1	749.9358	2996.721	2.61	0.00196	23.85591
P0C0L4; P0	0	0.7586	669.8612	1338.715	0.5	0.00034	6.654665
P0C0L4; P0	0	0.9747	854.4799	1707.952	0.85	0.00072	3.551121
P0C0L4; P0	0	1	569.9898	1707.955	2.16	0.00123	5.385142
P0C0L4; P0	0	0.9722	568.9744	1704.909	2.28	0.00129	5.693548
P0C0L4; P0	0	1	568.9751	1704.911	3.56	0.00203	9.272164
P0C0L4; P0	0	0.9672	852.9576	1704.908	1.96	0.00167	6.551302
P0C0L4; P0	0	1	568.9751	1704.911	3.56	0.00203	13.15989
P0C0L4; P0	0	1	782.4294	1563.851	1.63	0.00128	0
P0C0L4; P0	0	0.9474	568.9741	1704.908	1.85	0.00105	3.56933

P0C0L4; P0	0	0.9184	852.9573	1704.907	1.53	0.00131	6.473444
P0C0L4; P0	0	0.9362	780.9066	1560.806	2.07	0.00162	7.603855
P0C0L4; P0	0	1	568.9749	1704.91	3.14	0.00178	9.686429
P0C0L4; P0	0	0.9762	568.975	1704.911	3.46	0.00196	10.09111
P0C0L4; P0	0	1	816.5032	1631.999	1	0.00082	10.48337
P0C0L4; P0	0	0.9677	790.4136	2369.226	2.43	0.00192	6.811904
P0C0L4; P0	0	1	481.9554	1443.852	3.52	0.0017	0
P0C0L4; P0	0	0.7714	737.9129	1474.819	1.17	0.00086	4.098854
P0C0L4; P0	0	0.9333	492.2779	1474.819	1.57	0.00077	5.518109
P0C0L4; P0	0	0.9157	542.9428	1626.814	1.02	0.00056	0.859662
P0C0L4; P0	0	1	763.4246	2288.259	1.75	0.00134	30.67124
P0C0L4; P0	1	0.9677	621.8533	2484.391	2.4	0.00149	4.806307
P0C0L4; P0	0	1	592.3666	1775.085	2.33	0.00138	3.065492
P0C0L4; P0	0	1	791.7968	2373.376	0.63	0.0005	7.553562
P0C0L4; P0	0	0.875	594.0998	2373.377	1.24	0.00074	27.68506
P0C0L5	0	0.9796	649.1345	3241.644	2.37	0.00154	10.06449
P0C0L4; P0	0	0.7143	474.583	1421.734	3.31	0.00157	25.12729
P0C0L4; P0	0	0.988	711.371	1421.735	3.6	0.00256	7.464592
P0C0L4; P0	0	0.9434	474.5831	1421.735	3.5	0.00166	9.031528
P0C0L5	0	1	811.1661	3241.642	2.06	0.00167	3.946628
P0C0L4; P0	0	1	619.5472	3093.707	1.91	0.00118	20.76154
P0C0L4; P0	0	0.9189	442.9264	1326.765	1.94	0.00086	9.391974
P0C0L4; P0	0	0.9737	569.9888	1707.952	0.45	0.00025	31.71992
P0C0L4; P0	0	0.8736	663.8861	1326.765	2.25	0.00149	5.722034
P0C0L4; P0	0	0.625	442.9256	1326.762	0.28	0.00012	9.223516
P0C0L4; P0	0	1	774.1818	3093.705	1.4	0.00108	3.361939
P0C0L4; P0	0	0.8333	442.9268	1326.766	2.83	0.00125	22.21581
P0C0L4; P0	0	0.7561	442.9264	1326.765	2.01	0.00089	0
P0C0L4; P0	0	0.8197	492.2781	1474.82	1.88	0.00092	42.95472
P0C0L4; P0	0	0.8627	641.3524	1281.698	3.65	0.00234	12.25893
P0C0L4; P0	0	1	639.3195	1277.632	3.15	0.00201	5.319107
P0C0L4; P0	0	0.8889	562.6464	1685.925	3.27	0.00184	32.79515
P0C0L4; P0	0	1	598.7885	1196.57	2.7	0.00162	4.36656
P0C0L4; P0	0	1	598.7878	1196.568	1.68	0.00101	3.423467
P0C0L4; P0	0	1	744.4514	1487.895	0.04	0.00003	14.81127
P0C0L4; P0	0	1	644.3508	2574.381	0.99	0.00064	0.879733
P0C0L4; P0	0	1	598.7885	1196.57	2.7	0.00162	3.088728
P0C0L4; P0	0	1	598.7882	1196.569	2.3	0.00137	3.379773
P0C0L4; P0	0	1	516.6178	1547.839	3.04	0.00157	8.099505
P0C0L4; P0	1	0.8889	518.6359	1553.893	2.02	0.00105	11.67609
P0C0L4; P0	1	0.92	518.6356	1553.892	1.55	0.0008	20.36515
P0C0L4; P0	0	0.8654	641.3528	1281.698	4.22	0.00271	36.5496
P0C0L4; P0	0	1	598.7875	1196.568	1.07	0.00064	3.110818
P0C0L4; P0	0	1	598.789	1196.571	3.62	0.00217	0
P0C0L4; P0	0	1	598.7886	1196.57	3.01	0.0018	3.209586
P0C0L4; P0	0	1	598.7885	1196.57	2.7	0.00162	3.526876
P0C0L4; P0	0	0.9508	636.3505	1271.694	2.27	0.00144	100
P0C0L4; P0	0	1	919.8451	2757.521	1.91	0.00175	5.724564
P0C0L4; P0	0	0.9091	581.1309	2901.626	2.74	0.00159	4.158038
P0C0L4; P0	0	1	680.3757	2718.481	0.04	0.00002	2.192481
P0C0L4; P0	0	1	544.5027	2718.484	1.31	0.00071	3.460773
P0C0L4; P0	0	1	680.3768	2718.485	1.65	0.00112	2.076645

P0C0L4; P0	0	1	1029.58	3086.725	2.5	0.00258	3.345291
P0C0L4; P0	0	1	772.4356	3086.721	1.21	0.00094	5.352506
P0C0L4; P0	0	0.9778	568.975	1704.911	3.46	0.00196	8.411469
P0C0L4; P0	0	1	865.6581	4324.262	2.09	0.00181	2.478431
P0C0L4; P0	0	1	568.975	1704.911	3.46	0.00196	11.15509
P0C0L4; P0	0	0.975	568.9753	1704.911	3.99	0.00227	10.76766
P0C0L4; P0	0	0.9574	568.9755	1704.912	4.32	0.00245	12.43042
P0C0L4; P0	0	1	1081.82	4324.259	1.58	0.00171	2.319621
P0C0L4; P0	0	1	568.975	1704.91	3.35	0.0019	8.388101
P0C0L4; P0	0	1	568.975	1704.91	3.35	0.0019	7.974552
P0C0L4; P0	0	0.7067	610.3615	1219.716	1.65	0.00101	18.36178
P0C0L4; P0	0	0.7015	662.3806	1323.754	2.18	0.00145	5.777752
P0C0L4; P0	0	1	974.8261	2922.464	1.18	0.00115	2.592923
P0C0L4; P0	0	0.9846	1136.634	2272.262	0.5	0.00057	0
P0C0L4; P0	0	1	763.3895	1525.772	0.61	0.00047	8.361775
P0C0L4; P0	0	0.8571	691.3391	1381.671	1.48	0.00102	0
P0C0L4; P0	0	0.9121	758.0922	2272.262	0.68	0.00052	3.574229
P0C0L5	0	0.7632	531.9326	1593.783	1.23	0.00065	39.5839
P0C0L4; P0	1	0.697	470.6018	1409.791	2.15	0.00101	16.73803
P0C0L4; P0	0	0.9931	1314.205	2627.403	0.96	0.00127	0
P0C0L4; P0	0	1	729.1823	2913.707	0.96	0.0007	7.451555
P0C0L4; P0	0	1	971.9078	2913.709	1.43	0.00139	3.05197
P0C0L4; P0	0	1	592.3665	1775.085	2.02	0.0012	2.265197
P0C0L4; P0	1	0.9048	420.9138	1260.727	0.52	0.00022	17.07265
P0C0L4; P0	0	0.76	399.5595	1196.664	1.81	0.00072	67.22067
P0C0L4; P0	0	0.7143	468.5802	1403.726	2.14	0.001	12.54043
P0C0L4; P0	0	1	487.287	1459.846	3.35	0.00163	14.59365
P0C0L4; P0	0	0.1515	433.9207	1299.748	2.28	0.00099	19.51768
P0C0L4; P0	0	0.8611	852.9576	1704.908	1.96	0.00167	3.193368
P0C0L4; P0	0	0.95	463.0204	1849.06	1.18	0.00054	18.91823
P0C0L4; P0	0	1	598.788	1196.569	1.89	0.00113	3.521297
P0C0L4; P0	0	0.75	399.5589	1196.662	0.43	0.00017	6.72325
P0C0L5	0	0.8485	1033.185	3097.54	2.1	0.00217	1.242798
P0C0L4; P0	0	0.3429	885.9619	1770.917	1.29	0.00114	6.470485
P0C0L4; P0	0	0.8367	629.3591	1257.711	2.27	0.00143	10.64508
P0C0L4; P0	0	1	544.5025	2718.483	0.97	0.00053	29.77047
P0C0L4; P0	0	0.6286	463.0205	1849.06	1.51	0.0007	4.319591
P0C0L4; P0	0	1	399.5279	1196.569	2.29	0.00091	48.74908
P0C0L4; P0	0	0.6579	520.9397	1560.805	1.24	0.00065	3.713353
P0C0L4; P0	0	0.7436	468.5805	1403.727	2.79	0.00131	33.81586
P0C0L4; P0	0	0.8	441.9229	1323.754	2.36	0.00104	14.17325
P0C0L4; P0	0	0.8421	669.8644	1338.722	5.43	0.00363	17.00924
P0C0L4; P0	0	0.75	520.9398	1560.805	1.36	0.00071	4.419956
P0C0L4; P0	0	0.8182	409.8979	1227.679	3.48	0.00142	32.31134
P0C0L4; P0	0	1	899.1001	2695.286	2.5	0.00224	4.423165
P0C0L4; P0	0	0.9444	636.3513	1271.695	3.61	0.0023	100
P0C0L4; P0	0	0.9268	852.9576	1704.908	1.96	0.00167	6.797606
P0C0L4; P0	0	1	568.974	1704.907	1.63	0.00093	24.27141
P0C0L4; P0	1	1	421.0057	1681.001	0.65	0.00027	15.98904
P0C0L4; P0	0	1	542.2906	1083.574	1.04	0.00056	0
P0C0L4; P0	0	0.9535	602.325	1203.643	2.68	0.00161	4.264612
P0C0L4; P0	0	0.8667	662.0068	1984.006	1.22	0.00081	0

P0C0L4; P0	0	0.9714	569.99	1707.955	2.59	0.00147	14.00191
P0C0L4; P0	1	1	538.2952	1612.871	1.6	0.00086	41.40491
P0C0L4; P0	0	1	689.3657	1377.724	1.73	0.00119	2.458498
P0C0L4; P0	0	0.9375	662.006	1984.004	0.02	0.00001	83.18974
P0C0L4; P0	0	1	399.8803	1197.626	2.59	0.00104	7.288148
P0C0L4; P0	0	1	626.8574	1252.708	1.42	0.00089	4.032603
P0C0L4; P0	1	1	714.0414	2140.11	2.31	0.00165	12.5119
P0C0L4; P0	0	1	399.5277	1196.569	1.9	0.00076	51.43059
P0C0L4; P0	1	0.8485	418.0077	1669.009	2.36	0.00098	17.26229
P0C0L4; P0	0	1	409.8975	1227.678	2.59	0.00106	43.0858
P0C0L4; P0	0	0.7619	399.5596	1196.664	2.27	0.0009	39.83864
P0C0L4; P0	1	0.7556	806.9397	1612.872	2.21	0.00178	5.22815
P0C0L5	0	0.7714	531.9326	1593.783	1.35	0.00072	0
P0C0L4; P0	0	0.7895	698.9085	1396.81	1.35	0.00095	17.07978
P0C0L4; P0	0	0.871	852.9581	1704.909	2.53	0.00216	8.635193
P0C0L4; P0	0	0.4444	476.2759	1426.813	-0.25	-0.00012	11.83178
P0C0L4; P0	0	0.9512	780.9061	1560.805	1.53	0.00119	10.07023
P0C0L4; P0	1	1	459.2541	1833.994	3.3	0.00151	22.44029
P0C0L4; P0	0	1	906.833	2718.484	1.37	0.00124	2.811582
P0C0L4; P0	0	1	610.6805	1830.027	3.27	0.00199	9.225146
P0C0L4; P0	0	1	410.9166	1230.735	2.08	0.00085	37.53095
P0C0L4; P0	0	0.76	590.3304	1179.654	3.91	0.00231	0
P0C0L4; P0	0	0.7759	492.2392	983.4711	1.79	0.00088	0
P0C0L4; P0	0	0.8974	813.9114	1626.816	2.08	0.00169	11.69149
P0C0L4; P0	1	1	535.7828	2140.11	2.28	0.00122	34.20444
P0C0L4; P0	0	0.9062	542.9431	1626.815	1.47	0.0008	3.061351
P0C0L4; P0	1	1	476.6142	1427.828	2.2	0.00105	38.80282
P0C0L4; P0	0	0.4286	466.2755	1396.812	2.96	0.00138	7.190145
P0C0L4; P0	1	0.6957	592.8262	2368.283	2.89	0.00171	0
P0C0L4; P0	0	0.5312	814.434	1627.861	2.25	0.00183	10.15128
P0C0L4; P0	0	0.9583	401.8856	1203.642	2.41	0.00097	13.84584
P0C0L4; P0	0	0.7368	411.8785	1233.621	1.01	0.00042	5.811368
P0C0L4; P0	0	0.8222	511.3119	1021.617	3.26	0.00167	36.4212
P0C0L4; P0	0	1	401.8854	1203.642	1.88	0.00075	27.42783
P0C0L4; P0	0	0.8667	511.3113	1021.615	2.13	0.00109	7.841673
P0C0L4; P0	0	0.75	424.5696	1271.694	2.83	0.0012	14.03217
P0C0L4; P0	0	0.8571	409.8972	1227.677	1.77	0.00072	37.2449
P0C0L4; P0	1	1	420.9137	1260.727	0.3	0.00013	29.13715
P0C0L4; P0	0	1	899.0994	2695.284	1.75	0.00157	71.5943
P0C0L4; P0	0	0.9286	1348.146	2695.286	2.48	0.00334	0
P0C0L4; P0	0	0.5714	466.2754	1396.812	2.76	0.00129	0
P0C0L4; P0	0	0.9556	665.8625	1330.718	2.22	0.00148	3.229429
P0C0L4; P0	0	0.9259	441.9233	1323.755	3.26	0.00144	36.00274
P0C0L4; P0	0	0.7963	439.2596	877.512	1.03	0.00045	5.101516
P0C0L5	0	0.95	717.3466	1433.686	1.16	0.00083	7.366184
P0C0L4; P0	0	0.9355	575.6501	1724.936	2.8	0.00161	12.00104
P0C0L4; P0	0	1	598.7882	1196.569	2.3	0.00137	13.70312
P0C0L4; P0	1	1	501.5902	1502.756	1.24	0.00062	0
P0C0L4; P0	0	0.7805	780.9072	1560.807	2.85	0.00223	17.75122
P0C0L4; P0	1	1	592.8243	2368.276	-0.21	-0.00012	3.518496
P0C0L4; P0	1	0.8421	538.2929	1612.864	-2.83	-0.00152	86.75069
P0C0L4; P0	1	0.9474	577.6785	1731.021	1.22	0.0007	39.13348

P0C0L4; P0	0	0.7073	407.2438	1219.717	2.74	0.00111	40.93847
P0C0L4; P0	0	0.9	680.3768	2718.485	1.74	0.00118	0
P0C0L4; P0	0	0.7736	527.2651	1053.523	1.79	0.00094	31.35412
P0C0L4; P0	1	0.9412	508.9726	1524.903	0.28	0.00014	40.52543
P0C0L4; P0	1	0.9412	508.9726	1524.903	0.28	0.00014	40.52543
P0C0L5	0	0.8889	1081.218	3241.64	1.36	0.00147	1.881387
P0C0L4; P0	1	0.8056	835.0076	1669.008	1.86	0.00155	6.720405
P0C0L4; P0	0	0.5667	424.5695	1271.694	2.47	0.00105	16.01746
P0C0L4; P0	1	0.7143	518.6358	1553.893	1.9	0.00098	18.45007
P0C0L4; P0	0	0.95	548.3139	1095.621	2.33	0.00128	4.190257
P0C0L4; P0	1	1	535.7826	2140.109	1.82	0.00097	23.43074
P0C0L4; P0	0	1	526.7361	1052.465	0.53	0.00028	0
P0C0L4; P0	0	0.9091	458.2622	1830.027	3.3	0.00151	38.84898
P0C0L4; P0	0	0.2727	586.3351	1756.991	2.41	0.00141	7.217451
P0C0L4; P0	0	0.8571	511.3111	1021.615	1.65	0.00084	7.670605
P0C0L4; P0	0	0.6977	629.3596	1257.712	2.95	0.00185	5.895948
P0C0L4; P0	0	0.7551	554.8356	1108.664	3.08	0.0017	11.00247
P0C0L4; P0	0	1	617.3145	1233.622	1.64	0.00101	8.660937
P0C0L4; P0	0	0.7838	554.8355	1108.664	2.86	0.00158	35.13182
P0C0L4; P0	0	1	602.3248	1203.642	2.37	0.00143	6.424095
P0C0L4; P0	1	0.9	630.8679	1260.729	1.84	0.00116	7.584723
P0C0L4; P0	0	0.7143	722.4279	1443.848	1.27	0.00092	6.84609
P0C0L4; P0	0	1	598.7878	1196.568	1.58	0.00095	6.408106
P0C0L4; P0	0	0.9032	419.9082	1257.71	1.53	0.00064	17.20947
P0C0L4; P0	0	0.8444	630.3146	1259.622	0.64	0.0004	2.893304
P0C0L4; P0	0	0.8571	560.293	1119.579	2.17	0.00122	6.93324
P0C0L4; P0	0	0.9487	607.3416	1213.676	3.03	0.00184	21.54747
P0C0L4; P0	0	1	904.4326	2711.283	3.39	0.00306	0
P0C0L4; P0	0	1	610.6796	1830.024	1.77	0.00108	0
P0C0L4; P0	0	0.9583	590.9792	1770.923	4.93	0.00291	0
P0C0L4; P0	0	0.8723	492.2389	983.4705	1.17	0.00058	11.76828
P0C0L4; P0	0	1	852.9577	1704.908	2.03	0.00173	6.600808
P0C0L4; P0	0	0.7368	511.3101	1021.613	-0.2	-0.0001	30.00844
P0C0L4; P0	0	1	598.7905	1196.574	3.61	0.00216	0
P0C0L4; P0	0	0.9697	399.5274	1196.568	1.14	0.00045	11.45088
P0C0L4; P0	0	1	526.7377	1052.468	3.67	0.00193	0
P0C0L4; P0	1	1	493.3085	1477.911	1.12	0.00055	42.24174
P0C0L4; P0	1	0.75	592.8261	2368.282	2.68	0.00159	11.49942
P0C0L4; P0	1	1	697.4254	2090.262	1.52	0.00106	33.36861
P0C0L4; P0	0	0.8235	560.2942	1119.581	4.35	0.00244	15.10129
P0C0L4; P0	0	1	806.3716	2417.1	0.9	0.00072	0
P0C0L4; P0	0	0.9474	560.296	1119.585	7.52	0.00421	0
P0C0L4; P0	0	0.8611	774.425	1547.843	5.53	0.00428	27.70102
P0C0L4; P0	0	0.9778	488.7217	976.436	1.51	0.00074	25.58318
P0C0L4; P0	0	0.75	554.8341	1108.661	0.43	0.00024	100
P0C0L4; P0	0	0.8684	560.2941	1119.581	4.24	0.00238	2.274849
P0C0L4; P0	0	0.9348	492.2383	983.4694	0.05	0.00003	16.58234
P0C0L4; P0	0	0.7941	560.2938	1119.58	3.7	0.00207	0
P0C0L4; P0	0	1	687.9223	1374.837	1.89	0.0013	5.978024
P0C0L4; P0	1	0.9583	403.9734	1612.872	1.97	0.00079	21.62358
P0C0L4; P0	0	0.8545	554.8359	1108.664	3.63	0.00201	18.59108
P0C0L4; P0	1	0.8333	456.9215	1368.75	1.77	0.00081	16.56412

P0C0L4; P0	0	0.9091	482.7832	964.5591	0.7	0.00034	44.7948
P0C0L4; P0	0	0.8421	525.8064	1050.606	2.08	0.00109	11.52102
P0C0L4; P0	1	0.9	420.9141	1260.728	1.18	0.00049	37.17281
P0C0L4; P0	0	0.7353	458.9526	1374.843	6.07	0.00278	39.229
P0C0L4; P0	0	1	562.8051	1124.603	2.27	0.00128	11.01421
P0C0L4; P0	0	1	482.756	964.5048	1.57	0.00076	11.29565
P0C0L4; P0	0	0.9375	598.8365	1196.666	3.4	0.00203	16.29111
P0C0L4; P0	0	0.9524	742.382	1483.757	1.16	0.00086	0
P0C0L4; P0	0	0.8293	557.8166	1114.626	3.95	0.0022	13.47637
P0C0L4; P0	0		586.3353	1756.991	2.72	0.00159	0
P0C0L4; P0	0	1	399.5279	1196.569	2.21	0.00088	12.98866
P0C0L4; P0	0	0.76	544.6714	1632	1.33	0.00072	8.001515
P0C0L4; P0	0	0.9048	617.0269	1849.066	4.75	0.00293	29.99261
P0C0L4; P0	0	1	458.9506	1374.837	1.88	0.00086	44.57616
P0C0L4; P0	0	0.8684	525.806	1050.605	1.38	0.00072	21.83972
P0C0L4; P0	0	1	447.5626	1340.673	1.2	0.00053	25.67226
P0C0L4; P0	0	0	634.3692	1901.093	2.28	0.00145	16.75078
P0C0L4; P0	0	0.9677	560.2945	1119.582	4.9	0.00274	0
P0C0L4; P0	0	0.4	599.3169	1197.627	2.78	0.00167	3.641306
P0C0L4; P0	0	0.8846	542.9433	1626.815	1.92	0.00104	5.438027
P0C0L4; P0	0	0.6061	407.2438	1219.717	2.59	0.00105	13.25358
P0C0L4; P0	1	1	714.0369	2140.096	-3.93	-0.00281	34.70642
P0C0L4; P0	1	0.8125	621.8536	2484.393	2.99	0.00186	41.53924
P0C0L4; P0	1	1	420.915	1260.73	3.21	0.00135	58.33232
P0C0L4; P0	0	1	458.9503	1374.836	1.08	0.00049	76.27785
P0C0L4; P0	0	1	548.3137	1095.62	2	0.0011	0
P0C0L4; P0	0	0.75	419.9087	1257.711	2.62	0.0011	4.821672
P0C0L4; P0	0	0.76	862.9706	1724.934	1.82	0.00157	43.96324
P0C0L4; P0	0	1	674.577	2695.286	2.61	0.00176	0
P0C0L4; P0	1	0.8	420.9142	1260.728	1.47	0.00062	30.87623
P0C0L4; P0	0	1	730.4273	1459.847	3.99	0.00291	8.528147
P0C0L4; P0	0	0.25	576.7958	2304.161	2.44	0.0014	29.14127
P0C0L4; P0	0	0.9118	525.8063	1050.605	1.96	0.00103	3.130056
P0C0L4; P0	0	1	780.9059	1560.805	1.29	0.00101	13.83405
P0C0L4; P0	0	0.92	568.9929	1704.964	5.19	0.00295	8.115194
P0C0L4; P0	0	0.7586	419.9086	1257.711	2.55	0.00107	46.55788
P0C0L4; P0	0	1	542.291	1083.575	1.72	0.00093	6.112218
P0C0L4; P0	0	0.9375	1064.557	2128.107	0.65	0.00069	0
P0C0L4; P0	0	1	629.9681	1887.89	2.5	0.00157	78.46582
P0C0L4; P0	0	1	562.8051	1124.603	2.38	0.00134	0
P0C0L4; P0	0	1	548.3141	1095.621	2.67	0.00146	0
P0C0L4; P0	0	1	568.9743	1704.908	2.17	0.00123	0
P0C0L4; P0	1	1	421.0075	1681.008	4.86	0.00204	58.07848
P0C0L4; P0	0	1	590.9782	1770.92	3.17	0.00187	0
P0C0L4; P0	0	0.6207	630.3162	1259.625	3.25	0.00205	9.959315
P0C0L4; P0	0	1	665.8615	1330.716	0.75	0.0005	0
P0C0L4; P0	0	1	639.3193	1277.631	2.87	0.00183	14.56757
P00739	0	1	505.2966	1513.875	1.48	0.00075	22.24131
P00739; P0	0	1	545.2894	1633.854	2.41	0.00131	8.85826
P00739; P0	0	0.6667	404.5835	1211.736	0.76	0.00031	23.85787
P00739; P0	0	0.8594	606.3724	1211.737	2.03	0.00123	6.361173
P00739; P0	0	0.575	404.583	1211.734	-0.6	-0.00024	14.35788

P00739	0	0.9211	534.0665	2133.244	0.95	0.00051	8.713562
P00739; P0	0	0.6452	404.5835	1211.736	0.68	0.00028	27.89671
P00739	0	1	505.2962	1513.874	0.7	0.00035	28.60976
P00739; P0	0	1	789.972	1578.937	1.39	0.00109	5.505499
P00739; P0	0	1	526.9839	1578.937	1.65	0.00087	9.713168
P00739; P0	0	0.9362	717.9207	1434.834	1.11	0.00079	9.209297
P00739; P0	0	1	789.9721	1578.937	1.54	0.00122	7.52731
P00739; P0	0	0.9608	526.9834	1578.936	0.72	0.00038	6.662654
P00739; P0	0	1	533.9432	1599.815	2.84	0.00152	13.07789
P00739; P0	0	0.9863	800.4113	1599.815	3.08	0.00246	8.227809
P00739; P0	0	0.8714	800.411	1599.815	2.62	0.0021	0
P00739; P0	0	0.9821	545.2885	1633.851	0.73	0.0004	9.662613
P00739; P0	0	0.9565	545.2874	1633.848	-1.4	-0.00076	17.14644
P00739; P0	0	1	817.4298	1633.852	1.49	0.00122	5.912034
P00739; P0	0	1	545.2888	1633.852	1.18	0.00064	10.99026
P00739; P0	0	1	817.4294	1633.851	1.04	0.00085	9.215112
P00739; P0	0	0.9792	545.2889	1633.852	1.4	0.00077	40.41518
P00739; P0	0	1	545.2897	1633.855	2.97	0.00162	61.47524
P00739; P0	0	0.9211	545.2891	1633.853	1.74	0.00095	9.576406
P00739; P0	0	0.9615	545.2885	1633.851	0.73	0.0004	10.92377
P00739; P0	0	1	545.2891	1633.853	1.85	0.00101	7.647542
P00739; P0	0	0.9643	545.2882	1633.85	0.17	0.00009	7.670223
P00739; P0	0	0.9833	666.8856	1332.764	1.26	0.00084	6.644083
P00739; P0	0	1	607.3321	1819.982	2.58	0.00157	12.64681
P00739; P0	0	1	455.7507	1819.981	2.24	0.00102	11.57495
P00739; P0	1	0.4146	473.6367	1418.895	0.82	0.00039	28.42399
P00739; P0	1	0.9302	617.7175	1851.138	3.51	0.00217	19.44054
P00739; P0	0	0.8254	594.8354	1188.664	2.86	0.0017	10.16927
P00739	0	0.8684	725.0243	2173.058	2.47	0.00179	0
P00739; P0	0	0.9744	817.4287	1633.85	0.14	0.00012	0
P00739; P0	0	0.9189	526.9843	1578.938	2.46	0.00129	10.61268
P00739; P0	0	1	604.8381	1208.669	1.98	0.00119	0
P00739; P0	0	1	604.837	1208.667	0.06	0.00003	5.94458
P00739; P0	0	1	604.8383	1208.669	2.18	0.00132	6.355654
P00739; P0	0	0.8333	545.2889	1633.852	1.52	0.00083	7.819473
P00739; P0	0	0.5556	444.9262	1332.764	1.3	0.00058	16.70804
P00739; P0	0	0.8387	594.8349	1188.663	2.04	0.00121	15.26654
P00739; P0	0	0.3125	404.5837	1211.736	1.13	0.00046	38.3526
P00739; P0	0	0.8621	526.9837	1578.937	1.3	0.00068	30.90254
P00739; P0	0	0.7692	444.9258	1332.763	0.61	0.00027	20.90685
P00739; P0	0	0.8889	450.258	1348.759	1.68	0.00076	28.44966
P00739; P0	0	0.7059	404.5837	1211.737	1.29	0.00052	32.86251
P00739; P0	0	0.5957	501.8018	1002.596	1.12	0.00056	26.13306
P00739; P0	0	0.381	404.5838	1211.737	1.44	0.00058	34.80645
P00739; P0	0	0.7037	404.5843	1211.738	2.8	0.00113	25.39663
P00739; P0	0	1	728.3563	1455.705	-2.06	-0.0015	0
P00739; P0	0	0.3784	573.8538	1146.7	2.73	0.00156	48.05908
P00739; P0	0	0.7347	534.3206	1067.634	0.94	0.0005	0
P00739; P0	0	0.9231	403.5613	1208.669	2.14	0.00086	9.922118
P00739; P0	0	0.5833	396.8925	1188.663	2.36	0.00094	0
P00739; P0	0	0.7333	604.8378	1208.668	1.47	0.00089	16.93134
P00739; P0	0	0.7812	604.8389	1208.67	3.19	0.00193	6.641055

P00739	0	0.8	757.4417	1513.876	2.06	0.00156	0
P00739; P0	0	0.8	604.8384	1208.67	2.48	0.0015	9.656388
P00739; P0	0	0.55	533.9431	1599.815	2.61	0.00139	29.20991
P00739; P0	0	0.5333	450.2581	1348.76	1.95	0.00088	42.17896
P00739; P0	0	0.625	573.852	1146.697	-0.46	-0.00027	22.40576
P01861; P0	0	0.9744	477.9235	1431.756	1.71	0.00081	13.87715
P01861; P0	0	1	716.3814	1431.756	1.44	0.00103	9.843532
P01860	0	1	568.9728	1704.904	5.29	0.00301	12.98781
P01857; P0	0	0.8902	537.3005	1609.887	3.05	0.00164	13.45807
P01860	0	1	568.9716	1704.9	3.25	0.00185	10.98584
P01857; P0	0	0.9608	805.447	1609.887	2.87	0.00231	0
P01861; P0	0	0.9429	477.9231	1431.755	0.81	0.00039	14.94996
P01857; P0	0	0.6667	483.9505	1449.837	2.21	0.00107	9.518343
P01857; P0	0	1	483.9502	1449.836	1.51	0.00073	24.07588
P01860	0	1	500.585	1499.74	0.76	0.00038	0
P01857; P0	0	0.9643	563.8591	1126.711	3.07	0.00173	8.029201
P01860	0	0.6667	665.8052	1330.603	2.39	0.00159	3.261047
P01857; P0	0	1	715.8931	1430.779	2.09	0.00149	3.09548
P01857; P0	0	1	477.5974	1430.778	1.06	0.0005	11.75311
P01857; P0	0	0.9697	715.8933	1430.779	2.34	0.00168	2.854816
P01857; P0	0	0.9672	715.8933	1430.779	2.34	0.00168	5.368532
P01857; P0	0	1	715.8925	1430.778	1.15	0.00082	0
P01857; P0	0	1	477.598	1430.779	2.33	0.00111	7.543014
P01857; P0	0	0.8621	477.5976	1430.778	1.5	0.00072	19.59222
P01857; P0	0	0.5357	537.3008	1609.888	3.5	0.00188	75.55756
P01857; P0	0	0.8889	563.8597	1126.712	4.26	0.0024	9.855687
P01857; P0	0	0.6981	426.218	851.4287	-0.51	-0.00022	5.356847
P01857; P0	0	0.7619	477.5977	1430.779	1.76	0.00084	49.38679
P01861; P0	0	0.8525	571.3118	1711.921	4.14	0.00236	10.60563
P01861; P0	0	0.9231	571.3099	1711.915	0.72	0.00041	8.676208
P01861; P0	0	0.9744	477.9235	1431.756	1.71	0.00081	13.87715
P01861; P0	0	1	716.3814	1431.756	1.44	0.00103	9.843532
P01859	0	0.9138	732.0396	2194.104	2.77	0.00203	5.690258
P01861; P0	0	0.9429	477.9231	1431.755	0.81	0.00039	14.94996
P01857; P0	0	0.6667	483.9505	1449.837	2.21	0.00107	9.518343
P01857; P0	0	1	483.9502	1449.836	1.51	0.00073	24.07588
P01859	0	1	668.9476	3340.709	0.26	0.00018	1.571693
P01857; P0	0	1	715.8931	1430.779	2.09	0.00149	3.09548
P01857; P0	0	1	477.5974	1430.778	1.06	0.0005	11.75311
P01857; P0	0	0.9697	715.8933	1430.779	2.34	0.00168	2.854816
P01857; P0	0	0.9672	715.8933	1430.779	2.34	0.00168	5.368532
P01857; P0	0	1	715.8925	1430.778	1.15	0.00082	0
P01857; P0	0	1	477.598	1430.779	2.33	0.00111	7.543014
P01861; P0	0	0.8571	856.4638	1711.92	3.79	0.00324	6.989905
P01857; P0	0	0.8621	477.5976	1430.778	1.5	0.00072	19.59222
P01859	0	0.6735	556.8509	1112.695	2.47	0.00137	100
P01859	0	0.7292	556.8519	1112.696	4.23	0.00235	0
P01859	0	1	835.9345	3340.716	2.42	0.00202	4.225374
P01857; P0	0	0.6981	426.218	851.4287	-0.51	-0.00022	5.356847
P01859	0	0.4	732.0386	2194.101	1.44	0.00105	12.75352
P01857; P0	0	0.7619	477.5977	1430.779	1.76	0.00084	49.38679
Q13790	0	0.875	662.6901	1986.056	1.19	0.00079	7.805773

Q13790	0	0.7632	662.6874	1986.048	-2.96	-0.00196	21.35427
Q13790	0	0.9038	662.6915	1986.06	3.22	0.00213	34.26013
Q13790	0	1	740.8695	1480.732	1.92	0.00142	3.50881
Q13790	0	0.9342	805.3857	1609.764	1.52	0.00122	7.707602
Q13790	0	1	537.2594	1609.764	1.17	0.00063	11.6004
Q13790	0	0.8182	537.259	1609.762	0.37	0.0002	21.61834
P01034	0	0.9595	596.9708	1788.898	3.31	0.00197	2.723889
P01034	0	1	894.9514	1788.896	2.09	0.00187	0
P01034	0	1	602.3017	1804.891	2.1	0.00127	35.44664
P01034	0	0.7568	505.6083	1514.81	0.7	0.00035	31.58549
P01034	0	0.5938	408.8572	1224.557	1.87	0.00076	0
P02775	0	0.75	463.6178	1388.839	2.67	0.00124	19.3585
P02775	0	0.7969	694.9225	1388.838	1.85	0.00128	14.60299
P02775	0	0.8889	620.0085	1858.011	0.76	0.00047	14.23052
P02775	0	0.8846	376.8936	1128.666	4.15	0.00156	16.19851
P02775	0	0.9091	465.2593	1858.015	3.12	0.00145	45.91182
P14151	0	0.9189	445.2123	1333.622	2.05	0.00091	44.53681
P14151	0	0.9455	726.6799	2178.025	2.9	0.00211	28.84433
P14151	0	0.975	726.6803	2178.026	3.41	0.00247	4.053051
P14151	0	0.7857	601.3298	1201.652	3.15	0.00189	4.11567
Q15113	0	1	517.9089	1551.712	1.95	0.00101	28.86117
Q15113	0	1	625.642	1874.912	3.8	0.00238	37.26224
Q15113	0	0.8125	600.3443	1799.018	1.71	0.00102	44.85451
P49908	0	0.84	668.871	1336.735	2.14	0.00143	10.30466
P49908	0	0.8868	600.806	1200.605	2.46	0.00147	14.37117
P49908	0	1	446.249	1336.733	0.44	0.0002	23.66555
P49908	1	0.7857	426.2675	1276.788	4.15	0.00177	34.49136
P49908	0	0.9032	600.8051	1200.603	0.93	0.00056	10.72482
P16070	0	1	510.9573	1530.857	1.66	0.00085	6.603345
P16070	0	1	510.9571	1530.857	1.19	0.0006	42.37265
P16070	0	0.5263	441.2562	1321.754	2.55	0.00112	27.20519
P16070	0	0.4286	441.2565	1321.755	3.24	0.00143	6.555589
P16070	0	0.6207	399.2137	1195.627	2.86	0.00114	8.330242
Q02325; Q1	0	0.8	584.823	1168.639	2.92	0.00171	12.57676
Q15195; P0	1	0.9167	475.6111	1424.819	0.93	0.00044	26.07081
Q02325; Q1	0	0.5833	390.2174	1168.638	2.1	0.00082	27.11907
Q15195; P0	0	0.6585	569.3429	1137.679	2.41	0.00137	4.224358
Q15195; P0	0	0.6364	569.3433	1137.679	3.16	0.0018	14.98462
Q02325; Q1	0	0.8182	584.8233	1168.639	3.44	0.00201	7.360862
Q02325; Q1	0	0.5833	390.2172	1168.637	1.55	0.00061	10.91142
Q02325; Q1	0	0.44	390.2168	1168.636	0.38	0.00015	14.30095
Q15485	0	0.8889	664.34	1991.005	2.67	0.00178	0
Q15485	0	0.9245	664.3398	1991.005	2.49	0.00165	0
Q15485	0	1	601.3024	1201.597	0.41	0.00025	5.559122
Q15485; O0	0	0.4	525.2898	1049.572	0.58	0.00031	0
P63267; P6	0	0.7826	483.9488	1449.832	6.36	0.00307	34.84767
P63267; P6	0	1	487.2637	1459.777	0.94	0.00046	46.31744
A5A3E0; P6	0	0.8636	645.6713	1934.999	2.72	0.00175	55.86254
P63267; P6	0	0.7826	483.9488	1449.832	6.36	0.00307	34.84767
P63267; P6	0	1	487.2637	1459.777	0.94	0.00046	46.31744
A5A3E0; P6	0	0.8636	645.6713	1934.999	2.72	0.00175	55.86254
Q9UNW1	0	0.9333	619.2996	1855.884	3.59	0.00222	0

Q9UNW1	0	0.8621	393.9082	1179.71	1.87	0.00073	37.77554
Q9UNW1	0	1	694.9902	2082.956	0.44	0.00031	7.746798
P13645	0	1	503.9196	1509.744	1.8	0.00091	39.63369
P13645	0	0.5714	557.2896	1669.854	1.19	0.00066	0
Q14532; O7	0	0.875	404.2042	807.4011	1.86	0.00075	3.074318
O14786	0	0.6829	522.975	1566.911	0.77	0.0004	40.14544
O14786	0	1	593.3361	1777.994	3.67	0.00217	30.00705
O14786	0	0.6774	537.3516	1073.696	3.5	0.00188	23.04314
Q9Y5Y7	0	0.5897	397.8992	1191.683	0.54	0.00021	5.773323
Q9Y5Y7	0	0.5882	580.3881	1159.769	3.1	0.0018	43.75885
Q9Y5Y7	0	0.6667	625.3942	1249.781	1.49	0.00093	50.40883
Q9NPH3	0	0.5682	512.6077	1535.809	-0.66	-0.00034	22.06363
Q9NPH3	0	0.814	545.8578	1090.708	0.82	0.00045	10.02885
Q9NPH3	0	0.9	473.294	1417.867	1.18	0.00056	6.004278
Q9HDC9	0	1	592.8535	1184.7	3.91	0.00231	4.244822
Q9HDC9	0	0.4828	459.9312	1377.779	1.47	0.00067	22.26415
Q9HDC9	0	0.3333	525.6472	1574.927	1.75	0.00092	37.24336
P22105; Q1	0	1	584.3294	1750.974	3.98	0.00232	6.555482
P22105	0	0.6	596.6733	1788.005	3.35	0.002	23.84114
P22105; Q1	0	0.6667	621.864	1242.721	2.59	0.00161	50.187
A5A3E0; Q9	0	1	554.2729	1660.804	-0.37	-0.0002	38.75434
A5A3E0; P6	0	0.8636	645.6713	1934.999	2.72	0.00175	55.86254
A5A3E0; Q9	0	1	505.9223	1515.752	2.11	0.00107	29.73994
A5A3E0; Q9	0	1	554.2729	1660.804	-0.37	-0.0002	38.75434
A5A3E0; P6	0	0.8636	645.6713	1934.999	2.72	0.00175	55.86254
A5A3E0; Q9	0	1	505.9223	1515.752	2.11	0.00107	29.73994
A5A3E0; Q9	0	1	554.2729	1660.804	-0.37	-0.0002	38.75434
A5A3E0; P6	0	0.8636	645.6713	1934.999	2.72	0.00175	55.86254
A5A3E0; Q9	0	1	505.9223	1515.752	2.11	0.00107	29.73994
Q15063	0	1	453.9575	1359.858	0.56	0.00026	66.04424
Q15063	0	0.7955	579.8727	1158.738	3.85	0.00223	7.175214
Q15063	0	0.5333	573.3596	1145.712	-1.1	-0.00063	37.00546
P04040	0	1	421.8919	1263.661	0.74	0.00031	26.83989
P04040	0	0.9231	606.3483	1817.03	5.76	0.00349	31.67634
P04040	0	0.95	653.0134	1957.026	1.17	0.00076	0
P98160	0	0.8621	490.744	980.4806	1.07	0.00053	16.7303
P98160	0	0.9444	635.663	1904.974	4.56	0.00289	42.13707
P98160	0	0.973	575.2916	1149.576	5.93	0.00341	0
P0C0L4; P0	0	0.88	814.4339	1627.861	2.17	0.00177	11.91346
P0C0L4; P0	0	0.72	543.2917	1627.861	2.17	0.00117	2.514847
P0C0L4; P0	0	0.9589	814.4331	1627.859	1.2	0.00098	6.002271
P0C0L4; P0	0	0.7451	543.2906	1627.857	0.14	0.00008	11.02971
P0C0L4; P0	0	0.9467	543.2921	1627.862	2.84	0.00154	8.938625
P0C0L4; P0	0	0.1429	440.0023	1756.987	0.38	0.00017	13.3462
P0C0L4; P0	1	0.8929	790.0978	2368.279	1.16	0.00092	9.614325
P0C0L4; P0	0	0.8214	543.2907	1627.857	0.25	0.00014	0
P0C0L4; P0	0	0.9559	610.679	1830.022	0.67	0.00041	8.518244
P0C0L4; P0	0	0.8901	915.5165	1830.026	2.58	0.00236	6.086547
P0C0L4; P0	0	0.8864	458.2622	1830.027	3.16	0.00145	2.335641
P0C0L4; P0	0	1	610.6807	1830.027	3.47	0.00212	17.67647
P0C0L4; P0	0	1	610.6804	1830.027	3.07	0.00187	9.608068
P0C0L4; P0	0	1	636.3515	1271.696	3.9	0.00248	14.22901

P0C0L4; P0	0	0.8824	459.9129	1377.724	1.79	0.00082	0
P0C0L4; P0	0	0.7368	557.8151	1114.623	1.21	0.00068	0
P0C0L4; P0	0	0.9608	636.3503	1271.693	1.98	0.00126	7.495775
P0C0L4; P0	1	0.7333	592.8256	2368.281	1.96	0.00116	2.025505
P0C0L4; P0	1	0.88	790.0981	2368.28	1.55	0.00122	4.007072
P0C0L4; P0	1	0.7407	592.8265	2368.284	3.5	0.00207	65.63628
P0C0L4; P0	1	0.963	790.0977	2368.279	1.09	0.00086	5.731484
P0C0L4; P0	1	0.9706	790.0981	2368.28	1.63	0.00129	6.935635
P0C0L4; P0	1	1	571.8116	2284.225	7.79	0.00445	20.25506
P0C0L4; P0	1	0.9118	762.0757	2284.213	2.61	0.00199	0
P0C0L4; P0	0	1	710.0423	2128.112	3.17	0.00225	84.79305
P0C0L4; P0	0	0.8197	615.872	1230.737	3.21	0.00198	13.4027
P0C0L4; P0	0	1	542.2908	1083.574	1.49	0.00081	1.305774
P0C0L4; P0	1	0.8049	470.6024	1409.793	3.44	0.00162	17.80892
P0C0L4; P0	0	0.7	481.956	1443.854	4.79	0.00231	13.23638
P0C0L4; P0	0	0.7015	662.3807	1323.754	2.28	0.00151	3.729706
P0C0L4; P0	0	0.6579	524.311	1570.918	1.83	0.00096	6.544328
P0C0L4; P0	1	0.9074	490.2613	1468.769	1.87	0.00091	25.50551
P0C0L4; P0	1	0.9074	490.2613	1468.769	1.87	0.00091	25.50551
P0C0L4; P0	1	1	490.2612	1468.769	1.8	0.00088	2.070895
P0C0L4; P0	1	1	490.2612	1468.769	1.8	0.00088	2.070895
P0C0L4; P0	0	1	904.4314	2711.28	2.11	0.00191	1.925776
P0C0L4; P0	0	0.8333	399.5598	1196.665	2.57	0.00103	10.67162
P0C0L4; P0	0	0.697	481.9547	1443.85	2.13	0.00102	11.48204
P0C0L4; P0	0	1	899.0993	2695.283	1.62	0.00145	1.309225
P0C0L4; P0	0	1	899.0989	2695.282	1.21	0.00108	2.199146
P0C0L4; P0	0	1	851.0645	2551.179	0.73	0.00062	0
P0C0L4; P0	0	0.9524	862.9702	1724.933	1.32	0.00114	7.43602
P0C0L4; P0	0	0.9444	575.6502	1724.936	2.9	0.00167	28.3975
P0C0L4; P0	0	0.9298	575.65	1724.936	2.69	0.00155	8.106833
P0C0L4; P0	0	0.962	862.971	1724.935	2.24	0.00193	1.706079
P0C0L4; P0	0	0.9268	575.6498	1724.935	2.37	0.00136	11.78024
P0C0L4; P0	0	0.8571	862.9707	1724.934	1.89	0.00163	10.14675
P0C0L4; P0	0	0.9815	575.6504	1724.937	3.33	0.00191	8.231376
P0C0L4; P0	0	0.8571	590.9779	1770.919	2.76	0.00163	31.60502
P0C0L4; P0	0	0.9104	590.9768	1770.916	0.8	0.00047	3.83896
P0C0L4; P0	0	0.8462	885.9625	1770.918	1.91	0.00169	3.769225
P0C0L4; P0	0	0.92	590.9765	1770.915	0.38	0.00023	4.631679
P0C0L4; P0	0	0.5714	468.5796	1403.724	0.71	0.00033	27.22315
P0C0L4; P0	0	0.987	702.3669	1403.726	2.39	0.00168	6.447455
P0C0L4; P0	0	0.9189	459.9125	1377.723	0.72	0.00033	9.700747
P0C0L4; P0	0	0.7647	599.3157	1197.624	0.75	0.00045	4.685958
P0C0L4; P0	0	0.9167	459.9125	1377.723	0.72	0.00033	3.849536
P0C0L4; P0	0	0.8649	459.9129	1377.724	1.79	0.00082	3.418623
P0C0L4; P0	1	1	490.2617	1468.771	2.74	0.00134	68.65987
P0C0L4; P0	0	0.9831	458.9499	1374.835	0.21	0.0001	8.918069
P0C0L4; P0	0	0.7882	687.9224	1374.838	2.07	0.00142	6.157514
P0C0L4; P0	1	1	490.2617	1468.771	2.74	0.00134	68.65987
P0C0L4; P0	0	1	458.9507	1374.838	2.14	0.00098	8.437518
P0C0L4; P0	0	0.9808	662.0079	1984.009	2.79	0.00184	19.66835
P0C0L4; P0	0	1	572.7972	2288.167	2.69	0.00154	16.94717
P0C0L4; P0	0	0.8723	722.4288	1443.85	2.62	0.00189	8.394596

P0C0L4; P0	0	1	481.955	1443.85	2.64	0.00127	18.88451
P0C0L4; P0	0	0.8706	790.413	2369.224	1.66	0.00131	7.247535
P0C0L4; P0	1	1	538.2988	1612.882	8.3	0.00446	28.51182
P0C0L4; P0	0	0.6596	576.7955	2304.16	2.01	0.00116	5.662117
P0C0L4; P0	1	1	571.8084	2284.212	2.23	0.00128	2.234328
P0C0L4; P0	1	1	762.0748	2284.21	1.33	0.00101	2.679379
P0C0L4; P0	1	1	571.808	2284.21	1.59	0.00091	2.213109
P0C0L4; P0	1	1	762.075	2284.21	1.65	0.00126	2.43145
P0C0L4; P0	0	1	634.368	1901.09	0.45	0.00029	7.470258
P0C0L4; P0	0	1	476.0281	1901.091	0.99	0.00047	7.908507
P0C0L4; P0	1	0.9792	762.0773	2284.217	4.7	0.00358	0
P0C0L4; P0	1	1	571.8112	2284.223	7.04	0.00402	14.00352
P0C0L4; P0	0	0.8525	409.8971	1227.677	1.47	0.0006	10.92853
P0C0L4; P0	0	0.8636	614.3425	1227.678	2.4	0.00147	3.654341
P0C0L4; P0	0	0.8621	409.8967	1227.676	0.65	0.00027	5.003325
P0C0L4; P0	0	1	1126.59	2252.174	2.53	0.00285	1.71125
P0C0L4; P0	0	1	751.3956	2252.172	1.88	0.00141	2.23579
P0C0L4; P0	0	1	1215.597	3644.776	1.73	0.0021	0
P0C0L4; P0	0	1	911.949	3644.774	1.14	0.00104	0.705887
P0C0L4; P0	0	0.7143	399.5596	1196.664	2.19	0.00087	8.869975
P0C0L4; P0	0	0.5517	524.3106	1570.917	1.13	0.00059	53.46223
P0C0L4; P0	0	0.9231	698.9091	1396.811	2.23	0.00156	6.511384
P0C0L4; P0	0	0.9855	617.0257	1849.063	2.77	0.00171	25.65826
P0C0L4; P0	0	1	617.0252	1849.061	1.88	0.00116	47.37478
P0C0L4; P0	0	0.94	733.3654	2198.082	-1.93	-0.00141	65.47301
P0C0L4; P0	0	1	695.02	2083.046	1.08	0.00075	0
P0C0L4; P0	0	1	617.025	1849.06	1.58	0.00097	19.06164
P0C0L4; P0	0	1	617.0253	1849.061	2.08	0.00128	6.541273
P0C0L4; P0	0	1	706.8581	2824.411	1.52	0.00107	3.352633
P0C0L4; P0	0	1	763.3923	2288.162	0.74	0.00057	4.858709
P0C0L4; P0	0	1	572.7972	2288.167	2.69	0.00154	3.311056
P0C0L4; P0	0	1	763.3929	2288.164	1.46	0.00112	3.275803
P0C0L4; P0	0	1	572.7966	2288.165	1.73	0.00099	1.890797
P0C0L4; P0	0	1	942.143	2824.414	2.91	0.00274	1.905259
P0C0L4; P0	0	1	942.1425	2824.413	2.32	0.00219	2.277306
P0C0L4; P0	0	1	572.7976	2288.168	3.33	0.0019	3.885201
P0C0L4; P0	0	1	706.8582	2824.411	1.6	0.00113	2.626854
P0C0L4; P0	1	0.9091	418.0074	1669.008	1.63	0.00068	17.1066
P0C0L4; P0	0	1	572.7955	2288.16	-0.3	-0.00017	0
P0C0L4; P0	1	1	557.0079	1669.009	2.51	0.0014	10.70534
P0C0L4; P0	0	1	710.0408	2128.108	1.02	0.00072	1.937721
P0C0L4; P0	1	0.8864	418.006	1669.002	-1.66	-0.00069	8.256634
P0C0L4; P0	1	0.9737	557.0076	1669.008	2.07	0.00115	13.86559
P0C0L4; P0	0	1	532.7825	2128.108	1.15	0.00061	25.97721
P0C0L4; P0	0	0.9375	710.0416	2128.11	2.22	0.00158	12.2241
P0C0L4; P0	0	0.8421	679.3927	1357.778	2.88	0.00195	12.50909
P0C0L4; P0	0	0.5957	453.2638	1357.777	1.99	0.0009	14.11089
P0C0L4; P0	0	0.86	629.8673	1258.727	2.91	0.00183	22.45838
P0C0L4; P0	0	0.8197	453.2637	1357.777	1.72	0.00078	6.476308
P0C0L4; P0	0	0.9286	679.392	1357.777	1.89	0.00128	4.755651
P0C0L4; P0	1	0.9787	557.0079	1669.009	2.51	0.0014	11.11647
P0C0L4; P0	0	1	710.0415	2128.11	2.05	0.00145	0

P0C0L4; P0	0	1	706.8581	2824.411	1.52	0.00107	1.25589
P0C0L4; P0	0	0.9821	463.0205	1849.06	1.44	0.00067	8.049833
P0C0L4; P0	0	0.8714	925.0349	1849.062	2.71	0.00251	5.728807
P0C0L4; P0	0	0.9275	617.0245	1849.059	0.89	0.00055	9.125571
P0C0L4; P0	0	1	463.0206	1849.061	1.77	0.00082	10.18817
P0C0L4; P0	0	1	925.0341	1849.061	1.85	0.00171	0
P0C0L4; P0	0	1	617.025	1849.06	1.58	0.00097	7.924266
P0C0L4; P0	0	0.902	617.0258	1849.063	2.97	0.00183	39.13517
P0C0L4; P0	0	0.8367	568.9914	1704.96	2.51	0.00143	6.909508
P0C0L4; P0	0	0.3689	1063.531	3188.578	1.4	0.00148	3.788276
P0C0L4; P0	0	0.3882	797.8998	3188.577	1.03	0.00082	3.799753
P0C0L4; P0	0	1	710.0415	2128.11	2.05	0.00145	1.810176
P0C0L4; P0	0	0.7188	399.5593	1196.663	1.5	0.0006	23.17154
P0C0L4; P0	0	1	670.8411	1340.675	2.57	0.00172	4.709168
P0C0L4; P0	0	0.8298	629.8673	1258.727	3	0.00189	8.2719
P0C0L4; P0	0	0.8958	629.8668	1258.726	2.23	0.0014	32.32337
P0C0L4; P0	0	1	911.9504	3644.78	2.74	0.0025	5.921183
P0C0L4; P0	0	1	1215.599	3644.782	3.43	0.00417	0
P0C0L4	0	0.7759	531.9322	1593.782	0.54	0.00029	9.806005
P0C0L4; P0	0	0.9912	876.4736	2627.406	2.03	0.00178	2.412186
P0C0L4; P0	0	1	974.8275	2922.468	2.69	0.00262	0
P0C0L4; P0	0	1	926.7924	2778.363	1.6	0.00148	26.17738
P0C0L4; P0	0	1	1068.863	3204.576	2.1	0.00224	6.34409
P0C0L4; P0	0	1	733.3691	2198.093	3.24	0.00237	42.34372
P0C0L4; P0	0	1	1242.156	2483.305	2.4	0.00298	0
P0C0L4; P0	0	1	1314.207	2627.406	1.99	0.00261	0
P0C0L4; P0	0	0.9184	685.3339	2053.987	1.72	0.00117	2.506288
P0C0L4; P0	1	1	538.2955	1612.872	2.05	0.0011	19.50909
P0C0L4; P0	1	1	538.2947	1612.87	0.69	0.00037	38.42788
P0C0L4; P0	0	0.8095	409.8974	1227.678	2.29	0.00094	12.2222
P0C0L4; P0	0	0.7937	554.836	1108.665	3.85	0.00213	9.636339
P0C0L4; P0	1	0.9773	538.295	1612.871	1.26	0.00068	15.72817
P0C0L4; P0	1	0.9898	538.2946	1612.869	0.46	0.00025	8.549582
P0C0L4; P0	1	1	538.2954	1612.872	1.94	0.00104	9.702331
P0C0L4; P0	0	0.8	560.2935	1119.58	3.15	0.00177	11.31302
P0C0L4; P0	0	0.9333	610.6798	1830.025	1.97	0.0012	44.52637
P0C0L4; P0	1	0.9677	563.6411	1688.909	1.81	0.00102	4.968037
P0C0L4; P0	0	1	1209.052	2417.098	-0.21	-0.00025	0
P0C0L4; P0	0	1	806.3714	2417.1	0.59	0.00048	3.273516
P0C0L4	0	0.9762	819.8998	3276.577	1.87	0.00153	2.581899
P0C0L4	0	1	1092.862	3276.572	0.36	0.0004	1.932639
P0C0L4; P0	0	1	733.3657	2198.083	-1.43	-0.00105	36.81449
P0C0L4; P0	0	1	733.3678	2198.089	1.4	0.00103	14.15553
P0C0L4; P0	0	0.9783	733.3682	2198.09	1.9	0.00139	11.64252
P0C0L4; P0	0	1	733.3679	2198.089	1.49	0.00109	8.001216
P0C0L4; P0	0	1	629.9674	1887.888	1.43	0.0009	4.39021
P0C0L4; P0	0	0.9048	944.447	1887.887	0.89	0.00084	3.799334
P0C0L4; P0	0	0.9672	629.9689	1887.892	3.86	0.00243	21.66053
P0C0L4; P0	0	1	723.0315	2167.08	1.69	0.00122	1.818813
P0C0L4; P0	0	0.7586	669.8612	1338.715	0.5	0.00034	6.654665
P0C0L4; P0	0	0.9747	854.4799	1707.952	0.85	0.00072	3.551121
P0C0L4; P0	0	1	569.9898	1707.955	2.16	0.00123	5.385142

P0C0L4; P0	0	0.9722	568.9744	1704.909	2.28	0.00129	5.693548
P0C0L4; P0	0	1	568.9751	1704.911	3.56	0.00203	9.272164
P0C0L4; P0	0	0.9672	852.9576	1704.908	1.96	0.00167	6.551302
P0C0L4; P0	0	1	568.9751	1704.911	3.56	0.00203	13.15989
P0C0L4; P0	0	1	782.4294	1563.851	1.63	0.00128	0
P0C0L4; P0	0	0.9474	568.9741	1704.908	1.85	0.00105	3.56933
P0C0L4; P0	0	0.9184	852.9573	1704.907	1.53	0.00131	6.473444
P0C0L4; P0	0	0.9362	780.9066	1560.806	2.07	0.00162	7.603855
P0C0L4; P0	0	1	568.9749	1704.91	3.14	0.00178	9.686429
P0C0L4; P0	0	0.9762	568.975	1704.911	3.46	0.00196	10.09111
P0C0L4; P0	0	1	816.5032	1631.999	1	0.00082	10.48337
P0C0L4; P0	0	0.9677	790.4136	2369.226	2.43	0.00192	6.811904
P0C0L4; P0	0	1	481.9554	1443.852	3.52	0.0017	0
P0C0L4; P0	0	0.7714	737.9129	1474.819	1.17	0.00086	4.098854
P0C0L4; P0	0	0.9333	492.2779	1474.819	1.57	0.00077	5.518109
P0C0L4	0	0.9878	828.4674	1655.927	1.66	0.00137	22.2522
P0C0L4; P0	0	0.9157	542.9428	1626.814	1.02	0.00056	0.859662
P0C0L4; P0	0	1	763.4246	2288.259	1.75	0.00134	30.67124
P0C0L4; P0	1	0.9677	621.8533	2484.391	2.4	0.00149	4.806307
P0C0L4; P0	0	1	592.3666	1775.085	2.33	0.00138	3.065492
P0C0L4; P0	0	1	791.7968	2373.376	0.63	0.0005	7.553562
P0C0L4; P0	0	0.875	594.0998	2373.377	1.24	0.00074	27.68506
P0C0L4; P0	0	0.7143	474.583	1421.734	3.31	0.00157	25.12729
P0C0L4; P0	0	0.988	711.371	1421.735	3.6	0.00256	7.464592
P0C0L4; P0	0	0.9434	474.5831	1421.735	3.5	0.00166	9.031528
P0C0L4; P0	0	1	619.5472	3093.707	1.91	0.00118	20.76154
P0C0L4; P0	0	0.9189	442.9264	1326.765	1.94	0.00086	9.391974
P0C0L4; P0	0	0.9737	569.9888	1707.952	0.45	0.00025	31.71992
P0C0L4; P0	0	0.8736	663.8861	1326.765	2.25	0.00149	5.722034
P0C0L4; P0	0	0.625	442.9256	1326.762	0.28	0.00012	9.223516
P0C0L4; P0	0	1	774.1818	3093.705	1.4	0.00108	3.361939
P0C0L4; P0	0	0.8333	442.9268	1326.766	2.83	0.00125	22.21581
P0C0L4; P0	0	0.7561	442.9264	1326.765	2.01	0.00089	0
P0C0L4; P0	0	0.8197	492.2781	1474.82	1.88	0.00092	42.95472
P0C0L4; P0	0	0.8627	641.3524	1281.698	3.65	0.00234	12.25893
P0C0L4; P0	0	1	639.3195	1277.632	3.15	0.00201	5.319107
P0C0L4; P0	0	0.8889	562.6464	1685.925	3.27	0.00184	32.79515
P0C0L4; P0	0	1	598.7885	1196.57	2.7	0.00162	4.36656
P0C0L4; P0	0	1	598.7878	1196.568	1.68	0.00101	3.423467
P0C0L4; P0	0	1	744.4514	1487.895	0.04	0.00003	14.81127
P0C0L4; P0	0	1	644.3508	2574.381	0.99	0.00064	0.879733
P0C0L4; P0	0	1	598.7885	1196.57	2.7	0.00162	3.088728
P0C0L4; P0	0	1	598.7882	1196.569	2.3	0.00137	3.379773
P0C0L4; P0	0	1	516.6178	1547.839	3.04	0.00157	8.099505
P0C0L4; P0	1	0.8889	518.6359	1553.893	2.02	0.00105	11.67609
P0C0L4; P0	1	0.92	518.6356	1553.892	1.55	0.0008	20.36515
P0C0L4; P0	0	0.8654	641.3528	1281.698	4.22	0.00271	36.5496
P0C0L4; P0	0	1	598.7875	1196.568	1.07	0.00064	3.110818
P0C0L4; P0	0	1	598.789	1196.571	3.62	0.00217	0
P0C0L4; P0	0	1	598.7886	1196.57	3.01	0.0018	3.209586
P0C0L4; P0	0	1	598.7885	1196.57	2.7	0.00162	3.526876
P0C0L4; P0	0	0.9508	636.3505	1271.694	2.27	0.00144	100

P0C0L4; P0	0	1	919.8451	2757.521	1.91	0.00175	5.724564
P0C0L4; P0	0	0.9091	581.1309	2901.626	2.74	0.00159	4.158038
P0C0L4; P0	0	1	680.3757	2718.481	0.04	0.00002	2.192481
P0C0L4; P0	0	1	544.5027	2718.484	1.31	0.00071	3.460773
P0C0L4; P0	0	1	680.3768	2718.485	1.65	0.00112	2.076645
P0C0L4; P0	0	1	1029.58	3086.725	2.5	0.00258	3.345291
P0C0L4; P0	0	1	772.4356	3086.721	1.21	0.00094	5.352506
P0C0L4; P0	0	0.9778	568.975	1704.911	3.46	0.00196	8.411469
P0C0L4; P0	0	1	865.6581	4324.262	2.09	0.00181	2.478431
P0C0L4; P0	0	1	568.975	1704.911	3.46	0.00196	11.15509
P0C0L4; P0	0	0.975	568.9753	1704.911	3.99	0.00227	10.76766
P0C0L4; P0	0	0.9574	568.9755	1704.912	4.32	0.00245	12.43042
P0C0L4; P0	0	1	1081.82	4324.259	1.58	0.00171	2.319621
P0C0L4; P0	0	1	568.975	1704.91	3.35	0.0019	8.388101
P0C0L4; P0	0	1	568.975	1704.91	3.35	0.0019	7.974552
P0C0L4; P0	0	0.7067	610.3615	1219.716	1.65	0.00101	18.36178
P0C0L4; P0	0	0.7015	662.3806	1323.754	2.18	0.00145	5.777752
P0C0L4; P0	0	1	974.8261	2922.464	1.18	0.00115	2.592923
P0C0L4; P0	0	0.9846	1136.634	2272.262	0.5	0.00057	0
P0C0L4	0	0.7636	577.3285	1153.65	3.62	0.00209	5.31738
P0C0L4; P0	0	1	763.3895	1525.772	0.61	0.00047	8.361775
P0C0L4; P0	0	0.8571	691.3391	1381.671	1.48	0.00102	0
P0C0L4; P0	0	0.9121	758.0922	2272.262	0.68	0.00052	3.574229
P0C0L4; P0	1	0.697	470.6018	1409.791	2.15	0.00101	16.73803
P0C0L4; P0	0	0.9931	1314.205	2627.403	0.96	0.00127	0
P0C0L4; P0	0	1	729.1823	2913.707	0.96	0.0007	7.451555
P0C0L4; P0	0	1	971.9078	2913.709	1.43	0.00139	3.05197
P0C0L4; P0	0	1	592.3665	1775.085	2.02	0.0012	2.265197
P0C0L4; P0	1	0.9048	420.9138	1260.727	0.52	0.00022	17.07265
P0C0L4; P0	0	0.76	399.5595	1196.664	1.81	0.00072	67.22067
P0C0L4; P0	0	0.7143	468.5802	1403.726	2.14	0.001	12.54043
P0C0L4; P0	0	1	487.287	1459.846	3.35	0.00163	14.59365
P0C0L4; P0	0	0.1515	433.9207	1299.748	2.28	0.00099	19.51768
P0C0L4; P0	0	0.8611	852.9576	1704.908	1.96	0.00167	3.193368
P0C0L4; P0	0	0.95	463.0204	1849.06	1.18	0.00054	18.91823
P0C0L4; P0	0	1	598.788	1196.569	1.89	0.00113	3.521297
P0C0L4; P0	0	0.75	399.5589	1196.662	0.43	0.00017	6.72325
P0C0L4	0	0.9697	797.3947	1593.782	0.63	0.0005	2.018869
P0C0L4; P0	0	0.3429	885.9619	1770.917	1.29	0.00114	6.470485
P0C0L4; P0	0	0.8367	629.3591	1257.711	2.27	0.00143	10.64508
P0C0L4; P0	0	1	544.5025	2718.483	0.97	0.00053	29.77047
P0C0L4; P0	0	0.6286	463.0205	1849.06	1.51	0.0007	4.319591
P0C0L4; P0	0	1	399.5279	1196.569	2.29	0.00091	48.74908
P0C0L4; P0	0	0.6579	520.9397	1560.805	1.24	0.00065	3.713353
P0C0L4; P0	0	0.7436	468.5805	1403.727	2.79	0.00131	33.81586
P0C0L4; P0	0	0.8	441.9229	1323.754	2.36	0.00104	14.17325
P0C0L4; P0	0	0.8421	669.8644	1338.722	5.43	0.00363	17.00924
P0C0L4; P0	0	0.75	520.9398	1560.805	1.36	0.00071	4.419956
P0C0L4; P0	0	0.8182	409.8979	1227.679	3.48	0.00142	32.31134
P0C0L4; P0	0	1	899.1001	2695.286	2.5	0.00224	4.423165
P0C0L4; P0	0	0.9444	636.3513	1271.695	3.61	0.0023	100
P0C0L4; P0	0	0.9268	852.9576	1704.908	1.96	0.00167	6.797606

P0C0L4; P0	0	1	568.974	1704.907	1.63	0.00093	24.27141
P0C0L4; P0	1	1	421.0057	1681.001	0.65	0.00027	15.98904
P0C0L4; P0	0	1	542.2906	1083.574	1.04	0.00056	0
P0C0L4; P0	0	0.9535	602.325	1203.643	2.68	0.00161	4.264612
P0C0L4; P0	0	0.8667	662.0068	1984.006	1.22	0.00081	0
P0C0L4; P0	0	0.9714	569.99	1707.955	2.59	0.00147	14.00191
P0C0L4; P0	1	1	538.2952	1612.871	1.6	0.00086	41.40491
P0C0L4; P0	0	1	689.3657	1377.724	1.73	0.00119	2.458498
P0C0L4; P0	0	0.9375	662.006	1984.004	0.02	0.00001	83.18974
P0C0L4; P0	0	1	399.8803	1197.626	2.59	0.00104	7.288148
P0C0L4; P0	0	1	626.8574	1252.708	1.42	0.00089	4.032603
P0C0L4; P0	1	1	714.0414	2140.11	2.31	0.00165	12.5119
P0C0L4; P0	0	1	399.5277	1196.569	1.9	0.00076	51.43059
P0C0L4; P0	1	0.8485	418.0077	1669.009	2.36	0.00098	17.26229
P0C0L4; P0	0	1	409.8975	1227.678	2.59	0.00106	43.0858
P0C0L4; P0	0	0.7619	399.5596	1196.664	2.27	0.0009	39.83864
P0C0L4; P0	1	0.7556	806.9397	1612.872	2.21	0.00178	5.22815
P0C0L4; P0	0	0.7895	698.9085	1396.81	1.35	0.00095	17.07978
P0C0L4; P0	0	0.871	852.9581	1704.909	2.53	0.00216	8.635193
P0C0L4; P0	0	0.4444	476.2759	1426.813	-0.25	-0.00012	11.83178
P0C0L4; P0	0	0.9512	780.9061	1560.805	1.53	0.00119	10.07023
P0C0L4; P0	1	1	459.2541	1833.994	3.3	0.00151	22.44029
P0C0L4; P0	0	1	906.833	2718.484	1.37	0.00124	2.811582
P0C0L4; P0	0	1	610.6805	1830.027	3.27	0.00199	9.225146
P0C0L4; P0	0	1	410.9166	1230.735	2.08	0.00085	37.53095
P0C0L4; P0	0	0.76	590.3304	1179.654	3.91	0.00231	0
P0C0L4; P0	0	0.7759	492.2392	983.4711	1.79	0.00088	0
P0C0L4; P0	0	0.8974	813.9114	1626.816	2.08	0.00169	11.69149
P0C0L4; P0	1	1	535.7828	2140.11	2.28	0.00122	34.20444
P0C0L4; P0	0	0.9062	542.9431	1626.815	1.47	0.0008	3.061351
P0C0L4; P0	1	1	476.6142	1427.828	2.2	0.00105	38.80282
P0C0L4; P0	0	0.4286	466.2755	1396.812	2.96	0.00138	7.190145
P0C0L4; P0	1	0.6957	592.8262	2368.283	2.89	0.00171	0
P0C0L4; P0	0	0.5312	814.434	1627.861	2.25	0.00183	10.15128
P0C0L4; P0	0	0.9583	401.8856	1203.642	2.41	0.00097	13.84584
P0C0L4; P0	0	0.7368	411.8785	1233.621	1.01	0.00042	5.811368
P0C0L4; P0	0	0.8222	511.3119	1021.617	3.26	0.00167	36.4212
P0C0L4; P0	0	1	401.8854	1203.642	1.88	0.00075	27.42783
P0C0L4; P0	0	0.8667	511.3113	1021.615	2.13	0.00109	7.841673
P0C0L4; P0	0	0.75	424.5696	1271.694	2.83	0.0012	14.03217
P0C0L4; P0	0	0.8571	409.8972	1227.677	1.77	0.00072	37.2449
P0C0L4; P0	1	1	420.9137	1260.727	0.3	0.00013	29.13715
P0C0L4; P0	0	1	899.0994	2695.284	1.75	0.00157	71.5943
P0C0L4; P0	0	0.9286	1348.146	2695.286	2.48	0.00334	0
P0C0L4; P0	0	0.5714	466.2754	1396.812	2.76	0.00129	0
P0C0L4; P0	0	0.9556	665.8625	1330.718	2.22	0.00148	3.229429
P0C0L4; P0	0	0.9259	441.9233	1323.755	3.26	0.00144	36.00274
P0C0L4; P0	0	0.7963	439.2596	877.512	1.03	0.00045	5.101516
P0C0L4; P0	0	0.9355	575.6501	1724.936	2.8	0.00161	12.00104
P0C0L4; P0	0	1	598.7882	1196.569	2.3	0.00137	13.70312
P0C0L4; P0	1	1	501.5902	1502.756	1.24	0.00062	0
P0C0L4; P0	0	0.7805	780.9072	1560.807	2.85	0.00223	17.75122

P0C0L4; P0	1	1	592.8243	2368.276	-0.21	-0.00012	3.518496
P0C0L4; P0	1	0.8421	538.2929	1612.864	-2.83	-0.00152	86.75069
P0C0L4; P0	1	0.9474	577.6785	1731.021	1.22	0.0007	39.13348
P0C0L4; P0	0	0.7073	407.2438	1219.717	2.74	0.00111	40.93847
P0C0L4; P0	0	0.9	680.3768	2718.485	1.74	0.00118	0
P0C0L4; P0	0	0.7736	527.2651	1053.523	1.79	0.00094	31.35412
P0C0L4; P0	1	0.9412	508.9726	1524.903	0.28	0.00014	40.52543
P0C0L4; P0	1	0.9412	508.9726	1524.903	0.28	0.00014	40.52543
P0C0L4; P0	1	0.8056	835.0076	1669.008	1.86	0.00155	6.720405
P0C0L4; P0	0	0.5667	424.5695	1271.694	2.47	0.00105	16.01746
P0C0L4; P0	1	0.7143	518.6358	1553.893	1.9	0.00098	18.45007
P0C0L4; P0	0	0.95	548.3139	1095.621	2.33	0.00128	4.190257
P0C0L4; P0	1	1	535.7826	2140.109	1.82	0.00097	23.43074
P0C0L4; P0	0	1	526.7361	1052.465	0.53	0.00028	0
P0C0L4; P0	0	0.9091	458.2622	1830.027	3.3	0.00151	38.84898
P0C0L4; P0	0	0.2727	586.3351	1756.991	2.41	0.00141	7.217451
P0C0L4; P0	0	0.8571	511.3111	1021.615	1.65	0.00084	7.670605
P0C0L4; P0	0	0.6977	629.3596	1257.712	2.95	0.00185	5.895948
P0C0L4; P0	0	0.7551	554.8356	1108.664	3.08	0.0017	11.00247
P0C0L4; P0	0	1	617.3145	1233.622	1.64	0.00101	8.660937
P0C0L4	0	0.3	1092.866	3276.583	3.61	0.00394	2.290275
P0C0L4; P0	0	0.7838	554.8355	1108.664	2.86	0.00158	35.13182
P0C0L4; P0	0	1	602.3248	1203.642	2.37	0.00143	6.424095
P0C0L4; P0	1	0.9	630.8679	1260.729	1.84	0.00116	7.584723
P0C0L4; P0	0	0.7143	722.4279	1443.848	1.27	0.00092	6.84609
P0C0L4; P0	0	1	598.7878	1196.568	1.58	0.00095	6.408106
P0C0L4; P0	0	0.9032	419.9082	1257.71	1.53	0.00064	17.20947
P0C0L4; P0	0	0.8444	630.3146	1259.622	0.64	0.0004	2.893304
P0C0L4; P0	0	0.8571	560.293	1119.579	2.17	0.00122	6.93324
P0C0L4; P0	0	0.9487	607.3416	1213.676	3.03	0.00184	21.54747
P0C0L4; P0	0	1	904.4326	2711.283	3.39	0.00306	0
P0C0L4; P0	0	1	610.6796	1830.024	1.77	0.00108	0
P0C0L4; P0	0	0.9583	590.9792	1770.923	4.93	0.00291	0
P0C0L4; P0	0	0.8723	492.2389	983.4705	1.17	0.00058	11.76828
P0C0L4; P0	0	1	852.9577	1704.908	2.03	0.00173	6.600808
P0C0L4; P0	0	0.7368	511.3101	1021.613	-0.2	-0.0001	30.00844
P0C0L4; P0	0	1	598.7905	1196.574	3.61	0.00216	0
P0C0L4; P0	0	0.9697	399.5274	1196.568	1.14	0.00045	11.45088
P0C0L4; P0	0	1	526.7377	1052.468	3.67	0.00193	0
P0C0L4; P0	1	1	493.3085	1477.911	1.12	0.00055	42.24174
P0C0L4; P0	1	0.75	592.8261	2368.282	2.68	0.00159	11.49942
P0C0L4; P0	1	1	697.4254	2090.262	1.52	0.00106	33.36861
P0C0L4; P0	0	0.8235	560.2942	1119.581	4.35	0.00244	15.10129
P0C0L4; P0	0	1	806.3716	2417.1	0.9	0.00072	0
P0C0L4; P0	0	0.9474	560.296	1119.585	7.52	0.00421	0
P0C0L4; P0	0	0.8611	774.425	1547.843	5.53	0.00428	27.70102
P0C0L4; P0	0	0.9778	488.7217	976.436	1.51	0.00074	25.58318
P0C0L4; P0	0	0.75	554.8341	1108.661	0.43	0.00024	100
P0C0L4; P0	0	0.8684	560.2941	1119.581	4.24	0.00238	2.274849
P0C0L4; P0	0	0.9348	492.2383	983.4694	0.05	0.00003	16.58234
P0C0L4; P0	0	0.7941	560.2938	1119.58	3.7	0.00207	0
P0C0L4; P0	0	1	687.9223	1374.837	1.89	0.0013	5.978024

P0C0L4; P0	1	0.9583	403.9734	1612.872	1.97	0.00079	21.62358
P0C0L4; P0	0	0.8545	554.8359	1108.664	3.63	0.00201	18.59108
P0C0L4; P0	1	0.8333	456.9215	1368.75	1.77	0.00081	16.56412
P0C0L4; P0	0	0.9091	482.7832	964.5591	0.7	0.00034	44.7948
P0C0L4; P0	0	0.8421	525.8064	1050.606	2.08	0.00109	11.52102
P0C0L4; P0	1	0.9	420.9141	1260.728	1.18	0.00049	37.17281
P0C0L4; P0	0	0.7353	458.9526	1374.843	6.07	0.00278	39.229
P0C0L4; P0	0	1	562.8051	1124.603	2.27	0.00128	11.01421
P0C0L4; P0	0	1	482.756	964.5048	1.57	0.00076	11.29565
P0C0L4; P0	0	0.9375	598.8365	1196.666	3.4	0.00203	16.29111
P0C0L4; P0	0	0.9524	742.382	1483.757	1.16	0.00086	0
P0C0L4; P0	0	0.8293	557.8166	1114.626	3.95	0.0022	13.47637
P0C0L4; P0	0		586.3353	1756.991	2.72	0.00159	0
P0C0L4; P0	0	1	399.5279	1196.569	2.21	0.00088	12.98866
P0C0L4; P0	0	0.76	544.6714	1632	1.33	0.00072	8.001515
P0C0L4; P0	0	0.9048	617.0269	1849.066	4.75	0.00293	29.99261
P0C0L4; P0	0	1	458.9506	1374.837	1.88	0.00086	44.57616
P0C0L4; P0	0	0.8684	525.806	1050.605	1.38	0.00072	21.83972
P0C0L4; P0	0	1	447.5626	1340.673	1.2	0.00053	25.67226
P0C0L4; P0	0	0	634.3692	1901.093	2.28	0.00145	16.75078
P0C0L4	0	0.9259	1044.829	3132.473	1.28	0.00134	0.680275
P0C0L4; P0	0	0.9677	560.2945	1119.582	4.9	0.00274	0
P0C0L4; P0	0	0.4	599.3169	1197.627	2.78	0.00167	3.641306
P0C0L4; P0	0	0.8846	542.9433	1626.815	1.92	0.00104	5.438027
P0C0L4; P0	0	0.6061	407.2438	1219.717	2.59	0.00105	13.25358
P0C0L4; P0	1	1	714.0369	2140.096	-3.93	-0.00281	34.70642
P0C0L4; P0	1	0.8125	621.8536	2484.393	2.99	0.00186	41.53924
P0C0L4; P0	1	1	420.915	1260.73	3.21	0.00135	58.33232
P0C0L4; P0	0	1	458.9503	1374.836	1.08	0.00049	76.27785
P0C0L4; P0	0	1	548.3137	1095.62	2	0.0011	0
P0C0L4; P0	0	0.75	419.9087	1257.711	2.62	0.0011	4.821672
P0C0L4; P0	0	0.76	862.9706	1724.934	1.82	0.00157	43.96324
P0C0L4; P0	0	1	674.577	2695.286	2.61	0.00176	0
P0C0L4; P0	1	0.8	420.9142	1260.728	1.47	0.00062	30.87623
P0C0L4; P0	0	1	730.4273	1459.847	3.99	0.00291	8.528147
P0C0L4; P0	0	0.25	576.7958	2304.161	2.44	0.0014	29.14127
P0C0L4; P0	0	0.9118	525.8063	1050.605	1.96	0.00103	3.130056
P0C0L4; P0	0	1	780.9059	1560.805	1.29	0.00101	13.83405
P0C0L4; P0	0	0.92	568.9929	1704.964	5.19	0.00295	8.115194
P0C0L4; P0	0	0.7586	419.9086	1257.711	2.55	0.00107	46.55788
P0C0L4; P0	0	1	542.291	1083.575	1.72	0.00093	6.112218
P0C0L4; P0	0	0.9375	1064.557	2128.107	0.65	0.00069	0
P0C0L4; P0	0	1	629.9681	1887.89	2.5	0.00157	78.46582
P0C0L4; P0	0	1	562.8051	1124.603	2.38	0.00134	0
P0C0L4; P0	0	1	548.3141	1095.621	2.67	0.00146	0
P0C0L4; P0	0	1	568.9743	1704.908	2.17	0.00123	0
P0C0L4; P0	1	1	421.0075	1681.008	4.86	0.00204	58.07848
P0C0L4; P0	0	1	590.9782	1770.92	3.17	0.00187	0
P0C0L4; P0	0	0.6207	630.3162	1259.625	3.25	0.00205	9.959315
P0C0L4; P0	0	1	665.8615	1330.716	0.75	0.0005	0
P0C0L4; P0	0	1	639.3193	1277.631	2.87	0.00183	14.56757
P02655	0	0.8592	662.3746	1323.742	1.54	0.00102	14.8714

P02655	0	0.6415	441.919	1323.742	1.92	0.00085	10.41497
P02655	0	0.8462	662.3749	1323.743	2.09	0.00139	5.537033
P02655	0	0.8226	663.3707	1325.734	2.91	0.00193	13.41453
P02655	0	0.7872	442.5827	1325.734	2.44	0.00108	15.04031
P02655	0	0.8113	663.3702	1325.733	2.17	0.00144	28.09119
P02655	0	1	590.324	1179.641	2.57	0.00151	4.025693
P02655	0	0.7966	662.3749	1323.742	2	0.00133	7.405938
P02655	0	0.8923	715.8507	1430.694	1.42	0.00102	27.43355
P02655	0	0.7925	525.6036	1574.796	1.47	0.00077	14.19712
P02655	0	1	841.1215	2521.35	1.44	0.00121	4.876062
P02655	0	1	1261.179	2521.35	1.67	0.0021	0
P02655	0	1	787.9017	1574.796	1.36	0.00107	6.65253
P02655	0	1	787.9021	1574.797	1.82	0.00144	2.586982
P02655	0	0.7963	525.6037	1574.797	1.58	0.00083	9.362435
P02655	0	0.8108	442.5823	1325.732	1.47	0.00065	40.79031
P02655	0	0.8049	662.3783	1323.749	7.17	0.00474	0
P02655	0	0.5185	441.9187	1323.742	1.37	0.0006	10.65978
P02655	0	0.8788	591.3184	1181.629	1	0.00059	32.11076
P02655	0	0.1429	525.6038	1574.797	1.82	0.00095	4.033875
P02655	0	0.68	394.548	1181.63	1.09	0.00043	6.971853
P02655	0	0.6207	662.3726	1323.738	-1.41	-0.00093	18.63087
P02655	0	0.8571	631.0933	2521.351	2.09	0.00132	5.803618
P02745	1	1	687.1086	2745.413	0.04	0.00003	2.423619
P02745	1	1	915.8117	2745.421	2.89	0.00265	17.71249
P02745	0	1	825.0776	2473.218	1.04	0.00086	2.767003
P02745	0	0.9825	978.5327	2933.584	1.32	0.00129	4.815495
P02745	0	1	477.5781	1430.72	0.67	0.00032	42.39591
P35542	0	1	638.827	1276.647	2.49	0.00159	3.479472
P35542	0	0.8125	565.8333	1130.659	2.82	0.00159	6.147806
P35542	0	1	431.5523	1292.642	2.98	0.00129	26.02918
P35542	0	1	566.7759	1132.545	2.71	0.00154	10.89101
P35542	0	1	638.8268	1276.646	2.11	0.00135	3.803209
P35542	0	1	646.8243	1292.641	2.24	0.00145	0
P35542	0	1	426.2199	1276.645	1.09	0.00047	34.71687
P35542	0	0.6471	406.5799	1217.725	0.63	0.00026	9.542996
P35542	0	0.7778	406.5803	1217.726	1.46	0.00059	11.04507
P35542	0	0.8	377.5574	1130.658	1.51	0.00057	30.16945
P35542	0	1	519.7589	1038.511	1.99	0.00103	25.10227
P35542	0	0.3636	406.5811	1217.729	3.41	0.00139	19.70819
P35542	0	0.6364	406.5804	1217.727	1.91	0.00078	17.12051
P35542	0	0.6786	565.8326	1130.658	1.74	0.00098	42.02954
P35542	0	0.7097	565.8342	1130.661	4.44	0.00251	0
P02746	0	1	627.3399	1880.005	2.56	0.0016	8.28067
P02746	0	0.9818	627.3401	1880.006	2.85	0.00179	10.93156
P02746	0	0.8545	610.8233	1220.639	2.72	0.00166	3.670751
P02746	0	1	669.9885	2007.951	2.34	0.00157	11.55478
P02746	0	1	602.3099	1804.915	0.66	0.0004	31.65663
P02746	0	0.973	538.77	1076.533	-1.1	-0.00059	10.61958
P02746	0	0.7	407.5514	1220.64	3.04	0.00123	32.50603
P13727	0	1	464.2411	1390.709	2.5	0.00116	15.51724
P13727	0	1	602.3251	1804.961	1.87	0.00112	34.83067
P13727	0	1	425.5459	1274.623	1.42	0.0006	23.42044

P13727	0	0.9259	706.3731	2117.105	0.26	0.00018	23.72067
P13727	0	0.9118	706.3734	2117.106	0.69	0.00049	24.69586
P13727	0	0.975	565.7649	1130.523	2.79	0.00158	25.40351
P13727	0	1	695.857	1390.707	1.12	0.00078	0
P13727	0	1	377.5117	1130.521	1.02	0.00038	14.01315
P04180	0	1	831.645	3323.558	2.46	0.00204	20.63034
P04180	0	0.9219	725.8657	1450.724	0.87	0.00063	15.96875
P04180	0	1	790.4091	1579.811	0.93	0.00074	14.02369
P04180	0	1	1108.526	3323.563	3.8	0.00421	0
P04180	0	0.9412	510.2911	1528.859	1.45	0.00074	26.58681
P04180	0	0.5926	527.2747	1579.81	0.15	0.00008	0
P04180	0	0.85	527.2748	1579.81	0.27	0.00014	21.59788
P04180	0	0.6667	510.2898	1528.855	-0.94	-0.00048	47.79652
P08833	0	1	558.9869	1674.946	3.17	0.00177	44.31567
P08833	0	0.9167	733.884	1466.761	2.13	0.00156	5.571077
P08833	0	0.9796	489.5924	1466.763	3.41	0.00167	14.24485
P08833	0	1	489.5916	1466.76	1.66	0.00081	0
P08833	0	1	589.7824	1178.557	3.3	0.00194	0
P08833	0	0.9286	733.8842	1466.761	2.3	0.00169	5.355354
P08833	0	0.48	395.252	1183.741	1.09	0.00043	44.81193
P08833	0	0.9825	765.9247	1530.842	2.21	0.00169	0
P08833	0	0.9667	489.5909	1466.758	0.35	0.00017	59.20386
P08833	0	0.6	517.329	1033.651	1.09	0.00056	0
P08833	0	0.9444	558.9865	1674.945	2.41	0.00134	31.97291
Q12805	0	1	613.2905	1837.857	1.1	0.00067	14.49924
Q12805	0	1	691.9492	2073.833	-3.03	-0.0021	0
Q12805	0	1	734.3211	1467.635	1.7	0.00125	3.682209
Q12805	0	0.9474	682.906	1364.805	1.44	0.00099	4.025994
Q12805	0	0.7	455.6065	1364.805	1.68	0.00076	43.65178
Q12805	0	1	691.9495	2073.834	-2.51	-0.00173	0
Q12805	0	0.625	919.4302	1837.853	-1.05	-0.00097	18.64239
Q12805	0	0.4737	682.9071	1364.807	3.05	0.00208	6.377059
P55056	0	1	563.9338	1689.787	3.33	0.00188	14.24275
P55056	0	1	563.9336	1689.786	3	0.00169	49.68665
P55056	0	0.64	608.8557	1216.704	1.96	0.00119	2.6773
P55056	0	0.7885	584.8362	1168.665	4.07	0.00238	18.66071
P55056	0	0.8571	584.8353	1168.663	2.4	0.0014	17.84568
P55056	0	0.814	584.8357	1168.664	3.13	0.00183	8.575201
P55056	0	0.8261	516.3219	1031.637	2.54	0.00131	31.48803
P55056	0	0.8333	512.7836	1024.56	1.43	0.00073	27.56898
P55056	0	0.5455	390.2254	1168.662	1.03	0.0004	4.714448
P01591	0	1	863.8936	1726.78	0.88	0.00076	1.313522
P01591	0	0.9375	767.362	1533.717	1.18	0.00091	2.645135
P01591	0	0.8148	523.9502	1569.836	1.46	0.00076	5.896484
P01591	0	0.8621	511.9097	1533.714	-0.3	-0.00015	9.917511
P01591	0	0.3023	544.8503	1088.693	1.52	0.00083	30.48698
P01591	0	1	767.3604	1533.713	-0.96	-0.00074	0
Q7Z794; P3	0	0.9778	588.6707	1763.998	4.65	0.00273	64.9304
P35908	0	0.7234	499.6144	1496.829	5.88	0.00294	20.98262
P35908	0	1	440.8662	1320.584	0.95	0.00042	24.33473
P35908	0	0.7	495.9237	1485.756	1.95	0.00096	62.97563
P35908	0	0.5652	560.3561	1119.705	6.64	0.00372	5.87361

Q7Z794; P3	0	0.5	540.6342	1619.888	0.36	0.00019	5.406788
P43121	0	0.7907	566.3201	1696.946	3.45	0.00195	3.684172
P43121	0	1	581.9853	1743.941	3.2	0.00186	10.1243
P43121	0	0.7692	469.9576	1407.858	1.55	0.00073	35.11899
P43121	0	0.4211	602.8201	1204.633	3.21	0.00193	6.642983
P43121	0	0.9259	872.4731	1743.939	1.88	0.00164	11.6148
O43866	0	1	899.379	2696.122	4.23	0.0038	0
O43866	0	0.8333	414.8883	1242.65	0.12	0.00005	23.7085
O43866	0	0.9762	583.3112	1165.615	2.26	0.00132	8.060805
O43866	0	1	611.9738	1833.907	1.39	0.00085	49.74887
P32119	1	1	717.7642	2151.278	1.93	0.00139	35.17534
Q06830; P3	0	0.9773	678.3939	1355.781	3.13	0.00212	7.229448
Q06830; P3	0	0.7308	452.597	1355.776	0.12	0.00006	61.50156
P32119	0	0.871	575.8593	1150.711	3.43	0.00197	51.97497
P32119	0	1	468.9592	1404.863	3.89	0.00182	30.89872
P02741	0	0.9574	703.7158	2109.133	3.33	0.00234	7.756295
P02741	1	0.9688	613.017	1837.036	8.34	0.00511	29.84011
P02741	0	0.6129	475.5945	1424.769	1.7	0.00081	59.3195
P02741	0	1	433.9627	1299.874	0.9	0.00039	17.23426
P02741	0	1	712.8859	1424.765	-1.27	-0.0009	41.47656
Q6UX71	0	0.8205	481.6134	1442.826	1.48	0.00071	26.74689
Q6UX71	0	0.9286	608.9473	1824.827	-2.19	-0.00133	9.35117
Q6UX71	0	1	429.5543	1286.648	2.03	0.00087	0
Q6UX71	0	1	431.2501	1291.736	0.39	0.00017	4.652308
Q6UX71	0	1	643.8273	1286.647	1.31	0.00084	37.55793
P13796	0	0.9706	625.3565	1874.055	1.94	0.00121	14.79839
P13796	0	1	578.8624	1156.717	0.38	0.00022	23.36059
P13796	0	0.5667	573.9966	1719.975	4.75	0.00272	0
P13796	0	0.7714	480.2501	1438.736	1.3	0.00062	0
P63267; P6	0	0.7826	483.9488	1449.832	6.36	0.00307	34.84767
P63267; P6	0	1	487.2637	1459.777	0.94	0.00046	46.31744
A5A3E0; P6	0	0.8636	645.6713	1934.999	2.72	0.00175	55.86254
A5A3E0; Q9	0	1	505.9223	1515.752	2.11	0.00107	29.73994
P63267; P6	0	0.7826	483.9488	1449.832	6.36	0.00307	34.84767
P63267; P6	0	1	487.2637	1459.777	0.94	0.00046	46.31744
A5A3E0; P6	0	0.8636	645.6713	1934.999	2.72	0.00175	55.86254
A5A3E0; Q9	0	1	505.9223	1515.752	2.11	0.00107	29.73994
O00187	0	1	912.4517	1823.896	1.91	0.00175	15.7204
O00187	0	1	632.3079	1894.909	2.04	0.00129	16.74776
O00187	1	1	487.9421	1461.812	2.44	0.00119	32.54512
O00187	0	0.913	566.3087	1131.61	2.91	0.00165	3.02803
P18428	0	1	464.9697	1392.895	0.48	0.00022	37.52213
P18428	0	0.8222	696.3854	1391.763	-1.16	-0.00081	29.77236
P18428	0	1	383.8941	1149.668	2.47	0.00094	25.23315
P18428	0	1	572.3428	1715.014	2.55	0.00146	49.09436
P15144	0	0.9091	671.6872	2013.047	4.56	0.00306	0
P15144	1	1	434.9427	1302.813	2.95	0.00128	28.25023
P15144	1	1	545.3	1633.885	2.58	0.0014	4.215817
P15144	1	1	545.3	1633.885	2.58	0.0014	4.215817
P15144	0	0.7778	515.8112	1030.615	2.41	0.00124	14.16548
P07996	0	1	647.3102	1939.916	3.61	0.00233	14.82577
P07996	0	0.4524	695.9067	1390.806	2.87	0.002	27.48921

P07996	0	1	493.573	1478.704	4.97	0.00245	12.68804
P07996	0	0.48	540.9528	1620.844	2.03	0.00109	33.10731
P02763	0	1	714.3925	2141.163	1.19	0.00085	5.92102
P02763	0	1	536.0457	2141.161	0.36	0.00019	7.142251
P02763	0	1	714.3925	2141.163	1.28	0.00091	6.891572
P02763	0	1	536.0465	2141.164	1.84	0.00099	11.43514
P02763	0	1	714.3924	2141.163	1.11	0.00079	9.436403
P02763	0	1	943.9582	1886.909	0.86	0.00082	0
P02763	0	1	629.6424	1886.913	2.73	0.00172	6.885006
P02763	0	1	943.9593	1886.911	2.03	0.00191	0
P02763	0	1	871.9072	1742.807	1.01	0.00088	25.03623
P02763	0	0.9643	583.9594	1749.864	1.57	0.00092	11.7203
P02763	0	0.9048	867.441	1733.875	5.17	0.00449	60.56815
P02763	0	0.9718	867.4374	1733.867	0.95	0.00082	11.49671
P02763	0	0.8621	578.6281	1733.87	2.28	0.00132	7.274514
P02763	0	0.9048	475.0204	1897.06	1.78	0.00084	4.895586
P02763	0	0.9388	629.6416	1886.91	1.47	0.00093	8.506496
P02763; P1	0	0.7966	497.7645	994.5217	1.34	0.00066	5.573522
P02763; P1	0	1	652.8492	1304.691	0.31	0.0002	3.561763
P02763	0	0.7895	700.8693	1400.731	0.98	0.00069	7.169956
P02763	0	1	633.0246	1897.059	1.46	0.00093	33.07316
P02763	0	0.9375	475.0196	1897.057	0.1	0.00005	2.942715
P02763	0	0.9375	475.0204	1897.06	1.65	0.00078	2.675806
P02763	0	1	633.0249	1897.06	1.95	0.00123	2.284936
P02763; P1	1	0.6667	484.2695	1450.794	0.2	0.0001	12.35338
P02763; P1	0	0.8333	497.7651	994.5229	2.56	0.00127	0
P02763; P1	1	0.7059	484.2695	1450.794	0.27	0.00013	29.48372
P02763; P1	1	0.619	484.2691	1450.793	-0.55	-0.00027	12.07269
P02763	0	1	629.6422	1886.912	2.34	0.00147	25.50508
P02763; P1	1	0.6667	484.2699	1450.795	1.02	0.0005	28.49728
P02763; P1	1	0.7	484.269	1450.792	-0.87	-0.00042	17.58412
P02763	0	1	628.8189	1256.63	1.98	0.00124	5.176771
P02763; P1	0	1	652.8502	1304.693	1.71	0.00112	4.641958
P02763	0	1	666.3605	1997.067	4.43	0.00295	0
P02763; P1	1	0.8824	484.2694	1450.794	0.14	0.00007	47.73605
P02763	0	0.5263	467.5821	1400.732	1.24	0.00058	8.623795
P02763	0	1	666.3593	1997.063	2.6	0.00173	11.48709
P02763	0	1	500.0201	1997.058	0.15	0.00007	7.745005
P02763; P1	1	0.5	436.2366	1306.695	2.87	0.00125	16.76086
P02763	0	0.871	467.581	1400.729	-1.05	-0.00049	7.91581
P02763; P1	0	1	580.7996	1160.592	2.78	0.00161	13.73507
P02763	0	0.8	438.9952	1752.959	2.56	0.00112	5.344932
P02763	0	1	628.8197	1256.632	3.34	0.0021	52.48353
P02763	0	1	633.0247	1897.059	1.56	0.00099	25.11193
P02763; P1	1	0.5	436.2366	1306.695	2.87	0.00125	16.76086
P02763	0	1	629.6404	1886.907	-0.47	-0.0003	46.19625
P02763	0	1	666.3589	1997.062	1.96	0.0013	6.146193
P02763	0	0.0952	475.0215	1897.064	4.09	0.00194	0
P01857; P0	0	0.8902	537.3005	1609.887	3.05	0.00164	13.45807
P01857; P0	0	0.9608	805.447	1609.887	2.87	0.00231	0
P01857	0	0.8448	492.2893	1474.853	1.69	0.00083	16.05057
P01857	0	0.8873	737.9314	1474.856	3.21	0.00237	6.861941

P01857	0	0.8475	492.2898	1474.855	2.75	0.00135	14.36244
P01857	0	0.9815	656.0087	1966.011	2.72	0.00178	7.961033
P01857	0	1	656.0087	1966.012	2.81	0.00184	8.859803
P01857; P0	0	0.6667	483.9505	1449.837	2.21	0.00107	9.518343
P01857; P0	0	1	483.9502	1449.836	1.51	0.00073	24.07588
P01857	0	1	721.3821	2162.132	2.65	0.00191	7.10177
P01857	0	1	819.9475	3276.768	1.37	0.00112	3.988913
P01857	0	1	656.1607	3276.774	3.26	0.00214	25.85149
P01857	0	1	656.1602	3276.772	2.52	0.00165	7.613265
P01857	0	1	607.5648	2427.237	2.43	0.00147	21.24873
P01857	0	1	809.7507	2427.238	2.51	0.00203	5.750351
P01857; P0	0	0.9643	563.8591	1126.711	3.07	0.00173	8.029201
P01857; P0	0	1	715.8931	1430.779	2.09	0.00149	3.09548
P01857; P0	0	1	477.5974	1430.778	1.06	0.0005	11.75311
P01857; P0	0	0.9697	715.8933	1430.779	2.34	0.00168	2.854816
P01857; P0	0	0.9672	715.8933	1430.779	2.34	0.00168	5.368532
P01857; P0	0	1	715.8925	1430.778	1.15	0.00082	0
P01857	0	0.913	627.3385	3132.663	0.55	0.00035	79.04011
P01857; P0	0	1	477.598	1430.779	2.33	0.00111	7.543014
P01857; P0	0	0.8621	477.5976	1430.778	1.5	0.00072	19.59222
P01857; P0	0	0.5357	537.3008	1609.888	3.5	0.00188	75.55756
P01857	0	0.7857	721.3821	2162.132	2.74	0.00197	17.24101
P01857; P0	0	0.8889	563.8597	1126.712	4.26	0.0024	9.855687
P01857	0	0.6458	665.8794	1330.752	2.1	0.0014	12.72256
P01857	0	0.75	721.3826	2162.133	3.42	0.00246	55.10471
P01857	0	1	721.3831	2162.135	4.01	0.00289	11.25961
P01857	0	0.7143	673.3479	2018.029	2.61	0.00175	4.239983
P01857	0	0.85	656.0088	1966.012	2.9	0.0019	8.825878
P01857; P0	0	0.6981	426.218	851.4287	-0.51	-0.00022	5.356847
P01857	0	1	721.3808	2162.128	0.88	0.00063	11.32693
P01857	0	0.8182	737.9281	1474.849	-1.26	-0.00093	0
P01857	0	0.5	607.5663	2427.243	4.84	0.00294	28.7832
P01857; P0	0	0.7619	477.5977	1430.779	1.76	0.00084	49.38679
P19652	0	0.9302	574.9561	1722.854	2.29	0.00131	2.16749
P19652	0	1	853.9317	1706.856	0.7	0.0006	9.67487
P19652	0	0.9677	569.6246	1706.859	2.48	0.00141	10.18598
P19652	0	0.8095	569.6243	1706.858	1.94	0.00111	7.231671
P19652	0	1	460.274	1378.807	-0.11	-0.00005	52.58355
P19652	0	1	460.2744	1378.809	0.82	0.00037	5.731555
P19652	0	1	689.9083	1378.809	1.22	0.00084	3.456521
P19652	0	1	460.2745	1378.809	1.01	0.00047	16.47527
P19652	0	1	460.2746	1378.809	1.21	0.00056	43.86533
P19652	0	1	1129.043	2257.078	1.43	0.00161	0
P19652	0	1	753.0307	2257.078	1.15	0.00087	2.772164
P19652	0	1	548.0466	2189.164	1.91	0.00105	7.236839
P19652	0	1	730.3926	2189.163	1.42	0.00103	11.63029
P19652	0	1	548.0455	2189.16	0.02	0.00001	31.38284
P19652	0	1	1129.04	2257.073	-0.95	-0.00107	0
P19652	0	0.9889	753.0311	2257.079	1.64	0.00123	3.364546
P02763; P1	0	0.7966	497.7645	994.5217	1.34	0.00066	5.573522
P02763; P1	0	1	652.8492	1304.691	0.31	0.0002	3.561763
P02763; P1	1	0.6667	484.2695	1450.794	0.2	0.0001	12.35338

P02763; P1	0	0.8333	497.7651	994.5229	2.56	0.00127	0
P02763; P1	1	0.7059	484.2695	1450.794	0.27	0.00013	29.48372
P19652	0	1	412.241	1234.708	2.38	0.00098	32.31036
P02763; P1	1	0.619	484.2691	1450.793	-0.55	-0.00027	12.07269
P19652	0	0.9722	574.9558	1722.853	1.65	0.00095	0
P02763; P1	1	0.6667	484.2699	1450.795	1.02	0.0005	28.49728
P19652	0	1	460.2734	1378.805	-1.51	-0.00069	8.542577
P02763; P1	1	0.7	484.269	1450.792	-0.87	-0.00042	17.58412
P19652	0	0.9333	853.9325	1706.858	1.63	0.00139	33.86631
P02763; P1	0	1	652.8502	1304.693	1.71	0.00112	4.641958
P02763; P1	1	0.8824	484.2694	1450.794	0.14	0.00007	47.73605
P19652	0	1	574.9562	1722.854	2.5	0.00144	5.554844
P02763; P1	1	0.5	436.2366	1306.695	2.87	0.00125	16.76086
P02763; P1	0	1	580.7996	1160.592	2.78	0.00161	13.73507
P19652	0	1	689.9075	1378.808	0.16	0.00011	0
P19652	0	0.8333	478.2396	1432.704	1.45	0.00069	5.876993
P19652	0	0.9643	689.9085	1378.81	1.49	0.00103	0
P02763; P1	1	0.5	436.2366	1306.695	2.87	0.00125	16.76086
P01834	0	1	722.0504	2164.137	2.52	0.00182	9.780593
P01834	0	0.9	541.7892	2164.135	1.78	0.00096	8.307413
P01834	0	1	745.4172	2234.237	2.64	0.00196	5.107466
P01834	0	1	745.4171	2234.237	2.56	0.0019	5.193758
P01834	0	1	745.416	2234.233	1.08	0.0008	5.242318
P01834	0	1	745.4169	2234.236	2.23	0.00166	3.98656
P01834	0	0.9412	597.6598	1790.965	1.25	0.00075	0
P01834	0	0.8649	597.6612	1790.969	3.61	0.00215	5.476667
P01834	0	0.8462	697.3833	2090.135	3.03	0.00211	23.38654
P01834	0	1	648.0062	1942.004	3.52	0.00228	24.74631
P01834	0	0.9667	971.5058	1942.004	3.62	0.00351	16.38128
P01834	0	1	648.0059	1942.003	3.05	0.00197	61.85017
P01834	0	0.8953	808.7309	2424.178	2.18	0.00176	6.063765
P01834	0	0.99	808.7311	2424.179	2.48	0.00201	8.000682
P01834	0	0.973	808.7309	2424.178	2.18	0.00176	21.56556
P01834	0	0.8235	697.3823	2090.132	1.54	0.00107	0
P01834	0	0.8889	541.7896	2164.137	2.57	0.00139	25.89116
P01834	0	0.9688	722.0516	2164.14	4.3	0.0031	22.86751
P01834	0	1	606.801	2424.182	3.89	0.00236	19.96503
P01834	0	0.8696	597.661	1790.968	3.2	0.00191	35.47383
P01834	0	0.4211	597.6605	1790.967	2.48	0.00148	23.48841
P01834	0	0.7273	597.661	1790.968	3.3	0.00197	7.928895
P01834	0	0.9048	808.7326	2424.183	4.29	0.00347	5.926784
P01834	0	0.5294	697.3825	2090.133	1.8	0.00126	17.84052
P01876	0	0.8986	610.3815	1219.756	4.74	0.00289	9.082395
P01876	0	0.9062	538.3286	1075.65	1.87	0.00101	32.21341
P01876; P0	0	1	660.6904	1980.057	2.38	0.00157	2.719908
P01876; P0	0	1	660.6906	1980.057	2.75	0.00182	18.97561
P01876; P0	1	1	693.1183	2769.451	3.08	0.00213	12.61343
P01876	0	0.9326	610.3173	1828.937	2.99	0.00182	7.940055
P01876; P0	0	0.8421	453.2509	1357.738	2.75	0.00124	17.89252
P01876	0	1	961.1179	2881.339	2.8	0.00269	8.074305
P01876	0	0.7727	864.9357	3456.721	0.39	0.00033	9.644367
P01876	0	0.8333	864.9375	3456.728	2.51	0.00217	0

P01876; P0	0	0.75	542.798	1084.589	1.9	0.00103	16.38632
P01876	0	1	864.9373	3456.727	2.29	0.00198	0
P01876; P0	0	0.7941	542.7979	1084.588	1.56	0.00085	0
P01876; P0	1	0.95	693.1158	2769.442	-0.45	-0.00031	35.64758
P01876	0	0.8621	407.2557	1219.753	2.11	0.00086	37.25089
P01876	0	0.88	555.2813	1663.829	3.25	0.0018	9.320871
P01876	0	1	914.9714	1828.936	2	0.00183	8.756765
P01876; P0	0	0.8605	559.8174	1118.627	3.99	0.00223	40.71346
P35443; P4	0	0.975	675.0043	2022.998	3.06	0.00206	4.581845
P35443; P4	0	1	461.7607	1844.021	0.61	0.00028	43.29137
P49747	0	1	704.635	2111.89	2.6	0.00183	3.150305
P49747	0	0.987	648.6239	1943.857	2.09	0.00135	6.503608
P49747	0	0.9556	648.6247	1943.86	3.31	0.00214	4.213584
P49747	0	0.9091	770.3763	1539.745	2.42	0.00187	4.921598
P49747	0	0.7679	513.9195	1539.744	1.46	0.00075	10.19977
P49747	0	0.9344	513.9192	1539.743	0.87	0.00045	0
P35443; P4	0	0.8095	657.4075	1313.808	3.48	0.00228	6.450108
P49747	0	0.9231	588.3127	1175.618	3.86	0.00227	41.96267
P49747	0	1	704.6329	2111.884	-0.35	-0.00025	5.407792
P49747	0	0.8462	586.6285	1757.871	1.33	0.00078	28.75407
P02747	0	0.9	739.3748	2216.11	1.07	0.00079	11.37481
P02747	0	1	739.3756	2216.112	2.23	0.00165	22.72268
P02747	0	1	712.3843	1423.761	0.58	0.00041	7.910392
P02747	0	0.8431	475.2597	1423.765	2.86	0.00136	16.58792
P02747	0	0.9048	467.9371	1401.797	2.59	0.00121	28.81486
P02747	0	1	739.3759	2216.113	2.56	0.00189	9.761957
P02747	0	0.9667	554.7833	2216.111	1.76	0.00097	5.64569
P02747	0	1	486.9323	1458.782	0.88	0.00043	4.525704
P02747	0	1	534.967	1602.887	2.07	0.00111	0
P02747	0	0.8718	614.8451	1228.683	1.85	0.00114	17.26646
P02747	0	1	739.3761	2216.114	2.89	0.00214	7.130405
P02747	0	1	486.9307	1458.778	-2.44	-0.00119	57.93151
P02747	0	0.825	410.2329	1228.684	2.74	0.00112	0
P02747	0	1	691.3405	2072.007	0.83	0.00057	0
P02747	0	0.6364	614.8461	1228.685	3.54	0.00218	9.785963
Q96IY4	0	0.9574	504.933	1512.784	1.87	0.00095	2.262344
Q96IY4	0	1	756.8958	1512.784	1.9	0.00144	3.976117
Q96IY4	0	0.8125	435.9191	1305.743	1.72	0.00075	41.30577
Q96IY4	0	1	642.6412	1925.909	2.44	0.00157	24.68243
Q96IY4	0	1	523.9413	1569.809	1.06	0.00055	48.40847
Q96IY4	0	0.9	523.9415	1569.81	1.52	0.0008	5.358249
Q96IY4	1	0.9773	686.7086	2058.111	2.03	0.00139	22.70815
Q96IY4	1	0.9661	515.2824	2058.108	0.42	0.00022	8.380038
Q96IY4	1	1	515.2806	2058.101	-3.02	-0.00155	4.180572
Q96IY4	0	0.6429	504.9323	1512.782	0.54	0.00027	21.02876
Q96IY4	0	0.5	653.3752	1305.743	1.99	0.0013	28.11555
Q9NZP8	0	1	820.3797	2459.125	1.85	0.00151	0
Q9NZP8; P0	0	0.6491	480.274	959.5408	1.78	0.00085	8.877599
Q9NZP8	0	0.9231	705.9165	1410.826	1.58	0.00112	35.6143
Q9NZP8	0	1	568.0043	1701.998	3.08	0.00175	14.89096
Q9NZP8	0	0.9434	568.0041	1701.998	2.76	0.00156	11.80002
Q9NZP8	0	1	469.9152	1407.731	1.17	0.00055	17.19986

Q9NZP8	0	0.7812	376.8818	1128.631	2.44	0.00092	29.82521
Q9NZP8	0	0.9143	814.9291	1628.851	1.64	0.00133	11.67713
Q9NZP8	0	0.8605	564.8198	1128.632	3.69	0.00208	0
Q9NZP8	0	1	568.0045	1701.999	3.51	0.00199	9.614187
Q9NZP8	0	0.875	568.0026	1701.993	0.07	0.00004	20.91151
P05090	0	0.8571	856.4788	1711.95	1.98	0.00169	4.151644
P05090	0	0.9796	571.3221	1711.952	2.93	0.00167	9.000759
P05090	0	0.75	856.479	1711.951	2.26	0.00194	6.08145
P05090	0	0.8305	571.3222	1711.952	3.04	0.00173	15.43143
P05090	0	0.9111	571.3218	1711.951	2.4	0.00137	13.45836
P05090	0	0.9655	571.3221	1711.952	2.82	0.00161	10.15095
P05090	0	0.9661	649.3293	1945.973	2.72	0.00176	13.71424
P05090	0	0.8627	571.3221	1711.952	2.93	0.00167	13.82257
P05090	0	0.7857	506.9635	1518.876	1.96	0.00099	11.07617
P05090	0	1	571.3224	1711.953	3.36	0.00192	29.79873
P05090	0	0.9104	571.3239	1711.957	5.93	0.00338	24.95804
P05090	0	1	532.9379	1596.799	1.67	0.00089	0
P05090	0	0.8333	649.328	1945.969	0.74	0.00048	10.49364
P05090	0	0.5789	759.9419	1518.877	2.33	0.00177	7.085978
P05090	0	0.9667	571.3229	1711.954	4.32	0.00247	54.0492
P05090	1	0.9048	618.3394	1853.004	2.81	0.00174	10.93978
P05090	0	0.6333	856.4785	1711.95	1.62	0.00139	6.436097
P05090	0	0.4091	506.9633	1518.875	1.6	0.00081	26.18867
P05090	0	0.9655	856.4805	1711.954	3.97	0.0034	6.581279
P00746	0	1	520.3023	2078.187	1.13	0.00059	22.17647
P00746	0	0.974	770.3915	2309.16	1.02	0.00079	3.706459
P00746	0	1	476.2823	1902.107	1.61	0.00076	2.769631
P00746	0	0.8438	634.708	1902.109	2.8	0.00178	10.37494
P00746	0	0.9412	634.7071	1902.107	1.36	0.00086	58.39006
P00746	0	0.8636	743.1526	2969.589	2.13	0.00158	4.847384
P00746	0	1	693.4019	2078.191	3.08	0.00213	0
P00746	1	1	559.3275	2234.288	0.93	0.00052	15.41343
Q2TV78; P2	0	1	536.0283	2141.091	-0.32	-0.00017	41.41212
Q2TV78; P2	0	0.92	429.0256	2141.099	3.35	0.00143	21.05733
Q2TV78; P2	0	1	536.0294	2141.096	1.73	0.00092	41.93961
Q2TV78; P2	0	1	832.9111	3328.623	2.7	0.00225	30.29306
Q2TV78; P2	0	0.8571	580.8331	1160.659	3.83	0.00222	2.265286
Q2TV78; P2	0	0.8125	580.8327	1160.658	3.2	0.00186	2.265191
Q2TV78; P2	0	0.8148	506.6295	1517.874	1.42	0.00072	7.35443
Q2TV78; P2	0	1	822.382	2465.131	1.67	0.00137	3.663897
P11226	0	0.8636	620.3715	1239.736	1.46	0.00091	11.21213
P11226	0	0.9362	770.4123	1539.817	1.86	0.00143	1.798474
P11226	0	1	718.3438	1435.68	1.46	0.00105	11.42586
P11226	0	0.8205	740.8806	1480.754	1.76	0.0013	6.814324
P11226	0	0.875	494.2559	1480.753	1.33	0.00066	39.88413
P11226	0	0.7188	513.9434	1539.816	0.7	0.00036	7.559332
P11226	0	0.6452	479.2324	1435.683	3.13	0.0015	10.88133
P11226	0	1	521.3205	1041.634	4.06	0.00211	29.28679
P00915	0	0.925	591.0864	2361.324	4.26	0.00252	17.68858
P00915	0	0.9804	737.9497	1474.892	1.09	0.0008	43.50139
P00915	0	1	670.3009	1339.595	1.69	0.00113	2.367105
P00915	0	0.6923	629.9033	1258.799	1.56	0.00098	18.99691

P00915	0	0.7347	557.8524	1114.697	1.99	0.00111	35.94655
P00915	0	0.9836	670.3013	1339.595	2.32	0.00156	0
P00915	0	0.7714	492.3029	1474.894	2.42	0.00119	33.31845
P00915	0	0.9	575.6372	1724.897	2.05	0.00118	7.584534
Q9NQ79	0	0.98	845.4349	1689.863	3.65	0.00309	19.54707
Q9NQ79	0	0.9231	489.2755	1465.812	3.1	0.00152	0
Q9NQ79	0	1	811.0687	2431.191	2.34	0.00189	3.476779
Q9NQ79	0	0.7429	511.3004	1021.594	2.41	0.00123	23.89659
Q9NQ79	0	0.4138	603.3059	1807.903	4.4	0.00265	42.04036
Q9UGM5	0	0.75	546.627	1637.866	2.46	0.00134	10.6876
Q9UGM5	0	1	452.9001	1356.686	0.54	0.00024	11.5939
Q9UGM5	0	0.987	787.7648	2361.28	2.82	0.00222	5.240521
Q9UGM5	0	0.9167	678.8474	1356.688	1.83	0.00124	4.205239
Q9UGM5	0	0.9	555.9584	1665.861	3.42	0.0019	21.6245
Q9UGM5	0	1	569.7858	1138.564	2.94	0.00167	2.995152
Q9UGM5	0	0.8947	555.9575	1665.858	1.77	0.00098	17.51114
Q9UGM5	0	0.88	678.8482	1356.689	3	0.00203	23.19679
P22352	0	1	748.4141	2243.228	2.73	0.00204	5.704846
P22352	0	0.7231	729.9172	1458.827	0.88	0.00064	4.831704
P22352	0	1	615.0082	1843.01	2.65	0.00163	44.78246
P22352	0	0.5818	518.8162	1036.625	0.94	0.00049	19.93784
P22352	0	0.6296	486.9477	1458.829	1.85	0.0009	17.57738
P22352	0	0.9091	748.4114	2243.22	-0.87	-0.00065	7.934869
P22352	0	1	586.8012	1172.595	2.22	0.0013	41.96244
P55058	0	0.8462	452.2451	1354.721	2.01	0.00091	17.81859
P55058	0	0.9524	981.0424	1961.078	3.26	0.0032	4.468916
P55058	0	1	491.2564	1471.755	2.57	0.00126	4.221241
P55058	0	0.8065	591.3274	1181.648	3.38	0.00199	19.46548
P55058	0	0.8824	648.6837	1944.036	6.49	0.00421	18.43232
P22891	0	1	425.241	1273.708	1.21	0.00051	6.278729
P22891	1	0.9677	618.689	1854.053	2.67	0.00165	44.81833
P22891	0	0.7551	452.2589	1354.762	1.51	0.00068	10.16879
P22891	0	0.8108	437.591	1310.759	2.09	0.00091	15.46497
P22891	0	0.7222	655.8825	1310.758	1.47	0.00096	43.32256
P22891	0	1	626.6416	1877.91	-0.85	-0.00053	63.89486
P13591	0	0.875	602.3257	1804.963	3	0.0018	7.197617
P13591	0	0.7209	490.9449	1470.82	3.22	0.00158	72.08906
P13591	0	0.8627	499.6142	1496.828	5.46	0.00272	76.78727
P13591	0	0.7368	477.2618	1429.771	2.17	0.00103	22.81896
P13591	0	0.7105	518.3365	1035.666	0.47	0.00024	26.23435
P13591	0	1	748.9139	1496.821	0.49	0.00037	40.27512
Q9UHG3	0	0.5278	620.002	1857.991	2.31	0.00143	4.584499
Q9UHG3	0	0.8293	488.6217	1463.851	0.86	0.00042	27.06753
Q9UHG3	0	0.7419	753.9285	1506.85	3.42	0.00258	26.75832
Q9UHG3	0	0.6667	452.2666	1354.785	2.31	0.00104	18.81942
Q9UHG3	0	0.5517	411.9274	1233.768	1.69	0.0007	4.908089
Q9UHG3	0	0.6207	617.389	1233.771	4.17	0.00258	19.64588
Q15582	0	0.6857	491.2768	1471.816	1.34	0.00066	37.92909
Q15582	0	1	441.8982	1323.68	2.1	0.00093	10.06358
Q15582	1	1	464.9276	1392.768	1.02	0.00047	34.60479
Q15582	0	0.6061	456.614	1367.827	1.76	0.0008	10.51156
Q15582	0	0.3036	553.3403	1105.673	2.33	0.00129	9.373934

Q15582	0	1	441.8976	1323.678	0.79	0.00035	61.07894
Q15582	0	1	441.8986	1323.681	2.86	0.00126	0
P63267; P6	0	0.7826	483.9488	1449.832	6.36	0.00307	34.84767
P63267; P6	0	1	487.2637	1459.777	0.94	0.00046	46.31744
A5A3E0; Q9	0	1	554.2729	1660.804	-0.37	-0.0002	38.75434
A5A3E0; P6	0	0.8636	645.6713	1934.999	2.72	0.00175	55.86254
A5A3E0; Q9	0	1	505.9223	1515.752	2.11	0.00107	29.73994
P63267; P6	0	0.7826	483.9488	1449.832	6.36	0.00307	34.84767
P63267; P6	0	1	487.2637	1459.777	0.94	0.00046	46.31744
A5A3E0; Q9	0	1	554.2729	1660.804	-0.37	-0.0002	38.75434
A5A3E0; P6	0	0.8636	645.6713	1934.999	2.72	0.00175	55.86254
A5A3E0; Q9	0	1	505.9223	1515.752	2.11	0.00107	29.73994
Q92820	0	1	525.0145	1573.029	3.4	0.00178	31.22417
Q92820	1	1	639.6994	1917.084	1.61	0.00103	0
Q92820	0	0.9412	541.9752	1623.911	3.24	0.00175	26.37759
Q92820	0	0.8649	455.9276	1365.768	1.79	0.00082	42.14945
Q92820	0	0.5312	608.3486	1215.69	3	0.00183	22.03184
P02042; P6	0	1	424.2276	1270.668	1.66	0.0007	6.509261
P02042; P6	0	0.9136	479.9645	1437.879	0.68	0.00033	15.47896
P02042; P6	0	0.883	719.4435	1437.88	1.26	0.00091	7.142306
P02042; P6	0	0.825	479.9647	1437.88	1.06	0.00051	34.55009
P68871	0	0.9508	610.8676	1220.728	3.14	0.00192	14.74708
P68871	0	0.9821	407.5798	1220.725	0.6	0.00025	13.17877
P68871	0	0.973	610.8671	1220.727	2.34	0.00143	8.040556
P68871	0	0.9747	783.0566	2347.155	1.42	0.00111	5.412879
P02042; P6	0	0.9794	647.3928	1293.778	1.79	0.00116	26.37509
P02042; P6	0	0.5	414.2438	1240.717	2.42	0.001	30.8405
P02100; P0	0	0.9348	709.9196	1418.832	2.96	0.0021	14.76605
P02100; P0	0	0.9286	709.9183	1418.829	1.16	0.00082	31.45067
P02042; P6	0	0.9892	653.3716	1958.1	2.79	0.00182	13.43204
P68871	0	1	556.3072	1666.907	1.65	0.00092	5.811198
P68871	0	1	556.3079	1666.909	2.86	0.00159	12.83933
P68871	0	0.9851	589.6398	1766.905	3.63	0.00214	12.96153
P68871	0	1	589.639	1766.902	2.18	0.00128	17.68098
P02042; P6	0	1	424.2276	1270.668	1.8	0.00076	0
P68871	0	1	517.0553	2065.199	0.52	0.00027	9.718471
P68871	0	1	657.8375	1314.668	2.15	0.00142	18.45968
P68871	0	0.7778	729.8886	1458.77	2.02	0.00147	54.17877
P02042; P6	0	1	635.8383	1270.669	2.51	0.0016	11.28336
P68871	0	0.8421	689.0705	2065.197	-0.65	-0.00045	44.00559
P68871	0	0.9333	833.9583	1666.909	2.98	0.00248	20.78093
P02042; P6	0	1	431.931	1293.778	1.94	0.00084	37.24517
P02100; P0	0	1	637.8677	1274.728	1.97	0.00125	41.66899
P68871	0	0.913	556.3105	1666.917	7.58	0.00421	0
P68871	0	1	833.9558	1666.904	0.05	0.00004	33.34534
P02656	0	0.8444	620.9914	1860.96	3.47	0.00215	8.620186
P02656	1	0.9839	769.0842	2305.238	1.36	0.00105	3.286407
P02656	0	0.875	495.605	1484.8	1.11	0.00055	7.288025
P02656	0	0.9545	742.9041	1484.801	1.49	0.00111	9.683728
P02656	0	0.9828	742.9031	1484.799	0.17	0.00013	25.90226
P02656	0	0.907	495.6053	1484.801	1.73	0.00085	44.54293
P02656	0	0.9535	495.6046	1484.799	0.37	0.00018	7.707762

P02656	0	1	742.9025	1484.798	-0.73	-0.00054	5.829767
P02656	0	0.9362	495.6057	1484.802	2.53	0.00125	5.983118
P02656	0	1	742.9045	1484.802	1.98	0.00147	10.57703
P02656	0	1	761.0477	2281.129	1.28	0.00097	2.425762
P02656	0	0.8816	620.9913	1860.959	3.27	0.00203	20.52575
P02656	0	1	572.9587	1716.861	6.04	0.00346	50.35393
P02656	0	0.8598	930.9808	1860.954	0.59	0.00055	3.548863
P02656	0	0.8929	620.9894	1860.954	0.32	0.0002	4.585898
P02656	0	0.902	620.9909	1860.958	2.68	0.00166	36.51932
P02656	0	0.8148	620.9908	1860.958	2.58	0.0016	11.34842
P02656	0	0.6481	593.8217	1186.636	1.28	0.00076	15.3524
P02656	0	0.8718	495.6051	1484.801	1.36	0.00067	8.729263
P02656	0	0.8857	495.6046	1484.799	0.25	0.00012	7.777669
P02656	0	0.9818	670.8539	1340.7	2.84	0.0019	21.86194
P02656	1	1	890.1232	2668.355	2.04	0.00181	3.022247
P02656	0	1	495.6038	1484.797	-1.23	-0.00061	24.54805
P02656	0	0.619	593.8224	1186.638	2.41	0.00143	100
P02656	0	0.6522	620.9913	1860.959	3.37	0.00209	34.19717
P02656	0	0.4412	593.8218	1186.636	1.38	0.00082	4.131884
P02656	0	0.6923	593.8228	1186.638	3.03	0.0018	23.01978
P02656	0	0.6512	593.8217	1186.636	1.28	0.00076	5.762704
P02656	0	0.6071	593.822	1186.637	1.8	0.00107	32.81427
P02656	0	0.7895	620.9908	1860.958	2.48	0.00154	4.094033
P02656	0	1	858.9305	1716.854	1.5	0.00128	17.87755
P05452	0	0.9302	534.662	1601.971	-0.13	-0.00007	23.18858
P05452	0	0.9831	801.4912	1601.975	2.2	0.00176	33.21868
P05452	0	0.9821	534.6631	1601.975	1.93	0.00103	11.3616
P05452	0	0.9176	801.4913	1601.975	2.28	0.00182	7.669374
P05452	0	1	484.5462	1451.624	1.5	0.00073	15.35178
P05452	0	0.9844	611.9811	1833.929	3.69	0.00225	9.492293
P05452	0	0.931	917.466	1833.925	1.43	0.00131	4.670127
P05452	0	1	532.5801	1595.726	1.15	0.00061	12.67296
P05452	0	1	532.5805	1595.727	1.84	0.00098	16.49493
P05452	0	1	484.5461	1451.624	1.19	0.00057	2.882085
P05452	0	0.6071	535.9661	1605.884	-2.13	-0.00114	41.79853
P05452	0	1	726.3142	1451.621	-0.65	-0.00047	0
P05452	0	1	484.5463	1451.624	1.63	0.00079	4.569598
P05452	0	1	726.3152	1451.623	0.78	0.00056	0
P05452	1		546.337	1091.667	0.48	0.00026	6.690168
P05452	1	0.027	546.337	1091.667	0.48	0.00026	6.690168
P05452	0	1	484.5465	1451.625	2.01	0.00097	5.928984
P05452	1	0.7586	412.5949	1235.77	1.49	0.00061	32.89539
P05452	0	1	726.3167	1451.626	2.79	0.00203	0
P05452	0	0.7143	468.5916	1403.76	0.61	0.00029	0
P05452	1	0.6667	618.3879	1235.769	0.22	0.00013	5.200743
Q6UXB8	0	0.625	489.9291	1467.773	0.33	0.00016	38.85015
Q6UXB8	0	0.9024	489.9297	1467.774	1.52	0.00074	16.42676
Q6UXB8	0	1	968.5092	2903.513	2.35	0.00228	7.187781
Q6UXB8	0	1	658.8442	1316.681	3.05	0.00201	0
Q6UXB8	0	0.9655	439.565	1316.68	2.48	0.00109	13.92809
Q6UXB8	0	1	494.9077	1482.709	0.9	0.00044	0
Q6UXB8	0	0.95	477.9359	1431.793	2.19	0.00105	5.432574

Q6UXB8	0	1	563.2853	1687.841	2.17	0.00122	35.32702
Q6UXB8	0	0.9535	734.3914	1467.775	2.21	0.00162	10.76006
Q6UXB8	0	0.75	741.8581	1482.709	1.18	0.00087	0
O00391	1	0.75	828.7732	2484.305	2.15	0.00178	71.58312
O00391	0	1	731.0637	2191.176	1.71	0.00125	7.351919
O00391	0	0.5172	548.9667	1644.885	1.18	0.00065	26.38286
O00391	0	1	473.879	1419.623	-0.01	0	0
O00391	0	0.8182	518.3452	1035.683	2.48	0.00128	48.73775
O00391	0	1	476.6089	1427.812	1.66	0.00079	31.30605
O00391	0	1	1096.092	2191.177	2.11	0.00231	0
P05154	0	0.9811	582.3491	1745.033	5.32	0.0031	37.22974
P05154	0	1	568.3054	1702.902	5.65	0.00321	18.008
P05154	0	0.9559	653.3438	1305.68	2.81	0.00184	5.178348
P05154	0	0.9455	653.3428	1305.678	1.32	0.00086	8.775236
P05154	0	0.8837	653.3439	1305.681	3	0.00196	20.51149
P05154	0	0.9565	499.2495	1495.734	2.69	0.00134	18.63385
P05154	0	0.2222	530.2871	1588.847	0.32	0.00017	5.666491
P05154	0	0.625	646.3984	1291.789	2.39	0.00155	33.52577
P05154	0	0.2273	530.2882	1588.85	2.51	0.00133	4.106297
P05154	0	0.9615	499.2495	1495.734	2.76	0.00137	21.80467
P12111	0	0.931	778.4375	2333.298	2.54	0.00198	10.05069
P12111	0	0.9318	658.7308	1974.178	2.76	0.00182	9.931641
P12111	0	1	625.7172	1875.137	3.39	0.00212	52.2477
P12111	0	0.9688	573.9572	1719.857	2.56	0.00147	2.323016
P12111	0	1	496.2538	991.5004	1.43	0.00071	4.987773
P12111	1	1	379.4613	1514.823	1.88	0.00071	6.582224
P19320	0	0.8833	646.0363	1936.094	3.15	0.00204	6.83571
P19320	0	0.8409	677.3252	1353.643	2.09	0.00141	7.749067
P19320; P4	0	0.54	566.3649	1131.722	-0.23	-0.00013	8.492911
P19320	0	0.2778	573.3743	1145.741	2.63	0.0015	42.9141
P19320	0	0.7917	554.6185	1661.841	-2.21	-0.00122	0
P19320	0	1	552.6235	1655.856	0.94	0.00052	26.31883
P08571	0	1	706.375	2117.11	5.17	0.00365	11.24365
P08571	0	0.4545	624.8698	1248.732	2.64	0.00165	50.80756
P08571	0	0.7561	693.3868	1385.766	1.97	0.00136	21.41239
P08571	0	0.6087	462.594	1385.768	2.86	0.00132	75.718
P08571	0	0.1429	761.4362	2282.294	1.27	0.00097	11.28704
P08571	0	0.8	706.3733	2117.105	2.74	0.00194	6.2173
P08571	0	0.3158	556.8143	1112.621	2.05	0.00114	7.441019
P08571	0	1	715.3741	2144.108	1.78	0.00127	0
Q9UK55	0	1	547.7511	2734.726	2.59	0.00142	17.53612
Q9UK55	0	0.75	440.9165	1320.735	0.93	0.00041	12.63491
Q9UK55	0	1	412.2183	1234.64	2.57	0.00106	31.22696
Q9UK55	0	0.9756	747.4103	1493.813	2.97	0.00222	39.34453
Q9UK55	0	0.9706	747.4087	1493.81	0.76	0.00057	30.36753
Q9UK55	0	0.9583	574.9315	1722.78	3.6	0.00207	19.80302
Q9UK55	0	0.8261	688.39	2063.155	1.23	0.00085	39.98726
P07360	0	1	734.0147	2200.029	2.01	0.00148	2.233289
P07360	0	1	734.0137	2200.027	0.76	0.00056	4.952258
P07360	0	1	734.0145	2200.029	1.76	0.00129	10.54229
P07360	0	0.8	528.3232	1582.955	2.08	0.0011	9.060866
P07360	1	1	521.6293	1562.873	1.11	0.00058	9.402434

P07360	0	1	768.4039	2303.197	1.58	0.00121	2.703876
P07360	0	1	768.3994	2303.184	-4.23	-0.00325	37.52563
P07360	0	1	751.7325	2253.183	1.64	0.00123	4.190782
P07360	0	0.907	588.9813	1764.929	2.59	0.00153	5.927134
P07360	1	1	521.6298	1562.875	2.05	0.00107	21.03655
P07360	0	0.65	751.7352	2253.191	5.14	0.00386	29.73279
P07360	0	1	396.4942	1582.955	2.03	0.0008	42.47427
P07360	1	1	521.6304	1562.877	3.22	0.00168	24.38052
P07360	1	0.8966	391.4742	1562.875	2.31	0.0009	25.83555
P07360	0	1	588.9813	1764.929	2.49	0.00146	2.219802
P07360	0	0.9474	579.8124	1158.617	3.33	0.00193	0
P07360	0	0.8824	576.5553	2303.199	2.58	0.00148	22.05381
P07360	0	0.9048	579.8129	1158.618	4.18	0.00242	0
P07360	0	0.9091	386.8765	1158.615	1.22	0.00047	19.45648
P07360	0	0.7273	564.0516	2253.185	2.37	0.00133	7.591939
P07360	0	0.9231	386.8769	1158.616	2.08	0.00081	34.41246
P07360	0	0.8947	576.556	2303.202	3.85	0.00222	25.98046
P69905	0	0.9844	558.6175	1673.838	0.94	0.00052	0
P69905	1	1	535.329	1603.972	-1.2	-0.00064	36.94637
P69905	0	0.9535	531.2802	2122.099	1.32	0.0007	7.071931
P69905	0	1	495.255	1977.998	2.22	0.0011	20.77533
P69905	0	1	558.6185	1673.841	2.8	0.00156	31.79922
P69905	0	1	558.6181	1673.84	2.03	0.00113	8.392786
P69905	0	0.3396	657.7462	3284.702	2.47	0.00162	2.783596
P69905	0	1	680.3834	1359.76	0.82	0.00056	38.48998
P69905	0	1	558.6185	1673.841	2.69	0.0015	30.51131
P69905; P0	0	0.7719	553.8264	1106.646	0.68	0.00037	10.68246
P69905; P0	0	0.7719	553.8285	1106.65	4.43	0.00245	9.041749
P69905	0	1	608.3333	1215.659	2.33	0.00142	22.10156
P69905	0	0.9259	459.256	1375.754	0.15	0.00007	20.88878
P69905	0	0.84	514.3119	1540.921	1.47	0.00075	10.35098
P69905	0	1	514.3123	1540.922	2.18	0.00112	13.44237
P69905; P0	0	0.8462	481.7759	962.5444	1.74	0.00084	34.6242
P69905	0	0.8571	531.281	2122.102	2.93	0.00155	10.92075
P02654	0	0.878	497.2578	1489.759	1.57	0.00078	14.81891
P02654	0	0.8596	745.3829	1489.759	1.38	0.00103	5.934212
P02654	0	0.9804	745.3817	1489.756	-0.26	-0.0002	6.240797
P02654	0	0.8356	673.3322	1345.657	1.9	0.00128	0
P02654	1	0.8718	479.3005	1435.887	1.71	0.00082	10.77534
P02654	0	0.8462	670.8518	1340.696	1.95	0.0013	2.743253
P02654	0	0.8438	670.8512	1340.695	1.13	0.00075	5.239112
P02654	0	0.873	670.8502	1340.693	-0.42	-0.00028	5.598434
P02654	0	0.8986	670.8497	1340.692	-1.06	-0.00071	14.50503
P02654	1	1	575.9982	1725.98	3.05	0.00175	9.467617
P02654	1	0.8947	575.9977	1725.979	2.3	0.00133	15.25998
P02654	1	0.1026	383.2322	1147.682	1.38	0.00053	8.709124
P02654	1	0.8077	718.447	1435.887	1.63	0.00117	10.87461
P02654	0	1	497.2574	1489.758	0.65	0.00032	10.89803
P02654	0	0.8333	745.3795	1489.752	-3.29	-0.00245	37.86101
P02654	0	0.6842	447.5701	1340.696	1.66	0.00074	12.05745
P02654	0	0.6	525.8065	1050.606	2.31	0.00121	17.77835
P02654	2	1	395.487	1578.926	1.48	0.00058	0

P02654	0	1	497.2575	1489.758	0.84	0.00041	7.796483
P02654	0	0.902	598.8007	1196.594	2.19	0.00131	5.507139
P02654	1	1	592.9722	1776.902	2.15	0.00127	30.33248
P02654	0	0.6047	525.8063	1050.605	1.96	0.00103	9.785151
P02654	0	0.8636	497.2568	1489.756	-0.39	-0.0002	3.015112
P02654	0	0.875	497.2577	1489.759	1.39	0.00069	25.30824
P02654	0	0.6875	670.8527	1340.698	3.31	0.00222	0
P02654	2	1	395.487	1578.926	1.48	0.00058	0
P02654	0	0.7027	525.8064	1050.606	2.08	0.00109	10.22051
P02654	0	1	673.3328	1345.658	2.89	0.00195	19.67318
P00742	0	0.8235	495.9291	1485.773	1.42	0.0007	8.302235
P00742	0	1	536.9429	1608.814	1.62	0.00087	12.10183
P00742	0	1	701.3602	1401.713	2.25	0.00158	4.826756
P00742	0	0.963	467.9092	1401.713	2.33	0.00109	5.968043
P00742	0	1	688.0002	2061.986	2.01	0.00138	0
P00742	0	0.9767	687.9982	2061.98	-1.01	-0.00069	9.272799
P00742	0	0.8846	589.8268	1178.646	1.91	0.00113	9.58318
P00742	0	1	743.39	1485.773	1.47	0.00109	29.34441
P00742	0	0.5581	633.3389	1265.67	1.93	0.00122	3.963283
P00742	0	0.7419	422.5619	1265.671	2.52	0.00106	7.715523
P00742	0	0.8966	519.2976	1037.588	1.86	0.00097	24.02707
P00742	0	0.8636	517.776	1034.545	2.55	0.00132	24.67109
P00742	0	0.8649	447.2467	893.4861	2.38	0.00106	25.03457
P00742	0	0.6774	633.3394	1265.671	2.7	0.00171	4.380492
P00742	0	1	536.942	1608.812	-0.08	-0.00004	29.5193
Q96KN2	0	1	799.0972	2395.277	2.6	0.00208	9.669096
Q96KN2	0	1	718.3729	2153.104	1.66	0.00119	36.91257
Q96KN2	0	0.9167	764.4538	1527.9	1.22	0.00093	19.74153
Q96KN2	0	0.8958	764.4543	1527.901	1.86	0.00142	17.95419
Q96KN2	0	0.9091	457.5845	1370.739	1.42	0.00065	3.076419
Q96KN2	0	0.8889	450.906	1350.703	3.24	0.00146	40.52431
Q96KN2	0	1	432.2628	1294.774	1.36	0.00059	11.68979
Q96KN2	0	0.697	509.9712	1527.899	0.31	0.00016	11.6621
Q96KN2	0	0.9643	599.5761	2395.283	4.97	0.00297	10.66611
Q96KN2	0	1	440.5853	1319.741	2.16	0.00095	25.28683
Q96KN2	0	0.8438	457.5837	1370.736	-0.52	-0.00024	0
P17936	0	1	656.3506	1967.037	2.49	0.00164	2.203538
P17936	0	1	756.9196	1512.832	1.19	0.0009	36.48552
P17936	1	1	437.8799	1311.625	1.59	0.00069	9.573146
P17936	0	0.9556	545.3314	1089.656	1.72	0.00094	3.958177
P17936	0	1	656.3513	1967.039	3.61	0.00237	3.022174
P17936	0	0.8485	443.2396	1327.704	0.87	0.00039	16.73485
P17936	0	1	656.3521	1967.042	4.82	0.00316	47.64048
P17936	0	0.9355	385.2296	1153.674	1.96	0.00076	21.46969
P17936	0	1	656.3502	1967.036	1.84	0.00121	9.594666
P17936	0	0.8	780.6988	2340.082	4.31	0.00336	31.58427
P17936	0	1	577.3422	1153.677	4.46	0.00257	0
O00533	0	0.9756	840.4498	2519.335	1.75	0.00147	3.379233
O00533	0	0.9143	421.975	1684.878	1.65	0.00069	26.46294
O00533	0	1	481.9526	1443.843	1.61	0.00078	25.40402
O00533	0	0.9231	504.9055	1512.702	1.99	0.001	23.19218
O00533	0	1	517.9416	1551.81	3.65	0.00189	22.47573

O00533	0	0.8571	581.3198	1741.945	0.71	0.00041	0
O00533	0	0.88	524.9433	1572.815	0.35	0.00019	0
P02652	1	0.963	530.3388	1589.002	1.19	0.00063	25.10849
P02652	1	0.9583	530.3388	1589.002	1.19	0.00063	10.96668
P02652	1	0.8	398.0058	1589.001	0.91	0.00036	9.180206
P02652	1	0.9615	530.3392	1589.003	2	0.00106	26.62607
P02652	0	1	832.0704	2494.197	1.5	0.00125	2.82802
P02652	0	1	1247.6	2494.194	0.33	0.00042	0
P02652	0	1	1319.653	2638.299	1.42	0.00188	4.611726
P02652	1	1	544.662	1631.971	1.34	0.00073	8.625198
P02652	1	1	544.6608	1631.968	-0.9	-0.00049	7.235825
P02652	1	1	701.131	2801.502	2.14	0.0015	6.646177
P02652	0	0.807	630.8569	1260.707	2.23	0.00141	17.98664
P02652	0	0.9014	630.8552	1260.703	-0.58	-0.00036	5.575297
P02652	0	0.8929	630.8552	1260.703	-0.58	-0.00036	5.708681
P02652	0	0.8462	630.8552	1260.703	-0.48	-0.0003	5.530602
P02652	0	0.6591	420.9059	1260.703	-0.54	-0.00023	8.431663
P02652	0	0.8824	630.857	1260.707	2.33	0.00147	8.421275
P02652	0	0.8889	630.8572	1260.707	2.62	0.00165	6.389449
P02652	0	0.8889	630.8569	1260.707	2.23	0.00141	8.782099
P02652	0	0.9074	630.8568	1260.706	1.94	0.00122	19.80364
P02652	0	1	843.7726	2529.303	1.6	0.00135	2.054648
P02652	0	1	880.104	2638.297	0.98	0.00086	10.27286
P02652	0	0.8833	558.8059	1116.604	2.42	0.00135	3.238775
P02652	1	0.1389	496.628	1487.87	1.58	0.00079	13.77181
P02652	0	0.9322	558.806	1116.605	2.64	0.00147	36.40206
P02652	0	0.8868	630.8578	1260.708	3.59	0.00226	5.058565
P02652	0	0.9333	630.8575	1260.708	3.2	0.00202	68.03008
P02652	0	0.7381	615.3912	1229.775	3.53	0.00217	6.764525
P02652	0	0.6316	420.9059	1260.703	-0.54	-0.00023	7.916655
P02652	0	0.6552	420.9074	1260.707	2.95	0.00124	15.40078
P02652	1	0.5263	530.3388	1589.002	1.19	0.00063	46.0147
P02652	1	1	530.3373	1588.997	-1.46	-0.00077	34.3315
P02652	0	0.6053	420.906	1260.703	-0.32	-0.00013	8.649993
P02652	1	0.7436	795.0046	1589.002	1.32	0.00104	7.867902
P02652	0	0.5161	410.596	1229.774	2.3	0.00094	0
P02652	0	0.5152	410.5952	1229.771	0.22	0.00009	0
P02652	0	1	880.0996	2638.284	-4.02	-0.00354	29.53219
P02652	1	0.675	482.3048	1444.9	1.36	0.00066	10.43697
P02652	1	1	530.338	1588.999	-0.19	-0.0001	26.90374
P02652	1	0.675	482.3048	1444.9	1.36	0.00066	10.43697
P02652	0	0.7059	420.9065	1260.705	0.99	0.00041	5.477408
P02652	0	0.84	615.3889	1229.77	-0.25	-0.00015	14.45453
P02652	0	0.7143	420.9067	1260.706	1.49	0.00063	22.12584
P02652	1	0.7727	482.3044	1444.899	0.54	0.00026	44.00708
P02652	0	0.6829	543.3396	1085.672	2.99	0.00163	3.728235
P02652	0	0.9333	1265.158	2529.308	3.49	0.00441	9.201798
P02652	2	0.8824	466.0549	1861.198	0.52	0.00024	3.748977
P02652	0	0.5806	410.5951	1229.771	0.07	0.00003	8.954934
P02652	0	0.6207	420.9074	1260.707	2.95	0.00124	27.44842
P02652	1	0.5294	530.34	1589.005	3.5	0.00185	36.13976
P02652	0	0.8571	615.3889	1229.771	-0.15	-0.00009	5.542968

P02652	1	1	408.7473	1631.967	-1.1	-0.00045	37.86441
P02652	0	0.6538	420.9069	1260.706	1.86	0.00078	6.528193
P02652	1	0.7647	530.3383	1589	0.38	0.0002	43.63034
P02652	1	0.3571	530.3395	1589.004	2.57	0.00136	45.25055
P02652	0	0.7333	630.8572	1260.707	2.72	0.00171	51.25354
P02652	1	0.25	530.3381	1589	0.04	0.00002	39.17081
P02652	0	0.5517	420.9069	1260.706	1.93	0.00081	11.04731
P02652	0	0.5862	615.3909	1229.775	3.13	0.00192	0
P02652	1	0.6667	496.6276	1487.868	0.72	0.00036	59.65875
P02652	1	0.6667	496.6276	1487.868	0.72	0.00036	59.65875
P02652	0	0.75	420.9067	1260.705	1.35	0.00057	31.79425
P02652	0	0.7429	543.3394	1085.672	2.66	0.00144	44.34234
P02652	0	0.8636	420.9064	1260.705	0.62	0.00026	9.623656
O75636	0	0.9167	584.7983	1168.589	2.18	0.00127	3.107245
O75636	0	1	629.3304	1257.653	3.04	0.00191	1.396681
O75636	0	1	419.8889	1257.652	1.94	0.00081	7.103546
O75636	0	1	615.3573	1844.057	2.9	0.00178	8.683725
O75636	0	0.8923	445.5835	1334.736	3.95	0.00176	15.93118
O75636	0	1	1177.911	3531.718	0.65	0.00076	0
O75636	0	1	883.6854	3531.72	1.04	0.00092	3.123639
O75636	0	1	445.5833	1334.735	3.4	0.00151	53.92889
O75636	0	1	814.7298	2442.175	1.88	0.00153	10.49986
O75636	0	0.94	607.801	1214.595	2.11	0.00128	6.662632
O75636	0	1	651.6976	1953.078	3.28	0.00214	6.688876
O75636	0	1	419.8888	1257.652	1.72	0.00072	20.14346
O75636	0	1	419.8892	1257.653	2.66	0.00112	29.38592
O75636	0	1	512.7476	1024.488	3.09	0.00159	20.37806
O75636	0	0.7059	461.7705	1844.06	4.52	0.00208	24.66369
O75636	0	1	651.6966	1953.075	1.78	0.00116	67.19032
O75636	0	0.8571	629.3301	1257.653	2.65	0.00167	0
P43251	0	0.9873	612.3843	1223.761	1.78	0.00109	0
P43251	0	0.92	456.6261	1367.864	1.73	0.00079	44.87801
P43251	0	0.8286	456.6255	1367.862	0.39	0.00018	12.41496
P43251	0	0.825	451.5606	1352.667	1.68	0.00076	19.22727
P43251	0	1	1032.862	3096.571	4.04	0.00417	0
P43251	0	0.9744	456.6276	1367.868	5.01	0.00229	21.11179
P43251	0	1	684.4368	1367.866	3.73	0.00255	9.300961
P43251	0	0.8136	735.3832	1469.759	2.98	0.00219	10.31168
P43251	0	0.96	564.2695	1690.794	-0.36	-0.0002	15.92303
P43251	0	0.9459	564.2702	1690.796	0.94	0.00053	46.81824
P43251	0	0.8684	518.6259	1553.863	2.08	0.00108	25.06315
P43251	0	0.8462	798.433	1595.859	2.41	0.00192	0
P43251	0	0.8444	408.5918	1223.761	1.4	0.00057	9.877079
P43251	0	0.6176	484.6171	1451.837	1.99	0.00097	12.88708
P43251	0	0.4286	798.4324	1595.858	1.64	0.00131	7.004591
P43251	0	0.8	777.436	1553.865	3.03	0.00236	13.22548
P04278	0	0.9875	709.6945	2127.069	2.5	0.00177	9.663723
P04278	0	1	709.6943	2127.068	2.16	0.00153	17.69253
P04278	0	0.8868	553.3453	1105.683	1.23	0.00068	5.537842
P04278	0	0.9231	793.4822	1585.957	1.63	0.00129	5.632803
P04278	0	1	492.2718	1474.801	1.85	0.00091	22.55259
P04278	0	1	887.5464	2660.625	1.57	0.00139	15.37864

P04278	0	1	520.0789	2596.365	1.61	0.00083	33.87479	
P04278	0	1	520.0792	2596.367	2.2	0.00114	22.98778	
P04278	0	1	405.2369	1213.696	2.34	0.00094	8.898788	
P04278	0	1	648.8319	1296.656	2.87	0.00186	25.54552	
P04278	0	0.8276	553.3464	1105.685	3.21	0.00178	38.45863	
O95445	1	1	502.5279	2007.09	-0.21	-0.00011	9.390475	
O95445	0	1	578.9704	1734.897	2.01	0.00117	25.36809	
O95445	0	1	578.9706	1734.897	2.33	0.00135	9.093753	
O95445	0	0.9773	578.9707	1734.898	2.54	0.00147	16.11567	
O95445	0	0.9773	867.9512	1734.895	1.18	0.00102	0	
O95445	0	0.8182	850.4544	2549.348	2.12	0.0018	0	
O95445	0	1	808.4101	3230.619	2.81	0.00227	6.492002	
O95445	0	0.9365	649.8476	1298.688	1.16	0.00075	5.899256	
O95445	0	0.9722	578.9708	1734.898	2.65	0.00153	73.89592	
O95445	0	0.7368	433.5678	1298.689	1.97	0.00085	13.88651	
O95445	0	1	661.7928	1322.578	1.5	0.001	12.91227	
O95445	0	1	850.4528	2549.344	0.32	0.00027	5.890569	
O95445	0	1	407.8889	1221.652	1.67	0.00068	10.65826	
O95445	0	0.8182	407.8887	1221.651	1.22	0.0005	9.773503	
O95445	0	1	661.7927	1322.578	1.32	0.00087	56.37947	
O95445	0	0.898	409.2511	817.4949	2.21	0.0009	7.144684	
O95445	0	0.8108	577.798	1154.589	3.85	0.00222	31.20136	
P23142	0	0.9643	565.3195	1693.944	2.74	0.00155	8.126082	
P23142	0	0.8438	440.5541	1319.648	1.17	0.00052	11.44509	
P23142	0	1	694.0154	2080.032	1.61	0.00112	9.129058	
P23142	0	1	737.6536	2210.946	1.88	0.00138	5.394526	
P23142	0	1	737.6533	2210.945	1.55	0.00114	25.79776	
P23142	0	1	440.5546	1319.649	2.42	0.00107	43.00533	
P23142	0	1	727.8196	1454.632	0.92	0.00067	6.089577	
P23142	0	1	872.6451	3487.558	0.17	0.00014	0	
P23142	0	1	460.5366	1379.595	2.32	0.00107	39.54801	
P23142	0	0.7419	632.3632	1263.719	3.45	0.00218	0	
P23142	0	1	737.653	2210.944	1.05	0.00077	76.07219	
P23142	0	0.7742	421.9101	1263.716	0.91	0.00038	19.28104	
P15169	0	0.7955	376.2289	1126.672	1.88	0.00071	28.3273	
P15169	0	0.86	615.3716	1229.736	2.12	0.00131	100	
P15169	0	0.9032	509.0213	2033.063	1.89	0.00096	6.820088	
P15169	0	1	526.5929	1577.764	1.93	0.00102	0	
P15169	0	0.9535	615.3716	1229.736	2.03	0.00125	10.49718	
P15169	0	0.9091	509.0206	2033.06	0.45	0.00023	64.02462	
P15169	0	0.8125	439.8955	1317.672	1.38	0.00061	22.91768	
P15169	0	0.6538	440.9022	1320.692	2.65	0.00117	37.38174	
P15169	0	0.8636	543.3208	1085.634	2.75	0.0015	0	
P15169	0	0.7647	545.2876	1089.568	2.07	0.00113	0	
P15169	0	0.9231	509.0215	2033.064	2.31	0.00117	8.721296	
P15169	0	0.8462	838.949	3352.774	0.79	0.00066	5.757579	
P15169	0	0.7955	563.8401	1126.673	2.51	0.00142	3.50319	
P15169	0	0.7143	678.3601	2033.066	2.97	0.00202	9.448347	
P48740	0	0.9787	677.3333	2029.985	2.03	0.00137	7.780985	
P48740	0	0.8868	498.7614	1992.024	1.3	0.00065	0	
P48740	0	1	664.68	1992.025	2.17	0.00144	53.43182	
P48740	0	0.88	498.7629	1992.03	4.25	0.00211	16.06669	

P48740	0	0.9062	515.2734	1543.806	6.68	0.00344	6.147194
P48740; P0	0	1	564.259	1127.511	1.99	0.00112	2.876967
P48740	0	0.8947	647.8574	1294.708	0.63	0.00041	20.98335
P48740	0	0.9444	756.3977	1511.788	3.3	0.00249	3.826502
P48740	0	0.9429	606.3044	1211.601	1.03	0.00062	0
P48740	0	0.6333	509.2458	1525.723	-0.53	-0.00027	49.05505
P48740	0	1	515.2687	1543.791	-2.57	-0.00132	45.6151
P48740	0	1	577.3238	1729.957	-0.85	-0.00049	0
P09172	0	0.7391	475.6256	1424.862	0.4	0.00019	10.34492
P09172	0	1	505.6318	1514.881	1.79	0.00091	52.01717
P09172	0	0.6923	441.9221	1323.752	0.42	0.00019	19.84711
P09172	0	0.9667	775.0815	2323.23	1.11	0.00086	0
P09172	0	1	715.3629	2144.074	-1.58	-0.00113	0
P09172	1	0.7407	401.2796	1201.824	0.75	0.0003	7.528913
P09172	0	1	757.9434	1514.879	0.92	0.00069	10.35315
P09172	0	1	602.9788	1806.922	1.83	0.0011	43.25351
P09172	0	0.9286	455.7541	1819.995	0.55	0.00025	16.27107
P00748	0	1	824.7258	2472.163	1.39	0.00115	7.865944
P00748	0	1	714.0139	2140.027	1.04	0.00074	27.26283
P00748	0	1	473.5684	1418.691	2.76	0.0013	58.95689
P00748	0	0.8571	560.8054	2240.2	2.32	0.0013	0
P00748	0	1	385.8657	1155.583	1.37	0.00053	26.3201
P00748	0	1	473.5675	1418.688	0.82	0.00039	31.95839
P00748	0	1	381.8621	1143.572	1.68	0.00064	30.3688
P00748	0	1	514.3414	1027.676	1.13	0.00058	22.56754
P00748	0	0.6944	536.3004	1071.594	1.93	0.00103	6.621604
P04220; P0	0	1	465.2515	1393.74	1.57	0.00073	32.03212
P04220; P0	0	0.9273	697.3748	1393.742	3.16	0.0022	16.76457
P04220; P0	0	1	640.0442	1918.118	1.93	0.00123	5.983711
P04220; P0	0	1	480.2844	1918.116	0.81	0.00039	4.934416
P04220; P0	0	1	480.2846	1918.117	1.19	0.00057	26.15992
P04220; P0	0	1	625.3226	1249.638	1.78	0.00111	5.815742
P04220; P0	0	0.8333	502.0225	2005.068	0.74	0.00037	33.76444
P04220; P0	0	0.9848	669.0283	2005.07	1.78	0.00119	7.875226
P04220; P0	0	1	963.5733	1926.139	2.63	0.00253	9.84608
P04220; P0	0	1	582.2993	1744.883	1.35	0.00078	2.909007
P04220; P0	0	0.5556	585.8173	1170.627	2.38	0.00139	7.087362
P04220; P0	0	1	575.8695	1150.732	2.78	0.0016	5.647379
P04220; P0	0	1	500.2838	999.5604	1.23	0.00061	0
P04220; P0	0	0.9333	381.8922	1143.662	0.87	0.00033	23.20232
P04220; P0	0	0.5424	585.8171	1170.627	2.17	0.00127	20.0987
P04220; P0	0	0.8333	642.7177	1926.138	2.15	0.00138	8.946026
P04220; P0	0	0.8947	450.7702	900.5332	3.36	0.00151	18.34709
P04220; P0	0	1	582.3002	1744.886	3.02	0.00176	0
P04220; P0	0	1	575.8695	1150.732	2.68	0.00154	7.036704
P04220; P0	0	0.9333	669.0283	2005.07	1.87	0.00125	0
P04220; P0	0	1	502.0216	2005.064	-1.09	-0.00055	0
P04220; P0	0	0.6286	522.8208	1044.634	2.01	0.00105	27.21322
P04220; P0	0	1	500.2843	999.5613	2.15	0.00107	0
P04220; P0	0	1	640.0452	1918.121	3.46	0.00221	0
P04220; P0	0	0.9474	669.031	2005.078	5.89	0.00394	70.9072
P04220; P0	0	1	572.3357	1143.664	2.56	0.00146	23.70536

P04220; P0	0	0.8214	522.8209	1044.634	2.13	0.00111	0
P04220; P0	0	0.9688	384.2483	1150.73	1.47	0.00056	5.071266
P04220; P0	0	1	575.8692	1150.731	2.25	0.0013	0
P04220; P0	0	0.84	522.8204	1044.634	1.31	0.00068	53.48961
P04220; P0	0	1	572.3355	1143.664	2.13	0.00122	0
P04220; P0	0	0.8276	522.8203	1044.633	0.96	0.0005	23.53466
P04220; P0	0	0.7273	522.8204	1044.633	1.19	0.00062	6.409844
P20851	0	0.7188	614.989	1842.952	2.19	0.00134	16.67333
P20851	0	0.987	776.6318	3103.505	-1.75	-0.00136	2.447053
P20851	0	0.9423	1035.175	3103.511	-0.04	-0.00004	20.21488
P20851	0	1	792.0577	2374.158	0.95	0.00075	4.226348
P20851	0	0.9459	633.0182	1897.04	3.01	0.00191	44.35191
P20851	0	0.9535	633.0176	1897.038	2.14	0.00136	45.97803
P20851	0	0.9333	469.2657	1874.041	-0.37	-0.00017	11.42643
P20851	0	1	625.3538	1874.047	2.67	0.00167	10.20266
P20851	0	1	469.2657	1874.041	-0.5	-0.00023	30.311
P20851	0	1	1022.498	2043.989	-0.89	-0.00091	5.887505
P20851	0	0.8929	432.259	1294.763	1.27	0.00055	62.45133
P20851	0	0.8889	647.8854	1294.763	1.99	0.00129	28.60997
P20851	0	0.2121	620.3192	1858.943	-0.15	-0.00009	70.20193
P20851	0	0.9677	481.5755	1442.712	0.72	0.00035	7.258524
P20851	0	0.8837	531.3225	1061.638	2.98	0.00158	25.02668
P20851	0	1	744.0244	2230.059	1.95	0.00145	71.98204
P20851	0	1	792.0571	2374.157	0.18	0.00014	19.49184
Q14520	0	1	684.6924	2052.063	2.02	0.00138	33.1315
Q14520	0	1	684.6928	2052.064	2.56	0.00175	8.957685
Q14520	0	1	684.692	2052.062	1.49	0.00102	0
Q14520	0	1	576.2972	1726.877	3.98	0.00229	5.675166
Q14520	0	1	863.9403	1726.873	1.69	0.00146	3.268286
Q14520	0	1	576.2971	1726.877	3.66	0.00211	1.388424
Q14520	0	0.98	781.3747	1561.742	2.55	0.00199	6.9965
Q14520	0	1	521.2516	1561.74	1.33	0.00069	10.59657
Q14520	0	0.7778	634.8325	1268.658	2.4	0.00152	13.49475
Q14520	0	0.8596	634.8318	1268.656	1.24	0.00079	0
Q14520	1	1	406.9644	1624.836	2.18	0.00089	9.939571
Q14520	0	0.9211	629.3574	1886.058	2.52	0.00158	16.3625
Q14520	0	0.9697	472.2695	1886.056	1.77	0.00083	8.510356
Q14520	0	0.7273	513.7708	2052.061	1.45	0.00074	11.7863
Q14520	0	0.9167	555.9868	1665.946	2.71	0.0015	18.55164
Q14520	0	0.2353	513.7712	2052.063	2.28	0.00117	3.205098
Q14520	0	0.9231	423.5559	1268.653	-1.36	-0.00058	46.4451
Q14520	0	1	576.2955	1726.872	0.9	0.00052	45.60072
Q14520	0	0.4286	425.5907	1274.758	1.43	0.00061	2.937158
Q14520	0	0.814	581.3066	1161.606	1.22	0.00071	43.83019
Q14520	0	1	863.9406	1726.874	2.04	0.00176	5.942664
Q14520	1	1	406.9649	1624.838	3.46	0.00141	16.73187
Q14520	1	1	542.2825	1624.833	0.43	0.00023	32.54614
Q14520	0	1	423.5577	1268.658	2.89	0.00122	0
Q14520	0	0.5455	513.7701	2052.058	0.02	0.00001	12.21202
P02743	2	0.9412	739.3879	2954.53	1.57	0.00116	5.390469
P02743	0	0.9787	517.5961	1550.774	1.21	0.00062	42.67443
P02743	0	0.9041	775.8911	1550.775	1.84	0.00143	3.376059

P02743	0	1	517.5963	1550.774	1.44	0.00075	6.281616
P02743	1	0.9143	522.9638	1566.877	2.49	0.0013	12.12665
P02743	0	1	561.3062	1681.904	2.48	0.00139	11.08428
P02743	0	0.9241	841.4553	1681.903	1.98	0.00167	5.796036
P02743	0	0.9444	561.3066	1681.905	3.25	0.00182	33.52917
P02743	0	0.96	561.3059	1681.903	1.94	0.00109	13.65944
P02743	0	0.8393	578.8052	1156.603	2.92	0.00169	0
P02743	0	0.8372	715.949	1430.891	1.14	0.00081	7.349054
P02743	0	1	517.5966	1550.775	2.03	0.00105	59.45896
P02743	0	0.8333	561.3058	1681.903	1.72	0.00096	47.29276
P02743	0	0.9286	641.3712	1281.735	4.5	0.00288	12.6777
P02743	0	1	788.0008	1574.994	1.95	0.00154	27.7945
P02743	1	0.9697	783.9428	1566.878	3.35	0.00263	8.331266
P02743	0	0.8367	655.8479	1310.689	1.84	0.00121	0
P02743	1	0.5417	594.3405	1781.007	6.73	0.004	32.63989
P02743	0	0.3333	485.6024	1454.793	2.98	0.00145	10.14863
P02743	1	1	522.9635	1566.876	1.9	0.00099	18.26338
P02743	0	0.8485	641.3717	1281.736	5.36	0.00343	17.90226
P02743	1	0.8696	522.9653	1566.881	5.41	0.00283	32.80954
P27918	0	1	433.474	1730.874	1.9	0.00082	36.13181
P27918	0	1	433.4744	1730.876	2.75	0.00119	54.19877
P27918	0	0.9846	883.1094	2647.314	1.42	0.00125	11.13248
P27918	0	1	775.0263	2323.064	2.88	0.00223	0
P27918	0	1	581.521	2323.062	1.93	0.00112	16.24206
P27918	0	0.8718	433.4749	1730.878	4.02	0.00174	6.189634
P27918	0	1	545.2755	1633.812	2.18	0.00119	5.385627
P27918	0	1	603.9831	1809.935	2.89	0.00174	4.65006
P27918	0	1	382.2148	1525.837	2.5	0.00095	45.63492
P27918	0	1	509.2835	1525.836	1.65	0.00084	40.95387
P27918	0	0.5957	615.3825	1229.758	3.87	0.00238	11.22651
P27918	0	1	382.2148	1525.837	2.5	0.00095	45.63492
P27918	0	1	640.3327	1279.658	2.1	0.00134	3.360073
P27918	0	1	591.8191	1182.631	1.47	0.00087	2.869444
P27918	0	1	382.2134	1525.832	-1.18	-0.00045	71.17059
P27918	0	0.7368	410.5899	1229.755	1.85	0.00076	9.470749
P27918	0	0.8333	394.8819	1182.631	1.58	0.00062	16.05436
P02753	0	1	946.413	2837.225	0.55	0.00052	3.469536
P02753	0	1	710.0607	2837.221	-0.65	-0.00046	24.56946
P02753	0	1	946.4134	2837.226	0.94	0.00089	1.219239
P02753	0	0.9667	483.2467	1447.726	1.6	0.00077	35.96778
P02753	0	1	967.4628	2900.374	1.89	0.00183	4.658443
P02753	1	1	893.9609	3572.822	1.82	0.00162	7.559333
P02753	1	1	715.3693	3572.817	0.5	0.00036	11.00039
P02753	1	1	1191.616	3572.833	5.02	0.00597	0
P02753	0	1	967.4618	2900.371	0.82	0.00079	4.314328
P02753	1	0.9859	715.3678	3572.81	-1.55	-0.00111	11.54692
P02753	0	1	743.9188	1486.83	0.49	0.00036	32.72534
P02753	0	1	743.9199	1486.832	1.88	0.0014	6.990964
P02753	0	1	485.2683	1453.79	0.89	0.00043	60.75327
P02753	0	0.9444	485.2686	1453.791	1.58	0.00077	19.33685
P02753	0	1	737.0294	2209.074	0.96	0.00071	2.841416
P02753	0	1	1105.041	2209.074	1	0.0011	0

P02753	1	1	893.9612	3572.823	2.16	0.00193	2.906531
P02753	0	1	435.2127	1303.623	1.76	0.00077	29.57515
P02753	0	0.9643	653.3267	1305.646	3.25	0.00212	28.69491
P02753	0	0.4545	483.9196	1449.744	0.23	0.00011	10.13191
P02753	0	0.6667	388.2057	1162.603	1.66	0.00064	0
P02753	1	0.7568	434.8917	1302.661	1.71	0.00074	8.719847
P02753	0	0.8788	483.9203	1449.746	1.62	0.00078	12.45729
P02753	0	1	725.3771	1449.747	2.07	0.0015	11.12729
P02753	0	0.8214	509.7535	1018.5	1.18	0.0006	31.83038
P02753	0	0.8056	581.8051	1162.603	1.96	0.00114	3.496979
P02753	0	0.7872	509.7541	1018.501	2.19	0.00112	14.96634
P02753	0	0.9545	485.2685	1453.791	1.33	0.00064	58.43333
P02753	0	0.9615	496.2818	1486.831	0.81	0.0004	39.45337
P02753	0	0.8788	496.282	1486.832	1.3	0.00064	9.379045
P02753	0	0.6154	581.8052	1162.603	2.17	0.00126	43.0453
P02753	0	0.75	727.4004	1453.794	3.16	0.0023	14.88584
P02753	1	0.6857	482.926	1446.763	2.06	0.00099	13.81189
P02753	0	0.4	483.9201	1449.746	1.24	0.0006	21.14532
P02753	0	0.6957	489.2511	1465.739	-0.09	-0.00004	25.64811
P02753	0	1	972.793	2916.365	0.4	0.00039	0
P02753	0	0.8889	435.8867	1305.646	2.84	0.00124	8.729925
P02753	0	1	725.3743	1449.741	-1.8	-0.0013	5.332016
P02753	0	0.4583	489.252	1465.741	1.85	0.0009	9.247739
P02753	1	0.9286	651.8336	1302.66	1.18	0.00077	0
P01019	0	1	721.413	1441.819	1.14	0.00082	29.1785
P01019	0	0.8974	863.4635	1725.92	2.8	0.00242	17.11893
P01019	0	0.8571	575.978	1725.919	2.58	0.00148	9.490314
P01019	0	1	793.465	1585.923	2.19	0.00173	6.497079
P01019	0	0.9348	529.3123	1585.922	1.91	0.00101	8.895675
P01019	0	1	519.6599	1556.965	2.32	0.0012	28.77569
P01019	0	0.9649	519.6596	1556.964	1.85	0.00096	13.92412
P01019	0	1	778.9854	1556.964	1.39	0.00108	42.24855
P01019	0	1	681.4034	2042.196	1.81	0.00123	1.379567
P01019	0	1	1021.602	2042.196	2.06	0.0021	1.05458
P01019	0	0.7857	791.4114	1581.816	1.75	0.00139	14.4685
P01019	0	0.9556	471.6346	1412.889	1.65	0.00078	8.489688
P01019	0	0.7	706.9487	1412.89	2.42	0.00171	13.88592
P01019	0	0.7674	452.585	1355.74	1.5	0.00068	8.483099
P01019	0	0.7391	678.3744	1355.742	2.3	0.00156	8.849647
P01019	0	0.8214	678.3715	1355.736	-2.03	-0.00137	0
P01019	0	0.7419	678.3732	1355.739	0.59	0.0004	13.21946
P01019	0	0.914	623.0476	1867.128	2	0.00124	4.398575
P01019	0	1	934.0671	1867.127	1.25	0.00117	3.467736
P01019	0	1	912.1073	4556.507	0.74	0.00067	8.234467
P01019	0	0.9836	821.4415	1641.876	1.89	0.00155	17.70982
P01019	0	0.9487	863.4661	1725.925	5.77	0.00498	0
P01019	0	0.76	457.9163	1371.734	0.74	0.00034	22.36007
P01019	0	0.7143	634.8973	1268.787	2.13	0.00135	64.06026
P01019	0	1	519.6589	1556.962	0.56	0.00029	56.07894
P01019	0	0.9524	778.9864	1556.966	2.64	0.00206	14.87644
P01019	0	0.7818	606.3225	1211.638	1.07	0.00065	45.39406
P01019	0	1	721.4128	1441.818	0.8	0.00058	26.5273

P01019	0	0.7647	452.5837	1355.736	-1.47	-0.00067	5.465613
P01019	0	0.7714	529.3096	1585.914	-3.17	-0.00168	18.35841
P01019	0	0.9423	460.2463	919.4852	-0.08	-0.00004	40.67593
P01019	0	1	706.9492	1412.891	3.02	0.00214	44.78402
P01019	0	0.7059	532.2979	1063.589	1.13	0.0006	22.65428
P01019	0	0.9	529.3121	1585.922	1.68	0.00089	14.01908
P01019	0	0.8372	532.2992	1063.591	3.54	0.00188	46.90815
P01019	0	0.7381	606.3233	1211.639	2.48	0.0015	14.54782
P01019	0	0.8462	532.2993	1063.591	3.77	0.002	0
P01019	0	0.878	532.2978	1063.588	0.9	0.00048	14.88288
P08185	0	0.9592	384.9164	1152.735	0.26	0.0001	8.711287
P08185	0	0.8788	488.4626	2438.284	0.73	0.00036	18.96157
P08185	0	0.9571	610.3276	2438.288	2.7	0.00165	4.592879
P08185	0	0.9783	488.463	2438.286	1.67	0.00081	7.141043
P08185	0	0.9057	813.4345	2438.289	2.91	0.00236	3.636947
P08185	0	1	610.3276	2438.289	2.8	0.00171	6.779522
P08185	0	0.975	488.4629	2438.285	1.42	0.00069	17.37265
P08185	0	0.8571	504.8209	1008.634	2.2	0.00111	13.98915
P08185	0	1	854.7338	2562.187	1.08	0.00092	2.631478
P08185	0	1	854.7336	2562.186	0.93	0.0008	13.80791
P08185	0	0.8478	541.9475	1623.828	1.89	0.00102	0
P08185	0	0.9722	812.4172	1623.827	1.44	0.00117	4.045117
P08185	0	0.9706	685.0467	2053.126	2.3	0.00157	5.629933
P08185	0	0.975	384.9174	1152.738	2.88	0.00111	23.33887
P08185	0	0.8491	576.8732	1152.739	4.25	0.00245	5.176298
P08185	0	1	854.7339	2562.187	1.22	0.00104	0
P08185	0	0.9677	620.9898	1860.955	4.27	0.00265	32.90059
P08185	0	0.9643	631.0143	1891.028	4.29	0.00271	17.57139
P08185	0	0.9286	631.0132	1891.025	2.55	0.00161	40.74413
P08185	0	0.9	610.3524	1219.698	2.18	0.00133	20.08575
P08185	0	0.9167	610.3257	2438.281	-0.3	-0.00018	0
P08185	0	0.7857	488.4629	2438.285	1.42	0.00069	0
P08185	0	0.8919	620.9896	1860.954	3.98	0.00247	60.76868
P08185	0	1	740.3657	1479.724	0.93	0.00069	6.876392
P08185	0	0.6757	685.0466	2053.125	2.21	0.00151	6.749557
P08185	0	0.8889	576.8722	1152.737	2.45	0.00141	24.75664
P08185	0	0.9286	631.012	1891.021	0.52	0.00033	18.9997
P08185	0	0.8421	402.2434	1204.716	1.43	0.00057	19.74021
P08185	0	0.7742	631.0126	1891.023	1.48	0.00094	6.879228
P08185	0	0.7586	384.9166	1152.735	0.74	0.00028	31.6497
P08185	0	1	504.8208	1008.634	2.14	0.00108	14.25484
P08185	0	0.7805	570.339	1139.671	3.89	0.00222	0
P08185	0	0.7333	685.0471	2053.127	2.92	0.002	3.563947
P08185	0	1	574.3015	2294.184	1.81	0.00104	39.44958
P08185	0	0.9444	610.353	1219.699	3.08	0.00188	41.96978
P08185	0	1	631.0126	1891.023	1.48	0.00094	19.94828
P08185	0	0.65	541.9465	1623.825	0.08	0.00004	6.566654
P08185	0	0.3043	1027.067	2053.127	3.02	0.0031	0
P08185	0	0.4444	685.0463	2053.124	1.76	0.00121	0
P27169	0	0.9385	736.933	1472.859	1.65	0.00122	7.083886
P27169	0	0.9375	491.6243	1472.858	1.42	0.0007	7.632973
P27169	0	0.8654	439.2838	1315.837	2.05	0.0009	9.680024

P27169	0	1	643.0247	1927.06	1.82	0.00117	15.6372
P27169	0	0.8947	643.0252	1927.061	2.58	0.00166	20.84026
P27169	0	1	676.6788	2028.022	1.22	0.00083	4.777874
P27169	0	1	1014.516	2028.024	2.28	0.00231	0
P27169	0	1	676.6791	2028.023	1.58	0.00107	4.514667
P27169	0	1	536.652	1607.942	1.02	0.00055	3.577966
P27169	0	1	804.4758	1607.944	2.7	0.00217	12.39954
P27169	0	0.9767	536.6518	1607.941	0.56	0.0003	8.787539
P27169	0	1	774.5814	3868.878	1.28	0.00099	4.807421
P27169	0	1	967.9752	3868.879	1.49	0.00144	3.825256
P27169	0	0.9259	491.624	1472.857	0.73	0.00036	11.33763
P27169	0	0.9403	736.9329	1472.858	1.49	0.0011	3.595785
P27169	0	1	896.827	2688.467	1.7	0.00153	6.084014
P27169	0	1	1014.516	2028.024	2.16	0.00219	0.425983
P27169	0	1	804.4752	1607.943	1.94	0.00156	42.5558
P27169	0	0.8333	556.3351	1111.663	2.6	0.00144	14.96833
P27169	0	1	422.2087	1264.611	-2.91	-0.00123	39.48569
P27169	0	0.9459	618.3361	1235.665	2.1	0.0013	0
P27169	0	1	391.2498	1171.735	2.45	0.00096	43.53766
P27169	0	0.9375	491.6239	1472.857	0.55	0.00027	0
P27169	0	0.6552	658.4205	1315.834	-0.16	-0.00011	21.87804
P27169	0	1	439.2833	1315.835	0.94	0.00041	33.67272
P27169	0	0.9333	412.5596	1235.664	1.62	0.00067	65.45844
P27169	0	0.6842	491.6234	1472.856	-0.45	-0.00022	7.857613
P00740	0	1	804.691	2412.058	2.86	0.0023	15.32988
P00740	0	0.962	658.8306	1316.654	2.63	0.00173	2.931862
P00740	0	0.875	598.3423	1793.012	3.49	0.00208	0
P00740	0	0.9032	598.3424	1793.013	3.69	0.00221	0
P00740	0	0.9846	620.3428	1859.014	2.7	0.00167	8.645572
P00740	0	0.96	620.3412	1859.009	0.14	0.00009	64.45072
P00740	0	0.9808	620.3414	1859.01	0.43	0.00027	21.30162
P00740	0	1	613.821	2452.262	2.67	0.00164	5.017199
P00740	0	0.8704	562.3326	1123.658	1.59	0.00089	2.843107
P00740	0	1	449.0078	1793.01	1.95	0.00087	28.52361
P00740	0	0.8261	658.3815	1315.756	4.3	0.00283	0
P00740	0	1	551.9461	1653.824	3.58	0.00198	31.2692
P00740	0	0.7778	603.8712	1206.735	1.96	0.00118	41.89011
P00740	0	0.9444	449.0076	1793.009	1.47	0.00066	43.30343
P00740	0	0.8	439.5561	1316.654	2.46	0.00108	17.1138
P00740	0	0.8889	561.9593	1683.863	1.13	0.00063	2.786082
P00740	0	0.75	531.8189	1062.631	-0.06	-0.00003	20.90695
P00740	0	0.64	439.2573	1315.757	5.64	0.00247	36.78167
O14791	0	1	578.9901	1734.956	4.5	0.0026	18.51563
O14791	0	1	434.4933	1734.951	2	0.00087	14.75358
O14791	0	0.7333	545.3296	1089.652	1.18	0.00064	8.734852
O14791	0	0.8542	545.3295	1089.652	0.95	0.00052	4.997888
O14791	1	0.7143	489.6195	1466.844	0.79	0.00039	27.0965
O14791	0	0.9388	628.6855	1884.042	2.6	0.00163	14.59513
O14791	0	0.6364	560.2927	1119.578	1.74	0.00097	36.13098
O14791	0	0.7885	617.3512	1233.695	1.81	0.00112	6.741108
O14791	0	0.7778	465.2733	1393.805	0.88	0.00041	4.342271
O14791	1	0.4815	489.6196	1466.844	1.11	0.00054	32.63935

O14791	0	0.9615	617.3514	1233.696	2.21	0.00136	9.32138
O14791	0	0.7576	411.9031	1233.695	1.38	0.00057	5.815896
O14791	0	1	537.3018	1073.596	0.32	0.00017	7.688614
O14791	0	0.3143	495.7526	990.4979	0.67	0.00033	0
O14791	0	0.6818	411.9029	1233.694	1.01	0.00042	13.96441
O14791	0	0.7059	617.3513	1233.695	2.01	0.00124	0
O14791	0	0.5	398.8872	1194.647	1.31	0.00052	42.91328
O14791	0	1	537.3023	1073.597	1.22	0.00066	9.14649
O14791	0	0.75	697.4078	1393.808	3.04	0.00212	0
P06276	0	0.7368	420.6038	1259.797	3.33	0.0014	15.94152
P06276	0	0.8163	630.4023	1259.797	3.87	0.00244	6.036501
P06276	0	0.8367	426.2467	1276.726	0.37	0.00016	17.46573
P06276	0	0.9524	682.5798	2727.298	2.04	0.00139	4.929735
P06276	0	0.9857	649.6761	1947.014	1.81	0.00117	8.842005
P06276	0	0.913	505.9535	1515.846	0.69	0.00035	0
P06276	0	0.9057	758.4288	1515.85	3.58	0.00271	27.49824
P06276	0	0.8571	671.3618	1341.716	2.37	0.00159	6.694935
P06276	1	0.7308	511.3359	1531.993	2.24	0.00114	14.91957
P06276	1	0.8929	470.9498	1410.835	2.25	0.00106	37.8616
P06276	0	0.973	468.9056	1404.702	1.73	0.00081	16.42976
P06276	0	0.9286	674.3723	1347.737	2.52	0.0017	7.576026
P06276	0	0.9706	686.3773	1371.747	3.16	0.00217	45.10973
P02765	0	1	561.0378	2241.13	4.14	0.00232	11.08577
P02765	0	1	561.0381	2241.131	4.57	0.00256	11.12582
P02765	0	0.9697	561.0364	2241.124	1.52	0.00085	5.864598
P02765	0	1	557.038	2225.13	2.1	0.00117	4.588538
P02765	0	1	561.0374	2241.128	3.27	0.00183	8.537275
P02765	0	1	561.0361	2241.122	0.98	0.00055	25.00367
P02765	0	1	557.0371	2225.127	0.57	0.00032	28.35814
P02765	0	1	561.037	2241.126	2.61	0.00146	0
P02765	0	1	561.0377	2241.129	3.81	0.00214	35.15144
P02765	0	1	561.0361	2241.122	0.98	0.00055	8.205567
P02765	0	1	1008.541	2016.075	2.72	0.00275	0
P02765	0	1	672.6959	2016.073	1.75	0.00118	1.172322
P02765	0	1	1008.541	2016.074	2.18	0.0022	0
P02765	0	1	525.0108	2097.021	1.52	0.0008	0
P02765	0	1	561.037	2241.126	2.61	0.00146	15.84392
P02765	0	1	561.0372	2241.127	2.94	0.00165	20.07414
P02765	0	1	561.0347	2241.117	-1.53	-0.00085	25.51995
P02765	0	1	561.0372	2241.127	2.94	0.00165	21.34471
P02765	0	1	747.7125	2241.123	1.15	0.00086	0
P02765	0	1	720.7301	2160.176	1.94	0.0014	4.863881
P02765	0	1	557.038	2225.13	2.1	0.00117	7.35143
P02765	0	1	557.038	2225.13	2.21	0.00123	6.513257
P02765	0	1	557.0372	2225.127	0.79	0.00044	2.519679
P02765	0	1	742.381	2225.128	1.37	0.00101	2.457513
P02765	0	0.987	694.3471	2081.027	1.67	0.00116	1.334982
P02765	0	1	561.0376	2241.129	3.7	0.00207	27.17808
P02765	0	1	561.0365	2241.124	1.74	0.00098	14.63333
P02765	0	1	557.038	2225.13	2.1	0.00117	2.167
P02765	0	1	445.8317	2225.129	1.74	0.00077	32.19185
P02765	0	1	742.3806	2225.127	0.79	0.00059	2.380493

P02765	0	1	557.0377	2225.129	1.66	0.00093	0.171154
P02765	0	1	694.347	2081.026	1.5	0.00104	0
P02765	0	1	557.0382	2225.131	2.54	0.00141	4.674048
P02765	0	1	747.7116	2241.12	0.01	0	10.25762
P02765	0	1	561.036	2241.122	0.76	0.00043	5.47052
P02765	0	1	561.0367	2241.125	2.18	0.00122	16.90218
P02765	0	1	694.3477	2081.028	2.47	0.00171	3.961037
P02765	0	1	521.0125	2081.028	2.25	0.00117	13.20379
P02765	0	1	521.0126	2081.028	2.49	0.00129	0
P02765	0	0.9556	694.3468	2081.026	1.23	0.00086	1.896299
P02765	0	1	1217.924	3651.757	1.48	0.0018	3.22273
P02765	0	1	913.6953	3651.759	2.09	0.00191	0
P02765	0	1	540.7992	2160.175	1.56	0.00084	18.25223
P02765	0	1	1080.591	2160.175	1.52	0.00164	0
P02765	0	1	720.7288	2160.172	0.16	0.00011	2.420577
P02765	0	1	540.7985	2160.172	0.32	0.00017	41.36589
P02765	0	1	1274.646	3821.923	1.42	0.00181	0
P02765	0	0.96	765.1904	3821.923	1.41	0.00108	13.95909
P02765	0	1	720.7296	2160.174	1.17	0.00085	2.537074
P02765	0	1	672.6951	2016.071	0.57	0.00038	5.42997
P02765	0	1	956.2363	3821.923	1.46	0.0014	0
P02765	0	0.9722	956.5098	2867.515	1.62	0.00154	11.48514
P02765	0	0.75	495.6186	1484.841	6.81	0.00337	33.31622
P02765	0	0.7857	551.332	1101.657	1.95	0.00107	26.79151
P02765	0	1	908.4778	2723.419	3.95	0.00358	3.633132
P02765	0	1	717.6342	2867.515	1.65	0.00119	4.069098
P02765	0	1	1434.26	2867.513	0.94	0.00135	0
P02765	0	0.9808	487.9977	1948.969	0.56	0.00027	8.130723
P02765	0	1	974.9896	1948.972	2.14	0.00209	6.990175
P02765	0	1	650.3278	1948.969	0.54	0.00035	8.384644
P02765	0	0.9844	602.295	1804.87	2.65	0.0016	4.882636
P02765	0	0.9792	487.9979	1948.97	1.06	0.00052	8.446928
P02765	0	1	974.9897	1948.972	2.27	0.00221	5.025165
P02765	0	1	650.3283	1948.97	1.29	0.00084	7.577567
P02765	0	1	974.9899	1948.972	2.46	0.00239	9.155133
P02765	0	0.9815	650.3287	1948.972	2.04	0.00133	27.28166
P02765	0	0.9818	650.3284	1948.971	1.48	0.00096	10.36937
P02765	0	0.9344	650.3289	1948.972	2.23	0.00145	13.05327
P02765	0	1	650.3289	1948.972	2.23	0.00145	8.41076
P02765	0	0.975	487.998	1948.97	1.25	0.00061	7.311262
P02765	0	1	650.3277	1948.969	0.44	0.00029	8.011565
P02765	0	0.9672	602.2952	1804.871	2.96	0.00178	2.952495
P02765	0	0.8182	650.3286	1948.971	1.85	0.0012	6.408669
P02765	0	1	717.6335	2867.512	0.72	0.00051	1.95078
P02765	0	0.6486	495.616	1484.833	1.57	0.00078	17.82394
P02765	0	0.5625	495.6153	1484.831	0.15	0.00008	7.78282
P02765	0	0.8548	742.9205	1484.834	1.81	0.00134	5.984344
P02765	0	0.7792	742.9204	1484.833	1.56	0.00116	5.222761
P02765	0	0.7812	495.6153	1484.831	0.22	0.00011	8.701668
P02765	0	0.7826	670.8693	1340.731	1.65	0.0011	0
P02765	0	0.9592	650.329	1948.972	2.42	0.00157	16.47215
P02765	0	1	669.2681	1337.529	1.09	0.00073	0

P02765	0	1	650.3291	1948.973	2.61	0.00169	59.94659
P02765	0	0.9851	956.512	2867.521	3.85	0.00368	5.655369
P02765	0	0.7368	495.6168	1484.836	3.18	0.00157	28.15335
P02765	0	1	741.3184	1481.63	0.07	0.00005	0
P02765	0	1	561.0365	2241.124	1.74	0.00098	0
P02765	0	0.6	504.2837	1007.56	-1.08	-0.00055	0
P02765	0	1	672.6959	2016.073	1.75	0.00118	0
P02765	0	0.5208	504.2846	1007.562	0.8	0.0004	2.900759
P02765	0	0.9583	495.6166	1484.835	2.74	0.00136	36.50672
P02765	0	0.7143	495.6161	1484.834	1.82	0.0009	39.74815
P02765	0	0.8621	650.3298	1948.975	3.73	0.00243	0
P02765	0	0.8958	670.8692	1340.731	1.56	0.00104	31.0262
P02765	0	0.9375	487.9979	1948.97	1.13	0.00055	50.32969
P02765	0	0.8036	551.3305	1101.654	-0.82	-0.00045	0
P02765	0	1	742.381	2225.129	1.45	0.00108	2.163415
P02765	0	0.9444	495.6177	1484.838	4.96	0.00246	44.55171
P02765	0	0.8857	447.5821	1340.732	2.07	0.00093	27.93489
P02765	0	0.5818	504.284	1007.561	-0.42	-0.00021	3.437401
P02765	0	0.8036	551.3323	1101.657	2.5	0.00138	14.67101
P02765	0	0.5294	504.2843	1007.561	0.25	0.00013	4.740056
P02765	0	1	699.6809	2097.028	4.67	0.00326	0
P02765	0	1	561.0375	2241.128	3.49	0.00195	50.71252
P02765	0	0.9474	495.6158	1484.833	1.14	0.00056	78.73918
P02765	0	0.9655	747.7134	2241.126	2.37	0.00177	0
P02765	0	0.7193	551.3325	1101.658	2.83	0.00156	6.232641
P02765	0	0.7857	551.332	1101.657	1.83	0.00101	5.760327
P02765	0	0.6364	495.6164	1484.835	2.31	0.00114	30.36004
P02765	0	1	669.2687	1337.53	2	0.00134	8.910394
P02765	0	0.5818	504.2835	1007.56	-1.39	-0.0007	5.081807
P02765	0	1	720.7317	2160.181	4.14	0.00298	45.71784
P02765	0	0.7391	650.3295	1948.974	3.17	0.00206	24.6255
P02765	0	0.9667	479.2814	957.5555	3.02	0.00144	0
P02765	0	0.7857	551.3319	1101.657	1.72	0.00095	5.530228
P02765	0	1	561.0387	2241.133	5.66	0.00317	0
P02765	0	0.7222	504.2842	1007.561	-0.05	-0.00003	0
P02765	0	0.6905	504.2842	1007.561	-0.11	-0.00006	26.011
P02765	0	0.8605	551.3314	1101.655	0.73	0.0004	0
P02765	0	0.8	747.714	2241.127	3.19	0.00238	4.787404
P02765	0	0.8824	495.6167	1484.836	3.05	0.00151	78.09561
P02765	0	1	557.0371	2225.126	0.46	0.00025	21.53825
P02765	0	0.9615	561.0381	2241.131	4.57	0.00256	3.687115
P02765	0	0.7895	551.3314	1101.656	0.84	0.00046	15.15243
P02765	0	0.6129	504.2857	1007.564	2.98	0.0015	28.41929
P02765	0	0.7568	568.3248	1135.642	3.45	0.00196	0
P02765	0	0.9667	479.2808	957.5544	1.87	0.00089	8.087171
P02765	0	1	672.6964	2016.075	2.48	0.00166	1.396828
P02765	0	1	447.5819	1340.731	1.66	0.00074	36.11278
P02765	0	0.9667	479.2811	957.5549	2.38	0.00114	3.612337
P02765	0	0.8235	650.3292	1948.973	2.79	0.00181	0
P02765	0	1	557.0377	2225.129	1.55	0.00086	32.82062
P02765	0	1	447.5822	1340.732	2.27	0.00102	35.98244
P02765	0	0.7895	551.3306	1101.654	-0.71	-0.00039	13.36723

P02765	0	0.4286	487.9981	1948.97	1.38	0.00067	9.404763
P02765	0	1	742.3807	2225.127	0.96	0.00071	2.700198
P02765	0	1	1080.591	2160.175	1.63	0.00176	0
P02765	0	0.72	487.9981	1948.97	1.44	0.0007	10.21205
P02765	0	0.7895	551.3311	1101.655	0.17	0.0001	5.30816
P02765	0	1	447.5818	1340.731	1.32	0.00059	0
P02765	0	0.6667	602.2971	1804.877	6.1	0.00367	16.13634
P02765	0	0.8158	551.3318	1101.656	1.5	0.00083	26.7378
P02765	0	0.7	650.3291	1948.973	2.61	0.00169	30.76006
P02765	0	1	557.0379	2225.13	1.99	0.00111	28.35987
P02765	0	1	561.036	2241.122	0.76	0.00043	30.63366
P02765	0	1	717.6348	2867.517	2.51	0.0018	13.62547
P02765	0	0.6316	902.9365	1804.866	0.09	0.00008	4.724318
P02765	0	0.9333	741.3187	1481.63	0.48	0.00036	0
P02765	0	0.9756	479.2806	957.5539	1.42	0.00068	36.3133
P00738	0	0.9692	623.3119	1867.921	2.6	0.00162	5.841087
P00738	0	0.9231	623.3125	1867.923	3.58	0.00223	18.76155
P00739; P0	0	1	545.2894	1633.854	2.41	0.00131	8.85826
P00739; P0	0	0.6667	404.5835	1211.736	0.76	0.00031	23.85787
P00739; P0	0	0.8594	606.3724	1211.737	2.03	0.00123	6.361173
P00739; P0	0	0.575	404.583	1211.734	-0.6	-0.00024	14.35788
P00738	1	1	442.5029	1766.99	-0.96	-0.00043	4.16309
P00738	0	0.9216	497.9524	1491.843	1.19	0.00059	15.19254
P00739; P0	0	0.6452	404.5835	1211.736	0.68	0.00028	27.89671
P00738	0	0.8571	695.0775	2083.218	1.26	0.00087	7.109708
P00738	0	1	615.8231	2460.271	3.62	0.00223	4.733875
P00738	0	1	820.7597	2460.265	1.14	0.00094	4.056282
P00739; P0	0	1	789.972	1578.937	1.39	0.00109	5.505499
P00739; P0	0	1	526.9839	1578.937	1.65	0.00087	9.713168
P00738	0	1	615.8215	2460.264	1.04	0.00064	9.948783
P00738	0	1	772.7266	2316.165	2.43	0.00188	1.804138
P00738	0	0.7778	497.9528	1491.844	1.87	0.00093	11.74532
P00738	0	0.8778	746.4249	1491.843	1.1	0.00082	6.579601
P00738	0	0.7708	497.9518	1491.841	-0.04	-0.00002	8.481789
P00738	0	0.8889	746.4244	1491.842	0.44	0.00033	5.805256
P00738	0	0.9778	623.3116	1867.92	2.11	0.00131	68.85376
P00738	0	1	934.4636	1867.92	1.98	0.00185	0
P00738	0	1	623.3114	1867.92	1.81	0.00113	20.64974
P00738	0	0.8409	497.9523	1491.842	1.01	0.0005	4.707512
P00738	0	0.717	617.98	1851.926	2.28	0.00141	0
P00738	0	0.9762	617.9805	1851.927	3.07	0.0019	13.38197
P00738	0	0.9663	617.9797	1851.925	1.78	0.0011	5.030733
P00738	0	1	617.9806	1851.927	3.17	0.00196	51.99686
P00739; P0	0	0.9362	717.9207	1434.834	1.11	0.00079	9.209297
P00739; P0	0	1	789.9721	1578.937	1.54	0.00122	7.52731
P00739; P0	0	0.9608	526.9834	1578.936	0.72	0.00038	6.662654
P00738	1	1	427.4916	1706.944	0.44	0.00019	37.65122
P00738	0	0.6889	717.8783	1434.749	1.82	0.00131	5.371442
P00738	0	0.6818	717.878	1434.749	1.39	0.001	5.882028
P00738	0	0.64	478.9212	1434.749	1.7	0.00081	19.30486
P00738	0	0.7561	478.9214	1434.75	1.96	0.00094	9.009421
P00739; P0	0	1	533.9432	1599.815	2.84	0.00152	13.07789

P00739; P0	0	0.9863	800.4113	1599.815	3.08	0.00246	8.227809
P00738	0	0.9851	576.6287	1727.872	1.51	0.00087	10.05374
P00738	0	1	864.4396	1727.872	1.73	0.0015	5.701071
P00739; P0	0	0.8714	800.411	1599.815	2.62	0.0021	0
P00738	0	1	864.439	1727.871	1.02	0.00089	6.966609
P00738	0	0.9821	792.3877	1583.768	0.74	0.00059	0
P00738	0	0.9792	576.6295	1727.874	2.78	0.0016	8.598439
P00738	0	1	864.4403	1727.873	2.44	0.00211	21.27652
P00738	0	0.9833	576.6281	1727.87	0.45	0.00026	9.288018
P00738	0	1	576.6289	1727.872	1.72	0.00099	44.44397
P00739; P0	0	0.9821	545.2885	1633.851	0.73	0.0004	9.662613
P00739; P0	0	0.9565	545.2874	1633.848	-1.4	-0.00076	17.14644
P00739; P0	0	1	817.4298	1633.852	1.49	0.00122	5.912034
P00739; P0	0	1	545.2888	1633.852	1.18	0.00064	10.99026
P00739; P0	0	1	817.4294	1633.851	1.04	0.00085	9.215112
P00738	0	1	650.9179	3250.56	3.23	0.0021	3.063589
P00738	0	1	650.9176	3250.559	2.76	0.00179	6.028208
P00739; P0	0	0.9792	545.2889	1633.852	1.4	0.00077	40.41518
P00739; P0	0	1	545.2897	1633.855	2.97	0.00162	61.47524
P00739; P0	0	0.9211	545.2891	1633.853	1.74	0.00095	9.576406
P00738	0	1	650.9177	3250.56	2.95	0.00191	21.74493
P00739; P0	0	0.9615	545.2885	1633.851	0.73	0.0004	10.92377
P00739; P0	0	1	545.2891	1633.853	1.85	0.00101	7.647542
P00739; P0	0	0.9643	545.2882	1633.85	0.17	0.00009	7.670223
P00739; P0	0	0.9833	666.8856	1332.764	1.26	0.00084	6.644083
P00739; P0	0	1	607.3321	1819.982	2.58	0.00157	12.64681
P00739; P0	0	1	455.7507	1819.981	2.24	0.00102	11.57495
P00739; P0	1	0.4146	473.6367	1418.895	0.82	0.00039	28.42399
P00739; P0	1	0.9302	617.7175	1851.138	3.51	0.00217	19.44054
P00738	0	0.6579	497.9521	1491.842	0.58	0.00029	6.212155
P00738	0	0.6875	478.9204	1434.746	-0.15	-0.00007	9.035597
P00738	0	0.9167	562.8036	1124.6	2.67	0.0015	4.282324
P00738	0	0.6364	478.9206	1434.747	0.36	0.00017	0
P00739; P0	0	0.8254	594.8354	1188.664	2.86	0.0017	10.16927
P00738	0	0.6087	478.921	1434.749	1.26	0.0006	16.79485
P00738	0	0.6087	478.9213	1434.749	1.9	0.00091	7.888613
P00739; P0	0	0.9744	817.4287	1633.85	0.14	0.00012	0
P00739; P0	0	0.9189	526.9843	1578.938	2.46	0.00129	10.61268
P00739; P0	0	1	604.8381	1208.669	1.98	0.00119	0
P00738	0	0.8077	478.9202	1434.746	-0.4	-0.00019	15.9898
P00739; P0	0	1	604.837	1208.667	0.06	0.00003	5.94458
P00739; P0	0	1	604.8383	1208.669	2.18	0.00132	6.355654
P00739; P0	0	0.8333	545.2889	1633.852	1.52	0.00083	7.819473
P00738	0	1	926.4666	1851.926	2.48	0.0023	43.9442
P00738	1	1	427.4915	1706.944	0.22	0.0001	79.15483
P00738	0	0.913	478.9209	1434.748	1.07	0.00051	11.34952
P00738	0	0.85	478.9213	1434.749	1.83	0.00088	34.76339
P00739; P0	0	0.5556	444.9262	1332.764	1.3	0.00058	16.70804
P00738	0	0.7458	674.3785	1347.75	8.11	0.00546	12.56084
P00738	0	0.8125	478.9204	1434.747	-0.08	-0.00004	62.02139
P00739; P0	0	0.8387	594.8349	1188.663	2.04	0.00121	15.26654
P00739; P0	0	0.3125	404.5837	1211.736	1.13	0.00046	38.3526

P00738	0	0.6207	478.921	1434.749	1.26	0.0006	7.610994
P00739; P0	0	0.8621	526.9837	1578.937	1.3	0.00068	30.90254
P00739; P0	0	0.7692	444.9258	1332.763	0.61	0.00027	20.90685
P00739; P0	0	0.8889	450.258	1348.759	1.68	0.00076	28.44966
P00738	0	0.9655	576.6296	1727.874	3.1	0.00179	0
P00739; P0	0	0.7059	404.5837	1211.737	1.29	0.00052	32.86251
P00739; P0	0	0.5957	501.8018	1002.596	1.12	0.00056	26.13306
P00739; P0	0	0.381	404.5838	1211.737	1.44	0.00058	34.80645
P00738	0	0.9375	674.3762	1347.745	4.58	0.00308	20.71214
P00739; P0	0	0.7037	404.5843	1211.738	2.8	0.00113	25.39663
P00738	0	1	562.8035	1124.6	2.56	0.00144	41.50185
P00738	2	1	409.0085	1633.012	0.16	0.00007	27.51033
P00739; P0	0	1	728.3563	1455.705	-2.06	-0.0015	0
P00739; P0	0	0.3784	573.8538	1146.7	2.73	0.00156	48.05908
P00739; P0	0	0.7347	534.3206	1067.634	0.94	0.0005	0
P00738	0	1	934.4642	1867.921	2.64	0.00246	3.733863
P00738	0	0.72	617.9805	1851.927	2.97	0.00183	15.12336
P00738	0	0.3478	478.9211	1434.749	1.45	0.00069	9.637491
P00738	0	1	854.413	1707.819	-0.3	-0.00026	6.346492
P00738	1	1	427.4917	1706.945	0.8	0.00034	13.9704
P00739; P0	0	0.9231	403.5613	1208.669	2.14	0.00086	9.922118
P00739; P0	0	0.5833	396.8925	1188.663	2.36	0.00094	0
P00739; P0	0	0.7333	604.8378	1208.668	1.47	0.00089	16.93134
P00738	0	0.52	497.9518	1491.841	-0.1	-0.00005	17.20039
P00738	0	1	926.4667	1851.926	2.55	0.00236	21.70416
P00739; P0	0	0.7812	604.8389	1208.67	3.19	0.00193	6.641055
P00738	0	1	562.8035	1124.6	2.56	0.00144	22.53116
P00738	0	1	562.8019	1124.597	-0.27	-0.00015	3.374265
P00738	0	0.9231	478.9208	1434.748	0.87	0.00042	3.263679
P00738	0	1	854.4148	1707.822	1.77	0.00151	8.850186
P00738	0	1	490.7521	980.4969	2.14	0.00105	4.756501
P00739; P0	0	0.8	604.8384	1208.67	2.48	0.0015	9.656388
P00738	0	0.9677	562.8037	1124.6	2.77	0.00156	27.45691
P00739; P0	0	0.55	533.9431	1599.815	2.61	0.00139	29.20991
P00738	0	1	375.5381	1124.6	2.49	0.00093	0
P00738	0	0.3889	478.9212	1434.749	1.58	0.00075	15.42829
P00739; P0	0	0.5333	450.2581	1348.76	1.95	0.00088	42.17896
P00738	0	1	617.9791	1851.923	0.8	0.00049	28.43826
P00738	0	1	926.4666	1851.926	2.42	0.00224	37.28574
P00738	0	1	576.6289	1727.872	1.83	0.00105	14.48663
P00738	0	0.5556	521.5609	2083.222	2.98	0.00155	19.52695
P00738	0	0.7273	695.0773	2083.217	0.91	0.00063	25.58336
P00738	0	0.1579	449.9185	1347.741	1.51	0.00068	26.11288
P00738	0	1	569.9457	1707.822	1.87	0.00107	44.09102
P00738	1	1	427.4916	1706.945	0.58	0.00025	8.750884
P00738	0	0.4167	695.0778	2083.219	1.61	0.00112	4.576386
P00738	0	0.1	623.312	1867.921	2.69	0.00168	4.906315
P00738	0	0.9231	432.725	1727.878	5.18	0.00224	0
P00739; P0	0	0.625	573.852	1146.697	-0.46	-0.00027	22.40576
P35527	0	1	896.3699	1791.733	2.73	0.00244	0
P35527	0	1	616.8042	1232.601	2.69	0.00166	0
P35527	0	1	618.2692	1235.531	1.99	0.00123	0

P35527	0	0.9872	625.6638	1874.977	3.7	0.00231	5.989451
P35527	0	1	459.5715	1376.7	0	0	12.92555
P35527	0	1	460.5502	1379.636	3.82	0.00176	51.88376
P35527	0	0.725	602.8385	1204.67	3.61	0.00217	13.70412
P35527	0	0.5429	470.601	1409.789	0.52	0.00025	0
P35527	0	0.5385	376.1838	1126.537	0.98	0.00037	30.57482
P35527	0	1	498.9421	1494.812	2.74	0.00136	15.6873
P35527	0	0.8	605.8072	1210.607	5.32	0.00322	4.991724
P35527	0	1	541.2506	1081.494	-0.28	-0.00015	0
P35527	0	0.9286	616.8037	1232.6	1.9	0.00117	4.245039
P35527	0	1	618.2686	1235.53	0.9	0.00056	0
P35527	0	0.7568	521.2621	1041.517	1.3	0.00068	6.579479
P35527	0	1	533.2537	1065.5	0.78	0.00042	9.834361
P04264	0	0.8958	581.8569	1162.707	2.76	0.0016	49.20056
P04264	0	0.9688	540.6227	1619.854	1.68	0.00091	24.21158
P04264	0	0.9828	810.4312	1619.855	2.52	0.00204	3.679964
Q7Z794; P3	0	0.9778	588.6707	1763.998	4.65	0.00273	64.9304
P04264	0	0.8539	795.9645	1590.922	1.53	0.00121	7.791595
P04264	0	0.9512	518.6195	1553.844	1.7	0.00088	21.95787
P04264	0	0.88	589.3143	1177.621	2.69	0.00159	1.442891
P04264	0	0.9714	581.8554	1162.703	0.03	0.00002	64.09101
P04264	0	0.8605	589.3148	1177.622	3.52	0.00207	17.87667
P04264	0	0.8605	589.3153	1177.623	4.45	0.00262	20.09166
P04264	0	0.871	540.6227	1619.853	1.57	0.00085	0
P04264	0	1	429.8895	1287.654	3.67	0.00158	38.94283
P04264	0	0.9333	517.2623	1033.517	1.19	0.00061	14.48141
P04264	0	1	637.2846	1909.839	1.21	0.00077	0
P04264	0	0.7931	451.9145	1353.729	2.71	0.00122	22.81365
P04264	0	0.8611	488.7997	976.5921	1.36	0.00067	21.70732
P04264	0	0.5429	546.8114	1092.616	1.49	0.00081	41.32553
P04264	0	0.7619	557.9708	1671.898	2.09	0.00116	40.34591
P04264	0	0.7037	644.3298	1287.652	2.31	0.00149	5.924978
Q7Z794; P3	0	0.5	540.6342	1619.888	0.36	0.00019	5.406788
Q04756	0	1	489.8902	1467.656	0.33	0.00016	15.67211
Q04756	0	1	862.4514	2585.34	-1.06	-0.00091	5.12492
Q04756	1	1	580.6244	1739.859	3.47	0.00201	13.92158
Q04756	0	1	400.9426	1600.749	2.89	0.00116	22.17219
Q04756	0	1	702.3971	2105.177	2.11	0.00148	9.672229
Q04756	0	0.8837	543.2987	1627.882	2.12	0.00115	25.94655
Q04756	0	1	489.8906	1467.657	1.08	0.00053	33.91015
Q04756	0	1	647.2974	1939.878	2.45	0.00158	9.49091
Q04756	0	0.8333	862.4548	2585.35	2.83	0.00244	27.40079
Q04756	0	1	569.921	1707.749	2.18	0.00124	10.88539
Q04756	0	0.5862	647.2999	1939.885	6.41	0.00415	18.68185
Q04756	0	0.6562	430.5486	1289.631	2.71	0.00117	8.129224
Q04756	0	0.8571	694.0115	2080.02	1.85	0.00128	5.353076
Q04756	0	0.8611	645.3189	1289.631	2.27	0.00146	3.193021
Q04756	0	0.92	623.2983	1245.589	2.1	0.00131	0
Q04756	0	0.8387	645.319	1289.631	2.37	0.00153	0
Q04756	0	1	647.2971	1939.877	1.98	0.00128	0
P03951	0	1	457.2388	1369.702	0.36	0.00016	31.20362
P03951	0	0.8276	678.6613	2033.969	1.67	0.00113	11.89216

P03951	0	1	540.9323	1620.782	-0.04	-0.00002	16.12831
P03951	0	0.8182	386.5452	1157.621	2.38	0.00092	18.98826
P03951	0	1	540.2958	1618.873	1.05	0.00057	0
P03951	0	1	470.2455	1408.722	4.52	0.00212	9.023875
P03951	0	0.6944	533.6292	1598.873	1.81	0.00096	16.7834
P03951	0	0.9615	745.3693	2234.093	2.02	0.0015	19.55761
P03951	0	0.7273	562.8507	1124.694	2.12	0.00119	33.57146
P03951	0	0.8824	581.3174	1161.627	3.71	0.00216	10.41822
P03951	0	0.675	566.8511	1132.695	1.92	0.00109	7.214792
Q92954	0	0.7895	629.3302	1885.976	3.41	0.00215	0
Q92954	0	0.6042	523.8085	1046.61	1.31	0.00068	38.63663
Q92954	0	0.8431	548.3112	1095.615	1.34	0.00074	0
Q92954	0	1	802.9207	1604.834	-0.61	-0.00049	70.9383
Q92954	0	1	599.0241	1795.058	1.1	0.00066	30.46271
Q92954	1	0.8214	504.6029	1511.794	2	0.00101	43.43458
Q92954	0	0.7447	561.8171	1122.627	2.26	0.00127	7.51176
Q92954	0	0.7442	590.347	1179.687	3.71	0.00219	27.4754
Q92954	0	0.5714	505.2523	1513.742	1.37	0.00069	4.088009
Q92954	0	0.88	482.2738	1444.807	2.04	0.00098	12.76896
Q92954	0	0.8571	535.6168	1604.836	0.49	0.00026	63.43634
Q92954	0	1	452.5692	1355.693	2.26	0.00102	22.79264
Q92954	0	0.6579	523.8086	1046.61	1.42	0.00075	100
P02766	1	1	604.313	1810.924	1.68	0.00101	8.723731
P02766	1	1	604.313	1810.924	1.68	0.00101	8.723731
P02766	1	1	604.314	1810.927	3.29	0.00199	8.555555
P02766	1	1	604.314	1810.927	3.29	0.00199	8.555555
P02766	1	1	453.4865	1810.924	1.64	0.00074	11.30028
P02766	1	1	453.4865	1810.924	1.64	0.00074	11.30028
P02766	1	1	546.9898	1638.955	-0.7	-0.00038	69.78943
P02766	0	1	378.4712	1510.863	1.24	0.00047	0
P02766	0	1	504.291	1510.858	-1.76	-0.00089	36.4107
P02766	0	1	504.2926	1510.863	1.39	0.0007	46.18596
P02766	0	0.9474	561.6154	1682.832	3.03	0.0017	12.58949
P02766	0	1	504.2918	1510.861	-0.06	-0.00003	25.94839
P02766	1	0.9744	595.0262	1783.064	3.32	0.00197	18.96031
P02766	1	1	446.5211	1783.063	2.56	0.00114	30.60761
P02766	1	1	595.0264	1783.065	3.73	0.00222	20.62808
P02766	1	1	446.5211	1783.063	2.56	0.00114	23.687
P02766	0	1	504.2922	1510.862	0.61	0.00031	15.76781
P02766	0	1	504.2927	1510.863	1.58	0.00079	4.898794
P02766	0	1	755.9358	1510.864	2.15	0.00162	3.199323
P02766	0	1	456.2576	1366.758	-0.62	-0.00028	35.6737
P02766	0	1	504.2928	1510.864	1.88	0.00095	4.038014
P02766	0	1	504.2926	1510.863	1.45	0.00073	5.446237
P02766	0	1	456.2581	1366.76	0.52	0.00024	1.349752
P02766	0	1	683.8842	1366.761	1.49	0.00102	1.013427
P02766	0	1	456.2583	1366.76	1.06	0.00048	6.610992
P02766	0	1	683.8845	1366.762	1.94	0.00133	3.713634
P02766	0	1	456.2585	1366.761	1.46	0.00067	0
P02766	0	1	683.8846	1366.762	2.21	0.00151	1.616585
P02766	0	1	456.2587	1366.762	1.86	0.00085	22.64582
P02766	0	1	456.2582	1366.76	0.66	0.0003	20.15794

P02766	0	1	649.5837	2595.313	2.28	0.00148	33.72268
P02766	0	1	649.5843	2595.315	3.13	0.00203	15.1104
P02766	0	1	865.7759	2595.313	2.26	0.00196	0
P02766	0	1	649.5837	2595.313	2.19	0.00142	3.652207
P02766	1	1	551.0881	2751.412	1.18	0.00065	9.041449
P02766	0	0.9286	561.6155	1682.832	3.25	0.00182	8.779822
P02766	1	1	695.1278	2777.489	1.45	0.00101	13.59384
P02766	0	0.8548	841.9188	1682.83	2.28	0.00192	5.681471
P02766	0	0.8772	561.6163	1682.834	4.77	0.00268	27.52516
P02766	2	1	734.1531	2933.591	1.53	0.00112	6.366961
P02766	1	1	926.502	2777.491	2.29	0.00212	5.705649
P02766	0	0.9615	883.4907	2648.458	5.7	0.00503	3.644147
P02766	1	1	1317.195	2633.383	0.11	0.00014	0
P02766	1	1	878.4676	2633.388	1.96	0.00172	9.772348
P02766	0	0.975	915.1246	2743.359	1.43	0.00131	7.383872
P02766	2	1	930.5003	2789.486	0.79	0.00073	1.993159
P02766	2	0.975	978.5361	2933.594	2.59	0.00254	4.089589
P02766	2	1	734.1528	2933.59	1.11	0.00082	29.21631
P02766	1	0.9688	701.8913	2804.543	-0.1	-0.00007	30.91034
P02766	1	0.9815	604.3138	1810.927	2.99	0.00181	10.57843
P02766	1	0.9815	604.3138	1810.927	2.99	0.00181	10.57843
P02766	1	1	652.3477	1955.029	2.64	0.00172	23.58516
P02766	1	0.9412	489.5123	1955.027	2.07	0.00101	27.99917
P02766	1	0.7931	489.5127	1955.029	2.76	0.00135	67.56787
P02766	1	1	652.3485	1955.031	3.86	0.00251	21.81575
P02766	0	1	561.6154	1682.832	3.14	0.00176	13.93065
P02766	0	0.7755	841.92	1682.833	3.73	0.00314	6.925662
P02766	2	0.9302	734.1533	2933.591	1.7	0.00124	7.383788
P02766	0	0.8571	686.5949	2743.358	0.94	0.00064	53.97847
P02766	1	1	695.1276	2777.489	1.28	0.00089	7.305963
P02766	1	1	410.4949	1638.958	1.15	0.00047	28.81027
P02766	2	0.9487	930.501	2789.488	1.51	0.0014	2.755229
P02766	2	1	698.1273	2789.487	1.09	0.00076	5.908988
P02766	1	1	498.9571	1494.857	1.91	0.00095	23.97732
P02766	1	1	446.521	1783.062	2.21	0.00099	58.7527
P02766	0	0.9231	649.5831	2595.311	1.34	0.00087	39.86946
P02766	0	1	504.2936	1510.866	3.51	0.00177	26.67206
P02766	0	1	504.292	1510.862	0.3	0.00015	59.20649
P02766	2	0.9375	790.4258	3158.681	5.94	0.00469	38.37012
P02766	0	0.6744	769.8673	1538.727	1.87	0.00144	15.93497
P02766	0	1	378.4714	1510.864	1.81	0.00068	0
P02766	0	0.9231	378.4715	1510.864	2.13	0.00081	0
P02766	0	1	649.5842	2595.315	2.94	0.00191	37.9038
P02766	1	0.9565	926.5031	2777.495	3.48	0.00322	0
P02766	1	0.75	935.5219	2804.551	2.62	0.00245	3.852736
P02766	1	0.8889	551.0872	2751.407	-0.48	-0.00027	48.61059
P02766	0	1	649.5834	2595.312	1.81	0.00118	61.17326
P02760	1	0.9783	630.6621	1889.972	4.08	0.00257	6.066453
P02760	0	1	607.3416	1213.676	3.03	0.00184	4.711957
P02760	0	0.8929	607.3426	1213.678	4.74	0.00288	56.49397
P02760	1	1	630.6607	1889.968	1.95	0.00123	0
P02760	0	0.9074	607.342	1213.677	3.73	0.00226	13.06826

P02760	0	1	453.2635	1357.776	1.25	0.00057	5.807553
P02760	0	0.931	679.3909	1357.774	0.18	0.00012	3.238925
P02760	0	0.881	453.2635	1357.776	1.18	0.00054	13.44236
P02760	0	0.9804	453.2643	1357.778	3.07	0.00139	41.72086
P02760	0	0.9882	717.0421	2149.112	1.78	0.00127	6.641734
P02760	0	1	843.4295	2528.274	1.4	0.00118	4.289174
P02760	0	1	1264.64	2528.273	1.21	0.00154	0
P02760	0	1	722.3751	2165.111	3.66	0.00264	16.20148
P02760	0	0.9787	722.3735	2165.106	1.47	0.00106	2.092609
P02760	0	1	926.9303	1852.853	1.6	0.00148	0
P02760	0	1	618.29	1852.856	2.81	0.00174	0
P02760	0	1	1032.533	2064.059	1.9	0.00196	0
P02760	0	1	688.6907	2064.057	1.1	0.00076	4.418777
P02760	0	1	688.6912	2064.059	1.9	0.0013	1.572001
P02760	0	0.8421	417.2478	1249.729	2.6	0.00108	17.67913
P02760	0	0.9672	417.2473	1249.727	1.57	0.00066	14.3636
P02760	0	0.9206	625.3677	1249.728	2.15	0.00134	6.68641
P02760	0	0.9804	417.2475	1249.728	1.87	0.00078	21.90099
P02760	0	1	926.9312	1852.855	2.52	0.00234	4.79497
P02760	0	1	926.9294	1852.852	0.68	0.00063	3.448659
P02760	0	1	618.2897	1852.854	2.22	0.00137	4.206442
P02760	0	1	926.9309	1852.854	2.19	0.00203	3.418346
P02760	0	1	618.2911	1852.859	4.59	0.00284	3.601605
P02760	0	0.9722	666.358	1997.059	1.83	0.00122	3.832776
P02760	0	1	926.9312	1852.855	2.52	0.00234	8.143332
P02760	0	1	926.9301	1852.853	1.4	0.0013	3.587534
P02760	0	1	618.2894	1852.854	1.82	0.00113	4.871056
P02760	0	1	926.9308	1852.854	2.13	0.00197	2.679948
P02760	0	1	618.2902	1852.856	3.11	0.00192	8.575316
P02760	1	0.9787	630.6605	1889.967	1.56	0.00099	3.411857
P02760	1	0.878	473.2473	1889.967	1.88	0.00089	19.6838
P02760	1	1	630.6614	1889.97	3.11	0.00196	11.61899
P02760	0	1	926.9285	1852.85	-0.31	-0.00029	3.491387
P02760	0	1	618.2887	1852.852	0.64	0.00039	4.156075
P02760	0	1	854.8789	1708.751	1.31	0.00112	36.55689
P02760	1	1	841.7178	2523.139	2.02	0.0017	28.27684
P02760	1	1	631.5403	2523.14	2.26	0.00143	7.777102
P02760	0	1	809.3887	1617.77	2.07	0.00167	6.920965
P02760	0	1	848.9877	1696.968	0.94	0.0008	5.705739
P02760	0	0.8491	566.327	1696.966	-0.08	-0.00004	0
P02760	0	1	417.2467	1249.726	0.11	0.00004	47.01463
P02760	0	0.9286	607.3422	1213.677	4.03	0.00245	38.22062
P02760	0	0.9375	417.2466	1249.725	-0.33	-0.00014	24.35148
P02760	0	1	618.2913	1852.859	4.79	0.00296	0
P02760	0	0.9615	717.0418	2149.111	1.27	0.00091	47.31755
P02760	0	0.7	666.3572	1997.057	0.64	0.00043	67.65759
P02760	0	0.88	717.0411	2149.109	0.33	0.00024	44.92117
P02760	0	1	776.9355	1552.864	-0.46	-0.00036	8.139161
P02760	0	0.9667	618.2903	1852.856	3.21	0.00198	53.37071
P02760	0	1	539.9277	1617.768	1	0.00054	44.46853
P02760	1	0.9737	630.6621	1889.972	4.08	0.00257	14.84517
P02760	0	0.8372	607.3412	1213.675	2.42	0.00147	0

P02760	0	0.9091	583.3206	1165.634	0.52	0.0003	3.327689
P02760	1	1	841.7172	2523.137	1.23	0.00103	0
P02760	0	0.9643	926.931	1852.855	2.39	0.00222	7.145191
P02760	0	0.8696	625.3698	1249.732	5.37	0.00335	0
P02760	0	0.8636	679.3912	1357.775	0.72	0.00049	19.13122
P02760	0	0.8571	717.0406	2149.107	-0.35	-0.00025	14.13644
P02760	0	1	640.6572	1919.957	1.98	0.00127	3.6789
P02760	0	0.4762	666.3582	1997.06	2.2	0.00146	0
P02760	0	1	688.6912	2064.059	1.9	0.0013	3.363958
P02760	0	1	632.8254	2528.28	3.67	0.00232	4.224435
P02760	0	0.8095	679.3919	1357.776	1.62	0.0011	20.50048
P02760	0	0.8421	516.77	2064.058	1.48	0.00076	0
P02760	0	1	960.4819	1919.957	1.8	0.00173	0
P07357	0	0.9464	624.8417	1248.676	1.79	0.00112	2.9304
P07357	0	1	534.6154	1601.832	0.57	0.0003	5.95897
P07357	0	0.766	569.3078	1137.608	2.55	0.00145	4.53705
P07357	0	1	632.8389	1264.671	1.45	0.00092	4.179696
P07357	0	0.9756	534.6151	1601.831	-0.12	-0.00006	5.550167
P07357	0	0.619	447.947	1341.827	0.79	0.00035	13.31176
P07357	0	1	932.7454	2796.222	2.62	0.00245	0
P07357	0	1	781.7493	2343.233	2	0.00156	11.48596
P07357	0	1	781.7482	2343.23	0.51	0.0004	5.770126
P07357	0	1	742.3592	2225.063	-0.18	-0.00013	41.80221
P07357	0	1	742.361	2225.069	2.29	0.0017	7.027274
P07357	0	1	553.2611	1657.769	1.28	0.00071	13.85172
P07357	0	1	742.3605	2225.067	1.63	0.00121	18.31211
P07357	0	1	645.8277	1290.648	1.96	0.00126	21.30411
P07357	0	0.9655	670.3465	2009.025	1.88	0.00126	15.34272
P07357	0	0.6176	447.5982	1340.78	1.76	0.00079	14.16252
P07357	0	0.9804	486.5822	1457.732	2.37	0.00115	13.85847
P07357	0	1	699.8108	2796.221	2.49	0.00174	33.06617
P07357	0	0.7941	416.8966	1248.675	1.22	0.00051	13.37835
P07357	0	0.7941	379.874	1137.608	1.84	0.0007	16.89205
P07357	0	0.3514	671.4175	1341.828	1.75	0.00118	10.29956
P07357	0	0.7045	559.8325	1118.658	1.43	0.0008	0
P07357	0	0.7419	447.5984	1340.781	2.31	0.00103	29.75507
P07357	0	0.8824	742.3613	2225.069	2.62	0.00194	11.68507
P07357	0	1	694.3262	2080.964	1.25	0.00087	0
P07357	0	0.9	503.0112	2009.023	0.9	0.00045	13.56808
P80108	0	1	522.2871	1564.847	1.05	0.00055	3.513576
P80108	0	1	572.3293	1714.973	0.72	0.00041	52.53463
P80108	0	0.8485	528.9601	1584.866	0.07	0.00003	0
P80108	0	0.8909	600.6665	1799.985	3.78	0.00227	4.061333
P80108	0	0.5349	460.2595	1378.764	2.12	0.00098	7.805146
P80108	0	1	851.1769	3401.686	2.02	0.00171	14.04497
P80108	0	0.8358	670.853	1340.699	1.34	0.0009	20.02559
P80108	0	1	416.5677	1247.689	1.73	0.00072	11.38222
P80108	0	1	512.2664	1534.785	2.17	0.00111	10.16142
P80108	0	0.6562	624.3483	1247.689	2.25	0.0014	20.44608
P80108	0	0.6667	689.8848	1378.762	0.98	0.00068	4.286338
P80108	0	1	631.6928	1893.064	3.46	0.00218	13.07485
P80108	0	0.8846	732.3956	1463.784	4.58	0.00335	26.45258

P80108	0	1	535.287	1603.846	3.51	0.00188	9.654145
P80108	0	1	767.8955	1534.784	1.54	0.00118	43.34811
P80108	0	1	947.0353	1893.063	3.31	0.00313	55.70242
Q96PD5	0	0.9667	620.3179	1858.939	3.36	0.00208	18.83502
Q96PD5	0	0.9706	465.4898	1858.937	2.3	0.00107	4.512564
Q96PD5	0	1	520.2589	2078.014	1.4	0.00073	25.39145
Q96PD5	0	0.9697	693.3425	2078.013	1.15	0.00079	0
Q96PD5	0	0.9677	424.75	1695.978	0.4	0.00017	0
Q96PD5	0	1	565.9998	1695.985	4.26	0.00241	33.23626
Q96PD5	0	0.9667	424.7502	1695.979	0.91	0.00039	4.09347
Q96PD5	0	1	565.9989	1695.982	2.75	0.00156	5.156072
Q96PD5	0	1	746.8599	1492.713	1.69	0.00126	12.27376
Q96PD5	0	1	818.9115	1636.816	2.2	0.0018	15.02804
Q96PD5	0	1	630.3232	1888.955	2.94	0.00185	6.415331
Q96PD5	0	1	630.3231	1888.955	2.75	0.00173	2.169342
Q96PD5	0	1	823.4283	1645.849	2.22	0.00182	27.42413
Q96PD5	0	0.9348	549.2871	1645.847	0.61	0.00034	8.716537
Q96PD5	0	0.8039	549.2878	1645.849	1.95	0.00107	9.568415
Q96PD5	0	0.7414	388.206	1162.603	2.29	0.00089	4.936467
Q96PD5	0	1	800.6782	3199.691	0.86	0.00069	4.835224
Q96PD5	0	1	1067.234	3199.689	0.08	0.00009	0
Q96PD5	0	1	839.1868	3353.725	1.4	0.00117	1.40013
Q96PD5	0	1	1118.582	3353.73	2.77	0.0031	2.055639
Q96PD5	0	1	1009.218	3025.64	2.39	0.00241	3.646098
Q96PD5	0	1	891.0826	2671.233	1.75	0.00156	2.705177
Q96PD5	0	1	668.564	2671.234	2.09	0.00139	4.881982
Q96PD5	0	0.9792	843.0491	2527.133	2.38	0.00201	24.23079
Q96PD5	0	0.8846	549.2871	1645.847	0.5	0.00027	50.04065
Q96PD5	0	1	472.9939	1888.954	2.19	0.00104	10.7794
Q96PD5	0	0.7826	517.9647	1551.88	2.7	0.0014	6.63875
Q96PD5	0	0.6316	611.8694	1222.731	2.42	0.00148	6.246268
Q96PD5	0	0.1905	559.282	2234.106	1.55	0.00086	14.13651
Q96PD5	0	1	843.0472	2527.127	0.14	0.00012	3.754347
Q96PD5	0	0.9216	539.8176	1078.628	1.28	0.00069	3.471161
Q96PD5	0	0.8636	745.3749	2234.11	3.41	0.00254	26.89815
Q96PD5	0	1	693.3436	2078.016	2.65	0.00183	7.700942
Q96PD5	0	1	472.9935	1888.952	1.42	0.00067	3.604031
Q96PD5	0	0.8182	630.3242	1888.958	4.49	0.00283	30.50623
Q96PD5	0	0.8571	745.3766	2234.115	5.62	0.00419	5.958794
Q96PD5	0	0.6944	611.87	1222.733	3.42	0.00209	7.54864
Q96PD5	0	1	388.7247	1551.877	1	0.00039	4.440742
Q96PD5	0	0.5714	465.489	1858.934	0.73	0.00034	10.89546
P02750	0	0.84	662.3916	1323.776	0.5	0.00033	0
P02750	0	0.8627	662.3919	1323.776	0.87	0.00058	3.339704
P02750	1	1	471.0467	1881.165	0.2	0.00009	64.66068
P02750	1	1	392.2308	1565.902	1.45	0.00057	36.68343
P02750	0	0.8305	594.8835	1188.76	3.7	0.0022	26.769
P02750	0	0.9355	648.8622	1296.717	2.43	0.00158	4.41989
P02750	0	0.925	432.9106	1296.717	2.42	0.00105	9.370338
P02750	0	1	432.9104	1296.717	1.93	0.00083	38.42506
P02750	0	0.7867	495.2806	989.5539	1.26	0.00063	13.27008
P02750	0	0.551	378.5571	1133.657	1.67	0.00063	0

P02750	0	0.8605	567.3326	1133.658	2.76	0.00157	11.63438
P02750	0	1	1315.111	2629.215	1.69	0.00223	0
P02750	0	0.9804	877.0761	2629.214	1.09	0.00095	3.347218
P02750	0	1	1091.099	2181.191	1.21	0.00132	4.848787
P02750	0	1	727.7352	2181.191	1.29	0.00094	1.937458
P02750	0	0.9846	546.3091	2182.215	1.71	0.00093	7.362779
P02750	0	0.9474	1091.613	2182.219	3.45	0.00377	15.58062
P02750	0	1	728.0766	2182.215	1.94	0.00141	5.037887
P02750	0	1	680.0424	2038.113	1.84	0.00125	6.470344
P02750	0	0.6852	560.3084	1119.61	2.24	0.00125	6.07441
P02750	0	1	876.8477	2628.529	2.72	0.00238	19.44095
P02750	0	0.7436	441.9309	1323.778	2.06	0.00091	8.159548
P02750	0	0.8293	662.3955	1323.784	6.31	0.00418	13.92442
P02750	0	1	432.9105	1296.717	2.28	0.00099	15.79766
P02750	0	0.85	396.9238	1188.757	1.27	0.0005	13.10366
P02750	0	0.7647	662.3932	1323.779	2.9	0.00192	4.093449
P02750	0	0.9318	648.8626	1296.718	3	0.00194	0
P02750	1	1	532.9887	1596.951	3.12	0.00166	24.50469
P02750	1	0.2963	484.9537	1452.846	1.46	0.00071	3.553243
P02750	0	0.8049	576.8107	1152.614	1.9	0.00109	0
P02750	0	0.8966	628.8677	1256.728	3.15	0.00198	3.516373
P02750	0	1	419.5797	1256.725	0.44	0.00019	51.74453
P02750	1	1	484.9531	1452.845	0.2	0.0001	35.53529
P10909	0	0.8889	455.2685	1363.791	2.42	0.0011	10.12831
P10909	0	0.9412	455.2676	1363.788	0.34	0.00015	8.700597
P10909	0	0.9412	455.268	1363.789	1.21	0.00055	9.94537
P10909	0	0.91	703.4126	1405.818	2.75	0.00193	11.692
P10909	1	0.7727	560.0098	1678.015	2.18	0.00122	11.35592
P10909	0	0.9706	673.3716	2018.1	3.75	0.00252	45.11676
P10909	0	0.875	703.4116	1405.816	1.27	0.00089	17.49289
P10909	0	1	504.9937	2016.953	1.55	0.00078	21.30131
P10909	0	1	504.9937	2016.953	1.43	0.00072	11.56141
P10909	0	1	504.9938	2016.953	1.61	0.00081	17.09357
P10909	0	0.8947	513.2715	1537.8	1.33	0.00068	3.481364
P10909	0	0.9881	769.403	1537.799	0.51	0.00039	3.272267
P10909	0	0.6939	478.252	1432.741	0.98	0.00047	31.43902
P10909	0	0.7541	716.8752	1432.743	2.16	0.00155	8.44053
P10909	0	0.7895	478.2519	1432.741	0.72	0.00034	2.060853
P10909	0	0.9403	631.3608	1261.714	1.91	0.00121	38.42494
P10909	0	1	868.1334	2602.386	1.66	0.00144	5.16279
P10909	0	0.8148	609.8275	1218.648	2.45	0.00149	3.712485
P10909	0	0.8919	703.4115	1405.816	1.18	0.00083	5.882872
P10909	0	0.9123	621.8389	1242.671	5.12	0.00318	5.045342
P10909	0	0.697	469.2768	1405.816	1.2	0.00056	10.34423
P10909	0	1	684.3502	2051.036	2.04	0.0014	29.53649
P10909	0	0.7353	682.3986	1363.79	1.55	0.00106	10.072
P10909	0	0.8929	684.3504	2051.037	2.4	0.00164	0
P10909	0	0.6111	407.2342	1219.688	1.94	0.00079	36.75432
P10909	1	0.8696	420.2589	1678.014	1.63	0.00068	0
P10909	0	0.9	697.3527	1393.698	1.62	0.00113	4.176377
P10909	0	0.7097	682.3987	1363.79	1.82	0.00124	5.961411
P10909	1	0.9	530.2855	1588.842	0.57	0.0003	9.017067

P10909	0	0.9375	684.351	2051.038	3.29	0.00225	56.44411
P10909	0	0.6923	537.7752	1074.543	0.52	0.00028	34.07301
P10909	1	0.3571	447.2828	1339.834	2.05	0.00092	32.02862
P10909	0	0.8864	697.3503	1393.693	-1.71	-0.00119	20.84895
P10909	0	1	769.4052	1537.803	3.37	0.00259	66.33627
P10909	0	0.7586	769.4052	1537.803	3.45	0.00265	8.770552
P10909	0	0.8824	521.2825	2602.383	0.71	0.00037	27.60143
P22792	1	0.8889	385.9957	1540.961	2.92	0.00113	0
P22792	0	1	649.6686	1946.991	3.16	0.00205	20.21987
P22792	0	1	649.6679	1946.989	2.03	0.00132	12.15317
P22792	0	0.8333	414.257	1240.756	2.35	0.00097	0
P22792	0	0.931	462.2902	1384.856	0.33	0.00015	29.22101
P22792	0	0.9348	462.2908	1384.858	1.72	0.0008	11.61365
P22792	0	1	896.7375	2688.198	1.74	0.00156	1.324496
P22792	0	1	716.7336	2148.186	4.93	0.00353	25.80158
P22792	0	1	630.6971	1890.077	4.32	0.00272	37.39886
P22792	0	1	860.4635	2579.376	3.35	0.00288	9.340974
P22792	0	1	834.232	3333.906	1.12	0.00093	5.295148
P22792	0	0.9302	716.8699	1432.733	1.74	0.00124	13.93596
P22792	0	0.6727	595.885	1190.763	2.34	0.00139	20.07149
P22792	0	0.7551	448.2706	1342.797	2.97	0.00133	15.02739
P22792	0	0.3333	567.3383	1133.669	3.21	0.00182	7.596039
P22792	0	0.6765	692.9318	1384.856	0.65	0.00045	0
P22792	0	0.9394	478.249	1432.733	1.69	0.0008	11.58672
P22792	0	0.375	516.8127	1032.618	1.2	0.00062	22.07904
P22792	0	0.2439	495.2862	989.5652	1.65	0.00082	8.911083
P22792	0	0.3913	671.902	1342.797	2.55	0.00171	36.68053
P22792	0	0.913	692.9322	1384.857	1.18	0.00082	12.48927
Q2TV78; P2	0	1	536.0283	2141.091	-0.32	-0.00017	41.41212
Q2TV78; P2	0	0.92	429.0256	2141.099	3.35	0.00143	21.05733
Q2TV78; P2	0	1	536.0294	2141.096	1.73	0.00092	41.93961
P26927	0	0.9184	602.9983	1806.98	3.98	0.0024	6.463499
P26927	0	0.9167	477.9177	1431.739	1.7	0.00081	28.13566
P26927	0	1	730.708	2190.109	2.59	0.00189	26.10289
Q2TV78; P2	0	1	832.9111	3328.623	2.7	0.00225	30.29306
P26927	0	1	740.3362	2218.994	-0.1	-0.00007	22.37329
P26927	0	1	740.3339	2218.987	-3.32	-0.00245	23.03736
P26927	0	0.6949	593.0054	1777.002	3.57	0.00211	23.43148
Q2TV78; P2	0	0.8571	580.8331	1160.659	3.83	0.00222	2.265286
Q2TV78; P2	0	0.8125	580.8327	1160.658	3.2	0.00186	2.265191
Q2TV78; P2	0	0.8148	506.6295	1517.874	1.42	0.00072	7.35443
P26927	0	0.6471	863.0938	2587.267	3.11	0.00268	36.06442
P26927	0	1	448.2235	1342.656	1.42	0.00064	31.82818
Q2TV78; P2	0	1	822.382	2465.131	1.67	0.00137	3.663897
P26927	0	0.4839	586.8052	1172.603	2.49	0.00146	14.9315
P25311	0	0.825	507.9278	1521.769	2.58	0.00131	18.61318
P25311	0	0.6753	707.3818	1413.756	1.77	0.00125	7.541047
P25311	0	0.8261	643.0243	1927.058	7.14	0.00459	17.22633
P25311	0	0.9875	691.0548	2071.15	1.57	0.00108	5.381068
P25311	0	0.88	1036.079	2071.151	2.2	0.00227	0
P25311	0	1	705.3531	1409.699	1.13	0.00079	8.792508
P25311	0	1	532.6008	1595.788	1.37	0.00073	11.19711

P25311	0	1	798.3971	1595.787	0.84	0.00067	3.516333
P25311	0	1	849.777	2547.317	2.09	0.00178	3.683234
P25311	0	0.8714	566.3196	1696.944	2.58	0.00146	11.58587
P25311	0	0.9836	516.78	2064.098	0.81	0.00042	10.47461
P25311	0	1	688.705	2064.101	2	0.00137	9.214712
P25311	1	0.8837	547.6364	1640.895	1.64	0.0009	19.81544
P25311	0	1	1274.165	2547.323	4.64	0.00591	0
P25311	1	0.9143	547.6362	1640.894	1.31	0.00072	17.38868
P25311	1	1	607.6701	1820.996	3.31	0.00201	7.223686
P25311	0	0.8077	636.3405	1271.674	2.02	0.00128	3.727094
P25311	0	0.7593	636.3393	1271.671	0.1	0.00006	3.463441
P25311	0	0.8727	638.821	1276.635	2.15	0.00138	0.63061
P25311	0	1	726.3463	1451.685	1.27	0.00092	0
P25311	0	0.8462	564.29	1127.573	3.26	0.00184	0
P25311	0	0.7955	424.5623	1271.672	0.88	0.00037	4.625057
P25311	0	0.9318	608.8281	1216.649	3.1	0.00189	7.277424
P25311	0	0.8571	710.8725	1420.738	2.53	0.0018	5.626595
P25311	0	0.9394	470.5708	1409.698	0.45	0.00021	10.96612
P25311	1	0.9583	607.6694	1820.994	2.11	0.00128	3.749967
P25311	1	1	911	1820.993	1.64	0.0015	0
P25311	0	0.9756	475.9036	1425.696	2.87	0.00137	8.209058
P25311	0	0.6327	635.3309	1269.654	2.18	0.00138	6.597437
P25311	0	1	535.7977	1070.588	0.72	0.00038	8.109297
P25311	0	0.7368	636.3399	1271.673	1.06	0.00067	1.980797
P25311	0	0.9773	535.7981	1070.589	1.52	0.00081	14.65717
P25311	0	1	507.9269	1521.766	0.78	0.00039	13.00636
P25311	0	0.925	608.8265	1216.646	0.49	0.0003	14.90573
P25311	0	0.9	564.2892	1127.571	1.74	0.00098	7.343258
P25311	0	0.8333	477.2551	1429.751	1.5	0.00072	0
P25311	0	1	713.3513	1425.695	2.2	0.00157	6.390464
P25311	1	0.96	456.0031	1820.99	0.36	0.00016	62.4319
P25311	1	0.9032	499.6016	1496.79	0.2	0.0001	0
P25311	0	0.9737	535.7985	1070.59	2.2	0.00118	100
P25311	0	0.8889	518.5425	2071.148	0.71	0.00037	3.767303
P25311	0	0.6296	707.3825	1413.758	2.72	0.00193	0
P25311	0	0.8421	474.2499	1420.735	0.74	0.00035	0
P25311	0	0.3529	470.5714	1409.7	1.62	0.00076	64.80545
P25311	0	1	424.5623	1271.672	0.95	0.0004	76.60391
P51884	0	0.9014	757.4121	1513.817	1.83	0.00138	7.563082
P51884	0	0.8909	505.277	1513.817	1.66	0.00084	10.19699
P51884	0	1	652.3176	1954.938	0.55	0.00036	2.805971
P51884	0	1	652.3185	1954.941	2.05	0.00134	3.931847
P51884	0	1	977.9741	1954.941	1.94	0.00189	4.196792
P51884	0	1	652.3185	1954.941	1.96	0.00128	4.272217
P51884	0	1	977.9742	1954.941	2.06	0.00201	3.943734
P51884	0	1	652.3184	1954.941	1.77	0.00115	6.473813
P51884	0	1	652.3188	1954.942	2.43	0.00158	0
P51884	0	0.8772	942.1609	2824.468	2.62	0.00247	7.86319
P51884	0	0.7903	942.1602	2824.466	1.91	0.0018	3.031717
P51884	0	0.7667	633.3909	1265.774	2.94	0.00186	6.457979
P51884	0	0.9091	529.2984	1585.881	1.18	0.00062	47.50648
P51884	1	0.8	537.655	1610.95	1.61	0.00087	31.72026

P51884	1	1	537.6552	1610.951	1.95	0.00105	19.27387
P51884	1	0.92	403.4937	1610.953	3.35	0.00135	14.83369
P51884	1	1	537.6551	1610.951	1.84	0.00099	18.67451
P51884	1	0.8333	537.6554	1610.952	2.41	0.00129	23.79662
P51884	0	0.8864	505.2679	1513.789	2.72	0.00137	43.97705
P51884	0	0.5833	438.269	1312.792	1.75	0.00077	5.825379
P51884	0	0.6949	512.7836	1024.56	2.36	0.00121	5.954332
P51884	0	0.9057	656.9006	1312.794	2.97	0.00195	5.803599
P51884	0	0.5926	438.2694	1312.794	2.73	0.00119	9.033003
P51884	0	0.6341	438.2698	1312.795	3.64	0.00159	7.027594
P51884	0	0.8764	942.1601	2824.466	1.78	0.00168	4.770613
P51884	0	0.6757	906.9854	1812.963	1.21	0.0011	14.09965
P51884	0	0.9167	604.9939	1812.967	3.26	0.00197	6.498007
P51884	0	0.7241	505.2677	1513.789	2.48	0.00125	45.06312
P51884	0	0.8148	505.2676	1513.788	2.24	0.00113	10.88825
P51884	0	0.7872	505.2674	1513.788	1.82	0.00092	7.83718
P51884	0	0.8667	505.2675	1513.788	1.94	0.00098	10.3331
P51884	0	0.8772	505.2664	1513.785	-0.24	-0.00012	10.32465
P51884	0	0.8438	757.3976	1513.788	1.99	0.0015	63.60633
P51884	0	1	749.1072	2245.307	1.15	0.00086	6.281271
P51884	0	1	505.2677	1513.789	2.48	0.00125	18.5999
P51884	0	0.8571	505.2673	1513.787	1.64	0.00083	8.930131
P51884	0	1	1123.157	2245.307	0.94	0.00105	0
P51884	0	1	529.2991	1585.883	2.56	0.00136	9.4819
P51884	0	1	529.2989	1585.882	2.1	0.00111	7.240224
P51884	0	1	613.3396	1225.672	4.29	0.00263	55.86316
P51884	0	1	977.9741	1954.941	2	0.00195	2.231015
P51884	0	0.8919	604.9941	1812.968	3.56	0.00215	29.75869
P51884	0	0.9211	906.9858	1812.964	1.75	0.00159	28.95293
P51884	1	0.9259	403.4923	1610.948	-0.14	-0.00006	0
P51884	0	0.5938	584.835	1168.663	2.79	0.00163	0
P51884	0	0.8421	757.399	1513.791	3.84	0.00291	0
P51884	0	0.9412	793.4443	1585.881	1.65	0.00131	27.8183
P51884	0	0.8364	561.3401	1121.673	3.77	0.00211	15.32037
P51884	1	0.2292	489.6211	1466.849	2.07	0.00101	59.09682
P51884	0	0.9	422.5966	1265.775	3.46	0.00146	6.912282
P51884	1	0.9167	805.9799	1610.952	2.91	0.00234	10.30568
P51884	1	0.9167	403.4935	1610.952	2.74	0.0011	8.790588
P51884	0	0.8	633.3897	1265.772	1.11	0.0007	35.26887
P51884	0	0.6842	757.3984	1513.79	3.12	0.00236	6.541358
P51884	0	0.75	422.5967	1265.775	3.68	0.00155	26.25572
P51884	0	0.65	505.2669	1513.786	0.73	0.00037	0
P51884	1	0.6875	442.2594	1324.764	2.29	0.00101	63.33822
P51884	0	0.8846	834.9357	1668.864	2.94	0.00245	10.1926
P51884	0	0.7879	584.8356	1168.664	3.84	0.00224	20.97486
P51884	0	1	457.2332	1369.685	1.53	0.0007	1.778
P51884	0	0.9375	656.9	1312.793	2.04	0.00134	11.11731
P51884	0	1	584.8503	1168.693	4.6	0.00269	8.064291
P51884	0	0.5714	481.2645	1441.779	1.47	0.00071	29.65026
P51884	0	0.6471	502.2816	1003.556	1.79	0.0009	3.518802
P51884	0	0.6	706.8721	2824.466	2.04	0.00144	23.38015
P51884	0	1	604.9936	1812.966	2.76	0.00167	25.9606

P20742	0	0.973	509.2712	1525.799	4.4	0.00224	28.06613
P01023; P2	0	0.7586	624.3599	1247.712	3.91	0.00244	0
P01023; P2	0	1	622.3833	1865.135	3.18	0.00198	15.8727
P01023; P2	0	1	467.0389	1865.134	2.22	0.00104	53.79703
P20742	0	0.92	692.891	1384.775	3.03	0.0021	4.056625
P01023; P2	0	0.6061	653.9038	1306.8	2.34	0.00153	5.954266
P20742	0	0.6458	705.9096	1410.812	2.62	0.00185	48.15599
P01023; P2	0	0.9899	743.0449	2227.12	2.48	0.00184	2.667964
P01023; P2	0	1	1114.065	2227.122	3.53	0.00393	4.164521
P01023; P2	0	1	1114.064	2227.12	2.54	0.00283	0
P01023; P2	0	1	617.0515	1849.14	2.86	0.00177	16.97725
P01023; P2	0	1	743.0453	2227.121	3.06	0.00227	3.090556
P01023; P2	0	0.8621	463.0398	1849.137	1.54	0.00071	11.29244
P20742	0	0.7959	597.8378	1194.668	6.31	0.00377	0
P01023; P2	0	1	1095.084	2189.16	0.65	0.00071	0
P01023; P2	0	1	730.3897	2189.154	-1.9	-0.00138	15.28631
P01023; P2	0	1	1114.064	2227.121	2.98	0.00332	4.288647
P20742	0	1	850.7846	2550.339	2.77	0.00235	36.17917
P01023; P2	0	0.9655	464.609	1391.812	2.04	0.00094	50.13607
P20742	0	1	509.9017	1527.69	-2.61	-0.00133	17.29596
P01023; P2	0	0.7105	436.2716	1306.8	2.21	0.00096	9.604411
P01023; P2	0	0.6552	464.6081	1391.81	0	0	36.77136
P20742	0	0.8542	464.6187	1391.842	4.95	0.0023	38.44375
P01023; P2	0	1	617.0518	1849.141	3.36	0.00207	42.06173
P20742	0	1	487.9373	1461.797	2.98	0.00145	0
P01023; P2	0	0.8	464.6089	1391.812	1.77	0.00082	10.83583
P20742	0	0.6222	610.3637	1219.72	5.35	0.00326	17.69543
P20742	0	1	376.7014	1503.784	2.87	0.00108	13.63139
P01023; P2	0	0.5714	464.6095	1391.814	3.02	0.0014	67.77657
P01023; P2	0	0.75	436.2735	1306.806	6.63	0.00289	20.89936
P20742	0	0.3	567.6388	1700.902	3.8	0.00216	18.50627
P20742	0	1	553.2937	1657.867	5.76	0.00318	7.596171
P20742	0	0.8788	508.2888	1522.852	3.32	0.00169	28.32264
P01023; P2	0	0.7692	653.9042	1306.801	2.9	0.00189	44.47319
P20742	0	0.7692	421.2567	1261.756	3.83	0.00161	3.963704
P05543	0	0.9143	516.9409	1548.808	2.14	0.0011	12.70376
P05543	0	0.913	774.908	1548.809	2.42	0.00188	5.665292
P05543	0	0.8684	516.9407	1548.808	1.78	0.00092	25.64139
P05543	0	1	1063.881	3189.629	7.29	0.00775	0
P05543	0	0.9861	693.3495	1385.692	0.03	0.00002	0
P05543	0	0.9048	765.4028	1529.798	2.97	0.00228	6.586249
P05543	0	0.9348	510.6041	1529.798	2.69	0.00137	13.30512
P05543	0	0.9559	591.2883	1771.85	3.38	0.002	8.918624
P05543	0	0.7778	630.0282	1888.07	1.62	0.00102	7.909355
P05543	0	1	944.5399	1888.072	2.89	0.00273	29.22699
P05543	0	0.7917	674.8987	1348.79	2.59	0.00174	12.4781
P05543	0	0.9412	687.4433	1373.879	2.51	0.00173	35.87976
P05543	0	1	772.462	3086.826	3.13	0.00241	0
P05543	0	0.6667	674.8991	1348.791	3.13	0.00211	3.048711
P05543	1	0.5455	460.9299	1380.775	1.97	0.00091	10.72439
P05543	0	1	385.5767	1154.716	1.78	0.00069	9.842741
P05543	0	0.34	533.3506	1065.694	2.69	0.00143	13.70992

P05543	0	0.86	540.7895	1080.572	2.18	0.00118	0
P05543	0	1	413.6158	1238.833	1.7	0.0007	16.36514
P05543	0	0.3571	533.3497	1065.692	0.97	0.00052	14.33646
P05543	0	1	385.5773	1154.717	3.29	0.00127	26.64528
P05543	1	0.8667	527.0734	2105.272	1.16	0.00061	51.66877
P05543	0	1	577.8622	1154.717	3.17	0.00183	0
Q08380	0	1	1067.845	3201.52	2.87	0.00306	5.544907
Q08380	1	1	466.2779	1396.819	2.91	0.00136	63.07315
Q08380	1	0.9756	473.9653	1419.881	2.77	0.00131	10.32903
Q08380	0	0.8	579.639	1736.902	5.09	0.00295	0
Q08380	0	0.9831	868.9533	1736.899	3.27	0.00284	31.60339
Q08380	0	0.6591	548.6667	1643.986	2.02	0.00111	13.19628
Q08380	0	1	1298.607	2596.206	0.88	0.00114	0
Q08380	0	1	866.0728	2596.204	-0.14	-0.00012	4.024243
Q08380	0	0.8542	602.3149	1203.622	2.21	0.00133	4.190376
Q08380	0	1	769.38	2306.125	1.95	0.0015	35.94759
Q08380	0	0.8387	538.959	1614.862	2.9	0.00156	12.00686
Q08380	1	0.9487	698.9114	1396.815	0.3	0.00021	2.752107
Q08380	0	0.6571	490.9236	1470.756	0.44	0.00021	0
Q08380	0	0.5556	476.7713	952.5354	2.25	0.00107	3.449421
Q08380	1	1	473.9648	1419.88	1.87	0.00088	50.80263
Q08380	0	0.7368	421.9311	1263.779	1.91	0.00081	30.74195
Q08380	0	0.9091	675.8331	1350.659	2.21	0.00149	3.435979
Q08380	0	0.8929	801.1342	3201.515	1.36	0.00109	3.802016
Q08380	0	0.6765	632.3934	1263.78	2.67	0.00169	39.16169
Q08380	0	1	391.8837	1173.636	1.74	0.00068	74.52272
Q08380	0	0.48	548.6675	1643.988	3.47	0.0019	4.852496
Q08380	0	0.7812	562.8145	1124.622	3.2	0.0018	0
Q16610	0	1	727.6828	2181.034	1.59	0.00115	1.635897
Q16610	0	1	580.9659	1740.883	4.42	0.00256	23.02398
Q16610	0	0.963	630.7045	1890.099	2.64	0.00166	29.43839
Q16610	0	1	1021.471	3062.399	2.07	0.00212	0
Q16610	0	1	473.2304	1417.676	4.74	0.00224	68.70176
Q16610	0	0.9839	725.387	1449.767	1.8	0.0013	8.155114
Q16610	1	1	432.5599	1295.665	2.92	0.00126	24.70135
Q16610	1	0.8333	432.5595	1295.664	2.14	0.00092	0
Q16610	0	1	535.962	1605.871	1.72	0.00092	40.23181
Q16610	0	0.7377	702.8589	1404.711	2.38	0.00167	29.03466
Q16610	0	0.54	633.336	1265.665	2.48	0.00157	22.74282
Q16610	0	1	727.682	2181.031	0.41	0.0003	15.68935
Q16610	0	0.9655	630.7049	1890.1	3.32	0.00209	26.08136
Q16610	0	0.9062	671.4158	1341.824	1.53	0.00103	22.6251
Q16610	0	1	422.2026	1264.593	1.81	0.00076	0
Q16610	0	1	632.8302	1264.653	4.35	0.00275	64.93069
Q16610	0	1	422.2018	1264.591	0	0	5.274674
Q16610	0	0.4545	580.3445	1159.682	1.2	0.00069	0
Q16610	0	1	468.9082	1404.71	2	0.00094	47.07476
Q16610	0	0.8947	483.9256	1449.762	-1.18	-0.00057	37.13136
Q16610	0	1	580.9645	1740.879	2.1	0.00122	25.82335
P08519	0	0.9111	532.6395	1595.904	0.3	0.00016	7.990748
P08519	0	1	730.6848	2190.04	-0.33	-0.00024	17.81482
P08519	1	1	559.9907	1677.958	1.15	0.00064	21.83714

P08519	0	0.9138	620.7087	1860.112	1.73	0.00107	1.917884
P08519	0	0.9785	930.5585	1860.11	0.66	0.00061	28.1674
P08519	0	0.9737	734.6746	2202.009	2.09	0.00153	12.73291
P08519	0	0.5789	593.8146	1186.622	2.9	0.00172	0
P08519	0	0.8548	593.8138	1186.62	1.67	0.00099	3.077636
P08519	0	1	734.6737	2202.007	0.92	0.00068	25.90333
P08519	0	1	547.9332	1641.785	2.31	0.00126	34.38179
P08519	0	0.9574	585.8164	1170.625	1.62	0.00095	11.01561
P08519	0	0.8056	426.2254	1276.662	2.2	0.00093	20.12176
P08519	0	0.881	593.8134	1186.62	0.95	0.00056	5.617805
P08519	0	0.7857	593.8134	1186.619	0.85	0.0005	0
P08519	0	1	545.2564	1633.755	2.3	0.00125	45.62024
P08519	0	1	702.6686	2105.991	0.33	0.00023	34.01252
P08519	0	1	770.3529	2309.044	-0.24	-0.00018	0
P08519	0	0.9024	521.7627	1042.518	1.68	0.00088	46.44989
P08519	0	1	658.3561	2630.402	0.25	0.00016	7.382204
P08519	0	0.7647	561.8162	1122.625	1.48	0.00083	6.989403
P08519	0	0.6923	396.2115	1186.62	1.32	0.00052	54.46737
P04217	0	0.875	411.568	1232.689	2.15	0.00088	51.60205
P04217	0	1	467.7718	1868.065	1.63	0.00076	45.34072
P04217	0	0.9714	623.3612	1868.069	3.54	0.0022	8.988138
P04217	0	1	467.7721	1868.067	2.35	0.0011	33.64283
P04217	0	1	506.272	1516.801	1.72	0.00087	7.296523
P04217	0	0.9831	506.2717	1516.801	1.17	0.00059	4.769586
P04217	0	0.9828	758.9043	1516.801	1.63	0.00123	3.517689
P04217	0	1	506.2716	1516.8	0.81	0.00041	9.545256
P04217	0	0.9677	686.853	1372.699	1.45	0.001	0
P04217	0	0.9811	506.272	1516.801	1.66	0.00084	0
P04217	0	0.9434	758.9042	1516.801	1.47	0.00111	0
P04217	0	0.9831	506.2718	1516.801	1.36	0.00069	10.26194
P04217	0	0.9744	506.2721	1516.802	1.9	0.00096	15.74285
P04217	0	0.9434	686.8526	1372.698	0.83	0.00057	0
P04217	0	1	506.2718	1516.801	1.36	0.00069	44.69265
P04217	0	1	506.2724	1516.803	2.5	0.00127	3.744611
P04217	0	0.9302	506.2718	1516.801	1.36	0.00069	8.640786
P04217	0	0.9474	506.2732	1516.805	4.13	0.00209	56.73125
P04217	0	1	467.772	1868.066	2.03	0.00095	18.26283
P04217	0	0.9429	623.3602	1868.066	2.07	0.00129	8.748311
P04217	0	1	467.7719	1868.066	1.9	0.00089	9.963365
P04217	0	1	458.238	1372.699	1.82	0.00083	35.99899
P04217	0	1	686.8525	1372.698	0.74	0.00051	14.64235
P04217	0	1	467.7715	1868.064	0.98	0.00046	14.2805
P04217	0	0.8696	616.8488	1232.69	2.8	0.00173	3.360213
P04217	0	0.7826	616.8475	1232.688	0.82	0.00051	3.298528
P04217	0	0.7455	411.5677	1232.689	1.48	0.00061	0
P04217	0	0.7586	411.5678	1232.689	1.77	0.00073	5.301482
P04217	0	0.7	518.2913	1552.859	1.23	0.00064	17.84344
P04217	0	1	467.7719	1868.066	1.83	0.00085	0
P04217	0	1	467.7715	1868.064	0.92	0.00043	4.719209
P04217	0	1	623.3604	1868.067	2.36	0.00147	4.141427
P04217	0	1	467.7713	1868.063	0.52	0.00024	4.65883
P04217	0	1	467.7719	1868.066	1.9	0.00089	12.77589

P04217	0	1	467.7724	1868.068	2.94	0.00137	2.159503
P04217	0	0.9688	623.3607	1868.067	2.75	0.00171	8.596077
P04217	0	0.9333	623.3605	1868.067	2.56	0.00159	6.593993
P04217	0	1	467.7719	1868.066	1.83	0.00085	6.86235
P04217	0	1	467.7716	1868.065	1.31	0.00061	5.234429
P04217	0	1	623.3613	1868.069	3.73	0.00232	8.452997
P04217	0	1	467.7716	1868.064	1.11	0.00052	2.00179
P04217	0	0.9394	623.3605	1868.067	2.56	0.00159	6.611272
P04217	0	1	467.7713	1868.063	0.59	0.00027	2.607297
P04217	0	1	467.7718	1868.065	1.7	0.00079	16.70205
P04217	0	1	1308.156	2615.304	1.54	0.00202	0
P04217	0	1	872.4393	2615.303	1.35	0.00118	1.901769
P04217	0	1	506.2719	1516.801	1.42	0.00072	6.498134
P04217	0	0.9868	544.797	1088.587	1.84	0.001	1.619553
P04217	0	0.8969	776.9332	1552.859	1.12	0.00087	6.606741
P04217	0	0.75	518.2919	1552.861	2.41	0.00125	9.461235
P04217	0	0.8286	518.2914	1552.86	1.58	0.00082	12.2374
P04217	0	0.7963	579.8725	1158.738	3.54	0.00205	46.9997
P04217	0	0.7321	579.8728	1158.738	4.06	0.00235	8.94821
P04217	0	0.75	579.8725	1158.738	3.54	0.00205	12.87578
P04217	0	0.7581	579.8705	1158.734	0.17	0.0001	6.367818
P04217	0	1	467.7718	1868.065	1.63	0.00076	5.550165
P04217	0	1	623.361	1868.069	3.34	0.00208	3.685359
P04217	0	1	467.7717	1868.065	1.37	0.00064	3.915395
P04217	0	1	467.7716	1868.065	1.31	0.00061	5.165389
P04217	0	0.9667	623.3604	1868.067	2.36	0.00147	2.388265
P04217	0	1	467.7721	1868.067	2.35	0.0011	2.139393
P04217	0	0.973	623.3609	1868.068	3.14	0.00196	6.439563
P04217	0	1	467.7719	1868.066	1.76	0.00082	5.371679
P04217	0	1	467.7716	1868.065	1.31	0.00061	4.100005
P04217	0	1	467.7725	1868.068	3.07	0.00143	9.717034
P04217	0	1	895.4683	1789.929	0.99	0.00088	0
P04217	0	0.9592	597.3156	1789.932	2.68	0.0016	3.467098
P04217	0	1	814.0983	2440.28	-2.02	-0.00164	62.97336
P04217	0	0.9524	766.0663	2296.184	0.44	0.00034	0
P04217	0	0.988	814.1005	2440.287	0.68	0.00056	2.23003
P04217	0	0.9792	610.8279	2440.29	1.78	0.00109	1.612768
P04217	0	0.9508	814.1014	2440.29	1.81	0.00147	2.35806
P04217	0	0.9623	610.8284	2440.292	2.68	0.00163	10.53614
P04217	0	1	1010.557	2020.107	2.4	0.00243	0
P04217	0	1	674.04	2020.105	1.79	0.00121	31.06759
P04217	0	1	674.0401	2020.106	1.97	0.00133	2.034718
P04217	0	1	813.7937	2439.367	-1.91	-0.00156	38.38825
P04217	0	1	491.5285	1963.092	3.55	0.00174	26.65485
P04217	0	1	1220.192	2439.376	1.97	0.0024	0
P04217	0	1	610.6006	2439.381	3.81	0.00232	6.4293
P04217	0	1	764.7199	2292.145	-0.62	-0.00047	1.997291
P04217	0	1	573.7936	2292.153	2.75	0.00158	10.47242
P04217	0	1	573.7927	2292.149	1.05	0.0006	17.36331
P04217	0	1	819.1279	2455.369	1.19	0.00097	32.55411
P04217	0	1	764.7217	2292.151	1.86	0.00142	3.448748
P04217	0	1	573.7933	2292.151	2.12	0.00121	5.824782

P04217	0	1	610.829	2440.294	3.58	0.00218	28.4765
P04217	0	1	506.2703	1516.796	-1.72	-0.00087	43.00293
P04217	0	0.9688	506.2728	1516.804	3.29	0.00166	20.46011
P04217	0	1	458.2378	1372.699	1.55	0.00071	25.10944
P04217	0	1	814.1011	2440.289	1.36	0.00111	18.35939
P04217	0	0.9643	623.3609	1868.068	3.14	0.00196	10.4663
P04217	0	1	467.772	1868.066	2.09	0.00098	37.14836
P04217	0	1	623.3622	1868.072	5.3	0.0033	12.96531
P04217	0	1	467.7719	1868.066	1.83	0.00085	37.82217
P04217	0	0.8889	507.8203	1014.633	1.76	0.0009	25.31805
P04217	0	1	674.0389	2020.102	0.25	0.00017	46.2002
P04217	0	0.8367	386.9171	1158.737	2.73	0.00106	10.41233
P04217	0	0.7442	616.8486	1232.69	2.5	0.00154	3.25241
P04217	0	0.9796	544.7964	1088.586	0.72	0.00039	41.96601
P04217	0	0.5263	616.8487	1232.69	2.7	0.00166	9.019933
P04217	0	0.6667	386.9166	1158.735	1.47	0.00057	0
P04217	0	0.9355	431.7461	1723.963	1.35	0.00058	0
P04217	1	1	548.3064	1642.905	1.38	0.00076	34.05788
P04217	0	1	506.2724	1516.803	2.44	0.00123	63.71977
P04217	0	0.7843	579.8704	1158.733	-0.15	-0.00009	14.09051
P04217	0	1	814.1014	2440.29	1.81	0.00147	10.27606
P04217	0	0.6667	616.8485	1232.69	2.4	0.00148	0
P04217	0	0.8367	507.8211	1014.635	3.33	0.00169	18.4179
P04217	0	0.6471	616.8481	1232.689	1.71	0.00105	0
P04217	0	0.8	493.7586	986.5099	1.11	0.00055	24.85661
P04217	0	0.6923	467.7741	1868.075	6.67	0.00311	10.58773
P04217	0	0.75	507.8202	1014.633	1.52	0.00077	3.468971
P04217	0	0.4583	518.291	1552.858	0.76	0.00039	9.006186
P04217	0	1	506.2736	1516.806	4.92	0.00249	17.67278
P04217	0	0.9259	431.7462	1723.963	1.64	0.00071	12.52052
P04217	0	1	537.767	2148.046	0.9	0.00048	45.72462
P04217	0	0.8163	507.8203	1014.633	1.76	0.0009	2.283457
P04217	0	0.913	431.7465	1723.964	2.27	0.00098	0
P04217	0	1	579.8723	1158.737	3.11	0.0018	47.93823
P04217	0	0.5926	518.2913	1552.859	1.35	0.0007	8.909708
P04217	0	0.9231	579.8705	1158.734	0.17	0.0001	0
P04217	1	0.0833	395.5761	1184.714	1.48	0.00058	4.865179
P04217	0	1	758.9039	1516.801	1.14	0.00087	0
P04217	0	0.375	616.8486	1232.69	2.6	0.0016	9.254614
P04217	0	0.8125	575.3259	1723.963	1.75	0.00101	34.10489
P04217	0	1	575.3259	1723.963	1.75	0.00101	23.29911
P04217	0	0.5217	616.8486	1232.69	2.5	0.00154	3.731008
P04217	0	0.1538	616.8466	1232.686	-0.67	-0.00041	0
P04217	0	1	431.7459	1723.962	0.79	0.00034	0
P04217	0	1	758.9043	1516.801	1.63	0.00123	0
P04217	0	0.8333	507.8211	1014.635	3.33	0.00169	50.32187
P02749	0	1	891.1047	2671.3	1.66	0.00148	3.980671
P02749	0	1	668.5813	2671.303	3.05	0.00204	0
P02749	0	1	891.1041	2671.298	0.98	0.00087	5.805277
P02749	0	1	891.1046	2671.299	1.53	0.00136	5.273335
P02749	0	1	668.5804	2671.3	1.68	0.00112	4.773991
P02749	1	1	480.2802	1438.826	0.32	0.00015	22.63111

P02749	0	0.9615	464.9218	1392.751	-0.43	-0.0002	16.24735
P02749	0	0.8095	587.3342	2346.315	1.93	0.00113	14.52776
P02749	0	0.8125	782.7773	2346.317	2.98	0.00233	15.41712
P02749	0	0.7931	587.3356	2346.321	4.43	0.0026	16.3359
P02749	0	0.8837	583.8195	1166.632	2.61	0.00152	5.339758
P02749	0	0.9375	464.9224	1392.753	0.88	0.00041	0
P02749	0	0.9688	464.9227	1392.754	1.54	0.00072	7.969976
P02749	0	0.8889	696.8809	1392.754	2.19	0.00152	6.979035
P02749	0	0.9737	464.9227	1392.754	1.54	0.00072	7.401026
P02749	0	1	464.9224	1392.753	0.88	0.00041	32.51595
P02749	0	0.9355	587.3335	2346.312	0.79	0.00046	6.186659
P02749	0	0.9787	782.7758	2346.313	1.11	0.00087	5.84372
P02749	0	0.1579	551.3085	2202.212	1.73	0.00095	3.880956
P02749	0	0.2162	734.7422	2202.212	1.71	0.00126	4.253729
P02749	0	0.6098	734.7432	2202.215	3.13	0.00229	14.44538
P02749	0	0.6098	734.7432	2202.215	3.13	0.00229	14.44538
P02749	0	0.9318	782.7761	2346.314	1.42	0.00111	5.722111
P02749	0	0.931	587.3338	2346.313	1.31	0.00077	5.993733
P02749	0	0.9535	782.7763	2346.314	1.73	0.00135	5.95391
P02749	1	0.9444	662.335	2646.318	2.55	0.00169	6.928754
P02749	1	0.9592	882.777	2646.317	1.91	0.00169	10.25423
P02749	1	0.9643	882.7767	2646.316	1.57	0.00138	42.73511
P02749	0	1	844.0391	2530.103	1.8	0.00152	2.19392
P02749	1	0.7059	662.3345	2646.316	1.72	0.00114	19.73788
P02749	0	1	892.0722	2674.202	0.65	0.00058	0
P02749	0	1	669.3066	2674.205	1.65	0.0011	4.001714
P02749	1	0.8246	480.2808	1438.828	1.53	0.00073	100
P02749	0	1	892.0731	2674.205	1.67	0.00149	4.326416
P02749	0	1	892.0731	2674.205	1.61	0.00143	4.996123
P02749	0	1	892.0729	2674.204	1.4	0.00125	3.725558
P02749	0	1	669.3063	2674.204	1.19	0.0008	4.054362
P02749	0	1	892.0721	2674.202	0.51	0.00046	3.885424
P02749	0	1	892.0722	2674.202	0.65	0.00058	9.727498
P02749	0	1	669.3073	2674.207	2.56	0.00171	3.607074
P02749	0	1	669.307	2674.206	2.1	0.00141	5.63969
P02749	0	1	892.0731	2674.205	1.67	0.00149	4.319246
P02749	0	1	669.3068	2674.205	1.83	0.00122	3.584359
P02749	0	1	892.0734	2674.206	1.95	0.00174	3.166668
P02749	0	1	792.0443	2374.118	1.57	0.00124	5.913152
P02749	0	1	792.0446	2374.119	2.03	0.00161	6.049882
P02749	1	0.9077	730.4313	2189.279	1.64	0.0012	18.72259
P02749	0	1	891.1045	2671.299	1.39	0.00124	7.4634
P02749	1	0.9762	584.1017	2333.385	2.99	0.00175	7.213567
P02749	1	1	778.4655	2333.382	1.74	0.00136	9.083534
P02749	1	0.9077	730.4313	2189.279	1.64	0.0012	18.72259
P02749	1	1	584.1013	2333.383	2.36	0.00138	4.830279
P02749	1	1	778.4654	2333.382	1.59	0.00123	6.518513
P02749	0	1	640.8245	1280.642	1.71	0.00109	9.349539
P02749	0	1	741.6476	2963.569	1.48	0.0011	2.115092
P02749	0	1	988.5264	2963.565	0.07	0.00007	1.032423
P02749	0	1	940.4924	2819.463	0.16	0.00015	8.293025
P02749	0	1	1482.288	2963.568	1.2	0.00178	0

P02749	0	0.8611	511.7676	1022.528	1.37	0.0007	1.549752
P02749	1	0.7895	662.3356	2646.321	3.48	0.0023	0
P02749	0	1	843.0699	2527.195	0.77	0.00065	2.389004
P02749	0	1	959.0449	1917.083	1.95	0.00186	18.49173
P02749	0	1	891.1047	2671.3	1.66	0.00148	4.132637
P02749	0	1	668.5806	2671.3	1.95	0.00131	1.577475
P02749	0	1	669.3067	2674.205	1.74	0.00116	8.412983
P02749	0	1	892.0733	2674.205	1.88	0.00168	3.761561
P02749	1	1	719.9166	1438.826	0.24	0.00018	0
P02749	0	0.087	734.7422	2202.212	1.71	0.00126	32.23905
P02749	0	1	669.3067	2674.205	1.74	0.00116	32.63737
P02749	0	0.9048	782.7769	2346.316	2.43	0.0019	25.57878
P02749	0	1	892.0737	2674.207	2.36	0.0021	0
P02749	0	1	392.5419	1175.611	2.59	0.00102	14.53529
P02749	0	0.7714	583.819	1166.631	1.77	0.00103	0
P02749	0	0.8919	583.8201	1166.633	3.65	0.00213	0
P02749	0	1	568.7734	1136.54	1.93	0.0011	8.640901
P02749	0	0.8235	568.7736	1136.54	2.26	0.00128	3.052112
P02749	1	0.9474	493.7679	1972.05	1.06	0.00052	6.489329
P02749	0	0.3158	470.0682	2346.312	0.56	0.00026	9.968227
P02749	0	0.6087	734.7422	2202.212	1.71	0.00126	24.60968
P02749	0	0.7895	782.7753	2346.311	0.41	0.00032	38.91525
P02749	0	1	427.5524	1280.643	2.48	0.00106	2.404241
P02749	0	0.8611	583.8181	1166.629	0.31	0.00018	3.05824
P02749	0	1	427.552	1280.641	1.55	0.00066	6.83111
P02749	0	0.7895	583.8198	1166.632	3.13	0.00183	3.420103
P02749	0	0.1579	551.3083	2202.211	1.4	0.00077	14.06844
P02749	0	0.1579	551.3083	2202.211	1.4	0.00077	14.06844
P02749	0	1	513.5963	1538.774	2.88	0.00148	40.76123
P02749	0	1	427.5526	1280.643	2.84	0.00121	16.75869
P02749	0	1	669.3059	2674.202	0.55	0.00037	0
P02749	0	1	387.2096	1159.614	0.82	0.00032	42.21714
P02749	0	1	427.5517	1280.641	0.84	0.00036	14.1054
P02749	0	1	640.825	1280.643	2.47	0.00158	3.114318
P02749	0	0.7714	389.5492	1166.633	3.88	0.00151	0
P02749	0	1	427.5523	1280.642	2.2	0.00094	50.21577
P02749	0	0.7857	734.741	2202.209	0.13	0.0001	9.436034
P02749	0	0.7857	734.741	2202.209	0.13	0.0001	9.436034
P02749	0	1	464.9237	1392.756	3.65	0.00169	25.5619
P02749	0	1	511.7676	1022.528	1.43	0.00073	7.928835
P02749	1	0.5	730.4331	2189.285	4.07	0.00297	18.84471
P02749	1	0.5	730.4331	2189.285	4.07	0.00297	18.84471
P02749	0	1	511.7679	1022.529	1.97	0.00101	5.537322
P02749	0	0.7333	734.7416	2202.21	0.88	0.00065	13.83534
P02749	0	0.7333	734.7416	2202.21	0.88	0.00065	13.83534
P02749	0	0.7222	782.778	2346.319	3.84	0.003	4.200603
P02749	0	1	389.5484	1166.631	1.68	0.00065	35.17702
P02749	0	0.6471	734.7415	2202.21	0.71	0.00052	10.88852
P02749	0	0.6471	734.7415	2202.21	0.71	0.00052	10.88852
P02749	0	1	892.0754	2674.212	4.21	0.00375	6.960771
P02749	0	0.25	587.3348	2346.318	3.08	0.00181	4.048322
P04004	0	1	604.2967	1810.876	-1.1	-0.00066	20.63791

P04004	0	1	604.299	1810.882	2.64	0.0016	0
P04004	0	1	604.299	1810.882	2.74	0.00166	2.972012
P04004	0	0.973	905.944	1810.881	1.74	0.00158	3.939086
P04004	0	1	604.2977	1810.879	0.62	0.00037	4.435607
P04004	0	0.9753	905.9446	1810.882	2.41	0.00219	3.19997
P04004	0	1	604.2982	1810.88	1.43	0.00086	5.236632
P04004	0	1	453.4752	1810.879	0.85	0.00038	27.35274
P04004	0	0.8125	549.9863	1647.944	4.35	0.00239	8.535947
P04004	0	1	604.2985	1810.881	1.93	0.00117	7.679681
P04004	0	0.3684	392.5773	1175.717	2.87	0.00112	57.71684
P04004	0	1	783.8836	1566.76	2.74	0.00214	45.8703
P04004	0	1	783.8834	1566.76	2.5	0.00196	19.79913
P04004	0	1	609.6303	1826.876	2.16	0.00132	9.352267
P04004	0	1	604.3001	1810.886	4.46	0.00269	0
P04004	0	0.3889	392.5769	1175.716	2.01	0.00079	25.27228
P04004	0	0.7895	448.7658	1792.041	1.03	0.00046	43.70597
P04004	0	0.9529	823.9132	1646.819	1.04	0.00086	1.53857
P04004	0	0.9412	783.8828	1566.758	1.65	0.00129	4.879219
P04004	0	0.9831	522.9245	1566.759	2.1	0.0011	7.884763
P04004	0	0.9828	783.8831	1566.759	2.03	0.00159	5.261366
P04004	0	0.9429	522.9243	1566.758	1.75	0.00092	4.753265
P04004	0	0.9828	783.8816	1566.756	0.16	0.00013	3.355197
P04004	0	1	522.9238	1566.757	0.7	0.00037	4.358677
P04004	0	1	907.4637	1813.92	1.38	0.00125	3.616855
P04004	0	0.9551	711.8318	1422.656	1.91	0.00136	0
P04004	0	0.9863	711.8311	1422.655	0.96	0.00068	0
P04004	0	1	679.3377	1357.668	1.57	0.00106	5.780124
P04004	0	0.9474	531.5894	1592.754	1.91	0.00101	9.137148
P04004	1	0.8333	486.9327	1458.784	1.05	0.00051	10.16653
P04004	1	0.7368	486.9327	1458.784	0.98	0.00048	5.622293
P04004	0	1	679.3378	1357.668	1.75	0.00119	11.18523
P04004	1	0.7059	486.9326	1458.783	0.8	0.00039	5.069892
P04004	1	0.6053	486.9325	1458.783	0.54	0.00026	4.561823
P04004	1	0.9706	531.6266	1592.865	1.69	0.0009	7.233706
P04004	0	1	960.1405	2878.407	1.24	0.00119	7.251732
P04004	0	1	556.2645	1666.779	2.15	0.00119	0.168595
P04004	0	1	716.3583	2862.412	1.07	0.00076	8.136818
P04004	0	1	954.8101	2862.416	2.5	0.00238	7.33891
P04004	1	0.9231	486.9319	1458.781	-0.65	-0.00032	0
P04004	0	0.9474	876.3946	2627.169	4.08	0.00357	0
P04004	1	0.7619	486.9322	1458.782	-0.08	-0.00004	0
P04004	0	0.9487	531.589	1592.752	1.22	0.00065	14.80103
P04004	0	1	943.3971	3770.567	2.75	0.0026	2.132069
P04004	1	1	483.592	1448.762	0.77	0.00037	27.39853
P04004	0	1	605.3118	1813.921	1.84	0.00112	18.4481
P04004	0	0.9535	453.2278	1357.669	2.05	0.00093	8.854127
P04004	0	0.8286	651.8458	1302.684	2.57	0.00167	4.076452
P04004	1	0.775	438.8991	1314.683	1.91	0.00084	10.13515
P04004	0	0.55	392.5771	1175.717	2.32	0.00091	13.28334
P04004	0	1	453.4764	1810.884	3.34	0.00151	36.4066
P04004	0	1	556.2633	1666.775	-0.05	-0.00003	7.184883
P04004	0	0.5	597.6469	1790.926	3.81	0.00227	7.779567

P04004	1	1	408.2212	1629.863	0.01	0	13.36487
P04004	0	0.9	556.2651	1666.781	3.24	0.0018	59.78996
P04004	0	0.9474	651.8458	1302.684	2.48	0.00161	7.478293
P04004	1	0.875	438.8992	1314.683	2.19	0.00096	11.41148
P04004	0	0.9375	522.9243	1566.758	1.64	0.00085	10.10336
P04004	0	0.9688	783.8824	1566.757	1.18	0.00092	4.338346
P04004	0	0.9804	607.2875	1213.568	3.17	0.00192	0
P04004	0	0.8857	895.9642	1790.921	1.02	0.00092	6.269338
P04004	0	0.9697	783.8831	1566.759	2.03	0.00159	0
P04004	0	1	561.5977	1682.778	4.78	0.00268	25.54196
P04004	1	0.5294	486.9334	1458.786	2.36	0.00115	9.338683
P04004	0	0.9737	531.5862	1592.744	-4.07	-0.00216	0
P04004	1	0.8485	438.8989	1314.682	1.56	0.00069	13.23536
P04004	0	0.9394	605.3111	1813.919	0.63	0.00038	7.073317
P04004	0	0.9474	598.0199	1792.045	3.25	0.00194	36.62289
P04004	1	0.8696	438.8988	1314.682	1.36	0.00059	33.45156
P04004	0	0.7368	796.8785	1592.75	-0.42	-0.00033	22.40794
P04004	0	1	448.7665	1792.044	2.73	0.00122	57.48979
P04004	0	1	833.8924	1666.778	1.24	0.00103	2.185148
P04004	0	0.8421	895.9638	1790.92	0.48	0.00043	29.18975
P04004	0	1	392.5768	1175.716	1.7	0.00067	33.43949
P04220; P0	0	1	465.2515	1393.74	1.57	0.00073	32.03212
P04220; P0	0	0.9273	697.3748	1393.742	3.16	0.0022	16.76457
P04220; P0	0	1	640.0442	1918.118	1.93	0.00123	5.983711
P04220; P0	0	1	480.2844	1918.116	0.81	0.00039	4.934416
P04220; P0	0	1	480.2846	1918.117	1.19	0.00057	26.15992
P04220; P0	0	1	625.3226	1249.638	1.78	0.00111	5.815742
P01871	0	1	559.0185	1675.041	0.77	0.00043	7.077498
P01871	0	0.9706	559.019	1675.043	1.75	0.00098	35.56913
P04220; P0	0	0.8333	502.0225	2005.068	0.74	0.00037	33.76444
P04220; P0	0	0.9848	669.0283	2005.07	1.78	0.00119	7.875226
P01871	0	0.8649	522.6434	1565.916	0.93	0.00049	20.74918
P01871	0	1	602.6325	1805.883	3.63	0.00218	8.371262
P01871	0	0.8152	636.0099	1906.015	1.64	0.00104	7.662244
P01871	0	0.8361	953.512	1906.017	2.51	0.0024	6.028083
P01871	0	1	607.9641	1821.878	3.54	0.00215	0
P01871	0	0.9833	602.6322	1805.882	3.12	0.00188	5.250598
P04220; P0	0	1	963.5733	1926.139	2.63	0.00253	9.84608
P04220; P0	0	1	582.2993	1744.883	1.35	0.00078	2.909007
P04220; P0	0	0.5556	585.8173	1170.627	2.38	0.00139	7.087362
P04220; P0	0	1	575.8695	1150.732	2.78	0.0016	5.647379
P04220; P0	0	1	500.2838	999.5604	1.23	0.00061	0
P04220; P0	0	0.9333	381.8922	1143.662	0.87	0.00033	23.20232
P01871	0	0.8824	452.2239	1805.874	-1.52	-0.00068	69.11008
P04220; P0	0	0.5424	585.8171	1170.627	2.17	0.00127	20.0987
P01871	0	0.6944	522.6445	1565.919	3.04	0.00158	18.59729
P04220; P0	0	0.8333	642.7177	1926.138	2.15	0.00138	8.946026
P01871	0	0.9118	838.0234	1675.039	-0.1	-0.00008	3.986452
P04220; P0	0	0.8947	450.7702	900.5332	3.36	0.00151	18.34709
P04220; P0	0	1	582.3002	1744.886	3.02	0.00176	0
P01871	0	0.8148	559.019	1675.043	1.75	0.00098	2.936554
P01871	0	0.6207	522.6447	1565.919	3.39	0.00177	6.791483

P04220; P0	0	1	575.8695	1150.732	2.68	0.00154	7.036704
P04220; P0	0	0.9333	669.0283	2005.07	1.87	0.00125	0
P04220; P0	0	1	502.0216	2005.064	-1.09	-0.00055	0
P04220; P0	0	0.6286	522.8208	1044.634	2.01	0.00105	27.21322
P04220; P0	0	1	500.2843	999.5613	2.15	0.00107	0
P04220; P0	0	1	640.0452	1918.121	3.46	0.00221	0
P01871	0	0.7143	838.0233	1675.039	-0.17	-0.00014	5.722323
P04220; P0	0	0.9474	669.031	2005.078	5.89	0.00394	70.9072
P01871	0	0.8148	838.0248	1675.042	1.58	0.00132	4.8058
P04220; P0	0	1	572.3357	1143.664	2.56	0.00146	23.70536
P01871	0	1	394.5599	1181.665	1.83	0.00072	57.79884
P04220; P0	0	0.8214	522.8209	1044.634	2.13	0.00111	0
P04220; P0	0	0.9688	384.2483	1150.73	1.47	0.00056	5.071266
P01871	1	0.8333	740.7289	2220.172	0.49	0.00036	0
P01871	1	0.8333	740.7289	2220.172	0.49	0.00036	0
P04220; P0	0	1	575.8692	1150.731	2.25	0.0013	0
P01871	0	0.2353	636.0113	1906.019	3.85	0.00245	13.51828
P04220; P0	0	0.84	522.8204	1044.634	1.31	0.00068	53.48961
P04220; P0	0	1	572.3355	1143.664	2.13	0.00122	0
P04220; P0	0	0.8276	522.8203	1044.633	0.96	0.0005	23.53466
P04220; P0	0	0.7273	522.8204	1044.633	1.19	0.00062	6.409844
P07358	1	1	563.9639	1689.877	3.18	0.00179	10.43833
P07358	1	1	563.9641	1689.878	3.61	0.00203	26.54396
P07358	0	1	582.8198	1164.632	2.76	0.00161	10.924
P07358	0	1	792.3518	2375.041	1.68	0.00133	40.51714
P07358	0	1	792.3524	2375.043	2.38	0.00188	49.36799
P07358	0	0.9804	673.0809	2689.302	2.15	0.00144	3.30985
P07358	0	1	897.1053	2689.301	2.02	0.00181	4.317266
P07358	0	1	1055.542	4219.147	4.9	0.00516	0
P07358	1	1	563.9641	1689.878	3.5	0.00197	30.51527
P07358	0	1	1203.125	2405.243	3.24	0.00389	71.62
P07358	0	0.9867	756.3926	3022.548	0.36	0.00027	5.500639
P07358	0	1	734.8221	1468.637	1.46	0.00107	3.550969
P07358	0	1	734.8221	1468.637	1.46	0.00107	5.861677
P07358	0	1	734.8222	1468.637	1.54	0.00113	5.611843
P07358	0	1	767.3903	1533.773	1.74	0.00134	1.78136
P07358	0	0.9512	511.9285	1533.771	0.18	0.00009	0
P07358	0	1	582.8201	1164.633	3.29	0.00191	26.98871
P07358	0	0.6552	450.5805	1349.727	-2.56	-0.00115	30.12641
P07358	0	0.9286	527.6187	1580.842	2.56	0.00135	19.22535
P07358	0	0.9583	527.6179	1580.839	0.94	0.00049	36.62271
P07358	0	1	490.2177	1468.638	2.47	0.00121	0
P07358	0	0.3415	537.8425	1074.678	1.45	0.00078	38.65061
P07358	0	0.3721	537.843	1074.679	2.35	0.00127	23.17795
P07358	1	1	563.9636	1689.876	2.74	0.00154	13.86621
P07358	0	1	588.3222	1762.952	3.68	0.00216	0
P07358	0	1	582.8203	1164.633	3.49	0.00204	9.41748
P07358	0	1	543.7983	2172.171	2.4	0.0013	46.16141
P07358	0	1	476.8879	1428.649	0.74	0.00035	69.17966
P07358	1	0.7917	435.6021	1304.792	1.45	0.00063	15.58764
P07358	0	0.4615	673.0811	2689.302	2.42	0.00163	0
P07358	0	0.7586	767.3903	1533.773	1.74	0.00134	0

P07358	0	1	532.9471	1596.827	-3.61	-0.00192	31.03259
P07358	1	1	435.602	1304.792	1.31	0.00057	33.86082
P07358	0	0.6	388.8821	1164.632	2.17	0.00084	4.267658
P04196	0	0.8667	545.6033	1634.795	1.44	0.00078	1.871024
P04196	0	0.9221	705.0385	2113.101	3.07	0.00216	8.378069
P04196	0	0.9643	1057.054	2113.102	3.45	0.00365	0
P04196	0	1	392.629	1959.116	1.25	0.00049	50.52021
P04196	0	1	913.9526	1826.898	2.81	0.00256	3.03904
P04196	0	1	913.9532	1826.899	3.41	0.00311	0
P04196	0	1	490.5347	1959.117	1.72	0.00084	10.04774
P04196	0	0.1667	454.5088	1815.013	0.92	0.00042	15.18119
P04196	0	1	841.9001	1682.793	1.24	0.00104	0.884863
P04196	0	1	609.6363	1826.894	0.79	0.00048	4.865746
P04196	0	1	913.9499	1826.893	-0.2	-0.00018	3.49339
P04196	0	0.9467	529.0302	2113.099	2.11	0.00112	1.517609
P04196	0	0.9464	1057.053	2113.099	2.07	0.00218	0
P04196	0	0.8961	705.0366	2113.095	0.47	0.00033	4.539976
P04196	0	0.991	985.0024	1968.997	2.66	0.00262	4.738513
P04196	0	0.8	657.0036	1968.996	2.03	0.00133	2.401388
P04196	0	1	392.6297	1959.119	2.88	0.00113	36.28401
P04196	0	1	525.6365	1574.895	1.52	0.0008	29.59
P04196	0	0.9512	705.0418	2113.111	7.84	0.00552	14.25234
P04196	0	1	426.5501	1277.636	2.19	0.00093	5.333055
P04196	0	1	426.5497	1277.635	1.34	0.00057	0
P04196	1	1	603.6291	1808.873	1.87	0.00113	9.541204
P04196	1	1	603.6289	1808.872	1.56	0.00094	10.07456
P04196	1	1	697.4015	2786.584	4.44	0.00309	10.0829
P04196	1	0.9661	558.1213	2786.578	2.09	0.00117	13.27547
P04196	1	0.8824	558.1215	2786.578	2.31	0.00129	22.68667
P04196	1	0.9697	558.1208	2786.575	1.11	0.00062	7.354231
P04196	1	1	697.4008	2786.581	3.48	0.00242	6.285002
P04196	1	1	618.9623	1854.872	2.25	0.00139	10.96086
P04196	0	0.9623	418.2108	1252.618	2.18	0.00091	20.29903
P04196	1	1	618.9624	1854.873	2.35	0.00145	8.439034
P04196	1	1	618.9627	1854.874	2.84	0.00176	10.27925
P04196	0	1	716.0405	2146.107	1.62	0.00116	16.35294
P04196	0	0.9412	629.3509	2514.382	2.79	0.00175	5.761184
P04196	0	1	791.8404	1582.674	1.42	0.00112	4.009649
P04196	1	1	603.6299	1808.875	3.18	0.00192	11.6588
P04196	0	1	418.2102	1252.616	0.79	0.00033	10.56521
P04196	0	1	418.2101	1252.616	0.64	0.00027	10.00089
P04196	0	0.8846	477.6031	1430.795	2.88	0.00137	8.389368
P04196	0	0.9583	525.6362	1574.894	0.94	0.00049	50.52448
P04196	0	0.9615	525.6367	1574.896	1.87	0.00098	22.44906
P04196	0	1	394.4794	1574.896	1.98	0.00078	14.03827
P04196	0	1	696.1163	2781.443	2.83	0.00197	4.827563
P04196	0	1	557.0947	2781.445	3.28	0.00183	8.066754
P04196	0	0.9167	696.1167	2781.445	3.44	0.00239	14.55443
P04196	1	1	475.6103	1424.816	2.36	0.00112	19.41712
P04196	0	0.8	557.095	2781.446	3.72	0.00207	24.08427
P04196	0	1	426.55	1277.635	1.84	0.00078	0
P04196	0	1	927.8198	2781.445	3.42	0.00318	8.309294

P04196	0	0.8519	537.282	2146.106	1.38	0.00074	3.63231
P04196	0	1	525.6371	1574.897	2.57	0.00135	14.92081
P04196	0	1	525.6362	1574.894	0.94	0.00049	31.06642
P04196	1	1	464.4731	1854.871	1.19	0.00055	13.52289
P04196	0	0.9286	716.0397	2146.105	0.59	0.00042	0
P04196	0	1	426.5501	1277.636	2.12	0.0009	24.67882
P04196	0	0.4091	537.8424	1074.678	1.33	0.00072	12.1311
P04196	0	0.3333	537.8425	1074.678	1.45	0.00078	14.6035
P04196	0	1	528.2296	1582.674	1.91	0.00101	26.85953
P04196	0	1	528.2306	1582.677	3.76	0.00198	23.80724
P04196	0	0.7333	545.6041	1634.798	2.89	0.00158	11.83561
P04196	0	1	426.55	1277.635	1.84	0.00078	21.7995
P04196	0	1	554.7606	1108.514	0.86	0.00048	3.110776
P04196	0	0.26	537.8429	1074.679	2.24	0.0012	6.959748
P04196	0	1	426.55	1277.635	1.84	0.00078	27.40044
P04196	1	0.9688	464.4735	1854.872	2.18	0.00101	8.650406
P04196	0	0	537.8431	1074.679	2.7	0.00145	46.21024
P04196	0	0.3846	537.8422	1074.677	0.88	0.00047	6.377517
P04196	0	0.7674	567.2709	1133.534	3.14	0.00178	0
P04196	0	1	554.7615	1108.516	2.51	0.00139	8.653824
P04196	0	0.875	838.7961	2514.374	-0.34	-0.00028	0
P04196	0	0.9524	639.3216	1277.636	2.3	0.00147	0
P04196	0	0.8	545.6027	1634.794	0.32	0.00017	29.51368
P04196	0	0.9268	527.7933	1054.579	2.94	0.00155	30.509
P04196	0	0.9375	455.7412	910.4751	1.08	0.00049	48.12349
P04196	0	0.3191	537.8429	1074.679	2.24	0.0012	6.041926
P04196	1	1	904.9398	1808.872	1.6	0.00145	3.63531
P04196	0	1	817.8975	1634.788	-3.26	-0.00266	0
P04196	0	0.7895	567.272	1133.537	5.19	0.00294	7.07225
P04196	0	0.3125	537.8433	1074.679	2.92	0.00157	11.55303
P04196	0	1	817.9004	1634.794	0.25	0.0002	21.16752
P04196	1	0.8261	618.9627	1854.873	2.75	0.0017	10.69883
P04196	0	0.6129	626.8115	1252.616	0.56	0.00035	0
P04196	0	0.129	537.8434	1074.679	3.15	0.00169	35.42142
P04196	0	0.6562	527.7925	1054.578	1.55	0.00082	16.80746
P36955	0	0.7636	697.8879	1394.769	2.76	0.00192	9.999237
P36955	0	0.95	448.6222	1343.852	1.54	0.00069	15.83408
P36955	0	0.9714	672.4302	1343.853	2.34	0.00157	32.44493
P36955	0	1	448.6223	1343.852	1.67	0.00075	11.10554
P36955	0	0.9506	836.4478	1671.888	3.01	0.00252	15.31006
P36955	0	1	557.9673	1671.887	2.45	0.00137	10.5872
P36955	0	0.9333	557.9671	1671.887	2.13	0.00118	5.558527
P36955	0	1	748.7327	2244.184	2.04	0.00153	43.38021
P36955	0	0.9032	764.3956	1527.784	1.78	0.00136	19.39532
P36955	0	0.9667	769.9386	1538.87	2.02	0.00155	14.48951
P36955	0	0.9362	513.6278	1538.869	1.21	0.00062	7.95679
P36955	0	1	561.8021	2244.186	3.29	0.00184	2.098896
P36955	0	1	748.7325	2244.183	1.72	0.00128	4.604881
P36955	0	1	748.7336	2244.186	3.18	0.00238	18.07862
P36955	0	0.9722	554.2668	1660.786	2.13	0.00118	2.629496
P36955	0	1	830.8962	1660.785	1.77	0.00147	2.861014
P36955	0	1	830.896	1660.785	1.48	0.00123	0

P36955	0	0.871	657.3644	1313.721	1.82	0.0012	20.75306
P36955	0	1	568.6357	1703.892	3.03	0.00172	0
P36955	0	1	793.1458	2377.423	2.43	0.00193	8.732281
P36955	0	1	728.0969	2182.276	1.68	0.00122	5.081532
P36955	0	1	1039.24	3115.705	2.69	0.00279	4.694012
P36955	0	0.8772	501.6279	1502.869	1.61	0.00081	16.87963
P36955	1	1	644.9993	1932.983	2.05	0.00132	6.304137
P36955	1	1	644.9989	1932.982	1.48	0.00096	17.45774
P36955	0	0.8864	657.3649	1313.723	2.66	0.00174	21.59336
P36955	0	0.913	706.7137	2118.127	0.44	0.00031	27.39898
P36955	0	0.7818	758.8452	1516.683	1.87	0.00142	4.452028
P36955	0	1	633.3732	1265.739	3.82	0.00242	4.903155
P36955	0	0.7381	513.6283	1538.87	2.28	0.00117	6.366084
P36955	0	0.9268	852.4495	1703.892	2.61	0.00222	3.201324
P36955	0	1	633.3723	1265.737	2.37	0.0015	6.473435
P36955	0	0.7143	396.9399	1188.805	0.85	0.00034	16.32648
P36955	0	0.9091	482.5787	1445.722	2.16	0.00104	6.221749
P36955	0	0.931	554.2665	1660.785	1.58	0.00088	7.481616
P36955	0	0.881	594.907	1188.807	2.37	0.00141	41.41627
P36955	0	0.6538	465.5943	1394.768	2.57	0.0012	0
P36955	1	1	725.7218	2175.151	7.36	0.00533	6.280447
P36955	1	0.9394	596.9662	1788.884	3.79	0.00226	54.25206
P36955	1	0.9394	596.9662	1788.884	3.79	0.00226	54.25206
P36955	0	1	396.9403	1188.806	1.92	0.00076	11.23302
P36955	0	0.7083	438.5797	1313.725	4.25	0.00186	13.72674
P36955	0	0.75	513.627	1538.866	-0.21	-0.00011	4.669147
P36955	0	1	422.5834	1265.736	0.96	0.00041	45.36097
P36955	0	1	836.4507	1671.894	6.59	0.00551	33.16814
P36955	0	0.9286	672.4302	1343.853	2.25	0.00151	31.12382
P35858	0	1	533.6469	1598.926	0.91	0.00049	5.389612
P35858	0	1	400.4872	1598.927	1.52	0.00061	19.20371
P35858	0	1	400.4874	1598.928	1.9	0.00076	16.05134
P35858	0	1	493.628	1478.869	1.43	0.00071	25.24733
P35858	0	0.8636	594.3565	1187.706	3.29	0.00196	8.47803
P35858	0	1	463.5774	1388.718	-1.03	-0.00048	41.22989
P35858	0	1	463.5789	1388.722	2.07	0.00096	6.000164
P35858	0	0.9365	711.6901	2133.056	2.66	0.00189	8.242767
P35858	0	0.875	522.3046	1043.602	2.12	0.00111	3.951901
P35858	0	0.9583	415.5446	1244.619	1.78	0.00074	42.08899
P35858	0	1	445.5941	1334.768	1.85	0.00082	38.7625
P35858	0	0.875	541.624	1622.857	1.24	0.00067	3.943749
P35858	0	0.76	395.5487	1184.631	1.86	0.00074	6.30772
P35858	0	0.7551	395.5487	1184.632	1.94	0.00077	14.68786
P35858	0	0.871	929.1733	2785.505	2.89	0.00268	3.399588
P35858	0	0.9692	697.131	2785.502	1.77	0.00123	4.313786
P35858	0	1	804.467	1607.927	1.84	0.00148	5.22083
P35858	0	1	536.6472	1607.927	1.99	0.00107	8.078855
P35858	0	0.8958	697.1312	2785.503	1.94	0.00135	8.024984
P35858	0	1	732.4158	1463.824	1.87	0.00137	25.78742
P35858	0	0.8222	622.3333	1864.985	3.54	0.0022	3.53594
P35858	0	0.9333	872.0438	1743.08	1.88	0.00164	8.871238
P35858	0	1	785.7714	2355.3	4	0.00314	42.10468

P35858	0	1	908.0297	1815.052	1.39	0.00126	17.05075
P35858	0	0.974	605.69	1815.055	3.2	0.00194	1.539676
P35858	0	1	693.88	1386.753	2.13	0.00148	13.75502
P35858	0	1	953.5509	1906.095	1.41	0.00134	7.353093
P35858	0	1	406.4701	1622.859	1.98	0.00081	35.73656
P35858	0	0.7115	503.8006	1006.594	0.76	0.00038	4.715031
P35858	0	0.7778	711.6907	2133.058	3.6	0.00256	33.77448
P35858	0	0.7241	697.1315	2785.504	2.47	0.00172	8.069437
P35858	0	0.8462	929.1719	2785.501	1.31	0.00122	3.697706
P35858	0	0.875	594.3568	1187.706	3.81	0.00226	33.84117
P35858	0	0.7879	396.5729	1187.704	2.07	0.00082	0
P35858	0	1	621.8279	1242.649	0.72	0.00045	25.64685
P35858	0	1	533.647	1598.926	1.03	0.00055	41.89381
P35858	0	0.8293	525.8325	1050.658	2.65	0.00139	17.65205
P35858	0	1	693.8806	1386.754	3.01	0.00209	13.39086
P35858	0	1	463.5783	1388.72	0.95	0.00044	32.9597
P35858	0	0.9444	811.934	1622.861	3.36	0.00272	6.006824
P35858	0	0.5333	697.1307	2785.501	1.24	0.00086	6.41454
P35858	0	1	462.9224	1386.753	1.99	0.00092	42.73395
P09871	0	0.8308	1040.532	2080.057	3.51	0.00365	28.03457
P09871	0	0.9787	694.0219	2080.051	0.51	0.00036	0
P09871	1	1	743.8488	2972.373	-2.71	-0.00201	33.75748
P09871	1	0.95	991.4686	2972.391	3.4	0.00336	0
P09871	0	0.9216	694.0226	2080.053	1.57	0.00109	2.627485
P09871	0	0.8228	694.0229	2080.054	1.92	0.00133	63.77286
P09871	1	1	743.8514	2972.384	0.91	0.00067	0
P09871	0	0.9231	684.7112	2052.119	-1.24	-0.00085	11.42735
P09871	0	0.9855	684.7129	2052.124	1.26	0.00086	7.055655
P09871	0	1	803.0768	2407.216	2.19	0.00176	28.86472
P09871	0	0.5556	499.7745	1996.076	2.2	0.0011	17.65178
P09871	0	0.8652	846.9512	1692.895	1.6	0.00136	3.926589
P09871	0	0.8689	678.8663	1356.725	2.2	0.00149	7.856259
P09871	0	0.9524	684.7136	2052.126	2.24	0.00153	19.16875
P09871	0	0.8718	830.9255	1660.844	0.97	0.00081	3.706363
P09871	0	0.6905	442.221	1765.862	1.39	0.00061	15.40198
P09871	0	1	596.2973	1786.877	0.66	0.00039	42.90478
P09871	0	0.7344	638.8727	1276.738	2.45	0.00156	5.547648
P09871	0	0.6923	581.8585	1162.71	2.74	0.00159	3.94896
P09871	0	1	495.7754	1980.08	1.43	0.00071	12.07963
P09871	0	1	711.3799	1421.752	1.4	0.00099	30.30542
P09871	0	1	639.3295	1277.652	2.61	0.00167	24.91023
P09871	0	0.9667	825.4247	2474.259	1.66	0.00137	3.619407
P09871	0	1	497.2447	1489.72	1.75	0.00087	45.71563
P09871	0	0.8043	581.8586	1162.71	3.06	0.00178	6.365846
P09871	0	1	602.5602	2407.219	3.51	0.00211	7.649306
P09871	0	0.9545	883.4371	1765.867	4.17	0.00368	14.60784
P09871	0	0.8919	684.7139	2052.127	2.69	0.00184	31.0742
P48740; P0	0	1	564.259	1127.511	1.99	0.00112	2.876967
P09871	0	0.85	619.322	2474.266	4.29	0.00265	46.77302
P09871	0	0.9444	589.2943	1765.868	4.92	0.0029	5.180348
P09871	0	0.8462	581.8586	1162.71	3.06	0.00178	8.288093
P09871	0	0.9615	589.2937	1765.867	3.99	0.00235	32.24091

P09871	0	0.2667	1040.531	2080.055	2.34	0.00243	8.7313
P09871	0	0.6579	426.2496	1276.734	-0.64	-0.00027	14.45341
P09871	0	0.4667	499.773	1996.07	-0.74	-0.00037	25.86014
P09871	0	0.6667	495.7746	1980.077	-0.11	-0.00005	28.13392
P09871	0	0.6944	528.8132	1056.619	1.98	0.00105	11.61038
P09871	0	1	449.2111	1345.619	2.81	0.00126	43.92967
P09871	0	0.8	639.3295	1277.652	2.52	0.00161	9.942218
P09871	0	0.9615	589.2926	1765.863	2.12	0.00125	7.74291
P09871	0	0.56	554.2871	1660.847	2.73	0.00151	11.88295
P09871	0	1	803.0772	2407.217	2.57	0.00207	12.02987
P09871	0	0.8462	581.8585	1162.71	2.85	0.00166	24.34666
P09871	1	0.8462	394.6046	1181.799	1.59	0.00063	49.87727
P09871	0	0.4857	528.8131	1056.619	1.75	0.00093	20.8189
P09871	0	1	660.6975	1980.078	0.52	0.00034	46.0041
P05156	0	1	746.3646	2237.079	2.43	0.00181	51.57938
P05156	0	0.8302	582.3074	1744.908	2.65	0.00154	9.603717
P05156	0	0.9107	582.3075	1744.908	2.86	0.00166	22.03381
P05156	0	0.9412	582.3076	1744.908	3.07	0.00178	11.87974
P05156	0	0.9444	746.364	2237.077	1.69	0.00126	28.09837
P05156	0	0.9677	746.3649	2237.08	2.84	0.00212	2.860582
P05156	0	0.2439	722.1243	2885.475	0.23	0.00017	0
P05156	0	1	744.0195	2230.044	1.88	0.0014	15.36472
P05156	0	1	738.6876	2214.048	1.52	0.00113	5.621373
P05156	0	0.6739	558.298	1672.88	3.06	0.00171	16.23565
P05156	0	0.7021	558.2975	1672.878	2.08	0.00116	10.70521
P05156	1	0.8364	792.4341	1583.861	2.1	0.00166	0
P05156	0	0.9565	740.9257	1480.844	2.59	0.00192	6.642381
P05156	0	0.6562	437.8932	1311.665	3.48	0.00152	63.48926
P05156	0	1	704.3052	2110.901	1.91	0.00134	4.829153
P05156	0	1	698.9731	2094.905	1.36	0.00095	8.653643
P05156	0	1	1047.958	2094.908	2.85	0.00298	0.914089
P05156	0	0.9412	698.9736	2094.906	1.97	0.00137	14.00094
P05156	0	1	698.9736	2094.906	2.06	0.00144	18.73021
P05156	0	1	802.3301	2404.976	1.7	0.00136	19.2885
P05156	0	1	802.3301	2404.976	1.7	0.00136	4.820234
P05156	0	1	601.9999	2404.978	2.63	0.00158	7.230313
P05156	0	1	1202.992	2404.977	2.29	0.00275	0
P05156	0	1	768.0881	2302.25	0.99	0.00076	5.977662
P05156	0	1	802.3294	2404.974	0.86	0.00069	7.037961
P05156	0	1	754.2963	2260.874	2.17	0.00163	1.57138
P05156	0	1	602.0001	2404.979	2.94	0.00177	13.87017
P05156	0	1	1202.991	2404.975	1.58	0.0019	0
P05156	0	1	802.3297	2404.975	1.24	0.001	19.79661
P05156	0	1	802.3292	2404.973	0.56	0.00045	22.48761
P05156	0	0.9189	477.263	1429.774	2.25	0.00107	8.583925
P05156	0	0.7179	576.3433	1727.015	3.48	0.002	6.793579
P05156	1	0.8462	628.3768	1883.116	2.78	0.00175	14.15436
P05156	0	0.9535	477.2609	1429.768	-2.04	-0.00097	35.31183
P05156	0	0.8889	494.2853	1480.841	0.78	0.00039	11.48084
P05156	0	1	472.2299	1414.675	1.5	0.00071	37.17046
P05156	0	0.9524	494.2859	1480.843	1.9	0.00094	10.58906
P05156	0	0.8542	575.8345	1150.662	2.57	0.00148	2.802928

P05156	0	1	802.3297	2404.975	1.24	0.001	11.3441
P05156	0	0.5294	576.3426	1727.013	2.21	0.00127	4.145463
P05156	1	1	396.7206	1583.86	1.78	0.0007	12.06563
P05156	0	1	754.2958	2260.873	1.44	0.00108	0
P05156	0	1	632.8241	1264.641	2.53	0.0016	5.045718
P05156	0	0.5769	528.3077	1582.909	0.84	0.00044	5.235936
P05156	0	0.8333	738.6877	2214.049	1.61	0.00119	9.243632
P05156	0	0.8125	575.8367	1150.666	6.39	0.00368	27.92354
P29622	1	0.8261	470.6457	1409.922	1.43	0.00067	31.3341
P29622	0	0.6216	578.6372	1733.897	2.01	0.00116	0
P29622	0	0.9247	780.7452	2340.221	1.58	0.00123	8.01772
P29622	0	0.6349	867.4511	1733.895	0.89	0.00077	1.333021
P29622	0	0.8302	525.3164	1573.934	2.58	0.00135	12.09627
P29622	0	0.88	394.2394	1573.936	3.35	0.00132	47.2286
P29622	0	0.8793	525.3159	1573.933	1.65	0.00086	10.61154
P29622	0	1	446.2435	1336.716	2.14	0.00095	26.46877
P29622	0	0.6531	639.8701	1278.733	2.53	0.00162	29.13237
P29622	0	0.7297	652.409	1303.811	2.46	0.0016	7.788028
P29622	0	0.8361	658.381	1315.755	3.56	0.00234	37.92939
P29622	0	1	655.5989	2619.374	1.98	0.00129	42.28675
P29622	0	1	691.624	2763.474	1.25	0.00086	5.390159
P29622	0	1	599.0082	1795.01	6.09	0.00365	36.90794
P29622	0	1	920.0116	1839.016	1.44	0.00133	25.15557
P29622	0	0.9231	457.6551	2284.247	1.49	0.00068	4.56257
P29622	0	0.9762	571.8177	2284.249	2.55	0.00146	1.941242
P29622	0	1	541.3156	1621.932	1.15	0.00062	6.293672
P29622	0	0.7143	525.3155	1573.932	0.95	0.0005	23.56716
P29622	0	0.9545	571.8182	2284.251	3.41	0.00195	26.08594
P29622	0	0.6486	867.4524	1733.898	2.37	0.00205	6.558759
P29622	0	0.8846	559.6441	1676.918	2.38	0.00133	27.20576
P29622	0	0.7692	780.7452	2340.221	1.58	0.00123	0
P29622	0	1	541.3168	1621.936	3.29	0.00178	11.76832
P29622	0	0.7561	639.8701	1278.733	2.53	0.00162	46.18365
P29622	0	1	595.5383	2379.131	3.02	0.0018	1.527723
P29622	0	0.8095	379.9126	1137.723	-0.13	-0.00005	45.33997
P29622	0	0.8857	557.793	1114.579	2.24	0.00125	3.224221
P29622	0	1	578.637	1733.897	1.8	0.00104	45.69037
P29622	0	0.9394	668.8612	1336.715	1.6	0.00107	0
P29622	0	0.4167	511.6095	1532.814	1.4	0.00071	4.620315
P29622	0	0.9762	485.7413	970.4754	1.32	0.00064	15.5806
P29622	0	0.7647	379.9133	1137.725	1.72	0.00065	56.82906
P29622	0	0.625	426.915	1278.731	0.76	0.00032	44.48211
P29622	0	0.4375	379.9132	1137.725	1.48	0.00056	6.59517
P29622	0	1	485.7409	970.4745	0.44	0.00021	0
P07225	1	1	489.5554	1955.2	-0.76	-0.00037	52.09441
P07225	1	1	489.557	1955.206	2.42	0.00118	24.16482
P07225	1	1	652.4075	1955.208	3.43	0.00223	3.54352
P07225	1	1	489.5565	1955.204	1.48	0.00073	15.74467
P07225	0	1	548.0277	2189.089	2.02	0.00111	8.093472
P07225	0	1	730.3679	2189.089	2.22	0.00162	7.549598
P07225	0	0.8571	448.2464	1342.725	0.74	0.00033	0
P07225	0	1	726.0372	2176.097	1.49	0.00108	2.268145

P07225	0	0.9091	726.0364	2176.095	0.48	0.00035	0
P07225	0	0.9459	708.0433	2122.115	1.25	0.00088	69.45781
P07225	0	1	662.9539	1986.847	1.5	0.00099	7.562456
P07225	0	1	993.9279	1986.848	2.12	0.00211	2.930743
P07225	0	1	495.5996	1484.784	1.24	0.00061	46.86454
P07225	0	0.5862	425.2441	1273.718	0.1	0.00004	5.363454
P07225	1	1	391.8479	1955.21	4.55	0.00178	4.567848
P07225	0	1	618.5644	2471.236	2.16	0.00133	46.39608
P07225	0	0.3542	636.8674	1272.728	-0.31	-0.0002	54.32155
P07225	0	1	662.954	1986.848	1.68	0.00112	4.724596
P07225	0	1	453.9081	1359.71	1.13	0.00051	28.68501
P07225	0	0.75	548.0267	2189.085	0.35	0.00019	23.54591
P07225	0	1	581.7874	1162.567	2.15	0.00125	4.018451
P07225	1	0.44	453.531	1811.102	1.54	0.0007	0
P07225	1	0.44	453.531	1811.102	1.54	0.0007	0
P07225	0	0.8108	550.8049	1100.603	1.51	0.00083	3.523383
P07225	0	0.6429	436.8947	1308.67	2.34	0.00102	24.9109
P07225	0	0.6327	537.8423	1074.677	1.11	0.00059	6.927693
P07225	0	1	447.566	1340.683	2.17	0.00097	72.39617
P07225	0	0.84	632.02	1894.046	2.8	0.00177	5.218619
P07225	0	1	1030.787	3090.346	3.98	0.0041	0
P07225	0	0.9333	654.838	1308.669	1.76	0.00115	3.647795
P07225	0	0.9444	472.9489	1416.832	1.52	0.00072	33.89864
P07225	0	0.973	637.3641	1273.721	2.53	0.00161	3.731318
P07225	0	1	662.9535	1986.846	0.85	0.00057	69.39865
P07225	0	0.8	947.5237	1894.04	0	0	0
P07225	0	0.125	632.0192	1894.043	1.45	0.00091	3.703587
P07225	1	1	489.556	1955.202	0.36	0.00018	25.0977
P07225	0	1	680.3591	1359.711	1.99	0.00136	23.65166
Q06033	0	0.8333	717.364	1433.721	1.74	0.00124	5.095287
Q06033	0	0.7755	478.5783	1433.72	1.56	0.00074	4.124469
Q06033	0	0.2909	524.337	1047.667	1.4	0.00073	16.05836
Q06033	0	0.9848	674.3638	1347.72	2.44	0.00164	5.13249
Q06033	0	0.8571	449.9106	1347.717	0.14	0.00006	3.799736
Q06033	0	1	596.3308	1786.978	-1.31	-0.00078	27.80802
Q06033	1	1	515.5504	2059.18	1.25	0.00064	7.552632
Q06033	1	1	515.5504	2059.18	1.25	0.00064	27.59592
Q06033	1	1	412.6416	2059.179	0.73	0.0003	14.9729
Q06033	0	1	652.5955	2607.36	2.38	0.00155	15.97357
Q06033	0	0.9792	869.7908	2607.358	1.52	0.00132	7.197168
Q06033	0	0.875	652.5945	2607.356	0.89	0.00058	36.31327
Q06033	0	1	626.9993	1878.983	2.26	0.00142	6.623813
Q06033	0	0.9677	626.9994	1878.984	2.46	0.00154	4.493001
Q06033	0	1	884.1866	3533.725	1.81	0.0016	8.859504
Q06033	1	1	515.5504	2059.18	1.25	0.00064	14.88018
Q06033	0	1	602.3126	1203.618	2.44	0.00147	14.28543
Q06033	0	1	578.9656	1734.882	3.02	0.00175	29.57657
Q06033	0	1	533.6088	1598.812	2.23	0.00119	7.847265
Q06033	0	1	501.2535	1501.746	0.94	0.00047	54.43179
Q06033	0	1	562.8388	1124.67	4.06	0.00228	0
Q06033	0	1	632.6179	2527.45	1.61	0.00101	25.46742
Q06033	0	0.7222	525.8157	1050.624	2.01	0.00105	48.52787

Q06033	1	1	479.5247	1915.077	0.91	0.00043	18.26378
Q06033	1	1	479.5247	1915.077	0.91	0.00043	18.26378
Q06033	0	0.225	524.3376	1047.668	2.56	0.00134	26.33937
Q06033	0	1	406.8902	1218.656	2.11	0.00086	12.48371
Q06033	0	0.9231	412.2218	1234.651	2.15	0.00089	8.826192
Q06033	0	0.9444	406.8904	1218.657	2.71	0.0011	7.366235
Q06033	0	0.4681	490.2879	979.5686	2.42	0.00118	0
Q06033	0	1	406.8902	1218.656	2.26	0.00092	11.81282
Q06033	0	1	406.8903	1218.656	2.49	0.00101	0
Q06033	0	1	375.5609	1124.668	2.17	0.00081	7.676147
Q06033	0	0.3696	452.286	903.5647	1.77	0.0008	8.108923
Q06033	0	1	717.3626	1433.718	-0.22	-0.00016	32.01134
Q06033	1	0.7143	417.9268	1251.766	2.12	0.00089	35.27917
Q06033	0	0.6774	525.8158	1050.624	2.24	0.00118	56.26735
Q06033	0	1	724.6178	2895.449	1.97	0.00142	19.02348
Q06033	0	0.8333	724.6174	2895.448	1.38	0.001	43.64614
P05546	0	0.8983	684.3933	1367.779	1.42	0.00097	5.107193
P05546	0	0.8542	456.5982	1367.78	1.97	0.0009	10.54598
P05546	0	1	422.9582	1688.811	1.45	0.00061	21.37404
P05546	0	1	418.9596	1672.816	1.78	0.00074	20.61712
P05546	0	0.9459	390.8149	1950.045	0.89	0.00035	13.15316
P05546	0	0.9333	650.6873	1950.047	1.97	0.00128	12.74824
P05546	0	1	488.2671	1950.046	1.46	0.00071	17.90572
P05546	0	0.96	390.8151	1950.046	1.44	0.00056	49.93361
P05546	0	0.9474	488.2668	1950.045	0.96	0.00047	7.269555
P05546	0	0.8571	390.8152	1950.047	1.67	0.00065	38.90092
P05546	0	1	650.6874	1950.048	2.06	0.00134	4.116437
P05546	0	1	488.267	1950.046	1.34	0.00065	8.490896
P05546	0	1	390.8153	1950.047	1.91	0.00074	42.34333
P05546	0	0.8571	488.2669	1950.046	1.21	0.00059	31.47655
P05546	0	1	614.8198	1228.632	1.94	0.00119	4.21518
P05546	0	0.8222	456.5979	1367.779	1.3	0.00059	4.294487
P05546	0	1	1300.645	2600.283	1.01	0.00132	0
P05546	0	1	558.2769	1672.816	1.53	0.00085	8.989056
P05546	0	1	418.9593	1672.815	1.12	0.00047	66.43404
P05546	0	1	558.2771	1672.817	1.97	0.0011	31.87263
P05546	0	0.9333	390.8153	1950.047	1.91	0.00074	16.67837
P05546	0	1	754.3737	2261.107	1.31	0.00098	4.960749
P05546	0	0.8596	594.3485	1187.69	1.87	0.00111	5.523003
P05546	0		525.0418	2097.145	2.37	0.00124	30.4408
P05546	0	0.9104	704.9274	1408.847	2.29	0.00161	9.035565
P05546	0	0.831	514.7912	1028.575	1.59	0.00082	1.912749
P05546	0	0.8621	684.3937	1367.78	2.14	0.00146	6.342637
P05546	0	1	517.621	1550.849	2.45	0.00127	24.47589
P05546	0	0.8723	586.842	1172.677	0.97	0.00057	9.827888
P05546	0	0.8537	586.8425	1172.678	1.8	0.00106	8.092008
P05546	0	0.8209	612.343	1223.679	2.9	0.00177	26.70862
P05546	0	1	469.5863	1406.744	1.26	0.00059	8.773459
P05546	0	0.9459	517.6221	1550.852	4.46	0.00231	13.27002
P05546	0	0.7679	522.2977	1043.588	2.73	0.00142	8.028572
P05546	0	0.8387	525.041	2097.142	0.97	0.00051	2.637595
P05546	1	0.9655	390.8892	1170.653	2.5	0.00098	53.49796

P05546	0	0.5873	554.3557	1107.704	2.2	0.00122	11.93278
P05546	0	0.7838	704.9271	1408.847	1.94	0.00137	34.49294
P05546	0	0.7805	684.3939	1367.78	2.32	0.00158	10.88038
P05546	0	0.9	418.9595	1672.816	1.7	0.00071	65.35334
P05546	0	0.88	514.79	1028.573	-0.66	-0.00034	24.05058
P05546	0	1	422.9584	1688.812	2.02	0.00085	20.28531
P05546	0	1	706.3406	2117.007	2.64	0.00186	24.91445
P05546	0	1	542.7686	1084.53	1.88	0.00102	25.37009
P05546	0	0.6154	554.355	1107.703	0.99	0.00055	7.310923
P05546	0	0.8649	586.8435	1172.68	3.57	0.00209	20.9722
P05546	0	1	418.9595	1672.816	1.7	0.00071	50.979
P05546	0	0.8095	594.3494	1187.691	3.41	0.00203	34.46654
P05546	0	0.9032	819.3958	2456.173	-2.01	-0.00164	3.179378
P05546	0	0.5833	554.355	1107.703	0.99	0.00055	6.6423
P05546	0	0.9355	456.5972	1367.777	-0.1	-0.00005	8.576745
P05546	0	0.7778	470.2872	1408.847	1.93	0.00091	9.667555
P05546	0	0.88	632.8763	1264.745	2.56	0.00162	12.10985
P05546	0	0.8571	488.2669	1950.046	1.15	0.00056	13.32102
P05546	0	0.6226	482.3043	963.6013	1.84	0.00089	8.058602
P05546	0	0.5667	391.5641	1172.678	1.84	0.00072	20.48727
P05546	0	1	436.2154	1306.632	2	0.00087	59.08113
P05546	0	0.6538	456.5978	1367.779	1.23	0.00056	34.88422
P05546	0	0.8065	556.8062	1112.605	0.5	0.00028	17.30888
P05546	0	0.6341	537.35	1073.693	0.54	0.00029	9.001741
P05546	0	0.9143	537.7971	1074.587	1.45	0.00078	8.942545
P05546	0	0.6047	537.3502	1073.693	0.88	0.00048	14.5428
P05546	0	1	514.7903	1028.573	-0.19	-0.0001	48.03494
P05546	0	0.7188	594.3494	1187.691	3.41	0.00203	30.62728
P05546	0	0.4138	396.5682	1187.69	2.25	0.00089	12.97514
P05546	0	1	418.9597	1672.817	2.07	0.00087	37.033
P05546	0	1	422.958	1688.81	1.01	0.00043	23.03354
P05546	0	0.7742	537.7969	1074.587	1.23	0.00066	14.1712
P05546	0	1	558.2771	1672.817	1.97	0.0011	38.85274
O75882	0	1	629.9962	1887.974	2.67	0.00168	20.40479
O75882	0	1	629.9967	1887.976	3.45	0.00217	37.64902
O75882	0	1	397.8196	1985.069	5.06	0.00201	9.054237
O75882	0	1	497.0209	1985.062	1.55	0.00077	19.87938
O75882	0	1	497.0212	1985.063	2.23	0.00111	25.03966
O75882	0	0.9821	566.9973	1698.977	1.95	0.0011	0
O75882	0	0.8222	425.4996	1698.977	1.47	0.00063	5.870743
O75882	0	1	667.948	2001.829	1.65	0.0011	51.50071
O75882	0	0.8676	707.8694	1414.731	3.01	0.00213	5.005092
O75882	0	1	432.5839	1295.737	1.7	0.00073	18.61798
O75882	0	0.76	425.4992	1698.975	0.54	0.00023	18.25538
O75882	0	0.8857	635.8181	1270.629	2.98	0.00189	0
O75882	0	0.6809	515.8109	1030.614	1.82	0.00094	100
O75882	0	1	636.8228	1272.638	1.46	0.00093	36.89316
O75882	0	0.9737	746.8445	1492.682	1.02	0.00076	26.07495
O75882	0	0.973	498.2312	1492.679	-0.76	-0.00038	49.05568
O75882	0	1	572.4954	2286.96	1.62	0.00092	4.33848
O75882	0	1	472.9178	1416.739	0.11	0.00005	7.323316
O75882	0	1	472.9185	1416.741	1.59	0.00075	29.52758

O75882	0	1	826.8613	1652.715	1.68	0.00139	3.657782
O75882	0	1	551.5767	1652.715	1.78	0.00098	6.450633
O75882	0	1	826.8612	1652.715	1.61	0.00133	3.96211
O75882	0	1	551.577	1652.716	2.34	0.00129	4.07166
O75882	0	1	826.8613	1652.715	1.75	0.00145	3.485675
O75882	0	1	551.5757	1652.713	0.12	0.00007	17.29018
O75882	0	1	551.5765	1652.715	1.45	0.0008	30.92761
O75882	1	0.6905	432.2879	1294.849	2.55	0.0011	12.82202
O75882	0	0.8485	629.9966	1887.975	3.35	0.00211	23.78763
O75882	0	1	667.9496	2001.834	4.03	0.00269	38.54329
O75882	0	0.5741	557.7854	1114.564	1.49	0.00083	0
O75882	0	1	826.8616	1652.716	2.05	0.00169	0
O75882	0	0.8889	600.989	1800.952	4.48	0.00269	0
O75882	0	1	503.5645	1508.679	2.45	0.00123	32.19097
O75882	0	0.8718	515.8102	1030.613	0.63	0.00033	4.302829
O75882	0	1	447.2598	1339.765	2.69	0.0012	49.29813
O75882	0	0.7674	585.8184	1170.629	4.25	0.00249	6.118416
O75882	0	1	477.5803	1430.726	2.98	0.00142	33.68989
O75882	0	1	472.2478	1414.729	1.21	0.00057	14.18521
O75882	0	1	708.8743	1416.741	1.99	0.00141	12.73052
O75882	0	0.9	648.3741	1295.741	4.53	0.00294	6.079935
O75882	0	0.9714	759.3888	1517.77	3.43	0.0026	17.28933
O75882	0	0.4857	650.4002	1299.793	2.02	0.00131	11.32585
O75882	1	0.7391	432.2885	1294.851	4.03	0.00174	29.20449
O75882	0	0.9643	849.9913	1698.975	0.77	0.00066	0
O75882	0	1	506.5939	1517.767	1.29	0.00065	32.05256
O75882	0	0.7941	715.8666	1430.726	2.61	0.00187	0
O75882	0	1	762.9904	2286.957	0.26	0.0002	0
P01011	0	1	732.7279	2196.169	2.45	0.00179	27.02432
P01011	0	0.9878	727.3644	2180.079	1.66	0.0012	6.330095
P01011	0	0.9412	727.3654	2180.082	3	0.00218	9.782677
P01011	0	0.9111	727.3626	2180.073	-0.86	-0.00063	68.71194
P01011	0	0.6818	639.9624	1917.873	2.94	0.00188	7.27101
P01011	0	0.8507	639.962	1917.872	2.37	0.00152	5.345961
P01011	0	0.8485	639.9616	1917.87	1.7	0.00109	4.447939
P01011	0	0.7027	639.9614	1917.87	1.32	0.00084	5.952508
P01011	0	0.8571	461.618	1382.839	2.67	0.00123	6.237031
P01011	0	0.7667	480.7598	960.5123	1.43	0.00068	0
P01011	0	0.2041	646.3469	1937.026	2.51	0.00162	4.420014
P01011	0	0.8605	732.7282	2196.17	2.78	0.00204	0
P01011	0	0.6	646.3474	1937.028	3.18	0.00205	57.40021
P01011	0	0.4154	646.3469	1937.026	2.51	0.00162	8.768698
P01011	0	0.4773	969.0175	1937.028	3.31	0.0032	11.5645
P01011	0	0.8704	1098.588	2196.168	2.04	0.00224	7.102388
P01011	0	0.9806	732.7275	2196.168	1.95	0.00142	5.536043
P01011	0	0.377	646.3469	1937.026	2.42	0.00156	11.68077
P01011	0	1	732.7278	2196.169	2.28	0.00167	10.44922
P01011	0	0.2353	646.3472	1937.027	2.89	0.00187	11.58224
P01011	0	0.4773	646.3459	1937.023	0.91	0.00059	0
P01011	0	0.5738	646.3471	1937.027	2.8	0.00181	8.053593
P01011	0	0.8	481.0129	1921.03	1.69	0.00081	17.55138
P01011	0	0.9604	961.0207	1921.034	3.99	0.00384	11.11899

P01011	0	0.8769	641.0154	1921.032	2.78	0.00178	9.924909
P01011	0	0.1818	646.3487	1937.032	5.26	0.00339	0
P01011	0	0.5	646.3474	1937.028	3.18	0.00205	7.485449
P01011	0	0.2619	646.3468	1937.026	2.33	0.0015	19.36611
P01011	0	0.2683	646.3455	1937.022	0.34	0.00022	0
P01011	0	0.1111	646.3491	1937.033	5.92	0.00382	0
P01011	0	0.2162	646.3472	1937.027	2.99	0.00193	58.01707
P01011	0	0.1364	646.3477	1937.028	3.65	0.00236	13.72743
P01011	0	0.2903	646.3494	1937.034	6.39	0.00413	4.94283
P01011	0	0.4068	646.3465	1937.025	1.85	0.0012	5.867637
P01011	0	0.24	646.3467	1937.026	2.23	0.00144	4.32307
P01011	0	0.9143	549.7972	2196.167	1.52	0.00084	10.23917
P01011	0	0.6562	646.3472	1937.027	2.99	0.00193	24.55198
P01011	0	0.9732	1098.587	2196.167	1.59	0.00175	0
P01011	0	0.9806	732.7261	2196.164	0.03	0.00002	5.156268
P01011	0	0.4737	646.3469	1937.026	2.51	0.00162	2.307965
P01011	0	0.9683	727.361	2180.068	-3.05	-0.00221	45.86599
P01011	0	0.9775	732.728	2196.169	2.53	0.00185	29.4907
P01011	0	0.625	646.3466	1937.025	2.04	0.00132	0
P01011	0	0.9709	732.7277	2196.168	2.11	0.00155	4.90298
P01011	0	0.9709	732.7283	2196.17	3.03	0.00222	5.212932
P01011	0	0.9806	732.7274	2196.168	1.78	0.0013	4.810615
P01011	0	0.9074	727.3659	2180.083	3.76	0.00273	63.1729
P01011	0	0.9907	732.7275	2196.168	1.86	0.00136	6.13323
P01011	0	0.9231	727.3684	2180.091	7.2	0.00523	8.941708
P01011	0	0.8718	727.3637	2180.076	0.65	0.00047	13.91943
P01011	0	0.8889	727.3648	2180.08	2.24	0.00163	7.874072
P01011	0	0.8056	727.3655	2180.082	3.17	0.0023	22.79355
P01011	0	0.902	727.3649	2180.08	2.41	0.00175	23.50187
P01011	0	0.9355	727.3643	2180.078	1.49	0.00108	21.47308
P01011	0	0.9138	727.3662	2180.084	4.18	0.00304	4.732151
P01011	0	0.8864	727.3658	2180.083	3.59	0.00261	22.91838
P01011	0	0.8841	732.728	2196.17	2.61	0.00191	14.28222
P01011	0	0.873	732.7272	2196.167	1.53	0.00112	7.208468
P01011	0	0.9178	732.7252	2196.161	-1.31	-0.00096	28.62843
P01011	0	0.9296	732.725	2196.161	-1.47	-0.00108	32.45002
P01011	0	1	732.7276	2196.168	2.03	0.00149	0
P01011	0	0.9077	732.7289	2196.172	3.78	0.00277	17.94738
P01011	0	0.963	732.7278	2196.169	2.36	0.00173	10.48346
P01011	0	0.956	732.7279	2196.169	2.45	0.00179	13.35351
P01011	0	0.956	732.7275	2196.168	1.95	0.00142	12.45677
P01011	0	0.9697	732.7279	2196.169	2.45	0.00179	5.133433
P01011	0	0.9091	727.3635	2180.076	0.48	0.00035	8.529231
P01011	0	0.8871	732.7271	2196.167	1.28	0.00094	8.839244
P01011	0	0.8824	732.7262	2196.164	0.11	0.00008	23.46121
P01011	0	0.8864	727.3653	2180.081	2.92	0.00212	58.99309
P01011	0	0.9677	732.7281	2196.17	2.7	0.00197	5.998935
P01011	0	0.9348	732.7287	2196.172	3.53	0.00258	0
P01011	1	0.9538	779.3978	2336.179	1.22	0.00095	5.520219
P01011	1	0.9783	779.3947	2336.17	-2.78	-0.00217	39.36861
P01011	0	0.8846	675.401	1349.795	2.12	0.00143	6.799006
P01011	0	0.8929	675.4013	1349.795	2.48	0.00167	6.737877

P01011	0	0.8833	675.4005	1349.794	1.4	0.00094	18.78126
P01011	0	1	727.3643	2180.078	1.57	0.00114	10.39135
P01011	0	1	727.3644	2180.079	1.74	0.00127	9.483211
P01011	0	0.9867	727.3644	2180.079	1.66	0.0012	100
P01011	0	1	727.3643	2180.078	1.49	0.00108	18.07335
P01011	0	1	727.36	2180.065	-4.39	-0.00319	13.49717
P01011	0	0.9747	727.3648	2180.08	2.16	0.00157	6.657966
P01011	0	0.8974	727.3633	2180.075	0.15	0.00011	24.10193
P01011	0	0.8936	727.3638	2180.077	0.82	0.00059	30.60896
P01011	0	0.9524	727.3652	2180.081	2.75	0.002	9.344493
P01011	0	0.95	727.3641	2180.078	1.24	0.0009	6.835411
P01011	0	0.9565	727.3647	2180.08	2.08	0.00151	6.192315
P01011	0	0.9577	727.3682	2180.09	6.95	0.00505	9.001811
P01011	0	0.9524	727.3657	2180.083	3.5	0.00255	7.989721
P01011	0	0.9636	727.3679	2180.089	6.44	0.00468	30.03227
P01011	0	0.7778	552.8108	1104.614	1.23	0.00068	3.689874
P01011	0	0.875	461.618	1382.839	2.67	0.00123	51.83607
P01011	1	0.6458	475.6341	1424.888	2.83	0.00134	15.32195
P01011	0	0.8431	461.6179	1382.839	2.33	0.00108	59.2453
P01011	1	0.6579	475.6332	1424.885	1.03	0.00049	11.64417
P01011	0	0.9306	691.9236	1382.84	2.96	0.00205	7.514454
P01011	0	0.875	461.6173	1382.837	1.21	0.00056	10.50876
P01011	0	0.7556	552.8118	1104.616	3.11	0.00172	15.82358
P01011	0	0.78	461.6173	1382.837	1.14	0.00053	24.20318
P01011	0	0.9691	732.7285	2196.171	3.2	0.00234	6.700497
P01011	0	0.974	727.3649	2180.08	2.33	0.00169	5.278649
P01011	0	0.9677	732.7282	2196.17	2.86	0.0021	3.910068
P01011	0	0.971	727.3649	2180.08	2.41	0.00175	2.219801
P01011	0	0.931	727.3662	2180.084	4.18	0.00304	9.922313
P01011	0	0.8936	727.3643	2180.078	1.57	0.00114	3.000642
P01011	0	0.8767	641.0156	1921.032	2.97	0.0019	5.808604
P01011	0	0.9091	961.0204	1921.033	3.68	0.00353	19.02673
P01011	0	0.907	461.6178	1382.839	2.27	0.00105	45.93854
P01011	0	0.775	639.9617	1917.87	1.8	0.00115	31.56483
P01011	0	0.9302	727.3646	2180.079	1.99	0.00145	9.477134
P01011	0	0.9531	727.3594	2180.064	-5.23	-0.0038	12.40727
P01011	1	0.9615	683.9828	3415.885	1.51	0.00103	12.98392
P01011	0	0.7333	570.6362	1709.894	4.2	0.00239	9.848882
P01011	1	0.0909	818.7027	3271.789	3.47	0.00284	80.72858
P01011	0	1	608.3701	1215.733	1.85	0.00112	30.99804
P01011	0	1	838.4446	2513.319	-3.37	-0.00282	45.07218
P01011	0	0.9604	855.4495	1709.892	2.74	0.00234	7.78426
P01011	0	0.8627	570.6356	1709.892	3.02	0.00172	11.24312
P01011	0	1	1257.171	2513.335	2.95	0.00371	0
P01011	0	0.9808	783.4017	1565.796	7.21	0.00565	6.032496
P01011	0	1	680.4213	1359.835	1.91	0.0013	4.081489
P01011	0	0.8125	680.4214	1359.836	2	0.00136	26.18096
P01011	0	0.9206	680.4211	1359.835	1.55	0.00105	18.55902
P01011	0	0.898	453.95	1359.835	1.92	0.00087	6.862297
P01011	0	0.9344	680.4215	1359.836	2.09	0.00142	4.949018
P01011	0	0.8868	680.4219	1359.837	2.81	0.00191	7.349617
P01011	0	0.9062	862.1312	2584.379	1.58	0.00137	4.554194

P01011	0	0.9143	1292.694	2584.382	2.63	0.0034	0
P01011	0	1	1176.617	2352.227	1.89	0.00222	1.434845
P01011	0	1	784.7468	2352.226	1.52	0.00119	5.503526
P01011	0	1	983.2015	2947.59	2.8	0.00275	4.112854
P01011	0	0.9	737.6525	2947.588	2.17	0.0016	3.816468
P01011	0	0.6667	646.3487	1937.032	5.26	0.00339	4.58301
P01011	0	0.75	691.9226	1382.838	1.46	0.00101	7.544816
P01011	0	0.6098	559.322	1117.637	3.06	0.00171	14.97183
P01011	0	0.8696	679.3308	2035.978	2.44	0.00166	21.37796
P01011	0	0.5484	576.8489	1152.691	3.41	0.00196	7.310606
P01011	0	0.6364	684.6942	2052.068	3.01	0.00206	41.55156
P01011	0	0.8387	727.3648	2180.08	2.24	0.00163	11.10891
P01011	0	0.8333	732.7275	2196.168	1.86	0.00136	5.73735
P01011	1	0.8947	848.9765	3392.884	2.21	0.00188	4.747308
P01011	0	0.8065	413.5836	1238.736	2	0.00083	51.57315
P01011	0	0.9756	783.4021	1565.797	7.68	0.00601	36.22265
P01011	0	0.7561	450.6032	1349.795	2.25	0.00101	10.72296
P01011	0	0.5238	552.8117	1104.616	2.78	0.00153	0
P01011	0	0.9	545.7752	2180.079	1.85	0.00101	9.96628
P01011	0	0.8857	732.7275	2196.168	1.86	0.00136	8.462298
P01011	0	0.871	727.3668	2180.086	4.93	0.00358	9.234404
P01011	0	0.88	480.2226	1917.868	0.74	0.00036	3.580239
P01011	0	0.8889	549.7979	2196.17	2.75	0.00151	47.22902
P01011	0	0.913	727.3632	2180.075	-0.02	-0.00002	59.61802
P01011	0	0.5714	559.3209	1117.634	0.98	0.00055	3.94251
P01011	0	0.5526	552.812	1104.617	3.44	0.0019	12.99882
P01011	0	0.9429	727.3608	2180.068	-3.3	-0.0024	0
P01011	0	0.3939	732.7288	2196.172	3.61	0.00265	5.816994
P01011	1	0.7647	779.3991	2336.183	2.78	0.00217	1.928383
P01011	0	0.7895	675.4005	1349.794	1.31	0.00088	29.49121
P01011	0	0.973	691.9251	1382.843	5.08	0.00351	0
P01011	0	0.9091	727.3637	2180.076	0.65	0.00047	55.27946
P01011	0	0.9111	603.3504	1205.694	3.09	0.00186	3.656434
P01011	0	0.7273	727.3648	2180.08	2.24	0.00163	4.636286
P01011	0	0.75	461.6142	1382.828	-5.54	-0.00256	67.44629
P01011	0	0.8065	727.3663	2180.084	4.26	0.0031	69.36417
P01011	0	0.8214	450.6019	1349.791	-0.47	-0.00021	58.21704
P01011	0	0.7455	480.7582	960.509	-1.94	-0.00093	18.37774
P01011	0	0.8387	1098.59	2196.172	3.7	0.00407	1.41029
P01011	0	0.5217	547.8198	1094.632	0.53	0.00029	0
P01011	0	0.4667	592.9817	1776.931	3.46	0.00205	8.745883
P01011	0	0.6818	727.3641	2180.078	1.32	0.00096	9.722131
P01011	0	0.68	727.3658	2180.083	3.67	0.00267	4.955748
P01011	0	0.6667	727.3644	2180.079	1.74	0.00127	0
P01011	0	0.72	727.364	2180.077	1.07	0.00078	8.431595
P01011	0	0.3077	646.3475	1937.028	3.46	0.00223	0
P01011	0	0.7391	727.3669	2180.086	5.18	0.00377	6.854796
P01011	0	1	639.9608	1917.868	0.37	0.00023	5.430933
P01011	0	0.6842	576.849	1152.691	3.51	0.00203	35.08983
P01011	0	0.7778	732.7262	2196.164	0.11	0.00008	24.11256
P01011	0	0.4516	691.9232	1382.839	2.34	0.00162	7.880322
P01011	0	0.9167	727.3641	2180.078	1.32	0.00096	15.89617

P01011	0	0.4286	727.3633	2180.075	0.15	0.00011	58.01072
P01011	0	0.5769	504.7972	1008.587	2.45	0.00124	9.381697
P01011	0	1	645.2933	1933.865	1.83	0.00118	13.65664
P01011	0	0.7188	675.4021	1349.797	3.75	0.00253	39.30369
P01011	0	0.5385	522.6028	1565.794	5.8	0.00303	12.50836
P01011	0	0.5833	576.8491	1152.691	3.73	0.00215	27.61102
P01011	0	0.3488	576.8499	1152.692	5	0.00288	0
P01011	0	0.3846	855.4499	1709.893	3.24	0.00277	0
P01011	0	0.6	675.403	1349.799	5.1	0.00344	18.30736
P01011	0	0.359	576.8488	1152.69	3.09	0.00178	38.54438
P01011	0	0.6154	619.8718	1238.736	2.13	0.00132	0
P01011	0	0.7647	603.3508	1205.694	3.8	0.00229	27.74755
P01011	0	1	645.2944	1933.869	3.44	0.00221	0
P01011	0	1	959.4399	1917.872	2.86	0.00274	0
P01011	0	0.7714	680.4222	1359.837	3.26	0.00221	46.98319
P01011	0	0.4783	675.4006	1349.794	1.58	0.00106	30.54335
P01011	0	0.2308	732.7273	2196.167	1.61	0.00118	5.95366
P01011	0	0.9	732.7267	2196.166	0.78	0.00057	17.02778
P01011	0	0.3333	384.9011	1152.689	1.7	0.00065	5.01408
P01011	0	1	1090.545	2180.082	3.26	0.00355	0
P01011	0	0.2273	969.0176	1937.028	3.37	0.00327	11.49991
P08697	0	0.9841	620.9963	1860.974	2.73	0.00169	7.839658
P08697	0	1	459.9076	1377.708	1.16	0.00053	24.45631
P08697	0	1	747.7625	2241.273	1.58	0.00118	4.178572
P08697	0	1	408.8752	1224.611	1.84	0.00075	11.05856
P08697	0	0.9531	459.9077	1377.709	1.5	0.00069	11.22103
P08697	0	1	689.3569	1377.707	0.04	0.00003	3.31067
P08697	0	0.9459	459.9078	1377.709	1.76	0.00081	39.67487
P08697	0	0.9762	808.7187	2424.142	-1.2	-0.00097	6.840429
P08697	0	1	808.7206	2424.147	1.14	0.00092	1.0388
P08697	1	1	1147.98	3441.926	4.24	0.00486	0
P08697	0	1	806.4486	2417.331	2.19	0.00176	4.455618
P08697	0	1	605.0864	2417.324	-0.87	-0.00053	22.27456
P08697	0	1	758.4125	2273.223	-0.46	-0.00035	4.778725
P08697	0	1	1209.167	2417.326	-0.1	-0.00013	0
P08697	1	0.92	507.9724	1521.903	1.77	0.0009	31.3874
P08697	0	1	557.5633	2227.231	2.85	0.00159	2.113505
P08697	0	1	557.5609	2227.222	-1.54	-0.00086	43.05179
P08697	0	1	743.0809	2227.228	1.37	0.00102	53.70806
P08697	0	1	717.6367	2867.525	1.56	0.00112	3.556955
P08697	0	1	956.5137	2867.526	2.05	0.00196	1.590074
P08697	1	1	602.3309	1804.978	0.87	0.00052	14.54232
P08697	1	1	452.0002	1804.979	1.41	0.00063	13.73543
P08697	0	0.95	534.3026	1600.893	2.14	0.00114	13.17543
P08697	0	0.9853	534.3022	1600.892	1.45	0.00077	10.64095
P08697	0	0.9038	645.8314	1290.656	2.14	0.00138	6.25425
P08697	0	0.9524	808.7217	2424.15	2.5	0.00202	2.095165
P08697	0	0.9821	617.3068	1233.606	1.54	0.00095	7.332612
P08697	0	0.9375	747.7647	2241.279	4.52	0.00338	25.70563
P08697	0	0.7111	569.8397	1138.672	0.9	0.00051	10.01029
P08697	0	0.85	743.0811	2227.229	1.62	0.0012	1.427455
P08697	0	0.9455	426.7201	852.433	0.86	0.00036	16.26723

P08697	0	0.8438	930.9914	1860.976	3.29	0.00306	0
P08697	1	0.0303	450.5816	1349.73	0.59	0.00026	7.403651
P08697	0	0.7632	554.3007	1107.594	2.31	0.00128	12.01888
P08697	0	1	569.8389	1138.67	-0.49	-0.00028	12.47715
P08697	0	1	408.8751	1224.611	1.39	0.00057	34.50393
P08697	1		450.5816	1349.73	0.59	0.00026	7.403651
P08697	1	1	498.6156	1493.832	0.48	0.00024	3.019221
P08697	0	0.975	617.3097	1233.612	6.19	0.00382	76.93299
P08697	0	0.7045	539.3314	1077.656	0.86	0.00046	22.85773
P08697	0	1	708.0306	2122.077	0.22	0.00016	12.51516
P08697	0	0.8605	498.7713	996.5353	1.03	0.00051	1.454245
P08697	0	0.5789	534.3027	1600.893	2.37	0.00126	7.435753
P08697	0	0.8889	620.9967	1860.976	3.32	0.00206	32.792
P08697	0	0.8095	569.8411	1138.675	3.36	0.00192	8.838999
P08697	0	1	408.8759	1224.613	3.41	0.00139	47.78581
P08697	0	0.66	539.332	1077.657	1.88	0.00101	6.402196
P08697	0	0.9737	554.2999	1107.592	0.88	0.00049	6.456849
P02748	0	0.8971	721.3851	1441.763	2.05	0.00148	28.50583
P02748	0	0.8533	721.3855	1441.764	2.64	0.0019	13.72103
P02748	0	0.7143	481.2588	1441.762	1.32	0.00063	7.952652
P02748	1	1	661.3207	2642.261	2.66	0.00176	4.516679
Q96BY6; P0	1	0.6429	444.2751	1330.811	0.93	0.00041	11.91255
P02748	0	0.8478	585.3345	1753.989	3.58	0.0021	3.53109
P02748	0	0.88	585.3341	1753.988	2.96	0.00173	8.223726
P02748	0	0.8605	585.3344	1753.989	3.38	0.00197	0
Q96BY6; P0	1	0.6429	444.2752	1330.811	1.06	0.00047	11.27259
P02748	0	0.8235	655.3669	1309.726	2.49	0.00163	35.55614
P02748	0	0.9024	485.2819	1453.831	3.95	0.00191	16.73701
P02748	0	0.7556	485.2805	1453.827	1.18	0.00057	7.262913
P02748	0	0.9103	800.4114	1599.816	1.31	0.00105	4.19293
P02748	0	1	800.4117	1599.816	1.62	0.00129	0
P02748	0	0.9762	510.9893	1530.953	2.32	0.00118	9.499393
P02748	1	0.907	506.5877	1517.748	1.26	0.00064	8.212698
P02748	0	1	604.6417	1811.911	2.89	0.00174	11.40588
P02748	0	0.9608	765.9802	1530.953	2.23	0.00171	10.22955
P02748	0	1	604.6416	1811.91	2.68	0.00162	8.477773
P02748	0	0.973	604.6419	1811.911	3.19	0.00193	12.34804
P02748	0	0.8571	588.3236	1175.64	0.84	0.0005	3.074739
P02748	0	1	732.3498	2195.035	1.71	0.00125	4.295065
P02748	0	0.9796	622.3576	1865.058	3.18	0.00198	4.854928
P02748	0	1	622.357	1865.056	2.2	0.00137	8.400503
P02748	0	1	1031.071	3091.198	2.97	0.00306	0
P02748	0	0.74	605.8527	1210.698	4.87	0.00295	49.50336
P02748	0	0.95	622.357	1865.056	2.2	0.00137	22.77367
P02748	0	1	1031.069	3091.192	1.08	0.00111	1.76024
P02748	0	1	1031.069	3091.194	1.55	0.0016	2.476987
P02748	0	0.8776	588.3254	1175.644	4.06	0.00239	5.735367
P02748	0	0.5217	467.0195	1865.056	2	0.00093	8.701584
P02748	0	0.9048	623.8138	1246.62	1.49	0.00093	19.21581
P02748	0	1	549.5145	2195.036	2.37	0.0013	53.77746
P02748	0	0.5333	877.4957	1753.984	0.84	0.00074	23.87137
P02748	0	0.7609	613.3387	1225.67	2.8	0.00171	16.93781

P02748	1	0.5135	627.3484	1880.031	3.22	0.00202	59.19175
P02748	0	1	556.6071	1667.807	2.08	0.00116	18.06947
P02748	0	1	516.2722	1031.537	0.38	0.0002	2.426755
P02748	0	0.6	585.3356	1753.992	5.57	0.00326	6.639752
P02748	0	0.7742	392.5518	1175.641	1.69	0.00066	3.590029
P02748	0	1	541.2862	1081.565	0.47	0.00026	0
P02748	0	0.6944	649.3345	1297.662	2.94	0.00191	28.59999
P02748	0	1	551.762	1102.517	0.26	0.00014	13.45381
P02748	0	0.9143	672.3293	1343.651	0.05	0.00003	9.411049
P02748	0	1	773.5555	3091.2	3.66	0.00283	14.69446
P02748	0	0.92	510.9885	1530.951	0.82	0.00042	3.92548
P02748	0	0.9355	727.419	1453.831	3.75	0.00272	28.40584
P02748	0	0.5	463.2709	925.5345	0.97	0.00045	14.30839
P02748	0	0.9474	516.2721	1031.537	0.14	0.00007	20.68933
P02748	0	1	448.5568	1343.656	3.42	0.00153	38.84637
P02748	0	0.8696	877.497	1753.987	2.3	0.00202	24.86744
P05155	0	0.7808	813.9149	1626.823	1.63	0.00133	27.45401
P05155	0	0.75	630.8521	1260.697	4.15	0.00262	34.43736
P05155	0	1	502.9379	1506.799	0.9	0.00045	16.22123
P05155	0	0.9701	753.9038	1506.8	1.76	0.00132	6.8633
P05155	0	1	502.9377	1506.799	0.59	0.0003	9.515435
P05155	0	0.2308	678.3734	1355.74	0.86	0.00058	7.427076
P05155	0	1	502.9385	1506.801	2.05	0.00103	11.18597
P05155	0	0.8571	553.8557	1106.704	1.54	0.00085	17.3296
P05155	1	0.9388	498.7966	1992.164	0.11	0.00006	7.109019
P05155	1	1	498.7969	1992.166	0.78	0.00039	15.6536
P05155	0	1	776.9479	1552.889	3.95	0.00306	34.64158
P05155	0	0.7925	518.3001	1552.886	2.01	0.00104	8.647229
P05155	0	0.9861	776.9457	1552.884	1.04	0.00081	6.178887
P05155	0	0.9375	518.2991	1552.883	0.24	0.00013	11.20672
P05155	0	0.8621	573.9962	1719.974	4.01	0.0023	4.546702
P05155	0	0.9672	573.9957	1719.972	3.05	0.00175	8.324049
P05155	0	0.94	609.6642	1826.978	1.14	0.00069	40.27356
P05155	0	1	657.6989	1971.082	2.13	0.0014	70.74886
P05155	1	1	664.7274	1992.168	1.74	0.00116	48.67271
P05155	1	1	498.7972	1992.167	1.46	0.00073	6.137949
P05155	1	1	498.7969	1992.166	0.78	0.00039	11.33153
P05155	1	1	664.7276	1992.168	2.02	0.00134	6.572153
P05155	1	1	498.7971	1992.167	1.21	0.0006	9.56251
P05155	1	0.9286	664.7276	1992.168	2.02	0.00134	13.71631
P05155	1	0.9762	498.7975	1992.168	2.01	0.001	10.88901
P05155	1	0.9787	664.728	1992.169	2.57	0.00171	19.56452
P05155	1	1	498.7977	1992.169	2.32	0.00115	6.897486
P05155	1	1	664.7277	1992.169	2.2	0.00146	8.604988
P05155	1	1	498.7972	1992.167	1.4	0.0007	5.554324
P05155	1	1	725.7204	2175.147	-0.18	-0.00013	5.538439
P05155	0	0.8868	633.3319	1897.981	-0.49	-0.00031	45.75161
P05155	0	0.8913	633.3351	1897.991	4.72	0.00298	
P05155	0	0.5918	596.312	1786.921	2.47	0.00147	9.017102
P05155	0	0.5263	596.3126	1786.923	3.5	0.00208	8.668063
P05155	0	0.9892	941.4992	1881.991	2.13	0.002	5.764037
P05155	0	1	628.0012	1881.989	1.11	0.0007	9.961451

P05155	0	0.9592	628.0028	1881.994	3.55	0.00223	21.20113
P05155	0	0.9701	704.8952	1408.783	1.93	0.00136	100
P05155	0	0.6364	590.9809	1770.928	3.48	0.00206	9.38533
P05155	0	1	803.514	1606.021	1.63	0.00131	54.42621
P05155	0	1	665.405	1329.803	1.19	0.00079	3.418461
P05155	0	0.9655	657.6988	1971.082	1.95	0.00128	4.940819
P05155	0	1	657.6975	1971.078	-0.1	-0.00006	4.593298
P05155	0	0.9778	803.5136	1606.02	1.1	0.00088	8.854333
P05155	0	1	657.6995	1971.084	3.06	0.00201	5.545911
P05155	1	1	935.264	3738.034	1.89	0.00177	22.93683
P05155	0	0.9643	558.7997	1116.592	2.4	0.00134	2.467804
P05155	0	0.8696	553.8552	1106.703	0.66	0.00036	9.413018
P05155	0	0.7442	420.9031	1260.695	2.45	0.00103	3.548035
P05155	0	1	452.5848	1355.74	1.16	0.00052	33.89859
P05155	0	0.7111	630.8511	1260.695	2.7	0.0017	1.952793
P05155	0	1	502.9382	1506.8	1.56	0.00079	23.17642
P05155	0	1	502.9386	1506.801	2.29	0.00115	10.60185
P05155	1	1	616.696	1848.073	6.08	0.00374	47.06972
P05155	1	1	616.696	1848.073	6.08	0.00374	47.06972
P05155	0	0.9697	628.0021	1881.992	2.57	0.00161	6.722567
P05155	0	0.7692	377.4554	1506.8	1.5	0.00056	24.43747
P05155	0	1	493.5257	1971.081	1.51	0.00074	47.32162
P05155	0	0.7209	630.8511	1260.695	2.7	0.0017	8.381699
P05155	1	0.72	462.7719	1848.066	2.04	0.00094	28.85786
P05155	1	0.72	462.7719	1848.066	2.04	0.00094	28.85786
P05155	0	1	502.9379	1506.799	0.9	0.00045	20.95833
P05155	0	0.95	579.9674	1737.887	1.46	0.00085	4.890219
P05155	1	0.9167	498.7984	1992.172	3.85	0.00192	42.96174
P05155	0	1	454.9042	1362.698	1.85	0.00084	7.62984
P05155	0	0.8913	593.3541	1185.701	1.65	0.00098	12.90112
P05155	0	0.8462	628.0024	1881.993	3.06	0.00192	9.43808
P05155	0	0.8065	408.5546	1223.649	0.59	0.00024	8.87458
P05155	0	0.92	518.3002	1552.886	2.37	0.00122	46.51334
P05155	0	0.9143	454.9046	1362.699	2.59	0.00118	10.32163
P05155	0	0.9048	612.3295	1223.652	2.57	0.00157	15.76263
P05155	0	0.7407	408.555	1223.651	1.64	0.00067	5.195457
P05155	0	0.7297	704.8955	1408.784	2.37	0.00167	6.081885
P05155	0	0.9524	606.3232	1211.639	2.38	0.00144	20.57968
P05155	0	1	502.9376	1506.798	0.41	0.00021	35.68925
P05155	0	0.9216	455.7354	910.4634	0.61	0.00028	4.795763
P05155	0	0.8889	797.1044	2389.299	0.85	0.00067	3.498694
P05155	0	0.9615	573.9966	1719.975	4.65	0.00266	26.31799
P05155	0	0.7778	630.851	1260.695	2.41	0.00152	34.86462
P05155	0	1	413.8869	1239.646	2.27	0.00094	13.30621
P05155	0	0.8889	427.2237	853.4401	1.34	0.00057	0
P05155	0	0.7442	630.8501	1260.693	1.05	0.00066	3.153018
P05155	1	0.8636	664.731	1992.179	7.16	0.00476	0
P05155	0	1	657.6984	1971.081	1.39	0.00091	11.03835
P05155	1	1	412.2425	1234.713	1.96	0.00081	15.56115
P05155	0	0.8571	518.2988	1552.882	-0.35	-0.00018	27.90638
P05155	0	0.9355	493.5274	1971.088	4.97	0.00245	40.7527
P05155	0	0.8571	547.8005	1094.594	-0.21	-0.00012	31.73866

P05155	0	0.8571	558.7996	1116.592	2.18	0.00122	63.81391
P05155	0	1	502.9384	1506.801	1.99	0.001	10.98347
P05155	0	0.875	408.5553	1223.651	2.39	0.00097	10.37905
P05155	0	0.7857	869.4476	1737.888	1.75	0.00152	4.719529
P05155	0	1	536.0128	1606.024	3.45	0.00185	3.772555
P05155	0	0.7037	408.5558	1223.653	3.58	0.00146	11.29998
P05155	0	0.8974	455.7358	910.4643	1.61	0.00074	31.77118
P05155	1	1	725.7156	2175.132	-6.75	-0.00489	17.1963
P05155	0	0.6429	633.3344	1897.989	3.56	0.00225	36.11507
P05155	0	0.4444	454.9038	1362.697	0.84	0.00038	55.81579
P05155	0	1	869.4512	1737.895	5.89	0.00512	0
P05155	0	0.9592	455.7362	910.4651	2.49	0.00113	27.91975
P05155	0	1	986.0445	1971.082	1.89	0.00186	2.959084
P05155	0	0.4545	518.3004	1552.887	2.72	0.00141	23.34968
P05155	0	0.9535	455.7355	910.4637	0.94	0.00043	9.749778
P00736	0	0.95	511.0303	2041.099	3.38	0.00172	27.90041
P00736	0	1	961.4528	2882.344	-0.06	-0.00005	0
P00736	0	0.9318	617.9728	1851.904	3.33	0.00205	10.13322
P00736	0	0.8947	617.9717	1851.9	1.45	0.00089	20.09036
P00736	0	1	924.7883	2772.35	1.27	0.00117	8.43507
P00736	0	1	906.4727	1811.938	2.13	0.00193	0
P00736	0	1	604.6511	1811.939	2.39	0.00144	3.331318
P00736	0	1	606.327	1816.967	2.6	0.00158	0.160225
P00736	0	0.9615	454.9956	1816.961	-0.6	-0.00027	6.288332
P00736	0	1	826.6499	3303.578	1.7	0.0014	2.62586
P00736	0	1	708.0069	2122.006	1.4	0.00099	2.711348
P00736	0	1	708.0074	2122.008	2.09	0.00148	33.77083
P00736	0	0.975	757.3685	3026.452	2.1	0.00159	20.5592
P00736	0	1	1009.489	3026.452	2.1	0.00212	2.933997
P00736	0	1	924.7891	2772.353	2.13	0.00197	12.56448
P00736	0	0.9322	693.8444	2772.356	3.14	0.00217	7.017009
P00736	1	1	500.9468	1500.826	3.55	0.00178	73.07345
P00736	0	1	649.9982	1947.98	1.6	0.00104	15.28736
P00736	0	1	649.9985	1947.981	2.07	0.00135	21.4896
Q9NZP8; PC	0	0.6491	480.274	959.5408	1.78	0.00085	8.877599
P00736	0	0.9318	617.9724	1851.903	2.64	0.00163	6.600594
P00736	0	0.898	653.3421	1305.677	1.38	0.0009	46.35346
P00736	0	0.8429	800.0894	2398.254	0.5	0.0004	0
P00736	0	0.9783	715.69	2145.055	1.39	0.001	5.288332
P00736	0	1	568.0043	1701.998	3.08	0.00175	14.89096
P00736	0	0.9434	568.0041	1701.998	2.76	0.00156	11.80002
P00736	0	0.8485	715.6907	2145.057	2.33	0.00167	8.851177
P00736	0	0.5581	535.3534	1069.7	2.18	0.00117	12.6987
P00736	0	0.9394	522.9417	1566.81	1.79	0.00094	14.56185
P00736	0	1	604.6517	1811.941	3.5	0.00212	12.41443
P00736	0	0.625	719.4215	1437.836	0.85	0.00061	13.78449
P00736	0	0.8947	562.3063	1123.605	2.37	0.00133	100
P00736	0	1	708.0064	2122.004	0.62	0.00044	6.306197
P00736	0	0.64	506.298	1011.589	4.41	0.00223	19.29176
P00736	0	0.6	404.8818	1212.631	1.87	0.00076	13.69727
P00736	0	1	568.0045	1701.999	3.51	0.00199	9.614187
P00736	0	1	558.8004	1116.594	1.55	0.00087	0

P00736	0	0.6667	653.341	1305.675	-0.3	-0.00019	32.8207
P00736	0	0.875	568.0026	1701.993	0.07	0.00004	20.91151
P02649	0	1	547.9735	1641.906	1.07	0.00058	63.88305
P02649	0	1	484.7795	968.5518	-0.57	-0.00028	0
P02649	0	1	768.8624	1536.717	1.51	0.00116	0.67405
P02649	0	0.9882	768.8618	1536.716	0.71	0.00055	0
P02649	0	0.8916	597.974	1791.907	2.63	0.00157	5.035113
P02649	0	0.9643	896.4563	1791.905	1.45	0.0013	3.264533
P02649	0	0.9841	597.9753	1791.911	4.78	0.00286	58.71581
P02649	0	0.7347	589.3262	1177.645	0.52	0.0003	3.096913
P02649	0	0.9278	547.9735	1641.906	1.07	0.00058	4.750529
P02649	0	1	821.4564	1641.906	0.93	0.00076	3.390358
P02649	0	0.5741	609.8599	1218.712	3.27	0.00199	7.322783
P02649	0	0.6667	609.8588	1218.71	1.56	0.00095	6.276953
P02649	0	0.5918	546.8189	1092.63	2.03	0.00111	11.00255
P02649	0	0.9324	547.9738	1641.907	1.74	0.00095	6.992116
P02649	0	0.7937	517.2755	1033.544	1.07	0.00055	3.209466
P02649	0	1	840.9127	1680.818	0.57	0.00048	2.619284
P02649	0	1	560.9453	1680.821	2.49	0.00139	4.330383
P02649	0	0.9907	910.8026	2730.393	-0.5	-0.00045	2.133987
P02649	1	1	534.6459	1601.923	1.09	0.00058	9.328854
P02649	0	1	611.7642	1222.521	1.7	0.00104	21.93635
P02649	0	0.875	613.8236	1226.64	2.97	0.00182	5.634383
P02649	0	0.8511	865.9273	1730.847	1.76	0.00152	0
P02649	0	0.9796	625.6551	1874.951	2.32	0.00145	2.649611
P02649	0	0.9663	937.9768	1874.946	-0.08	-0.00007	3.215361
P02649	0	1	625.654	1874.947	0.57	0.00035	4.418406
P02649	0	0.9355	625.655	1874.951	2.23	0.00139	59.07252
P02649	0	1	603.3046	1807.899	0.93	0.00056	6.62564
P02649	0	0.875	603.3054	1807.902	2.15	0.0013	0
P02649	0	0.5472	546.7943	1092.581	3.4	0.00186	13.48895
P02649	0	1	588.973	1764.904	2.63	0.00155	36.52353
P02649	0	0.9318	696.3443	1391.681	2.1	0.00146	3.893229
P02649	0	1	696.3441	1391.681	1.75	0.00122	3.123729
P02649	0	0.875	557.7978	1114.588	2.86	0.0016	14.03979
P02649	0	0.9302	590.6411	1769.909	2.83	0.00167	3.302549
P02649	0	0.8936	885.4562	1769.905	0.74	0.00065	4.67049
P02649	0	1	885.4563	1769.905	0.88	0.00078	1.521629
P02649	0	0.52	546.7936	1092.58	2.28	0.00124	0
P02649	1	0.7736	533.7425	1066.478	0.83	0.00044	0
P02649	1	0.5417	388.2279	1162.669	1.78	0.00069	9.694468
P02649	1	0.225	453.6012	1358.789	1.33	0.0006	4.996053
P02649	0	0.9512	937.9791	1874.951	2.4	0.00225	3.426371
P02649	1	0.56	388.2278	1162.669	1.47	0.00057	6.137087
P02649	0	1	461.5438	1382.617	0.62	0.00029	27.8215
P02649	1	1	501.6345	1502.889	-0.24	-0.00012	26.98673
P02649	1	0.8235	401.2363	1601.923	1.2	0.00048	18.01991
P02649	0	0.8537	393.2204	1177.647	1.83	0.00072	17.36686
P02649	1	0.08	486.6118	1457.821	1.13	0.00055	9.721794
P02649	0	0.9388	556.8325	1112.658	2.9	0.00161	4.814276
P02649	0	0.6923	406.908	1218.709	0.7	0.00028	2.565988
P02649	0	0.6792	474.7669	948.5264	0.29	0.00014	5.1737

P02649	0	0.5	546.7932	1092.579	1.5	0.00082	4.18807
P02649	1	0.5714	388.2277	1162.669	1.31	0.00051	5.419596
P02649	0	0.8125	621.8214	1242.635	3.39	0.00211	7.383902
P02649	0	0.5714	406.9083	1218.71	1.6	0.00065	7.570036
P02649	0	0.7143	515.7924	1030.577	1.23	0.00064	17.74095
P02649	0	0.8571	629.8491	1258.691	2.91	0.00183	3.85592
P02649	0	1	624.2932	1247.579	2.25	0.00141	8.483586
P02649	0	0.9216	420.2348	1258.69	2.2	0.00092	8.112217
P02649	1	0.8529	408.5794	1223.724	1.39	0.00056	28.66732
P02649	0	0.9583	556.8312	1112.655	0.7	0.00039	2.878149
P02649	0	1	749.406	1497.805	1.84	0.00138	0
P02649	0	0.4583	546.7935	1092.58	1.94	0.00106	8.693021
P02649	0	1	556.8323	1112.657	2.57	0.00143	14.15296
P02649	0	0.8485	577.6211	1730.849	2.56	0.00148	0
P02649	0	0.413	546.7932	1092.579	1.5	0.00082	5.677302
P02649	1	0.55	388.2274	1162.668	0.44	0.00017	21.55532
P02649	0	0.8065	414.8829	1242.634	2.45	0.00101	41.44863
P02649	0	1	464.5653	1391.681	2.2	0.00102	44.11714
P02649	0	0.9167	590.6408	1769.908	2.32	0.00137	0
P02649	0	0.6444	474.7673	948.5273	1.25	0.00059	24.47377
P02649	0	0.6389	546.7936	1092.58	2.28	0.00124	16.5644
P02649	0	1	414.8838	1242.637	4.51	0.00187	10.56003
P02649	0	0.6389	546.7924	1092.577	-0.07	-0.00004	6.913381
P02649	0	0.8776	621.8213	1242.635	3.3	0.00205	9.90283
P02649	0	1	625.6552	1874.951	2.42	0.00151	54.70777
P02649	0	0.7	515.7929	1030.578	2.18	0.00112	31.20235
P02649	0	1	821.4567	1641.906	1.22	0.001	27.65627
P06681	0	1	410.5077	1639.009	1.99	0.00082	13.50525
P06681	0	1	410.5081	1639.01	2.96	0.00121	26.47408
P06681	0	1	547.0085	1639.011	3.27	0.00179	11.1284
P06681	0	0.8421	402.5571	1205.657	1.57	0.00063	5.090429
P06681	0	1	710.0172	2128.037	3.58	0.00254	32.14403
P06681	1	1	930.692	3719.746	1.48	0.00138	1.808168
P06681	0	1	547.0082	1639.01	2.71	0.00148	10.98159
P06681	0	0.8889	684.9292	1368.851	4.26	0.00291	0
P06681	0	0.9315	456.9541	1368.848	1.73	0.00079	12.28759
P06681	0	1	456.9535	1368.846	0.39	0.00018	32.97034
P06681	0	0.942	456.9542	1368.848	2.06	0.00094	11.08633
P06681	0	0.7377	513.633	1538.884	1.53	0.00079	13.54907
P06681	0	0.6545	769.9462	1538.885	1.94	0.00149	10.96665
P06681	0	0.9333	456.9539	1368.847	1.26	0.00057	9.532727
P06681	1	0.8974	790.4546	2369.349	2.18	0.00172	15.72494
P06681	0	1	1049.081	2097.155	3.96	0.00415	3.882048
P06681	0	1	699.7224	2097.153	2.61	0.00183	5.941878
P06681	1	0.8889	629.0203	1885.046	2.26	0.00142	7.991782
P06681	0	1	873.0729	2617.204	1.63	0.00142	4.471851
P06681	0	0.8857	655.058	2617.21	3.86	0.00253	12.95572
P06681	0	0.9706	467.253	1399.744	1.76	0.00082	9.149958
P06681	0	1	700.3761	1399.745	2.12	0.00148	13.18542
P06681	0	0.4921	589.3399	1177.673	4.98	0.00293	4.395614
P06681	0	1	415.2036	1243.596	2.15	0.00089	13.71723
P06681	0	0.9074	710.3599	1419.713	2.02	0.00144	2.862124

P06681	0	0.8966	473.909	1419.712	1.93	0.00091	17.3086
P06681	0	1	648.0213	1942.049	1.8	0.00116	18.31386
P06681	0	0.9429	486.2678	1942.049	1.85	0.0009	8.118219
P06681	0	1	486.2679	1942.05	1.91	0.00093	0
P06681	0	1	696.3769	1391.747	2.3	0.0016	12.51025
P06681	0	1	882.7937	2646.367	1.4	0.00124	17.86039
P06681	0	1	410.5084	1639.012	3.85	0.00158	5.635686
P06681	0	0.9302	633.3363	1265.665	3.72	0.00235	26.21051
P06681	0	0.6111	513.6334	1538.886	2.36	0.00121	0
P06681	0	0.9677	415.2033	1243.595	1.49	0.00062	9.01186
P06681	0	0.6333	454.2666	1360.785	1.94	0.00088	19.38161
P06681	0	1	408.92	1224.746	1.91	0.00078	29.35849
P06681	0	0.6207	402.557	1205.657	1.5	0.0006	12.21969
P06681	0	0.88	547.0072	1639.007	0.92	0.0005	12.62806
P06681	0	1	533.2835	1065.56	-0.33	-0.00018	33.82913
P06681	0	1	464.5863	1391.744	0.75	0.00035	7.04821
P06681	0	0.9149	628.3271	1255.647	5.58	0.0035	54.97927
P06681	0	1	467.2515	1399.74	-1.38	-0.00064	30.97646
P06681	0	0.7778	450.2425	1797.948	2.35	0.00105	0
P06681	0	0.5	454.2662	1360.784	1	0.00045	
P06681	0	1	467.2512	1399.739	-2.03	-0.00095	49.61073
P06681	0	0.5	454.2658	1360.783	-0.01	0	0
P06681	0	1	410.5083	1639.011	3.48	0.00143	14.3905
P06681	0	0.2727	630.3826	1259.758	1.36	0.00086	32.51718
P06681	0	0.8125	684.9275	1368.848	1.76	0.0012	22.25535
P06681	0	0.963	456.9551	1368.851	3.87	0.00176	78.72992
P06681	0	0.56	454.2658	1360.783	0.06	0.00003	56.19683
P06681	0	0.5769	422.5588	1265.662	1.05	0.00044	27.49483
P06681	0	1	498.9729	1494.904	0.51	0.00025	5.977794
P06681	0	0.7647	668.0067	2002.005	0.64	0.00043	14.67508
P06681	0	0.6818	668.0075	2002.008	1.83	0.00122	2.961311
P06681	0	1	624.3258	1247.644	2.47	0.00154	0
P06681	0	1	456.9544	1368.848	2.33	0.00106	60.27592
P06681	0	0.5556	680.8964	1360.785	2.02	0.00138	5.173426
P06681	0	0.9	668.0084	2002.011	3.2	0.00214	14.82103
P06681	0	1	415.2034	1243.596	1.71	0.00071	25.58758
P06681	0	0.9667	603.333	1205.659	3.31	0.00199	38.30157
P06681	0	1	873.0741	2617.208	2.96	0.00258	8.958677
P06681	0	0.35	769.9434	1538.88	-1.63	-0.00126	52.37825
P12259	0	0.8	823.4912	1645.975	1.5	0.00123	3.613659
P12259	0	0.9	549.3309	1645.978	3.27	0.00179	21.20807
P12259	0	1	430.759	1720.014	2.04	0.00088	16.85813
P12259	0	0.9545	728.3827	2183.134	5.16	0.00375	6.97278
P12259	0	1	464.9432	1392.815	2	0.00093	0
P12259	0	0.8393	614.0092	1840.013	2.49	0.00153	9.785065
P12259	0	0.7955	427.2419	1279.711	1.32	0.00056	18.57211
P12259	0	0.907	674.8799	1348.753	2.39	0.00161	6.708357
P12259	0	1	758.6854	2274.042	1.24	0.00094	4.256592
P12259	0	1	758.6852	2274.041	1.08	0.00082	0
P12259	0	0.875	449.8819	1347.631	1.12	0.0005	55.14321
P12259	1	1	555.6758	1665.013	2.2	0.00122	7.646373
P12259	0	1	613.8669	1226.727	3.35	0.00206	12.79606

P12259	1	0.8125	527.9365	1581.795	1.06	0.00056	23.79537
P12259	0	1	464.9422	1392.812	-0.1	-0.00005	27.47878
P12259	0	0.9091	538.2697	1075.532	-0.21	-0.00011	39.1588
P12259	0	0.9062	568.9397	1704.805	2.94	0.00167	0
P12259	0	0.7778	561.3566	1121.706	2.26	0.00127	0
P12259	0	0.7429	464.8075	928.6078	1.9	0.00088	25.38149
P12259	0	0.6111	516.9608	1548.868	3.54	0.00183	43.93911
P12259	1	1	553.965	1659.881	2.9	0.0016	45.93683
P12259	0	1	475.9036	1425.696	2.74	0.0013	18.40945
P12259	0	0.9375	592.6467	1775.926	-1.25	-0.00074	0
P12259	0	1	614.0098	1840.015	3.49	0.00214	7.862066
P12259	0	0.8372	542.3018	1083.596	0.51	0.00028	39.81616
P12259	0	0.4583	501.9313	1503.779	3.16	0.00158	39.90562
P02790	0	1	619.6309	1856.878	3.47	0.00215	0
P02790	0	1	619.6306	1856.877	2.88	0.00178	11.39527
P02790	0	0.9123	928.9419	1856.877	2.56	0.00238	0.986027
P02790	0	1	748.3444	1495.681	1.84	0.00137	3.297979
P02790	0	0.7714	661.3364	1981.995	2.97	0.00196	0
P02790	0	0.7561	661.3356	1981.992	1.68	0.00111	4.924888
P02790	0	0.75	477.6185	1430.841	3.6	0.00172	20.96952
P02790	0	0.8382	643.8718	1286.736	1.96	0.00126	2.749575
P02790	0	0.8525	715.9226	1430.838	1.5	0.00107	6.445424
P02790	0	0.7609	477.6169	1430.836	0.15	0.00007	10.08243
P02790	0	0.8167	715.9226	1430.838	1.5	0.00107	5.399431
P02790	0	0.8116	643.872	1286.737	2.34	0.00151	0
P02790	0	0.8364	477.6177	1430.838	1.87	0.00089	8.381349
P02790	0	0.9744	619.6303	1856.876	2.49	0.00154	8.118405
P02790	0	1	856.8897	1712.772	1.43	0.00122	0
P02790	0	0.9839	619.6305	1856.877	2.78	0.00172	0
P02790	0	0.9831	619.6304	1856.877	2.68	0.00166	3.010022
P02790	0	1	928.9409	1856.874	1.44	0.00134	11.41802
P02790	0	1	619.6305	1856.877	2.78	0.00172	27.65837
P02790	0	1	928.9413	1856.875	1.9	0.00177	3.730076
P02790	0	1	619.6302	1856.876	2.29	0.00142	5.1441
P02790	0	1	928.941	1856.875	1.57	0.00146	3.205738
P02790	0	1	619.6296	1856.874	1.3	0.00081	3.943996
P02790	0	1	856.8898	1712.772	1.57	0.00135	0
P02790	0	1	928.9392	1856.871	-0.4	-0.00037	3.468973
P02790	0	1	619.629	1856.872	0.32	0.0002	3.844419
P02790	0	1	928.9415	1856.876	2.1	0.00195	20.42489
P02790	0	0.9796	619.6278	1856.869	-1.56	-0.00096	0
P02790	0	1	619.6296	1856.874	1.4	0.00087	51.68941
P02790	0	0.9811	928.9406	1856.874	1.11	0.00103	0
P02790	0	0.6136	477.2701	1429.796	1.87	0.00089	16.11522
P02790	0	0.8125	715.4013	1429.795	1.68	0.0012	6.416716
P02790	0	0.7755	477.2695	1429.794	0.78	0.00037	9.378213
P02790	0	0.85	715.4006	1429.794	0.66	0.00047	6.504476
P02790	0	0.6731	477.2692	1429.793	0.07	0.00004	8.007553
P02790	0	0.6364	477.2698	1429.795	1.36	0.00065	14.97369
P02790	0	0.8167	715.4021	1429.797	2.79	0.002	1.351329
P02790	0	0.9756	619.6297	1856.875	1.5	0.00093	52.51019
P02790	0	1	881.4582	2642.36	8.92	0.00785	12.94534

P02790	0	1	836.7625	2508.273	2.19	0.00183	12.52815
P02790	0	1	881.4507	2642.338	0.46	0.00041	37.1097
P02790	0	1	788.7272	2364.167	0.8	0.00063	5.414624
P02790	0	0.9867	881.4523	2642.342	2.19	0.00193	11.92839
P02790	0	1	788.7276	2364.168	1.27	0.001	16.30295
P02790	0	1	881.4545	2642.349	4.76	0.00419	0
P02790	0	0.8889	881.4556	2642.352	5.94	0.00523	16.09009
P02790	0	1	788.7268	2364.166	0.26	0.0002	5.961622
P02790	0	0.9559	881.4538	2642.347	3.93	0.00346	26.74925
P02790	0	1	881.4539	2642.347	4	0.00352	7.547066
P02790	0	1	661.3407	2642.341	1.7	0.00113	2.296825
P02790	0	0.9623	661.3396	2642.337	0.04	0.00003	6.085531
P02790	0	1	881.4528	2642.344	2.75	0.00242	0
P02790	0	0.9444	833.4163	2498.234	0	0	3.577184
P02790	0	1	881.4544	2642.348	4.55	0.00401	9.295702
P02790	0	1	881.4535	2642.346	3.58	0.00315	0.963353
P02790	0	0.9683	881.4548	2642.35	5.04	0.00443	12.44493
P02790	0	0.875	596.969	1788.892	3.09	0.00184	10.15062
P02790	0	1	1254.638	2508.269	0.71	0.00089	0
P02790	0	1	627.8233	2508.271	1.62	0.00102	2.070103
P02790	0	1	1254.639	2508.271	1.29	0.00162	0.593852
P02790	0	1	836.7606	2508.267	-0.08	-0.00007	2.472323
P02790	0	1	627.823	2508.27	1.13	0.00071	2.384292
P02790	0	1	836.762	2508.272	1.67	0.0014	6.247859
P02790	0	1	836.7606	2508.267	0	0	2.162766
P02790	0	1	836.7606	2508.267	-0.08	-0.00007	2.263237
P02790	0	1	877.6769	3507.686	3.15	0.00276	20.54631
P02790	0	1	627.8232	2508.271	1.52	0.00095	2.410218
P02790	0	1	1254.639	2508.271	1.58	0.00198	2.55822
P02790	0	0.9737	881.4526	2642.343	2.54	0.00224	7.15334
P02790	0	0.8148	894.95	1788.893	3.2	0.00286	0
P02790	0	0.9783	881.4563	2642.354	6.77	0.00596	40.10157
P02790	0	0.9189	881.456	2642.353	6.42	0.00566	32.00636
P02790	0	0.7451	477.6181	1430.84	2.7	0.00129	14.96517
P02790	0	0.8148	477.6175	1430.838	1.42	0.00068	18.02268
P02790	0	1	833.4177	2498.239	1.69	0.00141	0
P02790	0	1	881.452	2642.342	1.92	0.00169	1.299401
P02790	0	0.9	661.3415	2642.344	2.9	0.00192	2.895188
P02790	0	0.9733	881.4523	2642.342	2.19	0.00193	2.08516
P02790	0	0.9032	709.3702	2126.096	2.39	0.00169	5.0551
P02790	0	0.907	532.2791	2126.094	1.64	0.00087	9.921781
P02790	0	0.9231	532.279	2126.094	1.52	0.00081	0
P02790	0	1	820.3936	1639.78	-0.49	-0.0004	3.125367
P02790	0	1	547.2662	1639.784	1.96	0.00107	23.6427
P02790	0	1	820.3953	1639.783	1.52	0.00125	3.587369
P02790	0	1	547.2653	1639.781	0.4	0.00022	6.6714
P02790	0	1	881.4582	2642.36	8.92	0.00785	18.69551
P02790	0	0.9722	661.3387	2642.333	-1.35	-0.00089	6.004994
P02790	0	1	881.4525	2642.343	2.4	0.00212	3.038657
P02790	0	0.8919	893.4361	3570.723	-0.28	-0.00025	0
P02790	0	1	661.34	2642.338	0.59	0.00039	2.249806
P02790	0	1	881.4503	2642.336	-0.09	-0.00008	2.252324

P02790	0	1	1321.674	2642.34	1.3	0.00171	3.218996
P02790	0	1	661.3404	2642.34	1.24	0.00082	2.291156
P02790	0	1	881.4506	2642.337	0.25	0.00022	2.101504
P02790	0	0.9091	532.2801	2126.099	3.59	0.00191	4.461025
P02790	0	0.974	1063.551	2126.096	2.17	0.0023	0
P02790	0	0.8478	496.253	1981.99	0.65	0.00032	5.877422
P02790	0	0.9123	661.3356	1981.992	1.68	0.00111	15.5322
P02790	0	0.9242	709.3687	2126.091	0.24	0.00017	4.61297
P02790	0	0.9615	661.3356	1981.992	1.77	0.00117	7.369062
P02790	0	1	532.2804	2126.1	4.16	0.00221	47.07515
P02790	0	0.9375	709.3687	2126.092	0.32	0.00023	4.586155
P02790	0	0.9167	661.334	1981.987	-0.72	-0.00048	6.218521
P02790	0	0.9574	532.2791	2126.095	1.75	0.00093	5.106207
P02790	0	0.8732	709.3699	2126.095	2.05	0.00145	12.23421
P02790	0	0.8913	532.2788	2126.093	1.06	0.00057	5.081725
P02790	0	0.9375	1063.552	2126.096	2.4	0.00255	0
P02790	0	0.9315	709.3691	2126.093	0.84	0.0006	4.511355
P02790	0	0.8854	814.9001	1628.793	1.82	0.00148	3.253555
P02790	0	0.8974	532.2796	2126.097	2.67	0.00142	4.465581
P02790	0	0.9077	1063.551	2126.095	1.94	0.00206	0
P02790	0	1	455.5747	1364.71	1.28	0.00058	5.373659
P02790	0	0.9677	682.8582	1364.709	0.82	0.00056	3.192588
P02790	0	1	610.8079	1220.608	2.13	0.0013	0
P02790	0	1	610.807	1220.607	0.63	0.00038	0.05162
P02790	0	0.9683	682.8586	1364.71	1.45	0.00099	3.220432
P02790	0	0.9242	682.8593	1364.711	2.52	0.00172	5.889195
P02790	0	0.8889	682.8591	1364.711	2.25	0.00154	3.467011
P02790	0	0.875	455.5751	1364.711	2.09	0.00095	4.505167
P02790	0	0.7551	477.27	1429.795	1.74	0.00083	12.82662
P02790	0	0.7385	715.4019	1429.797	2.54	0.00181	7.42549
P02790	0	1	516.9294	1548.774	1.73	0.00089	42.04683
P02790	0	0.6591	435.9205	1305.747	1.79	0.00078	0
P02790	0	0.7857	702.3431	3507.687	3.34	0.00234	33.13522
P02790	0	0.9474	877.6759	3507.682	1.97	0.00173	3.984314
P02790	0	1	881.4567	2642.356	7.25	0.00639	26.98557
P02790	0	1	836.7625	2508.273	2.26	0.00189	5.191063
P02790	0	1	836.7622	2508.272	1.82	0.00152	22.10536
P02790	0	1	788.7274	2364.168	1.03	0.00081	6.753419
P02790	0	0.942	877.675	3507.678	0.92	0.00081	3.088639
P02790	0	1	788.7271	2364.167	0.57	0.00045	0.146488
P02790	0	0.9667	702.3418	3507.68	1.42	0.001	4.398482
P02790	0	1	788.725	2364.161	-1.99	-0.00157	6.112413
P02790	0	0.6531	877.6744	3507.676	0.23	0.0002	11.04581
P02790	0	1	836.763	2508.274	2.84	0.00238	15.30525
P02790	0	1	627.8238	2508.273	2.4	0.0015	0
P02790	0	1	836.7622	2508.272	1.82	0.00152	2.725686
P02790	0	1	836.7615	2508.27	1.02	0.00085	7.160137
P02790	0	1	627.8237	2508.273	2.3	0.00144	2.590176
P02790	0	1	836.7623	2508.272	1.97	0.00164	2.579544
P02790	0	1	836.7624	2508.273	2.11	0.00177	11.04668
P02790	0	1	627.8259	2508.282	5.71	0.00358	10.06347
P02790	0	0.9765	881.4515	2642.34	1.36	0.0012	2.038263

P02790	2	1	809.9376	3236.728	1.14	0.00092	77.18871
P02790	0	0.9623	661.3405	2642.34	1.43	0.00094	16.29011
P02790	2	1	809.9376	3236.728	1.14	0.00092	77.18871
P02790	0	0.973	881.452	2642.342	1.92	0.00169	2.1777
P02790	0	1	659.3212	3292.577	1.7	0.00112	51.654
P02790	0	1	687.4067	2060.205	1	0.00069	3.696815
P02790	0	1	1030.608	2060.209	2.49	0.00257	0
P02790	0	1	661.3413	2642.343	2.63	0.00174	5.358066
P02790	0	0.5741	631.3828	1261.758	2.9	0.00183	18.94206
P02790	0	1	836.762	2508.271	1.6	0.00134	8.311328
P02790	0	0.8696	596.9689	1788.892	2.88	0.00172	9.381875
P02790	0	0.7377	631.3829	1261.758	3	0.00189	13.57712
P02790	0	0.5556	631.3826	1261.758	2.51	0.00158	26.15951
P02790	0	0.7778	702.3413	3507.677	0.73	0.00051	6.197073
P02790	0	0.9359	877.6748	3507.677	0.71	0.00063	4.171076
P02790	0	0.9512	596.9683	1788.89	1.96	0.00117	12.11103
P02790	1	1	608.3348	1822.99	3.13	0.0019	14.70978
P02790	1	1	608.3348	1822.99	3.03	0.00184	20.55173
P02790	0	1	547.2652	1639.781	0.17	0.00009	26.22812
P02790	0	0.7879	655.8397	1310.672	2.68	0.00176	13.77399
P02790	1	1	608.3342	1822.988	2.13	0.00129	8.693175
P02790	0	0.84	655.8376	1310.668	-0.58	-0.00038	5.257352
P02790	1	0.9783	560.3005	1678.887	2.9	0.00162	8.247183
P02790	1	0.9783	560.3005	1678.887	2.9	0.00162	8.247183
P02790	0	0.6515	643.351	1285.695	2.93	0.00188	16.66861
P02790	0	1	836.7646	2508.279	4.74	0.00396	3.534253
P02790	0	1	886.95	1772.893	0.43	0.00038	5.792911
P02790	0	1	591.6365	1772.895	1.73	0.00102	8.111058
P02790	0	1	886.9507	1772.894	1.25	0.00111	100
P02790	0	0.9167	619.6317	1856.881	4.76	0.00294	10.44557
P02790	0	0.9459	836.7592	2508.263	-1.76	-0.00147	5.125496
P02790	0	1	928.9392	1856.871	-0.33	-0.00031	0
P02790	0	0.975	774.8905	1548.774	1.81	0.0014	2.328142
P02790	0	1	610.8088	1220.61	3.63	0.00221	0
P02790	0	1	619.6301	1856.876	2.19	0.00136	5.682241
P02790	0	0.8936	653.3774	1305.748	2.36	0.00154	6.496942
P02790	0	0.9737	619.6301	1856.876	2.19	0.00136	5.828601
P02790	0	0.7	437.5617	1310.67	1.32	0.00058	9.659534
P02790	0	1	881.4524	2642.343	2.33	0.00205	75.46298
P02790	0	1	643.7941	1286.581	2.36	0.00152	14.05585
P02790	0	0.5902	631.3827	1261.758	2.71	0.00171	6.663101
P02790	0	0.9833	591.7863	1182.565	3.15	0.00186	7.322608
P02790	0	0.8	477.6177	1430.838	1.87	0.00089	15.00322
P02790	1	0.5357	473.2918	1417.861	3.45	0.00163	2.276918
P02790	0	0.9062	583.7886	1166.57	2.92	0.0017	6.038012
P02790	0	0.6389	477.2701	1429.796	1.93	0.00092	48.99046
P02790	0	0.7455	631.3826	1261.758	2.61	0.00165	22.1367
P02790	0	0.8378	709.3705	2126.097	2.82	0.002	8.961815
P02790	0	1	547.2653	1639.781	0.28	0.00015	39.89036
P02790	0	1	836.7612	2508.269	0.65	0.00055	37.55566
P02790	0	0.7105	477.2709	1429.798	3.6	0.00171	12.31586
P02790	0	1	836.7631	2508.275	2.92	0.00244	7.317331

P02790	0	0.9211	881.4454	2642.322	-5.57	-0.0049	0
P02790	0	1	434.8635	1302.576	2.38	0.00103	5.680776
P02790	1	0.4	473.2902	1417.856	0.22	0.0001	7.874603
P02790	0	0.7222	435.9208	1305.748	2.42	0.00105	4.18222
P02790	0	1	643.7944	1286.581	2.83	0.00182	14.018
P02790	0	0.8409	435.9207	1305.747	2.21	0.00096	9.81278
P02790	0	0.7419	435.9211	1305.749	3.19	0.00139	55.32776
P02790	0	1	619.6303	1856.876	2.39	0.00148	13.67699
P02790	0	0.7742	619.6301	1856.876	2.09	0.0013	15.08523
P02790	0	0.7619	682.8594	1364.712	2.7	0.00184	7.132895
P02790	1	0.5	473.2912	1417.859	2.29	0.00108	0
P02790	0	0.7143	715.4017	1429.796	2.2	0.00157	57.03716
P02790	0	0.7391	477.6173	1430.837	1.04	0.0005	40.99088
P02790	0	0.9688	596.9687	1788.892	2.58	0.00154	52.66348
P02790	0	0.7593	631.3835	1261.76	3.96	0.0025	31.83052
P02790	0	0.7297	715.4045	1429.802	6.12	0.00438	0
P02790	0	0.5135	429.5835	1286.736	1.64	0.0007	6.032457
P02790	0	0.8857	435.9211	1305.749	3.19	0.00139	0
P02790	0	0.9211	653.3779	1305.748	3.02	0.00197	6.853622
P02790	0	0.7879	435.9207	1305.747	2.21	0.00096	5.965112
P02790	0	0.9231	682.8591	1364.711	2.16	0.00148	7.372588
P02790	0	1	661.3391	2642.334	-0.79	-0.00052	74.81864
P02790	0	1	547.265	1639.78	-0.16	-0.00009	21.79093
P02790	0	1	643.7941	1286.581	2.36	0.00152	2.916619
P02790	0	1	1254.64	2508.272	1.87	0.00235	0
P02790	0	0.931	596.969	1788.892	3.09	0.00184	12.18355
P02790	0	0.8205	437.5619	1310.671	1.88	0.00082	17.87706
P02790	0	0.8889	653.3767	1305.746	1.15	0.00075	7.032547
P02790	0	0.8148	496.2521	1981.987	-1.07	-0.00053	7.094138
P02790	0	0.5667	619.6297	1856.875	1.5	0.00093	7.992166
P02790	0	0.7222	715.4023	1429.797	3.14	0.00224	5.107313
P02790	0	1	788.7274	2364.168	0.96	0.00075	26.47538
P02790	0	0.8649	653.377	1305.747	1.62	0.00105	40.61971
P02790	0	1	643.7953	1286.583	4.26	0.00274	34.49154
P02790	0	1	547.2658	1639.783	1.18	0.00064	9.830676
P02790	0	1	748.3438	1495.68	1.1	0.00083	30.44287
P02790	0	0.9808	610.8086	1220.61	3.33	0.00203	11.51673
P02790	0	0.85	477.6173	1430.837	0.98	0.00047	34.01669
P02790	0	0.9773	429.5318	1286.581	2.42	0.00104	5.244845
P02790	0	0.7586	591.6359	1772.893	0.7	0.00041	0
P02790	0	0.8333	435.9211	1305.749	3.12	0.00136	3.608953
P02790	0	0.8913	653.3756	1305.744	-0.44	-0.00029	5.760612
P02790	0	0.4706	429.5837	1286.736	2.07	0.00089	4.268757
P02790	0	1	610.808	1220.609	2.43	0.00148	14.43795
P02790	0	1	643.7931	1286.579	0.84	0.00054	9.038958
P02790	0	0.8108	653.3779	1305.749	3.11	0.00203	28.82355
P02790	1	0.95	526.6532	1577.945	2.22	0.00117	56.87579
P02790	0	0.8235	661.3311	1981.979	-5.07	-0.00335	80.61507
P02790	0	0.9167	655.8397	1310.672	2.68	0.00176	21.05737
P02790	0	0.6562	442.893	1326.665	0.74	0.00033	15.59977
P02790	0	1	610.8076	1220.608	1.73	0.00106	28.2862
P02790	1	1	526.6527	1577.944	1.29	0.00068	26.63778

P02790	0	1	547.2661	1639.784	1.85	0.00101	31.49541
P02790	0	0.697	477.2705	1429.797	2.76	0.00132	37.93758
P02790	0	0.9444	477.6194	1430.844	5.52	0.00263	82.64616
P02790	0	0.92	477.2708	1429.798	3.53	0.00168	38.29065
P02790	0	0.8478	663.837	1326.667	2.34	0.00155	6.187302
P02790	0	0.5	477.2701	1429.796	2	0.00095	29.65926
P02790	0	0.6774	437.5624	1310.672	2.93	0.00128	12.54712
P02790	0	0.6047	559.3315	1117.656	2.74	0.00153	33.61863
P02790	1	0.4783	473.2909	1417.858	1.7	0.00081	12.90563
P02790	0	0.8409	435.92	1305.746	0.81	0.00035	9.173021
P02790	0	0.7941	833.4172	2498.237	1.03	0.00086	2.598774
P02790	0	0.7879	653.377	1305.747	1.62	0.00105	7.524767
P02790	0	1	643.7937	1286.58	1.79	0.00115	6.727864
P02790	0	0.875	442.8934	1326.666	1.5	0.00066	12.3796
P02790	0	0.8958	653.3758	1305.744	-0.16	-0.0001	5.661051
P02790	0	1	748.3423	1495.677	-0.94	-0.0007	0
P02790	0	1	619.629	1856.872	0.32	0.0002	18.323
P02790	0	0.8	437.5611	1310.669	-0.01	0	8.671872
P02790	1	0.675	709.4328	1417.858	1.78	0.00126	8.354035
P02790	0	0.8333	881.4481	2642.33	-2.52	-0.00222	0
P02790	0	1	788.7275	2364.168	1.11	0.00087	13.26155
P02790	0	0.7586	442.8947	1326.67	4.61	0.00204	20.28485
P02790	1	1	405.5642	1214.678	1.45	0.00059	9.480793
P02790	0	0.6957	661.3417	2642.345	3.18	0.0021	46.17258
P02790	0	1	651.7924	1302.578	3.7	0.00241	27.38969
P02790	0	0.7	559.3322	1117.657	3.94	0.0022	0
P02790	1	1	471.5978	1412.779	1.57	0.00074	19.02959
P02790	0	0.625	715.4027	1429.798	3.56	0.00255	7.108372
P02790	0	0.7667	631.3807	1261.754	-0.39	-0.00025	5.886368
P02790	0	1	610.8067	1220.606	0.23	0.00014	23.12741
P02790	0	1	429.5312	1286.579	0.92	0.0004	63.26123
P02790	0	0.8519	682.859	1364.711	2.07	0.00142	0
P02790	0	1	651.7897	1302.572	-0.42	-0.00027	24.52917
P02790	0	0.8333	435.9198	1305.745	0.18	0.00008	10.08229
P02790	0	0.7742	653.3781	1305.749	3.3	0.00215	0
P02790	0	0.7209	559.3315	1117.656	2.85	0.00159	7.019646
P02790	0	1	455.5754	1364.711	2.62	0.00119	35.94758
P02790	0	1	643.7933	1286.579	1.22	0.00078	3.080237
P02790	0	0.8571	596.9692	1788.893	3.5	0.00209	9.414963
P02790	0	0.75	655.8402	1310.673	3.42	0.00224	13.53162
P02790	0	1	643.7958	1286.584	5.01	0.00323	17.61691
P02790	0	0.9545	610.8083	1220.609	2.83	0.00173	3.5931
P02790	0	0.6818	643.3547	1285.702	8.72	0.00561	52.81116
P02790	0	0.76	833.4227	2498.253	7.63	0.00635	73.57045
P02790	0	1	619.6304	1856.877	2.68	0.00166	42.66243
P02790	0	1	651.791	1302.575	1.55	0.00101	3.864536
P02790	0	0.9565	643.7939	1286.58	2.07	0.00133	17.37591
P02790	0	0.5833	437.5619	1310.671	1.95	0.00085	57.80194
P02790	0	1	619.6301	1856.876	2.19	0.00136	37.83699
P02790	0	0.4333	429.5848	1286.74	4.7	0.00202	19.87834
P02790	0	1	610.8078	1220.608	2.03	0.00124	0
P02790	0	1	596.9694	1788.894	3.7	0.00221	9.164256

P02790	0	0.6923	437.563	1310.674	4.32	0.00189	25.29589
P02790	0	1	663.8368	1326.666	2.06	0.00137	4.283311
P02790	0	1	687.405	2060.2	-1.48	-0.00102	54.21088
P02790	0	0.8667	881.4553	2642.351	5.66	0.00498	0
P02790	0	0.4444	627.8239	2508.274	2.5	0.00157	36.75036
P02790	0	1	651.792	1302.577	3.05	0.00198	13.76973
P02790	0	1	455.5758	1364.713	3.56	0.00162	56.38852
P02790	0	0.65	709.3707	2126.098	3.17	0.00224	9.560533
P02790	0	0.6667	886.9501	1772.893	0.57	0.0005	0
P02790	0	0.7083	442.8929	1326.664	0.47	0.00021	25.79791
P02790	0	0.8065	477.6173	1430.837	0.98	0.00047	9.734451
P02790	0	0.6316	477.2704	1429.797	2.57	0.00123	30.02251
P02790	0	0.7778	661.3417	2642.345	3.27	0.00216	8.619888
P02790	0	0.6364	715.9225	1430.838	1.33	0.00095	5.997162
P02790	0	0.9259	653.3778	1305.748	2.83	0.00185	14.76439
P02790	0	1	455.5748	1364.71	1.48	0.00067	17.8491
P02790	0	1	651.7924	1302.577	3.61	0.00235	0
P02790	0	1	748.3455	1495.684	3.31	0.00247	0
P02790	0	0.4118	661.3361	1981.994	2.51	0.00166	36.75528
P02790	0	1	651.7917	1302.576	2.58	0.00168	19.30686
P02790	0	1	651.7912	1302.575	1.83	0.00119	9.342721
P04003	0	0.8462	577.9735	1731.906	2.77	0.0016	39.93862
P04003	1	1	592.5422	2367.147	6.75	0.004	0
P04003	0	1	791.3607	1581.714	1.63	0.00129	30.35863
P04003	0	0.971	762.0198	2284.045	0.39	0.00029	4.122707
P04003	0	0.9062	571.7678	2284.049	2.44	0.00139	9.958784
P04003	0	0.9286	1142.527	2284.047	1.43	0.00164	0
P04003	0	0.9692	762.0204	2284.047	1.19	0.0009	3.49351
P04003	0	0.2703	840.7556	2520.252	2.3	0.00193	0
P04003	0	0.907	697.3949	1393.783	1.28	0.00089	3.804579
P04003	0	0.9821	697.3955	1393.784	2.15	0.0015	12.91592
P04003	0	1	465.2655	1393.782	0.87	0.0004	9.287104
P04003	0	0.9149	762.0212	2284.049	2.23	0.0017	10.20921
P04003	0	0.9062	456.9064	1368.705	0.57	0.00026	9.161044
P04003	0	0.9062	684.8564	1368.705	1.25	0.00086	5.453128
P04003	0	0.9286	456.9068	1368.706	1.44	0.00066	6.328815
P04003	0	1	575.9439	1725.817	2.01	0.00116	4.341281
P04003	0	1	863.4114	1725.815	1.14	0.00098	3.270589
P04003	0	1	575.9445	1725.819	3.07	0.00177	16.95031
P04003	0	1	575.944	1725.817	2.23	0.00128	44.79838
P04003	0	0.9592	665.1003	2657.38	1.42	0.00095	5.72896
P04003	0	0.9643	473.8943	1419.668	2.08	0.00098	25.94194
P04003	0	1	863.4118	1725.816	1.63	0.00141	5.102597
P04003	0	1	575.9436	1725.816	1.59	0.00091	6.121918
P04003	0	0.9756	886.4644	2657.379	1.11	0.00098	8.402198
P04003	0	0.9	532.2813	2657.377	0.52	0.00028	8.856483
P04003	0	1	866.4564	1731.906	2.54	0.0022	10.07373
P04003	0	1	577.9735	1731.906	2.77	0.0016	9.586963
P04003	0	1	665.1005	2657.38	1.61	0.00107	6.675801
P04003	0	0.5849	618.883	1236.759	2.66	0.00165	6.128533
P04003	0	1	723.0161	2167.034	1.53	0.0011	3.143506
P04003	0	1	665.6967	1995.075	1.5	0.001	8.57529

P04003	0	0.6207	499.5237	1995.073	0.36	0.00018	4.095746
P04003	0	1	665.6959	1995.073	0.4	0.00027	8.437095
P04003	0	0.825	617.6623	1850.972	1.07	0.00066	2.227891
P04003	0	1	583.3052	1747.901	2.9	0.00169	0
P04003	0	0.7273	538.9785	1614.921	1.67	0.0009	6.003868
P04003	0	0.8434	807.9638	1614.92	1.26	0.00102	3.590006
P04003	0	1	521.9287	1563.771	2.48	0.00129	31.26053
P04003	0	1	521.9275	1563.768	0.26	0.00013	44.10965
P04003	0	0.9808	521.9279	1563.769	0.96	0.0005	13.91652
P04003	0	1	737.6812	2211.029	-0.42	-0.00031	16.69851
P04003	0	1	521.928	1563.769	1.19	0.00062	30.64044
P04003	0	1	1006.484	3017.438	2.1	0.00211	3.853532
P04003	0	0.9778	594.2934	1780.866	3.14	0.00187	28.99681
P04003	0	1	594.293	1780.864	2.42	0.00144	7.631752
P04003	0	1	594.2941	1780.868	4.27	0.00254	10.54688
P04003	0	1	594.2935	1780.866	3.35	0.00199	8.394278
P04003	0	1	594.2934	1780.866	3.14	0.00187	11.27181
P04003	0	1	594.2935	1780.866	3.25	0.00193	4.428563
P04003	0	0.8889	594.2932	1780.865	2.73	0.00162	10.73281
P04003	0	1	594.2918	1780.861	0.37	0.00022	
P04003	0	1	594.2933	1780.865	2.94	0.00174	21.4029
P04003	0	0.9821	743.8709	1486.735	0.53	0.00039	5.917057
P04003	0	0.9804	496.2496	1486.734	0.4	0.0002	11.87973
P04003	0	0.7	743.8714	1486.736	1.19	0.00088	100
P04003	0	0.8571	743.8707	1486.734	0.28	0.00021	21.13439
P04003	0	0.8889	743.8712	1486.735	0.86	0.00064	6.260056
P04003	0	0.8958	496.2495	1486.734	0.03	0.00001	8.309007
P04003	0	0.9459	577.9731	1731.905	2.03	0.00117	53.43172
P04003	0	0.6538	412.9241	1236.758	1.96	0.00081	19.6584
P04003	0	0.98	654.3664	1307.726	1.32	0.00086	7.611247
P04003	0	1	424.2446	1270.719	0.99	0.00042	19.71972
P04003	0	0.9333	496.2497	1486.734	0.46	0.00023	8.840238
P04003	0	0.8837	697.395	1393.783	1.45	0.00101	18.66573
P04003	0	0.4286	571.3325	1141.658	4.13	0.00236	3.239827
P04003	0	0.641	496.2505	1486.737	2.18	0.00108	34.28121
P04003	0	1	666.8434	2664.352	1.27	0.00085	38.21416
P04003	0	1	665.6948	1995.07	-1.34	-0.00089	23.38381
P04003	0	0.5263	618.8817	1236.756	0.59	0.00037	0
P04003	0	1	654.3669	1307.726	2.07	0.00135	7.660088
P04003	0	1	521.9275	1563.768	0.26	0.00013	50.3068
P04003	0	0.8788	669.0999	2673.378	2.59	0.00173	6.195457
P04003	0	0.8947	697.3961	1393.785	3.03	0.00211	13.96212
P04003	0	0.9375	594.2937	1780.867	3.66	0.00217	9.182528
P04003	0	1	665.1005	2657.38	1.61	0.00107	38.72942
P04003	0	0.9677	697.3972	1393.787	4.52	0.00315	0
P04003	0	1	625.3438	1249.68	1.24	0.00077	53.08512
P04003	0	0.5741	557.3252	1113.643	2.15	0.0012	4.562491
P04003	0	0.4848	618.8818	1236.756	0.79	0.00049	0
P04003	0	0.8846	594.2935	1780.866	3.25	0.00193	4.399632
P04003	0	0.5	571.3317	1141.656	2.74	0.00156	16.66837
P04003	0	1	391.6986	1563.773	3.33	0.0013	44.70418
P04003	1	0.9048	443.2823	1327.832	1.04	0.00046	12.28446

P04003	0	1	782.3881	1563.769	0.85	0.00066	8.99669
P04003	0	0.6129	618.8828	1236.758	2.37	0.00146	41.83931
P04003	0	1	465.2658	1393.783	1.53	0.00071	4.642035
P04003	0	1	654.3666	1307.726	1.69	0.00111	6.419182
P04003	0	0.4872	412.9236	1236.756	0.63	0.00026	6.752341
P04003	1	0.1	710.6102	2839.419	2.87	0.00204	22.72827
P04003	0	1	710.3374	1419.668	1.46	0.00104	0
P04003	0	0.8276	424.8901	1272.656	2.26	0.00096	3.071523
P04003	0	1	529.9382	1587.8	0.54	0.00028	22.28356
P04003	0	0.9167	594.2911	1780.859	-0.66	-0.00039	0
P04003	0	0.5926	557.3262	1113.645	3.9	0.00217	4.750621
P04003	0	0.96	594.2925	1780.863	1.7	0.00101	12.30907
P04003	0	0.8421	594.2921	1780.862	0.98	0.00058	18.51723
P04003	0	0.9167	571.7678	2284.049	2.33	0.00133	16.62981
P04003	0	0.84	594.2941	1780.868	4.27	0.00254	22.99557
P04003	0	0.5862	412.9233	1236.755	0.04	0.00002	0
P04003	0	1	594.2938	1780.867	3.76	0.00223	45.42695
P04003	0	0.9556	582.3161	1163.625	2.75	0.0016	0
P04003	0	0.6818	666.8449	2664.358	3.38	0.00225	5.265999
P04003	0	0.8182	496.2497	1486.735	0.58	0.00029	32.4411
P04003	0	0.6	546.8314	1092.656	2.02	0.0011	2.89266
P04003	0	1	689.6487	2066.931	1.71	0.00118	38.6515
P04003	0	0.8857	636.8303	1272.653	0.43	0.00027	3.067272
P04003	0	1	723.0165	2167.035	2.12	0.00153	0
P04003	0	0.913	998.0417	1995.076	1.85	0.00184	4.148412
P04003	0	0.9	594.2933	1780.865	3.04	0.0018	12.26732
P04003	0	0.931	654.3668	1307.726	1.97	0.00129	18.28392
P04003	0	0.8846	436.5799	1307.725	1.1	0.00048	2.087198
P04003	0	0.2083	617.6627	1850.974	1.76	0.00109	11.55008
P04003	0	0.8846	436.5802	1307.726	1.66	0.00072	9.386792
P04003	0	0.8333	594.2935	1780.866	3.25	0.00193	19.40338
P04003	0	0.9545	436.5791	1307.723	-0.86	-0.00037	36.24191
P04003	0	1	782.3893	1563.771	2.41	0.00189	0
P04003	0	0.8571	665.6947	1995.07	-1.43	-0.00095	0
P04003	0	0.8276	636.8328	1272.658	4.45	0.00283	13.46358
P04003	0	0.9091	594.293	1780.865	2.53	0.0015	45.06875
P04003	0	0.8125	594.2929	1780.864	2.22	0.00132	37.84834
P04003	0	0.7143	496.254	1486.747	9.2	0.00456	21.89094
P04003	0	0.6667	630.8229	2520.27	9.3	0.00586	80.25001
P04003	0	0.9091	594.2943	1780.868	4.69	0.00278	21.22748
P03952	0	1	1007.493	3020.463	0.81	0.00082	3.181053
P03952	0	0.9583	802.4149	1603.822	1.74	0.00139	3.011365
P03952	0	1	535.2791	1603.823	1.85	0.00099	13.20701
P03952	1	0.9091	578.0175	1732.038	2.85	0.00164	21.81792
P03952	1	1	578.0176	1732.038	3.06	0.00177	12.43199
P03952	1	0.8571	578.0179	1732.039	3.69	0.00213	16.64993
P03952	0	1	703.3482	1405.689	-0.09	-0.00006	0
P03952	0	1	517.2692	1549.793	1.04	0.00054	10.85589
P03952	0	0.9839	661.3574	1321.707	1.5	0.00099	3.710219
P03952	0	0.8615	611.3337	1831.987	2.22	0.00136	7.481466
P03952	0	0.8103	562.3014	1684.89	2.24	0.00126	8.835663
P03952	0	1	842.9477	1684.888	1.35	0.00113	9.833282

P03952	1	1	455.9296	1365.774	1.01	0.00046	44.15098
P03952	0	0.9796	823.3984	2468.181	0.45	0.00037	2.843171
P03952	0	0.8776	603.354	1205.701	2.3	0.00138	6.096952
P03952	0	1	528.5988	1583.782	3.62	0.00191	5.541241
P03952	0	0.9778	452.9117	1356.72	1.56	0.0007	10.34233
P03952	0	1	731.66	2192.965	0.46	0.00034	36.83466
P03952	0	1	775.4006	1549.794	1.72	0.00133	7.716219
P03952	0	1	517.2687	1549.791	0.1	0.00005	10.97659
P03952	0	1	375.1787	1497.693	0.78	0.00029	17.94917
P03952	0	1	499.9023	1497.692	0.35	0.00017	18.98055
P03952	0	1	482.6175	1445.838	1.64	0.00079	10.01029
P03952	0	0.9286	528.6392	1583.903	2.15	0.00114	14.429
P03952	1	0.8929	433.7643	1732.036	1.54	0.00067	14.46412
P03952	0	0.8727	482.2323	963.4574	1.01	0.00049	16.44532
P03952	1	0.96	433.7646	1732.036	2.03	0.00088	5.421066
P03952	0	0.9655	622.8648	1244.722	1.8	0.00112	20.13895
P03952	0	0.9459	482.6176	1445.838	1.89	0.00091	13.68931
P03952	0	0.5517	444.2701	1330.796	1.89	0.00084	13.07454
P03952	0	0.7959	612.3477	1223.688	0.52	0.00032	9.290229
P03952	0	0.85	404.8775	1212.618	1.27	0.00051	30.5583
P03952	0	1	460.5992	1379.783	4.08	0.00188	30.37824
P03952; P2	0	0.9333	426.565	1277.681	3.41	0.00145	0
P03952	0	1	460.5978	1379.779	1.02	0.00047	42.17579
P03952	0	1	441.2408	1321.708	1.9	0.00084	0
P03952	0	0.9231	916.4963	1831.985	1.46	0.00134	6.034744
P03952	0	0.9429	775.4016	1549.796	2.98	0.00231	2.149021
P03952	0	0.8824	528.639	1583.903	1.92	0.00101	32.58677
P03952	0	1	452.9136	1356.726	5.74	0.0026	5.166926
P03952	0	1	482.2324	963.4575	1.08	0.00052	7.208748
P03952	0	0.7667	451.9029	1353.694	1.4	0.00063	20.73062
P03952	0	0.92	517.2681	1549.79	-1.08	-0.00056	23.66366
P03952	1	0.5714	433.7641	1732.035	0.97	0.00042	5.24967
P03952	1	0.7419	529.9819	1587.931	0.27	0.00014	67.18843
P03952	1	0.7419	529.9819	1587.931	0.27	0.00014	67.18843
P03952	0	1	404.8774	1212.618	1.2	0.00048	38.38169
P03952	0	1	605.3419	1209.676	3.88	0.00235	46.42304
P03952	0	0.8438	612.3497	1223.692	3.91	0.00239	41.04689
P03952	0	0.9667	775.4001	1549.793	1.01	0.00079	0
P03952	0	0.7778	612.3498	1223.692	4.01	0.00245	38.50381
P19827	0	1	502.2592	2006.015	2.88	0.00144	45.20381
P19827	0	1	689.3588	3442.765	2.68	0.00184	13.48158
P19827	0	1	861.4465	3442.764	2.47	0.00213	0
P19827	0	1	689.3613	3442.777	6.22	0.00429	34.94258
P19827	0	0.9882	669.3419	2006.011	1.09	0.00073	0
P19827	0	1	857.4479	3426.77	2.64	0.00226	13.56148
P19827	0	1	857.4478	3426.769	2.42	0.00207	1.49261
P19827	0	0.9615	686.1593	3426.767	1.9	0.0013	7.658159
P19827	0	1	857.4468	3426.766	1.35	0.00116	3.932991
P19827	0	1	686.1589	3426.766	1.37	0.00094	6.987109
P19827	0	1	857.4465	3426.764	0.93	0.00079	5.578273
P19827	0	1	861.446	3442.762	1.9	0.00164	0
P19827	0	1	717.3759	2150.113	0.98	0.00071	0

P19827	0	0.9831	717.3772	2150.117	2.77	0.00199	1.731905
P19827	0	1	455.8993	1365.683	1.36	0.00062	3.450308
P19827	0	0.8406	724.917	1448.827	1.94	0.00141	5.241786
P19827	0	0.6889	483.6135	1448.826	1.47	0.00071	9.13039
P19827	0	0.8769	512.2838	1534.837	1.39	0.00071	7.962925
P19827	0	0.9487	767.9216	1534.836	0.92	0.00071	3.609533
P19827	0	1	695.8709	1390.734	1.38	0.00096	5.172676
P19827	0	1	686.1582	3426.762	0.3	0.0002	9.128318
P19827	0	1	857.4466	3426.765	1.07	0.00092	7.652941
P19827	0	0.75	724.9168	1448.826	1.61	0.00116	41.33025
P19827	0	0.9143	717.3764	2150.115	1.67	0.00119	0
P19827	0	0.9881	717.3766	2150.115	1.92	0.00138	2.873098
P19827	0	1	538.2837	2150.113	0.86	0.00046	10.13554
P19827	0	1	538.2836	2150.113	0.75	0.0004	4.308006
P19827	0	1	717.3754	2150.112	0.3	0.00022	2.36122
P19827	0	1	1075.56	2150.113	0.82	0.00088	0
P19827	0	0.7	521.626	1562.864	2.09	0.00109	11.93568
P19827	0	0.6389	521.6256	1562.862	1.27	0.00066	29.19496
P19827	0	0.8889	455.8996	1365.684	2.16	0.00098	3.740248
P19827	1	1	525.3148	1573.93	2.23	0.00117	7.2367
P19827	1	1	525.3151	1573.931	2.69	0.00141	13.50332
P19827	0	0.9767	669.3391	2006.003	-3.2	-0.00214	8.538748
P19827	0	1	1003.511	2006.014	2.65	0.00266	0
P19827	0	1	669.3416	2006.01	0.64	0.00042	0
P19827	0	1	502.2577	2006.009	0.02	0.00001	12.30029
P19827	0	0.8636	717.3763	2150.114	1.5	0.00107	2.724067
P19827	0	0.9245	717.3799	2150.125	6.61	0.00473	7.377326
P19827	0	0.9	721.3932	2162.165	6.12	0.00441	22.2168
P19827	0	0.9697	541.2951	2162.159	3.15	0.0017	11.17634
P19827	0	1	721.3903	2162.156	2.14	0.00154	4.324872
P19827	0	1	721.3911	2162.159	3.15	0.00227	9.064602
P19827	0	0.8043	689.4046	1377.802	2.84	0.00196	0
P19827	0	0.8197	689.4047	1377.802	3.02	0.00208	7.309332
P19827	0	0.825	459.9379	1377.799	0.88	0.00041	9.876637
P19827	0	1	407.8652	1221.581	1.35	0.00055	33.46538
P19827	0	0.9268	455.899	1365.682	0.69	0.00031	37.74993
P19827	0	0.8302	689.4067	1377.806	5.94	0.00409	28.11077
P19827	1	1	477.281	1429.828	2.82	0.00135	50.93655
P19827	1	1	477.281	1429.828	2.82	0.00135	50.93655
P19827	0	0.775	521.6256	1562.862	1.27	0.00066	20.27046
P19827	0	0.8571	660.609	2639.414	2.58	0.0017	4.423915
P19827	0	0.9706	880.4749	2639.41	1.08	0.00095	32.80231
P19827	0	0.9242	586.6712	1757.999	3.59	0.0021	10.66379
P19827	0	0.931	586.6711	1757.999	3.48	0.00204	8.329605
P19827	0	0.9551	616.0784	2461.292	2.68	0.00165	2.491924
P19827	0	0.95	716.7116	2148.12	1.73	0.00124	5.98686
P19827	0	1	1074.563	2148.119	1.23	0.00132	0
P19827	0	1	716.7106	2148.117	0.28	0.0002	0.907566
P19827	0	1	716.7108	2148.118	0.54	0.00038	2.442147
P19827	0	0.9091	807.9176	1614.828	2.19	0.00177	0
P19827	0	0.987	538.9471	1614.827	1.42	0.00077	5.792378
P19827	0	0.9881	807.916	1614.825	0.22	0.00018	3.191833

P19827	0	1	538.9467	1614.826	0.74	0.0004	10.91788
P19827	0	0.9524	455.8996	1365.684	2.16	0.00098	16.29556
P19827	0	1	455.8993	1365.683	1.36	0.00062	41.6964
P19827	0	0.9892	821.0991	2461.283	-1.09	-0.00089	7.442227
P19827	1	1	523.9715	1569.9	2.47	0.00129	38.97852
P19827	0	1	790.9352	1580.863	2.79	0.00221	11.04541
P19827	0	0.2857	1004.143	3010.416	4.83	0.00484	31.44452
P19827	0	0.2157	581.3602	1161.713	4.35	0.00252	10.98513
P19827	0	1	461.2311	1381.679	1.66	0.00077	19.4267
P19827	0	1	461.2299	1381.675	-0.79	-0.00036	26.6718
P19827	0	1	461.231	1381.678	1.53	0.00071	4.753454
P19827	0	1	691.3434	1381.68	2.4	0.00166	2.939857
P19827	0	1	461.2312	1381.679	2	0.00092	20.77578
P19827	0	0.8932	579.3191	1157.631	3.11	0.0018	15.08549
P19827	0	1	461.2311	1381.679	1.73	0.0008	5.567979
P19827	0	1	489.2369	1465.696	1.45	0.00071	21.17375
P19827	0	1	683.3458	1365.684	2.1	0.00144	2.56214
P19827	0	1	461.2314	1381.68	2.46	0.00113	0
P19827	0	0.8846	455.8996	1365.684	2.16	0.00098	27.66081
P19827	0	0.6129	521.6267	1562.865	3.26	0.0017	27.20622
P19827	0	0.6897	521.6246	1562.859	-0.61	-0.00032	0
P19827	0	0.8039	521.6265	1562.865	2.91	0.00152	3.906552
P19827	0	0.8667	521.6262	1562.864	2.44	0.00127	15.79935
P19827	0	1	821.1016	2461.29	1.96	0.00161	5.867411
P19827	0	1	767.7294	2301.174	1.38	0.00106	2.659213
P19827	0	1	538.9471	1614.827	1.54	0.00083	35.01103
P19827	0	1	541.2948	2162.157	2.58	0.0014	24.73973
P19827	0	0.9701	617.3741	1850.108	2.56	0.00158	9.627854
P19827	0	1	1151.091	2301.174	1.54	0.00178	0
P19827	0	1	512.2834	1534.836	0.68	0.00035	0
P19827	0	1	407.8647	1221.58	0.23	0.00009	57.65248
P19827	0	0.7353	526.9575	1578.858	1.77	0.00093	29.93312
P19827	0	0.5556	526.9578	1578.859	2.23	0.00118	11.36375
P19827	0	1	461.2312	1381.679	1.93	0.00089	31.67815
P19827	0	0.875	434.5826	1301.733	2.86	0.00124	8.967201
P19827	0	0.9242	434.5827	1301.733	3.14	0.00136	20.19686
P19827	0	0.814	668.678	2004.02	2.53	0.00169	9.587754
P19827	0	0.7941	660.6072	2639.407	-0.01	-0.00001	6.559213
P19827	0	0.7941	459.9383	1377.8	1.61	0.00074	49.88604
P19827	0	0.6923	586.6691	1757.993	0.05	0.00003	28.33368
P19827	0	0.5641	651.3696	1301.732	2	0.0013	10.39654
P19827	0	0.9643	717.372	2150.101	-4.55	-0.00326	22.14915
P19827	0	0.6333	586.6704	1757.996	2.13	0.00125	0
P19827	0	1	695.8708	1390.734	1.29	0.0009	8.017869
P19827	0	1	541.2936	2162.152	0.32	0.00018	21.67015
P19827	0	0.8214	716.7107	2148.118	0.45	0.00032	20.02151
P19827	0	1	768.0705	2302.197	2.67	0.00205	6.141106
P19827	0	1	857.4502	3426.779	5.27	0.00452	24.66161
P19827	0	1	669.3433	2006.015	3.1	0.00207	17.90654
P19827	0	1	857.4487	3426.773	3.56	0.00305	3.807626
P19827	0	1	413.1972	1237.577	2.29	0.00094	37.50584
P19827	0	0.4545	651.3693	1301.731	1.53	0.001	3.310628

P19827	1	1	523.971	1569.898	1.42	0.00075	25.78511
P19827	0	1	790.9399	1580.872	8.74	0.00691	80.02103
P19827	0	0.1556	387.9087	1161.711	2.84	0.0011	11.47964
P19827	0	1	790.9354	1580.863	3.02	0.00239	46.53669
P19827	0	0.4483	669.3432	2006.015	3.01	0.00201	0
P19827	0	0.6857	617.3533	1233.699	2.79	0.00172	100
P19827	0	0.6923	669.3408	2006.008	-0.64	-0.00043	0
P19827	0	0.95	660.609	2639.414	2.67	0.00176	8.209394
P19827	1	0.913	564.3189	1690.942	3.84	0.00216	37.00291
P19827	0	0.8333	716.711	2148.118	0.88	0.00063	7.836177
P19827	0	0.9444	735.8668	1470.726	2.66	0.00196	0
P19827	0	0.9032	454.2737	1360.807	2.09	0.00095	0
P19827	0	1	538.2839	2150.114	1.21	0.00065	41.15789
P19827	0	1	538.2839	2150.114	1.21	0.00065	14.89626
P19827	0	0.3913	586.6699	1757.995	1.4	0.00082	7.913471
P19827	0	1	413.1973	1237.577	2.51	0.00103	0
P19827	0	1	512.2849	1534.84	3.66	0.00187	3.630345
P19827	0	0.8667	660.6094	2639.416	3.23	0.00213	2.544646
P19827	0	0.9444	669.3415	2006.01	0.45	0.0003	12.58709
P19827	0	0.9565	1003.512	2006.016	3.62	0.00363	38.91499
P19827	0	0.7895	689.4028	1377.798	0.18	0.00013	38.76474
P19827	0	1	861.4435	3442.752	-1.08	-0.00093	53.56005
P19827	0	0.9091	790.9343	1580.861	1.71	0.00135	42.16343
P19827	0	1	880.4799	2639.425	6.77	0.00596	44.87915
P01009	0	0.977	698.0612	2092.169	2.32	0.00162	5.433722
P01009	0	1	782.7242	2346.158	3.83	0.00299	6.141326
P01009	0	0.971	782.7229	2346.154	2.11	0.00165	5.524452
P01009	0	1	782.7232	2346.155	2.5	0.00195	14.48193
P01009	0	1	782.7235	2346.156	2.81	0.0022	0
P01009	0	1	384.708	1535.81	2.5	0.00096	8.622455
P01009	0	0.9111	512.6083	1535.81	2.59	0.00132	9.173017
P01009	0	0.9091	512.6075	1535.808	1.04	0.00053	8.395659
P01009	0	1	768.4086	1535.81	2.2	0.00169	6.984836
P01009	0	0.9783	708.0489	2122.132	2.41	0.0017	6.271567
P01009	0	1	517.9398	1551.805	2.14	0.00111	9.003087
P01009	1	0.92	423.7341	1691.915	4.12	0.00174	58.0178
P01009	0	0.9868	698.0613	2092.169	2.58	0.0018	4.925614
P01009	0	0.9483	523.7976	2092.169	2.17	0.00113	6.05226
P01009	0	1	698.061	2092.169	2.15	0.0015	4.687449
P01009	0	0.9259	523.7976	2092.168	2.05	0.00107	6.546816
P01009	0	0.931	523.7976	2092.168	2.05	0.00107	9.874651
P01009	0	1	698.0612	2092.169	2.41	0.00168	20.87814
P01009	0	0.9888	698.061	2092.169	2.15	0.0015	8.912704
P01009	0	0.9167	523.7976	2092.168	2.05	0.00107	11.11422
P01009	0	1	523.7973	2092.167	1.58	0.00083	10.89053
P01009	0	0.5556	523.7982	2092.171	3.22	0.00168	14.33731
P01009	0	0.7609	698.0609	2092.168	1.88	0.00131	9.910221
P01009	0	1	523.797	2092.166	1	0.00052	14.32156
P01009	0	0.9877	698.0608	2092.168	1.8	0.00125	10.55522
P01009	0	0.931	523.7976	2092.168	2.05	0.00107	8.526839
P01009	0	0.9483	523.7974	2092.168	1.7	0.00089	5.175604
P01009	0	1	698.061	2092.168	2.06	0.00143	8.604578

P01009	0	0.95	523.7974	2092.168	1.82	0.00095	4.959618
P01009	0	0.9802	698.061	2092.169	2.15	0.0015	4.42185
P01009	0	0.9429	782.7212	2346.149	-0.08	-0.00006	38.86
P01009	0	1	517.7491	2067.975	1.1	0.00057	4.4005
P01009	0	1	414.4007	2067.975	1.1	0.00046	11.48153
P01009	0	1	734.6884	2202.051	1.53	0.00112	9.848578
P01009	0	1	734.6887	2202.051	1.95	0.00143	8.407268
P01009	0	1	734.6893	2202.053	2.86	0.0021	7.605814
P01009	0	0.6863	588.8593	1176.711	3.02	0.00178	4.365268
P01009	0	1	414.4015	2067.978	2.88	0.00119	14.70936
P01009	0	1	517.7499	2067.978	2.75	0.00142	0
P01009	0	0.8235	512.6075	1535.808	1.04	0.00053	25.11739
P01009	0	1	464.5734	1391.706	0.87	0.0004	27.96925
P01009	0	1	464.5738	1391.707	1.79	0.00083	36.60539
P01009	0	0.9167	699.9091	1398.811	1.86	0.0013	26.5344
P01009	0	0.83	699.9085	1398.81	1.07	0.00075	6.723844
P01009	0	1	849.7878	2547.349	1.85	0.00157	9.545839
P01009	0	1	716.3893	2862.535	-3.42	-0.00245	44.16753
P01009	0	0.7344	855.1213	2563.349	4.01	0.00343	13.23696
P01009	0	0.9747	734.6877	2202.049	0.7	0.00051	1.286083
P01009	0	1	414.401	2067.976	1.69	0.0007	9.884032
P01009	0	0.7368	689.9969	2067.976	1.87	0.00129	12.52984
P01009	0	0.9565	517.7493	2067.975	1.45	0.00075	13.57144
P01009	0	1	414.4009	2067.975	1.55	0.00064	9.095506
P01009	0	1	414.4011	2067.976	1.92	0.00079	5.651325
P01009	0	0.9615	517.7497	2067.977	2.28	0.00118	21.97212
P01009	0	1	517.749	2067.974	0.86	0.00044	6.118406
P01009	0	1	517.7495	2067.976	1.92	0.00099	20.14589
P01009	0	1	414.401	2067.976	1.84	0.00076	35.59876
P01009	0	0.9545	689.9968	2067.976	1.69	0.00117	8.142431
P01009	0	0.9535	689.9965	2067.975	1.34	0.00092	8.560637
P01009	0	0.9355	727.3588	2180.062	1.01	0.00074	15.31083
P01009	0	0.9167	727.3613	2180.069	4.37	0.00318	18.02864
P01009	0	1	682.9161	1364.825	2.55	0.00174	37.2782
P01009	0	0.8475	682.9161	1364.825	2.64	0.0018	9.894423
P01009	0	0.9697	572.8274	2288.288	1.77	0.00101	5.600205
P01009	0	1	763.434	2288.288	1.67	0.00127	10.15979
P01009	0	0.8065	588.8605	1176.714	4.99	0.00294	6.339751
P01009	0	0.9831	727.3611	2180.069	4.12	0.00299	0
P01009	0	0.9355	727.3607	2180.067	3.53	0.00257	9.033413
P01009	0	1	727.3593	2180.063	1.6	0.00116	4.105151
P01009	0	0.9677	545.7716	2180.064	2.13	0.00116	17.75195
P01009	0	1	727.3594	2180.064	1.77	0.00128	8.700187
P01009	0	1	727.3601	2180.066	2.69	0.00196	8.861632
P01009	0	0.9111	487.7713	1948.064	0.77	0.00037	4.733218
P01009	0	0.8971	652.4141	1303.821	1.7	0.00111	7.254396
P01009	0	0.8542	435.2784	1303.821	1.48	0.00064	10.96723
P01009	0	0.9512	650.0268	1948.066	1.97	0.00128	0.507883
P01009	0	0.9636	650.0267	1948.065	1.78	0.00115	6.154496
P01009	0	0.9655	650.0266	1948.065	1.68	0.00109	8.665689
P01009	0	0.8364	588.8594	1176.712	3.23	0.0019	14.02577
P01009	0	0.8065	588.859	1176.711	2.5	0.00147	6.818508

P01009	0	0.8154	622.3547	1865.049	2.33	0.00145	11.06829
P01009	1	1	793.4399	2378.305	2.21	0.00175	5.169472
P01009	1	1	546.3451	1637.021	1.41	0.00077	13.22658
P01009	1	0.8478	523.6396	1568.904	2.17	0.00114	34.55547
P01009	0	1	893.488	1785.969	1.8	0.00161	19.74335
P01009	0	1	572.8281	2288.291	3.05	0.00174	0
P01009	0	0.8387	466.9416	1398.81	1.48	0.00069	10.98024
P01009	0	0.8421	435.2776	1303.818	-0.34	-0.00015	0
P01009	0	0.8448	648.8577	1296.708	3.39	0.0022	9.004613
P01009	0	0.8667	587.293	2346.15	0.46	0.00027	0
P01009	0	0.8983	580.364	1159.721	3.51	0.00203	24.51538
P01009	0	0.9286	727.3615	2180.07	4.62	0.00336	4.089424
P01009	0	1	689.9988	2067.982	4.7	0.00324	21.25354
P01009	0	0.9655	517.9399	1551.805	2.38	0.00123	14.28182
P01009	1	0.8966	595.332	2378.306	2.72	0.00162	5.649186
P01009	0	0.9688	512.6078	1535.809	1.51	0.00078	19.15731
P01009	0	0.8261	727.3598	2180.065	2.36	0.00171	7.234739
P01009	0	1	517.9406	1551.807	3.79	0.00196	17.10749
P01009	0	1	419.2385	2092.164	-0.26	-0.00011	18.0356
P01009	0	0.7692	487.7715	1948.064	1.08	0.00053	23.69799
P01009	0	0.9149	682.9153	1364.823	1.48	0.00101	15.09725
P01009	0	0.8333	727.3598	2180.065	2.36	0.00171	6.33804
P01009	1	0.9	423.7333	1691.912	2.32	0.00098	15.96248
P01009	0	0.973	435.2784	1303.821	1.34	0.00058	2.534157
P01009	1	1	427.7541	1707.995	2.36	0.00101	12.65208
P01009	0	0.7895	516.8072	1032.607	1.32	0.00068	2.489809
P01009	2	1	821.4711	4103.326	2.56	0.0021	54.49115
P01009	0	0.9697	481.7244	1923.876	2.96	0.00142	22.51917
P01009	1	0.9091	570.004	1707.997	3.94	0.00224	11.98104
P01009	0	0.963	517.9395	1551.804	1.55	0.0008	2.921595
P01009	0	1	512.6069	1535.806	-0.27	-0.00014	18.46076
P01009	0	0.7059	466.942	1398.812	2.33	0.00108	2.619329
P01009	1	0.9333	498.3108	1492.918	0.92	0.00046	56.97076
P01009	1	0.9333	498.3108	1492.918	0.92	0.00046	56.97076
P01009	0	1	782.7225	2346.153	1.64	0.00128	0
P01009	0	0.9706	768.4095	1535.812	3.39	0.0026	0
P01009	0	1	698.0629	2092.174	4.77	0.00333	46.18001
P01009	0	1	517.9391	1551.803	0.84	0.00044	16.23649
P01009	0	0.7727	487.7708	1948.062	-0.23	-0.00011	15.97938
P01009	0	0.5536	534.3134	1067.62	3.19	0.0017	52.66739
P01009	1	0.9048	564.6407	1691.908	-0.05	-0.00003	25.19209
P01009	0	0.8788	679.3271	2035.967	4.45	0.00302	23.23125
P01009	0	1	512.6085	1535.811	2.83	0.00145	49.13342
P01009	0	1	517.7485	2067.972	-0.08	-0.00004	10.75574
P01009	0	1	517.939	1551.802	0.61	0.00031	19.86617
P01009	0	1	512.6079	1535.809	1.75	0.0009	35.8756
P01009	0	0.9091	517.9392	1551.803	0.96	0.0005	0
P01009	0	1	517.7513	2067.983	5.35	0.00276	18.78118
P01009	0	0.6757	588.8591	1176.711	2.71	0.00159	9.181252
P01009	1	1	551.6777	1653.018	3.11	0.00171	32.65193
P01009	0	0.9167	517.7504	2067.98	3.69	0.00191	4.244313
P01009	0	0.6786	380.9051	1140.701	2.47	0.00094	7.936327

P01009	0	0.6744	576.8069	1152.606	4.15	0.00239	9.460225
P01009	0	0.4667	432.9063	1296.704	0.46	0.0002	0
P01009	0	0.8148	380.9052	1140.701	2.63	0.001	11.21393
P01009	0	1	469.9053	1407.701	1.44	0.00067	46.5127
P01009	0	0.566	534.311	1067.615	-1.27	-0.00068	0
P01009	0	0.6047	534.3125	1067.618	1.48	0.00079	0
P01009	0	0.8889	481.7241	1923.875	2.33	0.00112	0
P01009	0	1	734.6887	2202.052	2.03	0.00149	10.25356
P01009	0	0.5909	534.313	1067.619	2.39	0.00128	43.99587
P01009	0	1	464.5738	1391.707	1.72	0.0008	15.97469
P01009	0	0.9231	460.2635	1378.776	2.68	0.00123	14.56658
P01009	0	0.7917	380.9053	1140.701	3.03	0.00115	0
P01009	0	0.8621	679.3259	2035.963	2.65	0.0018	0
P01009	0	0.9375	481.7244	1923.876	2.83	0.00136	27.36067
P01009	0	1	580.3643	1159.721	3.93	0.00228	29.44305
P01009	0	0.75	588.8596	1176.712	3.43	0.00202	48.50661
P01009	0	0.9444	517.94	1551.805	2.61	0.00135	66.69722
P01009	0	1	460.263	1378.774	1.61	0.00074	65.71242
P01009	0	0.7143	380.9054	1140.702	3.35	0.00127	18.26698
P01009	0	0.6111	387.2445	1159.719	2.05	0.00079	0
P01009	0	0.8929	734.6871	2202.047	-0.13	-0.0001	43.43968
P01009	0	0.6444	542.311	1083.615	3.33	0.0018	8.749046
P01009	0	0.62	534.3124	1067.618	1.36	0.00073	14.71317
P01009	0	1	481.7239	1923.874	1.76	0.00084	19.45606
P01009	0	1	460.2625	1378.773	0.48	0.00022	13.02051
P01009	0	0.6078	534.3148	1067.622	5.71	0.00305	12.7456
P01009	0	0.6316	517.9397	1551.805	2.02	0.00105	9.649161
P01009	0	1	512.6086	1535.811	3.18	0.00163	18.42324
P01009	0	0.5938	570.8543	1140.701	2.91	0.00166	10.23295
P01009	0	0.7727	517.94	1551.805	2.61	0.00135	7.889136
P01009	0	1	460.263	1378.774	1.68	0.00077	10.6939
P01009	0	0.85	517.9397	1551.805	2.02	0.00105	36.80188
P01009	0	1	460.2644	1378.779	4.67	0.00215	27.10742
P01009	0	0.6875	587.2951	2346.159	4	0.00235	20.28561
P01009	1	0.9375	564.6428	1691.914	3.73	0.00211	0
P01009	0	0.5	466.9423	1398.812	2.92	0.00136	6.061275
P01009	0	0.6207	570.8543	1140.701	2.91	0.00166	0
P01009	0	0.9091	517.9396	1551.804	1.9	0.00099	5.780658
P01009	0	1	641.9638	1923.877	3.48	0.00223	0
P01009	0	0.7222	588.8605	1176.714	5.09	0.003	0
P01009	0	0.64	699.9097	1398.812	2.73	0.00191	15.97701
P01009	0	0.9474	517.9407	1551.807	3.91	0.00202	16.26337
P01009	0	0.5714	542.3112	1083.615	3.78	0.00205	0
P01009	0	0.75	460.2631	1378.775	1.81	0.00083	18.47471
P01009	0	0.8421	517.9398	1551.805	2.26	0.00117	0
P01009	0	0.75	782.7217	2346.15	0.55	0.00043	60.65384
P01009	0	0.7143	414.4016	2067.979	3.24	0.00134	19.46744
P01009	0	0.7273	380.9052	1140.701	2.71	0.00103	15.84191
P01009	0	0.9231	517.9398	1551.805	2.14	0.00111	19.50717
P01009	0	0.5	487.7712	1948.063	0.45	0.00022	24.09866
P01009	0	1	481.7243	1923.875	2.71	0.0013	49.24791
P01009	0	0.8421	517.9405	1551.807	3.56	0.00184	60.73491

P01009	0	0.9375	698.0612	2092.169	2.41	0.00168	62.37026
P00734	0	1	513.96	1539.866	2.24	0.00115	49.76067
P00734	0	1	770.4363	1539.865	2.17	0.00167	6.47825
P00734	0	0.95	513.9602	1539.866	2.59	0.00133	8.753925
P00734	1	1	859.423	2576.254	1.71	0.00147	4.394521
P00734	1	1	859.4229	2576.254	1.56	0.00134	2.067361
P00734	1	1	859.4241	2576.258	2.99	0.00256	6.401958
P00734	0	1	760.0465	2278.125	1.84	0.0014	11.75831
P00734	0	1	570.2874	2278.128	3.09	0.00176	6.360729
P00734	0	1	760.0465	2278.125	1.76	0.00133	5.774871
P00734	1	1	644.8192	2576.255	1.96	0.00126	2.082619
P00734	0	0.8043	646.8308	1292.654	0.35	0.00023	3.333771
P00734	0	0.7925	431.557	1292.656	2.09	0.0009	5.480378
P00734	1	1	859.4225	2576.253	1.14	0.00098	2.029225
P00734	0	0.8246	569.283	1705.835	2.52	0.00143	12.54033
P00734	0	1	853.4199	1705.833	1.38	0.00118	2.980192
P00734	0	1	705.0024	2112.993	0.23	0.00016	18.14559
P00734	0	1	705.0033	2112.995	1.44	0.00102	2.599225
P00734	0	1	1057.002	2112.997	2.06	0.00217	3.483287
P00734	0	1	705.0029	2112.994	0.84	0.00059	2.48871
P00734	0	1	781.369	1561.731	1.6	0.00125	0
P00734	0	0.931	669.855	1338.703	0.39	0.00026	3.307719
P00734	0	0.6863	669.8559	1338.705	1.76	0.00118	6.121565
P00734	0	1	767.0202	2299.046	1.88	0.00144	3.114969
P00734	0	0.9365	1108.044	2215.082	1.57	0.00173	0
P00734	0	1	739.0319	2215.081	1.31	0.00097	2.722798
P00734	0	1	445.2326	1333.683	1.72	0.00076	17.18909
P00734	1	1	864.7549	2592.25	2.08	0.0018	2.556294
P00734	0	1	703.3452	2108.021	5.27	0.0037	31.528
P00734	0	0.8367	581.8462	1162.685	2.45	0.00143	4.296452
P00734	0	1	576.3145	2302.236	4.61	0.00265	39.08431
P00734	0	1	576.3131	2302.231	2.17	0.00125	4.791936
P00734	0	0.9778	768.0817	2302.23	2.09	0.00161	5.875336
P00734	0	1	576.3126	2302.228	1.21	0.0007	4.861984
P00734	0	1	768.081	2302.228	1.22	0.00094	7.864848
P00734	0	1	527.7585	2108.012	1.01	0.00053	1.476094
P00734	0	1	895.4666	1789.926	1.39	0.00125	3.182745
P00734	0	0.9444	597.3143	1789.928	2.68	0.0016	5.261855
P00734	0	0.9726	703.3425	2108.013	1.45	0.00102	2.325186
P00734	0	1	655.3092	1963.913	2.62	0.00171	20.78196
P00734	0	1	703.3394	2108.004	-2.98	-0.0021	0
P00734	0	0.7959	597.3126	1789.923	-0.08	-0.00005	0
P00734	0	0.9718	703.3427	2108.013	1.62	0.00114	3.511152
P00734	1	0.9851	688.0467	2062.126	2.44	0.00168	7.226352
P00734	1	0.9773	1031.566	2062.124	1.74	0.00179	0
P00734	1	1	516.2864	2062.124	1.55	0.0008	8.826856
P00734	1	0.987	688.0452	2062.121	0.22	0.00015	8.166163
P00734	1	0.9219	516.286	2062.122	0.84	0.00043	4.71712
P00734	1	0.963	688.0465	2062.125	2.09	0.00143	20.99533
P00734	1	0.8519	640.0125	1918.023	2.38	0.00152	2.811429
P00734	1	0.9057	640.0141	1918.028	4.86	0.00311	2.680002
P00734	1	0.9057	640.0141	1918.028	4.86	0.00311	2.680002

P00734	1	0.8519	640.0125	1918.023	2.38	0.00152	2.811429
P00734	0	1	853.4196	1705.832	0.95	0.00081	3.597428
P00734	0	0.875	569.2823	1705.832	1.23	0.0007	4.208252
P00734	0	1	543.7813	2172.103	1.69	0.00092	0
P00734	0	1	724.7054	2172.102	0.93	0.00068	5.923318
P00734	0	1	676.6718	2028.001	1.58	0.00107	2.497174
P00734	0	1	724.7058	2172.103	1.44	0.00104	4.902156
P00734	0	1	543.7815	2172.104	2.02	0.0011	14.46146
P00734	1	1	609.3127	2434.229	2.88	0.00175	14.84324
P00734	0	1	504.8974	2520.458	0.13	0.00007	4.606958
P00734	0	0.9839	630.8705	2520.46	1.07	0.00067	4.616477
P00734	0	0.9487	840.825	2520.46	1.22	0.00102	4.698193
P00734	0	0.9744	605.6158	1814.833	2.12	0.00128	46.89226
P00734	0	1	907.9196	1814.832	1.56	0.00141	6.731294
P00734	0	1	605.6163	1814.834	2.83	0.00171	8.451031
P00734	0	0.9615	835.8682	1670.729	1.26	0.00105	0
P00734	0	1	668.6349	2003.89	2.04	0.00136	27.87104
P00734	0	0.9255	739.0323	2215.082	1.89	0.0014	35.10147
P00734	0	0.9519	690.9977	2070.979	1.26	0.00087	0
P00734	0	0.7397	597.8055	1194.604	3.11	0.00186	0
P00734	0	1	703.3422	2108.012	0.93	0.00065	8.029567
P00734	0	1	1035.993	2070.979	1.33	0.00137	1.041664
P00734	0	0.9221	1108.045	2215.082	1.68	0.00186	0.448723
P00734	0	0.9506	739.0313	2215.079	0.49	0.00036	2.463844
P00734	0	0.9516	690.9977	2070.978	1.17	0.00081	8.762603
P00734	0	1	994.451	1987.895	1.8	0.00179	1.049487
P00734	0	1	663.3029	1987.894	1.46	0.00097	2.437717
P00734	0	1	663.3027	1987.893	1.18	0.00078	27.02884
P00734	0	1	610.9478	1830.829	2.65	0.00162	12.4272
P00734	1	0.9474	657.65	1970.935	2.59	0.0017	6.036096
P00734	1	1	493.4888	1970.933	1.53	0.00075	7.657181
P00734	0	0.9286	753.4078	2258.209	2.45	0.00184	13.15956
P00734	0	1	610.9472	1830.827	1.65	0.001	18.44653
P00734	0	0.8571	565.3051	2258.199	-2.12	-0.0012	0
P00734	0	1	1205.109	2409.211	1.84	0.00222	0
P00734	0	1	698.3842	1395.761	0.82	0.00058	0
P00734	0	1	576.3134	2302.232	2.59	0.00149	3.996655
P00734	0	1	761.7562	2283.254	0.78	0.00059	8.970378
P00734	0	0.9787	571.5687	2283.253	0.29	0.00017	4.838962
P00734	0	0.8923	713.7227	2139.153	1.55	0.0011	35.75386
P00734	0	0.9714	576.3151	2302.238	5.56	0.0032	13.1512
P00734	0	0.9429	513.9597	1539.865	1.64	0.00084	9.704891
P00734	1	0.8571	644.8188	2576.253	1.3	0.00084	37.07825
P00734	0	0.7812	509.7947	1018.582	1.85	0.00094	0
P00734	0	0.8824	739.0329	2215.084	2.72	0.00201	68.18793
P00734	0	0.7963	581.8447	1162.682	-0.17	-0.0001	5.742953
P00734	0	1	676.6728	2028.004	3.02	0.00204	3.791553
P00734	1	0.8824	688.0481	2062.13	4.49	0.00308	34.32111
P00734	0	1	630.8732	2520.471	5.33	0.00336	49.17168
P00734	0	0.92	703.3426	2108.013	1.53	0.00108	26.67206
P00734	0	0.6857	575.3061	1149.605	2.49	0.00143	11.07978
P00734	0	0.7	431.5569	1292.656	1.73	0.00075	0

P00734	0	1	610.949	1830.832	4.55	0.00277	40.37957
P00734	0	1	610.9483	1830.83	3.45	0.0021	29.62363
P00734	0	1	669.8556	1338.704	1.31	0.00087	42.91862
P00734	0	0.8431	647.3538	1293.7	-2.9	-0.00187	31.49639
P00734	0	1	668.6356	2003.892	3.13	0.00209	23.29771
P00734	0	0.7903	509.7946	1018.582	1.73	0.00088	3.200408
P00734	0	1	397.1983	1189.58	1.3	0.00051	28.84335
P00734	0	1	705	2112.985	-3.24	-0.00228	69.70944
P00734	0	1	663.3024	1987.893	0.82	0.00054	65.98457
P00734	0	0.9444	570.2867	2278.125	1.91	0.00109	0
P00734	0	0.8864	509.7953	1018.583	2.99	0.00152	6.146496
P00734	0	0.7083	581.8462	1162.685	2.45	0.00143	25.93431
P00734	0	0.8611	558.2686	1115.53	2.39	0.00133	2.790368
P00734	0	1	513.9605	1539.867	3.19	0.00164	0
P00734	0	0.9048	739.0341	2215.088	4.37	0.00323	0
P00734	0	0.7895	509.7953	1018.583	2.99	0.00152	5.348005
P00734	0	0.7273	581.8453	1162.683	0.88	0.00051	62.92154
P00734	0	0.6522	540.2882	2158.131	3.34	0.0018	5.673255
P00734	0	0.6522	540.2882	2158.131	3.34	0.0018	5.673255
P00734	0	1	574.2844	2294.116	0.07	0.00004	20.45494
P00734	0	0.8298	581.8462	1162.685	2.45	0.00143	62.14581
P00734	0	1	576.3115	2302.224	-0.59	-0.00034	0
P00734	0	0.8824	739.0336	2215.086	3.71	0.00274	27.80495
P00734	0	0.0417	388.2329	1162.684	1.73	0.00067	9.63743
P00734	0	0.8889	570.2893	2278.135	6.41	0.00365	12.01935
P00734	0	0.7333	753.4132	2258.225	9.5	0.00715	54.92905
P00734	0	1	513.9604	1539.867	2.95	0.00151	33.1649
P00734	0	0.7727	669.8563	1338.705	2.31	0.00155	69.71092
P00734	0	1	605.6157	1814.832	1.82	0.0011	33.17545
P00734	0	0.7857	461.2517	2302.229	1.59	0.00073	12.70343
P00734	1	0.8333	688.0462	2062.124	1.73	0.00119	42.66354
P00734	0	0.9143	558.2693	1115.531	3.7	0.00206	12.96578
P00734	0	1	767.021	2299.048	2.91	0.00223	62.64981
P10643	0	1	1036.144	3106.418	2.19	0.00227	2.642919
P10643	0	1	777.359	3106.414	0.79	0.00062	50.86609
P10643	0	1	525.9396	1575.804	1.76	0.00092	28.08379
P10643	0	0.7368	469.9095	1407.714	1.69	0.00079	12.52279
P10643	0	0.9697	427.8866	1281.645	2.14	0.00092	14.3509
P10643	0	1	724.3167	2170.935	1.39	0.001	26.63113
P10643	0	0.9792	638.9906	1914.957	3.28	0.0021	0
P10643	0	1	801.0701	2401.196	2.17	0.00173	2.03494
P10643	0	0.9077	682.4271	1363.847	2.25	0.00154	10.14361
P10643	0	0.9062	682.4254	1363.843	-0.34	-0.00023	8.723853
P10643	0	0.8462	694.3905	1387.774	1.87	0.0013	8.763278
P10643	0	1	525.9394	1575.804	1.41	0.00074	20.85897
P10643	0	1	801.0695	2401.194	1.48	0.00118	2.164092
P10643	0	1	653.3319	1957.981	1.82	0.00119	2.687197
P10643	0	0.9394	733.8752	1466.743	2.25	0.00165	6.70365
P10643	0	1	489.586	1466.743	2.35	0.00115	18.54493
P10643	0	0.8824	401.9623	1604.827	0.21	0.00008	67.14551
P10643	0	0.9048	637.8518	1274.696	2.36	0.00151	3.344769
P10643	0	0.8367	425.5698	1274.695	1.25	0.00053	19.06293

P10643	0	1	644.3236	1930.956	5.38	0.00347	0
P10643	0	1	582.0019	1743.991	1.6	0.00093	7.38562
P10643	0	0.9818	698.7225	2094.153	2.63	0.00184	0
P10643	0	0.9348	698.7224	2094.153	2.46	0.00172	100
P10643	0	0.9733	907.1173	2719.337	1.06	0.00096	0
P10643	0	1	672.7707	3359.824	0.71	0.00048	23.96056
P10643	0	1	542.5854	1625.742	2.24	0.00121	31.23117
P10643	0	1	813.3738	1625.74	1.42	0.00116	1.472206
P10643	0	1	542.5847	1625.739	0.89	0.00048	4.334108
P10643	0	0.9231	813.3736	1625.74	1.2	0.00097	3.400315
P10643	1	1	563.3205	2250.26	5.2	0.00292	55.59766
P10643	0	0.9167	542.5851	1625.741	1.79	0.00097	13.58461
P10643	0	1	531.2704	1591.797	0.18	0.0001	24.6174
P10643	0	1	641.3257	1281.644	1.25	0.0008	2.998437
P10643	0	1	653.3331	1957.985	3.6	0.00235	56.40202
P10643	0	0.8056	455.287	1363.847	1.91	0.00087	9.55512
P10643	0	1	698.2927	1395.578	2.24	0.00156	14.00042
P10643; Q1	0	0.3182	552.8297	1104.652	1.58	0.00087	37.41586
P10643	0	1	840.7129	3359.83	2.31	0.00194	4.799808
P10643	0	1	394.707	1575.806	2.95	0.00116	0
P10643	0	0.5429	650.6876	1950.048	1.55	0.00101	3.61094
P10643	0	0.9778	622.3403	1243.673	3.48	0.00216	12.59542
P10643	0	0.7241	499.9193	1497.743	0.75	0.00038	5.025897
P10643	0	0.7333	393.2219	1177.651	1.81	0.00071	14.51093
P10643	0	0.95	411.2618	1231.771	1.68	0.00069	37.88374
P10643	0	1	555.7857	1110.564	3.34	0.00185	0
P10643	0	0.5556	704.3622	1407.717	4.04	0.00284	3.339082
P10643	0	1	821.3718	1641.736	2.13	0.00175	0
P10643	0	0.7188	667.879	1334.751	3.64	0.00243	0
P10643	0	1	547.9149	1641.73	-1.63	-0.00089	14.22023
P10643	0	0.9286	445.5875	1334.748	1.58	0.0007	36.26385
P10643; Q1	0	0.1795	552.8323	1104.657	6.33	0.00349	14.71871
P10643	0	1	445.5876	1334.748	1.78	0.00079	47.78157
P10643	0	0.88	489.5856	1466.742	1.54	0.00075	9.343733
P10643	0	1	813.3751	1625.743	3	0.00244	45.85756
P01008	1	1	655.0224	1963.053	2.01	0.00132	8.555601
P01008	1	1	558.9554	1674.852	4.19	0.00234	6.044933
P01008	1	1	472.2924	1414.863	0.49	0.00023	3.490838
P01008	0	1	1069.066	2137.125	7.66	0.00819	22.66182
P01008	0	0.9091	713.0447	2137.12	5.03	0.00358	12.47531
P01008	0	0.7727	665.0089	1993.012	2.67	0.00178	5.004417
P01008	0	0.9209	997.0103	1993.013	3.26	0.00325	7.580603
P01008	0	1	461.228	1381.67	0.73	0.00034	11.10926
P01008	1	0.9773	551.9613	1653.869	2.31	0.00127	12.91835
P01008	0	0.625	713.0425	2137.113	1.86	0.00132	0
P01008	0	0.975	459.6146	1376.829	0.67	0.00031	2.431956
P01008	0	0.7368	459.6141	1376.828	-0.39	-0.00018	4.859914
P01008	0	0.8864	606.322	1816.951	2.27	0.00138	65.34048
P01008	1	1	472.2929	1414.864	1.59	0.00075	7.565009
P01008	1	1	472.2932	1414.865	2.17	0.00103	53.76545
P01008	0	1	691.3391	1381.671	1.76	0.00122	8.470169
P01008	1	0.0208	424.2588	1270.762	1.4	0.00059	15.20503

P01008	0	0.9492	558.2877	1672.849	2.08	0.00116	4.363789
P01008	0	0.878	558.2885	1672.851	3.39	0.00189	8.292937
P01008	1	0.98	551.9605	1653.867	0.76	0.00042	6.685843
P01008	0	0.9444	777.3744	1553.742	1.3	0.00101	3.103992
P01008	0	0.9385	606.3212	1816.949	0.96	0.00058	8.578529
P01008	0	0.96	454.9929	1816.95	1.33	0.0006	8.737457
P01008	0	0.9375	908.9791	1816.951	2.01	0.00183	5.757835
P01008	0	0.931	606.3209	1816.948	0.56	0.00034	7.967163
P01008	0	0.9362	495.594	1484.767	1.35	0.00067	6.22054
P01008	0	0.9714	543.6278	1628.869	0.85	0.00046	18.1036
P01008	0	0.8727	814.9382	1628.869	0.97	0.00079	9.03714
P01008	0	0.8718	543.6282	1628.87	1.52	0.00083	8.595205
P01008	0	0.8511	814.9373	1628.867	-0.08	-0.00007	0
P01008	0	0.9167	543.6282	1628.87	1.63	0.00089	38.73887
P01008	0	1	691.3387	1381.67	1.14	0.00079	8.002794
P01008	1	1	625.7056	1875.102	4.24	0.00265	13.23384
P01008	0	0.807	528.3209	1055.635	1.53	0.00081	16.72213
P01008	0	0.825	718.3738	2153.107	1.37	0.00098	9.284603
P01008	0	0.9592	1069.06	2137.113	2.06	0.00221	0
P01008	0	0.7792	713.0421	2137.112	1.26	0.0009	4.931117
P01008	0	0.8514	860.0015	1718.996	1.57	0.00135	6.480305
P01008	0	0.8077	573.6694	1718.994	0.28	0.00016	8.617599
P01008	0	0.95	701.0816	2101.23	0	0	8.351913
P01008	0	0.9839	526.064	2101.234	1.91	0.001	6.438447
P01008	0	1	701.0826	2101.233	1.48	0.00104	10.83515
P01008	0	1	526.0634	2101.232	0.75	0.00039	18.27925
P01008	0	0.7586	799.4103	1597.813	2.03	0.00162	10.38351
P01008	0	1	533.276	1597.813	2.14	0.00114	9.060894
P01008	0	1	533.2753	1597.811	0.76	0.00041	10.33013
P01008	0	0.2037	685.6293	2054.873	1.32	0.0009	2.700342
P01008	2	1	763.45	3813.221	4.22	0.00322	16.86756
P01008	0	0.194	685.6296	2054.874	1.76	0.00121	6.667533
P01008	0	1	691.3392	1381.671	1.85	0.00128	12.77742
P01008	1	1	915.0343	3657.115	3.26	0.00298	18.34693
P01008	0	1	691.3398	1381.672	2.73	0.00189	0
P01008	0	1	767.4344	1533.862	2.32	0.00178	3.862999
P01008	0	1	767.4338	1533.86	1.52	0.00117	7.468942
P01008	0	1	680.298	2038.879	1.74	0.00118	39.52968
P01008	0	1	533.2748	1597.81	-0.15	-0.00008	70.36616
P01008	1	0.9744	551.96	1653.866	-0.02	-0.00001	18.82848
P01008	0	1	459.6142	1376.828	-0.12	-0.00006	15.79805
P01008	0	0.9802	812.4237	2435.256	1.21	0.00098	0
P01008	0	1	691.3391	1381.671	1.76	0.00122	5.048982
P01008	1	1	551.9601	1653.866	0.09	0.00005	30.0529
P01008	1	1	721.6984	2163.081	1.74	0.00126	6.939695
P01008	1		424.2588	1270.762	1.4	0.00059	15.20503
P01008	0	0.9756	461.2286	1381.671	1.86	0.00086	4.196344
P01008	0	0.6667	598.8439	1196.68	3.32	0.00198	5.639536
P01008	0	1	685.6298	2054.875	2.03	0.00139	29.25652
P01008	0	1	685.6298	2054.875	2.03	0.00139	29.25652
P01008	0	0.8444	495.5936	1484.766	0.61	0.0003	60.71108
P01008	0	1	691.3371	1381.667	-1.16	-0.0008	0

P01008	0	1	701.0823	2101.232	0.96	0.00067	36.74249
P01008	0	1	461.2286	1381.671	1.99	0.00092	37.48006
P01008	0	0.9091	495.5942	1484.768	1.66	0.00082	0
P01008	0	1	543.6285	1628.871	2.08	0.00113	42.52659
P01008	0	0.6667	454.9929	1816.95	1.33	0.0006	4.059965
P01008	0	0.9787	691.3397	1381.672	2.56	0.00177	4.04736
P01008	0	0.7391	407.9727	1628.869	0.95	0.00039	0
P01008	0	0.6905	598.8402	1196.673	-2.81	-0.00168	14.13431
P01008	0	0.6957	407.9727	1628.869	0.88	0.00036	0
P01008	1	1	472.2941	1414.868	4.05	0.00191	48.20914
P01008	0	0.3571	787.9496	1574.892	0.55	0.00044	11.68216
P01008	1		402.5805	1205.727	2.01	0.00081	13.49336
P01008	0	0.975	543.6279	1628.869	1.07	0.00058	24.526
P01008	0	0.7719	526.7916	1052.576	1.46	0.00077	2.342458
P01008	0	0.6842	564.3146	1127.622	2.97	0.00167	18.88368
P01008	0	1	543.6292	1628.873	3.43	0.00186	21.51781
P01008	0	0.8148	528.3214	1055.635	2.34	0.00123	3.301655
P01008	0	0.7857	713.0436	2137.116	3.49	0.00248	23.45052
P01008	0	0.5	606.3241	1816.958	5.8	0.00351	15.05096
P01008	0	0.8846	573.6714	1719	3.8	0.00217	3.99653
P01008	2	0.875	409.2416	1633.944	4.19	0.00171	21.16237
P01008	0	0.6735	564.3146	1127.622	2.86	0.00161	6.180216
P01008	1	0.7879	551.9616	1653.87	2.86	0.00158	12.15017
P01008	1	1	586.2687	2342.053	3.21	0.00188	0
P01008	0	0.7931	606.3229	1816.954	3.89	0.00235	6.560986
P01008	0	1	685.632	2054.881	5.15	0.00353	0
P01008	0	1	685.632	2054.881	5.15	0.00353	0
P01008	1	0.8333	625.7039	1875.097	1.51	0.00094	0
P01008	0	0.25	718.3731	2153.105	0.35	0.00025	0
P01008	0	1	695.3832	1389.759	2.39	0.00166	36.70161
P01008	1	0.7895	417.2541	1249.748	0.93	0.00039	6.831983
P01008	0	0.85	533.2758	1597.813	1.68	0.00089	5.076517
P01008	0	0.88	454.9936	1816.952	2.87	0.0013	41.03553
P01008	0	0.5172	376.5447	1127.619	0.74	0.00028	15.15065
P01008	0	1	691.3394	1381.671	2.11	0.00146	3.125758
P01008	0	0.6667	461.2298	1381.675	4.51	0.00208	25.69714
P01008	0	1	680.2991	2038.883	3.45	0.00234	43.75751
P01008	0	0.4375	718.374	2153.107	1.63	0.00117	12.90257
P01008	0	0.84	777.3729	1553.738	-0.67	-0.00052	12.10988
P01008	0	0.6364	564.3145	1127.622	2.64	0.00149	15.29742
P01008	0	0.8462	574.8502	1148.693	1.12	0.00064	11.74024
P01008	1	0	402.5804	1205.727	1.78	0.00071	23.94239
P01008	1	0.7353	625.3781	1249.749	1.84	0.00115	2.335834
P01008	0	0.625	407.9733	1628.871	2.3	0.00094	0
P01008	0	0.8125	533.2741	1597.808	-1.41	-0.00075	12.47126
P01008	1		402.5798	1205.725	0.26	0.0001	0
P01008	0	0.8065	688.9188	1376.83	1.5	0.00103	15.91041
P01008	0	0.9	574.8508	1148.694	2.29	0.00131	29.4141
P01008	0	0.7143	407.9739	1628.874	3.87	0.00158	0
P01008	0	1	518.5856	1553.742	1.84	0.00095	21.30715
P01008	0	0.9048	383.5687	1148.692	-0.12	-0.00004	7.819993
P01008	1	1	781.3564	2342.055	3.95	0.00308	8.452435

P13671	0	0.9516	638.3417	1275.676	2.86	0.00182	16.10202
P13671	0	0.973	826.1	2476.285	1.79	0.00148	3.049857
P13671	0	0.9746	826.1001	2476.286	1.94	0.0016	4.455562
P13671	0	0.989	826.1001	2476.286	1.94	0.0016	7.03271
P13671	0	0.9714	611.0492	2441.175	2.73	0.00166	30.82302
P13671	0	1	669.0273	2005.067	2.47	0.00165	19.04386
P13671	0	0.8286	570.6334	1709.886	4.55	0.00259	14.27956
P13671	0	1	669.0269	2005.066	1.83	0.00123	1.57153
P13671	0	1	669.0266	2005.065	1.47	0.00098	0
P13671	0	0.8333	570.6318	1709.881	1.77	0.00101	3.807343
P13671	0	0.7451	505.2769	1513.816	1.29	0.00065	10.28144
P13671	0	0.9048	757.4119	1513.817	1.66	0.00126	100
P13671	0	1	718.7366	2154.195	1.15	0.00083	2.044003
P13671	0	1	1077.602	2154.196	1.58	0.00171	0
P13671	0	1	981.1137	2941.326	1.12	0.0011	5.687417
P13671	1	0.95	426.9815	1704.904	1.37	0.00059	27.22173
P13671	0	0.6863	591.3431	1181.679	1.83	0.00108	15.73541
P13671	0	0.8462	390.8998	1170.685	1.33	0.00052	36.13808
P13671	1	0.9259	426.9824	1704.908	3.31	0.00141	24.37625
P13671	1	1	568.9727	1704.903	0.84	0.00048	0
P13671	0	1	686.318	2056.939	2.32	0.00159	32.20995
P13671	0	0.8367	512.2855	1534.842	1.92	0.00098	6.602458
P13671	0	0.8529	390.8997	1170.685	1.1	0.00043	42.50411
P13671	0	0.9423	585.848	1170.689	4.55	0.00266	0
P13671	0	0.9778	892.96	1784.913	2.06	0.00184	6.604506
P13671	0	0.939	595.6427	1784.914	2.49	0.00148	7.826789
P13671	0	0.7234	547.8019	1094.597	3.14	0.00172	7.152237
P13671	0	1	516.9406	1548.807	4.14	0.00214	53.79244
P13671	1	0.8889	375.9785	1500.892	-0.66	-0.00025	34.51408
P13671	1	1	375.9805	1500.9	4.63	0.00174	17.11764
P13671	0	1	390.9005	1170.687	3.06	0.00119	70.66444
P13671	0	0.85	669.0267	2005.066	1.65	0.0011	33.55584
P13671	1	0.9655	495.6384	1484.901	1.57	0.00078	0
P13671	0	0.6744	576.7957	1152.584	2.84	0.00163	14.9211
P13671	1	1	758.3466	2273.025	1.69	0.00128	18.26722
P13671	0	1	814.3945	2441.169	0.31	0.00025	0
P13671	0	0.7297	710.3926	1419.778	2.39	0.0017	0
P13671	0	1	667.6138	2000.827	1.16	0.00077	10.16739
P13671	0	0.7812	473.9306	1419.777	1.93	0.00091	22.29041
P13671	0	0.9024	517.2996	1033.592	0.73	0.00038	11.05105
P13671	0	0.6585	576.7957	1152.584	2.84	0.00163	5.53384
P13671	1	1	534.3165	1600.935	2.06	0.0011	19.86281
P13671	0	0.6	410.2375	1228.698	1.4	0.00057	15.49434
P13671	0	0.5714	620.9923	1860.962	1.03	0.00064	0
P13671	0	0.1667	492.2888	1474.852	0.7	0.00034	24.1463
P13671	0	1	523.794	1046.581	2.04	0.00107	41.30789
P13671	1	0.7429	742.9531	1484.899	0.45	0.00034	0
P13671	0	0.84	504.937	1512.796	1.87	0.00094	7.761663
P13671	1	1	400.9893	1600.935	2.25	0.0009	11.38574
P13671	0	0.7241	394.5646	1181.679	1.98	0.00078	30.83162
P13671	0	0.8846	512.2856	1534.842	2.27	0.00116	17.65926
P13671	0	1	397.5658	1190.683	1.93	0.00076	5.471869

P13671	0	0.5714	405.8893	1215.653	2.26	0.00092	29.38384
P13671	0	0.25	505.277	1513.817	1.66	0.00084	0
P13671	0	0.625	405.889	1215.652	1.51	0.00061	51.00243
P13671	0	0.963	608.3303	1215.653	2.25	0.00137	28.56708
P13671	0	0.56	757.4141	1513.821	4.57	0.00346	5.035923
P13671	0	0.5	504.9363	1512.794	0.36	0.00018	20.19027
P13671	0	0.7826	517.2994	1033.592	0.49	0.00026	44.12528
P43652	0	0.8	442.2485	1324.731	1.82	0.0008	30.10625
P43652	0	0.9254	638.8432	1276.679	3.69	0.00236	5.401321
P43652	0	1	486.9039	1458.697	1.42	0.00069	37.00771
P43652	0	1	1054.153	3160.445	2.96	0.00312	8.888538
P43652	0	1	671.9017	1342.796	2.09	0.0014	8.7066
P43652	0	0.9444	638.8427	1276.678	2.93	0.00187	6.047142
P43652	1	1	530.6473	1589.927	1.94	0.00103	5.831529
P43652	0	1	1054.153	3160.444	2.84	0.00299	8.709172
P43652	0	1	790.866	3160.442	2.2	0.00174	3.166996
P43652	0	0.7647	442.2485	1324.731	1.82	0.0008	8.517861
P43652	0	0.7812	442.2484	1324.731	1.68	0.00074	10.9256
P43652	0	1	865.1037	2593.297	2.44	0.00211	0
P43652	0	1	1016.526	2032.044	2.31	0.00235	1.8546
P43652	0	0.8605	544.7912	2176.143	0.63	0.00034	4.044751
P43652	0	1	486.903	1458.694	-0.33	-0.00016	8.392772
P43652	0	0.7222	478.6136	1433.826	2.04	0.00098	14.4332
P43652	1	0.8333	398.2375	1589.928	2.43	0.00096	7.338655
P43652	1	1	530.6474	1589.928	2.05	0.00109	4.593416
P43652	1	0.9412	398.2369	1589.926	0.81	0.00032	0
P43652	1	1	530.6473	1589.927	1.94	0.00103	0
P43652	1	0.9701	530.6473	1589.927	1.94	0.00103	4.436812
P43652	0	0.6875	440.9434	1320.816	2.1	0.00092	8.158227
P43652	0	0.7727	660.9116	1320.816	2.3	0.00152	6.18975
P43652	0	1	1088.577	2176.146	2.04	0.00222	0
P43652	1	0.9615	398.2376	1589.928	2.58	0.00103	4.560969
P43652	1	1	530.6474	1589.928	2.05	0.00109	4.689648
P43652	1	0.84	398.2377	1589.929	2.89	0.00115	4.004899
P43652	1	1	530.6473	1589.927	1.82	0.00096	4.000896
P43652	1	1	482.6125	1445.823	0.34	0.00017	2.008573
P43652	0	1	913.1375	2737.398	2.08	0.0019	3.910165
P43652	0	1	685.1052	2737.399	2.48	0.0017	5.704614
P43652	0	0.9048	478.9289	1434.772	2.32	0.00111	11.15421
P43652	0	1	1129.181	3385.529	1.08	0.00122	0
P43652	0	1	883.1658	3529.641	3.95	0.00349	9.399137
P43652	1	0.8182	612.8416	2448.344	2.35	0.00144	6.264833
P43652	1	0.8704	816.7854	2448.342	1.24	0.00101	6.135121
P43652	1	1	745.4	2978.578	2.34	0.00174	8.194013
P43652	1	1	745.4	2978.578	2.34	0.00174	8.194013
P43652	0	0.95	668.0504	2002.137	2.74	0.00183	0
P43652	0	0.7797	668.0509	2002.138	3.56	0.00237	23.41767
P43652	0	0.7708	1001.572	2002.137	2.74	0.00274	7.144054
P43652	0	1	668.0494	2002.134	1.36	0.00091	7.795515
P43652	1	0.9855	781.4256	3122.681	2.3	0.0018	4.736406
P43652	1	1	1041.572	3122.701	8.89	0.00926	0
P43652	0	1	707.8594	1414.711	1.57	0.00111	24.50251

P43652	0	0.9241	707.8599	1414.712	2.26	0.0016	6.334191
P43652	0	1	764.8453	1528.683	0.94	0.00072	3.37145
P43652	0	1	510.233	1528.684	1.71	0.00087	7.725793
P43652	0	1	486.9037	1458.696	0.98	0.00048	5.635805
P43652	0	1	486.9036	1458.696	0.92	0.00045	27.7282
P43652	0	1	534.9382	1602.8	1.82	0.00097	8.778147
P43652	0	1	534.9387	1602.802	2.85	0.00152	8.367504
P43652	0	0.8222	607.0431	1819.115	3.48	0.00211	17.13243
P43652	0	0.9091	867.9698	1734.932	1.6	0.00138	9.411367
P43652	0	1	637.6407	1910.908	1.62	0.00103	15.03099
P43652	0	1	578.9822	1734.932	1.41	0.00082	4.940291
P43652	0	1	955.9577	1910.908	1.95	0.00186	8.962317
P43652	0	0.9623	578.983	1734.935	2.89	0.00167	3.139548
P43652	0	0.9873	692.7949	1384.582	1.93	0.00134	14.58209
P43652	0	0.9455	692.7944	1384.581	1.23	0.00085	0
P43652	0	0.9057	640.3427	1279.678	2.58	0.00165	16.52666
P43652	0	1	661.3623	1321.717	2.8	0.00185	13.67644
P43652	0	0.8793	692.7947	1384.582	1.67	0.00115	3.215612
P43652	1	0.75	530.6485	1589.931	4.12	0.00219	46.61637
P43652	0	1	534.9378	1602.799	1.14	0.00061	0
P43652	0	1	486.9038	1458.697	1.23	0.0006	55.34235
P43652	0	0.9412	478.9287	1434.772	2.01	0.00096	27.00776
P43652	0	1	534.9371	1602.797	-0.12	-0.00006	9.675637
P43652	1	0.3125	479.2641	1435.778	1.33	0.00064	14.21353
P43652	0	0.5893	526.795	1052.583	0.09	0.00004	0
P43652	0	1	640.3427	1279.678	2.58	0.00165	24.31348
P43652	0	0.7	395.9058	1185.703	1.1	0.00044	20.36769
P43652	0	0.8163	560.8719	1120.737	2.92	0.00164	38.18472
P43652	0	1	426.2298	1276.675	0.34	0.00014	13.81138
P43652	0	0.7541	560.8721	1120.737	3.35	0.00188	6.374237
P43652	0	1	431.5622	1292.672	2.17	0.00093	14.10278
P43652	0	0.9444	442.2493	1324.733	3.61	0.0016	23.24922
P43652	0	0.963	559.8088	1118.61	1.66	0.00093	5.597795
P43652	0	0.8519	441.2442	1321.718	3.28	0.00144	15.65447
P43652	0	0.6333	426.2297	1276.675	0.12	0.00005	17.67797
P43652	1	0.9706	482.6133	1445.825	2.05	0.00099	43.69828
P43652	0	0.8947	707.8584	1414.71	0.19	0.00013	0
P43652	0	1	566.3311	1131.655	3.24	0.00183	5.560405
P43652	0	1	377.8893	1131.653	1.85	0.0007	3.336759
P43652	0	0.75	395.9059	1185.703	1.33	0.00053	29.40627
P43652	0	0.1739	395.9059	1185.703	1.18	0.00047	34.90573
P43652	0	0.6333	426.2311	1276.679	3.49	0.00149	4.011516
P43652	0	0.439	526.7961	1052.585	2.29	0.0012	0
P43652	0	0.7907	448.2709	1342.798	3.58	0.0016	4.487895
P43652	0	0.6111	660.9116	1320.816	2.3	0.00152	14.65388
P43652	0	0.9355	448.2701	1342.796	1.94	0.00087	0
P43652	0	0.8947	646.8407	1292.674	3.71	0.0024	7.852165
P43652	0	0.9524	635.8088	1270.61	2.43	0.00154	43.07589
P43652	0	0.5854	593.356	1185.705	2.66	0.00157	0
P43652	0	1	377.8898	1131.655	3.07	0.00116	26.66956
P43652	0	0.6111	534.9388	1602.802	3.08	0.00164	26.06594
P43652	0	1	377.8894	1131.654	2.1	0.00079	21.89651

P43652	0	0.9211	472.2422	1414.712	1.95	0.00092	8.636809
P43652	0	1	605.8128	1210.618	1.74	0.00105	11.60806
P43652	0	1	566.331	1131.655	3.02	0.00171	16.48912
P43652	0	1	478.614	1433.827	2.94	0.0014	32.40703
P43652	0	1	1006.116	3016.334	0.15	0.00015	0
P43652	0	0.9787	494.2794	987.5514	2.24	0.00111	0
P43652	0	0.88	637.6412	1910.909	2.39	0.00152	11.10922
P43652	0	0.6	442.2481	1324.73	0.78	0.00034	6.327705
P43652	0	0.4615	526.7959	1052.585	1.82	0.00096	7.326452
P43652	0	1	378.1959	1132.573	0.61	0.00023	35.69987
P43652	0	0.4359	526.7966	1052.586	3.22	0.00169	0
P43652	0	0.9375	589.3093	1177.611	-0.16	-0.0001	0
P43652	0	0.5263	440.9429	1320.814	0.99	0.00044	39.05444
P43652	0	0.8409	561.8089	1122.611	3.4	0.00191	10.14421
P43652	0	1	431.5616	1292.67	0.61	0.00026	28.0056
P43652	0	0.4483	526.7957	1052.584	1.48	0.00078	42.59482
P43652	0	1	494.279	987.5507	1.44	0.00071	6.296525
P43652	0	0.9189	635.8091	1270.611	3	0.00191	0
P43652	0	1	486.9034	1458.696	0.42	0.0002	79.00513
P43652	0	1	427.2311	1279.679	3.09	0.00132	27.60504
P43652	0	0.8718	561.8086	1122.61	2.85	0.0016	25.70295
P43652	0	1	431.562	1292.671	1.6	0.00069	74.15913
P43652	0	0.8462	427.2297	1279.675	-0.06	-0.00002	0
P43652	0	1	404.2108	1210.618	1.3	0.00053	17.00945
P43652	0	0.9375	637.6412	1910.909	2.49	0.00158	0
P43652	1	1	530.6478	1589.929	2.86	0.00151	45.22499
P43652	0	0.2941	377.8898	1131.655	3.07	0.00116	36.78856
P43652	0	1	494.2795	987.5516	2.43	0.0012	6.577944
P43652	0	0.4737	472.2421	1414.712	1.69	0.0008	48.90598
P43652	1	1	482.6135	1445.826	2.43	0.00117	37.36449
P43652	1	0.7143	479.2654	1435.782	4.01	0.00192	38.4207
P43652	1	0.9429	795.4673	1589.927	1.83	0.00145	0
P01042	0	0.9149	770.4023	1539.797	2.66	0.00205	6.681793
P01042	0	0.7846	513.9367	1539.796	1.46	0.00075	7.929009
P01042	0	1	770.4014	1539.795	1.39	0.00107	5.82401
P01042	0	0.9863	698.3505	1395.694	1.72	0.0012	5.75931
P01042	0	0.9853	698.3506	1395.694	1.89	0.00132	18.80093
P01042	0	0.766	402.2293	1204.673	2.04	0.00082	22.50484
P01042	0	0.6316	488.3074	975.6075	-0.17	-0.00008	27.3522
P01042	1	1	764.3976	2291.178	0.48	0.00037	13.80616
P01042	0	0.7442	402.2293	1204.673	2.19	0.00088	4.146805
P01042	0	0.7818	402.2287	1204.672	0.67	0.00027	5.28186
P01042	0	1	764.3867	1527.766	1.82	0.00139	2.835009
P01042	0	0.9583	764.3848	1527.762	-0.73	-0.00056	0
P01042	1	1	487.2952	1459.871	7.2	0.00351	23.69765
P01042	0	1	1009.996	2018.985	2.22	0.00224	3.021011
P01042	0	1	809.7669	2427.286	0.12	0.0001	4.31959
P01042	0	1	607.5783	2427.291	2.25	0.00137	4.088181
P01042	0	0.9692	809.7702	2427.296	4.27	0.00345	10.51965
P01042	0	1	607.5779	2427.29	1.65	0.001	4.421775
P01042	0	0.8387	809.7686	2427.291	2.31	0.00187	23.68766
P01042	0	0.978	809.7675	2427.288	0.95	0.00077	4.202127

P01042	0	0.9074	607.5794	2427.296	4.16	0.00253	0
P01042	0	1	761.734	2283.187	1.68	0.00128	2.382827
P01042	1	1	482.9506	1446.837	1.78	0.00086	10.22811
P01042	1	1	482.9506	1446.837	1.78	0.00086	8.97442
P01042	1	1	764.3993	2291.183	2.72	0.00208	0
P01042	1	1	764.3971	2291.177	-0.16	-0.00012	7.040606
P01042	0	1	673.6658	2018.983	1.43	0.00096	2.435777
P01042	0	1	673.6661	2018.984	1.79	0.0012	4.742405
P01042	0	0.6111	488.3085	975.6097	2.02	0.00099	5.461788
P01042	0	0.5789	494.8246	988.642	2.66	0.00131	50.31135
P01042	0	1	383.229	1147.673	1.87	0.00072	0
P01042	0	0.8971	383.229	1147.672	1.71	0.00065	2.838167
P01042	0	0.78	402.2294	1204.674	2.42	0.00097	14.09317
P01042	0	0.7818	402.2288	1204.672	0.97	0.00039	5.102438
P01042	0	0.8125	402.2298	1204.675	3.33	0.00134	3.405872
P01042	0	0.7963	402.2291	1204.673	1.73	0.0007	8.370627
P01042	0	0.8889	472.7434	1887.952	0.33	0.00016	5.556575
P01042	0	1	944.4809	1887.955	1.71	0.00161	6.019237
P01042	0	0.8594	629.9899	1887.955	1.99	0.00125	100
P01042	1	1	537.2917	1609.861	2.18	0.00117	21.31787
P01042	0	1	629.9895	1887.954	1.41	0.00089	100
P01042	0	0.7846	513.9374	1539.798	2.77	0.00142	9.036447
P01042	0	0.9318	770.4013	1539.795	1.31	0.00101	6.453742
P01042	0	0.9577	629.9898	1887.955	1.8	0.00113	9.478793
P01042	0	0.9697	799.0803	2395.226	2.35	0.00188	4.861084
P01042	0	0.963	799.0794	2395.224	1.28	0.00102	3.932043
P01042	0	0.9873	799.0799	2395.225	1.89	0.00151	3.761647
P01042	0	1	799.0787	2395.222	0.44	0.00035	100
P01042	0	1	1225.861	4900.423	3.04	0.00373	0
P01042	1	0.9333	631.586	2523.322	2.62	0.00165	0
P01042	1	0.9333	631.586	2523.322	2.62	0.00165	0
P01042	1	1	667.611	2667.422	1.74	0.00116	4.754493
P01042	1	0.9672	889.8123	2667.422	1.72	0.00153	5.378718
P01042	0	0.9857	836.437	1671.867	0.78	0.00065	6.189695
P01042	0	0.7931	557.9601	1671.866	0.15	0.00009	8.509763
P01042	0	1	836.4376	1671.868	1.51	0.00126	17.89953
P01042	1	0.96	632.1061	2525.403	0.38	0.00024	9.84887
P01042	1	0.8043	624.4069	1247.807	1.48	0.00092	8.60861
P01042	0	0.95	727.3826	1453.758	1.36	0.00099	5.150754
P01042	0	1	485.2575	1453.758	1.3	0.00063	53.24663
P01042	0	1	578.2933	2310.151	2.06	0.00119	0
P01042	0	1	485.2581	1453.76	2.69	0.0013	0
P01042	0	0.9231	485.2576	1453.758	1.68	0.00082	11.50979
P01042	0	0.9432	727.3829	1453.758	1.78	0.00129	7.38668
P01042	0	1	727.3818	1453.756	0.35	0.00025	5.586978
P01042	0	0.9062	485.2566	1453.755	-0.46	-0.00022	8.363006
P01042	0	1	462.8356	2310.149	1.1	0.00051	3.127691
P01042	0	0.7792	751.7402	2253.206	0.62	0.00047	26.10412
P01042	0	1	485.2579	1453.759	2.19	0.00106	39.89224
P01042	0	0.8684	407.2364	1219.695	1.75	0.00071	9.865144
P01042	0	1	541.6285	1622.871	2.91	0.00157	65.89758
P01042	0	1	922.4299	1843.852	2.99	0.00276	11.224

P01042	0	0.8551	655.332	1309.657	2.17	0.00142	7.84863
P01042	1	1	546.6893	2729.418	2.05	0.00112	15.58186
P01042	1	0.8571	416.6068	1247.806	0.93	0.00039	15.65512
P01042	0	0.8222	574.3392	1147.671	0.69	0.00039	3.194234
P01042	0	0.5789	494.824	988.6407	1.42	0.0007	28.28269
P01042	0	0.8333	574.3388	1147.67	-0.06	-0.00003	8.693646
P01042	0	0.8049	727.3818	1453.756	0.26	0.00019	0
P01042	0	1	709.3376	1417.668	1.33	0.00094	6.861341
P01042	0	0.7321	655.3306	1309.654	0.02	0.00001	0
P01042	0	0.8205	574.3404	1147.674	2.71	0.00155	3.178127
P01042	0	0.1471	751.0474	2251.128	4	0.003	8.722005
P01042	0	0.5789	494.824	988.6408	1.48	0.00073	13.36663
P01042	0	0.807	494.824	988.6407	1.36	0.00067	10.86702
P01042	0	0.8537	407.2368	1219.696	2.72	0.00111	9.465793
P01042	1	0.8333	487.2931	1459.865	2.75	0.00134	7.314584
P01042	0	1	629.9915	1887.96	4.51	0.00284	0
P01042	0	0.878	407.2361	1219.694	1	0.0004	9.054293
P01042	0	0.96	502.2886	1003.57	1.65	0.00083	15.08672
P01042	0	1	637.2874	1273.567	2.73	0.00174	0
P01042	0	0.8889	509.9267	1527.766	1.41	0.00072	10.12046
P01042	0	0.8333	574.3391	1147.671	0.47	0.00027	6.427654
P01042	0	1	473.2275	1417.668	1.32	0.00062	11.46027
P01042	0	0.5789	494.8244	988.6415	2.16	0.00107	18.16507
P01042	0	0.8519	407.2372	1219.697	3.7	0.0015	72.38647
P01042	1	0.9231	667.6115	2667.424	2.47	0.00165	14.86222
P01042	0	0.8333	799.0799	2395.225	1.89	0.00151	7.923137
P01042	1	0.7632	630.924	1260.841	3.39	0.00214	0
P01042	1	0.973	842.4697	2525.395	-2.83	-0.00238	34.30891
P01042	0	0.8649	509.927	1527.766	2.01	0.00103	0
P01042	0	0.7857	530.7886	1060.57	1.24	0.00066	0
P01042	0	0.5102	494.8245	988.6417	2.35	0.00116	63.07803
P01042	0	0.8966	557.9613	1671.869	2.34	0.00131	10.70925
P01042	0	0.2941	513.9371	1539.797	2.29	0.00118	15.62038
P01042	0	0.8214	407.2362	1219.694	1.22	0.0005	20.23017
P01042	0	0.875	1127.106	2253.206	0.38	0.00043	52.58852
P01042	0	1	402.2296	1204.674	3.02	0.00121	44.0244
P01042	0	1	673.6665	2018.985	2.43	0.00163	32.2427
P01042	0	0.9412	515.7723	1030.537	1.71	0.00088	0
P01042	1	1	482.9507	1446.838	1.97	0.00095	42.10269
P01042	0	0.84	557.9616	1671.87	2.89	0.00161	3.240114
P01042	1	0.7667	451.5751	1352.711	3.39	0.00153	9.794243
P01042	0	0.913	610.3519	1219.696	3.26	0.00199	6.050193
P01042	0	0.6383	494.8243	988.6413	1.98	0.00098	15.96551
P01042	0	1	629.9897	1887.955	1.7	0.00107	62.70786
P01042	0	0.5588	599.5635	2395.232	4.9	0.00293	5.377209
P01042	0	0.7097	513.936	1539.793	0.03	0.00002	0
P01042	0	0.9706	764.3868	1527.766	1.9	0.00145	35.20038
P01042	0	0.5833	799.0793	2395.223	1.13	0.0009	14.97971
P01042	0	0.8333	610.3518	1219.696	3.06	0.00187	15.85079
P01042	0	0.8293	587.8223	1174.637	-0.28	-0.00016	3.218603
P01042	0	0.9259	629.9885	1887.951	-0.24	-0.00015	17.76644
P01042	0	1	513.9365	1539.795	1.1	0.00057	32.67638

P01042	0	0.94	502.2883	1003.569	0.98	0.00049	0
P01042	0	0.8788	610.3516	1219.696	2.76	0.00168	9.72617
P01042	0	0.9444	629.9902	1887.956	2.57	0.00162	43.00935
P01042	0	0.9143	465.9028	1395.694	1.79	0.00083	9.618615
P01042	0	0.9429	396.5607	1187.668	3.41	0.00135	15.20208
P01042	1	0.9512	434.917	1302.736	2.88	0.00125	0
P01042	1	0.9512	434.917	1302.736	2.88	0.00125	0
P01042	0	0.9024	698.348	1395.689	-1.78	-0.00124	0
P01042	0	0.9062	392.2186	1174.641	2.93	0.00115	34.80474
P01042	0	0.6667	488.3095	975.6118	4.21	0.00206	14.78632
P01042	0	0.8913	610.3514	1219.696	2.56	0.00156	6.124978
P01042	0	1	610.3518	1219.696	3.06	0.00187	6.901563
P01042	0	0.6429	488.3085	975.6098	2.15	0.00105	5.037911
P01042	1	0.8293	434.9167	1302.736	2.25	0.00098	14.38482
P01042	1	0.8293	434.9167	1302.736	2.25	0.00098	14.38482
P01042	0	1	637.2868	1273.566	1.87	0.00119	9.049982
P01042	0	1	637.2873	1273.567	2.64	0.00168	13.94588
P01042	0	1	407.2365	1219.695	2.05	0.00083	46.67579
P01042	0	0.5357	757.072	2269.202	0.81	0.00062	12.88046
P01042	0	0.625	629.9914	1887.96	4.41	0.00278	20.89967
P01042	0	0.2963	513.9377	1539.799	3.36	0.00173	4.76165
P01042	0	0.8824	513.9366	1539.795	1.22	0.00063	22.72678
P01042	0	1	637.2873	1273.567	2.64	0.00168	3.627483
P01042	0	0.9444	757.0721	2269.202	0.98	0.00074	10.94758
P01042	0	0.6316	488.308	975.6088	1.09	0.00053	45.02955
P01042	0	1	709.3372	1417.667	0.81	0.00057	11.23495
P01042	0	0.675	494.824	988.6408	1.48	0.00073	7.152709
P01042	0	0.871	465.9024	1395.693	1.07	0.0005	7.832078
P01042	0	0.963	698.3494	1395.691	0.14	0.0001	28.54879
P01042	0	0.675	494.8242	988.641	1.73	0.00086	9.10708
P01042	1	1	573.5526	2291.189	5.03	0.00288	10.79374
P01042	0	1	637.2882	1273.569	3.98	0.00253	0
P01042	1	0.9333	667.612	2667.426	3.21	0.00214	34.76399
P06396	0	0.7692	1059.589	2118.171	1.28	0.00135	4.321578
P06396	0	1	706.7284	2118.171	1.01	0.00072	4.852993
P06396	0	0.8333	536.639	1607.902	1.78	0.00095	13.88729
P06396	0	0.9417	804.4551	1607.903	2.09	0.00168	7.370104
P06396	0	1	536.6391	1607.903	2.01	0.00108	21.10782
P06396	0	0.8148	564.3121	1127.617	4.21	0.00237	28.22671
P06396	0	1	854.1033	2560.295	1.28	0.00109	3.452702
P06396	0	0.9143	640.8293	2560.295	1.22	0.00078	0
P06396	0	1	854.1033	2560.295	1.28	0.00109	3.858436
P06396	0	1	640.8297	2560.297	1.8	0.00115	4.219265
P06396	1	1	428.2664	1282.785	0.23	0.0001	23.32552
P06396	0	1	854.1054	2560.301	3.64	0.0031	7.177437
P06396	0	1	854.1033	2560.295	1.28	0.00109	4.593058
P06396	0	1	640.8298	2560.297	1.99	0.00127	3.928206
P06396	0	1	854.1024	2560.293	0.2	0.00017	4.518157
P06396	0	1	1280.651	2560.295	1	0.00127	0.796913
P06396	0	0.9286	854.1049	2560.3	3.06	0.00261	0
P06396	0	0.9333	806.0695	2416.194	1.61	0.0013	1.268101
P06396	1	1	428.2679	1282.789	3.73	0.00159	18.03263

P06396	1	1	747.6265	2987.484	3.77	0.00281	37.11326
P06396	1	1	747.6273	2987.487	4.91	0.00367	7.945455
P06396	1	1	514.9722	1542.902	1.62	0.00083	9.065966
P06396	0	0.8955	473.9451	1419.821	1.39	0.00066	3.623176
P06396	0	0.9706	671.0206	2011.047	2.82	0.00189	13.79376
P06396	0	0.9825	671.0201	2011.046	2.09	0.0014	18.12832
P06396	0	0.9804	671.0207	2011.048	3	0.00201	10.81399
P06396	0	0.9375	709.3729	2126.104	1.53	0.00108	2.264488
P06396	0	0.8947	709.3728	2126.104	1.44	0.00102	7.818883
P06396	0	1	1063.557	2126.107	2.79	0.00297	7.132674
P06396	0	0.9412	709.3746	2126.109	3.94	0.00279	22.67377
P06396	0	0.9706	709.3735	2126.106	2.39	0.00169	10.92132
P06396	0	0.7447	709.3742	2126.108	3.42	0.00242	27.62198
P06396	0	0.9762	844.4219	2531.251	0.65	0.00055	3.692496
P06396	0	0.9792	671.0206	2011.047	2.91	0.00195	34.79568
P06396	0	1	844.4226	2531.253	1.45	0.00122	3.598106
P06396	0	1	844.4224	2531.253	1.23	0.00104	0
P06396	1	1	460.2446	1378.719	-0.28	-0.00013	0
P06396	1	1	413.9016	1239.69	2.7	0.00112	12.9063
P06396	0	0.7586	554.8276	1108.648	2.41	0.00134	21.00542
P06396	0	0.6923	536.9369	1608.796	1.93	0.00104	12.38954
P06396	0	0.8077	536.9368	1608.796	1.71	0.00092	35.3954
P06396	1	0.9815	700.0353	2098.091	1.78	0.00124	10.35054
P06396	0	1	488.9026	1464.693	1.55	0.00076	34.82188
P06396	0	0.9718	513.784	1026.561	1.88	0.00096	2.849683
P06396	0	1	998.2035	2992.596	0.3	0.0003	9.271144
P06396	0	1	473.9456	1419.822	2.3	0.00109	15.1963
P06396	0	0.9254	572.3044	1143.602	2.55	0.00146	5.04193
P06396	0	1	611.813	1222.619	0.22	0.00014	100
P06396	0	0.881	609.8143	1218.621	1.46	0.00089	4.664033
P06396	0	1	585.8348	1170.662	1.33	0.00078	14.24858
P06396	0	1	585.8355	1170.664	2.47	0.00145	19.3546
P06396	0	0.8286	640.8324	2560.308	6.09	0.0039	0
P06396	0	0.9444	1280.651	2560.295	1.28	0.00164	2.534681
P06396	0	0.5143	444.2516	887.4959	1.57	0.0007	4.244547
P06396	0	0.75	536.9388	1608.802	5.58	0.00299	13.70866
P06396	0	0.8571	505.798	1010.589	1.43	0.00072	9.834462
P06396	0	0.9032	640.8305	2560.3	3.13	0.002	0
P06396	0	0.9231	671.0201	2011.046	2.18	0.00146	44.88815
P06396	0	1	488.9019	1464.691	0.17	0.00009	29.6614
P06396	0	0.9375	408.2125	1222.623	3.68	0.0015	0
P06396	0	1	473.9454	1419.822	2.04	0.00096	31.01327
P06396	1	0.7368	525.2772	2098.087	-0.35	-0.00019	27.12704
P06396	0	0.5946	576.7869	1152.566	3.64	0.0021	3.903802
P06396	1	1	641.897	1282.787	1.93	0.00124	7.035858
P06396	0	0.775	482.7756	964.544	0.89	0.00043	0
P06396	1	0.7391	401.2678	1201.789	1.47	0.00059	9.425853
P06396	0	0.8605	523.3475	1045.688	1.65	0.00086	0
P06396	0	0.65	536.9367	1608.796	1.59	0.00085	58.99189
P06396	0	0.9032	609.8156	1218.624	3.56	0.00217	39.98104
P06396	1	1	436.2864	1306.845	2.1	0.00092	54.4599
P06396	0	0.5581	516.3026	1031.598	1.22	0.00063	2.898581

P06396	0	0.9767	571.8259	1142.645	0.63	0.00036	3.070138
P06396	0	0.9667	609.816	1218.625	4.36	0.00266	0
P06396	0	0.7209	523.3474	1045.687	1.42	0.00074	100
P06396	1	1	386.4808	1542.902	1.33	0.00051	14.56154
P06396	0	0.8919	564.3115	1127.616	3.12	0.00176	6.131098
P06396	0	1	484.249	967.4907	0.74	0.00036	2.149233
P06396	0	1	585.8364	1170.666	4.04	0.00236	22.41522
P06396	0	0.7027	381.5539	1142.647	2.87	0.00109	3.404617
P06396	0	0.75	522.3395	1043.672	1.44	0.00075	26.34965
P06396	0	0.8	482.7758	964.5442	1.14	0.00055	24.3253
P06396	0	0.8125	854.1025	2560.293	0.35	0.0003	5.999435
P06396	0	0.8857	523.3477	1045.688	2.12	0.00111	0
P06396	0	0.5	576.7859	1152.565	1.95	0.00112	0
P02647	0	1	844.9425	1688.878	2.59	0.00219	0
P02647	0	0.9583	844.9418	1688.876	1.72	0.00145	6.633591
P02647	0	0.9571	563.6304	1688.877	2	0.00113	9.600135
P02647	2	1	470.451	2348.226	1.72	0.00081	2.884918
P02647	1	0.7872	567.2975	1699.878	3.72	0.00211	3.558731
P02647	1	0.878	567.2968	1699.876	2.53	0.00143	7.240958
P02647	1	0.8409	567.297	1699.876	2.85	0.00162	4.783349
P02647	1	1	516.7697	2064.057	-2.46	-0.00127	13.59094
P02647	0	1	699.6626	2096.973	1.78	0.00125	1.946368
P02647	0	0.9464	699.6629	2096.974	2.22	0.00155	11.52986
P02647	0	0.9455	699.6625	2096.973	1.7	0.00119	2.609882
P02647	0	0.7045	563.6316	1688.88	4.06	0.00229	35.56144
P02647	0	0.9444	699.6629	2096.974	2.22	0.00155	8.594824
P02647	0	0.8511	699.6625	2096.973	1.7	0.00119	10.36652
P02647	0	0.9804	699.6631	2096.975	2.48	0.00174	8.636162
P02647	1	1	688.6932	2064.065	1.48	0.00102	5.538224
P02647	0	0.9559	699.6631	2096.975	2.48	0.00174	9.949135
P02647	0	0.9167	770.9185	1540.83	3.04	0.00234	16.92712
P02647	0	0.9184	514.2811	1540.829	2.4	0.00123	8.841324
P02647	0	0.9219	770.9183	1540.829	2.88	0.00222	5.868382
P02647	0	1	651.6285	1952.871	1.86	0.00121	20.37416
P02647	0	1	482.5895	1445.754	2.4	0.00116	12.40576
P02647	0	1	501.9474	1503.828	1.28	0.00064	34.04776
P02647	0	1	482.5893	1445.753	1.89	0.00091	0
P02647	0	1	723.3799	1445.753	1.46	0.00105	0
P02647	0	0.8387	434.2666	1300.785	2.03	0.00088	15.9908
P02647	2		467.7679	1868.05	1.5	0.0007	5.348342
P02647	2	0.0833	623.3552	1868.051	2.1	0.00131	3.255819
P02647	0	0.9787	434.555	1301.65	1.45	0.00063	15.69891
P02647	0	0.7925	690.4002	1379.793	2.1	0.00145	13.61261
P02647	0	0.8824	741.0517	2221.141	1.18	0.00088	6.397406
P02647	0	0.75	508.6362	1523.894	1.08	0.00055	2.333223
P02647	0	0.9302	762.4508	1523.894	1.33	0.00101	8.999641
P02647	0	0.806	508.6362	1523.894	1.08	0.00055	8.609177
P02647	0	1	762.4506	1523.894	1.09	0.00083	2.488898
P02647	0	0.9529	762.4507	1523.894	1.17	0.00089	3.445343
P02647	0	0.8676	741.0527	2221.144	2.58	0.00191	7.858632
P02647	0	0.9634	741.0523	2221.142	2.01	0.00149	0
P02647	0	0.9231	741.0522	2221.142	1.84	0.00136	10.81566

P02647	0	0.913	741.0504	2221.137	-0.55	-0.00041	0
P02647	0	0.9677	741.0513	2221.139	0.69	0.00051	8.675595
P02647	0	1	693.0176	2077.038	1.12	0.00078	1.507469
P02647	0	1	693.0179	2077.039	1.57	0.00108	27.82154
P02647	0	1	741.0518	2221.141	1.35	0.001	4.535439
P02647	0	0.8923	556.0413	2221.143	2.39	0.00133	8.563459
P02647	0	1	1111.075	2221.142	1.9	0.00211	0.702934
P02647	0	1	741.051	2221.138	0.19	0.00014	4.111861
P02647	0	1	556.0408	2221.142	1.62	0.0009	7.394027
P02647	0	1	1111.074	2221.14	1.13	0.00126	0.876546
P02647	0	1	741.0508	2221.138	-0.05	-0.00004	4.415444
P02647	0	1	556.0415	2221.144	2.83	0.00157	10.06732
P02647	0	1	1111.074	2221.141	1.24	0.00138	0
P02647	0	1	741.0507	2221.137	-0.22	-0.00016	4.368874
P02647	0	0.9167	741.0525	2221.143	2.25	0.00167	0
P02647	0	1	741.052	2221.141	1.6	0.00118	3.769292
P02647	0	0.9903	1039.023	2077.04	1.81	0.00188	0
P02647	0	0.9571	693.0172	2077.037	0.51	0.00035	2.379014
P02647	0	0.931	741.0532	2221.145	3.24	0.0024	13.2833
P02647	0	0.9565	741.0516	2221.14	1.1	0.00082	10.02585
P02647	0	1	741.0511	2221.139	0.36	0.00027	12.5403
P02647	0	1	741.0516	2221.14	1.1	0.00082	4.420992
P02647	0	0.8654	1111.075	2221.143	2.45	0.00272	0.703947
P02647	0	1	741.0517	2221.141	1.18	0.00088	4.647928
P02647	0	1	1111.075	2221.142	1.9	0.00211	0.23766
P02647	0	1	741.0509	2221.138	0.11	0.00008	4.506809
P02647	0	0.9861	1111.074	2221.142	1.68	0.00187	0
P02647	0	1	741.0508	2221.138	-0.05	-0.00004	4.306448
P02647	0	0.9322	556.0411	2221.142	2.06	0.00114	3.809138
P02647	0	0.9859	1111.074	2221.141	1.24	0.00138	6.381742
P02647	0	1	741.0508	2221.138	0.03	0.00002	4.472652
P02647	0	0.9892	1111.074	2221.14	0.91	0.00102	0.150761
P02647	0	1	741.0507	2221.137	-0.22	-0.00016	4.293705
P02647	0	0.8723	556.0411	2221.142	2.06	0.00114	3.845432
P02647	0	1	1111.074	2221.14	1.13	0.00126	0
P02647	0	1	741.0507	2221.138	-0.14	-0.0001	4.242296
P02647	0	1	741.052	2221.141	1.6	0.00118	6.544406
P02647	0	0.8889	741.0518	2221.141	1.35	0.001	13.04666
P02647	0	0.8788	741.0507	2221.137	-0.22	-0.00016	12.97937
P02647	0	1	741.0523	2221.142	1.93	0.00143	12.5852
P02647	0	1	563.6307	1688.877	2.44	0.00137	34.51334
P02647	0	0.9783	844.9421	1688.877	2.15	0.00182	7.286695
P02647	0	0.9362	563.6306	1688.877	2.22	0.00125	8.778948
P02647	0	1	844.941	1688.875	0.78	0.00066	6.123175
P02647	0	0.9744	563.6294	1688.874	0.16	0.00009	8.293533
P02647	0	1	844.9402	1688.873	-0.16	-0.00013	6.543588
P02647	0	0.9747	563.6293	1688.873	-0.06	-0.00003	7.92499
P02647	0	0.96	563.6317	1688.881	4.28	0.00241	20.01157
P02647	0	0.9434	563.6317	1688.881	4.28	0.00241	38.87373
P02647	0	0.7907	714.3414	1427.675	0.63	0.00045	4.203743
P02647	0	0.6727	476.5633	1427.675	0.6	0.00029	2.480297
P02647	0	0.9697	772.8882	1544.769	-1.35	-0.00104	0

P02647	0	0.9296	563.6308	1688.878	2.65	0.00149	18.27896
P02647	0	0.9778	844.9417	1688.876	1.58	0.00133	6.48517
P02647	0	0.9615	515.5961	1544.774	1.64	0.00085	4.429919
P02647	0	1	772.8904	1544.774	1.49	0.00115	2.761238
P02647	0	0.9483	741.0526	2221.143	2.42	0.00179	4.888702
P02647	0	0.9048	741.0505	2221.137	-0.38	-0.00028	0
P02647	0	0.62	560.3238	1119.64	0.22	0.00013	0
P02647	0	0.9722	693.0178	2077.039	1.48	0.00102	22.79571
P02647	0	0.9737	434.2667	1300.786	2.18	0.00094	12.08527
P02647	0	0.9655	434.267	1300.787	2.95	0.00128	16.34788
P02647	0	0.7778	434.267	1300.786	2.88	0.00125	30.12613
P02647	0	1	501.9486	1503.831	3.72	0.00186	0
P02647	0	0.8033	558.979	1674.922	1.92	0.00107	7.644935
P02647	0	0.9318	837.9647	1674.922	1.67	0.0014	5.781969
P02647	0	0.8065	434.2658	1300.783	0.13	0.00006	17.6015
P02647	0	1	434.2669	1300.786	2.67	0.00116	14.19855
P02647	0	0.7059	509.2768	1017.546	1.75	0.00089	2.482932
P02647	0	0.9756	765.9125	1530.818	0.4	0.00031	15.2144
P02647	0	1	482.5882	1445.75	-0.32	-0.00016	54.21256
P02647	0	0.8448	434.5554	1301.652	2.37	0.00103	4.590828
P02647	0	0.7719	586.301	1756.888	0.54	0.00032	3.970651
P02647	0	0.9386	878.9481	1756.889	0.83	0.00073	3.874524
P02647	0	0.8	762.4517	1523.896	2.53	0.00193	13.38053
P02647	0	0.7708	508.6368	1523.896	2.28	0.00116	2.558283
P02647	0	1	950.9997	1900.992	1.4	0.00133	5.849488
P02647	0	1	634.3356	1900.992	1.52	0.00096	9.524952
P02647	0	0.9254	634.3364	1900.995	2.68	0.0017	11.91869
P02647	1	0.9231	632.3914	1895.16	1.03	0.00065	14.93789
P02647	1	0.807	948.0842	1895.161	1.81	0.00172	7.26764
P02647	1	0.9787	474.5456	1895.161	1.48	0.0007	11.75959
P02647	1	0.9592	632.3916	1895.16	1.32	0.00084	17.75703
P02647	1	0.9032	632.3916	1895.16	1.32	0.00084	10.92754
P02647	1	0.9737	632.3903	1895.156	-0.71	-0.00045	66.89497
P02647	0	0.8947	634.3367	1900.996	3.25	0.00206	12.36575
P02647	0	0.9444	634.3366	1900.995	3.06	0.00194	6.441699
P02647	0	0.9231	634.3353	1900.991	1.04	0.00066	5.650203
P02647	0	1	501.9472	1503.827	0.98	0.00049	0
P02647	1	1	529.2749	1585.81	1.28	0.00067	8.377966
P02647	1	1	397.208	1585.81	1.15	0.00046	6.601691
P02647	0	0.8621	762.4525	1523.898	3.49	0.00266	38.10745
P02647	0	0.9789	837.9646	1674.922	1.6	0.00134	7.451027
P02647	0	0.8077	558.9781	1674.92	0.28	0.00015	8.65478
P02647	1	0.6061	830.7796	2490.324	0.47	0.00039	20.3045
P02647	0	0.98	837.9641	1674.921	0.94	0.00079	9.894595
P02647	0	0.8305	558.9781	1674.92	0.28	0.00015	8.035325
P02647	1	0.9804	830.781	2490.328	2.16	0.00179	8.870957
P02647	1	1	830.7801	2490.326	1.06	0.00088	4.209022
P02647	0	0.99	837.9655	1674.924	2.69	0.00225	15.72826
P02647	0	0.9074	558.9797	1674.925	3.23	0.0018	8.836884
P02647	0	0.9143	765.9134	1530.82	1.52	0.00116	5.603437
P02647	0	0.94	741.0517	2221.141	1.18	0.00088	9.548424
P02647	0	1	837.9639	1674.92	0.72	0.0006	19.68897

P02647	0	1	837.9639	1674.92	0.72	0.0006	29.99844
P02647	0	0.8657	558.978	1674.919	0.06	0.00003	8.076765
P02647	1	0.8358	584.3574	1751.058	1.16	0.00068	0
P02647	1	0.8358	584.3574	1751.058	1.16	0.00068	0
P02647	0	0.907	741.0521	2221.142	1.68	0.00124	3.771213
P02647	0	0.9231	762.4511	1523.895	1.73	0.00132	4.640319
P02647	0	0.8254	508.6364	1523.895	1.5	0.00076	8.419141
P02647	0	0.9333	762.4496	1523.892	-0.27	-0.00021	6.089044
P02647	0	0.8901	765.9135	1530.82	1.59	0.00122	4.138356
P02647	0	1	642.2914	1283.575	2.33	0.00149	23.06001
P02647	0	0.75	434.5545	1301.649	0.33	0.00014	26.70376
P02647	0	0.8684	434.555	1301.65	1.52	0.00066	16.79145
P02647	0	1	651.329	1301.651	1.82	0.00118	3.764178
P02647	0	0.8889	434.5551	1301.651	1.74	0.00075	1.296639
P02647	0	0.7826	434.555	1301.65	1.52	0.00066	8.923704
P02647	1	0.7442	792.9769	1584.946	1.97	0.00156	0
P02647	1	1	528.9865	1584.945	1.04	0.00055	16.22796
P02647	1	0.9796	632.3931	1895.165	3.74	0.00236	57.83123
P02647	0	0.9531	634.3359	1900.993	2	0.00127	12.15117
P02647	0	0.8636	634.3358	1900.993	1.81	0.00115	29.6317
P02647	0	1	687.9105	1374.814	1.7	0.00117	3.931486
P02647	0	1	501.9476	1503.828	1.65	0.00083	51.26196
P02647	0	0.9322	634.3357	1900.993	1.62	0.00102	15.06355
P02647	0	0.9464	634.3361	1900.994	2.29	0.00145	9.686314
P02647	0	1	634.3347	1900.99	0.08	0.00005	8.407166
P02647	0	0.974	759.9609	1518.914	0.72	0.00055	5.91537
P02647	0	0.9825	634.3348	1900.99	0.17	0.00011	19.31782
P02647	0	0.873	506.9766	1518.915	1.28	0.00065	8.104663
P02647	0	0.9833	476.0035	1900.992	1.38	0.00066	0
P02647	0	1	950.9995	1900.992	1.14	0.00109	5.783334
P02647	0	0.9846	759.9607	1518.914	0.48	0.00037	5.668058
P02647	0	0.98	506.9774	1518.918	2.78	0.00141	8.014697
P02647	0	0.9038	951.0004	1900.993	2.11	0.002	0
P02647	0	0.9846	759.9622	1518.917	2.41	0.00183	6.948265
P02647	0	1	501.9473	1503.827	1.1	0.00055	12.28884
P02647	0	1	501.9473	1503.827	1.1	0.00055	0
P02647	0	1	501.9492	1503.833	4.87	0.00244	16.91023
P02647	0	0.8333	634.3362	1900.994	2.48	0.00157	7.613402
P02647	0	0.9	634.3369	1900.996	3.54	0.00225	16.34534
P02647	1	1	528.9862	1584.944	0.34	0.00018	11.88418
P02647	0	1	501.9475	1503.828	1.4	0.0007	19.35593
P02647	0	0.8378	386.2325	1156.683	1.88	0.00072	20.14937
P02647	0	0.8378	386.2325	1156.683	1.88	0.00072	20.14937
P02647	0	1	482.589	1445.752	1.26	0.00061	7.066054
P02647	0	0.8571	434.5552	1301.651	1.88	0.00081	0
P02647	0	1	482.5887	1445.751	0.63	0.0003	49.77322
P02647	0	0.9796	482.5888	1445.752	0.88	0.00042	17.09196
P02647	0	0.8889	434.5547	1301.649	0.75	0.00033	0.094391
P02647	0	1	434.5547	1301.649	0.75	0.00033	51.20749
P02647	0	0.873	434.555	1301.65	1.45	0.00063	0.484491
P02647	0	1	482.5888	1445.752	0.88	0.00042	48.25791
P02647	0	0.875	434.5549	1301.65	1.31	0.00057	0.396241

P02647	0	1	482.5892	1445.753	1.83	0.00088	17.06632
P02647	0	1	482.5887	1445.752	0.75	0.00036	5.194019
P02647	0	1	482.5883	1445.75	-0.13	-0.00006	13.64258
P02647	0	1	482.589	1445.752	1.32	0.00064	4.406243
P02647	0	1	482.5892	1445.753	1.7	0.00082	10.64865
P02647	0	1	723.3798	1445.752	1.29	0.00093	3.092375
P02647	0	1	482.5884	1445.751	0.06	0.00003	4.649761
P02647	0	1	501.9469	1503.826	0.25	0.00012	15.99891
P02647	0	1	723.3804	1445.753	2.05	0.00148	0
P02647	1	1	567.2967	1699.876	2.31	0.00131	10.16533
P02647	1	1	567.296	1699.873	1.02	0.00058	8.106999
P02647	1	0.8409	567.2969	1699.876	2.64	0.00149	8.174891
P02647	1	0.8611	567.2974	1699.878	3.5	0.00198	7.827622
P02647	0	1	698.8668	1396.726	2.49	0.00174	3.336842
P02647	1	0.8919	567.2979	1699.879	4.47	0.00253	27.3749
P02647	1	1	567.2964	1699.875	1.78	0.00101	7.38408
P02647	1	0.8649	567.2972	1699.877	3.28	0.00186	8.639947
P02647	0	0.9091	588.3158	1175.624	2.76	0.00162	3.744691
P02647	0	0.8649	434.2664	1300.785	1.47	0.00064	14.38031
P02647	0	1	434.2667	1300.785	2.11	0.00091	13.65612
P02647	0	0.96	482.5882	1445.75	-0.39	-0.00019	44.09173
P02647	0	0.9688	434.2658	1300.783	-0.01	0	8.102496
P02647	0	0.8824	434.2658	1300.783	0.06	0.00003	8.281363
P02647	0	1	482.5892	1445.753	1.83	0.00088	5.38298
P02647	0	1	501.948	1503.83	2.56	0.00128	21.80379
P02647	2	1	503.7935	2012.152	1.57	0.00079	14.66811
P02647	0	0.6667	509.2764	1017.546	0.97	0.00049	3.552976
P02647	0	0.8966	434.2674	1300.788	3.72	0.00161	5.999357
P02647	0	0.898	514.2809	1540.828	2.16	0.00111	9.294912
P02647	0	1	770.916	1540.825	-0.21	-0.00016	6.178564
P02647	0	0.9667	434.2668	1300.786	2.32	0.001	21.32402
P02647	0	0.913	514.2806	1540.827	1.45	0.00074	6.598859
P02647	0	0.8864	514.2805	1540.827	1.21	0.00062	8.023529
P02647	0	1	770.9157	1540.824	-0.61	-0.00047	5.799671
P02647	0	1	723.3796	1445.752	0.95	0.00069	3.080201
P02647	0	0.9348	514.2802	1540.826	0.61	0.00032	9.670235
P02647	1	1	537.9701	1611.896	1.39	0.00075	20.0581
P02647	0	0.94	563.6308	1688.878	2.65	0.00149	13.14654
P02647	0	1	482.5885	1445.751	0.37	0.00018	4.284438
P02647	0	1	723.3804	1445.753	2.05	0.00148	3.325203
P02647	0	1	482.5889	1445.752	1.2	0.00058	4.731243
P02647	0	1	501.9477	1503.828	1.83	0.00092	24.31637
P02647	0	1	501.9466	1503.825	-0.36	-0.00018	8.028794
P02647	0	1	752.4171	1503.827	0.84	0.00063	5.547592
P02647	0	1	501.9465	1503.825	-0.48	-0.00024	6.869234
P02647	0	0.7021	509.2765	1017.546	1.21	0.00061	1.791536
P02647	0	0.9512	514.2809	1540.828	2.04	0.00105	29.47437
P02647	0	0.9444	693.0186	2077.041	2.62	0.00182	14.43799
P02647	0	0.913	516.2639	1031.521	1.5	0.00077	1.358717
P02647	0	0.9762	434.5554	1301.652	2.37	0.00103	21.27335
P02647	0	0.96	501.9468	1503.826	0	0	59.16982
P02647	0	1	699.6632	2096.975	2.66	0.00186	64.82291

P02647	0	0.8788	501.9486	1503.831	3.59	0.0018	54.46451
P02647	0	0.9149	772.8893	1544.771	-0.01	-0.00001	0
P02647	1	1	396.9912	1584.943	-0.36	-0.00014	12.45849
P02647	0	0.7895	476.5638	1427.677	1.69	0.0008	37.60398
P02647	0	0.925	563.6304	1688.877	2	0.00113	20.69114
P02647	0	0.78	651.3292	1301.651	2.1	0.00136	0
P02647	0	0.3529	714.3427	1427.678	2.51	0.00179	16.92611
P02647	0	1	434.2651	1300.781	-1.48	-0.00064	36.14016
P02647	0	1	651.3291	1301.651	1.91	0.00124	0
P02647	1	0.9048	519.2628	1555.774	2.81	0.00146	11.01931
P02647	1	0.9048	519.2628	1555.774	2.81	0.00146	11.01931
P02647	0	0.8333	741.0515	2221.14	0.85	0.00063	5.141618
P02647	0	1	482.5891	1445.753	1.45	0.0007	10.18239
P02647	0	0.6557	488.2729	975.5384	0.46	0.00022	0
P02647	0	0.6444	509.2767	1017.546	1.51	0.00077	0
P02647	2	1	587.8123	2348.227	2.3	0.00135	0.550985
P02647	0	0.8636	488.2727	975.5381	0.08	0.00004	4.785724
P02647	0	0.7391	714.3413	1427.675	0.46	0.00033	8.90386
P02647	1	0.8684	572.6286	1715.871	2.77	0.00158	9.342708
P02647	0	0.913	772.8919	1544.777	3.39	0.00262	0
P02647	0	0.8824	588.3145	1175.622	0.58	0.00034	3.178979
P02647	0	0.8378	434.5552	1301.651	2.09	0.00091	39.99739
P02647	0	1	434.5546	1301.649	0.61	0.00026	26.91794
P02647	1	1	537.9702	1611.896	1.62	0.00087	6.36654
P02647	0	1	482.5897	1445.754	2.71	0.00131	54.06726
P02647	0	0.7576	741.0509	2221.138	0.11	0.00008	5.77091
P02647	0	0.7647	509.2763	1017.545	0.73	0.00037	2.806286
P02647	0	0.7576	634.3372	1900.997	3.93	0.00249	31.43967
P02647	0	0.9189	514.2795	1540.824	-0.69	-0.00036	11.11472
P02647	0	0.7021	509.2766	1017.546	1.45	0.00074	28.26963
P02647	0	0.8571	634.3365	1900.995	2.87	0.00182	4.425326
P02647	1	1	552.7991	2208.174	4.66	0.00257	23.11695
P02647	0	0.9286	741.0516	2221.14	1.1	0.00082	11.00991
P02647	0	0.75	714.3409	1427.674	-0.05	-0.00004	3.121211
P02647	0	1	698.8661	1396.725	1.44	0.001	19.86818
P02647	1	0.9167	433.234	1729.914	2.18	0.00094	9.524178
P02647	0	0.7647	509.2764	1017.545	0.91	0.00046	3.059817
P02647	0	0.5926	714.3422	1427.677	1.74	0.00124	31.96611
P02647	0	0.9474	434.2661	1300.784	0.84	0.00036	55.02639
P02647	1	1	528.9883	1584.95	4.5	0.00238	8.280834
P02647	0	0.7297	563.6312	1688.879	3.41	0.00192	23.63972
P02647	0	0.7059	509.2762	1017.545	0.49	0.00025	3.314902
P02647	0	0.7442	509.2765	1017.546	1.09	0.00055	8.107929
P02647	0	0.9756	514.2791	1540.823	-1.41	-0.00072	0
P02647	0	0.7297	508.6368	1523.896	2.34	0.00119	9.470936
P02647	0	0.9474	651.3291	1301.651	1.91	0.00124	12.9716
P02647	0	1	723.3796	1445.752	0.95	0.00069	16.16673
P02647	0	0.963	514.2828	1540.834	5.84	0.003	51.7985
P02647	0	0.7778	509.2769	1017.546	1.87	0.00095	5.294312
P02647	0	1	482.5905	1445.757	4.42	0.00213	59.87727
P02647	0	0.913	588.3153	1175.623	1.93	0.00113	4.639504
P02647	0	0.55	509.2759	1017.544	-0.05	-0.00003	0

P02647	1	0.7778	504.7897	1008.572	2.43	0.00123	27.79346
P02647	0	0.2083	395.5682	1184.69	2.05	0.00081	10.88983
P02647	0	0.7674	772.8929	1544.778	4.66	0.0036	9.914625
P02647	2	0.037	467.7679	1868.05	1.5	0.0007	5.348342
P02647	1	1	433.2335	1729.912	1.19	0.00052	16.82448
P02647	0	0.4286	592.8486	1184.69	1.9	0.00113	4.501509
P02647	0	0.88	741.0529	2221.144	2.83	0.0021	16.25623
P02647	0	0.5	508.6364	1523.895	1.5	0.00076	4.924573
P02647	0	0.88	741.0513	2221.139	0.61	0.00045	15.99097
P02647	0	0.5161	693.0189	2077.042	2.98	0.00206	0
P02647	0	0.675	509.2759	1017.545	0.07	0.00003	0
P02647	0	1	651.3285	1301.65	0.97	0.00063	0.116527
P02647	0	0.8182	634.3353	1900.991	0.94	0.0006	36.89429
P02647	0	0.62	560.3256	1119.644	3.5	0.00196	0
P02647	0	0.9355	434.5542	1301.648	-0.3	-0.00013	0
P02647	0	0.9714	482.589	1445.753	1.39	0.00067	4.37337
P02647	0	1	651.6281	1952.87	1.2	0.00078	4.442463
P02647	0	1	651.3284	1301.65	0.88	0.00057	0
P02647	0	0.3333	714.342	1427.677	1.57	0.00112	22.07319
P02647	1	0.8	482.6171	1445.837	3.66	0.00177	5.811739
P02647	1	1	850.4404	1699.873	1.1	0.00094	6.32115
P02647	0	0.62	560.3231	1119.639	-0.97	-0.00055	0
P02647	1	0.1	480.9522	1440.842	0.62	0.0003	0
P02647	0	1	516.2639	1031.521	1.5	0.00077	15.98257
P02647	0	0.8462	563.6308	1688.878	2.65	0.00149	28.57557
P02647	0	0.4103	560.3253	1119.643	2.95	0.00165	23.7137
P02647	1	1	567.2966	1699.875	2.21	0.00125	25.82204
P02647	0	0.9483	524.2617	1047.516	2.14	0.00112	4.240848
P02647	0	1	650.2884	1299.57	1.61	0.00105	0
P02647	0	0.5172	476.0033	1900.991	0.94	0.00044	0
P02647	0	0.8889	558.9789	1674.922	1.7	0.00095	31.90879
P02647	1	1	482.9216	1446.75	0.84	0.00041	7.203422
P02647	0	0.8	741.0512	2221.139	0.44	0.00033	22.16891
P02647	0	0.8611	434.5548	1301.65	1.03	0.00045	8.633673
P02647	1	0.3871	434.582	1301.731	1.52	0.00066	36.35732
P02647	0	0.9375	741.0525	2221.143	2.25	0.00167	0
P02647	0	1	434.2675	1300.788	4.08	0.00177	5.041448
P02647	0	0.7955	722.3394	1443.671	1.36	0.00098	4.369184
P02647	0	0.8529	556.0423	2221.147	4.25	0.00236	3.510854
P02647	0	0.5714	634.3378	1900.999	4.99	0.00316	31.1857
P02647	0	0.3273	520.7971	1040.587	1.12	0.00058	29.46955
P02647	0	0.8	651.3291	1301.651	1.91	0.00124	0
P02647	0	0.375	395.5681	1184.69	1.66	0.00066	12.4829
P02647	0	0.95	376.7126	1503.828	1.8	0.00068	24.14646
P02647	0	1	482.5894	1445.754	2.21	0.00106	16.78702
P02647	0	0.7222	488.2736	975.5398	1.9	0.00093	0
P02647	0	0.4828	476.5635	1427.676	0.92	0.00044	0
P02647	1	0.8125	567.2976	1699.878	3.82	0.00217	11.03061
P02647	0	0.5806	563.6309	1688.878	2.76	0.00155	24.55579
P02647	0	0.4524	592.8482	1184.689	1.18	0.0007	6.240132
P02647	0	0.8333	1111.074	2221.14	1.13	0.00126	0
P02647	0	0.8056	693.02	2077.045	4.56	0.00316	9.656229

P02647	0	0.6071	563.6296	1688.874	0.59	0.00033	24.9904
P02647	0	0.5	395.5681	1184.69	1.74	0.00069	10.1567
P02647	0	0.9667	770.9175	1540.828	1.85	0.00143	5.913793
P02647	0	0.5	395.5681	1184.69	1.74	0.00069	10.96507
P02647	0	1	482.5887	1445.752	0.69	0.00033	81.23305
P02647	0	0.5405	509.2768	1017.546	1.75	0.00089	4.994334
P02647	0	0.4286	395.5681	1184.69	1.58	0.00063	46.72958
P02647	0	0.3333	395.5683	1184.69	2.2	0.00087	21.51325
P02647	0	0.8125	558.9797	1674.924	3.12	0.00174	56.40453
P02647	0	0.8824	1039.023	2077.039	1.69	0.00175	0
P02647	0	0.7083	634.3356	1900.992	1.42	0.0009	3.769731
P02647	0	0.7188	634.3357	1900.993	1.62	0.00102	20.47313
P02647	0	0.7547	488.2733	975.5394	1.4	0.00068	5.154588
P02647	0	0.65	453.9138	1359.727	2.28	0.00103	7.844263
P02647	0	0.5238	634.3355	1900.992	1.33	0.00084	4.862034
P02647	0	1	687.9103	1374.813	1.52	0.00104	26.27832
P02647	0	1	514.278	1540.819	-3.66	-0.00188	19.47607
P02647	1	0.8056	598.9979	1794.979	4.04	0.00242	61.71779
P02647	0	0.9062	690.3992	1379.791	0.68	0.00047	28.98508
P02647	1	0.6522	572.629	1715.872	3.41	0.00195	30.91411
P02647	0	0.2727	634.3365	1900.995	2.87	0.00182	6.061941
P02647	0	0.2941	395.5678	1184.689	1.04	0.00041	26.10808
P02647	0	1	514.2777	1540.819	-4.14	-0.00213	32.12918
P02647	0	0.7059	509.2764	1017.546	0.97	0.00049	42.57995
P02647	0	0.3056	510.9447	1530.819	1.49	0.00076	0
P02647	0	0.7381	509.277	1017.547	2.17	0.0011	29.90212
P02647	1	1	397.2089	1585.814	3.54	0.0014	6.875036
P02647	0	0.9524	434.2675	1300.788	3.93	0.00171	26.37
P02647	0	0.6346	437.2251	873.4429	0.44	0.00019	9.852448
P02647	0	0.7368	634.3366	1900.995	2.96	0.00188	40.59726
P02647	0	0.6444	509.2762	1017.545	0.55	0.00028	2.88381
P02647	0	0.8611	634.332	1900.981	-4.26	-0.0027	0
P02647	1	1	396.9921	1584.946	1.95	0.00077	48.15558
P02647	0	1	434.5547	1301.649	0.75	0.00033	41.53929
P02647	0	0.875	434.5553	1301.651	2.3	0.001	50.04819
P02647	1	1	482.6156	1445.832	0.5	0.00024	8.608278
P02647	0	0.7778	556.0409	2221.142	1.73	0.00096	5.272471
P02647	0	0.4231	693.0183	2077.04	2.09	0.00145	6.289457
P02647	0	0.92	563.6298	1688.875	0.81	0.00046	18.39999
P02647	0	0.3784	592.8486	1184.69	1.8	0.00107	10.95143
P02647	0	0.875	563.6303	1688.876	1.68	0.00094	26.71309
P02647	0	0.7368	434.2674	1300.788	3.86	0.00168	5.074654
P02647	0	0.5962	437.2254	873.4436	1.28	0.00056	15.28374
P02647	0	0.8621	514.2804	1540.827	1.09	0.00056	12.7544
P02647	0	0.3611	592.8494	1184.691	3.14	0.00186	9.508512
P02647	0	0.3571	592.8489	1184.69	2.32	0.00137	6.619897
P02647	1	0.6429	567.2987	1699.882	5.87	0.00333	0
P02647	0	0.9286	687.911	1374.815	2.41	0.00165	47.39672
P02647	1	0.931	480.9526	1440.843	1.38	0.00066	6.461298
P02647	1	0.931	480.9526	1440.843	1.38	0.00066	6.461298
P02647	0	0.9722	588.3162	1175.625	3.38	0.00199	17.43835
P02647	0	1	514.281	1540.828	2.28	0.00117	28.74172

P02647	0	0.6842	560.3248	1119.642	2.08	0.00116	27.27049
P02647	1	0.675	584.3588	1751.062	3.46	0.00202	11.80095
P02647	1	0.675	584.3588	1751.062	3.46	0.00202	11.80095
P02647	0	0.75	488.2731	975.539	1.02	0.0005	0
P02647	1	0.875	455.2765	1363.815	-0.56	-0.00025	70.0079
P02647	0	0.88	434.5542	1301.648	-0.37	-0.00016	34.28686
P02647	0	0.913	563.6314	1688.88	3.74	0.0021	74.66186
P02647	0	0.7368	506.9764	1518.914	0.74	0.00037	19.22167
P02647	0	0.9677	651.3289	1301.65	1.53	0.001	4.821892
P02647	0	0.9655	770.9158	1540.824	-0.37	-0.00028	0
P02647	0	0.8696	558.9796	1674.924	2.9	0.00162	5.813317
P02647	1	0.6087	580.6597	1739.964	4.26	0.00247	19.83234
P02647	1	0.6087	580.6597	1739.964	4.26	0.00247	19.83234
P02647	0	0.5	510.9447	1530.819	1.49	0.00076	6.291279
P02647	0	0.44	395.568	1184.69	1.51	0.00059	14.63811
P02647	0	0.8333	563.6303	1688.876	1.79	0.00101	62.86998
P02647	1	1	396.9924	1584.948	2.72	0.00108	33.51994
P02647	0	0.2	506.9763	1518.914	0.62	0.00031	0
P02647	0	0.8696	460.602	1379.791	0.78	0.00036	23.63401
P02647	0	0.6486	560.3249	1119.643	2.19	0.00122	0
P02647	0	0.8333	563.6289	1688.872	-0.82	-0.00046	53.00072
P02647	0	0.4167	395.5674	1184.688	-0.19	-0.00008	9.05993
P02647	0	0.8846	878.9481	1756.889	0.83	0.00073	6.310212
P02647	1	0.8919	651.37	1301.733	2.57	0.00167	0
P02647	0	1	698.8657	1396.724	0.91	0.00064	0
P02647	0	0.64	693.0196	2077.044	4.03	0.00279	39.19797
P02647	0	1	765.9137	1530.82	1.91	0.00146	66.56273
P02647	1	1	396.9915	1584.944	0.57	0.00022	58.12054
P02647	0	0.7241	509.2775	1017.548	3.19	0.00162	34.92838
P02647	0	0.9583	563.6306	1688.877	2.33	0.00131	19.33183
P02647	0	0.3529	395.5677	1184.688	0.58	0.00023	25.77604
P02647	0	0.8696	514.2807	1540.828	1.68	0.00086	3.271445
P02647	0	0.3333	592.8487	1184.69	2.01	0.00119	5.456939
P02647	0	0.913	951.0015	1900.996	3.26	0.0031	14.06745
P02647	0	1	563.6306	1688.877	2.22	0.00125	9.606052
P02647	2	1	470.4506	2348.224	0.81	0.00038	48.11077
P02647	1	0.9643	528.9871	1584.947	2.08	0.0011	4.057369
P02647	0	1	516.2635	1031.52	0.67	0.00035	0
P02647	1	1	567.2963	1699.874	1.67	0.00095	
P02647	0	0.9583	770.9184	1540.83	2.96	0.00228	4.30398
P02647	0	0.7059	741.0516	2221.14	1.02	0.00075	8.490401
P02647	0	0.9375	386.2326	1156.683	2.35	0.00091	6.323419
P02647	0	0.9375	386.2326	1156.683	2.35	0.00091	6.323419
P02647	0	0.8824	488.2726	975.538	-0.04	-0.00002	13.75586
P02647	0	0.6481	488.2736	975.5399	1.96	0.00096	4.857963
P02647	0	1	687.91	1374.813	1.08	0.00074	0
P02647	0	0.9286	651.3291	1301.651	1.91	0.00124	0
P02647	0	0.35	592.8496	1184.692	3.45	0.00204	13.72029
P02647	0	0.625	772.8914	1544.775	2.68	0.00207	6.276814
P02647	0	1	770.9172	1540.827	1.45	0.00112	4.724904
P02647	0	0.9259	651.3273	1301.647	-0.9	-0.00059	38.92931
P02647	0	1	453.9136	1359.726	2.01	0.00091	19.52173

P02647	0	0.9565	844.9372	1688.867	-3.7	-0.00312	5.385182
P02647	0	1	434.2658	1300.783	-0.01	0	20.01415
P02647	0	0.7222	741.0521	2221.142	1.76	0.0013	5.857788
P02647	0	0.625	524.9986	2096.973	1.46	0.00076	21.56983
P02647	0	0.6452	509.2768	1017.546	1.69	0.00086	0
P02647	0		510.9445	1530.819	1.07	0.00054	3.128819
P02647	0	1	651.3303	1301.653	3.69	0.0024	21.20005
P02647	0	1	481.8956	1443.672	1.85	0.00089	0
P02647	0	0.9565	651.329	1301.651	1.82	0.00118	0
P02647	0	0.34	520.7971	1040.587	1.12	0.00058	4.498678
P02647	0	1	524.9981	2096.971	0.53	0.00028	57.62544
P02774	1	0.6111	490.9566	1470.855	0.24	0.00012	0
P02774	1	1	538.9914	1614.96	1.65	0.00089	31.65588
P02774	1	0.8108	538.9918	1614.961	2.33	0.00125	13.57051
P02774	1	0.8108	490.9571	1470.857	1.23	0.0006	20.95879
P02774	0	0.8085	627.8627	1254.718	1.28	0.0008	0
P02774	0	1	466.945	1398.82	1.26	0.00059	31.71915
P02774	0	0.9556	486.9576	1458.858	1.72	0.00084	14.30832
P02774	0	1	466.9454	1398.822	2.18	0.00101	4.3955
P02774	0	1	521.9294	1563.774	1.43	0.00075	4.900436
P02774	1	0.9348	500.3008	1498.888	2.53	0.00126	8.855756
P02774	0	1	951.1099	2851.315	1.35	0.00128	0
P02774	0	1	951.1103	2851.316	1.74	0.00165	1.909381
P02774	0	1	820.8726	3280.468	-0.07	-0.00006	0
P02774	0	1	1094.164	3280.476	2.3	0.00251	2.736547
P02774	0	1	647.3232	1293.639	3.38	0.00219	0
P02774	0	1	820.8735	3280.472	1.12	0.00092	3.000134
P02774	0	1	1094.162	3280.473	1.18	0.00129	4.973901
P02774	0	1	1094.163	3280.475	2.07	0.00227	3.167392
P02774	0	1	1046.13	3136.374	2.37	0.00247	0
P02774	0	1	820.8739	3280.474	1.57	0.00128	2.32485
P02774	0	1	1094.163	3280.473	1.4	0.00153	4.092617
P02774	0	1	820.8738	3280.473	1.42	0.00116	3.706261
P02774	0	1	1094.162	3280.473	1.18	0.00129	3.366157
P02774	0	1	820.8741	3280.475	1.79	0.00147	3.563561
P02774	0	1	1094.161	3280.47	0.29	0.00031	0.371701
P02774	0	1	951.112	2851.321	3.53	0.00336	2.176339
P02774	0	1	1094.161	3280.469	0.06	0.00007	0
P02774	0	1	1094.163	3280.476	2.18	0.00239	0
P02774	0	1	903.0749	2707.21	0.3	0.00027	1.033127
P02774	0	1	903.0756	2707.212	1.04	0.00094	3.303947
P02774	0	1	903.0757	2707.213	1.25	0.00112	3.552674
P02774	0	1	903.0756	2707.212	1.11	0.001	0.90396
P02774	0	1	713.5844	2851.316	1.56	0.00111	17.41942
P02774	0	1	903.0754	2707.212	0.91	0.00082	8.398432
P02774	0	1	951.1096	2851.314	1.03	0.00098	2.100888
P02774	0	1	713.5841	2851.315	1.13	0.00081	3.630751
P02774	0	1	951.1101	2851.316	1.48	0.00141	2.136817
P02774	0	0.9524	1094.165	3280.481	3.63	0.00397	0
P02774	0	0.9556	1094.16	3280.465	-1.17	-0.00127	0
P02774	0	0.9722	1094.162	3280.473	1.18	0.00129	0
P02774	0	1	713.5848	2851.318	2.16	0.00154	1.929952

P02774	0	1	951.1091	2851.313	0.52	0.00049	2.119209
P02774	0	0.9737	781.6965	2343.075	2.62	0.00205	4.922868
P02774	0	1	781.6961	2343.074	2.07	0.00162	5.117416
P02774	0	1	781.6963	2343.074	2.31	0.0018	6.954242
P02774	0	1	713.5848	2851.318	2.16	0.00154	1.770994
P02774	0	1	951.1096	2851.314	0.97	0.00092	2.142505
P02774	0	1	951.109	2851.312	0.39	0.00037	7.917794
P02774	0	0.9412	486.9577	1458.859	1.91	0.00093	24.18926
P02774	0	1	466.9453	1398.821	1.91	0.00089	34.41379
P02774	0	1	466.9449	1398.82	1.13	0.00053	3.145844
P02774	0	1	466.9446	1398.819	0.41	0.00019	4.642865
P02774	0	0.9531	699.9139	1398.821	1.49	0.00104	3.947916
P02774	0	1	647.3221	1293.637	1.68	0.00109	0
P02774	1	0.84	500.3007	1498.888	2.29	0.00114	12.94992
P02774	1	0.9189	375.4781	1498.891	4.37	0.00164	4.137413
P02774	1	0.9268	538.9918	1614.961	2.44	0.00131	7.952909
P02774	1	0.6111	490.9569	1470.856	0.86	0.00042	6.215226
P02774	1	0.9333	404.4956	1614.96	2.17	0.00087	13.29116
P02774	1	1	538.9916	1614.96	1.99	0.00107	11.30998
P02774	1	0.5667	490.9565	1470.855	-0.01	-0.00001	14.19141
P02774	1	0.9783	538.9915	1614.96	1.87	0.00101	16.08534
P02774	1	1	538.9911	1614.959	1.08	0.00058	7.951202
P02774	1	0.9412	490.9571	1470.857	1.23	0.0006	7.538885
P02774	1	0.9394	490.9569	1470.856	0.8	0.00039	13.20278
P02774	1	0.8649	404.4957	1614.961	2.47	0.001	10.94688
P02774	0	0.973	438.9237	1314.757	2.1	0.00092	0
P02774	0	0.6032	547.8181	1094.629	-1.45	-0.00079	58.00685
P02774	1	1	538.9923	1614.962	3.35	0.0018	19.00097
P02774	1	0.9565	538.9913	1614.959	1.53	0.00082	50.19981
P02774	0	0.975	486.9581	1458.86	2.66	0.00129	6.638851
P02774	0	0.9787	486.9569	1458.856	0.21	0.0001	10.29313
P02774	0	0.8333	729.933	1458.859	1.94	0.00141	6.351958
P02774	0	0.9744	486.9584	1458.861	3.29	0.0016	21.30447
P02774	0	0.9722	486.958	1458.86	2.54	0.00123	17.04759
P02774	0	0.7857	729.9333	1458.859	2.36	0.00172	5.432534
P02774	0	0.963	486.9574	1458.858	1.22	0.00059	8.799095
P02774	0	0.9701	837.4489	1673.891	2.32	0.00194	12.35677
P02774	1	0.8085	456.2815	1366.83	1.88	0.00086	13.72063
P02774	1	0.9302	456.2805	1366.827	-0.33	-0.00015	11.19941
P02774	0	1	558.6353	1673.891	2.72	0.00152	8.994144
P02774	0	0.9848	837.4484	1673.89	1.74	0.00146	4.286998
P02774	1	0.9091	532.5604	2127.22	1.05	0.00056	37.95059
P02774	1	0.9375	709.7456	2127.222	2.29	0.00163	11.30849
P02774	0	0.9516	837.4638	2510.377	1.87	0.00157	10.14027
P02774	0	1	1327.742	2654.478	1.27	0.00168	0
P02774	1	0.6111	490.9574	1470.858	1.79	0.00088	11.71552
P02774	0	1	710.3397	1419.672	1.81	0.00128	0
P02774	0	0.9565	486.9576	1458.858	1.72	0.00084	9.88282
P02774	0	1	794.3482	2381.03	0.13	0.0001	3.922425
P02774	0	1	1191.021	2381.034	1.88	0.00223	0.430304
P02774	0	1	794.3496	2381.034	1.82	0.00144	3.735266
P02774	0	1	596.0147	2381.037	3.05	0.00181	3.678235

P02774	0	1	794.3492	2381.033	1.44	0.00114	10.76576
P02774	0	1	794.3468	2381.026	-1.64	-0.0013	22.57772
P02774	0	0.8491	619.0132	1855.025	2.5	0.00155	8.034148
P02774	0	1	781.6954	2343.072	1.14	0.00089	4.302599
P02774	0	1	1172.041	2343.075	2.39	0.0028	0.388275
P02774	0	1	781.696	2343.073	1.92	0.0015	4.072446
P02774	0	1	781.6957	2343.072	1.53	0.00119	3.675288
P02774	0	1	781.6962	2343.074	2.15	0.00168	7.841246
P02774	0	1	1172.041	2343.074	2.18	0.00256	0
P02774	0	1	781.6957	2343.072	1.53	0.00119	4.446748
P02774	0	1	781.6958	2343.073	1.68	0.00132	24.25493
P02774	0	0.9815	781.696	2343.074	2	0.00156	56.30687
P02774	0	1	781.6964	2343.075	2.39	0.00186	4.596306
P02774	0	0.9783	781.695	2343.07	0.59	0.00046	7.870258
P02774	0	0.9286	781.6952	2343.071	0.9	0.00071	11.2229
P02774	0	1	888.0463	2662.124	2.13	0.00189	33.88107
P02774	0	1	781.6964	2343.075	2.39	0.00186	9.46622
P02774	0	1	888.0462	2662.124	2	0.00177	35.21415
P02774	0	1	781.6962	2343.074	2.23	0.00174	4.246404
P02774	1	0.9	654.8546	2616.396	2	0.0013	8.424417
P02774	0	1	888.0451	2662.121	0.76	0.00067	2.272625
P02774	0	1	1331.564	2662.12	0.63	0.00083	0
P02774	0	1	888.0451	2662.121	0.76	0.00067	25.48631
P02774	0	1	661.569	2643.254	0.44	0.00029	2.907697
P02774	0	1	881.7516	2643.24	-4.8	-0.00423	6.772026
P02774	0	0.8305	620.8623	1240.717	2.73	0.00169	5.274197
P02774	0	0.9722	414.2438	1240.717	2.35	0.00097	10.40087
P02774; Q8	0	0.7759	483.298	965.5887	1.77	0.00086	4.257823
P02774	0	1	833.723	2499.155	1.47	0.00123	42.39676
P02774	0	1	803.686	2409.044	1.9	0.00153	2.184384
P02774	0	1	851.7206	2553.147	2.48	0.00211	6.023157
P02774	0	1	803.6861	2409.044	1.98	0.00159	2.764059
P02774	0	1	661.5695	2643.256	1.17	0.00078	3.03529
P02774	0	1	881.7567	2643.255	0.95	0.00084	4.300338
P02774	0	0.9756	521.9299	1563.775	2.37	0.00123	12.7579
P02774	0	1	521.9298	1563.775	2.02	0.00105	0
P02774	0	1	702.3131	1403.619	1.05	0.00074	3.290761
P02774	1	1	532.5607	2127.221	1.63	0.00086	6.384738
P02774	1	0.9512	709.7448	2127.22	1.09	0.00077	10.79277
P02774	1	0.9412	661.7117	1983.121	2.68	0.00177	13.48796
P02774	0	0.8	613.8485	1226.69	2.09	0.00128	5.952579
P02774	0	0.8689	613.8498	1226.692	4.27	0.00262	16.57302
P02774	1	0.8182	496.5348	1983.117	1.07	0.00053	3.301055
P02774	1	0.8182	496.5348	1983.117	1.07	0.00053	3.301055
P02774	1	0.9412	661.7117	1983.121	2.68	0.00177	13.48796
P02774	1	1	532.5609	2127.222	1.97	0.00105	11.83925
P02774	1	1	709.746	2127.224	2.9	0.00205	7.800463
P02774	0	1	647.3232	1293.639	3.38	0.00219	13.51746
P02774	0	1	873.6816	3491.705	-0.69	-0.00061	6.178287
P02774	1	1	532.562	2127.226	4.04	0.00215	5.654046
P02774	1	0.9556	709.7449	2127.22	1.35	0.00095	11.39899
P02774	0	1	1195.938	3585.799	2.03	0.00242	3.758381

P02774	0	0.8219	613.8474	1226.687	0.29	0.00018	6.339625
P02774	1	0.9298	661.7118	1983.121	2.77	0.00183	3.706608
P02774	1	0.7442	709.7462	2127.224	3.07	0.00217	29.39446
P02774	1	0.9298	661.7118	1983.121	2.77	0.00183	3.706608
P02774	0	1	579.2388	1735.702	1.25	0.00072	9.909491
P02774	0	0.8788	613.8489	1226.691	2.88	0.00177	20.09252
P02774	0	1	579.2389	1735.702	1.35	0.00078	6.410325
P02774	0	0.7541	619.8386	1238.67	3.68	0.00228	14.61842
P02774	0	0.973	521.9299	1563.775	2.37	0.00123	47.46926
P02774	0	1	639.0418	2553.145	1.61	0.00103	3.738093
P02774	0	1	851.7194	2553.144	1.05	0.00089	3.829121
P02774	0	1	851.7202	2553.146	1.98	0.00168	3.820195
P02774	0	1	647.3219	1293.637	1.4	0.00091	6.620681
P02774	0	1	782.3905	1563.774	1.4	0.00109	6.16034
P02774	0	0.9565	521.9299	1563.775	2.37	0.00123	12.92054
P02774	0	1	796.3024	1591.598	-0.08	-0.00006	18.70213
P02774	0	0.9286	1046.127	3136.367	0.26	0.00028	11.95869
P02774	1	0.8966	490.9575	1470.858	1.98	0.00097	37.11122
P02774	1	1	500.3004	1498.887	1.67	0.00084	48.35133
P02774	0	0.7692	414.2431	1240.715	0.58	0.00024	8.470369
P02774	0	1	733.6619	2198.971	1.91	0.0014	2.425757
P02774	0	1	466.9455	1398.822	2.44	0.00114	17.51867
P02774	0	1	1094.162	3280.47	0.51	0.00056	0
P02774	0	1	418.9112	1254.719	2.05	0.00086	6.323921
P02774	0	1	466.9453	1398.821	1.98	0.00092	59.28594
P02774; Q8	0	0.7759	483.2975	965.5876	0.7	0.00034	0
P02774	0	1	702.3134	1403.619	1.49	0.00104	7.404727
P02774; Q8	0	0.7538	483.2982	965.5892	2.28	0.0011	16.79695
P02774	0	1	431.8839	1293.637	1.98	0.00085	45.01679
P02774	0	0.8333	486.9577	1458.859	1.91	0.00093	63.07834
P02774	0	0.8696	619.8381	1238.669	2.9	0.00179	51.15693
P02774	0	1	1094.162	3280.47	0.51	0.00056	0
P02774	0	0.807	547.7863	1094.565	1.95	0.00107	4.801783
P02774	0	1	702.3138	1403.62	2.09	0.00147	0
P02774	0	0.8837	657.8814	1314.756	1.33	0.00087	24.88596
P02774	1	0.9	654.8553	2616.399	3.11	0.00204	9.704162
P02774	0	1	820.8737	3280.473	1.27	0.00104	24.73855
P02774	0	0.913	1094.16	3280.466	-0.83	-0.00091	0
P02774	0	0.9706	431.884	1293.637	2.05	0.00088	8.056562
P02774	0	0.7241	414.2438	1240.717	2.27	0.00094	15.98284
P02774	0	0.807	547.7866	1094.566	2.4	0.00131	5.446554
P02774	0	0.975	473.8958	1419.673	2.27	0.00108	0
P02774	0	0.8333	619.8365	1238.666	0.33	0.00021	5.273166
P02774	1	0.625	490.9571	1470.857	1.3	0.00064	47.86494
P02774; Q8	0	0.7538	483.2995	965.5917	4.87	0.00235	0
P02774	0	0.96	781.6963	2343.074	2.31	0.0018	0
P02774	0	0.8103	547.7869	1094.566	2.95	0.00162	0
P02774	0	1	702.3131	1403.619	1.05	0.00074	5.512931
P02774	0	1	702.3127	1403.618	0.53	0.00037	5.977122
P02774	0	0.9348	418.9108	1254.718	1.1	0.00046	5.872527
P02774	0	0.9375	521.93	1563.775	2.48	0.0013	12.33831
P02774	0	0.7949	547.8188	1094.63	-0.11	-0.00006	16.16144

P02774	0	0.9318	438.9238	1314.757	2.24	0.00098	6.194859
P02774	1	1	458.2897	1372.855	1.64	0.00075	39.88477
P02774	1	1	456.2831	1366.835	5.37	0.00245	45.50527
P02774; Q8	0	0.7925	483.298	965.5887	1.84	0.00089	8.885428
P02774	0	1	418.9107	1254.717	0.81	0.00034	34.32304
P02774; Q8	0	0.6458	483.2982	965.589	2.15	0.00104	8.608858
P02774	0	1	414.2435	1240.716	1.61	0.00067	25.7203
P02774	0	0.8667	438.9234	1314.756	1.41	0.00062	2.630655
P02774	0	0.9565	414.2441	1240.718	3.16	0.00131	17.4832
P02774	0	1	1094.163	3280.474	1.51	0.00166	1.546012
P02774	0	0.4286	544.3531	1087.699	2.2	0.0012	6.535117
P02774	0	1	781.6972	2343.077	3.48	0.00272	13.21325
P02774	1	0.9091	490.9569	1470.856	0.8	0.00039	22.97277
P02774	0	0.6111	414.2429	1240.714	0.13	0.00006	37.24466
P02774	1	0.95	404.4955	1614.96	1.86	0.00075	50.54002
P02774	0	0.5758	619.0153	1855.031	5.95	0.00368	34.67757
P02774	0	0.9524	781.6947	2343.07	0.28	0.00022	9.719802
P02774	1	0.9032	375.4775	1498.888	2.83	0.00106	4.185336
P02774	0	0.8444	619.8381	1238.669	2.9	0.00179	0
P02774; Q8	0	0.7895	483.2989	965.5904	3.61	0.00174	11.10543
P02774	1	0.7381	683.9185	1366.83	1.6	0.0011	9.03268
P02774	0	1	794.3481	2381.03	-0.03	-0.00002	2.984292
P02774	0	0.675	613.8497	1226.692	4.18	0.00256	12.94419
P02774	0	1	888.0439	2662.117	-0.62	-0.00055	9.919293
P02774	0	0.72	515.3135	1029.62	1.38	0.00071	29.11964
P02774	0	1	702.3129	1403.619	0.88	0.00062	9.475031
P02774	0	0.6667	414.2438	1240.717	2.42	0.001	15.24767
P02774	0	0.9545	781.6936	2343.066	-1.13	-0.00088	0
P02774	0	1	547.7875	1094.568	4.07	0.00223	10.71735
P02774	0	1	888.0454	2662.122	1.1	0.00098	9.207657
P02774	0	0.9615	486.959	1458.862	4.54	0.00221	26.85228
P02774	0	0.7879	657.8819	1314.757	2.07	0.00136	0
P02774	1	1	461.6135	1382.826	2.58	0.00119	31.08832
P02774	2	1	520.5311	2079.102	0.35	0.00018	29.02924
P02774	0	0.6333	443.2626	885.5179	1.96	0.00087	5.905873
P02774	0	1	794.3483	2381.03	0.28	0.00022	21.06667
P02774	0	0.8571	664.3753	2654.479	1.95	0.00129	12.76943
P02774	0	0.8621	541.7972	1082.587	2.04	0.0011	2.869317
P02774	0	0.7917	781.6955	2343.072	1.29	0.00101	5.975255
P02774	0	0.9259	820.8743	3280.475	2.01	0.00165	0.922815
P02774	0	0.8182	547.7864	1094.565	2.06	0.00113	19.53961
P02774	1	1	461.613	1382.824	1.39	0.00064	37.62499
P02774	1	1	500.302	1498.891	4.85	0.00242	60.25291
P02774; Q8	0	0.7458	483.2989	965.5906	3.73	0.0018	31.37271
P02774	0	1	888.0466	2662.125	2.41	0.00214	3.621063
P02774; Q8	0	0.7903	411.2469	821.4866	2.03	0.00083	12.27218
P02774	0	1	1191.012	2381.017	-5.51	-0.00655	28.76226
P02774	1	0.641	408.247	1222.726	0.74	0.0003	27.74141
P02774	1	0.641	408.247	1222.726	0.74	0.0003	27.74141
P02774	0	0.9231	781.697	2343.077	3.25	0.00254	6.079325
P02774	0	0.5	515.3143	1029.621	3.04	0.00156	12.08007
P02774	0	0.7143	627.8645	1254.722	4.2	0.00263	39.11585

P02774	0	1	521.9294	1563.774	1.31	0.00068	71.29767
P02774	0	0.9355	782.3889	1563.771	-0.63	-0.00049	0
P02774	0	1	951.1054	2851.301	-3.47	-0.00329	25.64327
P02774	0	1	868.3553	1735.703	2.03	0.00176	67.67986
P02774	0	0.8571	409.5681	1226.69	2.22	0.00091	10.41526
P02774	0	0.1538	544.353	1087.699	1.98	0.00107	31.47065
P02774	0	1	820.8715	3280.464	-1.34	-0.0011	20.50096
P02774	0	1	647.3211	1293.635	0.17	0.00011	0
P02774; Q8	0	0.7538	483.2971	965.5869	-0.06	-0.00003	11.14255
P02774	0	1	584.5713	1751.699	2.64	0.00154	48.57916
P02774	1	1	461.6138	1382.827	3.25	0.0015	39.0444
P02774	0	1	521.9285	1563.771	-0.33	-0.00017	26.97382
P02774	0	0.1081	544.3529	1087.699	1.86	0.00101	23.89579
P02774	1	0.88	532.5598	2127.217	0.02	0.00001	20.23253
P02774	0	0.6667	414.2437	1240.716	1.98	0.00082	3.051709
P02774	0	0.575	547.786	1094.565	1.39	0.00076	35.94297
P02774	0	0.8667	547.7872	1094.567	3.51	0.00192	20.08314
P02774	0	0.3871	613.8492	1226.691	3.38	0.00207	100
P02774	0	0.5957	544.3535	1087.7	2.87	0.00156	6.748145
P02774	1	0.72	709.746	2127.224	2.9	0.00205	7.030743
P02774	0	1	414.2434	1240.716	1.32	0.00054	42.03664
P02774	0	0.6667	547.7866	1094.566	2.4	0.00131	36.53055
P02774	0	0.875	431.8833	1293.635	0.49	0.00021	45.89429
P02774	1	0.6757	413.5793	1238.723	2.35	0.00097	32.35081
P02774	1	0.6757	413.5793	1238.723	2.35	0.00097	32.35081
P02774	0	0.8108	547.7855	1094.564	0.39	0.00021	74.05232
P02774	0	1	466.9455	1398.822	2.37	0.00111	55.68572
P02774	0	0.7778	885.498	2654.479	1.95	0.00173	39.33886
P02774	0	1	888.0461	2662.124	1.86	0.00165	4.083715
P02774	0	0.6897	438.9231	1314.755	0.64	0.00028	23.3421
P02774	0	1	796.3022	1591.597	-0.39	-0.00031	35.72642
P02774	0	0.8235	521.9302	1563.776	2.84	0.00148	55.44266
P02774	0	0.7647	547.7863	1094.565	1.95	0.00107	46.06146
P02774	0	1	468.5445	1403.619	1.19	0.00056	9.279737
P02774	1	0.5714	490.9572	1470.857	1.36	0.00067	63.94419
P02774	0	1	1094.163	3280.475	1.85	0.00202	0
P02774	0	0.5882	466.946	1398.824	3.62	0.00169	42.94089
P02774	1	1	456.2817	1366.83	2.22	0.00101	15.38469
P02774	0	0.7692	781.6976	2343.078	3.95	0.00309	50.58721
P02774; Q8	0	0.6809	411.2463	821.4853	0.54	0.00022	17.26967
P02774	0	1	888.0488	2662.132	4.95	0.0044	5.255212
P02774	0	1	466.9448	1398.82	1	0.00047	11.40425
P02774	0	1	833.7229	2499.154	1.33	0.0011	12.72684
P02774	0	0.825	547.7856	1094.564	0.61	0.00033	49.57756
P02774	0	0.6154	820.8718	3280.465	-0.96	-0.00079	22.74736
P02774	0	0.9286	547.8206	1094.634	3.23	0.00177	10.2222
P02774	1	0.4375	749.9471	1498.887	1.95	0.00146	14.02988
P02774	1	0.7778	532.5611	2127.223	2.43	0.00129	13.75479
P02774	0	1	733.6621	2198.972	2.16	0.00158	0
P02774	0	0.9286	438.9239	1314.757	2.59	0.00114	7.129221
P02774	0	1	579.2374	1735.698	-1.28	-0.00074	20.40243
P02774	0	1	468.5446	1403.619	1.39	0.00065	8.146728

P02774	0	0.7429	547.7871	1094.567	3.29	0.0018	15.91132
P02774	0	1	888.0449	2662.12	0.55	0.00049	70.35582
P06727	0	0.9492	680.3805	1359.754	0.17	0.00011	2.960161
P06727	0	0.8723	453.9234	1359.756	1.53	0.00069	9.45674
P06727	0	0.9508	680.3806	1359.754	0.26	0.00018	4.028257
P06727	0	0.8571	453.9232	1359.755	1.06	0.00048	4.073812
P06727	0	0.8235	508.632	1523.881	1.98	0.00101	6.12747
P06727	0	0.971	644.3378	1287.668	2.51	0.00161	0
P06727	0	0.6744	616.3438	1847.017	4.3	0.00265	37.90068
P06727	0	1	485.9407	1455.808	0.02	0.00001	26.61226
P06727	0	1	1227.941	3681.807	1.1	0.00135	1.713722
P06727	0	1	921.2083	3681.811	2.11	0.00194	3.172803
P06727	0	1	1233.274	3697.808	2.7	0.00333	0
P06727	0	0.9048	508.6312	1523.879	0.48	0.00025	10.60894
P06727	0	0.9556	508.6315	1523.88	1.02	0.00052	15.54411
P06727	0	1	459.2548	1375.75	0.97	0.00044	5.945319
P06727	0	0.6667	508.6325	1523.883	2.95	0.0015	23.82719
P06727	0	0.7895	508.6307	1523.878	-0.48	-0.00024	18.75703
P06727	1	1	432.758	1728.01	3.11	0.00134	12.96531
P06727	1	1	576.6749	1728.01	3.23	0.00186	26.38807
P06727	1	1	432.7571	1728.007	1.21	0.00052	6.554672
P06727	1	1	432.7561	1728.003	-1.2	-0.00052	0
P06727	0	0.971	644.3378	1287.668	2.51	0.00161	0
P06727	1	0.9362	557.795	2228.158	1.95	0.00109	7.521988
P06727	0	1	485.9406	1455.807	-0.3	-0.00014	4.45916
P06727	0	1	485.9414	1455.81	1.4	0.00068	17.3245
P06727	0	0.2069	568.3085	1702.911	2.56	0.00145	2.108785
P06727	0	0.9167	728.4081	1455.809	0.95	0.00069	18.70685
P06727	0	1	485.941	1455.808	0.58	0.00028	3.132308
P06727	0	0.2093	616.343	1847.015	3.11	0.00192	11.43126
P06727	0	1	698.713	2094.124	1	0.0007	36.06151
P06727	0	1	524.2882	2094.131	4.2	0.0022	34.54065
P06727	0	0.9815	524.2865	2094.124	0.94	0.00049	5.559986
P06727	0	0.2037	568.3082	1702.91	1.91	0.00109	16.58995
P06727	1	1	432.7581	1728.011	3.4	0.00147	33.37627
P06727	0	0.7385	488.2583	975.5093	-1.32	-0.00064	9.932269
P06727	0	1	437.9071	1311.707	0.91	0.0004	0
P06727	0	0.8571	680.3816	1359.756	1.78	0.00121	0
P06727	0	0.8795	644.3375	1287.668	2.03	0.00131	0
P06727	1	1	420.5622	1259.672	1.42	0.0006	5.253106
P06727	0	1	691.3559	2072.053	0.12	0.00008	2.560395
P06727	0	1	691.3562	2072.054	0.56	0.00039	2.336205
P06727	0	1	691.3561	2072.054	0.47	0.00032	11.38219
P06727	0	0.8788	477.9281	1431.77	1.7	0.00081	64.45287
P06727	0	0.8923	716.3876	1431.768	0.54	0.00039	3.175633
P06727	0	0.8545	477.9275	1431.768	0.55	0.00026	6.002842
P06727	0	0.8298	716.3881	1431.769	1.22	0.00088	2.696838
P06727	0	0.7826	551.7913	1102.575	1.24	0.00068	34.6492
P06727	0	0.6429	688.3881	1375.769	1.27	0.00087	35.07481
P06727	0	0.5636	459.2609	1375.768	0.81	0.00037	32.39775
P06727	0	0.6724	688.3873	1375.767	0.2	0.00014	10.79175
P06727	0	0.8039	623.8427	1246.678	1.77	0.0011	3.595778

P06727	0	0.9091	621.3596	1241.712	2.23	0.00138	5.21404
P06727	0	0.9211	414.5747	1241.71	0.3	0.00012	8.905455
P06727	0	0.8113	621.3595	1241.712	2.03	0.00126	4.161651
P06727	0	0.8108	414.5747	1241.709	0.22	0.00009	0
P06727	0	0.8163	567.8221	1134.637	1.16	0.00066	16.09152
P06727	1	1	547.6269	1640.866	1.62	0.00089	8.863574
P06727	1	1	547.6267	1640.866	1.28	0.0007	12.07576
P06727	1	1	547.627	1640.866	1.73	0.00095	11.96443
P06727	1	0.7179	421.9262	1263.764	0.58	0.00024	27.55602
P06727	0	0.8806	492.2805	983.5536	1.64	0.00081	2.910095
P06727	0	0.7455	560.3099	1119.612	-0.12	-0.00007	5.986587
P06727	0	0.9048	546.9584	1638.861	1.86	0.00101	8.813287
P06727	0	0.8442	819.9335	1638.86	1.27	0.00104	6.55032
P06727	0	0.9848	546.9592	1638.863	3.31	0.00181	8.178195
P06727	0	0.8923	848.4622	1695.917	0.94	0.0008	5.724376
P06727	0	0.9286	644.3373	1287.667	1.75	0.00113	12.63548
P06727	0	0.8919	644.3373	1287.667	1.65	0.00107	0
P06727	0	1	571.3062	2282.203	2.15	0.00122	4.440652
P06727	0	0.8675	644.3377	1287.668	2.32	0.00149	1.961263
P06727	0	1	571.3081	2282.211	5.57	0.00318	62.94411
P06727	0	0.9655	565.9764	1695.915	-0.47	-0.00026	8.078141
P06727	0	0.9231	686.3735	1371.74	-0.11	-0.00008	13.8649
P06727	1	0.5714	438.7477	1751.969	-0.25	-0.00011	33.4566
P06727	0	0.7564	488.2597	975.5121	1.56	0.00076	0
P06727	1	1	584.6623	1751.972	1.77	0.00104	13.97711
P06727	0	0.6	624.8391	1248.671	0.29	0.00018	6.674681
P06727	0	0.68	564.3319	1127.657	2.18	0.00123	3.19594
P06727	0	0.1316	462.508	1847.01	0.69	0.00032	13.38709
P06727	0	0.1731	616.3409	1847.008	-0.36	-0.00022	41.0845
P06727	1	1	547.6269	1640.866	1.62	0.00089	14.29256
P06727	1	0.6579	530.3025	1588.893	2.04	0.00108	7.031206
P06727	0	0.9677	546.9578	1638.859	0.74	0.0004	11.29132
P06727	0	0.881	621.3593	1241.711	1.73	0.00108	6.430236
P06727	0	0.6176	414.5739	1241.707	-1.69	-0.0007	83.02617
P06727	0	0.7778	565.9778	1695.919	1.91	0.00108	0
P06727	0	1	573.6406	1718.907	3.33	0.00191	43.21102
P06727	0	1	621.673	1863.005	0.48	0.0003	18.85314
P06727	0	0.9825	608.3307	1215.654	2.21	0.00134	0
P06727	0	0.7333	560.3109	1119.614	1.62	0.00091	0
P06727	0	1	761.405	2282.2	1.08	0.00082	3.00439
P06727	0	1	492.2794	983.5514	-0.59	-0.00029	9.874304
P06727	0	0.85	567.8228	1134.638	2.34	0.00133	24.86612
P06727	0	0.9211	414.5743	1241.708	-0.59	-0.00024	8.378055
P06727	0	0.92	641.687	1923.046	2.04	0.00131	6.386996
P06727	0	0.75	508.6315	1523.88	0.96	0.00049	14.29564
P06727	0	0.8125	460.5969	1379.776	-0.21	-0.0001	34.3982
P06727	1	1	547.6261	1640.864	0.17	0.00009	14.75041
P06727	0	0.9062	524.2867	2094.125	1.41	0.00074	18.60154
P06727	1	1	410.9719	1640.866	1.46	0.0006	16.50284
P06727	0	0.7105	455.5868	1364.746	0.35	0.00016	7.692957
P06727	0	0.8125	567.8209	1134.635	-0.99	-0.00056	5.167284
P06727	0	1	414.5749	1241.71	0.67	0.00028	68.42307

P06727	0	1	641.6881	1923.05	3.66	0.00235	0
P06727	0	0.74	488.2595	975.5116	1.12	0.00055	32.80059
P06727	0	0.907	688.3786	1375.75	1.11	0.00076	2.541368
P06727	0	0.76	560.3115	1119.616	2.82	0.00158	51.39714
P06727	0	0.8372	567.8225	1134.638	1.69	0.00096	0.810582
P06727	0	0.9231	567.8218	1134.636	0.62	0.00035	38.15883
P06727	0	1	690.3925	1379.778	0.95	0.00066	6.02578
P06727	0	1	485.9418	1455.811	2.28	0.00111	0
P06727	0	1	644.3386	1287.67	3.74	0.00241	50.02164
P06727	0	0.8235	623.8434	1246.679	2.85	0.00177	0
P06727	0	0.7297	616.3367	1231.666	0.93	0.00057	12.06413
P06727	1	0.963	378.212	1132.622	-0.54	-0.0002	25.40339
P06727	0	1	459.2536	1375.746	-1.56	-0.00072	62.52724
P06727	0	0.375	459.2616	1375.77	2.34	0.00107	49.72394
P06727	0	1	614.3251	1227.643	4.16	0.00255	61.49575
P06727	0	0.6571	624.8386	1248.67	-0.39	-0.00025	5.827014
P06727	1	0.5238	416.9348	1248.79	0.93	0.00039	13.72218
P06727	1	1	576.6737	1728.007	1.11	0.00064	68.68835
P06727	0	0.3793	429.8934	1287.666	0.46	0.0002	40.90447
P06727	0	0.6905	549.3079	1097.608	1.2	0.00066	24.27081
P06727	0	1	682.8772	1364.747	1.37	0.00093	21.45703
P06727	1	0.9167	584.6606	1751.967	-1.05	-0.00061	21.3231
P06727	0	1	567.822	1134.637	0.94	0.00053	46.50326
P06727	1	0.125	498.5348	1991.117	9.45	0.00471	27.9258
P06727	0	0.9118	437.9075	1311.708	1.82	0.0008	61.70761
P06727	0	0.8571	691.3562	2072.054	0.56	0.00039	7.475385
P06727	0	0.5526	552.789	1104.571	2.11	0.00116	13.54788
P06727	0	1	495.5926	1484.763	0.58	0.00029	47.19476
P06727	0	0.5	643.3225	1927.953	1.02	0.00065	6.272182
P06727	0	0.7391	680.3818	1359.756	2.05	0.0014	3.480474
P06727	0	0.9189	614.3234	1227.639	1.37	0.00084	7.348125
P06727	0	0.5429	564.3317	1127.656	1.75	0.00098	14.57245
P06727	1	0.55	416.9349	1248.79	1.07	0.00045	22.4493
P06727	0	0.95	477.9273	1431.767	0.04	0.00002	19.12751
P06727	1	0.9574	630.3394	1259.671	0.95	0.0006	5.222537
P06727	0	0.8929	560.3115	1119.616	2.82	0.00158	56.12635
P06727	0	1	457.918	1371.74	-0.21	-0.0001	8.617443
P06727	0	0.8	762.4434	1523.879	0.69	0.00053	5.255046
P06727	0	0.6667	623.8425	1246.678	1.48	0.00092	30.21508
P06727	0	0.7667	728.4088	1455.81	1.79	0.0013	18.20561
P06727	1	1	547.626	1640.864	0.06	0.00003	33.06539
P06727	1	0.5263	416.9347	1248.79	0.71	0.00029	27.50972
P06727	0	0.5	462.5078	1847.009	0.3	0.00014	39.25159
P06727	0	0.5556	552.7901	1104.573	4.1	0.00226	0
P06727	0	0.6389	488.2588	975.5104	-0.19	-0.00009	22.1055
P06727	0	1	742.8857	1484.764	1.25	0.00093	34.21469
P04275	0	0.9434	816.3906	2447.157	0.38	0.00031	2.21813
P04275	0	1	816.392	2447.162	2.1	0.00171	6.001658
P04275	0	0.9111	588.9815	1764.93	2.9	0.00171	7.378235
P04275	0	0.878	882.9679	1764.929	2.08	0.00184	7.120953
P04275	0	0.6389	510.3039	1528.897	2.18	0.00111	45.90638
P04275	0	1	853.4756	2558.412	2.48	0.00211	3.401361

P04275	0	1	767.7529	2301.244	1.05	0.00081	22.84845
P04275	0	1	500.2304	1498.677	0.66	0.00033	10.61148
P04275	0	1	605.6671	1814.987	4.56	0.00276	0
P04275	0	1	426.5566	1277.655	0.99	0.00042	59.24736
P04275	0	1	480.2385	1438.701	2.21	0.00106	58.1483
P04275	0	0.7805	442.2282	1324.67	0.87	0.00038	8.0851
P04275	0	0.7292	662.8394	1324.671	2.03	0.00134	21.44722
P04275	0	0.6604	598.3169	1195.627	2.87	0.00171	7.044303
P04275	0	0.9661	610.9149	1220.822	0.32	0.0002	46.55073
P04275	0	0.8095	576.6539	1727.947	3.16	0.00182	31.35785
P04275	1	1	561.9728	1683.904	3.55	0.00199	27.79349
P04275	0	1	509.2938	1525.867	1.73	0.00088	60.20037
P04275	0	1	591.8055	1182.604	3.7	0.00219	10.27722
P04275	0	0.898	641.3529	1281.699	4.41	0.00283	12.44727
P04275	0	1	441.2186	1321.641	2.18	0.00096	12.86118
P04275	0	0.8788	545.3131	1089.619	1.5	0.00082	33.31208
P04275	0	1	671.9686	2013.891	1.44	0.00096	34.09647
P04275	1	0.9474	521.9753	1563.911	1.02	0.00053	32.03893
P04275	0	1	488.2753	1462.811	0.01	0.00001	51.10168
P04275	0	0.9091	533.6024	1598.793	1.01	0.00054	19.93452
P04275	0	0.85	822.0936	2464.266	1.2	0.00099	9.721158
P04275	0	0.8286	387.2112	1159.619	4.93	0.0019	15.81307
P04275	0	0.7391	828.4188	3310.653	-0.49	-0.0004	3.680036
P04275	0	0.3333	488.276	1462.814	1.58	0.00077	39.33599
P04275	0	0.8293	399.2145	1195.629	5	0.00199	37.76895
P04275	0	0.7778	568.3416	1135.676	1.08	0.00061	44.65006
P04275	0	0.8462	796.4632	1591.919	0.97	0.00077	14.57251
P04275	0	0.6176	495.2976	989.5879	1.89	0.00094	22.03168
P04275	0	1	689.3414	1377.675	1.41	0.00097	0
P04275	0	0.2857	521.6254	1562.862	0.92	0.00048	10.50297
P04275	0	1	639.2921	1915.862	3.59	0.00229	6.513235
P04275	0	1	657.3539	1313.701	2.74	0.0018	24.04216
P00751	0	0.971	710.3867	1419.766	1.5	0.00107	0
P00751	0	0.5714	555.345	1109.683	1.63	0.0009	8.607278
P00751	0	0.9474	422.2594	1264.764	1.72	0.00072	11.96329
P00751	1	1	510.9753	1530.911	1.2	0.00061	29.18293
P00751	0	0.9661	533.5368	2131.125	2.47	0.00131	4.836981
P00751	0	0.977	1066.066	2131.125	2.42	0.00258	0
P00751	0	1	711.0461	2131.124	1.76	0.00125	4.960993
P00751	1	0.9032	394.9765	1576.884	2.1	0.00083	14.35872
P00751	0	0.8852	627.8516	1254.696	2.2	0.00138	5.725335
P00751	0	0.8983	627.8518	1254.696	2.49	0.00156	6.496327
P00751	0	1	422.2594	1264.764	1.57	0.00066	12.25582
P00751	0	1	711.046	2131.123	1.5	0.00106	4.382853
P00751	0	1	422.2596	1264.764	2.22	0.00094	13.90043
P00751	0	0.8889	422.2598	1264.765	2.66	0.00112	13.5151
P00751	1	0.7273	516.9888	1548.952	0.66	0.00034	13.35348
P00751	0	1	756.3848	2267.14	0.2	0.00015	2.150215
P00751	0	1	422.2618	1264.771	7.29	0.00307	21.17756
P00751	0	0.8571	396.5961	1187.774	1.97	0.00078	19.4771
P00751	0	0.8108	396.5956	1187.772	0.59	0.00023	11.55589
P00751	0	0.8667	396.5962	1187.774	2.28	0.0009	31.0502

P00751	0	0.8889	396.5964	1187.775	2.67	0.00106	23.52549
P00751	0	0.8462	396.5963	1187.774	2.51	0.00099	10.74182
P00751	1	0.7241	394.9762	1576.883	1.41	0.00055	6.322933
P00751	0	1	686.9892	2058.953	1.79	0.00123	9.820403
P00751	0	0.973	1029.981	2058.954	2.45	0.00252	0
P00751	0	1	686.9886	2058.951	0.9	0.00062	2.535333
P00751	0	1	686.9895	2058.954	2.23	0.00153	1.570058
P00751	0	0.7288	638.8823	1276.757	2.87	0.00183	5.540445
P00751	0	0.96	521.9609	1563.868	1.39	0.00072	6.597873
P00751	0	0.9722	782.4374	1563.867	0.89	0.00069	7.995002
P00751	1	1	510.9746	1530.909	0.01	0	12.64827
P00751	1	1	508.6362	1523.894	2.96	0.00151	5.860907
P00751	0	1	422.2596	1264.764	2.15	0.00091	4.865951
P00751	0	1	830.3937	3318.553	1.72	0.00143	11.6181
P00751	1	0.8649	601.5865	2403.324	2.9	0.00174	6.990106
P00751	1	0.9688	801.7785	2403.321	1.58	0.00127	4.910675
P00751	1	0.5897	801.7799	2403.325	3.26	0.00261	19.21603
P00751	0	1	987.1671	2959.487	2.89	0.00286	0
P00751	0	1	516.2597	1546.765	1.79	0.00092	20.6384
P00751	0	0.8929	481.9487	1443.831	1.21	0.00058	9.66556
P00751	0	0.8472	722.4196	1443.832	1.47	0.00106	8.152803
P00751	0	1	920.4788	2759.422	-0.06	-0.00005	14.83615
P00751	0	1	968.5154	2903.532	2.7	0.00261	2.420519
P00751	0	1	726.6376	2903.529	1.64	0.00119	2.85454
P00751	0	0.8657	898.95	1796.893	1.2	0.00108	6.526073
P00751	0	0.9733	599.6354	1796.892	0.72	0.00043	8.954595
P00751	0	0.9286	599.6362	1796.894	2.04	0.00122	31.33358
P00751	0	0.9054	670.3724	1339.737	2.04	0.00137	18.3385
P00751	0	0.8983	670.3723	1339.737	1.86	0.00124	11.03575
P00751	0	0.8824	708.3569	1415.706	1.39	0.00098	6.812879
P00751	0	0.8409	708.3585	1415.71	3.63	0.00257	0
P00751	1	0.75	526.2994	1576.884	1.92	0.00101	10.13564
P00751	1	0.6667	788.9436	1576.88	-0.51	-0.0004	0
P00751	0	1	1480.242	2959.477	-0.48	-0.00072	0
P00751	1	1	508.6351	1523.891	0.68	0.00035	63.22187
P00751	0	1	456.2074	1366.608	1.43	0.00065	16.23639
P00751	0	1	1452.265	2903.522	-0.58	-0.00084	0
P00751	0	1	690.6116	2759.425	1.02	0.00071	0
P00751	0	1	987.1656	2959.482	1.41	0.00139	1.56303
P00751	1	1	532.2913	1594.859	1.73	0.00092	13.60316
P00751	1	1	532.291	1594.858	1.04	0.00055	7.796257
P00751	0	0.9412	564.2938	1690.867	1.81	0.00102	35.69373
P00751	0	1	564.2949	1690.87	3.76	0.00212	9.032003
P00751	0	1	564.2948	1690.87	3.54	0.002	23.67762
P00751	0	0.973	408.5679	1223.689	2.14	0.00087	30.70784
P00751	1	0.8056	526.2996	1576.884	2.15	0.00113	27.09617
P00751	0	1	504.2412	1510.709	1	0.00051	4.939027
P00751	1	1	537.9826	1611.933	0.93	0.0005	15.40468
P00751	0	0.7609	580.3274	1159.648	3.1	0.0018	5.770638
P00751	0	0.963	638.6985	1914.081	2.67	0.0017	12.14157
P00751	0	1	957.5457	1914.084	4.3	0.00412	9.426233
P00751	0	0.9468	782.1772	3125.687	3.45	0.0027	7.368198

P00751	0	0.9925	1042.566	3125.684	2.5	0.00261	7.911452
P00751	0	0.973	456.6019	1367.791	1.86	0.00085	39.62049
P00751	0	1	456.6016	1367.79	1.19	0.00054	8.223703
P00751	0	0.8684	795.4335	1589.86	2.35	0.00186	6.455199
P00751	0	0.8571	795.4324	1589.857	0.96	0.00077	3.846954
P00751	0	1	456.6009	1367.788	-0.22	-0.0001	6.019475
P00751	0	0.9592	795.4333	1589.859	2.12	0.00168	4.24999
P00751	0	1	504.2414	1510.71	1.37	0.00069	47.33707
P00751	0	0.8519	636.3065	1271.606	2.51	0.0016	4.998684
P00751	0	0.9756	698.7278	2094.169	1.35	0.00094	4.181775
P00751	0	1	1047.589	2094.17	1.79	0.00187	0
P00751	0	0.9815	723.3821	1445.757	2.17	0.00157	0
P00751	0	0.875	664.5167	3318.555	2.21	0.00147	5.435781
P00751	0	1	756.3847	2267.14	0.12	0.00009	0
P00751	1	0.9355	394.9767	1576.885	2.57	0.00101	35.79996
P00751	0	1	686.9891	2058.953	1.7	0.00117	4.0786
P00751	0	0.7317	387.22	1159.645	1.25	0.00048	0
P00751	0	0.9273	597.3141	1193.621	3.24	0.00193	6.20182
P00751	0	0.8679	650.3694	1299.731	2.96	0.00192	7.51497
P00751	0	0.7647	533.5364	2131.124	1.66	0.00089	3.996831
P00751	1	1	797.9333	1594.859	1.66	0.00133	6.03642
P00751	0	0.8095	396.5959	1187.773	1.43	0.00057	16.52132
P00751	0	0.8448	795.433	1589.859	1.73	0.00138	4.13811
P00751	0	0.766	580.3259	1159.644	0.47	0.00027	8.886348
P00751	0	0.7778	580.3277	1159.648	3.63	0.0021	4.041078
P00751	0	0.6944	722.4201	1443.833	2.24	0.00161	18.94279
P00751	0	0.9524	472.5729	1415.704	-0.18	-0.00008	8.247107
P00751	0	0.7234	447.2507	1339.738	2.1	0.00094	13.06293
P00751	0	1	567.5424	2267.148	3.67	0.00208	8.801049
P00751	0	0.9412	472.5748	1415.71	3.71	0.00175	11.46249
P00751	0	1	698.7293	2094.173	3.44	0.0024	0
P00751	1	0.6176	526.2996	1576.884	2.27	0.00119	13.94493
P00751	0	0.9487	652.8472	1304.687	2.57	0.00167	0
P00751	0	0.3953	555.3447	1109.682	1.08	0.0006	19.87695
P00751	1	1	381.7286	1523.893	2.03	0.00077	48.87504
P00751	0	0.9623	597.3142	1193.621	3.44	0.00206	2.793723
P00751	0	1	638.9561	1914.854	3.39	0.00217	19.65021
P00751	0	1	472.5729	1415.704	-0.18	-0.00008	42.16849
P00751	0	0.7188	481.9486	1443.831	0.96	0.00046	9.288033
P00751	0	0.5745	555.3455	1109.684	2.62	0.00145	2.848327
P00751	0	1	632.8857	1264.764	2.09	0.00132	0
P00751	0	0.8478	627.8525	1254.698	3.56	0.00223	9.552679
P00751	1	0.8387	394.9766	1576.885	2.41	0.00095	15.53287
P00751	0	1	504.2412	1510.709	0.88	0.00045	17.72828
P00751	1	0.68	394.9763	1576.883	1.64	0.00065	6.605728
P00751	1	1	394.9765	1576.884	2.1	0.00083	19.84956
P00751	0	1	567.5418	2267.145	2.59	0.00147	38.75033
P00751	0	1	435.5667	1304.686	1.43	0.00062	34.62851
P00751	0	0.8333	521.9597	1563.865	-0.96	-0.0005	0
P00751	0	0.8889	482.2899	963.5726	1.3	0.00063	19.68523
P00751	1	0.9355	394.9765	1576.884	2.1	0.00083	22.0052
P00751	0	0.9583	522.3395	1043.672	2.36	0.00123	27.13828

P00751	0	1	435.567	1304.686	2	0.00087	16.81132
P00751	0	0.9535	795.4337	1589.86	2.58	0.00205	5.839256
P00751	0	0.6667	711.0465	2131.125	2.27	0.00161	40.5974
P00751	0	0.9167	521.9591	1563.863	-2.13	-0.00111	23.96799
P00751	0	0.8276	711.0472	2131.127	3.22	0.00228	15.48601
P00751	0	0.6	555.3458	1109.684	3.06	0.0017	31.50518
P00751	1	0.6923	394.976	1576.882	0.79	0.00031	60.94225
P00751	0	0.7619	599.6362	1796.894	1.94	0.00116	51.11195
P00751	0	0.9333	482.2907	963.574	2.82	0.00136	30.00072
P00751	0	0.7812	594.3909	1187.774	2.57	0.00153	7.733742
P00751	0	1	555.8005	1110.594	2.38	0.00132	38.21141
P00751	1	0.963	427.5829	1280.734	1.42	0.00061	15.70513
P00751	0	0.6098	555.3455	1109.684	2.62	0.00145	34.25141
P00751	0	0.9024	560.8347	1120.662	2.26	0.00127	2.26825
P00751	0	1	456.6009	1367.788	-0.35	-0.00016	60.15947
P00751	0	1	530.6243	1589.858	1.57	0.00083	8.884882
P00751	0	0.9333	482.2902	963.5731	1.87	0.0009	4.96421
P00751	1	0.9394	478.2647	1432.78	0.69	0.00033	18.21968
P00751	1	0.9394	478.2647	1432.78	0.69	0.00033	18.21968
P00751	0	1	530.6245	1589.859	1.91	0.00101	16.70636
P00751	0	1	632.8857	1264.764	1.99	0.00126	0
P00751	0	1	566.8311	1132.655	2.93	0.00166	40.41989
P00751	0	0.9643	530.6242	1589.858	1.34	0.00071	25.86917
P00751	0	0.2647	555.3464	1109.686	4.27	0.00237	22.60035
P00751	0	0.75	564.2934	1690.866	1.05	0.00059	52.05381
P00751	0	0.6579	580.3274	1159.648	3.1	0.0018	0
P00751	0	0.4583	426.256	1276.754	-0.1	-0.00004	0
P00751	0	0.8095	670.3723	1339.737	1.95	0.0013	15.45217
P00751	0	0.7826	564.2919	1690.861	-1.55	-0.00087	16.24173
P00751	0	0.5778	555.3444	1109.681	0.53	0.00029	5.686904
P00751	1	0.9524	532.2911	1594.859	1.39	0.00074	4.331127
P00751	0	1	396.5963	1187.774	2.43	0.00096	28.33862
P00751	0	0.9524	530.6251	1589.861	3.07	0.00163	35.17256
P00751	0	0.6875	580.3287	1159.65	5.42	0.00314	0
P00751	0	0.9615	684.3979	1367.789	-0.01	-0.00001	0
P00751	0	0.9512	723.3818	1445.756	1.66	0.0012	0
P00751	0	0.7308	530.6244	1589.859	1.68	0.00089	19.86831
P00751	0	0.7407	580.3283	1159.649	4.68	0.00271	22.41094
P00751	0	0.8571	722.4171	1443.827	-1.99	-0.00144	29.72464
P00751	0	0.9091	670.3724	1339.738	2.13	0.00143	45.99954
P00751	1	0.7333	383.4837	1530.913	2.29	0.00088	38.27532
P00751	0	0.75	482.2902	963.5732	1.93	0.00093	31.66308
P00751	0	1	580.3257	1159.644	0.26	0.00015	11.84299
P00751	0	0.5263	599.6369	1796.896	3.16	0.00189	23.02805
Q14624	0	0.7097	658.3845	1315.762	2.15	0.00141	4.113268
Q14624	0	0.7708	658.3846	1315.762	2.33	0.00153	35.02773
Q14624	0	0.375	665.6874	1995.048	0.83	0.00055	8.119791
Q14624	0	0.3288	998.0297	1995.052	3.07	0.00306	7.732388
Q14624	0	0.4545	665.6887	1995.051	2.76	0.00183	0
Q14624	0	0.96	671.02	2011.046	2.32	0.00156	8.135596
Q14624	0	1	546.3308	1636.978	1.98	0.00108	12.81206
Q14624	0	1	471.2581	1411.76	1.77	0.00083	24.70513

Q14624	0	1	546.3308	1636.978	1.98	0.00108	11.63118
Q14624	0	0.8393	746.9414	1492.876	1.98	0.00148	3.440629
Q14624	0	0.8393	746.9414	1492.876	1.98	0.00148	3.440629
Q14624	0	1	469.9257	1407.763	-1.5	-0.0007	5.428913
Q14624	0	0.8667	469.9266	1407.765	0.32	0.00015	4.595383
Q14624	0	1	471.2578	1411.759	1.25	0.00059	0.358704
Q14624	0	1	471.2575	1411.758	0.41	0.00019	10.37742
Q14624	0	0.9615	818.9929	1636.979	2.4	0.00196	9.2919
Q14624	0	1	546.3309	1636.978	2.09	0.00114	12.19857
Q14624	0	0.7826	419.2752	1255.811	1.77	0.00074	22.07832
Q14624	0	0.8462	419.2749	1255.81	0.96	0.0004	13.32171
Q14624	0	0.9048	419.2751	1255.811	1.55	0.00065	16.4774
Q14624	0	1	506.297	1516.876	2.55	0.00129	9.428925
Q14624	0	0.9355	469.9269	1407.766	0.97	0.00046	8.280146
Q14624	0	1	695.3903	2084.156	2.04	0.00142	20.23833
Q14624	0	1	521.7938	2084.153	0.63	0.00033	15.08535
Q14624	0	1	853.7694	2559.294	5.06	0.00432	9.482236
Q14624	0	1	853.7665	2559.285	1.7	0.00145	3.222097
Q14624	0	1	853.7659	2559.283	0.98	0.00084	2.260718
Q14624	0	1	521.7943	2084.155	1.45	0.00075	2.992401
Q14624	0	1	695.3904	2084.157	2.22	0.00154	3.472803
Q14624	0	1	805.7312	2415.179	0.16	0.00013	14.30131
Q14624	0	0.9487	521.7943	2084.155	1.45	0.00075	24.92296
Q14624	0	1	521.7944	2084.156	1.68	0.00088	30.26072
Q14624	0	0.9277	652.6903	1956.056	2.32	0.00151	4.608788
Q14624	0	0.8493	978.5319	1956.056	2.36	0.00231	3.452519
Q14624	0	1	652.6901	1956.056	1.95	0.00127	4.919642
Q14624	0	0.7358	478.281	1432.828	2.39	0.00114	11.84977
Q14624	0	0.9375	716.9171	1432.827	1.31	0.00094	6.107421
Q14624	0	0.725	478.2807	1432.828	1.81	0.00086	22.20437
Q14624	0	1	467.5686	1400.691	0.85	0.0004	5.109924
Q14624	0	0.9231	700.8499	1400.693	1.71	0.0012	3.438399
Q14624	0	1	467.5687	1400.692	1.05	0.00049	22.05987
Q14624	0	1	805.7322	2415.182	1.45	0.00117	3.124756
Q14624	0	0.918	746.9412	1492.875	1.74	0.0013	8.979471
Q14624	0	0.918	746.9412	1492.875	1.74	0.0013	8.979471
Q14624	0		498.2967	1492.876	1.98	0.00098	27.49652
Q14624	0	0.0476	498.2967	1492.876	1.98	0.00098	27.49652
Q14624	0	0.5667	471.2581	1411.76	1.83	0.00086	3.905124
Q14624	0	1	471.2582	1411.76	1.9	0.00089	10.49336
Q14624	0	0.9545	419.2753	1255.811	1.91	0.0008	18.31216
Q14624	0	1	776.7393	2328.203	1.22	0.00095	10.25939
Q14624	0	1	582.8076	2328.208	3.37	0.00196	2.16315
Q14624	0	1	776.7396	2328.204	1.61	0.00125	2.112437
Q14624	0	1	582.8066	2328.204	1.69	0.00098	2.080431
Q14624	0	1	466.4465	2328.203	1.22	0.00057	2.602665
Q14624	0	1	776.7395	2328.204	1.46	0.00113	2.405439
Q14624	0	1	582.806	2328.202	0.74	0.00043	2.54459
Q14624	0	1	546.7809	2184.102	1.58	0.00086	5.596146
Q14624	0	0.9697	679.6956	2037.072	2.16	0.00147	40.7143
Q14624	0	0.871	611.3776	1221.748	2.73	0.00167	6.566058
Q14624	0	0.9565	730.4354	1459.864	1.76	0.00128	7.97149

Q14624	0	0.9753	893.0194	1785.032	1.54	0.00137	10.76258
Q14624	0	0.8485	679.6955	2037.072	2.07	0.0014	21.9471
Q14624	0	0.9219	679.6955	2037.072	2.07	0.0014	7.640445
Q14624	0	0.9412	730.4355	1459.864	1.84	0.00134	12.77989
Q14624	0	0.6579	532.627	1595.866	1.99	0.00106	24.65868
Q14624	1	1	1239.607	2478.206	-1.13	-0.00139	0
Q14624	1	1	826.7421	2478.212	1.01	0.00084	3.265714
Q14624	0	0.746	596.3785	1191.75	2.42	0.00144	16.77308
Q14624	0	1	1183.959	3549.864	1.55	0.00184	0
Q14624	0	1	888.2216	3549.864	1.81	0.0016	4.160551
Q14624	0	1	786.9382	1572.869	1.76	0.00138	6.829504
Q14624	0	1	532.6267	1595.866	1.53	0.00081	45.11316
Q14624	0	0.7561	524.9615	1572.87	2.21	0.00116	7.873148
Q14624	0	0.9759	786.9382	1572.869	1.68	0.00132	5.99601
Q14624	0	0.8448	786.9389	1572.871	2.61	0.00205	9.835069
Q14624	0	0.9508	532.6276	1595.868	3.14	0.00167	11.59124
Q14624	1	0.766	666.3734	1997.106	1.47	0.00098	11.58917
Q14624	1	0.9091	500.0312	1997.103	0.03	0.00001	12.73623
Q14624	1	1	656.718	1968.139	2.07	0.00136	18.66892
Q14624	0	0.8696	532.6267	1595.866	1.53	0.00081	25.25371
Q14624	0	0.9483	532.6265	1595.865	1.07	0.00057	2.432889
Q14624	0	0.9783	798.4368	1595.866	2.04	0.00162	6.339597
Q14624	1	0.7755	666.3746	1997.109	3.3	0.0022	18.62988
Q14624	1	0.814	500.032	1997.106	1.8	0.0009	24.28378
Q14624	0	1	532.6272	1595.867	2.45	0.0013	9.502354
Q14624	0	1	532.6272	1595.867	2.45	0.0013	14.90127
Q14624	0	0.9677	524.9609	1572.868	1.17	0.00061	19.60567
Q14624	0	0.8	668.4295	1335.852	2.06	0.00137	6.727183
Q14624	0	0.9	583.3306	1165.654	4.25	0.00248	4.674911
Q14624	0	0.8889	583.3295	1165.652	2.47	0.00144	8.092896
Q14624	0	1	778.8792	3112.495	2.23	0.00173	4.01907
Q14624	0	1	1038.171	3112.497	3	0.00311	2.157008
Q14624	0	0.88	503.9585	1509.861	1.35	0.00068	18.8935
Q14624	0	0.96	755.4349	1509.862	2.37	0.00179	10.50709
Q14624	0	0.6667	532.6275	1595.868	3.02	0.00161	45.69793
Q14624	0	0.6176	532.6275	1595.868	3.02	0.00161	42.15372
Q14624	0	1	778.8787	3112.493	1.6	0.00124	6.993337
Q14624	1	1	906.5217	2717.551	0.97	0.00088	9.581242
Q14624	0	1	913.1631	2737.475	0.26	0.00023	1.695897
Q14624	0	1	685.124	2737.474	0.1	0.00007	4.204047
Q14624	0	0.5357	532.6257	1595.863	-0.31	-0.00016	52.55064
Q14624	0	1	777.1648	3105.637	1.18	0.00092	3.316844
Q14624	0	0.8837	410.2107	1228.617	1.92	0.00079	0
Q14624	0	1	1035.885	3105.64	2.19	0.00226	1.99959
Q14624	0	1	1038.17	3112.495	2.17	0.00225	
Q14624	0	0.88	611.3773	1221.747	2.33	0.00142	4.914336
Q14624	0	1	467.5689	1400.692	1.38	0.00064	52.34058
Q14624	0	1	471.258	1411.759	1.58	0.00074	64.20599
Q14624	0	0.8889	419.2746	1255.809	0.38	0.00016	70.10477
Q14624	0	0.8611	498.2974	1492.878	3.39	0.00168	18.18522
Q14624	0	1	471.2575	1411.758	0.47	0.00022	71.95668
Q14624	0	0.8913	786.9399	1572.872	3.85	0.00303	2.118155

Q14624	0	1	521.7943	2084.155	1.45	0.00075	1.620498
Q14624	0	1	421.8931	1263.665	1.72	0.00073	1.864329
Q14624	0	0.7759	539.3256	1077.644	1.4	0.00076	8.271564
Q14624	0	0.6316	487.2923	1459.862	0.91	0.00044	8.898244
Q14624	0	0.8649	536.8107	1072.614	2.03	0.00109	22.02081
Q14624	0	0.8611	498.2974	1492.878	3.39	0.00168	18.18522
Q14624	0	0.9	503.9594	1509.864	3.17	0.00159	47.41573
Q14624	0	0.8095	469.927	1407.767	1.3	0.00061	23.12851
Q14624	0	1	546.3294	1636.974	-0.59	-0.00032	7.700274
Q14624	0	0.8372	389.2219	1165.651	1.83	0.00071	4.971799
Q14624	0	0.5263	595.6823	1785.032	1.9	0.00113	8.592519
Q14624	0	0.9388	591.3278	1181.648	3.74	0.00221	6.210682
Q14624	0	1	485.7694	1940.056	3.01	0.00146	35.91664
Q14624	0	1	467.5691	1400.693	1.77	0.00083	10.19489
Q14624	0	0.9677	509.2912	1525.859	3.37	0.00171	67.67847
Q14624	0	0.8667	475.2587	1423.761	1.28	0.00061	5.333531
Q14624	0	0.2353	439.2584	1315.761	1.21	0.00053	0
Q14624	0	0.8491	509.2781	1017.549	1.31	0.00067	0
Q14624	0	0.1935	458.2614	1372.77	-0.73	-0.00034	0
Q14624	0	0.9024	716.9171	1432.827	1.39	0.001	6.125665
Q14624	0	0.7949	755.4349	1509.863	2.45	0.00185	8.199357
Q14624	0	0.8085	539.3254	1077.644	1.06	0.00057	25.46513
Q14624	0	1	409.9997	1636.977	1.49	0.00061	21.0881
Q14624	0	0.1333	498.2969	1492.876	2.4	0.0012	1.978593
Q14624	0	0.6765	658.3835	1315.76	0.57	0.00037	8.755822
Q14624	0	0.7619	652.6892	1956.053	0.54	0.00035	25.586
Q14624	1	0.6818	397.9319	1191.781	2.18	0.00087	28.94779
Q14624	0	0.6842	798.4371	1595.867	2.34	0.00187	4.637139
Q14624	0	0.913	469.9271	1407.767	1.37	0.00064	9.625572
Q14624	0	0.9	484.5925	1451.763	1.29	0.00062	64.89587
Q14624	0	0.9032	394.5537	1181.646	2.18	0.00086	13.45693
Q14624	0	1	582.8055	2328.2	-0.1	-0.00006	9.428074
Q14624	1	1	416.5948	1247.77	1.87	0.00078	31.57208
Q14624	0	1	485.7682	1940.051	0.49	0.00024	33.45097
Q14624	0	0.8545	509.2785	1017.55	2.09	0.00106	0
Q14624	0	0.6207	487.2925	1459.863	1.35	0.00066	12.06372
Q14624	0	0.6364	668.4291	1335.851	1.51	0.00101	18.77147
Q14624	0	0.75	419.2763	1255.814	4.24	0.00178	26.44579
Q14624	0	0.898	536.8098	1072.612	0.44	0.00023	3.065197
Q14624	0	0.7317	407.9203	1221.746	1.47	0.0006	7.850217
Q14624	0	1	1280.146	2559.285	1.85	0.00236	0
Q14624	0	0.8889	704.3878	1407.768	2.59	0.00182	18.75771
Q14624	0	0.8039	509.2785	1017.55	2.09	0.00106	12.98051
Q14624	0	1	503.958	1509.859	0.32	0.00016	13.33152
Q14624	0	1	506.2963	1516.874	1.1	0.00056	10.1642
Q14624	0	1	472.8998	1416.685	-0.2	-0.00009	66.51241
Q14624	0	0.6818	387.8886	1161.651	1.47	0.00057	25.12229
Q14624	0	1	379.9735	1516.872	-0.24	-0.00009	0
Q14624	0	1	464.7592	928.511	1.31	0.00061	10.03007
Q14624	0	0.8824	532.6263	1595.864	0.73	0.00039	25.12149
Q14624	0	0.875	546.3307	1636.978	1.76	0.00096	27.2962
Q14624	0	1	778.8787	3112.493	1.6	0.00124	46.57545

Q14624	0	0.6538	509.2899	1525.855	0.91	0.00046	25.1102
Q14624	0	0.75	387.8888	1161.652	1.94	0.00075	12.4556
Q14624	0	1	466.4466	2328.204	1.48	0.00069	0
Q14624	0	0.8621	786.9371	1572.867	0.28	0.00022	6.71518
Q14624	0	0.5385	387.8893	1161.653	3.12	0.00121	53.24963
Q14624	0	1	536.8107	1072.614	2.14	0.00115	25.961
Q14624	0	0.9167	532.6271	1595.867	2.22	0.00118	13.15745
Q14624	0	1	417.6367	2084.154	1.12	0.00047	0
Q14624	0	0.8182	685.0264	2053.065	1.02	0.0007	0
Q14624	0	0.6944	565.8273	1130.647	2.28	0.00129	7.318781
Q14624	0	1	419.5361	1256.594	4.43	0.00186	27.67778
Q14624	0	1	467.5691	1400.693	1.83	0.00086	59.47414
Q14624	0	0.7429	565.8274	1130.648	2.39	0.00135	24.7302
Q14624	0	1	421.8933	1263.665	2.16	0.00091	25.3494
Q14624	0	0.7333	658.3851	1315.763	2.98	0.00196	12.49684
Q14624	0	0.72	445.9554	1335.852	1.96	0.00087	9.324774
Q14624	1	0.1818	497.9888	1491.952	1.11	0.00055	0
Q14624	0	1	410.0009	1636.982	4.32	0.00177	27.01279
Q14624	0	0.7	503.9586	1509.861	1.59	0.0008	23.09921
Q14624	0	1	464.7593	928.5112	1.5	0.0007	28.10948
Q14624	0	1	410	1636.978	2.08	0.00085	28.79648
Q14624	0	0.8857	536.813	1072.619	6.35	0.00341	28.34657
Q14624	0	0.8636	679.6954	2037.072	1.98	0.00134	2.856497
Q14624	0	0.9375	410.0002	1636.979	2.68	0.0011	28.45953
Q14624	0	0.9268	556.3577	1111.708	1.14	0.00063	0
Q14624	0	0.8125	469.9278	1407.769	2.93	0.00137	16.69924
Q14624	0	1	475.2594	1423.764	2.76	0.00131	24.79189
Q14624	0	1	472.8998	1416.685	-0.26	-0.00012	72.11679
Q14624	0	1	409.9997	1636.977	1.34	0.00055	13.88865
Q14624	0	0.7647	387.8888	1161.652	1.94	0.00075	42.12729
Q14624	0	1	478.2814	1432.83	3.28	0.00157	46.48137
Q14624	0	0.8049	509.2777	1017.548	0.53	0.00027	5.478704
Q14624	0	1	472.9012	1416.689	2.78	0.00131	38.26564
Q14624	0	0.6	419.2752	1255.811	1.69	0.00071	31.67081
Q14624	0	1	419.2753	1255.811	2.06	0.00086	40.696
Q14624	0	0.9333	581.3295	1161.652	1.71	0.001	24.69508
Q14624	0	0.4286	532.6259	1595.863	-0.08	-0.00004	21.58272
Q14624	0	1	472.9008	1416.688	1.94	0.00091	55.13436
Q14624	0	1	475.2595	1423.764	3.01	0.00143	0
Q14624	0	0.4286	524.9604	1572.867	0.12	0.00006	0
Q14624	0	1	475.2595	1423.764	3.01	0.00143	32.8395
Q14624	0	1	498.2966	1492.875	1.85	0.00092	46.3236
Q14624	0	0.875	387.8884	1161.651	0.91	0.00035	46.10957
Q14624	0	1	419.5352	1256.591	2.32	0.00097	10.86871
Q14624	0	1	509.2792	1017.551	3.47	0.00176	9.521961
Q14624	0	0.8571	978.5327	1956.058	3.23	0.00316	2.977613
Q14624	0	0.8077	726.3837	1451.76	-0.7	-0.00051	0
Q14624	0	0.9259	611.3779	1221.748	3.23	0.00197	5.10031
Q14624	0	1	536.8119	1072.617	4.3	0.00231	17.07041
P08603; Q0	0	0.971	755.3972	1509.787	1.31	0.00099	3.569047
P08603; Q0	0	1	503.9338	1509.787	1.18	0.0006	3.008636
P08603	0	0.9024	562.0018	1683.991	1.44	0.00081	0

P08603	0	0.9655	562.0021	1683.992	1.98	0.00111	7.975345
P08603	0	1	562.0022	1683.992	2.2	0.00124	8.347581
P08603	0	1	842.4987	1683.99	1.01	0.00085	3.816402
P08603	0	1	562.0022	1683.992	2.2	0.00124	9.283036
P08603	0	1	611.9676	1833.888	0.89	0.00054	8.704879
P08603	0	0.925	467.2519	1399.741	1.19	0.00056	37.38911
P08603	0	0.9123	700.3748	1399.742	1.99	0.00139	4.915659
P08603	0	0.8387	467.2518	1399.741	1	0.00047	33.73383
P08603	0	0.9722	467.2519	1399.741	1.13	0.00053	7.840226
P08603	0	0.9783	723.369	2890.454	6.7	0.00484	14.22767
P08603	0	0.975	964.1514	2890.44	1.62	0.00156	4.037967
P08603	0	0.8846	964.1523	2890.442	2.63	0.00253	0
P08603	0	1	813.7198	2439.145	1.55	0.00126	4.398508
P08603	0	1	813.7191	2439.143	0.73	0.00059	67.18699
P08603	0	1	623.307	1245.607	1.72	0.00107	0
P08603	0	1	604.3511	1207.695	2.87	0.00173	8.337082
P08603	0	1	403.2355	1207.692	0.43	0.00017	9.428444
P08603	0	0.9333	454.7475	1815.968	0.82	0.00037	9.918261
P08603	0	0.9538	605.9952	1815.971	2.54	0.00154	7.482445
P08603	0	0.7333	454.7476	1815.969	1.16	0.00052	0
P08603	0	1	900.7938	2700.367	7.58	0.00682	31.01655
P08603	0	1	900.7893	2700.353	2.56	0.00231	4.1572
P08603	0	1	675.8434	2700.352	2.11	0.00142	2.242074
P08603	0	1	900.7889	2700.352	2.22	0.002	3.391243
P08603	1	1	524.3164	1570.935	2.49	0.0013	8.697868
P08603	1	1	524.3164	1570.934	2.37	0.00124	23.56487
P08603	1	0.5758	393.4884	1570.932	0.73	0.00029	7.132245
Q02985; P0	0	0.7879	474.622	1421.851	0.47	0.00022	9.169104
Q02985; P0	0	0.7391	474.6225	1421.853	1.5	0.00071	24.49958
Q02985; P0	0	0.8868	711.4302	1421.853	1.66	0.00118	7.879076
Q02985; P0	0	0.7273	474.6222	1421.852	0.92	0.00044	15.33165
P08603	0	0.8235	467.2524	1399.743	2.37	0.00111	0
P08603	0	0.8723	516.6213	1547.849	-0.08	-0.00004	7.515931
P08603	0	0.8308	774.4289	1547.851	0.72	0.00056	6.97052
P08603	0	0.8966	516.622	1547.851	1.22	0.00063	6.515483
P08603	0	1	727.3569	1453.707	1.22	0.00089	20.25361
P08603	0	1	463.9081	1389.71	2.27	0.00105	12.01466
P08603	0	1	695.3585	1389.71	2.24	0.00156	4.24159
P08603	0	0.4468	766.3857	2297.143	1.35	0.00104	5.645868
P08603	0	1	769.714	2307.127	3.39	0.00261	2.674431
P08603	0	1	777.3351	2329.991	-1.49	-0.00116	13.0928
P08603	0	1	725.6581	2174.96	1.26	0.00092	8.744031
P08603	0	1	617.2983	2466.171	3.3	0.00203	63.56646
P08603	0	0.9355	822.7285	2466.171	3.1	0.00255	13.01577
P08603	0	0.9583	654.3518	2614.385	4.99	0.00326	15.28613
P08603	1	1	637.8068	2548.205	1.5	0.00095	8.906884
P08603	0	0.8846	453.2239	1357.657	1.48	0.00067	41.73682
P08603	0	0.9362	481.919	1443.742	2.28	0.0011	12.34117
P08603	0	0.9	722.3746	1443.742	1.86	0.00135	6.220034
P08603	1	1	637.8069	2548.206	1.59	0.00102	5.408194
P08603	0	0.8684	517.6019	1550.791	2.27	0.00118	11.88309
P08603	0	1	775.8986	1550.79	1.5	0.00116	30.11355

P08603	0	1	648.3215	1942.95	2.15	0.00139	27.1526
P08603	0	1	621.7763	1242.545	2.74	0.0017	33.03737
P08603	0	1	621.7763	1242.545	2.65	0.00164	38.68909
P08603	0	1	693.8264	1386.645	1.04	0.00072	3.189405
P08603	0	1	693.8268	1386.646	1.75	0.00121	3.335537
P08603	0	1	741.8763	1482.745	1.84	0.00136	0
P08603	0	1	494.9194	1482.744	0.66	0.00032	8.316276
P08603	0	0.975	702.0218	2104.051	1.79	0.00126	4.786982
P08603	0	1	822.7271	2466.167	1.4	0.00115	5.072587
P08603	0	1	494.9195	1482.744	0.9	0.00045	31.73177
P08603	0	1	722.368	2165.09	5.47	0.00395	20.86158
P08603	0	1	494.9208	1482.748	3.37	0.00167	26.46812
P08603	0	0.7308	675.8431	2700.351	1.65	0.00112	4.018898
P08603	0	1	650.3234	1299.64	1.89	0.00123	37.06493
P08603	0	1	623.3316	1245.656	4.26	0.00266	15.46071
P08603	0	1	494.9205	1482.747	2.82	0.00139	19.47134
P08603	0	0.9667	648.0106	1942.017	1.71	0.00111	10.5841
P08603	0	1	667.321	1333.635	1.59	0.00106	3.304256
P08603	0	0.875	436.902	1308.692	2.39	0.00104	69.20187
P08603	0	0.9697	462.8868	1386.646	1.3	0.0006	9.885628
P08603; Q0	0	1	683.3453	1365.683	0.21	0.00014	12.80095
P08603	0	1	552.7719	1104.536	0.94	0.00052	20.77806
P08603	0	0.8182	702.0217	2104.051	1.7	0.0012	24.7922
P08603	0	0.8857	467.2535	1399.746	4.73	0.00221	0
P08603	0	1	741.8782	1482.749	4.31	0.00319	0
P08603	0	0.9375	675.8435	2700.352	2.2	0.00148	70.66231
P08603	0	1	631.8281	1262.649	3.82	0.00241	22.99215
P08603	0	0.5714	467.2525	1399.743	2.5	0.00117	11.81219
P08603	0	1	624.8234	1248.64	1.7	0.00106	8.022172
P08603	0	0.963	623.3304	1245.654	2.4	0.0015	6.733574
P08603	0	1	486.2596	1942.016	1.33	0.00064	27.70929
P08603	0	0.75	648.3214	1942.95	1.86	0.00121	4.978912
P08603	0	1	595.2709	1189.535	3.33	0.00198	48.93077
P08603	0	1	425.8789	1275.622	1.76	0.00075	43.38062
P08603	0	0.8537	604.3533	1207.699	6.51	0.00393	33.56851
P08603	0	0.9688	431.5703	1292.696	2.07	0.00089	8.20663
P08603	1	0.875	476.2829	1426.834	3.82	0.00182	28.02704
P08603	1	0.875	476.2829	1426.834	3.82	0.00182	28.02704
P08603	0	1	485.2408	1453.708	2.05	0.00099	18.50875
P08603	0	1	445.2164	1333.635	1.53	0.00068	22.57742
P08603	0	1	421.5528	1262.644	-0.18	-0.00008	53.92863
P08603	0	1	725.6587	2174.962	2.1	0.00153	0
P08603	0	1	431.5708	1292.698	3.35	0.00144	7.344255
P08603	0	1	617.2975	2466.168	2.01	0.00124	20.14208
P08603	0	0.8333	964.1525	2890.443	2.76	0.00266	0
P08603	0	0.875	481.9184	1443.741	1.07	0.00052	4.692261
P08603	0	1	458.5552	1373.651	0.79	0.00036	6.832651
P08603	0	1	425.8791	1275.623	2.19	0.00093	35.40481
P08603; Q0	0	0.9231	509.265	1525.78	0.14	0.00007	0
P08603; Q0	0	1	429.2259	1285.663	0.51	0.00022	17.08717
P08603	0	1	900.7936	2700.366	7.44	0.0067	8.963806
P08603	0	1	723.3629	2890.43	-1.75	-0.00126	30.87895

P08603	0	0.9583	559.7771	1118.547	4.32	0.00242	0
P08603	1	0.625	584.7961	2336.163	2.3	0.00134	4.327932
P08603	0	0.8293	592.3087	1183.61	4.08	0.00241	39.06714
P08603	0	0.7	824.0966	2470.275	1.95	0.00161	15.4316
P08603	0	1	669.8248	1338.642	1.23	0.00082	0
P08603	0	0.4211	654.3507	2614.381	3.31	0.00216	9.29794
P08603	1	1	785.9705	1570.934	1.84	0.00144	6.003601
P08603	0	1	393.2052	1177.601	-0.53	-0.00021	5.701189
P08603	0	1	393.2057	1177.603	0.79	0.00031	10.25868
P08603	0	1	621.7745	1242.542	-0.11	-0.00007	0
P08603	0	0.7778	611.9677	1833.888	0.99	0.0006	11.38106
P08603	0	1	458.5556	1373.652	1.65	0.00076	65.87532
P08603	0	1	1220.076	2439.146	1.86	0.00227	0
P08603	0	1	675.8447	2700.357	4	0.0027	0
P08603	1	1	637.8087	2548.213	4.37	0.00279	22.67767
P08603; QC	0	0.5	755.398	1509.789	2.28	0.00172	9.689037
P08603	0	0.6923	562.0028	1683.994	3.29	0.00185	18.94243
P08603	0	0.5882	421.752	1683.986	-1.18	-0.0005	66.43376
P02787	0	1	622.6262	1865.864	1.23	0.00077	0
P02787	0	1	622.6264	1865.865	1.53	0.00095	9.504395
P02787	0	1	933.4363	1865.865	1.93	0.0018	10.24657
P02787	0	1	447.2203	1339.646	1.39	0.00062	24.02737
P02787	0	1	561.9439	1683.817	2.46	0.00138	6.094108
P02787	0	1	749.8709	1498.735	1.16	0.00087	2.453113
P02787	1	1	543.288	1627.849	1.78	0.00097	21.66813
P02787	0	1	447.2205	1339.647	1.73	0.00077	35.3155
P02787	0	1	459.243	1833.95	-0.57	-0.00026	5.927568
P02787	1	1	537.9562	1611.854	1.52	0.00082	40.39764
P02787	0	1	391.2204	1561.86	1.35	0.00053	65.95879
P02787	0	1	521.2916	1561.86	1.71	0.00089	24.66203
P02787	0	0.8837	541.6174	1622.838	0.42	0.00023	37.237
P02787	0	1	811.9231	1622.839	1.26	0.00102	3.137086
P02787	0	1	559.2696	1675.794	2.52	0.00141	15.28788
P02787	0	0.95	522.2847	1564.839	2.08	0.00109	6.832556
P02787	0	0.9024	522.2844	1564.839	1.5	0.00078	14.73976
P02787	0	0.9615	521.2916	1561.86	1.71	0.00089	8.501781
P02787	0	0.8393	640.0125	1918.023	1.54	0.00099	25.1615
P02787	0	0.8594	640.0125	1918.023	1.54	0.00099	4.99184
P02787	0	0.88	523.5927	1568.763	1.08	0.00057	12.22315
P02787	0	0.8444	605.3244	1209.642	2.31	0.0014	31.93626
P02787	0	1	535.9366	1605.795	1.16	0.00062	19.7394
P02787	0	0.9268	622.678	1866.019	0.43	0.00027	5.828174
P02787	0	0.9846	622.6789	1866.022	1.9	0.00118	8.714314
P02787	0	0.9556	633.8505	1266.694	0.35	0.00022	33.82518
P02787	0	0.8113	644.8563	1288.705	1.97	0.00127	4.36728
P02787	1	1	675.9985	2025.981	1.41	0.00095	2.664398
P02787	0	0.7838	590.3154	2358.24	2.83	0.00167	4.696531
P02787	1	1	675.9993	2025.983	2.58	0.00175	23.73982
P02787	0	1	568.6486	1703.931	4.17	0.00237	23.50902
P02787	0	1	714.3408	1427.674	2.22	0.00159	7.965966
P02787	0	0.7255	513.2756	1537.812	1.51	0.00077	8.013424
P02787	0	0.9481	769.4105	1537.814	2.35	0.00181	13.05946

P02787	0	0.9815	469.5011	1874.983	2.23	0.00104	43.21069
P02787	0	1	459.2439	1833.954	1.43	0.00066	7.96967
P02787	0	1	459.2437	1833.953	1.1	0.0005	5.796868
P02787	0	1	535.9371	1605.797	2.07	0.00111	27.5948
P02787	0	1	469.5011	1874.982	2.1	0.00098	18.22964
P02787	0	1	469.5011	1874.982	2.1	0.00098	12.0648
P02787	0	1	563.2846	2250.116	2.55	0.00143	10.58652
P02787	0	1	750.7097	2250.115	1.77	0.00133	6.019434
P02787	0	1	750.7096	2250.114	1.61	0.00121	16.18424
P02787	0	1	563.2839	2250.114	1.47	0.00082	9.478635
P02787	0	1	750.7094	2250.114	1.28	0.00096	4.737927
P02787	0	1	563.2847	2250.117	2.88	0.00162	5.629054
P02787	0	1	750.7093	2250.113	1.2	0.0009	10.09671
P02787	0	0.8837	581.8284	1162.65	3.2	0.00186	11.71037
P02787	0	1	563.2846	2250.116	2.55	0.00143	7.735673
P02787	0	1	750.7092	2250.113	1.04	0.00078	5.89638
P02787	0	1	563.2847	2250.117	2.88	0.00162	9.054694
P02787	0	1	772.4049	2315.2	1.1	0.00085	1.781895
P02787	0	1	1158.104	2315.202	1.84	0.00213	0
P02787	0	1	563.2844	2250.116	2.33	0.00131	7.555831
P02787	0	1	485.6028	1454.794	-1.03	-0.0005	4.329264
P02787	0	1	459.2431	1833.95	-0.3	-0.00014	22.65432
P02787	0	1	563.2847	2250.117	2.77	0.00156	4.853487
P02787	0	0.9697	750.7097	2250.114	1.69	0.00127	6.579433
P02787	0	1	485.6036	1454.796	0.61	0.00029	4.794394
P02787	0	1	750.7091	2250.113	0.96	0.00072	9.400943
P02787	1	1	473.5981	1418.78	0.72	0.00034	33.83379
P02787	0	0.9388	727.902	1454.797	0.97	0.00071	0
P02787	0	1	670.325	1339.643	-1.47	-0.00099	15.96091
P02787	0	1	622.6259	1865.863	0.74	0.00046	5.040756
P02787	0	1	399.1865	1195.545	2	0.0008	35.61543
P02787	0	1	670.3281	1339.649	3.17	0.00213	3.322643
P02787	0	0.9091	571.2951	1141.583	3.27	0.00186	4.460028
P02787	0	1	485.6043	1454.798	1.99	0.00097	6.109419
P02787	0	0.84	554.8221	1108.637	2.29	0.00127	0
P02787	0	1	485.6043	1454.798	1.99	0.00097	36.76691
P02787	0	0.9444	521.2915	1561.86	1.48	0.00077	10.30852
P02787	0	0.093	516.2631	1031.519	1.64	0.00085	3.208266
P02787	0	0.9787	770.3615	1539.716	3.22	0.00248	0
P02787	0	1	402.2049	1605.798	2.82	0.00113	30.23007
P02787	0	0.9355	852.4713	1703.935	6.65	0.00567	0
P02787	0	0.8478	605.3264	1209.645	5.54	0.00335	0
P02787	1	0.92	423.9119	1269.721	1.08	0.00046	27.91417
P02787	0	1	485.6035	1454.796	0.42	0.0002	35.38224
P02787	0	1	473.2582	1417.76	3.03	0.00143	42.02169
P02787	1	1	604.0092	1810.013	3.34	0.00201	16.17004
P02787	0	0.8649	561.7996	1122.592	0.62	0.00035	0
P02787	0	0.8889	430.2397	1288.705	1.46	0.00063	6.041776
P02787	0	1	391.9646	1564.837	0.32	0.00013	32.35226
P02787	1	1	471.9307	1413.778	0.98	0.00046	8.847574
P02787	0	0.8421	605.3257	1209.644	4.43	0.00268	0
P02787	0	0.75	671.9962	2013.974	1.39	0.00094	11.34497

P02787	0	0.8788	605.3242	1209.641	1.9	0.00115	1.507185
P02787	0	0.9032	739.871	1478.735	-0.1	-0.00007	21.13222
P02787	0	1	512.3088	1023.61	2.53	0.00129	36.36041
P02787	0	0.9333	677.82	1354.633	1.38	0.00094	8.539092
P02787	0	1	533.2728	1065.538	1.49	0.00079	0
P02787	0	0.9091	541.6179	1622.839	1.32	0.00072	14.47225
P02787	0	0.6667	447.2231	1339.655	0.29	0.00013	5.948778
P02787	0	0.9706	512.3091	1023.611	3.12	0.0016	21.13083
P02787	0	0.9524	671.9953	2013.971	0.12	0.00008	0
P02787	0	0.9375	485.6043	1454.798	2.05	0.001	44.97449
P02787	0	1	521.2938	1561.867	5.81	0.00303	17.00948
P02787	1	0.913	675.9959	2025.973	-2.39	-0.00161	22.10481
P02787	0	0.8261	702.676	2106.013	2.27	0.0016	5.847476
P02787	0	1	512.3081	1023.609	1.21	0.00062	55.06925
P02787	0	0.8333	535.9362	1605.794	0.48	0.00025	78.04005
P02787	0	0.3333	422.9039	1266.697	3.18	0.00134	62.54387
P02751	0	0.8684	686.3742	2057.108	4.07	0.00279	5.326117
P02751	0	0.9	700.3784	2099.121	4.98	0.00348	10.42075
P02751	0	0.6667	527.6313	1580.879	1.22	0.00064	9.309264
P02751	0	1	535.9699	1605.895	1.36	0.00073	38.33139
P02751	0	0.7679	802.4449	2405.32	0.05	0.00004	5.803614
P02751	0	0.907	525.9559	1575.853	1.28	0.00067	8.228604
P02751	0	0.9737	525.9561	1575.854	1.63	0.00086	21.18682
P02751	0	0.7308	750.4022	1499.797	-0.19	-0.00014	12.56848
P02751	0	1	880.0772	2638.217	-0.35	-0.00031	86.89754
P02751	0	1	827.3877	2480.149	2.08	0.00172	6.165277
P02751	0	1	847.4017	2540.191	0.96	0.00081	9.204536
P02751	0	1	797.4309	3186.702	3.14	0.00251	36.04278
P02751	0	0.9118	588.6808	1764.028	2.89	0.0017	15.90574
P02751	0	0.9655	829.049	2485.132	1.24	0.00103	4.690374
P02751	0	0.8824	651.3449	1952.02	6.76	0.0044	22.98104
P02751	0	1	622.0371	2485.126	-1.15	-0.00071	18.22258
P02751	0	1	619.2675	1855.788	2.74	0.0017	14.78157
P02751	0	1	515.9291	1545.773	3.22	0.00166	0
P02751	0	0.975	456.2575	1366.758	0.87	0.00039	33.47849
P02751	0	0.8235	521.9442	1563.818	1.47	0.00077	6.625615
P02751	0	1	686.3699	2057.095	-2.16	-0.00148	21.71024
P02751	0	1	627.3427	1880.014	1.06	0.00067	17.1145
P02751	0	0.9524	451.8868	1353.646	-0.05	-0.00002	64.70397
P02751	0	0.8444	522.8189	1044.631	1.33	0.00069	15.40846
P02751	0	1	893.3959	1785.785	0.4	0.00036	0
P02751	0	0.9714	819.4238	2456.257	0.9	0.00073	9.832249
P02751	0	0.9211	453.9307	1359.778	0.97	0.00044	51.20701
P02751	0	0.9375	584.3232	1750.955	1.71	0.001	39.0821
P02751	0	0.9688	761.4058	2282.203	3.52	0.00268	40.41438
P02751	0	0.963	599.3019	1795.891	3.26	0.00195	2.837538
P02751	0	1	522.8188	1044.63	1.09	0.00057	21.77069
P02751	0	1	440.2506	1318.737	1.09	0.00048	18.58456
P02751	0	1	847.4052	2540.201	4.99	0.00423	10.75723
P02751	0	0.9167	539.7941	1078.581	1.34	0.00072	4.141369
P02751	0	0.9545	690.388	2758.53	2.63	0.00182	21.19614
P02751	0	1	470.7596	1880.017	2.68	0.00126	4.804381

P02751	0	0.9091	438.4943	1750.955	1.73	0.00076	35.49263
P02751	0	0.9333	576.2799	1151.552	0.91	0.00052	26.79073
P02751	0	1	508.7927	1016.578	2.36	0.0012	16.47688
P02751	0	1	624.3151	1870.931	3.76	0.00234	23.51587
P02751	0	1	625.627	1874.866	4.46	0.00279	11.34726
P02751	0	1	677.3266	1353.646	-0.02	-0.00001	0
P02751	0	1	624.3136	1870.926	1.31	0.00082	75.56871
P02751	0	1	500.605	1499.8	1.96	0.00098	5.081048
P02751	0	1	508.7925	1016.578	2	0.00102	14.37678
P02751	1	1	632.6921	1896.062	1.87	0.00118	38.25325
P02751	0	0.9062	627.8275	1254.648	2.48	0.00156	0
P02751	0	0.8333	614.8196	2456.257	0.82	0.0005	12.91154
P02751	0	0.5185	790.9413	1580.875	-1.32	-0.00105	61.37672
P02751	0	0.8333	515.9277	1545.769	0.49	0.00025	20.01289
P02751	0	0.5	802.4456	2405.322	0.97	0.00078	0
P02751	0	0.7805	467.7429	934.4786	1.16	0.00054	13.37445
P02751	0	0.5217	540.6455	1619.922	0.82	0.00044	11.20854
P02751	0	0.5217	540.6455	1619.922	0.82	0.00044	11.20854
P02751	0	0.7857	543.2712	1627.799	-1.67	-0.00091	3.663336
P02751	0	1	624.3139	1870.927	1.8	0.00112	0
P02751	0	0.871	539.7943	1078.581	1.56	0.00084	24.39417
P19823	0	1	650.3271	1948.967	1.72	0.00112	9.94997
P19823	0	1	650.3278	1948.969	2.85	0.00185	8.052189
P19823	0	1	487.997	1948.966	1.53	0.00074	4.265851
P19823	0	1	974.9878	1948.968	2.54	0.00248	2.881969
P19823	0	1	650.3263	1948.964	0.5	0.00033	4.392503
P19823	0	0.8857	487.9972	1948.967	1.78	0.00087	0
P19823	0	0.871	650.3254	1948.962	-0.91	-0.00059	0
P19823	0	0.898	892.7506	2676.237	1	0.00089	9.276464
P19823	0	0.9756	892.7502	2676.236	0.59	0.00052	6.30511
P19823	0	0.9896	892.7508	2676.238	1.2	0.00107	3.325036
P19823	1	1	537.3124	1609.923	2.7	0.00145	10.59046
P19823	0	0.9474	791.9324	1582.858	1.8	0.00142	28.17818
P19823	1	1	537.3124	1609.923	2.7	0.00145	10.59046
P19823	0	0.6567	487.2947	973.5821	2.28	0.00111	0
P19823	0	1	440.0078	1757.009	1.92	0.00084	35.53595
P19823	0	0.9298	646.6956	1938.072	2.45	0.00158	19.35045
P19823	0	0.9726	646.6954	1938.072	2.26	0.00146	8.4884
P19823	0	0.9846	646.6952	1938.071	1.88	0.00122	10.70938
P19823	0	0.9375	646.6954	1938.072	2.17	0.0014	3.963539
P19823	0	0.8649	646.6959	1938.073	3.02	0.00195	9.18661
P19823	0	0.9242	646.6954	1938.072	2.26	0.00146	3.92616
P19823	0	0.9412	646.6977	1938.079	5.76	0.00372	0
P19823	0	0.9808	646.6938	1938.067	-0.29	-0.00019	69.33345
P19823	0	0.7561	646.6937	1938.066	-0.48	-0.00031	4.899889
P19823	0	0.8704	646.6958	1938.073	2.83	0.00183	31.77669
P19823	0	0.8387	646.6953	1938.071	2.07	0.00134	0
P19823	0	0.875	646.6955	1938.072	2.35	0.00152	62.30809
P19823	0	0.6486	646.6951	1938.071	1.79	0.00116	5.113354
P19823	0	0.6216	646.6946	1938.069	1.03	0.00067	0
P19823	0	0.9155	646.695	1938.07	1.6	0.00103	7.7906
P19823	0	0.8871	646.696	1938.073	3.11	0.00201	1.868179

P19823	0	0.9365	646.6962	1938.074	3.49	0.00225	24.73537
P19823	0	0.9828	646.6954	1938.072	2.26	0.00146	11.13316
P19823	0	0.9153	646.6958	1938.073	2.83	0.00183	14.23139
P19823	0	0.8507	646.6956	1938.072	2.45	0.00158	8.314161
P19823	0	0.8571	646.6957	1938.072	2.64	0.0017	8.881416
P19823	0	0.9615	969.5386	1938.07	1.35	0.00131	5.24963
P19823	0	0.9254	646.6951	1938.071	1.79	0.00116	8.118793
P19823	0	0.9412	969.5389	1938.07	1.61	0.00156	8.203665
P19823	0	0.9385	646.6952	1938.071	1.88	0.00122	9.259316
P19823	0	0.9697	969.5399	1938.072	2.61	0.00253	5.975577
P19823	0	0.9344	646.6953	1938.071	2.07	0.00134	7.482445
P19823	0	0.9099	969.5392	1938.071	1.92	0.00186	6.717195
P19823	0	0.8507	646.6952	1938.071	1.88	0.00122	8.474336
P19823	0	0.9667	969.5394	1938.071	2.11	0.00204	6.14079
P19823	0	0.9365	646.6951	1938.071	1.79	0.00116	5.21696
P19823	0	0.9583	969.5398	1938.072	2.55	0.00247	0
P19823	0	1	892.7507	2676.238	1.14	0.00101	3.234253
P19823	0	0.9155	669.8155	2676.24	2.07	0.00138	3.85085
P19823	0	1	892.7514	2676.24	1.89	0.00168	3.579168
P19823	0	0.7818	669.817	2676.246	4.26	0.00285	0
P19823	1	1	585.3468	1754.026	3.16	0.00185	9.447807
P19823	0	0.9	649.8575	1298.708	2.13	0.00139	3.222637
P19823	0	1	461.5809	1382.728	1.72	0.00079	33.65332
P19823	0	1	691.8685	1382.73	2.96	0.00205	0
P19823	0	0.9375	646.6957	1938.072	2.64	0.0017	15.8395
P19823	0	0.8776	646.6956	1938.072	2.54	0.00164	2.94751
P19823	1	1	426.5794	1277.724	2.6	0.00111	65.19814
P19823	0	1	713.8437	2852.353	2.43	0.00173	5.87711
P19823	0	1	951.4559	2852.353	2.54	0.00242	3.243751
P19823	0	0.8036	657.8552	1314.703	2.45	0.00161	6.631094
P19823	0	0.8704	657.8552	1314.703	2.45	0.00161	6.388641
P19823	0	1	440.0079	1757.01	2.2	0.00097	13.27489
P19823	0	1	586.3419	1757.011	2.93	0.00172	7.835824
P19823	0	1	440.0075	1757.008	1.22	0.00054	9.294937
P19823	0	1	440.008	1757.01	2.34	0.00103	0
P19823	0	1	440.0079	1757.01	2.06	0.0009	19.87256
P19823	0	1	795.7504	2385.237	0.33	0.00026	2.153241
P19823	0	0.8	804.1174	2410.338	1.86	0.0015	11.58696
P19823	0	0.987	804.1174	2410.338	1.86	0.0015	4.697958
P19823	0	0.85	603.3404	2410.34	2.69	0.00162	24.76373
P19823	0	0.9062	633.0913	2529.343	2.13	0.00135	4.982163
P19823	0	1	993.7443	3971.955	-0.06	-0.00006	0
P19823	0	0.6923	633.0917	2529.345	2.71	0.00172	5.41684
P19823	0	0.9615	863.9825	1726.958	0.58	0.0005	3.752822
P19823	0	0.9429	576.3245	1726.959	1.17	0.00068	5.235427
P19823	0	1	413.5463	1238.624	0.57	0.00024	24.50912
P19823	0	0.9429	619.8174	1238.627	3.12	0.00193	0
P19823	0	0.9583	650.3269	1948.966	1.44	0.00094	20.58689
P19823	0	0.4	538.307	1612.906	1.54	0.00083	9.306217
P19823	0	0.68	569.9821	1707.932	0.47	0.00027	45.11551
P19823	1	0.9737	425.2676	1273.788	4.12	0.00175	9.015192
P19823	0	0.8205	569.9844	1707.939	4.54	0.00258	2.256958

P19823	0	0.6618	487.2946	973.582	2.16	0.00105	8.322962
P19823	0	0.9333	569.9841	1707.938	4.11	0.00234	19.01415
P19823	0	0.6	569.9837	1707.937	3.36	0.00191	10.5902
P19823	0	0.7179	427.7389	1707.934	1.64	0.0007	7.647493
P19823	0	0.8472	854.4705	1707.934	1.73	0.00147	4.508567
P19823	0	0.9804	569.9823	1707.932	0.9	0.00051	9.457501
P19823	0	0.8108	569.9836	1707.936	3.25	0.00185	37.84072
P19823	0	0.9636	645.3597	1934.065	1.58	0.00102	4.345253
P19823	0	1	645.3603	1934.066	2.44	0.00157	4.595862
P19823	0	1	645.3601	1934.066	2.06	0.00133	4.590316
P19823	1	1	694.6116	2775.425	2.34	0.00162	7.80178
P19823	0	0.9756	645.3604	1934.067	2.63	0.00169	
P19823	0	1	1087.904	3261.696	1.71	0.00186	62.70473
P19823	0	1	585.2677	1169.528	2.2	0.00129	24.66706
P19823	1	1	426.579	1277.722	1.6	0.00068	17.17769
P19823	1	0.9737	497.2603	1489.766	1.71	0.00085	14.27363
P19823	1	1	1060.628	2120.248	0.63	0.00067	14.49867
P19823	1	1	707.421	2120.249	0.71	0.0005	17.34978
P19823	0	0.619	559.3467	1117.686	3.72	0.00208	7.267285
P19823	0	0.4483	559.3458	1117.684	2.09	0.00117	4.152959
P19823	0	0.7193	559.3465	1117.686	3.4	0.0019	0
P19823	0	0.5357	633.0913	2529.343	2.13	0.00135	4.119233
P19823	0	0.6818	633.0914	2529.344	2.23	0.00141	40.51474
P19823	0	0.5405	633.0911	2529.343	1.84	0.00117	14.56382
P19823	0	0.9459	633.09	2529.338	0.11	0.00007	5.533314
P19823	0	1	843.7858	2529.343	1.87	0.00158	4.670127
P19823	0	0.6842	506.6739	2529.34	0.92	0.00047	6.745314
P19823	0	0.9383	685.0523	2053.142	1.34	0.00092	5.95778
P19823	0	0.9818	1027.075	2053.142	1.2	0.00123	0
P19823	0	0.7959	439.2667	1315.785	2.34	0.00102	10.8255
P19823	0	0.8816	658.3965	1315.786	2.53	0.00167	5.811566
P19823	0	0.8444	439.2681	1315.79	5.54	0.00243	9.061821
P19823	0	0.8714	924.5306	1848.054	2.11	0.00195	22.12577
P19823	0	0.8356	616.6895	1848.054	2.17	0.00134	2.916738
P19823	0	0.9016	657.3183	1313.629	1.3	0.00085	3.185106
P19823	0	1	438.5482	1313.63	1.76	0.00077	8.299137
P19823	0	1	741.4166	1481.826	1.82	0.00135	4.076958
P19823	0	0.9302	494.6137	1481.827	2.29	0.00113	3.902989
P19823	0	1	494.6136	1481.826	1.92	0.00095	26.34913
P19823	0	0.9231	808.6273	4039.107	0.99	0.0008	0
P19823	0	0.9508	547.9625	1641.873	2.57	0.00141	7.548377
P19823	0	1	821.4393	1641.871	1.62	0.00133	5.004364
P19823	0	1	1010.54	4039.137	8.32	0.0084	10.39841
P19823	0	1	898.0829	2692.234	1.7	0.00153	5.25773
P19823	0	1	413.5465	1238.625	1.09	0.00045	23.53882
P19823	0	0.4545	575.3409	1149.675	2.49	0.00143	29.68867
P19823	0	1	440.0078	1757.009	1.92	0.00084	6.392457
P19823	0	1	461.5809	1382.728	1.85	0.00085	61.52487
P19823	0	0.913	646.6968	1938.076	4.34	0.0028	0
P19823	0	1	879.0085	1757.01	2.07	0.00182	0
P19823	0	0.3261	559.3466	1117.686	3.51	0.00196	10.31867
P19823	0	0.5435	559.3462	1117.685	2.85	0.00159	22.53005

P19823	0	0.76	597.066	2385.242	2.58	0.00154	19.71237
P19823	0	0.8095	650.3284	1948.971	3.7	0.0024	0
P19823	0	0.48	597.0665	2385.244	3.39	0.00202	10.65519
P19823	0	0.8571	440.0079	1757.01	2.06	0.0009	10.94782
P19823	1	0.7273	531.6392	1592.903	2.05	0.00109	4.205298
P19823	0	0.8889	602.2933	1804.865	2.31	0.00139	4.622339
P19823	0	1	461.5804	1382.727	0.59	0.00027	30.95078
P19823	0	0.8148	645.3598	1934.065	1.68	0.00108	62.51734
P19823	0	0.8889	646.6947	1938.07	1.13	0.00073	33.59188
P19823	0	0.9091	969.5378	1938.068	0.47	0.00046	33.37907
P19823	0	1	650.329	1948.973	4.73	0.00307	30.2214
P19823	0	0.4444	403.9822	1612.907	1.8	0.00072	13.72105
P19823	0	0.7872	586.3457	1171.684	3.27	0.00192	4.841331
P19823	0	0.3778	510.801	1020.595	1.9	0.00097	7.314756
P19823	0	1	461.5802	1382.726	0.33	0.00015	26.13413
P19823	0	0.7632	560.8162	1120.625	1.83	0.00103	32.86502
P19823	0	0.5526	383.8964	1149.675	2.58	0.00099	11.09172
P19823	0	1	443.88	1329.626	2.28	0.00101	20.71251
P19823	0	1	440.0079	1757.01	2.06	0.0009	38.88612
P19823	0	1	479.723	958.4387	1.03	0.00049	1.236645
P19823	0	1	669.366	1337.725	2.67	0.00178	0
P19823	1	1	585.3467	1754.026	3.06	0.00179	20.01776
P19823	0	0.5385	510.8004	1020.593	0.71	0.00036	26.29689
P19823	0	0.4286	597.0659	2385.242	2.47	0.00147	0
P19823	0	0.88	669.8137	2676.233	-0.58	-0.00039	19.21533
P19823	0	1	898.0833	2692.235	2.11	0.00189	8.795095
P19823	0	1	593.2656	1185.524	2.96	0.00175	19.80198
P19823	2	0.0588	464.953	1392.844	2.28	0.00106	39.93721
P19823	1	0.6667	377.2325	1129.683	1.71	0.00064	21.77676
P19823	0	1	440.0073	1757.007	0.74	0.00032	50.02226
P19823	0	0.5122	495.2921	989.577	2.2	0.00109	10.51057
P19823	0	0.9167	646.6977	1938.079	5.76	0.00372	24.03932
P19823	1	1	513.6148	1538.83	2	0.00102	61.11507
P19823	0	0.8485	433.574	1298.708	1.94	0.00084	21.03227
P19823	0	0.7556	547.8106	1094.614	1.85	0.00101	17.59296
P19823	0	1	479.7232	958.4391	1.48	0.00071	1.961053
P19823	0	0.6	538.3073	1612.907	1.99	0.00107	33.26962
P19823	0	0.7826	645.3612	1934.069	3.86	0.00249	0
P19823	0	0.7632	547.8106	1094.614	1.96	0.00107	30.49237
P19823	0	1	993.7463	3971.964	2.03	0.00201	2.224888
P19823	0	0.8333	547.8115	1094.616	3.52	0.00193	46.23673
P19823	0	0.8966	446.2489	891.4905	1.24	0.00055	5.590342
P19823	0	0.5484	575.3414	1149.676	3.34	0.00192	44.35783
P19823	0	0.875	795.7543	2385.248	5.17	0.00411	21.14001
P19823	0	0.6364	559.3455	1117.684	1.65	0.00092	0
P19823	0	0.4516	438.9059	1314.703	2.46	0.00108	14.78954
P19823	0	0.6286	510.801	1020.595	1.84	0.00094	0
P19823	0	1	413.5466	1238.625	1.39	0.00057	78.91801
P19823	0	0.9565	499.9279	1497.769	1.77	0.00088	16.7561
P19823	0	0.9	518.2999	1035.593	1.06	0.00055	3.051961
P19823	0	1	518.2996	1035.592	0.47	0.00024	3.583137
P19823	0	0.7059	633.0897	2529.337	-0.47	-0.0003	28.20782

P19823	0	0.8571	969.5395	1938.072	2.24	0.00217	3.449175
P19823	0	0.5	403.9825	1612.908	2.48	0.001	23.94367
P19823	0	0.3684	495.2917	989.5761	1.34	0.00066	14.93933
P19823	0	0.8605	547.8107	1094.614	2.19	0.0012	5.805288
P19823	0	1	804.1176	2410.338	2.01	0.00162	22.72613
P19823	0	0.5789	804.1174	2410.338	1.86	0.0015	0
P19823	0	1	547.9625	1641.873	2.57	0.00141	72.589
P19823	0	1	892.7502	2676.236	0.59	0.00052	11.12249
P19823	0	0.7826	547.8106	1094.614	1.96	0.00107	14.67545
P19823	0	0.9286	619.8159	1238.625	0.76	0.00047	0
P19823	0	1	669.3663	1337.725	3.03	0.00203	5.97924
P19823	0	0.8	487.2945	973.5818	1.97	0.00096	24.56739
P19823	0	0.5714	575.3385	1149.67	-1.76	-0.00101	29.21977
P19823	0	1	586.3427	1757.013	4.19	0.00245	0
P19823	0	0.5	510.7988	1020.59	-2.34	-0.0012	0
P19823	0	0.6316	383.8958	1149.673	0.91	0.00035	50.16993
P19823	0	0.9231	413.5466	1238.625	1.24	0.00051	29.28199
P19823	0	1	650.3268	1948.966	1.35	0.00088	23.32343
P19823	0	1	440.007	1757.006	0.11	0.00005	8.228024
P19823	1	1	439.2585	1754.012	-4.53	-0.00199	38.0364
P19823	0	0.7234	487.297	973.5868	7.11	0.00346	0
P19823	0	0	633.0895	2529.336	-0.76	-0.00048	21.60957
P19823	0	1	547.81	1094.613	0.85	0.00046	7.859261
P19823	0	0.8214	438.9063	1314.704	3.37	0.00148	41.12018
P19823	0	0.8333	795.7526	2385.243	3.02	0.0024	35.26109
P19823	0	0.6	495.2914	989.5755	0.72	0.00036	3.220678
P19823	1	0.9412	497.2598	1489.765	0.67	0.00033	7.064361
P19823	0	0.9167	673.8136	2692.233	1.13	0.00076	6.691668
P19823	0	1	795.1981	3971.961	1.52	0.0012	0
P19823	0	1	586.343	1757.015	4.81	0.00282	28.12617
P00450	0	1	716.3232	1431.639	0.38	0.00027	22.10671
P00450	0	0.9434	555.2869	1663.846	0.75	0.00041	0
P00450	0	0.9884	760.3764	1519.746	1.84	0.0014	0
P00450	1	1	538.5002	2688.472	1.5	0.00081	17.78234
P00450	1	1	628.6398	1883.905	3.94	0.00247	5.385663
P00450	0	0.6	490.2775	1468.818	1.9	0.00093	11.74921
P00450	0	1	931.7825	2793.333	1.75	0.00163	3.441377
P00450	0	1	931.7838	2793.337	3.13	0.00291	3.357131
P00450	0	1	931.7835	2793.336	2.8	0.00261	5.249866
P00450	0	1	931.7819	2793.331	1.09	0.00102	3.424155
P00450	0	1	1397.171	2793.334	2.26	0.00315	5.354288
P00450	0	1	699.0904	2793.34	4.21	0.00294	20.40367
P00450	0	1	699.0888	2793.333	1.94	0.00136	2.919945
P00450	0	1	931.7818	2793.331	1.03	0.00096	3.541387
P00450	0	0.9841	662.8615	1324.716	1.98	0.00131	0
P00450	0	1	442.2433	1324.715	1.67	0.00074	11.73983
P00450	0	1	883.7482	2649.23	1.53	0.00135	3.212005
P00450	0	1	1142.187	3424.548	1.35	0.00154	0.848651
P00450	0	0.4375	490.2775	1468.818	1.96	0.00096	12.42609
P00450	0	1	662.861	1324.715	1.34	0.00089	6.421204
P00450	0	0.8983	442.2426	1324.713	0.15	0.00007	37.59397
P00450	0	0.8611	734.9134	1468.82	3.03	0.00222	7.48674

P00450	0	0.6552	490.278	1468.819	2.89	0.00142	30.14811
P00450	0	0.5	490.2771	1468.817	1.09	0.00053	12.09193
P00450	1	1	628.639	1883.903	2.77	0.00174	7.20571
P00450	1	1	862.9431	3448.75	2.95	0.00254	3.306401
P00450	1	1	1150.254	3448.748	2.12	0.00243	4.316582
P00450	0	1	442.2431	1324.715	1.4	0.00062	0
P00450	0	0.9825	643.338	1927.999	2.93	0.00189	8.923564
P00450	0	0.9828	643.3384	1928.001	3.6	0.00231	9.050024
P00450	0	1	1316.202	2631.396	2.44	0.00321	0
P00450	0	1	442.243	1324.714	1.12	0.0005	6.907844
P00450	1	1	628.639	1883.902	2.67	0.00168	3.255791
P00450	0	1	672.3326	2686.309	2.23	0.0015	6.145591
P00450	0	1	672.3323	2686.307	1.69	0.00113	3.358502
P00450	0	0.9296	672.3328	2686.309	2.5	0.00168	2.964321
P00450	0	0.9559	672.333	2686.31	2.78	0.00186	2.729214
P00450	0	0.9744	672.3314	2686.304	0.32	0.00022	0
P00450	0	0.9792	672.3326	2686.308	2.14	0.00144	4.476688
P00450	0	1	745.3688	2234.092	1.5	0.00111	15.52868
P00450	0	1	1117.55	2234.092	1.67	0.00186	0
P00450	0	1	1136.856	3408.554	1.87	0.00212	7.776856
P00450	0	1	852.8937	3408.553	1.49	0.00127	1.380633
P00450	0	1	1136.856	3408.553	1.33	0.00151	0
P00450	1	1	729.8887	2916.533	-0.25	-0.00018	10.62074
P00450	0	1	658.6039	2631.394	1.45	0.00096	3.434263
P00450	2	1	770.605	3848.996	-0.01	-0.00001	0
P00450	0	0.8684	705.6188	2819.454	1.46	0.00103	3.725968
P00450	0	0.9767	940.4902	2819.456	2.39	0.00225	3.935268
P00450	0	0.95	555.2874	1663.848	1.63	0.0009	4.835274
P00450	0	1	832.4258	1663.844	-0.31	-0.00026	3.414218
P00450	0	0.8718	705.6185	2819.452	1.02	0.00072	4.264041
P00450	0	0.9636	940.4898	2819.455	1.87	0.00176	4.228877
P00450	0	0.9118	940.4901	2819.456	2.2	0.00206	3.504708
P00450	0	0.8769	505.9606	1515.867	1.33	0.00067	5.002064
P00450	0	0.9524	832.4278	1663.848	2.11	0.00176	13.00554
P00450	0	0.8125	940.4903	2819.456	2.46	0.00231	30.20859
P00450	0	0.8056	705.6194	2819.456	2.24	0.00158	5.9952
P00450	0	0.8298	705.6186	2819.453	1.11	0.00078	0
P00450	0	0.8936	940.4902	2819.456	2.33	0.00219	7.487749
P00450	0	0.8286	758.4374	1515.868	1.62	0.00123	19.38717
P00450	0	0.9286	499.2859	1495.843	-0.03	-0.00001	53.20101
P00450	0	1	586.9836	1758.936	3.62	0.00212	9.275547
P00450	0	0.9302	879.9705	1758.934	2.17	0.00191	6.121106
P00450	0	1	586.9816	1758.93	0.28	0.00017	8.026421
P00450	0	0.86	499.2851	1495.841	-1.62	-0.00081	42.10028
P00450	1	1	969.4645	2906.379	2.23	0.00216	5.443057
P00450	1	1	727.3495	2906.376	1.22	0.00089	5.718077
P00450	0	1	785.8994	3140.576	3.3	0.00259	24.12132
P00450	1	1	969.4647	2906.38	2.42	0.00234	6.318775
P00450	1	1	727.3475	2906.368	-1.55	-0.00113	6.256988
P00450	1	1	969.4651	2906.381	2.8	0.00271	5.95311
P00450	1	1	727.35	2906.378	1.97	0.00143	5.856743
P00450	0	0.9444	879.9699	1758.933	1.54	0.00136	5.59929

P00450	0	0.9804	674.3188	1347.63	0.43	0.00029	3.096059
P00450	0	1	586.687	1758.046	1.56	0.00091	7.870058
P00450	0	0.9815	674.318	1347.629	-0.75	-0.00051	9.394844
P00450	0	0.8889	879.5267	1758.046	1.33	0.00116	5.536721
P00450	0	0.9508	674.3197	1347.632	1.79	0.0012	6.71616
P00450	0	1	785.9022	3140.587	6.88	0.0054	12.26911
P00450	1	1	974.7971	2922.377	3.19	0.0031	5.893039
P00450	1	1	731.3487	2922.373	1.87	0.00136	5.526311
P00450	1	1	974.7959	2922.373	1.93	0.00188	5.215619
P00450	1	1	731.3488	2922.373	2.03	0.00149	6.459257
P00450	0	1	662.8615	1324.716	1.98	0.00131	4.162364
P00450	0	1	442.2437	1324.716	2.57	0.00114	24.06189
P00450	0	0.127	1100.672	2200.338	1.33	0.00147	0
P00450	0	0.9787	734.1173	2200.337	1.11	0.00081	5.333075
P00450	2	0.5	513.6468	1538.926	1.47	0.00075	13.77847
P00450	0	0.9787	734.1173	2200.337	1.11	0.00081	5.333075
P00450	0	0.8033	490.2769	1468.816	0.65	0.00032	10.81433
P00450	0	1	782.1525	2344.443	2.65	0.00207	7.184777
P00450	0	0.9487	586.8665	2344.444	3.06	0.00179	2.36209
P00450	0	1	1172.725	2344.443	2.6	0.00305	0
P00450	0	1	1155.043	4617.15	-2.47	-0.00285	0
P00450	0	1	924.2405	4617.173	2.56	0.00237	7.549236
P00450	0	0.8043	493.9552	1479.851	1.83	0.0009	9.000023
P00450	0	0.8649	740.4293	1479.851	2.05	0.00152	7.005876
P00450	0	1	683.313	2047.924	0.88	0.0006	11.40783
P00450	0	1	1024.467	2047.926	1.81	0.00186	3.644149
P00450	0	1	683.3133	2047.925	1.33	0.00091	2.629423
P00450	0	1	952.4161	1903.825	2.28	0.00217	0
P00450	1	1	500.5142	1999.035	0.33	0.00016	43.95255
P00450	1	0.9211	667.0178	1999.039	2.26	0.00151	12.60032
P00450	1	0.9	500.5148	1999.037	1.43	0.00071	13.20689
P00450	1	0.9211	667.0175	1999.038	1.71	0.00114	11.05159
P00450	1	0.8846	500.5149	1999.038	1.61	0.00081	11.62384
P00450	1	0.9318	667.0176	1999.038	1.99	0.00132	11.43828
P00450	1	0.8276	500.5144	1999.036	0.76	0.00038	12.30724
P00450	1	1	667.0181	1999.04	2.72	0.00181	16.28873
P00450	0	0.9792	879.5293	1758.051	4.31	0.00379	29.65958
P00450	0	1	586.687	1758.046	1.46	0.00085	0
P00450	1	0.9048	667.0183	1999.04	2.99	0.00199	7.382736
P00450	1	1	500.5147	1999.037	1.37	0.00068	0
P00450	1	0.9091	667.0176	1999.038	1.89	0.00126	17.03421
P00450	1	0.9677	500.5144	1999.036	0.76	0.00038	10.08507
P00450	1	1	731.35	2922.378	3.62	0.00264	2.04298
P00450	1	1	731.349	2922.374	2.28	0.00167	7.070083
P00450	0	1	802.004	4005.991	2.02	0.00162	21.19365
P00450	1	0.9032	500.5146	1999.036	1	0.0005	8.621091
P00450	1	0.975	667.0179	1999.039	2.35	0.00157	11.03618
P00450	1	0.8857	500.5147	1999.037	1.18	0.00059	8.682019
P00450	1	0.9149	667.0176	1999.038	1.89	0.00126	10.40071
P00450	1	0.9302	667.0187	1999.042	3.63	0.00242	6.343308
P00450	1	0.975	577.9533	1731.845	1.93	0.00112	16.16106
P00450	0	1	785.8979	3140.57	1.36	0.00107	7.190181

P00450	1	0.8	667.0178	1999.039	2.17	0.00144	12.09949
P00450	1	1	500.5148	1999.037	1.43	0.00071	11.7835
P00450	1	0.9024	667.0177	1999.039	2.08	0.00138	10.93154
P00450	1	1	577.9537	1731.846	2.67	0.00154	19.3633
P00450	1	0.9535	1000.023	1999.039	2.05	0.00205	0
P00450	1	0.8889	577.9534	1731.846	2.15	0.00124	16.0069
P00450	1	1	667.0175	1999.038	1.71	0.00114	13.85439
P00450	1	0.931	500.5147	1999.037	1.18	0.00059	6.32183
P00450	1	0.8919	667.0176	1999.038	1.89	0.00126	10.53005
P00450	1	0.8667	577.9536	1731.846	2.46	0.00142	6.217479
P00450	1	0.9	500.515	1999.038	1.8	0.0009	9.34144
P00450	0	1	716.3237	1431.64	0.98	0.0007	0.999475
P00450	0	0.9245	525.9193	1575.743	1.68	0.00088	5.883184
P00450	0	0.9846	788.3753	1575.743	1.65	0.0013	3.334193
P00450	0	0.9556	788.3753	1575.743	1.65	0.0013	100
P00450	0	1	788.3751	1575.743	1.34	0.00106	100
P00450	0	0.8776	651.3198	1301.632	2.93	0.0019	6.181798
P00450	0	1	943.7922	2829.362	2.04	0.00193	2.960512
P00450	0	1	731.8798	1462.752	2.29	0.00168	6.474932
P00450	0	0.8103	594.8309	1188.654	3.52	0.00209	6.572426
P00450	0	1	604.8247	2416.277	2.46	0.00149	11.9157
P00450	0	0.875	806.0964	2416.275	1.46	0.00117	4.904213
P00450	1	1	628.6384	1883.901	1.7	0.00107	13.25065
P00450	1	1	628.6394	1883.904	3.35	0.00211	7.845357
P00450	1	1	942.4544	1883.901	2.16	0.00204	2.77908
P00450	1	1	628.6375	1883.898	0.24	0.00015	8.706073
P00450	1	1	942.4545	1883.902	2.36	0.00222	7.306626
P00450	1	0.9333	471.7304	1883.9	1.24	0.00058	6.87355
P00450	1	0.9444	636.8489	2544.374	3.11	0.00198	49.05018
P00450	0	0.9868	757.3682	1513.729	2.08	0.00157	5.889874
P00450	1	0.9444	636.8489	2544.374	3.11	0.00198	49.05018
P00450	1	0.9444	538.5002	2688.472	1.39	0.00075	14.23408
P00450	1	1	538.5005	2688.473	1.96	0.00105	8.268015
P00450	0	1	871.4406	4353.174	-0.21	-0.00018	0
P00450	1	1	538.5006	2688.474	2.18	0.00117	8.091995
P00450	1	1	672.8745	2688.476	2.94	0.00197	7.483015
P00450	1	1	672.8748	2688.477	3.48	0.00234	5.055246
P00450	1	1	538.5004	2688.473	1.73	0.00093	8.847569
P00450	1	1	538.5007	2688.474	2.3	0.00124	9.509255
P00450	1	1	672.8743	2688.475	2.67	0.00179	6.640608
P00450	1	1	538.5009	2688.475	2.75	0.00148	11.57976
P00450	1	1	672.874	2688.474	2.3	0.00155	7.855739
P00450	1	1	538.5013	2688.478	3.55	0.00191	11.23257
P00450	0	1	1407.186	2813.364	1.08	0.00151	0
P00450	0	1	938.4608	2813.368	2.29	0.00214	17.25761
P00450	0	1	704.0972	2813.367	1.98	0.00139	3.170245
P00450	0	1	1001.527	3002.566	3.93	0.00394	9.767673
P00450	1	0.093	618.9837	1854.937	2.28	0.00141	7.920289
P00450	1	0.9787	618.984	1854.937	2.67	0.00165	0
P00450	1	0.9787	618.984	1854.937	2.67	0.00165	0
P00450	0	0.8873	860.4841	1719.961	1.51	0.0013	3.624142
P00450	0	1	751.3945	3002.556	0.61	0.00046	4.879777

P00450	0	0.9811	643.3373	1927.997	1.89	0.00121	17.38432
P00450	0	0.963	643.3386	1928.001	3.79	0.00243	13.15815
P00450	0	0.8478	493.587	1478.746	1.71	0.00085	11.47195
P00450	0	1	807.4756	1613.944	1.38	0.00111	3.260042
P00450	0	0.8627	594.83	1188.653	2.08	0.00124	9.080737
P00450	1	1	538.5006	2688.474	2.07	0.00111	16.70759
P00450	0	1	802.0032	4005.987	1.03	0.00082	8.911998
P00450	1	1	628.6387	1883.901	2.19	0.00137	16.25809
P00450	1	0.0444	618.9841	1854.938	2.97	0.00184	10.26887
P00450	1	0.9091	921.4298	2762.275	1.51	0.00139	3.626569
P00450	0	0.8333	564.6967	2819.454	1.69	0.00095	31.19411
P00450	1	0.0612	580.6051	1739.801	3.15	0.00182	0
P00450	0	0.8958	449.8821	1347.632	1.39	0.00063	4.289437
P00450	1	1	471.7307	1883.901	2.02	0.00095	5.756726
P00450	1	0.9091	471.7307	1883.901	1.89	0.00089	12.30833
P00450	0	1	602.2698	1203.532	3.94	0.00237	30.25021
P00450	1		618.9841	1854.938	2.97	0.00184	10.26887
P00450	0	0.9756	586.689	1758.053	5	0.00293	4.026516
P00450	0	1	699.3066	1397.606	0.75	0.00052	0
P00450	0	0.95	538.9481	1614.83	1.15	0.00062	18.24586
P00450	0	0.825	525.9191	1575.743	1.22	0.00064	11.26662
P00450	1	0.9286	538.5006	2688.474	2.07	0.00111	46.29192
P00450	0	0.6786	422.2188	1264.642	0.63	0.00026	9.718333
P00450	0	0.8571	594.8311	1188.655	3.93	0.00233	26.8159
P00450	0	0.9091	555.2858	1663.843	-1.23	-0.00068	20.22786
P00450	0	1	488.2555	1462.752	2.05	0.001	10.00049
P00450	0	0.7561	505.9619	1515.871	4.05	0.00205	0
P00450	0	0.9818	716.3244	1431.642	2	0.00143	0
P00450	0	0.9697	493.5874	1478.748	2.46	0.00121	18.65011
P00450	0	0.9394	555.2871	1663.847	1.08	0.0006	31.31767
P00450	1	1	395.244	1577.954	1.53	0.0006	18.21799
P00450	0	0.7692	632.8265	1264.646	3.66	0.00231	5.300094
P00450	1	1	974.7965	2922.375	2.5	0.00243	6.809644
P00450	0	1	602.2694	1203.531	3.13	0.00188	5.035251
P00450	0	0.8605	594.8311	1188.655	3.83	0.00227	39.59385
P00450	0	1	509.2362	1017.465	1.57	0.0008	15.31666
P00450	0	0.7333	705.6195	2819.456	2.32	0.00164	9.356141
P00450	1	0.8667	471.7309	1883.902	2.28	0.00107	7.773092
P00450	0	0.9231	525.9194	1575.744	1.8	0.00095	18.44185
P00450	0	0.7297	740.4313	1479.855	4.69	0.00347	37.03231
P00450	0	0.9429	490.2773	1468.817	1.65	0.00081	9.253293
P00450	0	0.7925	748.4236	1495.84	-2.24	-0.00168	0
P00450	0	1	674.3192	1347.631	1.06	0.00072	45.57521
P00450	0	1	449.8821	1347.632	1.39	0.00063	37.55172
P00450	1	0.8333	500.5143	1999.035	0.51	0.00026	23.96867
P00450	0	0.9565	488.2549	1462.75	0.87	0.00042	17.34964
P00450	1	0.84	500.5153	1999.039	2.47	0.00123	8.39565
P00450	0	1	651.3192	1301.631	1.89	0.00123	6.318223
P00450	0	1	674.3201	1347.633	2.42	0.00163	2.63369
P00450	1	1	1000.023	1999.039	2.36	0.00236	2.108837
P00450	1	1	538.5012	2688.477	3.21	0.00172	17.27626
P00450	0	1	490.2787	1468.821	4.33	0.00212	90.85908

P00450	0	1	488.2552	1462.751	1.3	0.00064	8.839616
P00450	1	0.0556	618.9842	1854.938	3.07	0.0019	4.993354
P00450	1	0.9091	896.8301	2688.476	2.83	0.00254	8.922627
P00450	1	0.8667	727.3489	2906.374	0.38	0.00027	3.014759
P00450	1	0.8966	618.9828	1854.934	0.8	0.00049	26.0316
P00450	0	0.8485	586.9835	1758.936	3.51	0.00206	63.07257
P00450	1	0.8966	618.9828	1854.934	0.8	0.00049	26.0316
P00450	0	0.931	449.8824	1347.633	2.14	0.00096	5.200198
P00450	0	1	699.3067	1397.606	0.83	0.00058	0
P00450	0	0.6154	705.619	2819.454	1.63	0.00115	12.77
P00450	0	1	931.7817	2793.33	0.9	0.00084	1.699859
P00450	0	0.9677	586.9833	1758.935	3.2	0.00188	2.912965
P00450	0	0.9167	705.6196	2819.456	2.5	0.00176	38.67909
P00450	0	0.8148	396.8897	1188.654	3.53	0.0014	10.85422
P00450	0	1	505.2484	1513.731	3.02	0.00152	8.54055
P00450	1	0.9143	667.0195	1999.044	4.73	0.00315	3.584926
P00450	0	0.7778	422.2197	1264.645	2.66	0.00112	72.40382
P00450	1	1	672.8741	2688.475	2.39	0.00161	11.09231
P00450	1	1	667.0186	1999.041	3.36	0.00224	23.38348
P00450	0	0.55	422.2188	1264.642	0.56	0.00023	21.22042
P00450	0	1	509.2367	1017.466	2.47	0.00126	19.66953
P00450	1	0.9524	667.0172	1999.037	1.34	0.0009	0
P00450	0	1	509.2357	1017.464	0.55	0.00028	6.67155
P00450	0	1	699.3074	1397.607	1.79	0.00125	0
P00450	0	0.8378	594.8304	1188.654	2.8	0.00166	12.774
P00450	0	1	434.5487	1301.631	2.24	0.00097	16.7112
P00450	0	0.8222	686.3879	1371.768	3.94	0.0027	30.87147
P00450	0	0.6875	422.2197	1264.645	2.66	0.00112	30.70127
P00450	0	0.5769	499.2868	1495.846	1.75	0.00087	0
P00450	1	0.8148	667.0178	1999.039	2.26	0.00151	10.44033
P00450	1	1	471.7312	1883.903	3.05	0.00144	33.6981
P00450	0	0.9487	716.3253	1431.643	3.19	0.00229	0
P00450	0	0.9231	788.3754	1575.743	1.73	0.00136	2.933596
P00450	1	0.7083	667.0176	1999.038	1.89	0.00126	9.228986
P00450	0	0.875	525.919	1575.742	1.1	0.00058	4.901604
P00450	0	0.9302	686.3875	1371.768	3.32	0.00227	0
P00450	1	0.8182	628.637	1883.897	-0.44	-0.00028	41.99125
P00450	0	0.2	940.4899	2819.455	2.07	0.00194	4.724482
P00450	0	0.4706	422.219	1264.642	0.92	0.00039	53.66337
P00450	0	0	940.4904	2819.457	2.59	0.00243	8.52035
P00450	0	1	505.2471	1513.727	0.54	0.00027	16.5705
P00450	0	1	785.8976	3140.569	1.05	0.00082	0
P00450	0	0.75	493.5877	1478.749	3.14	0.00155	9.698661
P00450	0	0.9048	449.8828	1347.634	3.09	0.00139	8.997388
P00450	0	0.92	442.2438	1324.717	2.92	0.00129	21.13564
P00450	1	0.7308	577.9544	1731.849	3.94	0.00228	7.456669
P00450	0	0.7368	731.8792	1462.751	1.37	0.00101	12.10836
P00747	1	1	730.5664	3648.803	1.28	0.00094	4.895686
P00747	1	0.9167	912.9573	3648.808	2.54	0.00232	11.91792
P00747	1	1	730.567	3648.806	2.04	0.00149	3.862627
P00747	1	1	912.957	3648.806	2.21	0.00201	0.579091
P00747	0	1	1175.883	3525.635	1.9	0.00223	3.518208

P00747	0	1	882.1637	3525.633	1.22	0.00107	3.652641
P00747	0	1	676.1271	3376.606	1.5	0.00101	40.21682
P00747	0	1	1126.207	3376.608	1.9	0.00214	4.232334
P00747	0	1	844.907	3376.606	1.52	0.00128	4.722673
P00747	0	1	844.907	3376.606	1.45	0.00122	5.7482
P00747	0	1	882.1637	3525.633	1.22	0.00107	3.497389
P00747	1	0.9444	463.5806	1388.727	3.47	0.0016	3.494759
P00747	1	0.8814	463.5799	1388.725	1.82	0.00084	3.857638
P00747	1	1	598.3007	1792.888	2.85	0.0017	6.877428
P00747	0	1	452.2418	1354.711	0.8	0.00036	0.029963
P00747	0	1	512.8996	1536.684	2.5	0.00128	2.494043
P00747	0	0.9388	481.5888	1442.752	0.59	0.00028	2.695117
P00747	0	0.8269	507.5675	1520.688	1.57	0.0008	4.444267
P00747	0	1	481.5885	1442.751	0.14	0.00007	6.999498
P00747	0	0.875	507.5673	1520.687	1.15	0.00058	44.91856
P00747	0	1	481.5904	1442.757	4.08	0.00196	36.5256
P00747	0	1	721.8804	1442.754	1.91	0.00137	3.323581
P00747	1	1	486.9495	1458.834	-0.76	-0.00037	24.86668
P00747	1	1	703.3706	2108.097	1.1	0.00077	5.120021
P00747	1	1	527.7796	2108.097	0.78	0.00041	3.422945
P00747	0	0.9385	958.017	1915.027	2.09	0.002	2.786434
P00747	0	0.9508	639.0132	1915.025	1.16	0.00074	2.415774
P00747	0	0.9107	744.6068	2975.405	1.13	0.00084	3.812641
P00747	0	1	880.814	2640.427	1.81	0.00159	14.32488
P00747	0	1	660.8622	2640.427	1.73	0.00114	3.22368
P00747	0	0.9873	880.8138	2640.427	1.67	0.00147	2.613445
P00747	0	0.9836	660.8619	2640.426	1.27	0.00084	2.355811
P00747	0	1	783.8793	1566.751	1.37	0.00107	3.440332
P00747	0	1	660.8631	2640.431	3.03	0.002	5.836783
P00747	0	1	651.337	1951.996	1.3	0.00085	4.381309
P00747	0	0.975	806.4035	2417.196	1.32	0.00107	11.92917
P00747	0	1	854.4384	2561.301	2.22	0.0019	3.00357
P00747	0	1	792.1512	3165.583	8.43	0.00667	19.60836
Q02325; P0	0	1	513.2464	1537.725	1.77	0.00091	8.781481
P00747	0	1	808.0349	2422.09	1.67	0.00135	4.347972
Q02325; P0	0	1	769.3662	1537.725	2.02	0.00155	3.467448
Q02325; P0	0	1	513.2459	1537.723	0.82	0.00042	28.60907
P00747	0	1	808.0341	2422.088	0.69	0.00056	3.99156
Q02325; P0	0	0.9351	761.3681	1521.729	1.26	0.00096	3.723963
Q02325; P0	0	0.875	507.9149	1521.73	1.98	0.001	3.228438
P00747	0	0.8909	642.8688	1284.73	1.79	0.00115	39.82595
Q02325; P0	0	0.8372	507.9146	1521.729	1.43	0.00073	11.80114
P00747	1	1	645.3038	2578.194	2.5	0.00161	0
P00747	0	0.9672	854.4377	2561.299	1.51	0.00129	26.36061
P00747	1	1	860.0683	2578.19	1.27	0.00109	2.942597
P00747	0	0.9388	691.3762	1381.745	2.31	0.00159	5.08467
P00747	0	1	714.9196	1428.832	1.26	0.0009	6.917108
P00747	0	0.9038	476.9483	1428.83	0.22	0.00011	8.36368
P00747	1	1	486.9507	1458.837	1.63	0.00079	12.34657
P00747	0	1	1050.525	3149.562	0.1	0.00011	0
P00747	0	1	788.1453	3149.559	-0.66	-0.00052	22.21599
P00747	0	1	854.4377	2561.299	1.44	0.00123	8.06766

P00747	0	1	1281.152	2561.296	0.44	0.00056	0
P00747	0	0.967	739.3774	1477.748	2.64	0.00195	0
P00747	0	0.9608	541.2877	1621.849	1.75	0.00095	8.033704
P00747	0	0.9605	541.2886	1621.851	3.33	0.0018	8.39983
P00747	0	0.9903	811.4282	1621.849	2.02	0.00164	7.061496
P00747	0	1	760.001	2277.988	1.97	0.0015	22.67575
P00747	0	0.9136	659.8961	1318.785	2.22	0.00147	7.322577
P00747	0	1	683.9794	2049.924	1.49	0.00102	13.0671
P00747	0	1	1025.466	2049.925	2	0.00205	0
P00747	0	1	683.979	2049.922	0.96	0.00065	67.77734
P00747	0	0.9216	694.6808	2082.028	1.92	0.00133	9.263757
P00747	0	1	713.33	2137.975	1.12	0.0008	2.39857
P00747	0	1	568.2732	2270.071	-1.36	-0.00077	13.89618
P00747	0	0.9355	535.2493	2137.975	1.2	0.00064	28.11711
P00747	0	1	568.2754	2270.08	2.51	0.00142	2.780312
P00747	0	1	757.3637	2270.077	1.12	0.00084	2.605635
P00747	0	1	614.8353	1228.663	0.77	0.00047	49.98136
P00747	0	0.9149	759.8026	1518.598	1.39	0.00105	3.557089
P00747	0	0.9111	476.9482	1428.83	-0.1	-0.00005	18.68519
P00747	0	0.8889	772.7379	2316.199	2.15	0.00166	3.467148
P00747	0	0.9841	579.8046	2316.197	1.14	0.00066	2.971408
P00747	0	0.9296	772.7374	2316.198	1.59	0.00123	4.763189
P00747	0	1	759.8024	1518.597	1.15	0.00087	2.512161
P00747	1	1	784.3832	2351.135	0.99	0.00078	3.662739
P00747	1	0.9394	474.2664	1894.044	0.49	0.00023	13.24818
P00747	0	0.9036	587.8457	1174.684	3.55	0.00208	26.21953
P00747	0	0.9833	694.6807	2082.028	1.83	0.00127	6.399704
P00747	0	1	506.8709	1518.598	1.68	0.00085	0
P00747	0	0.973	534.2643	1600.778	0.88	0.00047	0
P00747	0	1	534.2653	1600.781	2.71	0.00145	2.800555
P00747	0	0.9672	578.7842	1156.561	2.99	0.00173	6.694562
P00747	1	1	586.2994	1756.884	3.07	0.0018	2.029201
P00747	0	0.9167	616.8159	1232.625	2.56	0.00158	3.412278
P00747	0	1	660.8616	2640.425	0.81	0.00053	20.54673
Q02325; P0	0	1	513.2466	1537.725	2.13	0.00109	6.229238
Q02325; Q1	0	0.8	584.823	1168.639	2.92	0.00171	12.57676
P00747	1	1	538.2646	1612.779	1.92	0.00103	9.582102
Q02325; P0	0	0.8824	689.3164	1377.626	0.43	0.0003	0
P00747	0	0.641	411.5462	1232.624	2.15	0.00088	3.035464
P00747	0	0.9167	641.0834	2561.312	6.58	0.00421	44.3318
P00747	0	0.9024	440.2664	1318.785	1.96	0.00086	10.54698
P00747	0	1	614.2825	1227.558	2.72	0.00167	2.705444
P00747	0	1	614.2819	1227.557	1.73	0.00106	3.260478
Q15195; P0	1	0.9167	475.6111	1424.819	0.93	0.00044	26.07081
P00747	0	1	579.8057	2316.201	3.04	0.00176	53.3778
P00747	1	0.8571	411.6152	1232.831	0.77	0.00032	23.10683
P00747	0	0.9783	640.8325	1280.658	2.38	0.00152	6.1394
P00747	0	0.9429	579.8061	2316.203	3.67	0.00212	14.0905
P00747	0	1	409.8572	1227.557	1.99	0.00081	15.82752
Q02325; Q1	0	0.5833	390.2174	1168.638	2.1	0.00082	27.11907
P00747	0	1	800.8936	1600.78	1.73	0.00138	29.56833
P00747	1	1	439.9757	1756.881	1.61	0.00071	20.73718

P00747	0	1	481.5891	1442.753	1.22	0.00059	32.79889
P00747	0	0.963	481.589	1442.752	1.09	0.00053	72.65716
P00747	0	1	1211.549	2422.091	2.13	0.00258	5.020582
P00747	0	0.8222	411.5459	1232.623	1.48	0.00061	4.400372
P00747	0	0.6923	759.9997	2277.985	0.28	0.00021	0
P00747	0	1	614.2817	1227.556	1.33	0.00082	13.94768
P00747	0	0.9667	808.0374	2422.098	4.85	0.00391	14.40323
P00747	0	1	759.8032	1518.599	2.19	0.00166	0
P00747	0	0.9783	434.2262	1300.664	3.18	0.00138	9.721385
P00747	0	0.8261	481.5898	1442.755	2.87	0.00138	54.97773
P00747	0	1	506.8715	1518.6	2.83	0.00143	0
P00747	0	0.9	493.2537	1477.746	1.85	0.00091	30.06171
Q15195; P0	0	0.6585	569.3429	1137.679	2.41	0.00137	4.224358
P00747	0	1	687.752	1374.497	2.25	0.00155	3.964253
P00747	1	0.7647	448.9766	1792.885	1.14	0.00051	48.01406
Q15195; P0	0	0.6364	569.3433	1137.679	3.16	0.0018	14.98462
Q02325; Q1	0	0.8182	584.8233	1168.639	3.44	0.00201	7.360862
P00747	0	1	534.2646	1600.779	1.34	0.00071	25.1992
P00747	0	0.7407	476.9487	1428.831	0.99	0.00047	12.95988
P00747	0	0.95	616.8146	1232.622	0.38	0.00023	3.19825
Q02325; P0	0	0.9756	689.3164	1377.625	0.34	0.00023	17.78435
P00747	1	1	860.0686	2578.191	1.63	0.0014	1.81482
P00747	0	1	614.2816	1227.556	1.13	0.00069	0
P00747	1	0.8261	463.5798	1388.725	1.62	0.00075	33.17683
P00747	0	0.9512	650.8346	1300.662	1.62	0.00105	6.612808
P00747	0	0.9211	554.8056	1108.604	1.48	0.00082	9.773003
Q02325; P0	0	1	513.2463	1537.724	1.65	0.00085	12.06906
P00747	0	1	542.23	1083.453	0.28	0.00015	20.15745
P00747	0	1	433.5553	1298.651	1.98	0.00086	9.238378
Q02325; Q1	0	0.5833	390.2172	1168.637	1.55	0.00061	10.91142
P00747	0	1	452.2422	1354.712	1.81	0.00082	18.0479
P00747	0	0.9429	691.3756	1381.744	1.51	0.00105	0
P00747	0	1	488.7545	1951.996	1.23	0.0006	8.990506
P00747	0	1	808.0363	2422.094	3.49	0.00281	6.695964
Q02325; P0	0	1	513.2466	1537.725	2.25	0.00115	59.04612
P00747	0	1	543.7791	2172.095	1.22	0.00066	54.74105
P00747	0	1	481.5891	1442.753	1.41	0.00068	53.9159
P00747	1	0.4615	431.2716	1291.8	2.05	0.00088	26.72187
P00747	0	0.8043	544.765	1088.523	3.13	0.00171	5.396366
P00747	0	0.96	427.5569	1280.656	1.08	0.00046	13.96915
P00747	0	0.7778	595.887	2975.406	1.41	0.00084	33.00044
P00747	0	1	808.0319	2422.081	-1.96	-0.00158	23.8973
P00747	0	1	409.8572	1227.557	1.99	0.00081	24.90139
P00747	0	1	542.2302	1083.453	0.62	0.00033	0
P00747	0	0.9	440.2658	1318.783	0.64	0.00028	58.24977
P00747	0	0.8214	659.8959	1318.784	1.85	0.00122	13.02505
P00747	0	1	759.8037	1518.6	2.83	0.00215	33.44288
Q02325; Q1	0	0.44	390.2168	1168.636	0.38	0.00015	14.30095
P00747	0	1	808.0354	2422.092	2.35	0.0019	8.092862
P00747	1	1	729.9213	1458.835	0.15	0.00011	6.708826
P00747	0	1	427.5574	1280.658	2.22	0.00095	8.818822
P00747	1	1	588.5406	2351.141	3.27	0.00192	8.100157

P00747	0	1	409.8571	1227.557	1.84	0.00075	36.23005
P02768	0	0.871	733.7176	2199.138	1.1	0.00081	21.3637
P02768	0	0.907	733.7178	2199.139	1.43	0.00105	10.30752
P02768	0	0.6279	549.617	1646.836	1.61	0.00088	18.50071
P02768	0	0.7759	549.6168	1646.836	1.28	0.0007	8.822502
P02768	0	1	823.9217	1646.836	1.4	0.00115	6.772108
P02768	0	1	649.325	1945.96	1.91	0.00124	30.70978
P02768	0	0.7692	549.6164	1646.835	0.5	0.00027	48.32998
P02768	0	1	823.9234	1646.84	3.47	0.00286	13.53656
P02768	2	1	580.8442	2320.355	3.03	0.00176	2.231667
P02768	2	1	580.8442	2320.355	3.03	0.00176	2.231667
P02768	2	1	580.8442	2320.355	3.03	0.00176	2.231667
P02768	0	0.8485	644.9079	1288.809	0.52	0.00034	4.788717
P02768	0	1	558.4926	2230.949	4.05	0.00226	15.2604
P02768	0	1	893.8962	1786.785	1.47	0.00131	0
P02768	1	0.8846	460.619	1379.843	1.49	0.00068	17.70241
P02768	0	1	821.8445	1642.682	0.79	0.00065	0
P02768	1	1	644.6516	1931.94	1.15	0.00074	0
P02768	1	0.88	448.6027	1343.794	0.74	0.00033	7.348913
P02768	0	0.8043	549.616	1646.833	-0.28	-0.00015	0
P02768	0	0.7843	549.6169	1646.836	1.5	0.00082	5.454378
P02768	0	0.8364	549.6162	1646.834	0.17	0.00009	0
P02768	0	0.8	549.6172	1646.837	1.95	0.00107	14.95914
P02768	0	0.7391	549.6169	1646.836	1.5	0.00082	7.448346
P02768	0	0.8594	544.2855	1630.842	1.8	0.00098	0
P02768	0	1	1088.191	3262.559	3.17	0.00344	0
P02768	0	0.7442	544.2846	1630.839	0.12	0.00006	0
P02768	0	0.9896	815.9249	1630.842	2.19	0.00178	2.43451
P02768	0	0.875	544.2852	1630.841	1.35	0.00074	2.697887
P02768	0	1	815.9241	1630.841	1.21	0.00099	5.672572
P02768	0	0.8644	544.2853	1630.841	1.47	0.0008	7.664101
P02768	0	1	815.9226	1630.838	-0.58	-0.00048	5.110016
P02768	0	0.8806	544.2844	1630.839	-0.22	-0.00012	6.372628
P02768	0	0.9388	815.9256	1630.844	3.08	0.00251	0
P02768	0	0.8438	544.2848	1630.84	0.57	0.00031	0
P02768	1	1	537.9871	1611.947	1.05	0.00056	17.51748
P02768	1	0.9623	489.9529	1467.844	0.7	0.00034	4.440347
P02768	1	0.7391	403.7423	1611.947	1.34	0.00054	8.245259
P02768	1	0.7442	403.7423	1611.948	1.49	0.0006	2.568454
P02768	1	1	537.9867	1611.946	0.25	0.00014	3.542316
P02768	1	1	537.9872	1611.947	1.28	0.00069	21.85099
P02768	1	1	537.9874	1611.948	1.5	0.00081	3.857814
P02768	1	1	537.987	1611.946	0.82	0.00044	4.672218
P02768	0	0.8974	733.717	2199.137	0.35	0.00026	3.277838
P02768	0	1	733.7169	2199.136	0.18	0.00013	8.457705
P02768	0	0.9286	733.717	2199.137	0.35	0.00026	3.277838
P02768	0	0.9032	733.7175	2199.138	0.93	0.00068	20.36963
P02768	0	0.9143	685.6838	2055.037	1.57	0.00107	3.619745
P02768	0	0.8837	550.5397	2199.137	0.59	0.00033	3.605744
P02768	0	0.8929	550.54	2199.138	1.04	0.00057	24.56736
P02768	0	0.9394	550.5402	2199.139	1.37	0.00075	5.131604
P02768	0	0.9245	733.7178	2199.139	1.35	0.00099	3.673026

P02768	0	0.8	719.4133	1437.819	0.08	0.00006	5.628948
P02768	0	0.9825	479.945	1437.82	0.92	0.00044	7.775204
P02768	0	0.9254	719.4133	1437.819	0.17	0.00012	5.613877
P02768	0	0.9434	479.9448	1437.82	0.48	0.00023	7.297089
P02768	0	1	823.9223	1646.837	2.14	0.00176	21.19716
P02768	0	0.7843	649.3251	1945.961	2.09	0.00136	5.278109
P02768	0	1	973.4839	1945.961	1.91	0.00186	4.78686
P02768	0	1	649.3241	1945.958	0.49	0.00032	6.30794
P02768	1	0.8889	489.9527	1467.843	0.27	0.00013	56.08204
P02768	1	0.9216	643.3865	1928.145	1.56	0.001	21.80333
P02768	1	0.766	472.973	1416.904	1.14	0.00054	5.472119
P02768	1	0.766	472.973	1416.904	1.14	0.00054	5.472119
P02768	1	1	643.3864	1928.145	1.46	0.00094	30.8559
P02768	0	1	596.2678	1786.789	3.55	0.00211	0
P02768	0	0.9588	653.3171	3262.556	2.35	0.00154	1.038449
P02768	0	0.99	1088.192	3262.563	4.29	0.00466	5.510254
P02768	1	1	537.9866	1611.945	0.14	0.00008	30.72844
P02768	0	0.8714	820.3919	3278.546	0.62	0.00051	4.463821
P02768	0	1	1093.523	3278.554	3.06	0.00335	0
P02768	0	0.8269	820.3926	3278.549	1.59	0.0013	0.894572
P02768	0	1	558.4915	2230.944	2.08	0.00116	0
P02768	1	1	643.3865	1928.145	1.65	0.00106	16.6461
P02768	1	1	643.3862	1928.144	1.08	0.00069	6.089679
P02768	1	1	482.7914	1928.144	1.01	0.00049	3.559402
P02768	1	1	964.5762	1928.145	1.72	0.00166	5.087317
P02768	1	1	643.3856	1928.142	0.23	0.00014	6.46519
P02768	1	1	482.7925	1928.148	3.35	0.00162	21.36076
P02768	1	1	643.3915	1928.16	9.34	0.006	36.09347
P02768	0	1	893.8964	1786.786	1.68	0.0015	2.820012
P02768	0	1	596.2673	1786.787	2.73	0.00162	7.067748
P02768	0	1	744.3204	2230.947	3.12	0.00232	4.436355
P02768	0	0.8226	653.3753	1305.743	2.17	0.00142	22.60959
P02768	0	1	435.9191	1305.743	1.79	0.00078	0
P02768	0	0.9412	435.9189	1305.742	1.16	0.0005	7.045504
P02768	0	0.9688	435.9187	1305.742	0.88	0.00038	7.138199
P02768	0	0.9	653.3757	1305.744	2.83	0.00185	14.84928
P02768	0	0.9412	435.9189	1305.742	1.16	0.0005	5.825669
P02768	0	0.7778	653.3754	1305.743	2.27	0.00148	11.64397
P02768	0	0.9375	435.9186	1305.741	0.6	0.00026	6.615524
P02768	0	0.9818	485.9536	1455.846	1.5	0.00073	1.54249
P02768	0	1	828.4772	1655.947	1.34	0.00111	2.927242
P02768	0	1	733.7159	2199.133	-1.15	-0.00084	60.95162
P02768	0	1	552.6542	1655.948	2	0.0011	4.395019
P02768	1	1	595.353	1784.045	2.65	0.00157	3.641001
P02768	1	1	643.3867	1928.146	1.93	0.00124	13.14684
P02768	1	0.619	521.0068	1561.006	0.69	0.00036	9.167002
P02768	1	0.6562	391.0069	1561.006	0.64	0.00025	31.00209
P02768	1	0.5455	521.0071	1561.007	1.16	0.0006	9.427256
P02768	1	0.6667	781.0068	1561.006	0.9	0.0007	8.717592
P02768	1	0.6154	650.9818	1950.931	0.78	0.00051	0
P02768	1	0.6154	650.9818	1950.931	0.78	0.00051	0
P02768	1	1	595.353	1784.045	2.65	0.00157	3.641001

P02768	0	1	596.2669	1786.786	2.01	0.0012	8.310125
P02768	1	1	482.7914	1928.144	1.01	0.00049	5.38532
P02768	1	1	643.3858	1928.143	0.51	0.00033	7.451637
P02768	1	1	482.7912	1928.143	0.63	0.0003	19.87566
P02768	1	1	643.3873	1928.147	2.88	0.00185	41.33638
P02768	0	0.9535	980.1622	2938.472	1.37	0.00134	4.230921
P02768	0	0.9167	735.3735	2938.472	1.35	0.00099	3.222918
P02768	0	0.9815	735.3735	2938.472	1.35	0.00099	2.546407
P02768	0	0.8529	735.3729	2938.47	0.52	0.00038	4.430845
P02768	0	0.9362	778.4389	2333.302	1.14	0.00089	4.090406
P02768	0	0.9362	778.4389	2333.302	1.14	0.00089	4.090406
P02768	0	0.8846	735.3733	2938.471	1.1	0.00081	3.44506
P02768	0	0.8947	980.1629	2938.474	2.06	0.00202	3.499089
P02768	0	0.9231	735.3734	2938.472	1.18	0.00087	7.363093
P02768	0	0.9375	980.1626	2938.473	1.75	0.00171	4.158357
P02768	0	0.9184	735.3731	2938.471	0.85	0.00062	2.467205
P02768	0	0.9429	980.1615	2938.47	0.62	0.00061	1.557643
P02768	0	0.9322	735.3731	2938.471	0.85	0.00062	2.388836
P02768	0	0.9245	735.3734	2938.472	1.18	0.00087	3.86522
P02768	0	1	620.1063	2477.403	0.67	0.00042	4.946644
P02768	0	1	826.4722	2477.402	0.16	0.00013	4.95781
P02768	0	1	826.4724	2477.403	0.38	0.00031	4.688102
P02768	0	1	620.1065	2477.404	1.06	0.00066	4.122445
P02768	0	1	826.473	2477.404	1.12	0.00092	42.86511
P02768	0	1	620.1069	2477.406	1.75	0.00109	5.987775
P02768	0	1	826.4728	2477.404	0.9	0.00074	38.59062
P02768	0	0.9697	739.3727	2954.469	1.99	0.00147	3.378802
P02768	0	1	850.081	2548.228	0.64	0.00054	7.304874
P02768	0	0.8857	637.8133	2548.231	1.78	0.00114	2.541619
P02768	0	1	850.0814	2548.23	1.14	0.00097	1.545759
P02768	0	0.8182	699.3486	2794.373	2.38	0.00166	1.65631
P02768	0	1	850.0814	2548.23	1.07	0.00091	5.446584
P02768	0	1	637.8133	2548.231	1.78	0.00114	31.80723
P02768	0	1	850.0812	2548.229	0.85	0.00073	22.62414
P02768	0	1	1016.055	2031.103	2.27	0.00231	14.02532
P02768	2	1	551.7893	2204.135	0.03	0.00002	11.82606
P02768	0	0.899	758.3395	1515.672	1.88	0.00143	5.358093
P02768	0	0.9677	677.7052	2031.101	1.39	0.00094	7.472669
P02768	0	1	696.2851	2086.841	1.53	0.00107	0.463014
P02768	0	1	522.4655	2086.84	1.18	0.00061	0.275699
P02768	0	1	696.2847	2086.84	1.01	0.0007	0
P02768	1	0.8936	681.8779	1362.748	1.29	0.00088	8.390205
P02768	0	0.9848	636.851	1272.695	1.64	0.00104	23.4501
P02768	0	0.8478	682.3554	1363.704	0.35	0.00024	4.203078
P02768	0	1	614.2736	1840.806	2.31	0.00142	3.627242
P02768	0	1	460.9561	1840.803	0.41	0.00019	17.01606
P02768	0	1	614.2729	1840.804	1.22	0.00075	6.553154
P02768	0	1	641.8046	1282.602	1.53	0.00098	5.355431
P02768	0	1	596.2679	1786.789	3.65	0.00217	18.91643
P02768	0	0.9718	830.3897	1659.772	0.76	0.00063	5.996527
P02768	0	0.9583	553.9296	1659.774	2.07	0.00115	7.297066
P02768	0	1	579.9804	1737.926	1.47	0.00085	10.43756

P02768	0	1	579.3555	1157.704	2.27	0.00131	1.320563
P02768	1	0.8611	547.5558	2187.201	0.91	0.0005	3.55343
P02768	1	0.88	729.7388	2187.202	1.19	0.00087	4.621848
P02768	1	0.8966	729.7386	2187.201	0.86	0.00063	21.7097
P02768	0	1	455.2398	1363.705	1.32	0.0006	7.525749
P02768	1	0.7143	547.5571	2187.206	3.25	0.00178	0
P02768	1	0.8824	729.7386	2187.201	0.86	0.00063	20.24694
P02768	1	1	681.7046	2043.099	0.96	0.00065	39.23035
P02768	0	1	577.9543	1731.848	1.24	0.00071	7.592054
P02768	0	1	866.4277	1731.848	1.1	0.00095	5.000917
P02768	0	1	577.955	1731.851	2.5	0.00145	4.271048
P02768	0	0.7021	620.3278	1239.648	1.96	0.00122	6.874068
P02768	0	0.7442	620.3271	1239.647	0.78	0.00049	5.200776
P02768	0	1	866.4279	1731.849	1.38	0.0012	5.51892
P02768	0	1	744.3187	2230.942	0.91	0.00068	2.018012
P02768	0	1	558.4911	2230.943	1.31	0.00073	7.571762
P02768	0	1	558.4915	2230.944	2.08	0.00116	2.285467
P02768	0	1	744.3188	2230.942	0.99	0.00074	1.424317
P02768	0	1	558.4904	2230.94	0.11	0.00006	3.343254
P02768	0	1	579.3544	1157.701	0.37	0.00021	2.687329
P02768	0	1	744.3192	2230.943	1.48	0.0011	4.571673
P02768	0	0.9592	584.8278	1168.648	2.71	0.00158	27.78719
P02768	0	1	744.3192	2230.943	1.48	0.0011	5.908863
P02768	0	1	558.4915	2230.944	1.97	0.0011	2.546326
P02768	0	1	579.3553	1157.703	1.95	0.00113	17.39894
P02768	0	1	558.4909	2230.942	0.98	0.00055	24.3815
P02768	0	1	596.2662	1786.784	0.78	0.00047	5.851484
P02768	1	1	553.642	1658.912	0.1	0.00006	10.05854
P02768	0	1	596.2664	1786.785	1.09	0.00065	6.068861
P02768	0	1	566.2394	1696.704	2.23	0.00126	21.46559
P02768	0	1	596.2669	1786.786	1.91	0.00114	6.373458
P02768	0	1	893.8954	1786.783	0.52	0.00046	4.145344
P02768	1	0.9773	553.6429	1658.914	1.76	0.00097	9.041361
P02768	0	1	569.754	1138.501	2.37	0.00135	11.57822
P02768	1	0.8667	544.2874	1630.848	0.28	0.00015	14.15329
P02768	0	0.8788	679.8294	1358.652	1.11	0.00075	4.70115
P02768	0	0.8769	679.8299	1358.653	1.83	0.00124	4.704859
P02768	0	0.8769	679.8292	1358.651	0.75	0.00051	4.650176
P02768	0	1	580.2503	1738.736	2.25	0.0013	6.976834
P02768	0	1	821.8452	1642.683	1.53	0.00126	8.730055
P02768	0	1	580.2504	1738.737	2.46	0.00142	12.78538
P02768	0	1	869.8706	1738.734	0.85	0.00074	8.303005
P02768	0	0.8769	679.8301	1358.653	2.19	0.00149	10.44248
P02768	1	1	477.3047	1429.9	1.08	0.00051	9.018848
P02768	0	1	893.8964	1786.786	1.68	0.0015	0
P02768	0	1	596.2669	1786.786	2.01	0.0012	4.968367
P02768	0	1	589.9696	1767.894	2.61	0.00154	6.634508
P02768	0	1	893.8958	1786.784	0.92	0.00083	7.419555
P02768	0	1	941.4791	2822.423	3.45	0.00325	14.59891
P02768	1	0.1111	495.5316	1979.104	0.73	0.00036	12.45738
P02768	1	1	483.7402	1931.939	0.42	0.0002	8.158053
P02768	0	1	936.1461	2806.424	2.01	0.00188	4.97189

P02768	0	1	702.3608	2806.421	1.17	0.00082	5.141513
P02768	1	0.9322	644.6512	1931.939	0.49	0.00032	8.407876
P02768	1	1	644.6494	1931.934	-2.35	-0.00152	71.44086
P02768	1	1	644.6503	1931.936	-0.84	-0.00054	63.50877
P02768	1	1	644.6505	1931.937	-0.55	-0.00036	0
P02768	1	1	966.4739	1931.94	1.25	0.00121	4.989229
P02768	1	1	483.74	1931.938	0.04	0.00002	9.644781
P02768	0	1	485.9534	1455.846	1.12	0.00055	10.22746
P02768	1	1	644.6506	1931.937	-0.46	-0.0003	8.38799
P02768	0	1	574.919	1722.742	2.86	0.00164	9.447586
P02768	0	1	574.9182	1722.74	1.37	0.00079	6.84208
P02768	0	1	861.8734	1722.739	1.1	0.00094	5.339647
P02768	0	1	574.9171	1722.737	-0.43	-0.00025	6.714633
P02768	0	1	861.8728	1722.738	0.46	0.0004	5.362154
P02768	0	1	574.9176	1722.738	0.42	0.00024	6.192368
P02768	0	1	861.8727	1722.738	0.32	0.00027	4.111784
P02768	1	1	644.6499	1931.935	-1.5	-0.00097	16.30419
P02768	1	1	644.651	1931.938	0.21	0.00013	20.82194
P02768	1	1	644.6494	1931.934	-2.26	-0.00145	19.64737
P02768	0	1	789.8226	1578.638	1.59	0.00126	30.54846
P02768	1	1	644.6484	1931.931	-3.87	-0.00249	21.65026
P02768	1	1	483.7401	1931.938	0.17	0.00008	41.05858
P02768	0	1	574.918	1722.739	1.05	0.0006	35.43832
P02768	0	1	435.2369	1737.926	1.01	0.00044	8.666703
P02768	1	1	489.954	1467.847	2.89	0.00141	14.78901
P02768	1	0.9355	644.6502	1931.936	-1.03	-0.00066	30.63243
P02768	0	0.8919	719.4152	1437.823	2.72	0.00195	39.27457
P02768	0	0.7719	539.3418	1077.676	0.51	0.00027	23.71292
P02768	0	0.7436	453.5551	1358.651	0.43	0.00019	0
P02768	0	0.7179	549.6159	1646.833	-0.39	-0.00021	0
P02768	0	0.7586	539.3428	1077.678	2.32	0.00125	7.428948
P02768	0	0.9661	512.7762	1024.545	1.91	0.00098	20.24165
P02768	0	1	455.2398	1363.705	1.32	0.0006	9.82896
P02768	1	1	448.6027	1343.793	0.67	0.0003	17.70633
P02768	0	1	743.8731	1486.739	1.42	0.00106	23.9387
P02768	0	0.8036	539.3414	1077.675	-0.29	-0.00015	10.49364
P02768	0	1	566.2388	1696.702	1.26	0.00071	73.08008
P02768	0	1	435.9193	1305.743	2.14	0.00093	13.98603
P02768	0	1	596.2671	1786.787	2.32	0.00138	7.788616
P02768	0	0.8571	636.8496	1272.692	-0.57	-0.00036	4.572615
P02768	0	0.7838	453.5553	1358.651	0.97	0.00044	9.872382
P02768	1	0.9412	681.8777	1362.748	1.02	0.00069	40.20883
P02768	0	0.9	512.7764	1024.546	2.38	0.00122	23.0758
P02768	0	1	553.9294	1659.774	1.74	0.00096	26.08163
P02768	0	0.625	549.6165	1646.835	0.72	0.0004	62.64732
P02768	0	0.8889	1093.521	3278.547	1.16	0.00127	4.071406
P02768	1	0.8542	806.4769	1611.947	0.91	0.00073	7.701343
P02768	0	0.8125	584.8261	1168.645	-0.32	-0.00019	5.558077
P02768	0	0.7857	539.3424	1077.678	1.64	0.00088	22.84634
P02768	0	1	744.3183	2230.94	0.33	0.00025	0
P02768	0	1	553.9291	1659.773	1.19	0.00066	47.67428
P02768	1	0.8333	547.5559	2187.202	1.02	0.00056	2.601221

P02768	0	1	821.8458	1642.684	2.35	0.00193	0
P02768	1	0.4286	388.5662	1163.684	2.97	0.00115	25.86027
P02768	0	1	455.2399	1363.705	1.52	0.00069	15.55305
P02768	0	1	435.9189	1305.742	1.3	0.00056	5.106997
P02768	0	0.7907	430.2745	1288.809	0.87	0.00037	7.16763
P02768	0	0.9815	610.3048	1219.602	1	0.00061	2.182498
P02768	0	0.7857	539.3412	1077.675	-0.63	-0.00034	28.31632
P02768	1	0.9318	596.6186	1787.841	2.93	0.00174	3.456623
P02768	1	0.125	596.6182	1787.84	2.31	0.00138	6.550745
P02768	1	0.5517	521.0073	1561.007	1.63	0.00085	15.29519
P02768	0	1	580.2504	1738.737	2.35	0.00136	20.15846
P02768	0	1	455.2396	1363.704	0.78	0.00036	49.98822
P02768	0	0.7941	552.8367	1104.666	1.44	0.0008	18.99468
P02768	0	0.8696	850.0805	2548.227	0.06	0.00005	0
P02768	1	0.9318	596.6186	1787.841	2.93	0.00174	3.456623
P02768	0	0.6667	430.2747	1288.81	1.29	0.00055	0
P02768	0	1	542.7798	1084.552	1.79	0.00097	2.749371
P02768	0	0.7857	539.3422	1077.677	1.19	0.00064	4.301626
P02768	0	1	542.7795	1084.552	1.34	0.00073	3.627981
P02768	1	1	644.6497	1931.934	-1.88	-0.00121	71.1075
P02768	0	0.8571	637.8138	2548.234	2.65	0.00169	59.17595
P02768	0	0.6389	544.2857	1630.843	2.25	0.00122	9.035712
P02768	1	0.85	644.6536	1931.946	4.19	0.0027	15.9724
P02768	0	1	577.9554	1731.852	3.24	0.00187	42.2208
P02768	0	0.8409	539.3417	1077.676	0.39	0.00021	28.96143
P02768	0	0.5957	620.3273	1239.647	1.18	0.00073	14.23868
P02768	0	0.6977	539.3143	1077.621	0.66	0.00035	0
P02768	0	1	435.9189	1305.742	1.3	0.00056	25.39859
P02768	0	0.8837	539.3144	1077.622	0.77	0.00042	100
P02768	1	1	596.6188	1787.842	3.23	0.00193	9.187243
P02768	1	1	537.9858	1611.943	-1.34	-0.00072	0
P02768	0	0.7407	549.6166	1646.835	0.83	0.00046	0
P02768	0	1	428.2077	1282.608	6.59	0.00282	31.12209
P02768	0	1	553.9331	1659.785	8.36	0.00463	45.86778
P02768	0	0.85	980.1629	2938.474	2.06	0.00202	3.013132
P02768	0	1	522.4659	2086.842	2.11	0.0011	10.09212
P02768	0	0.82	539.3433	1077.679	3.34	0.0018	31.63962
P02768	0	0.8889	544.2843	1630.838	-0.33	-0.00018	20.10644
P02768	1	0.9375	483.7404	1931.94	0.92	0.00045	3.093818
P02768	1	1	715.4534	1429.9	1.07	0.00076	28.4628
P02768	0	1	893.895	1786.783	0.11	0.00009	0
P02768	0	1	435.9181	1305.74	-0.67	-0.00029	4.225839
P02768	1	1	537.9888	1611.952	4.12	0.00221	51.33878
P02768	0	0.8409	539.3422	1077.677	1.19	0.00064	28.95014
P02768	0	1	542.7794	1084.552	1.12	0.00061	3.349341
P02768	2		551.7908	2204.141	2.8	0.00154	27.97959
P02768	1	1	596.6188	1787.842	3.23	0.00193	9.187243
P02768	0	0.8824	620.1074	2477.408	2.44	0.00151	15.47137
P02768	1	0.6522	403.7427	1611.949	2.48	0.001	46.24142
P02768	0	1	455.2394	1363.704	0.38	0.00017	3.60887
P02768	0	1	564.8002	1128.593	2.29	0.00129	24.55001
P02768	0	0.1579	778.4388	2333.302	1.06	0.00083	0

P02768	0	1	522.4655	2086.84	1.29	0.00067	0
P02768	0	0.8636	649.325	1945.96	1.81	0.00118	3.228806
P02768	0	0.8421	539.3146	1077.622	1.11	0.0006	100
P02768	0	0.7143	733.7184	2199.141	2.26	0.00166	32.43684
P02768	0	0.9259	564.7999	1128.592	1.75	0.00099	4.327916
P02768	0	0.5909	549.6186	1646.841	4.5	0.00247	80.52822
P02768	0	1	580.2507	1738.737	2.88	0.00167	23.58636
P02768	0	1	580.2496	1738.734	1.09	0.00063	16.43735
P02768	1	0.9333	454.9209	1362.748	0.99	0.00045	42.73316
P02768	0	1	580.2502	1738.736	2.14	0.00124	19.26483
P02768	0	0.8039	539.3419	1077.677	0.73	0.00039	23.47288
P02768	0	1	789.8221	1578.637	0.97	0.00077	44.37389
P02768	0	1	580.2506	1738.737	2.77	0.00161	16.0844
P02768	0	0.84	549.6177	1646.839	2.95	0.00162	0
P02768	0	1	596.267	1786.786	2.11	0.00126	59.69704
P02768	0	1	428.2061	1282.604	2.8	0.0012	52.10535
P02768	0	1	564.8007	1128.594	3.16	0.00178	0
P02768	1	0.9565	483.7406	1931.941	1.3	0.00063	28.05309
P02768	0	1	542.7778	1084.548	-1.81	-0.00098	0
P02768	1	1	483.7402	1931.939	0.42	0.0002	43.02632
P02768	1	1	483.7398	1931.937	-0.34	-0.00016	50.73358
P02768	1	1	483.7399	1931.938	-0.09	-0.00004	39.78196
P02768	1	0.92	483.7404	1931.94	0.86	0.00042	19.38438
P02768	0	1	529.9205	1587.747	1.74	0.00092	10.32073
P02768	1	0.875	436.5997	1307.784	1.39	0.00061	24.9861
P02768	0	1	435.9193	1305.743	2.14	0.00093	0
P02768	0	0.7674	413.8872	1239.647	0.85	0.00035	7.137344
P02768	2	0.9744	515.764	2060.034	0.52	0.00027	5.000806
P02768	0	0.6842	647.364	1293.721	2.74	0.00178	7.428512
P02768	0	0.5652	480.7853	960.5632	0.72	0.00035	29.4296
P02768	0	0.8696	850.0811	2548.229	0.71	0.0006	12.98415
P02768	0	0.6061	620.3275	1239.648	1.37	0.00085	49.46403
P02768	0	0.875	637.8133	2548.231	1.78	0.00114	14.4873
P02768	1	0.75	454.9201	1362.746	-0.76	-0.00034	7.269978
P02768	2	0.1579	507.0409	2025.142	2.28	0.00116	9.550075
P02768	0	0.8696	850.0813	2548.229	1	0.00085	9.173298
P02768	0	1	507.3041	1013.601	1.88	0.00095	7.372126
P02768	0	1	542.779	1084.551	0.33	0.00018	2.535678
P02768	1	0.68	644.6502	1931.936	-1.03	-0.00066	37.70908
P02768	0	0.9444	407.2057	1219.603	1.24	0.0005	4.587513
P02768	0	0.6977	548.2762	1095.545	1.12	0.00061	6.410838
P02768	0	0.8864	564.7999	1128.593	1.86	0.00105	6.011661
P02768	0	0.8571	550.5404	2199.14	1.81	0.001	13.19618
P02768	1	0.64	391.0072	1561.007	1.43	0.00056	27.2179
P02768	0	1	455.2401	1363.706	1.86	0.00084	0
P02768	0	1	744.3193	2230.943	1.65	0.00122	29.75278
P02768	0	0.3429	531.9458	1593.823	0.62	0.00033	7.30972
P02768	0	0.5238	980.1651	2938.481	4.3	0.00421	5.669075
P02768	0	0.7037	1239.204	2477.401	-0.25	-0.00032	0
P02768	0	1	679.8304	1358.654	2.64	0.00179	0
P02768	0	0.8148	553.9294	1659.774	1.63	0.0009	22.5912
P02768	0	0.7826	620.1069	2477.406	1.75	0.00109	1.669948

P02768	2	1	551.7914	2204.144	4.02	0.00222	4.86042
P02768	0	0.5714	588.5004	2938.473	1.67	0.00098	0
P02768	0	0.8333	850.0808	2548.228	0.42	0.00036	0
P02768	0	0.8148	467.2911	933.575	1.38	0.00065	23.97549
P02768	0	1	435.9202	1305.746	4.17	0.00182	19.71195
P02768	0	1	607.7795	1214.552	3.16	0.00192	0
P02768	0	0.9	390.2202	1168.646	0.76	0.0003	9.379023
P02768	0	1	815.924	1630.841	1.14	0.00093	40.3561
P02768	0	1	744.3176	2230.938	-0.57	-0.00042	0
P02768	1	1	483.7397	1931.937	-0.65	-0.00032	50.08033
P02768	0	1	558.4928	2230.949	4.38	0.00244	14.19997
P02768	0	1	580.2492	1738.733	0.25	0.00014	69.09566
P02768	0	0.2353	778.439	2333.302	1.22	0.00095	8.940798
P02768	0	0.7949	581.3242	1161.641	2.35	0.00136	8.965211
P02768	0	0.25	637.8142	2548.235	3.12	0.00199	51.99315
P02768	0	0.5926	453.5547	1358.65	-0.38	-0.00017	0
P02768	2	0.9744	515.764	2060.034	0.52	0.00027	5.000806
P02768	0	0.7073	647.3658	1293.724	5.57	0.00361	0
P02768	0	0.6842	572.8579	1144.709	2.41	0.00138	14.63735
P02768	0	0.5778	480.7862	960.5652	2.75	0.00132	11.97689
P02768	0	1	696.2856	2086.842	2.32	0.00162	0
P02768	0	0.9697	743.8736	1486.74	2.08	0.00154	6.001875
P02768	2	1	441.6334	2204.138	1.33	0.00059	10.21609
P02768	0	0.9	653.3763	1305.745	3.67	0.00239	18.04017
P02768	0	1	542.7791	1084.551	0.44	0.00024	2.516496
P02768	0	0.8235	826.475	2477.411	3.63	0.003	37.47957
P02768	0	1	564.8004	1128.594	2.72	0.00154	31.17029
P02768	0	1	596.2671	1786.787	2.32	0.00138	68.96686
P02768	0	0.2727	387.8844	1161.639	0.18	0.00007	71.88502
P02768	1	0.9091	643.3872	1928.147	2.6	0.00167	4.356931
P02768	0	0.9048	424.9024	1272.693	0.16	0.00007	3.625645
P02768	1	0.2083	521.0057	1561.002	-1.54	-0.0008	22.31177
P02768	0	0.7143	539.3145	1077.622	0.88	0.00048	5.960021
P02768	0	0.8	649.3222	1945.952	-2.42	-0.00157	46.14616
P02768	0	1	584.8269	1168.647	1.14	0.00067	10.77093
P02768	0	0.7059	699.3487	2794.373	2.47	0.00172	17.04577
P02768	1	1	644.6522	1931.942	2.1	0.00135	7.51436
P02768	0	1	861.8739	1722.741	1.73	0.00149	0
P02768	0	0.7949	552.8356	1104.664	-0.54	-0.0003	3.278935
P02768	0	0.6923	480.7854	960.5635	1.04	0.0005	3.813688
P02768	0	1	552.6544	1655.949	2.22	0.00122	36.04953
P02768	0	0.96	413.8877	1239.648	2.03	0.00084	0
P02768	2	1	441.6335	2204.138	1.54	0.00068	13.85383
P02768	0	1	580.2496	1738.734	0.98	0.00057	9.17481
P02768	0	1	580.2509	1738.738	3.2	0.00185	31.53621
P02768	1	0.9524	482.7922	1928.147	2.66	0.00128	0
P02768	0	0.4	850.08	2548.225	-0.58	-0.0005	0
P02768	0	1	455.2395	1363.704	0.72	0.00033	28.61316
P02768	0	0.8	637.8133	2548.231	1.78	0.00114	21.33987
P02768	2	1	580.8454	2320.36	5.14	0.00298	21.81264
P02768	2	1	580.8454	2320.36	5.14	0.00298	21.81264
P02768	2	1	580.8454	2320.36	5.14	0.00298	21.81264

P02768	0	1	485.9538	1455.847	2	0.00097	3.911112
P02768	0	1	1239.206	2477.404	1.12	0.00139	7.344357
P02768	0	0.8571	637.8118	2548.225	-0.52	-0.00033	
P02768	0	1	564.7995	1128.592	1.1	0.00062	51.88667
P02768	0	0.6667	644.9088	1288.81	1.94	0.00125	36.3767
P02768	0	0.7778	815.9248	1630.842	2.11	0.00172	31.97009
P02768	0	0.8636	539.3138	1077.62	-0.25	-0.00013	2.679921
P02768	0	1	539.3142	1077.621	0.32	0.00017	37.06232
P02768	0	1	580.2504	1738.737	2.35	0.00136	36.53078
P02768	1	0.8235	483.7402	1931.939	0.48	0.00023	13.91497
P02768	0	0.92	387.8849	1161.64	1.36	0.00053	18.04419
P02768	0	1	387.8849	1161.64	1.52	0.00059	4.82237
P02768	0	0.5	550.54	2199.138	1.15	0.00063	6.216532
P02768	0		588.5004	2938.473	1.57	0.00092	3.169176
P02768	0	0.6667	637.8136	2548.233	2.26	0.00144	67.94438
P02768	0	1	558.4919	2230.946	2.74	0.00153	11.08828
P02768	0	1	428.2068	1282.606	4.44	0.0019	14.55579
P02768	0	1	574.9189	1722.742	2.65	0.00152	21.98879
P02768	0	1	580.2506	1738.737	2.77	0.00161	19.36002
P02768	0	0.9259	542.7785	1084.55	-0.57	-0.00031	25.97374
P02768	0	0.25	544.2852	1630.841	1.24	0.00068	5.780238
P02768	0	0.5758	480.7852	960.5631	0.59	0.00029	54.5745
P02768	0	1	505.895	1515.67	0.99	0.0005	46.53205
P01031	0	0.7288	731.9316	1462.856	3.49	0.00255	13.47209
P01031	0	1	562.2818	1684.831	2.45	0.00137	7.348059
P01031	0	0.8947	842.9185	1684.83	1.8	0.00152	3.331119
P01031	0	1	562.2819	1684.831	2.56	0.00144	15.15485
P01031	0	0.85	513.9574	1539.858	1.36	0.0007	8.26269
P01031	0	0.7358	770.4342	1539.861	3.63	0.00279	0
P01031	0	1	589.9637	1767.877	6.62	0.0039	33.93773
P01031	0	0.9787	614.3645	1227.722	3.24	0.00199	9.977339
P01031	0	0.9792	409.9115	1227.72	1.79	0.00073	3.042572
P01031	0	0.8085	465.9236	1395.756	2.01	0.00094	4.820879
P01031	0	0.8	551.6604	1652.967	3.18	0.00175	41.33758
P01031	0	0.9048	413.9969	1652.966	2.55	0.00105	4.007162
P01031	0	0.2609	514.3294	1540.974	4.67	0.0024	36.49748
P01031	0	0.7333	385.9972	1540.967	0.3	0.00012	4.471771
P01031	0	0.3793	514.3288	1540.972	3.48	0.00179	8.48204
P01031	0	1	1219.63	3656.874	-0.03	-0.00004	0
P01031	0	1	665.8359	1330.664	2.2	0.00146	0
P01031	0	0.8889	675.8896	1350.772	1.88	0.00127	6.856209
P01031	0	0.5333	612.8778	1224.748	3.29	0.00202	6.191976
P01031	0	0.9884	709.7255	2127.162	2	0.00142	4.760758
P01031	0	1	1064.084	2127.162	1.92	0.00204	0
P01031	0	0.9664	1064.083	2127.159	0.89	0.00094	14.13525
P01031	0	1	1183.156	2365.304	1.46	0.00172	0
P01031	0	0.9737	789.107	2365.306	2.48	0.00196	5.878727
P01031	0	0.6818	697.8812	1394.755	2.43	0.00169	5.350134
P01031	0	0.8906	483.2304	1447.676	-3.25	-0.00157	25.57495
P01031	0	0.9538	684.3508	1367.694	1.42	0.00097	7.562445
P01031	0	1	1095.075	2189.143	5.75	0.0063	
P01031	0	1	730.382	2189.132	0.68	0.00049	6.877311

P01031	0	1	842.8656	2526.582	1.39	0.00117	12.85988
P01031	0	1	632.4011	2526.582	1.46	0.00092	11.64872
P01031	1	1	856.3585	2567.061	-0.44	-0.00038	0
P01031	1	0.7317	445.2861	1333.844	1.62	0.00072	19.04945
P01031	0	1	805.4287	2414.272	4.03	0.00325	2.2105
P01031	0	1	862.4313	2585.279	0.25	0.00022	19.74035
P01031	0	0.9104	531.2668	1591.786	1.6	0.00085	5.332033
P01031	0	1	599.0184	1795.041	4.57	0.00273	28.34602
P01031	0	0.9762	549.9952	1647.971	2.29	0.00126	15.34928
P01031	0	1	613.643	1838.914	3.28	0.00201	8.181591
P01031	0	1	753.7267	2259.166	2.15	0.00162	3.001609
P01031	0	1	1130.087	2259.167	2.61	0.00295	0
P01031	0	1	936.5189	2807.542	-0.39	-0.00037	17.29827
P01031	0	1	728.3403	1455.673	1.62	0.00118	0
P01031	0	1	1075.886	2150.765	1.11	0.00119	0
P01031	0	1	968.2512	2902.739	5.91	0.00572	6.715281
P01031	0	0.9048	635.7316	1905.18	1.47	0.00093	62.14106
P01031	0	1	726.4379	2902.73	2.74	0.00198	4.52687
P01031	1	0.7059	445.2858	1333.843	1.07	0.00048	32.00188
P01031	0	1	635.7313	1905.179	0.89	0.00057	46.2723
P01031	0	1	635.7323	1905.182	2.53	0.00161	52.74075
P01031	0	1	765.6279	2294.869	1.72	0.00131	26.09533
P01031	0	0.541	599.8602	1198.713	4.72	0.00283	9.887559
P01031	0	1	800.3909	1599.775	0.93	0.00075	3.621832
P01031	0	1	533.9301	1599.776	1.57	0.00084	6.26926
P01031	0	0.6596	488.2899	1462.855	2.96	0.00144	25.10291
P01031	0	0.6429	731.9318	1462.856	3.74	0.00274	21.23481
P01031	0	0.9231	492.2625	1474.773	1.99	0.00098	14.74348
P01031	0	1	416.8824	1248.633	2.04	0.00085	21.4586
P01031	0	0.7037	570.8423	1140.677	7.35	0.00419	0
P01031	0	1	665.8359	1330.664	2.2	0.00146	0
P01031	0	1	624.8196	1248.632	1.47	0.00091	7.261602
P01031	0	0.8387	466.2945	1396.869	3.17	0.00148	3.811645
P01031	0	0.9032	456.5696	1367.694	1.43	0.00065	0
P01031	0	0.9825	579.346	1157.685	3.35	0.00194	0
P01031	0	0.8148	474.9348	1422.79	2.47	0.00117	49.14931
P01031	0	0.7708	620.8576	1240.708	4.21	0.00261	0
P01031	0	0.4444	449.514	1795.034	0.99	0.00044	7.724353
P01031	0	1	562.2821	1684.832	2.99	0.00168	23.86173
P01031	1	1	488.54	1951.138	2.21	0.00108	8.901708
P01031	0	0.7317	474.7598	948.5123	0.44	0.00021	0
P01031	0	1	416.882	1248.632	1.16	0.00048	41.0483
P01031	1	0.85	541.6608	1622.968	0.95	0.00052	30.37296
P01031	0	1	465.9232	1395.755	1.16	0.00054	0
P01031	0	1	608.6951	1824.071	-0.77	-0.00047	0
P01031	0	0.7297	503.6252	1508.861	1.17	0.00059	2.684187
P01031	0	0.9231	591.7969	1182.586	1.31	0.00077	0
P01031	0	0.96	394.8672	1182.587	1.68	0.00066	39.39681
P01031	0	0.4359	531.3447	1061.682	1.13	0.0006	26.3804
P01031	0	1	385.7194	1539.856	0.2	0.00008	12.05418
P01031	1	0.5185	493.6268	1478.866	1.1	0.00054	24.74531
P01031	1	0.5185	493.6268	1478.866	1.1	0.00054	24.74531

P01031	0	1	765.6266	2294.865	0.04	0.00003	0
P01031	0	0.7097	465.5895	1394.754	1.63	0.00076	57.86474
P01031	0	0.5455	514.3279	1540.969	1.82	0.00093	12.11005
P01031	0	0.48	459.2937	917.5802	1.52	0.0007	7.131306
P01031	0	0.6829	546.8121	1092.617	2.83	0.00155	10.14931
P01031	0	1	800.3924	1599.778	2.77	0.00221	4.323926
P01031	0	1	728.34	1455.673	1.12	0.00081	21.39882
P01031	0	1	754.9342	1508.861	1.2	0.0009	40.02469
P01031	1	1	452.5136	1807.033	0.56	0.00025	29.44942
P01031	0	0.7667	546.8112	1092.615	1.15	0.00063	63.91224
P01031	0	1	591.8906	1182.774	3.35	0.00198	27.00819
P01031	1	0.9143	603.0169	1807.036	2.53	0.00152	0
P01031	0	0.6585	599.8593	1198.711	3.29	0.00197	31.02072
P01031	0	1	570.8397	1140.672	2.75	0.00157	15.68983
P01031	0	0.8182	513.9562	1539.854	-0.9	-0.00046	0
P01031	0	1	533.9302	1599.776	1.8	0.00096	41.23232
P01031	0	0.5778	503.3321	1005.657	2.19	0.0011	10.69921
P01031	1	0.75	406.4982	1622.971	2.9	0.00118	5.135925
P01031	0	0.931	503.6252	1508.861	1.17	0.00059	33.49602
P01031	0	0.92	531.2669	1591.786	1.71	0.00091	21.93405
P01031	0	0.6154	459.2754	917.5436	1.26	0.00058	33.00624
P01031	0	0.875	549.9953	1647.971	2.52	0.00138	0
P01031	0	0.3469	503.3328	1005.658	3.59	0.0018	0
P01031	0	0.3333	531.3451	1061.683	1.93	0.00103	47.7251
P01031	0	0.8462	413.9964	1652.964	1.51	0.00062	8.021682
P01031	1	1	562.5736	2247.273	3.25	0.00182	32.73191
P01031	0	1	728.3401	1455.673	1.29	0.00094	13.44284
P01031	1	0.5	474.9325	1422.783	2.05	0.00097	50.36946
P01031	0	0.8485	397.8684	1191.591	1.9	0.00075	14.85106
P01031	0	0.7179	593.8321	1186.657	3.07	0.00182	16.18551
P01031	0	0.64	527.8072	1054.607	1.67	0.00088	30.81952
P01031	0	0.9444	579.3447	1157.682	1.14	0.00066	0
P01031	0	0.3636	503.3313	1005.655	0.67	0.00034	5.938673
P01031	0	0.3158	394.9286	1182.771	1.1	0.00043	8.298322
P01031	0	0.8966	551.6621	1652.972	6.17	0.0034	40.58726
P01031	0	0.4651	531.3281	1061.649	4.13	0.00219	37.31525
P01031	0	0.8966	624.8206	1248.634	3.03	0.00189	4.87297
P01031	0	0.75	576.8466	1152.686	-0.62	-0.00036	38.39925
P01031	0	1	728.3397	1455.672	0.7	0.00051	20.83271
P01031	0	0.4545	384.902	1152.691	4.16	0.0016	5.812415
P01031	0	0.5152	612.8785	1224.75	4.59	0.00281	10.54679
P01031	0	0.6	531.2652	1591.781	-1.39	-0.00074	52.74384
P01031	0	0.8947	503.6243	1508.858	-0.65	-0.00033	9.638051
P01031	0	0.6786	546.8121	1092.617	2.72	0.00148	28.75905
P01031	0	0.25	414.2397	1240.705	1.44	0.0006	0
P01031	0	1	800.3919	1599.777	2.15	0.00172	13.5879
P01031	0	0.8462	579.3474	1157.688	5.78	0.00335	26.57383
P01023	0	0.7436	600.6645	1799.979	2.65	0.00159	65.51884
P01023	0	0.9024	606.3267	1816.965	2.9	0.00176	4.060271
P01023	0	0.8356	745.3494	2234.034	2.88	0.00215	3.139711
P01023	0	0.7541	745.348	2234.03	1	0.00074	2.057614
P01023	0	1	740.017	2218.036	1.79	0.00133	3.463063

P01023	0	1	555.2631	2218.031	-0.77	-0.00043	3.859825
P01023	0	1	740.0166	2218.035	1.3	0.00096	2.106216
P01023	0	1	740.0162	2218.034	0.72	0.00053	30.03285
P01023	0	1	752.0698	2254.195	6.82	0.00512	46.39114
P01023	0	1	702.3416	1403.676	0.9	0.00063	15.84251
P01023	0	0.973	600.6645	1799.979	2.65	0.00159	25.26567
P01023	0	0.9429	600.6644	1799.979	2.45	0.00147	6.787901
P01023; P2	0	0.7586	624.3599	1247.712	3.91	0.00244	0
P01023	0	1	423.2319	1689.906	1.1	0.00047	4.112817
P01023	0	0.9107	600.665	1799.981	3.57	0.00214	9.141077
P01023	0	0.9091	600.6645	1799.979	2.65	0.00159	9.499352
P01023	0	0.9118	636.841	1272.675	1.45	0.00092	0
P01023	0	1	600.665	1799.981	3.57	0.00214	39.26236
P01023	0	1	946.1033	2836.295	1.79	0.00169	5.058046
P01023	0	0.7895	600.6642	1799.978	2.15	0.00129	0
P01023	0	0.9318	600.6635	1799.976	1.03	0.00062	44.61521
P01023	0	0.8816	600.6651	1799.981	3.67	0.0022	11.29211
P01023	0	0.8841	600.6655	1799.982	4.38	0.00263	5.541611
P01023	0	0.8971	600.6646	1799.979	2.86	0.00171	5.962119
P01023	0	0.8889	817.407	2450.206	1.39	0.00114	0
P01023	0	0.9787	946.1035	2836.296	1.98	0.00187	3.996225
P01023	0	1	946.1028	2836.294	1.27	0.0012	5.956341
P01023	0	1	946.1038	2836.297	2.31	0.00218	3.119897
P01023	0	1	946.103	2836.295	1.53	0.00145	3.524234
P01023	0	1	709.8291	2836.295	1.55	0.0011	17.73108
P01023	0	1	946.1043	2836.298	2.82	0.00267	2.3199
P01023	0	0.9574	752.0655	2254.182	1.05	0.00079	0
P01023	0	1	738.1119	2212.321	2.18	0.00161	11.28763
P01023	0	0.9756	553.8356	2212.321	1.93	0.00107	7.029124
P01023	0	0.9259	600.6647	1799.98	3.06	0.00184	16.56869
P01023	0	0.8472	708.8914	1416.775	0.35	0.00025	3.838468
P01023	0	0.8364	472.9308	1416.778	2.02	0.00095	7.412191
P01023	0	0.9825	600.665	1799.981	3.57	0.00214	8.8484
P01023	0	0.9104	600.6645	1799.979	2.65	0.00159	3.630693
P01023	0	0.9077	600.6647	1799.98	3.06	0.00184	0
P01023	0	0.8889	600.6647	1799.98	3.06	0.00184	9.541169
P01023	0	0.9231	600.6648	1799.98	3.16	0.0019	14.34167
P01023	0	0.9318	600.6646	1799.979	2.76	0.00165	18.00625
P01023	0	0.9365	600.6649	1799.98	3.37	0.00202	8.552613
P01023	0	1	458.2787	1372.822	1.77	0.00081	75.03383
P01023	0	0.9385	600.6644	1799.979	2.55	0.00153	8.847381
P01023	0	0.9178	600.6646	1799.979	2.76	0.00165	9.871824
P01023	0	0.7925	600.6643	1799.978	2.25	0.00135	0
P01023	0	0.8475	467.2539	1399.747	1.53	0.00071	5.215059
P01023	0	0.9636	700.3765	1399.746	0.54	0.00038	3.086525
P01023	0	1	467.253	1399.744	-0.43	-0.0002	12.04316
P01023	0	1	549.0451	2193.159	2.42	0.00133	6.682052
P01023; P2	0	1	622.3833	1865.135	3.18	0.00198	15.8727
P01023; P2	0	1	467.0389	1865.134	2.22	0.00104	53.79703
P01023	0	1	549.0453	2193.159	2.76	0.00151	13.8818
P01023	0	1	518.3207	1552.947	3.35	0.00174	17.83922
P01023	0	1	518.32	1552.945	1.94	0.001	12.895

P01023	0	1	730.0338	2188.087	0.88	0.00065	9.869285
P01023	0	0.9268	724.757	2172.256	2.07	0.0015	14.60529
P01023	0	0.988	724.7572	2172.257	2.41	0.00175	6.73528
P01023	0	0.8222	467.2544	1399.749	2.64	0.00123	29.99298
P01023	0	1	686.9134	1372.82	0.31	0.00021	29.38097
P01023	0	1	458.2786	1372.821	1.57	0.00072	35.90863
P01023	0	0.9189	600.6641	1799.978	1.94	0.00117	5.935919
P01023	0	0.98	600.6642	1799.978	2.15	0.00129	0
P01023	0	0.9516	600.6641	1799.978	1.94	0.00117	0
P01023	0	0.9762	542.9575	1626.858	1.72	0.00093	6.041781
P01023	0	0.9184	549.0449	2193.158	2.09	0.00115	7.488667
P01023	0	0.9697	615.0026	1842.993	3.24	0.00199	17.85872
P01023	0	0.9701	713.0339	2137.087	1.24	0.00088	5.758836
P01023	0	0.9	542.9574	1626.858	1.49	0.00081	7.878911
P01023	0	1	813.9324	1626.857	1.39	0.00113	7.205064
P01023; P2	0	0.6061	653.9038	1306.8	2.34	0.00153	5.954266
P01023	0	1	669.6061	2675.402	1.27	0.00085	2.266198
P01023	0	0.9773	667.9011	1334.795	2.08	0.00139	11.58764
P01023	0	0.9792	615.0024	1842.993	2.84	0.00175	20.28931
P01023	0	0.6863	615.0032	1842.995	4.14	0.00254	5.433955
P01023	0	0.5484	615.0025	1842.993	3.04	0.00187	8.990729
P01023	0	0.6923	615.0021	1842.992	2.45	0.0015	7.049337
P01023	0	0.6154	615.0021	1842.992	2.45	0.0015	9.626925
P01023	0	0.9074	615.0026	1842.993	3.14	0.00193	12.21332
P01023	1	1	621.8855	2484.52	2.7	0.00168	8.62326
P01023	0	1	868.9287	1736.85	1.38	0.0012	6.373858
P01023	0	0.9714	579.6221	1736.852	2.36	0.00137	6.800303
P01023	0	1	892.4728	2675.404	1.75	0.00156	3.653236
P01023	0	0.9802	669.6061	2675.402	1.27	0.00085	5.482967
P01023	1	1	497.7099	2484.52	2.73	0.00136	14.90488
P01023	0	1	467.2535	1399.746	0.61	0.00029	0
P01023	0	1	387.2061	1545.803	0.34	0.00013	5.037571
P01023	0	1	423.2344	1689.916	6.95	0.00294	31.79756
P01023	0	0.9545	468.9421	1404.812	2.04	0.00095	7.900205
P01023	0	0.8611	702.9091	1404.811	1.37	0.00096	6.099411
P01023	0	0.9744	636.0238	1906.057	2.34	0.00149	18.56458
P01023	0	0.9756	636.0237	1906.057	2.25	0.00143	11.01091
P01023	0	1	519.5781	1556.72	1.27	0.00066	26.70448
P01023	0	0.9592	778.8635	1556.72	1.16	0.00091	0
P01023	0	1	636.0238	1906.057	2.34	0.00149	53.67189
P01023	0	0.5	636.0239	1906.057	2.44	0.00155	29.43455
P01023	1	1	719.9817	3595.879	2.09	0.0015	16.71869
P01023	1	1	1199.301	3595.887	4.34	0.0052	0
P01023	0	0.9394	672.0964	2685.364	4.02	0.0027	13.98514
P01023	0	1	865.4412	2594.309	1.57	0.00136	3.13237
P01023	0	1	895.79	2685.355	0.84	0.00075	3.725401
P01023	0	1	895.7903	2685.356	1.25	0.00112	4.04686
P01023	0	1	895.7903	2685.356	1.18	0.00106	6.600752
P01023	0	0.9189	895.7907	2685.357	1.59	0.00142	5.499063
P01023	0	0.9231	672.0948	2685.357	1.56	0.00105	21.43341
P01023	0	1	654.36	1961.065	1.62	0.00106	4.346309
P01023	0	1	654.3604	1961.067	2.18	0.00143	6.267948

P01023	0	1	691.6132	2763.431	1.62	0.00112	13.97519
P01023	0	1	921.8156	2763.432	2	0.00185	10.62755
P01023	0	0.9677	662.688	1986.049	1.92	0.00127	10.30199
P01023	0	1	1117.284	3349.838	2.61	0.00291	12.13171
P01023	0	1	838.2141	3349.834	1.64	0.00137	0.16785
P01023	1	0.9118	603.6962	1809.074	1.82	0.0011	31.64408
P01023	0	0.9808	561.634	1682.887	2.04	0.00115	8.237028
P01023	0	0.74	631.6892	1893.053	2.98	0.00188	17.55057
P01023	0	1	677.8773	1354.747	1.88	0.00127	2.220234
P01023	0	1	783.7739	2349.307	4.02	0.00315	29.02278
P01023	0	1	677.8772	1354.747	1.79	0.00121	2.754095
P01023	1	1	667.3023	2666.187	1.92	0.00128	5.623496
P01023	0	1	677.8773	1354.747	1.97	0.00134	5.492491
P01023	1	1	667.3023	2666.187	1.83	0.00122	12.86255
P01023	0	0.8684	768.9411	1536.875	0.72	0.00055	8.808555
P01023	0	1	606.6401	1817.906	2.1	0.00127	13.22038
P01023	0	1	455.2313	1817.903	0.65	0.0003	8.040416
P01023	0	1	778.864	1556.721	1.79	0.00139	40.12609
P01023	0	1	778.4401	2333.306	1.27	0.00098	4.35623
P01023	0	1	519.5778	1556.719	0.57	0.00029	7.213981
P01023	0	0.9583	778.8636	1556.72	1.32	0.00103	1.97534
P01023	0	1	1167.156	2333.304	0.59	0.00069	0
P01023	0	1	514.0389	2053.134	1.65	0.00085	23.81048
P01023; P2	0	0.9899	743.0449	2227.12	2.48	0.00184	2.667964
P01023	0	1	521.5719	1562.701	1.25	0.00065	5.452801
P01023; P2	0	1	1114.065	2227.122	3.53	0.00393	4.164521
P01023	0	1	521.572	1562.702	1.48	0.00077	23.34895
P01023	0	1	423.232	1689.906	1.25	0.00053	5.262959
P01023	0	1	563.9747	1689.909	3.2	0.0018	22.18463
P01023	0	0.931	676.7228	2028.154	2.08	0.0014	50.31225
P01023	0	1	515.9396	1545.804	1.42	0.00073	31.09629
P01023	0	0.9831	685.3319	1369.657	1.98	0.00136	4.076197
P01023	0	1	505.2575	1513.758	1.25	0.00063	16.81107
P01023	0	1	677.8773	1354.747	1.97	0.00134	3.194145
P01023	0	1	677.8773	1354.747	1.88	0.00127	3.806889
P01023	0	0.9857	711.8984	1422.789	2.12	0.00151	6.787572
P01023	0	0.7778	589.3388	1177.67	3.12	0.00184	9.470379
P01023	0	0.8148	589.3383	1177.669	2.29	0.00135	6.338728
P01023	0	0.7843	589.3382	1177.669	2.08	0.00123	14.11425
P01023	0	0.7963	589.3383	1177.669	2.29	0.00135	6.168824
P01023; P2	0	1	1114.064	2227.12	2.54	0.00283	0
P01023; P2	0	1	617.0515	1849.14	2.86	0.00177	16.97725
P01023; P2	0	1	743.0453	2227.121	3.06	0.00227	3.090556
P01023; P2	0	0.8621	463.0398	1849.137	1.54	0.00071	11.29244
P01023	0	1	607.07	2425.258	-0.9	-0.00055	43.2245
P01023	0	1	1154.147	2307.288	2.06	0.00238	0
P01023	0	1	769.7673	2307.287	1.9	0.00146	14.17074
P01023	0	1	677.8777	1354.748	2.51	0.0017	2.123139
P01023	0	1	1039.937	5195.658	3.87	0.00402	0
P01023	0	1	677.8771	1354.747	1.7	0.00115	3.34785
P01023	0	1	677.8771	1354.747	1.61	0.00109	3.531385
P01023	0	1	677.8774	1354.748	2.15	0.00146	3.123936

P01023	0	1	677.8773	1354.747	1.97	0.00134	2.478673
P01023; P2	0	1	1095.084	2189.16	0.65	0.00071	0
P01023; P2	0	1	730.3897	2189.154	-1.9	-0.00138	15.28631
P01023	0	1	702.3421	1403.677	1.69	0.00118	4.167675
P01023	0	0.9868	752.0662	2254.184	2.03	0.00152	2.466944
P01023	0	0.9661	752.0663	2254.184	2.11	0.00158	3.240089
P01023	0	1	1033.556	3098.652	1.61	0.00166	0
P01023	0	0.9701	838.2253	3349.88	1.68	0.0014	1.438799
P01023	0	0.8889	752.0663	2254.184	2.11	0.00158	2.501437
P01023; P2	0	1	1114.064	2227.121	2.98	0.00332	4.288647
P01023	0	1	752.0661	2254.184	1.86	0.0014	1.864408
P01023	0	1	521.572	1562.702	1.48	0.00077	51.19549
P01023	0	1	606.6404	1817.907	2.5	0.00152	17.77935
P01023	0	1	455.2316	1817.904	1.32	0.0006	9.142676
P01023	0	1	518.319	1552.942	0.05	0.00003	28.66989
P01023	0	1	455.2317	1817.905	1.52	0.00069	12.64895
P01023	0	0.9925	1067.125	2133.242	0.39	0.00041	9.694853
P01023	0	1	798.6685	2393.991	2.33	0.00186	61.75587
P01023	0	1	798.6679	2393.989	1.64	0.00131	5.110663
P01023	0	1	798.6679	2393.989	1.64	0.00131	7.813326
P01023	0	1	711.7529	2133.244	1.47	0.00105	1.89923
P01023	0	1	775.4192	3098.655	2.41	0.00187	0
P01023	0	1	711.7528	2133.244	1.3	0.00092	22.4913
P01023	0	0.4688	615.0017	1842.991	1.75	0.00108	5.753488
P01023	0	1	677.8773	1354.747	1.88	0.00127	2.515475
P01023	0	0.8696	477.2692	1906.055	1.41	0.00067	12.17062
P01023	0	1	468.9413	1404.809	0.28	0.00013	43.92423
P01023	0	1	471.544	1412.617	1.07	0.0005	0
P01023	0	0.9474	468.941	1404.808	-0.38	-0.00018	37.72394
P01023	0	0.9118	700.3777	1399.748	2.19	0.00154	6.289577
P01023; P2	0	0.9655	464.609	1391.812	2.04	0.00094	50.13607
P01023	0	0.9524	474.9347	1422.79	2.22	0.00105	10.07941
P01023	0	0.75	802.1895	3205.736	2.86	0.00229	15.47247
P01023	0	0.9756	452.2532	1354.745	0.21	0.00009	3.873488
P01023	0	1	752.0646	2254.179	-0.09	-0.00006	0
P01023	0	0.8182	405.2556	1213.752	1.34	0.00054	4.553275
P01023	0	0.7872	486.7842	972.5611	1.74	0.00084	18.88784
P01023	0	0.8571	468.5639	1403.677	1.85	0.00087	58.46535
P01023	0	0.5	405.2554	1213.752	0.81	0.00033	39.57986
P01023	0	0.5806	405.255	1213.751	-0.09	-0.00004	49.36704
P01023	0	0.9429	600.6643	1799.978	2.35	0.00141	0
P01023	0	0.814	677.8767	1354.746	1.07	0.00073	21.85669
P01023	1	0.8	393.2269	1962.106	2.28	0.00089	23.59902
P01023	0	0.8286	615.0046	1842.999	6.52	0.00401	0
P01023	0	0.7586	405.2553	1213.751	0.51	0.00021	8.979755
P01023	0	1	547.7781	2188.09	2.61	0.00143	32.73696
P01023	1	0.4375	1151.263	3451.776	1.73	0.00199	5.981826
P01023	0	0.5625	423.2327	1689.909	2.83	0.0012	46.02856
P01023	0	0.7143	726.1441	3626.691	0.7	0.00051	2.533976
P01023	0	0.9143	713.0324	2137.082	-0.9	-0.00064	0
P01023	0	0.6667	393.2276	1177.668	1.34	0.00053	4.583789
P01023	0	1	519.5779	1556.719	0.92	0.00048	44.75653

P01023	0	1	389.9351	1556.719	0.44	0.00017	30.94657
P01023	0	0.9429	468.9408	1404.808	-0.77	-0.00036	18.63815
P01023	1	1	631.2771	2522.087	2.52	0.00159	11.15758
P01023	1	1	631.2771	2522.087	2.52	0.00159	11.15758
P01023; P2	0	0.7105	436.2716	1306.8	2.21	0.00096	9.604411
P01023	0	0.6444	393.2274	1177.668	0.8	0.00031	29.76438
P01023	0	0.2564	470.2865	1408.845	3.29	0.00155	52.8643
P01023	0	0.68	405.2556	1213.752	1.34	0.00054	75.17595
P01023	0	0.6552	672.0945	2685.356	1.11	0.00074	2.29723
P01023	0	1	521.5721	1562.702	1.6	0.00083	32.62658
P01023	0	1	798.6696	2393.994	3.78	0.00302	8.278071
P01023	0	0.5185	439.2957	1315.873	4.01	0.00176	14.33673
P01023	0	0.8571	752.0658	2254.183	1.46	0.00109	11.14205
P01023	0	0.975	452.2538	1354.747	1.56	0.0007	8.734811
P01023	0	1	600.6636	1799.976	1.23	0.00074	32.24606
P01023	0	0.9474	423.2329	1689.91	3.48	0.00147	8.568277
P01023	0	0.9811	677.8775	1354.748	2.24	0.00152	2.232975
P01023; P2	0	0.6552	464.6081	1391.81	0	0	36.77136
P01023	0	0.6316	439.2942	1315.868	0.6	0.00026	18.97398
P01023	0	0.9524	628.3242	1255.641	-1.53	-0.00096	11.70377
P01023	0	1	900.4932	1799.979	2.8	0.00252	5.304621
P01023	0	0.6222	527.3134	1053.62	1.23	0.00065	38.46835
P01023	0	1	439.2946	1315.869	1.43	0.00063	34.20888
P01023	0	1	798.6631	2393.975	-4.4	-0.00352	9.168782
P01023; P2	0	1	617.0518	1849.141	3.36	0.00207	42.06173
P01023	0	0.9211	745.3464	2234.025	-1.14	-0.00085	0
P01023	0	1	752.066	2254.183	1.7	0.00128	4.003798
P01023	0	0.9655	600.6644	1799.979	2.55	0.00153	2.951984
P01023; P2	0	0.8	464.6089	1391.812	1.77	0.00082	10.83583
P01023	0	0.7674	535.3288	1069.65	1.6	0.00086	8.342375
P01023	0	0.7714	393.2278	1177.669	1.73	0.00068	15.26814
P01023	0	0.6667	405.2558	1213.753	1.72	0.00069	48.55184
P01023	0	0.9615	542.9572	1626.857	1.16	0.00063	0
P01023	1	1	621.8861	2484.523	3.68	0.00229	7.659868
P01023	0	0.9744	677.8779	1354.748	2.78	0.00189	4.563282
P01023	0	1	518.3225	1552.953	6.77	0.00351	16.96815
P01023	0	0.6842	491.0215	1961.064	0.9	0.00044	4.814834
P01023	0	0.9048	600.6649	1799.98	3.26	0.00196	0
P01023	0	0.9714	452.2538	1354.747	1.56	0.0007	5.141404
P01023	0	1	662.6881	1986.05	2.02	0.00134	9.43444
P01023	0	0.4444	745.3485	2234.031	1.65	0.00123	4.574406
P01023; P2	0	0.5714	464.6095	1391.814	3.02	0.0014	67.77657
P01023	0	0.3158	600.6645	1799.979	2.65	0.00159	53.22431
P01023	0	1	946.1049	2836.3	3.47	0.00328	8.920265
P01023	0	0.9524	477.2667	1906.045	-3.91	-0.00186	67.13512
P01023	0	1	702.342	1403.677	1.51	0.00106	7.564545
P01023	0	0.5938	708.8919	1416.777	1.12	0.0008	0
P01023	0	0.8667	505.2576	1513.758	1.49	0.00075	25.45726
P01023; P2	0	0.75	436.2735	1306.806	6.63	0.00289	20.89936
P01023	0	0.825	452.2535	1354.746	1.02	0.00046	2.969825
P01023	0	0.7	631.6887	1893.052	2.31	0.00146	44.51569
P01023	0	0.9615	505.2578	1513.759	1.92	0.00097	13.41684

P01023	0	0.9474	752.0668	2254.186	2.84	0.00213	7.429555
P01023	0	1	439.295	1315.87	2.41	0.00106	54.85983
P01023	0	1	605.8259	1210.645	1.61	0.00098	0
P01023	0	0.9048	649.3336	2594.312	2.83	0.00184	5.233872
P01023	0	0.95	410.2448	1228.72	2.26	0.00092	4.187112
P01023	0	1	730.0341	2188.088	1.39	0.00101	0
P01023	0	0.92	474.9343	1422.788	1.32	0.00062	39.5909
P01023	0	0.8182	486.7839	972.5605	1.05	0.00051	14.46875
P01023	0	0.7895	636.0246	1906.059	3.59	0.00228	6.227667
P01023	0	1	606.6391	1817.903	0.39	0.00024	0
P01023	0	0.6429	752.0676	2254.188	3.81	0.00286	8.550724
P01023	0	0.8421	639.8481	1278.689	3.51	0.00225	44.87497
P01023	0	1	599.2527	2393.989	1.53	0.00092	18.53495
P01023; P2	0	0.7692	653.9042	1306.801	2.9	0.00189	44.47319
P01023	0	0.8113	517.2868	1033.566	1.67	0.00087	9.392795
P01023	0	0.7143	575.7914	1150.576	3.01	0.00173	3.320632
P01023	0	0.7391	615.0016	1842.99	1.55	0.00095	61.68029
P01023	0	1	600.6661	1799.984	5.4	0.00324	21.15507
P01023	0	1	599.2553	2393.999	5.81	0.00348	14.46319
P01023	0	1	419.206	1673.802	1.37	0.00057	27.06233
P01023	0	1	452.2541	1354.748	2.17	0.00098	2.590594
P01023	0	0.5	549.0444	2193.156	1.09	0.0006	24.89062
P01023	0	0.8333	654.3607	1961.067	2.65	0.00173	27.65699
P01023	0	0.6207	393.2267	1177.666	-0.91	-0.00036	25.02374
P01023	0	1	706.8131	1412.619	2.16	0.00152	49.13583
P01023	0	0.6667	636.0241	1906.058	2.83	0.00179	49.4406
P01023	0	1	388.9911	1552.942	0.08	0.00003	41.41932
P01023	0	1	669.6071	2675.407	2.82	0.00188	25.44687
P01023	0	0.8571	600.6645	1799.979	2.65	0.00159	4.70071
P01023	0	1	615.002	1842.991	2.15	0.00132	67.14676
P01023	0	1	547.7774	2188.088	1.38	0.00076	54.64318
P01023	0	1	605.8272	1210.647	3.63	0.0022	13.26174
P01023	0	0.9333	387.2065	1545.804	1.29	0.0005	5.772464
P01023	0	0.7273	738.1112	2212.319	1.19	0.00088	5.444876
P01023	0	0.4706	731.7236	2193.156	1.4	0.00102	71.64941
P01023	0	1	521.5728	1562.704	2.89	0.0015	19.31788
P01023	0	0.8	384.1964	1150.575	2.28	0.00088	7.292885
P01023	0	1	662.6895	1986.054	4.14	0.00274	91.56047
P01023	0	1	702.3418	1403.676	1.25	0.00088	0
P01023	0	1	474.9342	1422.788	1.06	0.0005	38.92342
P01023	0	0.7692	564.3023	2254.187	3.48	0.00196	13.82237
P01023	0	1	745.3481	2234.03	1.16	0.00086	16.43969
P01023	0	0.6957	841.9491	1682.891	4.15	0.0035	7.468262
P01024	0	1	593.9952	1779.971	2.49	0.00148	37.95137
P01024	0	0.8214	552.332	1103.657	2.34	0.00129	22.45925
P01024	0	0.9434	648.6998	1944.085	6.86	0.00445	69.50312
P01024	0	0.9298	648.6968	1944.076	2.34	0.00152	11.41728
P01024	0	0.6047	601.295	1801.87	2.47	0.00148	23.21041
P01024	0	0.9423	648.6976	1944.078	3.57	0.00231	16.26524
P01024	0	1	545.5767	2179.285	2.16	0.00118	3.876989
P01024	0	0.9143	486.7739	1944.074	1.14	0.00055	29.08276
P01024	0	1	601.2952	1801.871	2.88	0.00173	25.90656

P01024	0	0.7447	509.5507	2035.181	1.42	0.00072	6.660112
P01024	0	0.8966	679.065	2035.18	1.19	0.00081	4.139227
P01024	0	1	545.5767	2179.285	2.16	0.00118	0
P01024	0	0.9833	648.6969	1944.076	2.44	0.00158	10.29946
P01024	0	0.9483	821.392	1641.777	4.69	0.00385	0
P01024	0	1	781.4164	2342.235	1.12	0.00087	2.074238
P01024	0	0.9464	829.4485	2486.331	-1.26	-0.00105	75.19083
P01024	0	0.9531	821.3898	1641.772	2.01	0.00165	0
P01024	0	1	764.4038	2291.197	9.63	0.00736	2.676936
P01024	0	1	667.8662	1334.725	1.89	0.00126	4.218095
P01024	0	1	773.0673	2317.187	2	0.00155	11.85977
P01024	0	0.913	1159.097	2317.187	1.93	0.00224	0
P01024	0	1	773.067	2317.186	1.61	0.00124	2.690153
P01024	0	0.9524	545.3073	1633.907	2	0.00109	4.448252
P01024	0	0.7321	545.3068	1633.906	1.1	0.0006	8.478786
P01024	0	1	713.8809	2852.502	1.72	0.00122	19.79784
P01024	0	0.9655	643.3654	1928.082	2.7	0.00173	10.92219
P01024	0	0.9375	482.7758	1928.081	2.44	0.00118	30.63818
P01024	0	0.9667	964.5443	1928.081	2.52	0.00243	6.61709
P01024	0	0.9315	643.3641	1928.078	0.7	0.00045	8.456351
P01024	0	1	677.8557	2708.401	2.26	0.00153	10.94703
P01024	0	1	677.8557	2708.401	2.26	0.00153	10.94703
P01024	0	0.9655	903.4715	2708.4	1.95	0.00176	1.543729
P01024	0	0.9655	903.4715	2708.4	1.95	0.00176	1.543729
P01024	0	0.7692	643.3655	1928.082	2.89	0.00186	23.44032
P01024	0	1	951.5064	2852.504	2.73	0.00259	5.373587
P01024	0	1	713.881	2852.502	1.89	0.00135	3.566138
P01024	0	0.9756	677.855	2708.398	1.36	0.00092	4.459158
P01024	0	0.9756	677.855	2708.398	1.36	0.00092	4.459158
P01024	0	1	571.3068	2852.505	2.91	0.00166	18.25558
P01024	0	1	717.88	2868.498	2.29	0.00164	17.22749
P01024	0	0.9877	685.8701	1370.733	1.11	0.00076	0
P01024	0	1	685.871	1370.735	2.44	0.00167	1.085531
P01024	0	1	436.6629	2179.285	2.44	0.00106	5.054229
P01024	0	1	727.0991	2179.283	1.16	0.00084	5.245312
P01024	0	1	545.577	2179.286	2.84	0.00154	100
P01024	0	1	727.0994	2179.284	1.67	0.00121	3.415468
P01024	0	1	545.5765	2179.284	1.83	0.001	0.07832
P01024	0	0.74	404.5566	1211.655	1.69	0.00068	7.581698
P01024	1	1	559.3052	2234.199	0.05	0.00003	0
P01024	1	1	559.3069	2234.206	3.11	0.00174	5.20824
P01024	0	1	604.6198	1811.845	3.11	0.00188	26.74462
P01024	1	0.7812	546.6722	1638.002	3.06	0.00167	32.58884
P01024	0	1	906.4251	1811.843	2.14	0.00194	3.773589
P01024	1	1	745.4066	2234.205	2.85	0.00212	5.490417
P01024	0	1	545.5769	2179.286	2.61	0.00142	30.59536
P01024	0	0.9394	604.6201	1811.846	3.72	0.00225	44.81252
P01024	0	1	604.6201	1811.846	3.72	0.00225	25.64127
P01024	0	1	517.9135	1551.726	1.94	0.00101	3.162104
P01024	1	0.8056	382.488	1526.93	2.96	0.00113	34.47271
P01024	1	1	500.6245	1499.859	1.09	0.00054	12.7227
P01024	1	1	500.6247	1499.859	1.45	0.00073	9.206025

P01024	1	1	500.6242	1499.858	0.54	0.00027	9.808579
P01024	1	1	500.6242	1499.858	0.6	0.0003	0.457518
P01024	1	1	500.6241	1499.858	0.36	0.00018	7.639125
P01024	1	0.7736	559.9736	1677.906	3.2	0.00179	2.693383
P01024	0	0.6176	479.6219	1436.851	3.31	0.00158	17.53735
P01024	0	0.8269	717.3597	1433.712	1.47	0.00106	0
P01024	0	0.1714	500.9683	1500.89	1.78	0.00089	36.92563
P01024	0	0.6579	479.6218	1436.851	3.05	0.00146	17.65978
P01024	0	1	404.7172	1615.847	0.58	0.00023	0
P01024	0	0.6579	479.6212	1436.849	1.78	0.00085	4.07452
P01024	1	0.6379	509.6471	1526.927	0.69	0.00035	8.713358
P01024	1	1	462.6297	1385.875	2.01	0.00093	42.38081
P01024	0	1	449.7461	1795.963	0.6	0.00027	14.97705
P01024	0	0.8182	593.9951	1779.971	2.39	0.00142	12.24853
P01024	2	0.475	505.3278	1513.969	1.39	0.0007	17.03609
P01024	2	0.6038	505.3278	1513.969	1.33	0.00067	8.007991
P01024	1	1	421.229	1681.894	1.84	0.00077	30.20753
P01024	0	1	404.7175	1615.848	1.26	0.00051	5.012847
P01024	0	1	539.2875	1615.848	1.08	0.00058	6.800458
P01024	0	1	539.2874	1615.848	0.96	0.00052	40.68475
P01024	1	0.25	509.6474	1526.928	1.35	0.00069	23.21803
P01024	0	1	808.428	1615.849	1.58	0.00127	4.033058
P01024	0	1	404.7176	1615.849	1.48	0.0006	25.83107
P01024	0	1	539.2876	1615.848	1.3	0.0007	3.4591
P01024	0	1	539.2878	1615.849	1.76	0.00095	10.41414
P01024	0	0.9149	404.7178	1615.849	1.94	0.00078	6.508315
P01024	0	0.9608	539.2878	1615.849	1.76	0.00095	10.07658
P01024	0	1	708.5606	3538.774	3.46	0.00245	7.77293
P01024	0	1	1079.048	2157.09	1.87	0.00202	0
P01024	0	1	719.7012	2157.089	1.55	0.00112	0.297972
P01024	0	1	1079.048	2157.09	1.87	0.00202	0
P01024	0	1	719.7011	2157.089	1.38	0.001	0
P01024	0	0.971	818.7418	2454.211	1.71	0.0014	7.653842
P01024	0	0.987	818.7424	2454.213	2.38	0.00195	5.231061
P01024	0	1	436.6633	2179.288	3.42	0.00149	51.87839
P01024	0	1	719.7012	2157.089	1.55	0.00112	6.236691
P01024	0	0.9859	723.0652	2167.181	1.55	0.00112	5.433462
P01024	0	1	723.0645	2167.179	0.62	0.00045	6.48631
P01024	0	1	545.5765	2179.284	1.94	0.00106	100
P01024	0	1	708.5594	3538.768	1.82	0.00129	9.243796
P01024	0	0.9565	885.4479	3538.77	2.41	0.00214	0
P01024	0	1	885.4472	3538.767	1.59	0.0014	0.341497
P01024	0	1	708.5595	3538.768	1.91	0.00135	5.899827
P01024	0	1	885.4482	3538.771	2.76	0.00244	5.168289
P01024	0	1	708.5593	3538.767	1.65	0.00117	3.159132
P01024	0	1	708.5593	3538.767	1.65	0.00117	6.626089
P01024	0	1	885.4475	3538.768	1.93	0.00171	0
P01024	0	1	708.5598	3538.77	2.34	0.00166	3.931624
P01024	0	1	885.4473	3538.767	1.72	0.00153	24.99987
P01024	0	0.973	818.7416	2454.21	1.49	0.00121	5.830852
P01024	0	1	818.7421	2454.212	2.08	0.0017	4.343169
P01024	0	1	889.446	3554.762	1.64	0.00145	7.040766

P01024	0	0.9545	614.3094	2454.216	3.67	0.00225	8.254519
P01024	0	1	1227.609	2454.211	1.84	0.00226	0
P01024	0	1	818.741	2454.209	0.74	0.0006	100
P01024	0	1	818.7412	2454.209	0.96	0.00079	4.311306
P01024	0	0.9423	614.3088	2454.213	2.67	0.00164	5.6736
P01024	0	1	1227.61	2454.212	2.14	0.00262	0
P01024	0	1	818.742	2454.211	1.93	0.00158	0.810201
P01024	0	0.9811	818.7422	2454.212	2.16	0.00176	7.391229
P01024	0	0.9737	770.7068	2310.106	0.5	0.00038	11.21178
P01024	0	1	885.4475	3538.768	1.86	0.00165	0
P01024	0	1	708.5595	3538.768	2	0.00141	3.006406
P01024	0	1	885.4473	3538.767	1.72	0.00153	23.51777
P01024	0	0.9	533.3051	2130.199	1.72	0.00091	17.39225
P01024	0	0.963	885.448	3538.77	2.48	0.0022	0
P01024	0	1	708.5591	3538.767	1.48	0.00105	3.553201
P01024	0	1	885.4476	3538.769	2.07	0.00183	0.507234
P01024	0	1	708.5595	3538.768	2	0.00141	3.487272
P01024	0	1	885.4474	3538.768	1.79	0.00159	4.917524
P01024	0	1	885.4476	3538.769	2.07	0.00183	0
P01024	0	1	708.5591	3538.767	1.48	0.00105	5.691597
P01024	0	1	885.448	3538.77	2.48	0.0022	0
P01024	0	1	708.5595	3538.768	1.91	0.00135	3.317864
P01024	0	0.8478	538.9664	1614.885	1.75	0.00094	10.92861
P01024	0	0.8947	538.9663	1614.884	1.41	0.00076	5.872104
P01024	0	0.9574	807.945	1614.883	0.51	0.00041	3.413648
P01024	0	0.9667	648.6973	1944.077	3	0.00194	7.514337
P01024	0	0.9661	545.3075	1633.908	2.45	0.00133	49.86577
P01024	0	0.9348	586.3138	2342.233	0.56	0.00033	0
P01024	0	0.9494	781.4172	2342.237	2.21	0.00173	2.119686
P01024	0	0.9794	538.9661	1614.884	1.07	0.00058	3.013411
P01024	0	1	1243.672	2486.336	0.98	0.00122	4.303097
P01024	0	1	622.3396	2486.337	1.02	0.00063	4.441894
P01024	0	1	829.4499	2486.335	0.43	0.00036	4.112293
P01024	0	0.9821	622.3399	2486.338	1.51	0.00094	13.27589
P01024	0	1	829.4497	2486.334	0.14	0.00011	4.470733
P01024	0	1	764.404	2291.197	9.87	0.00754	13.31554
P01024	0	1	764.4009	2291.188	5.88	0.00449	3.171816
P01024	0	0.8721	817.4567	1633.906	1.37	0.00112	7.211819
P01024	0	0.9355	545.3075	1633.908	2.45	0.00133	8.79669
P01024	0	0.9877	817.457	1633.907	1.67	0.00137	100
P01024	0	0.8772	817.4562	1633.905	0.7	0.00057	27.82294
P01024	0	0.9298	892.4932	1783.979	2.66	0.00237	6.015945
P01024	0	0.9667	545.3077	1633.909	2.9	0.00158	9.269956
P01024	0	0.9524	817.4569	1633.907	1.6	0.0013	6.406536
P01024	0	0.9508	545.3076	1633.908	2.67	0.00145	10.02113
P01024	0	0.987	817.458	1633.909	2.94	0.0024	11.87791
P01024	0	0.963	545.3074	1633.908	2.34	0.00127	8.653893
P01024	0	0.9762	817.4572	1633.907	1.97	0.00161	19.651
P01024	0	0.95	545.3076	1633.908	2.56	0.00139	12.01842
P01024	0	0.9828	817.4578	1633.908	2.72	0.00222	36.32286
P01024	0	1	545.3071	1633.907	1.66	0.00091	38.78336
P01024	0	0.9561	817.457	1633.907	1.75	0.00143	12.78953

P01024	0	0.8113	627.8696	1254.732	2.56	0.00161	2.607334
P01024	0	0.8511	614.3352	1227.663	2.36	0.00145	20.10637
P01024	0	0.48	493.8229	986.6385	1.86	0.00092	9.663264
P01024	1	0.2903	439.2824	1315.832	1.91	0.00084	8.797668
P01024	0	1	452.5906	1355.757	1.6	0.00072	45.51552
P01024	1	0.3077	439.2826	1315.833	2.47	0.00108	27.98564
P01024	0	1	452.5906	1355.757	1.6	0.00072	43.96957
P01024	0	0.9762	505.6262	1514.864	2.52	0.00127	23.80364
P01024	0	0.9649	757.9359	1514.865	2.81	0.00213	6.392227
P01024	0	0.8833	599.3278	1795.969	4.14	0.00248	0
P01024	0	0.8222	379.4718	1514.865	3.36	0.00127	55.39912
P01024	0	1	505.626	1514.863	1.97	0.001	15.49325
P01024	0	0.8636	599.3279	1795.969	4.24	0.00254	18.59867
P01024	0	1	539.2883	1615.85	2.66	0.00143	25.12082
P01024	0	0.7759	614.3356	1227.664	3.06	0.00188	2.981653
P01024	0	0.4154	565.8745	1130.742	2.58	0.00146	7.371479
P01024	0	1	436.6631	2179.286	2.79	0.00121	2.473919
P01024	0	1	727.0996	2179.284	1.92	0.00139	7.673331
P01024	0	1	545.5765	2179.284	1.83	0.001	6.238082
P01024	0	0.7797	552.3321	1103.657	2.56	0.00141	3.287616
P01024	0	0.7818	552.3318	1103.656	2.01	0.00111	2.873648
P01024	0	0.7609	627.8701	1254.733	3.34	0.00209	3.136741
P01024	0	0.7843	552.332	1103.657	2.45	0.00135	11.2597
P01024	0	0.7966	552.3323	1103.657	3	0.00166	2.322246
P01024	0	0.9792	678.3821	1355.757	1.34	0.00091	3.472347
P01024	0	0.9153	452.5902	1355.756	0.65	0.00029	4.996224
P01024	1	0.9444	398.9934	1592.952	2.8	0.00111	19.53598
P01024	0	1	452.5904	1355.757	1.12	0.00051	9.758556
P01024	1	0.85	531.6552	1592.951	2.15	0.00114	8.318783
P01024	1	0.6667	531.6557	1592.953	3.19	0.00169	23.33697
P01024	1	0.9286	398.9931	1592.951	2.03	0.00081	15.11184
P01024	0	0.7805	717.3588	1433.71	0.19	0.00014	13.20949
P01024	1	0.9412	398.9931	1592.951	1.95	0.00078	10.61745
P01024	0	0.9091	452.5912	1355.759	2.81	0.00127	14.27592
P01024	0	1	452.5906	1355.757	1.39	0.00063	15.19212
P01024	0	0.9796	452.5908	1355.758	1.93	0.00087	14.00225
P01024	1	0.7234	445.9579	1335.859	1.46	0.00065	25.19195
P01024	0	0.7045	479.6211	1436.849	1.58	0.00076	8.234904
P01024	0	0.7273	479.6212	1436.849	1.78	0.00085	9.625485
P01024	1	0.7	559.9732	1677.905	2.44	0.00136	32.8424
P01024	0	0.7179	479.6214	1436.85	2.35	0.00113	11.22186
P01024	1	0.9167	398.9931	1592.95	1.88	0.00075	21.71409
P01024	0	0.6585	479.6215	1436.85	2.41	0.00116	12.99561
P01024	0	0.7619	479.6214	1436.85	2.35	0.00113	27.29515
P01024	0	0.6667	479.6218	1436.851	3.11	0.00149	14.04478
P01024	0	0.6905	479.6212	1436.849	1.9	0.00091	16.52369
P01024	0	0.7333	479.6214	1436.85	2.29	0.00109	11.54833
P01024	1	0.7111	445.9574	1335.858	0.37	0.00016	14.46041
P01024	1	0.8974	761.4191	2282.243	1.99	0.00151	6.6009
P01024	0	1	880.0888	2638.252	0.65	0.00057	3.87396
P01024	0	0.5581	648.0346	1942.089	0.25	0.00016	39.22734
P01024	1	0.9375	761.4193	2282.243	2.15	0.00164	3.963768

P01024	0	0.1864	565.8757	1130.744	4.74	0.00268	40.80844
P01024	1	0.7609	761.4207	2282.247	4	0.00304	5.130354
P01024	0	1	880.0868	2638.246	-1.64	-0.00144	2.652258
P01024	1	0.9615	444.2821	1330.832	1.66	0.00074	9.334938
P01024	1	1	761.4192	2282.243	2.07	0.00157	37.35952
P01024	1	0.8889	761.4199	2282.245	2.95	0.00225	11.34416
P01024	0	1	880.0892	2638.253	1.14	0.001	29.35386
P01024	0	0.8519	593.9957	1779.972	3.31	0.00197	8.375484
P01024	0	1	445.7482	1779.971	2.41	0.00107	39.3562
P01024	1	0.8043	761.4177	2282.238	0.06	0.00005	20.46719
P01024	0	0.9524	452.5903	1355.756	0.92	0.00042	13.98257
P01024	1	0.9216	761.4191	2282.243	1.91	0.00145	4.502548
P01024	0	1	539.2879	1615.849	1.87	0.00101	13.56889
P01024	1	0.8824	761.4188	2282.242	1.51	0.00115	2.344328
P01024	0	0.8868	452.5901	1355.756	0.38	0.00017	3.983469
P01024	0	1	539.2878	1615.849	1.64	0.00088	13.87055
P01024	1	0.9355	761.4181	2282.24	0.63	0.00048	7.413558
P01024	1	0.9138	761.421	2282.249	4.48	0.00341	3.387928
P01024	0	1	880.0869	2638.246	-1.57	-0.00138	2.419894
P01024	1	0.9815	761.4202	2282.246	3.35	0.00255	5.263799
P01024	1	0.8462	761.4199	2282.245	3.03	0.00231	3.173436
P01024	1	0.9	761.4195	2282.244	2.47	0.00188	16.02841
P01024	0	0.9324	670.6868	2010.046	2.23	0.00149	0
P01024	0	0.8	648.0354	1942.092	1.48	0.00096	30.38441
P01024	1	0.9672	761.4216	2282.25	5.28	0.00402	12.49604
P01024	0	0.875	670.6866	2010.045	1.96	0.00131	0
P01024	0	0.9833	880.0895	2638.254	1.48	0.0013	2.842493
P01024	0	0.6579	648.0355	1942.092	1.67	0.00108	44.11722
P01024	0	0.9231	670.6868	2010.046	2.32	0.00156	2.802096
P01024	0	0.8986	648.0354	1942.092	1.48	0.00096	16.97169
P01024	0	1	880.0879	2638.249	-0.39	-0.00034	4.352894
P01024	1	0.9	761.4209	2282.248	4.32	0.00328	2.982988
P01024	0	1	1084.054	2167.102	3.06	0.00331	8.356995
P01024	0	0.1806	1042.547	2084.086	1.85	0.00193	5.602253
P01024	0	0.3418	695.3663	2084.084	1	0.00069	4.510385
P01024	0	1	1012.003	2022.999	2.86	0.00289	3.764683
P01024	0	0.963	723.0391	2167.103	3.53	0.00255	8.778992
P01024	0	0.4545	1031.486	3092.444	2.74	0.00283	9.352146
P01024	0	0.9487	648.0374	1942.098	4.59	0.00297	0
P01024	0	1	880.0918	2638.261	4.05	0.00356	5.807326
P01024	0	1	1026.153	3076.445	1.54	0.00158	3.8727
P01024	0	1	1080.466	2159.924	2.26	0.00245	9.413045
P01024	0	1	768.6799	2304.025	1.61	0.00124	2.971451
P01024	0	1	1152.516	2304.026	1.8	0.00207	3.204306
P01024	0	0.6	970.4957	1939.984	2	0.00194	0
P01024	0		773.8648	3092.437	0.63	0.00049	0
P01024	0	0.0306	773.8648	3092.437	0.63	0.00049	0
P01024	0	0.4545	1031.486	3092.444	2.74	0.00283	9.352146
P01024	1	0.9344	761.4182	2282.24	0.71	0.00054	3.485296
P01024	0	0.9306	670.6862	2010.044	1.32	0.00088	0
P01024	1	0.9286	761.419	2282.242	1.75	0.00133	6.640753
P01024	0	0.9355	670.6861	2010.044	1.23	0.00082	10.24611

P01024	1	0.902	761.4188	2282.242	1.59	0.00121	2.050617
P01024	0	1	599.328	1795.969	4.45	0.00266	29.71358
P01024	0	1	539.2877	1615.848	1.42	0.00076	17.5169
P01024	0	1	539.2866	1615.845	-0.62	-0.00034	0
P01024	0	0.9467	670.6867	2010.046	2.14	0.00143	3.880956
P01024	0	1	539.2877	1615.848	1.42	0.00076	18.17202
P01024	1	0.8906	761.419	2282.242	1.83	0.00139	4.562738
P01024	0	0.9455	880.0884	2638.251	0.16	0.00015	0
P01024	1	0.9535	761.4212	2282.249	4.72	0.00359	12.9066
P01024	0	1	880.0883	2638.25	0.03	0.00002	0
P01024	0	1	539.2873	1615.847	0.74	0.0004	4.549528
P01024	1	0.8913	761.4191	2282.243	1.99	0.00151	4.720218
P01024	0	0.9344	670.6871	2010.047	2.69	0.0018	6.167407
P01024	0	1	880.085	2638.241	-3.65	-0.00321	30.59231
P01024	0	0.8696	404.7176	1615.849	1.48	0.0006	42.89047
P01024	0	1	539.2873	1615.847	0.74	0.0004	8.734681
P01024	1	1	446.2946	1336.869	1.16	0.00052	20.85666
P01024	0	1	539.2877	1615.849	1.53	0.00082	24.84494
P01024	1	1	438.9669	1314.886	2.07	0.00091	12.78033
P01024	1	1	761.4183	2282.24	0.87	0.00066	27.87403
P01024	1	1	500.6249	1499.86	2	0.001	25.45837
P01024	0	0.9677	670.687	2010.046	2.5	0.00168	4.018288
P01024	0	0.9831	880.0894	2638.254	1.28	0.00112	2.684968
P01024	0	0.925	404.7176	1615.849	1.56	0.00063	30.38656
P01024	0	1	808.4278	1615.848	1.35	0.00109	3.145079
P01024	0	1	808.4282	1615.849	1.88	0.00152	4.862577
P01024	0	1	404.7175	1615.848	1.33	0.00054	19.44222
P01024	0	1	539.2875	1615.848	1.19	0.00064	4.629321
P01024	0	1	808.428	1615.849	1.65	0.00133	0
P01024	0	0.9565	670.6865	2010.045	1.78	0.00119	3.172601
P01024	0	1	404.7178	1615.849	1.94	0.00078	5.598609
P01024	0	1	539.2875	1615.848	1.08	0.00058	4.269525
P01024	0	1	808.4285	1615.85	2.18	0.00176	2.470858
P01024	0	0.8852	404.7175	1615.848	1.33	0.00054	4.594302
P01024	0	1	539.2877	1615.849	1.53	0.00082	4.341512
P01024	0	1	808.4282	1615.849	1.8	0.00146	2.873106
P01024	0	1	404.7173	1615.847	0.73	0.00029	4.789752
P01024	1	0.9	761.4196	2282.244	2.63	0.002	0
P01024	0	1	539.2875	1615.848	1.08	0.00058	4.012166
P01024	1	0.8793	761.4183	2282.24	0.87	0.00066	32.48347
P01024	0	0.975	648.0365	1942.095	3.18	0.00206	61.31683
P01024	1	0.8824	571.3174	2282.248	4.12	0.00235	6.924332
P01024	0	1	880.0885	2638.251	0.3	0.00027	12.97281
P01024	0	0.9839	616.3031	1846.895	-0.03	-0.00002	4.132455
P01024	0	0.5763	690.9221	1380.837	2.01	0.00139	36.86774
P01024	0	1	923.9509	1846.894	-0.19	-0.00017	3.650021
P01024	1	0.9167	761.419	2282.242	1.75	0.00133	7.377662
P01024	0	0.8254	648.0364	1942.095	3.08	0.002	12.78503
P01024	1	0.9333	761.4191	2282.243	1.91	0.00145	6.414845
P01024	0	0.675	648.0352	1942.091	1.1	0.00071	26.09475
P01024	0	0.9429	648.036	1942.093	2.42	0.00157	30.82415
P01024	1	0.9048	571.3163	2282.243	2.2	0.00125	8.829834

P01024	0	1	923.9526	1846.898	1.73	0.0016	4.3608
P01024	1	0.8261	761.4199	2282.245	2.95	0.00225	18.41693
P01024	1	0.92	761.4189	2282.242	1.67	0.00127	18.11844
P01024	0	0.6757	616.3038	1846.897	1.16	0.00071	3.551825
P01024	0	1	880.0889	2638.252	0.79	0.00069	2.832625
P01024	0	1	880.0894	2638.254	1.28	0.00112	2.80819
P01024	0	0.987	660.3187	2638.253	1.02	0.00067	17.67423
P01024	0	1	880.0898	2638.255	1.83	0.00161	2.018876
P01024	0	0.4	648.0354	1942.092	1.48	0.00096	22.53575
P01024	1	0.9787	707.0366	2119.095	1.64	0.00116	7.482587
P01024	0	0.806	662.7039	1986.097	2.16	0.00143	4.808304
P01024	1	0.9111	530.5298	2119.097	2.59	0.00137	17.67368
P01024	1	0.9737	707.0364	2119.095	1.38	0.00098	7.659843
P01024	0	1	1319.631	2638.255	2	0.00264	9.373337
P01024	1	1	530.5297	2119.097	2.36	0.00125	43.08799
P01024	0	0.9908	660.319	2638.254	1.48	0.00098	3.707511
P01024	1	0.9815	707.037	2119.096	2.16	0.00153	6.283272
P01024	0	0.8108	923.9515	1846.896	0.54	0.0005	0
P01024	1	0.9412	761.4189	2282.242	1.67	0.00127	5.917617
P01024	0	0.8429	648.0363	1942.094	2.8	0.00181	22.36613
P01024	0	0.8036	648.0354	1942.092	1.48	0.00096	9.21777
P01024	1	1	761.419	2282.242	1.75	0.00133	9.783814
P01024	0	0.9231	648.0363	1942.094	2.89	0.00187	11.99699
P01024	1	1	761.419	2282.242	1.75	0.00133	19.75056
P01024	1	0.7941	571.3159	2282.242	1.56	0.00089	11.30107
P01024	0	0.875	648.0355	1942.092	1.67	0.00108	3.824559
P01024	1	0.9608	761.4165	2282.235	-1.46	-0.00111	9.068006
P01024	1	0.8611	571.3149	2282.238	-0.26	-0.00015	14.39237
P01024	0	0.9242	648.0361	1942.094	2.61	0.00169	7.364186
P01024	1	0.9683	761.419	2282.242	1.75	0.00133	8.865488
P01024	0	0.9211	648.0358	1942.093	2.05	0.00132	12.63833
P01024	1	0.9383	761.4192	2282.243	2.07	0.00157	7.281392
P01024	1	0.9375	761.4184	2282.241	1.03	0.00078	6.09685
P01024	1	0.9344	761.4194	2282.244	2.31	0.00176	2.576102
P01024	0	0.8909	648.0353	1942.091	1.39	0.0009	20.91998
P01024	1	0.918	761.4193	2282.243	2.23	0.0017	7.300451
P01024	0	0.8462	648.0355	1942.092	1.67	0.00108	2.028035
P01024	1	0.8056	571.3177	2282.249	4.66	0.00266	9.397661
P01024	1	0.8462	761.4197	2282.244	2.71	0.00206	10.88882
P01024	0	0.8983	648.0366	1942.095	3.27	0.00212	8.427519
P01024	0	0.7073	648.0366	1942.095	3.27	0.00212	14.12922
P01024	1	0.9595	761.419	2282.242	1.75	0.00133	3.373013
P01024	0	0.8382	648.036	1942.093	2.33	0.00151	10.09873
P01024	0	0.9333	648.0361	1942.094	2.61	0.00169	35.28796
P01024	1	0.9706	761.4194	2282.244	2.31	0.00176	8.841187
P01024	0	0.9412	648.036	1942.093	2.42	0.00157	45.84824
P01024	0	0.8043	648.0361	1942.094	2.61	0.00169	42.89344
P01024	0	0.9474	648.0373	1942.097	4.4	0.00285	9.958298
P01024	0	0.8816	648.0362	1942.094	2.71	0.00175	12.58057
P01024	1	0.9062	761.4197	2282.244	2.71	0.00206	37.12178
P01024	0	0.8769	648.0362	1942.094	2.71	0.00175	19.07158
P01024	0	0.9	692.7304	2076.177	1.68	0.00117	11.53498

P01024	1	1	761.4197	2282.244	2.71	0.00206	39.31753
P01024	1	1	761.4191	2282.243	1.99	0.00151	22.55211
P01024	1	0.75	571.3154	2282.24	0.59	0.00034	17.10268
P01024	0	0.8657	648.0366	1942.095	3.27	0.00212	15.68917
P01024	1	1	761.419	2282.242	1.83	0.00139	4.333237
P01024	1	0.9661	571.318	2282.25	5.19	0.00296	29.19534
P01024	0	0.8333	648.0356	1942.092	1.76	0.00114	5.695469
P01024	1	1	761.4186	2282.241	1.35	0.00103	11.09221
P01024	1	0.9074	571.317	2282.246	3.37	0.00193	3.669082
P01024	0	0.9306	648.0364	1942.095	3.08	0.002	7.510995
P01024	1	0.9677	761.4182	2282.24	0.79	0.0006	9.209319
P01024	0	0.9265	648.0361	1942.094	2.52	0.00163	19.23264
P01024	1	0.92	761.419	2282.242	1.83	0.00139	6.783161
P01024	1	0.8627	571.3165	2282.244	2.63	0.0015	64.50167
P01024	0	0.8551	648.0359	1942.093	2.23	0.00145	7.416413
P01024	1	0.9143	571.3154	2282.24	0.59	0.00034	28.90779
P01024	1	0.9074	761.4202	2282.246	3.43	0.00261	4.877081
P01024	1	0.9429	761.4189	2282.242	1.67	0.00127	0
P01024	0	0.8824	648.0367	1942.095	3.46	0.00224	39.88945
P01024	0	0.9853	880.0902	2638.256	2.18	0.00192	1.615159
P01024	0	0.8667	480.9416	1440.81	0.84	0.0004	62.54494
P01024	0	0.8902	720.9092	1440.811	1.51	0.00109	6.512867
P01024	0	0.7308	480.9422	1440.812	2.11	0.00101	8.009255
P01024	0	0.625	480.9419	1440.811	1.48	0.00071	12.71878
P01024	1	0.9688	761.4196	2282.244	2.55	0.00194	6.01217
P01024	1	0.6111	761.4182	2282.24	0.71	0.00054	4.595315
P01024	1	0.9149	761.4197	2282.245	2.79	0.00212	2.546978
P01024	0	0.7407	587.8704	1174.734	4.28	0.00252	100
P01024	0	0.6809	480.9418	1440.811	1.35	0.00065	10.30435
P01024	0	0.8868	714.3781	1427.749	2.74	0.00196	7.10457
P01024	0	0.9048	757.9217	1514.836	1.72	0.0013	3.729705
P01024	1	0.9355	761.4182	2282.24	0.79	0.0006	7.376333
P01024	0	0.8493	648.0371	1942.097	4.03	0.00261	42.1275
P01024	0	1	880.0893	2638.253	1.21	0.00106	2.005251
P01024	0	1	880.0894	2638.254	1.28	0.00112	2.189461
P01024	1	0.9333	761.4188	2282.242	1.59	0.00121	5.802393
P01024	0	1	880.0898	2638.255	1.83	0.00161	2.663171
P01024	1	0.8605	761.4194	2282.244	2.39	0.00182	3.411147
P01024	0	1	880.0891	2638.253	1	0.00088	19.3798
P01024	0	0.7879	504.9399	1512.805	2.67	0.00135	3.554398
P01024	0	1	880.089	2638.252	0.86	0.00076	2.795041
P01024	1	0.9388	761.4191	2282.243	1.99	0.00151	12.38545
P01024	0	1	676.8574	1352.707	2.49	0.00169	3.526554
P01024	0	1	880.0894	2638.254	1.34	0.00118	1.781836
P01024	1	0.8302	761.4189	2282.242	1.67	0.00127	9.75845
P01024	1	0.8636	761.4189	2282.242	1.67	0.00127	19.41426
P01024	0	0.9672	649.0171	1945.037	1.73	0.00112	2.948561
P01024	0	0.9906	880.0892	2638.253	1.07	0.00094	2.259118
P01024	1	0.8571	761.4183	2282.24	0.95	0.00072	3.701891
P01024	0	1	1273.161	2545.314	1.75	0.00223	0
P01024	0	0.8727	697.8679	1394.728	2.31	0.00161	2.820423
P01024	0	1	1026.154	3076.447	2.02	0.00207	0

P01024	0	1	854.4403	2561.306	0.65	0.00055	20.43663
P01024	0	1	1152.515	2304.023	0.85	0.00097	0
P01024	0	1	768.6802	2304.026	1.93	0.00148	20.91209
P01024	0	0.9481	849.1099	2545.315	2.06	0.00175	13.00192
P01024	0	0.8571	505.6162	1514.834	0.26	0.00013	4.45005
P01024	0	0.9836	757.9207	1514.834	0.35	0.00026	3.265729
P01024	0	0.8833	505.6161	1514.834	0.14	0.00007	34.00026
P01024	0	1	714.3777	1427.748	2.23	0.00159	5.633142
P01024	0	0.7037	697.8673	1394.727	1.52	0.00106	4.278603
P01024	0	0.8679	465.5812	1394.729	2.63	0.00122	2.14466
P01024	0	0.8889	465.5808	1394.728	1.84	0.00086	7.897058
P01024	0	0.6923	697.8668	1394.726	0.82	0.00057	3.40301
P01024	0	1	717.8798	2868.497	2.03	0.00146	21.74616
P01024	0	1	713.8815	2852.504	2.57	0.00184	7.306866
P01024	1	1	1132.541	3395.607	0.67	0.00076	2.328988
P01024	0	0.3544	695.3674	2084.088	2.58	0.00179	7.613627
P01024	0	1	571.3061	2852.501	1.63	0.00093	35.7038
P01024	0	1	951.5057	2852.502	2.02	0.00192	4.075742
P01024	0	1	713.8803	2852.499	0.95	0.00068	4.116827
P01024	0	0.6071	618.8715	1236.736	2.95	0.00182	2.426491
P01024	1	1	421.2294	1681.896	2.93	0.00123	9.3031
P01024	0	1	601.2946	1801.869	1.86	0.00112	29.28682
P01024	0	1	901.438	1801.869	1.53	0.00138	2.430489
P01024	2	1	477.5171	1907.047	1.59	0.00076	7.908171
P01024	2	1	477.5171	1907.047	1.59	0.00076	7.908171
P01024	0	0.9038	593.9949	1779.97	1.97	0.00117	13.00532
P01024	2	0.0385	636.3539	1907.047	1.82	0.00116	10.6707
P01024	2		636.3539	1907.047	1.82	0.00116	10.6707
P01024	0	0.8955	593.9943	1779.968	0.95	0.00056	7.69036
P01024	2	0.0556	477.5169	1907.046	1.08	0.00052	2.756554
P01024	0	1	445.748	1779.97	2.07	0.00092	39.82608
P01024	0	1	445.7482	1779.971	2.41	0.00107	28.93469
P01024	2	1	477.5171	1907.047	1.59	0.00076	7.908171
P01024	0	0.3953	438.2444	1312.719	0.91	0.0004	13.07735
P01024	2	0.1017	636.3543	1907.048	2.4	0.00153	7.948243
P01024	2	1	513.542	2051.146	0.22	0.00012	8.315079
P01024	0	0.8714	584.8135	1168.62	3.89	0.00227	26.30187
P01024	0	0.7826	614.3361	1227.665	3.85	0.00237	4.752878
P01024	0	0.7674	627.8696	1254.732	2.66	0.00167	6.644507
P01024	0	0.6731	418.9152	1254.731	1.97	0.00082	8.502866
P01024	0	0.7544	627.8685	1254.73	0.91	0.00057	3.284029
P01024	1	1	421.2282	1681.891	-0.05	-0.00002	5.48739
P01024	0	0.551	438.2443	1312.718	0.77	0.00034	0
P01024	0	1	645.3098	1289.612	3.35	0.00216	0
P01024	0	0.6901	656.8638	1312.72	2.34	0.00153	20.74958
P01024	0	0.2833	565.8744	1130.742	2.47	0.0014	8.799871
P01024	0	0.1967	565.8749	1130.743	3.44	0.00195	7.904404
P01024	0	0.1967	565.8751	1130.743	3.77	0.00213	7.201677
P01024	1	1	591.0024	1770.993	5.4	0.00319	35.79666
P01024	0	0.2456	565.8745	1130.742	2.69	0.00152	23.16597
P01024	0	0.3433	565.8745	1130.742	2.69	0.00152	33.31018
P01024	0	0.6912	656.8627	1312.718	0.66	0.00043	6.39124

P01024	0	0.3636	565.8747	1130.742	3.01	0.0017	19.92287
P01024	0	0.2222	565.8762	1130.745	5.6	0.00317	10.83633
P01024	0	0.5172	565.8757	1130.744	4.74	0.00268	31.21321
P01024	0	1	713.8805	2852.5	1.2	0.00086	4.37348
P01024	0	1	951.5114	2852.52	7.99	0.0076	8.863057
P01024	0	1	571.3069	2852.505	3.02	0.00172	6.307643
P01024	0	0.9688	880.0892	2638.253	1.07	0.00094	0
P01024	1	0.9524	761.4188	2282.242	1.59	0.00121	6.822721
P01024	1	0.92	571.3165	2282.244	2.63	0.0015	14.75939
P01024	1	0.9444	761.4199	2282.245	2.95	0.00225	6.834059
P01024	0	1	880.0909	2638.258	3.08	0.00271	1.977817
P01024	1	0.7273	571.3174	2282.248	4.12	0.00235	29.9663
P01024	0	0.6296	648.0359	1942.093	2.23	0.00145	2.570074
P01024	0	0.9524	700.6989	2100.082	2.25	0.00157	7.063157
P01024	1	1	786.103	2356.294	6.35	0.00498	25.93839
P01024	0	1	767.7357	2301.193	2.14	0.00164	0
P01024	0	1	700.6978	2100.079	0.68	0.00048	6.258474
P01024	1	0.9149	691.3665	2762.444	1.49	0.00103	9.406418
P01024	1	1	921.4932	2762.465	8.98	0.00827	53.4462
P01024	0	1	832.0497	2494.135	-5.38	-0.00447	37.25452
P01024	1	0.9556	691.3676	2762.448	2.99	0.00206	7.548436
P01024	0	1	880.0878	2638.249	-0.46	-0.0004	37.87069
P01024	1	0.7	571.3165	2282.244	2.52	0.00144	17.90386
P01024	1	1	761.4188	2282.242	1.59	0.00121	8.225058
P01024	0	1	1151.099	2301.19	1.01	0.00116	0
P01024	0	1	767.7338	2301.187	-0.33	-0.00025	2.216884
P01024	0	1	767.7341	2301.188	0.07	0.00006	0
P01024	1	0.7674	571.3159	2282.242	1.56	0.00089	43.54082
P01024	1	0.9733	761.4196	2282.244	2.55	0.00194	16.02232
P01024	0	0.9667	670.6864	2010.045	1.68	0.00113	0
P01024	0	0.9362	723.0656	2167.182	2.14	0.00155	30.98116
P01024	0	0.9296	648.0353	1942.091	1.29	0.00084	0
P01024	0	1	767.7347	2301.189	0.79	0.00061	2.489722
P01024	0	1	1151.099	2301.192	1.75	0.00201	2.411083
P01024	0	1	767.7343	2301.188	0.31	0.00024	2.613189
P01024	0	1	773.0669	2317.186	1.53	0.00118	3.321414
P01024	0	1	773.0666	2317.185	1.13	0.00088	7.997076
P01024	0	0.7167	690.9208	1380.834	0.24	0.00017	16.69075
P01024	0	1	713.8829	2852.51	4.54	0.00324	32.98532
P01024	0	1	951.5057	2852.503	2.09	0.00198	5.438453
P01024	0	1	713.8809	2852.502	1.8	0.00129	6.980599
P01024	0	0.6774	690.9211	1380.835	0.6	0.00041	0.43442
P01024	0	0.4222	690.9227	1380.838	2.98	0.00206	19.08362
P01024	0	0.4375	460.95	1380.835	1.03	0.00047	10.35044
P01024	0	0.9388	880.0903	2638.256	2.32	0.00204	1.309608
P01024	0	1	667.8666	1334.726	2.62	0.00175	23.94862
P01024	0	1	595.9641	1785.878	3.78	0.00225	15.42492
P01024	0	1	851.9009	1702.795	1.09	0.00093	0
P01024	0	0.9559	880.0897	2638.254	1.62	0.00143	5.393572
P01024	1	0.963	761.4205	2282.247	3.83	0.00292	22.39546
P01024	0	1	893.4394	1785.872	0.27	0.00024	3.290036
P01024	0	1	595.9619	1785.871	0.09	0.00005	4.768652

P01024	1	0.9583	761.4193	2282.243	2.15	0.00164	7.155581
P01024	1	0.9545	559.9734	1677.906	2.77	0.00155	13.46286
P01024	0	1	803.0867	2407.246	2.02	0.00162	35.29527
P01024	1	1	559.9726	1677.903	1.35	0.00075	2.592273
P01024	0	0.9286	672.719	2016.142	3.27	0.0022	14.07725
P01024	1	1	1015.173	3043.504	3.16	0.0032	3.560711
P01024	1	0.8167	559.9728	1677.904	1.78	0.001	8.383908
P01024	0	0.9759	812.4726	1623.938	1.89	0.00154	8.380633
P01024	1	1	761.6304	3043.5	1.67	0.00127	6.169547
P01024	1	1	1015.172	3043.502	2.31	0.00235	3.614882
P01024	1	1	761.6302	3043.499	1.43	0.00109	5.421591
P01024	1	1	1015.172	3043.501	2.19	0.00223	3.446305
P01024	1	0.9315	839.4554	1677.903	1.5	0.00126	6.330214
P01024	0	1	552.6459	1655.923	-0.28	-0.00016	4.693666
P01024	0	1	828.4653	1655.923	-0.2	-0.00016	2.995376
P01024	0	0.942	552.647	1655.927	1.71	0.00094	6.001133
P01024	0	1	964.4166	2891.235	1.63	0.00157	3.977208
P01024	1	1	761.6304	3043.5	1.67	0.00127	3.098587
P01024	0	0.9286	828.4669	1655.926	1.64	0.00136	1.62189
P01024	0	1	723.5653	2891.239	3.01	0.00218	1.558357
P01024	0	1	964.4141	2891.228	-0.91	-0.00087	5.327418
P01024	0	0.7843	541.9837	1623.937	1.06	0.00057	8.524255
P01024	1	1	761.6303	3043.499	1.59	0.00121	5.496209
P01024	0	1	760.1396	3037.537	5.63	0.00428	18.62157
P01024	0	0.9091	828.4673	1655.927	2.16	0.00179	0
P01024	1	1	1015.172	3043.502	2.43	0.00247	2.975655
P01024	1	0.9167	839.4554	1677.903	1.5	0.00126	6.012403
P01024	1	0.8636	559.9721	1677.902	0.47	0.00026	9.145682
P01024	1	1	761.6303	3043.499	1.59	0.00121	4.715016
P01024	1	1	839.4559	1677.904	2.08	0.00175	6.738241
P01024	1	1	1015.172	3043.501	2.01	0.00204	2.69429
P01024	1	0.8649	559.9725	1677.903	1.13	0.00063	8.912168
P01024	1	0.8611	559.9727	1677.903	1.46	0.00081	10.37407
P01024	1	1	761.63	3043.498	1.19	0.0009	3.646046
P01024	0	1	735.8944	1470.782	1.15	0.00085	3.146883
P01024	1	0.878	559.972	1677.901	0.26	0.00014	8.496011
P01024	1	1	1015.171	3043.5	1.71	0.00174	3.635164
P01024	1	1	761.6304	3043.5	1.67	0.00127	3.745147
P01024	1	1	1015.172	3043.501	2.19	0.00223	2.810037
P01024	1	1	761.6304	3043.5	1.67	0.00127	3.033229
P01024	1	1	559.9721	1677.902	0.47	0.00026	0.523854
P01024	1	0.8594	559.9721	1677.902	0.47	0.00026	8.91062
P01024	1	1	1015.172	3043.501	1.95	0.00198	3.862218
P01024	1	0.8676	559.9721	1677.902	0.47	0.00026	10.73318
P01024	1	1	839.4556	1677.904	1.72	0.00144	6.822971
P01024	1	0.9762	681.169	3401.816	2.32	0.00158	6.227491
P01024	0	1	803.0862	2407.244	1.34	0.00107	11.63925
P01024	0	0.9605	602.5671	2407.246	2.37	0.00143	1.873599
P01024	1	0.9762	681.169	3401.816	2.32	0.00158	6.227491
P01024	0	0.9815	602.567	2407.246	2.27	0.00137	1.536593
P01024	0	0.7647	530.2702	1588.796	2.59	0.00137	9.449334
P01024	0	1	794.9012	1588.795	1.98	0.00158	6.675832

P01024	0	0.8043	530.2701	1588.796	2.47	0.00131	8.618893
P01024	0	1	794.8999	1588.793	0.37	0.00029	6.300881
P01024	0	1	803.0841	2407.238	-1.25	-0.001	51.87331
P01024	0	0.9231	648.0375	1942.098	4.69	0.00303	22.20974
P01024	0	1	803.0864	2407.245	1.64	0.00132	14.36591
P01024	0	0.8519	672.7173	2016.137	0.73	0.00049	36.51883
P01024	0	0.98	602.5666	2407.245	1.56	0.00094	32.23099
P01024	1	0.9722	709.9887	3545.914	1.14	0.00081	24.29012
P01024	1	0.8936	761.4219	2282.251	5.68	0.00432	24.39528
P01024	0	0.9104	648.0366	1942.095	3.27	0.00212	26.27517
P01024	0	1	681.3621	2722.427	6.01	0.00409	2.187102
P01024	0	1	803.0864	2407.245	1.57	0.00126	2.662729
P01024	0	1	908.1432	2722.415	1.73	0.00157	1.670474
P01024	0	1	803.0863	2407.244	1.49	0.0012	11.99712
P01024	0	1	672.7195	2016.144	4.09	0.00275	7.898105
P01024	1	0.9362	761.42	2282.245	3.11	0.00237	13.20814
P01024	1	0.8605	503.9526	1509.843	0.9	0.00045	21.90457
P01024	0	0.9091	717.36	1433.713	1.81	0.0013	0
P01024	0	0.9688	648.0371	1942.097	4.03	0.00261	12.40545
P01024	0	1	672.718	2016.139	1.73	0.00116	7.794504
P01024	1	1	398.9937	1592.953	3.56	0.00142	32.30318
P01024	1	0.6552	531.6546	1592.949	1.12	0.00059	8.137566
P01024	0	1	624.6841	1872.038	2.09	0.00131	43.13214
P01024	0	0.9783	595.9641	1785.878	3.78	0.00225	3.09141
P01024	1	1	398.9934	1592.952	2.57	0.00102	22.51931
P01024	0	0.8226	648.0361	1942.094	2.52	0.00163	29.06031
P01024	0	0.8889	479.6198	1436.845	-0.96	-0.00046	21.67955
P01024	0	1	733.4278	2198.269	0.62	0.00046	6.076848
P01024	1	0.8421	761.4195	2282.244	2.47	0.00188	4.966627
P01024	0	0.5417	479.6213	1436.849	2.16	0.00103	0
P01024	0	0.7	479.621	1436.848	1.46	0.0007	9.365727
P01024	0	1	1099.639	2198.271	1.74	0.00191	19.44694
P01024	1	0.9259	398.9937	1592.953	3.49	0.00139	8.838181
P01024	1	0.8462	531.6558	1592.953	3.42	0.00181	8.318171
P01024	1	0.6333	531.6553	1592.951	2.5	0.00133	20.203
P01024	0	0.9787	645.3292	1933.973	3.73	0.00241	17.64808
P01024	0	1	786.7088	2358.112	2.27	0.00178	4.452007
P01024	0	0.96	786.7091	2358.113	2.66	0.00209	1.724487
P01024	0	1	908.4822	1815.957	1.49	0.00136	20.59525
P01024	0	1	605.9898	1815.955	0.28	0.00017	8.500375
P01024	0	1	672.7178	2016.139	1.45	0.00098	27.34234
P01024	0	0.4259	819.1858	3273.721	2.52	0.00207	14.39419
P01024	0	0.6667	504.79	2016.138	1.05	0.00053	0
P01024	0	1	624.6846	1872.039	2.88	0.0018	5.494987
P01024	0	0.9344	603.8002	1206.593	2.13	0.00128	5.173339
P01024	0	1	672.7168	2016.136	0	0	0
P01024	0	1	672.7169	2016.136	0.18	0.00012	2.600743
P01024	0	0.9315	624.6843	1872.038	2.39	0.00149	0
P01024	1	1	511.9381	1533.8	0.45	0.00023	52.28294
P01024	1	1	511.9381	1533.8	0.45	0.00023	52.28294
P01024	0	0.3651	1018.51	3053.515	0.07	0.00008	64.10399
P01024	0	0.4359	1091.911	3273.717	1.2	0.00131	0

P01024	0	1	672.718	2016.14	1.82	0.00122	2.394669
P01024	0	0.5429	648.0356	1942.092	1.76	0.00114	0
P01024	1	0.9474	761.4205	2282.247	3.75	0.00286	20.58779
P01024	0	1	981.0034	1960.999	2.58	0.00253	4.209519
P01024	0	0.9789	654.3379	1960.999	2.42	0.00158	4.123317
P01024	0	1	908.952	1816.897	2.39	0.00217	5.564895
P01024	0	0.9419	606.3043	1816.898	3.36	0.00203	0
P01024	0	0.9902	786.7083	2358.11	1.57	0.00123	12.13895
P01024	0	0.9875	786.7087	2358.111	2.11	0.00166	4.081446
P01024	0	0.9342	786.7084	2358.111	1.72	0.00135	10.20641
P01024	0	1	786.7087	2358.112	2.19	0.00172	13.58159
P01024	2	0.8333	513.5431	2051.151	2.37	0.00121	8.848928
P01024	0	0.9881	786.7081	2358.11	1.41	0.00111	2.713516
P01024	0	0.8958	564.0178	1690.039	-0.23	-0.00013	7.73945
P01024	0	1	786.708	2358.109	1.26	0.00099	2.540077
P01024	0	1	786.7079	2358.109	1.1	0.00087	11.85513
P01024	0	1	803.088	2407.249	3.54	0.00284	22.6765
P01024	0	1	1179.559	2358.111	2.07	0.00244	0
P01024	0	1	786.7072	2358.107	0.25	0.0002	2.276945
P01024	0	1	738.6737	2214.006	0.88	0.00065	15.34129
P01024	0	1	786.7071	2358.107	0.09	0.00007	0
P01024	0	1	916.3831	2747.135	2.27	0.00208	0
P01024	0	1	786.7083	2358.11	1.57	0.00123	2.2667
P01024	0	1	723.5643	2891.235	1.66	0.0012	3.960311
P01024	0	1	964.4171	2891.237	2.2	0.00212	3.911639
P01024	0	1	786.7084	2358.111	1.8	0.00142	2.630527
P01024	0	1	916.3826	2747.133	1.74	0.00159	1.890722
P01024	0	1	723.5649	2891.238	2.51	0.00181	4.850716
P01024	0	0.9868	1179.559	2358.11	1.65	0.00195	0
P01024	0	1	964.4173	2891.237	2.39	0.0023	4.045831
P01024	0	1	1179.559	2358.11	1.55	0.00183	0.632811
P01024	0	0.9881	786.7078	2358.109	0.95	0.00074	2.167222
P01024	0	1	738.6737	2214.006	0.88	0.00065	2.17446
P01024	0	1	1179.559	2358.11	1.65	0.00195	0
P01024	0	1	786.7075	2358.108	0.64	0.0005	2.748203
P01024	0	1	1179.559	2358.111	1.96	0.00231	0
P01024	0	1	776.3668	1551.726	2.19	0.0017	3.667263
P01024	0	1	1179.559	2358.11	1.55	0.00183	0
P01024	0	1	738.6736	2214.006	0.79	0.00059	0
P01024	0	1	786.7073	2358.107	0.4	0.00032	0
P01024	0	1	786.7073	2358.107	0.4	0.00032	10.53674
P01024	0	1	738.6737	2214.006	0.88	0.00065	0
P01024	0	1	1179.558	2358.11	1.34	0.00158	0.44973
P01024	0	1	1107.506	2214.006	0.55	0.00061	0
P01024	0	1	786.7072	2358.107	0.25	0.0002	2.515662
P01024	0	1	517.9131	1551.725	1.12	0.00058	17.80295
P01024	0	1	738.6741	2214.008	1.46	0.00107	0.813458
P01024	0	1	1179.559	2358.111	1.76	0.00207	0
P01024	0	1	1107.508	2214.008	1.76	0.00195	0
P01024	0	1	786.7075	2358.108	0.56	0.00044	2.434725
P01024	0	1	964.4158	2891.233	0.87	0.00083	0
P01024	0	1	517.9133	1551.725	1.59	0.00082	5.601008

P01024	0	0.0556	500.9685	1500.891	2.33	0.00117	17.86632
P01024	0	0.8451	675.8524	1350.697	3.61	0.00244	5.715099
P01024	1	1	561.3035	1681.896	2.9	0.00163	11.41621
P01024	0	0.9535	450.9035	1350.696	2.51	0.00113	9.107911
P01024	0	0.9467	605.9912	1815.959	2.5	0.00151	4.37279
P01024	1	1	421.2293	1681.896	2.71	0.00114	46.18562
P01024	0	1	803.083	2407.234	-2.69	-0.00216	5.200674
P01024	0	0.9762	908.4826	1815.958	1.96	0.00178	1.333601
P01024	1	0.9677	561.304	1681.898	3.88	0.00218	19.84244
P01024	0	1	450.9023	1350.692	-0.06	-0.00003	8.526272
P01024	0	0.8571	675.8502	1350.693	0.36	0.00024	5.892132
P01024	0	1	845.5238	1690.04	0.61	0.00051	5.861838
P01024	1	1	503.9525	1509.843	0.66	0.00033	9.706235
P01024	0	0.8727	692.8614	1384.716	2.28	0.00158	3.602351
P01024	0	1	462.2421	1384.712	-0.44	-0.0002	25.17028
P01024	0	0.9878	605.9916	1815.96	3.3	0.002	14.41915
P01024	0	0.9718	605.9915	1815.96	3	0.00182	3.277436
P01024	0	0.956	605.9907	1815.957	1.69	0.00102	5.034089
P01024	0	0.8	564.0195	1690.044	2.69	0.00152	13.88159
P01024	0	0.0541	500.9681	1500.89	1.48	0.00074	31.71945
P01024	0	1	916.3817	2747.13	0.74	0.00068	0
P01024	0	0.8889	523.9392	1569.803	1.93	0.00101	11.74787
P01024	0	0.8873	785.4058	1569.804	2.79	0.00219	6.450924
P01024	0	0.9423	523.9397	1569.805	2.98	0.00156	10.41501
P01024	0	0.9054	793.8826	1586.758	2.19	0.00174	7.39928
P01024	0	1	529.5907	1586.758	1.91	0.00101	7.958789
P01024	0	0.908	785.4047	1569.802	1.47	0.00115	5.638725
P01024	0	0.9683	523.939	1569.802	1.58	0.00083	9.620114
P01024	0	0.9302	803.0861	2407.244	1.19	0.00095	29.9456
P01024	0	0.8941	785.4053	1569.803	2.25	0.00176	6.207455
P01024	0	0.9831	785.4056	1569.804	2.56	0.00201	8.044439
P01024	0	0.7917	430.922	1290.751	0.48	0.00021	9.421391
P01024	0	0.85	645.8794	1290.752	0.61	0.00039	6.023853
P01024	0	0.9545	565.947	1695.826	0.76	0.00043	14.45208
P01024	0	0.975	565.949	1695.833	4.44	0.00251	6.368908
P01024	0	1	565.9471	1695.827	1.09	0.00062	13.99445
P01024	0	1	565.9454	1695.822	-1.93	-0.00109	37.97032
P01024	0	0.8036	523.9392	1569.803	2.05	0.00107	12.92473
P01024	0	0.7442	523.9388	1569.802	1.35	0.0007	9.152535
P01024	0	1	529.5893	1586.753	-0.74	-0.00039	9.545547
P01024	0	0.85	645.8804	1290.753	2.12	0.00137	13.54526
P01024	0	0.8	645.8803	1290.753	2.03	0.00131	23.30298
P01024	0	1	880.0892	2638.253	1.14	0.001	3.777273
P01024	0	1	803.0862	2407.244	1.41	0.00113	18.91265
P01024	1	1	763.3936	2288.166	1.89	0.00144	9.494504
P01024	1	1	572.7972	2288.167	2.23	0.00128	5.214916
P01024	0	1	803.0863	2407.244	1.49	0.0012	17.26485
P01024	0	1	713.3535	1425.7	1.37	0.00098	0
P01024	0	1	523.9382	1569.8	0.06	0.00003	16.93076
P01024	0	0.7568	523.9393	1569.803	2.16	0.00113	12.72792
P01024	0	0.6486	648.0358	1942.093	2.14	0.00139	61.4591
P01024	0	1	565.9466	1695.825	0.12	0.00007	18.00137

P01024	0	1	723.5641	2891.235	1.41	0.00102	3.882621
P01024	0	1	964.4165	2891.235	1.56	0.00151	4.002057
P01024	0	1	723.5643	2891.235	1.66	0.0012	4.522698
P01024	0	1	964.4165	2891.235	1.56	0.00151	4.111323
P01024	0	0.9545	786.7083	2358.11	1.65	0.00129	1.802467
P01024	0	1	964.4174	2891.238	2.51	0.00242	3.996088
P01024	0	0.9756	786.7078	2358.109	0.95	0.00074	9.148158
P01024	0	0.9667	786.7075	2358.108	0.56	0.00044	12.89709
P01024	0	1	565.9479	1695.829	2.38	0.00135	7.956236
P01024	0	0.6111	722.8492	1444.691	0.84	0.00061	0
P01024	0	0.7746	722.8498	1444.692	1.68	0.00122	4.950117
P01024	0	1	565.9478	1695.829	2.28	0.00129	8.069776
P01024	0	0.973	424.7125	1695.828	1.91	0.00081	10.01504
P01024	0	1	529.5915	1586.76	3.41	0.0018	59.37828
P01024	0	1	848.4178	1695.828	1.92	0.00163	6.612415
P01024	0	1	565.9495	1695.834	5.19	0.00293	7.797422
P01024	0	1	803.0863	2407.244	1.49	0.0012	5.790777
P01024	1	1	493.9352	1479.791	1.27	0.00062	9.327494
P01024	0	0.8182	662.7036	1986.096	1.7	0.00113	8.396692
P01024	0	1	523.0395	2089.136	0.35	0.00018	33.54731
P01024	1	1	903.5106	3611.021	2.2	0.00199	7.571024
P01024	1	1	493.9352	1479.791	1.2	0.00059	8.739074
P01024	0	0.6	497.2794	1986.096	1.36	0.00067	0
P01024	1	0.9	740.3995	1479.792	1.69	0.00125	5.822781
P01024	1	0.6957	493.9352	1479.791	1.08	0.00053	7.895748
P01024	1	0.7143	493.935	1479.79	0.77	0.00038	8.352705
P01024	0	1	697.0501	2089.136	0.18	0.00012	4.121869
P01024	0	0.9375	451.2563	1351.754	3.67	0.00165	12.64542
P01024	0	0.88	676.381	1351.755	3.95	0.00267	5.984123
P01024	1	1	511.9386	1533.801	1.58	0.00081	10.0011
P01024	1	1	511.9386	1533.801	1.58	0.00081	10.0011
P01024	1	0.0192	767.4037	1533.8	0.77	0.00059	0
P01024	1		767.4037	1533.8	0.77	0.00059	0
P01024	1	0.9775	511.9387	1533.802	1.7	0.00087	10.11606
P01024	0	0.8182	552.332	1103.657	2.34	0.00129	3.80226
P01024	0	0.8	662.7039	1986.097	2.07	0.00137	17.05764
P01024	0	0.5909	497.2801	1986.098	2.77	0.00138	26.10733
P01024	1	0.9775	511.9387	1533.802	1.7	0.00087	10.11606
P01024	1	0.0508	767.4042	1533.801	1.49	0.00114	2.393142
P01024	1	1	511.9383	1533.8	0.92	0.00047	5.676223
P01024	1	1	511.9383	1533.8	0.92	0.00047	5.676223
P01024	1	0.0189	767.4045	1533.802	1.81	0.00139	4.351643
P01024	1		767.4045	1533.802	1.81	0.00139	4.351643
P01024	0	0.8889	717.3589	1433.711	0.28	0.0002	25.6715
P01024	0	1	717.3594	1433.711	0.96	0.00069	3.758771
P01024	0	1	523.0405	2089.14	2.33	0.00122	2.793945
P01024	0	0.8511	697.0518	2089.141	2.63	0.00183	8.278879
P01024	0	0.9365	697.0513	2089.139	1.93	0.00134	11.01583
P01024	0	1	604.6191	1811.843	2.1	0.00127	26.4688
P01024	0	1	906.4241	1811.841	1.06	0.00096	3.07593
P01024	0	1	697.0515	2089.14	2.19	0.00153	5.108894
P01024	0	1	523.0397	2089.137	0.7	0.00036	7.734283

P01024	1	0.9138	740.3995	1479.792	1.69	0.00125	7.956345
P01024	1	0.85	493.9354	1479.792	1.64	0.00081	11.75138
P01024	0	1	1045.073	2089.14	2.05	0.00214	0
P01024	0	0.9184	478.5754	1433.711	0.97	0.00047	0
P01024	0	1	717.3599	1433.713	1.73	0.00124	7.452931
P01024	0	0.9464	478.5754	1433.712	1.16	0.00056	5.117208
P01024	1	1	511.9386	1533.801	1.58	0.00081	18.14629
P01024	1	0.9302	591.0009	1770.988	2.82	0.00166	7.884392
P01024	0	1	908.4843	1815.961	3.78	0.00343	11.60523
P01024	0	1	1253.224	2505.441	2.17	0.00272	1.435377
P01024	0	1	834.3733	1667.739	1.38	0.00115	2.135336
P01024	0	1	714.978	3570.861	2.87	0.00205	7.213333
P01024	1	1	761.4207	2282.248	4.08	0.0031	16.4874
P01024	1	1	590.9993	1770.983	0.13	0.00008	5.098004
P01024	0	1	803.0869	2407.246	2.18	0.00175	0
P01024	0	1	1190.957	3570.856	1.6	0.0019	3.82868
P01024	0	0.9794	835.818	2505.439	1.66	0.00138	4.077807
P01024	0	1	893.4719	3570.866	4.31	0.00385	1.44973
P01024	0	0.9189	404.7183	1615.851	3.22	0.0013	0
P01024	1	1	591.0018	1770.991	4.37	0.00258	0
P01024	1	0.9608	591.0002	1770.986	1.78	0.00105	44.57871
P01024	1	1	591.0009	1770.988	2.82	0.00166	3.603768
P01024	1	1	591.0011	1770.989	3.23	0.00191	11.03367
P01024	1	1	594.017	1780.036	0.22	0.00013	100
P01024	1	1	591.0014	1770.99	3.75	0.00221	6.844754
P01024	0	0.8776	627.1162	2505.443	2.99	0.00187	7.642929
P01024	1	0.8214	586.8345	2344.316	4.85	0.00284	15.3471
P01024	0	1	717.3601	1433.713	1.98	0.00142	3.097755
P01024	0	0.6596	552.332	1103.657	2.34	0.00129	12.32221
P01024	0	1	647.8582	2588.411	0.7	0.00045	15.99419
P01024	0	1	649.0156	1945.032	-0.53	-0.00034	22.79784
P01024	0	1	863.4756	2588.412	1.15	0.001	2.189114
P01024	0	1	478.5763	1433.714	2.95	0.00141	2.969398
P01024	0	0.7213	552.3322	1103.657	2.67	0.00147	2.507487
P01024	1	1	511.9386	1533.801	1.58	0.00081	18.14629
P01024	0	0.7222	552.3317	1103.656	1.89	0.00105	3.611325
P01024	0	0.9091	649.0176	1945.038	2.49	0.00161	13.73777
P01024	0	0.7963	552.3319	1103.656	2.12	0.00117	5.563288
P01024	0	0.8649	533.306	2130.202	3.44	0.00183	22.98233
P01024	0	1	697.0519	2089.141	2.81	0.00195	17.67736
P01024	0	1	523.04	2089.138	1.4	0.00073	20.161
P01024	0	0.2826	580.3702	1159.733	3.63	0.0021	11.92777
P01024	0	1	1019.58	2038.153	1.88	0.00192	0
P01024	0	0.9524	680.0558	2038.153	1.54	0.00105	5.07559
P01024	0	0.7416	872.7719	2616.301	1.97	0.00172	3.75364
P01024	0	1	631.3056	1261.604	1.62	0.00102	0
P01024	0	1	1039.54	2078.073	2.65	0.00275	6.867674
P01024	0	0.8906	555.8184	1110.63	2.68	0.00149	29.48924
P01024	0	0.5385	479.6216	1436.85	2.67	0.00128	69.31403
P01024	0	1	644.6965	1932.075	2.04	0.00131	1.960783
P01024	0	0.8358	648.0357	1942.093	1.95	0.00126	32.6348
P01024	0	0.9398	748.9081	1496.809	1.84	0.00138	6.352702

P01024	0	0.7143	479.6211	1436.849	1.65	0.00079	16.92912
P01024	0	1	565.9473	1695.827	1.41	0.0008	37.40307
P01024	0	1	604.6191	1811.843	2	0.00121	9.552627
P01024	0	1	693.3632	2078.075	3.53	0.00244	11.78732
P01024	0	1	1013.18	3037.524	1.56	0.00158	3.17395
P01024	0	0.94	520.2738	2078.073	2.7	0.0014	8.665817
P01024	0	1	786.7077	2358.109	0.87	0.00068	2.414361
P01024	0	1	738.6743	2214.008	1.79	0.00132	0
P01024	0	1	549.0034	1644.996	3.7	0.00203	12.73141
P01024	0	0.9701	1179.559	2358.112	2.17	0.00256	1.179302
P01024	0	1	692.7307	2076.177	2.04	0.00141	6.744061
P01024	0	1	1179.558	2358.109	1.24	0.00146	0
P01024	0	1	786.7083	2358.11	1.65	0.00129	2.17309
P01024	0	1	1107.507	2214.007	1.1	0.00122	0
P01024	0	1	786.707	2358.106	-0.06	-0.00005	10.78449
P01024	0	0.9875	786.7071	2358.107	0.09	0.00007	2.401644
P01024	0	0.9818	738.6736	2214.006	0.79	0.00059	0
P01024	0	0.907	1179.558	2358.108	0.62	0.00073	7.491054
P01024	0	1	786.7083	2358.11	1.57	0.00123	1.757187
P01024	0	0.9516	786.7087	2358.112	2.19	0.00172	2.697949
P01024	0	0.9512	786.7091	2358.113	2.66	0.00209	0
P01024	0	1	1179.558	2358.109	1.24	0.00146	0
P01024	0	1	1179.559	2358.111	2.07	0.00244	0
P01024	0	1	786.7083	2358.11	1.57	0.00123	2.31205
P01024	0	0.9403	648.0364	1942.095	3.08	0.002	11.95694
P01024	0	1	760.1368	3037.526	2.01	0.00153	2.866978
P01024	0	1	1179.559	2358.11	1.55	0.00183	0
P01024	0	1	676.8575	1352.708	2.77	0.00187	8.078828
P01024	0	1	786.7084	2358.111	1.72	0.00135	12.62063
P01024	0	1	786.7081	2358.11	1.34	0.00105	2.66931
P01024	0	1	412.0035	1644.992	1.43	0.00059	11.60258
P01024	0	1	738.6763	2214.014	4.43	0.00327	0
P01024	0	1	786.7083	2358.11	1.57	0.00123	2.493541
P01024	0	0.9832	1179.559	2358.111	1.96	0.00231	0
P01024	0	0.9804	786.7082	2358.11	1.49	0.00117	12.65049
P01024	0	1	1107.507	2214.007	1.32	0.00147	0
P01024	0	1	786.7073	2358.107	0.4	0.00032	0
P01024	0	1	549.0028	1644.994	2.58	0.00142	12.55894
P01024	1	0.9231	761.4193	2282.243	2.15	0.00164	5.187278
P01024	0	1	693.3612	2078.069	0.53	0.00037	100
P01024	0	1	786.7083	2358.11	1.65	0.00129	2.381968
P01024	0	1	644.6955	1932.072	0.43	0.00027	7.762418
P01024	0	1	786.708	2358.109	1.26	0.00099	4.247067
P01024	0	0.9828	786.7084	2358.111	1.8	0.00142	0
P01024	0	1	519.7998	2076.177	2.03	0.00105	4.927312
P01024	0	0.95	648.0366	1942.095	3.27	0.00212	51.28757
P01024	0	1	692.7293	2076.173	0.1	0.00007	4.260423
P01024	0	1	693.3629	2078.074	3	0.00208	0
P01024	0	1	908.4837	1815.96	3.17	0.00288	6.799383
P01024	0	0.7963	648.0359	1942.093	2.23	0.00145	0
P01024	0	1	1107.508	2214.008	1.54	0.00171	0
P01024	0	1	786.7076	2358.108	0.79	0.00062	2.086887

P01024	0	0.9787	738.6735	2214.006	0.63	0.00046	0
P01024	0	0.9912	1179.558	2358.108	0.62	0.00073	0
P01024	0	1	1107.507	2214.007	1.1	0.00122	1.135485
P01024	0	1	549.0018	1644.991	0.69	0.00038	55.38434
P01024	0	0.9905	786.7073	2358.107	0.4	0.00032	11.98888
P01024	0	1	693.3613	2078.069	0.79	0.00055	7.245576
P01024	0	1	1179.558	2358.109	1.13	0.00134	0
P01024	0	1	786.7079	2358.109	1.1	0.00087	2.193598
P01024	0	1	786.708	2358.109	1.26	0.00099	0
P01024	0	0.9412	1179.559	2358.11	1.55	0.00183	0
P01024	0	1	786.7089	2358.112	2.34	0.00184	3.404822
P01024	0	0.6889	648.0358	1942.093	2.14	0.00139	10.80001
P01024	0	1	710.7368	2130.196	0.43	0.00031	6.630315
P01024	0	1	906.4243	1811.841	1.27	0.00115	3.480999
P01024	0	0.9592	533.3053	2130.199	2.06	0.0011	4.179342
P01024	0	0.7	479.6211	1436.849	1.71	0.00082	9.988125
P01024	0	0.931	710.7381	2130.2	2.24	0.00159	20.30905
P01024	0	0.8125	533.3049	2130.198	1.37	0.00073	3.872327
P01024	0	1	756.4155	1511.824	1.32	0.001	0
P01024	1	0.8039	761.419	2282.242	1.83	0.00139	4.587765
P01024	0	1	703.3564	1405.705	1.1	0.00077	2.935766
P01024	0	0.9268	469.2397	1405.704	0.33	0.00015	5.110014
P01024	0	0.9552	703.3562	1405.705	0.84	0.00059	3.24194
P01024	0	0.9778	710.7373	2130.197	1.12	0.0008	41.96458
P01024	0	0.6071	533.3054	2130.2	2.18	0.00116	8.330234
P01024	0	0.939	469.2402	1405.706	1.5	0.0007	8.418099
P01024	0	0.8986	469.2401	1405.706	1.24	0.00058	11.80439
P01024	0	0.9848	756.4179	1511.828	4.47	0.00338	0
P01024	0	1	505.6265	1514.865	3.06	0.00155	31.84282
P01024	1	0.9474	761.4187	2282.242	1.43	0.00109	6.781034
P01024	0	1	697.0507	2089.137	0.97	0.00067	41.91027
P01024	0	0.7143	479.621	1436.849	1.52	0.00073	9.121378
P01024	0	0.9839	604.6186	1811.841	1.19	0.00072	4.647392
P01024	0	0.6977	648.0353	1942.091	1.39	0.0009	3.402536
P01024	0	1	989.8251	2967.461	0.59	0.00058	24.44282
P01024	0	0.675	479.6217	1436.85	2.86	0.00137	14.99168
P01024	0	1	693.3626	2078.073	2.56	0.00177	8.533365
P01024	0	1	604.6196	1811.844	2.91	0.00176	4.846468
P01024	0	0.8537	648.036	1942.093	2.33	0.00151	14.80847
P01024	0	1	906.4246	1811.842	1.6	0.00145	3.176332
P01024	1	1	545.9832	1635.935	0.62	0.00034	6.662004
P01024	0	0.7692	718.9288	1436.85	2.72	0.00195	10.50089
P01024	0	0.9	604.6193	1811.843	2.3	0.00139	1.624027
P01024	0	0.8684	880.0891	2638.253	1	0.00088	5.065908
P01024	0	1	450.9034	1350.696	2.24	0.00101	12.3407
P01024	1	1	438.9665	1314.885	1.23	0.00054	9.635981
P01024	0	1	692.7321	2076.182	4.07	0.00281	7.86514
P01024	0	0.9756	644.697	1932.076	2.7	0.00174	25.59362
P01024	0	1	601.2949	1801.87	2.27	0.00136	24.68689
P01024	0	0.3721	580.3699	1159.733	3.21	0.00186	10.01686
P01024	0	0.9512	504.9394	1512.804	1.64	0.00083	11.66531
P01024	0	0.973	539.2869	1615.846	0.06	0.00003	0

P01024	1	0.9032	571.3172	2282.247	3.8	0.00217	19.10826
P01024	1	1	605.3342	1813.988	2.06	0.00124	9.836341
P01024	0	0.7213	565.8727	1130.738	-0.44	-0.00025	5.664781
P01024	0	0.8462	523.0407	2089.141	2.68	0.0014	0
P01024	0	0.7818	547.3486	1093.69	3.59	0.00196	34.84159
P01024	0	0.8974	622.3387	2486.333	-0.46	-0.00028	6.755883
P01024	0	0.6316	452.59	1355.755	0.18	0.00008	59.01131
P01024	0	1	885.4464	3538.764	0.69	0.00061	0
P01024	1	0.5	464.6212	1391.849	1.52	0.00071	21.85379
P01024	0	1	738.6733	2214.005	0.46	0.00034	5.27091
P01024	0	0.9286	404.7161	1615.843	-2.14	-0.00087	67.11542
P01024	0	0.7027	648.0366	1942.095	3.37	0.00218	14.99859
P01024	0	0.7273	409.8924	1227.663	2.03	0.00083	0
P01024	0	0.9375	600.6632	1799.975	3.18	0.00191	46.48963
P01024	0	1	549.0018	1644.991	0.69	0.00038	0
P01024	1	1	421.2282	1681.891	0.1	0.00004	12.58134
P01024	0	0.6296	583.8662	1166.725	3.6	0.0021	31.76363
P01024	0	0.9375	523.9383	1569.8	0.3	0.00015	0
P01024	0	0.8718	552.6473	1655.927	2.15	0.00119	25.54733
P01024	0	0.6222	722.8503	1444.693	2.36	0.0017	9.415826
P01024	1	1	493.9346	1479.789	0.03	0.00001	8.371916
P01024	0	0.9643	404.7177	1615.849	1.79	0.00072	52.982
P01024	0	0.7609	409.8924	1227.663	1.88	0.00077	6.827866
P01024	0	0.6857	648.0349	1942.09	0.73	0.00047	31.79472
P01024	0	0.7347	587.8698	1174.732	3.14	0.00184	32.21711
P01024	0	1	786.7064	2358.105	-0.76	-0.0006	12.56548
P01024	0	1	685.871	1370.735	2.44	0.00167	0
P01024	0	0.7727	710.7374	2130.198	1.21	0.00086	19.86711
P01024	0	0.95	601.2952	1801.871	2.78	0.00167	0
P01024	1	1	470.6114	1409.82	1.36	0.00064	52.75338
P01024	0	0.9516	603.7999	1206.592	1.62	0.00098	0
P01024	2	1	511.5511	2043.183	1.21	0.00062	16.72536
P01024	0	0.6727	614.3343	1227.661	0.87	0.00054	3.231944
P01024	2	0.7895	397.7635	1588.032	3.3	0.00131	32.61517
P01024	0	0.2609	580.3684	1159.73	0.58	0.00033	15.04164
P01024	0	0.46	377.5851	1130.741	1.76	0.00066	8.214984
P01024	0	0.9706	539.2885	1615.851	2.89	0.00156	5.965941
P01024	0	1	602.5659	2407.242	0.45	0.00027	69.37263
P01024	0	1	552.6475	1655.928	2.59	0.00143	2.689092
P01024	2	0.913	513.5429	2051.15	2.01	0.00103	38.46426
P01024	1	0.75	571.316	2282.242	1.77	0.00101	17.36365
P01024	0	0.8286	648.0356	1942.092	1.86	0.0012	25.22486
P01024	1	1	421.2298	1681.897	3.8	0.0016	12.73531
P01024	0	1	670.6868	2010.046	2.23	0.00149	5.594165
P01024	0	1	595.9638	1785.877	3.17	0.00188	74.90744
P01024	0	1	667.8663	1334.725	2.07	0.00138	15.26435
P01024	0	1	605.9918	1815.961	3.6	0.00218	19.28808
P01024	0	1	697.0502	2089.136	0.26	0.00018	73.80787
P01024	0	0.4595	648.0366	1942.095	3.27	0.00212	0
P01024	0	0.7778	519.7997	2076.177	1.8	0.00093	19.37876
P01024	0	1	786.7055	2358.102	-1.93	-0.00151	27.17006
P01024	1	0.8889	571.3151	2282.239	0.16	0.00009	40.56333

P01024	0	0.2941	460.9499	1380.835	0.76	0.00035	13.82021
P01024	1	1	761.4188	2282.242	1.51	0.00115	42.94876
P01024	0	0.5405	438.2447	1312.72	1.74	0.00076	44.26881
P01024	0	0.7419	880.0883	2638.25	0.1	0.00008	4.718264
P01024	0	0.9667	693.3631	2078.075	3.35	0.00232	32.59631
P01024	1	0.9583	1015.172	3043.501	1.95	0.00198	3.932519
P01024	0	1	906.4251	1811.843	2.08	0.00188	0
P01024	1	0.7647	531.6553	1592.951	2.38	0.00127	11.42001
P01024	0	0.5312	648.0356	1942.092	1.76	0.00114	46.21769
P01024	0	0.7857	829.4521	2486.342	3.08	0.00256	0
P01024	0	0.931	450.903	1350.694	1.29	0.00058	26.43
P01024	1	1	445.7653	1780.039	1.9	0.00084	35.36129
P01024	0	1	803.086	2407.243	1.11	0.00089	0
P01024	0	0.725	499.608	1496.809	2.16	0.00108	9.003232
P01024	0	0.9545	885.4476	3538.769	2.07	0.00183	4.642412
P01024	0	0.9375	889.4466	3554.765	2.32	0.00206	7.138779
P01024; O9	0	0.64	532.3343	1063.661	1.11	0.00059	0
P01024	0	1	595.8157	1190.624	3.16	0.00188	0
P01024	0	0.7321	587.8703	1174.733	4.08	0.00239	5.006826
P01024	0	0.3061	618.8714	1236.736	2.85	0.00176	0
P01024	0	0.9512	523.9369	1569.796	-2.39	-0.00125	20.28429
P01024	0	0.6757	599.3275	1795.968	3.63	0.00218	9.194969
P01024	0	0.9474	538.9665	1614.885	1.86	0.001	0
P01024	0	0.8276	786.7083	2358.11	1.65	0.00129	10.04483
P01024	1	1	493.9359	1479.793	2.57	0.00127	50.85025
P01024	1	0.88	571.3156	2282.241	1.02	0.00058	3.871119
P01024	0	0.7843	587.8693	1174.731	2.31	0.00136	19.15297
P01024	0	0.7561	418.915	1254.73	1.31	0.00055	25.02604
P01024	0	0.9412	807.9462	1614.885	2.02	0.00163	4.310209
P01024	0	0.8667	880.0895	2638.254	1.48	0.0013	11.69024
P01024	0	0.8857	545.3077	1633.909	2.9	0.00158	4.725809
P01024	0	1	711.7586	3554.764	2.15	0.00153	6.804381
P01024	0	0.9512	451.2561	1351.754	3.33	0.0015	4.115806
P01024	0	0.6111	700.6988	2100.082	2.16	0.00151	4.487957
P01024	1	1	444.2823	1330.832	2	0.00089	10.5811
P01024	0	0.9259	786.7091	2358.113	2.66	0.00209	12.55509
P01024	0	0.7391	404.7174	1615.848	1.11	0.00045	10.01746
P01024	0	0.8261	697.0527	2089.144	3.95	0.00275	0
P01024	0	0.5122	460.9497	1380.835	0.37	0.00017	4.090551
P01024	1	1	542.9669	1626.886	3.11	0.00169	42.1939
P01024	0	0.7727	552.3318	1103.656	2.01	0.00111	0
P01024	0	0.8	452.5906	1355.757	1.6	0.00072	3.779116
P01024	1	0.6875	493.9352	1479.791	1.27	0.00062	15.88511
P01024	1	0.2857	480.9438	1440.817	3.2	0.00154	18.81922
P01024	0	0.619	501.7972	1002.587	1.8	0.0009	3.435703
P01024	0	0.6667	648.0366	1942.095	3.27	0.00212	38.26043
P01024	1	0.4444	513.2687	1537.792	1.79	0.00092	7.464835
P01024	1	0.7619	841.4518	1681.896	3.16	0.00266	0
P01024	0	0.8065	880.0885	2638.251	0.3	0.00027	2.900106
P01024	0	0.8889	648.6973	1944.077	3.1	0.00201	6.263824
P01024	0	0.9429	757.922	1514.837	2.12	0.00161	3.076235
P01024	1	1	493.9353	1479.791	1.33	0.00066	14.62449

P01024	0	0.4688	382.9013	1146.689	1.85	0.00071	8.958314
P01024	0	0.7	392.2488	1174.732	2.69	0.00105	12.31742
P01024	0	0.8077	693.3602	2078.066	-0.79	-0.00055	0
P01024	0	0.1579	431.5867	1292.746	1.04	0.00045	62.15388
P01024	0	0.8261	547.3486	1093.69	3.7	0.00202	24.98479
P01024	0	1	533.3055	2130.2	2.41	0.00128	63.28512
P01024	1	0.7692	464.6213	1391.849	1.65	0.00077	15.02949
P01024	0	1	545.5769	2179.286	2.61	0.00142	23.56545
P01024	0	0.2558	580.3687	1159.73	1.1	0.00064	5.791723
P01024	0	0.2857	648.0354	1942.092	1.48	0.00096	36.76591
P01024	0	0.9697	670.6865	2010.045	1.87	0.00125	3.212496
P01024; O9	0	0.62	532.3347	1063.662	1.8	0.00096	29.72895
P01024	0	0.8611	692.7316	2076.18	3.36	0.00233	6.759931
P01024	0	0.8846	519.8016	2076.184	5.44	0.00282	26.76847
P01024	0	1	713.3527	1425.698	0.26	0.00018	18.99341
P01024	0	1	714.38	1427.753	5.48	0.00391	7.388453
P01024	0	0.86	606.331	1211.655	1.3	0.00079	0
P01024	0	0.32	618.8709	1236.734	1.96	0.00121	1.957112
P01024	1	0.7391	571.3159	2282.242	1.45	0.00083	1.791034
P01024	0	1	687.5389	2747.134	1.93	0.00133	5.373046
P01024	0	0.8974	595.3314	1783.98	2.86	0.0017	7.454174
P01024	0	0.6667	545.3093	1633.913	5.7	0.0031	25.47194
P01024	0	0.6905	718.9286	1436.85	2.47	0.00177	11.08955
P01024	0	0.5312	648.0363	1942.094	2.8	0.00181	36.31767
P01024	0	0.8222	547.3475	1093.688	1.69	0.00092	45.18101
P01024	0	0.4483	603.8007	1206.594	2.94	0.00177	27.39233
P01024	0	0.9524	603.7999	1206.593	1.72	0.00104	11.77612
P01024	0	0.7636	510.3035	1019.6	2.21	0.00113	20.77922
P01024	0	0.7959	484.7924	968.5774	1.72	0.00083	24.91194
P01024	0	0.8	418.915	1254.731	1.53	0.00064	20.23393
P01024	2	1	696.4076	2782.609	6.07	0.00422	24.44337
P01024	0	0.68	546.8306	1092.654	3.27	0.00179	20.75942
P01024	0	0.7419	499.6073	1496.807	0.75	0.00037	33.14555
P01024	0	0.6279	656.8652	1312.723	4.38	0.00288	25.65546
P01024	0	0.2353	438.245	1312.72	2.37	0.00104	9.080698
P01024	0	0.9091	1005.526	2010.045	1.78	0.00179	0
P01024	1	0.9714	503.9535	1509.846	2.6	0.00131	17.91397
P01024	0	0.8333	807.9468	1614.886	2.7	0.00218	0
P01024	0	0.4146	377.5851	1130.741	1.84	0.00069	5.956144
P01024	1	0.7143	493.9356	1479.792	1.89	0.00093	24.89801
P01024	0	1	491.2533	1471.745	0.86	0.00042	0
P01024	1	1	421.2299	1681.898	4.02	0.00169	11.76248
P01024	1	0.5806	509.6469	1526.926	0.33	0.00017	11.51297
P01024	0	1	708.5617	3538.779	5.02	0.00355	6.348145
P01024	0	1	818.7404	2454.207	-0.08	-0.00007	3.260694
P01024	0	0.3846	648.0365	1942.095	3.18	0.00206	11.15637
P01024	0	1	595.815	1190.623	1.93	0.00115	0.988152
P01024	1	0.9487	659.0031	1974.995	2.45	0.00161	4.105151
P01024	0	0.8621	818.7399	2454.205	-0.68	-0.00056	3.202533
P01024	0	1	667.8659	1334.725	1.53	0.00102	3.419898
P01024	1	0.9487	659.0031	1974.995	2.45	0.00161	4.105151
P01024	0	0.5882	690.9219	1380.837	1.83	0.00127	13.61996

P01024	0	0.4483	382.9015	1146.69	2.33	0.00089	8.019503
P01024	0	0.9565	708.5599	3538.771	2.6	0.00184	4.174822
P01024	1	1	668.9388	1336.87	2.06	0.00138	5.169005
P01024	0	1	705.8653	1410.723	2.26	0.00159	3.73944
P01024	1	0.913	378.2163	1509.843	0.99	0.00037	13.65654
P01024	0	0.8409	484.7918	968.5762	0.46	0.00022	41.4712
P01024	1	0.4286	509.647	1526.927	0.57	0.00029	27.55706
P01024	0	1	880.0903	2638.256	2.39	0.0021	2.396597
P01024	0	0.8444	547.3482	1093.689	2.92	0.0016	0
P01024	1	0.4048	410.2561	1638.003	3.39	0.00139	3.976753
P01024	0	0.9302	807.9479	1614.889	4.14	0.00334	0
P01024	0	1	505.6141	1514.828	-3.85	-0.00194	28.33048
P01024	0	0.7414	587.8704	1174.733	4.18	0.00246	8.474478
P01024	0	0.3514	387.2486	1159.731	2.04	0.00079	11.84036
P01024	0	0.7021	546.831	1092.655	4.05	0.00221	3.266834
P01024	0	0.7692	418.9153	1254.731	2.04	0.00085	14.52722
P01024	0	0.2778	438.2442	1312.718	0.49	0.00021	8.690989
P01024	0	0.9636	1107.508	2214.01	2.32	0.00256	0
P01024	0	1	510.9579	1530.859	2.6	0.00133	14.25692
P01024	0	0.641	656.8637	1312.72	2.15	0.00141	54.50566
P01024	0	0.8788	880.0895	2638.254	1.41	0.00124	14.36848
P01024	0	1	1090.146	2179.285	2.34	0.00255	0
P01024	0	0.7105	718.9287	1436.85	2.63	0.00189	12.14149
P01024	1	0.7308	559.9744	1677.909	4.62	0.00258	30.26162
P01024	0	1	445.5797	1334.724	1.49	0.00066	5.667696
P01024	0	0.9796	645.3084	1289.609	1.08	0.0007	26.4501
P01024	0	0.8846	505.6169	1514.836	1.71	0.00086	51.20999
P01024	1	0.5	509.6472	1526.927	0.93	0.00048	70.21364
P01024	0	1	504.6129	1511.824	1.68	0.00085	5.873374
P01024	0	0.9394	499.6079	1496.809	2.03	0.00101	6.715208
P01024	1	1	421.229	1681.894	1.91	0.0008	22.77313
P01024	2	1	702.1777	3506.859	-1.73	-0.00121	0.276747
P01024	0	0.8667	880.0887	2638.252	0.51	0.00045	2.49393
P01024	0	0.5385	482.7749	1928.078	0.6	0.00029	9.641109
P01024	0	0.5538	493.8236	986.6399	3.28	0.00162	12.41327
P01024	0	0.9516	603.8004	1206.594	2.53	0.00153	34.70613
P01024	1	1	493.9356	1479.792	1.89	0.00093	49.40816
P01024	0	1	764.3989	2291.182	3.16	0.00241	0
P01024	0	0.7292	404.5564	1211.655	1.17	0.00047	0
P01024	1	0.6786	691.3656	2762.441	0.16	0.00011	11.3296
P01024	0	1	595.8152	1190.623	2.23	0.00133	0.933831
P01024	0	0.8611	418.9151	1254.731	1.67	0.0007	56.51604
P01024	0	1	667.8658	1334.724	1.43	0.00096	19.19712
P01024	0	0.7903	510.3032	1019.599	1.62	0.00082	7.884241
P01024	0	1	595.8157	1190.624	3.05	0.00182	0
P01024	0	0.8	499.6081	1496.81	2.4	0.0012	15.24384
P01024	0	0.9643	786.7085	2358.111	1.88	0.00148	11.44798
P01024	0	0.875	648.0361	1942.094	2.52	0.00163	45.95354
P01024	0	0.8667	599.3306	1795.977	8.83	0.00529	5.760993
P01024	0	0.4167	382.9012	1146.689	1.45	0.00055	14.87472
P01024	0	0.9348	573.8296	1146.652	2.82	0.00162	24.53609
P01024	0	0.7174	583.8094	1166.612	3.2	0.00186	4.421603

P01024	0	0.7059	555.8184	1110.629	2.57	0.00143	0
P01024	0	0.871	649.0172	1945.037	1.92	0.00125	21.66901
P01024	1	0.8723	885.9965	1770.986	1.48	0.00131	3.572867
P01024	0	0.4091	583.8655	1166.724	2.45	0.00143	2.912181
P01024	0	0.4318	618.8712	1236.735	2.45	0.00152	0
P01024	0	0.9706	504.6119	1511.821	-0.26	-0.00013	7.411877
P01024	0	0.7297	599.3269	1795.966	2.61	0.00156	48.49725
P01024	1	0.9535	839.4558	1677.904	2.01	0.00169	7.570782
P01024	0	0.8929	1159.095	2317.182	-0.18	-0.00021	0
P01024	1	0.6471	464.6212	1391.849	1.46	0.00068	11.90851
P01024	1	1	761.4187	2282.242	1.43	0.00109	6.976895
P01024	0	0.4062	648.0374	1942.098	4.5	0.00291	29.0237
P01024	2	1	513.544	2051.154	4.03	0.00207	31.61909
P01024	0	0.7321	587.8702	1174.733	3.87	0.00227	10.16192
P01024	0	0.7895	450.9031	1350.695	1.57	0.0007	25.40508
P01024	1	1	561.3036	1681.896	3.12	0.00175	23.94676
P01024	0	0.875	885.4478	3538.769	2.28	0.00201	5.602897
P01024; O9	0	0.6735	532.3348	1063.662	2.14	0.00114	12.74101
P01024	0	0.8889	547.3476	1093.688	1.8	0.00099	27.57678
P01024	0	0.5833	885.4472	3538.767	1.59	0.0014	5.304733
P01024	0	0.9118	452.5892	1355.753	-1.58	-0.00071	40.30692
P01024	0	0.619	602.5674	2407.248	2.88	0.00173	8.66717
P01024	0	1	738.6745	2214.009	2.03	0.0015	68.09644
P01024	0	0.9333	504.7896	2016.136	0.27	0.00013	28.92328
P01024	0	0.7692	648.6968	1944.076	2.25	0.00146	13.05819
P01024	0	0.881	532.8397	1064.672	1.45	0.00077	10.64838
P01024	0	0.3333	431.5873	1292.747	2.46	0.00106	14.96847
P01024	1	0.6429	410.2564	1638.004	3.91	0.0016	39.34185
P01024	1	0.5789	509.648	1526.929	2.43	0.00124	40.71249
P01024	0	0.7021	546.83	1092.653	2.26	0.00124	5.814992
P01024	0	0.2955	580.3701	1159.733	3.42	0.00198	0
P01024	0	1	738.6751	2214.011	2.78	0.00205	0
P01024	0	0.9455	584.8134	1168.62	3.68	0.00215	5.499272
P01024	0	1	539.2888	1615.852	3.57	0.00192	48.38189
P01024	1	0.1081	683.375	1365.743	2.11	0.00144	34.94502
P01024	1	0.9545	526.0393	2101.135	0.45	0.00024	29.17065
P01024	0	0.7381	404.5559	1211.653	0.11	0.00004	10.16623
P01024	0	0.4	530.2695	1588.794	1.32	0.0007	4.461038
P01024	0	0.8036	510.3033	1019.599	1.86	0.00095	5.634512
P01024	0	1	786.7092	2358.113	2.73	0.00215	26.34435
P01024	0	0.6667	546.8294	1092.652	1.15	0.00063	43.82499
P01024	0	0.7	656.8638	1312.72	2.24	0.00147	10.86452
P01024	0	0.7321	516.7971	1032.587	2.28	0.00118	4.9656
P01024	0	0.75	476.5879	1427.749	2.88	0.00137	9.500804
P01024	0	1	818.7427	2454.213	2.75	0.00225	29.58549
P01024	0	0.8788	643.3632	1928.075	-0.81	-0.00052	25.97827
P01024	0	0.9167	1179.559	2358.11	1.44	0.0017	0.870138
P01024	0	1	738.6742	2214.008	1.62	0.0012	2.592724
P01024	2	1	587.3208	2346.261	5.37	0.00315	0
P01024	0	0.5588	487.0146	1945.036	1.57	0.00076	0
P01024	0	0.8125	573.8293	1146.651	2.29	0.00131	12.43238
P01024	0	0.5833	431.5877	1292.748	3.23	0.00139	67.68532

P01024; O9	0	0.58	532.3359	1063.664	4.09	0.00218	14.26944
P01024	0	1	450.9027	1350.694	0.75	0.00034	46.27835
P01024	0	0.9565	450.9034	1350.696	2.38	0.00107	15.38576
P01024	1	1	561.3032	1681.895	2.47	0.00138	13.82616
P01024; O9	0	0.5814	532.3342	1063.661	0.88	0.00047	6.692185
P01024	1	1	421.2289	1681.894	1.62	0.00068	46.6876
P01024	0	0.5172	648.0355	1942.092	1.67	0.00108	30.70085
P01024	0	0.4375	382.9014	1146.69	2.09	0.0008	7.968709
P01024	0	1	499.6076	1496.808	1.36	0.00068	19.43667
P01024	0	1	478.5756	1433.712	1.55	0.00074	0
P01024	0	0.8298	532.8407	1064.674	3.28	0.00175	25.85593
P01024; O9	0	0.4091	532.3358	1063.664	3.86	0.00206	60.61178
P01024	1	0.8214	572.797	2288.166	1.91	0.00109	16.93087
P01024	0	0.7692	515.8178	1030.628	1.82	0.00094	27.12353
P01024	1	0.619	668.4338	1335.86	2.43	0.00163	10.67565
P01024	0	0.7917	599.3273	1795.967	3.33	0.00199	0
P01024	0	0.3654	493.8231	986.639	2.35	0.00116	20.43558
P01024	0	0.7812	515.818	1030.629	2.17	0.00112	8.192772
P01024	0	1	1446.123	2891.239	2.81	0.00407	0
P01024	0	0.8214	643.3683	1928.09	7.16	0.0046	16.33719
P01024	0	0.44	648.0344	1942.089	-0.12	-0.00008	11.52841
P01024	0	0.3846	549.0026	1644.993	2.14	0.00117	65.45551
P01024	0	1	478.576	1433.714	2.38	0.00114	13.83794
P01024	2	1	702.1763	3506.853	-3.64	-0.00255	11.5659
P01024	0	0.619	588.8493	1176.691	3.96	0.00233	14.91845
P01024	0	0.25	479.6211	1436.849	1.71	0.00082	8.092014
P01024	2	0.0345	477.517	1907.046	1.4	0.00067	47.09988
P01024	0	0.9429	717.3608	1433.714	2.92	0.00209	0
P01024	0	0.5333	583.8097	1166.612	3.72	0.00217	2.005855
P01024	0	0.6842	392.249	1174.732	3.23	0.00127	7.749425
P01024	0	0.7838	645.8811	1290.755	3.26	0.0021	28.04943
P01024	0	0.8077	781.4174	2342.238	2.37	0.00185	2.017628
P01024	0	0.925	532.8394	1064.672	0.87	0.00046	45.72959
P01024	0	0.4815	382.9014	1146.69	2.09	0.0008	8.235432
P01024	0	0.7317	546.8295	1092.652	1.37	0.00075	16.09895
P01024; O9	0	0.7091	460.2835	919.5597	1.83	0.00084	3.409078
P01024	1	0.7692	571.3178	2282.249	4.76	0.00272	7.314544
P01024	0	0.7568	627.8693	1254.731	2.17	0.00136	19.40027
P01024	0	0.5455	480.9409	1440.808	-0.49	-0.00024	7.730431
P01024	0	0.8214	622.3397	2486.337	1.11	0.00069	33.68331
P01024	1	0.9091	503.9522	1509.842	0.17	0.00009	36.15313
P01024	1	0.95	571.3153	2282.239	0.38	0.00022	10.12941
P01024	0	0.6341	588.8485	1176.69	2.51	0.00148	0
P01024	0	0.7143	588.8495	1176.692	4.27	0.00251	20.67884
P01024	0	0.7667	552.3311	1103.655	0.68	0.00037	11.63104
P01024	0	0.9062	828.4669	1655.927	1.72	0.00142	6.508906
P01024	1	1	561.3042	1681.898	4.21	0.00236	0
P01024	0	0.9231	470.9121	1410.722	1.23	0.00058	46.46935
P01024	0	0.775	404.5564	1211.655	1.24	0.0005	7.829526
P01024	0	0.8696	885.4484	3538.772	2.97	0.00262	4.292865
P01024	0	0.9062	451.2558	1351.753	2.65	0.0012	2.493932
P01024	0	0.931	504.9394	1512.804	1.7	0.00086	9.625627

P01024	0	1	622.3408	2486.341	2.88	0.00179	56.91244
P01024	1	1	567.9521	1701.842	1.79	0.00101	4.987516
P01024	0	0.5217	523.9392	1569.803	1.93	0.00101	9.810185
P01024	0	1	595.8147	1190.622	1.41	0.00084	29.86287
P01024	0	1	774.0101	2320.016	-0.26	-0.0002	64.8847
P01024	0	0.9615	667.8656	1334.724	1.07	0.00071	36.45114
P01024	0	0.4286	648.0367	1942.096	3.55	0.0023	27.95452
P01024	0	0.6905	591.8643	1182.721	4.55	0.00269	15.44733
P01024	0	0.3889	509.5517	2035.185	3.34	0.0017	39.1162
P01024	0	0.8276	880.0883	2638.25	0.1	0.00008	1.559483
P01024	0	0.4643	648.0376	1942.098	4.87	0.00316	12.78673
P01024	0	0.9474	803.0861	2407.244	1.19	0.00095	77.91226
P01024	0	1	885.4446	3538.757	-1.31	-0.00116	17.65957
P01024	1	1	375.7207	1499.861	2.4	0.0009	30.85749
P01024	1	0.9722	740.4	1479.793	2.27	0.00168	10.09031
P01024	0	0.8913	532.8399	1064.673	1.79	0.00095	24.00579
P01024	0	0.6875	552.3311	1103.655	0.68	0.00037	0
P01024	1	1	493.935	1479.79	0.71	0.00035	14.55597
P01024	0	0.7755	573.8312	1146.655	5.59	0.00321	35.92996
P01024	0	0.6279	588.8482	1176.689	2.09	0.00123	6.332741
P01024	0	0.8049	547.3478	1093.688	2.25	0.00123	34.99136
P01024; O9	0	0.7234	532.3346	1063.662	1.68	0.0009	5.901869
P01024	0	1	465.5803	1394.726	0.73	0.00034	70.46812
P01024	0	1	714.3776	1427.748	2.14	0.00153	6.180002
P01024	0	0.6829	722.8483	1444.689	-0.35	-0.00025	0
P01024	0	0.9545	604.6194	1811.844	2.51	0.00151	39.38744
P01024	0	1	470.9125	1410.723	2.07	0.00097	31.41291
P01024	1	1	615.9874	1845.948	3.79	0.00233	9.956991
P01024	0	0.5	382.9011	1146.689	1.29	0.00049	13.73068
P01024	0	0.3148	573.8496	1146.692	3.91	0.00224	5.263508
P01024	1	1	594.017	1780.036	0.22	0.00013	0.140251
P01024	0	1	452.5899	1355.755	0.04	0.00002	18.00065
P01024	0	0.625	595.3309	1783.978	2.14	0.00127	24.50599
P01024	0	1	625.8164	1250.626	1.9	0.00119	0
P01024	0	0.8182	817.4566	1633.906	1.22	0.001	0
P01024	0	0.7778	588.8489	1176.691	3.34	0.00196	31.07891
P01024	0	0.65	583.8093	1166.611	2.99	0.00174	5.231005
P01024	0	1	693.3605	2078.067	-0.35	-0.00024	12.40254
P01024	1	0.1053	439.2823	1315.832	1.7	0.00075	36.19715
P01024	0	0.6667	504.9395	1512.804	1.82	0.00092	6.677652
P01024	0	0.9	676.8573	1352.707	2.4	0.00163	0
P01024	0	1	738.6732	2214.005	0.21	0.00016	0
P01024	0	0.7333	392.2481	1174.73	1.05	0.00041	9.140336
P01024	0	0.7368	431.588	1292.749	3.87	0.00167	20.75518
P01024	0	1	450.9038	1350.697	3.26	0.00147	44.20295
P01024	1	0.9062	707.0376	2119.098	3.03	0.00214	26.8794
P01024	0	0.7812	392.2481	1174.73	0.9	0.00035	5.281031
P01024	0	1	545.5762	2179.283	1.38	0.00075	18.94294
P01024	0	0.75	476.5867	1427.745	0.38	0.00018	15.49583
P01024	0	0.6061	690.9211	1380.835	0.6	0.00041	0.43442
P01024	0	0.7143	627.8698	1254.732	2.85	0.00179	28.77794
P01024	0	0.0976	565.8764	1130.746	6.04	0.00341	0

P01024	0	0.6279	588.8492	1176.691	3.75	0.00221	10.89429
P01024	0	0.775	475.9045	1425.699	0.7	0.00033	5.071131
P01024	0	0.9111	542.2837	1083.56	1.79	0.00097	14.06775
P01024	0	0.7556	546.8302	1092.653	2.6	0.00142	37.75628
P01024	1	0.8077	571.3159	2282.242	1.56	0.00089	11.17065
P01024	0	0.7959	510.304	1019.601	3.05	0.00156	24.16283
P01024	0	1	803.0869	2407.246	2.18	0.00175	45.0176
P01024	2	1	877.4714	3506.864	-0.47	-0.00041	25.37721
P01024	1	0.6957	503.9533	1509.845	2.17	0.00109	17.82155
P01024	0	0.7857	509.5507	2035.181	1.36	0.00069	24.83649
P01024	0	0.84	532.84	1064.673	1.9	0.00101	18.29566
P01024	0	0.4839	552.3323	1103.657	3	0.00166	8.732166
P01024	1	1	588.3184	2350.252	-0.9	-0.00053	52.29385
P01024	1	1	500.6247	1499.859	1.45	0.00073	17.85411
P01024	0	0.6	573.829	1146.651	1.87	0.00107	0
P01024	0	1	774.0101	2320.016	-0.26	-0.0002	0
P01024	0	0.9	849.4223	3394.667	2.38	0.00202	8.229027
P01024	0	0.6154	530.2708	1588.798	3.74	0.00198	0
P01024	0	0.6364	690.9221	1380.837	2.01	0.00139	49.46531
P01024	0	1	773.0664	2317.185	0.9	0.00069	0
P01024	1	1	528.9768	1584.916	0.96	0.00051	15.75557
P01024	0	1	773.065	2317.18	-0.92	-0.00071	14.31365
P01024	1	1	375.7208	1499.861	2.81	0.00105	8.276955
P01024	0	0.4643	1065.603	2130.2	2.13	0.00227	3.804996
P01024	0	0.7719	516.797	1032.587	2.04	0.00105	9.618594
P01024	1	0.7742	396.2483	1186.73	2.31	0.00091	3.043154
P01024	0	1	545.9609	1635.868	2.19	0.0012	7.475727
P01024	0	0.1667	500.9681	1500.89	1.36	0.00068	61.18779
P01024	0	0.8636	786.7084	2358.111	1.8	0.00142	5.683824
P01024	0	0.9	738.6745	2214.009	2.03	0.0015	0
P01024	0	0.75	573.8301	1146.653	3.68	0.00211	13.30647
P01024	0	0.6818	573.8301	1146.653	3.68	0.00211	4.15098
P01024	0	0.6364	885.4469	3538.766	1.24	0.0011	1.33282
P01024	1	1	461.6141	1382.828	3.07	0.00141	46.19294
P01024	0	0.2826	580.3693	1159.731	2.16	0.00125	5.839186
P01024; O9	0	0.6591	532.3352	1063.663	2.83	0.00151	0
P01024	0	0.8889	552.647	1655.926	1.6	0.00088	7.625573
P01024	0	1	738.6738	2214.007	1.12	0.00083	30.875
P01024	1	0.1176	571.3165	2282.244	2.52	0.00144	9.758818
P01024	0	0.7692	530.2703	1588.796	2.82	0.00149	63.37735
P01024	1	1	461.6141	1382.828	3.07	0.00141	46.19294
P01024	0	0.7045	547.3479	1093.689	2.36	0.00129	35.03806
P01024	0	0.7949	588.8486	1176.69	2.72	0.0016	15.17854
P01024	0	0.5306	377.5849	1130.74	1.19	0.00045	7.857436
P01024	0	0.6667	583.8093	1166.611	2.99	0.00174	5.105981
P01024	0	0.6341	648.8601	1296.713	4.6	0.00298	9.97804
P01024	0	1	436.6629	2179.285	2.37	0.00103	7.806824
P01024	0	0.6562	546.8296	1092.652	1.48	0.00081	26.69226
P01024	0	0.8889	654.3379	1960.999	2.42	0.00158	28.03566
P01024	0	1	803.0845	2407.239	-0.79	-0.00064	17.11488
P01024	0	0.7241	456.9049	1368.7	0.8	0.00036	45.79394
P01024	1	0.2188	390.9329	1170.784	2.38	0.00093	32.48123

P01024	0	0.8824	645.329	1933.973	3.54	0.00228	2.558857
P01024	1	0.625	761.4202	2282.246	3.35	0.00255	37.87062
P01024	0	0.7059	476.588	1427.749	3.14	0.00149	21.40702
P01024	0	0.9722	1107.507	2214.007	1.1	0.00122	0
P01024	0	1	549.0041	1644.998	4.92	0.0027	8.657015
P01024	0	0.8857	773.4728	1545.938	0.67	0.00052	18.10392
P01024	0	0.8696	510.9579	1530.859	2.54	0.0013	15.93233
P01024	0	0.9444	672.7202	2016.146	5.09	0.00342	10.68467
P01024	0	0.6531	583.8099	1166.613	4.14	0.00241	6.83206
P01024	0	0.7885	587.8677	1174.728	-0.29	-0.00017	5.546717
P01024	0	0.551	573.8508	1146.694	6.04	0.00346	0
P01024	0	1	667.8652	1334.723	0.43	0.00029	3.290244
P01024	0	0.6286	718.9289	1436.851	2.89	0.00208	7.18483
P01024; Q7	1	1	417.5987	1250.782	1.91	0.0008	11.28629
P01024	0	0.8636	880.0898	2638.255	1.76	0.00155	1.77588
P01024	0	1	642.3265	1283.646	2.2	0.00141	100
P01024	0	0.8444	542.284	1083.561	2.35	0.00127	40.16504
P01024	0	0.973	532.8395	1064.672	1.1	0.00059	59.57505
P01024	1	0.381	513.2692	1537.793	2.74	0.00141	11.07192
P01024	0	0.9375	532.8399	1064.673	1.79	0.00095	36.03547
P01024	0	0.3333	648.0373	1942.097	4.4	0.00285	37.46886
P01024	1	1	493.9353	1479.791	1.45	0.00072	17.96102
P01024	1	0.7857	559.9728	1677.904	1.78	0.001	42.28728
P01024	0	0.9444	571.3041	2852.491	-1.9	-0.00109	37.61379
P01024	0	0.5593	493.8253	986.6433	6.74	0.00333	8.27927
P01024	0	0.3478	493.8229	986.6385	1.92	0.00095	14.97456
P01024	0	0.5455	660.3183	2638.251	0.46	0.0003	0
P01024	0	0.9744	684.8541	1368.701	1.45	0.00099	22.58687
P01024	0	0.6667	404.5563	1211.654	1.01	0.00041	0
P01024	0	0.8571	648.0364	1942.095	3.08	0.002	56.59838
P01024	0	1	654.338	1960.999	2.61	0.0017	31.65231
P01024	0	0.6744	546.8297	1092.652	1.7	0.00093	10.98269
P01024	0	0.8636	545.3074	1633.908	2.22	0.00121	5.323332
P01024	0	1	545.5767	2179.285	2.16	0.00118	6.366291
P01024	0	1	499.6072	1496.807	0.5	0.00025	37.56438
P01024	0	1	964.4171	2891.237	2.2	0.00212	0
P01024	0	0.6591	573.8298	1146.652	3.25	0.00186	7.657873
P01024	1	0.8182	571.3152	2282.239	0.27	0.00016	0
P01024	0	0.65	643.3654	1928.082	2.7	0.00173	10.68926
P01024	0	1	764.4017	2291.19	6.84	0.00522	59.9624
P01024	0	0.9583	465.5806	1394.727	1.45	0.00067	0
P01024	0	1	923.9529	1846.898	1.99	0.00184	8.27962
P01024	0	0.8	431.5872	1292.747	2.03	0.00088	48.54261
P01024	1	0.4688	763.9675	1526.928	1.38	0.00105	6.767378
P01024	0	1	738.6746	2214.009	2.2	0.00162	9.524781
P01024	0	0.5588	583.8088	1166.61	2.25	0.00132	0
P01024	1	0.7917	480.7777	960.5481	0.85	0.00041	0
P01024	0	0.7368	546.8302	1092.653	2.6	0.00142	7.386324
P01024	1	1	923.4755	1845.944	1.58	0.00146	0
P01024	0	1	545.5764	2179.284	1.6	0.00087	0
P01024	0	0.8182	605.9907	1815.957	1.69	0.00102	0
P01024	1	0.6667	420.2318	1677.905	2.5	0.00105	6.231005

P01024	0	0.9048	719.702	2157.091	2.66	0.00191	17.48965
P01024; O9	0	0.7	532.3342	1063.661	0.88	0.00047	13.69359
P01024	0	1	504.9383	1512.8	-0.42	-0.00021	70.8731
P01024	0	0.9048	552.647	1655.927	1.71	0.00094	10.06548
P01024	0	0.5439	511.814	1022.621	1.79	0.00091	13.52388
P01024	0	0.7692	892.4914	1783.976	0.61	0.00054	0
P01024	0	0.6061	499.7617	998.5161	2.54	0.00127	37.72398
P01024	0	0.8846	721.8314	1442.656	2.16	0.00156	0
P01024	0	0.6275	501.7972	1002.587	1.92	0.00096	15.65265
P01024	0	0.88	893.4403	1785.873	1.22	0.00109	3.576018
P01024	1	1	461.6135	1382.826	1.75	0.0008	43.02078
P01024	0	0.8462	409.8927	1227.663	2.63	0.00107	17.33604
P01024	1	1	461.6135	1382.826	1.75	0.0008	43.02078
P01024	0	0.36	583.8642	1166.721	0.14	0.00008	6.085474
P01024	0	0.7561	588.8494	1176.692	4.17	0.00245	46.93943
P01024	0	0.9032	717.3599	1433.712	1.64	0.00118	7.014114
P01024	1	0.75	571.3176	2282.249	4.55	0.0026	46.35453
P01024	0	0.7451	516.7985	1032.59	5	0.00258	20.12809
P01024	0	0.75	404.5563	1211.654	1.01	0.00041	12.67189
P01024	0	0.7857	552.3328	1103.658	3.78	0.00208	0
P01024	0	0.4828	382.901	1146.688	0.97	0.00037	8.258221
P01024	0	0.3158	387.2485	1159.731	1.72	0.00067	8.386262
P01024	0	0.8077	552.3323	1103.657	2.89	0.00159	33.58055
P01024	1	0.8125	921.4885	2762.451	3.88	0.00357	27.81056
P01024	0	1	738.6743	2214.008	1.79	0.00132	0
P01024	0	0.9091	849.423	3394.67	3.24	0.00275	0
P01024	0	1	539.2858	1615.843	-1.98	-0.00107	26.92057
P01024	1	0.9333	483.6208	1448.848	1.66	0.0008	19.15943
P01024	0	0.64	552.3325	1103.658	3.22	0.00178	65.16335
P01024	1	0.75	707.0364	2119.095	1.38	0.00098	16.93656
P01024	0	0.5952	573.8501	1146.693	4.87	0.00279	17.21188
P01024	0	0.92	530.2697	1588.795	1.67	0.00088	26.71694
P01024	0	0.9231	539.2875	1615.848	1.08	0.00058	64.33107
P01024	0	0.7778	545.3074	1633.908	2.34	0.00127	46.42766
P01024	1	0.5517	509.6466	1526.925	-0.27	-0.00014	13.50613
P01024	0	0.7586	1179.559	2358.11	1.65	0.00195	0
P01024	0	1	606.3318	1211.656	2.61	0.00158	0
P01024	0	0.5667	546.8301	1092.653	2.49	0.00136	33.79175
P01024	1	0.8108	397.9238	1191.757	1.47	0.00058	31.14958
P01024	0	1	595.9642	1785.878	3.99	0.00237	53.43063
P01024	1	0.8108	397.9238	1191.757	1.47	0.00058	31.14958
P01024	0	0.7907	510.3038	1019.6	2.81	0.00143	40.43241
P01024	0	1	667.8677	1334.728	4.18	0.00279	9.618825
P01024	0	1	539.2864	1615.845	-0.85	-0.00046	10.45022
P01024	0	0.6538	624.684	1872.037	1.9	0.00118	70.01386
P01024	0	0.7292	588.8472	1176.687	0.33	0.00019	5.46774
P01024	0	0.375	885.4479	3538.77	2.41	0.00214	12.32391
P01024	0	1	648.0393	1942.103	7.42	0.0048	46.0386
P01024	0	0.5	648.0366	1942.095	3.37	0.00218	45.49703
P01024	0	0.7297	515.8174	1030.628	1.1	0.00057	10.45339
P01024	1	0.9091	586.8334	2344.312	2.98	0.00174	29.16484
P01024	0	0.9762	603.7993	1206.591	0.71	0.00043	0

P01024	0	0.9091	565.9467	1695.826	0.33	0.00019	12.09396
P01024	0	1	545.9604	1635.867	1.3	0.00071	28.86658
P01024	0	0.6818	648.6968	1944.076	2.34	0.00152	6.403956
P01024	0	1	595.9636	1785.876	2.86	0.0017	11.40218
P01024	1	0.8333	1015.172	3043.502	2.31	0.00235	0
P01024	0	1	721.8312	1442.655	1.82	0.00132	0
P01024	1	1	591.001	1770.988	3.02	0.00178	9.871649
P01024	0	0	648.0371	1942.097	4.03	0.00261	25.08841
P01024	0	1	667.8669	1334.726	2.99	0.00199	33.37525
P01024	0	0.8182	828.4662	1655.925	0.83	0.00069	0
P01024	0	1	625.8154	1250.624	0.34	0.00021	0
P01024	0	0.7692	484.793	968.5787	3.04	0.00147	0
P01024	0	0.7353	583.8091	1166.611	2.67	0.00156	25.27555
P01024	1	0.6579	696.4278	1391.848	0.96	0.00067	10.75634
P01024	0	0.3333	438.2441	1312.718	0.28	0.00012	34.31989
P01024	0	1	785.4064	1569.805	3.57	0.0028	0
P01024	0	0.6154	697.0513	2089.139	1.93	0.00134	7.758032
P01024	0	0.8261	817.4573	1633.907	2.12	0.00173	2.249765
P01024	2		477.517	1907.046	1.4	0.00067	47.09988
P01024	0	0.48	476.5878	1427.749	2.69	0.00128	34.0836
P01024	2		477.517	1907.046	1.4	0.00067	47.09988
P01024	1	1	559.9732	1677.905	2.44	0.00136	42.87915
P01024	0	0.6486	588.8486	1176.69	2.82	0.00166	35.02979
P01024	0	1	642.3266	1283.646	2.39	0.00154	9.54295
P01024	0	1	738.6741	2214.008	1.5	0.00111	
P01024	0	0.6774	627.8698	1254.732	2.95	0.00185	5.324896
P01024	0	0.8286	532.8407	1064.674	3.28	0.00175	59.54812
P01024	1	0.875	503.9526	1509.843	0.9	0.00045	44.28659
P01024	0	0.6923	510.3031	1019.599	1.32	0.00067	48.52712
P01024	0	0.9697	773.4727	1545.938	0.51	0.0004	25.30762
P01024	0	1	1319.63	2638.253	1.16	0.00154	0
P01024	0	0.7391	430.9224	1290.753	1.4	0.0006	12.97479
P01024	0		377.5855	1130.742	2.73	0.00103	22.03847
P01024	0	0.4118	523.0403	2089.139	1.86	0.00097	0
P01024	0	1	764.4038	2291.197	9.55	0.0073	3.894335
P01024	1	0.96	420.2315	1677.904	1.77	0.00074	22.23221
P01024	0	0.1351	583.866	1166.725	3.28	0.00192	15.43483
P01024	0	0.6857	510.3047	1019.602	4.55	0.00232	22.23052
P01024	0	0.75	573.8297	1146.652	2.93	0.00168	0
P01024	1	1	511.9389	1533.802	2.12	0.00108	28.30771
P01024	1	0.7143	657.9472	1314.887	2.91	0.00191	8.861692
P01024	1	1	511.9389	1533.802	2.12	0.00108	28.30771
P01024	0	1	510.9577	1530.859	2.19	0.00111	35.90475
P01024	1	0.8889	761.6302	3043.499	1.43	0.00109	9.393605
P01024	0	0.92	605.9892	1815.953	-0.73	-0.00044	65.62952
P01024	0	0.3659	493.8229	986.6386	1.98	0.00098	43.50087
P01024	0	0.963	510.9578	1530.859	2.3	0.00118	34.0215
P01024	0	1	708.5582	3538.762	0.1	0.00007	2.903505
P01024	0	0.913	517.9133	1551.725	1.47	0.00076	4.754052
P01024	0	0.9091	614.3358	1227.664	3.36	0.00206	9.028971
P01024	0	0.5882	587.8706	1174.734	4.6	0.0027	55.9561
P01024	0	0.6842	519.8005	2076.18	3.32	0.00173	23.49956

P01024	0	0.1364	662.7061	1986.104	5.39	0.00357	59.12737
P01024	0	0.5789	418.9145	1254.729	0.29	0.00012	55.80648
P01024	0	0.6512	511.8141	1022.621	1.97	0.00101	24.91138
P01024	0	1	923.9516	1846.896	0.61	0.00056	6.842917
P01024	0	0.9231	627.8695	1254.732	2.46	0.00155	18.4508
P01024	0	0.9412	605.9926	1815.963	4.92	0.00298	12.95977
P01024	0	0.6087	504.9394	1512.804	1.76	0.00089	10.91038
P01024	0	0.975	603.8007	1206.594	3.04	0.00183	40.90488
P01024	1	1	1015.172	3043.501	1.95	0.00198	0
P01024	0	1	808.4291	1615.851	2.93	0.00237	5.879544
P01024	0	0.7097	678.3828	1355.758	2.24	0.00152	0
P01024	0	0.8	591.8637	1182.72	3.62	0.00214	11.66604
P01024	1	1	740.3993	1479.791	1.36	0.001	0
P01024	1	0.6111	571.3167	2282.245	2.95	0.00168	6.78936
P01024	0	0.8222	546.8323	1092.657	6.51	0.00356	23.04784
P01024	1	1	511.9396	1533.804	3.43	0.00175	54.79261
P01024	1	1	511.9396	1533.804	3.43	0.00175	54.79261
P01024	0	0.2727	614.3093	2454.215	3.47	0.00213	71.10449
P01024	0	0.7727	545.3074	1633.908	2.22	0.00121	4.673029
P01024	0	0.5	382.9013	1146.689	1.69	0.00064	20.9894
P01024	0	0	648.0358	1942.093	2.14	0.00139	15.02305
P01024	0	0.7419	476.5877	1427.749	2.56	0.00122	35.07749
P01024	0	0.2	482.2353	1444.691	1	0.00048	0
P01024	1	0.8824	483.6196	1448.844	-0.87	-0.00042	70.98856
P01024	0	0.6471	599.3267	1795.965	2.21	0.00132	7.656238
P01024	0	0.6944	510.304	1019.601	3.05	0.00156	47.45063
P01024	0	0.7895	1005.524	2010.041	-0.1	-0.0001	31.16234
P01024	0	1	756.904	1512.801	-0.23	-0.00017	0
P01024	0	1	738.6748	2214.01	2.45	0.00181	0
P01024	1	0.5385	513.2687	1537.791	1.67	0.00086	35.55772
P01024	0	1	517.9134	1551.726	1.71	0.00088	0
P01024	0	0.7778	627.8707	1254.734	4.31	0.00271	0
P01024	0	1	576.0538	2301.193	2.43	0.0014	3.697288
P01024	0	1	675.8515	1350.696	2.34	0.00158	0
P01024	1	1	750.4333	1499.859	1.36	0.00102	5.85879
P01024	0	0.8696	1005.526	2010.045	2.09	0.0021	6.91111
P01024	0	1	565.949	1695.833	4.44	0.00251	25.1817
P01024	0	0.4091	382.9016	1146.69	2.56	0.00098	0
P01024	0	1	738.6745	2214.009	2.03	0.0015	7.746558
P01024	0	0.7742	627.8712	1254.735	5.19	0.00325	9.546235
P01024	0	0.8378	510.3045	1019.602	4.19	0.00214	37.3288
P01024	0	0.8889	757.9358	1514.864	2.65	0.00201	6.703103
P01024	0	1	885.4495	3538.776	4.21	0.00372	38.04567
P01024	0	1	667.8658	1334.724	1.34	0.0009	42.41177
P01024	0	1	565.9482	1695.83	3.03	0.00171	50.1368
P01024	0	0.75	476.5885	1427.751	4.29	0.00204	17.43116
P01024	0	0.3529	389.5414	1166.61	1.56	0.00061	25.66426
P01024	0	1	803.0871	2407.247	2.48	0.00199	91.35889
P01024	0	0.6857	484.7923	968.5773	1.53	0.00074	0
P01024	0	0.3889	648.6958	1944.073	0.74	0.00048	8.421431
P01024	1	1	740.3995	1479.792	1.69	0.00125	1.892336
P01024	0	0.9	685.8709	1370.735	2.26	0.00155	0

P01024	2		379.2481	1513.97	2.34	0.00089	6.425385
P01024	2		379.2481	1513.97	2.34	0.00089	6.425385
P01024	0	1	880.0888	2638.252	0.65	0.00057	38.10561
P01024	1	0.65	571.317	2282.246	3.48	0.00199	5.637302
P01024	0	0.7778	475.9046	1425.699	1.08	0.00052	4.100976
P01024	1	0.6154	761.4181	2282.24	0.63	0.00048	7.989108
P01024	0	0.68	547.3478	1093.688	2.14	0.00117	6.111754
P01024	0	1	645.3283	1933.97	2.41	0.00155	8.058928
P01024	0	1	624.6843	1872.038	2.49	0.00155	20.54414
P01024	0	0.7838	499.7615	998.5157	2.11	0.00105	4.442496
P01024	1	0.375	483.6207	1448.847	1.41	0.00068	50.56187
P01024	0	1	738.6741	2214.008	1.46	0.00107	16.46152
P01024	0	0.5	504.9392	1512.803	1.22	0.00061	76.93637
P01024	0	0.6667	1038.592	2076.177	1.99	0.00206	0
P01024	0	0.6471	773.0701	2317.196	5.64	0.00436	18.98074
P01024	0	0.7895	533.3093	2130.215	9.51	0.00507	40.89769
P01024	0	1	604.6192	1811.843	2.2	0.00133	13.40726
P01024	0	0.3	387.2478	1159.729	-0.01	0	15.91447
P01024	0	0.4	648.0372	1942.097	4.21	0.00273	10.10804
P01024	1	0.0857	767.4038	1533.8	0.93	0.00072	0
P01024	1	0.2258	390.933	1170.784	2.62	0.00102	35.96939
P01024	1	0.913	438.9665	1314.885	1.16	0.00051	48.83235
P01024	0	0.8095	505.626	1514.863	1.97	0.001	53.23386
P01024	0	1	595.8145	1190.622	1.11	0.00066	3.525381
P01024	1	0.75	921.4867	2762.446	1.96	0.0018	44.47349
P01024	0	0.8333	424.7119	1695.826	0.4	0.00017	78.40486
P01024	0	0.7	546.8297	1092.652	1.59	0.00087	6.653565
P01024; O9	0	0.7333	532.3348	1063.662	2.14	0.00114	6.09545
P01024	0	0.1	500.9684	1500.891	2.15	0.00108	0
P01024	0	0.4857	510.3042	1019.601	3.53	0.0018	43.21545
P01024	0	0	479.6219	1436.851	3.24	0.00155	4.861784
P01024	0	0.7143	605.9917	1815.961	3.4	0.00206	13.00969
P01024	0	0.4138	583.8095	1166.612	3.3	0.00193	6.351749
P01024	1	1	745.407	2234.207	3.5	0.00261	6.842923
P01024	0	0.9231	685.8706	1370.734	1.82	0.00125	17.04296
P01024	0	0.9444	530.2687	1588.791	-0.29	-0.00015	39.2995
P01024	0	0.7059	603.7993	1206.591	0.71	0.00043	32.7053
P01024	0	1	676.8594	1352.711	5.47	0.0037	22.19567
P04114	0	0.9254	562.3297	1684.974	1.78	0.001	7.678353
P04114	0	0.9091	671.8355	1342.664	1.54	0.00103	3.294633
P04114	0	1	888.4462	1775.885	1.52	0.00135	0
P04114	0	1	489.6132	1466.825	0.37	0.00018	66.47633
P04114	0	0.6379	562.3297	1684.975	1.89	0.00106	6.588665
P04114	0	1	475.6133	1424.825	1.78	0.00084	22.38718
P04114	0	0.9091	712.9164	1424.825	1.79	0.00127	17.83965
P04114	0	0.9535	475.6125	1424.823	0.04	0.00002	4.203761
P04114	0	0.931	712.9156	1424.824	0.67	0.00048	3.233114
P04114	0	0.9556	475.6128	1424.824	0.69	0.00033	0
P04114	0	0.8909	589.82	1178.633	3.51	0.00207	2.316912
P04114	0	1	668.3922	1335.777	0.7	0.00047	6.438231
P04114	0	0.9839	661.8252	1322.643	1.66	0.0011	8.574846
P04114	0	1	489.5864	1466.745	1.07	0.00052	3.751423

P04114	0	0.9833	733.8754	1466.744	0.41	0.0003	3.531101
P04114	0	0.95	733.8756	1466.744	0.66	0.00048	9.95089
P04114	0	0.9744	719.0294	2155.074	0.15	0.00011	0
P04114	0	1	719.0317	2155.081	3.46	0.00249	39.48687
P04114	0	0.8913	648.3423	1295.677	4.71	0.00305	5.423463
P04114	0	0.9688	494.9185	1482.741	1.97	0.00098	11.24194
P04114	0	0.9608	498.2756	1990.08	2.82	0.0014	0
P04114	0	0.9123	656.3834	1311.759	3.33	0.00218	9.090432
P04114	0	0.8478	437.2508	1309.738	1.84	0.00081	8.858871
P04114	0	1	655.3726	1309.738	1.85	0.00121	6.296742
P04114	0	0.9857	498.2746	1990.076	0.79	0.0004	7.190231
P04114	0	0.9861	664.032	1990.082	3.38	0.00224	11.43456
P04114	0	0.9	429.2437	1285.717	2.46	0.00105	8.121521
P04114	0	0.7963	606.8307	1212.654	3.15	0.00191	16.50888
P04114	0	0.6	765.9476	1530.888	1.34	0.00103	3.660219
P04114	0	0.6875	525.6519	1574.941	3.57	0.00188	12.50804
P04114	0	0.9796	434.2431	1300.715	1.42	0.00062	9.703751
P04114	0	0.875	765.9256	1530.844	2.17	0.00166	8.095461
P04114	0	0.9464	510.9532	1530.845	2.81	0.00144	8.287645
P04114	0	0.7292	652.3796	1303.752	1.54	0.001	58.58208
P04114	0	1	452.8837	1356.637	2.36	0.00107	52.1926
P04114	1	1	431.4948	1722.957	2.52	0.00109	36.84549
P04114	0	0.75	678.8218	1356.636	2.19	0.00149	21.04242
P04114	1	1	857.4137	2570.227	0.64	0.00055	5.86468
P04114	0	1	484.2579	1450.759	2.29	0.00111	21.3595
P04114	0	0.9167	380.5767	1139.716	1.51	0.00057	3.633371
P04114	0	0.9268	752.8569	1504.706	1.9	0.00143	0
P04114	0	1	469.919	1407.743	2.61	0.00123	39.37512
P04114	0	0.9714	651.3749	1952.11	3.58	0.00233	13.91869
P04114	0	1	488.782	1952.106	1.5	0.00073	10.98878
P04114	0	0.9643	725.8831	1450.759	2.09	0.00152	2.580118
P04114	0	1	484.2572	1450.757	0.78	0.00038	5.257349
P04114	0	1	484.2577	1450.758	1.73	0.00083	32.02574
P04114	0	1	463.6114	1388.82	4.11	0.0019	64.51563
P04114	0	0.5818	581.3535	1161.7	3.17	0.00184	12.29018
P04114	0	1	484.2573	1450.757	0.9	0.00044	36.95274
P04114	1	0.907	809.3797	2426.125	0.71	0.00057	3.396551
P04114	1	0.907	809.3797	2426.125	0.71	0.00057	3.396551
P04114	0	0.963	389.9592	1556.815	3.89	0.00152	33.56322
P04114	0	0.8889	381.8849	1143.64	0.88	0.00034	4.497948
P04114	0	0.8286	381.8852	1143.641	1.76	0.00067	27.46516
P04114	0	0.8923	633.007	1897.006	4.43	0.0028	8.623389
P04114	0	0.878	633.0074	1897.008	5.11	0.00323	3.617055
P04114	0	0.9608	806.0969	2416.276	2.69	0.00217	8.592494
P04114	0	0.8077	694.8818	1388.756	1.59	0.0011	10.83513
P04114	0	1	389.9584	1556.812	1.7	0.00066	11.19557
P04114	0	1	519.609	1556.812	2.22	0.00115	44.70229
P04114	2	0.987	706.3796	2822.497	2.53	0.00178	5.943409
P04114	2	1	565.3049	2822.495	2	0.00113	7.988802
P04114	2	0.9821	706.379	2822.494	1.66	0.00117	6.002202
P04114	2	0.9722	941.5031	2822.495	1.84	0.00173	7.3026
P04114	0	0.9659	680.3347	2038.99	2.37	0.00161	5.095828

P04114	0	0.913	616.3399	1847.005	2.78	0.00171	0
P04114	0	0.9647	1019.998	2038.99	2.41	0.00245	5.225675
P04114	0	0.8861	680.333	2038.984	-0.23	-0.00016	11.99382
P04114	0	0.8713	924.0053	1847.003	1.73	0.0016	6.383188
P04114	0	0.875	616.3391	1847.003	1.4	0.00086	8.113494
P04114	0	0.9444	616.3388	1847.002	1	0.00062	0
P04114	1	0.8519	889.4671	2666.387	-0.65	-0.00058	6.4054
P04114	1	0.871	889.4705	2666.397	3.13	0.00278	0
P04114	1	0.8824	889.4724	2666.403	5.26	0.00467	3.917206
P04114	1	0.8571	667.3539	2666.394	1.95	0.0013	10.02872
P04114	1	0.9545	667.3539	2666.394	1.95	0.0013	4.581666
P04114	1	0.9821	889.4702	2666.396	2.78	0.00247	7.462214
P04114	1	1	667.3541	2666.394	2.23	0.00149	5.646883
P04114	1	0.9375	667.3561	2666.402	5.25	0.0035	2.791625
P04114	1	1	889.4688	2666.392	1.27	0.00113	5.8158
P04114	1	1	667.3533	2666.391	1.04	0.00069	5.912683
P04114	0	0.8478	747.4257	1493.844	-0.8	-0.0006	12.95047
P04114	0	1	712.6957	2136.072	1.21	0.00086	12.31516
P04114	0	1	596.3419	1191.677	2.02	0.00121	0
P04114	0	0.9487	639.9927	1917.963	6.7	0.00428	19.18038
P04114	0	1	631.0175	1891.038	3.39	0.00213	76.19498
P04114	0	0.8958	673.3828	1345.758	2.05	0.00138	4.090156
P04114	0	1	449.2577	1345.759	2.24	0.00101	30.16427
P04114	0	0.7143	435.9387	1305.802	2.75	0.0012	8.807224
P04114	0	0.8454	653.4045	1305.802	2.91	0.0019	29.94927
P04114	0	0.6809	435.9389	1305.802	3.25	0.00141	15.37336
P04114	0	0.9574	550.3106	1648.917	3.92	0.00215	31.4659
P04114	0	0.6056	653.4054	1305.804	4.21	0.00275	48.72275
P04114	0	0.8704	587.3279	1759.969	3.79	0.00222	20.13526
P04114	0	1	440.7473	1759.968	2.92	0.00128	44.98894
P04114	0	1	624.0187	1870.042	5.56	0.00347	10.91581
P04114	0	0.8462	424.5693	1271.693	1.97	0.00083	10.99942
P04114	0	0.8333	636.3508	1271.694	2.84	0.00181	6.333439
P04114	0	0.871	598.6691	1793.993	4.43	0.00265	17.94276
P04114	0	1	544.9708	1632.898	2.52	0.00137	46.553
P04114	0	0.8679	713.8829	1426.759	1.2	0.00086	5.914031
P04114	0	0.9091	408.98	1632.898	2.55	0.00104	6.54188
P04114	0	0.8667	386.2091	1156.613	1.43	0.00055	2.983732
P04114	0	0.7222	386.2089	1156.612	1.03	0.0004	0
P04114	1	1	496.6307	1487.877	3.07	0.00152	27.34733
P04114	0	0.9873	760.3953	2279.171	2.95	0.00224	4.200677
P04114	0	0.9851	610.3635	1829.076	1.3	0.00079	7.89661
P04114	0	1	436.2236	1306.656	1.76	0.00077	8.513081
P04114	0	0.9583	458.0248	1829.078	2.23	0.00102	6.325736
P04114	1	1	504.044	2013.154	0.81	0.00041	13.48341
P04114	0	1	619.8549	1238.703	3.44	0.00213	0
P04114	0	1	425.4966	1698.964	4.36	0.00185	15.14044
P04114	0	1	458.0247	1829.077	1.9	0.00087	29.18206
P04114	1	0.8689	809.4819	1617.957	2.46	0.00199	6.531079
P04114	1	0.9595	539.9903	1617.956	2.3	0.00124	9.001056
P04114	1	0.9831	539.9904	1617.957	2.41	0.0013	0
P04114	0	1	610.3646	1829.079	3.2	0.00195	14.39931

P04114	1	0.8333	379.7365	1515.924	1.63	0.00062	8.929523
P04114	0	1	566.9913	1698.959	1.46	0.00082	9.002501
P04114	0	1	566.9924	1698.963	3.29	0.00186	12.35811
P04114	0	1	610.3641	1829.078	2.4	0.00147	2.496487
P04114	0	0.9846	881.001	1760.995	1.89	0.00167	33.71863
P04114	0	0.9375	643.3617	1285.716	2.16	0.00139	3.883469
P04114	0	1	429.2428	1285.714	0.47	0.0002	5.591251
P04114	0	1	429.2422	1285.712	-1.1	-0.00047	4.216781
P04114	0	1	389.9582	1556.811	1.15	0.00045	5.762279
P04114	0	1	519.6086	1556.811	1.39	0.00072	21.18047
P04114	0	0.7895	389.9584	1556.812	1.86	0.00072	30.3497
P04114	0	0.931	563.3434	1688.016	4.27	0.0024	12.26031
P04114	0	1	519.6086	1556.811	1.39	0.00072	24.63093
P04114	0	0.8205	562.3298	1684.975	2.11	0.00118	33.1318
P04114	0	0.9412	509.6253	1526.861	2.85	0.00145	8.345437
P04114	0	1	489.614	1466.827	1.93	0.00095	27.09693
P04114	0	0.9804	389.9584	1556.812	1.86	0.00072	7.009489
P04114	0	1	519.6083	1556.81	0.81	0.00042	21.66059
P04114	0	1	389.9585	1556.812	1.93	0.00075	5.231515
P04114	1	1	778.0864	2332.245	1.07	0.00083	12.5846
P04114	1	1	727.0456	2179.122	1.76	0.00128	8.178112
P04114	0	0.9483	408.9796	1632.897	1.8	0.00073	2.958793
P04114	1	1	545.5361	2179.123	1.97	0.00108	48.91193
P04114	0	0.9455	408.9798	1632.897	2.1	0.00086	8.520015
P04114	0	0.8769	544.9705	1632.897	1.96	0.00106	4.175641
P04114	0	0.8939	408.9798	1632.897	2.25	0.00092	2.026433
P04114	0	0.9828	408.9801	1632.899	2.92	0.00119	0
P04114	0	1	713.6856	2139.042	2.11	0.0015	3.288271
P04114	0	1	652.3825	1955.133	1.27	0.00083	8.768548
P04114	0	1	652.3817	1955.131	0.15	0.00009	7.621233
P04114	0	1	978.0712	1955.135	2.49	0.00244	5.949591
P04114	1	1	727.0458	2179.123	2.09	0.00152	5.67016
P04114	0	0.9296	408.98	1632.898	2.77	0.00113	0
P04114	1	1	545.5359	2179.122	1.53	0.00083	14.62965
P04114	0	0.8043	517.9525	1551.843	1.32	0.00068	14.2568
P04114	0	1	548.3088	1642.912	2.52	0.00138	43.39756
P04114	0	0.9429	548.3082	1642.91	1.4	0.00077	19.71157
P04114	0	0.8519	517.9527	1551.844	1.68	0.00087	10.19203
P04114	0	0.9762	776.4258	1551.844	2.16	0.00167	5.909917
P04114	0	0.775	517.9534	1551.846	3.09	0.0016	23.72988
P04114	0	0.8101	653.4041	1305.801	2.16	0.00141	38.96466
P04114	1	0.6667	494.0317	1973.105	6.39	0.00315	46.87059
P04114	0	0.96	726.8959	1452.785	-0.62	-0.00045	16.091
P04114	0	1	885.4732	2654.405	0.86	0.00076	33.80442
P04114	1	1	431.494	1722.954	0.75	0.00032	13.90142
P04114	1	0.8636	483.5366	1931.125	3.59	0.00173	36.22338
P04114	0	1	484.2576	1450.758	1.6	0.00077	27.59557
P04114	0	1	484.2579	1450.759	2.23	0.00108	3.419087
P04114	0	0.8478	381.8851	1143.641	1.44	0.00055	5.363652
P04114	0	0.8654	381.8853	1143.641	2	0.00076	4.556586
P04114	0	0.9565	945.5362	1890.065	4.04	0.00382	6.96443
P04114	0	0.9403	630.692	1890.062	2.13	0.00134	10.12971

P04114	0	0.9623	566.3161	1696.934	2.16	0.00122	8.412881
P04114	0	0.9153	671.3865	2012.145	1.49	0.001	3.607776
P04114	0	0.9375	778.4688	1555.93	0.91	0.00071	3.638077
P04114	0	0.8824	778.4694	1555.932	1.78	0.00138	7.476841
P04114	0	0.8254	519.3152	1555.931	1.41	0.00073	5.91614
P04114	0	0.9655	671.3854	2012.142	-0.15	-0.0001	4.702208
P04114	0	0.9167	1006.577	2012.147	2.5	0.00251	0
P04114	0	0.9437	720.7217	2160.15	2.06	0.00149	10.2127
P04114	0	0.9077	720.7244	2160.159	5.79	0.00417	11.03343
P04114	0	0.8413	595.3588	1189.71	3.47	0.00207	22.21321
P04114	0	0.4318	655.0149	1963.03	3.59	0.00235	32.08146
P04114	0	0.7179	533.3072	1597.907	1.93	0.00103	38.52685
P04114	0	0.8448	813.963	1626.919	3.63	0.00295	13.15825
P04114	0	0.8793	542.9766	1626.915	1.48	0.0008	8.905892
P04114	0	0.8684	813.9615	1626.916	1.83	0.00149	5.554574
P04114	0	0.963	542.9769	1626.916	2.04	0.00111	16.62893
P04114	0	0.9535	780.4491	1559.891	1.69	0.00132	4.984789
P04114	0	0.94	520.6346	1559.889	0.58	0.0003	6.763074
P04114	0	0.7436	533.3071	1597.907	1.7	0.00091	8.366426
P04114	0	0.9545	780.4496	1559.892	2.31	0.0018	6.224965
P04114	0	0.8983	520.6354	1559.892	2.23	0.00116	10.14761
P04114	0	0.8592	533.307	1597.906	1.47	0.00078	19.34507
P04114	0	0.973	510.9523	1530.842	1.08	0.00055	18.78366
P04114	0	0.9149	681.9167	1362.826	1.91	0.0013	36.35106
P04114	0	0.2051	731.7229	2193.154	2.01	0.00147	4.467676
P04114	0	0.9167	681.917	1362.827	2.35	0.0016	6.684518
P04114	0	1	864.45	2591.335	2.21	0.0019	15.50795
P04114	0	1	640.6694	1919.994	4.86	0.00311	23.15222
P04114	0	0.9885	960.4981	1919.989	2.35	0.00226	3.749805
P04114	0	1	636.3137	1906.927	2.74	0.00174	9.12881
P04114	0	0.92	953.9672	1906.927	2.98	0.00284	6.30678
P04114	0	0.9194	640.6671	1919.987	1.14	0.00073	5.123522
P04114	0	0.9804	636.3136	1906.926	2.55	0.00162	4.215791
P04114	0	0.9412	509.625	1526.86	2.31	0.00117	22.15171
P04114	0	1	733.9171	1466.827	1.63	0.0012	10.91737
P04114	0	0.8103	434.2433	1300.715	1.7	0.00074	0
P04114	0	0.9841	434.2428	1300.714	0.58	0.00025	4.837309
P04114	0	0.8525	650.8615	1300.716	2.11	0.00137	5.013338
P04114	0	0.9841	434.2434	1300.716	2.13	0.00092	2.068902
P04114	0	0.8125	652.3801	1303.753	2.28	0.00149	22.2399
P04114	0	0.8197	636.3507	1271.694	2.65	0.00168	13.77838
P04114	0	0.8163	553.3215	1105.636	2.1	0.00116	100
P04114	0	0.7213	592.3464	1775.025	4.48	0.00265	21.15675
P04114	0	0.9231	444.5111	1775.022	3.24	0.00144	14.91415
P04114	0	0.6383	553.3217	1105.636	2.54	0.0014	10.48274
P04114	0	0.9524	444.5114	1775.024	3.93	0.00174	35.04399
P04114	0	1	436.2229	1306.654	0.22	0.0001	24.99941
P04114	0	0.9718	444.5109	1775.022	2.96	0.00131	24.08275
P04114	0	1	509.6255	1526.862	3.33	0.00169	37.81144
P04114	0	0.8261	489.6133	1466.825	0.43	0.00021	39.5967
P04114	0	0.9804	408.9789	1632.894	-0.07	-0.00003	50.33401
P04114	0	0.9556	408.9794	1632.896	1.12	0.00046	0

P04114	0	0.5676	567.6376	1700.898	1.76	0.001	0
P04114	0	0.973	552.2963	1103.585	3.57	0.00197	0
P04114	0	0.9231	548.3088	1642.912	2.63	0.00144	23.13322
P04114	0	0.6	616.8793	1232.751	4.98	0.00307	13.23632
P04114	0	0.7347	434.2436	1300.716	2.55	0.00111	0
P04114	0	0.6452	548.3094	1642.914	3.63	0.00199	47.60517
P04114	0	1	544.9698	1632.895	0.61	0.00033	29.77523
P04114	0	0.9556	1018.062	2035.116	2.9	0.00295	1.110583
P04114	0	0.8654	679.0435	2035.116	2.74	0.00186	4.767134
P04114	0	0.9672	978.0712	1955.135	2.49	0.00244	7.043578
P04114	0	1	652.3825	1955.133	1.27	0.00083	7.669722
P04114	0	0.6667	739.7089	2217.112	2.53	0.00187	6.887114
P04114	0	1	990.186	2968.543	1.68	0.00166	1.265898
P04114	0	1	885.4752	2654.411	3.06	0.00271	10.83279
P04114	0	0.8947	766.9329	1532.858	1.43	0.0011	21.28372
P04114	0	0.8767	766.9331	1532.859	1.75	0.00134	100
P04114	0	0.8846	766.9333	1532.859	1.99	0.00152	2.278606
P04114	1	0.9286	661.3771	2642.487	2.34	0.00154	24.58482
P04114	0	0.9753	726.3913	2177.159	2.07	0.0015	4.326257
P04114	1	0.931	881.4991	2642.483	0.82	0.00072	38.10486
P04114	1	1	881.4999	2642.485	1.79	0.00158	5.298659
P04114	1	0.9231	661.3764	2642.484	1.23	0.00081	4.992674
P04114	0	0.8873	766.9341	1532.861	3.02	0.00232	10.58554
P04114	0	0.8235	511.6248	1532.86	2.32	0.00118	9.111108
P04114	0	1	990.1854	2968.542	1.06	0.00105	3.258094
P04114	1	0.9615	688.1147	2749.437	-1.09	-0.00075	21.76972
P04114	0	0.9878	664.3563	2654.403	0.16	0.00011	17.53648
P04114	0	1	946.0019	1890.997	1.9	0.00179	0.81828
P04114	0	0.9872	631.004	1890.997	2.34	0.00148	2.842151
P04114	0	1	1018.053	2035.1	2.24	0.00228	0
P04114	0	0.7755	653.3458	1958.023	3.98	0.0026	7.281023
P04114	0	1	664.358	2654.41	2.83	0.00188	5.125243
P04114	0	1	990.1862	2968.544	1.86	0.00184	3.054319
P04114	0	0.8696	739.7086	2217.111	2.12	0.00156	6.029101
P04114	0	1	990.1868	2968.546	2.48	0.00245	2.412564
P04114	0	0.9394	679.0427	2035.114	1.66	0.00113	6.690235
P04114	0	0.961	739.7089	2217.112	2.53	0.00187	6.297403
P04114	0	1	885.4745	2654.409	2.3	0.00204	6.865523
P04114	0	1	781.1198	2341.345	3.39	0.00265	10.12054
P04114	0	1	798.9486	1596.89	1.42	0.00113	7.018042
P04114	0	0.9	532.9681	1596.89	1.45	0.00077	9.043949
P04114	0	0	679.0385	2035.101	2.86	0.00194	3.180454
P04114	0	0.7969	668.3933	1335.779	2.34	0.00157	23.95877
P04114	0	1	997.5424	1994.078	4.58	0.00456	6.783185
P04114	0	1	665.3634	1994.076	3.62	0.00241	5.099755
P04114	0	0.9825	648.0007	1941.987	4.04	0.00261	20.39226
P04114	0	1	876.468	2627.389	3.66	0.0032	4.093039
P04114	0	0.9275	609.3458	1826.023	4.37	0.00266	8.175531
P04114	0	1	649.3659	1946.083	1.8	0.00117	57.68742
P04114	0	0.9661	697.3496	2786.376	1.47	0.00102	4.691191
P04114	0	0.7619	860.0028	1718.998	3.06	0.00263	6.921578
P04114	0	0.871	573.6707	1718.997	2.52	0.00144	9.282678

P04114	0	0.8689	559.0013	1674.989	0.77	0.00043	8.829419
P04114	0	0.7624	837.9992	1674.991	1.87	0.00157	9.554386
P04114	0	0.8393	559.0024	1674.993	2.85	0.00159	8.097444
P04114	0	0.9518	973.5458	1946.084	2.43	0.00237	6.359791
P04114	0	0.8961	649.3652	1946.081	0.76	0.00049	8.948442
P04114	0		634.6584	1901.961	2.57	0.00163	9.278855
P04114	1	0	634.6584	1901.961	8.98	0.00569	9.278855
P04114	0	0.8293	559.002	1674.991	2.08	0.00116	19.47608
P04114	0	0.9333	649.3657	1946.083	1.52	0.00098	5.197229
P04114	0	0.8367	509.5968	1526.776	1.92	0.00098	5.553577
P04114	0	1	763.892	1526.777	2.53	0.00193	7.115397
P04114	0	1	818.3889	2453.152	2.49	0.00203	5.508376
P04114	0	0.9412	714.3754	1427.743	1.8	0.00128	5.961722
P04114	0	1	766.6821	2298.032	1.73	0.00132	3.830698
P04114	0	0.9605	660.8481	1320.689	1	0.00066	3.41485
P04114	1	0.75	489.6361	1466.894	2.28	0.00112	11.27147
P04114	1	1	744.4418	1487.876	2.32	0.00173	0
P04114	0	1	1171.174	2341.34	1.47	0.00172	0
P04114	1	1	496.6309	1487.878	3.62	0.0018	13.93522
P04114	0	1	781.1181	2341.34	1.2	0.00094	3.618087
P04114	1	1	448.5964	1343.775	2.98	0.00133	28.67429
P04114	1	1	448.5964	1343.775	2.98	0.00133	28.67429
P04114	1	1	531.6339	1592.887	1.62	0.00086	15.81919
P04114	0	1	924.5021	2771.492	3.64	0.00336	5.623271
P04114	0	1	693.6268	2771.485	1.26	0.00087	3.825375
P04114	0	1	779.423	2336.255	2.44	0.0019	74.60046
P04114	0	1	731.5827	2923.309	0.91	0.00067	25.40523
P04114	0	1	975.1092	2923.313	2.3	0.00224	29.14009
P04114	0	0.2333	969.7773	2907.317	2.03	0.00197	3.722632
P04114	0	0.1346	969.7779	2907.319	2.66	0.00258	4.022875
P04114	0	0.6607	624.3495	1247.692	6.67	0.00416	7.128069
P04114	0	0.9726	751.0908	2251.258	-1.46	-0.00109	16.65745
P04114	0	0.7742	679.9194	1358.831	2.12	0.00144	100
P04114	0	0.9412	521.978	1563.92	1.56	0.00082	7.950328
P04114	0	0.898	453.6159	1358.833	3.37	0.00153	11.71059
P04114	0	0.9273	785.4158	2354.233	4.13	0.00324	6.047787
P04114	0	0.7674	679.9201	1358.833	3.2	0.00217	20.12064
P04114	0	0.9574	570.6398	1709.905	2.15	0.00122	2.478134
P04114	0	0.8596	613.3511	1225.695	2.66	0.00163	4.925251
P04114	0	0.7656	613.3511	1225.695	2.56	0.00157	9.570259
P04114	1	1	531.6342	1592.888	2.2	0.00117	14.52502
P04114	0	0.975	495.601	1484.788	1.1	0.00054	19.16586
P04114	0	0.5333	438.5636	1313.676	3.34	0.00146	49.12705
P04114	1	0.9714	531.6345	1592.889	2.66	0.00141	9.830604
P04114	1	1	796.9476	1592.888	2.05	0.00163	5.304429
P04114	1	0.9571	531.6343	1592.888	2.43	0.00129	8.540796
P04114	1	0.96	531.6337	1592.886	1.16	0.00062	11.44808
P04114	0	0.962	855.4556	1709.904	1.59	0.00136	9.472838
P04114	0	1	635.7042	1905.098	2.41	0.00153	9.50951
P04114	0	1	713.6849	2139.04	1.17	0.00083	0.614829
P04114	0	0.9231	492.2772	1474.817	4.51	0.00222	13.09839
P04114	0	0.9167	635.7041	1905.098	2.22	0.00141	24.07677

P04114	0	1	713.6854	2139.042	1.85	0.00132	6.269534
P04114	0	1	408.8998	1224.685	2.52	0.00103	43.7624
P04114	0	0.7778	405.8976	1215.678	2.13	0.00086	46.19752
P04114	0	0.8649	408.8997	1224.685	2.37	0.00097	11.07607
P04114	0	0.6471	849.9897	1698.972	1.65	0.0014	3.543408
P04114	0	0.6349	849.9908	1698.974	3.02	0.00256	28.83894
P04114	0	0.6774	566.9968	1698.976	3.91	0.00221	5.67531
P04114	0	0.9756	752.8569	1504.706	1.9	0.00143	0
P04114	0	0.7705	566.9961	1698.974	2.61	0.00148	5.134623
P04114	0	0.9828	844.4316	1687.856	1.8	0.00152	6.408613
P04114	0	0.9242	690.3572	1379.707	2.64	0.00182	8.18026
P04114	0	0.9841	563.2903	1687.856	1.98	0.00111	8.797332
P04114	0	1	680.8049	1360.603	0.76	0.00052	39.95712
P04114	1	1	809.3804	2426.127	1.54	0.00125	2.680119
P04114	1	1	809.3804	2426.127	1.54	0.00125	2.680119
P04114	0	0.7297	465.2469	1393.726	4.34	0.00201	12.91025
P04114	0	0.8837	460.5734	1379.706	1.44	0.00066	29.12798
P04114	0	0.7451	678.8217	1356.636	1.92	0.0013	6.564109
P04114	0	0.8913	690.3566	1379.706	1.67	0.00115	5.964774
P04114	0	0.9762	751.0942	2251.268	3.1	0.00233	27.40767
P04114	0	0.8732	823.0147	1645.022	2.61	0.00215	8.732327
P04114	0	1	752.8572	1504.707	2.38	0.00179	0.540075
P04114	0	1	502.2406	1504.707	2.48	0.00124	13.3591
P04114	0	0.5	611.3527	1221.698	4.23	0.00258	10.08818
P04114	0	0.7727	753.0786	2257.221	-0.28	-0.00021	16.30729
P04114	0	0.8491	565.063	2257.23	3.64	0.00205	6.513497
P04114	0	0.8286	565.0628	2257.229	3.31	0.00187	10.84741
P04114	0	0.8806	753.0805	2257.227	2.24	0.00168	5.544158
P04114	0	0.9825	612.0926	2445.349	2.93	0.00179	3.301458
P04114	0	1	815.7875	2445.348	2.66	0.00217	6.388143
P04114	0	0.4634	730.8984	1460.79	1.43	0.00105	53.46321
P04114	0	0.9091	683.8521	1366.697	2.16	0.00147	6.45603
P04114	0	1	922.5156	1844.024	2.87	0.00265	6.768218
P04114	0	0.9815	456.2371	1366.697	1.99	0.00091	10.52851
P04114	0	1	615.3461	1844.024	2.8	0.00172	4.282447
P04114	0	1	903.4734	2708.406	4.54	0.0041	5.410085
P04114	0	1	870.8308	2610.478	0.63	0.00055	2.62994
P04114	0	1	774.3927	2321.164	1.03	0.00079	2.451459
P04114	0	1	774.3935	2321.166	2.05	0.00159	5.009547
P04114	1	1	857.4145	2570.229	1.57	0.00134	4.126486
P04114	1	1	643.3135	2570.232	2.8	0.0018	23.2908
P04114	0	1	774.3938	2321.167	2.45	0.00189	3.006981
P04114	0	1	924.1262	2770.364	1.67	0.00154	0
P04114	0	1	581.0476	2321.169	3.2	0.00186	68.08711
P04114	0	0.9903	814.7851	2442.341	1.53	0.00124	17.68
P04114	0	1	1243.662	3728.971	4.22	0.00525	2.97666
P04114	0	1	932.9959	3728.962	1.68	0.00157	2.676947
P04114	0	1	623.6594	1868.964	2.31	0.00144	35.53183
P04114	0	1	859.1171	2575.337	0.84	0.00072	6.398591
P04114	0	1	918.8663	2754.584	2.23	0.00205	11.35834
P04114	0	1	1377.797	2754.586	2.97	0.00409	0
P04114	0	1	689.4013	2754.583	1.76	0.00121	3.151737

P04114	0	0.973	787.7615	2361.27	1.96	0.00155	5.434047
P04114	0	0.8788	542.9771	1626.917	2.38	0.00129	8.469782
P04114	0	1	591.0743	2361.276	4.36	0.00257	9.845611
P04114	0	1	660.0316	1978.08	1.87	0.00124	1.182949
P04114	0	0.8481	796.4785	2387.421	1.07	0.00085	3.365304
P04114	0	1	526.5791	1577.723	1.15	0.0006	9.925909
P04114	0	1	789.3655	1577.724	1.81	0.00143	5.763126
P04114	0	1	526.5798	1577.725	2.54	0.00134	4.983511
P04114	0	1	678.3239	2032.957	4	0.00271	9.880599
P04114	0	0.9875	989.5439	1978.08	1.97	0.00195	0.833564
P04114	0	1	1038.602	3113.792	2.46	0.00255	0
P04114	0	1	779.2028	3113.789	1.65	0.00128	2.874561
P04114	0	0.9865	745.7618	2235.271	2.18	0.00163	17.97917
P04114	0	1	650.9267	1300.846	1.45	0.00094	25.32581
P04114	0	1	1118.138	2235.268	0.91	0.00101	5.473608
P04114	0	1	789.3654	1577.723	1.58	0.00125	32.11262
P04114	0	0.8625	982.0609	1963.114	-1.63	-0.0016	0
P04114	0	1	727.4221	2180.252	1.7	0.00124	3.574387
P04114	0	0.8	452.8833	1356.635	1.35	0.00061	4.046149
P04114	1	1	857.4147	2570.229	1.78	0.00153	5.172739
P04114	1	1	643.3137	2570.233	3.08	0.00198	5.755325
P04114	0	0.9588	1009.583	2018.159	1.54	0.00155	4.124088
P04114	0	1	1090.63	2180.252	1.91	0.00208	0
P04114	0	1	1087.244	3259.716	1.93	0.0021	2.341294
P04114	1	0.8837	459.2771	1375.817	1.54	0.0007	50.32549
P04114	0	0.9753	727.3933	2180.165	3.46	0.00252	8.947624
P04114	0	1	1090.584	2180.161	1.4	0.00153	0
P04114	0	0.8876	1194.215	2387.423	1.84	0.0022	0
P04114	0	1	401.5526	1202.643	2.95	0.00118	15.63825
P04114	0	1	935.4973	2804.477	3.22	0.00301	7.74254
P04114	0	0.878	434.2429	1300.714	0.93	0.0004	38.63764
P04114	0	1	489.5869	1466.746	2.19	0.00107	3.740947
P04114	0	1	520.6357	1559.892	2.7	0.0014	30.20678
P04114	0	0.75	668.3933	1335.779	2.34	0.00157	13.40576
P04114	0	0.6596	599.3337	1197.66	5.19	0.00311	18.97291
P04114	1	0.9688	531.6335	1592.886	0.93	0.0005	9.844166
P04114	0	1	990.1861	2968.544	1.8	0.00178	0
P04114	0	0.9091	616.8431	2464.35	4.34	0.00267	10.355
P04114	0	0.8667	408.98	1632.898	2.55	0.00104	0
P04114	1	0.7917	505.9801	1515.926	2.56	0.00129	65.27874
P04114	0	0.9756	576.2882	1726.85	7.3	0.0042	0
P04114	0	0.7907	553.322	1105.637	3.09	0.00171	13.05781
P04114	0	0.6512	765.9483	1530.889	2.22	0.0017	10.05616
P04114	0	0.9767	778.9092	1556.811	1.32	0.00103	0
P04114	0	0.92	454.947	1362.826	2.08	0.00094	11.81164
P04114	0	1	777.9386	1554.87	1.73	0.00135	2.255943
P04114	0	0.8627	553.3215	1105.636	2.1	0.00116	18.25963
P04114	0	0.9474	774.3926	2321.163	0.87	0.00067	5.593933
P04114	0	1	468.2591	1402.763	-3.77	-0.00176	43.77733
P04114	0	1	706.418	1411.829	1.36	0.00096	43.27026
P04114	0	1	468.2642	1870.035	1.96	0.00092	42.51616
P04114	0	1	778.9088	1556.81	0.78	0.0006	4.790524

P04114	0	0.9231	572.3258	1143.644	4.57	0.00262	21.38144
P04114	0	0.8857	690.357	1379.707	2.29	0.00158	16.29279
P04114	1	1	515.301	1543.889	0.47	0.00024	5.1334
P04114	0	0.7419	437.9236	1311.756	0.92	0.0004	15.40873
P04114	0	0.9762	502.2417	1504.711	4.61	0.00231	46.9518
P04114	0	0.5397	524.3379	1047.669	3.15	0.00165	7.991071
P04114	0	0.8485	708.8687	2832.453	1.91	0.00135	5.477514
P04114	0	1	519.3155	1555.932	2	0.00104	21.14695
P04114	0	1	429.2431	1285.715	1.04	0.00044	18.06917
P04114	0	1	872.0322	1743.057	3.7	0.00323	45.23229
P04114	0	0.6061	559.0025	1674.993	2.96	0.00165	41.08651
P04114	0	1	937.5317	1874.056	1.47	0.00138	7.625896
P04114	0	0.8974	435.9377	1305.798	0.3	0.00013	0
P04114	1	1	643.3131	2570.231	2.23	0.00143	5.152909
P04114	0	0.8235	510.9532	1530.845	2.87	0.00147	43.13586
P04114	0	0.7963	606.8294	1212.651	0.93	0.00056	18.81751
P04114	0	0.7561	437.2502	1309.736	0.45	0.00019	4.857244
P04114	0	0.8205	572.3248	1143.642	2.76	0.00158	4.332971
P04114	0	0.9429	490.2695	1468.794	1.37	0.00067	28.98824
P04114	0	0.7826	432.5632	1295.675	2.91	0.00126	0
P04114	0	0.9444	631.0177	1891.039	3.68	0.00232	27.89413
P04114	0	1	476.5859	1427.743	1.66	0.00079	7.74175
P04114	0	0.549	584.3593	1167.711	5.47	0.00319	24.44175
P04114	0	0.9048	589.3124	2354.228	2.08	0.00122	7.929016
P04114	0	0.625	524.3361	1047.665	-0.23	-0.00012	14.24474
P04114	0	0.9143	868.4918	1735.976	3.06	0.00265	29.69016
P04114	0	0.72	494.8002	988.5932	2.46	0.00122	7.122599
P04114	0	0.98	640.8657	1280.724	2.57	0.00165	29.27769
P04114	0	0.9722	456.2364	1366.695	0.38	0.00017	13.97264
P04114	0	0.8485	990.1873	2968.547	2.97	0.00294	0
P04114	1	0.7037	661.3765	2642.484	1.32	0.00087	60.38203
P04114	0	0.0625	518.9573	1554.857	1.52	0.00079	22.36887
P04114	0	1	789.3651	1577.723	1.27	0.001	67.85486
P04114	0	0.8182	714.3757	1427.744	2.22	0.00159	12.4142
P04114	0	1	606.7704	1212.533	1.76	0.00107	31.30492
P04114	0	0.9149	618.3055	1235.604	1.88	0.00116	15.64159
P04114	1	0.8571	437.5664	1310.684	1.83	0.0008	21.10795
P04114	0	0.6857	434.2434	1300.716	2.13	0.00092	23.97023
P04114	0	0.3636	539.3002	1077.593	2.09	0.00112	5.928447
P04114	0	0.8611	449.2574	1345.758	1.5	0.00067	7.368335
P04114	0	0.9836	642.3247	1283.642	2.58	0.00165	8.905486
P04114	0	0.875	678.8218	1356.636	2.1	0.00143	4.43446
P04114	0	0.8125	630.693	1890.064	3.68	0.00232	20.57629
P04114	1	1	543.9334	1629.786	1.24	0.00067	0
P04114	1	1	398.9776	1592.889	2.57	0.00102	21.53441
P04114	0	0.8545	596.3422	1191.677	2.43	0.00145	22.90174
P04114	0	1	458.2147	1372.629	0.79	0.00036	35.23486
P04114	0	0.7222	559.0021	1674.992	2.19	0.00122	0
P04114	0	0.8824	668.3947	1335.782	4.35	0.00291	0
P04114	0	0.7959	693.8738	1386.74	1.25	0.00087	17.36086
P04114	1	0.8125	649.0673	1945.187	1.34	0.00087	31.59378
P04114	0	0.5	592.3461	1775.024	3.97	0.00235	13.28751

P04114	0	0.8333	408.9791	1632.894	0.38	0.00015	35.73292
P04114	0	0.878	844.5118	1688.016	4.74	0.004	9.772592
P04114	0	0.7812	475.6134	1424.826	1.84	0.00087	16.68567
P04114	0	1	652.3848	1955.14	4.83	0.00315	17.38629
P04114	0	1	519.3153	1555.931	1.53	0.00079	18.9867
P04114	0	1	429.2436	1285.716	2.32	0.00099	43.51598
P04114	1	1	531.6339	1592.887	1.62	0.00086	5.577809
P04114	1	1	405.2441	1617.954	1.16	0.00047	29.13526
P04114	0	0.7576	388.5386	1163.601	1.21	0.00047	25.29027
P04114	0	0.8837	693.8739	1386.741	1.43	0.00099	28.97383
P04114	0	0.8923	479.2869	957.5665	2.76	0.00132	10.59847
P04114	0	0.6444	658.9141	1316.821	5.2	0.00343	10.47006
P04114	0	0.8387	641.0216	1921.05	4.22	0.0027	17.33551
P04114	0	1	549.012	1645.022	2.24	0.00123	10.1757
P04114	0	0.9302	476.586	1427.743	1.79	0.00085	26.06529
P04114	0	0.8966	696.3746	2087.109	3.94	0.00274	12.27354
P04114	0	0.5636	584.3486	1167.69	3.02	0.00177	35.23802
P04114	0	0.7667	408.9797	1632.897	1.95	0.00079	0
P04114	0	0.7907	503.8115	1006.616	2.25	0.00113	29.56282
P04114	0	0.8261	481.2699	961.5325	1.22	0.00059	28.24526
P04114	0	0.9737	741.874	1482.741	1.86	0.00138	5.334628
P04114	0	0.9744	797.9594	1594.912	2.04	0.00163	7.560391
P04114	0	0.8519	559.3023	1117.597	0.23	0.00013	3.2438
P04114	0	0.9744	589.8195	1178.632	2.58	0.00152	16.99992
P04114	0	0.6977	598.851	1196.695	2.4	0.00144	5.689356
P04114	0	0.8158	413.5717	1238.701	1.78	0.00074	45.46464
P04114	0	0.9375	504.2763	1510.814	2.71	0.00136	34.117
P04114	0	0.6222	508.3247	1015.642	3.1	0.00157	0
P04114	0	1	476.2578	1426.759	1.41	0.00067	17.83496
P04114	0	0.8667	648.0002	1941.986	3.28	0.00213	7.412726
P04114	0	0.8846	564.0787	2253.293	3.28	0.00185	9.35809
P04114	0	0.6765	490.2697	1468.795	1.8	0.00088	10.6375
P04114	0	0.7241	726.0536	2176.146	2.42	0.00176	0
P04114	0	1	471.5745	1412.709	1.33	0.00063	29.49665
P04114	0	1	610.3635	1829.076	1.4	0.00085	20.99583
P04114	0	0.7586	388.7166	1551.845	2.3	0.00089	0
P04114	0	0.7692	593.2959	1185.585	3.59	0.00213	16.28871
P04114	0	0.6667	568.3062	1702.904	3.6	0.00204	9.471786
P04114	0	1	379.1987	1135.581	1.39	0.00053	3.941931
P04114	0	0.963	442.242	1324.711	1.09	0.00048	11.06562
P04114	0	1	379.1989	1135.582	1.96	0.00074	18.39009
P04114	0	0.9487	568.2955	1135.584	3.45	0.00196	11.92844
P04114	0	0.4615	415.2477	1243.729	3.08	0.00127	53.5704
P04114	0	0.8286	510.9526	1530.843	1.74	0.00089	31.4304
P04114	0	0.9783	547.8032	1094.599	2.68	0.00147	0
P04114	0	0.4375	598.669	1793.992	4.33	0.00259	9.169598
P04114	0	1	471.5744	1412.709	1.26	0.0006	82.83998
P04114	0	0.88	563.3138	1125.62	2.49	0.0014	2.415234
P04114	0	0.55	671.3873	2012.147	2.76	0.00185	66.67792
P04114	0	0.9211	424.569	1271.692	1.32	0.00056	3.705432
P04114	0	0.7656	554.3385	1107.67	4.08	0.00226	6.78148
P04114	0	0.8095	650.8615	1300.716	2.11	0.00137	3.730811

P04114	0	0.6977	599.3345	1197.662	6.41	0.00384	0
P04114	1	1	464.7579	1856.01	1.97	0.00091	23.24651
P04114	0	0.5345	528.8574	1056.708	5.34	0.00282	29.44617
P04114	0	0.9231	712.916	1424.825	1.19	0.00085	36.33414
P04114	0	0.8974	592.6345	1775.889	3.64	0.00216	26.70787
P04114	0	0.8974	572.3249	1143.643	2.97	0.0017	3.701596
P04114	0	0.5294	498.6209	1493.848	1.95	0.00097	18.04885
P04114	0	0.8571	606.8292	1212.651	0.73	0.00044	5.654098
P04114	1	0.64	725.3915	2174.16	3.37	0.00244	20.1981
P04114	0	0.963	667.4108	1333.814	4.55	0.00304	9.346652
P04114	0	1	586.0907	2341.341	1.72	0.00101	4.296774
P04114	0	0.3125	679.0392	2035.103	3.94	0.00267	38.06491
P04114	0	0.975	589.82	1178.633	3.51	0.00207	67.9922
P04114	0	0.8571	647.9993	1941.983	1.96	0.00127	17.42842
P04114	0	0.8529	532.3088	1594.912	2.3	0.00122	8.794916
P04114	1	0.8929	857.4152	2570.231	2.42	0.00208	5.513754
P04114	0	0.8529	563.3138	1125.62	2.49	0.0014	100
P04114	0	0.6829	462.9183	1386.74	1.38	0.00064	11.29119
P04114	0	0.8636	647.9996	1941.984	2.34	0.00152	15.24193
P04114	0	1	440.9013	1320.689	1.36	0.0006	16.92626
P04114	0	0.8	693.6301	2771.498	6.01	0.00417	43.9791
P04114	0	1	683.8517	1366.696	1.53	0.00105	17.61644
P04114	0	0.7805	559.3042	1117.601	3.72	0.00208	18.39595
P04114	0	0.7021	555.3033	1109.599	3.06	0.0017	27.91577
P04114	0	0.775	472.6013	1415.789	2.12	0.001	3.065603
P04114	0	0.6154	523.8268	1046.646	1.42	0.00074	100
P04114	0	0.7	527.3103	1579.916	2.76	0.00145	38.10035
P04114	0	0.7273	452.8834	1356.636	1.69	0.00076	58.14102
P04114	1	0.9565	574.9907	1722.957	2.6	0.00149	6.556283
P04114	1	0.7727	502.3195	1504.944	1.03	0.00052	22.05148
P04114	1	1	577.2983	1729.88	1.25	0.00072	4.087327
P04114	0	1	583.322	1165.637	2.83	0.00165	12.67647
P04114	0	0.9815	487.2513	973.4954	0.52	0.00025	9.615032
P04114	0	1	519.6096	1556.814	3.39	0.00176	43.07314
P04114	0	0.8857	844.5092	1688.011	1.63	0.00138	22.93782
P04114	0	0.8667	605.3206	1813.947	5.97	0.00361	62.41048
P04114	0	0.9655	456.2378	1366.699	3.6	0.00164	32.05995
P04114	0	0.6897	623.3543	1868.048	4.58	0.00285	41.39274
P04114	0	0.8158	627.3582	1253.709	3.79	0.00237	11.91647
P04114	0	0.8571	389.9578	1556.809	0.29	0.00011	8.657466
P04114	0	0.8065	671.8374	1342.668	4.45	0.00299	24.3339
P04114	0	1	436.2239	1306.657	2.46	0.00107	59.89769
P04114	0	0.9714	476.2578	1426.759	1.34	0.00064	9.184903
P04114	0	1	566.9929	1698.964	4.15	0.00235	8.691909
P04114	0	1	449.257	1345.757	0.68	0.0003	20.25302
P04114	0	1	496.9359	1488.793	0.96	0.00048	13.4424
P04114	0	0.7632	568.295	1135.583	2.48	0.00141	3.606152
P04114	0	0.6538	381.885	1143.641	1.28	0.00049	19.10435
P04114	0	1	586.9474	1758.828	0.28	0.00016	4.946571
P04114	1	0.5333	649.0688	1945.192	3.69	0.00239	54.63821
P04114	0	0.9091	487.6022	1460.792	3.11	0.00152	60.62325
P04114	0	0.9167	798.9584	1596.91	1.17	0.00093	0

P04114	0	0.7241	431.2463	861.4853	2.78	0.0012	0
P04114	0	0.8125	578.8761	1156.745	2.38	0.00137	18.65723
P04114	0	0.9062	741.8738	1482.74	1.61	0.0012	4.356606
P04114	0	0.35	476.9353	1428.791	3.02	0.00144	10.94873
P04114	0	0.8148	473.2625	945.5178	1.64	0.00078	
P04114	0	0.8919	499.2629	997.5185	0.16	0.00008	16.28428
P04114	0	0.5938	662.8601	1324.713	2.27	0.00151	0
P04114	0	0.4286	731.7217	2193.151	0.43	0.00031	17.52582
P04114	0	1	798.9507	1596.894	4.17	0.00333	60.04685
P04114	0	0.7895	742.891	2968.542	1.26	0.00094	0
P04114	1	0.8611	491.9564	1473.855	2.82	0.00139	25.16206
P04114	1	0.8611	491.9564	1473.855	2.82	0.00139	25.16206
P04114	0	0.8077	720.7206	2160.147	0.62	0.00045	25.93238
P04114	0	0.9565	547.8021	1094.597	0.67	0.00037	14.14915
P04114	0	0.9412	552.2977	1103.588	6.11	0.00337	0
P04114	0	0.5143	544.3123	1630.922	4.7	0.00256	62.94799
P04114	0	0.6071	584.3499	1167.693	5.32	0.00311	29.66083
P04114	0	0.5814	529.8646	1058.722	4.04	0.00214	13.16629
P04114	0	1	384.5305	1151.577	1.76	0.00068	7.865205
P04114	0	0.8649	606.3475	1211.688	0.32	0.0002	40.49014
P04114	0	0.5652	509.5335	2035.112	0.97	0.00049	0
P04114	0	0.6591	528.8544	1056.701	-0.44	-0.00023	24.29427
P04114	0	0.913	498.2733	1990.071	-1.72	-0.00086	15.98617
P04114	0	0.8611	624.3461	1247.685	1.19	0.00074	4.575662
P04114	0	0.7381	619.8216	1238.636	3.18	0.00197	8.0049
P04114	0	0.7143	742.8911	2968.543	1.43	0.00106	5.74543
P04114	1	1	456.6258	1367.863	0.5	0.00023	66.83944
P04114	0	0.6735	598.8509	1196.695	2.3	0.00137	9.345694
P04114	0	0.9512	499.2633	997.5193	1.02	0.00051	100
P04114	0	0.7692	490.7814	980.5555	2.26	0.00111	12.19601
P04114	1	1	545.5355	2179.12	0.85	0.00046	32.61387
P04114	0	0.3478	603.3401	1808.006	2.48	0.0015	4.793478
P04114	0	1	408.9797	1632.897	2.02	0.00083	9.401805
P04114	0	0.963	379.199	1135.583	2.36	0.00089	30.31279
P04114	0	1	500.2723	999.5372	0.18	0.00009	21.24076
P04114	0	0.8837	624.3478	1247.688	4.03	0.00251	12.60859
P04114	0	1	575.627	1724.866	5.31	0.00305	7.452607
P04114	0	0.3226	581.6907	1743.057	3.94	0.00229	25.37302
P04114	0	0.3673	539.3005	1077.594	2.65	0.00143	0
P04114	0	0.5135	512.7725	1024.538	1.01	0.00052	0
P04114	0	0.9565	424.5695	1271.694	2.47	0.00105	47.45816
P04114	0		951.4844	1901.962	3.09	0.00294	0
P04114	0	0.4815	509.5967	1526.776	1.8	0.00092	3.944944
P04114	1	0.6875	433.2789	1297.822	1.37	0.00059	26.54815
P04114	0	1	389.958	1556.81	0.76	0.00029	29.78618
P04114	0	0.3947	523.8275	1046.648	2.82	0.00147	8.292784
P04114	0	0.9286	461.5909	1382.758	2.27	0.00105	49.00355
P04114	0	0.8235	636.3516	1271.696	3.99	0.00254	48.80053
P04114	0	1	566.315	1696.93	0.22	0.00012	10.78045
P04114	0	0.881	503.812	1006.617	3.1	0.00156	66.16414
P04114	0	0.92	733.8771	1466.747	2.66	0.00195	10.82374
P04114	0	0.9091	384.5305	1151.577	1.84	0.00071	51.3965

P04114	0	0.8235	655.0134	1963.026	1.26	0.00082	20.16067
P04114	0	0.8286	586.6242	1757.858	2.41	0.00141	8.220073
P04114	1	1	537.3273	1609.967	2.89	0.00155	16.62476
P04114	0	0.7381	406.9135	1218.726	3.58	0.00146	36.20231
P04114	0	0.7619	532.3087	1594.911	1.96	0.00104	2.207071
P04114	0	0.6923	456.7618	912.5163	1.57	0.00072	18.58581
P04114	0	0.7333	487.2514	973.4955	0.59	0.00028	0
P04114	0	0.9474	488.9186	1464.741	3.51	0.00171	27.57136
P04114	1	1	483.5997	1448.785	1.46	0.0007	29.26477
P04114	0	0.875	575.9712	1725.899	1.64	0.00095	15.17875
P04114	1	1	483.5997	1448.785	1.46	0.0007	29.26477
P04114	0	1	456.2367	1366.696	1.12	0.00051	2.481965
P04114	0	0.76	609.3475	1826.028	7.18	0.00437	14.39218
P04114	0	0.8621	863.9245	1726.842	2.57	0.00222	0
P04114	0	0.6829	644.3485	1287.69	3.15	0.00203	2.884801
P04114	0	1	484.257	1450.756	0.27	0.00013	0
P04114	0	0.9655	448.9077	1344.709	1.01	0.00045	6.714863
P04114	0	0.7917	537.6313	1610.879	0.78	0.00042	9.955923
P04114	1	1	545.5356	2179.121	1.08	0.00059	57.07236
P04114	0	0.6	652.3795	1303.752	1.35	0.00088	16.76585
P04114	0	0.8333	913.5129	1826.018	1.94	0.00177	0
P04114	0	0.5926	511.6245	1532.859	1.84	0.00094	3.979715
P04114	0	0.8857	772.4589	1543.911	2.74	0.00212	23.75401
P04114	0	0.9744	606.7705	1212.534	1.86	0.00113	7.611712
P04114	0	0.6667	525.6348	1574.89	-0.81	-0.00043	62.46359
P04114	0	0.9412	589.8203	1178.633	3.93	0.00231	17.3235
P04114	0	0.9	424.5689	1271.692	1.03	0.00044	17.66537
P04114	0	1	564.2968	1127.586	-2.09	-0.00118	12.27932
P04114	0	1	544.9678	1632.889	-2.98	-0.00162	69.80656
P04114	0	0.3929	671.8353	1342.663	1.27	0.00085	0
P04114	0	0.8696	510.9531	1530.845	2.75	0.00141	12.49575
P04114	0	1	379.1987	1135.581	1.39	0.00053	28.08025
P04114	0	0.7222	445.9309	1335.778	1.53	0.00068	24.0488
P04114	0	0.8824	640.6674	1919.988	1.62	0.00103	49.58657
P04114	0	0.963	789.3654	1577.724	1.66	0.00131	39.02721
P04114	0	0.9524	610.364	1829.077	2.2	0.00134	15.99836
P04114	0	0.6452	510.9532	1530.845	2.93	0.0015	0
P04114	0	0.7273	639.9907	1917.957	3.55	0.00227	13.02419
P04114	0	1	712.9158	1424.824	1.02	0.00072	48.95623
P04114	1	0.9375	692.7001	2076.086	0.07	0.00005	0
P04114	0	1	384.5303	1151.576	1.44	0.00055	37.56325
P04114	0	0.7857	503.8104	1006.614	0.01	0	4.539833
P04114	0	1	787.7613	2361.269	1.73	0.00136	9.233197
P04114	0	0.68	533.3076	1597.908	2.73	0.00145	10.16836
P04114	1	0.8929	736.0996	2941.377	0.9	0.00066	0
P04114	1	0.6429	403.2472	1609.967	2.58	0.00104	30.25116
P04114	0	0.8462	668.3924	1335.778	0.97	0.00065	6.343143
P04114	0	0.7857	465.2463	929.4853	0.41	0.00019	0
P04114	0	1	683.8525	1366.698	2.78	0.0019	5.507437
P04114	0	0.7778	776.4274	1551.848	4.28	0.00332	0
P04114	0	1	680.856	1360.705	1.98	0.00135	4.593351
P04114	0	0.64	1109.06	2217.113	2.7	0.00299	0

P04114	0	0.95	751.7687	2253.291	2.67	0.002	16.13864
P04114	0	0.3333	443.7839	886.5605	1.66	0.00074	6.513032
P04114	0	0.8857	679.047	2035.126	7.96	0.0054	66.55428
P04114	0	0.8462	499.2637	997.5201	1.81	0.0009	26.86415
P04114	0	0.5	782.4641	1563.921	2.39	0.00187	32.53048
P04114	0	0.5556	855.4542	1709.901	-0.06	-0.00005	30.09697
P04114	0	1	454.2395	1360.704	1.32	0.0006	42.21049
P04114	0	0.7609	578.8763	1156.745	2.69	0.00156	0
P04114	0	0.95	567.6401	1700.906	6.17	0.0035	0
P04114	0	0.8684	499.2632	997.5192	0.9	0.00045	0
P04114	0	0.8788	589.8195	1178.632	2.58	0.00152	34.24921
P04114	0	1	683.8519	1366.697	1.89	0.00129	5.584842
P04114	1	0.5	493.2609	1477.768	4.45	0.00219	0
P04114	0	0.9149	619.8203	1238.633	1.02	0.00063	3.62824
P04114	1	0.65	1014.548	3041.63	0.92	0.00093	5.633653
P04114	0	0.8485	528.8129	1056.619	1.52	0.0008	12.03749
P04114	0	0.9259	413.5711	1238.699	0.38	0.00016	26.96435
P04114	0	0.8276	572.3242	1143.641	1.69	0.00097	7.07499
P04114	0	0.9231	405.8975	1215.678	1.75	0.00071	42.82643
P04114	0	0.8519	778.9095	1556.812	1.72	0.00134	0
P04114	0	0.8065	416.5671	1247.687	2.57	0.00107	29.07967
P04114	0	0.9355	683.8524	1366.697	2.51	0.00172	5.671254
P04114	1	0.5517	483.6	1448.786	2.09	0.00101	9.429806
P04114	1	0.5517	483.6	1448.786	2.09	0.00101	9.429806
P04114	0	0.5556	381.8854	1143.642	2.32	0.00088	44.92138
P04114	0	0.625	837.9999	1674.992	2.68	0.00224	27.47167
P04114	0	0.4706	668.3938	1335.78	3.07	0.00205	15.5709
P04114	0	0.7	554.3377	1107.668	2.65	0.00147	29.56052
P04114	0	0.2105	559.0017	1674.991	1.54	0.00086	21.04757
P04114	0	0.625	598.8518	1196.696	3.73	0.00223	8.297427
P04114	0	1	460.574	1379.707	2.77	0.00127	5.296625
P04114	0	1	384.5308	1151.578	2.56	0.00098	46.23136
P04114	0	0.8158	445.9312	1335.779	2.08	0.00093	13.01136
P04114	0	1	490.2721	1468.802	6.67	0.00326	37.78534
P04114	0	1	456.2363	1366.694	0.32	0.00014	23.92336
P04114	1	0.8	401.9031	1203.695	0.94	0.00038	74.92777
P04114	0	1	379.1987	1135.582	1.47	0.00056	12.7689
P04114	0	0.3548	435.2566	1303.755	4.08	0.00177	14.78458
P04114	0	0.3333	671.3861	2012.144	0.94	0.00063	21.15609
P04114	0	1	458.0238	1829.073	-0.04	-0.00002	37.48754
P04114	0	0.7037	671.8359	1342.664	2.18	0.00146	5.378081
P04114	1	0.6667	857.4135	2570.226	0.43	0.00037	22.92661
P04114	0	1	544.9698	1632.895	0.61	0.00033	13.73358
P04114	0	0.8519	381.2086	1141.611	0.03	0.00001	51.81804
P04114	0	0.875	797.9578	1594.908	0.05	0.00004	0
P04114	1	0.875	667.3571	2666.406	6.72	0.00448	25.33017
P04114	1	1	483.6002	1448.786	2.4	0.00116	43.83767
P04114	1	1	483.6002	1448.786	2.4	0.00116	43.83767
P04114	0	1	623.6604	1868.967	3.98	0.00248	60.07015
P04114	0	0.9231	484.2579	1450.759	2.17	0.00105	80.39879
P04114	0	0.875	408.9796	1632.896	1.57	0.00064	40.50221
P04114	0	1	576.2868	1726.846	4.96	0.00286	5.545444

P04114	0	0.4688	523.8263	1046.645	0.48	0.00025	47.28819
P04114	0	0.28	592.3466	1775.025	4.9	0.0029	4.92606
P04114	0	0.9697	456.2374	1366.698	2.73	0.00124	34.58175
P04114	0	0.8421	563.3136	1125.62	2.06	0.00116	9.567071
P04114	0	1	448.2268	1342.666	3.28	0.00147	20.17022
P04114	0	0.5714	611.3518	1221.696	2.83	0.00173	0
P04114	0	0.8077	416.5668	1247.686	1.99	0.00083	34.19727
P04114	1	0.2222	658.3705	1973.097	2.38	0.00156	5.32258
P04114	0	1	495.601	1484.789	1.16	0.00057	36.85239
P04114	0	0.6667	503.8104	1006.613	-0.05	-0.00003	50.7957
P04114	0	1	384.5307	1151.578	2.4	0.00092	16.65363
P04114	0	0.7333	445.276	1333.813	3.95	0.00176	26.54824
P04114	0	0.7027	563.3146	1125.622	3.9	0.0022	12.23474
P04114	0	0.6429	503.7909	2012.142	-0.06	-0.00003	0
P04114	1	0.5357	539.9907	1617.957	2.98	0.00161	18.57733
P04114	0	0.9091	614.0441	2453.155	3.58	0.00219	11.70769
P04114	0	0.8571	741.8737	1482.74	1.53	0.00113	5.908511
P04114	1	1	574.9906	1722.957	2.5	0.00143	0
P04114	0	0.8421	570.3633	1139.719	4.84	0.00276	9.484889
P04114	0	0.6667	616.8785	1232.75	3.7	0.00228	48.82201
P04114	1	0.9091	857.4175	2570.238	5.13	0.0044	16.86457
P04114	0	0.1852	697.3662	1393.725	3.58	0.00249	13.03004
P04114	0	1	429.2433	1285.715	1.61	0.00069	50.12146
P04114	0	1	656.3823	1311.757	1.74	0.00114	24.46258
P04114	1	1	607.2871	2426.127	1.56	0.00095	12.72344
P04114	1	1	607.2871	2426.127	1.56	0.00095	12.72344
P04114	0	0.8182	408.9793	1632.895	0.98	0.0004	0
P04114	0	0.9412	639.9904	1917.957	3.17	0.00202	21.40736
P04114	1	1	667.3552	2666.399	3.88	0.00258	0
P04114	0	0.6154	624.3468	1247.686	2.36	0.00148	0
P04114	0	0.6897	439.6128	1316.824	7.53	0.00331	19.38299
P04114	0	0.5625	731.7227	2193.154	1.76	0.00129	16.50322
P04114	0	0.8	563.3138	1125.62	2.49	0.0014	2.540552
P04114	0	0.5385	411.5878	1232.749	2.99	0.00123	5.259064
P04114	0	0.9231	924.5079	2771.509	9.85	0.0091	10.15452
P04114	0	0.7391	799.4573	1597.907	2.09	0.00167	7.449416
P04114	0	0.8684	503.8117	1006.616	2.49	0.00125	6.917277
P04114	0	1	487.2519	973.4965	1.59	0.00077	38.9526
P04114	0	0.931	568.2952	1135.583	2.91	0.00165	0
P04114	0	1	990.1876	2968.548	3.28	0.00325	0

Average Rep	Ion Inject Ti	RT [min]	First Scan	Ions Score	Expectation	Percolator (	Percolator I
136.5	42.62	42.7956	15305	34	0.011235	0	0.000676
128.4	38.098	56.5524	18661	81	1.77E-07	0	1.04E-10
32.5	91.42	56.529	18651	94	8.47E-09	0	8.18E-10
153.3	100	56.0523	18469	40	0.002274	0	1.52E-06
5	100	32.578	10000	97	5.35E-09	0	1.41E-06
218	60.917	45.2531	15660	40	0.003666	0	0.000843
52.2	100	116.2611	43618	46	0.000882	0	0.000708
15.6	100	115.7594	43412	42	0.001962	0	0.000598
32.3	51.95	118.5193	44604	93	1.93E-08	0	3.4E-10
19.6	100	42.2908	15115	27	0.05579	0.000273	0.002107
24.7	100	59.4108	19742	43	0.002247	0.000951	0.003631
14.6	100	42.8426	15324	38	0.00531	0.001576	0.008689
50.5	100	32.2894	9900	34	0.012222	0.002543	0.01623
3.4	100	101.6235	37492	28	0.058758	0.00337	0.0228
12.3	100	60.6426	24327	34	0.013326	0.004192	0.03059
5.3	100	98.9441	37101	49	0.000584	0	0.000667
10.1	100	113.316	41150	62	2.86E-05	0	2.45E-06
45	58.884	113.8198	41372	76	9.65E-07	0	2.66E-08
2.9	100	97.0732	36701	78	7.96E-07	0	6.46E-08
5.8	100	96.5653	36497	53	0.000234	0	3.22E-06
28.4	100	55.7724	20330	79	3.37E-07	0	2.07E-08
23.6	100	55.2665	20128	72	1.67E-06	0	1.07E-08
10.9	100	98.4331	36902	32	0.02995	0.000868	0.003363
2.5	100	97.9273	36713	32	0.030294	0.001114	0.006369
29.4	100	55.3231	20150	12	1.406851	0.002376	0.01427
11	100	33.9012	11471	39	0.0029	0.002543	0.01566
12.4	100	33.396	11283	37	0.004419	0.00337	0.02421
41.7	100	33.4702	11309	38	0.003502	0.003892	0.02834
6.6	100	54.7591	19919	14	0.94506	0.006926	0.05025
7.2	100	108.7074	39281	21	0.379501	0.008559	0.06472
24.5	100	52.3207	18952	50	0.000152	0	7.24E-05
30.3	100	52.3185	18951	55	5.07E-05	0	5.12E-05
13.6	100	51.8075	18740	23	0.057439	0	0.001086
26.4	53.888	66.088	24503	77	4.22E-07	0	2.12E-06
22	100	66.1143	24515	48	0.000322	0	3.98E-05
8.7	100	65.6044	24306	43	0.001071	0	0.000133
3.9	100	52.3586	18920	28	0.019184	0.000407	0.002133
107.9	100	15.9979	4815	55	0.000106	0.000525	0.002384
0.8	100	61.5478	22368	51	6.4E-05	0.000758	0.002779
365.3	88.265	15.9601	4796	36	0.008971	0.001022	0.005551
210.7	100	15.8502	5016	55	0.000111	0.002012	0.01077
5	100	62.0585	22569	41	0.000623	0.002079	0.01135
3.3	100	51.8548	18726	25	0.041198	0.002777	0.01707
320.3	6.942	15.8454	5013	36	0.008892	0.003178	0.02046
20.4	100	58.6704	21487	12	0.876437	0.003533	0.0245
51.7	100	16.0092	4409	25	0.092658	0.007572	0.05456
9.7	100	97.3901	36844	71	1.05E-06	0	1.58E-08
5.7	100	96.8811	36621	90	1.51E-08	0	1.64E-11
234.2	43.252	141.1588	54528	75	8.74E-07	0	2.96E-08
0.5	100	96.3715	36415	16	0.356223	0.006721	0.04914
1.3	100	119.0966	44520	40	0.003033	0	0.000181

24.4	100	67.7032	23257	23	0.228884	0	2.61E-05
0	100	120.1973	45830	56	2.38E-05	0	4.15E-05
35.2	100	74.2911	27597	31	0.031801	0.000758	0.003183
17.2	100	50.0373	19768	42	0.001075	0.000951	0.00395
9.7	100	50.3476	16394	38	0.002314	0	0.000785
28.4	100	50.5343	16469	64	5.49E-06	0	1.03E-07
22.6	100	50.0302	16273	35	0.005165	0	0.00048
18.5	100	64.4695	21909	39	0.002796	0	0.000194
48.2	100	63.1396	22912	56	8.97E-05	0	0.00127
18.1	100	35.3676	13546	28	0.078746	0	0.000248
36.4	100	34.8637	13324	37	0.00876	0	2.93E-05
32.8	100	30.6673	10306	47	0.000365	0.000951	0.0039
17.2	100	58.0573	23222	23	0.182746	0.002012	0.01027
2.2	100	140.7153	54672	89	1.64E-08	0	9.37E-09
18	100	23.9633	8807	55	9.91E-05	0.000273	0.001952
20.7	100	53.9178	17735	33	0.026087	0	0.001006
12.4	100	58.7243	19640	48	0.00061	0	1.54E-05
21.5	100	64.9608	23657	33	0.018417	0	0.001432
20.4	100	64.4565	23453	13	2.092643	0.009514	0.07636
12.9	100	118.0987	44172	57	8.33E-05	0	3.55E-08
50.5	100	17.6964	5330	24	0.118408	0.002012	0.01044
47.3	100	77.4056	27322	52	9.06E-05	0	0.000235
66.1	100	48.415	19061	42	0.000957	0.001114	0.006252
10.8	100	62.7018	21210	19	0.236561	0.002376	0.01486
6.8	100	76.9582	27123	29	0.016286	0.002843	0.01833
18.3	100	76.9048	27099	20	0.130298	0.00337	0.02383
45.8	100	66.4856	24477	31	0.031461	0	0.000925
3.1	100	96.9058	34495	37	0.006925	0	0.00052
13	100	95.3582	35986	32	0.024652	0	3.74E-05
2.5	100	94.8476	35772	35	0.011273	0	0.00103
24.7	100	65.9842	24284	35	0.014679	0.00065	0.002668
3.3	100	97.4106	34694	25	0.124149	0.001022	0.005181
1.4	100	95.8668	36207	23	0.198384	0.001576	0.008698
7	100	66.7057	24562	19	0.504421	0.002079	0.01178
23.9	100	18.1952	5870	67	5.62E-06	0	5.06E-05
25.2	100	29.1246	10949	23	0.188989	0.000758	0.003223
11.8	100	16.5014	5332	55	0.000101	0.000758	0.003191
259.1	100	17.4153	5494	18	0.266877	0	7.56E-05
13.7	100	99.1469	37626	65	1.01E-05	0	0.00013
112.7	100	55.4456	22108	20	0.353985	0	0.00047
5.9	100	58.0737	23229	17	0.833107	0.001758	0.009141
129.2	100	38.2793	13140	42	0.000552	0.002543	0.0159
36.1	100	51.8987	16992	31	0.019621	0	0.000838
17.3	100	77.5913	27404	49	0.000383	0	0.000169
4.3	100	93.5095	34038	34	0.010931	0	0.00031
2.5	100	121.2913	47378	34	0.015099	0.001022	0.004976
18.6	100	88.6122	31184	40	0.004639	0	0.000697
10.4	100	89.0056	33863	25	0.092403	0.001114	0.006149
73	100	18.662	6086	19	0.577141	0	0.000657
0	100	109.3979	44927	22	0.255757	0.002079	0.01141
4.9	100	108.901	40498	33	0.018575	0.000758	0.002977
0.9	100	119.4946	45023	22	0.149549	0.001022	0.005158

40.6	100	61.6062	22285	34	0.017153	0.000273	0.002083
4.7	100	102.3394	36650	42	0.002614	0.001945	0.01004
32.8	100	34.5593	10611	33	0.011432	0	0.001138
15	100	68.8122	25409	29	0.036753	0.003674	0.0266
16.1	100	62.5352	23029	27	0.042997	0	0.001487
17.3	100	42.1698	13276	31	0.018206	0.003892	0.0283
16.1	100	62.5352	23029	27	0.042997	0	0.001487
17.3	100	42.1698	13276	31	0.018206	0.003892	0.0283
49.8	100	44.1063	15188	51	0.000218	0.001022	0.005549
12.8	100	74.6121	27734	37	0.006367	0.001576	0.008165
6.2	100	103.3135	39989	30	0.025771	0.001114	0.006055
15.6	100	27.5498	8286	14	1.613118	0.001758	0.009665
16.2	100	62.636	22694	7	7.252389	0.005792	0.04291
27.1	100	26.4444	9863	1	29.2611	0.007836	0.0569
49.7	100	35.2718	13501	26	0.114444	0.001022	0.005355
0.4	100	86.507	32194	17	0.8561	0.002079	0.01143
1.4	100	93.8515	33306	42	0.003124	0.000525	0.002379
68.5	100	52.9124	19247	24	0.127393	0.004192	0.0301
62.6	100	23.5803	8060	36	0.009112	0.001022	0.004895
38.2	100	46.3238	16141	25	0.051912	0.002543	0.01612
30.3	100	65.1075	22174	44	8.29E-05	0.000868	0.003471
22.1	100	27.8216	9655	21	0.323192	0.003533	0.02459
439.9	100	17.362	5783	22	0.057444	0.001022	0.004849
0	100	120.7123	45555	18	0.049604	0.002843	0.01837
46.1	100	17.2032	5135	19	0.413638	0.001022	0.004812
23.9	100	88.3487	31925	16	1.003451	0.002843	0.01872
14	100	60.4298	22140	44	0.000925	0.001114	0.006175
36.4	100	53.0498	21093	37	0.0076	0.00337	0.0242
14.8	100	62.9565	23114	27	0.067586	0.002315	0.01333
31.7	100	57.0989	19001	18	0.590237	0.003674	0.0256
4	100	89.2679	33309	50	0.000365	0	0.000292
10.2	100	89.2582	33305	50	0.000341	0	0.000111
32.7	9.911	88.2598	32920	73	1.7E-06	0	2.7E-07
8.1	100	88.7576	33115	51	0.000317	0	0.000428
40.2	5.167	88.254	32917	75	1.1E-06	0	2.22E-07
28.1	100	51.1727	16713	21	0.342394	0	0.00128
5.1	100	83.0203	30853	28	0.057249	0	0.000185
18.4	100	87.7462	32706	56	9.99E-05	0	4.01E-06
31.3	1.662	68.5723	25304	68	6.49E-06	0	6.19E-07
29.6	58.28	68.6392	25332	91	2.73E-08	0	6.23E-10
9.4	100	68.7342	25374	44	0.001599	0	0.001198
6.3	100	69.5855	25716	41	0.002806	0	0.000797
25.7	100	69.0773	25515	65	1.33E-05	0	1.53E-07
49.3	43.781	63.7359	23418	46	0.000922	0	0.00108
46.9	100	57.6402	19219	34	0.014759	0	0.000641
4.1	100	64.3835	23662	57	5.05E-05	0	0.000423
77.6	17.356	64.239	23609	51	0.00025	0	0.001127
3.8	100	82.1746	30536	30	0.04226	0	0.00028
9.3	100	82.0119	30476	25	0.125128	0	6.58E-05
9.6	100	81.6708	30348	27	0.084903	0	0.000145
8.3	100	81.5063	30286	27	0.073003	0	0.000182
10.1	100	82.514	30664	34	0.014137	0	2.67E-05

7.6	100	55.121	18211	48	0.000783	0	8.29E-05
0.6	100	55.1236	18212	34	0.017944	0	0.000289
17.6	100	75.0969	27841	27	0.082775	0	0.000712
4.5	100	38.4029	11951	61	2.45E-05	0	0.001428
6.9	100	24.343	7906	58	3.01E-05	0	0.000982
21.8	100	39.3984	12297	41	0.002884	0	0.00143
38.4	100	23.0512	7414	40	0.003138	0	0.00093
14.7	100	29.8338	9961	67	7.81E-06	0	0.000122
33.1	100	35.4424	12046	38	0.004392	0	0.000236
11.5	100	45.9594	14674	54	0.00016	0	6.7E-05
11.5	100	45.9594	14674	54	0.00016	0	6.7E-05
20.2	100	46.4667	14867	65	1.17E-05	0	2.29E-05
20.2	100	46.4667	14867	65	1.17E-05	0	2.29E-05
9	100	119.4643	44656	57	4E-05	0	1.08E-05
32	100	15.1434	4255	30	0.034953	0	0.00116
36.4	100	14.8534	4147	33	0.015535	0	0.001312
1.8	100	132.4457	49151	43	0.001043	0	9.68E-05
23.5	35.082	131.9448	48972	53	9.9E-05	0	1.48E-08
0	100	130.3155	48421	29	0.015557	0	0.000455
6.1	100	108.8976	44732	63	2.03E-05	0	1.88E-05
5.3	100	108.7816	44688	54	0.000178	0	3.86E-05
5.9	100	109.2922	44884	57	8.93E-05	0	2.75E-05
8.2	100	109.4081	44932	79	5.35E-07	0	3.08E-08
20.6	100	110.3017	45328	41	0.003104	0	0.000444
5.4	100	109.9185	45151	70	4.03E-06	0	1.31E-06
24.3	100	109.7951	45095	54	0.000186	0	5.1E-06
3.2	100	102.4165	42057	42	0.002612	0	0.000411
6.8	12.361	101.9126	41863	67	8.38E-06	0	5.19E-06
7	85.262	101.5135	41704	65	1.24E-05	0	2.72E-06
9.3	3.983	101.4086	41662	75	1.23E-06	0	7.99E-07
22.2	100	43.3436	15514	35	0.009998	0	0.001017
17.6	100	43.1498	15436	77	7.29E-07	0	9.73E-07
23.1	100	59.1549	19798	37	0.007404	0	0.000254
84.1	4.349	60.1605	22041	51	0.000238	0	0.001305
135	11.242	58.1428	19424	48	0.000632	0	0.000298
53.1	100	58.645	19610	37	0.008415	0	0.000548
21.2	100	51.4576	18688	35	0.01139	0	0.000856
148.9	4.277	60.4305	20307	59	3.53E-05	0	4.71E-05
47	57.244	60.4589	20318	85	7.56E-08	0	7.63E-07
21.2	100	51.4576	18688	35	0.01139	0	0.000856
85.2	100	59.9213	20092	45	0.000782	0	8.08E-05
2.5	100	69.1152	23831	52	0.000221	0	7.54E-05
12	100	69.7858	24100	46	0.000868	0	1.33E-06
35.6	100	14.6067	4178	47	0.000648	0	0.001371
114.9	100	14.5851	4169	45	0.000957	0	0.000106
3.7	100	140.6238	57525	85	1.46E-07	0	1.47E-09
101.4	100	52.8074	17229	41	0.003715	0	6.14E-05
9.5	100	65.7441	22168	47	0.000832	0	4.49E-07
2.6	29.228	62.7411	20996	57	8.74E-05	0	1.45E-08
4.3	86.101	62.261	20810	56	0.000126	0	1.91E-09
2.2	17.673	62.2385	20800	55	0.000158	0	1.11E-08
2.4	100	62.7664	21006	62	2.99E-05	0	1.03E-09

61	7.949	64.4971	21692	42	0.001872	0	5.86E-07
61.6	100	64.4036	21656	28	0.054819	0	0.000122
0.3	100	63.2738	21208	48	0.000685	0	8.97E-06
13.8	100	63.2467	21197	47	0.000872	0	2.33E-07
91.8	36.913	27.7745	8361	61	2.54E-05	0	6.43E-05
90.4	36.259	27.4831	8265	44	0.001116	0	0.00114
81.8	2.313	27.2708	8193	58	5.54E-05	0	0.000169
67.9	94.792	140.8249	55398	83	2.48E-07	0	9.42E-10
31.9	71.454	140.8228	55397	70	4.35E-06	0	1.64E-09
40.3	100	140.767	55367	111	2.67E-10	0	3.12E-14
9.8	21.931	140.764	55365	102	2.03E-09	0	1.67E-11
23.9	100	15.2336	4161	35	0.009444	0	0.000265
10.5	100	36.2184	11223	29	0.031967	0	0.00066
70.2	25.217	97.3035	34650	65	9.96E-06	0	0.000237
7.9	100	89.41	31524	69	4.46E-06	0	5.63E-06
81.6	45.86	88.9059	31315	18	0.50117	0	4.47E-05
6.2	100	93.3989	33136	50	0.0003	0	0.000187
1.3	100	107.5418	38773	39	0.004615	0	0.000353
71	21.14	88.7522	31248	46	0.000913	0	1.78E-06
57	19.079	88.7406	31242	73	1.68E-06	0	2.44E-08
0.4	100	122.3613	44907	33	0.017932	0	2.38E-05
0	100	77.461	26758	37	0.007894	0	4.22E-05
40.3	98.337	77.2925	26698	30	0.036704	0	1.26E-06
0.9	100	76.9597	26568	58	6.44E-05	0	3.17E-08
2.7	33.561	76.7827	26502	52	0.000249	0	8.53E-09
7.2	100	122.012	44770	67	8.67E-06	0	5.41E-08
14	100	121.5059	44563	67	7.16E-06	0	2.01E-10
4.7	100	77.7994	26890	50	0.000388	0	2.32E-06
17.6	57.805	121.8591	44705	46	0.001035	0	1.31E-10
83.3	100	73.2864	25097	33	0.010149	0	0.000423
9.7	100	73.2003	25063	51	0.000313	0	1.03E-06
84.8	11.655	73.1817	25056	51	0.000162	0	6.44E-05
20.2	19.658	73.025	24994	68	6.72E-06	0	1.35E-09
17.5	100	72.7833	24890	44	0.000971	0	0.00061
11.8	100	72.6715	24849	38	0.003195	0	0.000192
12.6	100	72.6106	24825	20	0.460521	0	0.000975
1.6	100	72.5145	24786	32	0.029017	0	0.00059
22.8	100	73.338	25120	38	0.005127	0	0.000671
19.2	100	73.3893	25141	47	0.000678	0	9.08E-05
40.2	100	74.6971	25688	50	0.00034	0	0.000697
22.4	100	73.8929	25361	61	2.7E-05	0	9.8E-06
45.3	25.014	73.8421	25338	56	7.89E-05	0	2.84E-05
77.7	58.628	73.6921	25276	47	0.000343	0	3.77E-05
7	100	73.5365	25204	59	5.98E-05	0	7.6E-07
9.5	45.774	121.3538	44498	70	3.79E-06	0	2.63E-12
20.7	100	88.3457	31076	56	7.92E-05	0	2.72E-05
3.3	100	88.3158	31063	70	3.05E-06	0	1E-05
24.6	8.961	88.2375	31032	69	4.47E-06	0	1.08E-07
39.7	83.577	87.8389	30874	60	3.73E-05	0	4.86E-07
4.1	100	87.8135	30864	53	0.000175	0	8.15E-05
40.5	8.926	87.734	30829	70	3.92E-06	0	6.55E-08
12	100	87.2234	30620	51	0.00029	0	3.88E-05

7.5	100	82.965	28922	49	0.000515	0	0.000184
28.1	21.104	141.219	52667	103	1.97E-09	0	9.18E-13
24.5	25.115	141.2199	52668	85	1.22E-07	0	1.58E-09
12.1	100	72.5705	26651	58	4.53E-05	0	7.12E-05
5.4	100	72.2036	26489	54	0.00016	0	3.66E-06
29.3	100	15.0902	4200	32	0.018934	0	0.001467
18	100	72.4209	26587	47	0.000512	0	7.6E-06
41	39.332	75.7345	27926	79	3.14E-07	0	1.13E-06
31.8	60.692	74.1639	27309	47	0.000772	0	0.00134
53.1	39.716	73.6574	27114	48	0.000568	0	0.000118
15	100	140.8376	52461	100	3.33E-09	0	1.18E-11
14.4	100	140.8508	52467	100	3.17E-09	0	3.54E-12
16.6	17.954	140.8955	52491	114	1.51E-10	0	1.99E-13
13.7	53.167	140.782	52430	96	1.05E-08	0	4.32E-15
10.7	100	140.812	52447	61	2.6E-05	0	4.74E-08
9.6	100	141.0493	52573	86	7.35E-08	0	9.94E-09
29.4	100	83.6126	31114	39	0.004503	0	9.91E-05
4.2	100	140.9086	52498	112	2.33E-10	0	2.96E-10
14.1	100	140.9108	52499	93	1.81E-08	0	7.19E-10
5.9	89.163	69.8371	25536	49	0.000269	0	4.38E-05
20	100	54.2322	19276	41	0.003124	0	2.12E-05
15.6	100	54.7428	19488	39	0.004963	0	0.00039
20.2	100	26.8409	8079	42	0.00194	0	0.000292
50.9	100	49.2511	17310	63	1.29E-05	0	0.001194
61.4	86.948	51.2579	18094	88	7.3E-08	0	1.62E-09
37.9	5.492	50.751	17900	98	6.7E-09	0	1.06E-09
32	6.798	50.4588	17783	81	3.37E-07	0	7.65E-09
31.7	100	15.5013	4347	45	0.001132	0	0.00056
12.9	100	66.7191	24310	30	0.03666	0	0.000186
13.3	100	17.6396	5107	31	0.038553	0	0.000449
80	100	140.4503	55184	117	1.79E-11	0	4.57E-10
28.8	33.859	140.4313	55174	92	6.54E-09	0	3.8E-10
6.7	29.945	99.4279	37757	85	8.12E-08	0	1.81E-07
26.7	10.163	99.4035	37746	91	2.02E-08	0	1.47E-09
3.8	100	92.3529	34757	37	0.005719	0	0.001518
31.6	47.186	91.3458	34343	77	5.65E-07	0	2.11E-08
8	100	90.6739	34092	46	0.00076	0	5.77E-05
17.6	35.41	91.8496	34551	81	2.65E-07	0	8.98E-09
6	15.115	105.9902	40583	64	5.25E-06	0	1.72E-05
31.4	9.136	105.9557	40566	84	5.47E-08	0	3.26E-07
3.4	100	105.4805	40349	61	1.12E-05	0	3.97E-05
23.2	100	109.8544	40878	84	1.35E-07	0	7.11E-11
23.1	100	139.6837	53746	53	4.69E-05	0	4.91E-08
67.1	2.788	89.1158	33913	58	6.36E-05	0	6.91E-05
24.1	22.964	113.7589	42540	79	5.58E-07	0	1.02E-08
4.8	23.623	113.773	42546	70	4.57E-06	0	6.44E-07
2.5	100	96.2288	36939	36	0.012345	0	0.000803
20	92.912	95.72	36730	48	0.000681	0	0.000201
12.8	92.397	95.3405	36570	61	3.21E-05	0	2.74E-06
21.7	58.098	95.2157	36514	48	0.000715	0	6.71E-05
0.4	100	111.5192	41585	96	1.25E-08	0	4.37E-07
6.1	100	96.7351	37142	38	0.006612	0	0.000427

13.7	100	94.8343	36346	49	0.000542	0	0.000166
0.6	100	93.0413	35567	47	0.000636	0	0.001342
10	49.412	94.7118	36292	44	0.001673	0	4.04E-05
10.5	100	94.2086	36078	42	0.002927	0	0.000223
172.1	3.96	140.5927	54187	66	4.58E-06	0	3.7E-06
2.4	100	141.2964	54969	31	0.037107	0	0.000972
116.1	100	13.6894	3914	34	0.013462	0	0.000401
33.2	30.596	102.8952	38052	70	4.45E-06	0	3.77E-06
3.8	100	102.945	38072	45	0.00162	0	0.000409
0	100	134.2669	52336	83	1.43E-07	0	1.69E-06
50	88.716	140.3717	54077	59	4.14E-05	0	1.31E-10
23.1	100	106.0869	39346	31	0.025658	0	3.04E-05
20.4	24.272	131.8755	50352	61	9.79E-06	0	3.44E-06
2.4	100	131.7344	50295	29	0.021095	0	0.00037
14.1	100	131.6865	50276	32	0.010348	0	5.66E-05
2.2	100	129.1129	49172	49	0.000493	0	4.19E-05
35.3	100	56.0867	20017	42	0.002163	0	0.000466
18.3	100	55.6399	19839	83	1.58E-07	0	5.33E-07
115.7	15.021	55.5809	19816	53	0.000179	0	4.01E-05
4.9	100	129.4087	49306	93	1.93E-08	0	3.44E-09
10.2	100	118.6687	44672	30	0.024394	0	7.31E-07
31.3	100	65.8261	24019	37	0.007226	0	7.79E-05
1.2	100	115.5712	43327	38	0.008063	0	0.000513
34.3	41.416	66.3411	24232	87	6.31E-08	0	1.04E-06
85.1	3.804	66.3295	24227	40	0.003611	0	0.000572
47.9	67.416	118.6178	44648	65	6.55E-06	0	6.01E-10
31.4	100	66.8323	24445	42	0.002197	0	4.43E-05
14.8	100	69.5341	25561	41	0.002798	0	9.15E-05
6.9	100	102.4402	37855	61	3.75E-05	0	0.000131
104.4	100	70.5957	28621	51	0.000339	0	8.28E-05
7.5	100	44.7688	15971	51	0.000164	0	0.00095
32.7	100	49.5078	19545	36	0.010699	0	0.000364
37.5	100	51.3075	18517	47	0.000273	0	0.000473
67.8	8.102	50.7373	18286	46	0.000346	0	0.000732
28.6	36.117	141.0307	54449	57	5.05E-05	0	0.000335
1.2	93.779	78.7188	27907	15	1.441885	0	1.12E-05
48.2	37.822	48.5438	17432	48	0.000252	0	0.000265
53.3	100	49.0478	17625	52	9.6E-05	0	9.06E-05
43	2.841	44.4835	17360	50	0.00044	0	6.17E-05
183.8	3.731	45.7493	17926	36	0.0089	0	0.00031
199.1	100	45.2421	17691	25	0.107395	0	2.5E-05
16.7	100	70.0835	28396	52	0.000254	0	0.001392
69.3	9.763	50.0654	18030	55	5.19E-05	0	0.000517
15.3	100	86.9561	32052	56	3.54E-05	0	0.000238
55	100	86.4548	31884	47	0.000338	0	0.000453
52.8	100	85.9505	31714	46	0.000376	0	0.000823
19.1	100	62.8898	25310	61	2.43E-05	0	0.000224
22.8	100	141.5457	54757	104	1.13E-09	0	5.24E-11
0.8	94.784	141.5358	54752	44	0.000857	0	0.000107
48.6	5.024	85.4972	30729	31	0.027214	0	9.03E-07
40.6	46.488	85.5514	30753	36	0.009587	0	8.5E-07
12.4	100	86.0002	30941	29	0.047697	0	4.7E-06

97.7	68.421	141.3667	54655	104	7.86E-10	0	9.21E-13
28.2	28.035	141.365	54654	77	3.58E-07	0	1.59E-10
8.7	49.164	56.4759	22547	45	0.001395	0	9.46E-05
59.1	56.71	141.3641	54653	61	2.07E-05	0	2.86E-11
32.2	27.704	55.971	22331	45	0.001404	0	0.000263
15.4	100	57.4837	22988	40	0.004929	0	0.000451
12	100	56.9805	22763	47	0.000826	0	9.79E-05
49.4	100	141.3746	54659	80	2.54E-07	0	2.66E-15
66.7	89.696	54.965	21914	36	0.011077	0	0.000423
63.2	35.493	55.4691	22120	47	0.000963	0	8.63E-05
186.9	100	55.4094	22092	75	1.36E-06	0	1.29E-05
29	6.483	29.8773	11246	67	8.03E-06	0	3.55E-05
37.3	5.899	140.8143	54315	98	5.94E-09	0	3.78E-16
44.7	100	140.7981	54306	65	1.03E-05	0	1.05E-10
329	23.386	140.7637	54285	50	0.000276	0	0.000596
2.4	100	140.7477	54275	63	9.58E-06	0	0.000346
11.2	8.762	140.7857	54299	91	2.69E-08	0	8.89E-13
11.2	100	71.792	29141	38	0.004087	0	0.000235
37.9	100	39.4948	15245	33	0.018954	0	0.000892
359.7	25.892	140.9153	54380	144	1.57E-13	0	8.74E-16
26.8	16.464	140.8922	54365	80	6.63E-08	0	1.12E-09
75.8	4.214	140.8914	54364	77	1.12E-07	0	1.31E-11
2.6	100	131.3715	50145	38	0.001849	0.000141	0.001645
143	14.753	20.9613	6517	21	0.316589	0.000141	0.001624
241.2	100	15.1572	4420	25	0.10876	0.000141	0.001795
79.5	100	42.6934	15268	42	0.001954	0.000141	0.001766
180.1	9.349	12.9888	3512	35	0.01421	0.000141	0.001562
135.5	100	14.4527	4114	33	0.015796	0.000141	0.001755
5.7	100	94.3311	36131	36	0.012243	0.000141	0.001813
5.8	100	87.3363	30665	40	0.003688	0.000141	0.001617
92.4	9.298	49.5573	17827	46	0.00035	0.000273	0.001872
175.8	1.368	15.2833	4700	32	0.02222	0.000273	0.002096
0	100	126.0139	46381	33	0.019225	0.000273	0.001933
1.3	100	102.0194	41908	35	0.01246	0.000273	0.001849
123	6.27	55.4692	20273	49	0.000428	0.000273	0.002032
18	100	86.0611	30966	4	14.60519	0.000273	0.00203
12.6	100	88.8532	31293	35	0.010982	0.000525	0.002525
9.1	100	50.9512	18371	40	0.001641	0.000525	0.002376
4.4	100	92.9945	35547	38	0.005154	0.000525	0.002223
55.5	100	42.6591	15255	39	0.003971	0.000525	0.00228
35.2	100	30.184	11381	40	0.003689	0.000525	0.002257
10	100	80.8186	30321	38	0.005858	0.000525	0.00234
9.5	100	92.486	35325	28	0.047809	0.00065	0.002717
56.2	100	25.9858	8517	22	0.173172	0.00065	0.002753
1.5	100	130.2483	50770	32	0.012544	0.00065	0.0027
30.9	100	64.3464	23648	36	0.009021	0.00065	0.002661
6.6	100	95.8415	36781	41	0.003176	0.000758	0.003231
26.4	100	54.453	21682	18	0.636048	0.000758	0.002837
24.9	100	34.9889	12437	21	0.201893	0.000758	0.003208
1.4	100	25.3718	8739	49	0.00028	0.000758	0.002786
21.8	100	36.9918	12421	43	0.001681	0.000758	0.002814
0.7	100	68.6113	23616	30	0.036682	0.000758	0.003169

16.9	100	107.0417	39736	35	0.014632	0.000758	0.003046
20	100	50.2477	17699	20	0.439636	0.000758	0.002866
40.1	100	58.1853	19441	42	0.002178	0.000758	0.003197
3.3	100	72.4125	24746	32	0.025305	0.000868	0.003547
9	100	59.8076	21909	28	0.048458	0.000868	0.003453
14.2	100	87.9178	32927	54	0.00015	0.000868	0.003304
0	100	61.3458	22127	34	0.019248	0.000868	0.003345
7.9	100	49.9392	17979	38	0.002521	0.000951	0.00458
10	100	73.7936	25318	33	0.01012	0.000951	0.003884
37.6	100	26.769	8017	22	0.198996	0.000951	0.004389
151.3	100	28.4412	9169	21	0.274255	0.000951	0.004184
29.5	60.601	50.8013	17922	45	0.001265	0.000951	0.003781
0.6	100	71.294	26119	35	0.008176	0.000951	0.003998
8.9	100	96.8023	34454	38	0.004987	0.000951	0.00385
6.2	100	55.9974	22343	31	0.031363	0.000951	0.004134
23.4	100	23.3841	7544	36	0.008608	0.000951	0.003672
2.5	100	92.5365	35348	41	0.002789	0.000951	0.003737
20.8	100	44.9661	14298	19	0.592911	0.000951	0.004407
20.4	50.624	85.5502	30752	14	1.296896	0.001022	0.005598
6.5	100	68.066	25090	32	0.024082	0.001022	0.004993
90.3	100	38.3931	11947	41	0.003016	0.001022	0.005471
1.4	100	19.6052	6893	50	0.000478	0.001022	0.00572
2.1	100	21.9151	6486	58	1.68E-05	0.001022	0.005546
0.4	100	134.3722	52377	39	0.003568	0.001022	0.004713
21.5	100	64.0793	21525	15	1.415521	0.001114	0.005895
0	100	134.781	52522	32	0.019526	0.001114	0.006015
177.9	60.041	51.0796	16580	12	1.611266	0.001114	0.006232
38.2	88.529	97.3359	34663	35	0.009783	0.001114	0.006373
5.9	100	82.6757	30724	23	0.197078	0.001114	0.006186
1.3	100	88.7653	33118	32	0.023972	0.001114	0.006019
147.2	100	36.5873	12254	24	0.126205	0.001114	0.005914
5.5	100	56.714	18722	38	0.0034	0.00121	0.006567
33.8	100	21.5627	7263	45	0.000419	0.00121	0.006694
215.3	100	37.1717	12859	21	0.311047	0.00121	0.006606
75.5	100	21.2287	7631	45	0.000471	0.00121	0.006727
72	100	64.2058	23597	32	0.019505	0.00121	0.006836
49.9	100	27.2881	9435	21	0.243	0.00121	0.006795
71.9	100	20.4583	6326	15	1.37718	0.001308	0.006963
0.5	100	130.7487	50973	35	0.007438	0.00141	0.007362
0.4	100	131.6762	48876	28	0.038588	0.001498	0.007716
2.5	100	96.833	34467	35	0.008792	0.001498	0.007756
0.8	100	97.8545	34864	45	0.001426	0.001498	0.008047
254.9	88.162	29.6852	11172	27	0.086053	0.001498	0.007525
15.7	100	17.2362	4959	54	3.87E-05	0.001498	0.007704
1.9	100	93.634	35012	40	0.001604	0.001498	0.007948
0.8	100	108.9006	40900	31	0.037496	0.001576	0.008488
23	100	50.8572	18352	35	0.004509	0.001576	0.008377
5.2	100	23.4608	6987	25	0.095026	0.001576	0.00848
2.7	100	93.5444	35787	41	0.002885	0.001758	0.009304
6.8	100	83.1816	30912	10	4.180216	0.001758	0.009008
21.8	100	48.789	15780	19	0.459333	0.001758	0.009064
15.7	100	54.6071	17910	19	0.370237	0.001854	0.009877

85.3	100	55.2853	22039	41	0.003421	0.001854	0.009886
2.3	100	84.9915	30515	20	0.381069	0.001854	0.00974
9.4	100	87.535	33203	53	0.000114	0.001854	0.009884
18.1	100	69.9052	23791	34	0.009677	0.001945	0.009944
18.1	100	69.9052	23791	34	0.009677	0.001945	0.009944
0	100	129.4264	49313	18	0.603525	0.002012	0.01056
18.3	100	73.2829	25095	36	0.004325	0.002012	0.01027
25.7	100	39.3319	14021	30	0.034473	0.002012	0.01105
24.9	60.269	46.2559	18148	28	0.055187	0.002012	0.01113
22	100	27.3098	9019	40	0.00291	0.002079	0.01236
4.3	100	60.9451	21966	16	1.164599	0.002079	0.01157
1.4	100	76.8884	28560	47	0.000106	0.002079	0.01179
8.5	100	68.2285	25155	22	0.239715	0.002079	0.01146
12.7	100	54.9115	18025	22	0.23456	0.002079	0.01171
160.6	100	21.7472	7848	49	0.000175	0.002079	0.01235
20.3	100	55.9748	20474	43	0.001514	0.002079	0.01214
158.7	100	48.3914	16964	49	0.000344	0.002079	0.01162
2.7	100	51.8746	16981	42	0.001511	0.002257	0.01254
40.6	100	48.9467	17724	37	0.005065	0.002315	0.01353
53.4	100	37.1228	12838	39	0.004542	0.002315	0.01399
28.1	100	21.0003	6533	40	0.003814	0.002315	0.01396
17.9	100	14.5627	4339	42	0.002059	0.002315	0.01414
5.6	100	20.1403	5925	29	0.017399	0.002315	0.01331
15.7	100	54.1497	19245	31	0.027445	0.002315	0.01389
2.6	100	89.5486	33417	45	0.000499	0.002315	0.01337
13.5	100	29.6322	9894	42	0.001941	0.002376	0.0142
0.8	100	67.9922	24809	39	0.004051	0.002376	0.01426
0.4	100	118.9592	44468	26	0.048103	0.002376	0.01538
5.6	100	67.6383	22906	22	0.228133	0.002376	0.0149
0.4	100	103.4072	38920	24	0.148157	0.002543	0.01589
11.1	100	49.3995	17315	47	0.000202	0.002543	0.01627
5.3	100	55.4912	22131	29	0.059686	0.002719	0.0165
49.5	100	51.7941	18295	38	0.002531	0.002719	0.01643
3.3	100	73.039	27067	41	0.001198	0.002777	0.01764
7.1	100	49.4342	17774	33	0.007658	0.002777	0.01714
2.3	100	76.389	28377	45	0.000183	0.002777	0.01811
17.3	100	38.6792	12049	15	0.62586	0.002843	0.01912
4.2	100	81.1647	30152	16	0.96557	0.002843	0.01835
10	100	115.4404	43267	25	0.026486	0.002843	0.01882
24.3	100	29.3442	9909	34	0.012605	0.002843	0.0185
0.9	100	131.3227	48755	17	0.199802	0.00302	0.01957
19.8	100	15.6188	4443	38	0.005685	0.003096	0.01998
3.5	100	44.7733	17486	36	0.011362	0.003096	0.01975
5.9	100	32.5455	12362	45	0.000111	0.003178	0.02042
286.5	14.86	48.1105	16851	44	0.001151	0.003254	0.02108
18.1	100	15.508	4245	38	0.005123	0.00337	0.02365
16.8	100	51.4392	18589	46	0.000214	0.00337	0.02393
28.1	100	15.6606	4651	34	0.012852	0.00337	0.02427
7.4	100	59.9512	20104	31	0.02295	0.00337	0.02188
23.4	100	50.429	17771	24	0.149969	0.00337	0.0225
166.3	11.56	47.8893	16761	55	8.87E-05	0.00337	0.0224
93.4	100	21.7412	6815	12	2.278812	0.00337	0.02261

35.6	100	36.7923	12568	44	0.000909	0.00337	0.02225
122.2	100	27.9267	9366	38	0.005278	0.00337	0.0232
80.9	100	22.024	6927	10	4.226422	0.003736	0.02679
27.3	100	63.9559	21476	34	0.010451	0.003736	0.02759
30.9	100	28.2297	9360	38	0.00443	0.003736	0.02684
12.2	100	42.5484	13410	42	0.001484	0.003736	0.02702
28.1	100	15.167	4424	32	0.019411	0.003892	0.02823
0.9	100	86.4726	35311	42	0.001948	0.003967	0.02875
4.3	100	64.888	23854	41	0.001969	0.003967	0.02896
8.6	100	51.0118	16650	20	0.400611	0.004049	0.02917
10	100	48.4464	17396	26	0.039457	0.004124	0.02978
0.3	15.136	140.5935	54188	25	0.051774	0.004192	0.03068
9.2	100	86.0083	32098	21	0.239751	0.004192	0.03066
13.2	100	29.0411	8782	25	0.086254	0.004192	0.03053
47.9	100	28.4348	9558	38	0.004582	0.004266	0.03082
6.1	100	75.3261	27753	19	0.356023	0.004266	0.03096
11.2	100	65.0011	21883	7	5.690342	0.004581	0.03243
21.8	100	52.222	19525	31	0.02764	0.004643	0.03271
16.3	100	60.6649	22232	30	0.02877	0.004643	0.03275
0	100	133.765	52164	26	0.078683	0.005325	0.03677
5.8	100	54.7821	21826	33	0.023721	0.005325	0.03714
5.2	100	60.8432	21921	25	0.149431	0.005478	0.03803
8	100	105.5827	39145	16	0.685319	0.005478	0.03775
56.8	100	22.6155	7140	14	1.516766	0.005618	0.03883
53.3	100	61.3033	20651	24	0.102806	0.00573	0.04136
18.6	100	30.2243	10595	32	0.018423	0.00573	0.03989
35.6	100	54.9905	20080	28	0.054372	0.005792	0.04246
4.3	100	108.396	44538	25	0.123153	0.005792	0.04165
0	100	131.8233	48928	18	0.356005	0.00599	0.04512
62.3	100	20.9505	6395	15	1.15836	0.00599	0.04532
56.6	25.2	12.9905	3514	34	0.01483	0.00599	0.04398
6.7	100	65.241	21972	16	0.83758	0.006069	0.04556
31.3	100	27.8292	9152	34	0.010727	0.006428	0.04667
0.7	100	92.0328	35141	26	0.074499	0.006497	0.04826
4.6	100	82.4575	28704	25	0.145766	0.006497	0.0482
24.7	100	55.9955	20482	29	0.044195	0.006872	0.04986
0.9	100	25.6499	7625	38	0.003352	0.006991	0.05108
1.5	100	73.1533	25043	16	1.00751	0.006991	0.05113
1	100	101.7878	38776	18	0.207229	0.008361	0.06276
14.3	100	29.0904	8797	36	0.007078	0.0085	0.06326
16.8	100	27.8143	9210	30	0.02613	0.00861	0.06494
8.6	100	93.7085	35861	15	1.416968	0.00861	0.06521
23.2	100	35.4991	12630	17	0.439393	0.008997	0.06881
3.4	100	96.9093	36296	17	0.817572	0.009514	0.07616
1.3	100	39.2194	15131	29	0.023047	0.009818	0.07924
0.8	100	97.3501	34669	26	0.110113	0.009858	0.08095
6.3	100	44.2689	15775	32	0.014163	0.009858	0.08013
83.8	100	47.9199	15425	39	0.004006	0	2.34E-05
41.8	100	61.9521	22726	50	0.000412	0	1.38E-05
176.2	100	29.7766	9940	27	0.036387	0	0.000385
87.5	58.906	29.5153	9844	64	8.24E-06	0	4.33E-05
204.6	1.479	29.1783	9715	40	0.002451	0	0.00037

16.9	100	76.8309	27070	38	0.003847	0	2.59E-06
104.7	100	30.1225	10551	31	0.017966	0	0.000834
26.9	100	52.428	17085	34	0.011764	0	0.00071
46.1	36.558	105.3527	37843	71	2.19E-06	0	3.77E-06
54.7	21.426	105.3515	37842	59	3.2E-05	0	1.31E-05
0.8	100	112.23	43158	47	0.000654	0	0.001119
12.5	100	103.7631	39632	54	0.000105	0	0.000141
10	100	104.1645	39795	51	0.000205	0	0.000382
22.5	46.021	76.936	28955	51	0.000244	0	0.000609
39.2	49.782	76.9442	28959	73	1.53E-06	0	3.82E-07
13.7	100	79.2378	29922	70	3.33E-06	0	7.58E-06
31.5	9.794	60.1176	24082	56	0.000103	0	1.67E-06
8	100	65.0286	26215	46	0.000926	0	0.000326
24	48.09	60.4262	24228	81	3.08E-07	0	7.23E-09
67.3	39.152	60.6296	24322	61	2.92E-05	0	8.86E-07
15	100	60.9354	24456	73	1.8E-06	0	1.54E-07
13.5	100	62.6598	25213	48	0.000603	0	3.09E-05
13.5	100	63.4511	25546	33	0.019397	0	0.001098
6.1	100	63.9631	25762	38	0.00639	0	0.000561
11.9	100	61.6377	24771	52	0.000224	0	1.74E-05
65.2	100	61.1345	24549	64	1.56E-05	0	9E-07
16.9	100	62.1464	24997	56	0.000104	0	3.72E-06
42.8	13.883	80.7425	28783	60	4.23E-05	0	0.000264
18.2	13.002	28.2075	10570	39	0.006137	0	0.000256
40.7	59.936	28.2619	10595	34	0.017458	0	5.54E-05
141.2	100	26.9988	10085	41	0.001384	0	0.000271
9	100	80.6862	28027	43	0.000528	0.000141	0.001599
7.2	100	70.2742	23933	63	2.17E-05	0.000273	0.001951
0.8	100	123.3889	46046	38	0.004607	0.000525	0.00229
7.5	100	59.9177	23999	39	0.005046	0.000525	0.002238
6	100	105.8523	38045	37	0.004907	0.00065	0.002753
33.2	100	54.5066	19392	42	0.002465	0.000758	0.003006
54	6.598	54.0023	19187	39	0.00479	0.000951	0.004348
67.9	8.063	55.0972	18100	41	0.003356	0.000951	0.004289
2.2	100	61.4478	22527	30	0.037149	0.000951	0.003709
69.3	50.212	80.8002	28808	27	0.083917	0.001022	0.005243
18.8	100	68.8243	23710	62	2.76E-05	0.00121	0.006489
84.9	100	30.7926	9388	16	0.489291	0.001308	0.007109
7.5	100	104.8459	37623	29	0.03327	0.001758	0.009312
35.1	100	80.2994	28596	26	0.108803	0.002012	0.01063
24.9	100	59.2607	19836	27	0.120416	0.002079	0.01231
63.1	100	29.6178	10345	17	0.352831	0.002079	0.01161
25.5	100	73.5652	27273	47	0.00047	0.002079	0.01138
48.4	100	30.2916	9209	21	0.168061	0.002257	0.01259
24.4	100	29.9415	9074	27	0.037779	0.002376	0.01445
6.1	100	75.142	28195	38	0.003263	0.002376	0.01422
90.3	100	80.6072	30228	37	0.007019	0.002376	0.01476
12.1	100	19.6926	6510	49	0.000239	0.002376	0.01521
85.6	79.972	54.0554	19208	26	0.096335	0.002777	0.01785
6.3	100	70.3953	23979	36	0.010075	0.002777	0.0179
13.9	100	54.5896	17903	30	0.03623	0.002777	0.01812
14	100	55.008	19593	32	0.024811	0.003096	0.02013

5.6	100	47.9353	15431	30	0.029761	0.003674	0.02634
14.5	100	53.4978	18986	25	0.109846	0.004124	0.0295
31	100	75.8602	28505	20	0.284486	0.004424	0.03175
32.9	100	60.8055	20255	15	1.707911	0.005325	0.03727
163.2	2.232	80.1062	30007	40	0.002613	0.009858	0.08064
47.6	100	76.8754	27087	39	0.004285	0	0.000311
19.6	22.619	76.6704	27003	66	9.95E-06	0	6.4E-05
16.1	100	56.2003	20563	44	0.001742	0	0.00058
51.9	21.696	55.4247	20254	82	2.53E-07	0	2.96E-08
11	100	56.7029	20759	59	5.66E-05	0	2.6E-05
11	100	55.2124	20173	51	0.000354	0	0.000237
12.7	100	78.1928	27040	35	0.012459	0	0.001405
14.4	100	65.6113	23851	42	0.002258	0	0.000265
50.5	100	65.1101	23656	35	0.013226	0	0.00016
26.7	100	42.7484	14642	51	0.000187	0	0.000367
29.7	100	43.364	16892	56	2.72E-05	0	0.001211
30.9	100	36.8472	12903	45	0.000212	0	0.000556
12.8	100	52.8489	21002	54	0.000179	0	0.001206
28.7	100	51.79	20537	35	0.012749	0	0.000155
16.6	100	52.3398	20774	66	1.08E-05	0	8.87E-05
16.2	100	51.8301	20555	61	3.34E-05	0	0.000185
10.4	94.931	53.3526	21225	49	0.000609	0.000141	0.001565
10.2	100	52.2993	20756	34	0.017672	0.000273	0.001838
38.5	100	52.8003	20982	29	0.050402	0.000758	0.003159
12.6	100	53.9362	19161	28	0.061152	0.000951	0.003629
183.3	22.091	43.8666	17103	63	5.02E-06	0.001022	0.004811
3.8	100	58.0078	21234	53	4.68E-05	0.003674	0.02645
15.9	100	50.7685	20092	21	0.33301	0.006795	0.04974
19.8	62.074	62.4234	22910	61	3.84E-05	0	7.87E-06
11.5	100	61.9195	22713	52	0.000283	0	4.88E-05
47.6	100	76.8754	27087	39	0.004285	0	0.000311
19.6	22.619	76.6704	27003	66	9.95E-06	0	6.4E-05
19.8	100	129.5368	47724	58	6.13E-05	0	1.05E-07
12.7	100	78.1928	27040	35	0.012459	0	0.001405
14.4	100	65.6113	23851	42	0.002258	0	0.000265
50.5	100	65.1101	23656	35	0.013226	0	0.00016
9.9	100	112.3147	41919	26	0.090747	0	3.86E-06
12.8	100	52.8489	21002	54	0.000179	0	0.001206
28.7	100	51.79	20537	35	0.012749	0	0.000155
16.6	100	52.3398	20774	66	1.08E-05	0	8.87E-05
16.2	100	51.8301	20555	61	3.34E-05	0	0.000185
10.4	94.931	53.3526	21225	49	0.000609	0.000141	0.001565
10.2	100	52.2993	20756	34	0.017672	0.000273	0.001838
5	100	62.4348	22914	43	0.002401	0.000525	0.002404
38.5	100	52.8003	20982	29	0.050402	0.000758	0.003159
127.8	100	42.4985	16516	49	0.000197	0.000758	0.00319
84.9	100	43.0023	16740	48	0.000209	0.001114	0.005931
1.7	100	112.2493	41888	16	0.999812	0.001758	0.009118
3.8	100	58.0078	21234	53	4.68E-05	0.003674	0.02645
5	100	129.0358	47516	15	1.060492	0.003967	0.02868
15.9	100	50.7685	20092	21	0.33301	0.006795	0.04974
22.9	28.841	104.4696	37470	72	2.98E-06	0	1.81E-08

5.1	100	103.9647	37272	38	0.007638	0	0.000567
6.5	100	104.9717	37673	52	0.000296	0	2.62E-05
31.7	9.257	141.2702	54957	66	6.91E-06	0	0.000479
58.7	83.044	57.3447	20768	76	5.07E-07	0	1.13E-05
59.7	41.802	57.3428	20767	47	0.00034	0.000951	0.003695
10.6	100	56.8346	20572	11	1.194285	0.009764	0.0784
5.9	100	112.7956	43892	74	1.23E-06	0	2.77E-07
12.5	100	112.7736	43882	54	0.000131	0	2.85E-05
4.5	100	94.6246	36255	25	0.093227	0.000525	0.002289
9.9	100	79.7727	29599	37	0.007344	0.000951	0.004017
7.9	100	13.368	3619	32	0.004778	0.002376	0.01452
28.9	100	70.6141	26128	48	0.000477	0	8.39E-05
38	100	70.6283	26133	64	1.2E-05	0	9.39E-06
5.3	100	51.4773	18696	36	0.011539	0.000525	0.002294
261.6	46.28	21.8078	6720	26	0.07192	0.001308	0.007055
11.5	100	51.5024	18705	22	0.281107	0.002257	0.0129
97.6	100	35.8417	11969	37	0.002362	0	0.000183
12.8	100	105.6297	40427	55	4.49E-05	0	6.03E-06
1.7	100	106.1336	40611	40	0.0017	0	0.001089
19.2	100	90.7859	32067	42	0.001977	0.000758	0.002991
27.8	100	29.4237	9810	30	0.009081	0	0.000133
11.5	100	63.5749	23162	54	9.45E-05	0	4.6E-05
3.6	100	88.5391	33200	32	0.023555	0.000525	0.002401
14.1	100	58.264	19324	50	0.00045	0	0.0006
27.5	100	117.6114	45870	53	0.000105	0	0.001307
51	100	58.4244	19382	24	0.155186	0.00065	0.002749
52.9	100	21.3431	7680	28	0.037843	0.002315	0.01357
4.5	100	117.1056	45662	31	0.013315	0.004803	0.03382
17.9	100	83.3873	29096	64	1.7E-05	0	5.26E-07
55.9	100	82.8847	28887	33	0.022685	0	0.000113
68.4	100	57.6834	19100	38	0.00548	0.000868	0.00358
57.6	100	57.1817	18901	28	0.055727	0.000951	0.00382
19.6	100	47.679	17106	29	0.043582	0.003674	0.02658
107.9	100	15.9979	4815	55	0.000106	0.000525	0.002384
28.5	100	60.0657	21587	24	0.139404	0.000951	0.004127
365.3	88.265	15.9601	4796	36	0.008971	0.001022	0.005551
81.7	95.302	64.8119	26118	41	0.001768	0.001758	0.009255
95.1	100	65.3198	26345	44	0.001045	0.002012	0.01083
210.7	100	15.8502	5016	55	0.000111	0.002012	0.01077
320.3	6.942	15.8454	5013	36	0.008892	0.003178	0.02046
51.7	100	16.0092	4409	25	0.092658	0.007572	0.05456
4.1	100	125.8763	46905	45	0.001043	0	0.000516
4	100	125.3696	46724	53	0.000155	0	0.000144
142.5	100	55.1347	21979	39	0.00193	0	0.000488
23.5	100	55.9589	20917	35	0.011063	0.003254	0.0214
13.4	100	62.9015	23093	46	0.001236	0	0.000318
73	100	18.662	6086	19	0.577141	0	0.000657
0	100	109.3979	44927	22	0.255757	0.002079	0.01141
13.4	100	62.9015	23093	46	0.001236	0	0.000318
73	100	18.662	6086	19	0.577141	0	0.000657
0	100	109.3979	44927	22	0.255757	0.002079	0.01141
3.8	100	127.6068	52375	45	0.000538	0	0.000346

36.7	100	28.0045	8439	29	0.037882	0.000525	0.002438
0.8	100	102.3787	39228	25	0.036952	0.001308	0.007067
25.6	100	65.6895	24340	27	0.049312	0	0.000238
3.4	100	89.0439	33983	35	0.012436	0.000141	0.001807
11.3	100	27.3472	8248	48	9.39E-05	0.003967	0.02877
35.2	100	59.9049	19914	41	0.002605	0	0.00012
13.2	100	64.0816	21753	22	0.285968	0.002079	0.01174
21.2	100	30.4135	10674	31	0.01148	0.002376	0.01526
11.9	100	61.3362	22542	39	0.00578	0.000758	0.003126
24.5	100	89.7989	32499	51	8.37E-05	0.000758	0.003163
21.6	100	87.0039	31377	42	0.001444	0.000951	0.004101
7.1	100	97.159	36407	44	0.001925	0	0.00063
40.3	100	46.4351	16183	43	0.000541	0.001498	0.007678
3.5	100	99.9078	35663	20	0.255139	0.004643	0.03359
14.5	100	61.2807	20437	55	7.22E-05	0.000273	0.001891
3.3	100	103.5879	37130	29	0.048017	0.001758	0.009674
7.3	100	81.0321	30098	21	0.292307	0.002012	0.01114
1.4	100	93.8515	33306	42	0.003124	0.000525	0.002379
8.4	100	76.8767	28506	25	0.10949	0.001022	0.004974
68.5	100	52.9124	19247	24	0.127393	0.004192	0.0301
16.1	100	62.5352	23029	27	0.042997	0	0.001487
0	100	109.3979	44927	22	0.255757	0.002079	0.01141
17.3	100	42.1698	13276	31	0.018206	0.003892	0.0283
16.1	100	62.5352	23029	27	0.042997	0	0.001487
0	100	109.3979	44927	22	0.255757	0.002079	0.01141
17.3	100	42.1698	13276	31	0.018206	0.003892	0.0283
16.1	100	62.5352	23029	27	0.042997	0	0.001487
0	100	109.3979	44927	22	0.255757	0.002079	0.01141
17.3	100	42.1698	13276	31	0.018206	0.003892	0.0283
234.9	100	46.6629	16237	7	3.772812	0.001114	0.006152
15.9	100	52.2947	19014	44	0.000674	0.002315	0.01318
34.8	100	42.0243	16308	30	0.025828	0.004581	0.03248
39.3	100	42.1858	14559	31	0.026874	0.001576	0.008164
8.9	100	95.6035	34898	26	0.095888	0.00337	0.02267
5.7	100	84.426	30288	20	0.478467	0.003674	0.02512
5.4	100	17.7369	5142	58	2.47E-05	0.001576	0.008311
3.1	100	85.3497	31744	18	0.579149	0.004344	0.03159
12.3	100	57.2791	20739	37	0.004633	0.004643	0.03294
4	100	89.2679	33309	50	0.000365	0	0.000292
10.2	100	89.2582	33305	50	0.000341	0	0.000111
32.7	9.911	88.2598	32920	73	1.7E-06	0	2.7E-07
8.1	100	88.7576	33115	51	0.000317	0	0.000428
40.2	5.167	88.254	32917	75	1.1E-06	0	2.22E-07
28.1	100	51.1727	16713	21	0.342394	0	0.00128
5.1	100	83.0203	30853	28	0.057249	0	0.000185
18.4	100	87.7462	32706	56	9.99E-05	0	4.01E-06
31.3	1.662	68.5723	25304	68	6.49E-06	0	6.19E-07
29.6	58.28	68.6392	25332	91	2.73E-08	0	6.23E-10
9.4	100	68.7342	25374	44	0.001599	0	0.001198
6.3	100	69.5855	25716	41	0.002806	0	0.000797
25.7	100	69.0773	25515	65	1.33E-05	0	1.53E-07
49.3	43.781	63.7359	23418	46	0.000922	0	0.00108

46.9	100	57.6402	19219	34	0.014759	0	0.000641
4.1	100	64.3835	23662	57	5.05E-05	0	0.000423
77.6	17.356	64.239	23609	51	0.00025	0	0.001127
3.8	100	82.1746	30536	30	0.04226	0	0.00028
9.3	100	82.0119	30476	25	0.125128	0	6.58E-05
9.6	100	81.6708	30348	27	0.084903	0	0.000145
8.3	100	81.5063	30286	27	0.073003	0	0.000182
10.1	100	82.514	30664	34	0.014137	0	2.67E-05
7.6	100	55.121	18211	48	0.000783	0	8.29E-05
0.6	100	55.1236	18212	34	0.017944	0	0.000289
17.6	100	75.0969	27841	27	0.082775	0	0.000712
4.5	100	38.4029	11951	61	2.45E-05	0	0.001428
6.9	100	24.343	7906	58	3.01E-05	0	0.000982
21.8	100	39.3984	12297	41	0.002884	0	0.00143
38.4	100	23.0512	7414	40	0.003138	0	0.00093
14.7	100	29.8338	9961	67	7.81E-06	0	0.000122
33.1	100	35.4424	12046	38	0.004392	0	0.000236
11.5	100	45.9594	14674	54	0.00016	0	6.7E-05
11.5	100	45.9594	14674	54	0.00016	0	6.7E-05
20.2	100	46.4667	14867	65	1.17E-05	0	2.29E-05
20.2	100	46.4667	14867	65	1.17E-05	0	2.29E-05
9	100	119.4643	44656	57	4E-05	0	1.08E-05
32	100	15.1434	4255	30	0.034953	0	0.00116
36.4	100	14.8534	4147	33	0.015535	0	0.001312
1.8	100	132.4457	49151	43	0.001043	0	9.68E-05
23.5	35.082	131.9448	48972	53	9.9E-05	0	1.48E-08
0	100	130.3155	48421	29	0.015557	0	0.000455
6.1	100	108.8976	44732	63	2.03E-05	0	1.88E-05
5.3	100	108.7816	44688	54	0.000178	0	3.86E-05
5.9	100	109.2922	44884	57	8.93E-05	0	2.75E-05
8.2	100	109.4081	44932	79	5.35E-07	0	3.08E-08
20.6	100	110.3017	45328	41	0.003104	0	0.000444
5.4	100	109.9185	45151	70	4.03E-06	0	1.31E-06
24.3	100	109.7951	45095	54	0.000186	0	5.1E-06
3.2	100	102.4165	42057	42	0.002612	0	0.000411
6.8	12.361	101.9126	41863	67	8.38E-06	0	5.19E-06
7	85.262	101.5135	41704	65	1.24E-05	0	2.72E-06
9.3	3.983	101.4086	41662	75	1.23E-06	0	7.99E-07
22.2	100	43.3436	15514	35	0.009998	0	0.001017
17.6	100	43.1498	15436	77	7.29E-07	0	9.73E-07
23.1	100	59.1549	19798	37	0.007404	0	0.000254
84.1	4.349	60.1605	22041	51	0.000238	0	0.001305
135	11.242	58.1428	19424	48	0.000632	0	0.000298
53.1	100	58.645	19610	37	0.008415	0	0.000548
21.2	100	51.4576	18688	35	0.01139	0	0.000856
148.9	4.277	60.4305	20307	59	3.53E-05	0	4.71E-05
47	57.244	60.4589	20318	85	7.56E-08	0	7.63E-07
21.2	100	51.4576	18688	35	0.01139	0	0.000856
85.2	100	59.9213	20092	45	0.000782	0	8.08E-05
2.5	100	69.1152	23831	52	0.000221	0	7.54E-05
12	100	69.7858	24100	46	0.000868	0	1.33E-06
35.6	100	14.6067	4178	47	0.000648	0	0.001371

114.9	100	14.5851	4169	45	0.000957	0	0.000106
3.7	100	140.6238	57525	85	1.46E-07	0	1.47E-09
101.4	100	52.8074	17229	41	0.003715	0	6.14E-05
9.5	100	65.7441	22168	47	0.000832	0	4.49E-07
2.6	29.228	62.7411	20996	57	8.74E-05	0	1.45E-08
4.3	86.101	62.261	20810	56	0.000126	0	1.91E-09
2.2	17.673	62.2385	20800	55	0.000158	0	1.11E-08
2.4	100	62.7664	21006	62	2.99E-05	0	1.03E-09
61	7.949	64.4971	21692	42	0.001872	0	5.86E-07
61.6	100	64.4036	21656	28	0.054819	0	0.000122
0.3	100	63.2738	21208	48	0.000685	0	8.97E-06
13.8	100	63.2467	21197	47	0.000872	0	2.33E-07
91.8	36.913	27.7745	8361	61	2.54E-05	0	6.43E-05
90.4	36.259	27.4831	8265	44	0.001116	0	0.00114
81.8	2.313	27.2708	8193	58	5.54E-05	0	0.000169
67.9	94.792	140.8249	55398	83	2.48E-07	0	9.42E-10
31.9	71.454	140.8228	55397	70	4.35E-06	0	1.64E-09
40.3	100	140.767	55367	111	2.67E-10	0	3.12E-14
9.8	21.931	140.764	55365	102	2.03E-09	0	1.67E-11
23.9	100	15.2336	4161	35	0.009444	0	0.000265
10.5	100	36.2184	11223	29	0.031967	0	0.00066
70.2	25.217	97.3035	34650	65	9.96E-06	0	0.000237
7.9	100	89.41	31524	69	4.46E-06	0	5.63E-06
81.6	45.86	88.9059	31315	18	0.50117	0	4.47E-05
6.2	100	93.3989	33136	50	0.0003	0	0.000187
1.3	100	107.5418	38773	39	0.004615	0	0.000353
71	21.14	88.7522	31248	46	0.000913	0	1.78E-06
57	19.079	88.7406	31242	73	1.68E-06	0	2.44E-08
0.4	100	122.3613	44907	33	0.017932	0	2.38E-05
0	100	77.461	26758	37	0.007894	0	4.22E-05
40.3	98.337	77.2925	26698	30	0.036704	0	1.26E-06
0.9	100	76.9597	26568	58	6.44E-05	0	3.17E-08
2.7	33.561	76.7827	26502	52	0.000249	0	8.53E-09
7.2	100	122.012	44770	67	8.67E-06	0	5.41E-08
14	100	121.5059	44563	67	7.16E-06	0	2.01E-10
4.7	100	77.7994	26890	50	0.000388	0	2.32E-06
17.6	57.805	121.8591	44705	46	0.001035	0	1.31E-10
83.3	100	73.2864	25097	33	0.010149	0	0.000423
9.7	100	73.2003	25063	51	0.000313	0	1.03E-06
84.8	11.655	73.1817	25056	51	0.000162	0	6.44E-05
20.2	19.658	73.025	24994	68	6.72E-06	0	1.35E-09
17.5	100	72.7833	24890	44	0.000971	0	0.00061
11.8	100	72.6715	24849	38	0.003195	0	0.000192
12.6	100	72.6106	24825	20	0.460521	0	0.000975
1.6	100	72.5145	24786	32	0.029017	0	0.00059
22.8	100	73.338	25120	38	0.005127	0	0.000671
19.2	100	73.3893	25141	47	0.000678	0	9.08E-05
40.2	100	74.6971	25688	50	0.00034	0	0.000697
22.4	100	73.8929	25361	61	2.7E-05	0	9.8E-06
45.3	25.014	73.8421	25338	56	7.89E-05	0	2.84E-05
77.7	58.628	73.6921	25276	47	0.000343	0	3.77E-05
7	100	73.5365	25204	59	5.98E-05	0	7.6E-07

9.5	45.774	121.3538	44498	70	3.79E-06	0	2.63E-12
20.7	100	88.3457	31076	56	7.92E-05	0	2.72E-05
3.3	100	88.3158	31063	70	3.05E-06	0	1E-05
24.6	8.961	88.2375	31032	69	4.47E-06	0	1.08E-07
39.7	83.577	87.8389	30874	60	3.73E-05	0	4.86E-07
4.1	100	87.8135	30864	53	0.000175	0	8.15E-05
40.5	8.926	87.734	30829	70	3.92E-06	0	6.55E-08
12	100	87.2234	30620	51	0.00029	0	3.88E-05
7.5	100	82.965	28922	49	0.000515	0	0.000184
28.1	21.104	141.219	52667	103	1.97E-09	0	9.18E-13
24.5	25.115	141.2199	52668	85	1.22E-07	0	1.58E-09
5.4	100	72.2036	26489	54	0.00016	0	3.66E-06
29.3	100	15.0902	4200	32	0.018934	0	0.001467
41	39.332	75.7345	27926	79	3.14E-07	0	1.13E-06
31.8	60.692	74.1639	27309	47	0.000772	0	0.00134
53.1	39.716	73.6574	27114	48	0.000568	0	0.000118
15	100	140.8376	52461	100	3.33E-09	0	1.18E-11
14.4	100	140.8508	52467	100	3.17E-09	0	3.54E-12
8.7	100	86.6666	32376	58	4.49E-05	0	1.55E-05
16.6	17.954	140.8955	52491	114	1.51E-10	0	1.99E-13
13.7	53.167	140.782	52430	96	1.05E-08	0	4.32E-15
10.7	100	140.812	52447	61	2.6E-05	0	4.74E-08
9.6	100	141.0493	52573	86	7.35E-08	0	9.94E-09
29.4	100	83.6126	31114	39	0.004503	0	9.91E-05
4.2	100	140.9086	52498	112	2.33E-10	0	2.96E-10
14.1	100	140.9108	52499	93	1.81E-08	0	7.19E-10
5.9	89.163	69.8371	25536	49	0.000269	0	4.38E-05
20	100	54.2322	19276	41	0.003124	0	2.12E-05
15.6	100	54.7428	19488	39	0.004963	0	0.00039
20.2	100	26.8409	8079	42	0.00194	0	0.000292
50.9	100	49.2511	17310	63	1.29E-05	0	0.001194
61.4	86.948	51.2579	18094	88	7.3E-08	0	1.62E-09
37.9	5.492	50.751	17900	98	6.7E-09	0	1.06E-09
32	6.798	50.4588	17783	81	3.37E-07	0	7.65E-09
31.7	100	15.5013	4347	45	0.001132	0	0.00056
12.9	100	66.7191	24310	30	0.03666	0	0.000186
13.3	100	17.6396	5107	31	0.038553	0	0.000449
80	100	140.4503	55184	117	1.79E-11	0	4.57E-10
28.8	33.859	140.4313	55174	92	6.54E-09	0	3.8E-10
4.3	77.959	136.9296	53583	84	1.06E-07	0	4.48E-09
6.8	18.544	136.8392	53546	96	6.89E-09	0	1.17E-09
3.8	100	92.3529	34757	37	0.005719	0	0.001518
31.6	47.186	91.3458	34343	77	5.65E-07	0	2.11E-08
8	100	90.6739	34092	46	0.00076	0	5.77E-05
17.6	35.41	91.8496	34551	81	2.65E-07	0	8.98E-09
6	15.115	105.9902	40583	64	5.25E-06	0	1.72E-05
31.4	9.136	105.9557	40566	84	5.47E-08	0	3.26E-07
3.4	100	105.4805	40349	61	1.12E-05	0	3.97E-05
23.2	100	109.8544	40878	84	1.35E-07	0	7.11E-11
67.1	2.788	89.1158	33913	58	6.36E-05	0	6.91E-05
24.1	22.964	113.7589	42540	79	5.58E-07	0	1.02E-08
4.8	23.623	113.773	42546	70	4.57E-06	0	6.44E-07

2.5	100	96.2288	36939	36	0.012345	0	0.000803
20	92.912	95.72	36730	48	0.000681	0	0.000201
12.8	92.397	95.3405	36570	61	3.21E-05	0	2.74E-06
21.7	58.098	95.2157	36514	48	0.000715	0	6.71E-05
0.4	100	111.5192	41585	96	1.25E-08	0	4.37E-07
6.1	100	96.7351	37142	38	0.006612	0	0.000427
13.7	100	94.8343	36346	49	0.000542	0	0.000166
0.6	100	93.0413	35567	47	0.000636	0	0.001342
10	49.412	94.7118	36292	44	0.001673	0	4.04E-05
10.5	100	94.2086	36078	42	0.002927	0	0.000223
172.1	3.96	140.5927	54187	66	4.58E-06	0	3.7E-06
2.4	100	141.2964	54969	31	0.037107	0	0.000972
116.1	100	13.6894	3914	34	0.013462	0	0.000401
33.2	30.596	102.8952	38052	70	4.45E-06	0	3.77E-06
3.8	100	102.945	38072	45	0.00162	0	0.000409
158.8	48.697	140.6731	54233	82	2.79E-07	0	2.39E-07
0	100	134.2669	52336	83	1.43E-07	0	1.69E-06
50	88.716	140.3717	54077	59	4.14E-05	0	1.31E-10
23.1	100	106.0869	39346	31	0.025658	0	3.04E-05
20.4	24.272	131.8755	50352	61	9.79E-06	0	3.44E-06
2.4	100	131.7344	50295	29	0.021095	0	0.00037
14.1	100	131.6865	50276	32	0.010348	0	5.66E-05
35.3	100	56.0867	20017	42	0.002163	0	0.000466
18.3	100	55.6399	19839	83	1.58E-07	0	5.33E-07
115.7	15.021	55.5809	19816	53	0.000179	0	4.01E-05
10.2	100	118.6687	44672	30	0.024394	0	7.31E-07
31.3	100	65.8261	24019	37	0.007226	0	7.79E-05
1.2	100	115.5712	43327	38	0.008063	0	0.000513
34.3	41.416	66.3411	24232	87	6.31E-08	0	1.04E-06
85.1	3.804	66.3295	24227	40	0.003611	0	0.000572
47.9	67.416	118.6178	44648	65	6.55E-06	0	6.01E-10
31.4	100	66.8323	24445	42	0.002197	0	4.43E-05
14.8	100	69.5341	25561	41	0.002798	0	9.15E-05
6.9	100	102.4402	37855	61	3.75E-05	0	0.000131
104.4	100	70.5957	28621	51	0.000339	0	8.28E-05
7.5	100	44.7688	15971	51	0.000164	0	0.00095
32.7	100	49.5078	19545	36	0.010699	0	0.000364
37.5	100	51.3075	18517	47	0.000273	0	0.000473
67.8	8.102	50.7373	18286	46	0.000346	0	0.000732
28.6	36.117	141.0307	54449	57	5.05E-05	0	0.000335
1.2	93.779	78.7188	27907	15	1.441885	0	1.12E-05
48.2	37.822	48.5438	17432	48	0.000252	0	0.000265
53.3	100	49.0478	17625	52	9.6E-05	0	9.06E-05
43	2.841	44.4835	17360	50	0.00044	0	6.17E-05
183.8	3.731	45.7493	17926	36	0.0089	0	0.00031
199.1	100	45.2421	17691	25	0.107395	0	2.5E-05
16.7	100	70.0835	28396	52	0.000254	0	0.001392
69.3	9.763	50.0654	18030	55	5.19E-05	0	0.000517
15.3	100	86.9561	32052	56	3.54E-05	0	0.000238
55	100	86.4548	31884	47	0.000338	0	0.000453
52.8	100	85.9505	31714	46	0.000376	0	0.000823
19.1	100	62.8898	25310	61	2.43E-05	0	0.000224

22.8	100	141.5457	54757	104	1.13E-09	0	5.24E-11
0.8	94.784	141.5358	54752	44	0.000857	0	0.000107
48.6	5.024	85.4972	30729	31	0.027214	0	9.03E-07
40.6	46.488	85.5514	30753	36	0.009587	0	8.5E-07
12.4	100	86.0002	30941	29	0.047697	0	4.7E-06
97.7	68.421	141.3667	54655	104	7.86E-10	0	9.21E-13
28.2	28.035	141.365	54654	77	3.58E-07	0	1.59E-10
8.7	49.164	56.4759	22547	45	0.001395	0	9.46E-05
59.1	56.71	141.3641	54653	61	2.07E-05	0	2.86E-11
32.2	27.704	55.971	22331	45	0.001404	0	0.000263
15.4	100	57.4837	22988	40	0.004929	0	0.000451
12	100	56.9805	22763	47	0.000826	0	9.79E-05
49.4	100	141.3746	54659	80	2.54E-07	0	2.66E-15
66.7	89.696	54.965	21914	36	0.011077	0	0.000423
63.2	35.493	55.4691	22120	47	0.000963	0	8.63E-05
186.9	100	55.4094	22092	75	1.36E-06	0	1.29E-05
29	6.483	29.8773	11246	67	8.03E-06	0	3.55E-05
37.3	5.899	140.8143	54315	98	5.94E-09	0	3.78E-16
44.7	100	140.7981	54306	65	1.03E-05	0	1.05E-10
93.8	9.067	31.7019	12009	55	0.000112	0	0.001177
329	23.386	140.7637	54285	50	0.000276	0	0.000596
2.4	100	140.7477	54275	63	9.58E-06	0	0.000346
11.2	8.762	140.7857	54299	91	2.69E-08	0	8.89E-13
37.9	100	39.4948	15245	33	0.018954	0	0.000892
359.7	25.892	140.9153	54380	144	1.57E-13	0	8.74E-16
26.8	16.464	140.8922	54365	80	6.63E-08	0	1.12E-09
75.8	4.214	140.8914	54364	77	1.12E-07	0	1.31E-11
2.6	100	131.3715	50145	38	0.001849	0.000141	0.001645
143	14.753	20.9613	6517	21	0.316589	0.000141	0.001624
241.2	100	15.1572	4420	25	0.10876	0.000141	0.001795
79.5	100	42.6934	15268	42	0.001954	0.000141	0.001766
180.1	9.349	12.9888	3512	35	0.01421	0.000141	0.001562
135.5	100	14.4527	4114	33	0.015796	0.000141	0.001755
5.7	100	94.3311	36131	36	0.012243	0.000141	0.001813
5.8	100	87.3363	30665	40	0.003688	0.000141	0.001617
92.4	9.298	49.5573	17827	46	0.00035	0.000273	0.001872
175.8	1.368	15.2833	4700	32	0.02222	0.000273	0.002096
8	100	87.0985	32559	33	0.011793	0.000273	0.002031
1.3	100	102.0194	41908	35	0.01246	0.000273	0.001849
123	6.27	55.4692	20273	49	0.000428	0.000273	0.002032
18	100	86.0611	30966	4	14.60519	0.000273	0.00203
12.6	100	88.8532	31293	35	0.010982	0.000525	0.002525
9.1	100	50.9512	18371	40	0.001641	0.000525	0.002376
4.4	100	92.9945	35547	38	0.005154	0.000525	0.002223
55.5	100	42.6591	15255	39	0.003971	0.000525	0.00228
35.2	100	30.184	11381	40	0.003689	0.000525	0.002257
10	100	80.8186	30321	38	0.005858	0.000525	0.00234
9.5	100	92.486	35325	28	0.047809	0.00065	0.002717
56.2	100	25.9858	8517	22	0.173172	0.00065	0.002753
1.5	100	130.2483	50770	32	0.012544	0.00065	0.0027
30.9	100	64.3464	23648	36	0.009021	0.00065	0.002661
6.6	100	95.8415	36781	41	0.003176	0.000758	0.003231

26.4	100	54.453	21682	18	0.636048	0.000758	0.002837
24.9	100	34.9889	12437	21	0.201893	0.000758	0.003208
1.4	100	25.3718	8739	49	0.00028	0.000758	0.002786
21.8	100	36.9918	12421	43	0.001681	0.000758	0.002814
0.7	100	68.6113	23616	30	0.036682	0.000758	0.003169
16.9	100	107.0417	39736	35	0.014632	0.000758	0.003046
20	100	50.2477	17699	20	0.439636	0.000758	0.002866
40.1	100	58.1853	19441	42	0.002178	0.000758	0.003197
3.3	100	72.4125	24746	32	0.025305	0.000868	0.003547
9	100	59.8076	21909	28	0.048458	0.000868	0.003453
14.2	100	87.9178	32927	54	0.00015	0.000868	0.003304
0	100	61.3458	22127	34	0.019248	0.000868	0.003345
7.9	100	49.9392	17979	38	0.002521	0.000951	0.00458
10	100	73.7936	25318	33	0.01012	0.000951	0.003884
37.6	100	26.769	8017	22	0.198996	0.000951	0.004389
151.3	100	28.4412	9169	21	0.274255	0.000951	0.004184
29.5	60.601	50.8013	17922	45	0.001265	0.000951	0.003781
8.9	100	96.8023	34454	38	0.004987	0.000951	0.00385
6.2	100	55.9974	22343	31	0.031363	0.000951	0.004134
23.4	100	23.3841	7544	36	0.008608	0.000951	0.003672
2.5	100	92.5365	35348	41	0.002789	0.000951	0.003737
20.8	100	44.9661	14298	19	0.592911	0.000951	0.004407
20.4	50.624	85.5502	30752	14	1.296896	0.001022	0.005598
6.5	100	68.066	25090	32	0.024082	0.001022	0.004993
90.3	100	38.3931	11947	41	0.003016	0.001022	0.005471
1.4	100	19.6052	6893	50	0.000478	0.001022	0.00572
2.1	100	21.9151	6486	58	1.68E-05	0.001022	0.005546
0.4	100	134.3722	52377	39	0.003568	0.001022	0.004713
21.5	100	64.0793	21525	15	1.415521	0.001114	0.005895
0	100	134.781	52522	32	0.019526	0.001114	0.006015
177.9	60.041	51.0796	16580	12	1.611266	0.001114	0.006232
38.2	88.529	97.3359	34663	35	0.009783	0.001114	0.006373
5.9	100	82.6757	30724	23	0.197078	0.001114	0.006186
1.3	100	88.7653	33118	32	0.023972	0.001114	0.006019
147.2	100	36.5873	12254	24	0.126205	0.001114	0.005914
5.5	100	56.714	18722	38	0.0034	0.00121	0.006567
33.8	100	21.5627	7263	45	0.000419	0.00121	0.006694
215.3	100	37.1717	12859	21	0.311047	0.00121	0.006606
75.5	100	21.2287	7631	45	0.000471	0.00121	0.006727
72	100	64.2058	23597	32	0.019505	0.00121	0.006836
49.9	100	27.2881	9435	21	0.243	0.00121	0.006795
71.9	100	20.4583	6326	15	1.37718	0.001308	0.006963
0.5	100	130.7487	50973	35	0.007438	0.00141	0.007362
0.4	100	131.6762	48876	28	0.038588	0.001498	0.007716
2.5	100	96.833	34467	35	0.008792	0.001498	0.007756
0.8	100	97.8545	34864	45	0.001426	0.001498	0.008047
254.9	88.162	29.6852	11172	27	0.086053	0.001498	0.007525
15.7	100	17.2362	4959	54	3.87E-05	0.001498	0.007704
0.8	100	108.9006	40900	31	0.037496	0.001576	0.008488
23	100	50.8572	18352	35	0.004509	0.001576	0.008377
5.2	100	23.4608	6987	25	0.095026	0.001576	0.00848
2.7	100	93.5444	35787	41	0.002885	0.001758	0.009304

6.8	100	83.1816	30912	10	4.180216	0.001758	0.009008
21.8	100	48.789	15780	19	0.459333	0.001758	0.009064
15.7	100	54.6071	17910	19	0.370237	0.001854	0.009877
85.3	100	55.2853	22039	41	0.003421	0.001854	0.009886
2.3	100	84.9915	30515	20	0.381069	0.001854	0.00974
9.4	100	87.535	33203	53	0.000114	0.001854	0.009884
18.1	100	69.9052	23791	34	0.009677	0.001945	0.009944
18.1	100	69.9052	23791	34	0.009677	0.001945	0.009944
18.3	100	73.2829	25095	36	0.004325	0.002012	0.01027
25.7	100	39.3319	14021	30	0.034473	0.002012	0.01105
24.9	60.269	46.2559	18148	28	0.055187	0.002012	0.01113
22	100	27.3098	9019	40	0.00291	0.002079	0.01236
4.3	100	60.9451	21966	16	1.164599	0.002079	0.01157
1.4	100	76.8884	28560	47	0.000106	0.002079	0.01179
8.5	100	68.2285	25155	22	0.239715	0.002079	0.01146
12.7	100	54.9115	18025	22	0.23456	0.002079	0.01171
160.6	100	21.7472	7848	49	0.000175	0.002079	0.01235
20.3	100	55.9748	20474	43	0.001514	0.002079	0.01214
158.7	100	48.3914	16964	49	0.000344	0.002079	0.01162
2.7	100	51.8746	16981	42	0.001511	0.002257	0.01254
0	100	137.3487	53753	20	0.265607	0.002257	0.01245
40.6	100	48.9467	17724	37	0.005065	0.002315	0.01353
53.4	100	37.1228	12838	39	0.004542	0.002315	0.01399
28.1	100	21.0003	6533	40	0.003814	0.002315	0.01396
17.9	100	14.5627	4339	42	0.002059	0.002315	0.01414
5.6	100	20.1403	5925	29	0.017399	0.002315	0.01331
15.7	100	54.1497	19245	31	0.027445	0.002315	0.01389
2.6	100	89.5486	33417	45	0.000499	0.002315	0.01337
13.5	100	29.6322	9894	42	0.001941	0.002376	0.0142
0.8	100	67.9922	24809	39	0.004051	0.002376	0.01426
0.4	100	118.9592	44468	26	0.048103	0.002376	0.01538
5.6	100	67.6383	22906	22	0.228133	0.002376	0.0149
0.4	100	103.4072	38920	24	0.148157	0.002543	0.01589
11.1	100	49.3995	17315	47	0.000202	0.002543	0.01627
5.3	100	55.4912	22131	29	0.059686	0.002719	0.0165
49.5	100	51.7941	18295	38	0.002531	0.002719	0.01643
3.3	100	73.039	27067	41	0.001198	0.002777	0.01764
7.1	100	49.4342	17774	33	0.007658	0.002777	0.01714
2.3	100	76.389	28377	45	0.000183	0.002777	0.01811
17.3	100	38.6792	12049	15	0.62586	0.002843	0.01912
4.2	100	81.1647	30152	16	0.96557	0.002843	0.01835
10	100	115.4404	43267	25	0.026486	0.002843	0.01882
24.3	100	29.3442	9909	34	0.012605	0.002843	0.0185
0.9	100	131.3227	48755	17	0.199802	0.00302	0.01957
19.8	100	15.6188	4443	38	0.005685	0.003096	0.01998
3.5	100	44.7733	17486	36	0.011362	0.003096	0.01975
5.9	100	32.5455	12362	45	0.000111	0.003178	0.02042
286.5	14.86	48.1105	16851	44	0.001151	0.003254	0.02108
18.1	100	15.508	4245	38	0.005123	0.00337	0.02365
16.8	100	51.4392	18589	46	0.000214	0.00337	0.02393
28.1	100	15.6606	4651	34	0.012852	0.00337	0.02427
7.4	100	59.9512	20104	31	0.02295	0.00337	0.02188

23.4	100	50.429	17771	24	0.149969	0.00337	0.0225
166.3	11.56	47.8893	16761	55	8.87E-05	0.00337	0.0224
93.4	100	21.7412	6815	12	2.278812	0.00337	0.02261
35.6	100	36.7923	12568	44	0.000909	0.00337	0.02225
122.2	100	27.9267	9366	38	0.005278	0.00337	0.0232
80.9	100	22.024	6927	10	4.226422	0.003736	0.02679
27.3	100	63.9559	21476	34	0.010451	0.003736	0.02759
30.9	100	28.2297	9360	38	0.00443	0.003736	0.02684
12.2	100	42.5484	13410	42	0.001484	0.003736	0.02702
28.1	100	15.167	4424	32	0.019411	0.003892	0.02823
0.9	100	86.4726	35311	42	0.001948	0.003967	0.02875
4.3	100	64.888	23854	41	0.001969	0.003967	0.02896
8.6	100	51.0118	16650	20	0.400611	0.004049	0.02917
10	100	48.4464	17396	26	0.039457	0.004124	0.02978
0.3	15.136	140.5935	54188	25	0.051774	0.004192	0.03068
9.2	100	86.0083	32098	21	0.239751	0.004192	0.03066
13.2	100	29.0411	8782	25	0.086254	0.004192	0.03053
47.9	100	28.4348	9558	38	0.004582	0.004266	0.03082
6.1	100	75.3261	27753	19	0.356023	0.004266	0.03096
11.2	100	65.0011	21883	7	5.690342	0.004581	0.03243
0	100	129.303	50374	27	0.033701	0.004643	0.0331
21.8	100	52.222	19525	31	0.02764	0.004643	0.03271
16.3	100	60.6649	22232	30	0.02877	0.004643	0.03275
0	100	133.765	52164	26	0.078683	0.005325	0.03677
5.8	100	54.7821	21826	33	0.023721	0.005325	0.03714
5.2	100	60.8432	21921	25	0.149431	0.005478	0.03803
8	100	105.5827	39145	16	0.685319	0.005478	0.03775
56.8	100	22.6155	7140	14	1.516766	0.005618	0.03883
53.3	100	61.3033	20651	24	0.102806	0.00573	0.04136
18.6	100	30.2243	10595	32	0.018423	0.00573	0.03989
35.6	100	54.9905	20080	28	0.054372	0.005792	0.04246
4.3	100	108.396	44538	25	0.123153	0.005792	0.04165
0	100	131.8233	48928	18	0.356005	0.00599	0.04512
62.3	100	20.9505	6395	15	1.15836	0.00599	0.04532
56.6	25.2	12.9905	3514	34	0.01483	0.00599	0.04398
6.7	100	65.241	21972	16	0.83758	0.006069	0.04556
31.3	100	27.8292	9152	34	0.010727	0.006428	0.04667
0.7	100	92.0328	35141	26	0.074499	0.006497	0.04826
4.6	100	82.4575	28704	25	0.145766	0.006497	0.0482
24.7	100	55.9955	20482	29	0.044195	0.006872	0.04986
0.9	100	25.6499	7625	38	0.003352	0.006991	0.05108
1.5	100	73.1533	25043	16	1.00751	0.006991	0.05113
1	100	101.7878	38776	18	0.207229	0.008361	0.06276
14.3	100	29.0904	8797	36	0.007078	0.0085	0.06326
16.8	100	27.8143	9210	30	0.02613	0.00861	0.06494
8.6	100	93.7085	35861	15	1.416968	0.00861	0.06521
23.2	100	35.4991	12630	17	0.439393	0.008997	0.06881
3.4	100	96.9093	36296	17	0.817572	0.009514	0.07616
1.3	100	39.2194	15131	29	0.023047	0.009818	0.07924
0.8	100	97.3501	34669	26	0.110113	0.009858	0.08095
6.3	100	44.2689	15775	32	0.014163	0.009858	0.08013
57.8	37.263	89.2607	33976	71	3.62E-06	0	8.08E-05

30.4	100	89.633	34142	53	0.000246	0	0.000189
42.6	29.447	89.5989	34126	65	1.33E-05	0	0.000118
19.9	100	56.6738	20256	62	2.69E-05	0	2.2E-05
49.9	100	56.6452	20246	47	0.000722	0	0.000339
24.2	100	56.1722	20052	53	0.000175	0	6.4E-05
9	100	83.142	31287	61	3.59E-05	0	0.001331
37.1	28.831	50.157	19823	59	5.6E-05	0	0.000425
11.5	100	70.4702	28564	65	5.58E-06	0	0.000123
24.3	100	71.3738	28955	53	0.000141	0	6.6E-05
137.9	22.594	141.0246	54444	102	2.62E-09	0	3.11E-12
74.5	90.01	141.0608	54469	109	6.11E-10	0	5.71E-13
30.8	19.887	71.9292	29206	79	3.93E-07	0	4.54E-07
17.8	100	71.4242	28978	78	4.89E-07	0	3.02E-06
38.1	10.32	71.8825	29184	54	0.000124	0.000273	0.001972
19.9	100	56.1431	20040	37	0.007258	0.001114	0.005996
17.2	100	89.0956	33903	41	0.003423	0.002012	0.01063
28.7	100	49.8388	19681	27	0.086952	0.002543	0.01606
11.3	100	50.7103	18298	33	0.019234	0.003736	0.02692
9	100	73.853	27369	21	0.252496	0.007499	0.05419
22	100	50.7665	18320	25	0.121288	0.007836	0.05657
7.4	100	51.1602	20260	29	0.050639	0.009655	0.07707
2.8	76.799	141.0573	54467	14	1.892211	0.00971	0.07786
8.6	100	140.4507	54115	76	1.14E-06	0	5.03E-08
70.4	52.549	140.4341	54107	82	2.63E-07	0	5.23E-11
28.4	86.347	141.1367	54513	82	2E-07	0	6.97E-10
68.3	81.802	140.9471	54401	114	1.33E-10	0	3.41E-13
40.5	100	34.646	13228	35	0.008069	0.00065	0.00267
16.8	100	39.2516	12243	45	0.000707	0	0.000593
101.2	61.59	34.1915	12144	64	1.45E-05	0	0.000118
163	100	53.8451	19143	29	0.028469	0	0.000681
5.2	100	72.9695	27286	67	2.02E-06	0	0.000204
45.8	30.486	77.7878	29020	54	8.98E-05	0	6.11E-05
18.1	71.832	54.1543	19256	52	0.000138	0.000273	0.001878
5.5	100	77.806	29028	34	0.010763	0.000868	0.003573
133.6	6.834	30.9259	10873	17	0.747409	0.000951	0.003663
75.8	100	31.4821	11095	18	0.573387	0.000951	0.004332
82.1	100	33.7971	11992	45	0.001205	0.001022	0.004661
116.6	100	13.7347	3937	24	0.076571	0.003533	0.02469
33.1	100	32.0171	11298	11	2.64482	0.004643	0.0332
50	100	30.4216	10678	11	2.555336	0.006926	0.05016
24.6	100	33.6871	11948	28	0.065591	0.006991	0.0514
19.3	100	34.6937	12324	31	0.031121	0.006991	0.05138
11	56.424	86.767	35439	58	7.77E-05	0	1.37E-06
8.4	100	87.2763	35665	55	0.000129	0	1.14E-05
31.8	69.667	55.1729	19655	55	0.000105	0	4.26E-05
9.8	100	63.9662	23188	65	7.16E-06	0	6.22E-08
15.2	100	86.6608	31228	55	0.000108	0	1.25E-05
8.8	100	51.4858	16829	37	0.002923	0.002079	0.01194
3.2	100	54.9919	19586	30	0.034558	0.002777	0.01805
19.6	100	38.7827	12087	36	0.006828	0	0.000467
24.1	100	75.0395	26309	52	0.00029	0	2.01E-06
14.6	100	15.1376	4214	47	0.000345	0	3.06E-05

2.1	100	98.4341	36096	54	0.000224	0	0.000163
2.9	100	98.9394	36320	34	0.020411	0	0.001018
0.3	100	14.5949	4029	40	0.000711	0.002543	0.01571
11.3	100	38.7294	12066	20	0.268735	0.003736	0.02776
15.7	100	14.5792	4023	25	0.016919	0.009514	0.07642
0.4	100	140.0325	51774	35	0.004915	0	0.000135
18.9	100	73.1251	26886	64	8.81E-06	0	4.41E-05
33.7	100	95.4321	36613	66	9.97E-06	0	6.57E-07
0.5	100	140.0066	51763	25	0.050426	0.000868	0.003268
7.5	100	52.8546	19225	34	0.017225	0.000951	0.004473
2.1	100	95.2564	36532	27	0.082233	0.001758	0.009293
17.1	100	55.9853	22337	20	0.421643	0.002843	0.01833
15.2	100	52.3501	19034	18	0.590287	0.003456	0.02431
40.3	100	48.4278	15625	32	0.02376	0	4.08E-05
13.9	100	17.5441	5557	48	0.000658	0	0.000541
18.7	100	17.4255	4896	49	0.000464	0	0.000153
35	100	41.6295	14759	41	0.003075	0	0.000548
0	100	14.2605	3916	59	1.87E-05	0.000273	0.001898
11.7	100	17.4375	4901	42	0.002232	0.000525	0.002574
157.1	100	21.3037	6754	25	0.044678	0.000525	0.002256
0	100	42.7366	13478	57	7.38E-05	0.000868	0.003433
49.1	100	17.4755	5227	30	0.036872	0.000951	0.004386
31.2	100	29.7739	11205	45	0.000589	0.002257	0.0124
16.2	100	51.1621	16612	18	0.56988	0.006222	0.04591
4.2	100	105.7324	40462	34	0.005639	0	0.00022
2.1	100	92.0457	33705	40	0.000147	0	0.00148
50	100	72.1026	26445	61	2.58E-06	0	1.62E-06
20.8	100	61.9011	22705	38	0.00577	0.001022	0.005062
62.4	100	61.6623	22606	20	0.418011	0.00121	0.006776
1.5	100	91.5401	33530	22	0.008538	0.004803	0.03388
0.3	100	105.7668	40475	24	0.041936	0.005325	0.03643
3.5	100	61.395	22508	19	0.432064	0.009764	0.07821
31.4	77.255	71.25	26382	62	6.52E-06	0	1.57E-06
14.6	100	70.7463	26188	42	0.000678	0	0.000274
71.9	100	67.0819	27078	50	0.000438	0	0.000176
14.8	100	76.5692	28272	52	0.000166	0.000407	0.002137
74.4	100	77.5421	26789	42	0.001583	0.001498	0.007876
125.8	71.582	78.0431	26982	43	0.001303	0.001576	0.008154
42.8	100	38.5268	14849	46	0.000664	0.002079	0.01136
9	100	68.7889	25129	42	0.001706	0.00337	0.02315
12.9	100	77.6029	26812	22	0.153289	0.009436	0.07121
43.4	83.507	140.9162	54769	81	6.17E-08	0	4.98E-05
64.9	100	109.5931	39341	80	1.07E-07	0	4.7E-07
11.6	100	70.9961	24210	27	0.08719	0.001022	0.005081
9.4	100	109.3417	39260	29	0.013345	0.001114	0.00643
180.8	82.736	44.6091	16014	43	0.00102	0.00382	0.02785
6.2	100	109.0915	39176	25	0.028134	0.00861	0.06513
30.4	100	62.3152	22519	45	0.001283	0	3.56E-05
12.2	100	59.9815	24025	47	0.000801	0.000273	0.001975
7.7	100	17.5011	5053	33	0.002071	0.000758	0.002872
45.6	100	40.6196	14248	30	0.029962	0.000951	0.004424
19	100	37.7807	12945	46	0.000698	0.001114	0.006217

10.9	100	50.8872	18466	24	0.188628	0.00382	0.02795
1.5	100	96.0684	35974	43	0.002132	0	0.001065
7.3	100	65.7674	24197	55	0.000129	0	0.000113
24.9	100	53.5892	19023	39	0.003032	0	0.00065
29.8	100	48.3666	17366	38	0.007343	0.002777	0.01701
0.3	100	65.7791	24202	27	0.088611	0.00337	0.02297
1.7	100	140.245	54465	52	9.04E-06	0	1.53E-05
135.4	100	27.4884	8817	36	0.006943	0	0.000114
20.2	100	40.4052	14267	42	0.002462	0.000758	0.003217
11	100	58.8799	23563	25	0.096411	0.001945	0.009926
11.7	100	128.1578	48741	23	0.056439	0	0.000871
12.8	100	68.1603	25125	44	0.001643	0.000525	0.002627
14.4	100	68.3014	25188	26	0.093625	0.000868	0.003359
18.1	100	84.5682	31503	31	0.016686	0.001758	0.009587
116.2	100	31.6683	11168	17	0.372747	0.002079	0.01221
5.9	100	124.3954	45735	47	0.000762	0	0.000213
2.6	100	103.4197	37066	32	0.024397	0.000868	0.003544
10.6	100	83.2404	29034	31	0.032923	0.001022	0.004602
56.8	100	22.5653	7230	20	0.037074	0.001677	0.008912
4	100	83.2136	29024	23	0.197793	0.008559	0.06453
14.3	100	76.6978	27015	39	0.004755	0	0.000919
7.8	100	80.5492	29635	42	0.000665	0	0.000857
57.2	100	38.7393	13612	26	0.056355	0.000951	0.004397
17.7	100	18.7837	5503	16	1.09259	0.00337	0.02164
13.2	100	38.6907	13592	20	0.216923	0.004803	0.03413
3.1	100	113.6338	42594	34	0.013336	0	0.000802
22.2	100	98.7279	36227	46	0.000349	0	0.000889
2.7	100	82.5601	28748	30	0.04363	0.000273	0.001906
7.5	100	113.9333	43488	35	0.010905	0.000525	0.002293
13.4	100	62.9015	23093	46	0.001236	0	0.000318
73	100	18.662	6086	19	0.577141	0	0.000657
0	100	109.3979	44927	22	0.255757	0.002079	0.01141
17.3	100	42.1698	13276	31	0.018206	0.003892	0.0283
13.4	100	62.9015	23093	46	0.001236	0	0.000318
73	100	18.662	6086	19	0.577141	0	0.000657
0	100	109.3979	44927	22	0.255757	0.002079	0.01141
17.3	100	42.1698	13276	31	0.018206	0.003892	0.0283
6.5	100	58.8689	21566	42	0.001536	0	0.000169
2.7	100	69.9363	28333	36	0.005275	0.000951	0.003755
26.1	100	49.6113	16110	17	0.844982	0.001945	0.01016
65.7	100	87.6353	30786	46	0.000875	0.002079	0.01221
212.6	100	44.0211	13963	25	0.024571	0	0.000388
38	100	121.742	46016	45	0.00147	0.000525	0.002649
20.8	100	37.7844	11732	18	0.583036	0.001945	0.01022
32.9	100	90.2737	32709	14	0.977648	0.00337	0.02168
1.5	100	101.9061	38343	44	0.001604	0	0.000877
144.8	100	16.454	5026	26	0.048057	0.001498	0.007717
2.2	100	17.4562	5035	41	0.003807	0.001945	0.01007
2.2	100	17.4562	5035	41	0.003807	0.001945	0.01007
41.5	100	21.7222	7326	36	0.009299	0.003736	0.02687
2.4	100	81.8546	30994	30	0.017722	0.000758	0.003064
8.4	100	86.605	30359	42	0.002538	0.000868	0.003516

21.1	100	24.3935	7994	16	0.370451	0.002543	0.01607
8.2	100	43.2427	15475	25	0.142027	0.003254	0.021
79.5	100	68.4802	25264	60	5.04E-05	0	1.07E-10
100.4	56.383	68.4195	25237	40	0.004552	0	1.66E-06
58.6	23.98	67.9752	25056	56	0.000123	0	1.51E-09
60.9	11.112	67.9068	25031	41	0.003336	0	1.01E-05
28.2	100	67.4712	24850	55	0.000134	0	6.54E-07
42.8	100	140.4495	51958	83	1.13E-07	0	1.14E-09
13.3	62.764	140.4167	51942	75	7.14E-07	0	2.1E-06
3.7	100	139.9244	51733	37	0.00466	0	0.000978
0.3	100	135.3865	55520	58	1.69E-05	0	0.000114
23	100	80.8845	32947	56	7.8E-05	0	1.82E-05
28.8	100	99.0496	40682	63	1.69E-05	0	2.01E-06
18.4	100	98.5372	40459	71	2.45E-06	0	1.05E-06
17.4	44.707	98.4929	40440	58	4.95E-05	0	3.81E-05
10.2	100	93.6122	33218	63	1.76E-05	0	2.38E-08
0.4	100	137.9499	54026	49	0.000258	0	0.000271
0.9	100	93.9485	35966	59	2.65E-05	0	0.001191
75.5	12.584	129.0238	49132	50	0.000398	0	0.000471
41.7	14.234	60.318	24179	57	6.09E-05	0	0.00013
27.8	100	82.4284	29476	36	0.009208	0	2.4E-06
24.3	11.135	81.8922	29264	64	1.61E-05	0	8.8E-08
25	37.196	82.3928	29463	64	1.6E-05	0	2.11E-08
17.7	43.374	81.9237	29277	60	3.65E-05	0	2.46E-08
122.9	61.841	39.9993	14111	24	0.166088	0	0.000339
1.3	100	93.4376	35735	54	0.000108	0.000141	0.001804
196.3	29.962	38.9866	13712	17	0.940458	0.000141	0.001571
146.8	17.387	39.4949	13918	21	0.382031	0.000141	0.00166
0.9	100	139.9017	51725	33	0.011463	0.000273	0.001857
79.5	100	40.5096	14307	21	0.378837	0.000273	0.002093
210.4	3.963	38.4845	13519	20	0.412561	0.000868	0.003477
20.4	100	97.3393	37398	48	0.000343	0.000951	0.004484
79.9	85.256	128.5138	48905	27	0.079526	0.001022	0.00496
9	100	67.8259	25003	27	0.083231	0.001022	0.005688
58	100	39.8452	13932	17	0.846862	0.00141	0.007295
25.9	36.139	60.349	24193	38	0.005377	0.001576	0.008277
9.5	100	67.3233	24793	17	0.977263	0.002012	0.01079
16.1	100	67.4355	24835	9	6.013092	0.002777	0.01695
27.1	100	39.5735	13948	28	0.062791	0.003178	0.0204
8	100	59.8402	23964	31	0.021465	0.003178	0.02063
2.3	100	112.0842	41816	43	0.001136	0.003254	0.02109
2.9	100	74.1449	25921	35	0.013227	0.00337	0.02246
4.1	100	96.8307	37181	30	0.023582	0.003892	0.02813
3.7	100	93.7172	33256	15	1.177691	0.006926	0.05099
27.1	100	39.5735	13948	28	0.062791	0.007836	0.05686
3.3	100	115.6314	44616	11	1.834149	0.007969	0.05837
2.5	100	62.6338	22660	13	2.091418	0.009818	0.07931
3	100	81.3816	29043	21	0.261973	0.009858	0.08003
51.9	21.696	55.4247	20254	82	2.53E-07	0	2.96E-08
11	100	55.2124	20173	51	0.000354	0	0.000237
98.4	100	73.0335	24997	58	6.58E-05	0	5.59E-06
26.1	79.917	73.4464	25165	71	3.03E-06	0	2.48E-06

55.6	100	73.5343	25203	59	4.72E-05	0	8.09E-06
10.4	100	79.8694	29637	54	0.000155	0	5.8E-05
17	100	80.3755	29829	82	2.35E-07	0	8.48E-08
14.4	100	65.6113	23851	42	0.002258	0	0.000265
50.5	100	65.1101	23656	35	0.013226	0	0.00016
15.6	100	125.6491	48848	56	0.000115	0	1.87E-06
3.6	100	112.7181	42092	29	0.039904	0	0.000706
2.6	100	113.1209	42257	31	0.022456	0	0.000979
11	100	112.614	42047	44	0.001156	0	4.05E-07
7.3	100	126.2449	49360	48	0.000699	0	2.21E-05
10.8	100	126.1125	49308	69	4.65E-06	0	5.99E-08
29.7	100	43.364	16892	56	2.72E-05	0	0.001211
12.8	100	52.8489	21002	54	0.000179	0	0.001206
28.7	100	51.79	20537	35	0.012749	0	0.000155
16.6	100	52.3398	20774	66	1.08E-05	0	8.87E-05
16.2	100	51.8301	20555	61	3.34E-05	0	0.000185
10.4	94.931	53.3526	21225	49	0.000609	0.000141	0.001565
1.7	100	113.6579	42493	23	0.188063	0.000273	0.001824
10.2	100	52.2993	20756	34	0.017672	0.000273	0.001838
38.5	100	52.8003	20982	29	0.050402	0.000758	0.003159
12.6	100	53.9362	19161	28	0.061152	0.000951	0.003629
1.3	100	126.2269	49087	28	0.071156	0.001022	0.005575
183.3	22.091	43.8666	17103	63	5.02E-06	0.001022	0.004811
8.8	100	68.5224	25020	48	0.000669	0.001114	0.006225
0.7	100	125.1428	48639	16	1.197664	0.001308	0.00705
0	100	126.032	46388	21	0.320874	0.001758	0.009541
1.9	100	124.5934	48413	35	0.013117	0.002079	0.01227
5.3	100	81.2286	28240	20	0.399655	0.002376	0.01498
3.8	100	58.0078	21234	53	4.68E-05	0.003674	0.02645
0.3	100	129.0433	50271	13	2.323874	0.004192	0.03013
3.3	100	72.942	24958	33	0.023669	0.005618	0.0387
1.7	100	125.7424	49147	16	1.109329	0.006371	0.04638
15.9	100	50.7685	20092	21	0.33301	0.006795	0.04974
7.2	100	90.4712	33795	43	0.001506	0	0.000724
40.2	100	104.5551	39338	91	2.8E-08	0	5.08E-09
22.8	62.046	104.5218	39325	62	2.45E-05	0	3.11E-05
1.7	100	104.0174	39145	42	0.002321	0	0.00097
28.6	100	56.2473	18648	36	0.008913	0	0.000126
112.4	47.58	55.2042	18244	45	0.001223	0	3.59E-06
18	100	55.2577	18267	58	6.03E-05	0	5.72E-05
118.2	100	55.7243	18448	38	0.005339	0	3.82E-06
20.2	100	62.5019	20901	40	0.003935	0	4.68E-05
202.6	15.699	140.7391	55350	99	3.54E-09	0	1.19E-11
35.3	4.802	140.7376	55348	89	3.13E-08	0	1.94E-10
49.1	42.897	77.0014	28454	39	0.006243	0	2.92E-06
114.4	77.865	76.9224	28418	61	3.62E-05	0	2.53E-10
13.5	100	76.4934	28243	29	0.062797	0	0.000497
12.9	100	140.833	52457	51	0.000218	0	3.01E-06
32.8	100	140.8142	52448	91	2.29E-08	0	1.33E-10
0.9	100	93.9485	35966	59	2.65E-05	0	0.001191
75.5	12.584	129.0238	49132	50	0.000398	0	0.000471
122.9	61.841	39.9993	14111	24	0.166088	0	0.000339

1.3	100	93.4376	35735	54	0.000108	0.000141	0.001804
196.3	29.962	38.9866	13712	17	0.940458	0.000141	0.001571
12	100	53.3878	17537	35	0.012853	0.000141	0.001702
146.8	17.387	39.4949	13918	21	0.382031	0.000141	0.00166
7.3	100	89.9657	33585	36	0.007575	0.000273	0.002053
79.5	100	40.5096	14307	21	0.378837	0.000273	0.002093
36.8	100	54.7014	18047	27	0.077531	0.000868	0.003589
210.4	3.963	38.4845	13519	20	0.412561	0.000868	0.003477
7	100	104.0518	39157	30	0.037829	0.000951	0.004298
79.9	85.256	128.5138	48905	27	0.079526	0.001022	0.00496
58	100	39.8452	13932	17	0.846862	0.00141	0.007295
5.3	100	89.4506	33378	21	0.240738	0.002376	0.01535
27.1	100	39.5735	13948	28	0.062791	0.003178	0.0204
2.3	100	112.0842	41816	43	0.001136	0.003254	0.02109
7.5	100	55.7631	18464	28	0.054801	0.00337	0.02288
7.5	100	63.982	25769	24	0.087334	0.004643	0.03304
6.3	100	54.7546	18069	28	0.063451	0.005031	0.03522
27.1	100	39.5735	13948	28	0.062791	0.007836	0.05686
28	82.083	54.6995	19957	83	2.02E-07	0	5.12E-10
78.4	100	54.321	19816	40	0.004599	0	1.35E-06
8.9	100	129.3131	47633	68	6.55E-06	0	8.67E-08
8.4	100	130.8226	48239	77	7.24E-07	0	1.09E-07
28	77.294	129.82	47844	74	1.39E-06	0	2.9E-08
21.6	97.887	130.3213	48044	78	6.61E-07	0	4.75E-08
25.8	100	87.5083	32744	51	0.000359	0	1.28E-05
18.2	100	87.0013	32518	37	0.00915	0	0.00066
2.3	100	127.0867	49433	39	0.005764	0	0.00079
14.9	100	139.7262	53767	68	7.26E-06	0	4.45E-08
38.5	100	139.7126	53760	90	3.97E-08	0	4.11E-10
12.3	100	139.2217	53532	33	0.020711	0	0.000315
7.9	100	76.5268	28787	86	8.11E-08	0	5.08E-08
7.5	100	77.0313	28993	100	2.79E-09	0	1.36E-10
2.9	100	76.0224	28577	37	0.006473	0	0.00059
0	100	128.1006	49871	34	0.019766	0.000141	0.001597
25.1	100	54.824	20009	18	0.728246	0.000868	0.003596
1.8	100	54.196	19768	32	0.028134	0.000951	0.003838
10.5	100	76.1585	28630	27	0.065294	0.001576	0.008628
24.1	100	88.0161	32972	23	0.225976	0.002257	0.01294
5	100	86.4982	32301	19	0.642648	0.003533	0.0249
4.1	100	88.5252	33194	22	0.275694	0.005271	0.03593
1	100	73.5454	27533	21	0.239419	0.005618	0.03877
0	100	127.5921	49641	17	0.895192	0.00861	0.065
20.8	100	45.9077	15977	69	2.69E-06	0	0.000173
15.4	100	37.6595	13408	64	1.13E-05	0	0.000572
8.4	100	100.6661	35962	62	3.34E-05	0	7.86E-06
22	100	100.1648	35768	54	0.000182	0	1.97E-05
5.6	100	103.3487	37037	35	0.012529	0	2.49E-05
13	41.378	80.1619	29746	89	4.88E-08	0	2.22E-07
73.1	100	65.973	23997	38	0.005175	0	0.000881
0.3	100	101.6244	38708	32	0.009717	0	0.000815
3.5	100	125.3552	46129	22	0.199218	0.000525	0.002217
0.9	100	124.3449	45714	36	0.008067	0.000525	0.002555

21.4	100	31.3358	10728	32	0.015945	0.001022	0.00522
0.6	100	124.8511	45914	22	0.216935	0.001022	0.005438
28.8	100	31.848	10926	34	0.009411	0.001022	0.005401
2.1	100	102.8443	36847	20	0.401029	0.002257	0.0127
92.7	100	45.4742	15805	29	0.032136	0.002315	0.01346
6.2	100	39.3149	15169	25	0.08661	0.002543	0.01572
3.7	100	80.285	29791	29	0.044731	0.003736	0.02698
20.5	100	22.299	6908	43	0.00215	0.003736	0.02718
1.3	100	119.0966	44520	40	0.003033	0	0.000181
24.4	100	67.7032	23257	23	0.228884	0	2.61E-05
7.1	100	112.4138	40252	67	2.47E-07	0	1.99E-06
16.1	100	96.0345	35605	77	1.1E-07	0	4.49E-07
6.6	100	95.5303	35427	45	0.000185	0	0.000283
24.3	100	98.8681	36651	77	3.85E-07	0	4.22E-07
23.3	100	98.7775	36618	56	4.73E-05	0	7.72E-05
7.7	100	98.2749	36435	61	1.61E-05	0	5.01E-05
17.2	100	50.0373	19768	42	0.001075	0.000951	0.00395
57.5	100	32.9842	11389	39	0.00402	0.001114	0.005873
1.3	100	111.9105	40084	22	0.006882	0.002257	0.01253
24.3	100	29.9484	11277	13	1.484461	0.002777	0.01796
1.4	100	77.6495	28789	40	0.003553	0	0.000176
46.8	100	78.1538	28971	78	6.61E-07	0	1.8E-08
13	100	20.3227	6383	63	2.11E-05	0	0.000125
44.6	100	20.2618	6360	51	0.000384	0	0.000261
20.5	100	36.4459	12964	42	0.002441	0	0.000303
6.9	100	76.6455	28305	66	1.08E-05	0	3.11E-06
14.3	100	76.2822	28157	30	0.03608	0	0.001524
7.5	100	23.9423	7145	31	0.026312	0	0.000409
20.7	100	24.1141	8872	40	0.004709	0	6.16E-06
47.9	100	74.8315	26219	39	0.005797	0.00065	0.002757
2	100	76.1426	28096	25	0.13803	0.001022	0.005126
72.9	100	22.957	8374	23	0.173184	0.001114	0.00643
6.7	100	74.9135	26256	40	0.004201	0.001114	0.005886
1.1	100	63.9447	21694	25	0.090849	0.002543	0.01618
44.5	100	75.3335	26441	22	0.2961	0.007213	0.05274
7.7	100	99.2167	37214	47	0.000595	0	1.53E-05
61.3	88.908	99.1318	37182	67	6.35E-06	0	9.22E-08
184	83.233	35.8417	12199	32	0.030685	0	0.000706
11.2	100	44.124	15820	47	0.000352	0	8.68E-05
14.2	100	58.0097	20769	33	0.01952	0	0.000684
33.7	100	58.5146	20971	60	3.45E-05	0	1.48E-07
5.8	100	48.1247	18925	44	0.001924	0	4.31E-05
6.8	100	48.5357	19115	59	5.62E-05	0	9.16E-06
11.6	100	48.022	18880	41	0.003759	0.000273	0.001828
2.1	100	98.7147	37011	14	1.354738	0.005325	0.03733
11.9	100	35.8341	12196	30	0.04373	0.009498	0.07205
0	100	88.3771	32969	57	2.27E-05	0	8.46E-08
154.1	100	50.2359	17655	57	4.1E-05	0	0.000508
46.3	100	117.5633	44175	78	4.11E-07	0	3.3E-06
14.7	100	91.6309	33258	39	0.00356	0	0.000351
34.5	94.541	91.1255	33057	53	0.000146	0	7.45E-05
43.1	100	41.5776	16106	32	0.022183	0.000951	0.004543

302.8	84.671	18.981	6608	33	0.016814	0.000951	0.004006
1.2	100	56.9556	20816	35	0.010727	0.001022	0.0052
33.4	100	19.0246	6630	43	0.001377	0.002079	0.0119
12	100	86.8666	31319	15	0.918082	0.004643	0.03336
13.4	100	90.6188	32859	16	0.706669	0.009858	0.08111
11.6	100	103.0948	39896	49	0.000541	0	0.000649
13	75.842	102.8782	39799	49	0.000555	0	5.67E-06
9.5	100	102.5952	39676	48	0.000595	0	0.000531
19.2	100	102.3768	39575	59	4.72E-05	0	1.03E-06
2.7	100	103.8873	40227	45	0.001319	0	0.000287
14.4	78.728	103.3825	40015	58	7.09E-05	0	2.67E-05
8.8	100	94.3776	36150	59	3.98E-05	0	3.29E-06
13.7	100	65.661	26483	51	0.000297	0	6.12E-06
25.3	100	58.7667	23516	56	0.000109	0	2.08E-05
59.6	70.708	65.1597	26276	24	0.171759	0	0.000433
27.8	91.447	64.6566	26056	67	8.15E-06	0	4.11E-06
67.5	100	19.6155	6898	48	0.000411	0	9.61E-05
3.8	100	93.874	35932	24	0.124119	0.000141	0.00167
7.2	100	58.7689	23517	38	0.006837	0.000758	0.002813
13.9	100	64.1483	25835	30	0.042355	0.000868	0.003483
5.6	100	20.5268	7323	21	0.310449	0.002315	0.01378
6.7	100	103.5982	40102	30	0.039327	0.002543	0.0158
6.7	100	58.2604	23310	22	0.298753	0.002777	0.01793
2.5	100	64.6607	26058	29	0.054663	0.00337	0.02168
22	100	64.3957	21879	35	0.008589	0	1.62E-05
4.4	100	103.9546	37268	77	7E-07	0	1.09E-06
6.3	100	86.2769	31060	41	0.002292	0	3.69E-07
0.5	100	85.8551	30883	32	0.017171	0	0.000892
10.6	100	86.3651	31101	17	0.632448	0.000525	0.002349
1.3	100	125.0634	46619	22	0.218324	0.001854	0.009806
2.3	100	64.3705	21869	20	0.263865	0.002315	0.0132
13	100	50.4518	16437	15	0.627275	0.002315	0.01346
18.5	100	42.9023	14813	17	0.86333	0	0.00139
49.7	100	43.1511	14908	25	0.111888	0	9.3E-05
28.6	100	43.4067	15001	13	2.020192	0	0.001448
18.4	100	140.1763	52108	67	6.01E-06	0	4.12E-11
37.9	100	66.3641	24431	49	0.000565	0.000525	0.002625
35.3	100	65.862	24235	48	0.00064	0.000758	0.003066
3.4	100	101.2367	37330	27	0.086585	0.001498	0.007682
19.8	100	102.7374	39738	7	2.803667	0.002843	0.01914
26	100	109.6187	40781	66	5.13E-06	0	0.000186
7.1	100	115.2885	44921	47	0.000822	0	0.000748
40.5	100	82.2932	30526	63	7.54E-06	0	7.23E-05
16.3	92.994	56.9317	22743	39	0.004431	0	0.001032
14.6	100	56.8272	22701	40	0.003156	0.000141	0.001647
6.9	100	115.2344	44898	32	0.022516	0.000951	0.003624
3.2	100	82.1692	30479	31	0.014581	0.001498	0.007761
68.5	100	18.5543	6402	37	0.002912	0.002543	0.01587
27.8	100	48.8484	17154	40	0.003086	0	2.97E-05
26.5	100	130.126	49609	51	0.000167	0	8.7E-05
15.8	100	114.2409	43616	87	1.22E-08	0	0.000264
11.6	100	96.4654	35285	52	8.5E-05	0	0.000965

14.2	100	74.1847	25488	49	0.000211	0.000525	0.002497
2.8	100	113.7294	43411	61	4.74E-06	0.000758	0.003192
8.3	100	130.0814	49590	35	0.007041	0.000758	0.00311
3	100	82.5821	30690	30	0.04597	0.001308	0.007132
9.6	100	123.5173	50732	50	0.000376	0	0.000998
25.6	100	40.6325	12746	39	0.00542	0	0.000729
16.2	100	140.3841	54519	81	2.63E-07	0	5.36E-11
31.2	100	64.1371	21777	35	0.007399	0.001022	0.004778
3.6	100	108.7208	42197	29	0.036458	0.001576	0.008367
2.6	100	77.4727	31534	52	0.000283	0	0.00012
13.2	100	61.3436	20462	34	0.010648	0	0.001334
43.7	58.739	141.1075	54494	77	8.36E-07	0	2.3E-08
11.8	100	61.6778	20587	36	0.005957	0.000758	0.003188
6.9	100	93.6636	33237	30	0.040208	0.002315	0.01339
32.1	100	36.964	12947	32	0.015961	0.002376	0.01542
13	100	93.1627	33036	19	0.530861	0.003674	0.02596
7	100	61.1724	20395	25	0.077992	0.004124	0.02983
11.7	100	114.0058	42648	37	0.007331	0	0.000047
70.8	100	121.6721	45985	65	1.47E-05	0	4.89E-05
14	85.046	72.5279	29469	54	0.000146	0	4.37E-05
61.9	100	30.81	10076	55	6.92E-05	0.00065	0.002747
11.8	100	121.6008	45951	27	0.095495	0.001576	0.008143
4.4	100	113.5003	42419	22	0.276997	0.001576	0.0081
114	100	104.9778	38883	27	0.043355	0.003533	0.02472
38.6	100	37.4313	11637	39	0.005064	0	0.00033
21.6	100	140.899	52493	84	1.9E-07	0	2.83E-09
7.9	100	66.9073	24444	42	0.002027	0	0.000718
25.5	100	81.5196	28349	31	0.029197	0.00337	0.02272
0.4	100	127.2304	49493	17	0.876307	0.004643	0.03332
18.6	100	19.7741	5689	36	0.01091	0	6.29E-05
26.2	100	91.566	32398	31	0.02587	0	0.000127
3.6	100	77.8843	28858	49	0.000472	0	6.73E-05
26.6	100	50.204	19844	37	0.007464	0.001022	0.005504
12.1	100	50.2177	19850	36	0.00916	0.001758	0.009325
31.9	100	77.5162	26779	18	0.375032	0.003254	0.02113
5.6	100	98.971	37543	56	9.58E-05	0	0.00011
28.1	100	58.4162	20948	43	0.001955	0	0.000142
12.7	100	74.4036	30266	51	0.0003	0	0.000179
15.1	100	64.2068	23348	38	0.005357	0.000525	0.002259
58.2	100	38.6899	13291	38	0.000517	0.002012	0.0104
14.2	100	73.9242	30072	20	0.399233	0.002777	0.01694
1.4	100	106.0371	39888	36	0.011662	0	0.001172
70	100	67.4844	22844	41	0.002888	0	0.000171
15.8	100	105.6182	37947	62	2.67E-05	0	5.94E-05
19.9	100	57.2712	20469	24	0.127857	0.002012	0.01108
3.4	100	67.9139	24778	29	0.029532	0.003736	0.02682
9	100	67.8863	24768	29	0.026203	0.006721	0.0496
31.9	100	50.0925	16200	35	0.011393	0	0.00036
19.9	100	39.3013	13840	36	0.009126	0.000141	0.001735
173.5	100	16.8062	5197	35	0.015027	0.000273	0.002112
39.1	100	49.1151	17257	33	0.011	0.000525	0.002632
116	100	45.1784	17661	56	7.39E-05	0.000868	0.003519

36.1	100	39.8918	14069	26	0.083217	0.001308	0.006999
21.8	100	40.3894	14152	19	0.432354	0.00337	0.02328
13.4	100	62.9015	23093	46	0.001236	0	0.000318
73	100	18.662	6086	19	0.577141	0	0.000657
16.1	100	62.5352	23029	27	0.042997	0	0.001487
0	100	109.3979	44927	22	0.255757	0.002079	0.01141
17.3	100	42.1698	13276	31	0.018206	0.003892	0.0283
13.4	100	62.9015	23093	46	0.001236	0	0.000318
73	100	18.662	6086	19	0.577141	0	0.000657
16.1	100	62.5352	23029	27	0.042997	0	0.001487
0	100	109.3979	44927	22	0.255757	0.002079	0.01141
17.3	100	42.1698	13276	31	0.018206	0.003892	0.0283
27.5	100	76.3248	26855	43	0.000157	0	3.45E-05
5.5	100	62.4476	21103	33	0.016185	0.000951	0.004542
13.3	100	29.8816	11248	34	0.014152	0.000951	0.00448
57.1	100	66.1255	24063	37	0.008384	0.002315	0.01352
11.3	100	86.9952	35539	32	0.019858	0.003967	0.02867
136.5	42.62	42.7956	15305	34	0.011235	0	0.000676
128.4	38.098	56.5524	18661	81	1.77E-07	0	1.04E-10
32.5	91.42	56.529	18651	94	8.47E-09	0	8.18E-10
153.3	100	56.0523	18469	40	0.002274	0	1.52E-06
14.3	100	66.4007	22420	61	2.75E-05	0	5.34E-05
161.8	37.529	65.9704	22251	56	8.64E-05	0	0.000736
69.9	100	65.8921	22218	74	1.53E-06	0	2E-05
40	100	137.9664	51097	79	3.54E-07	0	6.84E-09
5	100	32.578	10000	97	5.35E-09	0	1.41E-06
218	60.917	45.2531	15660	40	0.003666	0	0.000843
52.2	100	116.2611	43618	46	0.000882	0	0.000708
15.6	100	115.7594	43412	42	0.001962	0	0.000598
32.3	51.95	118.5193	44604	93	1.93E-08	0	3.4E-10
44.3	100	60.8018	24393	34	0.017019	0	0.000113
39.8	32.837	61.3013	24619	54	0.000175	0	3.46E-06
13.5	90.28	56.2712	22450	67	8.22E-06	0	2.61E-06
57.1	100	55.7691	22241	60	4.42E-05	0	3.54E-07
19.6	100	42.2908	15115	27	0.05579	0.000273	0.002107
0	100	136.2321	52206	29	0.026509	0.000868	0.003465
2.1	100	90.8906	34681	44	0.000885	0.000951	0.004187
62.5	3.81	61.6254	24765	27	0.073294	0.001022	0.00542
14.6	100	42.8426	15324	38	0.00531	0.001576	0.008689
2.2	100	135.9832	52103	19	0.297089	0.002012	0.01115
5.3	100	61.3658	24648	30	0.048457	0.002315	0.01337
50.5	100	32.2894	9900	34	0.012222	0.002543	0.01623
3.4	100	101.6235	37492	28	0.058758	0.00337	0.0228
13.9	100	61.8522	22685	23	0.213535	0.004344	0.03128
5.8	100	60.8601	24421	23	0.218359	0.00728	0.05297
6.1	100	47.9894	15454	45	0.001234	0	0.000114
2.6	100	49.4564	17925	62	2.59E-05	0	1.75E-06
14.8	100	94.0005	33364	40	0.004031	0	0.000367
15.3	100	93.9898	33359	44	0.001412	0	0.000568
27.5	100	90.7902	32069	58	5.63E-05	0	1.48E-05
8.4	100	89.4578	31544	43	0.001747	0	0.000924
74.7	2.353	93.4972	33174	43	0.002068	0	0.000443

55.9	2.676	93.4856	33169	65	1.35E-05	0	9.61E-06
9.6	100	92.9958	32965	47	0.00077	0	0.000742
37.6	100	90.2248	33896	67	8.18E-06	0	3.71E-06
81.9	13.797	140.5206	55221	24	0.126161	0	3.32E-05
11.5	100	88.7781	33755	76	1.02E-06	0	3E-08
8.5	100	89.6973	34168	46	0.000836	0	0.000771
21.6	6.825	88.283	33538	107	8.28E-10	0	1.65E-10
12.7	1.127	88.2707	33532	84	1.62E-07	0	7.63E-08
21.4	100	87.7645	33306	51	0.000375	0	2.21E-06
10.3	100	80.6539	30249	54	0.000191	0	1.05E-05
31	100	47.6281	18704	54	0.000121	0	0.001297
7	100	90.721	32043	39	0.005454	0.000273	0.001966
4.9	100	100.1507	35762	35	0.012963	0.000525	0.002246
7.9	100	93.4007	35172	55	9.79E-05	0.000758	0.00287
1.5	100	133.4642	52146	22	0.233728	0.000758	0.002896
29	100	89.9341	33772	31	0.032867	0.001576	0.008183
80.9	100	47.6882	18731	42	0.002081	0.001758	0.00925
7.6	100	89.2848	33987	23	0.222838	0.002543	0.01616
18.8	100	48.7102	19197	34	0.010588	0.003736	0.02723
125.7	100	43.1906	16811	39	0.004031	0.003967	0.02884
144.8	6.568	48.196	18959	43	0.00157	0.00573	0.03994
14.9	100	49.2256	17835	28	0.047534	0.006428	0.04719
2.3	100	56.9923	22768	19	0.547631	0.006926	0.05047
0	100	90.1814	34382	20	0.335013	0.0081	0.05957
5.7	100	137.1938	50734	43	0.001138	0	0.000277
18.2	100	137.2299	50751	59	2.59E-05	0	5.37E-07
17	79.665	137.6973	50968	56	4.98E-05	0	2.25E-06
53.4	100	137.7428	50990	85	7.07E-08	0	7.28E-09
3.7	100	17.2132	4951	46	5.89E-05	0	0.001439
18.8	100	100.8911	38946	64	1.21E-05	0	5.44E-06
15.5	100	100.5627	38798	58	4.97E-05	0	3.75E-05
30.2	100	52.8174	19155	51	6.39E-05	0	1.91E-06
11.6	100	52.3147	18949	30	0.009418	0	0.000274
15.1	100	45.5901	16298	66	5.89E-07	0	8.43E-05
21.5	100	51.8222	20551	28	0.075391	0.000758	0.002888
0.3	100	45.9531	16446	54	8.6E-06	0.000951	0.004545
4.2	100	17.2476	4833	39	0.000295	0.001022	0.004642
0	100	17.2744	4843	48	3.57E-05	0.001022	0.00571
18.1	100	19.4926	5964	36	0.007415	0.001576	0.008494
18.1	100	19.4926	5964	37	0.006458	0.001576	0.008111
22.3	100	17.2076	5106	35	0.000733	0.002012	0.01028
149.4	100	25.9327	8205	29	0.026764	0.002257	0.01255
0.7	100	17.2318	5117	43	0.000113	0.003096	0.01998
25.9	100	73.4919	25633	29	0.061163	0.004643	0.03278
33.9	100	25.9743	8220	33	0.009051	0.00599	0.04532
3.9	100	110.9783	41647	40	0.004176	0	0.001535
7	100	111.4796	41849	41	0.003119	0	0.001319
12.7	100	140.2549	55085	40	0.004192	0	2.32E-05
24.2	100	15.7655	4969	43	0.001541	0	0.001246
153.3	17.749	15.7488	4961	29	0.038264	0	0.00088
204.2	100	17.9588	6101	59	2.12E-05	0	1.61E-06
11.2	100	79.2814	28147	40	0.004441	0	0.000831

18.5	100	55.3122	20210	23	0.16199	0.000758	0.003189
11.6	100	111.5108	41860	43	0.001976	0.000868	0.003363
8	100	17.9896	6116	28	0.023237	0.00822	0.06026
0	100	119.6979	46316	32	0.028859	0	0.00097
45.6	100	140.5019	52271	81	3.86E-07	0	2.85E-08
33.1	100	60.7362	24366	29	0.054924	0	0.001536
39.2	100	20.5434	6572	46	0.000108	0	1.78E-05
53.2	100	63.7983	21637	55	9.97E-06	0.000868	0.003459
18	100	33.56	12779	25	0.112088	0.001576	0.008586
13.5	100	140.5139	52277	18	0.872246	0.003674	0.02548
10.8	100	130.5766	49812	53	0.000108	0	0.000178
23.5	100	118.334	44519	34	0.015033	0	0.000313
29.6	100	61.6567	22410	68	5.02E-06	0	3.14E-06
26.6	100	61.1513	22216	55	9.71E-05	0	2.95E-05
8.6	100	60.6409	22012	43	0.001698	0.000951	0.004486
8	100	89.7226	34180	23	0.109928	0.00121	0.006808
4.1	100	78.3761	29062	27	0.074288	0.001945	0.01003
15.7	100	85.9187	32061	32	0.01403	0.002843	0.01842
10.1	100	80.5487	29900	22	0.260427	0.004049	0.02914
11.1	100	49.0932	19360	26	0.05904	0.007358	0.05342
7.1	100	123.0246	46574	58	5.48E-05	0	5.2E-06
11.7	100	115.1847	43148	44	0.000599	0	0.000729
14.5	100	93.66	34099	35	0.004559	0	2.97E-05
2.9	100	99.15	36758	32	0.020334	0.00121	0.006459
5	100	17.4626	5038	46	0.000478	0.003674	0.02644
18.1	100	14.8019	4100	17	0.796785	0.003674	0.02547
13.7	100	132.4872	51712	60	3E-05	0	1.11E-06
17.4	100	79.0972	29064	44	0.00065	0	0.001135
16.1	100	97.4248	36859	50	0.000117	0.000951	0.003829
32.2	100	95.3626	35988	36	0.004155	0.00121	0.00685
5.4	100	75.4899	30703	24	0.119253	0.001945	0.01018
8.8	100	74.0655	27443	15	1.156644	0.002315	0.01371
9	100	135.1742	49900	54	0.000221	0	0.000194
23.8	100	76.6813	31206	44	0.001844	0	0.001454
2.7	100	51.3803	16788	41	0.00273	0.000951	0.003952
38.2	100	51.1902	16720	23	0.177432	0.001022	0.004628
55.7	100	141.0936	54486	7	5.512935	0.001758	0.009568
0	100	134.6692	49698	35	0.016764	0.001758	0.009424
110.5	100	39.8312	13926	38	0.00398	0.002376	0.01446
1	100	68.9828	23776	29	0.058191	0.002376	0.01429
5.3	100	106.2164	39406	17	0.011544	0	0.000533
71.9	100	28.8117	9577	28	0.07736	0.000525	0.002256
32.6	100	43.4486	15557	28	0.063548	0.000868	0.003511
12.5	100	78.5069	31974	41	0.003231	0.000951	0.004296
11.5	100	78.4759	31962	34	0.017976	0.00337	0.0229
5.8	100	103.9452	39703	24	0.033262	0.003674	0.02567
6.4	100	110.4924	41150	23	0.170133	0.003736	0.02753
16.1	100	120.4967	45036	90	1.74E-08	0	3.73E-10
36	100	119.9928	44856	85	4.7E-08	0	3.7E-12
31.7	100	119.4885	44666	92	1.14E-08	0	4.91E-11
33.4	17.807	29.0837	10134	30	0.018833	0	0.00011
75	28.801	61.3312	20457	46	0.001041	0	2.52E-06

8.1	100	78.1788	27034	91	3.01E-08	0	4.29E-12
11.3	100	77.0674	28485	40	0.004211	0	0.000211
32.3	100	102.2871	38997	49	0.00059	0	1.23E-07
6.7	100	107.5189	41221	43	0.002448	0	0.000726
34.1	100	60.8275	20265	31	0.034747	0.000141	0.00168
3.7	100	101.783	38774	20	0.420585	0.000758	0.00294
51.3	100	29.0399	10117	9	2.77281	0.000868	0.003397
20.4	100	56.639	18694	35	0.014347	0.00121	0.006599
11.4	100	61.0235	20338	29	0.062572	0.00121	0.006689
0.4	100	107.0088	40993	27	0.081239	0.001498	0.007717
42.4	100	17.0988	5338	38	0.004896	0.001677	0.008929
3.4	100	78.2558	27064	17	0.933861	0.002012	0.01104
632.9	100	16.9586	5565	42	0.00188	0.002777	0.0174
163.5	2.261	16.9655	5570	44	0.001336	0.00337	0.02395
0	100	102.371	39036	22	0.307548	0.003674	0.0265
198.9	100	17.0901	5334	26	0.08779	0.008171	0.05995
6.6	100	77.7533	26871	19	0.559569	0.008361	0.06236
4	100	32.0657	9770	64	1.09E-05	0	2.49E-06
68.9	100	24.9659	8595	20	0.212201	0	0.001013
62	67.859	76.9715	26573	43	0.002084	0	8.2E-08
17	100	73.1782	25054	39	0.004273	0	0.000478
19.8	100	73.1669	27098	26	0.072692	0	0.000525
6.3	88.575	73.6697	27318	71	2.55E-06	0	6.35E-08
11.3	100	137.6248	52809	53	0.000204	0	1.52E-07
39.1	100	97.0872	35541	66	1.27E-05	0	2.83E-05
23.1	100	32.3884	12302	63	1.66E-05	0	2.22E-07
71	100	46.9287	16906	57	7.84E-05	0.000868	0.00352
189.9	100	47.4365	17105	57	7.17E-05	0.001022	0.005795
32.5	100	90.2841	32714	47	0.000663	0.001114	0.006108
10.6	100	79.0452	28049	27	0.092522	0.001677	0.008941
4.1	100	107.2705	39838	25	0.06507	0.002079	0.01218
4	100	107.7775	40043	22	0.127678	0.002843	0.01867
12.5	100	36.5171	14025	52	0.000207	0.002843	0.01833
13.8	100	70.249	24293	21	0.346777	0.004344	0.03155
11.5	100	88.9242	33180	41	0.002694	0	0.001308
55.1	20.145	88.4078	32983	57	6.97E-05	0	5.15E-05
105.7	3.53	87.9058	32773	51	0.000244	0	0.000737
4	100	83.3846	33969	73	9.17E-07	0	0.000134
214	28.094	17.4912	5851	39	0.001751	0	5.72E-05
19.9	100	76.1665	28634	65	1.03E-05	0	0.000138
28.9	100	75.1477	28198	64	1.14E-05	0	6.93E-05
109.3	1.895	74.6382	27977	63	1.46E-05	0	0.000122
121.1	2.38	74.3511	27868	69	3.84E-06	0	0.000104
81.2	36.034	48.1625	18944	40	0.002977	0	1.99E-05
30.1	100	47.6427	18711	38	0.005507	0	0.000825
15.5	100	13.6646	3716	39	0.004694	0.001022	0.00534
13.3	100	17.5092	5861	52	8.69E-05	0.001022	0.004787
72.3	16.753	88.4177	32987	34	0.01396	0.001114	0.005998
13.7	100	82.3981	30620	42	0.001805	0.00121	0.006584
61.4	22.582	74.6893	27999	38	0.005075	0.00121	0.006751
59.5	100	30.378	10194	55	8.51E-05	0.001498	0.007672
21.5	100	18.0118	5243	18	0.543264	0.001945	0.01005

112.8	2.658	87.9159	32778	34	0.014839	0.002315	0.01322
30.1	92.798	70.0459	25640	51	0.000136	0.002376	0.01534
34.4	100	79.3611	28180	14	1.359536	0.002376	0.01424
73.5	100	30.6093	10429	43	0.001417	0.002843	0.01842
9.8	100	87.4044	32563	22	0.180895	0.00337	0.02199
10.2	100	86.3189	30231	16	0.752577	0.003892	0.02839
11.9	100	75.6563	28413	32	0.02293	0.005271	0.03573
21.5	100	18.0118	5243	18	0.543264	0.006721	0.04933
41.6	100	31.113	10637	37	0.005464	0.006926	0.05078
14.5	100	85.1751	31677	33	0.01027	0.006991	0.0515
7	100	115.0378	43104	51	0.000254	0	0.00024
79.9	81.873	51.8639	18850	40	0.00301	0	0.000225
15.2	100	52.9756	19272	46	0.000635	0	0.00039
11.4	100	52.5359	19106	54	9.98E-05	0	0.00026
6	100	137.3458	50803	41	0.001671	0	0.001387
1	100	136.8185	50579	43	0.000878	0.000141	0.00158
26.9	100	39.2271	13286	52	0.000265	0.000758	0.002823
10.4	100	115.309	43196	34	0.013765	0.000951	0.0043
98.3	70.344	86.6203	32240	43	0.001946	0.001576	0.008521
2.5	100	86.2692	32099	31	0.028165	0.002376	0.01429
79.2	100	40.4817	13950	29	0.030175	0.002777	0.01714
51.4	100	25.2772	8183	44	0.001282	0.003254	0.02111
12.8	100	37.6375	13400	37	0.002635	0.00337	0.02352
41.9	100	86.1143	32036	31	0.027772	0.003892	0.02806
15.8	100	51.3592	18651	19	0.422211	0.003967	0.02884
5.3	100	92.6248	34594	29	0.05622	0	0.000571
6.1	100	135.9466	55719	35	0.012508	0	0.000186
21.5	100	82.4489	33583	60	2.98E-05	0	1.71E-05
26.4	100	82.3978	33558	48	0.000392	0	0.000274
2.4	100	99.5292	35513	44	0.001527	0	0.000584
7.8	100	64.6269	23456	45	0.001394	0	0.000619
291.3	88.834	17.9704	5761	30	0.027286	0.000407	0.002156
4.3	100	82.2476	33497	33	0.013642	0.00121	0.006511
1.6	100	92.167	34421	28	0.074287	0.00141	0.007328
58	100	47.6305	16657	19	0.635684	0.001498	0.007531
1.4	100	99.0165	35313	32	0.022356	0.001498	0.007528
9.1	71.294	83.7071	31107	58	6.26E-05	0	2.29E-07
43.4	100	46.4074	18212	27	0.076038	0	0.001271
32.2	100	24.7965	8153	19	0.1632	0	0.00097
132	70.019	84.1472	30161	45	0.001022	0.000141	0.001665
1.9	100	82.5985	30708	25	0.144123	0.000273	0.00208
61.7	100	45.9754	15953	33	0.017576	0.000951	0.003743
4.3	100	83.2031	30920	16	0.977634	0.001114	0.0061
59	100	15.1256	4248	31	0.022048	0.002376	0.01517
0.4	100	82.096	30510	13	1.91797	0.004424	0.03169
2.8	100	83.6883	31523	15	0.477326	0.004643	0.03314
16.2	100	15.1821	4270	37	0.005492	0.006222	0.04609
2.2	100	133.0226	54592	41	0.003126	0	0.00014
37.6	100	41.1915	14218	35	0.011782	0.000273	0.001857
41.8	100	60.8174	21960	7	6.864832	0.000868	0.003551
6	100	54.0388	21507	26	0.0324	0.001308	0.006885
34.6	100	66.1089	24335	25	0.113507	0.002079	0.01177

12.5	100	87.967	32950	21	0.398302	0.002376	0.01422
0.4	100	108.5492	42130	25	0.114888	0.00337	0.02236
50	100	38.011	13033	27	0.024215	0	0.00085
75.1	3.187	37.5093	12847	24	0.046568	0	0.000269
100.8	24.409	37.441	12819	25	0.042071	0	0.000148
106.6	11.673	37.0037	12651	26	0.030038	0	0.000372
38.6	15.936	141.0471	52572	88	3.97E-08	0	2.86E-08
1.4	100	141.057	52577	92	1.77E-08	0	1.16E-07
199.4	31.711	140.983	52538	88	5.7E-08	0	5.02E-11
37.2	85.652	54.4155	19362	30	0.033166	0	0.000531
44.9	99.842	53.9164	19168	31	0.025574	0	0.000186
5.3	69.748	141.671	54827	42	0.002369	0	5.44E-06
67.8	100	52.0155	18787	57	8.92E-05	0	0.000445
70.6	2.515	51.5123	18591	71	4.08E-06	0	0.000393
112.1	0.646	50.5008	18190	56	0.000116	0	0.001436
89.4	3.284	51.0062	18393	65	1.42E-05	0	0.000367
74.5	5.266	51.0068	18394	44	0.001901	0	0.001518
32.8	100	52.5184	18982	51	0.000341	0	0.001088
24.9	100	53.021	19170	54	0.000179	0	0.000652
18.5	100	54.0329	19542	54	0.000189	0	0.000956
42.3	100	49.9975	18000	54	0.000162	0	0.000511
5.8	27.548	142.8874	55531	42	0.002917	0	1.08E-07
31.3	100	140.9078	54375	36	0.00925	0	1.11E-05
38.9	100	43.1347	15344	60	2.82E-05	0.000141	0.001599
40.6	100	44.3989	15309	36	0.009938	0.000273	0.002081
30.9	67.803	42.6293	15152	59	3.95E-05	0.000525	0.002553
22	100	53.5227	19353	53	0.000197	0.000525	0.002256
20.8	100	57.2706	20735	45	0.00128	0.000525	0.002409
41	100	55.981	22335	42	0.001096	0.000525	0.002454
99.9	1.088	50.5014	18191	38	0.008166	0.000951	0.003665
34.1	100	54.2884	19641	29	0.050728	0.001022	0.005519
25.1	100	38.5168	13226	19	0.164851	0.001022	0.005769
34.8	100	38.621	12047	22	0.075681	0.001022	0.005079
89.2	3.381	51.513	18592	38	0.008045	0.001022	0.004931
24.2	53.677	37.4424	12820	39	0.001683	0.001114	0.006235
17.3	100	56.5222	22569	31	0.016949	0.00121	0.006757
8.5	100	55.0074	21931	33	0.009245	0.001308	0.006967
49.5	90.933	140.4956	55208	16	0.836322	0.001498	0.007699
14.4	100	36.4285	12957	40	0.001712	0.001498	0.007577
17.4	100	38.431	13684	25	0.038814	0.001498	0.007941
14.4	100	36.4285	12957	40	0.001712	0.001498	0.007577
19.6	100	53.0326	19174	34	0.015393	0.001758	0.009267
169.5	1.369	55.3552	22071	50	0.000214	0.001854	0.009717
63.7	100	52.0217	18790	28	0.062617	0.002079	0.0121
22.4	100	33.0851	11172	22	0.129522	0.002257	0.01277
16.8	100	51.257	20307	41	0.001583	0.002315	0.01393
0	100	142.925	55557	30	0.040547	0.002315	0.01416
26	100	28.7063	9535	17	0.053599	0.002777	0.01816
37.3	3.554	55.5119	22141	31	0.020272	0.002843	0.01869
27.8	100	54.826	19832	29	0.056656	0.00337	0.02269
40.4	100	41.5078	14335	17	0.236273	0.00362	0.02497
149.8	1.304	55.4774	22124	42	0.001353	0.003736	0.02677

181	100	53.8751	19155	8	4.551777	0.004124	0.02965
55.2	100	51.425	18583	26	0.103432	0.004124	0.02929
41.4	100	42.4031	14634	17	0.238774	0.004266	0.03075
27.3	100	39.0795	13437	14	0.440923	0.005031	0.03519
22.9	100	55.5489	20092	30	0.047477	0.005114	0.03544
18.3	100	39.5911	13620	16	0.31841	0.00573	0.0402
27.4	100	52.5287	18987	29	0.052681	0.005792	0.04224
13.8	100	56.4882	22553	29	0.023703	0.007423	0.05398
22.3	100	44.2537	15246	24	0.173952	0.007636	0.05483
22.3	100	44.2537	15246	24	0.173952	0.007636	0.05483
96.2	100	50.109	17600	24	0.185987	0.008962	0.06775
23.9	100	52.8547	21005	35	0.005577	0.009818	0.07916
27.5	100	53.5361	19358	22	0.246977	0.009858	0.08106
100.8	24.605	64.5538	23728	48	0.000413	0	0.000899
35	100	38.1495	11873	54	9.92E-05	0	0.000277
131.4	64.436	38.0925	11854	45	0.000754	0	0.000241
34.4	37.971	98.8038	35233	63	1.44E-05	0	1.54E-07
228.2	30.671	17.6612	5948	65	1.39E-05	0	9.75E-06
61.2	100	140.3448	54064	100	2.81E-09	0	1.97E-13
60.2	100	140.3404	54062	62	1.59E-05	0	6.09E-10
73.5	100	45.3813	15714	36	0.011316	0	8.52E-05
146.4	33.958	141.0257	54445	66	7.44E-06	0	1.53E-08
47.5	100	61.9851	24922	50	0.000176	0	0.00027
4.9	100	77.6478	28744	32	0.027476	0.000951	0.004419
18.3	100	35.6722	10998	31	0.019237	0.000951	0.004162
39.2	100	37.5896	11688	27	0.048728	0.00121	0.006481
3.6	100	57.8041	23117	47	0.000291	0.001498	0.007955
7.9	100	98.5944	35154	17	0.586302	0.001758	0.009613
5.7	100	78.4924	27157	16	1.055062	0.004581	0.03259
8.8	100	37.6474	11708	28	0.038175	0.00599	0.04486
2	100	33.442	10228	79	2.14E-07	0	5.44E-06
47.4	100	34.4253	11668	25	0.042094	0	0.000754
18.2	100	35.4055	10906	35	0.005268	0	0.000852
37.9	100	35.9457	12786	40	0.002753	0	0.000584
31	100	140.6321	55290	109	5.41E-10	0	1.74E-12
119.3	100	36.8131	11431	39	0.001371	0	0.000117
18.6	100	36.4021	11293	60	1.3E-05	0	0.000105
17	100	50.7639	20090	59	4.3E-05	0	0.000625
14.8	100	30.8289	10517	50	0.000116	0	0.000132
7.5	100	56.5877	21175	37	0.002741	0	0.000921
13.8	100	101.9283	36482	38	0.006731	0.000141	0.001623
6.4	100	133.5308	51045	39	0.005494	0.000758	0.003008
13.3	100	33.3653	10204	45	0.00056	0.000868	0.003398
0.8	100	100.3211	36947	34	0.013252	0.001022	0.004748
4.3	100	133.0267	50825	21	0.383249	0.006721	0.04928
2.6	100	101.9575	36495	25	0.109747	0.007636	0.05518
14.8	100	120.2427	44945	80	3.2E-07	0	5.86E-09
31.9	100	119.7383	44763	76	7.92E-07	0	1.19E-08
19.6	100	20.5148	6842	53	0.000166	0	0.001165
104.6	48.425	140.6586	54226	91	1.5E-08	0	1.97E-08
17.2	100	80.0733	29992	44	0.001648	0	2.65E-05
48.2	66.033	141.4243	54687	101	1.23E-10	0	2.6E-12

11.7	100	66.4021	22726	5	12.04597	0.000273	0.001916
12	100	66.9021	22934	10	3.901145	0.001022	0.004749
427	100	21.6583	7804	23	0.151315	0.00121	0.006544
31.9	100	137.4345	50845	26	0.06706	0.002177	0.01237
15.6	100	21.5356	7252	29	0.036733	0.007836	0.05606
19.3	100	74.4858	25606	42	0.002559	0	7.08E-06
24	100	86.6148	30363	31	0.028185	0	0.001149
47.3	57.426	86.1141	30142	51	0.000324	0	8.49E-06
7.1	100	85.6104	29940	44	0.001446	0	0.000255
8	100	86.2328	30193	44	0.001395	0	9.38E-05
2	100	132.7655	50722	33	0.02374	0	0.000355
35.8	94.559	141.038	54453	27	0.072323	0	1.97E-06
50.6	14.87	34.2074	13047	63	1.65E-05	0	6.6E-05
6.9	100	87.1187	30575	36	0.009716	0.000273	0.002098
40.3	92.422	34.1874	13039	38	0.005668	0.00065	0.002765
38.4	36.327	77.5443	28809	48	6.58E-05	0.000758	0.002791
2.5	100	132.2643	50512	14	1.916305	0.002079	0.01134
266	100	23.2404	7548	11	2.941853	0.002079	0.01233
281	52.425	22.733	7365	11	2.90892	0.002843	0.01903
15.9	100	77.0382	28619	28	0.005587	0.00337	0.02313
7.5	100	54.8787	18013	49	0.000182	0.003674	0.02519
9.6	100	64.6812	23545	37	0.005962	0.003674	0.02629
11	100	102.3096	36639	28	0.075043	0	0.00054
50	100	34.8932	11971	32	0.010466	0	0.000655
25.8	100	96.7312	37006	84	1.39E-07	0	7.23E-10
19.8	100	74.5237	27356	109	2.73E-11	0	9.28E-10
1.8	100	74.0225	27172	41	0.000171	0	0.00019
21.6	100	32.9084	10858	19	0.258604	0.001022	0.005014
10	100	66.2028	24178	30	0.004732	0.001022	0.005109
0	100	83.4476	31011	20	0.068049	0.001022	0.005522
193.9	100	17.3918	5800	18	0.049019	0.002012	0.01053
15.8	100	39.7033	13877	31	0.035028	0.002719	0.01681
0.5	100	75.0309	27539	15	0.078536	0.004344	0.0316
107.9	100	39.7584	13898	31	0.034454	0.005031	0.03487
17.4	100	40.2906	14370	44	0.000939	0	0.000555
43.5	100	76.7125	26472	50	0.000306	0	0.000138
7.2	100	63.8631	23146	31	0.034977	0	0.000215
52.6	100	17.3598	5782	49	0.00026	0	5.43E-06
56.2	100	76.3815	26344	43	0.001637	0.00065	0.002703
15.1	100	63.3628	22949	11	3.521782	0.00065	0.002696
11.3	100	52.1179	18863	32	0.017268	0.001022	0.005559
22.1	100	36.7053	12846	26	0.086762	0.001114	0.006125
11.3	100	44.1105	13995	44	0.000974	0.001758	0.009029
11.7	100	14.3999	3960	34	0.011262	0.002777	0.0172
24.4	100	64.418	23369	13	2.139941	0.004192	0.03039
0.7	100	100.8617	37177	13	1.453592	0.004643	0.03327
80.6	25.888	40.52	14450	44	0.000931	0.006926	0.05061
1.7	100	63.6304	23058	14	2.003758	0.007423	0.05356
6.6	100	108.5048	44579	47	0.000455	0	9.64E-05
25.2	100	71.0423	26200	53	0.000169	0	5.63E-06
8.8	100	70.8276	26105	25	0.116698	0	0.000216
34.1	100	70.4932	25963	50	0.000422	0	1.15E-05

18.2	100	36.7984	12882	32	0.021538	0.000525	0.002422
39.7	100	52.5486	19680	43	0.000293	0.000525	0.002616
14.6	100	55.6847	19854	38	0.005782	0.001576	0.008698
4.1	100	118.928	46421	36	0.008671	0.002012	0.01086
7.6	100	56.7511	20732	35	0.005092	0.002315	0.01414
22.9	100	67.7293	24807	30	0.015391	0.002315	0.01297
40	100	35.9454	12550	20	0.29823	0.002843	0.01829
21.8	100	96.0027	35077	27	0.092975	0.003967	0.0288
31.7	100	61.0309	20544	46	0.000427	0	0.000143
69.5	100	46.9405	16911	36	0.009068	0	0.00066
14	100	55.5805	20320	39	0.00493	0	0.000181
0	100	90.9377	34192	30	0.047314	0	0.000644
5.6	100	63.737	25668	34	0.013938	0.000951	0.004212
30.3	100	31.5029	9581	27	0.004046	0.001308	0.006975
6	100	47.0101	16941	40	0.00329	0.001576	0.008158
1.9	100	90.4305	33777	24	0.149281	0.002177	0.01238
11.1	100	66.762	24328	14	1.845064	0.002257	0.01261
0.9	100	61.7035	22622	47	0.000381	0	7.06E-07
5.6	100	77.3619	28683	35	0.008202	0	8.31E-05
22.5	100	42.5407	13407	34	0.006744	0.000525	0.002429
7.6	100	95.7873	34980	21	0.360072	0.000951	0.004556
35.7	100	16.532	4895	23	0.090662	0.000951	0.003998
75.6	100	46.8101	15004	24	0.060096	0.001308	0.007031
49.1	100	32.2757	10942	21	0.116284	0.002257	0.01269
58.2	100	69.1655	23851	33	0.002811	0.002257	0.01265
95.3	82.303	51.7021	18696	36	0.006373	0.00337	0.024
94.5	100	61.7901	20837	31	0.031853	0	0.00099
20.8	100	62.155	20987	55	0.000132	0	4.39E-05
10.5	100	68.3011	23490	38	0.003948	0	2.28E-06
25.9	64.543	68.2676	23477	30	0.031417	0	1.09E-05
35.6	100	67.7641	23281	8	4.04808	0	0.000348
2.1	100	42.3346	13353	59	4E-05	0	0.000805
44.5	100	73.6842	25272	12	3.298147	0	0.000284
27.1	20.973	73.5238	25198	66	1.39E-05	0	3.17E-08
78.5	29.051	141.1555	54525	68	3.79E-06	0	9.4E-06
10.7	100	60.3675	24201	38	0.005717	0	0.001144
116.4	100	35.3919	12337	54	0.000117	0	0.000421
117.4	66.029	41.4192	14301	50	6.97E-05	0.000141	0.00167
6.2	100	17.2477	4963	61	1.34E-05	0.000273	0.001992
83.8	100	42.6872	15089	30	0.030711	0.000525	0.002637
107.5	40.56	35.1858	12247	59	3.3E-05	0.000758	0.00315
11	20.778	141.1576	54527	36	0.005901	0.000951	0.003801
11.2	100	45.2236	15705	57	4.68E-05	0.001022	0.00556
21.4	100	60.8762	24429	27	0.068138	0.001022	0.004643
19.9	100	40.9143	14112	49	8.56E-05	0.001308	0.006988
3.5	100	74.0386	25424	30	0.049752	0.001576	0.008857
27.7	100	73.1069	25023	12	3.440076	0.001576	0.008455
81.4	100	58.3334	19349	35	0.010747	0.001854	0.009804
14.5	100	37.9719	13320	47	0.000391	0.002012	0.01095
4	100	67.7951	23294	26	0.065645	0.002376	0.01545
6.2	100	73.0068	24986	19	0.62097	0.002843	0.01851
34.2	100	42.6323	15065	27	0.070428	0.00337	0.02319

43.5	100	56.4419	18728	28	0.051559	0.00337	0.02421
26.1	100	41.1537	14203	32	0.003547	0.004049	0.02921
14.9	100	41.9238	14474	37	0.001121	0.005618	0.03866
50.3	100	57.9553	19349	25	0.118001	0.00599	0.04541
18.4	100	43.1379	15272	29	0.033816	0.006872	0.04992
231.5	51.448	56.9458	18937	29	0.045174	0.007057	0.05236
229.8	72.166	57.4492	19140	33	0.017575	0.009514	0.07399
21.7	100	87.1428	30586	32	0.025446	0	0.001182
21.5	100	139.9686	54950	77	5.42E-07	0	4.51E-08
3.7	100	139.9485	54940	52	0.0002	0	7.05E-06
15.7	100	98.3261	37248	64	1.32E-05	0	5.22E-09
9.6	100	105.6524	40426	37	0.007907	0	0.001204
7.2	100	105.4053	40317	43	0.002079	0	0.000115
57	100	68.0964	24956	30	0.042832	0	2.39E-05
57.9	66.954	67.9079	24878	36	0.010316	0	1.41E-06
55.2	75.569	67.5938	24752	19	0.501129	0	0.001146
101.3	100	140.8478	54340	27	0.046421	0	2.68E-05
10.6	100	62.5251	22610	28	0.049368	0.00141	0.007232
67.3	100	62.4954	22596	27	0.06022	0.001677	0.008911
9.7	100	71.9617	24574	33	0.019629	0.001758	0.009233
7.2	100	85.354	32258	31	0.017148	0.002079	0.0116
180.5	100	17.8962	6070	43	0.001441	0.002843	0.01928
10.9	100	97.9794	37090	11	1.425125	0.003254	0.02123
1.8	100	97.8185	37020	15	0.903909	0.007785	0.05549
38.9	100	84.6544	31469	22	0.238936	0	0.000186
50.1	100	84.1516	31282	32	0.029648	0	1.83E-05
30.5	100	83.6468	31083	31	0.032272	0	0.000329
11.2	100	65.3551	24036	42	0.001924	0	2.94E-06
6.5	100	64.8765	23850	51	0.00023	0	2.89E-05
16.9	100	64.8524	23840	35	0.009102	0	3.69E-05
22.7	100	51.9827	18894	50	0.000161	0	0.000351
123.3	28.888	51.7169	18795	34	0.005846	0	0.001306
14.6	100	51.7172	18277	63	1.33E-05	0	0.000132
23.7	100	51.2134	18074	57	5.22E-05	0	0.000435
57.8	100	45.7989	15880	28	0.055272	0	0.000356
8.2	100	22.9548	8373	38	0.006019	0	0.000128
129.4	82.725	22.8299	8317	33	0.018088	0	7.34E-06
11.2	100	84.2012	31303	11	3.312943	0.000951	0.003894
6.8	100	67.5322	27283	24	0.154993	0.000951	0.003687
6.3	100	84.705	31491	17	0.784971	0.001022	0.005508
23.8	100	52.2195	18987	39	0.003286	0.001576	0.008345
10.8	100	64.3485	23649	12	2.207131	0.002079	0.01167
9.6	100	50.9855	20182	28	0.052919	0.002079	0.01186
9.9	100	27.9683	9265	43	0.001677	0.002177	0.0124
3.1	100	65.3793	24046	25	0.09863	0.002257	0.01269
30.3	100	45.295	15678	19	0.455919	0.00496	0.03471
23.6	100	45.3614	15705	14	1.348465	0.00496	0.03459
44	100	51.3619	18139	32	0.020012	0.006654	0.04867
7.3	100	83.6973	31103	11	3.030896	0.00861	0.06505
8.9	100	101.0621	36117	34	0.015577	0	0.000577
10.8	100	76.3122	26317	47	0.000519	0	0.000474
23.6	100	76.2205	26281	73	1.39E-06	0	1.31E-06

14.1	100	75.8077	26119	61	2.16E-05	0	6.13E-05
21.6	100	81.3337	30533	35	0.014187	0	0.000486
89.5	35.648	50.5442	19989	87	8.61E-08	0	1.38E-07
19.6	47.22	50.5883	20009	79	5.53E-07	0	2.39E-06
38.2	49.436	51.0483	20209	72	2.93E-06	0	6.46E-06
12.3	100	52.2786	20746	75	1.33E-06	0	9.73E-06
10.4	100	66.4131	26796	56	5.49E-05	0	0.000886
49	100	140.6041	54193	43	0.000859	0.000951	0.004445
13.5	100	41.7666	13128	31	0.025942	0.000951	0.003682
63.4	100	50.0416	19770	24	0.176582	0.001022	0.004827
14.7	100	97.1496	37315	42	0.002346	0.001114	0.005883
78.8	15.485	140.7066	54252	28	0.016917	0.001576	0.008809
6.3	100	39.9608	12493	33	0.02288	0.001758	0.009012
4.1	100	37.2712	11551	49	0.000444	0.001758	0.008996
7.3	100	41.7332	14908	24	0.180432	0.002376	0.01509
7.7	100	38.5828	14872	21	0.280609	0.00337	0.02191
30.3	100	39.76	12421	23	0.230472	0.00599	0.04521
6.4	100	96.6463	37103	33	0.018092	0.007636	0.05457
24.3	100	39.9002	15412	23	0.20323	0.007636	0.05498
70.4	100	18.4134	5649	26	0.073408	0	0.000404
56.7	100	18.7542	6122	20	0.348688	0	0.000943
2.4	100	121.5418	44578	65	1.09E-05	0	2.37E-06
1.1	100	18.1813	6212	50	0.000121	0	1.52E-05
20.7	100	18.167	6205	52	7.66E-05	0	1.09E-06
302.6	100	17.4171	5814	39	0.005259	0	6.71E-06
18	45.05	68.6179	25181	54	0.000103	0	6.57E-06
50	100	61.9248	24895	31	0.032678	0	0.000333
200.9	100	19.4263	6402	12	2.687089	0.000868	0.003399
19.1	100	19.461	6419	22	0.25981	0.000951	0.004577
36.8	100	72.1971	24664	47	0.000563	0.001022	0.005558
200.9	100	19.4294	6404	12	2.813728	0.001498	0.00756
14.9	100	35.2371	12270	35	0.010893	0.002719	0.01664
85.7	17.67	13.1325	3671	37	0.005823	0.002777	0.01742
46.4	100	20.3184	6767	12	2.992649	0.00337	0.02204
15.2	100	71.7439	24488	19	0.319089	0.00861	0.06528
189.2	4.607	13.1189	3657	18	0.44476	0.009655	0.07665
4.7	100	106.0222	43553	65	9.78E-07	0	1.93E-10
0.8	100	105.6599	43387	30	0.00296	0	0.000129
4.3	70.358	105.5142	43324	72	2.19E-07	0	1.69E-10
49	100	23.9098	8187	30	0.025393	0	0.000445
41.1	5.872	140.5953	55268	108	3.58E-10	0	8.09E-12
55.7	12.815	140.2691	55092	101	2.94E-09	0	3.46E-14
46.7	50.443	140.2732	55095	82	2.46E-07	0	5E-10
39.9	100	140.2754	55096	100	4.22E-09	0	6.57E-14
36.9	36.979	140.6642	52363	101	1.59E-09	0	9.01E-12
21.7	100	140.3662	52200	71	3.03E-06	0	3.16E-08
18.4	100	113.6391	42484	52	0.000246	0	0.000233
51.2	100	114.142	42706	59	4.86E-05	0	6.12E-06
23.6	100	64.9732	24036	33	0.022407	0	0.000423
17.1	100	65.4761	24252	36	0.012175	0	0.000959
56.5	61.579	140.6573	54225	95	7.85E-09	0	4.13E-10
366.4	25.735	140.654	54222	93	1.36E-08	0	1.56E-11

17.8	100	142.7729	49689	78	6.8E-07	0	5.83E-10
11	100	17.57	5080	37	0.00262	0.000141	0.001571
6.5	100	72.224	24673	56	5.92E-05	0.00065	0.002714
19.6	100	79.4157	28204	33	0.017499	0.000951	0.003914
5.3	100	67.4532	25058	45	0.001034	0.000951	0.00415
7.2	100	18.1539	5291	37	0.004816	0.001022	0.005691
28.3	100	79.9277	28437	33	0.018463	0.00121	0.006523
15.5	100	79.8943	28422	35	0.011521	0.001308	0.007184
24.9	76.038	62.9786	23220	56	3.07E-05	0.001308	0.007038
79.3	28.657	67.8749	25232	36	0.006842	0.001498	0.00782
13.3	100	62.9831	22929	47	0.000289	0.001758	0.00908
12.7	100	64.4637	23827	22	0.290344	0.002079	0.0119
47.1	100	78.1821	27668	26	0.094394	0.002543	0.01567
9	31.881	114.1116	42693	33	0.019296	0.002777	0.01703
106.2	30.711	67.3722	25026	26	0.084754	0.002777	0.01772
6.5	100	64.9635	24032	32	0.024944	0.00337	0.02189
6.7	100	18.7607	5495	35	0.012306	0.00337	0.02355
23	100	83.2077	29021	25	0.104205	0.003736	0.02766
23.6	100	58.9604	19727	23	0.158346	0.003736	0.02661
0	100	129.392	50412	13	0.797555	0.003967	0.0289
11.4	100	72.4048	24743	36	0.005712	0.00496	0.03474
13	100	83.1247	28988	37	0.00685	0.005687	0.03939
20.3	100	61.2267	20416	24	0.111073	0.00599	0.0444
0	100	18.1917	5305	42	0.001629	0.00822	0.06028
2.4	100	94.8375	35481	56	7.61E-05	0	0.000717
17.2	100	106.8462	43906	78	6.49E-07	0	1.12E-07
7.8	30.539	106.8165	43894	49	0.000467	0	0.000447
18.5	65.284	86.7889	32429	54	0.000124	0	0.000576
19.7	17.629	86.7726	32422	46	0.000778	0	0.000358
2.6	100	85.3238	31813	40	0.000921	0	0.000777
15.4	50.226	92.2426	34716	57	2.31E-05	0	1.31E-06
28.7	42.377	91.8422	34548	62	7.61E-06	0	1.51E-06
7.1	55.337	140.6801	54238	89	2.35E-08	0	4.58E-10
421.4	27.074	140.677	54236	80	2.2E-07	0	1.78E-08
1.7	100	139.692	54258	56	8.01E-05	0	0.0002
26.4	81.452	121.876	46079	45	0.000299	0	0.001268
14.5	100	121.9335	46106	60	8.86E-06	0	0.000218
15.8	100	86.6883	31240	43	0.001727	0	0.00047
71.2	77.079	86.6981	31244	69	4.01E-06	0	3.65E-05
22.1	100	87.2556	31482	56	8.05E-05	0	0.000448
41.8	100	86.1963	31023	62	2.21E-05	0	2.6E-05
2.2	5.522	141.0491	54461	93	8.29E-09	0	2.45E-10
124.4	2.839	141.0478	54459	108	2.29E-10	0	8.46E-11
13.6	100	141.4356	54693	51	8.47E-05	0	1.22E-10
62.8	31.568	141.3909	54668	61	3.35E-05	0	1.14E-07
8.4	100	107.3694	44127	39	0.005216	0.00065	0.002693
29.5	100	59.3501	19869	25	0.112169	0.000758	0.002956
6	100	105.4742	39097	49	0.000173	0.000951	0.004322
8.3	100	91.7434	34506	25	0.034481	0.000951	0.004193
2.9	100	92.3452	34754	42	0.000625	0.001022	0.005213
13.9	100	64.388	21876	55	8.46E-05	0.001022	0.005419
3	100	95.3433	35691	41	0.002918	0.001022	0.004965

7.3	100	86.1794	31017	34	0.012151	0.001576	0.008243
7.1	100	87.4066	32564	35	0.009474	0.001945	0.009928
22.7	100	51.0699	18988	52	0.000126	0.002376	0.01526
69.6	100	121.4262	45875	25	0.027223	0.002777	0.01766
32.4	100	29.7832	9955	34	0.015701	0.004266	0.03077
2.5	100	86.2677	32204	20	0.279043	0.004643	0.0332
34.3	100	31.2427	10692	43	0.001845	0.004803	0.03398
9.7	100	64.8937	22088	42	0.002046	0.007057	0.05262
24	100	15.9056	4553	39	0.004657	0.00728	0.05288
185.2	37.977	56.412	21096	41	0.002952	0.00971	0.07747
223.8	4.005	24.6205	8011	49	0.000103	0	0.000183
15.6	100	76.7155	27023	33	0.023539	0	4.07E-05
34.5	45.418	77.6445	27427	70	4.84E-06	0	5E-09
57.8	36.059	77.7302	27466	46	0.001098	0	1.09E-08
5	100	77.3731	27308	53	0.000233	0	2.3E-05
32.4	100	77.1406	27202	69	5.67E-06	0	1.32E-09
96.8	95.75	77.2201	27238	40	0.003981	0	5.22E-08
17.6	100	23.1	7871	56	1.68E-05	0	0.00056
3.6	100	132.1848	48737	92	9.54E-09	0	1.39E-10
5	100	132.6902	48933	91	1.14E-08	0	4.71E-11
15.9	100	96.4669	34328	46	0.000786	0	7.36E-05
25.4	20.972	96.3897	34297	72	2.4E-06	0	2.34E-06
28	59.755	120.5193	44142	68	6.59E-06	0	7.9E-08
37.9	100	25.1774	7551	40	0.000748	0	0.001476
13.7	100	25.3203	7594	53	3.78E-05	0	0.001278
3.7	100	138.8665	51507	51	0.000103	0	2.62E-06
17.9	100	112.4719	43745	31	0.030067	0	0.001304
48.9	100	140.18	53984	28	0.062586	0	0.000461
5.9	100	128.7642	49017	42	0.002685	0	0.000668
37.4	100	83.1451	29763	50	0.000399	0	0.00014
6.6	100	78.152	27655	36	0.012541	0	0.000613
17.5	100	78.2332	27693	14	1.566556	0	0.000718
4.1	100	111.961	43511	37	0.008261	0.000273	0.001835
1.9	100	95.9178	34094	55	6.83E-05	0.000273	0.001972
5.8	100	121.0256	44350	37	0.007867	0.000525	0.002216
17.8	100	24.4032	7929	45	0.000223	0.000758	0.002791
3.9	100	129.2736	49245	28	0.068134	0.000951	0.00447
122.7	100	26.642	9949	38	0.005837	0.000951	0.004237
0	100	127.304	48390	31	0.032016	0.000951	0.004586
31.9	100	25.6475	7624	29	0.010244	0.00121	0.006841
17.8	100	22.4346	7180	46	0.000173	0.00121	0.006608
107.6	100	23.4111	8567	41	0.001766	0.00141	0.007283
4.4	100	120.0161	43930	30	0.042748	0.001498	0.007872
25.5	100	73.409	25599	23	0.215019	0.001945	0.01
12	100	83.7501	30000	36	0.011902	0.002012	0.01123
1	100	127.8113	48590	16	1.173183	0.006991	0.05152
8	100	95.9637	34112	20	0.376691	0.007785	0.05567
1.2	100	120.5466	44153	23	0.195137	0.008746	0.06655
2.8	100	111.8241	41712	9	4.857019	0.009858	0.08176
53.6	76.286	69.6783	25750	65	1.22E-05	0	1.93E-06
72.8	45.772	69.665	25744	32	0.02281	0	0.000454
47.3	100	77.4056	27322	52	9.06E-05	0	0.000235

15	100	82.4842	33600	64	1.69E-05	0	5.55E-07
4.8	100	81.9695	33389	38	0.006683	0	0.000127
62.9	31.856	122.8432	45109	63	1.64E-05	0	5.84E-06
16.3	66.657	122.8814	45125	78	5.75E-07	0	7.71E-08
13	100	122.1348	47392	63	1.97E-05	0	8.32E-07
9.2	100	110.2791	41059	40	0.002878	0	0.000195
7.1	100	110.415	41115	54	0.000122	0	8.35E-05
15.7	36.553	110.7829	41272	43	0.00151	0	9.78E-05
92.6	77.993	140.2341	54010	67	6.08E-06	0	2.94E-12
133.5	90.932	140.2323	54009	91	2.44E-08	0	1.28E-13
27.1	100	68.5198	27728	27	0.074874	0	0.001018
44.7	100	68.5036	27720	67	8.12E-06	0	1.96E-06
4	59.668	141.082	55534	27	0.064447	0.000141	0.001634
8.1	100	122.1587	47402	32	0.024732	0.000868	0.003301
8.2	100	110.4371	41124	32	0.017839	0.000951	0.003758
66.1	100	48.415	19061	42	0.000957	0.001114	0.006252
16.8	100	5.932	1686	24	0.07762	0.001576	0.008162
13.5	100	18.6438	6447	37	0.009438	0.002315	0.01327
10.8	100	62.7018	21210	19	0.236561	0.002376	0.01486
22.4	100	69.1633	25547	16	0.904215	0.002719	0.01668
6.8	100	76.9582	27123	29	0.016286	0.002843	0.01833
18.3	100	76.9048	27099	20	0.130298	0.00337	0.02383
103.9	100	46.2788	16077	30	0.038086	0.00337	0.02237
3.5	100	68.0154	27507	19	0.492846	0.008171	0.06001
1.6	100	131.2987	53854	51	2.89E-05	0	0.000533
45.6	100	70.4416	23998	79	2.44E-07	0	1.94E-06
13.8	100	31.0091	9462	56	0.000103	0	2.14E-06
9.6	100	30.5017	9287	31	0.036652	0	0.000769
27.4	100	81.956	28507	65	1.57E-05	0	5.88E-08
25.9	100	81.452	28324	50	0.000473	0	4.38E-06
11.1	100	82.4634	28707	52	0.000318	0	7.9E-05
18.7	100	84.9385	32079	55	0.000128	0	1.3E-07
239	51.42	16.8353	5498	54	0.000114	0	0.001409
48.8	100	31.0069	9461	17	0.97598	0.000141	0.001795
25.7	100	77.2703	28577	46	0.001024	0.000273	0.00192
10.4	100	88.8559	33792	38	0.00421	0.000273	0.002017
14.2	100	92.5773	33638	36	0.006804	0.000758	0.002851
43.2	100	30.5038	9288	18	0.762936	0.000951	0.003623
12.2	100	70.4919	24019	25	0.062109	0.00121	0.006611
3.5	100	65.971	26605	18	0.537714	0.002719	0.01672
5.5	100	84.4958	30316	32	0.01819	0.004192	0.03022
13.1	100	77.4704	28663	25	0.12986	0.007213	0.05274
26.8	100	16.8417	5214	36	0.012105	0	2.64E-06
88.9	100	16.8287	5208	37	0.008495	0	1.44E-06
52.2	100	49.9775	17547	45	0.001125	0	0.000372
33.6	100	48.0393	16782	48	0.000498	0	0.000288
113.8	100	38.0669	13354	28	0.063966	0	0.000178
32.7	100	85.651	30794	49	0.000571	0	5.02E-07
39.9	100	57.2663	20733	44	0.001314	0	0.001257
106.3	49.509	67.0484	24499	52	0.000291	0	0.000718
79.6	100	64.2215	21582	36	0.010678	0.000758	0.002918
50.9	100	37.061	12981	27	0.084908	0.000868	0.003324

139.3	69.098	66.9478	24461	26	0.112766	0.001758	0.009325
28	100	66.7177	24368	33	0.020606	0.002315	0.01301
4.8	100	121.1095	47298	38	0.004648	0.002376	0.01483
26.5	100	56.676	21217	35	0.004636	0.002843	0.01843
22.1	100	65.6603	24329	22	0.294315	0.00337	0.02189
9.4	100	65.4643	24246	34	0.019778	0.003736	0.02676
16.1	100	73.406	27315	24	0.135193	0.004581	0.03251
7.4	100	121.6124	47501	36	0.009446	0.005196	0.0356
14.8	100	63.9868	21488	24	0.152344	0.005792	0.04212
65.9	100	45.7949	15932	38	0.003173	0	0.000541
9	100	45.7654	15920	49	0.000223	0	0.000856
39.5	100	35.5825	12661	49	0.000585	0	0.000847
1.6	100	111.0808	42674	42	0.001348	0	5.99E-05
11.6	100	114.1841	43981	70	3.8E-06	0	1.44E-06
10.5	100	118.2799	44492	46	0.001086	0	0.000655
18.2	100	118.3262	44515	53	0.000196	0	0.000352
25	100	50.5977	18354	42	0.001909	0.000273	0.002055
14.4	100	31.4657	9620	26	0.014434	0.000273	0.001823
228.8	19.86	19.1422	6687	28	0.045934	0.000758	0.002854
46.9	100	59.8709	21564	37	0.004983	0.000951	0.003903
39.7	100	124.0453	47010	42	0.002946	0.002543	0.01592
4.5	100	109.4456	40714	34	0.013379	0.003674	0.02653
7.9	100	49.512	16072	28	0.066702	0	0.000331
10.7	100	50.0547	16281	40	0.004456	0	0.000512
8.5	100	52.2412	17120	33	0.020522	0	0.000593
15.8	65.298	48.9569	15844	42	0.002528	0	0.000201
25.4	100	56.3767	18704	39	0.004956	0	2.07E-07
24.9	100	56.9317	18930	27	0.086039	0	9.03E-05
21.3	100	57.0824	18995	48	0.000593	0	1.43E-06
9.9	100	53.2994	17505	36	0.010175	0	0.000301
14.5	100	55.1054	18204	44	0.001604	0	5.18E-05
10.1	100	55.6118	18403	33	0.022372	0	0.000223
0	100	74.5862	27646	60	4.43E-05	0	0.000248
0	23.367	74.1169	27463	80	4.62E-07	0	2.54E-09
0	65.235	74.0828	27450	95	1.42E-08	0	6.84E-07
8.6	100	43.4083	13726	37	0.006369	0	0.000556
8.2	100	47.7874	15374	39	0.005291	0	0.000105
8.8	100	47.2399	15171	45	0.001454	0	2.18E-05
6.6	100	44.7897	14234	47	0.000795	0	8.21E-06
94.4	100	45.2952	14418	58	6.45E-05	0	1.26E-08
3.5	100	45.3867	14453	80	4.35E-07	0	1.16E-09
1.3	100	76.9802	27133	43	0.001875	0	0.00022
32.6	100	61.5921	20764	78	7.46E-07	0	9.98E-10
21.3	59.057	61.0871	20567	64	1.71E-05	0	3.45E-08
33.8	11.075	60.5863	20374	70	3.85E-06	0	1.64E-09
10.3	100	60.7349	20431	87	8.19E-08	0	7.1E-11
0	100	59.0637	19767	77	6.2E-07	0	2.12E-07
9.7	100	59.4098	19890	50	0.00042	0	1.22E-05
9.1	100	58.905	19706	40	0.004408	0	0.000106
26.2	2.582	60.0796	20157	74	1.83E-06	0	1.37E-08
64.3	67.581	60.1601	20194	64	1.86E-05	0	2.26E-09
17	17.239	60.2309	20226	88	7.08E-08	0	4.42E-11

278	100	60.0638	20149	47	0.000868	0	6.25E-08
0.4	100	59.5673	19950	60	3.07E-05	0	2.34E-05
22.8	100	56.3824	18592	36	0.010862	0	0.000163
2.1	100	50.5509	16381	28	0.070701	0	0.000544
35.5	100	50.4592	16344	74	1.76E-06	0	7.84E-10
9.6	100	49.9553	16148	51	0.000302	0	5.51E-05
0.4	100	66.1211	22307	53	0.000157	0	0.00012
21.8	100	66.4338	22434	58	4.95E-05	0	3.73E-07
9	100	65.9311	22235	35	0.009401	0	0.000245
0.8	100	66.6238	22509	45	0.001041	0	0.000444
20.7	100	141.2692	55638	89	2.87E-08	0	5.19E-13
11.6	79.604	141.267	55637	73	1.33E-06	0	7.94E-11
40.9	67.678	77.5213	28687	87	7.62E-08	0	1.28E-10
11.4	17.399	77.279	28582	75	1.29E-06	0	1.27E-06
27.3	2.931	77.2678	28576	97	7.64E-09	0	5.89E-12
31.6	52.218	77.0141	28460	42	0.002798	0	1.16E-05
318.7	100	141.1629	52635	83	1.54E-07	0	5.65E-17
14.9	100	141.1721	52640	25	0.091685	0	3.02E-05
7.3	25.353	77.7768	28807	92	2.32E-08	0	3.46E-13
0.4	100	73.3476	26985	48	0.000736	0	0.000129
44.9	17.061	141.1606	52634	79	3.44E-07	0	2.95E-13
9.8	100	140.9451	52517	36	0.010616	0	0.000418
33.7	100	24.3028	7268	24	0.131191	0	0.001487
56.1	100	49.5631	17431	56	7.59E-05	0	0.001308
57.4	100	140.4113	52223	84	1.81E-07	0	1.14E-07
92.5	7.596	140.4341	52234	76	1.01E-06	0	9.94E-08
264	100	140.4379	52236	89	5.51E-08	0	4.63E-09
19.8	39.444	102.7654	39750	52	0.00018	0	1.68E-06
23.8	81.391	102.7591	39747	73	1.43E-06	0	6.94E-07
45.3	4.548	102.7014	39723	56	7.44E-05	0	4.96E-08
17.1	89.901	102.4234	39596	64	8.45E-06	0	6.15E-06
21.2	15.431	102.262	39521	48	0.00055	0	6.69E-06
18.3	41.328	102.2493	39515	83	1.49E-07	0	8.47E-08
26.1	7.056	103.204	39944	59	3.84E-05	0	2.08E-07
14.7	100	103.2627	39967	72	2.03E-06	0	1.27E-06
9.2	100	105.2285	40761	54	0.00012	0	3.35E-06
11.7	100	104.7232	40564	55	9.83E-05	0	4.26E-06
7	100	104.2164	40360	61	2.69E-05	0	1.67E-06
37.4	44.078	103.7104	40148	76	7.29E-07	0	2.21E-09
21.3	98.47	103.2687	39970	40	0.00318	0	4.88E-06
48	2.068	102.1928	39492	55	8.82E-05	0	9.91E-08
8.8	100	101.9112	39366	61	1.48E-05	0	6.52E-05
3.3	100	101.1383	39043	33	0.015361	0	0.000628
53.4	100	140.4327	54106	67	7.29E-06	0	1.4E-07
36.1	100	62.8044	22765	37	0.008002	0	0.000392
103.3	1.839	58.5008	20985	32	0.022807	0	0.001462
29.4	19.634	58.5266	20997	62	2.51E-05	0	0.000133
23.2	16.596	59.031	21217	77	8.13E-07	0	1.68E-05
88.2	2.186	59.0065	21205	32	0.025355	0	0.000825
2.9	100	55.3794	19735	69	4.7E-06	0	0.000162
11	100	64.9538	26180	49	0.000434	0	5.66E-06
0	83.324	123.5543	43712	70	4.63E-08	0	0.001356

91.8	100	61.9292	24897	35	0.009044	0	1.52E-06
4.4	100	141.0554	50470	67	7.82E-06	0	2.37E-07
108.8	100	19.1062	6669	38	0.006571	0	0.000807
12.1	100	66.3507	24809	36	0.000666	0.000141	0.00158
8.8	100	48.4729	15645	27	0.076248	0.000141	0.001563
64.5	100	18.735	5622	55	0.000117	0.000141	0.001643
1.3	100	74.6181	27659	43	0.001956	0.000273	0.002042
66.4	100	41.8225	15295	48	0.000566	0.000525	0.002545
61.8	100	23.1774	8472	24	0.13661	0.000525	0.002302
51.5	100	57.9913	20775	21	0.285826	0.000525	0.002421
2.3	100	59.5481	19943	29	0.044827	0.000525	0.002624
5.3	100	58.1493	21290	48	0.000602	0.00065	0.002751
15.8	100	62.1101	24979	16	0.783858	0.000758	0.002925
111.9	100	41.7342	14410	56	8E-05	0.000758	0.002962
0.4	100	61.7385	20817	29	0.053978	0.000868	0.003573
73	100	24.7121	8500	18	0.549913	0.000868	0.003351
75.6	100	55.363	19728	35	0.011568	0.000951	0.004536
56.6	100	13.1685	3599	55	0.000102	0.000951	0.004398
67.6	100	52.3267	17045	56	9.12E-05	0.000951	0.003668
92	11.013	41.318	15094	51	0.000264	0.000951	0.004368
2.3	100	43.307	13690	31	0.028849	0.000951	0.004478
16.6	100	54.1872	17844	22	0.283907	0.001022	0.004873
44.9	100	52.5148	20851	19	0.528687	0.001022	0.005106
0	100	50.048	16184	29	0.057405	0.001022	0.00512
141.8	24.207	50.0672	17632	57	7.2E-05	0.001022	0.005773
248.5	100	51.2978	16661	56	7.71E-05	0.001114	0.006044
75.3	100	50.4037	17726	22	0.252973	0.001114	0.006286
0.8	100	120.8279	42864	49	6.12E-06	0.001114	0.006132
25.3	100	13.1254	3548	55	0.000112	0.001114	0.00588
10.7	100	76.7577	28348	17	0.75459	0.001114	0.006366
5.3	100	64.4517	25969	23	0.150036	0.001114	0.006189
41.7	100	41.4272	14797	60	2.42E-05	0.001308	0.00704
255.4	50.377	50.2867	16274	56	7.75E-05	0.00141	0.007347
6.3	100	52.7941	17328	29	0.052043	0.001498	0.007851
20.3	100	13.2182	3563	36	0.008014	0.001498	0.007492
27	100	40.8077	14886	42	0.002041	0.001498	0.007852
27.8	100	45.854	15956	43	0.001542	0.001576	0.00835
0.4	100	44.8807	14267	25	0.14212	0.001854	0.009824
52.8	100	57.0647	20407	17	0.655641	0.002012	0.01122
5.6	100	67.5717	22879	21	0.334886	0.002079	0.01221
10.6	100	58.4012	19521	26	0.110892	0.002315	0.01373
167.9	6.1	49.852	17547	57	7.13E-05	0.002315	0.01335
33.9	100	17.9809	5618	31	0.025492	0.002315	0.01325
18.8	100	30.1485	11365	37	0.007311	0.002315	0.01345
85.3	100	40.9766	14137	60	2.33E-05	0.002376	0.01523
1.5	100	72.8611	25373	32	0.023897	0.002543	0.01624
28.2	100	58.1368	21284	20	0.327723	0.002543	0.01612
52.8	100	41.93	14983	60	2.25E-05	0.002719	0.01679
5.6	100	62.5793	20930	17	0.670461	0.002777	0.01703
7.1	100	66.3054	22682	21	0.343727	0.002843	0.01883
20.5	100	57.6323	21084	21	0.285561	0.003096	0.02019
176.4	1.071	50.5819	16394	57	6.98E-05	0.003674	0.02542

2.9	100	101.7584	39297	14	1.144513	0.003892	0.02823
0.4	100	61.2359	20624	18	0.629434	0.003967	0.02886
0.9	100	77.792	28815	20	0.388557	0.004192	0.03007
2.4	100	103.7724	40176	25	0.110058	0.004504	0.03207
201	1.051	50.7933	16473	57	7.53E-05	0.004803	0.03418
31.3	100	54.8554	19536	20	0.386212	0.004803	0.03399
1.2	100	102.9454	39826	24	0.084153	0.005325	0.03695
157	100	51.802	16849	38	0.005208	0.005556	0.0381
1.8	100	106.2895	41201	20	0.327622	0.00599	0.04496
13.9	100	57.5373	19040	19	0.478905	0.006371	0.0465
52.4	100	45.8024	14616	5	13.78874	0.006428	0.04698
21.2	100	142.7938	49699	12	2.429005	0.006428	0.04696
0	100	102.4573	39612	19	0.22279	0.007836	0.05651
3.4	100	39.4188	13887	30	0.002432	0.007969	0.05826
41.3	100	40.745	12771	41	0.001616	0.0085	0.06378
8.4	100	83.5074	31031	65	8.07E-06	0	9.17E-07
1.5	100	83.0026	30846	39	0.003554	0	0.00055
41.8	100	61.9521	22726	50	0.000412	0	1.38E-05
176.2	100	29.7766	9940	27	0.036387	0	0.000385
87.5	58.906	29.5153	9844	64	8.24E-06	0	4.33E-05
204.6	1.479	29.1783	9715	40	0.002451	0	0.00037
65.2	100	37.2643	12750	20	0.384054	0	0.001143
22.9	100	68.3849	23522	51	0.000359	0	0.000343
104.7	100	30.1225	10551	31	0.017966	0	0.000834
4.5	100	97.4068	34692	35	0.006245	0	0.000111
10.2	100	89.1531	31415	52	0.000287	0	1.16E-05
67.9	20.986	88.823	31279	73	2.4E-06	0	1.13E-10
46.1	36.558	105.3527	37843	71	2.19E-06	0	3.77E-06
54.7	21.426	105.3515	37842	59	3.2E-05	0	1.31E-05
54.1	27.133	88.6446	31199	70	4.58E-06	0	6.54E-08
2.2	100	84.963	29701	51	0.000343	0	3.79E-05
38.2	100	83.2834	29052	54	0.000177	0	0.0002
57.7	18.817	82.9768	28927	90	4.76E-08	0	5.18E-07
85.2	1.849	82.7756	28841	48	0.000795	0	0.000783
63.8	13.642	82.4732	28711	90	4.6E-08	0	1.72E-07
6.8	100	87.1236	32570	45	0.000774	0	3.41E-05
7.9	100	82.2799	30582	36	0.006006	0	0.001201
15.5	83.672	82.2638	30575	77	5.77E-07	0	2.41E-07
11.6	100	69.0388	25226	44	0.001733	0	0.001208
5.2	100	98.1898	37185	53	0.000164	0	3.32E-05
18.8	100	95.3957	36002	42	0.001985	0	0.000101
9.6	32.944	94.8586	35777	89	4.15E-08	0	9.36E-08
10.9	90.631	94.3554	35556	36	0.007855	0	0.001165
0.8	100	112.23	43158	47	0.000654	0	0.001119
12.5	100	103.7631	39632	54	0.000105	0	0.000141
10	100	104.1645	39795	51	0.000205	0	0.000382
172	68.845	14.8614	4489	19	0.568499	0	0.000562
72	100	39.8986	13954	45	0.001004	0	0.000236
88	73.106	39.397	13755	45	0.00116	0	0.000784
210.2	71.516	16.1587	5175	25	0.093803	0	0.000734
106	36.347	37.0359	12437	41	0.002488	0	0.000584
22.5	46.021	76.936	28955	51	0.000244	0	0.000609

39.2	49.782	76.9442	28959	73	1.53E-06	0	3.82E-07
28.1	7.917	75.8908	28519	67	6.54E-06	0	4.01E-06
32.4	37.66	75.9076	28527	72	1.88E-06	0	4.36E-07
13.7	100	79.2378	29922	70	3.33E-06	0	7.58E-06
37.2	17.689	75.3979	28306	80	3.3E-07	0	7.67E-08
3.8	100	73.6639	27582	56	4.86E-05	0	0.000238
13.5	100	74.8835	28079	48	0.000589	0	6.84E-05
14.3	100	74.8943	28084	70	3.28E-06	0	4.85E-07
25.2	6.714	75.3862	28301	60	2.88E-05	0	2.99E-05
32.8	100	74.3812	27879	42	0.001841	0	0.000274
31.5	9.794	60.1176	24082	56	0.000103	0	1.67E-06
8	100	65.0286	26215	46	0.000926	0	0.000326
24	48.09	60.4262	24228	81	3.08E-07	0	7.23E-09
67.3	39.152	60.6296	24322	61	2.92E-05	0	8.86E-07
15	100	60.9354	24456	73	1.8E-06	0	1.54E-07
7.1	100	63.3337	25494	31	0.018898	0	2.9E-05
7.6	100	62.8267	25285	46	0.000554	0	2.54E-07
13.5	100	62.6598	25213	48	0.000603	0	3.09E-05
13.5	100	63.4511	25546	33	0.019397	0	0.001098
6.1	100	63.9631	25762	38	0.00639	0	0.000561
13	100	63.8367	25713	11	1.571736	0	0.000868
11.9	100	61.6377	24771	52	0.000224	0	1.74E-05
65.2	100	61.1345	24549	64	1.56E-05	0	9E-07
16.9	100	62.1464	24997	56	0.000104	0	3.72E-06
42.8	13.883	80.7425	28783	60	4.23E-05	0	0.000264
18.2	13.002	28.2075	10570	39	0.006137	0	0.000256
40.7	59.936	28.2619	10595	34	0.017458	0	5.54E-05
141.2	100	26.9988	10085	41	0.001384	0	0.000271
9	100	80.6862	28027	43	0.000528	0.000141	0.001599
22.2	100	82.2698	28628	38	0.006343	0.000141	0.001678
109.7	6.139	39.3169	13722	32	0.018402	0.000273	0.001868
69.5	100	53.5851	17526	48	0.000464	0.000273	0.002125
13.4	100	16.2154	4472	22	0.183522	0.000273	0.001842
7.2	100	70.2742	23933	63	2.17E-05	0.000273	0.001951
44.7	100	40.3523	14135	23	0.145724	0.000525	0.002182
31.2	100	16.1623	4656	23	0.180531	0.000525	0.00253
7.5	100	59.9177	23999	39	0.005046	0.000525	0.002238
6	100	105.8523	38045	37	0.004907	0.00065	0.002753
33.2	100	54.5066	19392	42	0.002465	0.000758	0.003006
85.2	5.259	39.8197	13921	26	0.080771	0.000868	0.003424
54	6.598	54.0023	19187	39	0.00479	0.000951	0.004348
67.9	8.063	55.0972	18100	41	0.003356	0.000951	0.004289
2.2	100	61.4478	22527	30	0.037149	0.000951	0.003709
11.7	24.352	94.4456	35592	23	0.155755	0.000951	0.004356
39.2	100	14.8882	4160	17	0.920266	0.001022	0.004954
102.1	100	36.5275	12232	23	0.148083	0.001022	0.005483
62.4	100	40.8572	14345	20	0.350658	0.001022	0.005656
69.3	50.212	80.8002	28808	27	0.083917	0.001022	0.005243
7	100	68.934	25184	59	5.25E-05	0.001114	0.006182
40.2	100	41.3702	14546	16	0.712646	0.001114	0.006261
18.8	100	68.8243	23710	62	2.76E-05	0.00121	0.006489
84.9	100	30.7926	9388	16	0.489291	0.001308	0.007109

12.6	100	16.15	4571	29	0.043705	0.001758	0.009342
7.5	100	104.8459	37623	29	0.03327	0.001758	0.009312
35.1	100	80.2994	28596	26	0.108803	0.002012	0.01063
24.9	100	59.2607	19836	27	0.120416	0.002079	0.01231
9.2	100	76.4016	28736	29	0.04507	0.002079	0.01158
63.1	100	29.6178	10345	17	0.352831	0.002079	0.01161
25.5	100	73.5652	27273	47	0.00047	0.002079	0.01138
48.4	100	30.2916	9209	21	0.168061	0.002257	0.01259
1.5	100	69.4377	25379	48	0.000772	0.002315	0.01368
24.4	100	29.9415	9074	27	0.037779	0.002376	0.01445
76.9	100	51.1167	16692	30	0.028895	0.002376	0.01473
86.5	100	16.5173	4805	1	10.31754	0.002376	0.01421
6.1	100	75.142	28195	38	0.003263	0.002376	0.01422
90.3	100	80.6072	30228	37	0.007019	0.002376	0.01476
12.1	100	19.6926	6510	49	0.000239	0.002376	0.01521
4.1	100	83.5285	31038	25	0.074952	0.002543	0.01595
4.3	100	95.9164	35914	25	0.092411	0.002719	0.01642
15	100	26.4972	8416	23	0.154714	0.002777	0.01819
0.4	100	84.7361	31502	33	0.008449	0.002777	0.01807
66.5	100	14.7706	4248	11	3.235773	0.002777	0.01817
85.6	79.972	54.0554	19208	26	0.096335	0.002777	0.01785
6.3	100	70.3953	23979	36	0.010075	0.002777	0.0179
13.9	100	54.5896	17903	30	0.03623	0.002777	0.01812
9.5	100	83.7869	29253	25	0.135596	0.002843	0.01905
3.8	100	94.9488	35813	22	0.192683	0.002843	0.01861
14	100	55.008	19593	32	0.024811	0.003096	0.02013
149	50.003	51.8436	18332	36	0.007995	0.00337	0.02167
127.4	1.429	53.0807	17337	47	0.00059	0.00337	0.02161
25	100	29.8196	9972	13	1.447122	0.003533	0.02447
0.4	100	85.2436	31703	35	0.00583	0.00382	0.02785
10.3	100	41.1902	12934	42	0.001097	0.00382	0.02794
14.5	100	53.4978	18986	25	0.109846	0.004124	0.0295
116.3	23.07	51.891	18352	31	0.025518	0.004192	0.03004
31	100	75.8602	28505	20	0.284486	0.004424	0.03175
23.9	100	52.7989	17225	33	0.016026	0.004643	0.03362
30.4	100	38.8158	13529	18	0.468195	0.004643	0.03349
32.9	100	60.8055	20255	15	1.707911	0.005325	0.03727
5.8	100	97.1932	36755	9	3.858318	0.00573	0.041
1.8	100	96.0071	35951	14	1.263937	0.005792	0.04191
5.8	100	77.3762	29126	21	0.225767	0.006428	0.04672
2.2	100	97.4642	34714	9	2.02431	0.006721	0.04895
1.9	100	92.0983	33451	11	1.432298	0.007636	0.05524
7.8	100	68.9768	25201	19	0.625089	0.0081	0.05955
0	100	84.8904	31567	14	0.717912	0.008293	0.06214
22	100	30.822	10081	13	2.379477	0.008361	0.06304
1.6	100	97.9077	34886	12	1.135098	0.00861	0.06583
0.6	100	81.75	30371	10	2.580956	0.009514	0.07629
14.4	100	75.6588	28414	26	0.086429	0.009818	0.07881
163.2	2.232	80.1062	30007	40	0.002613	0.009858	0.08064
0	100	15.0342	4180	68	1.56E-07	0	0.000112
0	100	17.0124	4876	79	2.06E-07	0	1.66E-05
0	100	15.5038	4348	65	7.28E-07	0	0.00019

8.7	100	101.9199	39370	78	6.99E-07	0	1.81E-06
136.6	100	39.5232	13809	28	0.049486	0	4.63E-05
36.3	100	42.3383	14482	48	0.000151	0	0.000132
94.6	100	62.0563	24954	40	0.004359	0	0.000645
31.6	100	53.5234	21291	35	0.011817	0	0.000697
59.6	100	20.9054	6498	39	0.00147	0	0.000618
23.7	100	50.8804	20136	42	0.003153	0.000141	0.00167
53	100	45.649	17878	35	0.009364	0.000525	0.002474
0	100	13.5445	3675	58	1.29E-05	0.000868	0.003269
1.9	100	35.8936	12530	56	4.72E-05	0.000951	0.004345
0.4	100	30.412	10210	48	4.61E-05	0.001758	0.009113
86.7	100	65.034	23908	37	0.003361	0.00337	0.02272
3.6	100	17.375	5006	41	0.000945	0.00337	0.02358
21	100	38.3498	13654	48	0.000519	0	0.000984
10.8	100	100.5054	35900	32	0.023494	0	0.000382
23.2	100	100.4019	35859	58	6.75E-05	0	1.33E-05
30.4	100	62.3152	22519	45	0.001283	0	3.56E-05
43.3	13.859	140.5494	55242	89	4.06E-08	0	2.52E-08
8.5	100	94.2129	36080	41	0.00322	0	0.000583
71.7	100	62.0538	22567	50	0.000408	0	0.000115
22	100	37.7589	12936	35	0.012157	0.000141	0.001657
28.6	100	61.5453	22367	43	0.002265	0.000525	0.002461
35.7	100	61.0425	22170	43	0.001863	0.001022	0.004763
3.2	100	99.9667	35686	31	0.032505	0.001022	0.004837
22	100	66.4378	24274	38	0.003494	0.001576	0.008765
5.6	100	22.2287	6586	45	0.000525	0.001576	0.008635
3.7	100	54.9441	18141	23	0.025078	0.002257	0.0126
24.8	100	64.786	23586	29	0.044251	0.002315	0.01401
56.4	100	37.6224	13395	36	0.00525	0.002543	0.01635
89.1	100	42.9202	15261	35	0.014497	0.002719	0.01682
4.2	100	95.0026	35835	21	0.322829	0.002777	0.01787
5.2	100	66.363	24242	27	0.042112	0.00337	0.02356
10.9	100	50.8872	18466	24	0.188628	0.00382	0.02795
30.8	100	18.943	6201	28	0.011033	0	0.00076
1.8	100	101.1524	36154	34	0.016839	0	0.000576
64.2	100	15.8357	5008	29	0.03494	0	0.001459
147.9	100	13.9719	4049	72	8.34E-07	0	2.18E-07
4.2	97.901	134.9207	51654	72	2.68E-06	0	2.57E-07
8	100	67.7042	27361	43	0.002095	0	0.000833
159.6	100	18.5951	6423	30	0.006216	0	3.82E-05
23	100	60.2335	21860	90	9.46E-09	0	4.2E-10
0	100	101.6612	36367	24	0.150442	0.001308	0.00703
10	100	40.652	14261	18	0.076075	0.001498	0.007868
0.9	100	64.7324	23607	29	0.014145	0.001576	0.008123
12.7	100	73.2767	27420	32	0.009322	0.002315	0.01402
7.2	100	66.2485	26724	14	1.272187	0.00337	0.0232
52	100	73.4745	27502	36	0.003943	0.004124	0.02972
48.7	100	77.646	29245	25	0.032577	0.004344	0.03164
56.3	42.834	73.2184	27395	31	0.014131	0.005792	0.04182
2.1	100	59.7332	21677	17	0.181987	0.00861	0.06497
16.1	100	63.4516	21278	42	0.001971	0	0.000722
1.3	100	124.1181	47042	29	0.025922	0	0.000641

36.5	100	22.2584	7010	32	0.013288	0	6.41E-05
23.7	100	25.4092	8755	44	0.001115	0.000141	0.001581
47.6	100	55.1884	21999	35	0.015485	0.000141	0.001581
16	100	65.6531	22132	41	0.002478	0.000525	0.002229
8.1	100	96.7174	36221	36	0.009025	0.000758	0.003011
0	100	107.3369	40338	26	0.072353	0.001022	0.00503
52.9	100	62.186	20781	44	0.000579	0.001022	0.004627
22.5	100	69.4861	25893	34	0.015992	0.002257	0.01278
20.6	100	23.5119	8034	40	0.002873	0.003533	0.02454
51.3	100	53.2898	21200	38	0.006136	0	2.35E-05
50	100	34.1234	11664	48	0.000577	0.000951	0.003834
17.7	100	52.059	19449	51	0.000187	0.001022	0.004904
10.3	100	117.7234	43029	35	0.012496	0.001022	0.005767
2.3	100	130.2226	49657	17	0.470779	0.001022	0.005652
18	100	43.5686	14965	28	0.056349	0.001114	0.006148
81.1	100	57.0329	21380	47	0.000757	0.001114	0.006117
43.9	86.041	53.7291	19091	43	0.001751	0.001758	0.009292
0.4	100	99.3097	38260	28	0.038139	0.002079	0.01202
5.5	100	58.871	21567	25	0.11601	0.002315	0.01312
6.4	100	117.366	42868	21	0.335784	0.002376	0.01426
105.8	100	27.9591	10469	20	0.235883	0.004504	0.03182
140.2	100	34.289	11734	38	0.00624	0.004643	0.03321
17.8	100	48.3777	15607	53	0.000187	0	8.86E-05
17.8	100	48.3777	15607	53	0.000187	0	8.86E-05
34.1	82.327	48.8817	15816	64	1.33E-05	0	2.25E-05
34.1	82.327	48.8817	15816	64	1.33E-05	0	2.25E-05
104.7	100	48.7699	15772	60	3.62E-05	0	3.29E-05
104.7	100	48.7699	15772	60	3.62E-05	0	3.29E-05
17.9	100	56.6071	18792	43	0.001567	0	0.000563
7.2	100	46.8584	15023	44	0.001313	0	0.000412
60.6	100	71.5641	24838	37	0.007456	0	0.000116
16.2	100	72.4133	25191	34	0.015244	0	0.000972
4.7	100	77.8208	31679	57	4.73E-05	0	0.000248
18.2	100	73.9068	25819	35	0.012063	0	0.001266
18.7	100	60.5211	20344	39	0.002184	0	0.000588
35.2	100	59.4949	19923	34	0.008292	0	3.34E-05
32.5	100	60.0117	20127	54	6.91E-05	0	1.4E-08
76.3	44.062	60.0016	20123	33	0.009219	0	2.21E-05
33.7	100	69.5479	24005	58	5.31E-05	0	2.15E-07
20.5	5.393	70.0519	24213	88	5.66E-08	0	5.1E-08
21	87.595	70.0923	24228	93	1.94E-08	0	2.75E-08
17.8	100	68.7027	23656	39	0.00606	0	0.001484
16.9	100	71.06	24633	63	1.96E-05	0	2.3E-06
24.9	44.488	70.558	24424	88	5.65E-08	0	2.58E-09
5.5	25.385	66.2711	22667	99	5.22E-09	0	6.95E-08
0	45.237	66.2829	22672	105	1.41E-09	0	2.45E-07
14.3	100	66.7727	22880	99	5.2E-09	0	2.7E-08
0.6	100	66.7865	22886	61	3.36E-05	0	0.000421
11	100	65.7653	22458	99	5.39E-09	0	3.75E-08
1.4	100	65.7816	22465	73	1.97E-06	0	6.73E-05
5.9	100	67.7797	23288	74	1.64E-06	0	1.2E-05
10.6	100	67.2753	23089	48	0.000763	0	0.000234

2.4	100	90.7017	32036	35	0.012328	0	0.000169
3.1	100	89.5853	31592	65	1.31E-05	0	4.21E-08
7.8	100	89.1591	31418	68	6.6E-06	0	1.1E-09
7.5	100	89.0835	31387	68	6.02E-06	0	7.51E-10
9.5	100	82.7206	28819	65	1.34E-05	0	2.06E-08
4.2	100	111.7949	43437	42	0.001527	0	0.000533
6.9	100	113.4699	42404	27	0.081002	0	0.000742
10.1	100	112.2857	43660	62	1.57E-05	0	1.95E-05
27.2	100	112.3141	43672	57	4.7E-05	0	0.0001
1.9	100	103.5974	38339	40	0.003328	0	0.00068
63	13.125	140.676	54235	99	4.95E-09	0	4.89E-09
128.1	90.439	140.6324	54209	104	1.3E-09	0	3.51E-11
52.4	100	140.4736	54127	111	3.95E-10	0	2.71E-09
77.1	14.02	140.4658	54123	89	5.64E-08	0	2.6E-06
3.2	100	140.3737	54514	40	0.003577	0	0.000107
4.5	26.808	120.4865	45457	64	1.74E-05	0	4.57E-06
0	56.739	123.5398	46799	40	0.0033	0	0.000113
6.2	100	123.0879	46603	41	0.002782	0	0.000323
13.4	100	122.0253	46148	32	0.01878	0	0.000295
12.6	100	50.2718	19872	54	0.000143	0	0.00019
12.6	100	50.2718	19872	54	0.000143	0	0.00019
17.5	100	51.1178	20241	25	0.125965	0	0.001514
61.3	100	51.1703	20265	34	0.01768	0	1.59E-05
24.5	100	51.6726	20484	29	0.050383	0	0.000982
17.9	61.635	51.6248	20464	49	0.000575	0	2.59E-05
109.9	42.046	74.3348	30236	42	0.001555	0	0.000227
3	100	74.764	30413	49	0.000313	0	0.000905
1.7	100	103.0957	38130	43	0.001739	0.000273	0.00184
1.7	100	140.364	54510	28	0.049199	0.000273	0.001829
6.7	100	140.6887	54242	38	0.006262	0.000525	0.002186
19.4	100	56.1781	18619	34	0.011618	0.000758	0.003061
0.5	100	120.9907	45676	39	0.005808	0.000868	0.003293
3.5	100	120.6632	45535	33	0.022706	0.000868	0.00329
36.7	100	50.0983	16299	34	0.014019	0.000951	0.004344
67.8	100	60.511	20339	23	0.101813	0.000951	0.003982
12.6	100	90.0867	31793	13	1.968185	0.001677	0.008916
13.5	100	72.9775	25419	30	0.034377	0.001758	0.009675
21	100	74.603	26116	25	0.125862	0.002012	0.01064
23.3	100	137.8373	52907	16	0.863935	0.002079	0.01138
1.5	100	111.8159	43446	43	0.000734	0.002079	0.01211
3.5	100	69.7663	24093	26	0.088973	0.002376	0.01469
2.2	100	70.2757	24304	26	0.101276	0.00337	0.02273
3.9	100	80.3391	28615	24	0.146914	0.003736	0.02753
0.3	100	113.6514	42490	23	0.193481	0.004192	0.03062
0	100	122.1244	46192	24	0.112755	0.004504	0.03218
19.7	100	69.9167	24156	9	5.27867	0.004581	0.03259
15	100	90.0944	31796	4	16.09279	0.005792	0.04168
8	100	17.09	5056	46	0.00105	0	9.12E-05
4.5	43.402	55.0383	20101	73	1.5E-06	0	0.000119
3.7	100	54.5394	19895	56	8.72E-05	0	0.001537
11.5	100	17.1499	5362	42	0.002372	0	3.33E-05
0	100	54.1362	19240	54	0.000135	0	0.001476

21.3	20.015	61.1325	22046	55	0.000104	0	2.19E-05
40	5.017	61.1058	22034	58	5.48E-05	0	3.67E-05
6.8	100	60.6276	21832	42	0.001939	0	0.000227
6.8	100	61.7017	22269	51	0.000264	0	0.00016
18.2	100	130.1485	50730	85	1.47E-07	0	6.1E-10
26.6	23.466	140.27	55093	121	3.17E-11	0	8.29E-14
17.5	100	140.2842	55100	129	4.98E-12	0	2.64E-14
4.3	100	113.206	43565	47	0.000916	0	0.000364
1.1	100	113.711	43780	47	0.000848	0	0.000185
4.6	100	117.4538	45806	37	0.002366	0	0.001408
6	100	113.2467	44092	40	0.001102	0	0.000162
16.8	67.643	126.0063	49264	63	2.22E-05	0	2.92E-06
9.6	17.696	125.9978	49260	67	8.45E-06	0	1.25E-06
4.1	100	125.4944	49041	50	0.000414	0	7.69E-05
295.8	100	18.9687	5705	57	9.29E-05	0	6.94E-05
188.6	36.593	19.4684	5874	61	3.46E-05	0	4.4E-05
10.1	100	19.486	5880	63	2E-05	0	0.000569
130.6	100	19.9702	6046	51	0.000361	0	9.17E-05
12.5	100	115.8445	44213	45	0.000372	0	9.77E-05
48.9	17.908	114.8405	43841	82	7.46E-08	0	3.82E-07
22.4	26.101	114.8843	43856	59	1.53E-05	0	2.58E-05
13.6	100	115.3445	44025	63	5.8E-06	0	1.88E-06
10.9	100	115.3895	44042	55	4.48E-05	0	4.33E-06
11.4	63.744	134.2012	51346	72	3.03E-06	0	9.76E-07
12.6	100	116.3465	44407	59	1.55E-05	0	9.04E-06
44.6	39.729	118.0384	45057	85	3.93E-08	0	8.39E-08
24.9	91.165	118.3341	45170	59	1.39E-05	0	1.28E-05
14.4	100	118.5439	45246	73	5.97E-07	0	6.76E-07
10.4	100	116.3486	44408	53	5.85E-05	0	1.4E-05
3.3	100	44.3175	15277	47	0.000695	0	0.00133
53	100	44.7302	15442	41	0.003041	0	0.00041
20.2	100	44.823	15483	39	0.004438	0	0.000105
60.3	2.864	114.3392	43654	78	1.72E-07	0	2.49E-06
37.8	4.664	114.3798	43669	59	1.47E-05	0	1.13E-05
22.3	20.144	113.3982	43290	47	0.000114	0	0.001025
0	100	48.1647	18945	58	1.33E-05	0	2.25E-06
9.8	100	47.7128	18743	56	2.12E-05	0	1.48E-07
42.9	100	56.817	20566	83	7.38E-08	0	1.31E-09
320.7	10.097	140.7621	54283	70	3.69E-06	0	4.95E-06
10.4	66.382	140.775	54293	53	0.000175	0	3.34E-05
40.5	100	13.1616	3560	32	0.02673	0.000141	0.001772
7.3	100	53.5602	17598	56	8.5E-05	0.000141	0.001713
56.6	100	20.8674	6365	32	0.024801	0.000273	0.001943
2.6	100	113.8749	43466	38	0.002123	0.000273	0.001951
2.7	100	132.6326	48910	26	0.111578	0.000525	0.002344
3.2	100	133.6977	51120	20	0.518147	0.000525	0.002184
3.4	100	129.6434	50519	25	0.1517	0.000525	0.002589
29.7	100	140.6971	54246	32	0.026839	0.00065	0.002708
2.2	100	117.1712	44729	30	0.012687	0.000758	0.003095
67.6	79.659	56.4039	20412	22	0.106839	0.000868	0.003491
4.1	100	17.1304	4788	38	0.005889	0.000868	0.0035
3.3	100	54.6229	17916	43	0.001504	0.00121	0.0065

76.8	9.674	72.8541	29609	44	0.001779	0.001576	0.008552
0	100	47.647	18713	25	0.026818	0.002079	0.01222
2.9	100	113.6441	44270	28	0.016971	0.002257	0.01282
24.4	100	18.9851	5711	46	0.001112	0.002315	0.01306
6.8	100	60.6025	21822	22	0.222576	0.002376	0.01441
0	100	126.2979	49115	21	0.32582	0.002376	0.01513
0.5	100	125.3728	48994	29	0.036718	0.002777	0.01696
1.8	100	132.6065	50654	21	0.369574	0.002843	0.01908
0	100	126.5021	49461	30	0.049927	0.003533	0.02472
1.7	100	140.2929	55104	22	0.291775	0.004266	0.03082
10.3	100	61.8607	20655	21	0.246311	0.007785	0.05553
0.9	100	125.6671	49114	19	0.553809	0.008559	0.06464
0	100	125.7749	49162	26	0.089235	0.008962	0.06762
70.5	37.931	77.197	28622	56	0.000101	0	2.52E-05
18	100	25.9158	8489	40	0.003742	0	0.000369
97.5	51.508	43.9861	15766	47	0.000715	0	0.000644
25.4	100	57.8318	21190	41	0.003459	0	0.001193
17.3	100	26.877	9280	41	0.002879	0	0.000185
294.8	62.495	36.498	11324	42	0.001083	0	0.00055
0	100	102.4451	36691	56	1.21E-05	0	3.28E-06
13.3	100	138.1016	51161	96	1.28E-08	0	1.56E-11
10.7	100	138.6072	51389	89	5.77E-08	0	4.02E-11
7	100	96.6328	37098	35	0.008156	0	7.24E-05
11.9	100	97.1338	37309	64	9.91E-06	0	1.68E-07
14.2	100	43.5665	15442	42	0.000711	0	0.000407
4	100	98.0776	37567	62	1.54E-05	0	1.17E-05
36.4	100	69.2212	25429	53	0.000101	0	0.000623
9.5	100	43.2351	16833	58	5.59E-05	0	1.8E-06
171.5	94.734	23.4928	8603	34	0.013606	0	0.000803
46.3	100	18.9848	6610	51	0.000188	0	6.96E-06
1.8	100	102.1366	36572	15	0.157529	0.001022	0.005259
5.8	100	77.2081	28627	34	0.016518	0.001022	0.005775
12.6	100	44.0627	15796	34	0.015371	0.001758	0.009297
50.3	100	36.6534	11377	37	0.002889	0.002079	0.01229
119.4	100	16.6469	5120	44	0.001954	0.002376	0.01475
215.9	90.609	17.1428	5667	31	0.024021	0.002543	0.01567
1.3	100	99.7687	38471	17	0.519158	0.002777	0.01806
0.8	100	92.3573	35308	29	0.021127	0.002777	0.01724
19.2	100	42.8387	16665	10	3.562792	0.009514	0.07639
11.8	100	64.7204	23791	39	0.00585	0	0.000282
17.1	100	73.8914	25812	49	0.00047	0	0.000199
18.6	100	62.2528	20806	66	1.14E-05	0	3.51E-06
5.5	100	107.5719	38787	55	0.000149	0	0.000191
20.8	100	71.326	24332	43	0.002099	0	0.000277
45.5	84.63	140.3442	52190	77	6.02E-07	0	5.67E-12
16.2	100	73.6072	27291	67	7.07E-06	0	1.62E-05
27.9	100	65.1237	21928	34	0.017202	0.000951	0.004388
24	100	52.7101	20941	24	0.120503	0.000951	0.003829
33.3	100	64.9675	21869	32	0.027141	0.002079	0.01229
3.5	100	71.0337	24225	27	0.094618	0.002719	0.01667
2.2	100	90.5169	33812	23	0.184388	0.002777	0.01711
9.9	100	78.7111	32053	26	0.116812	0.003674	0.02549

3.7	100	92.6855	34616	25	0.152045	0.003674	0.02599
16.1	100	52.8526	21004	19	0.388571	0.004192	0.02992
3.1	100	90.7959	33916	18	0.590735	0.008034	0.05874
3.1	100	38.1622	11866	30	0.031275	0	0.000904
10.3	100	38.1017	11843	34	0.014682	0	1.02E-05
8.3	100	34.4533	10572	32	0.019475	0	0.000164
1.8	100	34.4558	10573	33	0.013117	0	0.000287
8.3	100	66.5933	22807	31	0.027435	0	0.000376
29.4	100	65.6344	22401	35	0.010165	0	0.000149
33.8	23.828	66.0887	22592	30	0.032251	0	1.87E-05
42	56.264	66.1399	22612	59	3.91E-05	0	2.58E-08
1.4	35.455	140.8576	55415	99	2.35E-09	0	2.99E-06
668.1	65.523	140.717	55337	73	1.35E-06	0	5.59E-08
12.4	100	90.9708	32140	65	9.69E-06	0	1.11E-05
7.3	100	88.5753	33214	56	7.72E-05	0	4.86E-05
21.6	76.028	86.5128	32773	74	1.34E-06	0	7.42E-07
57.3	34.813	86.1363	32602	46	0.000874	0	7.03E-06
15.9	100	86.6435	32826	51	0.000272	0	3.51E-06
13.5	100	62.4535	22994	58	5.08E-05	0	0.00074
137.3	71.817	140.3055	54044	40	0.003166	0	2.44E-08
36.3	100	140.3513	54067	43	0.001921	0	7.03E-06
8.3	39.351	141.4742	54717	102	2.14E-09	0	4.43E-11
81.9	100	141.4709	54715	111	2.68E-10	0	2.74E-12
16.2	20.171	141.4483	54701	114	1.3E-10	0	4.17E-15
16.6	54.809	67.6873	27353	100	1.24E-09	0	7.83E-15
2.8	62.571	67.7356	27376	60	1.2E-05	0	1.39E-07
0	100	64.7442	22025	48	0.000139	0.000273	0.001832
8.6	100	83.7029	31530	26	0.091854	0.000951	0.004206
3.2	100	88.2053	33051	31	0.024427	0.00121	0.006788
9.3	100	60.8673	20479	23	0.189684	0.002315	0.01394
57.7	18.064	75.2689	27728	38	0.004453	0.002315	0.01377
3.2	100	95.2725	36539	21	0.254071	0.002543	0.01614
0	100	67.6033	27315	29	0.010229	0.002843	0.01898
28.2	100	72.5133	26867	51	0.000234	0.003096	0.01971
0.9	100	95.0552	36446	22	0.208852	0.003254	0.02109
0.4	100	34.9655	10752	19	0.38494	0.00337	0.0234
2	100	90.7783	32064	22	0.212533	0.003674	0.02501
3	100	90.4674	31942	22	0.211205	0.003674	0.02649
0	100	95.8426	36653	21	0.277911	0.00599	0.04541
12.6	100	75.7701	27941	36	0.007855	0.006428	0.04736
6.2	100	60.9597	20515	24	0.169516	0.006872	0.04987
21	100	46.4506	14873	7	7.023205	0.009436	0.07121
10	100	100.8323	37902	50	0.000397	0	0.000481
40.4	13.154	97.9684	36728	51	0.000277	0	0.000948
57.5	100	57.787	19278	16	0.146013	0	0.000271
21.1	100	41.2569	12957	28	0.057777	0	0.000894
33.1	100	45.3379	15752	59	2.21E-05	0	8.18E-05
5	100	34.3871	11652	62	2.48E-05	0	0.000697
70.5	100	34.3698	11646	40	0.003526	0	0.000346
51.2	100	33.8646	11455	25	0.114445	0	0.001446
12.5	100	37.0366	11468	75	1.18E-06	0	0.00026
19.2	100	51.3162	16669	49	0.000494	0	0.001125

165.4	50.281	51.3138	16668	43	0.002059	0	0.000932
36.7	33.931	140.6833	55318	89	1.7E-08	0	1.78E-11
14.7	7.096	140.6815	55316	102	9.21E-10	0	1.04E-11
192.9	48.95	140.4338	55176	121	3.39E-11	0	1.02E-11
82.9	20.166	140.4303	55173	75	1.43E-06	0	4.74E-09
10.7	36.979	140.2748	52156	65	1.1E-05	0	5.26E-09
62.1	100	140.2771	52157	114	1.27E-10	0	6.87E-11
119.6	10.914	140.2722	52154	86	7.75E-08	0	1.29E-12
15.5	100	140.3943	52214	70	4.29E-06	0	2.2E-07
73.4	100	36.6388	12650	54	0.000171	0	0.000962
173.9	87.616	141.1619	54530	102	6.24E-10	0	5.51E-10
20.2	100	98.018	36746	39	0.004239	0.000273	0.002067
13.5	100	97.4644	36530	41	0.002284	0.000758	0.003167
18.6	100	34.4844	10583	30	0.041456	0.000951	0.004565
27.7	100	45.3162	15742	40	0.001807	0.000951	0.004311
3.9	100	98.4724	36916	34	0.013742	0.001114	0.006365
3.2	100	33.8867	11464	44	0.001555	0.00121	0.006763
30.9	100	65.7472	22450	20	0.230853	0.001758	0.009278
38	100	57.7929	19281	27	0.059273	0.002079	0.01214
0.4	100	28.1272	9770	41	0.003183	0.002719	0.01678
13.5	100	68.3792	23194	29	0.041316	0.003254	0.02093
30.6	100	68.5098	23244	22	0.182516	0.007636	0.05513
48.1	100	57.2915	19075	15	0.949419	0.009818	0.07951
31	100	43.9648	13942	27	0.079964	0	0.001234
82.1	14.206	44.469	14124	34	0.014557	0	0.000256
82.3	76.265	44.9737	14301	34	0.015014	0	0.00022
17.9	100	77.8183	31678	100	4.09E-09	0	5.79E-07
24.6	92.946	59.7403	21882	44	0.000838	0	4.14E-05
6.4	100	137.891	51060	34	0.017756	0	0.000491
65.5	26.482	77.4554	31527	80	4.14E-07	0	5E-07
29.9	66.913	57.4047	20527	48	0.000252	0	1E-07
27.8	93.584	57.3545	20504	50	0.000142	0	5.55E-08
24.3	100	57.2813	20473	53	8.44E-05	0	1.54E-06
1.6	68.614	136.2877	53316	57	8.35E-05	0	4.59E-05
32.7	8.165	136.2272	53292	84	1.74E-07	0	2.6E-07
2	100	95.1023	36374	49	0.00039	0	0.000312
24.7	100	95.5718	36551	61	2.9E-05	0	4.14E-06
5.6	100	95.6031	36563	57	7.28E-05	0	0.000215
28.2	57.731	70.9995	28804	67	9.14E-06	0	0.000866
5.3	100	69.9723	28348	67	8.87E-06	0	2.93E-08
98.3	26.999	66.5059	26836	54	0.000166	0	0.000853
43.7	12.885	77.3073	31465	74	1.69E-06	0	3.09E-06
16.7	100	45.9598	18016	57	7.26E-05	0.000141	0.001567
8.6	55.315	77.3762	31494	33	0.022506	0.000525	0.002358
6.9	100	89.232	33963	32	0.026506	0.000758	0.003186
10.1	100	44.0232	13964	34	0.01422	0.001022	0.004958
0.4	100	87.2985	33102	28	0.067739	0.001114	0.006077
31.5	100	43.4058	13725	18	0.819792	0.001945	0.009989
6.6	100	59.4596	21781	23	0.111136	0.001945	0.0102
0.3	100	130.3965	50834	40	0.003185	0.002079	0.01175
16.9	100	44.5234	14143	31	0.030938	0.002315	0.01393
3.2	100	78.0902	29438	20	0.393408	0.002376	0.01451

1.7	100	85.1535	32172	16	0.950259	0.003178	0.02069
4	100	76.962	28966	39	0.002388	0.003736	0.02679
84.9	100	24.3584	8980	28	0.026334	0.004192	0.02992
0	100	129.8921	50624	44	0.000995	0.004266	0.03088
4.5	100	96.3099	34260	19	0.53515	0.008962	0.06759
7.7	100	131.8604	51425	29	0.055141	0.008997	0.06916
4	100	69.6589	28213	17	0.9397	0.009514	0.07367
17.5	100	51.0964	16683	27	0.018566	0	0.001045
17.4	100	91.2488	34088	54	0.000137	0	1.24E-06
26.7	85.136	90.7473	33899	64	1.4E-05	0	5.08E-08
15	100	54.1475	17829	60	1.95E-05	0	0.000214
39.3	100	69.0282	23795	29	0.022393	0	0.001052
140.6	19.91	69.538	24001	46	0.000392	0	9.81E-06
0.8	100	104.1773	40345	71	6.24E-07	0	8.15E-08
27.7	100	139.696	53752	62	2.49E-05	0	2.75E-06
13.9	100	105.513	39114	36	0.009862	0	0.000864
39.7	100	137.4512	52729	21	0.317287	0	4.4E-05
24.5	51.66	141.4438	54697	75	2.12E-07	0	7.24E-13
43.9	87.952	58.8135	23535	86	8.36E-08	0	7.58E-09
76.7	100	93.0326	33834	55	4.7E-05	0	0.001222
18.2	100	37.6067	14482	49	0.00038	0	0.000544
34.3	100	100.1786	38637	48	0.000603	0.000868	0.003434
7.5	100	69.1442	23843	34	0.006172	0.00121	0.006464
5.8	100	58.8596	23555	33	0.014163	0.001498	0.007485
408.3	100	48.8316	17148	40	0.003199	0.002257	0.01258
7.1	100	102.4359	39251	41	0.002833	0.00382	0.02793
12.5	100	37.5909	14475	23	0.158899	0.008559	0.06449
9.6	100	69.6524	24047	23	0.089768	0.009514	0.0727
18.5	100	42.9023	14813	17	0.86333	0	0.00139
49.7	100	43.1511	14908	25	0.111888	0	9.3E-05
28.6	100	43.4067	15001	13	2.020192	0	0.001448
9.7	100	60.5745	20368	49	0.000529	0	2.4E-05
105.1	100	23.4092	7991	36	0.009036	0	0.000221
5.4	100	71.1126	24256	47	0.000839	0	2.82E-05
18.4	100	140.1763	52108	67	6.01E-06	0	4.12E-11
13.3	100	118.605	46285	48	0.000128	0	1.29E-05
11.4	100	118.5057	46241	37	0.001546	0	0.000291
55.6	100	90.5661	32838	59	4.96E-05	0	2.23E-05
37.9	100	66.3641	24431	49	0.000565	0.000525	0.002625
35.3	100	65.862	24235	48	0.00064	0.000758	0.003066
3.4	100	101.2367	37330	27	0.086585	0.001498	0.007682
3.2	100	140.4257	54538	17	0.711325	0.002376	0.01527
21.2	100	59.4842	21406	26	0.047045	0.002543	0.01608
19.8	100	102.7374	39738	7	2.803667	0.002843	0.01914
43.7	100	50.8048	18434	31	0.01627	0.003674	0.02575
9.8	100	83.4092	30995	40	0.003093	0	0.000368
28.9	41.609	93.7512	38418	77	6.37E-07	0	3.03E-06
9.9	33.644	140.5812	52318	69	5.27E-06	0	7.53E-05
109.3	3.133	140.4945	52266	80	3.62E-07	0	9.98E-07
153.3	12.909	140.4953	52267	75	1.31E-06	0	3.3E-07
35	100	89.5432	34099	47	0.000518	0	0.000123
29	38.508	97.6104	37526	69	3.2E-06	0	1.14E-05

31.4	9.737	97.5819	37513	65	7.15E-06	0	2.98E-06
118.4	100	140.6865	54241	97	8.85E-09	0	8.15E-12
33.5	100	107.2492	39830	70	3.58E-06	0	4.82E-05
16.2	18.393	66.0517	24116	61	3.51E-05	0	1.17E-05
24.3	18.921	66.0169	24100	64	1.87E-05	0	9.57E-08
27.8	100	68.784	27834	43	0.001986	0	8.18E-05
377.3	100	140.7167	54257	108	6.87E-10	0	1.77E-12
7.5	100	68.2802	27621	35	0.01306	0	0.000836
40.3	25.547	42.0747	16331	57	9.2E-05	0	4.51E-07
55	83.075	67.3361	24619	52	0.000181	0	0.000166
66.8	3.783	66.8327	24414	54	9.89E-05	0	0.000305
0	100	91.746	35027	55	7.06E-05	0.000141	0.001649
0	100	91.2124	34858	49	0.000189	0.000273	0.001834
0	100	25.5577	7673	52	0.000144	0.000758	0.003058
31.3	13.491	66.9461	24460	44	0.001021	0.000951	0.004466
79	94.106	93.9773	35979	44	0.001311	0.001022	0.005031
26.4	100	62.4375	25122	35	0.012149	0.001022	0.005778
23.1	100	89.5453	34100	33	0.01242	0.001114	0.006113
11.4	100	40.3959	12647	24	0.191487	0.001114	0.006221
17.9	100	42.1996	16383	30	0.040743	0.001308	0.007089
48.9	100	62.7811	22754	41	0.001543	0.001576	0.008871
1.8	100	128.2126	50116	49	0.000378	0.001576	0.00884
192	71.783	35.6744	12694	43	0.000927	0.001758	0.009158
10.6	100	66.4893	24668	38	0.004722	0.001758	0.009402
124.9	100	36.1772	12868	44	0.000863	0.001945	0.01018
7.5	100	82.9074	30807	17	0.623037	0.002079	0.01134
100	100	93.4751	35753	40	0.003417	0.002257	0.01291
9.2	100	60.032	21786	40	0.002034	0.002843	0.01859
22.7	100	74.3612	30248	30	0.036825	0.00337	0.02207
18.7	100	62.7592	22744	37	0.003563	0.003456	0.02436
10.2	100	41.9454	16271	25	0.143277	0.003674	0.02644
15.7	100	67.3412	27200	31	0.032899	0.003967	0.02903
292.3	58.639	35.9092	12773	38	0.002771	0.004344	0.03131
1.5	74.735	140.5058	52273	18	0.644016	0.005114	0.0354
4.8	100	129.7491	50680	27	0.063007	0.00573	0.04063
3.5	100	61.9898	24924	19	0.49454	0.006721	0.04906
6.7	100	90.0522	34326	17	0.491038	0.007423	0.05384
5.9	100	66.4428	24267	16	0.62254	0.007785	0.05549
74.9	15.918	85.4479	31781	71	3.11E-06	0	2.12E-05
84.8	24.485	85.4187	31770	55	0.000121	0	0.000767
4.7	100	104.942	39483	34	0.0084	0	0.000745
10	100	104.4398	39295	54	8.58E-05	0	4.4E-06
8.1	100	104.4204	39288	50	0.000222	0	0.000157
17.4	90.503	103.9357	39114	53	9.89E-05	0	1.09E-05
11.6	100	103.9166	39107	61	1.59E-05	0	1.05E-06
11.2	100	103.4325	38931	57	4.1E-05	0	7.31E-07
1.8	100	102.9285	38744	41	0.001521	0	0.000473
0.6	100	139.0527	51438	57	8.76E-05	0	8.4E-07
3.3	100	138.549	51269	62	2.6E-05	0	4.79E-07
87.7	48.204	73.9583	25841	60	2.1E-05	0	0.000317
27.3	100	42.1892	15079	22	0.269884	0	0.000806
24.7	100	44.9952	16168	35	0.008172	0	0.000782

119.6	46.973	54.2303	19782	43	0.001303	0	5.45E-07
59.9	100	53.7599	19593	25	0.082212	0	0.001559
89.4	17.35	53.7248	19577	36	0.006545	0	4.96E-06
37.1	100	53.2213	19370	30	0.028476	0	0.000531
37.8	100	17.5131	5542	44	0.001278	0	0.000058
23.8	100	68.3333	23174	48	0.000344	0	9.52E-05
1.8	100	85.8884	30050	59	3.26E-05	0	0.000759
40.6	59.62	67.535	24628	53	0.000112	0	0.000597
29.5	98.345	67.529	24625	54	0.000104	0	0.000377
5.2	100	67.0259	24435	41	0.001848	0	0.000905
14.9	83.794	135.935	53162	89	5.88E-08	0	1.77E-10
10.9	100	132.522	50618	37	0.009204	0	0.000363
1.5	100	132.973	50802	48	0.000735	0	0.000686
64.4	100	42.3006	14466	29	0.042433	0	0.000516
61.8	100	44.8042	15984	54	0.000157	0	7.04E-05
49.6	100	44.3017	15789	47	0.000694	0	1.42E-05
98.5	83.114	45.3203	16185	60	3.85E-05	0	9.24E-06
129.9	42.047	45.8236	16400	57	6.8E-05	0	1.7E-05
24.2	100	45.987	16461	64	1.55E-05	0	1.7E-06
167.1	86.011	141.1914	54550	75	5.46E-07	0	1.65E-09
35.3	100	43.6511	15545	25	0.102261	0	0.001418
85.5	21.457	46.3364	16595	63	1.66E-05	0	5.56E-06
150.1	100	141.2087	54563	97	3.97E-09	0	1.85E-09
65.5	8.496	40.4657	15655	52	0.000269	0	2.88E-06
17.2	100	40.97	15854	49	0.000436	0	2.52E-05
32.4	100	78.8559	29478	30	0.033377	0.000273	0.001975
4.9	100	103.4148	38923	40	0.002251	0.000273	0.001928
0.9	100	135.6465	50091	37	0.009299	0.000273	0.001952
6.4	100	133.0245	50824	38	0.007128	0.000525	0.002159
11.4	100	53.2529	19383	27	0.052316	0.000951	0.0038
98.4	100	90.1847	32673	32	0.016232	0.001022	0.005688
6.5	100	46.4925	16656	38	0.006388	0.001022	0.005226
6.3	100	40.4143	15633	34	0.016289	0.001498	0.007632
13.7	100	58.3899	19369	55	0.000109	0.001498	0.00768
11.2	100	35.0762	10792	48	0.000644	0.001498	0.00781
24.1	100	74.0082	25861	40	0.002281	0.001758	0.009012
12.6	100	53.752	19589	36	0.007	0.002012	0.01088
27.3	100	54.263	19795	25	0.088526	0.002012	0.01114
19.8	100	73.4546	25618	40	0.002578	0.002012	0.01083
6.9	100	45.4854	16255	38	0.005349	0.002012	0.0104
27.5	100	73.5072	25640	36	0.006197	0.002079	0.01135
15.7	100	17.4704	4912	20	0.313475	0.002257	0.01288
18.2	100	60.2675	20241	16	0.781167	0.002315	0.01353
0	100	132.3902	48812	26	0.107002	0.002376	0.01481
27.4	100	89.6756	32447	33	0.012847	0.00337	0.02355
12.4	100	39.8239	14041	31	0.018064	0.00337	0.02232
6.5	100	68.2284	23132	32	0.014758	0.003736	0.02674
14.8	100	62.2774	22502	42	0.001299	0.004124	0.02988
10.4	100	36.6655	14089	21	0.312134	0.004192	0.03052
23.5	34.222	88.5065	33639	51	0.000304	0.004643	0.03352
0.9	100	135.6732	53057	10	3.832161	0.005687	0.03934
1	100	132.4699	50596	21	0.338054	0.009514	0.07475

25.8	100	71.7732	26590	37	0.007512	0	0.001256
1.7	100	41.1617	12924	58	6.53E-05	0	0.001093
18.8	100	69.453	23964	38	0.001885	0	0.00038
30.3	100	68.8945	23740	26	0.027292	0	0.000813
11	100	75.91	26159	50	0.000331	0	0.00066
59.7	24.029	82.5673	28752	66	5.72E-06	0	6.42E-05
7.7	100	101.3962	38621	48	0.000567	0	0.000866
4.1	100	140.7393	52647	99	4.58E-09	0	1.31E-11
6.7	100	140.7415	52648	72	2.34E-06	0	2.04E-07
12.8	100	142.6867	49649	88	6.12E-08	0	2.46E-10
31	74.751	82.7526	29614	45	0.000303	0	3.7E-05
13.6	44.195	142.6845	49648	101	3.3E-09	0	1.44E-10
31.7	72.683	82.7185	29599	29	0.010851	0	0.000388
76.1	100	53.6865	21359	49	0.00051	0	0.001356
46	100	141.2897	54612	55	0.000144	0	2.45E-07
22.2	100	141.2919	54613	21	0.378657	0	6.34E-05
5.6	100	140.7002	50323	60	4.06E-05	0	2.24E-06
2.5	100	133.4412	51005	33	0.021403	0.000141	0.001689
40.5	100	45.0903	16203	29	0.048543	0.000273	0.002043
34.8	100	42.9885	15213	27	0.015208	0.000525	0.002515
103.8	27.461	82.5734	28755	38	0.003734	0.000868	0.003534
107.8	3.22	44.4921	15966	29	0.051258	0.001022	0.004747
14.4	100	80.1689	27814	48	0.000473	0.001022	0.005595
25.3	100	82.2423	29402	22	0.050176	0.001022	0.005519
95.6	100	41.5383	14346	19	0.560735	0.001022	0.004838
28.9	100	43.5981	15074	30	0.043765	0.001114	0.006038
33.1	100	50.0151	19759	45	0.001352	0.001308	0.007129
47.3	100	18.0397	5426	17	0.640395	0.001677	0.008913
26.1	100	43.9908	15768	28	0.05474	0.001758	0.009068
18.1	100	82.0668	28552	40	0.00273	0.002012	0.01066
3.6	100	99.6521	35562	30	0.038748	0.002079	0.01198
10.6	100	39.4447	13374	24	0.1388	0.002543	0.01599
24.4	100	40.0085	14260	33	0.025014	0.002777	0.01691
23.3	100	82.0647	28551	26	0.059215	0.003254	0.02131
29.8	100	61.7047	20596	26	0.090065	0.004344	0.03127
6.5	100	78.3965	29071	35	0.011893	0	0.001276
14.5	100	78.2752	29021	46	0.001033	0	0.000712
63.4	100	77.8901	28861	38	0.005456	0	0.000353
0.9	100	140.1032	55013	74	1.68E-06	0	1.03E-07
10.1	100	98.1903	37791	72	1.56E-06	0	5.89E-06
59.5	66.345	99.7408	38459	84	1.48E-07	0	1.31E-08
8.1	100	99.7745	38474	46	0.000837	0	0.001192
16.9	81.538	100.6117	38822	69	2.77E-06	0	4.61E-07
4.5	100	140.5286	54154	63	1.88E-05	0	1.45E-05
244.5	68.6	140.5019	54142	100	3.49E-09	0	1.58E-10
15.2	100	120.8313	45607	48	0.000578	0	0.000854
10.9	100	122.8992	46523	51	0.00011	0	0.000117
42.6	65.034	140.9276	54389	56	8.19E-06	0	4.76E-11
16.2	100	121.3309	45831	48	0.000631	0.000141	0.001584
129.4	82.855	23.5461	8625	22	0.263063	0.001022	0.005118
120.1	14.223	23.6849	8688	37	0.002524	0.00121	0.006835
68.6	100	82.1362	29361	50	4.43E-05	0.001498	0.007855

5.2	100	33.4981	10246	50	0.000246	0.002315	0.01381
101.4	100	15.5312	4411	20	0.021341	0.002315	0.01342
139.6	100	81.6367	29153	42	0.000279	0.002376	0.01436
190.8	100	24.8339	8542	31	0.008466	0.002543	0.01622
2	100	107.4725	39920	15	0.226622	0.004124	0.0294
9.3	100	24.8619	8553	32	0.008	0.004803	0.03412
1	100	117.9073	48417	42	0.001302	0	0.00026
148.6	34.134	16.4499	5024	31	0.026203	0	0.001054
26.5	100	56.997	18831	41	0.001574	0	0.000789
0.8	100	130.7131	48200	50	0.000435	0	0.000154
8.8	100	130.2357	48008	59	5.73E-05	0	2.29E-05
1.4	100	119.6006	46273	44	0.000952	0	0.000455
9.9	100	140.7117	52392	80	1.51E-07	0	2.98E-08
17.4	15.885	140.6966	52382	101	1.18E-09	0	1.6E-10
60.9	100	31.6285	10686	48	0.000408	0	0.001329
39.2	100	137.3711	52691	48	0.000422	0	5.19E-06
49.1	73.31	67.5843	24748	62	2.75E-05	0	3.98E-07
66.1	100	16.4605	5029	39	0.003937	0.000273	0.002079
10.6	100	65.4267	24231	35	0.009868	0.000525	0.002354
131.5	18.969	20.5225	7321	45	0.00054	0.001022	0.005226
35.1	100	56.4959	18637	28	0.02703	0.001022	0.004774
9.3	100	81.0363	28168	38	0.003167	0.001022	0.0053
15	100	73.1667	27111	33	0.009652	0.00121	0.00676
0	100	117.9462	48433	28	0.029762	0.00141	0.007228
17.1	100	81.0003	28155	34	0.007648	0.002079	0.01146
53	100	39.0321	13205	14	1.412267	0.002376	0.01512
0.5	100	119.0979	46055	25	0.074204	0.002843	0.01848
19.3	100	19.6589	6921	32	0.025356	0.00362	0.02492
11.5	100	74.6597	27678	59	2.24E-05	0	8.9E-08
11.4	100	46.5541	14902	43	0.001714	0	0.000383
5.9	100	86.5774	35359	27	0.052574	0	0.000162
28.3	100	140.4135	52224	88	1.38E-08	0	2.65E-10
97.6	100	13.5307	3837	19	0.176493	0	0.000661
63.8	58.936	100.551	37050	62	2.58E-05	0	6.27E-06
44.8	100	42.0486	14818	29	0.039333	0	0.000921
48	100	41.5457	14618	30	0.027641	0	0.000668
64.8	100	70.5136	25973	58	7.69E-05	0	1.21E-05
32.5	94.935	64.7717	26102	61	2.4E-05	0	4.16E-05
29.5	100	89.5122	34084	50	0.000408	0.000525	0.002616
1.8	100	75.1611	27868	26	0.039835	0.000525	0.002183
4.1	100	87.0847	35579	29	0.031897	0.000951	0.003693
7.5	100	130.1238	49608	32	0.011054	0.001022	0.004684
15	100	12.4938	3374	21	0.075303	0.002012	0.01027
13.8	100	82.8176	30797	33	0.01464	0.002079	0.0122
8.7	100	12.2447	3350	25	0.025989	0.002315	0.01353
13.2	100	97.0228	36347	33	0.020733	0.002376	0.01507
26.5	100	64.8302	26126	18	0.405656	0.005792	0.04247
7.8	100	100.6257	37081	19	0.467761	0.009514	0.07282
9.3	100	49.8933	16124	13	1.697678	0.009858	0.08125
9.1	100	72.5447	26879	45	0.001064	0	0.001327
8.7	100	99.792	40995	44	0.000763	0	2.69E-05
9.6	100	68.4082	23531	39	0.004736	0	0.000296

18.9	58.173	140.473	52255	58	3.14E-05	0	5.31E-05
132.4	79.427	140.4693	52253	93	9.6E-09	0	4.95E-10
4	100	106.6469	41349	38	0.001634	0	0.000784
58.2	100	17.733	5985	57	6.34E-05	0	0.000184
33	42.157	42.1173	14849	62	1.75E-05	0	5.04E-05
6	100	68.9325	27899	40	0.00092	0	0.000117
89.8	100	49.3419	19469	28	0.029228	0	0.000124
69.8	100	25.83	9621	47	0.000576	0	0.000612
46	100	28.0806	9753	36	0.008018	0.000141	0.001565
21.3	100	17.7778	5369	42	0.001643	0.000141	0.001762
35.6	100	17.8308	5696	42	0.001903	0.000273	0.001923
95.3	100	48.2512	17330	26	0.024537	0.000273	0.002032
1.1	100	106.5853	40826	28	0.030009	0.000273	0.002028
0.5	100	113.2772	46515	25	0.042308	0.000758	0.003042
27	100	36.0843	12602	41	0.001142	0.00121	0.0066
4	100	84.4661	31463	18	0.588449	0.002543	0.01615
21.9	100	47.3507	16505	34	0.015111	0.003254	0.02113
35	100	41.7372	14700	26	0.070529	0.004266	0.03101
23.8	100	56.5201	18757	48	0.000703	0	0.000174
84.2	32.787	57.6069	19205	26	0.091457	0	7.45E-05
17.5	100	57.6911	19241	35	0.009917	0	2E-06
44.5	100	57.0555	18984	18	0.489585	0	0.000741
37.9	100	31.5318	10591	44	0.00162	0	2.39E-05
60	22.68	31.0301	10407	59	5.03E-05	0	9.06E-06
50.4	95.093	31.0202	10403	58	5.89E-05	0	4.04E-05
101.1	9.017	30.5832	10242	55	0.00011	0	1.44E-05
0	100	30.2448	10111	62	1.79E-05	0	0.000772
14.1	100	32.0397	10780	53	0.000201	0	1.61E-05
15.9	100	31.1378	9464	53	0.000178	0	0.000251
45.5	68.953	31.2536	9499	59	4.7E-05	0	4.46E-06
29.1	100	31.1204	9458	39	0.004641	0	5.98E-05
0	100	30.7376	9334	53	0.000125	0	0.000542
30.9	100	34.6165	11738	35	0.012884	0	0.000102
19	100	34.0651	11532	48	0.000636	0	1.39E-05
15.1	100	33.0506	11158	43	0.001799	0	5.13E-05
26.6	100	32.5473	10975	38	0.006688	0	0.000161
97.3	56.1	58.1103	19410	25	0.108197	0	0.000116
14.3	100	58.1928	19444	35	0.010962	0	2.08E-05
61.9	100	58.6139	19599	28	0.050029	0	5.52E-05
29.3	100	30.6397	10769	59	3.5E-05	0	0.00034
5.2	100	30.6303	10765	46	0.000582	0	0.000959
14.5	100	60.479	20129	18	0.489493	0	0.000758
61.6	95.323	58.9319	19569	69	6.32E-06	0	1.04E-06
100.5	1.487	58.4219	19381	69	5.84E-06	0	3.55E-05
13.8	100	57.9929	19221	55	0.000148	0	0.000012
56.8	38.172	58.4942	19411	58	6.91E-05	0	1.09E-05
39.2	100	65.9386	22238	40	0.004275	0	0.001051
16.6	100	65.7021	22151	35	0.009732	0	1.67E-05
66.7	4.417	62.6785	20971	28	0.056461	0	0.000111
33.2	21.698	62.6348	20952	37	0.006469	0	3.46E-06
60.4	7.528	62.1742	20776	26	0.076117	0	4.17E-05
20.6	100	61.6739	20585	23	0.147645	0	0.000233

17.8	100	61.168	20393	22	0.215716	0	0.000163
49.2	46.765	62.1318	20759	32	0.022293	0	2.91E-06
33.9	36.084	63.1358	21150	30	0.03151	0	5.09E-06
38.4	100	64.695	21770	25	0.114873	0	4.29E-05
41	35.671	64.1922	21569	27	0.069482	0	6.81E-05
47.8	100	64.1433	21548	32	0.019693	0	2.03E-06
64.6	14.388	63.6869	21370	25	0.109359	0	0.00012
35.9	68.153	63.6382	21351	33	0.018512	0	3.66E-06
73.6	8.399	63.1814	21170	28	0.048734	0	6.9E-05
20.9	100	65.201	21956	21	0.258951	0	0.000153
24	54.378	141.1302	55562	61	2.65E-05	0	6.87E-06
14	7.024	141.1241	55556	56	9.65E-05	0	8.38E-09
11.7	100	31.7849	9731	48	0.0006	0	6.58E-05
13.6	100	50.6277	17853	76	6.88E-07	0	0.00123
28.1	11.854	65.1755	23683	97	8.25E-09	0	2.35E-09
58.1	3.863	65.1658	23679	48	0.000635	0	0.000312
26.6	100	64.6619	23471	35	0.012387	0	0.001194
25.6	100	66.4289	24185	54	6.95E-05	0	0.001058
12.6	100	67.9385	24788	56	4.33E-05	0	0.000735
28.8	100	67.4353	24593	56	3.99E-05	0	0.000366
129.5	2.915	66.9314	24401	62	1.31E-05	0	0.001533
33.4	22.8	61.0868	22026	32	0.018759	0	3.73E-05
28.9	100	61.044	22006	35	0.009939	0	6.33E-06
33.8	15.988	60.574	21810	28	0.055446	0	5.49E-05
38.4	17.449	60.0715	21590	35	0.011478	0	1.82E-05
41.9	80.733	60.0269	21572	30	0.034106	0	3.29E-06
35	100	59.5666	21385	25	0.111899	0	0.000329
30.6	84.8	60.5299	21790	37	0.006112	0	1.52E-06
26.6	75.246	61.5904	22224	29	0.045243	0	6.08E-05
8	100	62.5987	22643	21	0.236523	0	0.001523
22.2	100	62.0957	22427	25	0.115403	0	0.00023
136.1	4.393	140.4294	55172	49	0.000458	0	3.97E-05
2	52.383	140.3752	55144	49	0.000473	0	0.000322
18.2	100	139.2361	53539	52	0.000257	0	3.25E-06
0	100	101.9142	37627	63	2.25E-05	0	1.48E-05
11.1	6.633	105.127	38953	83	2.09E-07	0	2.25E-11
16.2	24.565	105.185	38978	96	9.74E-09	0	4.15E-12
2.7	100	105.6283	39166	61	3.14E-05	0	9.93E-08
2.4	100	105.6865	39189	53	0.0002	0	5.34E-06
109.5	71.178	138.224	53083	86	1.02E-07	0	1.11E-08
43.4	100	138.3708	53148	48	0.000596	0	9.87E-07
25.5	8.359	137.8665	52921	63	1.94E-05	0	1.26E-07
205.1	54.98	141.2557	54593	76	8.3E-07	0	2.12E-09
31.1	100	51.3492	20347	17	0.819106	0	0.000429
261.8	22.895	141.2732	54603	88	3.88E-08	0	1.71E-10
11.3	100	141.2792	54607	75	8.25E-07	0	1.93E-07
12.9	100	92.8452	33752	73	1.89E-06	0	4.04E-11
31.7	39.448	92.8064	33733	55	0.000112	0	7.22E-08
18.1	100	96.2359	35180	52	0.000254	0	4.05E-07
40.5	71.723	140.9591	54407	62	1.71E-05	0	2.06E-06
1.8	100	92.3347	33539	54	0.000165	0	8.2E-06
23	45.617	92.2968	33524	59	4.82E-05	0	2.69E-08

7.4	100	106.1982	39397	24	0.161283	0.000141	0.001723
114.3	58.943	30.5266	10218	28	0.063583	0.000141	0.001675
12.4	100	33.559	11344	32	0.023903	0.000141	0.001755
3.7	100	30.7834	9349	43	0.001269	0.000407	0.002135
0.5	100	107.6559	39992	28	0.071576	0.000525	0.002412
9.8	100	61.5461	22207	28	0.047448	0.00065	0.002658
38.8	100	59.1216	19787	16	0.818245	0.00065	0.002717
11.7	100	64.645	21751	26	0.086628	0.00065	0.002678
15.6	100	66.3636	22403	12	1.841695	0.000868	0.003446
30.8	100	64.7581	21791	45	0.000508	0.000868	0.003308
10.6	100	137.3616	52687	32	0.028366	0.000868	0.003488
31.8	23.717	66.981	24420	49	0.000213	0.000868	0.003324
21.6	100	59.4358	19751	43	0.002544	0.000951	0.003736
57.7	100	51.5048	18706	49	0.000401	0.001022	0.005553
28.3	100	59.9383	19927	38	0.008017	0.001022	0.004804
9.8	100	66.4785	24206	36	0.004492	0.001022	0.00527
4.4	100	53.8444	19125	31	0.034737	0.001022	0.005325
24.8	100	73.8759	25805	26	0.091846	0.001114	0.006317
24.9	100	34.9731	10755	20	0.378785	0.001114	0.006022
172.6	3.552	66.6678	24287	51	0.000151	0.001114	0.006055
0	100	106.1367	39369	24	0.156109	0.001308	0.007163
13.2	100	60.9431	20311	36	0.010389	0.001576	0.008629
15	100	50.6236	18364	49	0.000197	0.001758	0.009441
12.8	100	56.8529	18896	34	0.015863	0.002012	0.01125
7.9	100	66.3895	24254	45	0.00042	0.002012	0.01075
15.4	100	59.06	21186	13	1.347838	0.002079	0.01219
51.2	67.321	49.6608	17470	56	4.7E-05	0.002376	0.01441
9.8	100	63.5991	23046	24	0.178641	0.002376	0.01518
24.3	100	38.054	13053	22	0.261252	0.002376	0.01489
12.7	100	54.3557	19330	27	0.090348	0.002543	0.01565
24.2	100	84.9438	30495	14	1.090532	0.002777	0.01746
22.9	100	50.1651	17668	49	0.000227	0.002777	0.01759
1.7	100	53.3399	18919	23	0.206463	0.002777	0.01691
18.8	100	68.6194	25061	37	0.003531	0.00337	0.02402
12	100	62.8139	22731	27	0.087089	0.00337	0.02243
179.2	5.758	66.9111	24392	39	0.002142	0.004049	0.02921
14.3	100	27.2984	8230	36	0.005339	0.004643	0.03305
10.1	100	31.8727	9760	29	0.04357	0.005404	0.03765
7	100	57.1796	20434	32	0.029688	0.006299	0.04623
8.8	100	53.5611	19012	16	1.141499	0.007836	0.05692
9.5	100	53.0562	18809	14	1.511734	0.007836	0.05601
13.9	100	60.4395	20114	23	0.223654	0.008683	0.06604
9.7	100	63.7049	21376	26	0.113005	0.008746	0.06621
8.7	100	52.8347	18722	19	0.550004	0.008997	0.06791
8.3	100	31.522	10587	28	0.064041	0.008997	0.06796
70.1	100	49.7447	16164	42	0.000963	0.009764	0.07818
16.3	100	80.1851	29756	34	0.010986	0	2.39E-05
10.3	100	80.2304	29776	37	0.0066	0	0.000276
14.2	100	78.3852	31924	28	0.044284	0	0.000136
38.1	42.479	77.8811	31706	58	4.45E-05	0	3.11E-10
23.2	98.941	77.7485	31650	55	8.91E-05	0	5E-08
63.9	100	16.5058	5051	43	0.001956	0	2.07E-05

35.7	100	53.4067	17463	26	0.107956	0	0.00152
8.1	100	102.0551	36536	21	0.260472	0	0.000251
9.5	100	102.0407	36530	32	0.020614	0	9.93E-05
7.2	100	101.5494	36317	29	0.042223	0	0.000249
7.9	100	20.0112	5886	43	0.0018	0	0.001148
50.4	100	51.9766	18387	32	0.029544	0	0.000543
103.1	28.307	51.4662	18181	32	0.028704	0	0.000387
37.9	84.774	51.2191	18077	63	2.33E-05	0	5.66E-06
113.6	3.999	50.9634	17985	38	0.007095	0	0.000229
58.3	100	50.4579	17782	21	0.342353	0	0.000886
38.4	5.205	100.1783	38086	31	0.022419	0	2.72E-05
53.2	12.005	100.1578	38076	47	0.00061	0	2.6E-07
47.8	69.187	100.1191	38060	19	0.486397	0	0.000393
23	41.934	100.0616	38035	37	0.008843	0	2.75E-05
3.1	100	100.5675	38268	41	0.003477	0	0.000775
3.1	100	100.5675	38268	41	0.003477	0	0.000483
18	100	101.171	38525	44	0.00139	0	1.21E-06
33.2	18.953	100.6813	38320	29	0.043807	0	1.56E-05
49	25.89	100.6614	38311	43	0.001683	0	1.09E-06
11.9	100	109.7545	42608	36	0.010714	0	7.93E-06
15.5	100	109.5407	42524	49	0.000515	0	4.35E-07
10.7	100	110.0472	42727	28	0.056589	0	5.74E-05
13.3	100	110.3982	42867	75	1.33E-07	0	2.5E-08
3	100	110.2577	42810	34	0.015482	0	0.001361
2.7	100	112.1842	43616	41	0.000583	0	2.59E-05
32.2	36.072	112.3882	43706	59	1.1E-05	0	3.38E-09
59.1	22.764	16.5762	5369	57	7.26E-05	0	5.29E-06
7	100	116.7573	45526	46	0.000195	0	1.43E-05
7.5	100	117.2639	45727	53	4.18E-05	0	3.64E-08
25	22.71	113.1891	44064	82	5.59E-08	0	2.8E-11
42.5	27.23	112.8938	43936	61	7.6E-06	0	1.95E-09
41	14.214	112.6868	43844	88	1.42E-08	0	3.31E-12
2.9	100	114.7052	44684	50	8.23E-05	0	6.46E-06
2.7	100	114.4128	44560	33	0.00399	0	0.000172
10.4	100	113.9086	44373	43	0.000407	0	1.04E-05
25.7	80.342	113.695	44291	87	1.89E-08	0	6.48E-13
46.5	63.544	113.4039	44166	61	6.32E-06	0	2.53E-10
10.1	100	114.1971	44481	82	5.68E-08	0	1.69E-09
10.1	100	121.2195	47347	51	0.000132	0	9.44E-08
23	59.512	120.7157	47148	77	3.09E-07	0	3.13E-09
6.5	100	134.3365	51406	65	4.96E-06	0	5.5E-05
25.7	100	77.3784	31495	46	0.000632	0	9.57E-08
9	100	136.0603	52136	42	0.000698	0	2E-05
16.7	100	136.083	52146	38	0.00168	0	6.32E-07
6.5	100	134.3365	51406	65	4.96E-06	0	5.5E-05
48.1	56.593	135.5503	51920	34	0.004401	0	5.32E-06
25.7	68.496	135.5748	51932	42	0.000646	0	4.03E-07
88.4	34.362	43.3242	15420	38	0.004324	0	0.001069
28.3	25.792	141.0515	54464	69	5.37E-06	0	2.51E-08
107	14.455	141.0498	54462	63	2.01E-05	0	7.61E-09
39.3	72.473	141.0784	54479	45	0.001427	0	1.08E-05
1513	100	141.0731	54475	71	3.45E-06	0	1.29E-08

4.1	38.968	37.6111	13187	72	1.49E-06	0	0.00057
8.2	100	67.4478	27245	19	0.455388	0	0.001314
4.9	100	73.5867	29926	41	0.001605	0	4.46E-06
70.5	100	140.9493	54402	96	8.71E-09	0	1.55E-06
11.8	100	76.8709	31285	28	0.0493	0	0.000112
7	100	77.2375	31438	45	0.000924	0	6.86E-05
3.6	100	72.8213	29594	52	5.09E-05	0	7.07E-06
8.9	100	72.8321	29599	71	7.08E-07	0	4.38E-08
56.1	100	16.4851	5325	37	0.007276	0.000141	0.001706
9.8	100	101.5842	36333	23	0.205871	0.000273	0.001875
5.6	100	117.036	45634	22	0.051463	0.000525	0.002372
5.9	100	101.5316	36310	21	0.26565	0.000758	0.002941
2.2	100	115.2202	44892	23	0.04853	0.001022	0.004627
111.6	100	13.5753	3804	37	0.005878	0.001022	0.004648
13.1	100	19.9324	6239	35	0.010573	0.001114	0.00612
40.4	100	19.9824	7078	37	0.007912	0.001114	0.005954
46.4	57.436	40.7013	14385	45	0.000428	0.00121	0.006482
34.4	19.345	40.9554	14491	51	0.00011	0.00121	0.00657
13.6	100	67.3707	22797	19	0.529084	0.001576	0.008239
0.9	100	100.3093	38145	19	0.431891	0.002079	0.01204
1.1	100	99.79	35617	23	0.193016	0.002079	0.01229
3.4	100	99.6541	37852	19	0.399418	0.002177	0.01238
41.2	100	43.2584	15395	22	0.157389	0.002315	0.0141
69.5	2.949	50.1119	18059	36	0.009088	0.002315	0.01361
285.2	44.916	44.057	15656	23	0.134444	0.002376	0.01462
59.7	22.558	48.8991	17115	38	0.005952	0.002376	0.01485
1.3	100	101.5273	36308	19	0.522273	0.002376	0.0146
1.3	100	101.5273	36308	19	0.522273	0.002376	0.0146
65.2	100	66.4597	22443	22	0.21184	0.002376	0.01433
72	100	44.5711	15859	21	0.217483	0.002777	0.01816
2.2	100	100.7069	38867	16	0.194753	0.002843	0.0183
112.5	100	25.7277	8123	15	0.889239	0.003254	0.02128
220.8	31.591	44.0662	15661	22	0.154211	0.003254	0.0211
33.7	100	44.9831	16034	27	0.054322	0.00337	0.02315
18.4	100	48.9706	17146	35	0.011784	0.00337	0.02192
32.7	100	39.6427	13975	19	0.303599	0.003736	0.0269
0.9	100	99.5565	37810	14	1.494066	0.003736	0.02679
0.9	100	99.5565	37810	14	1.494066	0.003736	0.02679
36.4	100	48.2614	16913	13	2.390134	0.004424	0.03169
18.3	100	38.2979	13319	41	0.00163	0.004643	0.03288
4.3	100	133.8334	51182	30	0.015744	0.00573	0.04052
4.3	100	133.8334	51182	30	0.015744	0.00573	0.04052
4.2	100	17.4343	5026	41	0.001913	0.005792	0.04176
1.5	100	96.9077	35463	15	1.281328	0.006428	0.04732
1.5	100	96.9077	35463	15	1.281328	0.006428	0.04732
2.3	100	94.195	35491	18	0.567271	0.007836	0.05598
40	100	49.9414	17991	20	0.301895	0.007969	0.05841
0.9	100	101.0711	38480	17	0.843828	0.008034	0.05869
0.9	100	101.0711	38480	17	0.843828	0.008034	0.05869
0	100	72.3192	29375	9	1.229486	0.008559	0.06417
0.4	100	101.187	38532	12	1.853354	0.009498	0.07178
10.8	100	68.2577	25168	22	0.156714	0	0.000456

6.6	100	70.2701	25981	49	0.000289	0	2.2E-05
18.9	83.072	69.7683	25786	62	1.58E-05	0	3.43E-08
8.7	100	69.3993	25640	37	0.00421	0	0.000609
21.3	5.436	69.2644	25589	64	1E-05	0	8.17E-08
16.9	100	68.8984	25444	81	1.97E-07	0	5.95E-07
20.6	9.104	68.7614	25386	70	2.26E-06	0	7.06E-09
12.6	100	69.2743	25594	39	0.00314	0	8.48E-05
30.3	100	56.7098	18833	32	0.024672	0	0.000739
2.2	100	78.029	28923	34	0.009843	0	0.000431
93.6	100	15.8974	4549	19	0.370731	0	0.001391
19.6	100	86.6661	35398	36	0.00542	0	0.000372
14.9	100	87.1729	35618	39	0.002582	0	0.000464
21.1	42.365	43.7517	15676	49	0.000212	0	1.51E-06
7.2	100	62.3097	21049	41	0.001838	0	1.35E-05
282.6	67.707	15.9436	4788	18	0.489157	0	0.001273
24.9	100	68.2775	23481	19	0.374937	0	0.001288
0	100	140.9008	55436	85	9.3E-08	0	2.4E-06
20.4	100	124.6214	48696	51	0.00017	0	4.07E-05
9.3	100	123.1848	48129	59	2.55E-05	0	9.03E-06
44.2	31.068	123.114	48103	58	3.41E-05	0	3.31E-05
27	8.789	122.6803	47929	70	2.21E-06	0	2.1E-05
72	2.713	122.6078	47896	58	3.23E-05	0	1.06E-05
3.5	100	122.1704	47718	40	0.00186	0	0.000205
6.8	59.435	119.8214	45168	62	2.44E-05	0	2.85E-05
0.3	93.757	115.7038	44157	89	1.28E-08	0	6.57E-06
0.4	100	116.2076	44354	73	4.35E-07	0	2.57E-05
38.6	94.607	65.5262	23889	66	4.3E-06	0	3.54E-06
42.2	55.104	81.6529	30914	38	0.002067	0	0.000959
16.7	100	68.1108	24962	30	0.036873	0	0.000941
12.8	15.377	67.606	24758	38	0.006233	0	0.000871
47.2	100	66.029	24105	56	4.17E-05	0	4.51E-05
8.2	14.177	66.5876	24340	34	0.015407	0	0.000655
9.5	3.413	67.0944	24556	38	0.005779	0	0.000162
84.9	100	18.5234	6386	34	0.0199	0	3.64E-05
36.8	100	141.0958	54487	40	0.003365	0	7.32E-05
0.9	54.154	70.0464	28380	74	4.98E-07	0	1.92E-06
45.1	57.136	141.317	54628	65	9.5E-06	0	2.29E-06
196.6	30.087	141.303	54619	91	2.59E-08	0	9.67E-10
104.3	100	29.6058	11140	26	0.097832	0	0.001339
0.4	100	142.8727	53567	38	0.001094	0.000141	0.00157
5.9	100	29.8792	9053	21	0.295515	0.000141	0.001815
27	43.183	81.1508	30705	39	0.001938	0.000141	0.001642
0	100	74.1912	30177	17	0.029103	0.000273	0.001945
4.8	100	17.7521	5147	44	0.001687	0.000273	0.002008
16.3	86.167	119.8034	45160	32	0.021096	0.000525	0.002632
66.7	53.069	65.6088	23928	43	0.000954	0.000525	0.002537
49.5	68.183	87.7035	33277	35	0.010309	0.000758	0.003213
10.3	100	26.5593	7990	40	0.002704	0.000758	0.002783
38.1	100	15.9429	4388	20	0.326925	0.000758	0.00286
16	100	68.77	25390	31	0.020852	0.000868	0.003338
8.6	100	67.1718	23047	33	0.005871	0.000951	0.003719
2.2	100	128.9449	47480	30	0.044345	0.001022	0.005643

103.3	100	52.2816	18503	26	0.100506	0.001022	0.004608
0	100	109.1072	42349	30	0.012243	0.001022	0.005479
49.6	41.132	48.1614	18943	38	0.005088	0.001022	0.00482
26.5	100	64.2762	23744	32	0.017057	0.001022	0.00553
0.6	100	124.2116	48533	32	0.013924	0.001022	0.004789
13.2	100	123.6168	48296	32	0.012614	0.001022	0.005372
6.6	100	62.6934	23096	51	8.58E-05	0.001114	0.006185
1.3	100	129.4009	48109	35	0.013189	0.001114	0.006158
8.8	100	124.1205	48498	33	0.011024	0.00121	0.006498
37.5	100	46.0978	18075	33	0.005826	0.002012	0.01051
9.1	100	29.0951	10937	17	0.770092	0.002079	0.01141
28.2	100	79.976	29952	38	0.001957	0.002376	0.0154
9.4	100	26.5254	7979	33	0.013743	0.002543	0.01603
4.6	100	85.8072	30862	33	0.018548	0.00302	0.01957
21.3	100	68.1461	23432	19	0.406348	0.00337	0.02292
88.6	100	63.7636	23539	23	0.119516	0.00337	0.02229
10.6	100	81.046	30664	19	0.190164	0.003674	0.02548
24.6	100	44.0764	13982	9	3.808522	0.003674	0.0264
0	100	70.1145	28409	25	0.034703	0.007836	0.0569
1.8	100	128.874	47449	19	0.533235	0.007836	0.05685
28.8	100	15.9277	4497	10	3.609508	0.008361	0.06271
94.5	100	61.7901	20837	31	0.031853	0	0.00099
20.8	100	62.155	20987	55	0.000132	0	4.39E-05
10.5	100	68.3011	23490	38	0.003948	0	2.28E-06
25.9	64.543	68.2676	23477	30	0.031417	0	1.09E-05
35.6	100	67.7641	23281	8	4.04808	0	0.000348
2.1	100	42.3346	13353	59	4E-05	0	0.000805
23.8	100	116.4874	42502	56	3.28E-05	0	9.2E-08
16.5	82.16	115.9779	42281	34	0.004781	0	3.53E-05
44.5	100	73.6842	25272	12	3.298147	0	0.000284
27.1	20.973	73.5238	25198	66	1.39E-05	0	3.17E-08
19.2	100	65.2564	23713	37	0.006952	0	0.000865
13.9	100	68.4738	25468	55	7.36E-05	0	9.6E-05
23.8	26.188	71.4655	26668	92	3.19E-08	0	9.39E-09
9.2	100	71.5283	26695	61	3.98E-05	0	1.79E-05
20.2	100	48.4273	16928	50	0.000187	0	2.44E-05
10.6	100	66.5942	24343	60	2.48E-05	0	1.01E-05
78.5	29.051	141.1555	54525	68	3.79E-06	0	9.4E-06
10.7	100	60.3675	24201	38	0.005717	0	0.001144
116.4	100	35.3919	12337	54	0.000117	0	0.000421
117.4	66.029	41.4192	14301	50	6.97E-05	0.000141	0.00167
6.2	100	17.2477	4963	61	1.34E-05	0.000273	0.001992
83.8	100	42.6872	15089	30	0.030711	0.000525	0.002637
8.5	100	66.7227	24396	34	0.008269	0.00065	0.002671
107.5	40.56	35.1858	12247	59	3.3E-05	0.000758	0.00315
21.7	100	65.8483	22201	36	0.007082	0.000951	0.004057
11	20.778	141.1576	54527	36	0.005901	0.000951	0.003801
10.5	100	116.1543	42355	34	0.005563	0.000951	0.004026
11.2	100	45.2236	15705	57	4.68E-05	0.001022	0.00556
21.4	100	60.8762	24429	27	0.068138	0.001022	0.004643
0.8	100	116.9916	42709	27	0.025675	0.001022	0.005648
15.9	100	64.7539	23505	29	0.032745	0.00121	0.006456

19.9	100	40.9143	14112	49	8.56E-05	0.001308	0.006988
3.5	100	74.0386	25424	30	0.049752	0.001576	0.008857
27.7	100	73.1069	25023	12	3.440076	0.001576	0.008455
81.4	100	58.3334	19349	35	0.010747	0.001854	0.009804
14.5	100	37.9719	13320	47	0.000391	0.002012	0.01095
4	100	67.7951	23294	26	0.065645	0.002376	0.01545
2.3	100	115.6445	42147	21	0.120941	0.002843	0.0192
6.2	100	73.0068	24986	19	0.62097	0.002843	0.01851
4.9	100	116.6639	42579	27	0.023278	0.002931	0.01956
34.2	100	42.6323	15065	27	0.070428	0.00337	0.02319
106.5	100	13.1293	3646	24	0.16581	0.00337	0.02211
43.5	100	56.4419	18728	28	0.051559	0.00337	0.02421
26.1	100	41.1537	14203	32	0.003547	0.004049	0.02921
0.4	100	23.6592	7053	30	0.04265	0.004266	0.03071
0.4	100	23.6592	7053	30	0.04265	0.004266	0.03071
14.9	100	41.9238	14474	37	0.001121	0.005618	0.03866
4	100	70.9623	26466	17	0.857335	0.005865	0.04306
50.3	100	57.9553	19349	25	0.118001	0.00599	0.04541
18.4	100	43.1379	15272	29	0.033816	0.006872	0.04992
231.5	51.448	56.9458	18937	29	0.045174	0.007057	0.05236
229.8	72.166	57.4492	19140	33	0.017575	0.009514	0.07399
9.4	100	68.0796	25096	45	0.001259	0	0.000326
37.6	100	67.5743	24895	48	0.000695	0	8.69E-06
83.9	100	73.352	25573	46	0.001122	0	0.000793
18.2	100	81.5478	33209	40	0.000615	0	1.19E-05
13.7	100	81.4456	33168	26	0.016447	0	0.001072
8.9	100	98.4645	35106	51	0.000222	0	1.18E-05
17.5	100	98.3873	35075	49	0.000377	0	3.15E-06
6.3	100	140.7465	52410	68	4.7E-06	0	1.32E-11
15.3	100	62.2221	22478	30	0.038	0	0.000486
61.4	100	141.1778	54538	86	9.65E-08	0	4.11E-10
11	59.552	141.3185	54629	75	1.13E-06	0	1.76E-09
57.9	100	92.4398	34268	59	4.39E-06	0	6.13E-06
30.3	100	93.4498	34651	52	2.68E-05	0	8.88E-06
70.5	61.22	92.9464	34468	59	5.28E-06	0	9.45E-06
53	100	89.816	33521	45	0.001005	0.000141	0.001661
5.9	100	89.9512	33578	41	0.00267	0.000273	0.002026
133.3	100	75.6032	26040	40	0.004092	0.000525	0.002384
103.9	81.63	58.816	21123	29	0.049037	0.000525	0.002368
4.8	100	82.8979	29669	28	0.060799	0.000758	0.00293
15.8	100	83.4004	29858	24	0.179343	0.000868	0.003324
7.8	100	92.5632	34319	35	0.001084	0.000951	0.003976
48	100	52.5735	20878	41	0.001591	0.001022	0.004888
71.3	100	52.0698	20656	43	0.000952	0.001114	0.00632
3.9	100	67.069	24697	31	0.031453	0.001576	0.008837
0.4	100	128.7265	49000	26	0.123742	0.002543	0.01611
26.6	100	76.1086	26234	42	0.00229	0.002719	0.01683
18.6	100	33.3035	12672	9	5.627518	0.002777	0.01801
80.9	100	61.5067	22243	15	0.210055	0.002843	0.01899
77.7	30.231	28.9103	9360	24	0.121355	0.003533	0.02477
0.3	100	97.9572	34904	13	1.285647	0.003674	0.02505
11.5	100	90.5554	33827	29	0.038745	0.003736	0.02757

26.6	100	74.5122	26077	16	0.894323	0.004643	0.03321
102.8	38.165	28.7912	9315	21	0.266361	0.005325	0.03633
13.3	100	73.3806	25586	25	0.118791	0.007423	0.05399
15.3	100	99.8616	37490	45	0.000605	0	0.000151
92.6	100	140.5921	52024	77	8.66E-07	0	8.86E-10
14.6	100	140.6052	52030	84	1.86E-07	0	8.02E-09
48.8	100	14.5975	4054	15	1.063023	0	7.84E-05
59.9	100	140.7792	52110	83	1.28E-07	0	9.85E-09
7.7	100	132.2009	54242	39	0.003037	0	0.000247
130.8	100	14.367	4076	15	1.128359	0	1.91E-05
49.4	100	14.0427	3937	6	10.51831	0	0.000254
0	48.004	141.1338	57776	74	5.56E-07	0	2.99E-06
53.8	1.614	140.8073	57617	78	3.42E-07	0	1.47E-07
155.7	0.822	140.8066	57616	87	4.84E-08	0	2.29E-08
29.5	60.246	140.6253	57526	75	1.39E-06	0	3.06E-08
273.7	6.363	140.6159	57521	112	2.96E-10	0	6.86E-12
113.5	0.879	140.6152	57520	77	8.4E-07	0	3.12E-09
19.7	66.46	140.5867	57506	111	2.51E-10	0	6.22E-09
8.1	25.63	140.585	57505	55	0.000117	0	0.000112
76.7	100	14.3034	4049	6	9.066675	0	0.000958
13.2	100	26.508	9150	24	0.175947	0	0.001056
18	40.632	140.6277	55288	41	0.004154	0	0.000213
96.8	56.484	41.3537	13005	42	0.001672	0	0.000666
21.8	100	41.8561	13181	42	0.001435	0	0.000949
79.3	13.604	88.0696	33442	45	0.000594	0	1.77E-06
48.6	5.161	87.5655	33217	42	0.00129	0	2.31E-06
16.6	100	100.0027	36806	46	0.000395	0	5E-07
11.7	55.092	100.1162	36859	59	1.92E-05	0	6.48E-08
3.2	100	101.1257	37286	34	0.006973	0	2.36E-05
7.3	52.128	100.6214	37079	66	4.91E-06	0	4.3E-08
21.4	100	100.5056	37029	52	0.000108	0	3.07E-08
25.7	60.731	62.75	22740	49	0.000187	0	7.52E-07
32.4	100	12.833	3518	53	0.000103	0	5.38E-05
18.8	14.861	63.2557	22964	52	0.000111	0	4.01E-07
13.1	100	63.7582	23167	30	0.015802	0	0.000994
37.3	60.331	123.8437	46922	70	4.21E-06	0	2.93E-09
2.9	100	114.7761	42981	68	5.82E-06	0	1.86E-06
42.5	75.467	83.4601	31423	71	1.66E-07	0	6.29E-07
80.6	100	45.2629	17702	30	0.021109	0	1.5E-05
407	100	13.0403	3719	59	2.49E-05	0	1.88E-05
88.1	99.409	12.7396	3520	50	0.000218	0	4.43E-05
89	100	20.2535	7201	26	0.114549	0	0.001126
10	100	25.8963	9645	24	0.145415	0	0.001228
15.4	100	24.8043	9171	26	0.093208	0	0.001332
74.7	57.967	24.8959	9212	31	0.031299	0	0.000134
18	100	40.4257	15638	38	0.006536	0	1.09E-06
67.2	100	40.7074	15751	33	0.018374	0	1.45E-07
13	100	40.9289	15838	36	0.010182	0	5.29E-06
22.6	100	90.2194	32687	28	0.054335	0	0.000477
25	100	40.2028	15535	10	3.664882	0	0.000597
25.3	100	40.847	12821	41	0.00205	0.000141	0.001578
4.8	100	40.6722	15736	29	0.055759	0.000141	0.001645

6.8	72.681	124.1821	47072	27	0.07371	0.000273	0.002039
34.8	100	25.3084	9398	20	0.360273	0.000525	0.002594
10.4	100	22.9802	8384	19	0.525545	0.00065	0.002775
19.6	100	62.8878	22801	23	0.087401	0.000758	0.002852
8.5	100	124.3488	47151	28	0.06817	0.000951	0.003936
121.2	100	38.7622	12078	35	0.008021	0.000951	0.004207
39.9	100	57.1424	20927	44	0.000824	0.001022	0.005172
28.1	100	56.3657	20118	36	0.004817	0.00141	0.007278
9.6	100	83.5555	31467	30	0.001893	0.001576	0.008288
4.8	100	83.0457	31246	32	0.001493	0.001576	0.008329
1	100	99.3578	37272	30	0.023084	0.002012	0.01064
51.1	100	39.2844	12254	35	0.007974	0.002079	0.01158
0	100	11.6413	3190	49	0.000115	0.002079	0.01177
196	29.656	57.6465	21120	50	0.0002	0.002257	0.01292
166.5	100	38.7806	12086	30	0.021168	0.002315	0.01296
12.3	100	63.3893	23018	32	0.011155	0.002315	0.01337
67.4	100	57.3785	20515	43	0.001008	0.002543	0.01562
28.4	100	58.1667	21320	39	0.002726	0.002777	0.01744
2.5	100	39.1122	12218	43	0.000526	0.002777	0.01806
0	100	12.0376	3431	43	0.000469	0.002777	0.01754
0.8	100	114.3744	42804	24	0.14266	0.003254	0.02097
30.5	100	41.3878	13019	42	0.001483	0.00337	0.02391
0.9	100	96.1175	35992	20	0.203437	0.00337	0.02315
59.8	100	43.9777	15132	41	0.001807	0.003674	0.02499
115.3	99.407	27.5936	9067	48	0.000143	0.003736	0.02762
134.8	5.462	56.8697	20312	47	0.000366	0.003736	0.02762
15	100	88.013	33419	30	0.021216	0.004192	0.03045
7.8	100	100.2625	37663	36	0.004674	0.004643	0.03313
2.1	100	37.2152	11532	38	0.001967	0.005196	0.03557
22.9	100	52.8426	19220	32	0.01149	0.005325	0.03708
5.1	100	99.2557	37229	25	0.067988	0.00573	0.04024
2.8	100	64.262	23371	23	0.079382	0.00573	0.04077
11.6	100	13.029	3715	31	0.015698	0.006428	0.047
26.1	100	51.9198	17000	31	0.015829	0.007969	0.05831
49	100	43.4737	14929	32	0.015297	0.009291	0.07084
1.1	100	115.7123	43333	55	0.000137	0	0.001281
49.6	100	65.7194	22159	40	0.001103	0	3.69E-05
10.2	100	65.7096	22155	70	1E-06	0	2.13E-05
18.5	100	65.2152	21962	36	0.002665	0	0.000153
19.3	100	111.4411	40393	81	3.23E-07	0	1.52E-06
35.4	79.766	111.4106	40380	65	1.41E-05	0	9.34E-06
3.5	100	110.9045	40184	45	0.001221	0	0.000706
30.1	100	105.881	38058	26	0.106482	0	4.89E-05
4	100	94.1289	35462	62	2.02E-05	0	0.000185
7.2	100	103.5835	39557	60	4.4E-05	0	7.73E-05
9.6	100	103.5717	39551	47	0.000704	0	0.00082
20.1	100	106.0158	40595	71	3.21E-06	0	3.67E-07
13.3	100	105.9715	40574	76	1.08E-06	0	4.08E-08
5.8	100	105.4605	40340	44	0.001929	0	2.9E-05
6.6	100	132.1756	51579	72	8.41E-07	0	7.68E-06
48	67.587	132.1407	51565	78	2.12E-07	0	3.01E-08
14	100	131.6389	51374	51	0.000113	0	0.000338

48.8	8.903	75.7576	28219	62	2.14E-05	0	2.97E-05
5.2	100	116.663	43806	42	0.002576	0	0.000642
62.4	100	141.6444	54814	77	1.67E-07	0	5.19E-10
54.2	9.749	141.1287	54507	93	8.32E-09	0	2.43E-11
5.6	100	141.6778	54831	95	7.74E-09	0	2.23E-12
25.5	100	63.8282	25709	57	7.13E-05	0	1.22E-05
9.9	38.988	73.4818	29880	63	1.89E-05	0	9.76E-07
12.1	100	72.9802	29661	60	3.95E-05	0	1.29E-06
40.6	100	36.7817	14141	44	0.001405	0	0.000871
6	98.381	140.8456	54339	23	0.228514	0.000141	0.001622
0	100	133.6718	52132	55	2.09E-05	0.000273	0.001819
18.5	100	54.2479	21595	40	0.003186	0.000525	0.002274
9.7	100	105.1271	39550	42	0.00284	0.00065	0.002759
5.4	100	116.6303	43790	41	0.00292	0.000868	0.003548
17.5	100	54.7264	19968	36	0.008552	0.000951	0.003955
434.1	63.645	17.3816	5183	14	0.039939	0.001022	0.005385
18.3	100	50.3731	19915	33	0.013144	0.001576	0.008649
1.2	100	131.6703	51386	29	0.017487	0.002012	0.01033
89.7	100	17.3966	5190	42	5.59E-05	0.002079	0.01141
3.8	100	115.82	43368	26	0.12077	0.002315	0.01409
3.3	100	109.0284	40549	44	0.001886	0.002376	0.0142
1.1	100	112	43530	33	0.014453	0.00337	0.02297
1.1	100	112	43530	33	0.014453	0.00337	0.02297
63.4	100	17.5208	4930	14	0.042285	0.003456	0.02428
16.1	100	76.0217	28326	24	0.153705	0.004192	0.03069
0.5	100	103.0679	39336	20	0.448928	0.004643	0.03328
121.4	100	54.7949	19997	23	0.168929	0.005271	0.03614
7.8	100	110.939	40198	32	0.02351	0.007636	0.05502
6.4	100	65.2075	21959	28	0.015297	0.009514	0.07389
36.7	40.966	48.1017	15497	53	0.000191	0	2.58E-07
25.9	100	48.2927	15574	30	0.030297	0	7.45E-06
13.1	100	47.7917	15376	33	0.016841	0	0.000193
32.6	100	30.768	9343	26	0.081303	0	0.000105
55.5	100	72.3707	25174	44	0.001325	0	0.000568
25.9	100	63.7906	21634	34	0.014241	0	0.00072
76.8	70.521	64.2935	21839	47	0.000741	0	8.4E-05
17.5	100	130.9727	48297	63	1.45E-05	0	4.1E-07
7.6	100	67.3533	22791	64	1.04E-05	0	0.001496
3.5	100	61.7287	20606	48	0.000311	0	0.000157
25.4	100	22.5282	6603	37	0.009172	0	0.000638
52.3	73.772	44.1248	14002	72	2.75E-06	0	8.42E-09
169.1	43.489	48.1722	15498	50	0.00029	0	0.000654
30.1	100	47.6692	15314	49	0.000376	0	0.000818
5.3	100	135.0439	49844	31	0.032586	0	0.000286
10.6	100	135.0625	49852	65	1.13E-05	0	3.92E-09
23.6	100	113.5407	41247	77	6.76E-07	0	1.33E-06
7.8	100	113.5614	41257	39	0.004052	0	5.88E-05
2.3	100	135.1922	52852	48	0.000592	0	2.04E-05
14.7	100	98.7222	37428	49	0.000552	0	0.001295
0.8	100	96.432	36440	45	0.001379	0	3.96E-05
284.7	86.078	140.6843	54240	105	3.04E-10	0	7.43E-09
7.3	100	116.6207	43786	33	0.01497	0	0.001324

176.8	19.404	141.1999	54556	87	6.36E-08	0	3.76E-09
3.7	47.853	141.2065	54562	77	5.45E-07	0	1.18E-06
38.1	100	57.621	23045	48	0.00064	0	0.000551
100.6	79.246	141.0004	54431	69	4.46E-06	0	4.53E-08
209.8	100	43.9643	13942	23	0.205965	0.000141	0.001589
2.1	100	85.7928	30012	52	9.57E-05	0.000141	0.00171
5.3	100	130.47	48103	27	0.064437	0.000141	0.001754
1.4	100	135.5697	50058	29	0.049979	0.000273	0.002055
2.6	100	135.1222	52823	26	0.084698	0.001022	0.005525
15.2	100	71.8618	24965	40	0.003202	0.001114	0.006024
9.1	100	71.9386	24997	33	0.018025	0.001576	0.008295
11.2	100	52.356	20781	43	0.001336	0.001945	0.01023
18.8	100	47.5924	15304	19	0.419458	0.002777	0.01786
75.3	100	60.0492	20144	41	0.000851	0.00337	0.02234
18.3	100	58.1478	21313	25	0.114268	0.003736	0.02723
20.7	100	67.2303	22747	15	1.246425	0.004124	0.02977
5.5	100	43.9452	13936	36	0.01087	0.004192	0.03001
0.5	100	134.5602	49653	15	1.17411	0.005271	0.03597
30.7	100	58.2481	21347	16	0.964949	0.00573	0.04036
12.8	100	140.3059	51889	65	1.22E-05	0	1.75E-07
6.6	100	140.2949	51884	94	1.57E-08	0	1.53E-09
0	100	140.058	51784	89	1.71E-08	0	1.27E-08
0	100	140.0402	51777	40	0.001903	0	0.000163
7.6	100	135.4507	50235	102	2.6E-09	0	1.63E-09
0.5	100	134.9487	50067	79	5.24E-07	0	3.68E-08
0.8	100	140.0643	57261	101	1.25E-09	0	4.17E-11
8.8	100	136.9287	50622	39	0.006067	0	0.000827
41.7	100	137.4301	50843	69	5.77E-06	0	8.75E-08
35.9	100	96.5513	34361	39	0.005646	0	2.67E-05
6.4	100	74.8865	25763	27	0.084902	0	0.000261
25.3	77.835	119.6132	46279	89	5.73E-08	0	1.52E-06
109.4	51.521	98.6049	37375	61	2.78E-05	0	0.000989
36.7	100	140.5463	52294	42	0.002825	0	6.61E-05
7.9	100	89.027	33873	39	0.00408	0	0.001438
30.9	100	74.373	27876	42	0.001584	0	0.00011
28.9	100	48.6539	19171	36	0.006066	0	0.000588
84.9	12.871	42.285	16424	64	1.58E-05	0	5.7E-06
37.9	50.594	82.2976	29425	52	0.00021	0	0.000501
31.9	50.244	80.167	28536	43	0.0023	0	6.99E-06
180.5	57.295	75.7077	30800	41	0.003281	0	5.75E-05
0	100	73.1705	29742	68	5.33E-06	0	6.56E-05
6.2	100	74.4874	30300	60	3.61E-05	0	1.14E-07
203.1	100	19.5734	6880	39	0.002118	0	2.96E-05
16.8	100	82.7991	29631	46	0.000887	0.000141	0.00157
16.1	100	96.8756	34483	19	0.56323	0.000273	0.00189
2.3	100	74.205	27801	44	0.000989	0.000525	0.00227
1.7	100	134.8749	51634	37	0.008346	0.000525	0.002487
39.7	100	52.5486	19680	43	0.000293	0.000525	0.002616
4.5	100	74.4201	30274	20	0.384501	0.000525	0.002342
6.4	100	74.6915	28000	36	0.0071	0.000868	0.003374
16.8	100	83.3045	29825	39	0.004486	0.000951	0.0044
22	100	75.4185	28316	26	0.063983	0.001022	0.005549

1.1	100	135.5368	50266	30	0.040373	0.001022	0.005499
14.1	100	42.0221	16307	38	0.007815	0.001114	0.006298
30.1	100	67.0601	23001	15	1.467149	0.00121	0.006744
64.9	100	79.6625	28319	6	11.70361	0.002257	0.0125
110.5	99.946	54.2942	21616	36	0.004832	0.002376	0.01522
50.4	100	46.4809	16162	15	0.274184	0.002376	0.01477
0	100	110.7807	43030	35	0.011549	0.002719	0.01646
7	100	74.1776	27789	26	0.060945	0.002777	0.01734
6.4	100	89.3866	34031	25	0.103637	0.002777	0.01794
4.9	100	97.0579	34557	8	5.802223	0.003674	0.02577
13.3	100	83.8121	30025	26	0.074509	0.003967	0.02869
33	100	37.5907	11662	26	0.003821	0.004424	0.03172
45.5	100	54.8003	21835	35	0.006474	0.004581	0.03253
11	100	79.707	28340	6	13.17151	0.007057	0.05223
2.2	100	92.9987	34743	33	0.014081	0	0.001475
9.8	100	80.2526	29785	53	0.000183	0	0.000142
33	100	80.7586	29986	56	9.68E-05	0	1.27E-05
9.5	100	79.3926	32345	51	0.000255	0	0.000139
0	100	93.4793	38300	36	0.00582	0	0.000748
0	100	92.9762	38069	62	1.79E-05	0	8.93E-06
3	100	128.8482	47437	41	0.003995	0	0.000111
18.9	100	98.5179	37335	45	0.000574	0	8.22E-06
17.4	100	113.6553	43757	84	7.34E-08	0	1.71E-09
34.5	100	55.747	20320	46	0.001047	0	1.01E-05
32.7	52.3	55.2351	20115	47	0.000899	0	1.65E-05
32.2	100	14.75	4432	55	0.000124	0	0.000201
34.6	48.992	102.0753	37702	69	4.39E-06	0	3.48E-06
114	100	16.7179	5439	32	0.015876	0	0.001167
14.5	100	76.1541	28628	64	1.36E-06	0	5.77E-07
12.5	100	89.6274	34226	75	9E-08	0	1.7E-07
7.5	100	90.038	34380	45	0.000108	0	5.38E-05
4.2	100	90.6337	34622	51	2.62E-05	0	3.88E-05
22.6	89.454	90.1295	34416	60	3.56E-06	0	1.09E-06
4.4	100	82.8524	30577	78	8.11E-09	0	1.8E-07
51	94.401	82.3479	30386	87	9.88E-10	0	4.07E-09
8.8	100	82.2989	30366	40	4.83E-05	0	0.000493
7.6	100	82.1889	30326	53	2.4E-06	0	4.87E-05
76.4	10.114	140.817	54319	77	8.98E-07	0	7.65E-11
66.8	63.818	81.8435	30186	84	2.05E-09	0	1.54E-09
3.4	100	79.5449	29236	59	2.39E-07	0	7.02E-05
14.5	100	81.7922	30163	36	0.000144	0	0.000932
4.7	100	81.6864	30119	41	3.78E-05	0	0.000309
10.8	100	81.3389	29980	68	7.59E-08	0	6.9E-07
23.4	100	81.2459	29940	72	3.3E-08	0	1.98E-08
61.6	100	21.8631	7900	37	0.00728	0	0.000869
4.5	100	119.1545	44890	39	0.003207	0.000525	0.002516
7	100	93.442	34009	26	0.054419	0.000758	0.002859
20.8	100	21.9291	6991	43	0.001904	0.000868	0.003308
16.2	100	101.578	37472	36	0.008085	0.000868	0.00358
74.4	100	57.7138	20668	26	0.034925	0.000951	0.004092
79.8	60.045	102.0854	37707	42	0.001957	0.000951	0.00386
98.6	31.724	72.1791	26744	48	0.000548	0.000951	0.003822

1.3	100	80.7384	29709	21	0.004168	0.000951	0.004032
2	100	118.6527	44664	34	0.010943	0.000951	0.003925
91.9	100	14.9799	4340	15	1.158006	0.00141	0.007234
0.8	100	79.0408	29042	29	0.000231	0.001945	0.01007
70.8	27.193	57.2429	19059	36	0.006005	0.002315	0.01342
3.7	100	120.4214	44100	26	0.081996	0.002843	0.01898
1.7	100	113.1506	43540	18	0.302478	0.003674	0.02645
36.2	100	71.6786	26552	32	0.026264	0.0085	0.06343
59.5	100	43.2574	14947	23	0.019871	0	0.001199
0	100	133.5748	49564	37	0.007685	0	0.000677
2	100	119.5644	49121	93	2.48E-08	0	2.47E-09
5	100	133.6109	54831	63	2.06E-05	0	1.88E-06
29.2	100	65.1926	22208	53	9.92E-05	0	2.56E-05
13.1	100	65.435	22317	50	0.000208	0	1.14E-05
68.5	46.259	65.6941	22427	58	3.85E-05	0	4.7E-07
25	100	79.8938	27708	37	0.007085	0	0.000246
82.8	45.139	79.9828	27743	49	0.000591	0	0.000175
41.3	54.612	116.6374	45054	74	7.38E-07	0	9.81E-06
17.7	100	113.8774	42595	61	2.88E-05	0	0.00034
7.1	100	111.6716	41647	50	0.000446	0	3.58E-07
5	100	119.3246	44953	48	0.000579	0	7.75E-06
39.2	67.704	116.3585	43664	29	0.047744	0	0.0007
416.7	100	140.7434	54273	85	1.38E-07	0	1.27E-10
4	100	91.8218	33340	39	0.005168	0	3.53E-05
3.5	100	91.8158	33337	42	0.00235	0	1.05E-06
25	100	72.7342	25318	22	0.215379	0.000273	0.001892
11.1	100	70.5561	24043	35	0.00705	0.000525	0.002364
7.8	100	91.314	33134	22	0.268159	0.000525	0.00234
2.5	100	133.5361	49550	37	0.008519	0.00065	0.002746
24.5	100	99.7349	36681	26	0.122658	0.000951	0.004377
1.8	100	120.068	49331	26	0.117267	0.000951	0.003833
22.8	100	73.2394	25526	17	0.678225	0.000951	0.00389
28.4	100	78.8172	29230	41	0.003338	0.001022	0.005295
4.3	100	85.6491	29955	22	0.129269	0.001758	0.009079
29.3	100	58.1231	19271	21	0.079146	0.002543	0.01578
157.4	54.119	32.1923	9869	35	0.005689	0.003096	0.01973
0	100	133.6281	54838	17	0.809097	0.003254	0.02122
26.3	100	80.2496	27848	33	0.018535	0.00337	0.02392
3.2	100	87.159	30593	24	0.180936	0.00337	0.02218
15.3	100	20.0471	6280	42	0.000588	0.003736	0.0278
65.7	100	58.169	19289	17	0.211985	0.006145	0.04574
16.5	100	79.8819	27703	24	0.177534	0.008034	0.05915
8.2	100	56.7004	20243	16	0.255496	0.008361	0.06249
3.6	100	20.136	5923	35	0.002309	0.008962	0.06771
45.9	100	44.4942	15967	5	1.733213	0	0.000141
31.4	100	45.5032	16354	21	0.04374	0	0.000232
12.3	100	45.017	16176	26	0.014369	0	0.000764
97.1	89.648	44.9991	16170	28	0.008921	0	4.76E-08
66	100	38.9207	13871	32	0.019277	0	3.74E-05
33.8	100	38.8946	13859	29	0.038494	0	3.05E-05
13.6	100	71.0976	24649	49	0.000377	0	0.001359
17.8	100	87.0015	30528	87	7.99E-08	0	8.21E-09

4.4	100	87.5061	30733	44	0.001569	0	0.000133
3.5	100	118.0773	45632	37	0.008449	0	0.000297
31.2	82.838	106.9309	41474	68	4.79E-07	0	3.34E-06
17.5	100	106.929	41473	89	3.95E-09	0	4.7E-08
100.5	18.423	30.4591	11492	33	0.018889	0	0.000387
18.8	100	19.841	5833	29	0.055112	0.000141	0.001637
18.7	100	44.6262	16022	25	0.014101	0.000273	0.001823
19.1	60.306	57.936	23172	7	7.146631	0.000868	0.003567
6.3	100	56.5977	22602	48	0.000634	0.001022	0.005037
2.1	100	106.4156	41252	30	0.002531	0.001022	0.004952
308.6	39.884	17.5833	5904	27	0.063994	0.001114	0.006417
11.6	100	38.4097	13676	12	2.286729	0.001114	0.006057
53.9	100	85.7489	31990	37	0.003103	0.00121	0.006807
22.8	100	36.561	13010	25	0.040925	0.001308	0.006904
22.8	100	36.561	13010	25	0.040925	0.001308	0.006904
108	21.072	81.9666	29295	37	0.004661	0.001576	0.008203
10.5	100	62.1051	22854	28	0.044173	0.001758	0.009416
179.6	23.173	42.6865	16599	49	0.000238	0.001758	0.009628
36.9	100	52.1214	18435	20	0.334868	0.002079	0.01168
0	100	135.9996	53192	25	0.136451	0.002777	0.01747
0	100	84.9018	34603	12	0.290569	0.002931	0.01954
12.9	100	62.5213	23023	30	0.031778	0.003892	0.02855
9.7	100	58.4838	23402	18	0.559047	0.003967	0.02871
10.8	100	18.8416	6541	37	0.008366	0.004192	0.03054
0.5	100	107.4663	41705	14	0.105035	0.005325	0.03665
1.7	100	136.743	53507	20	0.473645	0.005618	0.0383
0	100	136.5075	53404	16	1.085149	0.006926	0.05034
13.7	100	46.9187	16902	7	1.2922	0.007423	0.05389
23.6	100	17.6883	5962	23	0.182133	0.009514	0.0752
32.3	100	98.6182	36974	48	0.000363	0	0.000309
5	100	98.153	36798	49	0.000344	0	0.00061
22.5	100	63.1842	23205	55	4.75E-05	0	0.001491
32.7	73.091	65.0074	23896	66	9.96E-06	0	1.02E-06
3.6	100	64.6849	23778	49	0.000503	0	0.000472
19	100	23.1456	7452	15	1.48312	0	0.001209
21.5	100	22.3306	7142	14	1.000981	0	0.000511
26.2	100	21.8247	6955	8	3.93936	0	0.001418
52.3	100	21.5375	6845	26	0.062173	0	0.000151
3.4	100	95.346	35980	30	0.043634	0	0.000379
3.8	100	95.2963	35957	48	0.000629	0	4.81E-05
9.5	100	94.838	35768	16	1.105935	0	0.001273
14.7	100	74.8292	27832	58	7.02E-05	0	4.17E-07
12.1	43.998	75.331	28034	62	2.55E-05	0	2.59E-08
47.2	100	140.9834	54421	13	1.353309	0	0.000547
30.7	100	22.8356	7327	7	5.746795	0.000141	0.001655
15.9	100	60.567	24295	52	0.000142	0.000273	0.002062
8.2	100	74.0155	27727	26	0.081847	0.000758	0.003074
3.8	100	102.6506	36772	39	0.004128	0.000758	0.003227
33.4	100	45.9885	18027	29	0.029185	0.000951	0.004174
62.9	100	16.3068	5252	38	0.00316	0.001022	0.005717
17.1	100	60.3598	22116	10	1.855954	0.001022	0.004615
69.9	100	38.0014	13029	36	0.005763	0.001022	0.004621

19	100	20.9613	6627	17	0.686245	0.00121	0.006445
19	100	20.9613	6627	17	0.686245	0.00121	0.006445
35.6	100	62.2382	22485	40	0.00163	0.001498	0.007902
35.1	100	26.0235	8531	20	0.399428	0.001576	0.008594
73.9	100	17.7437	5012	26	0.0916	0.001576	0.008511
112.7	100	27.3614	8227	18	0.649618	0.001576	0.008691
63.3	100	31.7236	10726	47	0.000397	0.001945	0.009995
251.7	100	27.8664	8394	20	0.43246	0.002012	0.01093
26.8	100	26.8733	8090	19	0.51398	0.002079	0.01143
211.5	61.905	16.2962	5247	28	0.035174	0.002315	0.01304
6.1	100	56.9159	20841	46	0.000256	0.002543	0.01615
24.9	100	98.1139	36784	27	0.049185	0.002843	0.01931
104.4	100	26.8828	8566	21	0.176647	0.00337	0.02245
43.6	100	37.4972	12841	31	0.018178	0.004643	0.03276
2.3	100	96.0309	34138	11	3.031713	0.004643	0.03306
2	100	95.5241	33937	6	8.640889	0.004643	0.03371
145.3	15.049	70.6037	26123	59	4.69E-05	0	0.000172
74.3	100	70.65	26143	48	0.000633	0	0.000189
75.1	100	21.6228	6880	28	0.02909	0	0.001394
111.8	100	40.0464	13787	34	0.008659	0	0.000181
28.9	100	39.0986	13443	37	0.008801	0	7.08E-07
2.7	100	38.9946	13403	45	0.001584	0	5.42E-05
72.8	100	38.9719	13393	40	0.004634	0	5.32E-08
18.1	100	38.5949	13257	25	0.145784	0	4.74E-05
26.7	100	38.4684	13209	38	0.007115	0	1.53E-06
111.7	100	37.9841	13022	28	0.07778	0	1.48E-06
9.9	100	37.982	13021	48	0.00077	0	3.07E-07
42.2	45.23	37.9645	13015	46	0.0011	0	2.15E-09
20.9	100	37.4816	12835	12	3.244231	0	0.000786
70	100	37.4641	12828	7	10.33994	0	0.000945
71.2	34.239	76.9241	27108	39	0.003605	0	0.001347
15.6	100	50.8563	18455	45	0.001051	0	0.00075
140.8	49.229	140.5997	55272	91	2.36E-08	0	3.04E-11
23.1	100	43.4479	13757	52	0.000135	0	3.15E-05
154	100	43.4385	13753	27	0.047019	0	0.00021
87.1	57.767	42.9406	13572	51	0.000171	0	5.54E-06
17.1	100	42.567	13434	30	0.04933	0	0.000107
26.7	25.774	89.4663	34064	72	2.06E-06	0	2.91E-08
64.3	15.545	85.5916	32362	57	6.35E-05	0	0.001166
160.1	100	125.0127	47417	7	8.363072	0	0.000514
58.9	57.168	129.6008	49382	67	5.94E-06	0	7.3E-05
16.4	100	70.9311	26153	71	1.76E-06	0	0.000159
26.7	55.744	50.2348	19857	58	5.34E-05	0	2.91E-05
55.8	37.731	86.4083	31120	54	0.000182	0	3.95E-06
117.1	6.816	42.2006	16384	47	0.000643	0	0.001145
108	100	42.4963	16515	41	0.002469	0	0.001128
22	100	66.6142	26883	67	8.51E-06	0	0.000477
10.3	100	78.2111	27682	42	0.00292	0	0.001265
13.3	100	85.8971	30901	37	0.008393	0.000141	0.00163
8.2	100	86.5915	32804	56	6.99E-05	0.000273	0.001916
2.5	66.174	125.5146	47631	31	0.030512	0.000525	0.002568
196.3	100	24.5017	8427	29	0.041035	0.000525	0.002503

219.9	42.251	99.9243	36769	63	5.58E-06	0.000525	0.002303
16.3	100	129.0964	49164	37	0.005352	0.000951	0.004494
8.6	100	68.2393	24907	41	0.002918	0.000951	0.003975
62.3	100	42.4028	13376	10	2.26719	0.000951	0.004578
42.5	100	70.4191	25930	50	0.000213	0.000951	0.004154
48.1	100	24.1931	7156	29	0.020833	0.001022	0.005011
11.8	100	89.0452	33882	23	0.093641	0.001022	0.004636
40.5	100	73.8156	25778	47	0.0003	0.001022	0.005309
18	100	99.4189	36534	39	0.001375	0.001114	0.006224
73.6	100	41.8624	16232	37	0.006892	0.00121	0.006694
173.5	25.526	42.9332	13569	12	1.597673	0.00141	0.007383
15.3	100	72.7692	26924	42	0.00208	0.00141	0.007458
15.8	88.833	140.6445	55296	31	0.014934	0.001576	0.008714
11.9	100	94.4815	33546	36	0.003084	0.001758	0.009433
6.2	100	70.1447	25929	31	0.025686	0.001758	0.009039
9.6	40.098	129.5774	49372	36	0.007136	0.001758	0.009235
10.4	100	128.7962	49033	50	0.000408	0.002012	0.0108
15.8	100	35.7378	11018	14	1.858283	0.002079	0.01164
5.2	100	89.2819	31470	53	6.22E-05	0.002079	0.0116
4.6	100	41.932	16264	30	0.031445	0.002257	0.01276
24.8	100	45.3149	16168	21	0.137612	0.002543	0.01604
13.4	100	50.3564	19907	26	0.097649	0.002843	0.01871
24.1	100	25.2196	8685	31	0.015588	0.002931	0.01949
326.6	95.032	55.1629	18125	41	0.001276	0.003967	0.02882
65.6	100	83.7313	29992	35	0.010231	0.004266	0.03077
224.3	14.676	55.2742	18173	43	0.000676	0.005325	0.03649
43.3	100	70.3671	25910	30	0.020215	0.005478	0.03775
14.8	100	85.0821	32143	32	0.025009	0.005687	0.03985
11.3	100	85.6444	32384	29	0.053694	0.005687	0.03976
21	100	37.3478	11580	10	2.235403	0.006926	0.05048
78.2	100	21.6517	6891	16	0.406733	0.007572	0.05445
85	100	84.2416	30202	31	0.027356	0.0085	0.06404
19.5	100	42.4391	13390	11	1.643126	0.008997	0.06835
29	100	66.9279	24645	95	1.31E-08	0	1.78E-08
14.6	100	66.7391	24575	63	1.93E-05	0	1.78E-06
24.7	100	53.3313	17517	23	0.234143	0	0.000599
98.4	100	53.7619	17675	27	0.09727	0	3.78E-07
52.3	100	53.8862	17722	19	0.484682	0	1.42E-05
12.7	100	46.6784	14951	56	8.13E-05	0	1.77E-06
31	100	46.7231	14970	45	0.001079	0	6.96E-06
3.3	100	115.9879	43425	39	0.000133	0	8.67E-05
16.4	100	24.1513	8285	68	4.98E-06	0	9.76E-05
152.1	65.445	21.7086	7321	34	0.013374	0	0.000647
26.9	100	50.8921	16511	25	0.117446	0	0.00046
3	100	16.8622	4819	70	1.94E-06	0	6.28E-05
74.3	100	47.6715	16634	47	0.000656	0	0.000828
9.3	100	75.9457	28291	52	0.000123	0	0.00088
10.6	100	68.767	25240	38	0.001315	0	0.000616
25.6	100	68.694	25212	37	0.001604	0	0.000701
26.3	62.246	71.5117	26398	61	1.3E-06	0	2.89E-09
32.7	100	41.8804	16241	44	0.001258	0	0.000383
35.5	100	41.3683	16016	38	0.005072	0	0.000279

45.6	100	123.636	43737	76	1.21E-07	0	9.48E-08
10.4	98.349	123.7251	43769	53	2.53E-05	0	4.55E-05
49.7	73.349	124.1378	43888	79	6.21E-08	0	2.83E-08
14.8	76.209	124.2279	43918	53	2.52E-05	0	2.88E-05
23.6	100	124.6395	44044	76	1.08E-07	0	6.06E-09
3.7	100	123.2211	43609	41	0.000323	0	0.000182
15.6	100	68.2699	24973	35	0.001391	0	0.000553
141.2	100	41.1613	14206	42	0.000193	0.000141	0.001748
12.5	100	67.428	24832	33	0.018952	0.000141	0.001767
2.7	100	115.3367	43206	27	0.00251	0.000141	0.00167
30.3	100	58.2782	21866	54	6.67E-05	0.000273	0.002028
2.3	100	123.132	43577	40	0.000507	0.000273	0.002007
4.8	100	130.2534	48016	36	0.009637	0.000525	0.002277
33.4	100	50.6276	17818	22	0.045135	0.000758	0.003033
55.1	75.25	47.5436	16583	39	0.003065	0.000868	0.003535
120.4	100	36.1116	12297	18	0.590339	0.001022	0.00512
90.2	100	52.6495	19090	43	0.001273	0.001022	0.004645
176.9	100	17.387	5480	24	0.109571	0.001114	0.006179
26.8	100	23.9196	8191	28	0.04638	0.001114	0.006362
5.9	100	41.3587	16012	32	0.020166	0.001576	0.008789
4.3	100	21.6988	7317	40	0.003786	0.001576	0.008695
8.7	100	62.8618	22749	35	0.009274	0.001576	0.008359
73.8	81.656	121.4063	45866	35	0.006638	0.002012	0.01122
44.6	100	40.6589	14016	23	0.01361	0.002257	0.0125
4.3	100	46.8008	14998	28	0.048903	0.002376	0.01449
20.2	100	62.8716	22753	21	0.236781	0.003674	0.02598
20.2	100	17.0205	5301	34	0.010627	0.009141	0.0706
0.8	100	71.5852	26428	11	0.089931	0.009514	0.07348
3.6	100	100.7838	37880	31	0.03206	0	0.001152
29	100	100.7164	37850	82	2.09E-07	0	1.1E-08
4.2	100	100.2056	37637	68	5.48E-06	0	5.52E-06
2.3	100	99.7018	37414	45	0.001077	0	0.000743
7.9	100	82.3567	30605	44	0.000406	0	0.000273
28.5	37.158	81.8518	30418	67	1.85E-06	0	2.91E-08
39.5	39.224	81.3461	30222	66	2.42E-06	0	1.93E-08
5.4	100	80.8408	30020	37	0.001917	0	0.000224
88.5	100	30.7404	10303	63	1.01E-05	0	3.18E-07
6.1	100	32.9355	10066	60	1.58E-05	0	0.001261
2.2	100	119.9972	44858	49	0.000636	0	5.97E-05
1.4	100	109.9466	41265	43	0.002415	0	0.000348
2.8	100	116.9522	43764	51	0.000422	0	1.29E-06
18	100	119.4787	44662	65	1.62E-05	0	6.45E-07
3.4	100	119.4181	44638	44	0.001874	0	0.000428
2.8	100	119.2188	44564	54	0.000181	0	1.64E-05
45.5	62.349	119.1067	44524	103	2.17E-09	0	1.48E-10
23.1	71.932	118.9746	44474	61	3.95E-05	0	1.14E-06
14.6	100	118.6014	44343	95	1.34E-08	0	1.2E-09
14.9	100	118.4712	44298	51	0.000359	0	5.62E-06
1.4	100	117.9677	44129	44	0.001684	0	0.000108
13.9	100	117.4596	43945	61	3.45E-05	0	3.5E-07
13	100	140.4798	51972	25	0.136961	0	0.000629
121.6	100	140.4217	51945	101	3.62E-09	0	1.65E-09

71.3	12.946	140.4195	51944	65	1.66E-05	0	9.94E-07
1.3	100	139.6453	51642	33	0.024862	0	0.000926
2	100	132.5193	49177	38	0.008116	0	0.000804
4.5	100	133.54	49552	43	0.002708	0	4.67E-05
0.5	100	138.2414	51167	41	0.004135	0	0.000394
1	100	137.0875	50794	45	0.001483	0	0.000626
1.8	100	136.5578	50615	37	0.00984	0	0.000591
4.1	100	136.0533	50442	44	0.001913	0	0.0003
26.2	100	135.5468	50270	31	0.039162	0	0.000676
3.3	100	135.0455	50100	59	6.31E-05	0	5.97E-06
6.5	100	134.544	49928	50	0.00044	0	3.5E-05
6.2	100	118.6352	48725	70	3.91E-06	0	9.66E-07
3.9	100	118.5964	48707	32	0.03083	0	0.001208
14.3	77.674	118.5777	48698	112	2.51E-10	0	8.21E-12
40.5	4.367	118.5759	48697	103	2.11E-09	0	1.44E-10
0.5	100	118.0916	48485	38	0.006787	0	0.001152
12.3	100	118.6796	48745	63	1.48E-05	0	3.5E-05
18.3	100	118.0668	48476	89	5.76E-08	0	1.75E-09
0	100	116.5895	47858	40	0.004326	0	0.00046
11.9	100	120.5973	49546	103	2.25E-09	0	1.01E-10
21.9	100	120.0905	49339	103	2.42E-09	0	6.42E-10
24.9	100	119.5856	49129	103	2.31E-09	0	2.37E-10
4.3	100	119.1825	48965	54	0.000128	0	2.11E-05
36	38.074	119.08	48923	107	8.58E-10	0	8.06E-11
1.8	100	119.6875	49169	52	0.000257	0	0.000167
1.1	100	108.9445	44749	39	0.004472	0	0.001172
2	100	107.9402	44353	36	0.007843	0	0.000859
3.3	100	106.9222	43935	36	0.007974	0	0.001016
6.2	100	106.4095	43720	51	0.000279	0	9.87E-05
7.2	100	107.4317	44153	62	2.19E-05	0	1.2E-05
4.4	100	109.4512	44951	58	5.05E-05	0	1.91E-05
2.6	100	114.0145	46831	44	0.001317	0	0.000825
1.8	100	113.0848	46439	69	5.23E-06	0	1.34E-06
3.5	100	109.7094	45058	63	2.37E-05	0	2.03E-05
4.6	100	131.2101	53815	73	2.02E-06	0	7.62E-07
4	100	129.0954	52970	71	3.29E-06	0	2.48E-06
10.1	100	131.7289	54035	43	0.002221	0	0.000196
1.4	100	123.1179	50573	65	1.29E-05	0	2.44E-06
5.3	100	122.6146	50381	108	6.49E-10	0	5.85E-10
4.3	100	122.1133	50176	91	3.12E-08	0	5.98E-08
6.3	100	121.6071	49959	91	3.11E-08	0	2.91E-09
11.7	100	121.1033	49763	99	6.01E-09	0	8.8E-10
5.1	100	121.0587	49745	44	0.001243	0	0.000251
0.9	100	123.6227	50774	62	2.88E-05	0	1.03E-05
1.9	100	126.411	51889	34	0.016009	0	0.000433
0.9	100	126.0079	51717	44	0.001387	0	0.000537
1.7	100	124.6275	51174	62	2.96E-05	0	5.85E-06
2.3	100	127.5715	52361	46	0.001049	0	0.000253
6.2	100	78.8797	32122	65	1.17E-05	0	5.72E-08
23.5	100	78.3777	31921	46	0.000993	0	8.64E-06
54.9	100	83.2161	33896	52	0.00021	0	0.000292
114.9	2.628	82.7088	33689	56	7.21E-05	0	0.000495

80.7	11.324	82.4144	33567	60	3.78E-05	0	0.000113
39.2	83.693	100.3763	41246	93	1.58E-08	0	1.72E-09
54.3	19.56	99.8714	41030	92	2.23E-08	0	3.67E-09
29.8	100	99.3658	40812	75	1.16E-06	0	3.15E-08
53.1	100	99.1363	40711	97	6.53E-09	0	5.68E-10
12.6	100	98.8979	40616	56	7.61E-05	0	2.23E-05
11.7	100	100.8801	41450	79	3.85E-07	0	5.03E-08
1.9	100	104.9354	43070	39	0.003686	0	0.000447
5.3	100	103.9012	42643	47	0.000677	0	0.000109
5.7	100	103.4004	42436	63	1.93E-05	0	1.17E-05
3.9	100	102.8972	42241	60	3.51E-05	0	1.91E-05
7	100	102.3943	42048	69	4.37E-06	0	3.51E-06
6.6	100	101.8879	41852	71	3.25E-06	0	2.21E-06
7.4	100	101.3847	41652	63	1.81E-05	0	1.19E-05
6.2	100	95.9074	39327	55	0.000111	0	6.88E-05
103.8	3.073	41.5714	14852	72	2.22E-06	0	1.34E-05
102.1	100	32.9165	11657	32	0.012899	0	0.000197
148	30.222	32.8678	11637	48	0.000181	0	3.84E-05
148	100	32.3864	11445	51	0.000153	0	2.3E-06
48.5	100	32.3604	11434	38	0.001947	0	0.000984
25.5	47.361	31.9162	11257	72	1.2E-06	0	5.97E-07
113.6	6.547	31.884	11246	72	1.23E-06	0	1.73E-07
79.5	100	41.0678	14656	45	0.001407	0	0.001132
36.3	100	31.3818	11055	50	0.000217	0	1.3E-05
11	100	140.2053	57326	97	8.51E-09	0	7.79E-09
10.4	100	140.1463	57299	77	6.38E-07	0	9.59E-08
8.8	100	139.7024	57105	93	2.03E-08	0	1.59E-09
7.2	100	139.6402	57082	69	4.05E-06	0	4.47E-07
5.3	100	139.1364	56883	58	5.09E-05	0	3.01E-05
4.6	100	138.6356	56692	47	0.000731	0	0.000145
36.4	100	140.4649	57447	73	2.38E-06	0	5.57E-07
50.8	100	140.4693	57449	77	1.06E-06	0	1.6E-07
115.4	100	26.768	9245	43	0.001027	0	4.72E-05
9.2	100	78.61	29154	40	0.000999	0	0.000287
11	100	98.5896	37369	43	0.001776	0	0.000223
7.5	100	98.0165	37107	64	1.37E-05	0	0.000149
17.3	100	140.1851	52112	26	0.046456	0	7.94E-09
10.2	100	111.8467	43460	45	0.001171	0	5.44E-05
44	100	139.9479	53873	44	0.000899	0	3.25E-07
7.7	100	113.8347	42576	70	3.34E-06	0	0.000157
19.7	100	139.1152	53482	69	6.22E-06	0	9.88E-08
28.2	91.872	116.5081	45429	101	3.06E-09	0	5.97E-10
30.6	20.801	116.491	45422	51	0.000343	0	1.87E-05
61	100	140.1362	53963	107	7.86E-10	0	5.26E-12
9	100	121.7138	47538	52	0.000239	0	0.001084
18	100	128.966	49107	40	0.002409	0	0.001098
10.4	100	129.4676	49330	48	0.000426	0	0.000665
50.8	17.347	127.9595	48647	63	1.3E-05	0	9.78E-06
1	64.112	128.377	48845	49	0.000305	0	0.00154
69	57.32	128.4643	48883	61	2.06E-05	0	1.31E-05
26.3	100	115.4287	43262	53	0.000115	0	0.000535
60.2	10.137	141.5564	54763	96	8.67E-09	0	1.51E-11

43.6	100	141.5694	54772	105	1.1E-09	0	5.06E-12
17.1	100	141.9045	54960	100	4.85E-09	0	2.31E-10
27.7	11.569	141.8964	54955	70	4.23E-06	0	9.27E-09
47.8	23.405	140.9163	54381	57	7E-05	0	1.49E-08
33.1	30.56	140.9229	54386	40	0.003759	0	1.9E-06
0.8	100	117.5822	48275	33	0.023525	0.000141	0.001582
7.5	100	31.9094	9772	40	0.002246	0.000141	0.001696
65.5	100	55.2796	22036	41	0.002887	0.000141	0.001597
4.5	100	92.3653	37797	46	0.000567	0.000273	0.002075
95.1	13.619	51.092	18547	62	1.71E-05	0.000273	0.002099
1.5	100	111.6354	41903	44	0.001883	0.000273	0.001877
1.3	100	108.443	44556	31	0.024461	0.000273	0.002101
0.9	100	124.1247	50979	30	0.040971	0.000273	0.001987
13.6	100	141.5979	54788	19	0.17473	0.000525	0.002443
26.7	100	28.5915	9944	31	0.018855	0.000525	0.002428
11.6	100	121.7281	47544	41	0.002824	0.000525	0.002204
62.1	3.683	82.7299	33698	41	0.002396	0.000525	0.002245
16.8	100	41.3477	13002	42	0.002433	0.000758	0.002812
8.5	100	99.5427	40887	30	0.036957	0.000758	0.002787
1.6	100	117.0966	43815	35	0.012749	0.000868	0.003539
1.6	100	118.1137	48495	31	0.030501	0.000868	0.003451
3.8	100	81.657	30342	25	0.025141	0.000951	0.0044
8.6	100	119.0552	44505	27	0.096486	0.000951	0.00406
4.3	100	129.2054	53022	23	0.165532	0.000951	0.003982
112	2.451	55.7869	22249	49	0.00058	0.001022	0.005204
40.4	100	42.3222	15127	38	0.006334	0.001022	0.004732
5.1	100	109.9603	45170	35	0.00937	0.001022	0.00526
0.5	100	139.2004	56908	33	0.023878	0.001022	0.005122
0.9	100	76.8016	31257	34	0.014815	0.001022	0.005768
18.3	100	83.8945	31183	38	0.005808	0.001022	0.004666
3.5	100	30.7482	10307	37	0.004248	0.001022	0.00485
6.7	100	132.5146	54377	22	0.191662	0.00121	0.006793
13.3	91.169	78.9739	32162	45	0.001184	0.001308	0.007062
0.8	100	115.0244	47223	22	0.200207	0.001308	0.007186
35.3	100	31.7873	9732	32	0.013798	0.001308	0.006938
0.7	100	116.4781	47809	31	0.030855	0.00141	0.007249
11.7	100	83.8398	31160	28	0.055392	0.001498	0.007999
14.6	100	32.9455	12523	55	7.32E-05	0.001576	0.008416
3.4	100	119.0822	48924	31	0.036514	0.001677	0.008977
0.4	100	20.6102	6073	46	0.000651	0.002012	0.01093
0.3	100	126.7295	52010	30	0.047342	0.002257	0.01295
1	100	117.5376	48255	22	0.206056	0.002315	0.01371
1	100	121.5839	49949	25	0.11146	0.002315	0.01395
0.9	100	101.2252	38070	24	0.12284	0.002315	0.01388
0.9	100	110.9706	45620	25	0.105875	0.002376	0.01503
0.4	100	137.6404	50967	26	0.131643	0.002543	0.01615
0	100	138.1327	56504	23	0.164842	0.002543	0.01625
0.5	100	85.4072	31766	18	0.142214	0.002719	0.0164
118.1	87.219	49.7691	19650	38	0.004183	0.002719	0.01656
0.4	100	128.0746	52565	18	0.630037	0.002843	0.01872
4.7	100	31.4117	11067	31	0.017201	0.00337	0.02355
5.7	100	94.8549	35775	24	0.131215	0.00337	0.02412

0.9	100	122.0886	50166	14	1.266332	0.00337	0.02363
43.7	100	33.9362	12935	52	0.000169	0.003736	0.02724
4.1	100	54.9884	20079	12	0.464612	0.003736	0.02718
12.1	100	82.2051	33479	32	0.020824	0.003967	0.02905
7.6	100	121.7953	47568	26	0.084941	0.004504	0.03201
129.5	76.557	49.785	19658	36	0.005591	0.004504	0.03182
24.8	100	48.8783	17167	43	0.001159	0.004581	0.03237
4.5	100	111.9297	43497	26	0.105472	0.00496	0.03471
6.7	100	84.3979	31373	35	0.01062	0.005031	0.0351
16.3	100	48.9618	19303	39	0.003269	0.00573	0.04055
1.6	100	28.5894	9943	26	0.07185	0.005792	0.04237
1.6	100	78.4693	31959	34	0.016763	0.005792	0.04271
2.8	100	53.8421	19626	16	0.192602	0.005865	0.0433
3.9	100	81.7431	30376	22	0.06421	0.00861	0.06524
5.1	100	133.5662	51062	35	0.007062	0.008746	0.06685
7.8	100	83.7264	34109	23	0.163045	0.008897	0.06717
0	100	126.9134	52081	13	2.152649	0.008997	0.06812
1.5	100	129.6116	53187	10	4.534081	0.008997	0.0685
30.8	100	50.7018	18393	30	0.023079	0.008997	0.06892
2.6	100	99.5006	40872	23	0.161581	0.00971	0.07759
3.5	100	118.9167	44453	22	0.274715	0.009764	0.07862
19.6	24.805	98.5599	40469	63	2.21E-05	0	4.33E-06
41.8	100	67.6246	23227	41	0.002564	0	0.000287
18.1	100	108.9666	39389	107	5.79E-10	0	9.32E-10
54.9	100	13.0122	3494	46	0.000529	0	0.000434
42.6	100	68.5638	25037	64	1.34E-05	0	4.14E-06
62.7	6.832	68.3057	24934	47	0.000608	0	0.000439
8.5	100	68.0578	24835	37	0.006638	0	0.001253
0.8	100	129.8026	50585	42	0.001329	0	1.29E-05
0.4	100	130.3078	50795	53	0.000116	0	1.99E-07
19.6	100	139.9589	53878	63	6.77E-06	0	4.65E-10
411.9	32.013	140.5633	54172	79	4.68E-07	0	7.3E-09
12.9	100	140.583	54182	57	8.39E-05	0	1.07E-05
26.8	100	140.5362	54158	67	8.05E-06	0	5.71E-07
207.2	100	140.5975	54190	68	6.96E-06	0	6.35E-09
42.1	100	45.8016	17951	25	0.088971	0	0.000602
21	26.02	87.9683	31775	97	8.08E-09	0	7.5E-12
73.6	100	87.468	31566	40	0.00345	0	4.89E-07
41.4	100	87.5696	31607	32	0.026254	0	2.87E-09
20.3	28.24	141.4212	54685	68	5.78E-06	0	2.15E-10
31	29.364	141.4194	54683	106	1.01E-09	0	1.82E-14
36.9	100	32.6671	11260	25	0.146801	0	0.001001
49	100	32.7831	11307	30	0.040822	0	0.001462
23.6	100	30.1197	11351	40	0.005272	0	0.000609
74.1	46.77	30.1935	11385	68	7.98E-06	0	1.05E-06
45.6	100	37.4132	13360	52	0.000157	0	0.000128
0.7	100	130.8123	50998	21	0.177507	0.000525	0.00231
2.3	100	54.3828	19840	56	4.61E-05	0.000758	0.002938
7.4	100	108.466	39172	32	0.015385	0.000758	0.003053
8.6	100	82.9302	30844	45	0.000989	0.000868	0.003559
1.6	100	88.0753	31818	20	0.396336	0.001758	0.009321
5.4	100	13.107	3533	55	1.77E-05	0.001758	0.009646

2.6	100	98.6338	40499	32	0.027985	0.002079	0.01156
12.6	100	24.6791	7390	33	0.019674	0.002257	0.01243
72.3	100	42.179	15075	38	0.005624	0.002257	0.01267
23.7	100	72.7725	26737	36	0.006234	0.002257	0.01247
34.4	100	39.4843	14319	33	0.011496	0.002315	0.01389
12.6	100	24.6791	7390	32	0.027599	0.002315	0.01393
48.6	100	25.7864	9602	17	0.903278	0.002315	0.01383
69.3	100	53.3645	21230	40	0.001857	0.002843	0.01855
114.3	100	65.2414	23708	44	0.0014	0.00337	0.02211
1.6	100	107.7631	40492	23	0.233878	0.003533	0.02443
72.6	37.891	47.7108	17698	43	0.000889	0.004124	0.02943
6.3	100	30.6253	11558	19	0.630904	0.004581	0.03242
5.6	100	98.053	40253	18	0.767991	0.00496	0.0346
176	22.87	73.2734	26953	42	0.00193	0.005325	0.03709
40.7	100	16.3815	4999	25	0.077501	0.008034	0.05858
155.8	51.764	65.7498	23907	50	0.000291	0.00822	0.06094
128.9	18.681	41.6777	14889	38	0.006028	0.009514	0.07586
30.3	100	73.0147	27057	68	4.95E-06	0	1.92E-06
59.7	81.563	73.5171	27239	75	1.23E-06	0	2.25E-06
92.8	13.085	73.3398	27177	42	0.002262	0	0.000503
1.1	100	36.3224	11211	42	0.001421	0	4.27E-05
134.6	100	37.5403	12859	42	0.001735	0	0.000351
4.4	100	118.5393	48680	46	0.000991	0	0.000519
4.4	70.837	118.0375	48466	50	0.000349	0	0.000372
2.5	100	119.0402	48907	43	0.001957	0	0.001259
112.7	100	39.6605	14140	42	0.001679	0	0.000463
6.8	100	58.0378	20781	68	7.04E-06	0	0.000185
28.5	100	61.0915	22028	41	0.002895	0	7.65E-05
21.6	100	61.6002	22228	45	0.001384	0	5.4E-05
35.8	22.738	126.1005	49035	78	5.52E-07	0	2.72E-07
10.3	100	125.5919	48823	44	0.001338	0	0.000221
15.8	53.692	110.4215	41118	42	0.000835	0	0.001216
15	36.426	56.1026	20461	43	0.001031	0	1.47E-05
38.2	85.201	102.1467	39473	49	0.000377	0	3.58E-06
21.1	100	110.4431	41127	51	0.000101	0	0.000315
27.8	56.465	101.595	39226	55	9.72E-05	0	2.23E-06
2.4	100	101.0915	39024	37	0.006298	0	0.000464
100.7	4.838	68.4464	25457	49	0.000535	0	0.000752
4.1	100	68.8233	25261	75	6.23E-07	0	1.18E-07
18.8	100	94.847	34580	49	0.000444	0	3.65E-07
21.1	30.976	94.3436	34379	49	0.000436	0	4.01E-07
0.4	100	96.2838	35694	33	9.57E-05	0	0.000589
21.3	100	31.6569	11989	50	0.000322	0	0.001477
12.7	100	93.8411	34170	40	0.00338	0	1.44E-05
13	33.883	96.7876	35886	87	4.19E-10	0	9.53E-09
2.5	100	97.2913	36073	64	7.21E-08	0	5.31E-07
22.5	100	68.954	25661	49	0.000574	0.000273	0.001936
2.2	100	94.3754	34392	23	0.162069	0.000407	0.002132
31	17.298	68.8254	25606	42	0.001268	0.000525	0.002596
20.3	100	68.4531	25111	19	0.255772	0.000525	0.002335
0.6	100	117.5885	48278	30	0.039234	0.000868	0.003272
14.9	100	20.6272	7366	46	0.000825	0.001022	0.005696

4.1	100	86.6124	31207	37	0.008619	0.001114	0.005959
2.2	100	101.0501	38716	28	0.03172	0.001114	0.006412
9	100	48.6447	17472	51	0.000153	0.00121	0.006752
0	100	117.9065	44109	30	0.037085	0.001576	0.00831
7.4	100	68.4039	25439	31	0.030633	0.001576	0.008148
13.8	100	37.2801	12907	48	0.000457	0.001576	0.008574
2.6	100	56.8997	22730	36	0.006438	0.001758	0.00919
10.3	100	73.1925	27383	40	0.000893	0.001854	0.009871
7.1	100	44.1012	15266	35	0.004578	0.002257	0.01282
0	100	96.6292	35826	18	0.003228	0.002719	0.01682
1.4	100	109.9163	40902	25	0.045256	0.002777	0.01788
10.1	100	61.0382	22003	31	0.026533	0.002843	0.01872
39.7	100	34.3918	11419	36	0.002135	0.003096	0.02035
9.5	100	48.1402	17278	38	0.003538	0.003967	0.02891
77.6	100	43.6623	15099	17	0.345849	0.006497	0.04814
2.1	100	118.2824	48564	23	0.186891	0.008559	0.06485
4.5	100	90.563	33830	73	1.34E-06	0	4.87E-05
29.9	100	70.2625	25978	48	0.000803	0	0.001257
23.1	100	32.302	10874	26	0.110657	0	0.000817
11.7	100	32.8409	11078	67	9.17E-06	0	3.19E-05
79.8	18.195	32.8072	11067	37	0.00913	0	0.000309
17.2	100	45.5961	14533	65	1.23E-05	0	1.61E-05
79.9	33.312	33.8855	12025	37	0.008943	0	0.000376
21.5	100	31.2117	10990	70	1.17E-06	0	0.000551
18.5	100	67.6073	23221	49	0.000277	0	3.95E-05
44.1	100	67.1075	23021	35	0.006996	0	3.38E-05
13.7	100	97.0181	34540	41	0.002446	0	0.00089
38.5	3.916	96.5357	34355	53	0.000185	0	1.69E-05
44.6	8.148	96.5143	34346	72	1.97E-06	0	1.41E-06
12.2	100	96.0334	34139	32	0.02201	0	0.001426
28.3	100	90.0628	31783	58	5.93E-05	0	2.75E-06
48.6	100	89.5598	31582	61	3.27E-05	0	1.36E-06
6.9	100	101.5779	36330	50	0.000484	0	9.88E-05
9.1	100	105.5758	37931	48	0.000682	0	0.000197
49.3	85.848	77.1764	26653	31	0.016613	0	1.51E-05
69.3	24.064	77.1481	26641	42	0.00144	0	1.59E-06
41.1	59.196	77.651	26829	42	0.001361	0	2.41E-06
37.1	54.997	76.6736	26457	54	8.35E-05	0	4.46E-07
57.9	23.627	76.6473	26446	48	0.000376	0	9.65E-07
31.8	100	77.6806	26841	42	0.001284	0	5.13E-06
21.4	100	79.1597	27412	42	0.001438	0	3.35E-05
10.9	100	78.6867	27235	47	0.000422	0	2.32E-05
74.4	94.642	78.657	27223	45	0.000613	0	5.84E-07
21.9	100	78.1826	27036	47	0.000381	0	3.88E-05
64.8	70.103	78.153	27023	46	0.000613	0	3.3E-07
13.9	100	87.7033	32831	41	0.003565	0	1.05E-05
4.6	100	87.7835	32869	53	0.000191	0	2.67E-05
4.4	100	88.3083	33100	46	0.000909	0	0.000635
13.7	100	64.6936	23484	49	0.000433	0	1.01E-05
11.8	100	64.1882	23274	57	7.94E-05	0	6.13E-06
33.1	81.501	121.6926	47206	93	1.96E-08	0	1.05E-09
17.6	7.735	121.6724	47197	62	2.79E-05	0	6.49E-07

2.3	100	117.5386	45413	49	0.000473	0	0.000148
5.7	100	93.0161	35016	67	8.24E-06	0	8.05E-05
19.8	68.141	95.637	36109	66	1.07E-05	0	9.94E-07
163.6	85.218	139.7572	53782	64	3.19E-06	0	2.54E-06
51.9	21.399	103.4016	38261	60	2.53E-05	0	0.00041
3.6	100	101.2582	37339	87	7.63E-08	0	4.47E-07
17.4	3.592	100.2488	36913	93	2.24E-08	0	4.36E-11
149.5	63.299	140.6506	54218	90	7.71E-09	0	2.33E-07
5.6	100	100.7534	37132	43	0.001867	0	0.000169
54.3	100	141.1452	54518	29	0.020883	0	1.09E-06
8.5	100	49.5517	19563	56	6.78E-05	0	0.001063
79.6	100	42.1028	16343	69	1.38E-06	0	8.41E-05
12.3	100	70.8547	26229	43	0.002132	0.000141	0.001684
44	100	45.5297	14505	19	0.455186	0.000141	0.001687
15.5	100	74.7105	27698	45	0.001527	0.000273	0.001844
30.3	100	31.8025	11216	18	0.732424	0.000273	0.001933
12.9	100	32.7253	9997	29	0.06285	0.000273	0.001915
37.4	100	75.4029	25961	27	0.063004	0.000525	0.002459
37.4	100	75.4029	25961	27	0.063004	0.000525	0.002459
0	100	122.1768	47410	33	0.020341	0.000758	0.002831
22.6	100	33.5034	11873	26	0.106974	0.000758	0.002987
45.4	100	74.1317	25916	25	0.121375	0.000758	0.003126
29.3	100	71.2736	26392	43	0.002477	0.000758	0.002797
9.9	100	75.1406	25861	25	0.10544	0.000868	0.003334
9.9	100	75.1406	25861	25	0.10544	0.000868	0.003334
30.3	100	33.3136	11251	23	0.197688	0.000868	0.003454
1.8	100	106.5221	40058	40	0.003997	0.000951	0.00365
26.8	100	54.15	17830	24	0.075625	0.001022	0.005039
32.3	100	34.439	12234	40	0.003188	0.001022	0.005042
9.9	100	96.2524	35187	46	0.000607	0.001114	0.006311
0.5	100	119.1593	46081	26	0.100653	0.001114	0.006192
216.7	8.493	49.6213	17455	31	0.029515	0.00121	0.006459
20.5	100	97.0441	34551	25	0.122167	0.00121	0.006592
36.9	100	33.9357	12046	35	0.009787	0.00141	0.007402
58.2	94.195	49.7033	17487	42	0.002504	0.00141	0.00729
69.1	100	50.1252	17655	27	0.077606	0.001576	0.008583
1.6	100	93.5201	35221	37	0.007263	0.001758	0.009448
5	100	32.5476	9989	42	0.001773	0.001758	0.00931
21.4	100	33.3813	11828	17	0.823906	0.001854	0.009792
5.7	100	16.6159	4732	51	5.44E-05	0.002012	0.01037
0.9	100	133.5214	49261	18	0.669518	0.002012	0.01107
9.2	100	89.0561	31376	26	0.092211	0.002079	0.01196
10.5	100	71.7769	26592	36	0.010464	0.002079	0.01164
135.6	100	31.3774	10530	33	0.013277	0.002376	0.01431
5.2	100	67.6607	23241	54	1.5E-05	0.002543	0.01616
97.7	2.021	70.768	26197	43	0.002358	0.002543	0.01573
7	100	79.1954	27428	22	0.11088	0.002777	0.01801
5.6	100	96.2863	35203	24	0.146913	0.002931	0.01953
125.5	100	13.189	3704	12	2.930764	0.003736	0.02669
6.5	100	82.6464	30728	21	0.31342	0.003892	0.02805
8.4	100	71.5072	24814	31	0.031463	0.004424	0.03175
20.3	100	30.3578	10151	35	0.010704	0.004581	0.03257

36.8	100	50.0574	19778	35	0.009448	0.004803	0.03402
14.1	100	33.82	11439	16	1.116823	0.005478	0.03782
15	100	50.6853	17875	24	0.135376	0.005687	0.03941
0.5	100	106.5745	40077	28	0.064344	0.00573	0.04159
0.7	100	140.1637	53976	20	0.076904	0.00573	0.04032
26.6	100	49.1173	17258	27	0.070662	0.006222	0.04591
9.8	100	16.6589	4625	39	0.000934	0.006371	0.04634
23.8	100	88.2031	33050	9	5.820561	0.006428	0.04707
2.3	100	88.3742	36145	14	1.534022	0.007636	0.05465
40.3	100	33.4842	11314	9	3.529884	0.007836	0.05629
4.3	100	122.4234	47521	29	0.055226	0.00822	0.06192
38.5	100	16.8105	5199	49	0.000129	0.008361	0.0628
0	100	100.3284	36950	22	0.256443	0.0085	0.064
4.2	100	99.0673	35332	11	2.423208	0.009858	0.08112
19.9	100	34.7069	11895	43	0.000426	0.009858	0.08143
17.5	100	45.6375	15868	20	0.487905	0	0.00091
0	100	140.241	51859	58	2.48E-05	0	1.43E-06
10.1	100	49.0328	17759	44	0.000912	0	0.000984
28	100	48.5305	17557	38	0.003329	0	5.84E-05
32.5	63.525	140.6631	55307	99	3.82E-09	0	1.3E-10
16.2	100	97.4599	34712	46	0.000875	0	0.000254
5.9	100	97.4897	34724	52	0.000262	0	5.78E-05
36.6	26.214	74.1135	25457	42	0.003265	0	7.81E-06
16.3	100	73.9278	25376	52	0.000323	0	7.39E-05
0.4	100	118.1667	43210	52	0.000127	0	8.32E-06
1	100	125.4044	48744	69	2.68E-06	0	1.51E-07
29.7	100	140.0578	54992	37	0.004637	0	9.59E-07
0	100	136.3265	53331	40	0.00241	0	0.000595
0.9	100	136.1481	53259	72	1.43E-06	0	3.97E-09
215.5	76.48	140.7176	52396	103	1.56E-09	0	8.51E-10
12.8	100	140.7422	52408	59	4.21E-05	0	5.68E-06
166.2	100	16.5753	5368	22	0.25876	0	0.001547
2.1	100	119.4149	44988	51	0.000289	0	0.0001
1.5	100	118.9144	44785	44	0.00141	0	0.00049
154.1	100	50.2359	17655	57	4.1E-05	0	0.000508
21.4	45.854	44.9666	17567	44	0.000983	0	9.78E-05
28.2	100	53.2933	21202	49	0.000417	0	0.000847
43.1	43.738	140.7661	54287	70	5.39E-06	0	1.91E-09
7.8	100	72.2645	29348	46	0.00081	0	1.18E-05
14.7	100	91.6309	33258	39	0.00356	0	0.000351
34.5	94.541	91.1255	33057	53	0.000146	0	7.45E-05
1.3	100	71.7606	29127	33	0.017375	0.000273	0.00208
28.3	100	37.5809	11685	43	0.000262	0.000951	0.004547
61.9	100	45.1096	17630	33	0.018374	0.00121	0.006766
2	100	96.9884	34528	30	0.042379	0.001945	0.01005
10.6	100	114.9996	43070	32	0.019853	0.002079	0.01151
266.7	100	35.1811	13460	38	0.004559	0.002843	0.01909
0	100	125.9127	48958	23	0.103812	0.00337	0.02207
10.5	100	65.4208	23844	50	0.00015	0.00337	0.02309
67.3	100	30.6232	11557	30	0.022951	0.003967	0.02882
12	100	86.8666	31319	15	0.918082	0.004643	0.03336
117.5	100	17.6235	5927	37	0.005945	0.004643	0.03371

8.9	100	52.7871	20976	30	0.027747	0.00573	0.04033
13.4	100	90.6188	32859	16	0.706669	0.009858	0.08111
28.1	100	53.0831	17428	29	0.049158	0	0.000525
6.7	100	30.1279	9131	66	2.34E-06	0	0.001264
0.3	100	130.9689	48635	78	1.82E-07	0	2.09E-05
0.3	100	130.4653	48471	85	3.45E-08	0	5.02E-06
0	54.598	83.593	34056	83	2.03E-07	0	5.68E-08
5.8	27.924	83.534	34031	56	8.33E-05	0	1.44E-06
8	100	83.0836	33839	63	1.7E-05	0	8.55E-07
77.1	8.074	39.0728	13928	49	0.000504	0	0.000392
8.7	9.349	54.8123	20004	97	8.08E-09	0	8.26E-08
19.8	13.015	54.7398	19974	72	2.5E-06	0	2.39E-07
43.8	100	58.0238	21264	54	0.000147	0	0.000974
64.8	12.533	57.5188	21070	51	0.000345	0	0.000994
68.3	100	31.4365	11076	49	0.000497	0	0.000611
50.7	54.549	54.3127	19812	74	1.83E-06	0	9.85E-08
3.4	100	25.8138	7756	63	1.41E-05	0	0.000199
41.8	10.312	132.7414	51820	74	8.64E-07	0	8.18E-07
5.4	27.271	132.781	51838	69	3.14E-06	0	9.09E-06
15.1	84.995	140.6402	52350	107	8.9E-10	0	4.23E-11
24.5	71.928	53.9821	19190	41	0.00267	0	1.14E-05
2.2	100	76.9075	28943	75	7.71E-08	0	6.37E-05
69.3	34.275	77.8341	29041	56	7.67E-05	0	0.000355
0	100	106.7068	40830	47	0.000433	0	0.000748
2	100	110.5917	42265	49	0.0004	0	0.00029
44.1	9.582	111.0722	42442	89	3.74E-08	0	2.26E-09
13.6	6.111	111.0921	42450	73	1.46E-06	0	1.8E-07
1.8	100	111.5951	42633	62	1.88E-05	0	1E-05
8.9	100	50.4238	19937	56	7.26E-05	0	8.84E-07
19.2	100	50.9243	20155	32	0.018002	0	5.36E-05
32.6	100	64.8149	24248	53	0.000155	0	0.001234
67	79.088	43.8956	17117	47	0.000731	0	5.49E-06
63.7	73.945	35.1921	12250	44	0.000812	0	0.000452
24.7	100	35.6951	12452	55	7.18E-05	0	0.00021
8.7	100	39.4004	13879	56	5E-05	0	0.000512
2.7	100	96.6126	35820	43	0.001961	0	0.001052
3.3	100	96.1237	35638	47	0.000825	0	0.000139
8.5	100	96.6267	35825	65	1.14E-05	0	3.02E-06
42.5	100	64.31	24072	50	0.000306	0.000141	0.001756
0	100	13.2094	3566	53	2.62E-05	0.000141	0.001737
125.3	72.376	40.2159	14195	24	0.202526	0.000141	0.00169
12.3	100	16.9628	4856	40	0.004253	0.000273	0.001818
1.9	100	111.5758	42626	41	0.002758	0.000273	0.002069
124.4	100	39.2079	13803	25	0.163893	0.000525	0.002215
15.3	100	60.8565	21975	30	0.005654	0.000525	0.002637
23.3	100	25.4668	8775	30	0.03413	0.000525	0.002504
25.1	100	53.7872	19117	34	0.013819	0.000525	0.002491
28.5	100	38.7493	13803	41	0.00355	0.000525	0.002592
8.4	100	17.5837	5085	50	0.000375	0.000525	0.002638
98.4	100	44.4489	15403	49	0.000249	0.00065	0.002736
11.7	100	57.0989	20910	39	0.005276	0.00065	0.002718
3.7	100	21.6662	6405	53	0.000155	0.000951	0.00458

18.9	100	17.5107	4926	48	0.00051	0.000951	0.004217
161.1	100	38.7002	13597	21	0.348786	0.001022	0.00581
16.7	100	54.1872	19269	48	0.000356	0.001022	0.005603
108.1	91.969	57.6044	21103	28	0.065438	0.001022	0.004919
101.4	50.419	32.4315	11167	49	0.000357	0.001022	0.005577
29.2	97.98	60.6015	21868	35	0.010051	0.001022	0.005021
7.5	100	30.2563	10281	46	0.000206	0.001022	0.005671
52.8	86.234	60.256	21722	51	0.000251	0.001022	0.005617
9.2	100	25.1449	7539	34	0.013117	0.001022	0.004652
137.9	4.735	43.9479	15207	48	0.000256	0.001114	0.006133
0	100	46.8365	15013	42	0.002379	0.001576	0.008227
180.2	100	38.624	13567	48	0.000457	0.001576	0.008791
81.6	100	44.9437	16149	44	0.000784	0.001576	0.008072
0.3	100	106.3251	40688	33	0.012256	0.001758	0.009307
151.7	58.259	63.8071	23890	46	0.000761	0.002012	0.01037
73.9	100	39.7137	14004	20	0.497097	0.002079	0.01175
37.9	100	55.1113	19632	31	0.0208	0.002257	0.01295
33.8	100	34.7527	12078	18	0.333092	0.002543	0.01604
2.1	100	96.1083	35632	24	0.164427	0.002719	0.01645
58.4	100	21.8401	7889	45	0.000879	0.00337	0.02233
42.3	100	17.6087	5588	36	0.008327	0.003674	0.02655
44.6	100	58.4021	20942	34	0.01045	0.003736	0.02715
30.4	100	17.5283	5252	36	0.008038	0.003892	0.02833
18.9	100	58.8345	21132	49	0.00032	0.004192	0.03022
0.4	100	109.6753	42579	16	0.761281	0.004504	0.03212
28.5	100	32.935	11369	30	0.027077	0.004504	0.03213
3.1	100	53.0314	17411	21	0.290175	0.0085	0.06389
150.2	50.766	37.8926	11769	24	0.041042	0	0.001212
71.2	100	37.3896	11593	27	0.02363	0	0.000747
65.5	100	37.8791	11764	32	0.006966	0	2.74E-05
38.8	59.676	76.4079	26887	38	0.007269	0	0.00106
16.8	100	87.9803	35970	54	9.8E-05	0	6.72E-07
1.7	100	105.9087	43500	58	2.44E-05	0	2.15E-07
19.7	100	43.6121	13816	28	0.016159	0	0.00031
15.8	100	48.5201	15629	54	4.51E-05	0	0.000375
167.3	100	48.4768	15611	73	7.07E-07	0	5.85E-06
86.9	100	47.9752	15425	40	0.001598	0	0.000186
183.1	91.04	48.9801	15792	69	1.97E-06	0	8.6E-06
14	100	109.8862	39774	61	2.96E-05	0	4.72E-05
10.8	100	109.8748	39770	55	0.000116	0	0.00017
73.4	100	46.9808	16403	45	0.000483	0	0.000191
3.5	100	128.5395	50069	39	0.003407	0	0.000171
91.8	86.161	140.574	55257	82	2.74E-07	0	6.48E-10
86.3	41.806	140.5696	55254	74	1.48E-06	0	5.22E-09
10.5	100	94.0658	35436	45	0.001414	0	2.42E-06
20.3	30.529	116.3144	45351	96	3.43E-09	0	1.11E-09
2.3	100	116.0736	45246	35	0.004309	0	0.000565
103.8	17.288	51.1305	18462	34	0.014194	0	0.000244
18.8	100	51.2954	18530	55	9.22E-05	0	2.74E-05
26.8	100	63.0716	22887	63	2.01E-05	0	9.18E-05
112.5	48.28	46.9527	16345	40	0.000959	0	0.001019
13.7	100	52.5645	18627	54	8.75E-05	0	5.24E-05

30.3	100	52.5289	18612	29	0.033541	0	0.000446
31.1	100	86.9187	31341	68	6.12E-06	0	1.93E-09
13.1	100	86.8551	31313	35	0.013449	0	6.47E-05
38.3	100	87.3682	31526	54	0.000151	0	2.66E-07
33.8	100	77.759	27480	71	3.17E-06	0	3.16E-06
56.7	71.81	141.0054	54434	76	1.02E-06	0	3.32E-09
183.2	86.789	43.2817	13693	21	0.08373	0.000141	0.001608
26.9	100	81.3032	30520	43	0.002212	0.000525	0.00232
8.7	100	109.3817	39558	36	0.009447	0.000758	0.002872
13.2	100	49.6681	17882	31	0.007366	0.000868	0.003492
162.4	100	32.2583	11396	30	0.07813	0.000951	0.003745
26.6	100	43.9852	15222	43	0.000984	0.000951	0.004426
8	100	75.907	26680	29	0.055651	0.001022	0.004601
17.4	100	43.0972	13626	25	0.031065	0.001022	0.004938
31	100	21.8865	6875	38	0.004748	0.001022	0.00579
16	100	77.7511	27476	35	0.014404	0.001022	0.004912
9.5	100	53.2752	19333	47	0.000515	0.001114	0.005924
50.8	100	48.4131	16922	22	0.182489	0.00121	0.00662
7.3	100	82.6327	28782	18	0.698233	0.001308	0.007187
104.5	100	32.7616	11597	28	0.129943	0.001498	0.007809
38.6	100	50.6222	18258	22	0.19783	0.001576	0.008059
17.9	100	31.75	9666	28	0.119872	0.001576	0.008429
30.7	100	42.7791	13512	16	0.303416	0.001576	0.008668
12.5	100	42.3894	16470	33	0.013705	0.001758	0.009549
8.5	100	48.0141	15439	32	0.009127	0.002079	0.01209
50.4	100	46.4785	16200	27	0.02274	0.002777	0.01803
40	100	31.7542	11198	25	0.286092	0.002777	0.01815
4.8	100	81.3554	30542	26	0.097738	0.002931	0.01938
12.4	100	37.0156	11499	26	0.051159	0.003254	0.02126
0	100	123.4165	47929	17	0.639522	0.003674	0.0257
2.3	100	123.923	48138	22	0.194141	0.003674	0.02656
1.1	100	57.6142	19072	38	0.005836	0.004643	0.03283
83.3	100	47.6443	16662	25	0.044876	0.005271	0.0361
9.8	100	32.3293	11423	36	0.017976	0.005325	0.03748
2	100	126.223	47025	20	0.325334	0.005687	0.03979
13	100	49.1652	17687	16	0.231209	0.00573	0.04044
15.2	100	76.0094	26723	30	0.04013	0.008962	0.06745
0	100	115.8094	45127	6	3.498387	0.009514	0.07286
2.5	100	109.3713	39553	20	0.393724	0.009858	0.08159
3.5	100	61.7831	22655	45	0.00095	0	0.000394
6.6	100	61.5832	22576	40	0.002509	0	0.001199
50.7	100	28.9113	9613	39	0.003551	0	1.65E-06
3.1	100	131.704	54023	66	1.01E-05	0	5.66E-08
32.8	100	59.5267	19784	43	0.001722	0	0.000808
3.7	100	99.3859	37738	56	0.000105	0	7.99E-06
79.9	100	33.7985	11536	44	0.001876	0	0.000546
15.5	100	94.8851	34596	43	0.002401	0	0.000717
4.9	100	105.6579	39050	79	1.6E-07	0	8.87E-08
0.4	100	105.1557	38873	40	0.001175	0	0.000491
18.9	100	62.8725	23176	40	0.001238	0.000141	0.001731
12.6	100	39.9054	12473	32	0.009292	0.000141	0.001626
10.5	100	91.4521	34166	39	0.002697	0.000273	0.001996

82	100	54.9637	19575	16	0.69225	0.00065	0.002668
42.3	100	57.7892	19279	33	0.016955	0.000758	0.002875
46.9	100	52.4372	18999	33	0.007249	0.001022	0.00553
5.2	100	114.1353	44456	32	0.009952	0.001022	0.004784
23.2	100	15.7782	4705	54	6.17E-05	0.001114	0.005954
81.1	100	64.8037	22051	35	0.001036	0.001308	0.00701
36.1	100	58.4933	20962	18	0.684674	0.001576	0.008834
7	100	63.4079	21263	29	0.053891	0.001758	0.00956
13.9	100	70.8618	26430	26	0.046588	0.002079	0.01233
41.2	100	32.6419	11549	16	1.023294	0.003096	0.01969
7.4	100	99.9007	37961	13	1.708026	0.003674	0.02548
81.9	100	46.6119	16220	43	0.001376	0.004192	0.03047
16.4	100	76.8071	28368	24	0.141478	0.004344	0.03115
1.3	100	88.8869	33165	54	6.86E-05	0	9.5E-05
1.9	100	92.3739	34497	64	6.72E-06	0	5.36E-07
2.6	100	92.2857	34463	57	2.96E-05	0	1.76E-05
0.5	100	84.3499	31356	58	1.28E-05	0	0.000368
0	100	95.0387	35569	35	0.010478	0	0.000495
1.4	100	94.5326	35365	41	0.002959	0	0.000663
48.5	100	33.8621	11454	28	0.049056	0	0.000961
13.1	100	35.6455	12124	68	5.95E-06	0	0.001128
53.4	100	36.6682	12518	61	2.8E-05	0	3.55E-06
81.9	8.255	36.5065	12452	46	0.000984	0	0.000173
42	100	36.165	12322	60	3.76E-05	0	1.01E-05
4.1	100	36.1544	12317	69	4.45E-06	0	0.000226
88.9	25.495	36.0035	12255	55	0.000108	0	3.1E-05
1.9	100	87.9846	35972	39	0.001987	0	0.001147
0	64.743	90.8844	37166	95	2.95E-09	0	3.6E-06
3.4	100	99.3741	40815	62	1.06E-05	0	2E-06
0	100	92.9538	38058	59	2.07E-05	0	1.66E-05
38.9	100	92.9517	38057	78	2.69E-07	0	3.04E-09
2.2	51.205	92.4423	37829	50	0.000153	0	6.81E-06
14.5	79.24	91.9455	37617	80	1.58E-07	0	6.05E-09
2.1	42.267	91.9373	37613	65	5.35E-06	0	3.59E-06
15	25.465	91.4396	37411	92	9.94E-09	0	6.41E-10
1.8	13.185	91.4314	37407	72	1.03E-06	0	1.95E-07
0	100	91.3884	37388	82	5.05E-08	0	1.78E-06
39.7	1.785	90.9359	37188	95	4.28E-09	0	4.17E-10
9.6	1.411	90.928	37184	70	1.37E-06	0	4.37E-08
13.6	100	92.4463	37831	59	2.15E-05	0	3.54E-07
4.9	100	97.7019	40092	49	0.000179	0	0.000265
8.1	100	95.9959	39368	62	1.09E-05	0	1.95E-06
6.1	100	94.9829	38931	53	6.95E-05	0	7.15E-05
35.4	100	56.3437	20619	44	0.001645	0	0.000237
25	8.353	55.8491	20422	64	1.62E-05	0	5.35E-06
57.2	4.191	55.8417	20419	49	0.000533	0	0.000738
57	1.765	55.3465	20223	60	4.31E-05	0	3.31E-05
78.6	0.965	55.3395	20220	52	0.000247	0	0.001452
29.3	100	52.9734	19271	44	0.00148	0	0.000566
14.1	100	52.9424	19259	60	3.81E-05	0	6.73E-06
0	100	139.4321	57001	41	0.001155	0	0.000523
2.1	100	129.4199	47680	71	3.48E-06	0	2.22E-08

0.9	100	129.598	47750	40	0.003917	0	0.000643
2.9	100	129.6079	47754	27	0.085104	0	2.4E-05
0	100	129.2899	47625	81	2.56E-07	0	3.07E-06
0	100	128.3867	47248	75	1.39E-06	0	9.36E-09
0	100	128.7783	47408	86	7.23E-08	0	1.28E-07
1.7	100	128.921	47469	70	4.62E-06	0	5.76E-08
0	100	130.5387	48132	45	0.001619	0	0.000118
1.1	100	129.7923	47832	48	0.000529	0	0.000997
0	100	127.8796	47059	68	6.83E-06	0	2.79E-07
1.2	100	125.5172	46198	76	1.08E-06	0	5.94E-09
1.4	100	125.4938	46188	48	0.000701	0	2.8E-05
2.2	100	124.9879	45971	53	0.000225	0	2.31E-06
3.7	100	125.0137	45982	73	2.05E-06	0	9.19E-11
0	100	125.0724	46007	54	0.000136	0	0.000421
1.1	100	126.0204	46384	56	0.000104	0	6.55E-07
0.8	100	126.5291	46568	74	1.65E-06	0	2.28E-08
1.2	100	127.035	46751	63	2.45E-05	0	2.8E-07
18.7	55.837	132.4413	48832	56	7.93E-05	0	3.84E-05
10.1	67.712	136.9996	50651	62	2.21E-05	0	3.5E-07
40.2	12.731	137.4022	50830	107	7.79E-10	0	4.58E-10
19.5	100	137.5134	50881	83	1.97E-07	0	6.05E-10
36.1	2.448	137.2696	50770	102	2.3E-09	0	1.28E-10
34.1	21.78	136.8914	50607	107	7.74E-10	0	1.05E-09
24.1	100	136.439	50420	63	1.85E-05	0	5.11E-08
28.5	5.849	136.76	50556	107	7.57E-10	0	3.61E-11
35	2.687	137.7727	51003	100	3.81E-09	0	3.89E-11
26.7	100	137.7771	51005	53	0.000114	0	1.41E-06
35.1	18.541	137.9134	51071	107	7.56E-10	0	4.81E-10
9.5	100	138.0164	51121	71	3.08E-06	0	1.75E-06
1.3	100	134.1009	49482	38	0.006344	0	0.000535
17.4	100	132.5171	48863	54	0.000108	0	0.000737
1.8	100	132.5197	48864	46	0.0012	0	0.000355
1	100	133.0356	49069	37	0.009257	0	0.000539
51.4	100	38.5322	12013	51	0.000266	0	5.29E-05
68.1	100	38.0285	11832	27	0.067679	0	0.000538
0	100	124.5718	45806	75	1.09E-06	0	8.85E-08
5.9	100	124.5089	45780	78	6.36E-07	0	5.8E-12
0	100	124.4851	45770	40	0.004856	0	0.001444
1.8	100	115.0585	41898	75	1.38E-06	0	5.83E-09
8.8	100	90.5384	31973	62	2.56E-05	0	9.16E-08
3.9	100	90.4936	31953	43	0.00214	0	0.000111
2.5	100	89.9886	31752	26	0.120633	0	0.000627
104.1	1.48	91.4343	32344	54	5.74E-05	0	6.49E-05
45.4	71.097	91.9663	32560	40	0.001756	0	0.000304
29.2	23.627	91.937	32549	46	0.000453	0	0.000141
37.8	3.998	91.4651	32357	51	0.00012	0	9.32E-05
1	100	122.4787	44956	70	4.92E-06	0	8.55E-08
1.4	100	123.9797	45571	36	0.010613	0	0.001077
9.3	87.997	124.0072	45583	80	4.52E-07	0	4.4E-13
2.6	100	124.2118	45660	37	0.005901	0	0.000729
11.3	7.061	122.9745	45164	82	2.54E-07	0	8.17E-12
19.6	2.473	122.991	45171	81	3.61E-07	0	6.31E-13

7.1	95.108	123.0668	45205	102	2.54E-09	0	7.31E-11
3.6	24.77	123.4787	45383	81	3.17E-07	0	2.75E-11
11.4	7.378	123.4972	45391	88	7.24E-08	0	7.45E-13
30.9	3.076	87.3637	32679	44	0.001947	0	6.61E-06
9.5	66.784	87.3514	32673	77	8.86E-07	0	5.34E-07
5.5	100	87.319	32657	46	0.000916	0	2.79E-05
11.7	75.297	87.2383	32620	57	7.48E-05	0	3.09E-06
23.1	2.58	87.2134	32608	66	1.22E-05	0	1.49E-08
4.3	23.064	87.2076	32605	78	5.37E-07	0	6.18E-07
11.1	89.419	86.8579	32458	19	0.556998	0	0.000471
22.5	2.646	87.7214	32840	64	1.61E-05	0	2.21E-09
10.3	71.193	87.7426	32851	60	3.51E-05	0	1.16E-05
3.9	100	88.8887	33348	47	0.000967	0	1.4E-06
6.3	27.561	88.7404	33283	71	3.75E-06	0	7.24E-09
18	8.183	88.3802	33133	46	0.00114	0	2.96E-06
2.1	100	88.3663	33127	48	0.000621	0	0.000109
14.9	5.293	88.2321	33064	73	1.94E-06	0	1.12E-08
33.8	6.012	140.8049	52442	96	5.43E-09	0	7.62E-06
19.4	3.706	87.8743	32908	39	0.005508	0	1.12E-05
7.9	73.75	87.8603	32902	65	1.43E-05	0	3.77E-06
20.7	7.215	83.4633	31060	51	0.000292	0	0.000137
121	0.306	83.2795	30992	62	2.43E-05	0	0.000466
1.1	38.189	83.1962	30956	56	4.8E-05	0	0.001038
0	14.21	82.688	30745	62	1.32E-05	0	0.000962
129.5	1.185	83.7918	31189	63	1.96E-05	0	0.000228
51.6	100	84.797	31600	66	8.43E-06	0	3.73E-05
48.8	91.459	84.2945	31390	54	0.000148	0	0.001263
4.5	100	83.9684	31268	64	1.33E-05	0	7.36E-05
45.3	43.562	54.1088	19230	49	0.000526	0	0.000121
11.3	100	54.1932	19262	65	1.12E-05	0	4.53E-05
41.7	100	56.6918	20239	48	0.000487	0	1.29E-05
3.8	100	68.192	24887	44	0.00173	0	0.001076
1.9	100	139.5941	51843	28	0.040241	0	0.001219
28.6	100	139.62	51854	76	5.62E-07	0	2.09E-13
8.8	100	138.9071	51526	71	3.36E-06	0	8.9E-10
3.3	100	129.2326	50345	70	4.16E-06	0	4.44E-08
0.3	100	128.7295	50147	44	0.001632	0	0.000185
0	100	128.67	50123	77	6.65E-07	0	2.43E-06
29.8	4.623	139.0679	51603	69	3.14E-06	0	1.01E-11
0	17.203	128.1658	49902	98	5E-09	0	4.25E-10
14.9	9.338	139.0839	51610	60	2.14E-05	0	2.36E-10
0	100	139.8536	51958	54	0.000128	0	0.001237
0.6	100	138.9525	54483	49	0.000322	0	1.34E-05
6.5	100	137.9658	54034	86	9.56E-08	0	3.22E-08
7.8	100	137.6225	53875	102	2.38E-09	0	6.71E-10
22.6	100	137.4198	53783	73	1.81E-06	0	6.96E-09
46.1	4.191	138.2775	51242	78	6.14E-07	0	7.51E-10
10.3	18.621	138.4238	51303	95	1.29E-08	0	4.98E-10
17.4	44.165	137.4617	53802	98	5.51E-09	0	1.33E-10
78.1	100	140.2122	52125	92	2.58E-08	0	1.12E-09
5.3	100	140.2693	52152	57	6.99E-05	0	1.41E-05
1.8	100	114.747	44233	85	1.33E-07	0	4.04E-09

14.8	100	139.5134	53666	48	0.000588	0	1.46E-05
5.3	100	112.0662	41808	53	0.000212	0	1.08E-06
14.8	100	139.5134	53666	48	0.000588	0	3.17E-06
2.1	100	112.1161	41829	74	1.93E-06	0	1.1E-08
28.2	71.567	100.1087	36855	10	2.356131	0	0.00024
40.9	7.668	140.6512	54219	72	1.31E-06	0	3.74E-08
3064.2	100	140.6368	54211	90	2.32E-08	0	5.23E-09
1.7	100	119.6403	45090	39	0.005417	0	0.001485
26	100	131.7925	50319	54	0.000131	0	0.00147
1.5	100	134.1633	51330	82	2.07E-07	0	2.95E-08
12.7	56.902	129.2832	49250	46	0.000754	0	0.000305
17	100	136.8736	52469	61	2.74E-05	0	0.001452
26.7	100	134.8486	51622	54	0.000137	0	0.000974
2.5	100	134.4676	51461	45	0.000838	0	1.02E-05
13.4	100	134.3905	51428	78	3.6E-07	0	1.37E-11
16.1	100	135.2614	51794	41	0.002613	0	0.000623
33.2	100	86.7003	31245	38	0.006804	0	2.3E-05
31.6	38.673	86.1941	31022	42	0.002696	0	4.75E-05
72	100	87.4495	31559	26	0.045169	0	0.001262
32.6	84.488	43.4298	16921	66	8.08E-06	0	7.54E-06
51.6	100	87.8287	31716	36	0.011336	0	9.6E-06
131.5	2.535	42.92	16702	50	0.000294	0	0.0003
5.6	100	82.9719	29699	46	0.001106	0	0.000231
5.6	100	82.9719	29699	46	0.001106	0	0.000231
19.5	55.334	52.7321	20952	66	8.88E-06	0	0.001363
2.6	100	140.9388	50416	71	3.36E-06	0	1.79E-07
325.4	1.251	140.7361	54267	79	3.75E-07	0	4.24E-06
45.2	3.877	140.7367	54268	55	0.000109	0	8.3E-05
267.8	24.275	140.7866	54300	82	1.87E-07	0	4.13E-07
87.8	100	89.1157	36445	24	0.068531	0.000141	0.001708
0	100	136.9592	53596	37	0.006863	0.000141	0.001644
4.4	100	140.1207	57287	31	0.011561	0.000141	0.001773
29.7	92.928	56.9635	20348	40	0.002771	0.000141	0.001727
8.7	100	83.7021	31146	64	8.13E-06	0.000141	0.00177
0	100	95.489	39141	37	0.003142	0.000141	0.001579
37.2	49.219	67.2274	24513	47	0.000814	0.000273	0.001976
0.3	100	94.4762	38713	38	0.002607	0.000273	0.001961
13.6	100	43.5628	16974	40	0.003215	0.000273	0.002003
6.6	100	138.7604	51457	28	0.072432	0.000273	0.001879
14.9	100	62.5895	25183	43	0.000317	0.000273	0.00201
78.4	22.558	135.8614	52052	61	2.99E-05	0.000273	0.001863
13.9	96.698	43.7089	15023	60	1.38E-05	0.000525	0.002492
23.8	100	37.523	11666	30	0.032276	0.000525	0.002335
20.9	100	140.5321	52556	28	0.049048	0.000525	0.002441
72	39.564	68.0553	24937	64	9.74E-06	0.000525	0.002389
19.8	100	52.4712	19080	36	0.00873	0.000525	0.002228
48.5	100	136.3679	52263	55	0.000113	0.00065	0.0027
0.4	100	83.7015	29979	37	0.007952	0.000758	0.0028
34.5	100	57.7462	19125	21	0.13351	0.000758	0.003173
4.7	100	136.9105	52486	25	0.125749	0.000758	0.002821
21.2	100	35.6316	12680	38	0.006846	0.000758	0.003227
1.6	100	127.0925	48307	29	0.046566	0.000758	0.002927

3.6	100	135.1136	49874	38	0.005265	0.000758	0.002839
14.5	100	41.192	15945	39	0.000444	0.000758	0.002821
47.6	3.04	101.8996	37620	30	0.031465	0.000758	0.002875
4.5	100	67.6845	24687	36	0.012116	0.000868	0.003521
11.9	100	63.5983	25606	37	0.00132	0.000868	0.003389
41.3	73.493	67.1775	24493	44	0.001803	0.000868	0.003492
9.2	100	65.1565	23675	31	0.039232	0.000868	0.003502
11.7	100	93.4573	38290	29	0.019914	0.000951	0.004168
0	100	93.9677	38507	31	0.012167	0.000951	0.004462
16	100	85.405	31846	42	0.002073	0.000951	0.004262
13.8	100	140.5092	50258	22	0.185115	0.000951	0.004472
15.1	100	54.1409	21548	35	0.011052	0.000951	0.004042
67.6	100	37.0119	12654	23	0.189246	0.000951	0.003983
2.5	100	135.767	52015	32	0.020259	0.000951	0.004405
23.8	100	137.4261	52717	54	0.000141	0.000951	0.003613
6.1	100	54.9924	18057	37	0.007532	0.001022	0.00552
85.2	39.718	35.5653	12092	37	0.007242	0.001022	0.004758
21.3	100	66.7662	22568	35	0.016659	0.001022	0.005471
22.7	100	67.1566	22717	38	0.006637	0.001022	0.005418
16.2	100	67.8019	22968	33	0.021232	0.001022	0.004897
34.7	100	84.5143	29531	39	0.004606	0.001022	0.004662
8.6	100	122.471	44953	25	0.137974	0.001022	0.005126
21.2	100	60.7382	20228	20	0.173318	0.001022	0.004905
55.3	40.931	62.9839	23125	40	0.000584	0.001114	0.006258
2.5	100	138.5257	51352	26	0.097649	0.001114	0.006118
6.6	100	136.7764	52426	29	0.037161	0.00121	0.006513
120.5	69.489	42.5541	16537	39	0.003756	0.001308	0.007005
50.3	3.921	66.7227	24312	45	0.001469	0.001308	0.006994
4.1	100	87.8372	32891	27	0.07483	0.00141	0.007288
0.4	100	139.9856	57224	30	0.017195	0.001498	0.007937
4.6	100	53.117	17440	36	0.009086	0.001498	0.008009
6.3	100	128.0288	49837	17	0.670611	0.001498	0.007902
15.8	100	65.207	23695	37	0.009046	0.001498	0.007907
17.9	100	61.5746	24741	38	0.001012	0.001498	0.007759
12.9	100	52.6928	17289	24	0.064478	0.001498	0.007755
35.7	18.102	82.6576	28793	41	0.000532	0.001576	0.008538
4.4	100	85.4011	31844	52	0.000149	0.001576	0.008754
40.3	100	33.3589	11268	20	0.354835	0.001576	0.008665
7.7	20.954	62.1237	24986	44	0.000264	0.001576	0.008479
2.3	100	134.1107	49486	29	0.035797	0.001576	0.008828
45.3	100	67.269	22760	36	0.012682	0.001576	0.008095
72.9	3.286	65.7098	23890	46	0.001183	0.001758	0.009198
99.8	100	36.068	12279	34	0.013179	0.001854	0.009917
3.9	100	85.9208	32062	51	0.000152	0.001854	0.009761
130.9	2.917	62.1471	24998	41	0.000439	0.001854	0.009914
17.1	100	67.9272	24783	37	0.009096	0.001854	0.009866
32.9	100	45.1457	15674	20	0.272583	0.001945	0.01022
10.6	100	88.2894	33091	34	0.012558	0.001945	0.01021
11.9	100	43.9343	17134	48	0.00044	0.002012	0.01031
34.8	100	31.6228	11976	32	0.016708	0.002012	0.0103
5.3	100	94.6691	35691	51	0.000164	0.002012	0.0111
62	100	45.4601	15799	17	0.552461	0.002012	0.011

46.8	100	54.7858	18082	22	0.10581	0.002079	0.01146
36.8	100	53.0487	17417	33	0.017849	0.002079	0.01189
60.8	100	35.5009	12069	18	0.576036	0.002079	0.01205
26.2	100	33.7077	11399	25	0.124144	0.002079	0.01142
22.6	25.528	25.5516	9503	46	0.000677	0.002257	0.01254
18	100	56.8471	20815	32	0.02136	0.002257	0.01266
23.8	100	43.0258	15391	31	0.021678	0.002315	0.01296
0.4	100	131.8417	50338	43	0.002306	0.002315	0.0132
21.2	54.352	102.4054	37839	23	0.124651	0.002315	0.01381
84.4	4.078	66.6757	24291	44	0.001768	0.002315	0.01347
0	100	124.0644	45606	34	0.014186	0.002315	0.01307
13.4	100	68.9318	25183	33	0.021109	0.002315	0.01305
12.6	100	62.4787	20892	31	0.004788	0.002376	0.01457
95.1	15.728	25.6187	9531	40	0.002634	0.002376	0.01469
128.3	1.297	66.2198	24102	48	0.000678	0.002543	0.01579
0.3	100	78.8264	27951	34	0.00312	0.002777	0.018
0	100	97.1893	39869	17	0.300893	0.002777	0.01789
115.2	0.829	43.0564	16762	40	0.003019	0.002777	0.01811
37.8	42.407	101.9741	37655	40	0.002653	0.002777	0.0171
0.4	100	114.0468	41475	24	0.172026	0.002843	0.01905
0.6	100	126.0759	47880	38	0.005165	0.003096	0.02009
23.2	100	35.1914	13465	29	0.030268	0.003254	0.02083
38.7	100	43.1699	15285	17	0.694749	0.00337	0.02291
1.4	100	111.1143	41420	23	0.238878	0.00337	0.02241
9.8	100	41.6237	16125	42	0.000227	0.003674	0.02625
1.8	100	130.3295	49706	40	0.003438	0.003674	0.02592
29.1	100	40.0612	14282	14	1.436458	0.003736	0.02755
4.6	100	56.3518	20623	32	0.025404	0.003736	0.02709
195.6	0.664	135.3566	51831	60	3.4E-05	0.003892	0.0283
1.3	100	91.7314	34502	40	0.001924	0.003892	0.02824
5.2	100	62.6804	23004	22	0.034635	0.003967	0.02883
6.1	100	77.2632	28646	27	0.067655	0.004124	0.02959
13.5	100	56.4638	22541	34	0.001315	0.004266	0.03081
88	5.478	65.6598	23870	36	0.01097	0.004266	0.03113
10.8	100	67.662	22915	31	0.035261	0.004344	0.03156
2.1	100	131.3416	50132	43	0.001825	0.004344	0.03133
25.1	100	47.7771	16712	23	0.171201	0.004643	0.03308
128.9	1.049	62.0842	24968	41	0.000484	0.004643	0.03291
2.4	100	137.2805	52649	21	0.22342	0.004643	0.03307
8.1	100	44.715	17460	36	0.007668	0.004803	0.03402
17.3	100	61.5608	24735	29	0.00744	0.005031	0.03518
0.3	100	82.1865	30544	44	0.00081	0.005271	0.03577
34.2	100	53.2401	21177	44	0.001397	0.005618	0.0391
0.8	100	123.7647	48071	25	0.119856	0.00573	0.04053
4.9	100	96.686	39666	15	0.518614	0.00573	0.04153
35.8	20.436	41.1168	15912	29	0.004519	0.00573	0.04073
9.7	100	63.9882	23516	23	0.031648	0.005792	0.04282
6.7	100	44.0688	17190	24	0.128447	0.005865	0.04298
3.5	100	90.333	33941	18	0.255969	0.00599	0.04488
36.2	100	35.0666	11904	30	0.035703	0.006145	0.04573
14.8	100	100.1378	38068	44	0.00077	0.006299	0.04616
2.3	100	136.2705	52221	20	0.321903	0.006428	0.04787

29.4	100	43.6154	15622	26	0.08239	0.006428	0.04685
27.3	100	41.7113	16164	31	0.021414	0.006721	0.04933
12.3	100	143.4775	55823	11	1.564357	0.007057	0.05223
0	100	114.5532	41682	15	1.401658	0.007213	0.05273
2.7	100	136.3871	50399	9	4.946416	0.00728	0.05315
9.4	100	41.9624	14996	31	0.002712	0.007836	0.05679
11	100	82.5131	29515	20	0.420932	0.007836	0.05586
1.3	100	89.9082	31722	20	0.487482	0.007836	0.05669
2.1	100	134.1319	49494	21	0.214706	0.007836	0.05694
38.7	100	40.4799	15660	24	0.108345	0.008171	0.05983
30.1	100	22.944	7368	31	0.029446	0.008293	0.0621
27	100	54.8363	20015	19	0.463939	0.008559	0.06449
1.3	100	114.0896	41495	18	0.737646	0.00861	0.06515
9.2	100	37.678	11720	22	0.2581	0.00861	0.06525
10.8	100	68.4295	24983	27	0.085399	0.008683	0.066
10.5	100	76.2335	28135	24	0.158379	0.008997	0.06834
4.6	100	41.4522	14809	32	0.002308	0.008997	0.06893
1.1	100	83.1757	29008	37	0.001708	0.009069	0.07042
2.4	100	86.6965	32389	17	0.745557	0.009587	0.07651
7.8	100	42.6407	16580	32	0.002029	0.009655	0.07675
15.6	100	48.7849	19228	27	0.006855	0.009764	0.0782
9.4	100	88.8636	33156	39	0.005638	0	0.000751
10.7	100	57.4758	19152	34	0.011095	0	0.000602
0	100	64.7841	23814	71	5.01E-07	0	2.15E-05
46.9	18.16	81.1781	30157	69	1.32E-06	0	3.56E-09
12.1	100	81.1326	30137	32	0.008393	0	0.000362
22.9	100	81.1025	30124	42	0.000716	0	0.000248
48.5	49.106	80.6766	29954	65	3.81E-06	0	3.8E-09
3.5	100	33.7473	10331	37	0.006874	0	0.001288
21.3	24.473	88.3388	36129	43	0.002338	0	0.000455
12	100	87.8289	35902	56	9.46E-05	0	2.63E-05
5.5	100	88.3799	36148	49	0.000502	0	0.00028
13.1	100	79.6086	32431	47	0.000256	0	2.11E-05
127.5	100	51.9961	18899	32	0.018841	0	0.001215
70.2	26.57	52.249	18999	64	1.23E-05	0	4.92E-05
45.3	100	52.5027	19093	43	0.001771	0	0.000127
12.3	34.842	75.6603	26065	63	8.84E-06	0	2.71E-05
32.8	47.462	75.644	26058	84	6.88E-08	0	5.43E-08
7.6	100	79.649	27606	57	3.37E-05	0	0.000202
18.6	100	75.1569	25868	39	0.002127	0	0.000497
5.8	100	80.0991	29723	49	0.000521	0	4.78E-05
11.5	100	14.8841	4130	56	3.55E-05	0	0.000336
18.6	31.388	74.7109	27516	64	5.96E-06	0	2.04E-05
9.5	21.383	74.7007	27512	60	1.44E-05	0	3.46E-05
4.2	100	80.4425	29856	41	0.003059	0	9.35E-06
4.5	100	80.4543	29860	30	0.044237	0	0.000825
10.8	72.615	87.2278	32615	47	0.000754	0	0.000176
55.3	25.13	87.1748	32592	47	0.000877	0	8.49E-05
14.3	100	80.6036	29920	54	0.000161	0	9.82E-08
26.2	100	46.8043	16331	53	0.000111	0	3.93E-05
7.6	100	49.6761	17476	63	1.12E-05	0	1.92E-06
11.2	57.774	65.3424	23749	47	0.000805	0	2.54E-06

52	27.254	64.9102	23573	29	0.049688	0	6.58E-06
15.1	6.807	64.8374	23540	57	7.66E-05	0	5.54E-07
3	100	64.3869	23354	40	0.004063	0	0.000811
11.3	100	66.1359	24067	29	0.046135	0	0.000496
2.1	100	117.3151	45327	66	9.84E-06	0	1.44E-05
26.3	83.957	117.2755	45309	83	2.3E-07	0	1.32E-08
124.5	100	15.8294	5005	39	0.003349	0	0.000112
30.7	100	34.8449	11951	22	0.154352	0	0.000694
42.9	44.98	35.3489	12141	52	0.00015	0	4.31E-06
2.4	100	114.1533	44463	40	0.001723	0	0.000294
7.7	100	33.7972	11200	45	0.000852	0	0.000106
33.5	80.289	141.0556	54466	111	1.72E-10	0	4.26E-13
14.5	100	58.332	23338	45	0.000884	0	0.000323
11.1	96.445	57.8286	23129	51	0.000196	0	3.88E-05
14.5	100	58.8411	23546	47	0.000507	0	0.000234
12.6	68.891	56.3108	22469	43	0.001385	0	0.000393
20.2	59.818	56.8188	22697	52	0.000169	0	0.000157
10.9	53.682	57.3208	22917	50	0.000244	0	6.43E-05
50.6	100	54.7071	21793	36	0.005904	0	4.56E-05
86.3	100	55.2155	22010	47	0.000508	0	3.31E-05
51.9	54.979	55.7231	22223	35	0.007821	0	0.000458
28.6	100	82.4451	30423	56	6.72E-05	0	2.56E-05
41	100	82.472	30435	51	0.000212	0	0.000487
25.3	100	81.9404	30225	40	0.002522	0	0.000408
75.4	100	80.9725	29817	49	0.000345	0	9.67E-05
77.3	15.859	81.4737	30033	54	0.000106	0	6.92E-05
115.6	17.76	81.4598	30027	48	0.00037	0	0.000562
8.2	100	86.6687	32377	37	0.007532	0.000141	0.001605
103.8	100	46.7941	16327	52	0.000141	0.000273	0.001881
70.6	100	67.2775	27172	50	0.00043	0.000407	0.002148
154.1	56.112	33.9418	11253	16	0.732627	0.000525	0.002572
73.6	70.057	81.9632	30236	45	0.00075	0.000525	0.002196
4.8	100	88.9362	33185	43	0.002373	0.000525	0.002425
48	100	55.9727	20473	49	0.000336	0.000758	0.002875
10.8	100	82.9798	30629	39	0.003703	0.000758	0.002957
8.7	100	34.2258	13055	8	6.094226	0.000868	0.003358
31.2	100	65.9175	23975	20	0.478068	0.000951	0.004343
9.6	100	47.2518	15163	38	0.004053	0.000951	0.004121
90.9	100	68.2649	25171	41	0.003169	0.001022	0.004763
63.9	100	33.9576	11599	18	0.431034	0.001022	0.004645
3.4	100	68.2491	24911	33	0.019496	0.001022	0.00575
10.5	100	93.3631	34897	38	0.006276	0.001022	0.004863
4.1	100	59.3479	23749	32	0.015433	0.001114	0.005976
6	100	81.1064	30120	23	0.203764	0.001114	0.005961
5.2	100	88.864	36344	31	0.029349	0.001308	0.006978
0	100	86.2921	35224	38	0.007545	0.001576	0.00877
58.8	5.609	12.9228	3517	54	9.87E-05	0.001576	0.008214
7.7	100	46.3004	16132	33	0.010832	0.001758	0.009329
1.4	100	57.2152	20957	26	0.064223	0.001758	0.00914
140.2	17.404	55.6868	20362	48	0.000356	0.001758	0.009187
39.1	100	35.0588	12034	14	1.103304	0.001758	0.009582
147	100	19.2207	5971	21	0.122775	0.001854	0.009912

2.3	100	35.3891	12159	35	0.008685	0.001945	0.009971
16.2	100	47.4328	17103	31	0.020349	0.002012	0.01126
1.1	100	89.131	33258	27	0.076448	0.002079	0.01183
60.3	17.351	66.7677	26947	41	0.003276	0.002079	0.01191
36.6	100	47.2616	15167	39	0.002909	0.002079	0.01173
0.9	100	36.87	11410	20	0.416893	0.002257	0.0127
1.3	100	14.9109	4139	40	0.001427	0.002315	0.01373
13.4	100	59.4806	21791	29	0.033294	0.002376	0.01447
16.3	100	72.0976	26714	31	0.021621	0.002376	0.01432
9.1	100	57.7237	21152	24	0.088478	0.002376	0.01509
114.9	38.485	12.9948	3519	54	9.88E-05	0.002543	0.01607
24.2	100	59.9358	24007	25	0.079348	0.002543	0.01627
11.5	100	54.2041	21576	19	0.266582	0.002719	0.01679
8.1	100	80.6278	29934	24	0.054755	0.002777	0.01749
18.5	100	54.1349	21545	25	0.08806	0.002843	0.01852
21.2	100	46.2893	16128	29	0.03098	0.002843	0.01843
22.6	100	54.3713	21649	19	0.316291	0.003096	0.01982
8.1	54.226	63.1283	25400	45	0.001421	0.003178	0.02064
1.3	100	35.2379	13486	22	0.249065	0.00337	0.02348
2	100	80.7573	30542	22	0.160893	0.00337	0.02312
13.7	100	42.4747	15186	35	0.010557	0.003533	0.02455
30.3	100	113.1197	44035	14	0.383236	0.003674	0.02505
71.5	6.802	59.3347	21738	35	0.009383	0.003674	0.02543
2.3	100	50.7778	16467	14	0.972369	0.005031	0.03479
1.1	100	65.0788	23645	23	0.246861	0.005031	0.03484
4.6	100	56.7106	20762	20	0.269228	0.005404	0.03755
18.6	100	66.2622	26730	29	0.05023	0.005618	0.03834
36.3	100	68.3093	25192	26	0.116099	0.00599	0.04526
1.5	100	63.8827	23154	24	0.163686	0.006497	0.04832
29.5	20.658	66.8053	26963	26	0.110004	0.007572	0.05449
1	100	58.2253	21339	18	0.370414	0.0081	0.05937
33.5	100	67.8025	24994	22	0.31643	0.008171	0.05976
1.2	100	33.8323	11214	21	0.20071	0.00822	0.06038
1.6	100	62.3675	21071	14	1.925225	0.00861	0.06536
16	100	58.858	19541	29	0.035087	0.008746	0.06654
2.6	100	58.7429	21522	11	2.207159	0.008997	0.06886
2.5	100	58.727	21516	16	0.661512	0.008997	0.0684
67.2	100	80.9559	29809	28	0.042216	0.009436	0.07142
4.9	100	33.6106	10285	12	2.488187	0.009498	0.072
7.3	100	56.2085	20567	11	1.921057	0.009985	0.08299
4.3	100	88.2637	32922	84	1.15E-07	0	9.04E-08
29.3	100	52.0353	18914	48	0.000654	0	8.48E-05
40.4	100	51.9262	18873	54	0.000148	0	7.34E-06
41.8	48.083	53.4567	19467	33	0.009635	0	0.000101
42.3	62.819	52.9523	19263	25	0.057544	0	0.00031
35.9	100	52.4475	19071	28	0.030414	0	0.000159
2.1	100	72.2517	24683	55	6.18E-05	0	0.000789
34	100	86.5791	30347	50	0.000342	0	0.000144
73.2	27.205	85.3642	29851	62	3.03E-05	0	3.63E-05
13.9	21.666	76.8212	28374	65	1.39E-05	0	4.05E-06
9.1	12.278	140.5223	55223	58	6.4E-05	0	0.000133
168.4	6.779	140.5198	55220	74	1.64E-06	0	8.21E-07

80	30.625	31.7674	10744	26	0.103131	0	0.00013
5.3	100	131.2485	50092	49	0.000285	0	6.7E-06
60.9	100	65.5531	23902	49	0.000551	0	0.000515
16.3	100	50.2957	17682	47	0.000591	0	0.000496
111	12.736	40.7156	13866	45	0.000938	0	8.81E-05
12.5	100	69.6995	28231	30	0.005385	0	0.000554
31.9	100	84.2945	30227	60	3.11E-05	0	2.02E-05
15.5	100	84.3149	30236	55	0.0001	0	6.36E-05
250.8	13.883	13.1112	3649	38	0.001856	0	2.3E-05
88.1	18.753	13.1128	3651	33	0.005872	0	0.000254
37.6	100	27.3783	10235	41	0.003181	0	0.000351
18.2	100	39.4647	15232	42	0.002383	0	6.82E-05
18.6	100	53.102	19320	28	0.030735	0.000407	0.002137
2	100	19.3521	5683	55	2.94E-05	0.000525	0.002646
18.4	100	53.6079	19529	25	0.062158	0.000525	0.0024
62.5	100	15.4622	4794	29	0.044034	0.000758	0.003152
14	100	28.2021	9800	37	0.007644	0.000868	0.003528
18.2	100	60.4777	22157	29	0.031315	0.000868	0.00342
29.6	100	48.1427	18934	49	0.000499	0.000951	0.004367
111.8	100	33.3955	11381	40	0.002499	0.001022	0.004847
63.9	100	33.9767	12063	31	0.032022	0.001022	0.005631
46.6	100	52.611	18648	30	0.032244	0.001022	0.004982
105.6	54.927	33.4913	12750	26	0.131544	0.00121	0.006742
5.2	100	84.9916	29711	32	0.030724	0.00121	0.006842
8.5	100	76.8931	28405	39	0.005319	0.00121	0.006758
9.6	100	86.9101	30489	35	0.010059	0.001576	0.008409
19.7	100	39.9652	15439	17	0.739766	0.001758	0.009499
33	100	40.2115	13667	32	0.023773	0.001945	0.009945
18.4	100	46.7101	16725	42	0.000522	0.002012	0.01082
52.7	100	42.6124	15056	30	0.029448	0.002079	0.01137
14.7	100	83.8082	30023	25	0.095181	0.002315	0.0135
9	100	52.5951	19130	21	0.156925	0.002376	0.01475
21.2	100	42.6062	16563	31	0.02802	0.002543	0.01571
21.2	100	42.6062	16563	31	0.02802	0.002543	0.01571
10.3	100	15.5326	4358	22	0.133901	0.002543	0.01582
32.4	100	45.3866	16200	36	0.009491	0.002719	0.01655
62.8	91.571	47.6405	18710	32	0.025557	0.002777	0.01724
5.6	100	86.4051	30268	30	0.035838	0.002777	0.01746
37.9	100	47.6071	18694	36	0.009152	0.009498	0.0721
26.5	100	51.1415	16701	26	0.089474	0	0.001526
2.5	100	87.4667	32588	17	0.791041	0	0.000123
12.7	100	87.263	32508	12	2.622911	0	0.000246
14.9	100	86.9601	32386	12	2.587306	0	0.000267
0.3	67.398	51.384	16790	85	1.14E-07	0	2.85E-08
8	100	101.7262	38275	16	1.00906	0	0.000642
24	100	103.7345	39036	40	0.003876	0	1.03E-08
1.5	100	103.4284	38929	26	0.090676	0	9.05E-05
38.2	83.296	103.2323	38856	48	0.000594	0	2.25E-10
6.3	100	102.9209	38741	32	0.026003	0	7.67E-07
[truncated: 552,872 more chars]
